# Identification of the relationship between 1400 blood metabolites and urolithiasis: A bidirectional Mendelian randomization study

**DOI:** 10.1097/MD.0000000000041911

**Published:** 2025-03-21

**Authors:** Haoyang Zhang, Haojie Mo, Peng Li, Qi Zhou, Gang Shen, Jiale Sun

**Affiliations:** a Department of Urology, The Fourth Affiliated Hospital of Soochow University, Suzhou, Jiangsu, China; b Department of Reproductive Medicine Center, The First Affiliated Hospital of Soochow University, Suzhou, Jiangsu, China.

**Keywords:** blood metabolites, causality, Mendelian randomization, single-nucleotide polymorphisms, urolithiasis

## Abstract

Relationships between blood metabolites and urolithiasis have been identified in few previous observational studies, and causality remains uncertain. We tried to examine whether blood metabolites were causally associated with upper and lower urinary stones in this bidirectional Mendelian randomization (MR) study. The causal relationship between 1400 blood metabolites and upper and lower urinary stones was investigated using genome-wide association study data. The primary analysis for causality analysis was the inverse variance weighted method, with 4 other methods used as complementary analyses. Intersection was then conducted to show the shared metabolites between upper and lower urinary tract stones, followed by the MR-Egger intercept test, Cochran Q test, leave-one-out analysis, MR-PRESSO and the linkage disequilibrium score regressions. The metabolic pathway analysis was conducted to identify potential metabolic pathways. Lastly, reverse MR analyses were also performed. We identified 15 metabolites as potential causal predictors of urinary stones in forward MR analyses. These metabolites consisted of 1 azole, 2 carbohydrates, 6 lipids, 1 nucleotide, 1 peptide, 1 urea, and 3 metabolites with unknown chemical properties. Additionally, urinary stones were found to be significantly associated with some of the above metabolites in reverse MR analyses. Metabolic pathway analysis identified several pathways that may be implicated in the development of urolithiasis. This MR study has established a causal relationship between 12 blood metabolites and the risk of upper and lower urinary tract stones. The identification of these blood metabolites provides valuable insights into early screening, prevention, and treatment of urolithiasis.

## 1. Introduction

Urolithiasis is a common disease encountered in urology, with an overall prevalence of 7% to 13% in North America.^[1]^ Over the last few decades, there has been an increasing incidence of urinary stones in both developed and developing countries.^[2]^ The occurrence of urinary stones increases with age and the recurrence rate can reach 50% within 5 years after initial treatment,^[3]^ imposing a huge public health and financial burden.^[4]^ Although treatments for urolithiasis have improved in terms of efficacy and safety, it has not been cured. Therefore, it is crucial to identify potential risk factors for urinary stones. Various factors have been reported to be associated with the development of urinary stones, including gender, coffee consumption, body mass index (BMI), and diabetes.^[5–7]^ Many studies indicated that urolithiasis is a chronic metabolic disorder.^[8]^

Nonetheless, limited research has been conducted on metabolic changes in urinary stones.

Metabolites serves as functional intermediates to understand the occurrence and development of several diseases.^[9]^ Although the mechanism was not fully understood, urolithiasis was thought to be influenced by blood metabolites,^[10]^ which could act as inhibitors or promoters for the development of urinary stones. Metabolomics is a systematic study of metabolites associated with metabolic processes in organisms, providing a novel approach to investigating the biological pathogenesis of urinary stones. This technique is able to elucidate the mechanisms of disease by identifying specific biomarkers related to environmental exposures and diseases.^[11]^ Exploration of the metabolites connected with the development of urolithiasis can not only contribute to early screening and prevention of urinary stones, but also help to understand the mechanisms for treatment. The causal association between blood metabolites and urinary stones remains to be established.

Mendelian randomization (MR) is an epidemiological method of analysis that uses single nucleotide polymorphisms (SNPs) as instrumental variables (IVs) to simulate randomized controlled trials. MR analysis can be a reliable tool for assessing causality between exposure to risk factors and outcomes, with the advantage of reducing bias from reverse causality and confounding factors.^[12]^ In this study, the causal relationship between 1400 blood metabolites and urolithiasis was investigated using genome-wide association studies (GWAS) data. The primary analysis for causality analysis was the inverse variance weighted (IVW) method, with 4 other methods used as complementary analyses. Intersection was then conducted to show the shared metabolites between upper and lower urinary tract stones, followed by the MR-Egger intercept test, Cochran Q test, leave-one-out analysis, MR-PRESSO and the linkage disequilibrium score (LDSC) regressions. The metabolic pathway analysis was conducted to identify potential metabolic pathways.

## 2. Methods

### 2.1. Study design

**Figure 1.**
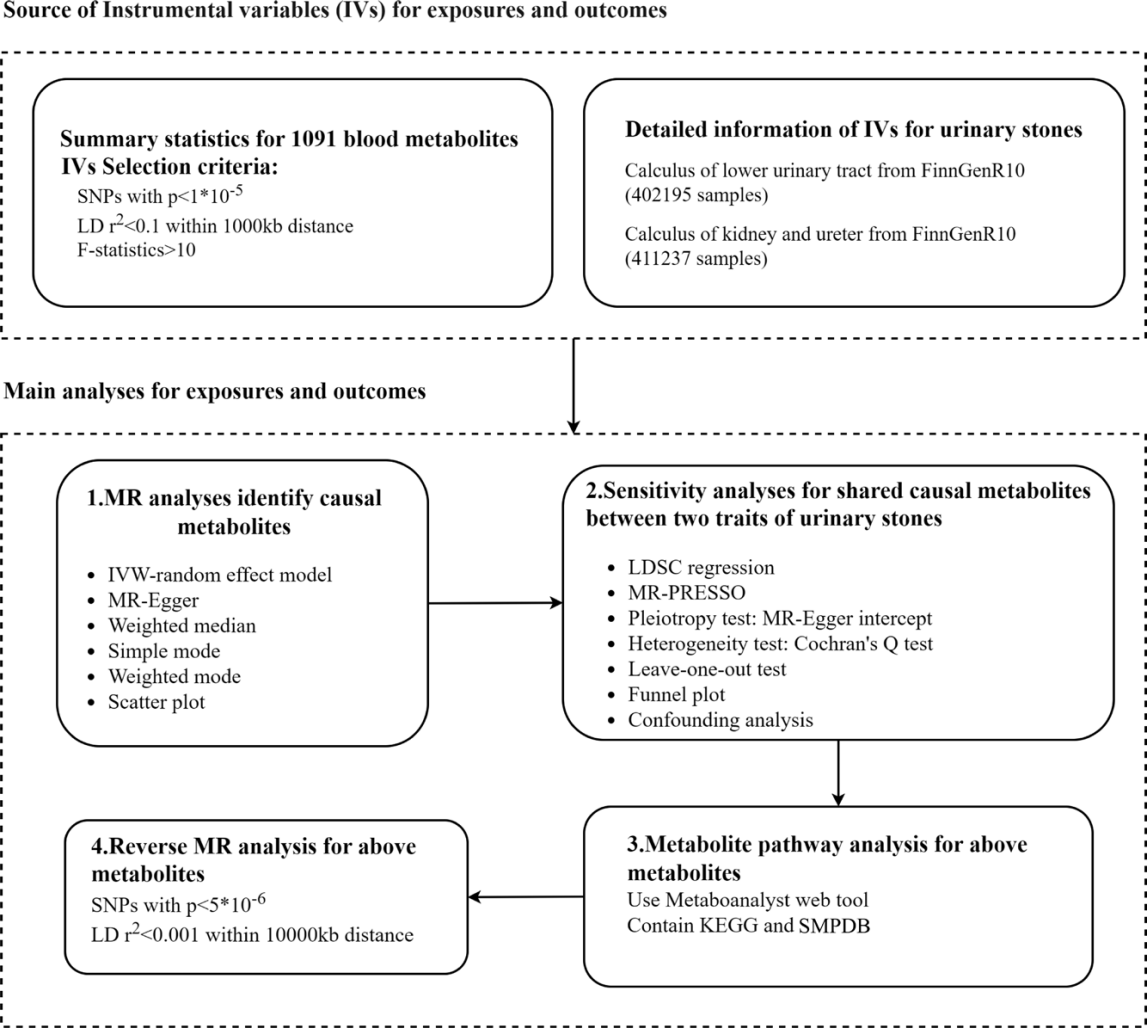
The flowchart of Mendelian Randomization analysis of 1400 metabolites and risk of urinary stones. Among the above metabolites, 309 which were defined as metabolite ratios were excluded in our study. IV = instrumental variables, SNPs = single nucleotide polymorphisms, LD = linkage disequilibrium, IVW = inverse variance weighted, LDSC = linkage disequilibrium score, MR = Mendelian randomization, MR-PRESSO = MR-Pleiotropy RESidual sum and outlier, KEGG = Kyoto Encyclopedia of Genes and Genomes, SMPDB = The Small Molecule Pathway Database.

Figure 1 shows our two-sample MR design comparing 1400 human blood metabolites with the risk of urinary stones. We used summary statistics from a GWAS to investigate the causal relationship between blood metabolites and upper and lower urinary stones. The chosen IVs needed to comply with 3 assumptions: (1) IVs must be strongly associated with the exposure of interest; (2) IVs must be independent of unmeasured confounders; and (3) IVs must be related to outcomes solely through the exposure of interest rather than through confounders. In this MR study, the risk of urinary stones and human blood metabolites were respectively considered as the exposure and outcome.

### 2.2. Sources of GWAS data

The metabolite database was sourced from one of the most comprehensive metabolite studies by Chen et al.^[13]^ By conducting studies on 1091 metabolites and 309 metabolite ratios in 8299 individuals of European descent from the Canadian longitudinal study on aging cohort, this report eventually identified almost 15.4 million SNPs for GWAS testing. The detailed information of 1400 metabolites is presented in Table S1, Supplemental Digital Content, http://links.lww.com/MD/O573. The urinary stones datasets were obtained from FinnGen R10,^[14]^ with 10,566 cases and 400,681 controls in calculus of kidney and ureter, and 1514 cases and 400,681 controls in calculus of lower urinary tract.

### 2.3. Filtering of IVs for MR analyses and confounding analysis

We chose a lenient threshold for genome-wide significance using *P* < 5 × 10^–5^, because of the limited number of SNPs reaching GWAS. Then we conducted linkage disequilibrium analyses (r^2^ = 0.1 and with 1000kb window) among the included IVs to filter independent instrument SNPs. The F-statistic was evaluated to quantify the strength of genetic variation using the formula: F = (β/SE)^2^, where β denotes the estimated genetic effect of each SNP, and SE is the standard error of β. SNPs with an F-statistic of <10 were abandoned, indicativing weak association. Additionally, we conducted confounding analysis utilizing the Phenoscanner V2 website (http://www.phenoscanner.medschl.cam.ac.uk/) to investigate whether metabolite-associated SNPs were also connected to common risk factors that might influence MR estimates, including BMI^[15]^, diabetes mellitus^[16]^, serum calcium, and serum 1,25(OH)_2_D3.^[17]^

### 2.4. MR analysis

Several MR analyses including IVW, MR-Egger, Weighted median, Simple mode, and Weighted mode were employed to evaluate the causal association between human blood metabolites and urinary stones. Additionally, 2 reverse MR analyses were performed to investigate the causal association of upper and lower urinary stones on blood metabolites. A *P*-value < 0.05 was considered statistically significant.

### 2.5. Sensitivity analysis and LDSC analysis

We performed MR-Egger intercept test to detect potential horizontal or pleiotropic biases for significant estimates. Secondly, we employed Cochran Q test to identify heterogeneity among the selected SNPs. To assess the robustness of the causal estimates, we also presented leave-one-out plots to identify strong influences of SNPs. In addition, the LDSC regressions were performed to assess whether the genetic associations are influenced by common genetic factors.^[18]^ The odds ratio (OR) and 95% confidence intervals (CIs) were used to estimate the degree of metabolic impact. All statistical analyses were conducted using the “TwoSampleMR,” “MRPRESSO” package and “ldscr” packages in R software (version 4.3.0, https://www.r-project.org/). A significant difference was considered when *P* < .05.

### 2.6. Metabolic pathway analysis

We used Web-based metaconflict 5.0 (https://www.Metaboanalyst.ca/) to estimate metabolic pathways. The pathway and enrichment analysis modules were utilized to identify clusters of metabolites or superpathways that may be associated with metabolic processes and urinary stone associations. We made use of the small molecule pathway database and the Kyoto Encyclopedia of Genes and Genomes database as reference.^[19,20]^

## 3. Results

### 3.1. Selected genetic IVs

After removing 309 metabolite ratios, a total of 1091 metabolites were retained for MR analysis in the instrument SNP selection procedure (Table S1, Supplemental Digital Content, http://links.lww.com/MD/O573). Fifteen metabolites were detected to be both significantly associated with upper and lower urinary stones. The number of SNPs for each metabolite ranged from 15 to 90. None of the F-values for SNP inclusion were <10, indicating no potentially weak instruments were employed (Tables S2 and S3, Supplemental Digital Content, http://links.lww.com/MD/O574). We also examined several common factors including BMI, diabetes mellitus, serum calcium, and serum 1,25(OH)_2_D3 to assess their potential impact as confounding variables, and several SNPs associated with any confounding factors were excluded from the instrument SNP before conducting MR analysis (Tables S4, Supplemental Digital Content, http://links.lww.com/MD/O575 and S5, Supplemental Digital Content, http://links.lww.com/MD/O576).

### 3.2. Primary analysis

**Figure 2.**
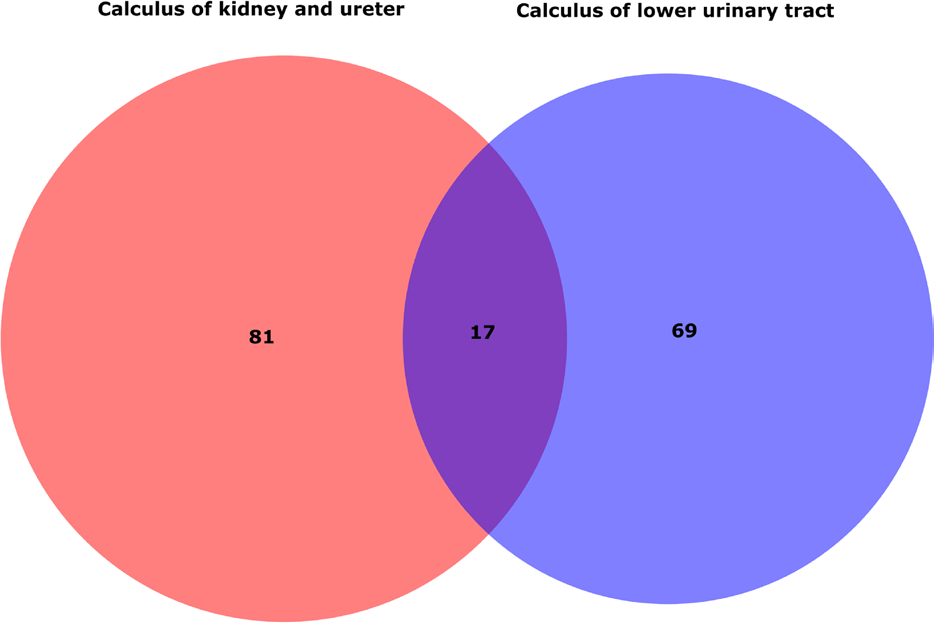
Intersection analysis of significantly associated metabolites on upper and lower urinary stones. The Venn diagram showed the shared 17 metabolites screened by forward MR analyses. MR = Mendelian randomization.

IVW results identified 98 metabolites related to calculus of kidney and ureter, and 86 metabolites related to calculus of lower urinary tract. Intersection analysis showed 17 shared casual metabolites between upper and lower urinary tract stones (Fig. 2). Among them, 2 were removed due to lack of LDSC analysis results and 15 metabolites showed weak evidence of genetic correlation (Tables S6 and S7, Supplemental Digital Content, http://links.lww.com/MD/O577). Finally, these 15 metabolites were detected significantly associated to upper and lower urinary stones, of which 3 metabolites remained chemically unknown.

### 3.3. Tightened MR analysis

**Figure 3.**
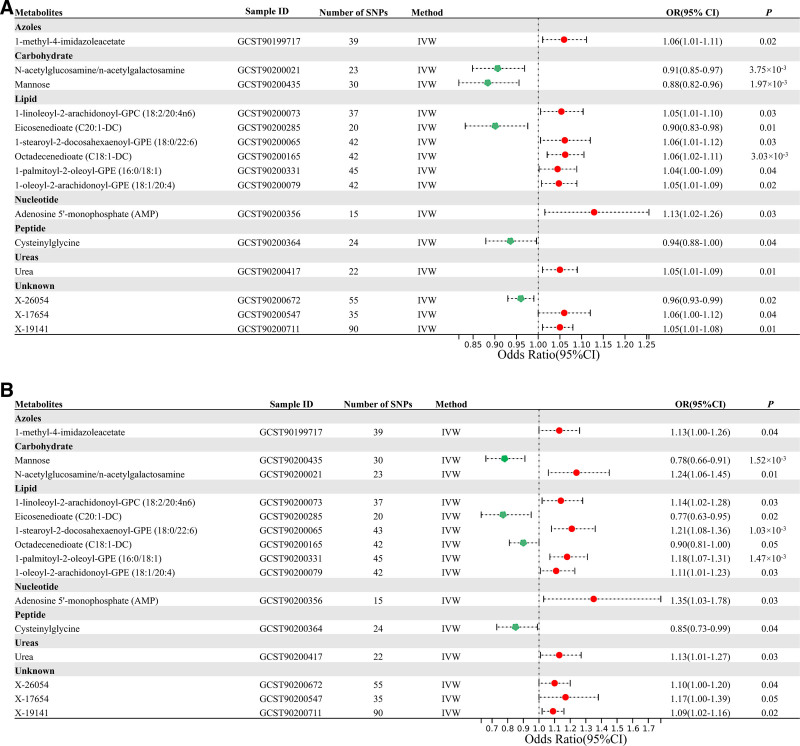
Forest plot of the causal effects of 15 metabolites on the risk of urinary stones derived from the IVW method. (A) Causal effects of 15 metabolites on the risk of upper urinary stones. (B) Causal effects of 15 metabolites on the risk of lower urinary stones. CI = confidence interval, IVW = inverse variance weighted, OR = odds ratio.

Among all above metabolites, 10 metabolites increased the risk of upper urinary stones, including 1-methyl-4-imidazoleacetate (OR = 1.06, 95%CI = 0.95–1.16, *P* = .0174), 1-linoleoyl-2-arachidonoyl-GPC (OR = 1.05, 95%CI = 0.95–1.14, *P* = .0284), 1-stearoyl-2-docosahexaenoyl-GPE (OR = 1.06, 95%CI = 0.92–1.15, *P* = .0311), octadecenedioate (OR = 1.06, 95%CI = 0.99–1.13, *P* = .0030), 1-palmitoyl-2-oleoyl-GPE (OR = 1.04, 95%CI = 0.94–1.11, *P* = .0392), 1-oleoyl-2-arachidonoyl-GPE (OR = 1.05, 95%CI = 0.92–1.06, *P* = .0193), adenosine 5′-monophosphate (OR = 1.13, 95%CI = 0.84–1.42, *P* = .0250), urea (OR = 1.05, 95%CI = 1.00–1.09, *P* = .0095), X-17654 (OR = 1.06, 95%CI = 0.92–1.19, *P* = .0413), and X-19141 (OR = 1.05, 95%CI = 0.99–1.13, *P* = .0052). Five metabolites decreased the risk of upper urinary stones, including mannose (OR = 0.88, 95%CI = 0.69–0.97, *P* = .0020), N-acetylglucosamine/n-acetylgalactosamine (OR = 0.91, 95%CI = 0.81–1.05, *P* = .0037), eicosenedioate (OR = 0.90, 95%CI = 0.81–1.11, *P* = .0105), cysteinylglycine (OR = 0.94, 95%CI = 0.84–1.15, *P* = .0373), and X-26054 (OR = 0.96, 95%CI = 0.93–1.05, *P* = .0200). Detailed information was available in Fig. 3 and Table 1. MR-Egger regression analyses and MR-PRESSO tests were performed, and no incidence of potential pleiotropy was identified, validating the reliability of MR analyses. Cochran Q *P*-value indicated the absence of heterogeneity (Table 1). In Figs. S1–S4, Supplemental Digital Content, http://links.lww.com/MD/O578, forest plots, funnel plots, scatter plots, and leave-one-out plots were displayed.

**Table 1:** MR analysis and sensitivity analyses for causality from 15 blood metabolites on upper urinary stones.

Name	Exposure	Metabolites	Number of SNPs	MR analysis					Heterogeneity				Pleiotropy		MR-PRESSO
				Method	OR (95% CI)	Lower CI	Upper CI	P	IVW		MR Egger		Intercept	P	P
									Q	P	Q	P			
1-methyl-4-imidazoleacetate	GCST90199717	Azoles	39	IVW	1.06	0.95	1.16	0.0174	44.55	0.2154	44.46	0.1863	0.0024	0.7856	0.2448
Mannose	GCST90200435	Carbohydrate	30	IVW	0.88	0.69	0.97	0.0020	49.00	0.0115	47.23	0.0130	0.0118	0.3141	0.0650
N-acetylglucosamine/n-acetylgalactosamine	GCST90200021	Carbohydrate	23	IVW	0.91	0.81	1.05	0.0037	26.45	0.2330	26.32	0.1946	-0.0030	0.7502	0.2870
1-linoleoyl-2-arachidonoyl-GPC (18:2/20:4n6)	GCST90200073	Lipid	37	IVW	1.05	0.95	1.14	0.0284	43.84	0.1731	43.74	0.1477	0.0022	0.7715	0.1626
Eicosenedioate (C20:1-DC)	GCST90200285	Lipid	20	IVW	0.90	0.81	1.11	0.0105	17.71	0.5416	17.24	0.5069	-0.0096	0.4984	0.6176
1-stearoyl-2-docosahexaenoyl-GPE (18:0/22:6)	GCST90200065	Lipid	42	IVW	1.06	0.92	1.15	0.0311	63.30	0.0184	62.75	0.0160	0.0051	0.5521	0.5110
Octadecenedioate (C18:1-DC)	GCST90200165	Lipid	42	IVW	1.06	0.99	1.13	0.0030	37.63	0.6211	37.54	0.5816	0.0018	0.7607	0.6326
1-palmitoyl-2-oleoyl-GPE (16:0/18:1)	GCST90200331	Lipid	45	IVW	1.04	0.94	1.11	0.0392	48.77	0.2870	48.25	0.2691	0.0047	0.4968	0.3034
1-oleoyl-2-arachidonoyl-GPE (18:1/20:4)	GCST90200079	Lipid	42	IVW	1.05	0.92	1.06	0.0193	46.72	0.2492	43.19	0.3367	0.0113	0.0781	0.2926
Adenosine 5’-monophosphate (AMP)	GCST90200356	Nucleotide	15	IVW	1.13	0.84	1.42	0.0250	8.01	0.8888	7.94	0.8478	0.0037	0.7893	0.9012
Cysteinylglycine	GCST90200364	Peptide	24	IVW	0.94	0.84	1.15	0.0373	28.82	0.1864	28.32	0.1653	-0.0067	0.5401	0.1962
Urea	GCST90200417	Ureas	22	IVW	1.05	1.00	1.09	0.0095	12.09	0.9372	11.72	0.9252	0.0034	0.5535	0.9640
X-26054	GCST90200672	Unknown	55	IVW	0.96	0.93	1.05	0.0200	47.09	0.7358	45.77	0.7489	‐0.0059	0.2560	0.7630
X-17654	GCST90200547	Unknown	35	IVW	1.06	0.92	1.19	0.0413	35.69	0.3890	35.64	0.3453	0.0019	0.8329	0.4124
X-19141	GCST90200711	Unknown	90	IVW	1.05	0.99	1.13	0.0052	143.00	0.0002	142.86	0.0002	-0.0018	0.7677	0.5020

CI = confidence interval, IVW = inverse variance weighted, MR = Mendelian randomization, MR-PRESSO = MR-Pleiotropy RESidual sum and outlier, OR = odds ratio, SNPs = single nucleotide polymorphisms.

Besides, 11 metabolites increased the risk of lower urinary stones, including 1-methyl-4-imidazoleacetate (OR = 1.13, 95%CI = 1.00–1.26, *P* = .0410), N-acetylglucosamine/n-acetylgalactosamine (OR = 1.24, 95%CI = 1.06–1.45, *P* = .0068), 1-linoleoyl-2-arachidonoyl-GPC (OR = 1.14, 95%CI = 1.02–1.28, *P* = .0254), 1-stearoyl-2-docosahexaenoyl-GPE (OR = 1.21, 95%CI = 1.08–1.36, *P* = .0010), 1-palmitoyl-2-oleoyl-GPE (OR = 1.18, 95%CI = 1.01–1.50, *P* = .0015), 1-oleoyl-2-arachidonoyl-GPE (OR = 1.11, 95%CI = 1.01–1.23, *P* = .0255), Adenosine 5′-monophosphate (OR = 1.35, 95%CI = 1.03–1.78, *P* = .0309), urea (OR = 1.13, 95%CI = 0.97–1.27, *P* = .0299), X-26054 (OR = 1.10, 95%CI = 0.97–1.36, *P* = .0401), X-17654 (OR = 1.17, 95%CI = 1.00–1.38, *P* = .0491), and X-19141 (OR = 1.09, 95%CI = 0.91–1.17, *P* = .0160). On the other hand, 4 metabolites decreased the risk of lower urinary stones, including mannose (OR = 0.78, 95%CI = 0.51–1.03, *P* = .0015), eicosenedioate (OR = 0.77, 95%CI = 0.52–1.19, *P* = .0167), octadecenedioate (OR = 0.90, 95%CI = 0.81–1.00, *P* = .0454), and cysteinylglycine (OR = 0.85, 95%CI = 0.51–1.13, *P* = .0429) (Fig. 3 and Table 2). No risk of potential pleiotropy was found in the MR-Egger regression analyses or MR-PRESSO tests (Table 2). Cochran Q *P*-value indicated heterogeneity in some causal associations, but this was acceptable in the MR study (Table 2). Forest plots, funnel plots, scatter plots and leave-one-out plots were presented in Figs. S5–S8, Supplemental Digital Content, http://links.lww.com/MD/O578, demonstrating that the estimates were unaffected by individual SNPs and there were no violations of assumptions.

**Table 2:** MR analysis and sensitivity analyses for causality from 15 blood metabolites on lower urinary stones.

Name	Exposure	Metabolites	Number of SNPs	MR analysis					Heterogeneity				Pleiotropy		MR-PRESSO
				Method	OR (95% CI)	Lower CI	Upper CI	P	IVW		MR Egger		Intercept	P	P
									Q	P	Q	P			
1-methyl-4-imidazoleacetate	GCST90199717	Azoles	39	IVW	1.13	1.00	1.26	0.0410	32.54	0.7195	31.60	0.7197	0.0200	0.3392	0.7404
Mannose	GCST90200435	Carbohydrate	30	IVW	0.78	0.51	1.03	0.0015	14.69	0.9873	14.53	0.9830	0.0094	0.6875	0.9916
N-acetylglucosamine/n-acetylgalactosamine	GCST90200021	Carbohydrate	23	IVW	1.24	1.06	1.45	0.0068	20.20	0.5706	20.20	0.5087	0.0000	0.9986	0.6320
1-linoleoyl-2-arachidonoyl-GPC (18:2/20:4n6)	GCST90200073	Lipid	37	IVW	1.14	1.02	1.28	0.0254	38.61	0.3524	38.25	0.3240	‐0.0103	0.5694	0.3674
Eicosenedioate (C20:1-DC)	GCST90200285	Lipid	20	IVW	0.77	0.52	1.19	0.0167	15.21	0.7093	15.20	0.6483	‐0.0035	0.9247	0.7106
1-stearoyl-2-docosahexaenoyl-GPE (18:0/22:6)	GCST90200065	Lipid	43	IVW	1.21	1.08	1.36	0.0010	28.05	0.9514	27.65	0.9449	0.0114	0.5329	0.9596
Octadecenedioate (C18:1-DC)	GCST90200165	Lipid	42	IVW	0.90	0.81	1.00	0.0454	41.44	0.4514	41.36	0.4110	-0.0042	0.7820	0.4790
1-palmitoyl-2-oleoyl-GPE (16:0/18:1)	GCST90200331	Lipid	45	IVW	1.18	1.01	1.50	0.0015	38.71	0.6970	38.49	0.6671	-0.0079	0.6374	0.7234
1-oleoyl-2-arachidonoyl-GPE (18:1/20:4)	GCST90200079	Lipid	42	IVW	1.11	1.01	1.23	0.0255	39.64	0.5311	39.59	0.4886	-0.0036	0.8217	0.5690
Adenosine 5’-monophosphate (AMP)	GCST90200356	Nucleotide	15	IVW	1.35	1.03	1.78	0.0309	10.69	0.7102	9.74	0.7147	0.0343	0.3485	0.7212
Cysteinylglycine	GCST90200364	Peptide	24	IVW	0.85	0.51	1.13	0.0429	26.67	0.2703	26.23	0.2419	0.0162	0.5510	0.3110
Urea	GCST90200417	Ureas	22	IVW	1.13	0.97	1.27	0.0299	29.27	0.1077	28.79	0.0919	0.0101	0.5715	0.3722
X-26054	GCST90200672	Unknown	55	IVW	1.10	0.97	1.36	0.0401	60.64	0.2489	60.27	0.2296	-0.0082	0.5705	0.2650
X-17654	GCST90200547	Unknown	35	IVW	1.17	1.00	1.38	0.0491	45.49	0.0901	43.48	0.1048	0.0309	0.2258	0.0934
X-19141	GCST90200711	Unknown	90	IVW	1.09	0.91	1.17	0.0160	82.05	0.6860	81.11	0.6854	0.0122	0.3342	0.6796

CI = confidence interval, IVW = inverse variance weighted, MR = Mendelian randomization, MR-PRESSO = MR-Pleiotropy RESidual sum and outlier, OR = odds ratio, SNPs = single nucleotide polymorphisms.

According to the forward MR analyses, 3 metabolites (N-acetylglucosamine/n-acetylgalactosamine, Octadecenedioate and X-26054) were removed before the reverse MR analyses due to their opposite effects on upper and lower tract urinary stones. The upper urinary tract stones increase mannose production in the blood (OR = 1.06, 95%CI = 1.00–1.13, *P* = .0449). Additionally, the upper urinary tract stones increase the generation of 1-oleoyl-2-arachidonoyl-GPE (OR = 1.03, 95%CI = 1.00–1.05, *P* = .0238) and 1-stearoyl-2-docosahexaenoyl-GPE (OR = 1.03, 95%CI = 1.01–1.05, *P* = .0097). Detailed information was available in Tables 3 and 4.

**Table 3:** MR analysis and sensitivity analyses for causality from upper urinary stones on 12 blood metabolites

Name	Outcome	Metabolites	Number of SNPs	MR analysis					Heterogeneity				Pleiotropy		MR-PRESSO
				Method	OR (95% CI)	Lower CI	Upper CI	P	IVW		MR Egger		Intercept	P	P
									Q	P	Q	P			
1-methyl-4-imidazoleacetate	GCST90199717	Azoles	55	IVW	1.03	0.98	1.09	0.2144	53.45	0.4954	53.27	0.4638	‐0.0031	0.6695	0.2175
Mannose	GCST90200435	Carbohydrate	55	IVW	1.06	1.00	1.13	0.0449	66.38	0.1202	66.33	0.1033	0.0018	0.8325	0.1154
1-linoleoyl-2-arachidonoyl-GPC (18:2/20:4n6)	GCST90200073	Lipid	55	IVW	0.96	0.91	1.01	0.1403	53.69	0.4864	53.69	0.4478	0.0002	0.9787	0.1449
Eicosenedioate (C20:1-DC)	GCST90200285	Lipid	55	IVW	1.02	0.96	1.08	0.5097	53.53	0.4923	51.73	0.5237	0.0101	0.1850	0.5107
1-stearoyl-2-docosahexaenoyl-GPE (18:0/22:6)	GCST90200065	Lipid	55	IVW	1.04	0.99	1.10	0.1236	51.11	0.5867	51.07	0.5495	0.0013	0.8596	0.1193
1-palmitoyl-2-oleoyl-GPE (16:0/18:1)	GCST90200331	Lipid	55	IVW	1.05	0.99	1.11	0.1123	56.21	0.3921	56.07	0.3604	0.0028	0.7197	0.1182
1-oleoyl-2-arachidonoyl-GPE (18:1/20:4)	GCST90200079	Lipid	55	IVW	1.00	0.95	1.05	0.9470	49.11	0.6629	49.11	0.6263	-0.0004	0.9565	0.9447
Adenosine 5’-monophosphate (AMP)	GCST90200356	Nucleotide	55	IVW	0.98	0.93	1.03	0.4519	55.80	0.4070	55.79	0.3704	-0.0007	0.9217	0.4552
Cysteinylglycine	GCST90200364	Peptide	55	IVW	0.98	0.93	1.03	0.4040	48.16	0.6981	47.92	0.6716	-0.0036	0.6306	0.3808
Urea	GCST90200417	Ureas	55	IVW	1.05	0.99	1.11	0.1282	63.49	0.1768	63.34	0.1563	0.0027	0.7306	0.1341
X-17654	GCST90200547	Unknown	55	IVW	0.97	0.92	1.02	0.2403	40.55	0.9123	40.54	0.8951	-0.0004	0.9547	0.1811
X-19141	GCST90200711	Unknown	55	IVW	1.00	0.94	1.05	0.8769	48.62	0.6811	48.47	0.6511	0.0029	0.6936	0.2175

CI = confidence interval, IVW = inverse variance weighted, MR = Mendelian randomization, MR-PRESSO = MR-Pleiotropy RESidual sum and outlier, OR = odds ratio, SNPs = single nucleotide polymorphisms.

**Table 4:** MR analysis and sensitivity analyses for causality from lower urinary stones on 12 blood metabolites.

Name	Outcome	Metabolites	Number of SNPs	MR analysis					Heterogeneity				Pleiotropy		MR-PRESSO
				Method	OR (95% CI)	Lower CI	Upper CI	P	IVW		MR Egger		Intercept	P	P
									Q	P	Q	P			
1-methyl-4-imidazoleacetate	GCST90199717	Azoles	11	IVW	1.01	0.98	1.03	0.5667	12.01	0.2847	8.45	0.4898	-0.0183	0.0919	0.5793
Mannose	GCST90200435	Carbohydrate	11	IVW	1.01	0.98	1.04	0.6419	20.15	0.0279	19.80	0.0192	-0.0059	0.7012	0.6519
1-oleoyl-2-arachidonoyl-GPE (18:1/20:4)	GCST90200079	Lipid	11	IVW	1.03	1.00	1.05	0.0238	6.98	0.7274	4.91	0.8423	-0.0148	0.1840	0.5178
1-linoleoyl-2-arachidonoyl-GPC (18:2/20:4n6)	GCST90200073	Lipid	11	IVW	0.99	0.97	1.02	0.5510	12.72	0.2398	12.49	0.1870	0.0048	0.6941	0.5643
1-palmitoyl-2-oleoyl-GPE (16:0/18:1)	GCST90200331	Lipid	11	IVW	1.01	0.98	1.04	0.5139	18.03	0.0544	10.29	0.3279	‐0.0284	0.0286	0.5286
1-stearoyl-2-docosahexaenoyl-GPE (18:0/22:6)	GCST90200065	Lipid	11	IVW	1.03	1.01	1.05	0.0097	6.91	0.7343	6.53	0.6863	‐0.0061	0.5534	0.5392
Eicosenedioate (C20:1-DC)	GCST90200285	Lipid	11	IVW	0.99	0.97	1.01	0.2391	7.92	0.6368	7.90	0.5443	0.0014	0.8926	0.2153
Adenosine 5’-monophosphate (AMP)	GCST90200356	Nucleotide	11	IVW	1.00	0.98	1.02	0.8203	3.32	0.9728	3.32	0.9503	0.0004	0.9704	0.7017
Cysteinylglycine	GCST90200364	Peptide	11	IVW	0.99	0.97	1.01	0.2056	5.91	0.8229	4.82	0.8499	‐0.0105	0.3236	0.1307
Urea	GCST90200417	Ureas	11	IVW	1.00	0.98	1.02	0.7455	10.01	0.4399	9.75	0.3712	0.0050	0.6372	0.7522
X-17654	GCST90200547	Unknown	11	IVW	1.00	0.98	1.02	0.8169	9.83	0.4553	9.81	0.3662	0.0016	0.8859	0.8201
X-19141	GCST90200711	Unknown	11	IVW	1.01	0.99	1.03	0.5551	8.31	0.5983	8.25	0.5096	0.0026	0.8007	0.5320

CI = confidence interval, IVW = inverse variance weighted, MR = Mendelian randomization, MR-PRESSO = MR-Pleiotropy RESidual sum and outlier, OR = odds ratio, SNPs = single nucleotide polymorphisms.

### 3.4. Metabolic pathway analysis

**Figure 4.**
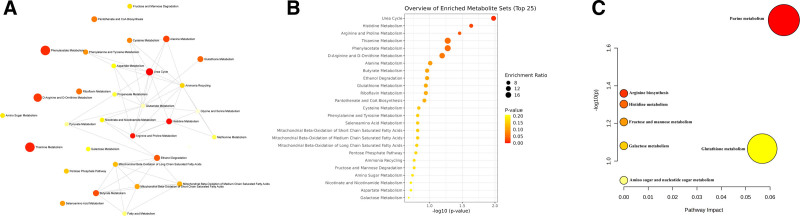
(A) Network analysis of significantly associated metabolites on upper and lower urinary stones. (B) Enrichment analysis of significantly associated metabolites on upper and lower urinary stones. (C) Pathway analysis of significantly associated metabolites on upper and lower urinary stones. The different colors indicated the level of significance, and the size of the circle reflected the level of the ratio.

Metabolites significantly associated with upper and lower urinary stones were entered into the Metabolic Analyzer 5.0 platform to determine potential metabolic pathways involved in the pathogenesis of urinary stones. The network, enrichment, and pathway analyses of interactions among the metabolic pathways involved in this study were exhibited in Fig. 4. Detailed information was displayed in Tables S8 and S9, Supplemental Digital Content, http://links.lww.com/MD/O577.

## 4. Discussion

In the current study, bidirectional MR analysis provided clues that could contribute to the search for causal effects between blood metabolites and urolithiasis. We identified 15 metabolites, of which 3 remained chemically unknown, as potential causal predictors of urinary stones in forward MR analyses. Additionally, urinary stones were found to be significantly associated with 12 metabolites. Limited research has focused on metabolic changes in urinary stones. Our research is notable for incorporating the most comprehensive blood metabolite GWAS data for bidirectional MR analysis, allowing us to explore the causal connection between blood metabolites and both upper and lower tract urinary stones.

Metabolomics is a powerful analytical technique that allows for the simultaneous quantification of a wide range of small molecule metabolites within biological systems.^[21]^ It is high-throughput and unbiased, making it extremely valuable for studying urolithiasis and uncovering the complex metabolic changes associated with this condition.^[22]^ In the forward MR analyses, we identified 15 blood metabolites that are associated with the risk of both upper and lower tract urinary stones. These metabolites consist of 1 azole, 2 carbohydrates, 6 lipids, 1 nucleotide, 1 peptide, 1 urea, and 3 metabolites with unknown chemical properties. However, 3 metabolites were excluded from the reverse MR analyses due to their contradictory effects on upper and lower tract urinary stones. The results of the reverse MR analyses revealed that urolithiasis can indeed impact the expression of some metabolites in the blood, suggesting a potential interaction between blood metabolites and urinary stones. These findings contribute to a more comprehensive understanding of the role of these blood metabolites in urinary stones and their associated health outcomes.

We observed that lipids accounted for nearly half of the metabolites in our study. It has been established that disruptions in lipid metabolism are linked to the development and progression of various diseases that impact multiple bodily systems.^[23–25]^ In recent years, there has been a growing interest in the connection between metabolic disorders and the occurrence of urinary stones, suggesting a link between circulating lipids and urolithiasis.^[26–28]^ In a study conducted by Tan et al,^[29]^ a causal relationship was discovered between an elevated risk of urolithiasis and increased serum triglyceride levels. Membrane lipids play a critical role in the development of kidney stones, particularly those composed of calcium oxalate (CaOx) and calcium phosphate. Individuals who form CaOx and uric acid stones show considerably elevated levels of cholesterol, cholesterol ester, and triglycerides in their urine compared to healthy individuals, and the urine of CaOx stone formers contains higher levels of acidic phospholipids.^[30]^ Our results indicated that 4 acidic phospholipids in the blood, including 1-linoleoyl-2-arachidonoyl-GPC, 1-stearoyl-2-docosahexaenoyl-GPE, 1-palmitoyl-2-oleoyl-GPE, and 1-oleoyl-2-arachidonoyl-GPE increases the likelihood of developing upper and lower urinary tract stones, which is in line with previous studies. In reverse MR analysis, we also observed an increase in the expression of 1-oleoyl-2-arachidonoyl-GPE and 1-stearoyl-2-docosahexaenoyl-GPE in blood with lower urinary tract stones. This finding suggests a mutual relationship between metabolites and urinary stones.

Octadecenedioate, a long-chain fatty acid, has been potentially associated with a decreased risk of coronary heart disease.^[31]^ In our study, we found that octadecenedioate also reduced the risk of stones in both upper and lower urinary tract. Research on the relationship between lipid metabolites and urolithiasis is currently limited, and further discussion is still required.

Several other metabolites were also identified as causal risk factors in urolithiasis in our study. 1-methyl-4-imidazole acetate is the primary metabolite of histamine and can be utilized to estimate the production and release of histamine.^[32]^ Mannose is a carbohydrate that plays a role in the formation of Tamm-Horsfall glycoprotein. It has the ability to prevent the aggregation of calcium oxalate and calcium phosphate crystals, ultimately helping to prevent the formation of kidney stones.^[33]^ Upper urinary tract stones seem to cause an increase in the level of Mannose in the bloodstream, as indicated by our reverse MR analysis. N-acetylglucosamine/N-acetylgalactosamine is a sugar residue commonly found in living organisms. It plays a vital role as a component of various essential polysaccharides and sugar complexes.^[34,35]^ Metformin contributes to the alleviation of urolithiasis by indirectly activating 5′ adenosine monophosphate-activated protein kinase.^[36]^ Cysteinylglycine is a prooxidant that has been shown to cause oxidative damage to DNA bases.^[37]^ Higher levels of cysteinylglycine may indicate an increased risk of developing breast cancer in women who are exposed to environments that are prone to peroxidation.^[38]^ Urea is the final product of protein metabolism in the body. Previous studies have not revealed any evidence to support the claim that a long-term high protein intake leads to kidney stones.^[39]^ However, we do have data that indicates that elevated levels of urea in the blood may potentially contribute to the formation of urolithiasis. Although few studies have previously explored the relationship between the above metabolites and urinary stones, our findings present a novel approach to investigating the causes of urolithiasis.

However, it is important to acknowledge several limitations in this investigation. Firstly, all participants we obtained were exclusively from European populations, which may restrict the generalization of our conclusions to other ethnic backgrounds. Secondly, although we included 1091 metabolites in our MR study through rigorous selection, we still have 220 metabolites that remain chemically unknown. We still include 3 unknown metabolites in our findings. Thirdly, our analysis did not differentiate between the composition of stones, which is an important factor that could potentially influence the associations between blood metabolites and urolithiasis. Therefore, further investigation is necessary to explore this aspect.

## 5. Conclusion

This MR study has established a causal relationship between 15 blood metabolites and the risk of upper and lower urinary tract stones. Furthermore, it has been found that urolithiasis affects the generation of 12 of these metabolites. The identification of these blood metabolites provides valuable insights into early screening, prevention, and treatment of urolithiasis.

## Author contributions

**Conceptualization:** Haoyang Zhang, Haojie Mo, Jiale Sun.

**Data curation:** Haoyang Zhang, Haojie Mo, Peng Li, Jiale Sun.

**Formal analysis:** Haoyang Zhang, Haojie Mo, Peng Li.

**Funding acquisition:** Gang Shen.

**Investigation:** Haoyang Zhang, Jiale Sun.

**Methodology:** Haoyang Zhang, Jiale Sun.

**Project administration:** Haoyang Zhang, Haojie Mo, Jiale Sun.

**Resources:** Haoyang Zhang, Haojie Mo.

**Software:** Haoyang Zhang, Haojie Mo, Peng Li, Qi Zhou.

**Supervision:** Haoyang Zhang, Haojie Mo, Peng Li, Qi Zhou.

**Validation:** Haoyang Zhang, Haojie Mo, Qi Zhou.

**Visualization:** Haoyang Zhang, Haojie Mo, Qi Zhou.

**Writing – original draft:** Haoyang Zhang, Haojie Mo, Jiale Sun.

**Writing – review & editing:** Haoyang Zhang, Haojie Mo, Jiale Sun.

## Supplementary Material

SUPPLEMENTARY MATERIAL
