# Supplementary material for: Identification of the relationship between 1400 blood metabolites and urolithiasis: A bidirectional Mendelian randomization study
Source: Medicine (Baltimore). 2025 Mar 21;104(12):e41911. doi: 10.1097/MD.0000000000041911 (PMC11936622; doi:10.1097/MD.0000000000041911)
Supplement: SUPPLEMENTARY MATERIAL [file medi-104-e41911-s006.pdf]

Supporting information

Figure S1. Forest plot of causal associations between 98 blood metabolites and upper urinary stones using MR - Egger method.
Figure S2. Funnel plots were applied to detect whether the observed association between 98 blood metabolites and upper urinary stones was along with obvious heterogeneity using IVW and MR-Egger methods. IVW, inverse variance weighted.
Figure S3. Scatter plots of causal associations between 98 blood metabolites and upper urinary stones. The five methods applied in this study were all demonstrated. Lines in different colors represent IVW, MR-Egger, weighted median, simple mode and weight mode methods. IVW, inverse variance weighted; SNP, single nucleotide polymorphism.
Figure S4. Leave-one-out analyses were applied to evaluate whether any single instrumental variable was driving the causal effect between 98 blood metabolites and upper urinary stones.
Figure S5. Forest plot of causal associations between 86 blood metabolites and lower urinary stones using MR - Egger method.
Figure S6. Funnel plots were applied to detect whether the observed association between 86 blood metabolites and lower urinary stones was along with obvious heterogeneity using IVW and MR-Egger methods. IVW, inverse variance weighted.
Figure S7. Scatter plots of causal associations between 86 blood metabolites and lower urinary stones. The five methods applied in this study were all demonstrated. Lines in different colors represent IVW, MR-Egger, weighted median, simple mode and weight mode methods. IVW, inverse variance weighted; SNP, single nucleotide polymorphism.
Figure S8. Leave-one-out analyses were applied to evaluate whether any single instrumental variable was driving the causal effect between 86 blood metabolites and lower urinary stones.

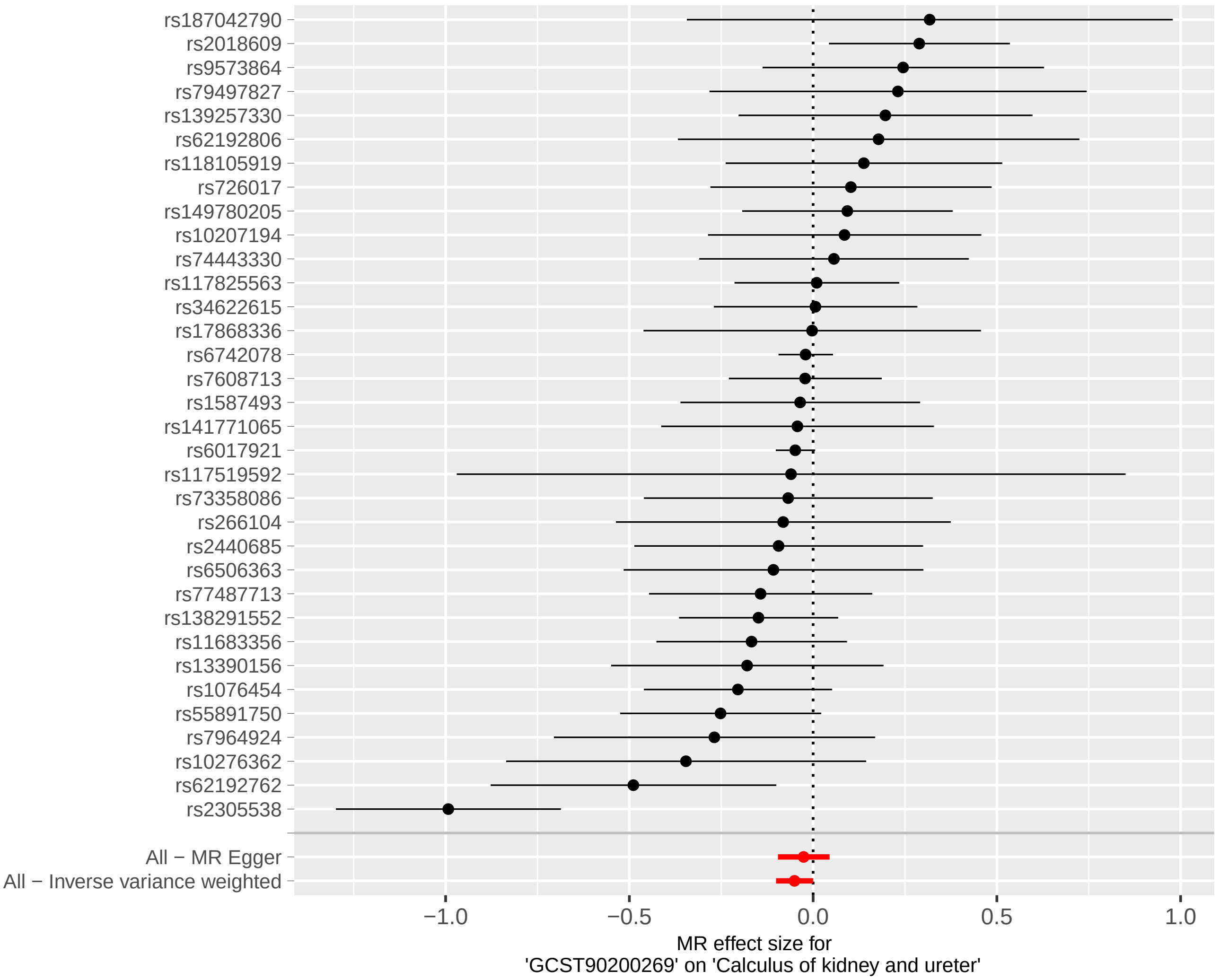
rs187042790
rs2018609
rs9573864
rs79497827
rs139257330
rs62192806
rs118105919
rs726017
rs149780205
rs10207194
rs74443330
rs117825563
rs34622615
rs17868336
rs6742078
rs7608713
rs1587493
rs141771065
rs6017921
rs117519592
rs73358086
rs266104
rs2440685
rs6506363
rs77487713
rs138291552
rs11683356
rs13390156
rs1076454
rs55891750
rs7964924
rs10276362
rs62192762
rs2305538
All − MR Egger
All − Inverse variance weighted
−1.0
−0.5
0.0
0.5
1.0
MR effect size for
'GCST90200269' on 'Calculus of kidney and ureter'

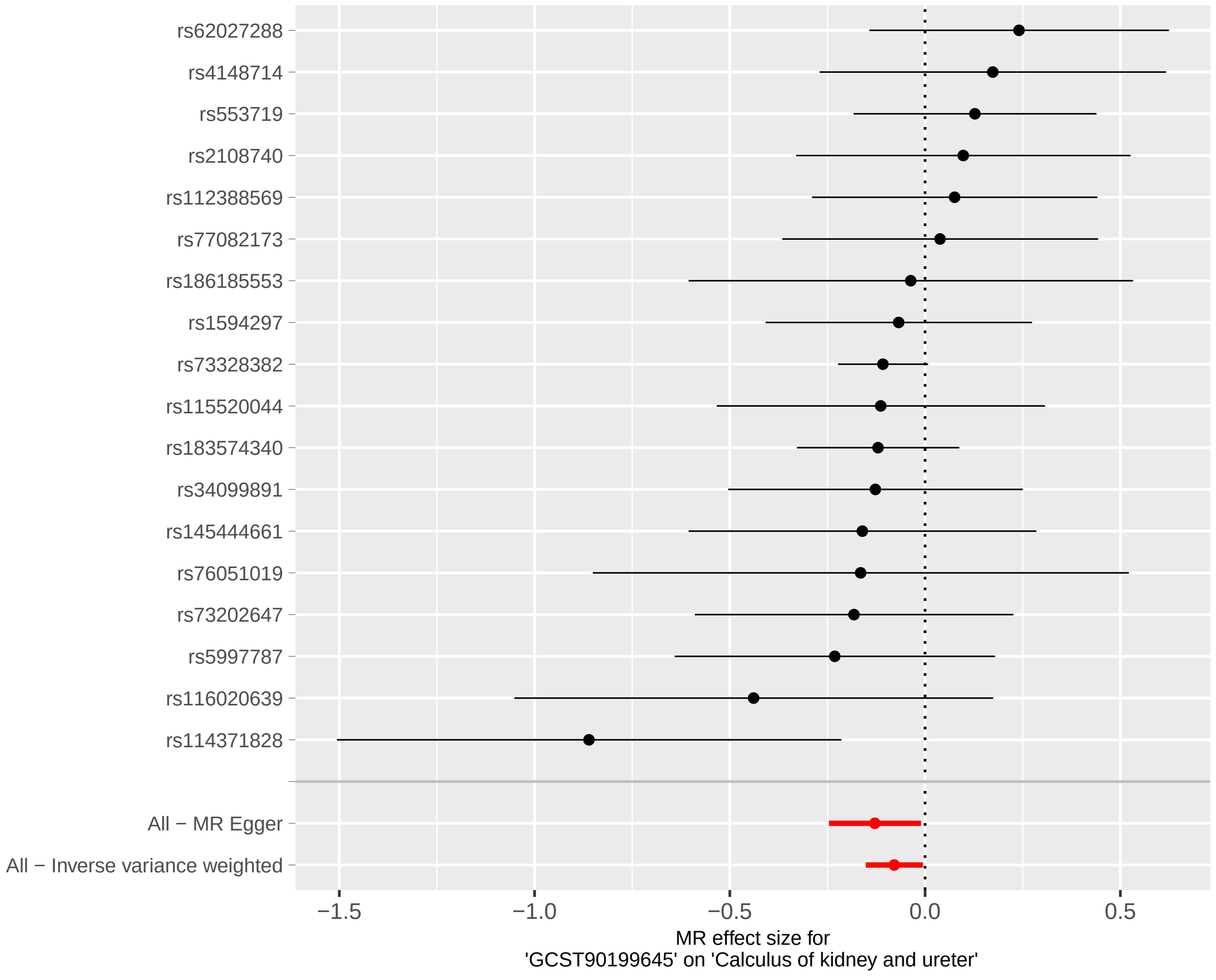
rs62027288
rs4148714
rs553719
rs2108740
rs112388569
rs77082173
rs186185553
rs1594297
rs73328382
rs115520044
rs183574340
rs34099891
rs145444661
rs76051019
rs73202647
rs5997787
rs116020639
rs114371828
All − MR Egger
All − Inverse variance weighted
−1.5
−1.0
−0.5
0.0
0.5
MR effect size for
'GCST90199645' on 'Calculus of kidney and ureter'

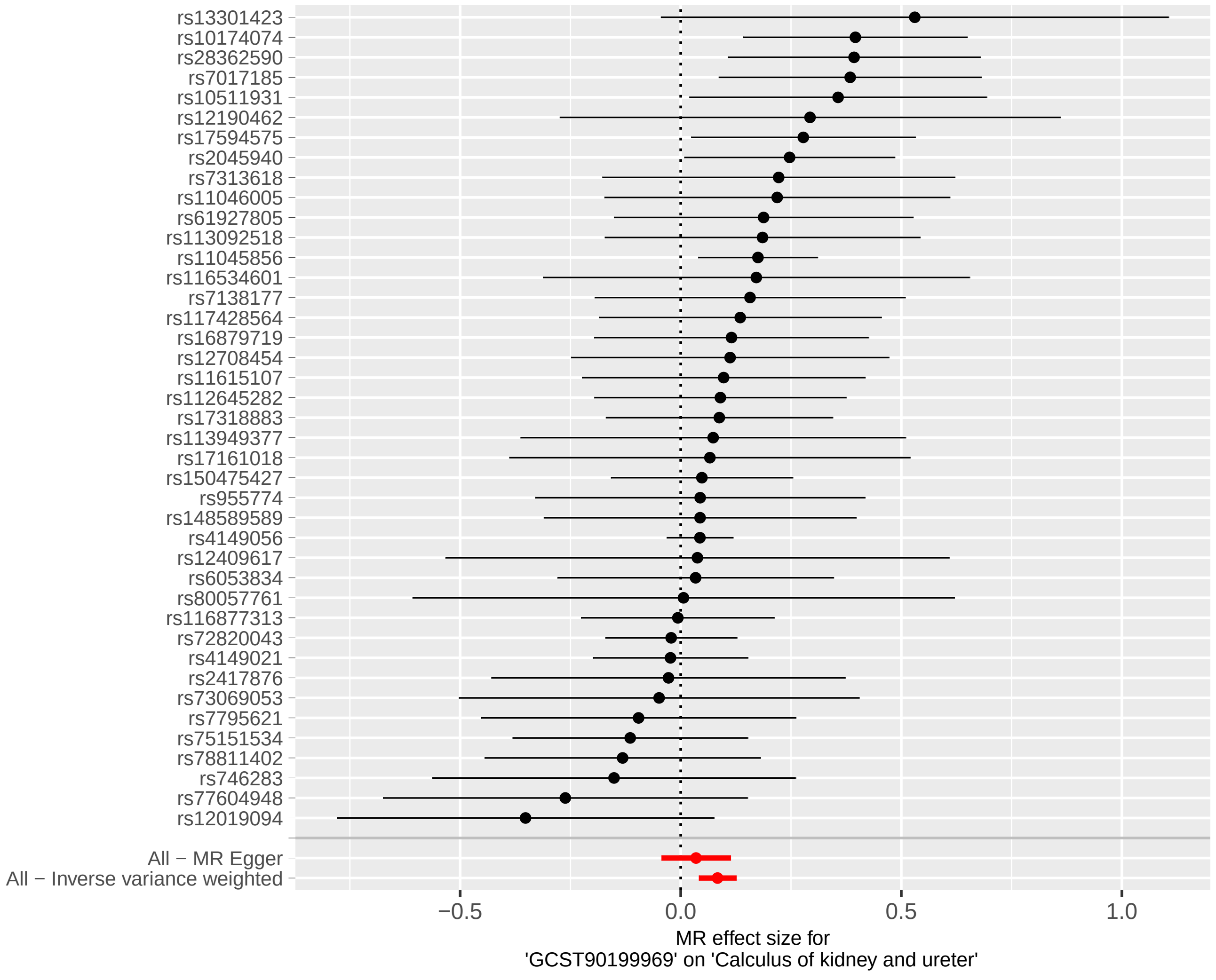

rs13301423
rs10174074
rs28362590
rs7017185
rs10511931
rs12190462
rs17594575
rs2045940
rs7313618
rs11046005
rs61927805
rs113092518
rs11045856
rs116534601
rs7138177
rs117428564
rs16879719
rs12708454
rs11615107
rs112645282
rs17318883
rs113949377
rs17161018
rs150475427
rs955774
rs148589589
rs4149056
rs12409617
rs6053834
rs80057761
rs116877313
rs72820043
rs4149021
rs2417876
rs73069053
rs7795621
rs75151534
rs78811402
rs746283
rs77604948
rs12019094
All − MR Egger
All − Inverse variance weighted
−0.5
0.0
0.5
1.0
MR effect size for
'GCST90199969' on 'Calculus of kidney and ureter'

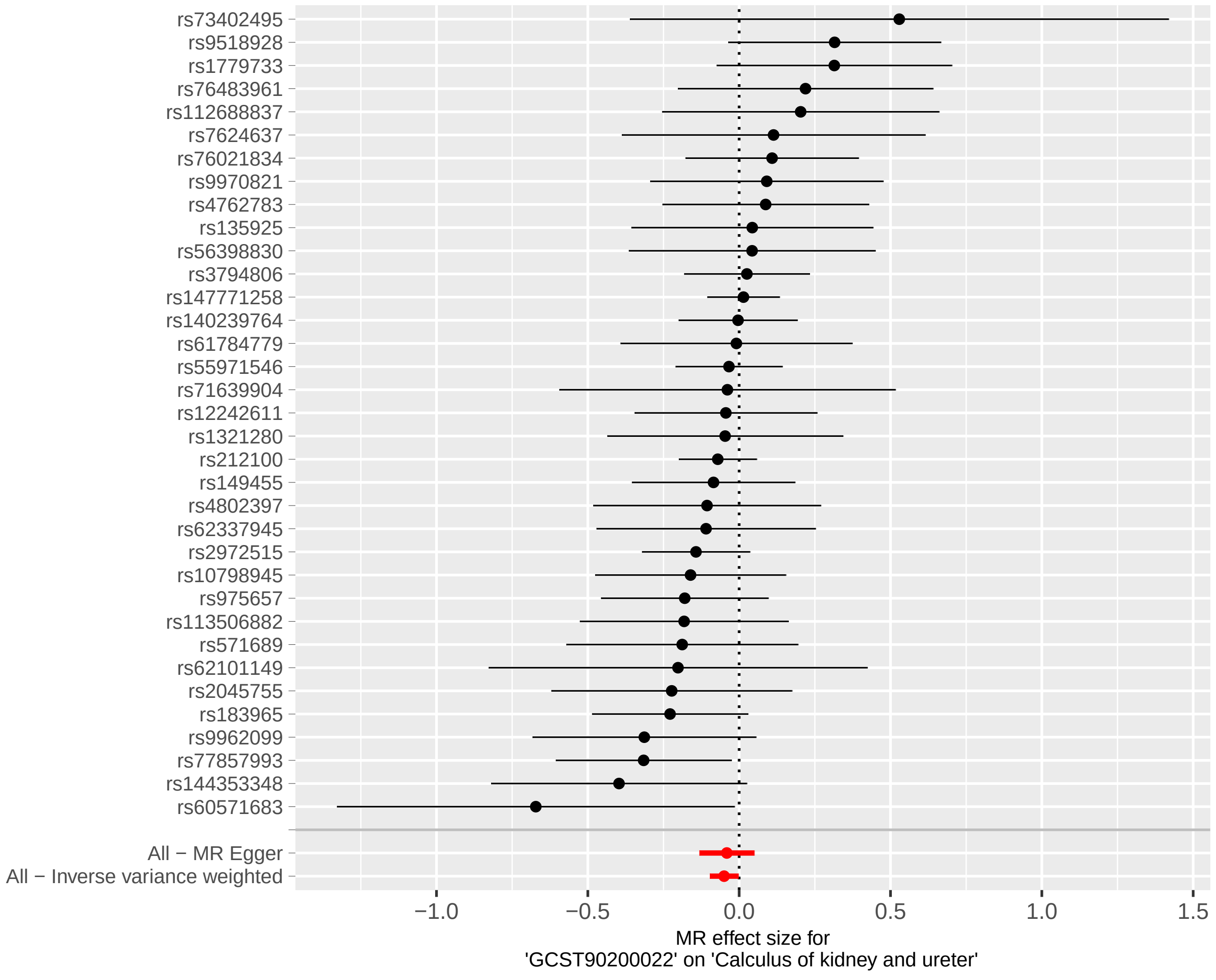
rs73402495
rs9518928
rs1779733
rs76483961
rs112688837
rs7624637
rs76021834
rs9970821
rs4762783
rs135925
rs56398830
rs3794806
rs147771258
rs140239764
rs61784779
rs55971546
rs71639904
rs12242611
rs1321280
rs212100
rs149455
rs4802397
rs62337945
rs2972515
rs10798945
rs975657
rs113506882
rs571689
rs62101149
rs2045755
rs183965
rs9962099
rs77857993
rs144353348
rs60571683
All − MR Egger
All − Inverse variance weighted
−1.0
−0.5
0.0
0.5
1.0
1.5
MR effect size for
'GCST90200022' on 'Calculus of kidney and ureter'

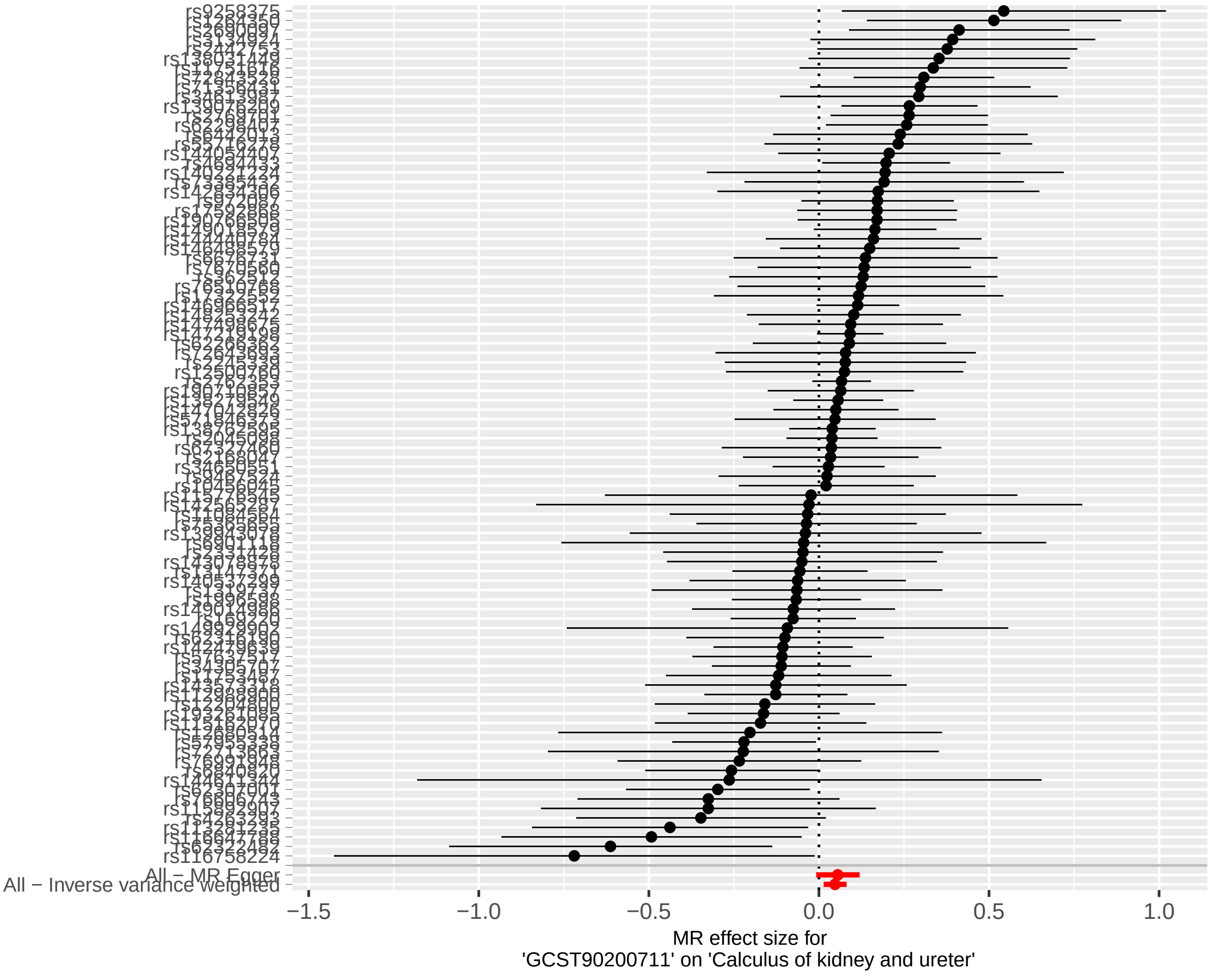
rs9258375
rs116758224
All − MR Egger
All − Inverse variance weighted
−1.5
−1.0
−0.5
0.0
0.5
1.0
MR effect size for
'GCST90200711' on 'Calculus of kidney and ureter'

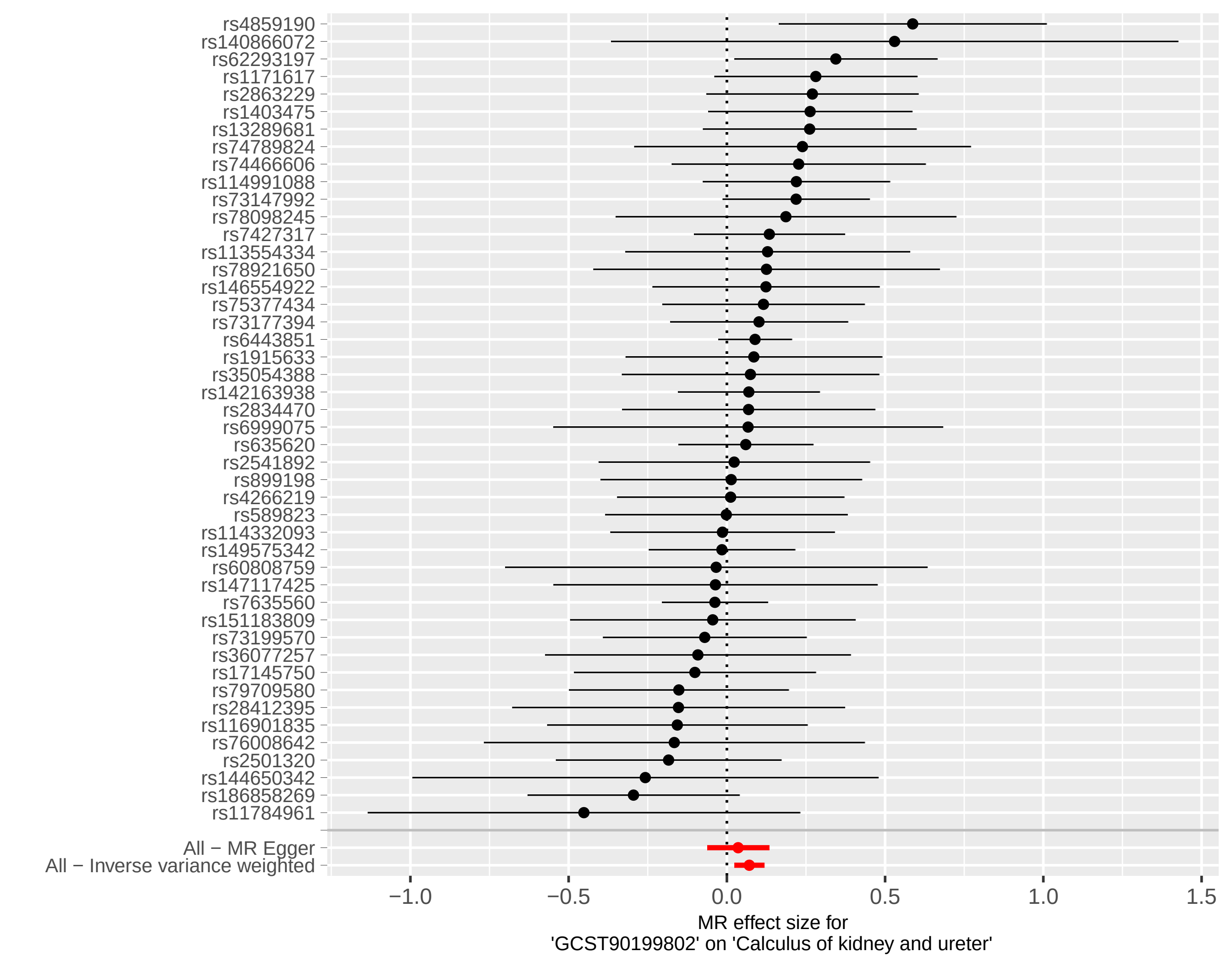
rs4859190
rs140866072
rs62293197
rs1171617
rs2863229
rs1403475
rs13289681
rs74789824
rs74466606
rs114991088
rs73147992
rs78098245
rs7427317
rs113554334
rs78921650
rs146554922
rs75377434
rs73177394
rs6443851
rs1915633
rs35054388
rs142163938
rs2834470
rs6999075
rs635620
rs2541892
rs899198
rs4266219
rs589823
rs114332093
rs149575342
rs60808759
rs147117425
rs7635560
rs151183809
rs73199570
rs36077257
rs17145750
rs79709580
rs28412395
rs116901835
rs76008642
rs2501320
rs144650342
rs186858269
rs11784961
All − MR Egger
All − Inverse variance weighted
−1.0
−0.5
0.0
0.5
1.0
1.5
MR effect size for
'GCST90199802' on 'Calculus of kidney and ureter'

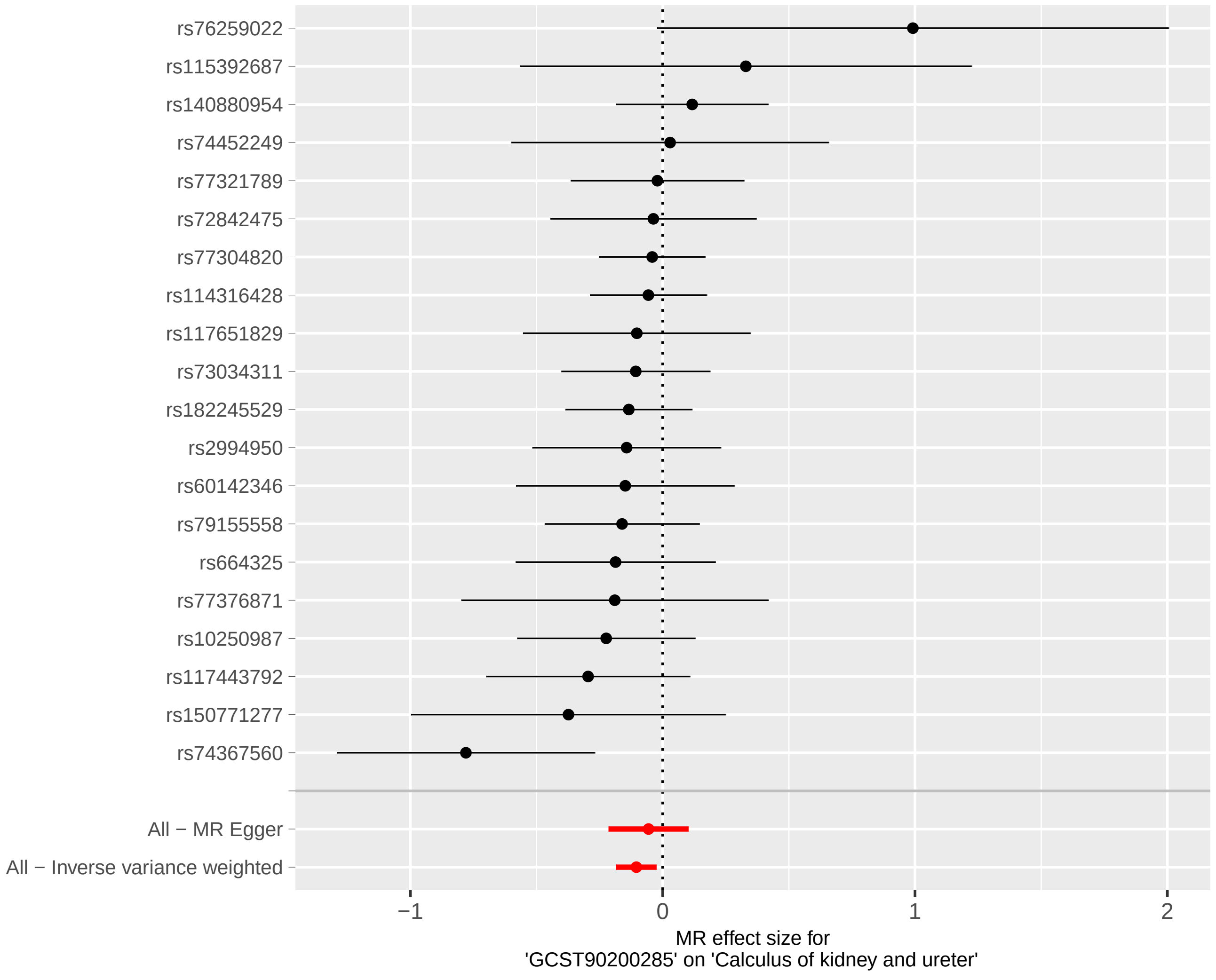

rs76259022
rs115392687
rs140880954
rs74452249
rs77321789
rs72842475
rs77304820
rs114316428
rs117651829
rs73034311
rs182245529
rs2994950
rs60142346
rs79155558
rs664325
rs77376871
rs10250987
rs117443792
rs150771277
rs74367560
All − MR Egger
All − Inverse variance weighted
−1
0
1
2
MR effect size for
'GCST90200285' on 'Calculus of kidney and ureter'

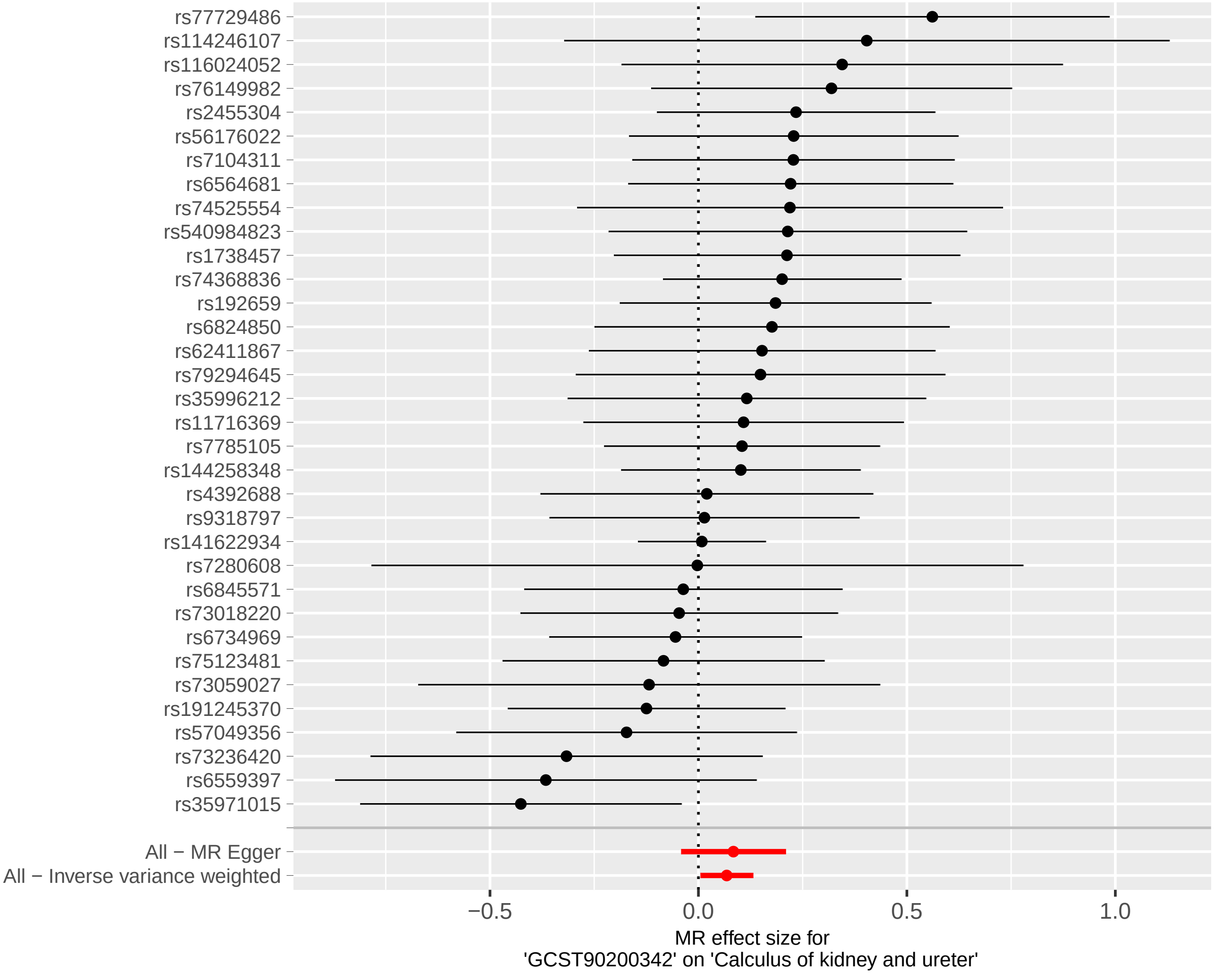

rs77729486
rs114246107
rs116024052
rs76149982
rs2455304
rs56176022
rs7104311
rs6564681
rs74525554
rs540984823
rs1738457
rs74368836
rs192659
rs6824850
rs62411867
rs79294645
rs35996212
rs11716369
rs7785105
rs144258348
rs4392688
rs9318797
rs141622934
rs7280608
rs6845571
rs73018220
rs6734969
rs75123481
rs73059027
rs191245370
rs57049356
rs73236420
rs6559397
rs35971015
All − MR Egger
All − Inverse variance weighted
−0.5
0.0
0.5
1.0
MR effect size for
'GCST90200342' on 'Calculus of kidney and ureter'

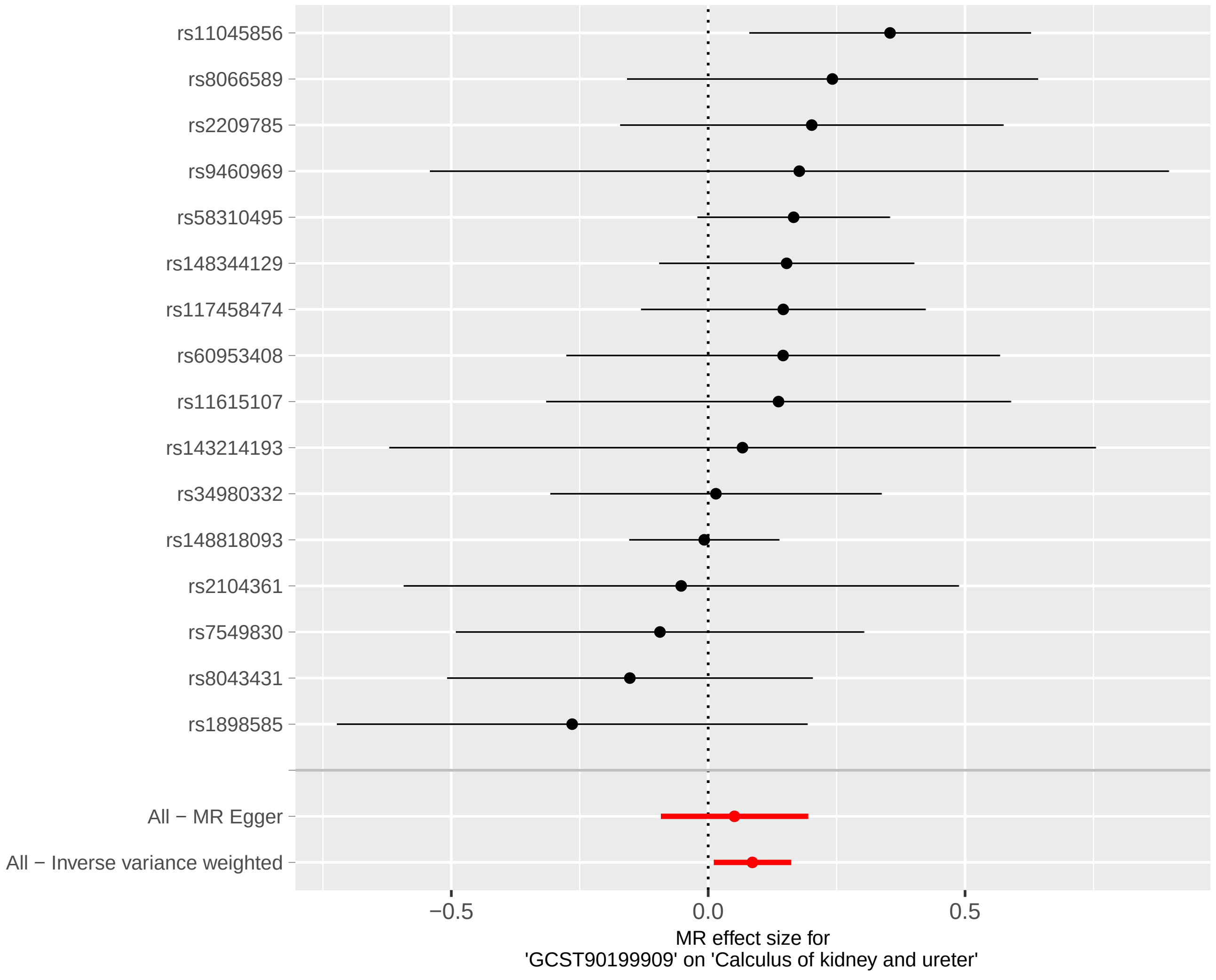
rs11045856
rs8066589
rs2209785
rs9460969
rs58310495
rs148344129
rs117458474
rs60953408
rs11615107
rs143214193
rs34980332
rs148818093
rs2104361
rs7549830
rs8043431
rs1898585
All − MR Egger
All − Inverse variance weighted
−0.5
0.0
0.5
MR effect size for
'GCST90199909' on 'Calculus of kidney and ureter'

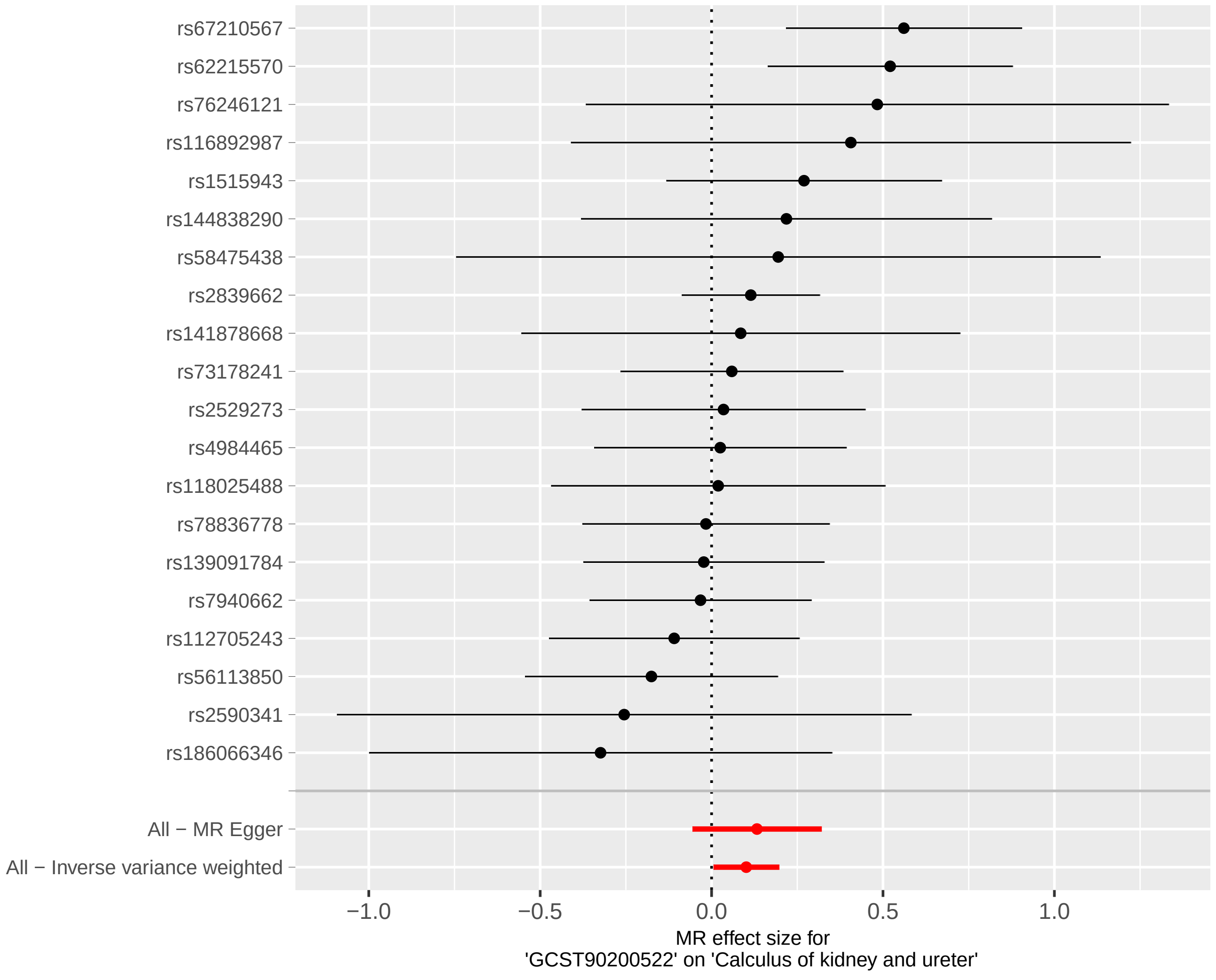

rs67210567
rs62215570
rs76246121
rs116892987
rs1515943
rs144838290
rs58475438
rs2839662
rs141878668
rs73178241
rs2529273
rs4984465
rs118025488
rs78836778
rs139091784
rs7940662
rs112705243
rs56113850
rs2590341
rs186066346
All − MR Egger
All − Inverse variance weighted
−1.0
−0.5
0.0
0.5
1.0
MR effect size for
'GCST90200522' on 'Calculus of kidney and ureter'

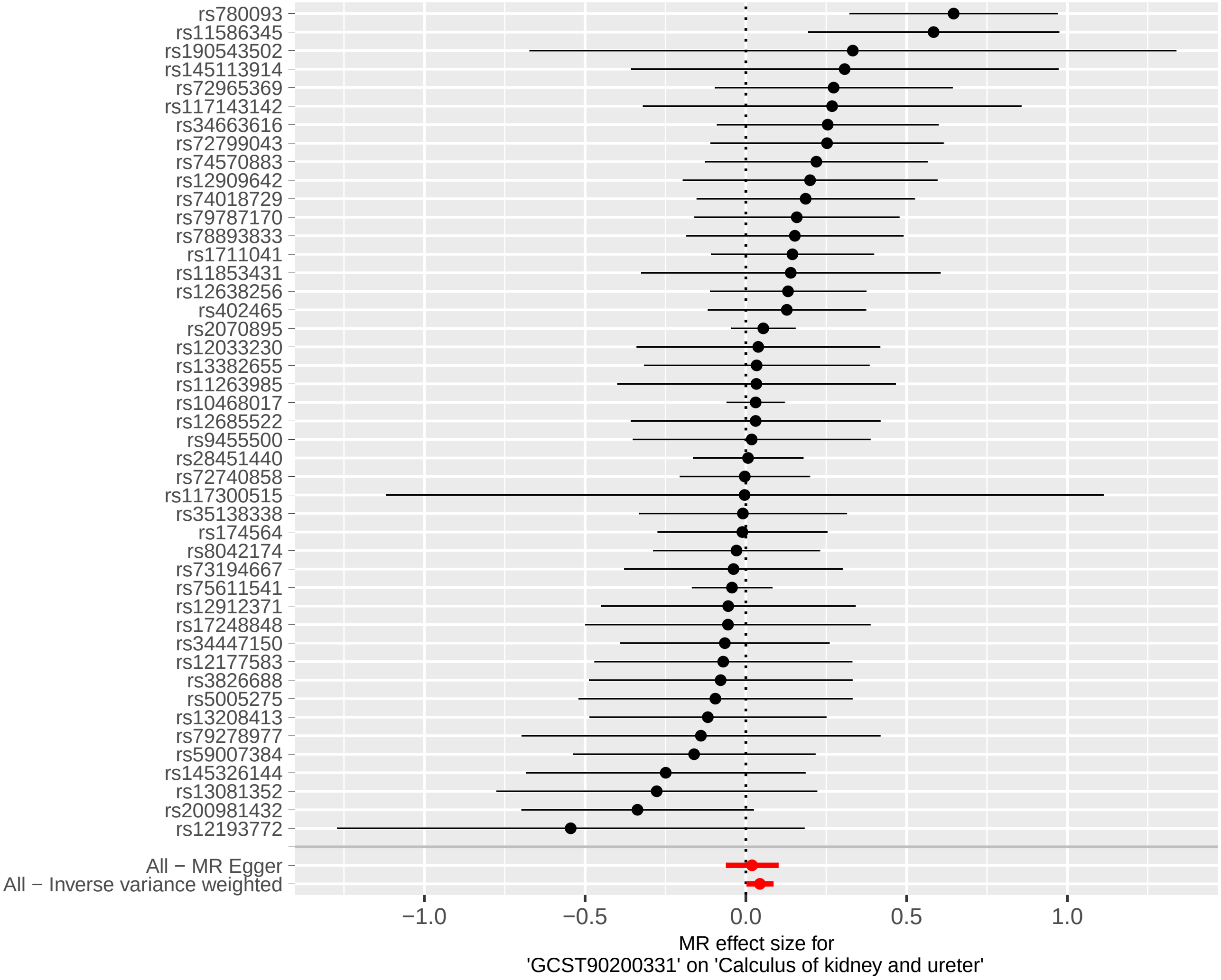
rs780093
rs11586345
rs190543502
rs145113914
rs72965369
rs117143142
rs34663616
rs72799043
rs74570883
rs12909642
rs74018729
rs79787170
rs78893833
rs1711041
rs11853431
rs12638256
rs402465
rs2070895
rs12033230
rs13382655
rs11263985
rs10468017
rs12685522
rs9455500
rs28451440
rs72740858
rs117300515
rs35138338
rs174564
rs8042174
rs73194667
rs75611541
rs12912371
rs17248848
rs34447150
rs12177583
rs3826688
rs5005275
rs13208413
rs79278977
rs59007384
rs145326144
rs13081352
rs200981432
rs12193772
All − MR Egger
All − Inverse variance weighted
−1.0
−0.5
0.0
0.5
1.0
MR effect size for
'GCST90200331' on 'Calculus of kidney and ureter'

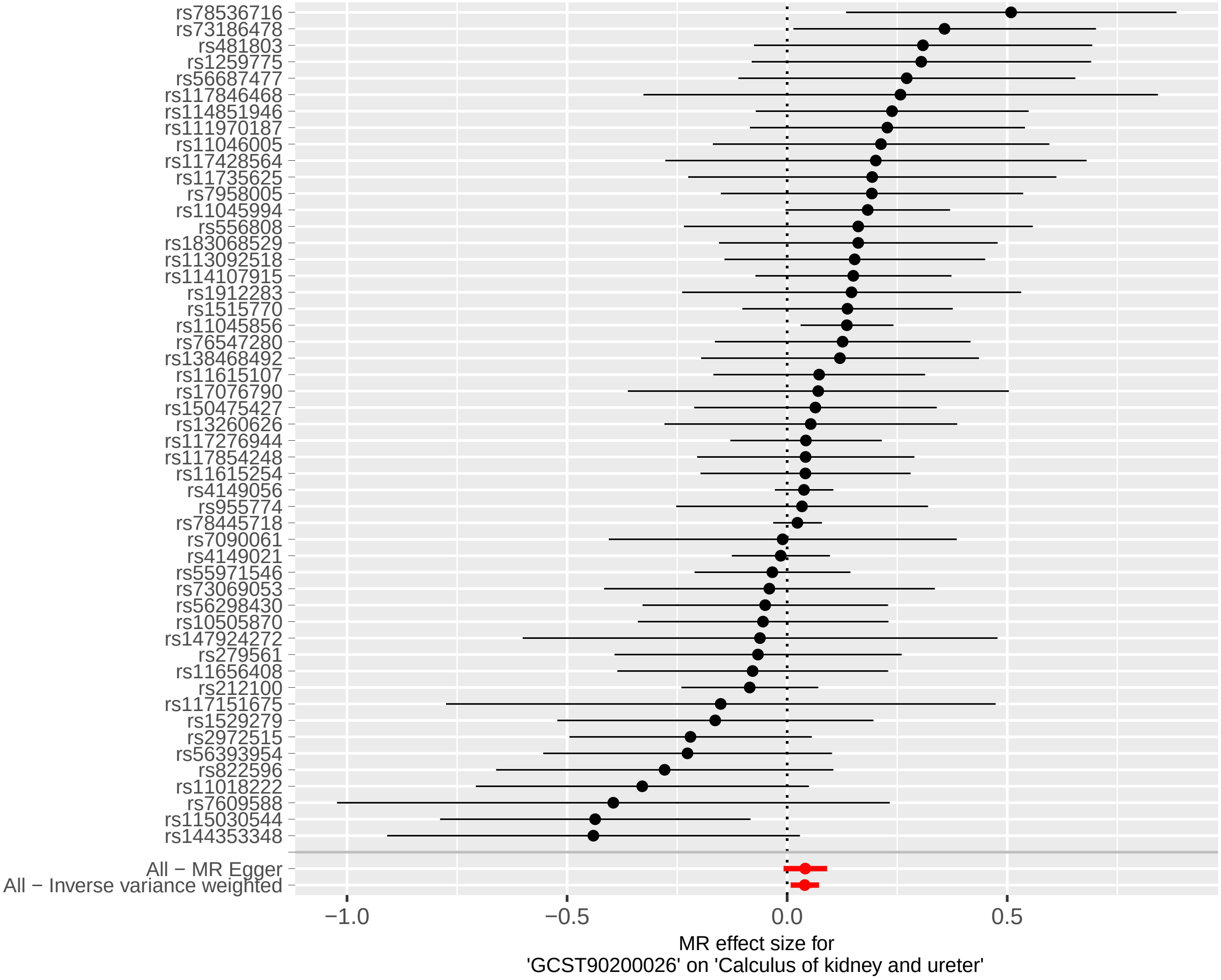
rs78536716
rs73186478
rs481803
rs1259775
rs56687477
rs117846468
rs114851946
rs111970187
rs11046005
rs117428564
rs11735625
rs7958005
rs11045994
rs556808
rs183068529
rs113092518
rs114107915
rs1912283
rs1515770
rs11045856
rs76547280
rs138468492
rs11615107
rs17076790
rs150475427
rs13260626
rs117276944
rs117854248
rs11615254
rs4149056
rs955774
rs78445718
rs7090061
rs4149021
rs55971546
rs73069053
rs56298430
rs10505870
rs147924272
rs279561
rs11656408
rs212100
rs117151675
rs1529279
rs2972515
rs56393954
rs822596
rs11018222
rs7609588
rs115030544
rs144353348
All − MR Egger
All − Inverse variance weighted
−1.0
−0.5
0.0
0.5
MR effect size for
'GCST90200026' on 'Calculus of kidney and ureter'

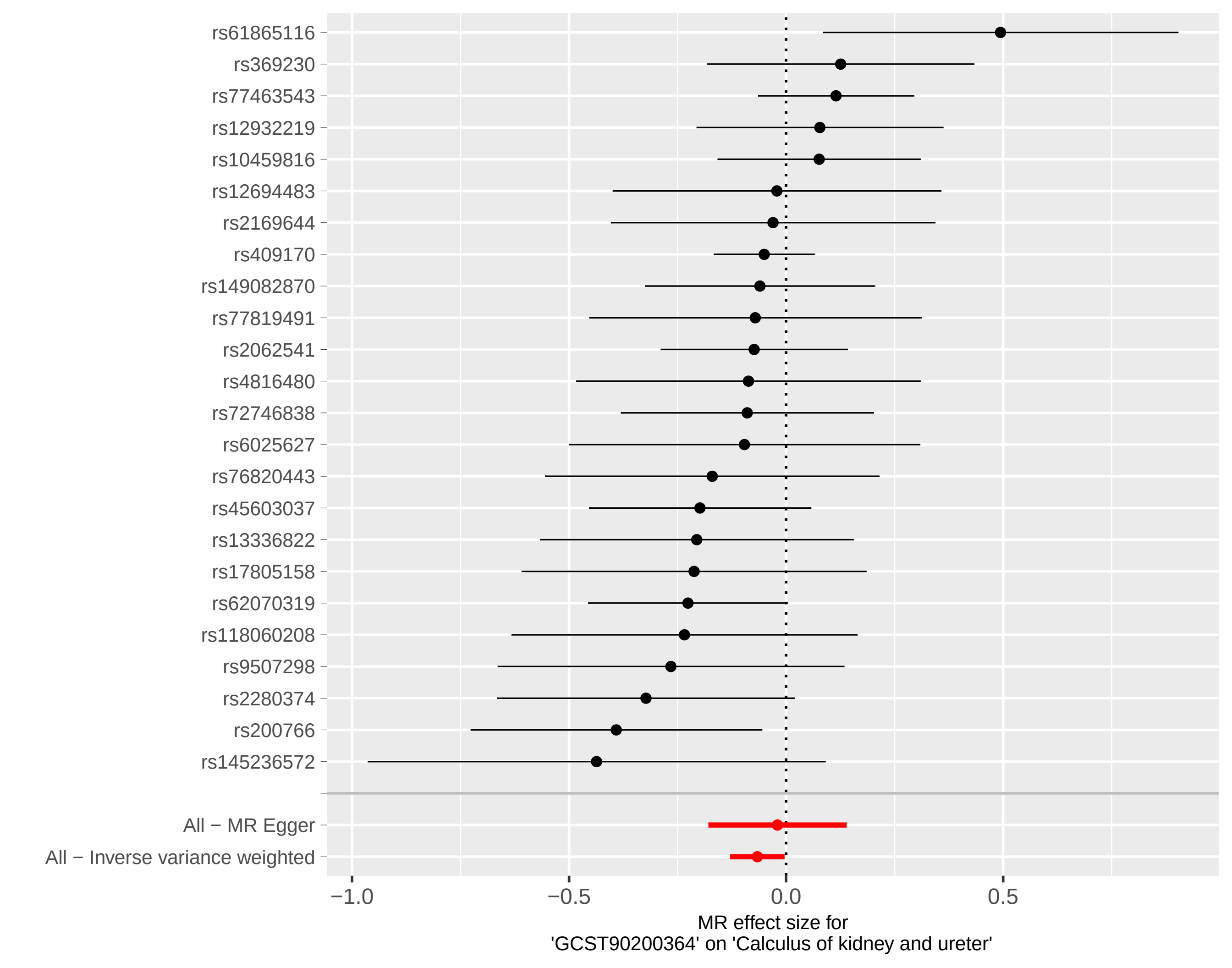
rs61865116
rs369230
rs77463543
rs12932219
rs10459816
rs12694483
rs2169644
rs409170
rs149082870
rs77819491
rs2062541
rs4816480
rs72746838
rs6025627
rs76820443
rs45603037
rs13336822
rs17805158
rs62070319
rs118060208
rs9507298
rs2280374
rs200766
rs145236572
All − MR Egger
All − Inverse variance weighted
−1.0
−0.5
0.0
0.5
MR effect size for
'GCST90200364' on 'Calculus of kidney and ureter'

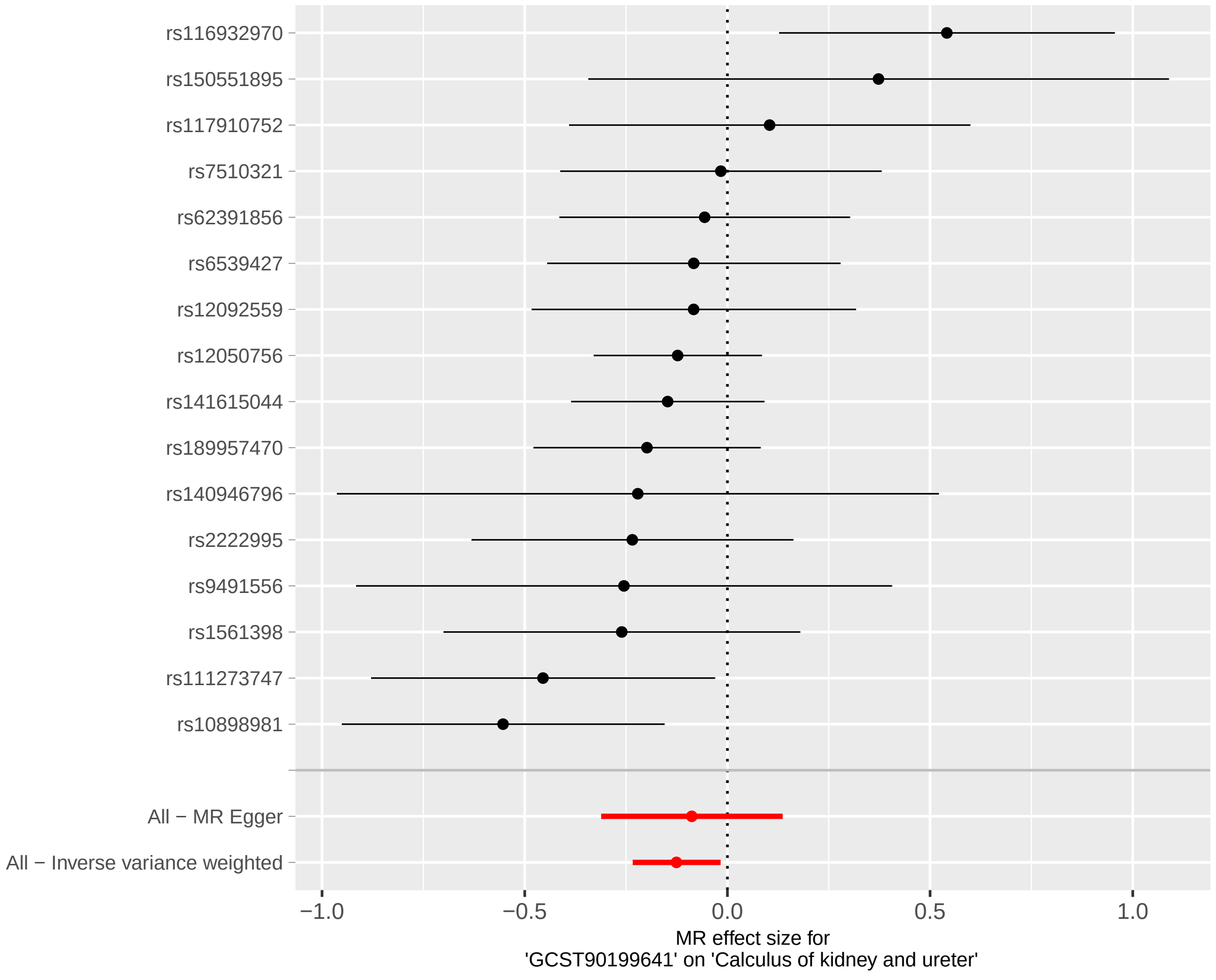

rs116932970
rs150551895
rs117910752
rs7510321
rs62391856
rs6539427
rs12092559
rs12050756
rs141615044
rs189957470
rs140946796
rs2222995
rs9491556
rs1561398
rs111273747
rs10898981
All − MR Egger
All − Inverse variance weighted
−1.0
−0.5
0.0
0.5
1.0
MR effect size for
'GCST90199641' on 'Calculus of kidney and ureter'

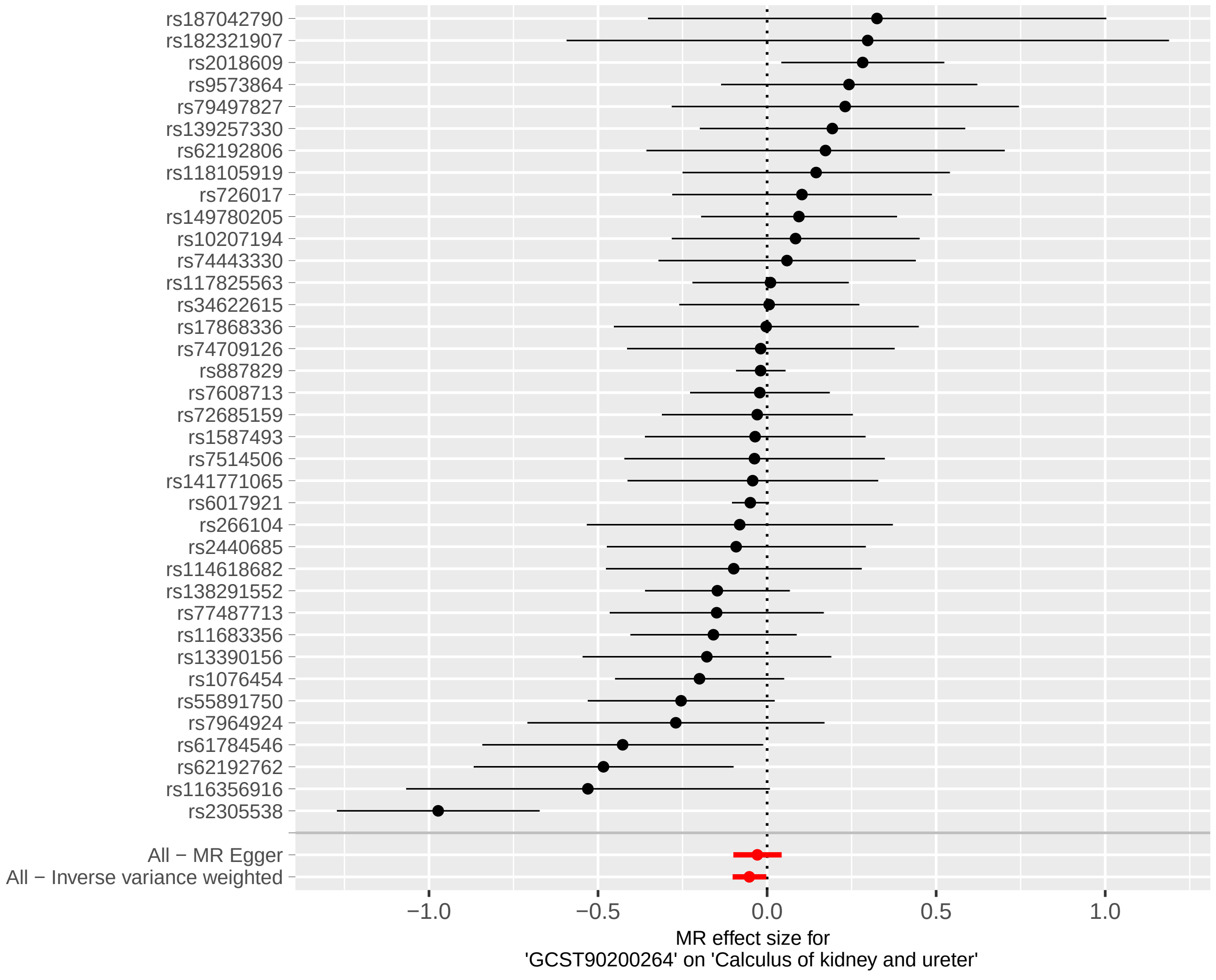
rs187042790
rs182321907
rs2018609
rs9573864
rs79497827
rs139257330
rs62192806
rs118105919
rs726017
rs149780205
rs10207194
rs74443330
rs117825563
rs34622615
rs17868336
rs74709126
rs887829
rs7608713
rs72685159
rs1587493
rs7514506
rs141771065
rs6017921
rs266104
rs2440685
rs114618682
rs138291552
rs77487713
rs11683356
rs13390156
rs1076454
rs55891750
rs7964924
rs61784546
rs62192762
rs116356916
rs2305538
All − MR Egger
All − Inverse variance weighted
−1.0
−0.5
0.0
0.5
1.0
MR effect size for
'GCST90200264' on 'Calculus of kidney and ureter'

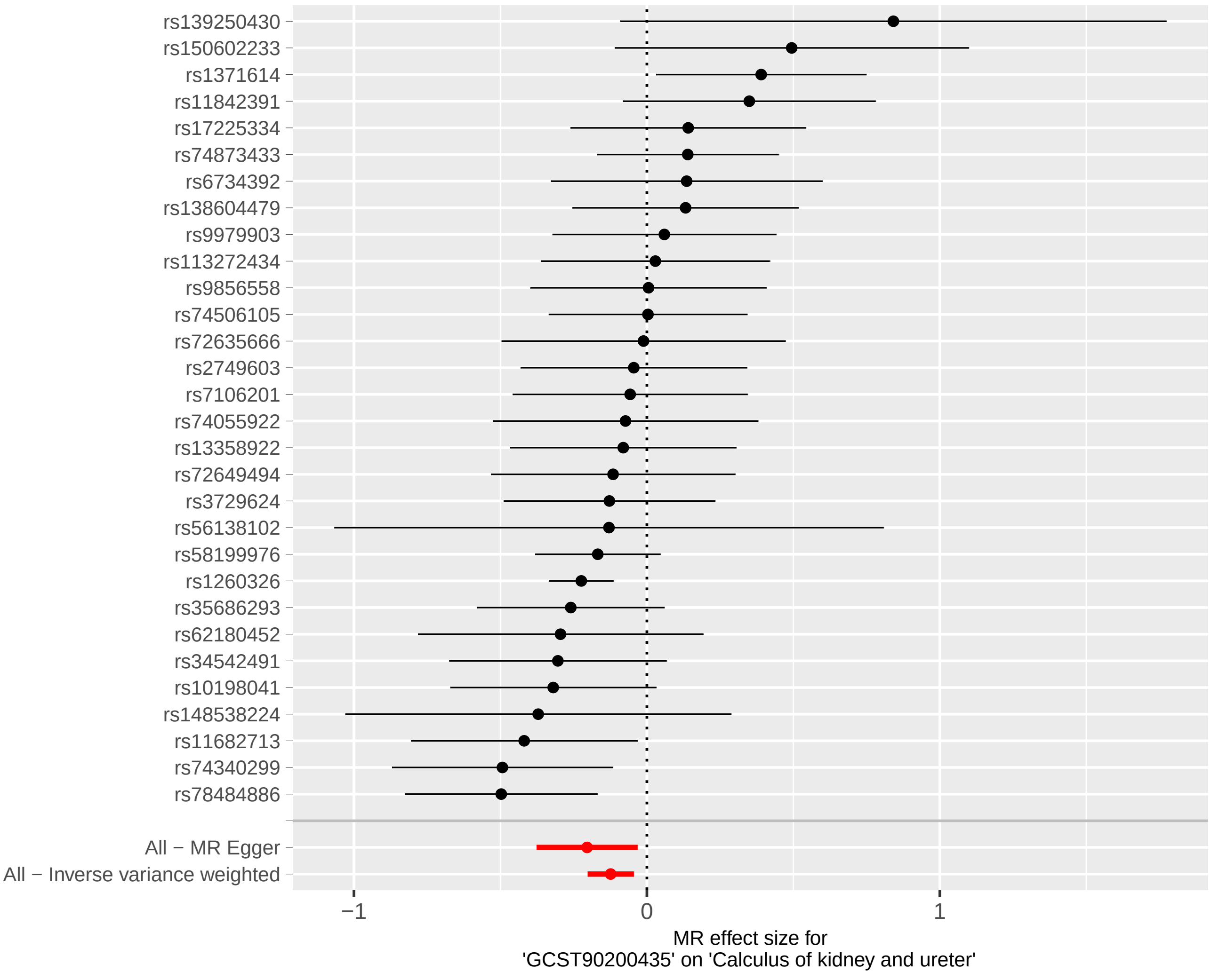
rs139250430
rs150602233
rs1371614
rs11842391
rs17225334
rs74873433
rs6734392
rs138604479
rs9979903
rs113272434
rs9856558
rs74506105
rs72635666
rs2749603
rs7106201
rs74055922
rs13358922
rs72649494
rs3729624
rs56138102
rs58199976
rs1260326
rs35686293
rs62180452
rs34542491
rs10198041
rs148538224
rs11682713
rs74340299
rs78484886
All − MR Egger
All − Inverse variance weighted
−1
0
1
MR effect size for
'GCST90200435' on 'Calculus of kidney and ureter'

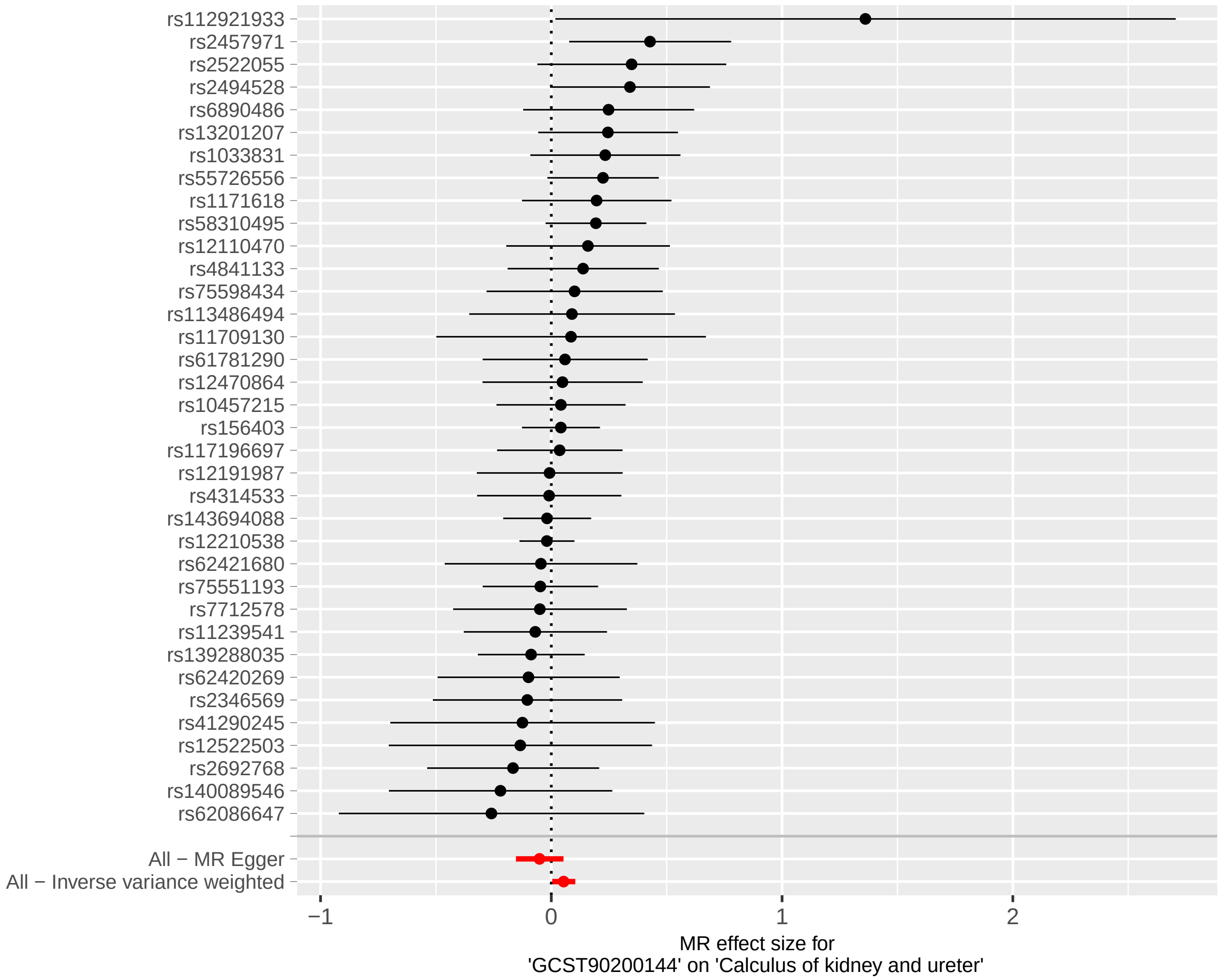
rs112921933
rs2457971
rs2522055
rs2494528
rs6890486
rs13201207
rs1033831
rs55726556
rs1171618
rs58310495
rs12110470
rs4841133
rs75598434
rs113486494
rs11709130
rs61781290
rs12470864
rs10457215
rs156403
rs117196697
rs12191987
rs4314533
rs143694088
rs12210538
rs62421680
rs75551193
rs7712578
rs11239541
rs139288035
rs62420269
rs2346569
rs41290245
rs12522503
rs2692768
rs140089546
rs62086647
All − MR Egger
All − Inverse variance weighted
−1
0
1
2
MR effect size for
'GCST90200144' on 'Calculus of kidney and ureter'

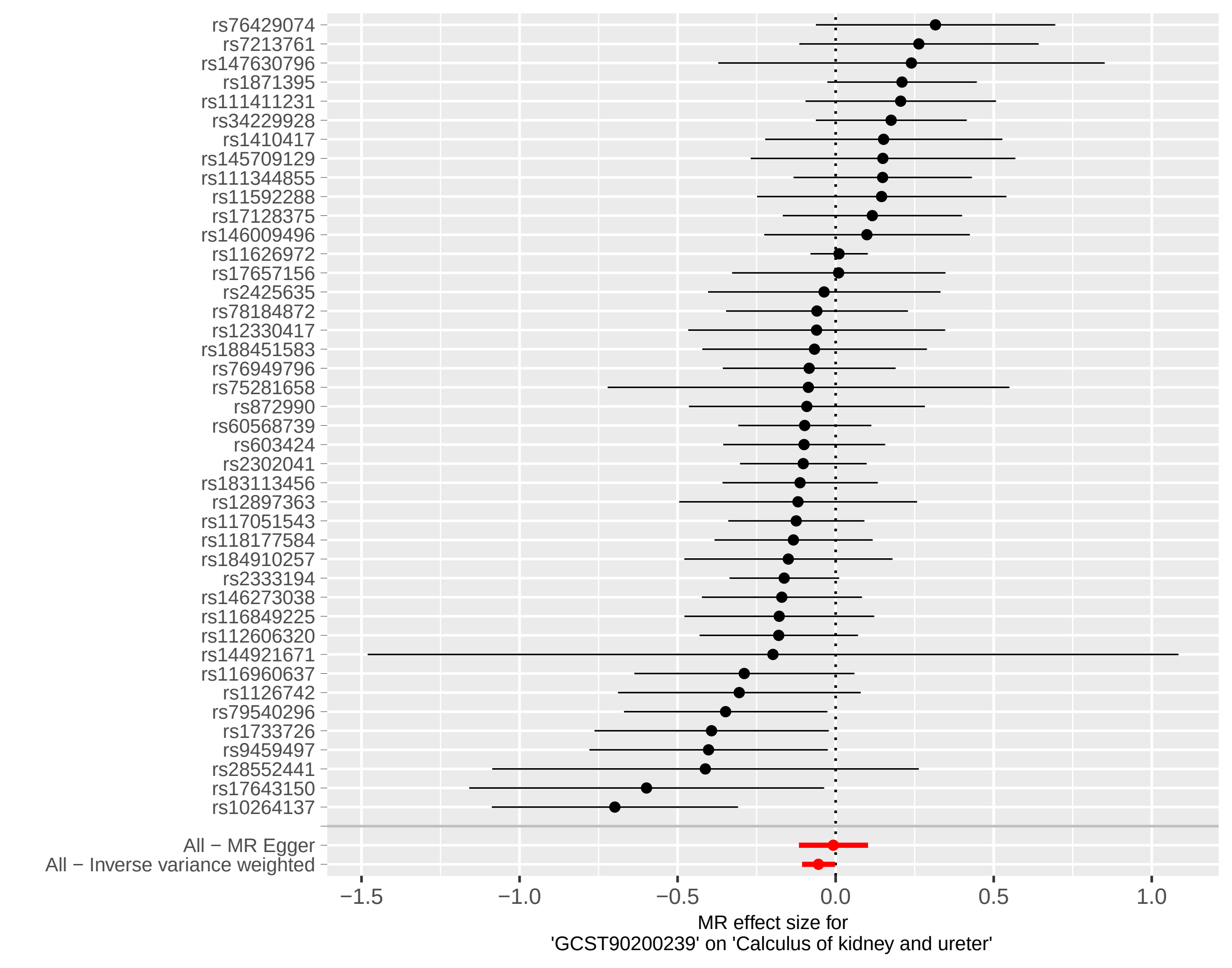
rs76429074
rs7213761
rs147630796
rs1871395
rs111411231
rs34229928
rs1410417
rs145709129
rs111344855
rs11592288
rs17128375
rs146009496
rs11626972
rs17657156
rs2425635
rs78184872
rs12330417
rs188451583
rs76949796
rs75281658
rs872990
rs60568739
rs603424
rs2302041
rs183113456
rs12897363
rs117051543
rs118177584
rs184910257
rs2333194
rs146273038
rs116849225
rs112606320
rs144921671
rs116960637
rs1126742
rs79540296
rs1733726
rs9459497
rs28552441
rs17643150
rs10264137
All − MR Egger
All − Inverse variance weighted
−1.5
−1.0
−0.5
0.0
0.5
1.0
MR effect size for
'GCST90200239' on 'Calculus of kidney and ureter'

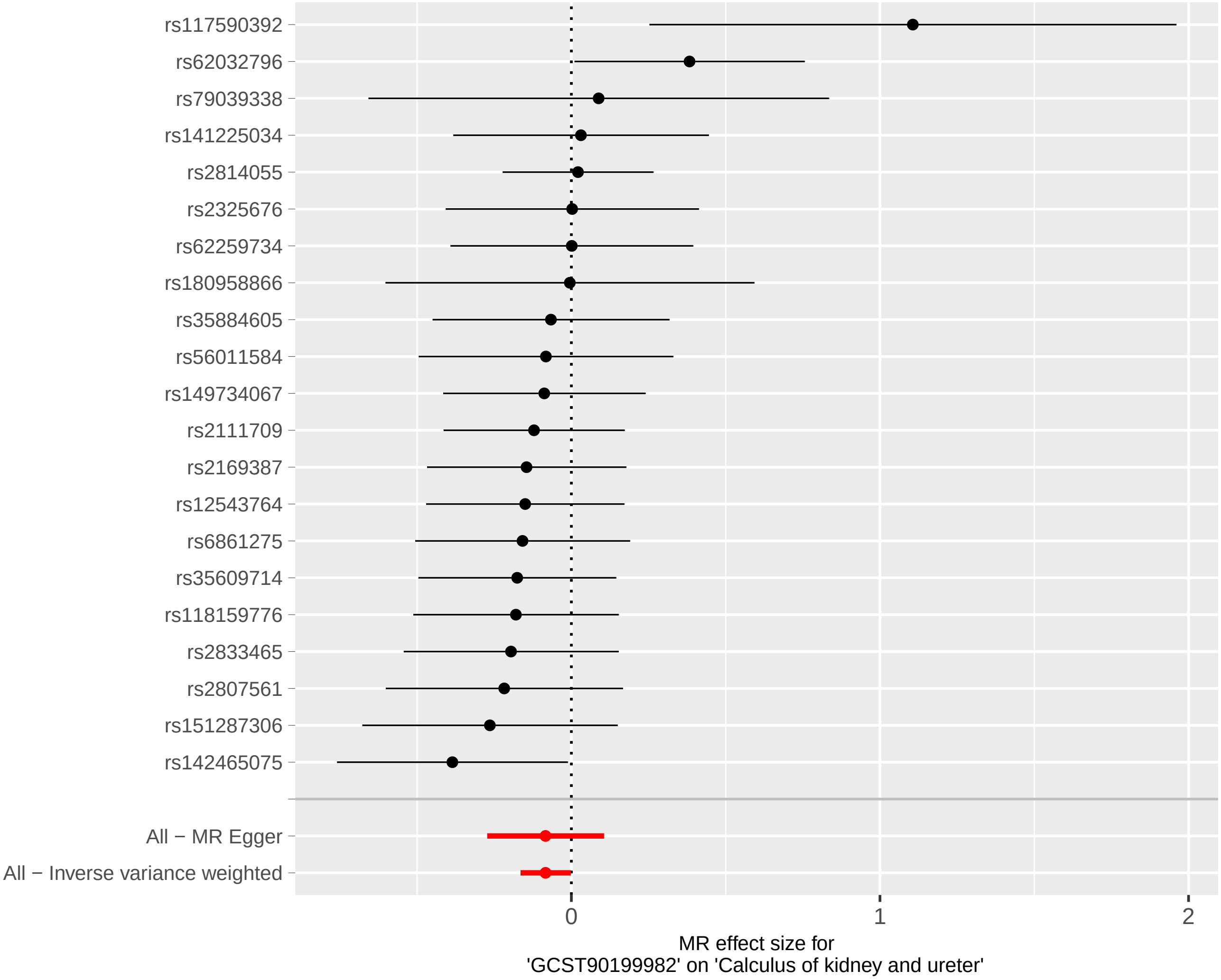
rs117590392
rs62032796
rs79039338
rs141225034
rs2814055
rs2325676
rs62259734
rs180958866
rs35884605
rs56011584
rs149734067
rs2111709
rs2169387
rs12543764
rs6861275
rs35609714
rs118159776
rs2833465
rs2807561
rs151287306
rs142465075
All − MR Egger
All − Inverse variance weighted
0
1
2
MR effect size for
'GCST90199982' on 'Calculus of kidney and ureter'

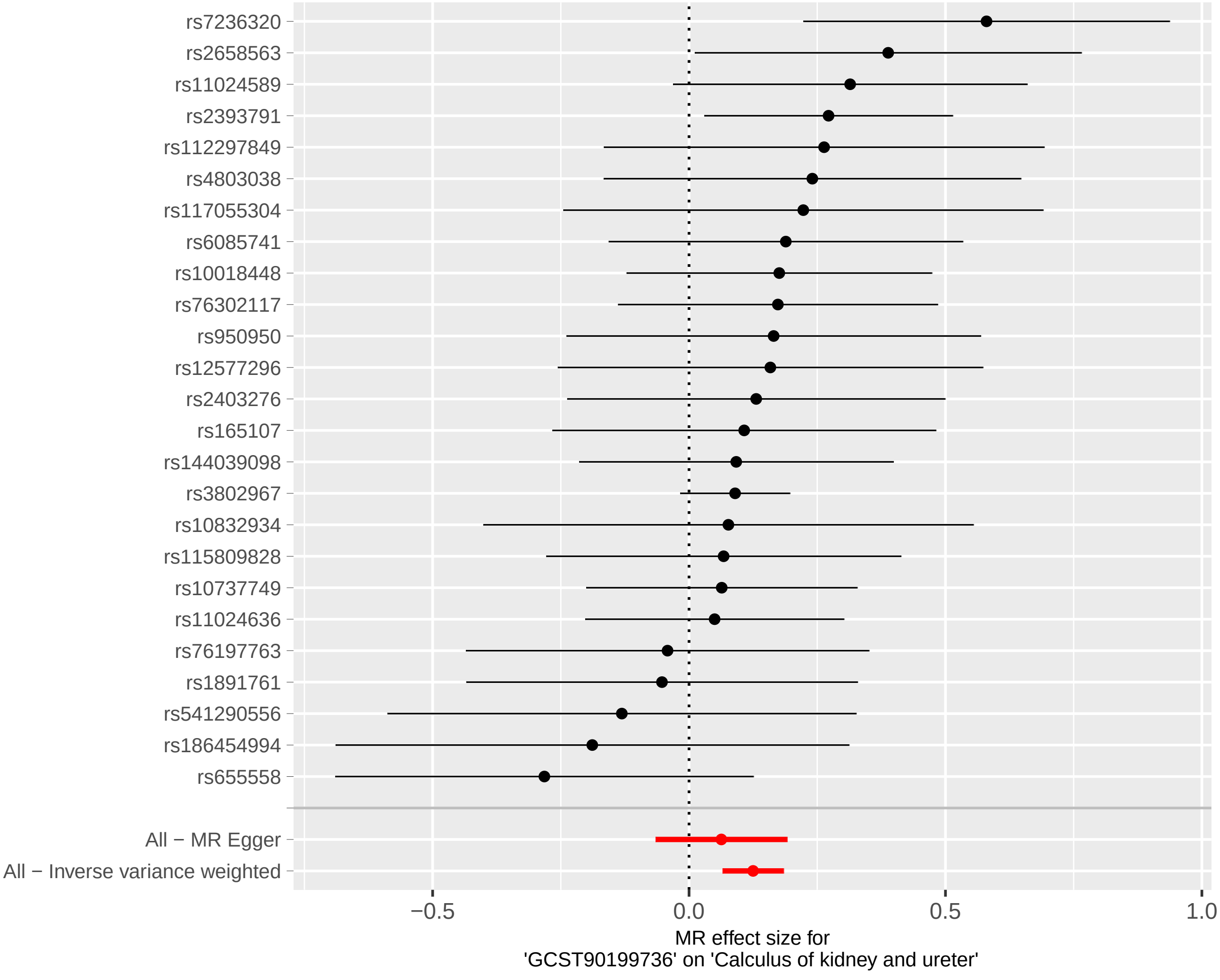

rs7236320
rs2658563
rs11024589
rs2393791
rs112297849
rs4803038
rs117055304
rs6085741
rs10018448
rs76302117
rs950950
rs12577296
rs2403276
rs165107
rs144039098
rs3802967
rs10832934
rs115809828
rs10737749
rs11024636
rs76197763
rs1891761
rs541290556
rs186454994
rs655558
All − MR Egger
All − Inverse variance weighted
−0.5
0.0
0.5
1.0
MR effect size for
'GCST90199736' on 'Calculus of kidney and ureter'

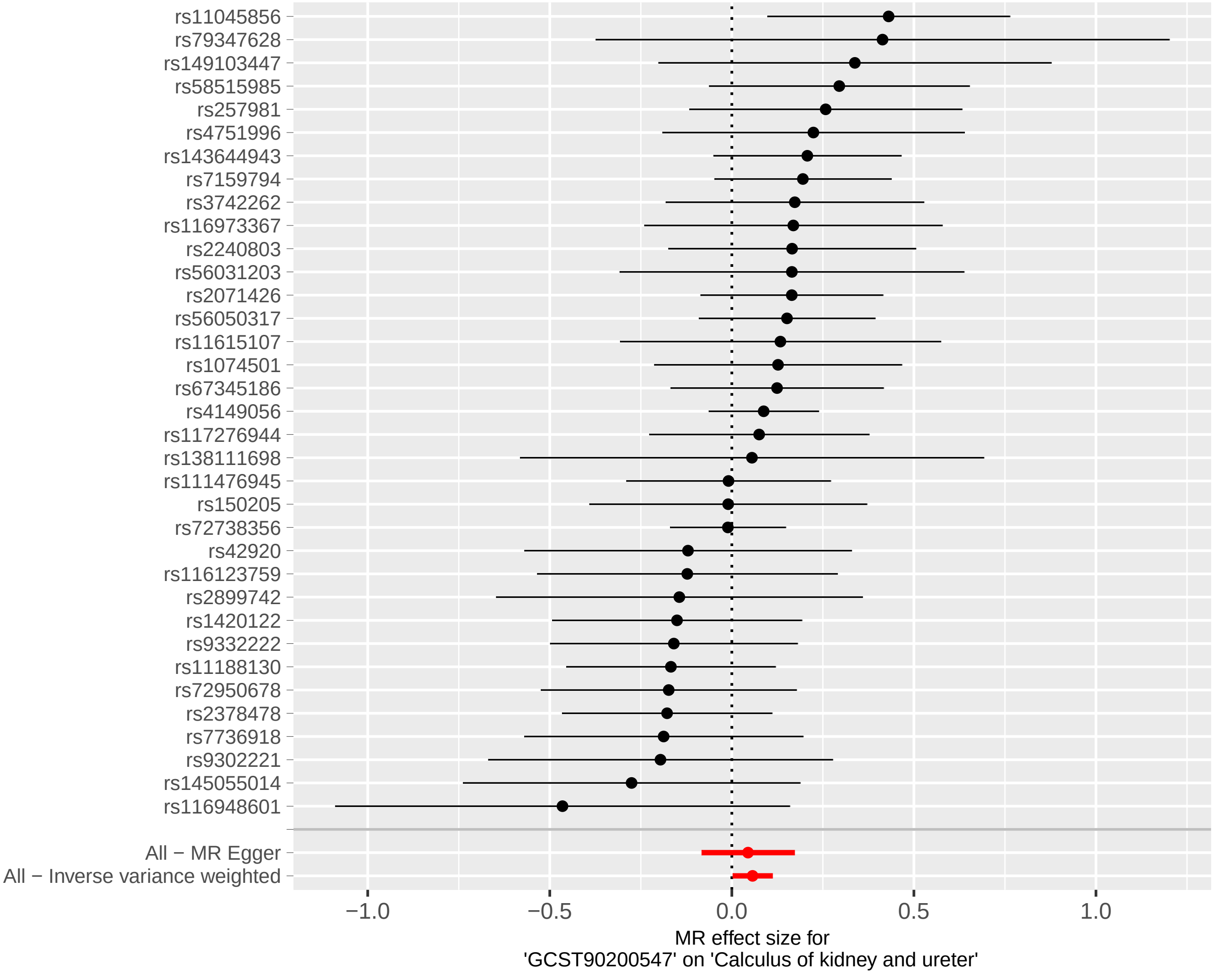

rs11045856
rs79347628
rs149103447
rs58515985
rs257981
rs4751996
rs143644943
rs7159794
rs3742262
rs116973367
rs2240803
rs56031203
rs2071426
rs56050317
rs11615107
rs1074501
rs67345186
rs4149056
rs117276944
rs138111698
rs111476945
rs150205
rs72738356
rs42920
rs116123759
rs2899742
rs1420122
rs9332222
rs11188130
rs72950678
rs2378478
rs7736918
rs9302221
rs145055014
rs116948601
All − MR Egger
All − Inverse variance weighted
−1.0
−0.5
0.0
0.5
1.0
MR effect size for
'GCST90200547' on 'Calculus of kidney and ureter'

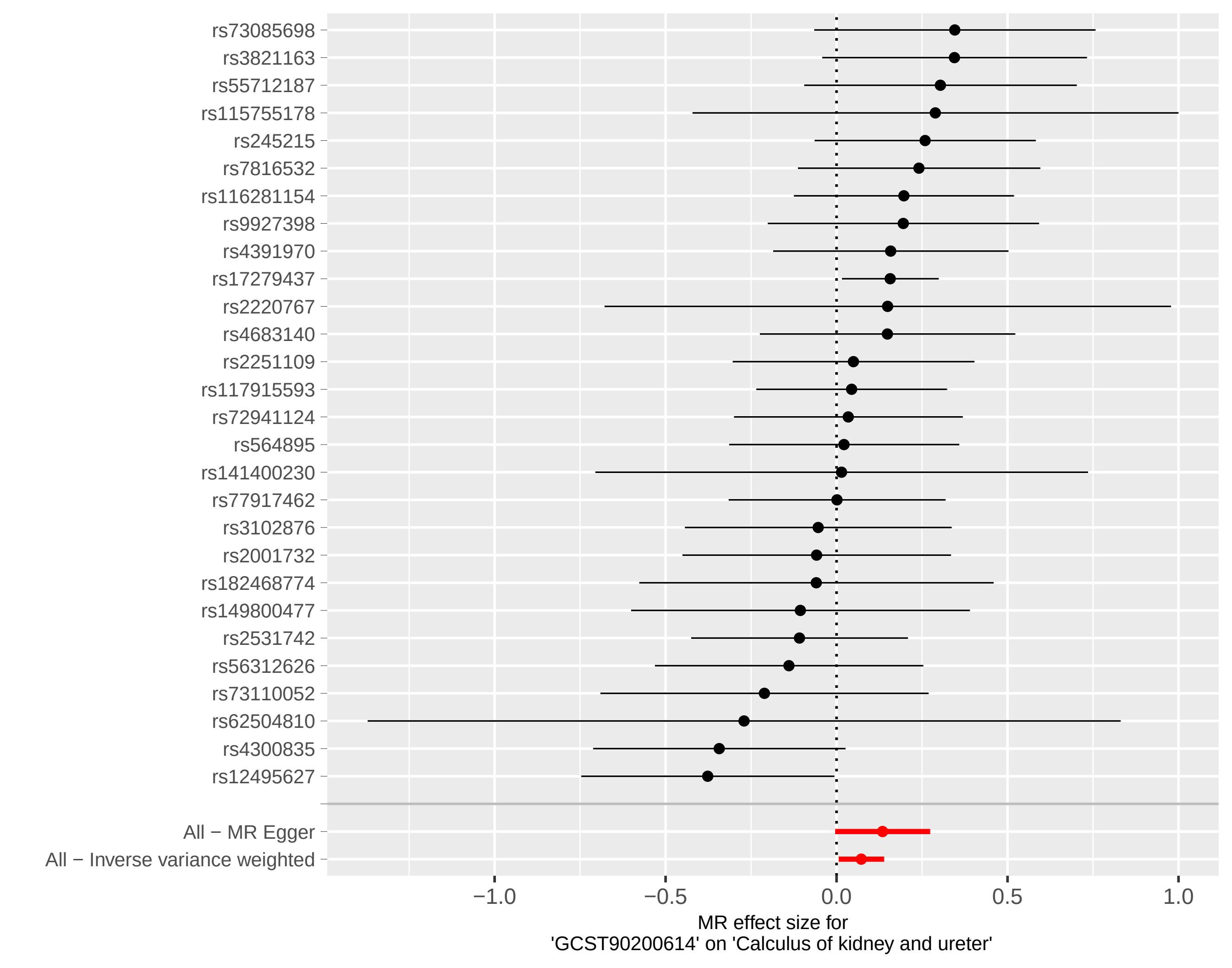
rs73085698
rs3821163
rs55712187
rs115755178
rs245215
rs7816532
rs116281154
rs9927398
rs4391970
rs17279437
rs2220767
rs4683140
rs2251109
rs117915593
rs72941124
rs564895
rs141400230
rs77917462
rs3102876
rs2001732
rs182468774
rs149800477
rs2531742
rs56312626
rs73110052
rs62504810
rs4300835
rs12495627
All − MR Egger
All − Inverse variance weighted
−1.0
−0.5
0.0
0.5
1.0
MR effect size for
'GCST90200614' on 'Calculus of kidney and ureter'

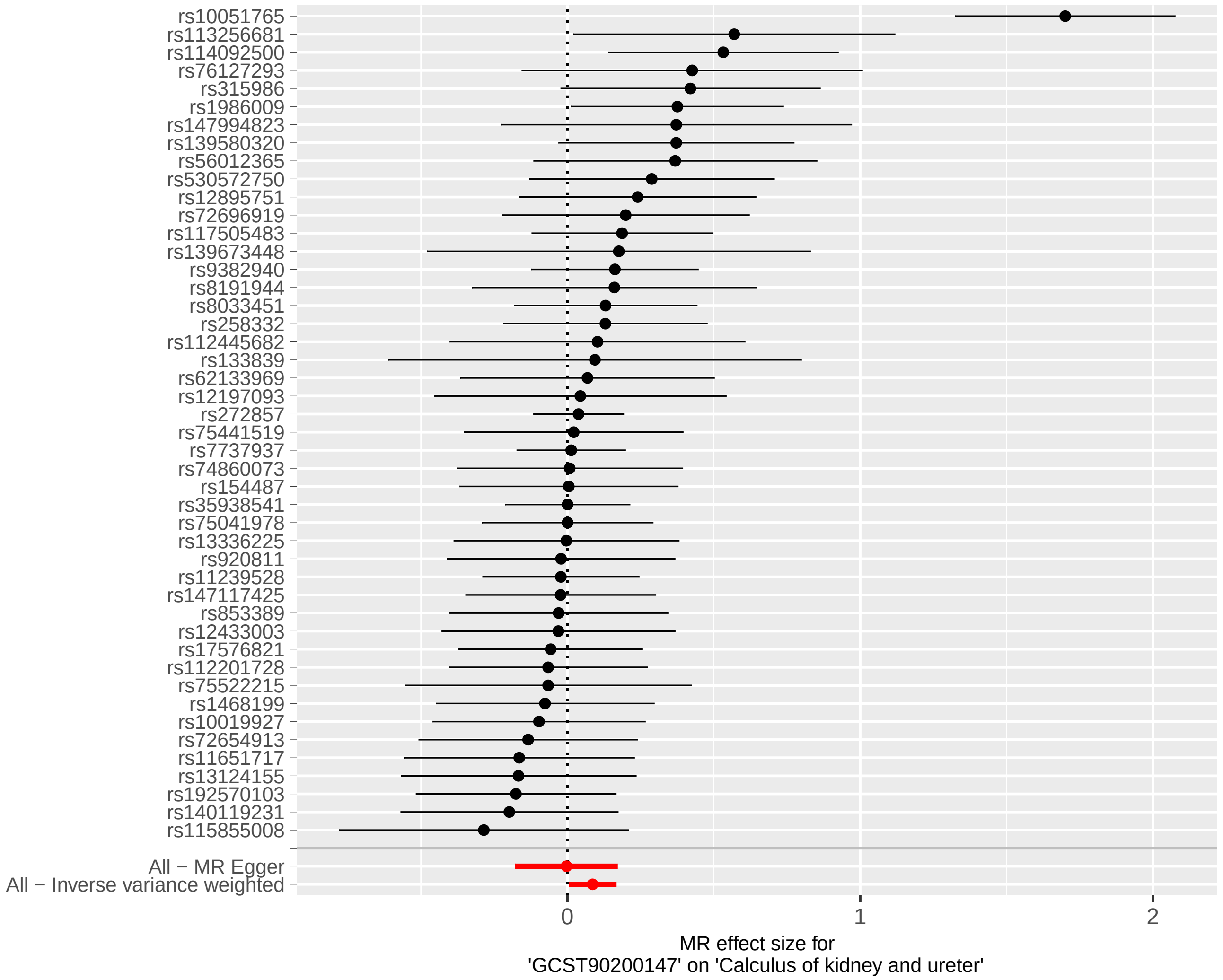
rs10051765
rs113256681
rs114092500
rs76127293
rs315986
rs1986009
rs147994823
rs139580320
rs56012365
rs530572750
rs12895751
rs72696919
rs117505483
rs139673448
rs9382940
rs8191944
rs8033451
rs258332
rs112445682
rs133839
rs62133969
rs12197093
rs272857
rs75441519
rs7737937
rs74860073
rs154487
rs35938541
rs75041978
rs13336225
rs920811
rs11239528
rs147117425
rs853389
rs12433003
rs17576821
rs112201728
rs75522215
rs1468199
rs10019927
rs72654913
rs11651717
rs13124155
rs192570103
rs140119231
rs115855008
All − MR Egger
All − Inverse variance weighted
0
1
2
MR effect size for
'GCST90200147' on 'Calculus of kidney and ureter'

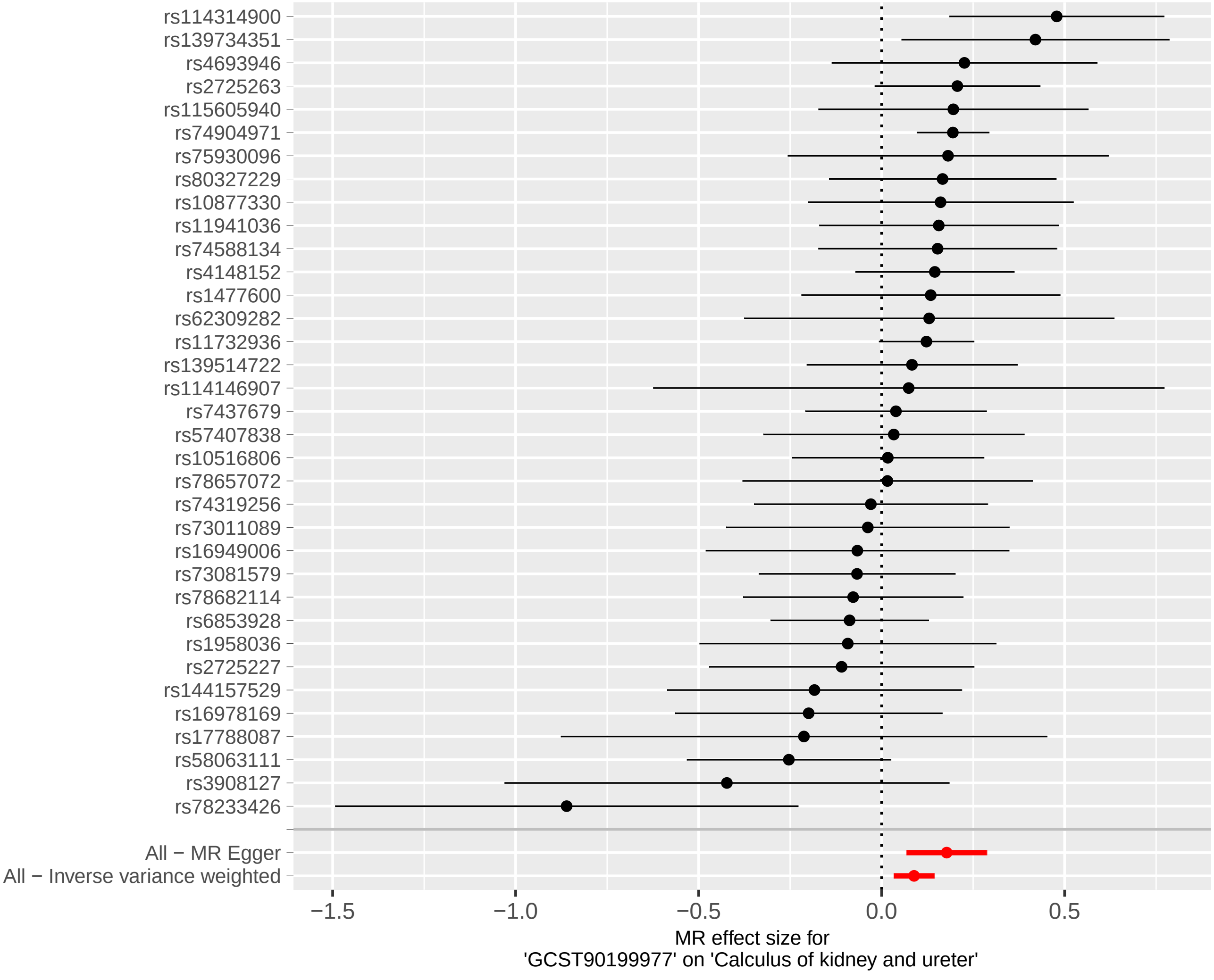
rs114314900
rs139734351
rs4693946
rs2725263
rs115605940
rs74904971
rs75930096
rs80327229
rs10877330
rs11941036
rs74588134
rs4148152
rs1477600
rs62309282
rs11732936
rs139514722
rs114146907
rs7437679
rs57407838
rs10516806
rs78657072
rs74319256
rs73011089
rs16949006
rs73081579
rs78682114
rs6853928
rs1958036
rs2725227
rs144157529
rs16978169
rs17788087
rs58063111
rs3908127
rs78233426
All − MR Egger
All − Inverse variance weighted
−1.5
−1.0
−0.5
0.0
0.5
MR effect size for
'GCST90199977' on 'Calculus of kidney and ureter'

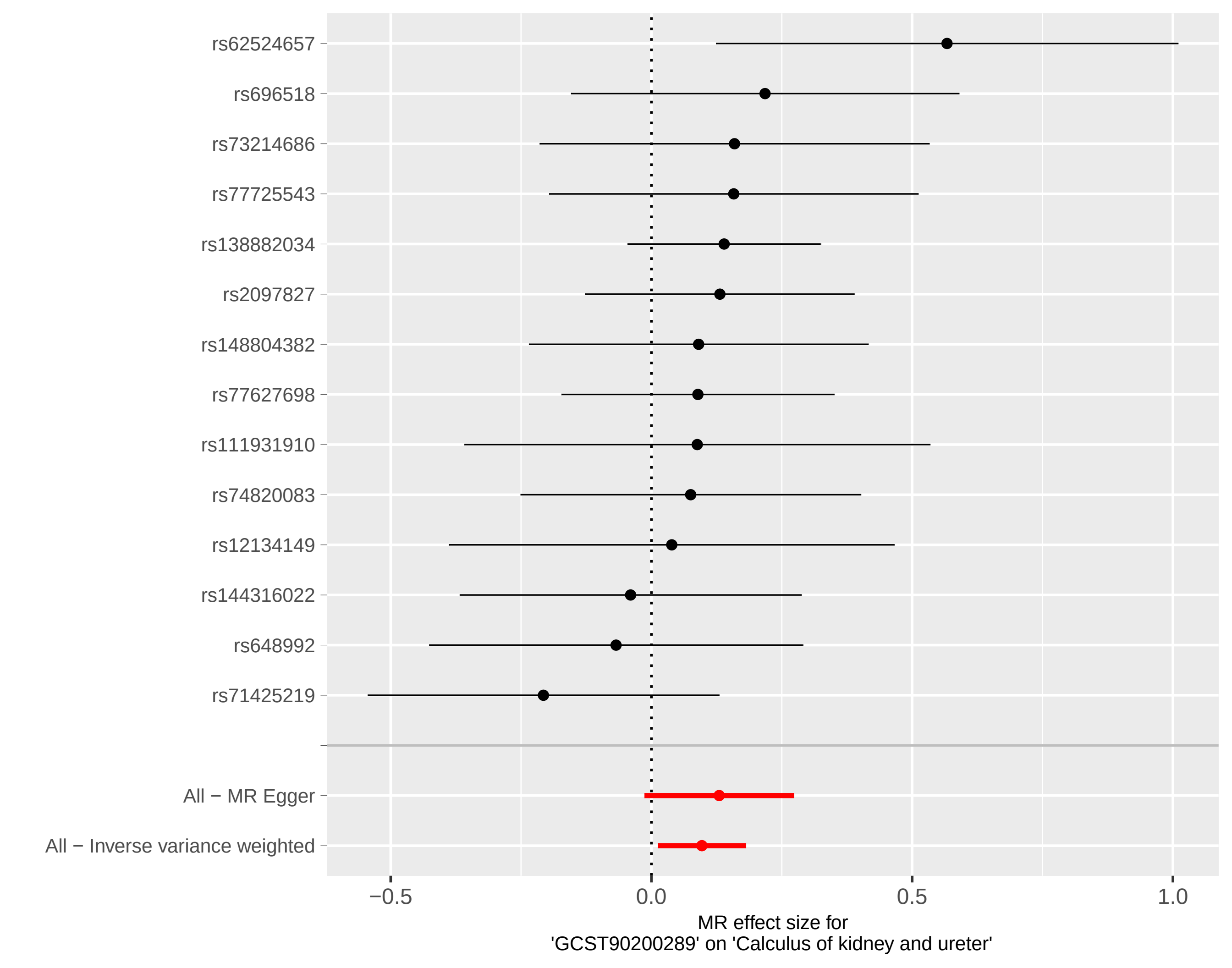
rs62524657
rs696518
rs73214686
rs77725543
rs138882034
rs2097827
rs148804382
rs77627698
rs111931910
rs74820083
rs12134149
rs144316022
rs648992
rs71425219
All − MR Egger
All − Inverse variance weighted
−0.5
0.0
0.5
1.0
MR effect size for
'GCST90200289' on 'Calculus of kidney and ureter'

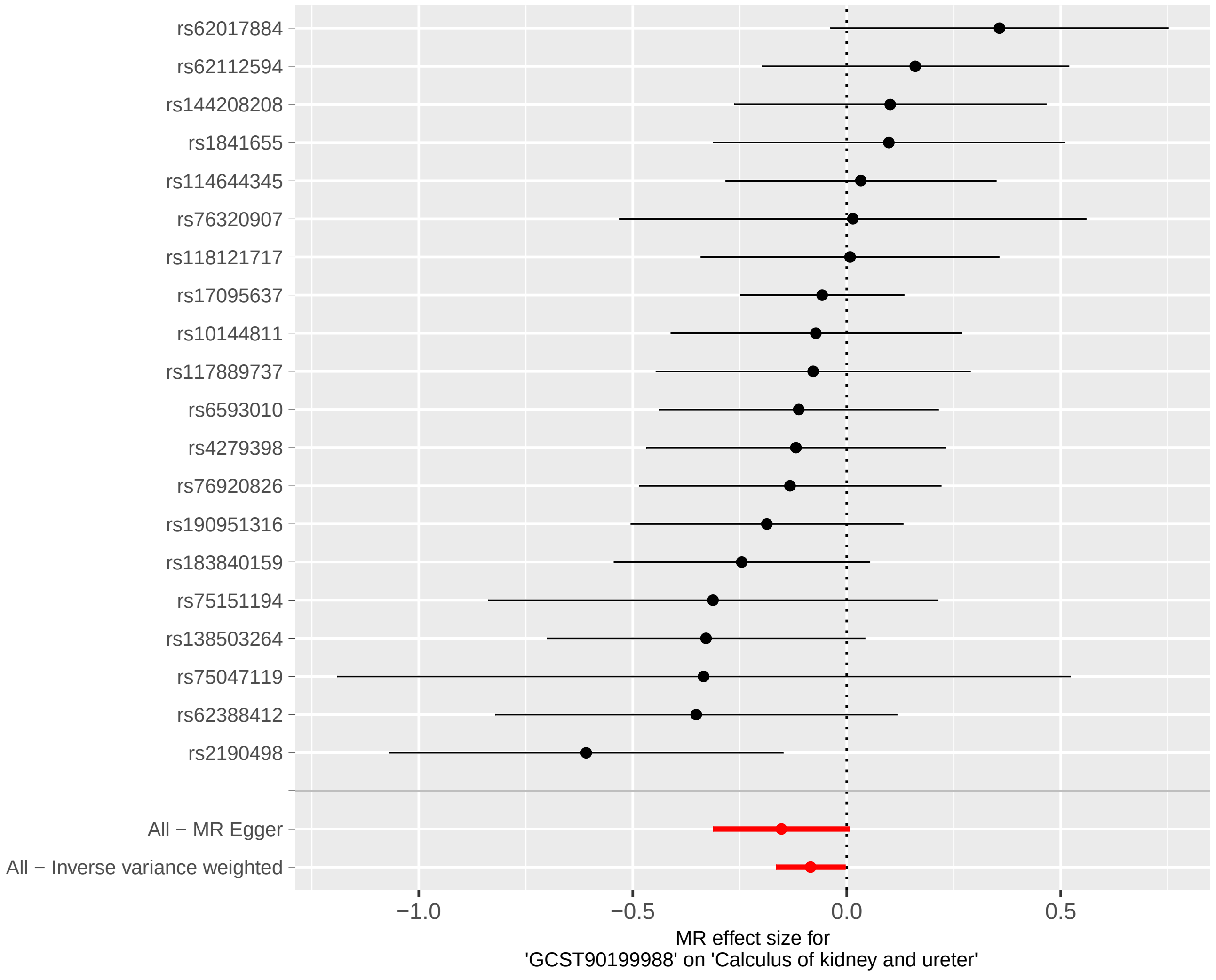
rs62017884
rs62112594
rs144208208
rs1841655
rs114644345
rs76320907
rs118121717
rs17095637
rs10144811
rs117889737
rs6593010
rs4279398
rs76920826
rs190951316
rs183840159
rs75151194
rs138503264
rs75047119
rs62388412
rs2190498
All − MR Egger
All − Inverse variance weighted
−1.0
−0.5
0.0
0.5
MR effect size for
'GCST90199988' on 'Calculus of kidney and ureter'

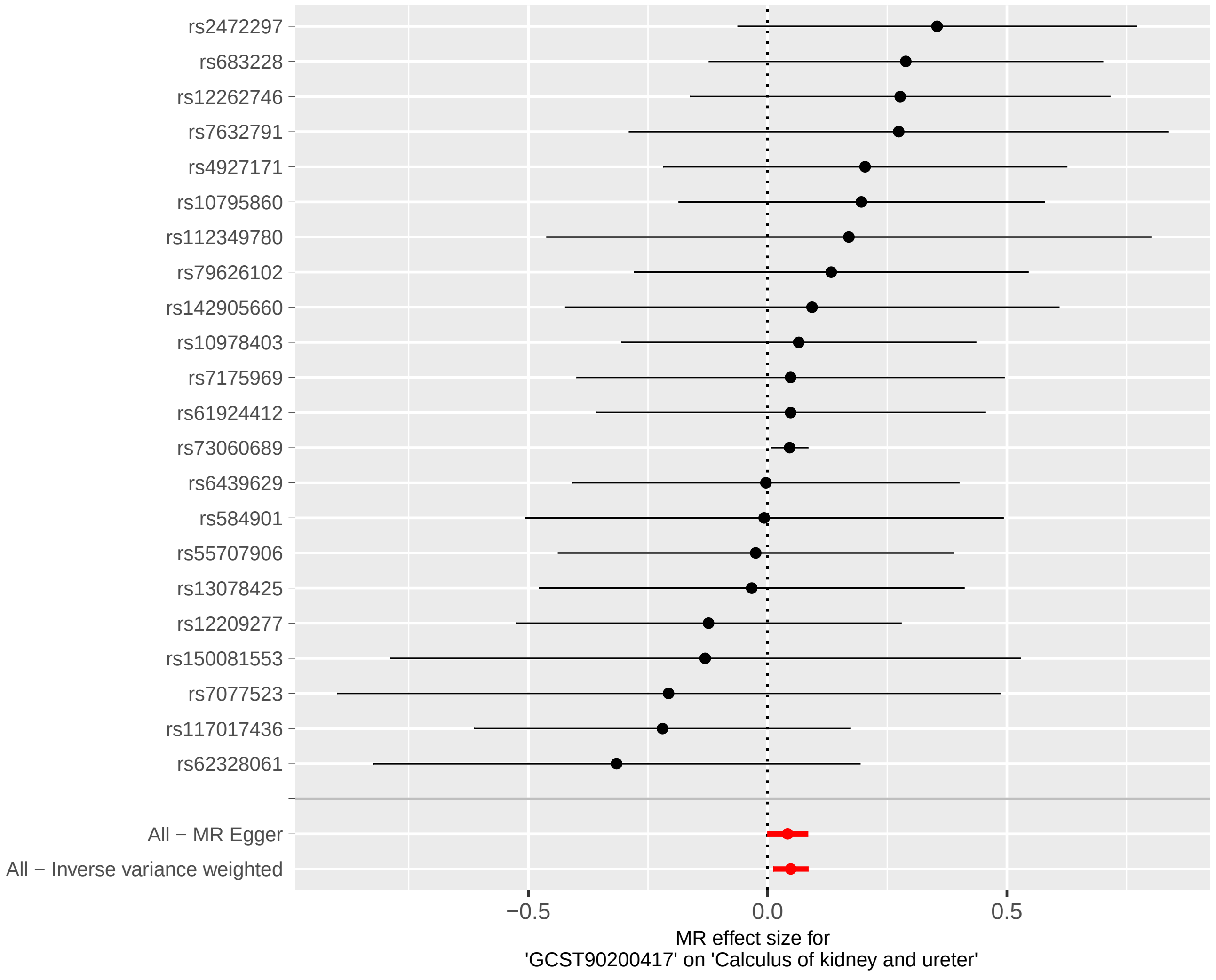

rs2472297
rs683228
rs12262746
rs7632791
rs4927171
rs10795860
rs112349780
rs79626102
rs142905660
rs10978403
rs7175969
rs61924412
rs73060689
rs6439629
rs584901
rs55707906
rs13078425
rs12209277
rs150081553
rs7077523
rs117017436
rs62328061
All − MR Egger
All − Inverse variance weighted
−0.5
0.0
0.5
MR effect size for
'GCST90200417' on 'Calculus of kidney and ureter'

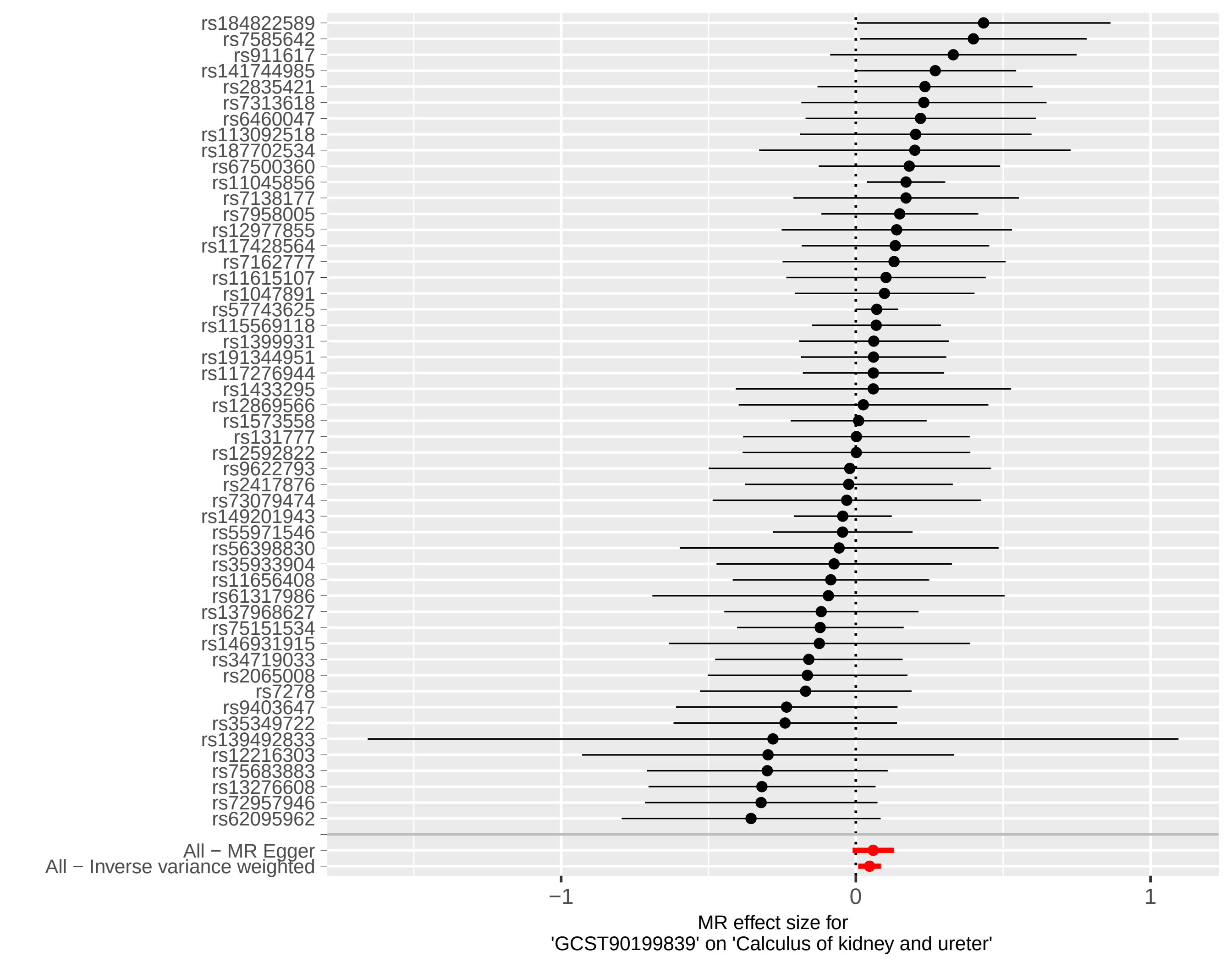
rs184822589
rs7585642
rs911617
rs141744985
rs2835421
rs7313618
rs6460047
rs113092518
rs187702534
rs67500360
rs11045856
rs7138177
rs7958005
rs12977855
rs117428564
rs7162777
rs11615107
rs1047891
rs57743625
rs115569118
rs1399931
rs191344951
rs117276944
rs1433295
rs12869566
rs1573558
rs131777
rs12592822
rs9622793
rs2417876
rs73079474
rs149201943
rs55971546
rs56398830
rs35933904
rs11656408
rs61317986
rs137968627
rs75151534
rs146931915
rs34719033
rs2065008
rs7278
rs9403647
rs35349722
rs139492833
rs12216303
rs75683883
rs13276608
rs72957946
rs62095962
All − MR Egger
All − Inverse variance weighted
−1
0
1
MR effect size for
'GCST90199839' on 'Calculus of kidney and ureter'

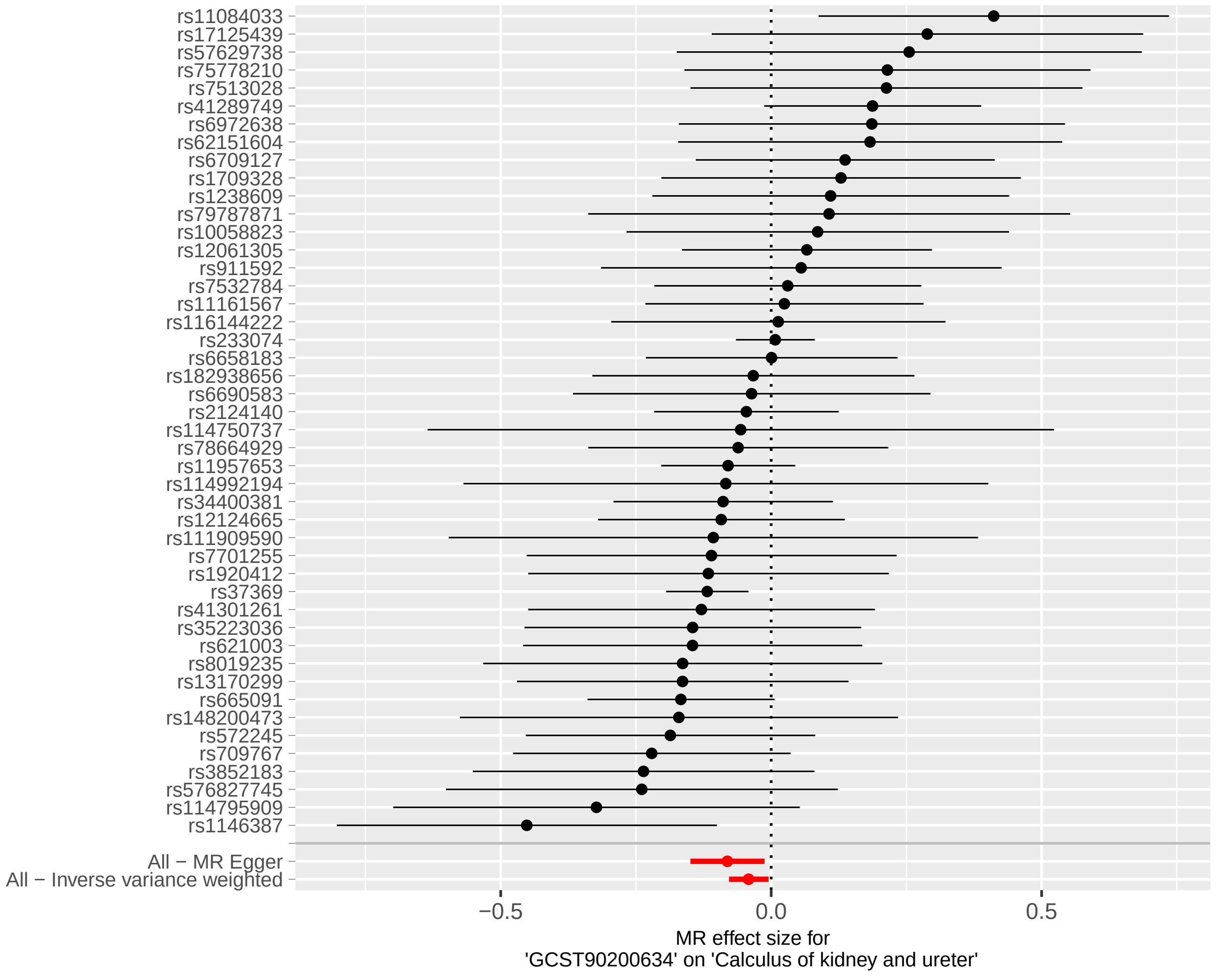
rs11084033
rs17125439
rs57629738
rs75778210
rs7513028
rs41289749
rs6972638
rs62151604
rs6709127
rs1709328
rs1238609
rs79787871
rs10058823
rs12061305
rs911592
rs7532784
rs11161567
rs116144222
rs233074
rs6658183
rs182938656
rs6690583
rs2124140
rs114750737
rs78664929
rs11957653
rs114992194
rs34400381
rs12124665
rs111909590
rs7701255
rs1920412
rs37369
rs41301261
rs35223036
rs621003
rs8019235
rs13170299
rs665091
rs148200473
rs572245
rs709767
rs3852183
rs576827745
rs114795909
rs1146387
All − MR Egger
All − Inverse variance weighted
−0.5
0.0
0.5
MR effect size for
'GCST90200634' on 'Calculus of kidney and ureter'

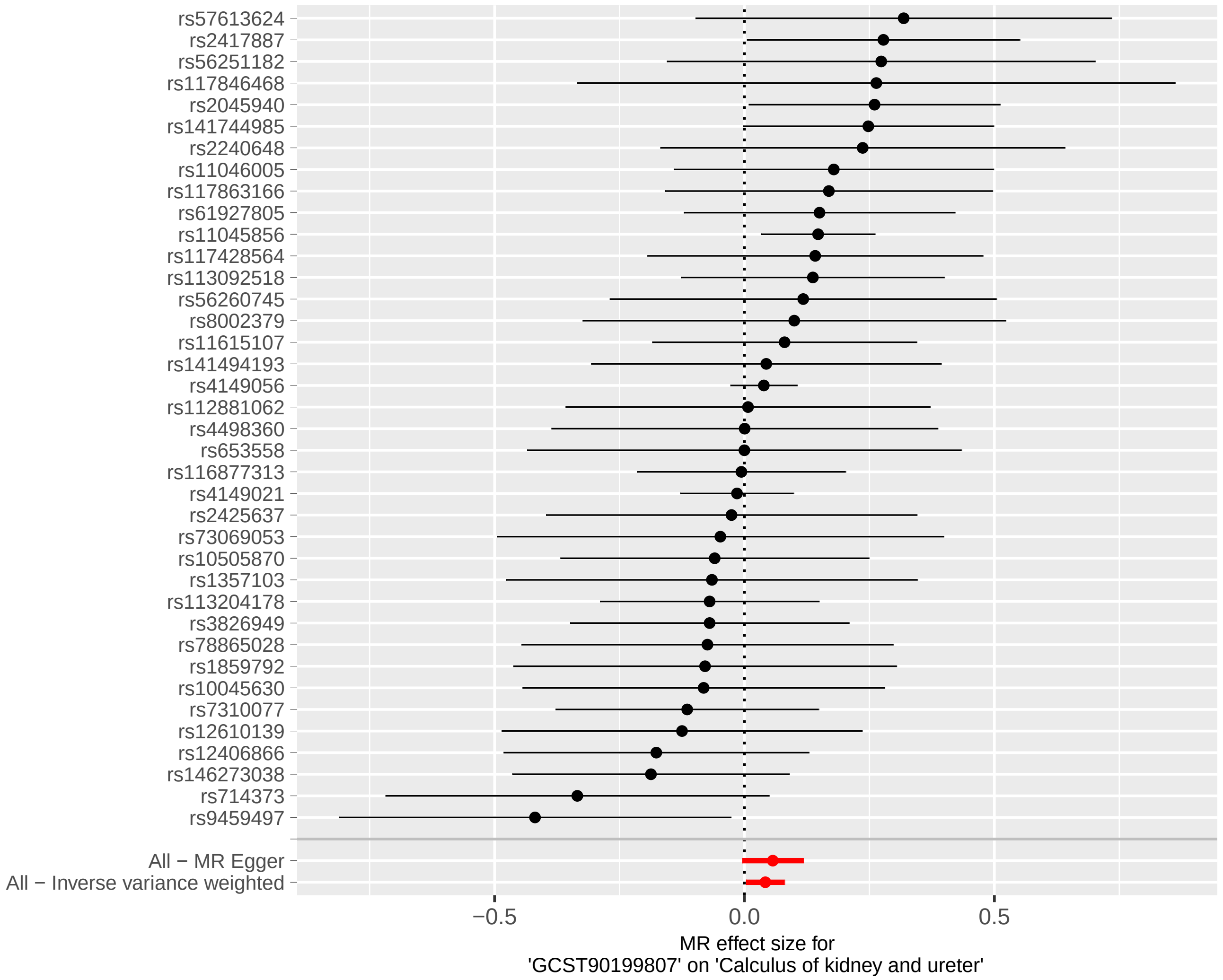

rs57613624
rs2417887
rs56251182
rs117846468
rs2045940
rs141744985
rs2240648
rs11046005
rs117863166
rs61927805
rs11045856
rs117428564
rs113092518
rs56260745
rs8002379
rs11615107
rs141494193
rs4149056
rs112881062
rs4498360
rs653558
rs116877313
rs4149021
rs2425637
rs73069053
rs10505870
rs1357103
rs113204178
rs3826949
rs78865028
rs1859792
rs10045630
rs7310077
rs12610139
rs12406866
rs146273038
rs714373
rs9459497
All − MR Egger
All − Inverse variance weighted
−0.5
0.0
0.5
MR effect size for
'GCST90199807' on 'Calculus of kidney and ureter'

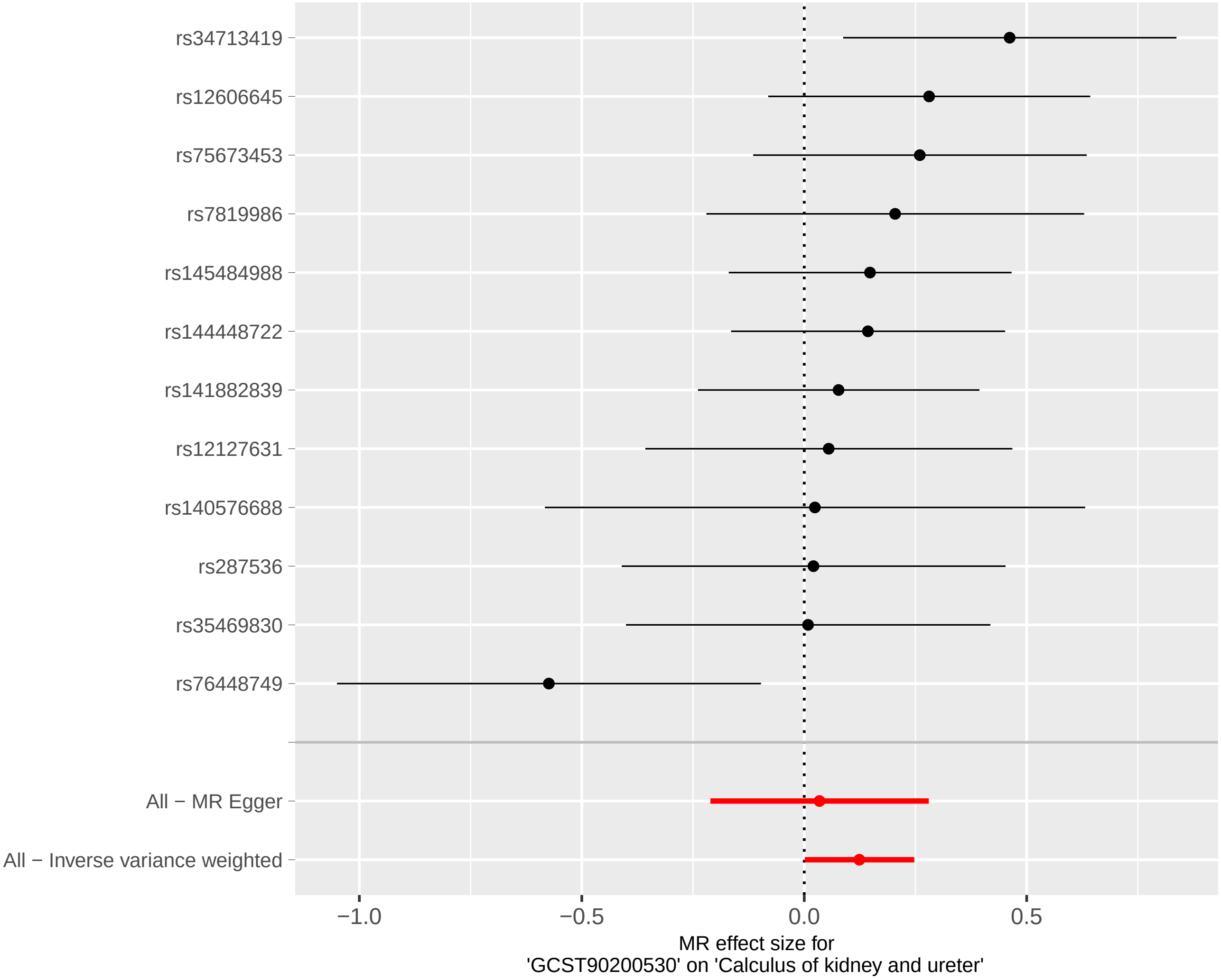
rs34713419
rs12606645
rs75673453
rs7819986
rs145484988
rs144448722
rs141882839
rs12127631
rs140576688
rs287536
rs35469830
rs76448749
All − MR Egger
All − Inverse variance weighted
−1.0
−0.5
0.0
0.5
MR effect size for
'GCST90200530' on 'Calculus of kidney and ureter'

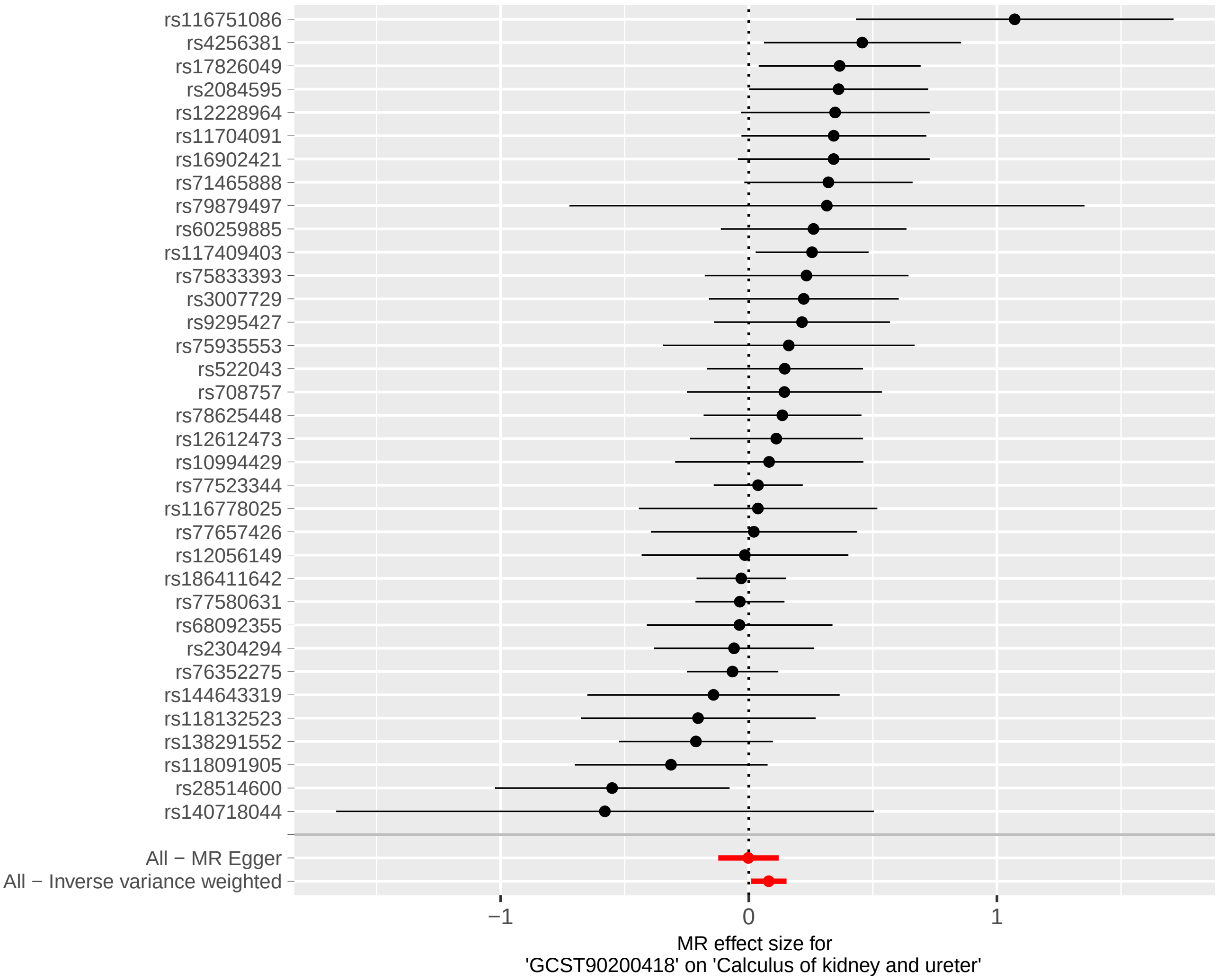
rs116751086
rs4256381
rs17826049
rs2084595
rs12228964
rs11704091
rs16902421
rs71465888
rs79879497
rs60259885
rs117409403
rs75833393
rs3007729
rs9295427
rs75935553
rs522043
rs708757
rs78625448
rs12612473
rs10994429
rs77523344
rs116778025
rs77657426
rs12056149
rs186411642
rs77580631
rs68092355
rs2304294
rs76352275
rs144643319
rs118132523
rs138291552
rs118091905
rs28514600
rs140718044
All − MR Egger
All − Inverse variance weighted
−1
0
1
MR effect size for
'GCST90200418' on 'Calculus of kidney and ureter'

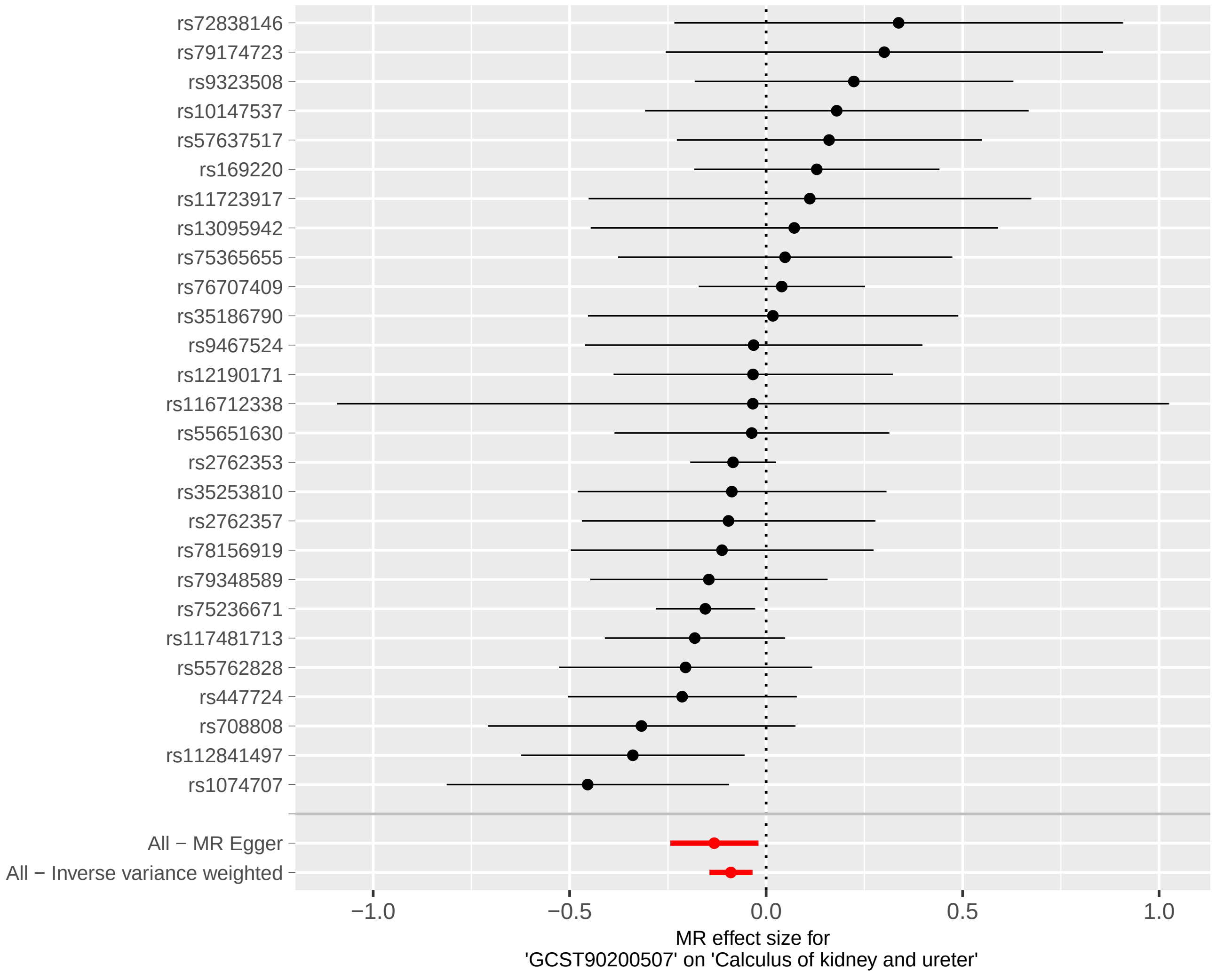

rs72838146
rs79174723
rs9323508
rs10147537
rs57637517
rs169220
rs11723917
rs13095942
rs75365655
rs76707409
rs35186790
rs9467524
rs12190171
rs116712338
rs55651630
rs2762353
rs35253810
rs2762357
rs78156919
rs79348589
rs75236671
rs117481713
rs55762828
rs447724
rs708808
rs112841497
rs1074707
All − MR Egger
All − Inverse variance weighted
−1.0
−0.5
0.0
0.5
1.0
MR effect size for
'GCST90200507' on 'Calculus of kidney and ureter'

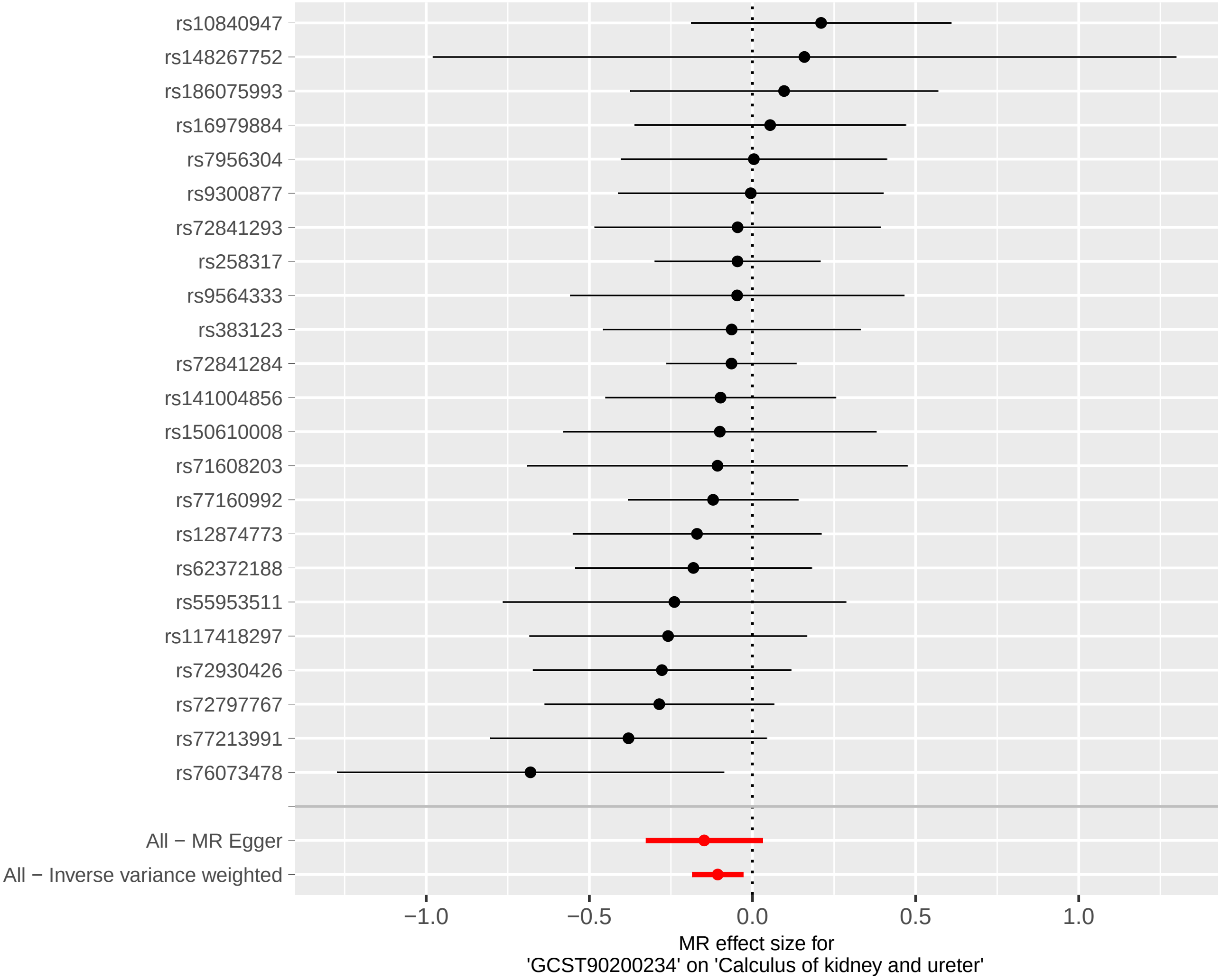
rs10840947
rs148267752
rs186075993
rs16979884
rs7956304
rs9300877
rs72841293
rs258317
rs9564333
rs383123
rs72841284
rs141004856
rs150610008
rs71608203
rs77160992
rs12874773
rs62372188
rs55953511
rs117418297
rs72930426
rs72797767
rs77213991
rs76073478
All − MR Egger
All − Inverse variance weighted
−1.0
−0.5
0.0
0.5
1.0
MR effect size for
'GCST90200234' on 'Calculus of kidney and ureter'

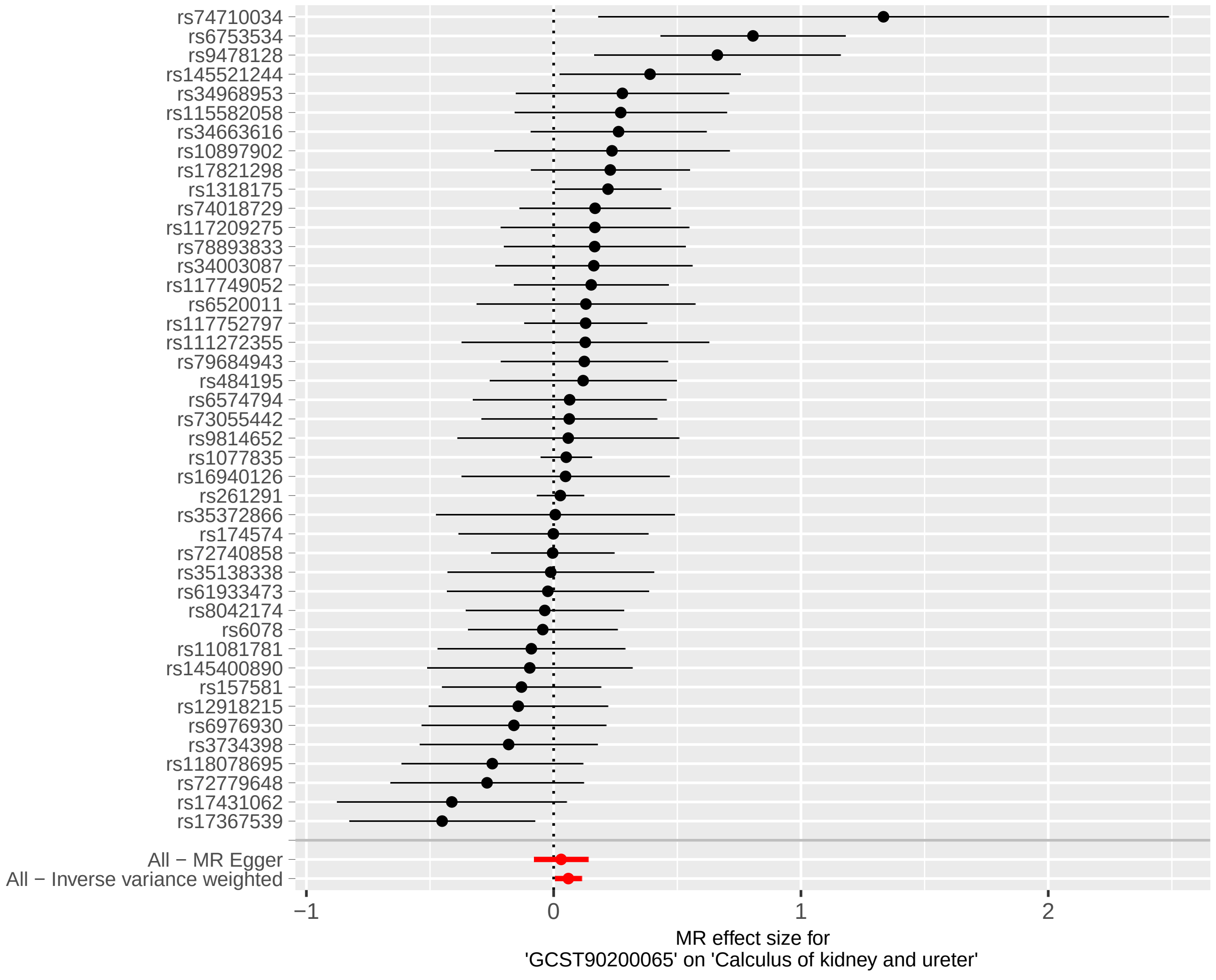
rs74710034
rs6753534
rs9478128
rs145521244
rs34968953
rs115582058
rs34663616
rs10897902
rs17821298
rs1318175
rs74018729
rs117209275
rs78893833
rs34003087
rs117749052
rs6520011
rs117752797
rs111272355
rs79684943
rs484195
rs6574794
rs73055442
rs9814652
rs1077835
rs16940126
rs261291
rs35372866
rs174574
rs72740858
rs35138338
rs61933473
rs8042174
rs6078
rs11081781
rs145400890
rs157581
rs12918215
rs6976930
rs3734398
rs118078695
rs72779648
rs17431062
rs17367539
All − MR Egger
All − Inverse variance weighted
−1
0
1
2
MR effect size for
'GCST90200065' on 'Calculus of kidney and ureter'

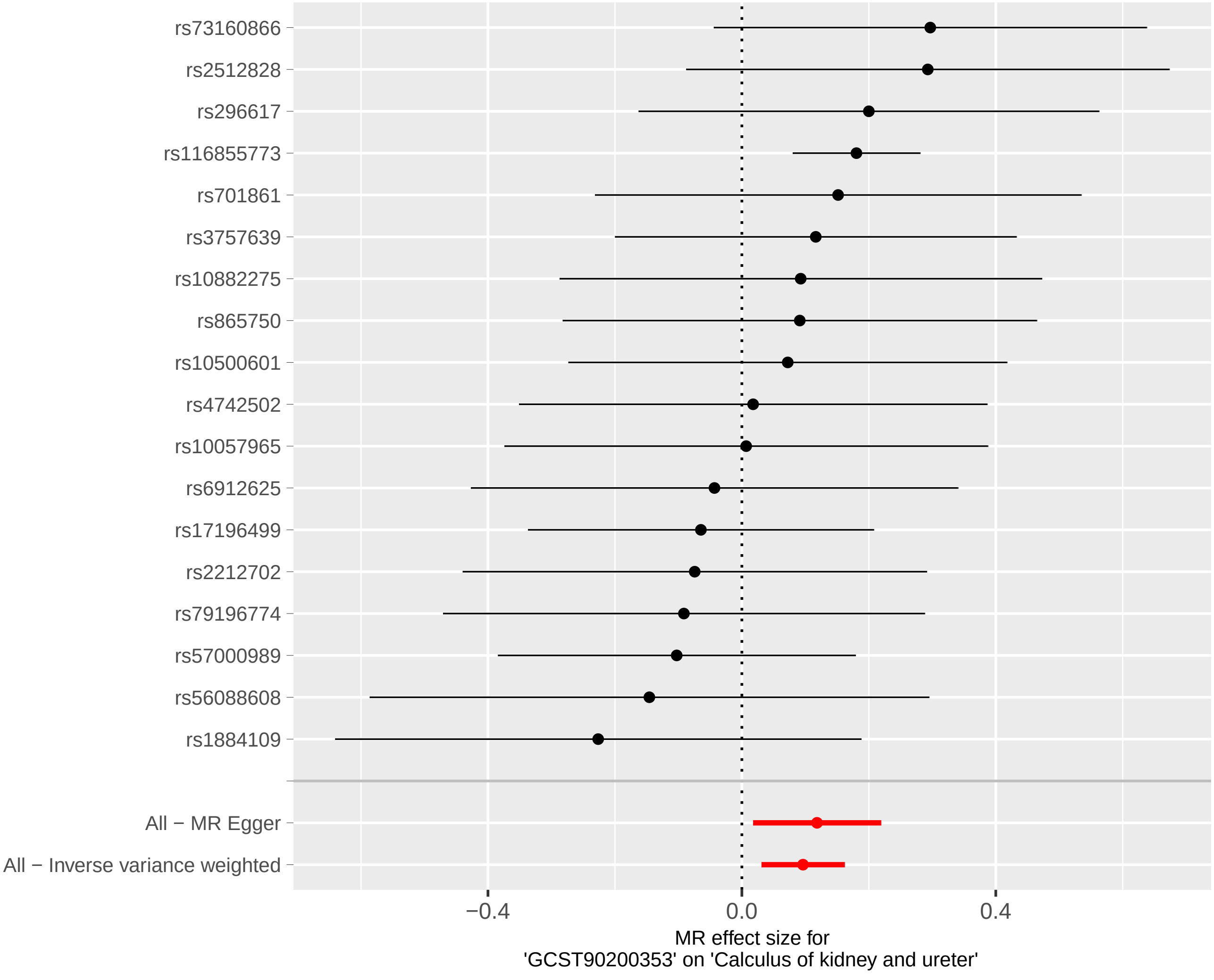
rs73160866
rs2512828
rs296617
rs116855773
rs701861
rs3757639
rs10882275
rs865750
rs10500601
rs4742502
rs10057965
rs6912625
rs17196499
rs2212702
rs79196774
rs57000989
rs56088608
rs1884109
All − MR Egger
All − Inverse variance weighted
−0.4
0.0
0.4
MR effect size for
'GCST90200353' on 'Calculus of kidney and ureter'

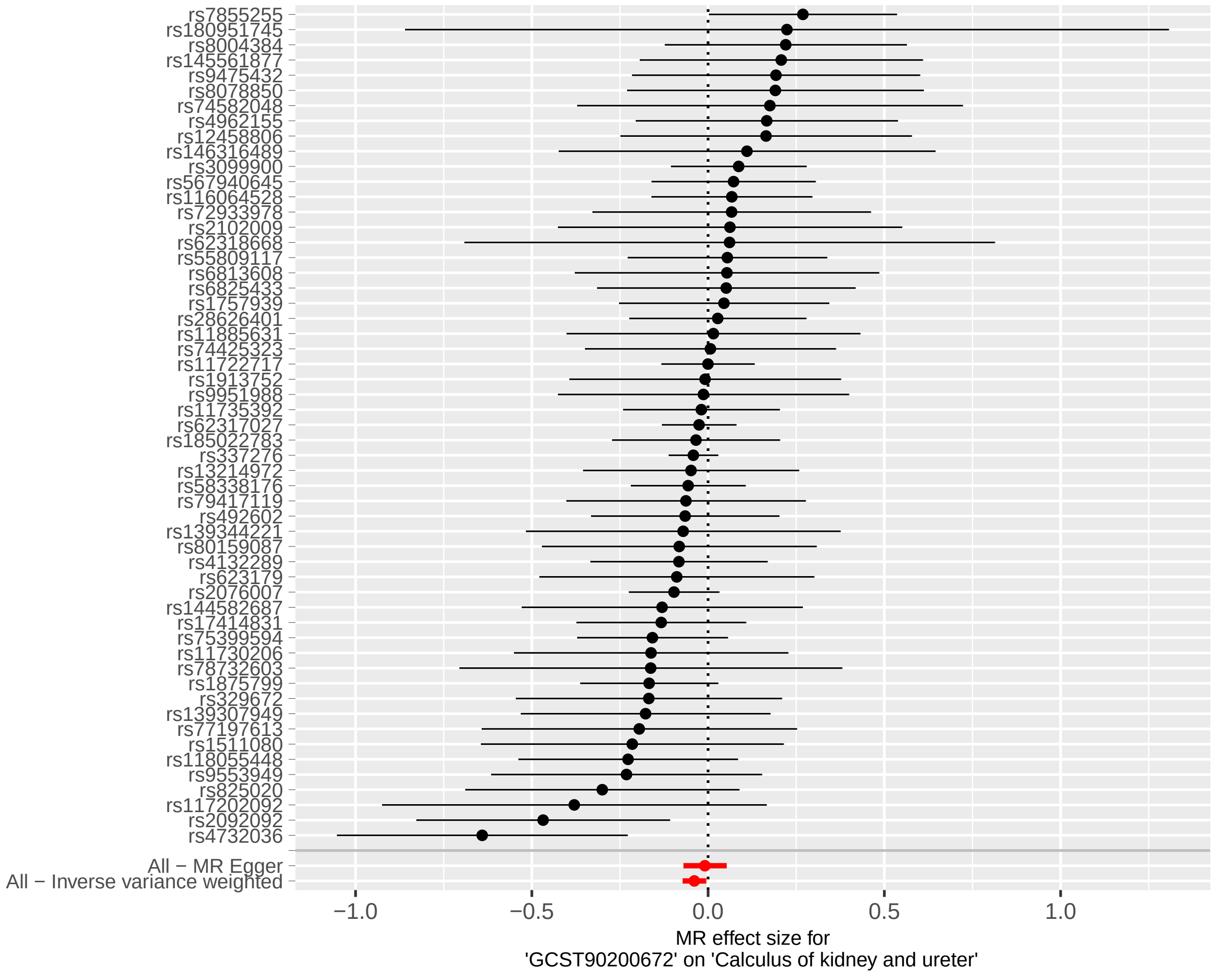
rs7855255
rs180951745
rs8004384
rs145561877
rs9475432
rs8078850
rs74582048
rs4962155
rs12458806
rs146316489
rs3099900
rs567940645
rs116064528
rs72933978
rs2102009
rs62318668
rs55809117
rs6813608
rs6825433
rs1757939
rs28626401
rs11885631
rs74425323
rs11722717
rs1913752
rs9951988
rs11735392
rs62317027
rs185022783
rs337276
rs13214972
rs58338176
rs79417119
rs492602
rs139344221
rs80159087
rs4132289
rs623179
rs2076007
rs144582687
rs17414831
rs75399594
rs11730206
rs78732603
rs1875799
rs329672
rs139307949
rs77197613
rs1511080
rs118055448
rs9553949
rs825020
rs117202092
rs2092092
rs4732036
All − MR Egger
All − Inverse variance weighted
−1.0
−0.5
0.0
0.5
1.0
MR effect size for
'GCST90200672' on 'Calculus of kidney and ureter'

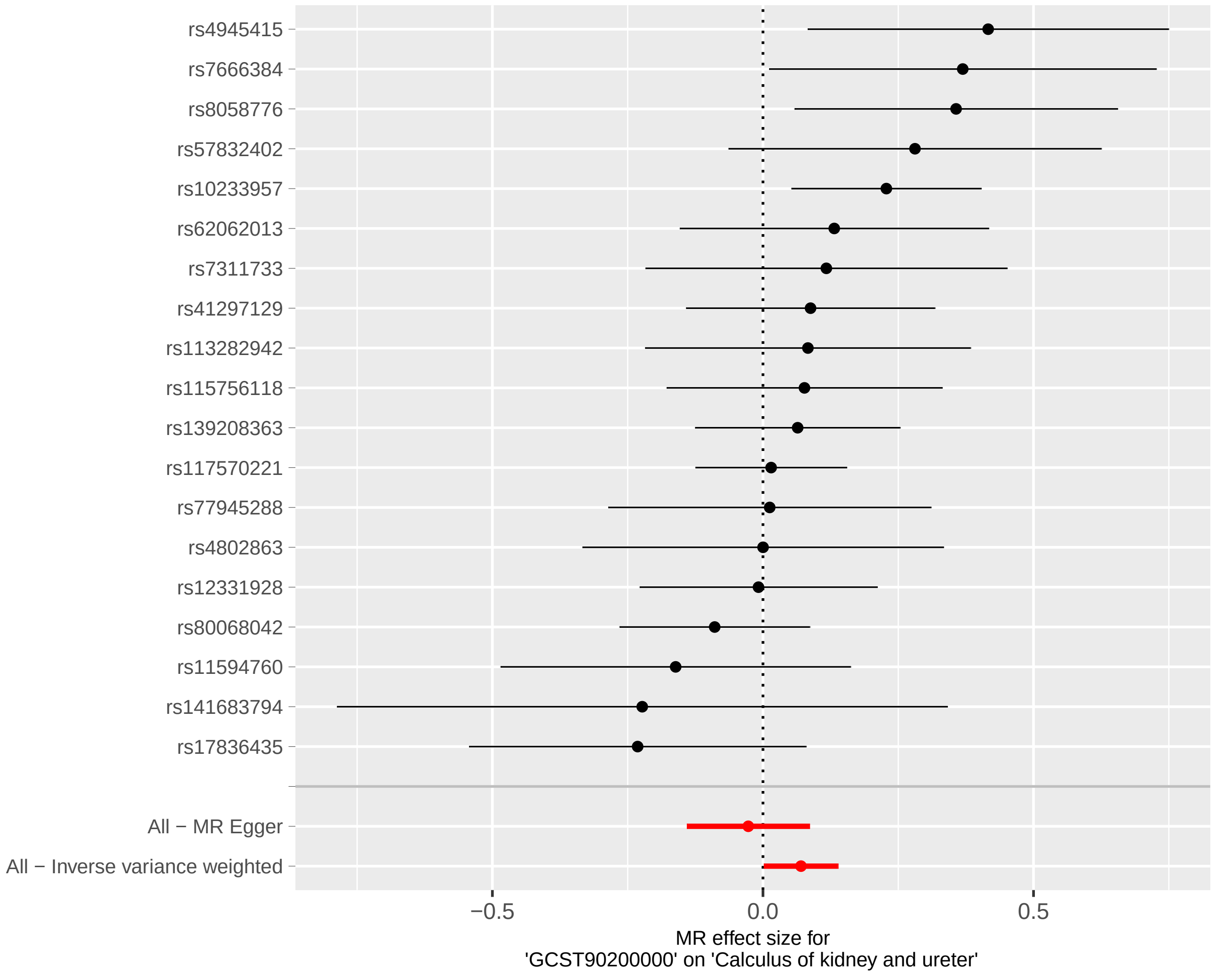

rs4945415
rs7666384
rs8058776
rs57832402
rs10233957
rs62062013
rs7311733
rs41297129
rs113282942
rs115756118
rs139208363
rs117570221
rs77945288
rs4802863
rs12331928
rs80068042
rs11594760
rs141683794
rs17836435
All − MR Egger
All − Inverse variance weighted
−0.5
0.0
0.5
MR effect size for
'GCST90200000' on 'Calculus of kidney and ureter'

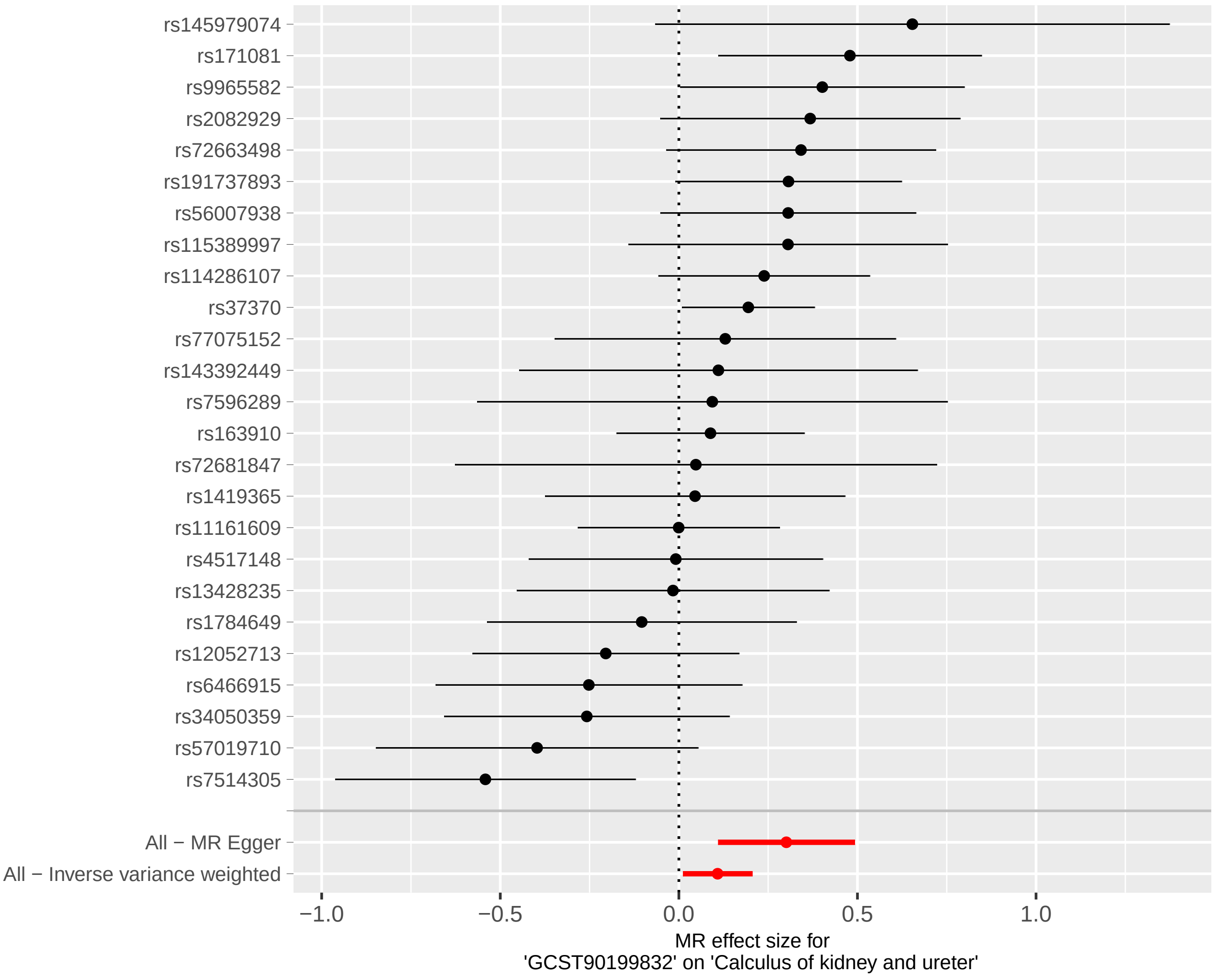

rs145979074
rs171081
rs9965582
rs2082929
rs72663498
rs191737893
rs56007938
rs115389997
rs114286107
rs37370
rs77075152
rs143392449
rs7596289
rs163910
rs72681847
rs1419365
rs11161609
rs4517148
rs13428235
rs1784649
rs12052713
rs6466915
rs34050359
rs57019710
rs7514305
All − MR Egger
All − Inverse variance weighted
−1.0
−0.5
0.0
0.5
1.0
MR effect size for
'GCST90199832' on 'Calculus of kidney and ureter'

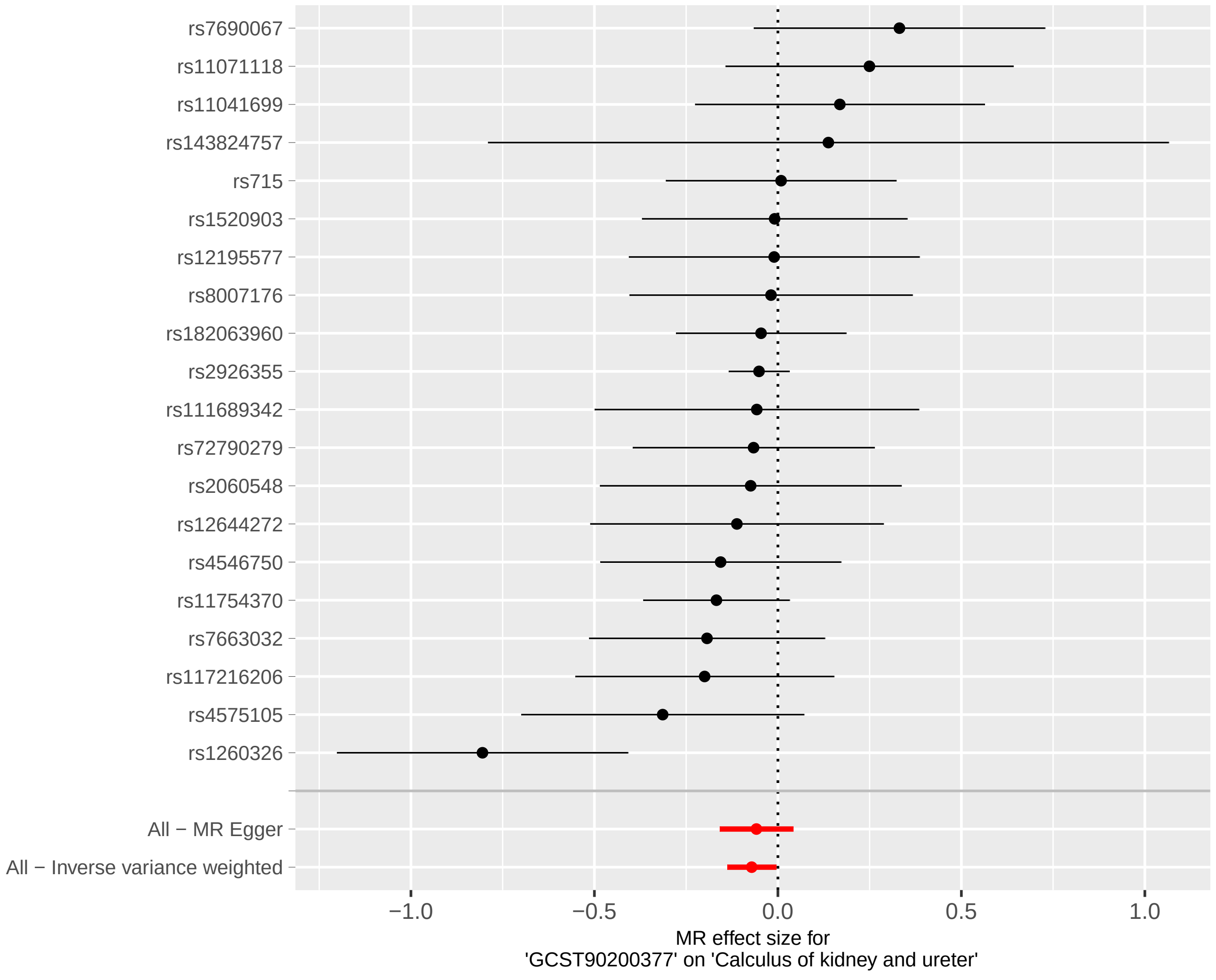

rs7690067
rs11071118
rs11041699
rs143824757
rs715
rs1520903
rs12195577
rs8007176
rs182063960
rs2926355
rs111689342
rs72790279
rs2060548
rs12644272
rs4546750
rs11754370
rs7663032
rs117216206
rs4575105
rs1260326
All − MR Egger
All − Inverse variance weighted
−1.0
−0.5
0.0
0.5
1.0
MR effect size for
'GCST90200377' on 'Calculus of kidney and ureter'

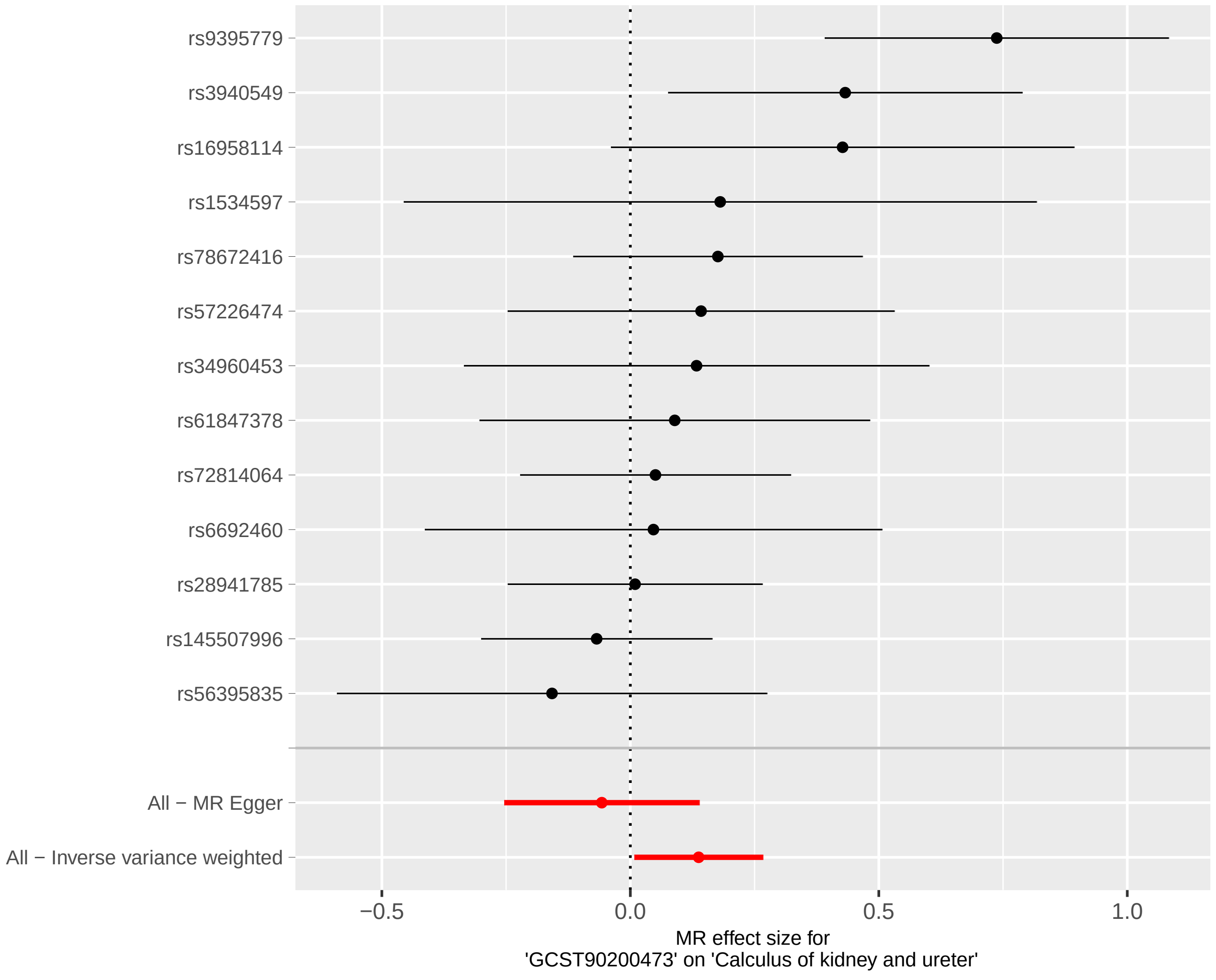
rs9395779
rs3940549
rs16958114
rs1534597
rs78672416
rs57226474
rs34960453
rs61847378
rs72814064
rs6692460
rs28941785
rs145507996
rs56395835
All − MR Egger
All − Inverse variance weighted
−0.5
0.0
0.5
1.0
MR effect size for
'GCST90200473' on 'Calculus of kidney and ureter'

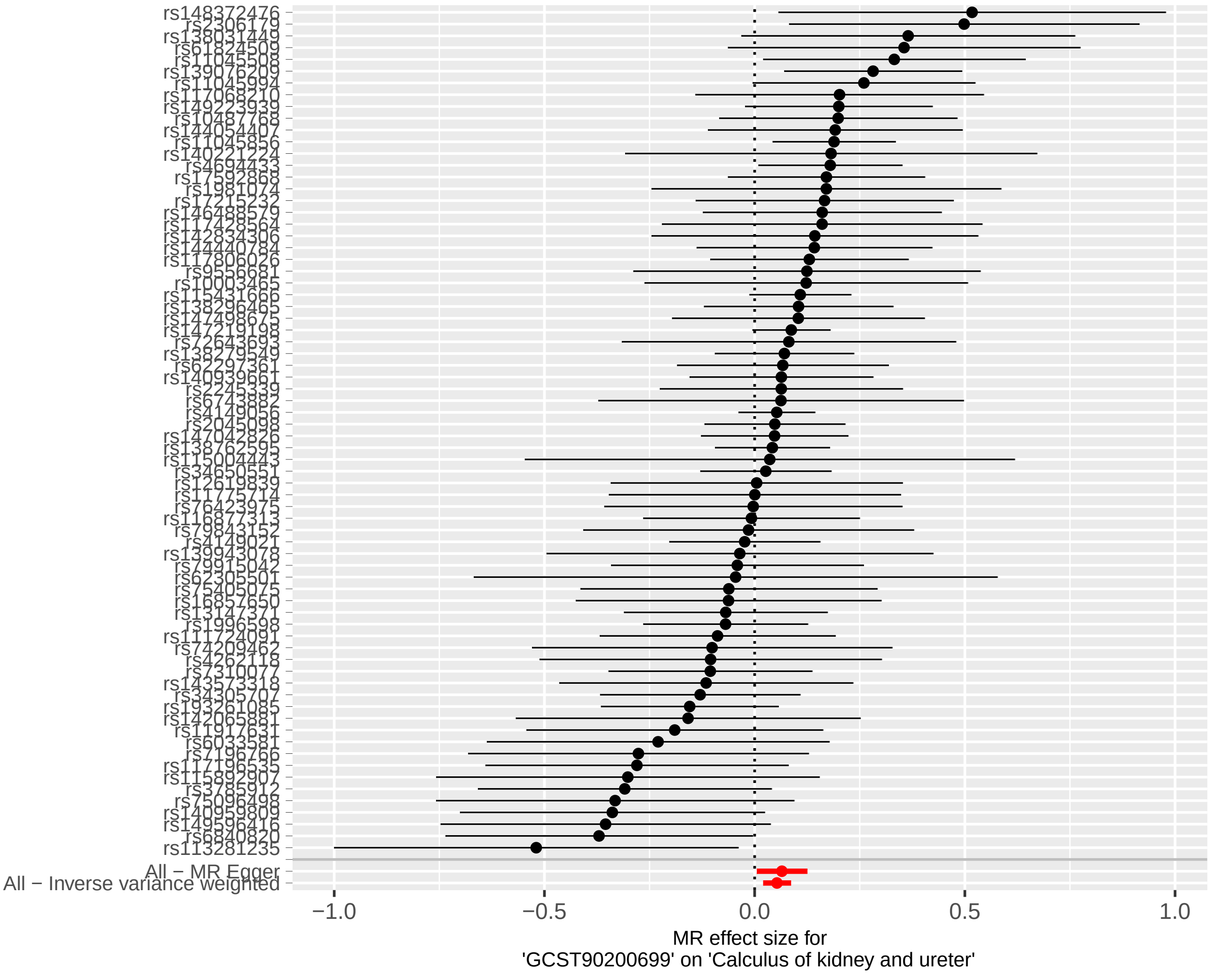
rs148372476
rs2306179
rs138031449
rs61824509
rs11045508
rs139076209
rs11045994
rs117068210
rs149223939
rs10487768
rs144054407
rs11045856
rs140221224
rs4694433
rs17592868
rs1981074
rs17215232
rs146488579
rs117428564
rs142834306
rs144440784
rs117806026
rs9556681
rs10003465
rs115431666
rs138296465
rs147498675
rs147219198
rs72643693
rs138279549
rs62297361
rs140939661
rs2245339
rs6743882
rs4149056
rs2045098
rs147042826
rs138762595
rs115004443
rs34650551
rs12619839
rs11775714
rs76423975
rs116877313
rs79843152
rs4149021
rs139943078
rs79915042
rs62305501
rs75405075
rs16857650
rs13147371
rs1996598
rs111724091
rs74209462
rs4262118
rs7310077
rs143573318
rs34305707
rs193261085
rs142065881
rs11917631
rs6033581
rs7196766
rs117196535
rs115892907
rs3785912
rs75096498
rs140959809
rs149596416
rs6840820
rs113281235
All − MR Egger
All − Inverse variance weighted
−1.0
−0.5
0.0
0.5
1.0
MR effect size for
'GCST90200699' on 'Calculus of kidney and ureter'

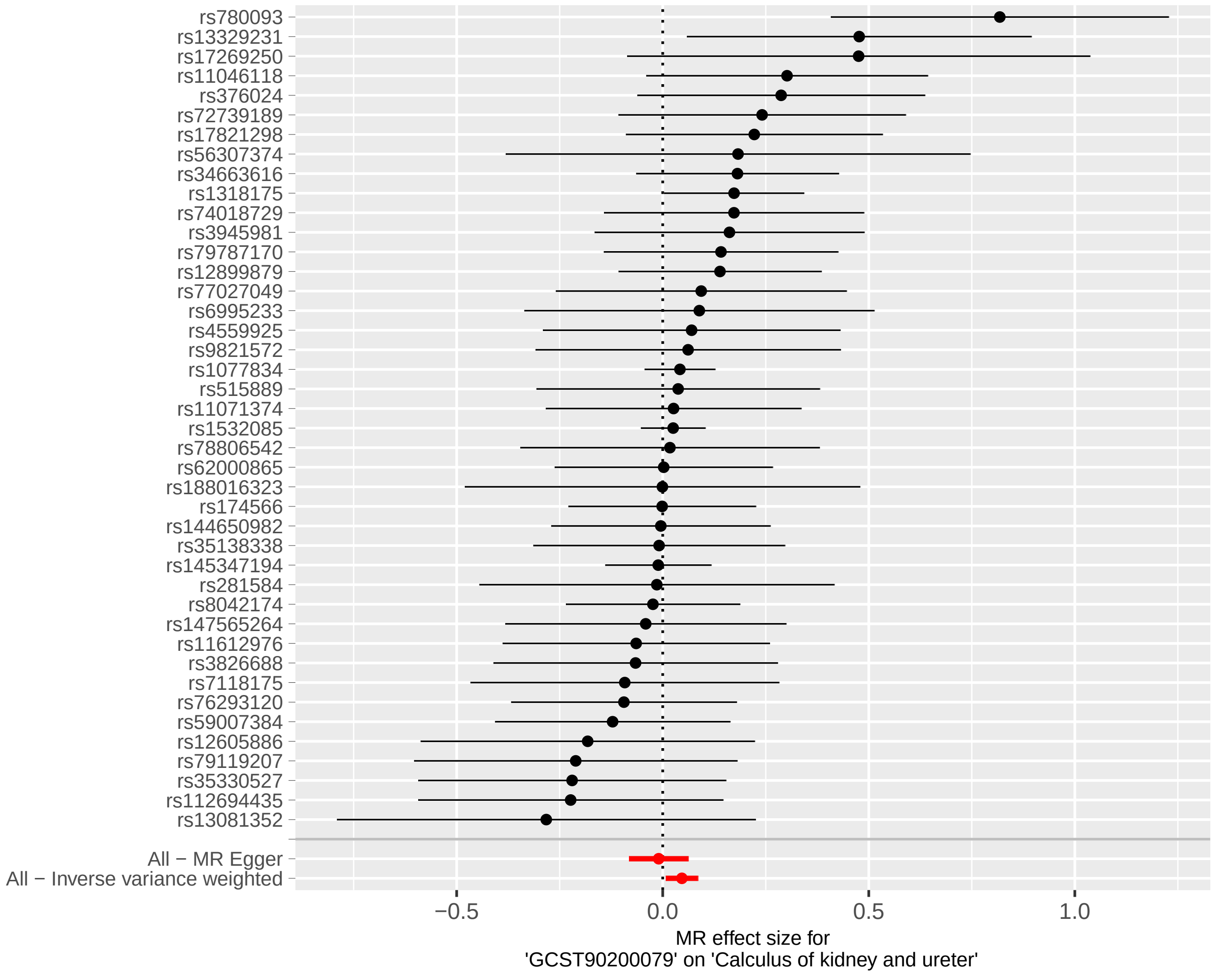
rs780093
rs13329231
rs17269250
rs11046118
rs376024
rs72739189
rs17821298
rs56307374
rs34663616
rs1318175
rs74018729
rs3945981
rs79787170
rs12899879
rs77027049
rs6995233
rs4559925
rs9821572
rs1077834
rs515889
rs11071374
rs1532085
rs78806542
rs62000865
rs188016323
rs174566
rs144650982
rs35138338
rs145347194
rs281584
rs8042174
rs147565264
rs11612976
rs3826688
rs7118175
rs76293120
rs59007384
rs12605886
rs79119207
rs35330527
rs112694435
rs13081352
All − MR Egger
All − Inverse variance weighted
−0.5
0.0
0.5
1.0
MR effect size for
'GCST90200079' on 'Calculus of kidney and ureter'

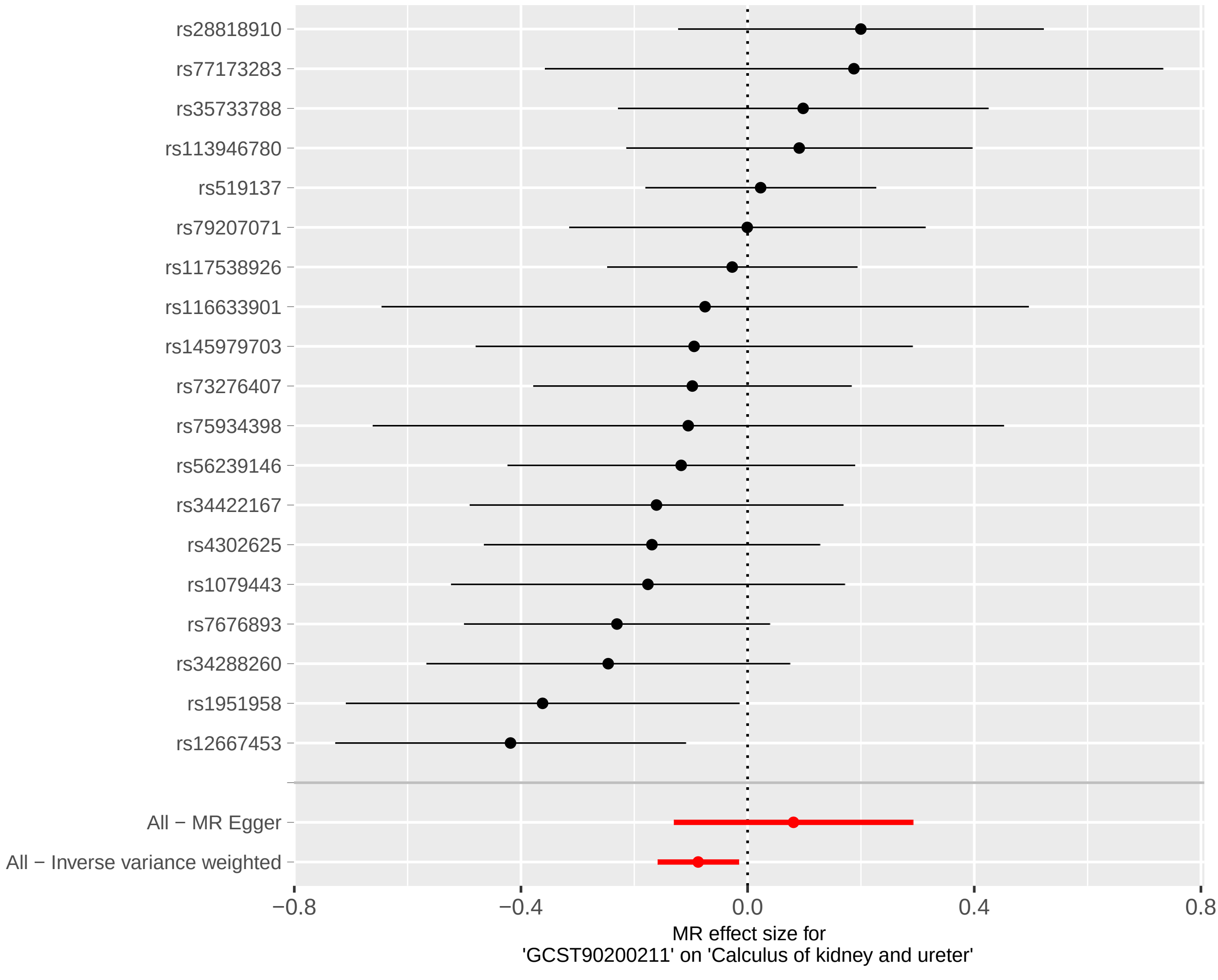
rs28818910
rs77173283
rs35733788
rs113946780
rs519137
rs79207071
rs117538926
rs116633901
rs145979703
rs73276407
rs75934398
rs56239146
rs34422167
rs4302625
rs1079443
rs7676893
rs34288260
rs1951958
rs12667453
All − MR Egger
All − Inverse variance weighted
−0.8
−0.4
0.0
0.4
0.8
MR effect size for
'GCST90200211' on 'Calculus of kidney and ureter'

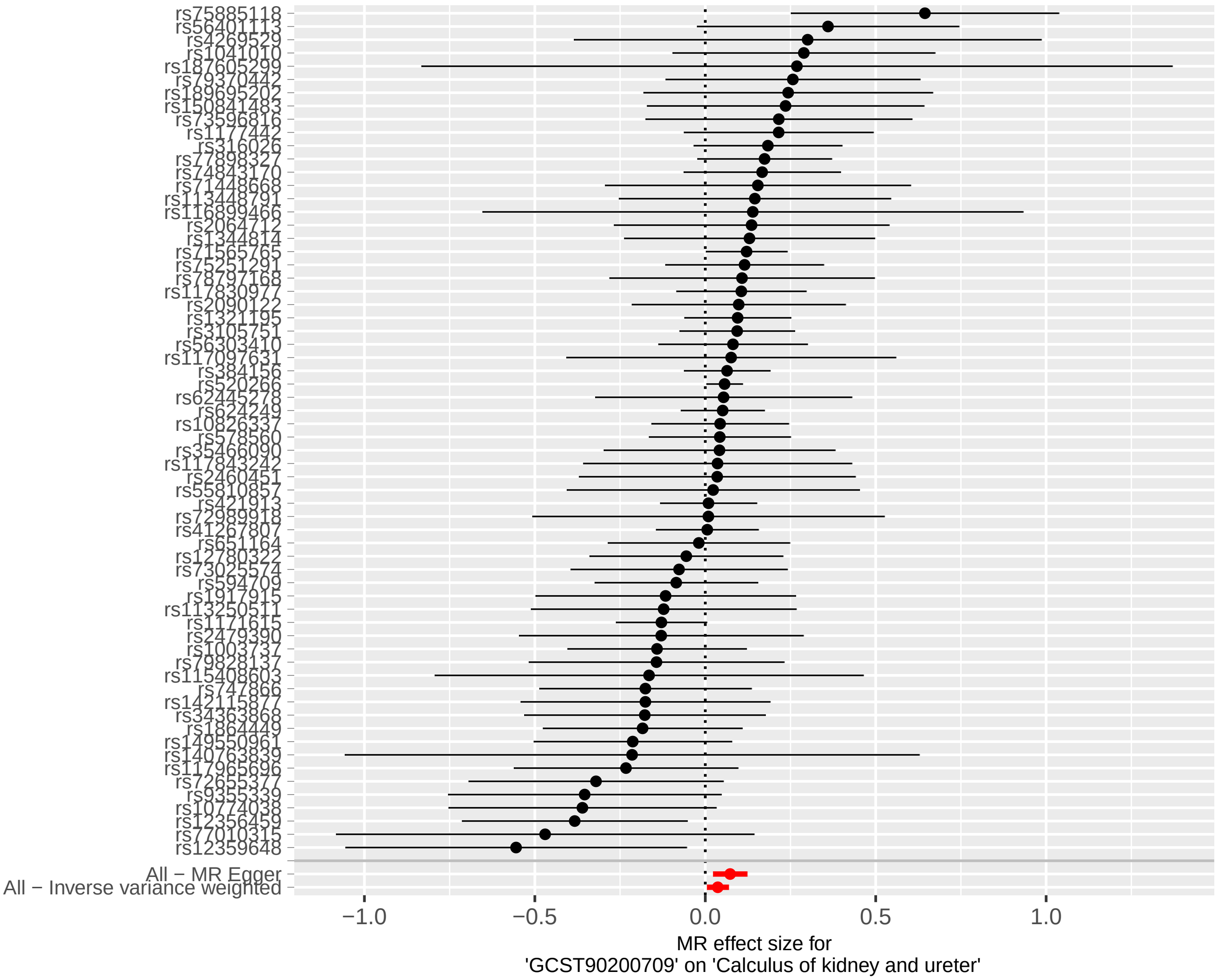
rs75885118
rs56401113
rs4269529
rs1041010
rs187605299
rs79370442
rs189695202
rs150841483
rs73596816
rs1177442
rs316026
rs77898327
rs74843170
rs71448668
rs113448791
rs116899466
rs2064712
rs1344814
rs71565765
rs75251291
rs78797168
rs117830977
rs2090122
rs1321195
rs3105751
rs56303410
rs117097631
rs384156
rs520266
rs62445278
rs624249
rs10826337
rs578560
rs35466090
rs117843242
rs2460451
rs55810857
rs421913
rs72989918
rs41267807
rs651164
rs12780322
rs73025574
rs594709
rs1917915
rs113250511
rs1171615
rs2479390
rs1003737
rs79828137
rs115408603
rs747866
rs142115877
rs34363868
rs1864449
rs149550961
rs140763839
rs117965696
rs72655377
rs9355339
rs10774038
rs12356459
rs77010315
rs12359648
All − MR Egger
All − Inverse variance weighted
−1.0
−0.5
0.0
0.5
1.0
MR effect size for
'GCST90200709' on 'Calculus of kidney and ureter'

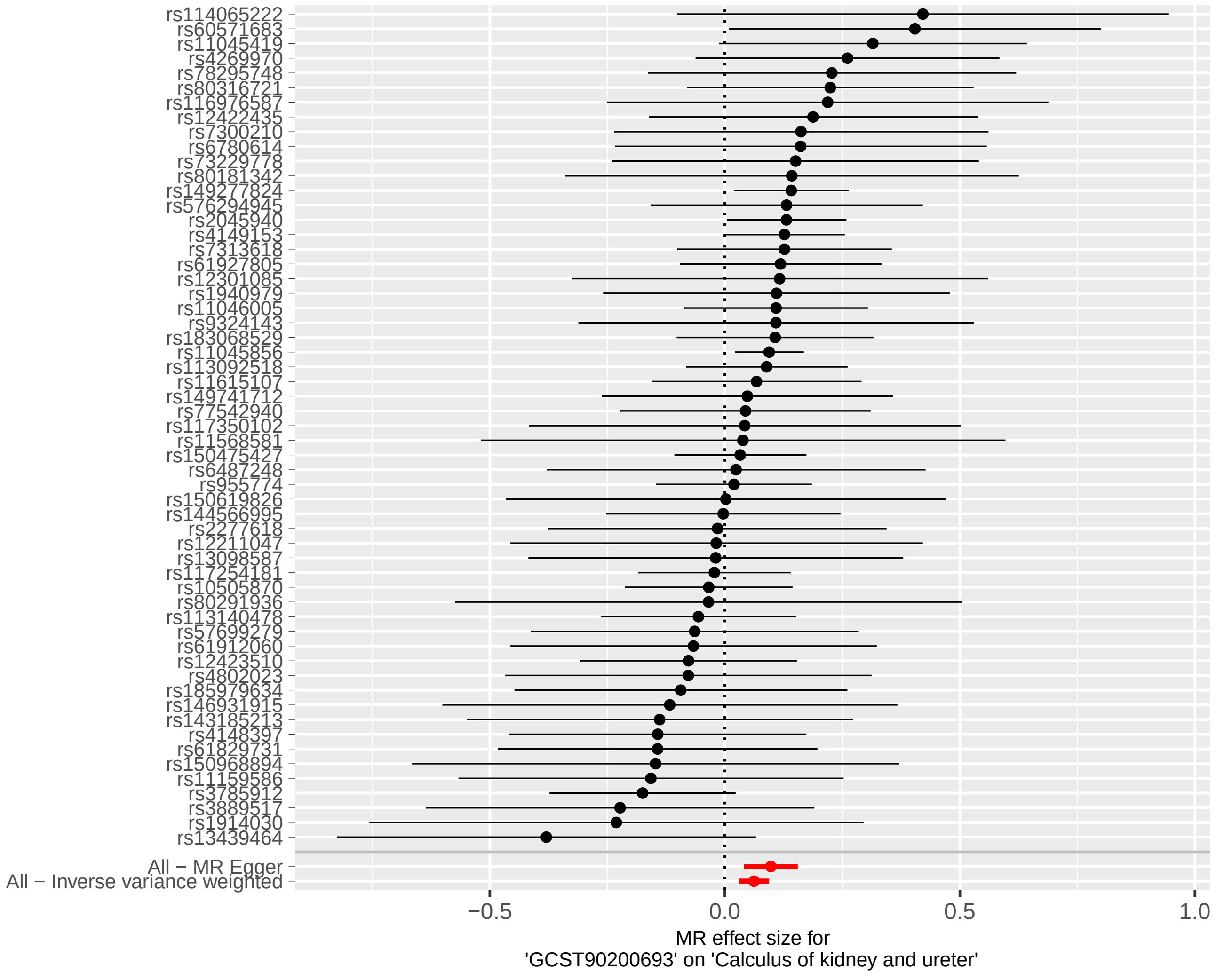

rs114065222
rs60571683
rs11045419
rs4269970
rs78295748
rs80316721
rs116976587
rs12422435
rs7300210
rs6780614
rs73229778
rs80181342
rs149277824
rs576294945
rs2045940
rs4149153
rs7313618
rs61927805
rs12301085
rs1940979
rs11046005
rs9324143
rs183068529
rs11045856
rs113092518
rs11615107
rs149741712
rs77542940
rs117350102
rs11568581
rs150475427
rs6487248
rs955774
rs150619826
rs144566995
rs2277618
rs12211047
rs13098587
rs117254181
rs10505870
rs80291936
rs113140478
rs57699279
rs61912060
rs12423510
rs4802023
rs185979634
rs146931915
rs143185213
rs4148397
rs61829731
rs150968894
rs11159586
rs3785912
rs3889517
rs1914030
rs13439464
All − MR Egger
All − Inverse variance weighted
−0.5
0.0
0.5
1.0
MR effect size for
'GCST90200693' on 'Calculus of kidney and ureter'

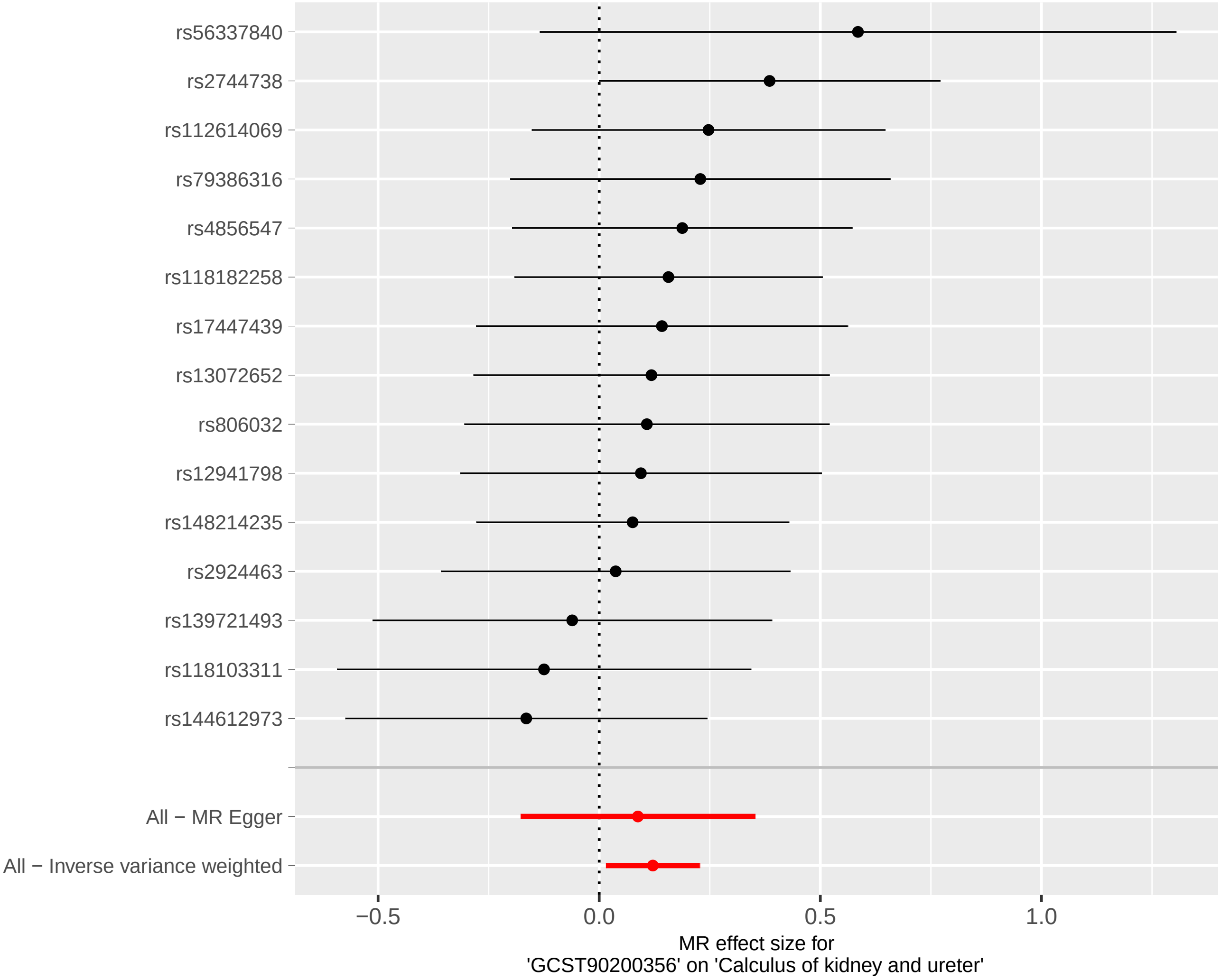
rs56337840
rs2744738
rs112614069
rs79386316
rs4856547
rs118182258
rs17447439
rs13072652
rs806032
rs12941798
rs148214235
rs2924463
rs139721493
rs118103311
rs144612973
All − MR Egger
All − Inverse variance weighted
−0.5
0.0
0.5
1.0
MR effect size for
'GCST90200356' on 'Calculus of kidney and ureter'

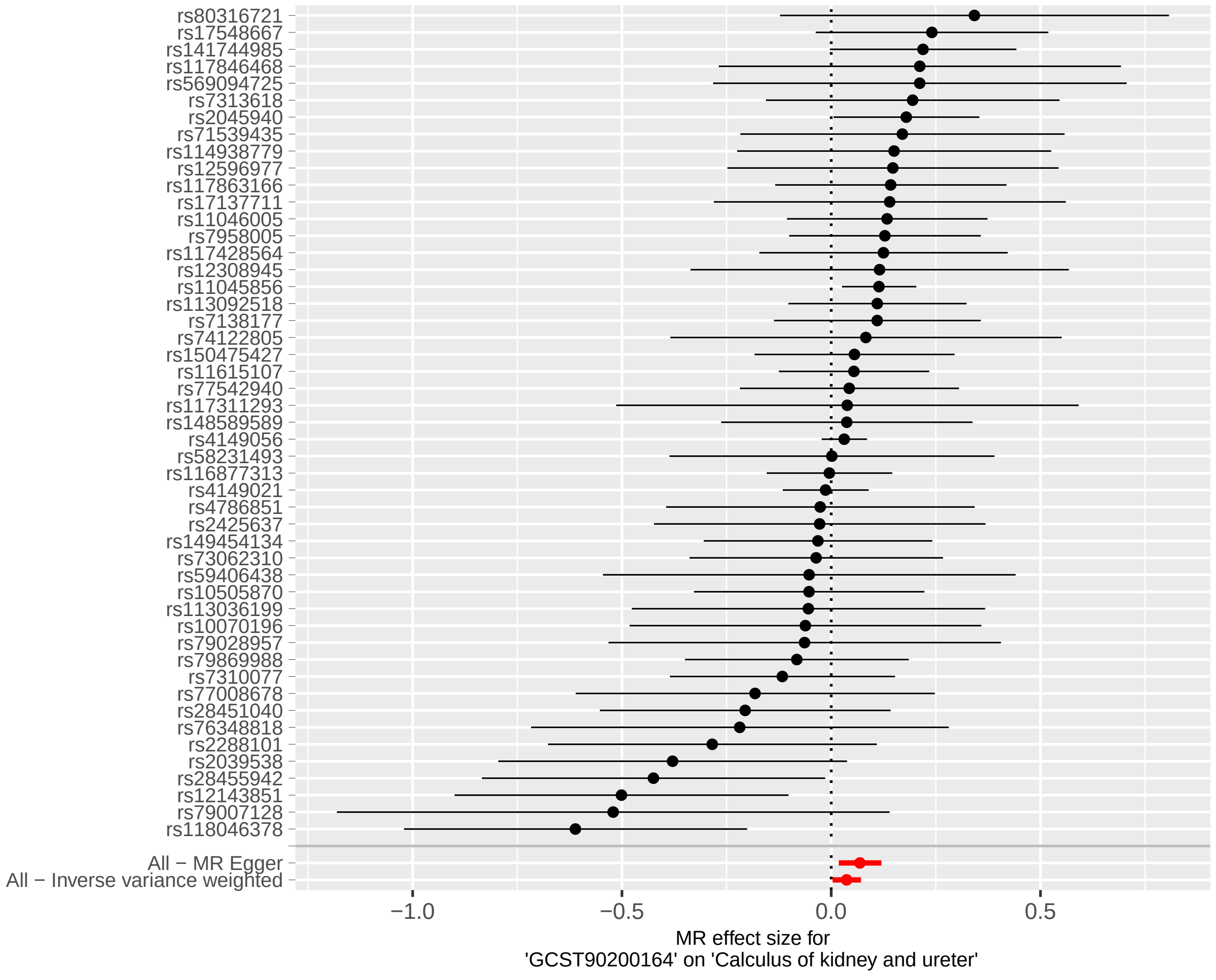

rs80316721
rs17548667
rs141744985
rs117846468
rs569094725
rs7313618
rs2045940
rs71539435
rs114938779
rs12596977
rs117863166
rs17137711
rs11046005
rs7958005
rs117428564
rs12308945
rs11045856
rs113092518
rs7138177
rs74122805
rs150475427
rs11615107
rs77542940
rs117311293
rs148589589
rs4149056
rs58231493
rs116877313
rs4149021
rs4786851
rs2425637
rs149454134
rs73062310
rs59406438
rs10505870
rs113036199
rs10070196
rs79028957
rs79869988
rs7310077
rs77008678
rs28451040
rs76348818
rs2288101
rs2039538
rs28455942
rs12143851
rs79007128
rs118046378
All − MR Egger
All − Inverse variance weighted
−1.0
−0.5
0.0
0.5
MR effect size for
'GCST90200164' on 'Calculus of kidney and ureter'

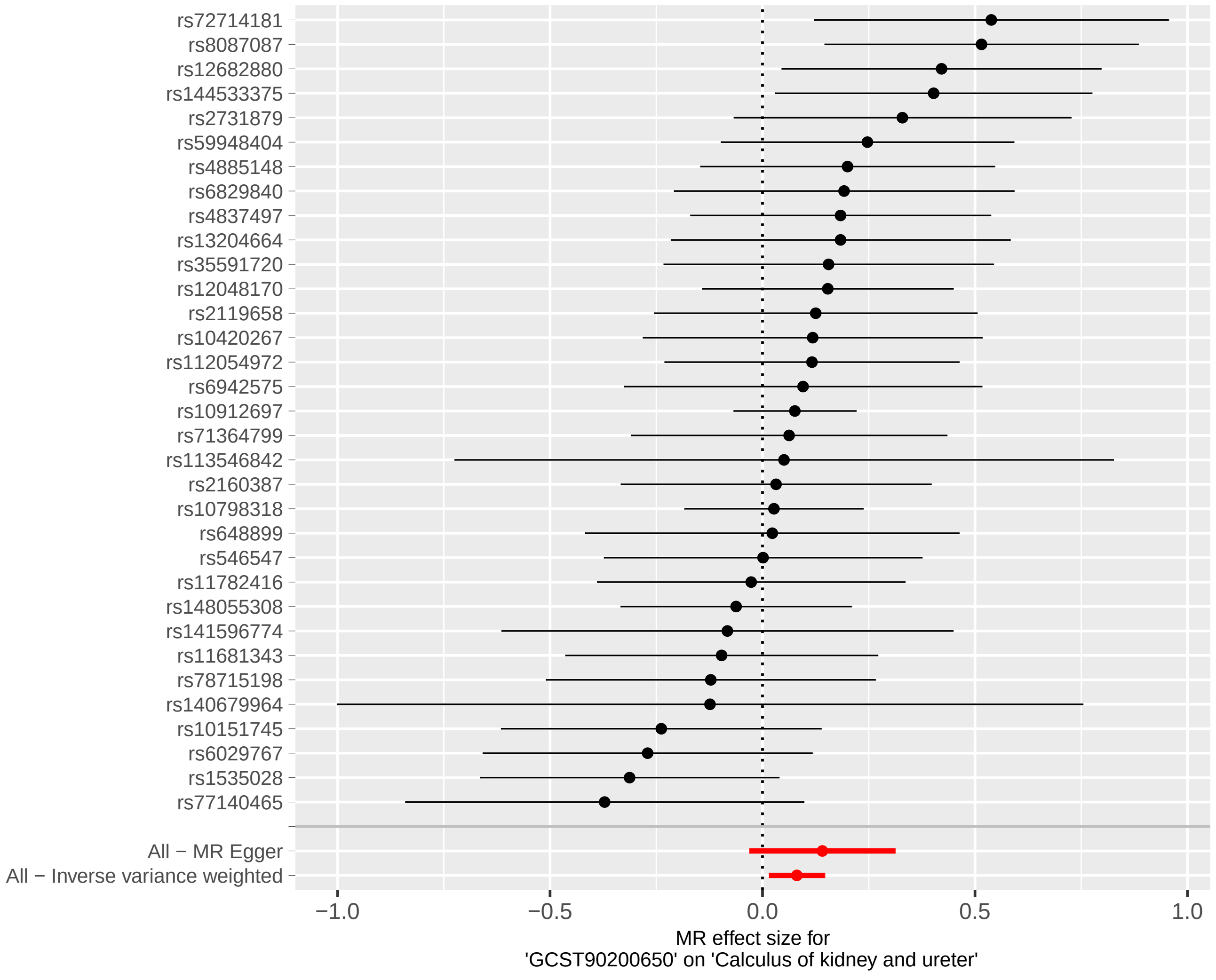

rs72714181
rs8087087
rs12682880
rs144533375
rs2731879
rs59948404
rs4885148
rs6829840
rs4837497
rs13204664
rs35591720
rs12048170
rs2119658
rs10420267
rs112054972
rs6942575
rs10912697
rs71364799
rs113546842
rs2160387
rs10798318
rs648899
rs546547
rs11782416
rs148055308
rs141596774
rs11681343
rs78715198
rs140679964
rs10151745
rs6029767
rs1535028
rs77140465
All − MR Egger
All − Inverse variance weighted
−1.0
−0.5
0.0
0.5
1.0
MR effect size for
'GCST90200650' on 'Calculus of kidney and ureter'

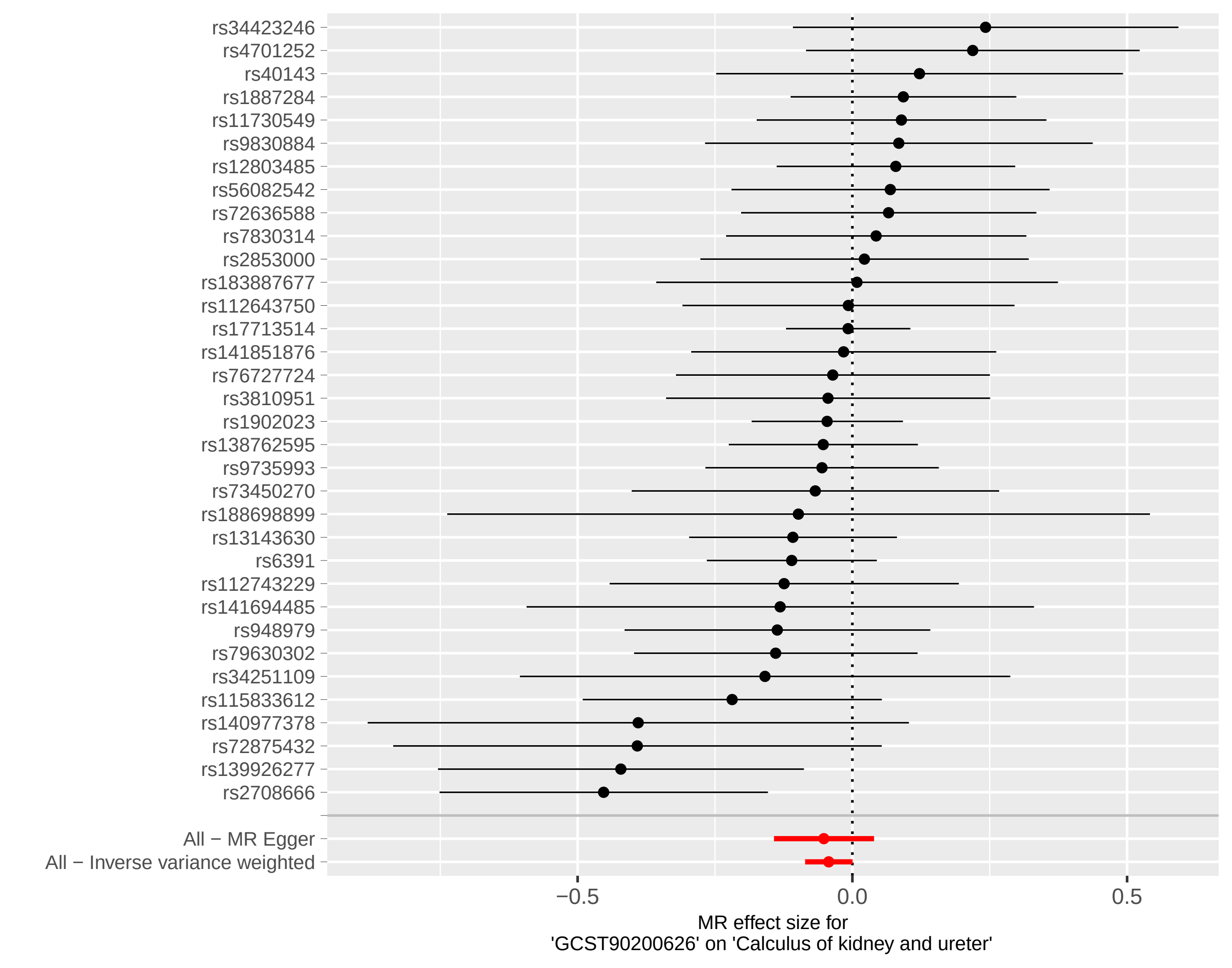
rs34423246
rs4701252
rs40143
rs1887284
rs11730549
rs9830884
rs12803485
rs56082542
rs72636588
rs7830314
rs2853000
rs183887677
rs112643750
rs17713514
rs141851876
rs76727724
rs3810951
rs1902023
rs138762595
rs9735993
rs73450270
rs188698899
rs13143630
rs6391
rs112743229
rs141694485
rs948979
rs79630302
rs34251109
rs115833612
rs140977378
rs72875432
rs139926277
rs2708666
All − MR Egger
All − Inverse variance weighted
−0.5
0.0
0.5
MR effect size for
'GCST90200626' on 'Calculus of kidney and ureter'

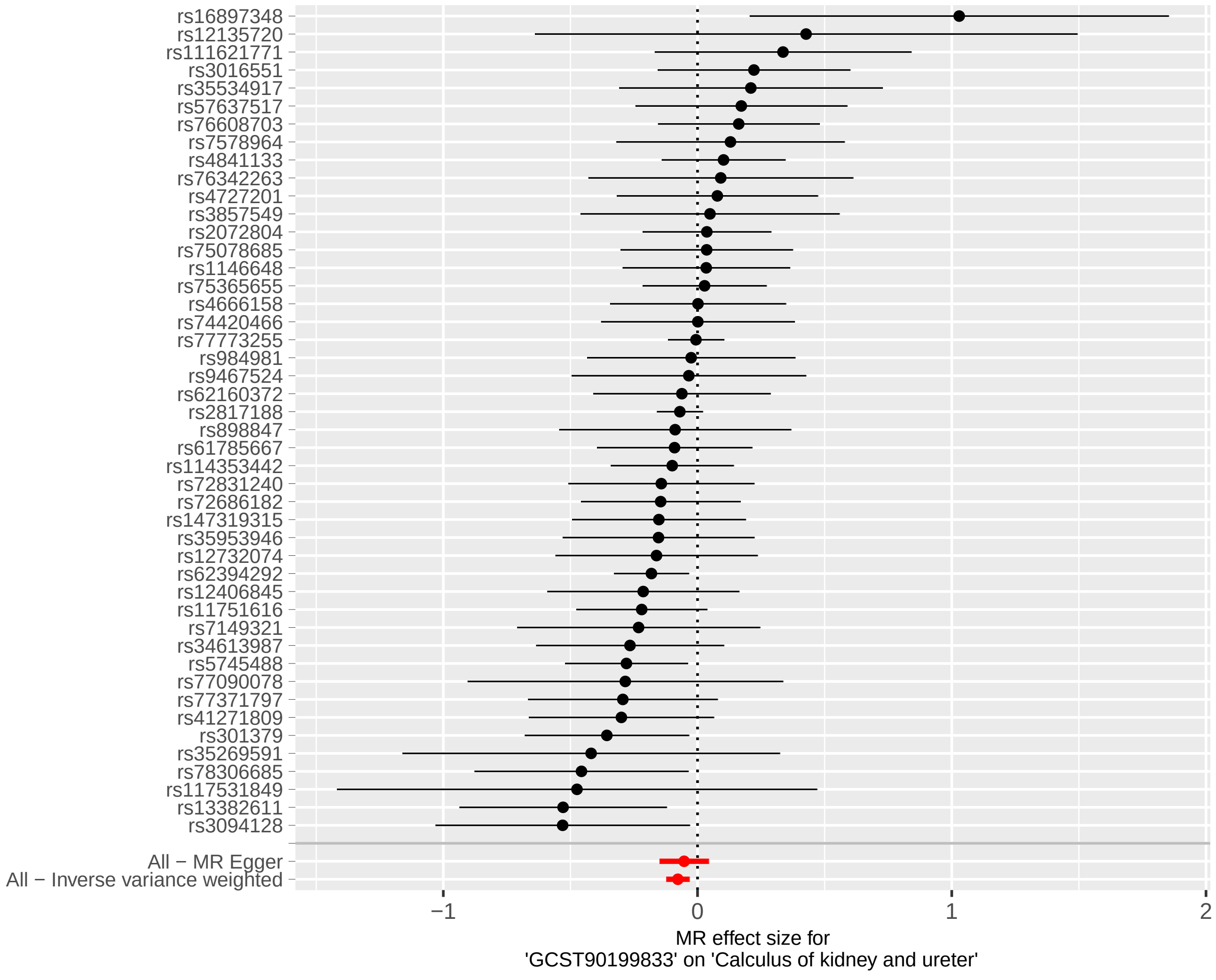
rs16897348
rs12135720
rs111621771
rs3016551
rs35534917
rs57637517
rs76608703
rs7578964
rs4841133
rs76342263
rs4727201
rs3857549
rs2072804
rs75078685
rs1146648
rs75365655
rs4666158
rs74420466
rs77773255
rs984981
rs9467524
rs62160372
rs2817188
rs898847
rs61785667
rs114353442
rs72831240
rs72686182
rs147319315
rs35953946
rs12732074
rs62394292
rs12406845
rs11751616
rs7149321
rs34613987
rs5745488
rs77090078
rs77371797
rs41271809
rs301379
rs35269591
rs78306685
rs117531849
rs13382611
rs3094128
All − MR Egger
All − Inverse variance weighted
−1
0
1
2
MR effect size for
'GCST90199833' on 'Calculus of kidney and ureter'

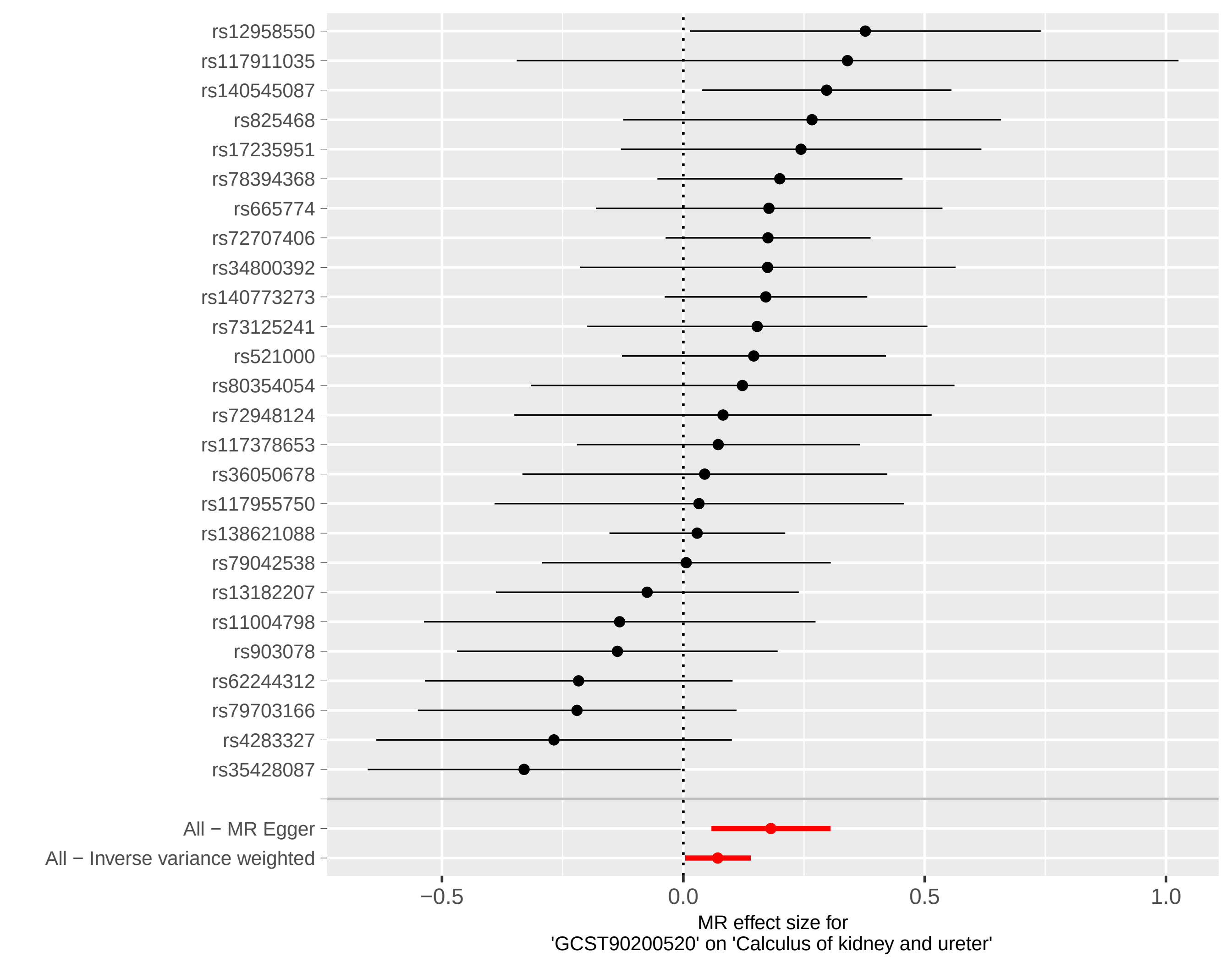
rs12958550
rs117911035
rs140545087
rs825468
rs17235951
rs78394368
rs665774
rs72707406
rs34800392
rs140773273
rs73125241
rs521000
rs80354054
rs72948124
rs117378653
rs36050678
rs117955750
rs138621088
rs79042538
rs13182207
rs11004798
rs903078
rs62244312
rs79703166
rs4283327
rs35428087
All − MR Egger
All − Inverse variance weighted
−0.5
0.0
0.5
1.0
MR effect size for
'GCST90200520' on 'Calculus of kidney and ureter'

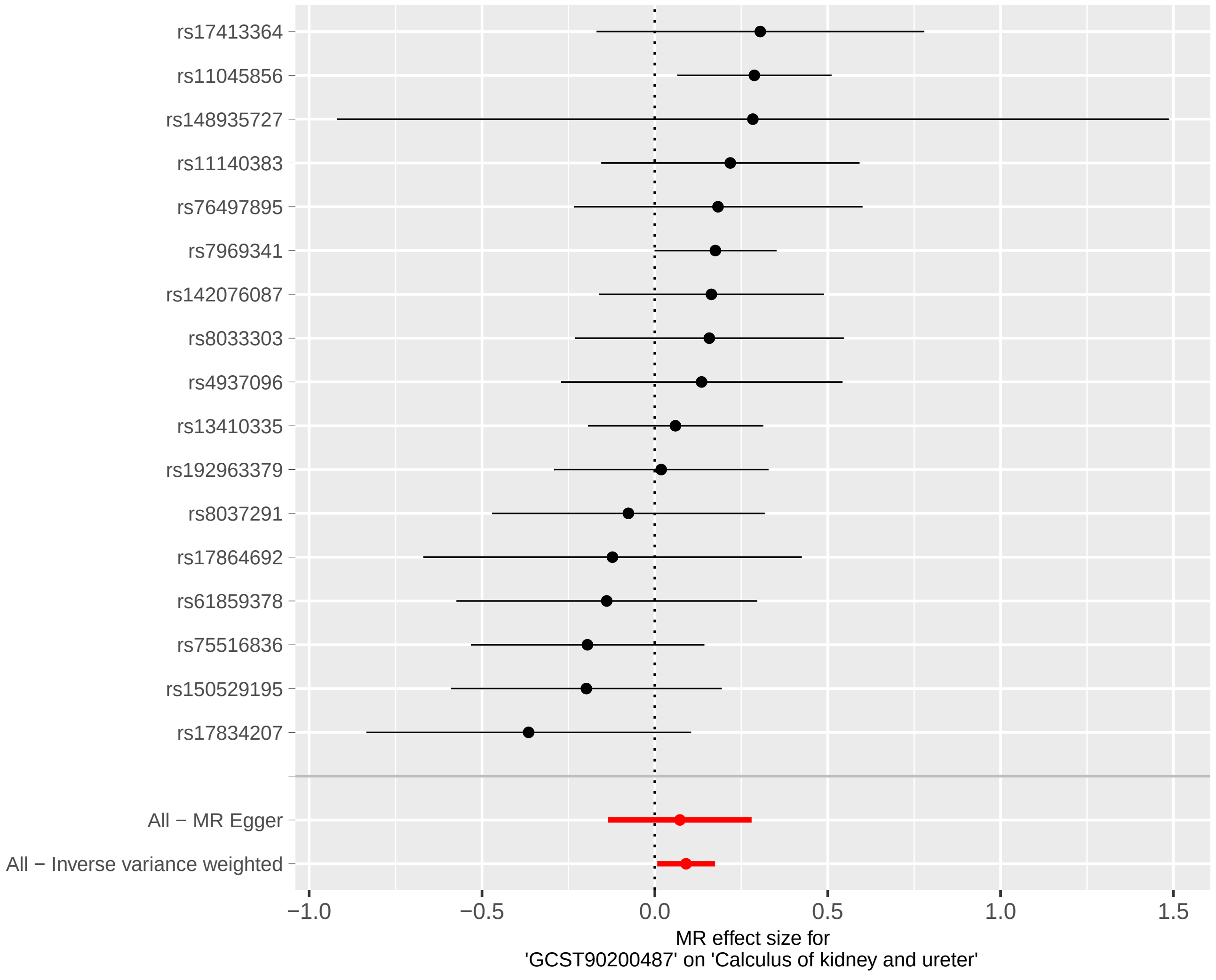
rs17413364
rs11045856
rs148935727
rs11140383
rs76497895
rs7969341
rs142076087
rs8033303
rs4937096
rs13410335
rs192963379
rs8037291
rs17864692
rs61859378
rs75516836
rs150529195
rs17834207
All − MR Egger
All − Inverse variance weighted
−1.0
−0.5
0.0
0.5
1.0
1.5
MR effect size for
'GCST90200487' on 'Calculus of kidney and ureter'

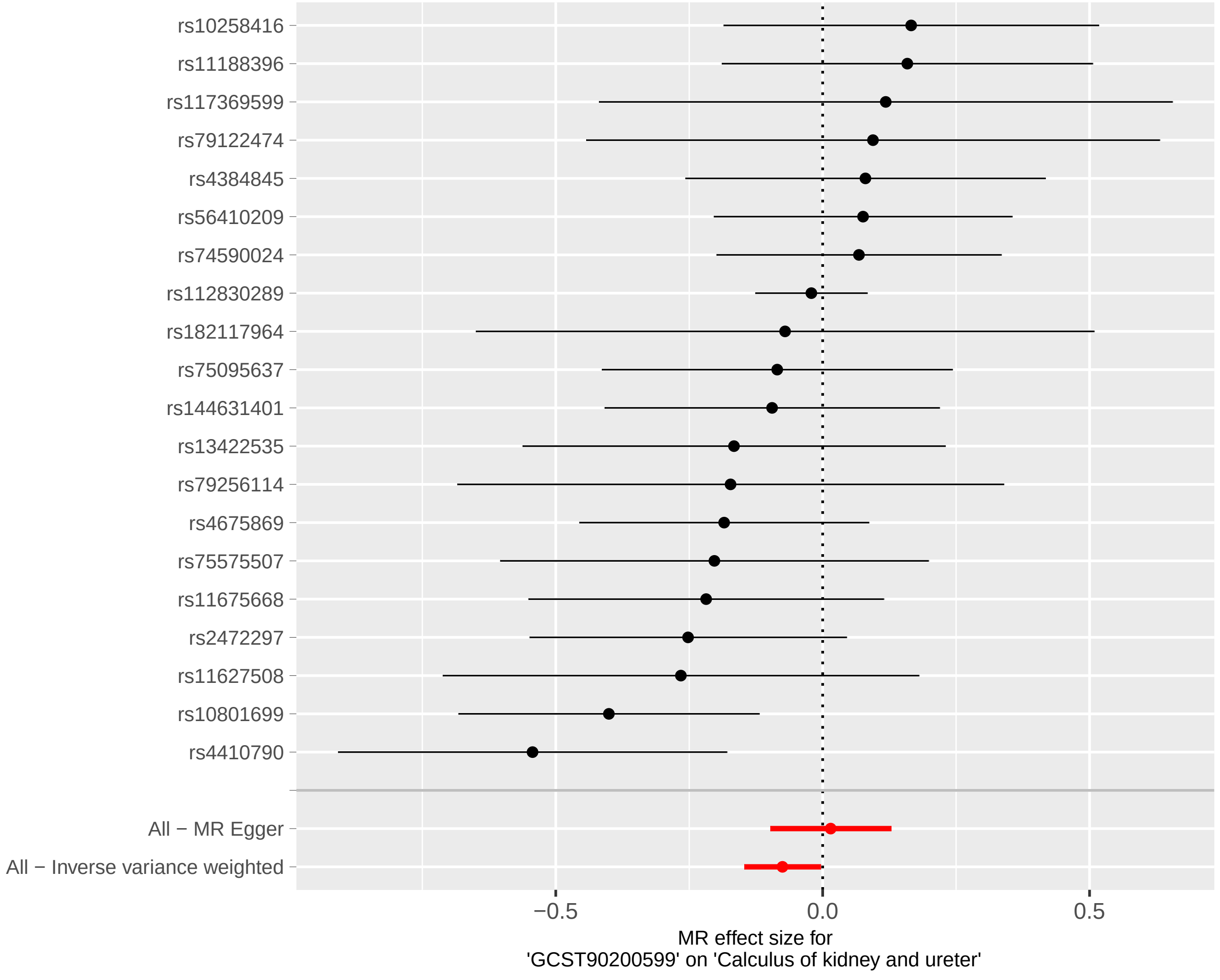

rs10258416
rs11188396
rs117369599
rs79122474
rs4384845
rs56410209
rs74590024
rs112830289
rs182117964
rs75095637
rs144631401
rs13422535
rs79256114
rs4675869
rs75575507
rs11675668
rs2472297
rs11627508
rs10801699
rs4410790
All − MR Egger
All − Inverse variance weighted
−0.5
0.0
0.5
MR effect size for
'GCST90200599' on 'Calculus of kidney and ureter'

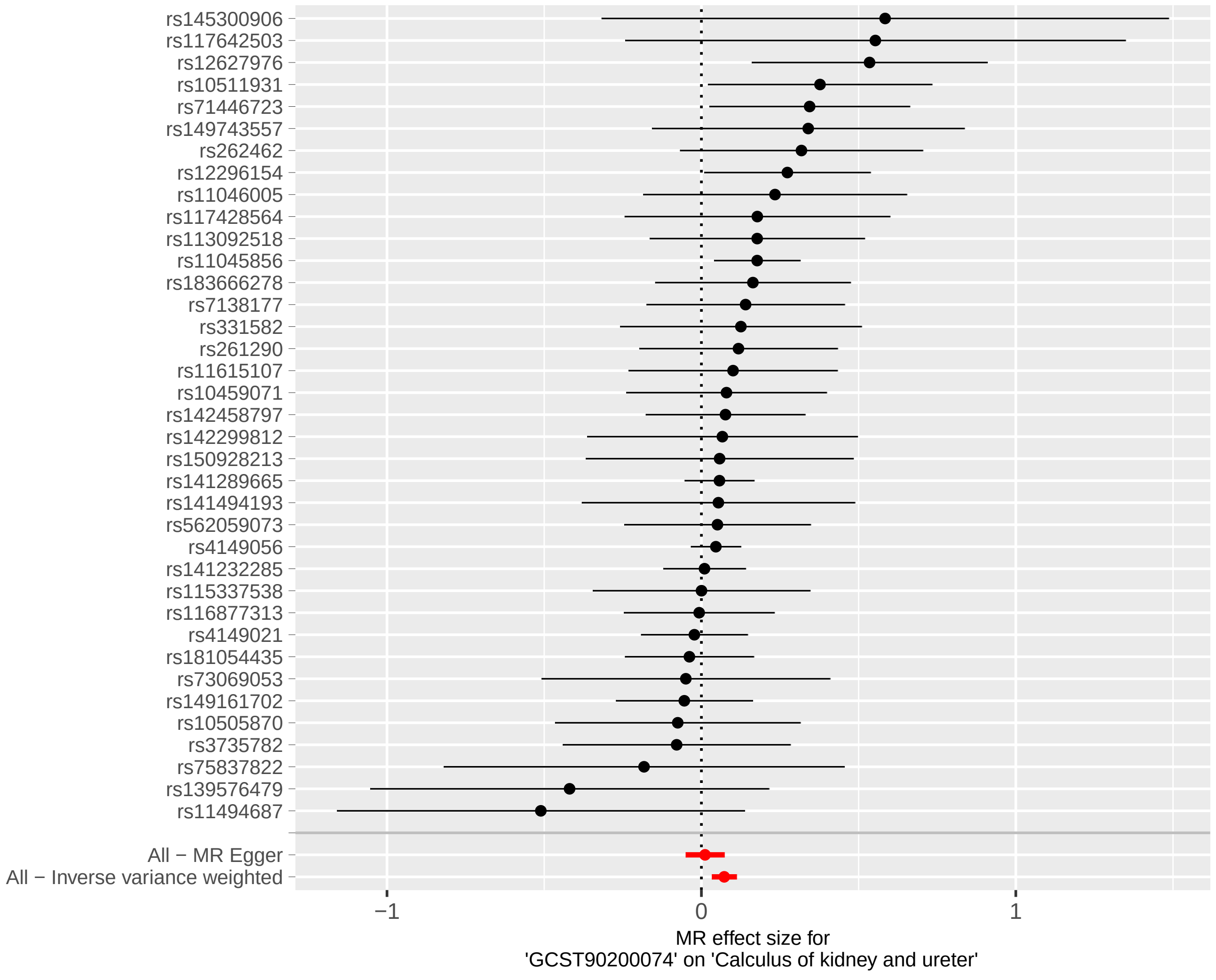
rs145300906
rs117642503
rs12627976
rs10511931
rs71446723
rs149743557
rs262462
rs12296154
rs11046005
rs117428564
rs113092518
rs11045856
rs183666278
rs7138177
rs331582
rs261290
rs11615107
rs10459071
rs142458797
rs142299812
rs150928213
rs141289665
rs141494193
rs562059073
rs4149056
rs141232285
rs115337538
rs116877313
rs4149021
rs181054435
rs73069053
rs149161702
rs10505870
rs3735782
rs75837822
rs139576479
rs11494687
All − MR Egger
All − Inverse variance weighted
−1
0
1
MR effect size for
'GCST90200074' on 'Calculus of kidney and ureter'

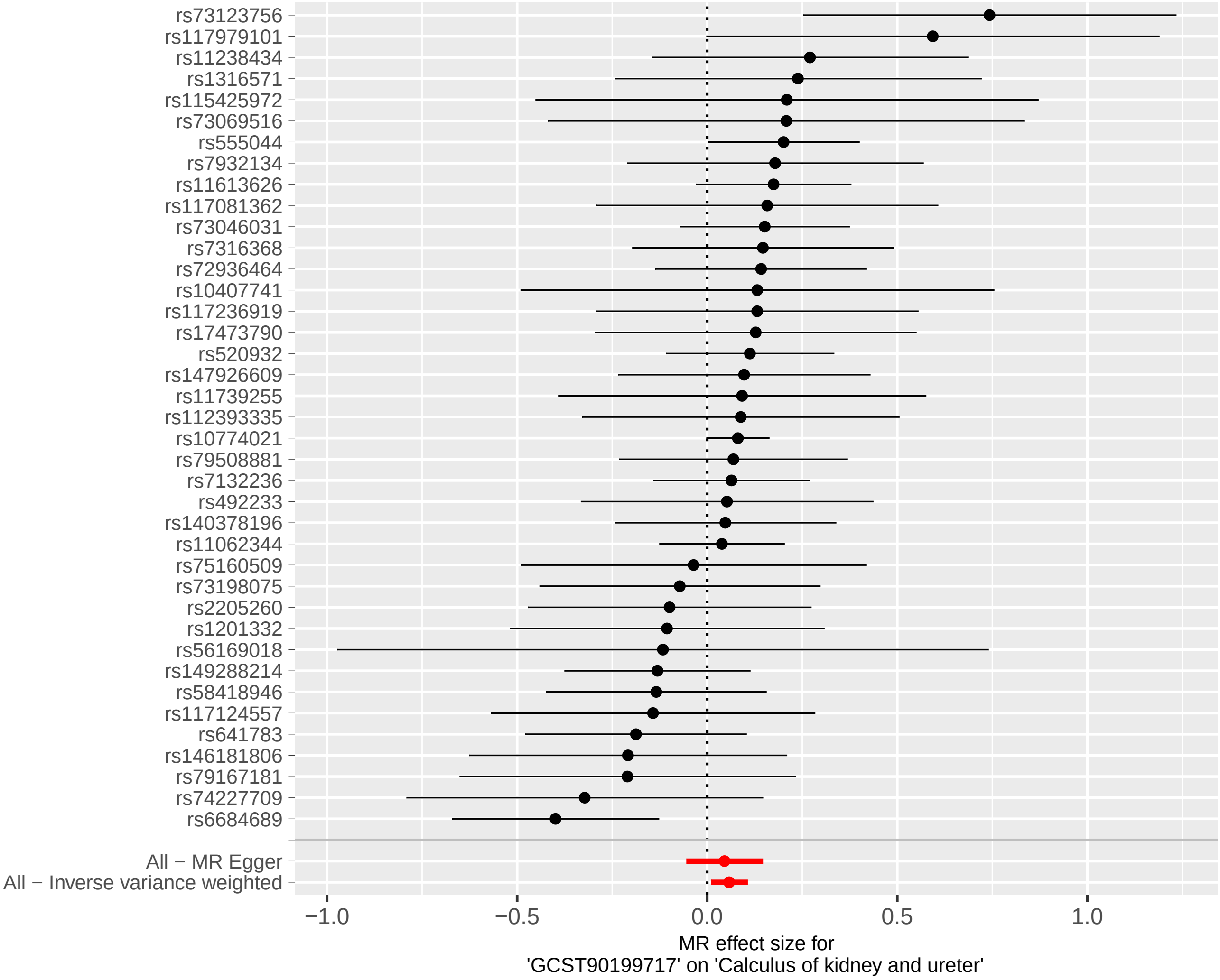
rs73123756
rs117979101
rs11238434
rs1316571
rs115425972
rs73069516
rs555044
rs7932134
rs11613626
rs117081362
rs73046031
rs7316368
rs72936464
rs10407741
rs117236919
rs17473790
rs520932
rs147926609
rs11739255
rs112393335
rs10774021
rs79508881
rs7132236
rs492233
rs140378196
rs11062344
rs75160509
rs73198075
rs2205260
rs1201332
rs56169018
rs149288214
rs58418946
rs117124557
rs641783
rs146181806
rs79167181
rs74227709
rs6684689
All − MR Egger
All − Inverse variance weighted
−1.0
−0.5
0.0
0.5
1.0
MR effect size for
'GCST90199717' on 'Calculus of kidney and ureter'

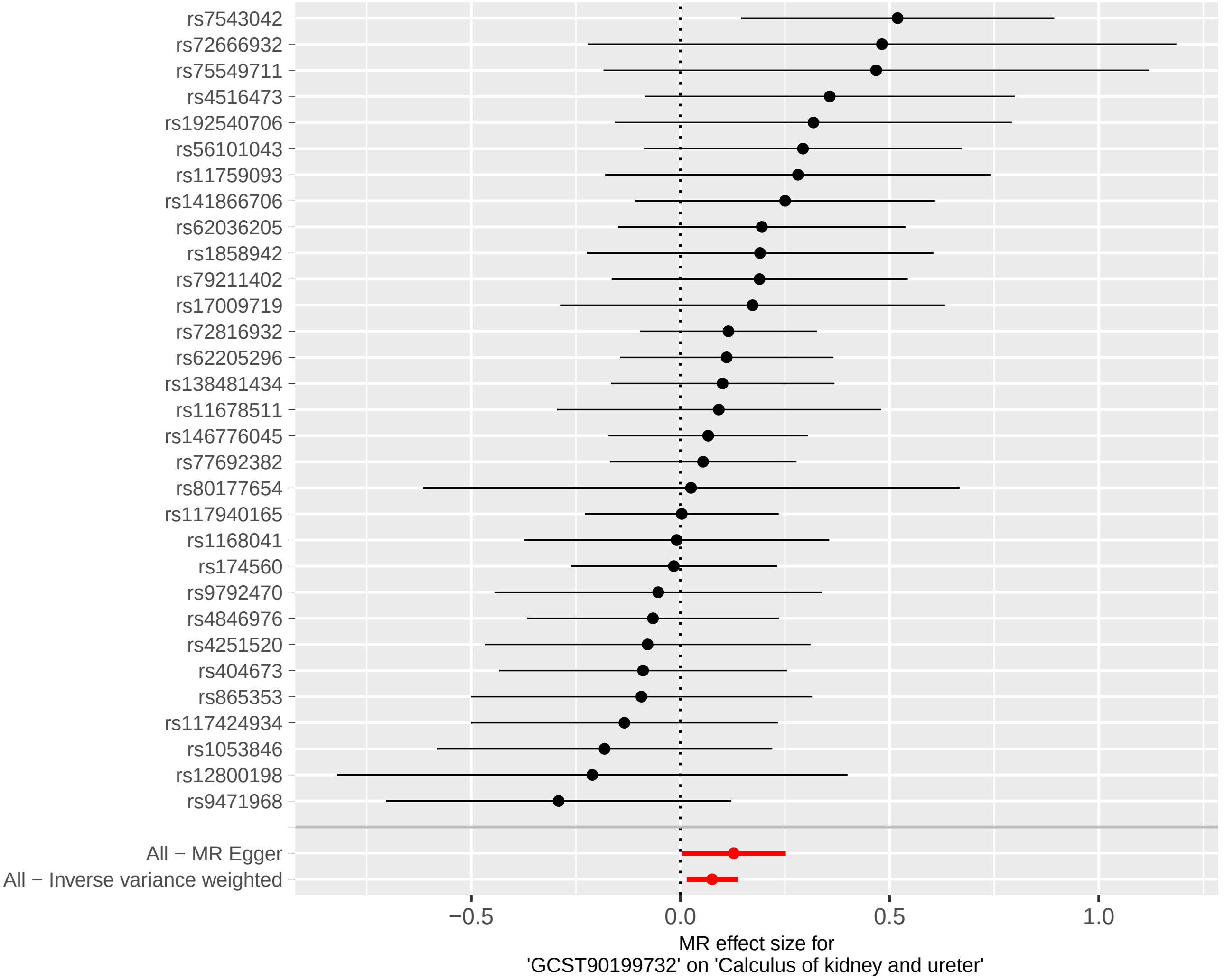
rs7543042
rs72666932
rs75549711
rs4516473
rs192540706
rs56101043
rs11759093
rs141866706
rs62036205
rs1858942
rs79211402
rs17009719
rs72816932
rs62205296
rs138481434
rs11678511
rs146776045
rs77692382
rs80177654
rs117940165
rs1168041
rs174560
rs9792470
rs4846976
rs4251520
rs404673
rs865353
rs117424934
rs1053846
rs12800198
rs9471968
All − MR Egger
All − Inverse variance weighted
−0.5
0.0
0.5
1.0
MR effect size for
'GCST90199732' on 'Calculus of kidney and ureter'

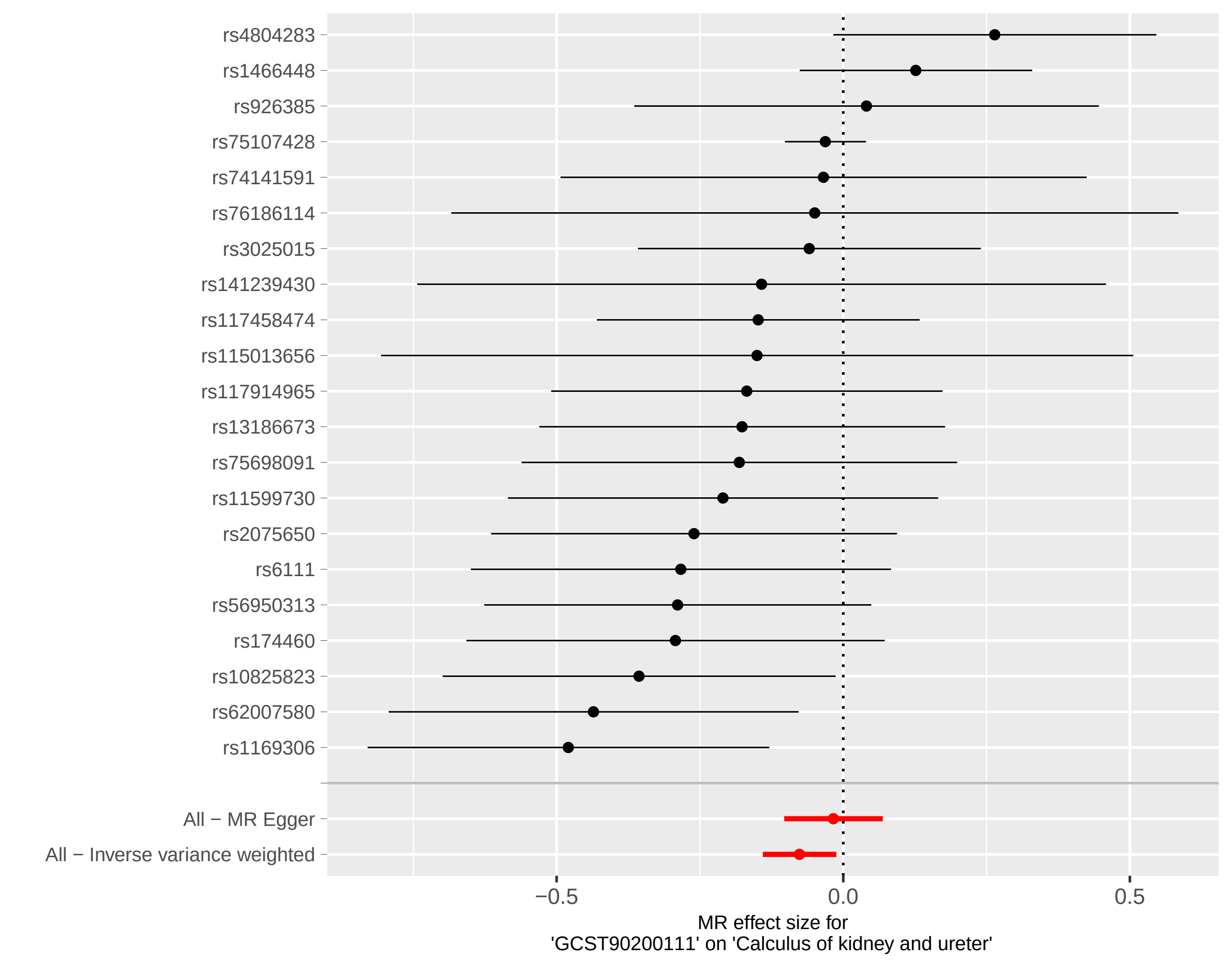
rs4804283
rs1466448
rs926385
rs75107428
rs74141591
rs76186114
rs3025015
rs141239430
rs117458474
rs115013656
rs117914965
rs13186673
rs75698091
rs11599730
rs2075650
rs6111
rs56950313
rs174460
rs10825823
rs62007580
rs1169306
All − MR Egger
All − Inverse variance weighted
−0.5
0.0
0.5
MR effect size for
'GCST90200111' on 'Calculus of kidney and ureter'

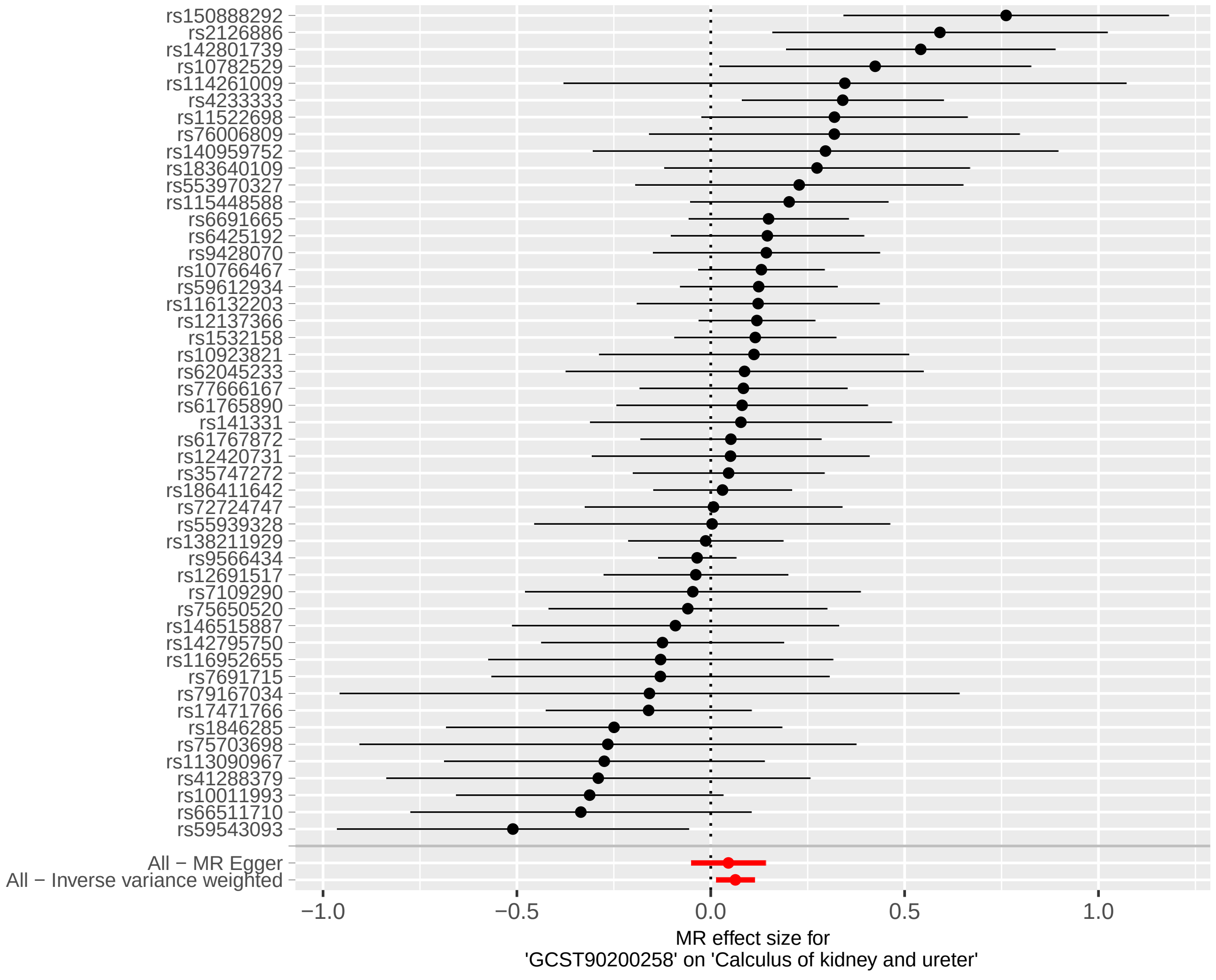
rs150888292
rs2126886
rs142801739
rs10782529
rs114261009
rs4233333
rs11522698
rs76006809
rs140959752
rs183640109
rs553970327
rs115448588
rs6691665
rs6425192
rs9428070
rs10766467
rs59612934
rs116132203
rs12137366
rs1532158
rs10923821
rs62045233
rs77666167
rs61765890
rs141331
rs61767872
rs12420731
rs35747272
rs186411642
rs72724747
rs55939328
rs138211929
rs9566434
rs12691517
rs7109290
rs75650520
rs146515887
rs142795750
rs116952655
rs7691715
rs79167034
rs17471766
rs1846285
rs75703698
rs113090967
rs41288379
rs10011993
rs66511710
rs59543093
All − MR Egger
All − Inverse variance weighted
−1.0
−0.5
0.0
0.5
1.0
MR effect size for
'GCST90200258' on 'Calculus of kidney and ureter'

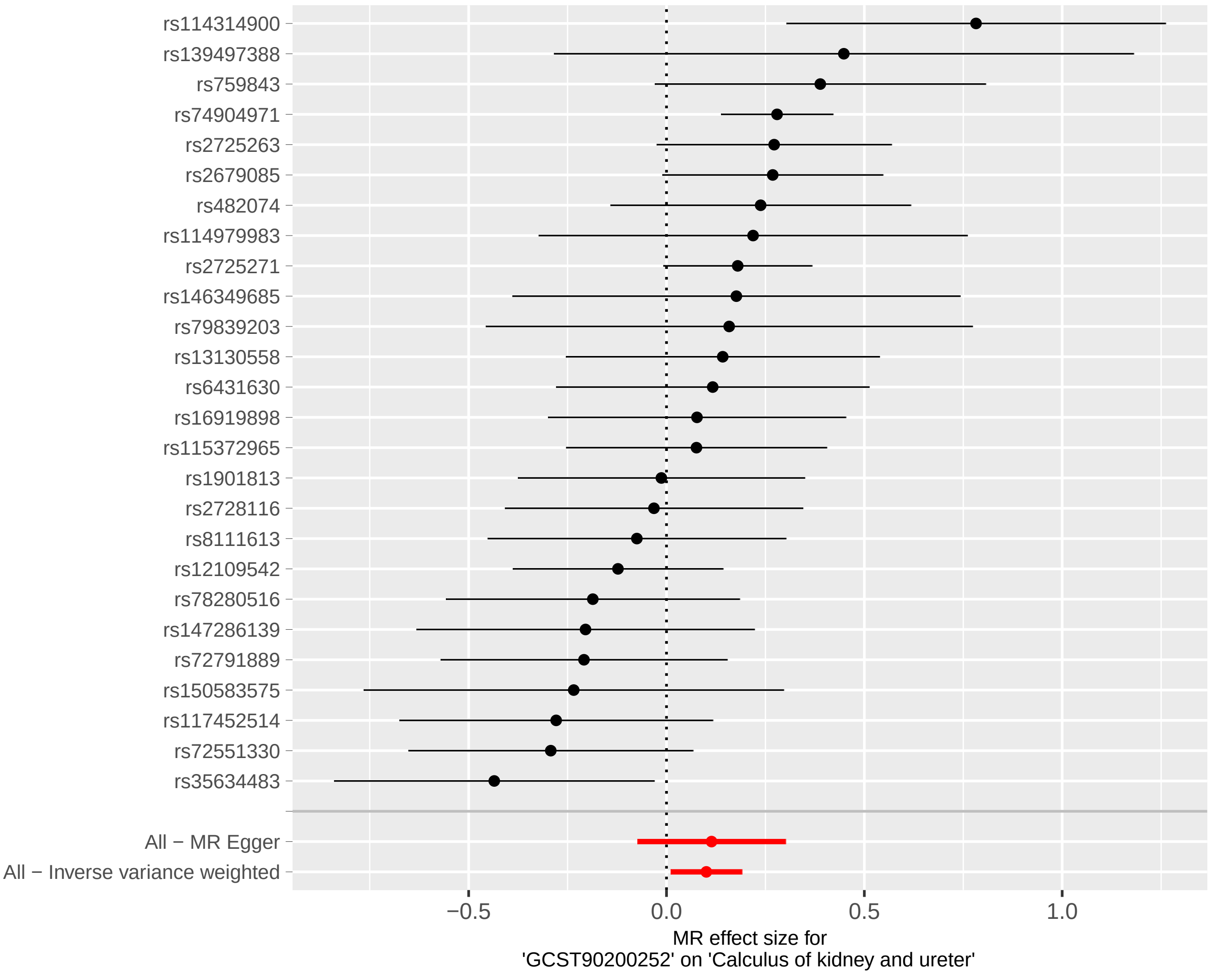

rs114314900
rs139497388
rs759843
rs74904971
rs2725263
rs2679085
rs482074
rs114979983
rs2725271
rs146349685
rs79839203
rs13130558
rs6431630
rs16919898
rs115372965
rs1901813
rs2728116
rs8111613
rs12109542
rs78280516
rs147286139
rs72791889
rs150583575
rs117452514
rs72551330
rs35634483
All − MR Egger
All − Inverse variance weighted
−0.5
0.0
0.5
1.0
MR effect size for
'GCST90200252' on 'Calculus of kidney and ureter'

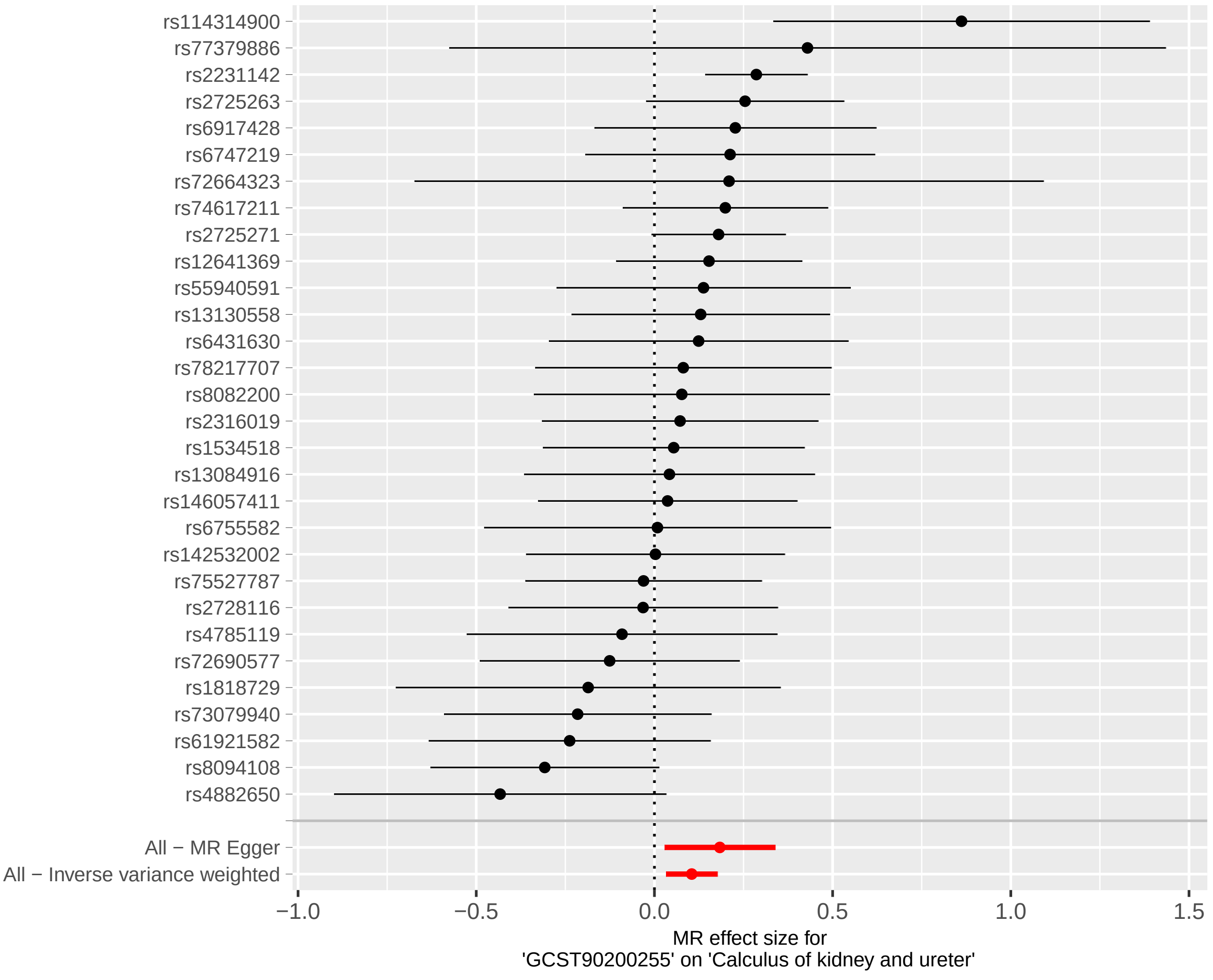
rs114314900
rs77379886
rs2231142
rs2725263
rs6917428
rs6747219
rs72664323
rs74617211
rs2725271
rs12641369
rs55940591
rs13130558
rs6431630
rs78217707
rs8082200
rs2316019
rs1534518
rs13084916
rs146057411
rs6755582
rs142532002
rs75527787
rs2728116
rs4785119
rs72690577
rs1818729
rs73079940
rs61921582
rs8094108
rs4882650
All − MR Egger
All − Inverse variance weighted
−1.0
−0.5
0.0
0.5
1.0
1.5
MR effect size for
'GCST90200255' on 'Calculus of kidney and ureter'

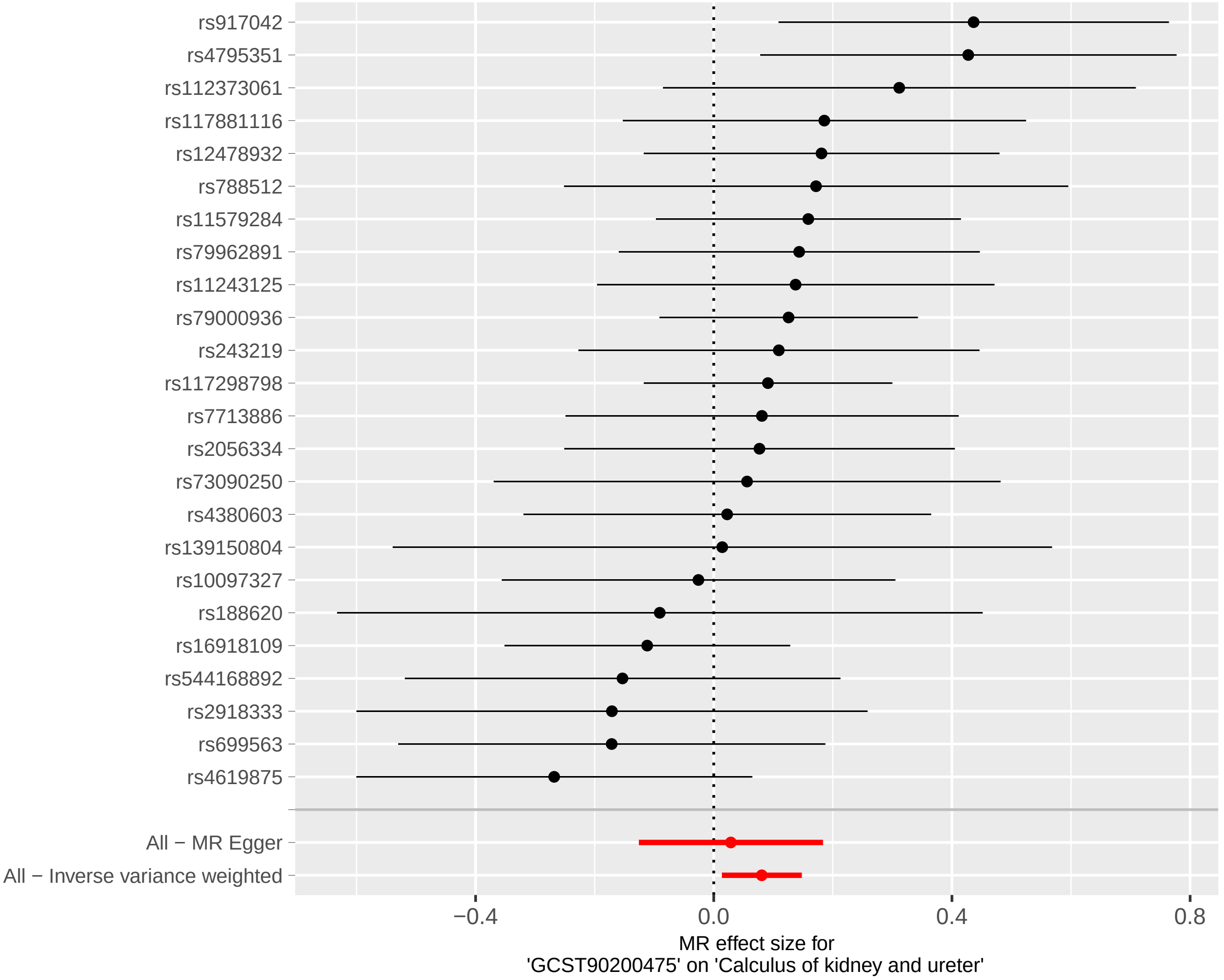
rs917042
rs4795351
rs112373061
rs117881116
rs12478932
rs788512
rs11579284
rs79962891
rs11243125
rs79000936
rs243219
rs117298798
rs7713886
rs2056334
rs73090250
rs4380603
rs139150804
rs10097327
rs188620
rs16918109
rs544168892
rs2918333
rs699563
rs4619875
All − MR Egger
All − Inverse variance weighted
−0.4
0.0
0.4
0.8
MR effect size for
'GCST90200475' on 'Calculus of kidney and ureter'

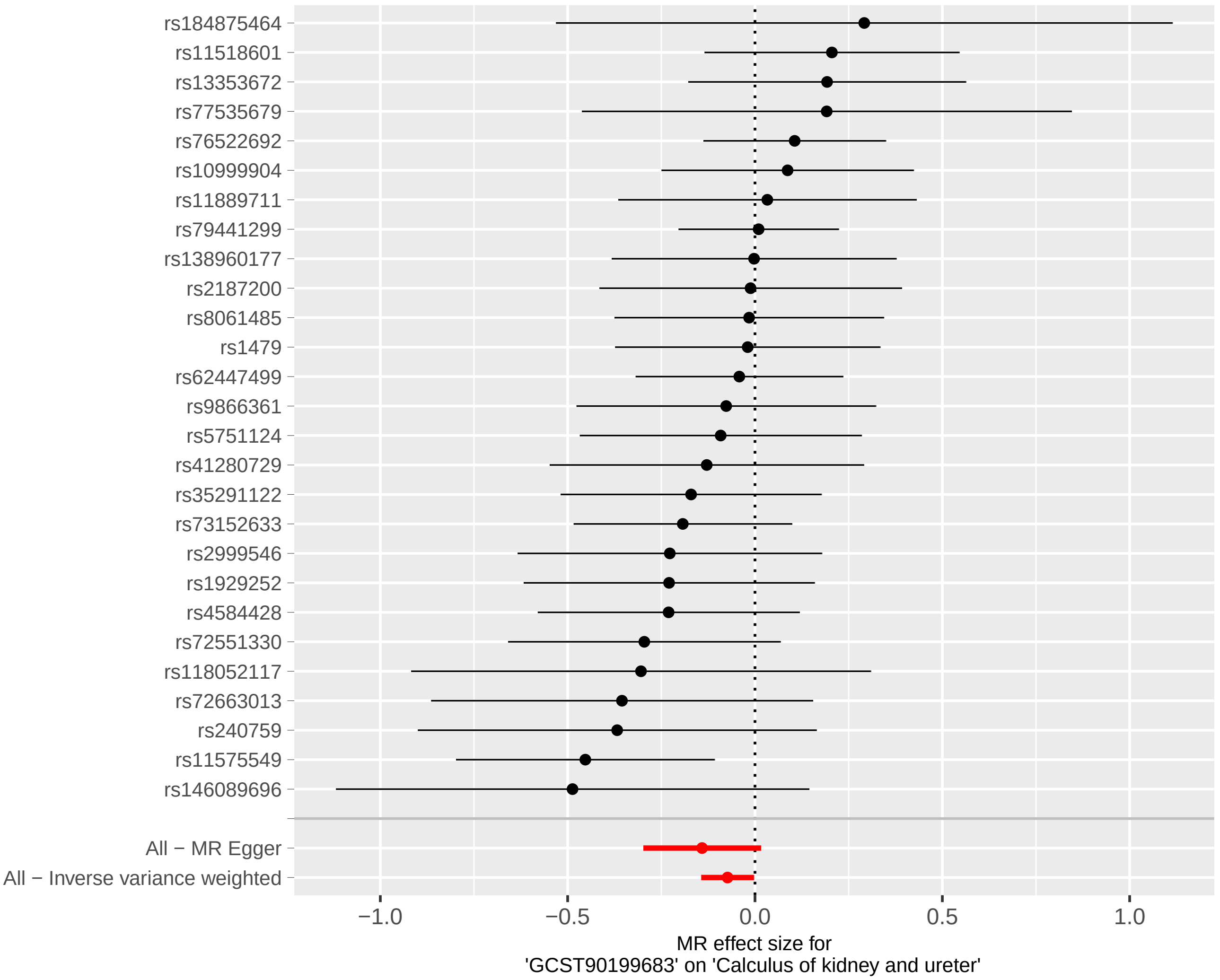
rs184875464
rs11518601
rs13353672
rs77535679
rs76522692
rs10999904
rs11889711
rs79441299
rs138960177
rs2187200
rs8061485
rs1479
rs62447499
rs9866361
rs5751124
rs41280729
rs35291122
rs73152633
rs2999546
rs1929252
rs4584428
rs72551330
rs118052117
rs72663013
rs240759
rs11575549
rs146089696
All − MR Egger
All − Inverse variance weighted
−1.0
−0.5
0.0
0.5
1.0
MR effect size for
'GCST90199683' on 'Calculus of kidney and ureter'

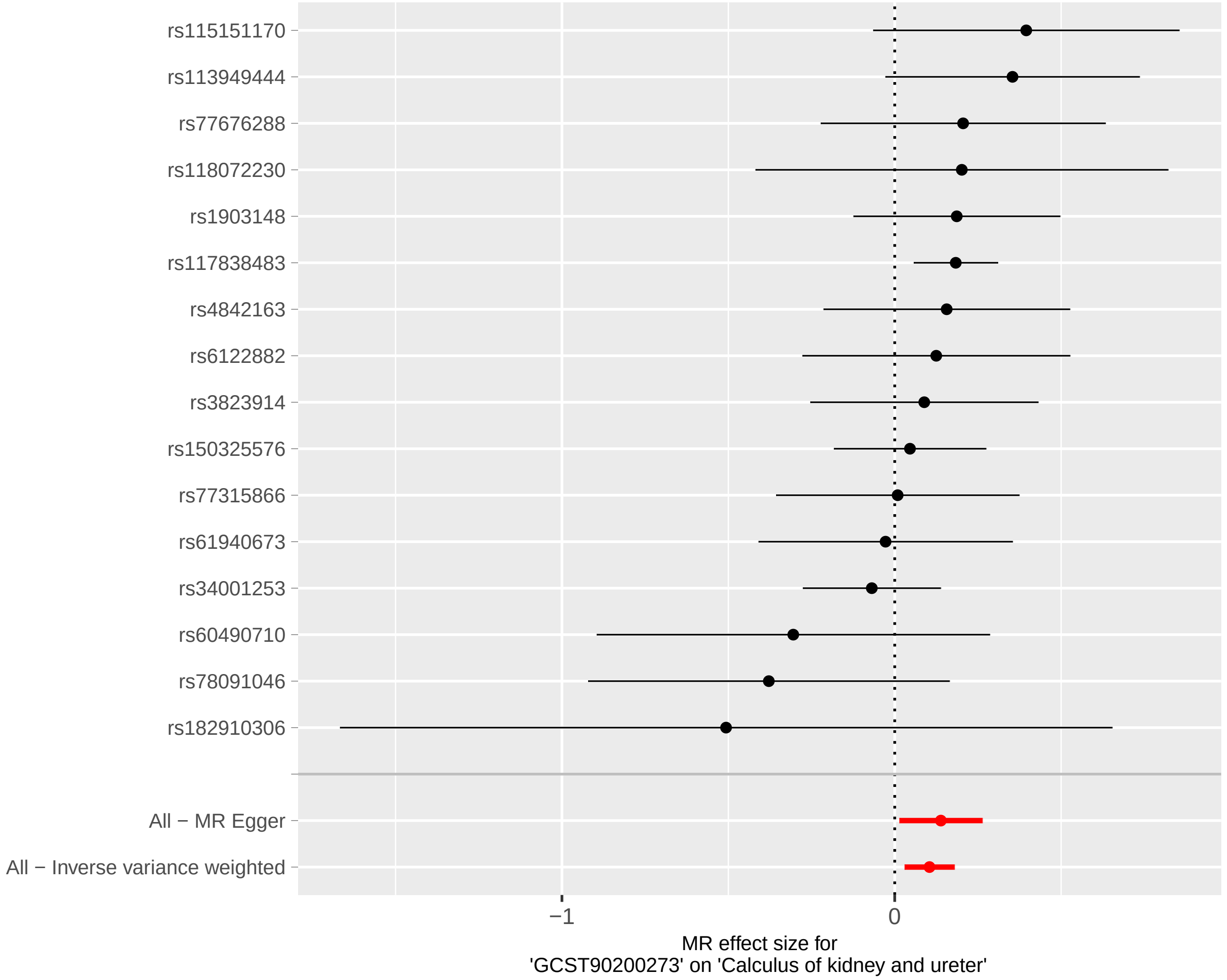
rs115151170
rs113949444
rs77676288
rs118072230
rs1903148
rs117838483
rs4842163
rs6122882
rs3823914
rs150325576
rs77315866
rs61940673
rs34001253
rs60490710
rs78091046
rs182910306
All − MR Egger
All − Inverse variance weighted
−1
0
MR effect size for
'GCST90200273' on 'Calculus of kidney and ureter'

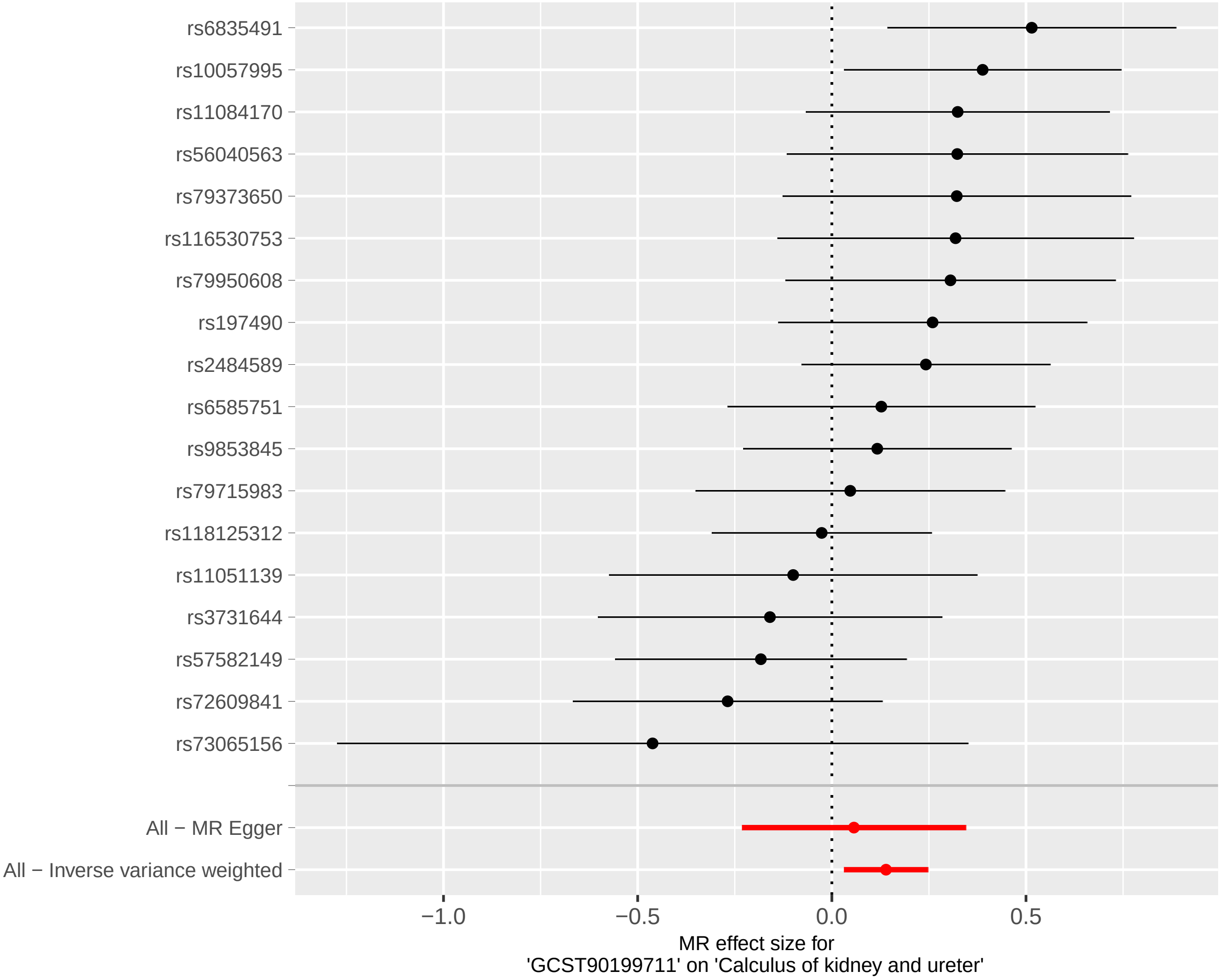
rs6835491
rs10057995
rs11084170
rs56040563
rs79373650
rs116530753
rs79950608
rs197490
rs2484589
rs6585751
rs9853845
rs79715983
rs118125312
rs11051139
rs3731644
rs57582149
rs72609841
rs73065156
All − MR Egger
All − Inverse variance weighted
−1.0
−0.5
0.0
0.5
MR effect size for
'GCST90199711' on 'Calculus of kidney and ureter'

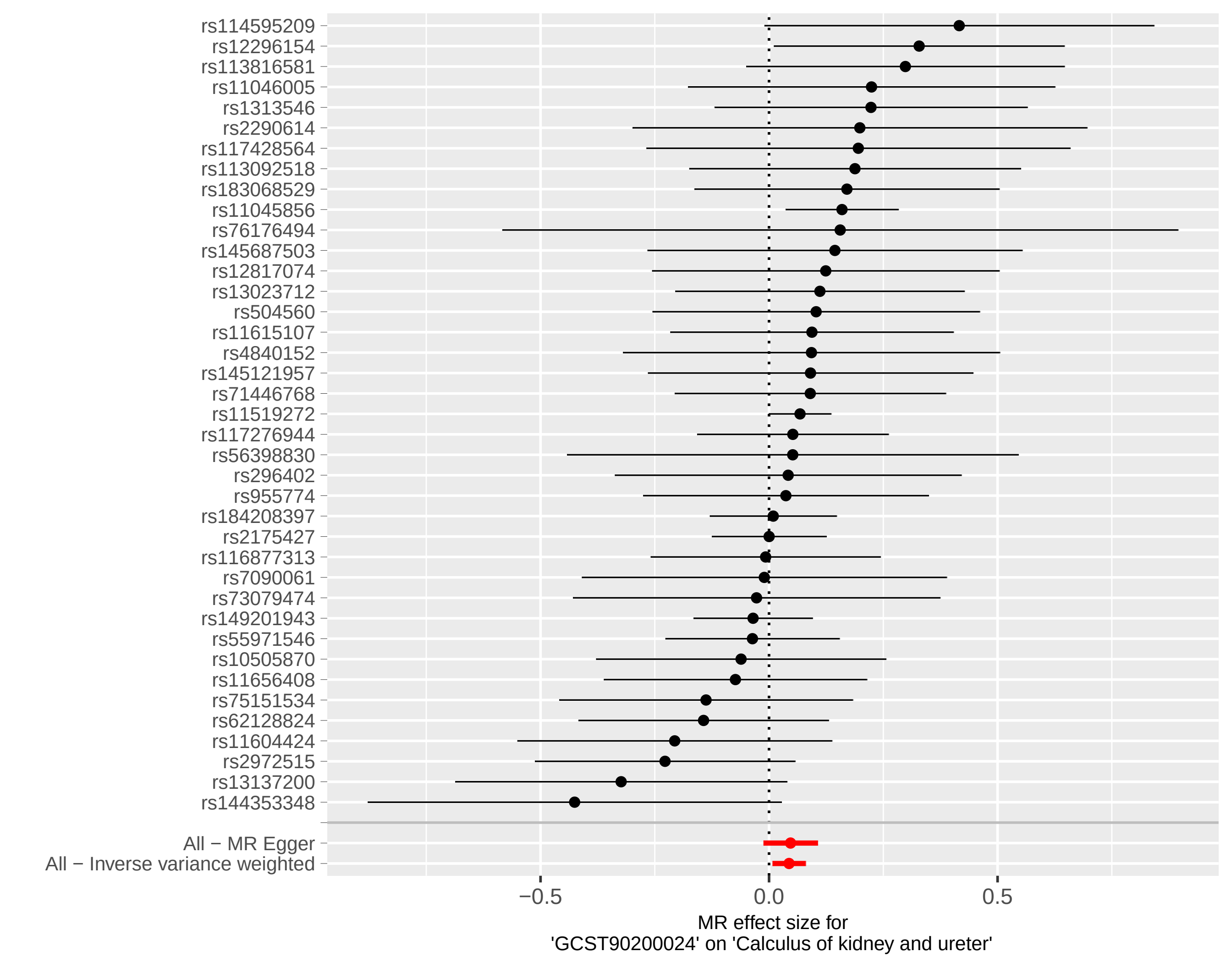
rs114595209
rs12296154
rs113816581
rs11046005
rs1313546
rs2290614
rs117428564
rs113092518
rs183068529
rs11045856
rs76176494
rs145687503
rs12817074
rs13023712
rs504560
rs11615107
rs4840152
rs145121957
rs71446768
rs11519272
rs117276944
rs56398830
rs296402
rs955774
rs184208397
rs2175427
rs116877313
rs7090061
rs73079474
rs149201943
rs55971546
rs10505870
rs11656408
rs75151534
rs62128824
rs11604424
rs2972515
rs13137200
rs144353348
All − MR Egger
All − Inverse variance weighted
−0.5
0.0
0.5
MR effect size for
'GCST90200024' on 'Calculus of kidney and ureter'

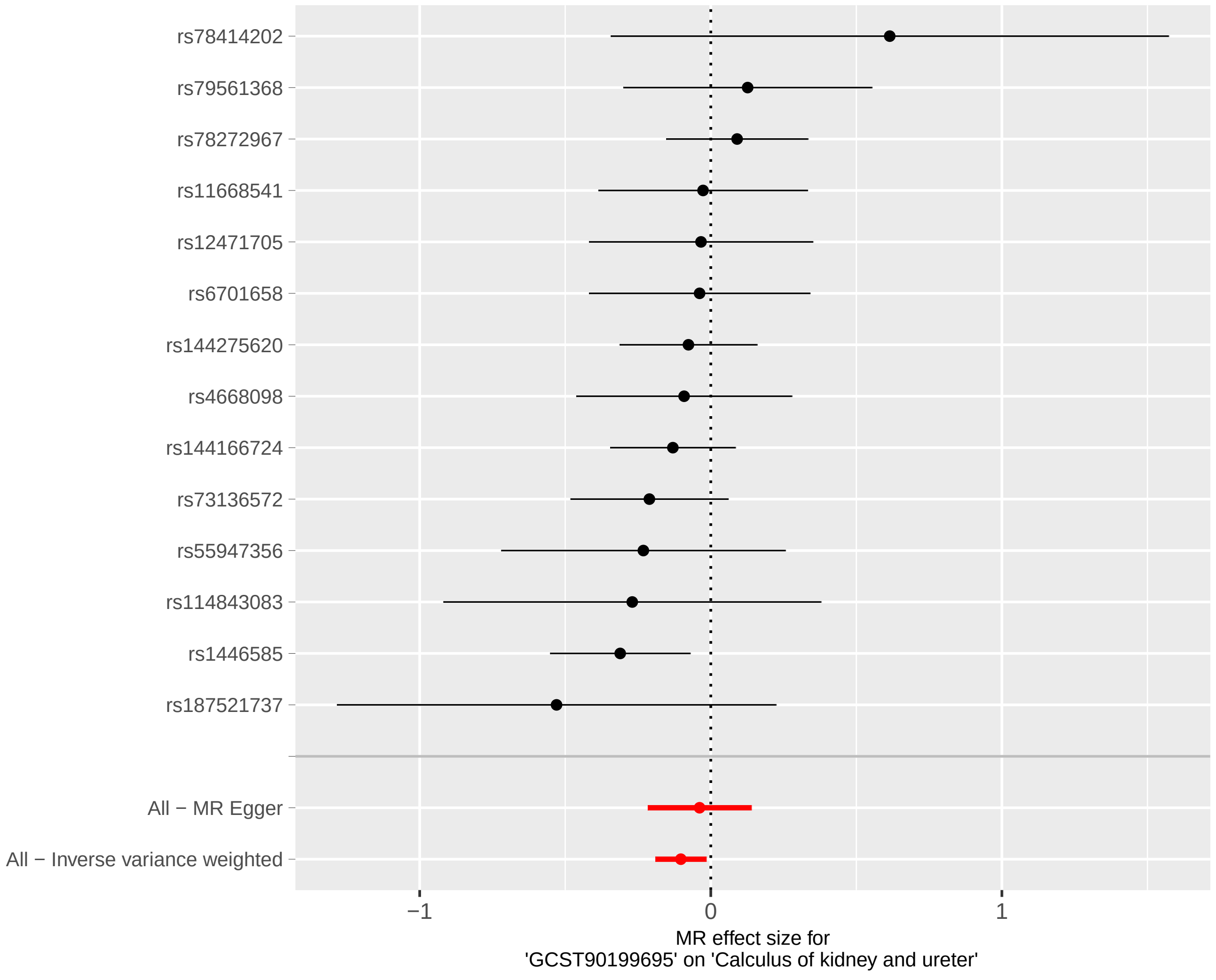
rs78414202
rs79561368
rs78272967
rs11668541
rs12471705
rs6701658
rs144275620
rs4668098
rs144166724
rs73136572
rs55947356
rs114843083
rs1446585
rs187521737
All − MR Egger
All − Inverse variance weighted
−1
0
1
MR effect size for
'GCST90199695' on 'Calculus of kidney and ureter'

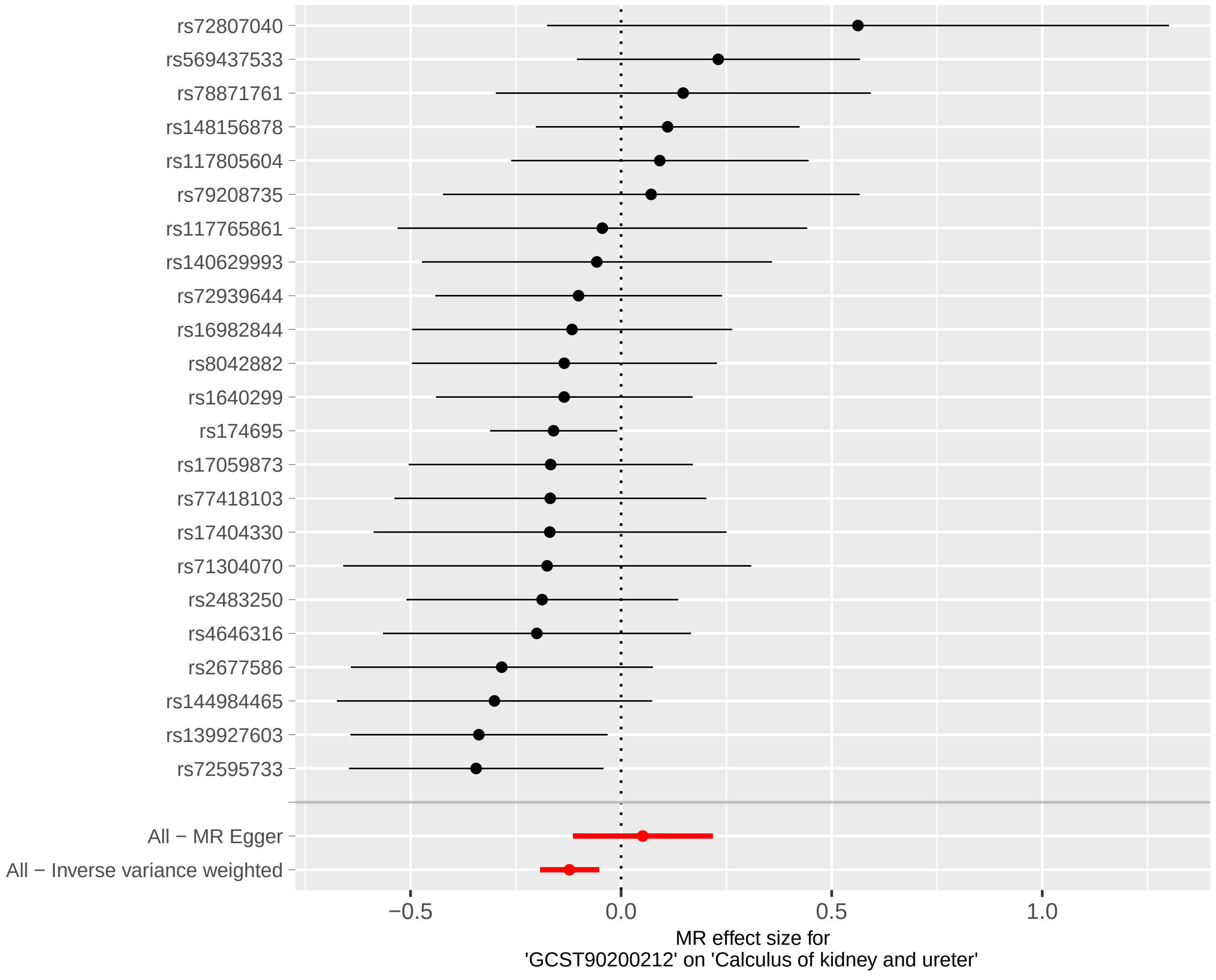
rs72807040
rs569437533
rs78871761
rs148156878
rs117805604
rs79208735
rs117765861
rs140629993
rs72939644
rs16982844
rs8042882
rs1640299
rs174695
rs17059873
rs77418103
rs17404330
rs71304070
rs2483250
rs4646316
rs2677586
rs144984465
rs139927603
rs72595733
All − MR Egger
All − Inverse variance weighted
−0.5
0.0
0.5
1.0
MR effect size for
'GCST90200212' on 'Calculus of kidney and ureter'

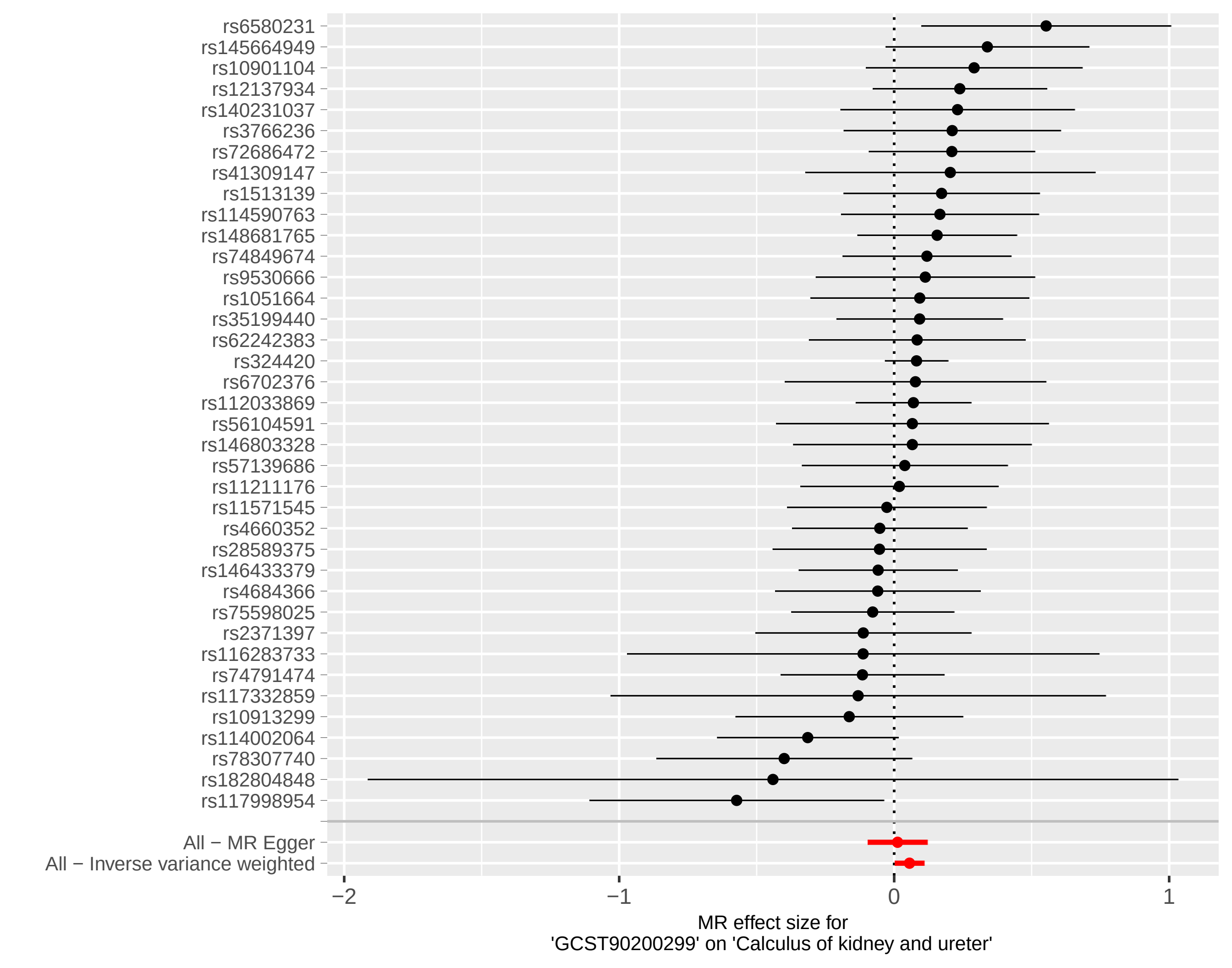
rs6580231
rs145664949
rs10901104
rs12137934
rs140231037
rs3766236
rs72686472
rs41309147
rs1513139
rs114590763
rs148681765
rs74849674
rs9530666
rs1051664
rs35199440
rs62242383
rs324420
rs6702376
rs112033869
rs56104591
rs146803328
rs57139686
rs11211176
rs11571545
rs4660352
rs28589375
rs146433379
rs4684366
rs75598025
rs2371397
rs116283733
rs74791474
rs117332859
rs10913299
rs114002064
rs78307740
rs182804848
rs117998954
All − MR Egger
All − Inverse variance weighted
−2
−1
0
1
MR effect size for
'GCST90200299' on 'Calculus of kidney and ureter'

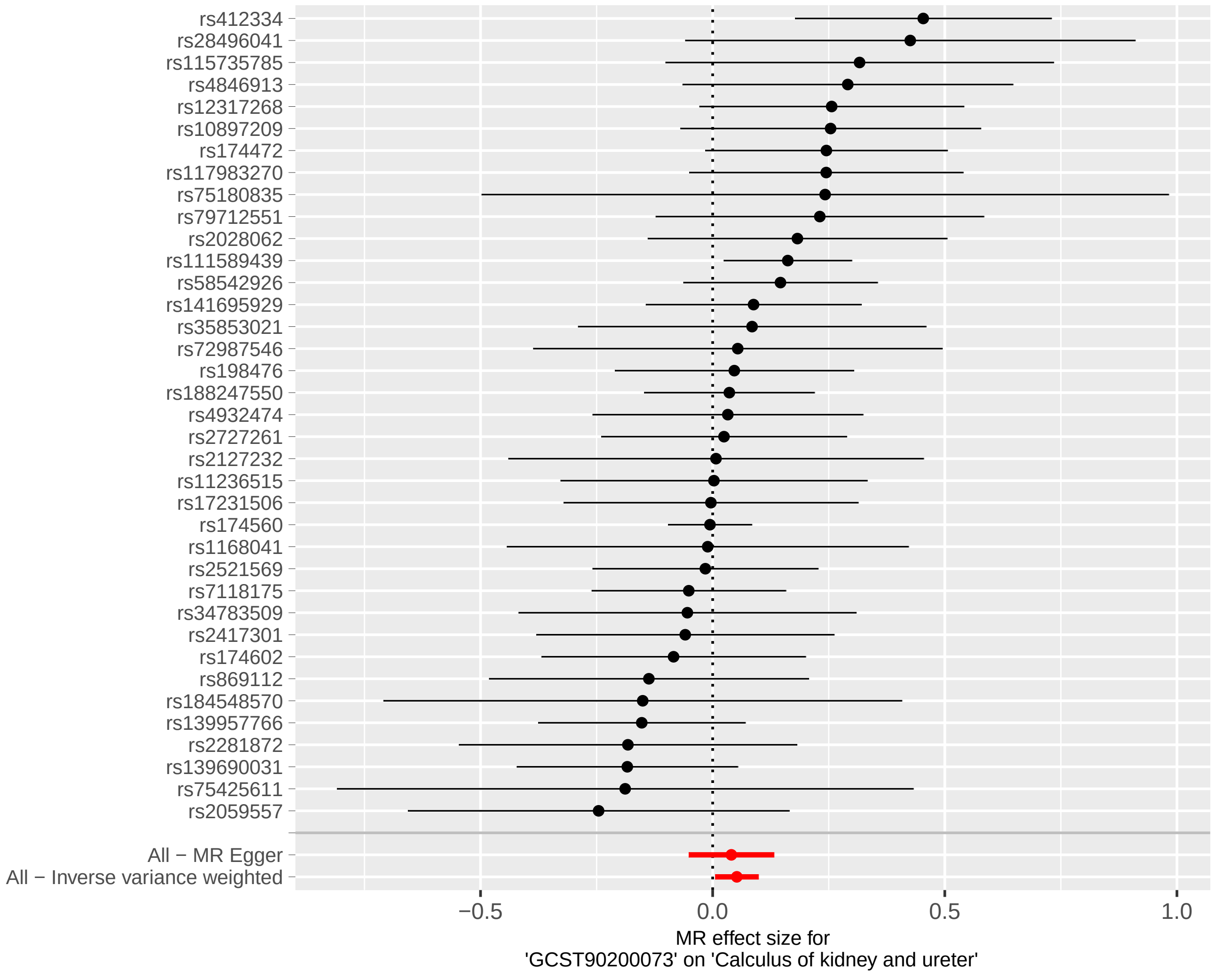

rs412334
rs28496041
rs115735785
rs4846913
rs12317268
rs10897209
rs174472
rs117983270
rs75180835
rs79712551
rs2028062
rs111589439
rs58542926
rs141695929
rs35853021
rs72987546
rs198476
rs188247550
rs4932474
rs2727261
rs2127232
rs11236515
rs17231506
rs174560
rs1168041
rs2521569
rs7118175
rs34783509
rs2417301
rs174602
rs869112
rs184548570
rs139957766
rs2281872
rs139690031
rs75425611
rs2059557
All − MR Egger
All − Inverse variance weighted
−0.5
0.0
0.5
1.0
MR effect size for
'GCST90200073' on 'Calculus of kidney and ureter'

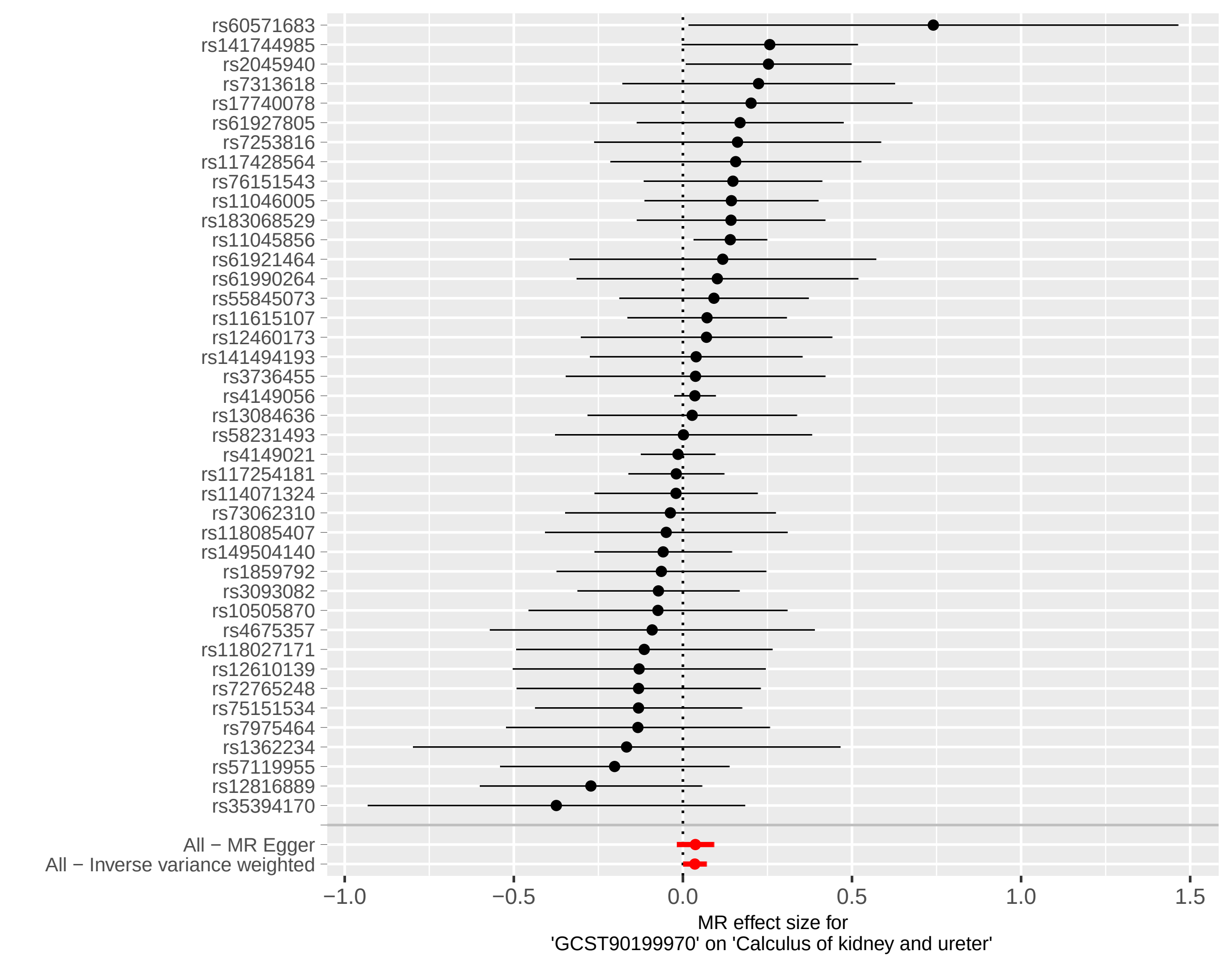
rs60571683
rs141744985
rs2045940
rs7313618
rs17740078
rs61927805
rs7253816
rs117428564
rs76151543
rs11046005
rs183068529
rs11045856
rs61921464
rs61990264
rs55845073
rs11615107
rs12460173
rs141494193
rs3736455
rs4149056
rs13084636
rs58231493
rs4149021
rs117254181
rs114071324
rs73062310
rs118085407
rs149504140
rs1859792
rs3093082
rs10505870
rs4675357
rs118027171
rs12610139
rs72765248
rs75151534
rs7975464
rs1362234
rs57119955
rs12816889
rs35394170
All − MR Egger
All − Inverse variance weighted
−1.0
−0.5
0.0
0.5
1.0
1.5
MR effect size for
'GCST90199970' on 'Calculus of kidney and ureter'

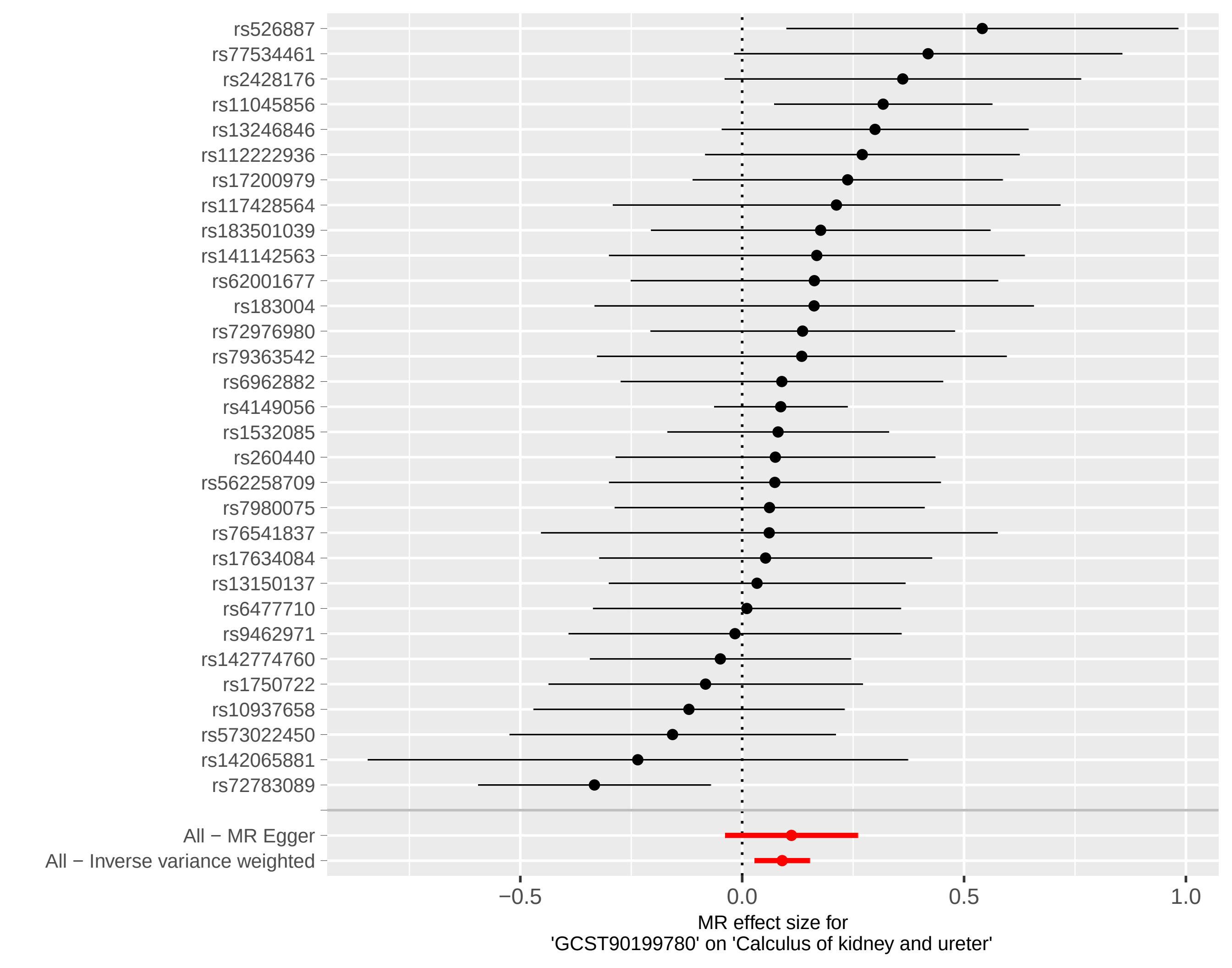
rs526887
rs77534461
rs2428176
rs11045856
rs13246846
rs112222936
rs17200979
rs117428564
rs183501039
rs141142563
rs62001677
rs183004
rs72976980
rs79363542
rs6962882
rs4149056
rs1532085
rs260440
rs562258709
rs7980075
rs76541837
rs17634084
rs13150137
rs6477710
rs9462971
rs142774760
rs1750722
rs10937658
rs573022450
rs142065881
rs72783089
All − MR Egger
All − Inverse variance weighted
−0.5
0.0
0.5
1.0
MR effect size for
'GCST90199780' on 'Calculus of kidney and ureter'

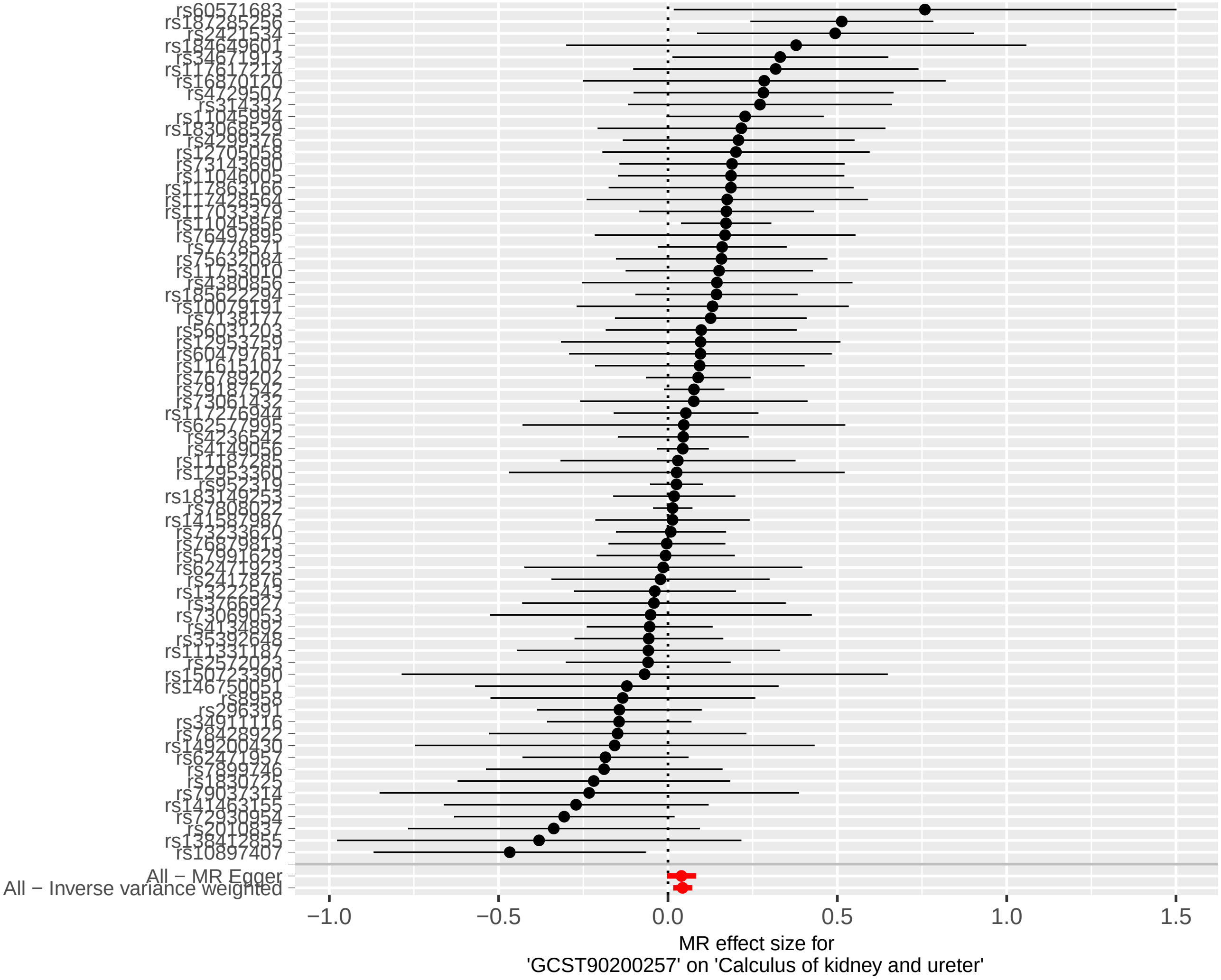

rs60571683
rs187285256
rs2421534
rs184649601
rs34671913
rs117617214
rs16870120
rs4729507
rs314332
rs11045994
rs183068529
rs4299376
rs12705058
rs73143690
rs11046005
rs117863166
rs117428564
rs117033379
rs11045856
rs76497895
rs7778571
rs75632084
rs11753010
rs4380856
rs185622294
rs10079191
rs7138177
rs56031203
rs12953759
rs60479761
rs11615107
rs76789202
rs79187542
rs73061432
rs117276944
rs62577995
rs4236542
rs4149056
rs1187285
rs12953360
rs952319
rs183149253
rs7808022
rs141587987
rs73233620
rs76879813
rs57991629
rs62471923
rs2417876
rs13222543
rs3766927
rs73069053
rs4134892
rs35392648
rs11133187
rs2572023
rs150723390
rs146750051
rs8958
rs296391
rs34911116
rs78428922
rs149200430
rs62471957
rs7899746
rs1830725
rs79037314
rs141463155
rs72930954
rs2010837
rs138412855
rs10897407
All − MR Egger
All − Inverse variance weighted
−1.0
−0.5
0.0
0.5
1.0
1.5
MR effect size for
'GCST90200257' on 'Calculus of kidney and ureter'

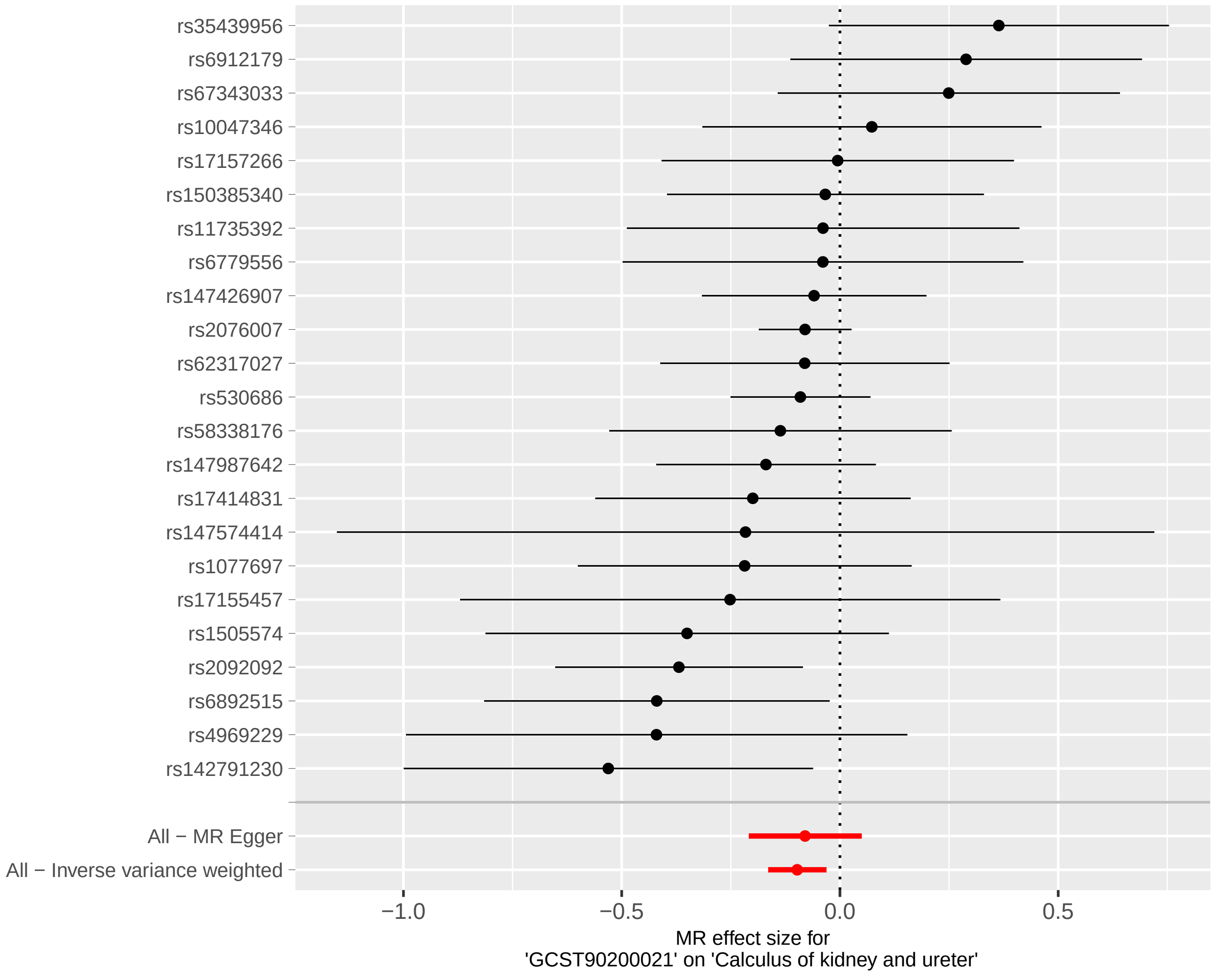

rs35439956
rs6912179
rs67343033
rs10047346
rs17157266
rs150385340
rs11735392
rs6779556
rs147426907
rs2076007
rs62317027
rs530686
rs58338176
rs147987642
rs17414831
rs147574414
rs1077697
rs17155457
rs1505574
rs2092092
rs6892515
rs4969229
rs142791230
All − MR Egger
All − Inverse variance weighted
−1.0
−0.5
0.0
0.5
MR effect size for
'GCST90200021' on 'Calculus of kidney and ureter'

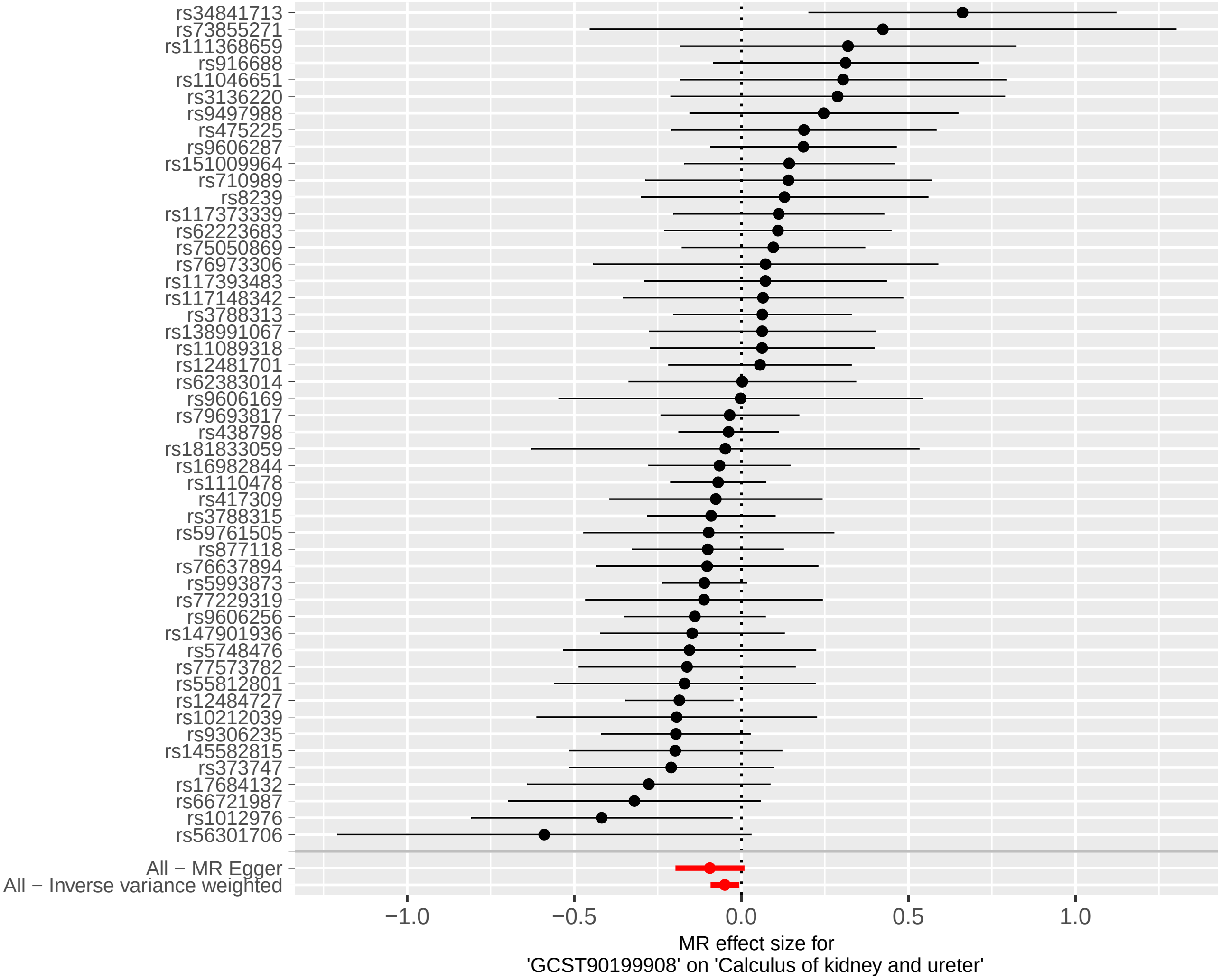
rs34841713
rs73855271
rs111368659
rs916688
rs11046651
rs3136220
rs9497988
rs475225
rs9606287
rs151009964
rs710989
rs8239
rs117373339
rs62223683
rs75050869
rs76973306
rs117393483
rs117148342
rs3788313
rs138991067
rs11089318
rs12481701
rs62383014
rs9606169
rs79693817
rs438798
rs181833059
rs16982844
rs1110478
rs417309
rs3788315
rs59761505
rs877118
rs76637894
rs5993873
rs77229319
rs9606256
rs147901936
rs5748476
rs77573782
rs55812801
rs12484727
rs10212039
rs9306235
rs145582815
rs373747
rs17684132
rs66721987
rs1012976
rs56301706
All − MR Egger
All − Inverse variance weighted
−1.0
−0.5
0.0
0.5
1.0
MR effect size for
'GCST90199908' on 'Calculus of kidney and ureter'

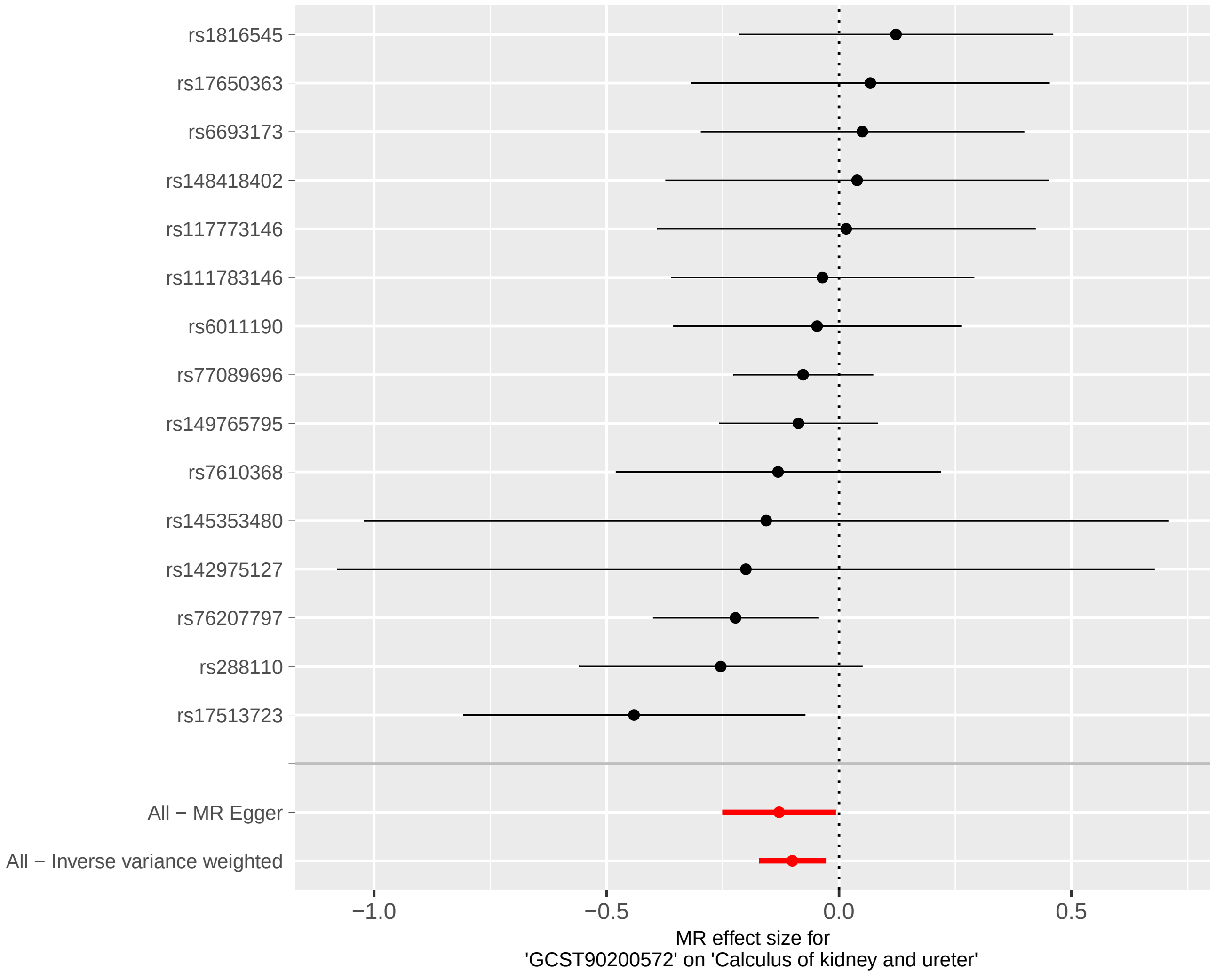
rs1816545
rs17650363
rs6693173
rs148418402
rs117773146
rs111783146
rs6011190
rs77089696
rs149765795
rs7610368
rs145353480
rs142975127
rs76207797
rs288110
rs17513723
All − MR Egger
All − Inverse variance weighted
−1.0
−0.5
0.0
0.5
MR effect size for
'GCST90200572' on 'Calculus of kidney and ureter'

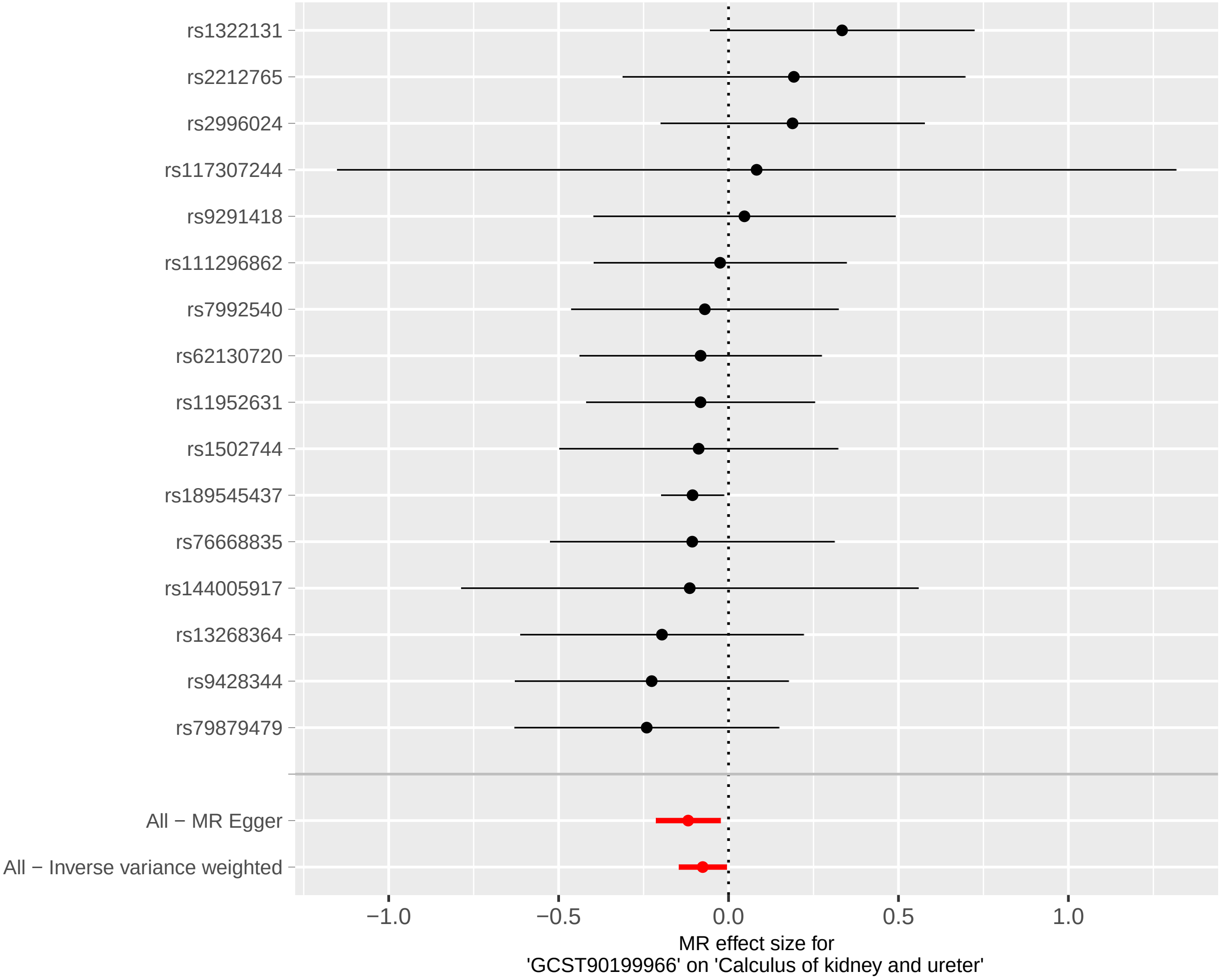
rs1322131
rs2212765
rs2996024
rs117307244
rs9291418
rs111296862
rs7992540
rs62130720
rs11952631
rs1502744
rs189545437
rs76668835
rs144005917
rs13268364
rs9428344
rs79879479
All − MR Egger
All − Inverse variance weighted
−1.0
−0.5
0.0
0.5
1.0
MR effect size for
'GCST90199966' on 'Calculus of kidney and ureter'

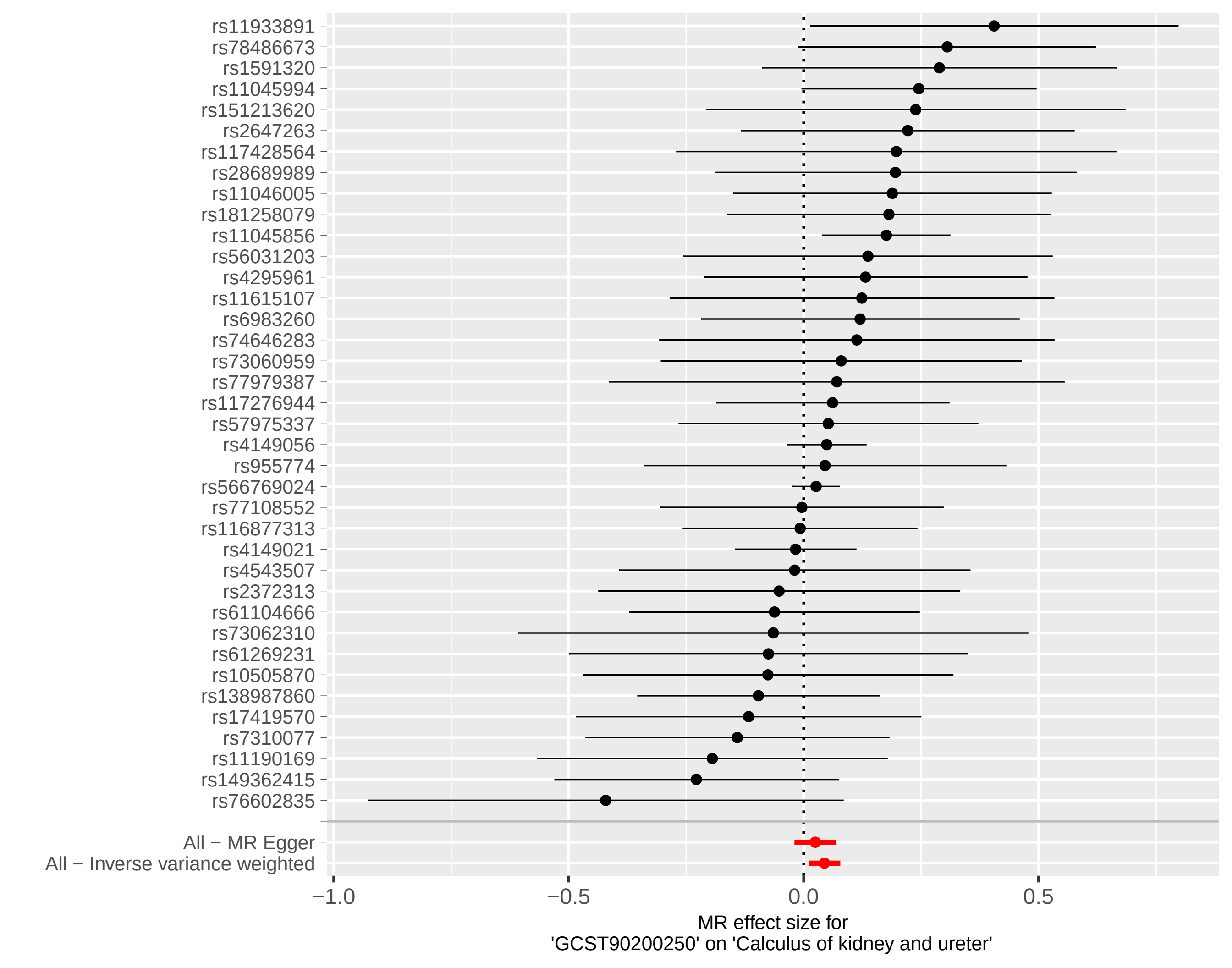
rs11933891
rs78486673
rs1591320
rs11045994
rs151213620
rs2647263
rs117428564
rs28689989
rs11046005
rs181258079
rs11045856
rs56031203
rs4295961
rs11615107
rs6983260
rs74646283
rs73060959
rs77979387
rs117276944
rs57975337
rs4149056
rs955774
rs566769024
rs77108552
rs116877313
rs4149021
rs4543507
rs2372313
rs61104666
rs73062310
rs61269231
rs10505870
rs138987860
rs17419570
rs7310077
rs11190169
rs149362415
rs76602835
All − MR Egger
All − Inverse variance weighted
−1.0
−0.5
0.0
0.5
MR effect size for
'GCST90200250' on 'Calculus of kidney and ureter'

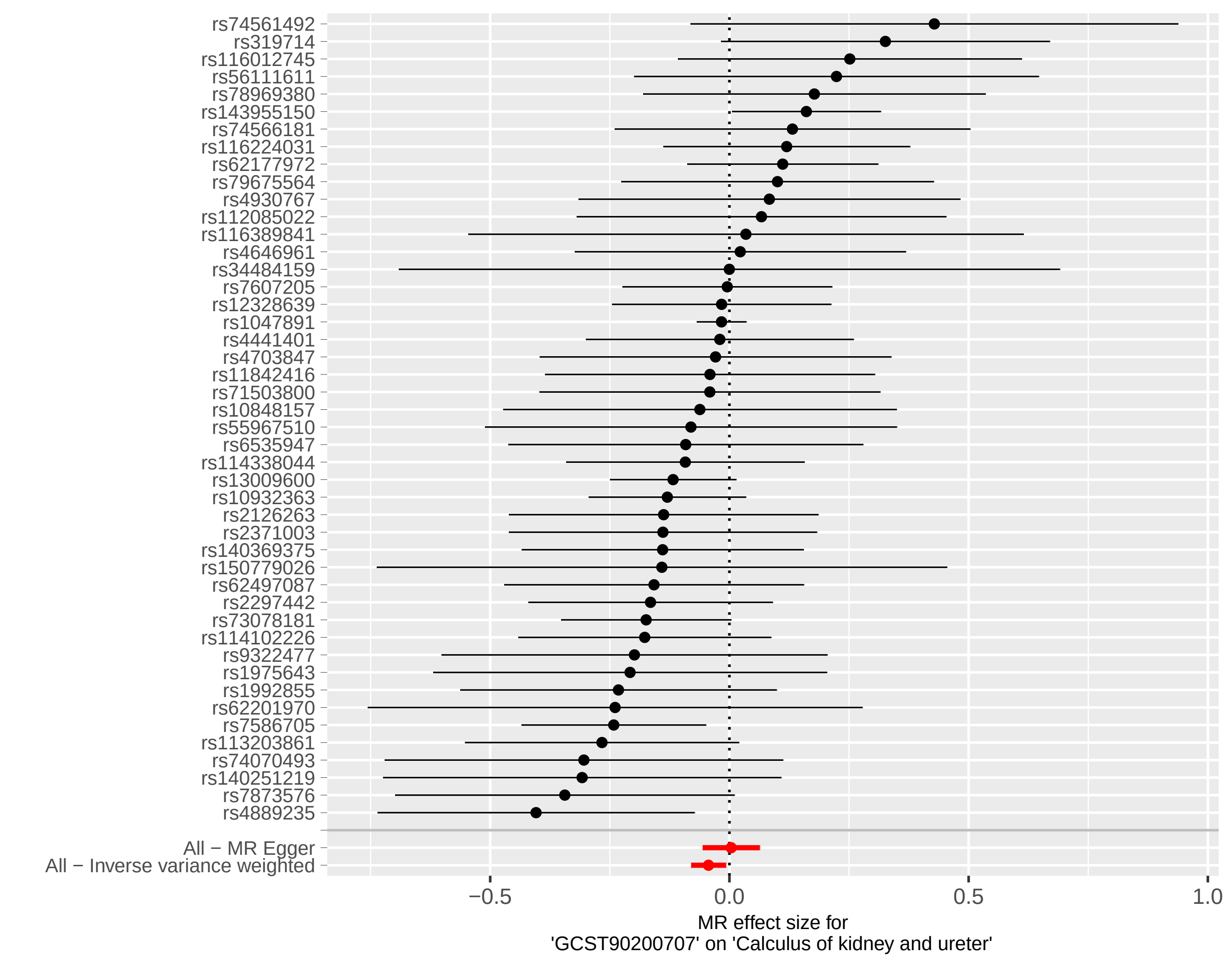
rs74561492
rs319714
rs116012745
rs56111611
rs78969380
rs143955150
rs74566181
rs116224031
rs62177972
rs79675564
rs4930767
rs112085022
rs116389841
rs4646961
rs34484159
rs7607205
rs12328639
rs1047891
rs4441401
rs4703847
rs11842416
rs71503800
rs10848157
rs55967510
rs6535947
rs114338044
rs13009600
rs10932363
rs2126263
rs2371003
rs140369375
rs150779026
rs62497087
rs2297442
rs73078181
rs114102226
rs9322477
rs1975643
rs1992855
rs62201970
rs7586705
rs113203861
rs74070493
rs140251219
rs7873576
rs4889235
All − MR Egger
All − Inverse variance weighted
−0.5
0.0
0.5
1.0
MR effect size for
'GCST90200707' on 'Calculus of kidney and ureter'

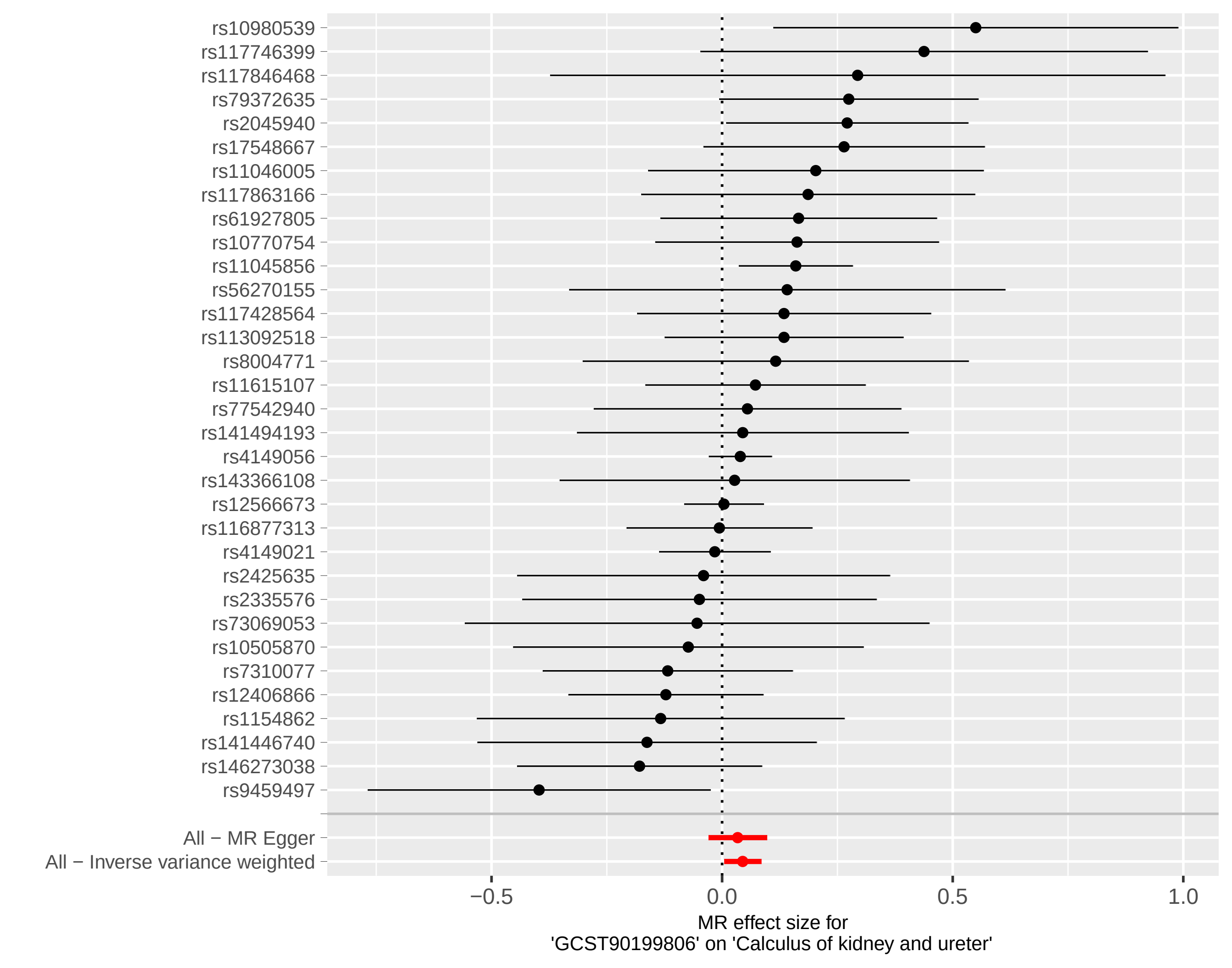
rs10980539
rs117746399
rs117846468
rs79372635
rs2045940
rs17548667
rs11046005
rs117863166
rs61927805
rs10770754
rs11045856
rs56270155
rs117428564
rs113092518
rs8004771
rs11615107
rs77542940
rs141494193
rs4149056
rs143366108
rs12566673
rs116877313
rs4149021
rs2425635
rs2335576
rs73069053
rs10505870
rs7310077
rs12406866
rs1154862
rs141446740
rs146273038
rs9459497
All − MR Egger
All − Inverse variance weighted
−0.5
0.0
0.5
1.0
MR effect size for
'GCST90199806' on 'Calculus of kidney and ureter'

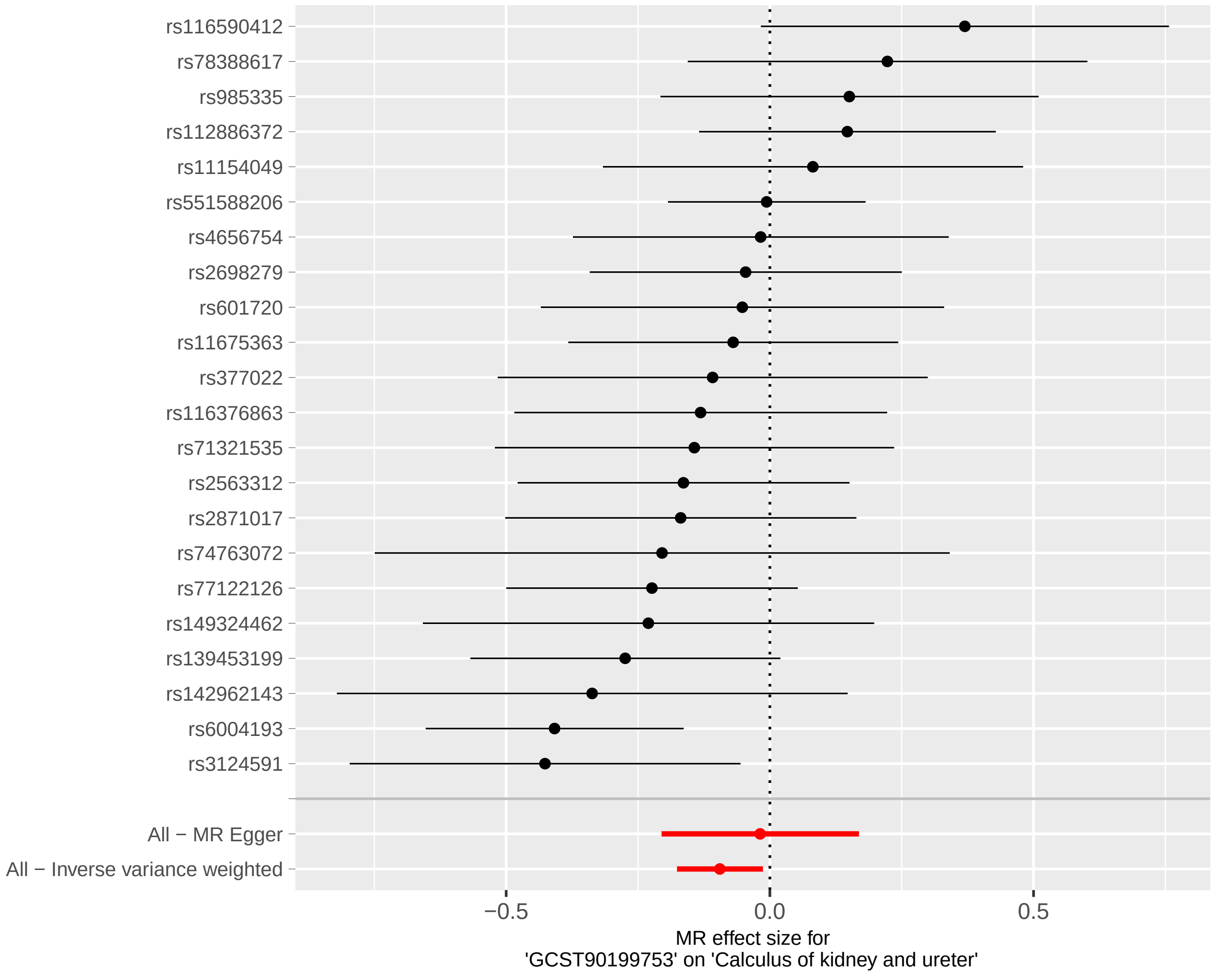
rs116590412
rs78388617
rs985335
rs112886372
rs11154049
rs551588206
rs4656754
rs2698279
rs601720
rs11675363
rs377022
rs116376863
rs71321535
rs2563312
rs2871017
rs74763072
rs77122126
rs149324462
rs139453199
rs142962143
rs6004193
rs3124591
All − MR Egger
All − Inverse variance weighted
−0.5
0.0
0.5
MR effect size for
'GCST90199753' on 'Calculus of kidney and ureter'

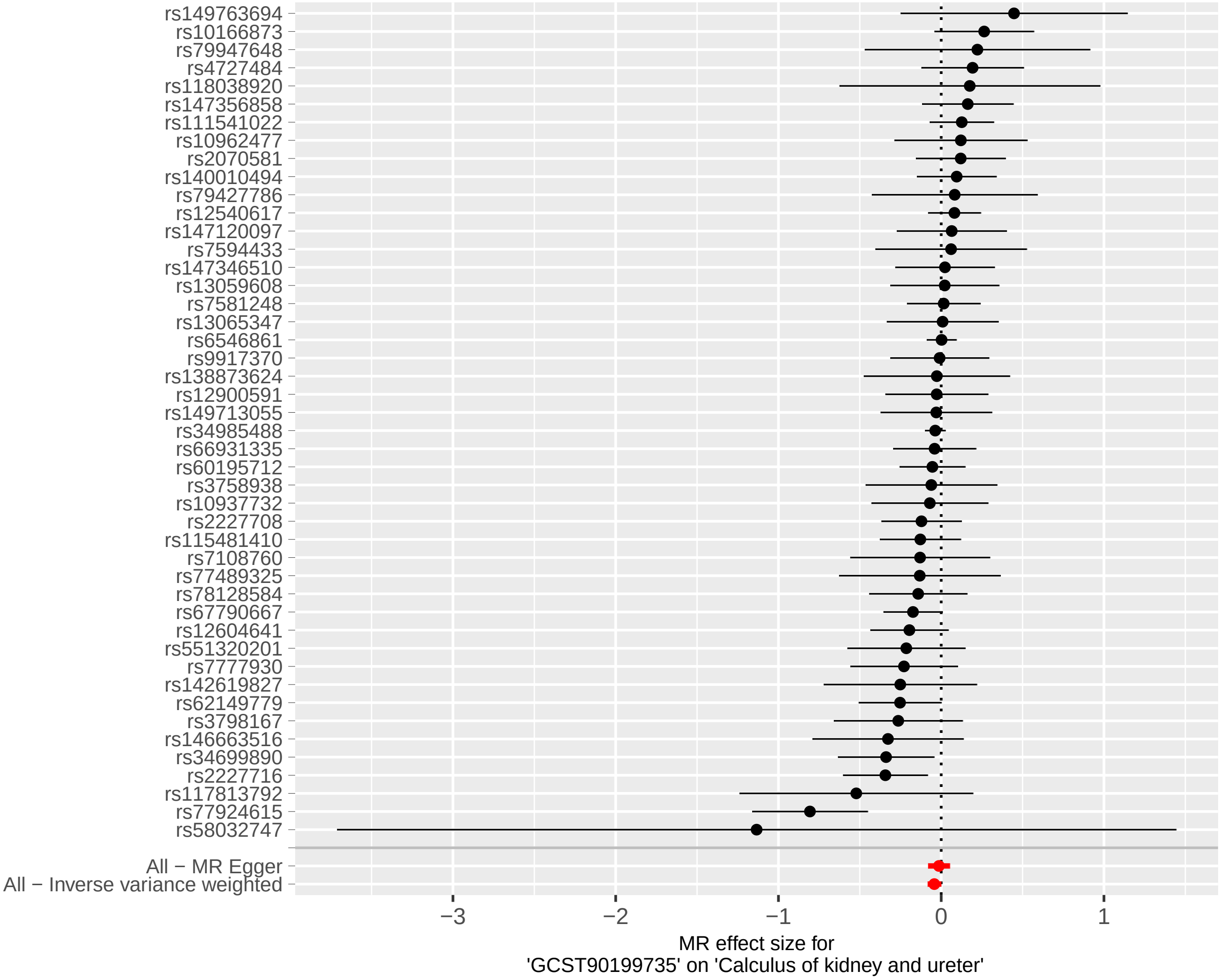

rs149763694
rs10166873
rs79947648
rs4727484
rs118038920
rs147356858
rs111541022
rs10962477
rs2070581
rs140010494
rs79427786
rs12540617
rs147120097
rs7594433
rs147346510
rs13059608
rs7581248
rs13065347
rs6546861
rs9917370
rs138873624
rs12900591
rs149713055
rs34985488
rs66931335
rs60195712
rs3758938
rs10937732
rs2227708
rs115481410
rs7108760
rs77489325
rs78128584
rs67790667
rs12604641
rs551320201
rs7777930
rs142619827
rs62149779
rs3798167
rs146663516
rs34699890
rs2227716
rs117813792
rs77924615
rs58032747
All − MR Egger
All − Inverse variance weighted
−3
−2
−1
0
1
MR effect size for
'GCST90199735' on 'Calculus of kidney and ureter'

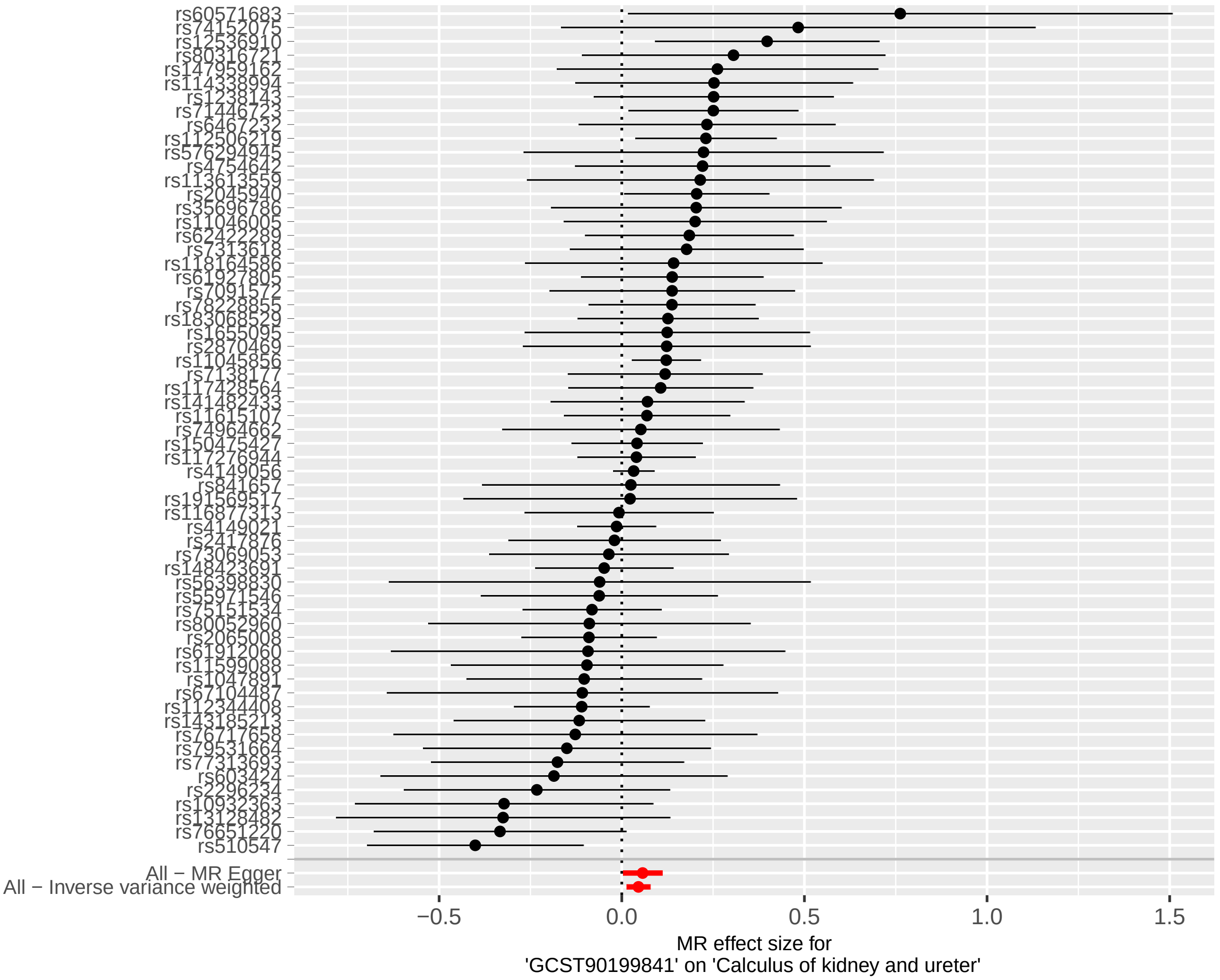
rs60571683
rs74152075
rs12536910
rs80316721
rs147959162
rs114338994
rs1238143
rs71446723
rs6467232
rs112506219
rs576294945
rs4754642
rs113613559
rs2045940
rs35696786
rs11046005
rs62422289
rs7313618
rs118164586
rs61927805
rs7091572
rs78228855
rs183068529
rs1655095
rs2870469
rs11045856
rs7138177
rs117428564
rs141482433
rs11615107
rs74964662
rs150475427
rs117276944
rs4149056
rs841657
rs191569517
rs116877313
rs4149021
rs2417876
rs73069053
rs148423691
rs56398830
rs55971546
rs75151534
rs80052960
rs2065008
rs61912060
rs11599088
rs1047891
rs67104487
rs112344408
rs143185213
rs76717658
rs79531664
rs77313693
rs603424
rs2296234
rs10932363
rs13128482
rs76651220
rs510547
All − MR Egger
All − Inverse variance weighted
−0.5
0.0
0.5
1.0
1.5
MR effect size for
'GCST90199841' on 'Calculus of kidney and ureter'

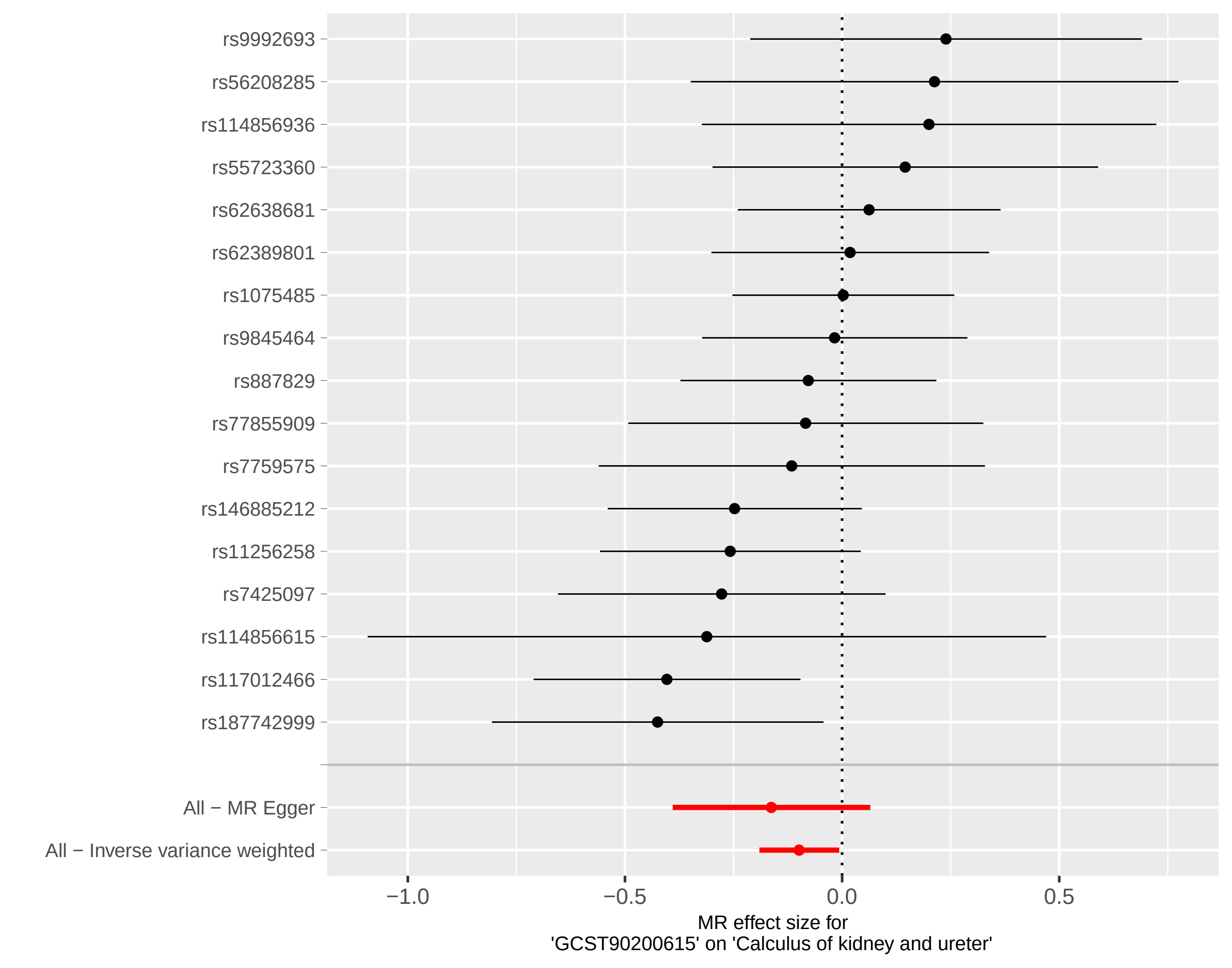
rs9992693
rs56208285
rs114856936
rs55723360
rs62638681
rs62389801
rs1075485
rs9845464
rs887829
rs77855909
rs7759575
rs146885212
rs11256258
rs7425097
rs114856615
rs117012466
rs187742999
All − MR Egger
All − Inverse variance weighted
−1.0
−0.5
0.0
0.5
MR effect size for
'GCST90200615' on 'Calculus of kidney and ureter'

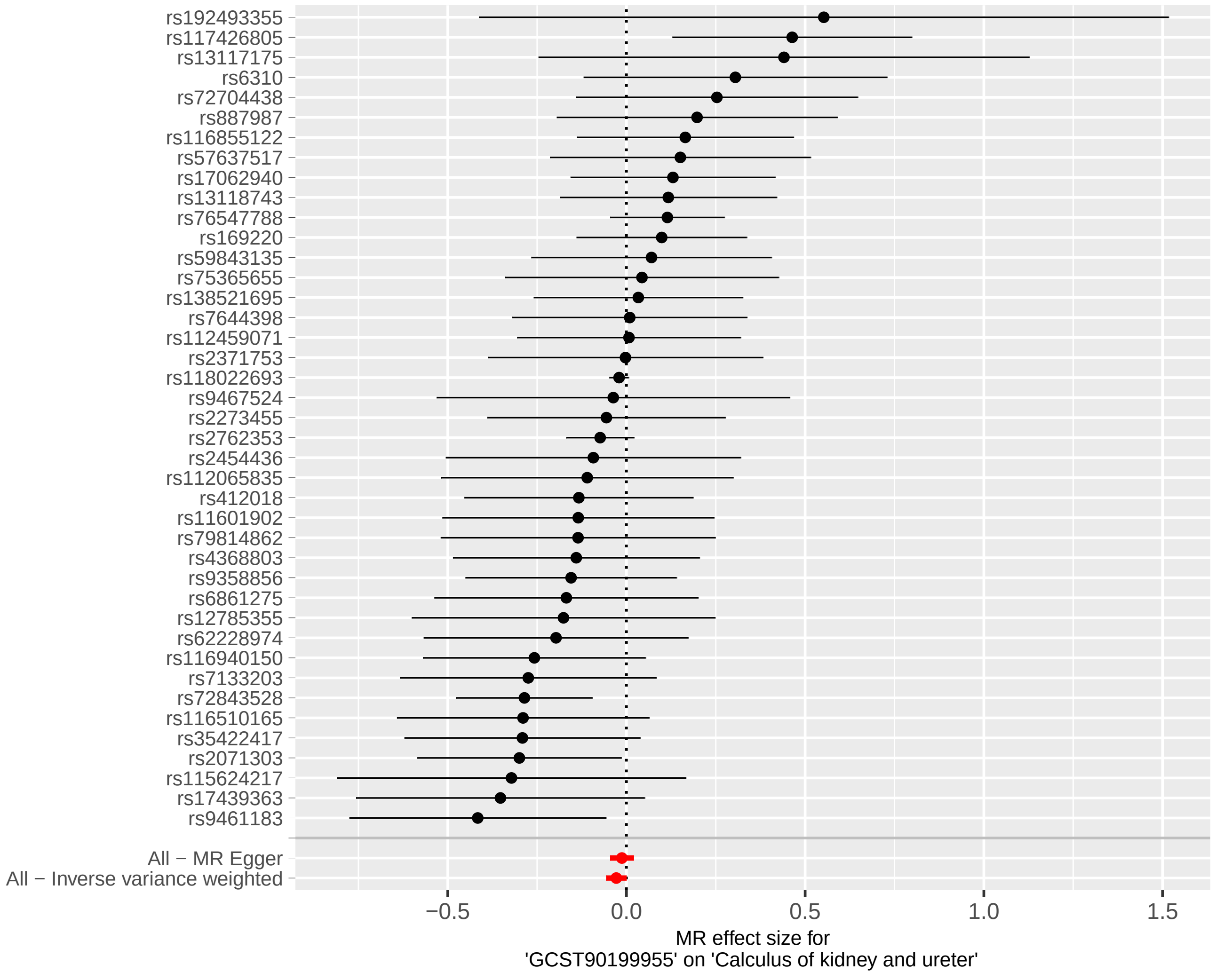
rs192493355
rs117426805
rs13117175
rs6310
rs72704438
rs887987
rs116855122
rs57637517
rs17062940
rs13118743
rs76547788
rs169220
rs59843135
rs75365655
rs138521695
rs7644398
rs112459071
rs2371753
rs118022693
rs9467524
rs2273455
rs2762353
rs2454436
rs112065835
rs412018
rs11601902
rs79814862
rs4368803
rs9358856
rs6861275
rs12785355
rs62228974
rs116940150
rs7133203
rs72843528
rs116510165
rs35422417
rs2071303
rs115624217
rs17439363
rs9461183
All − MR Egger
All − Inverse variance weighted
−0.5
0.0
0.5
1.0
1.5
MR effect size for
'GCST90199955' on 'Calculus of kidney and ureter'

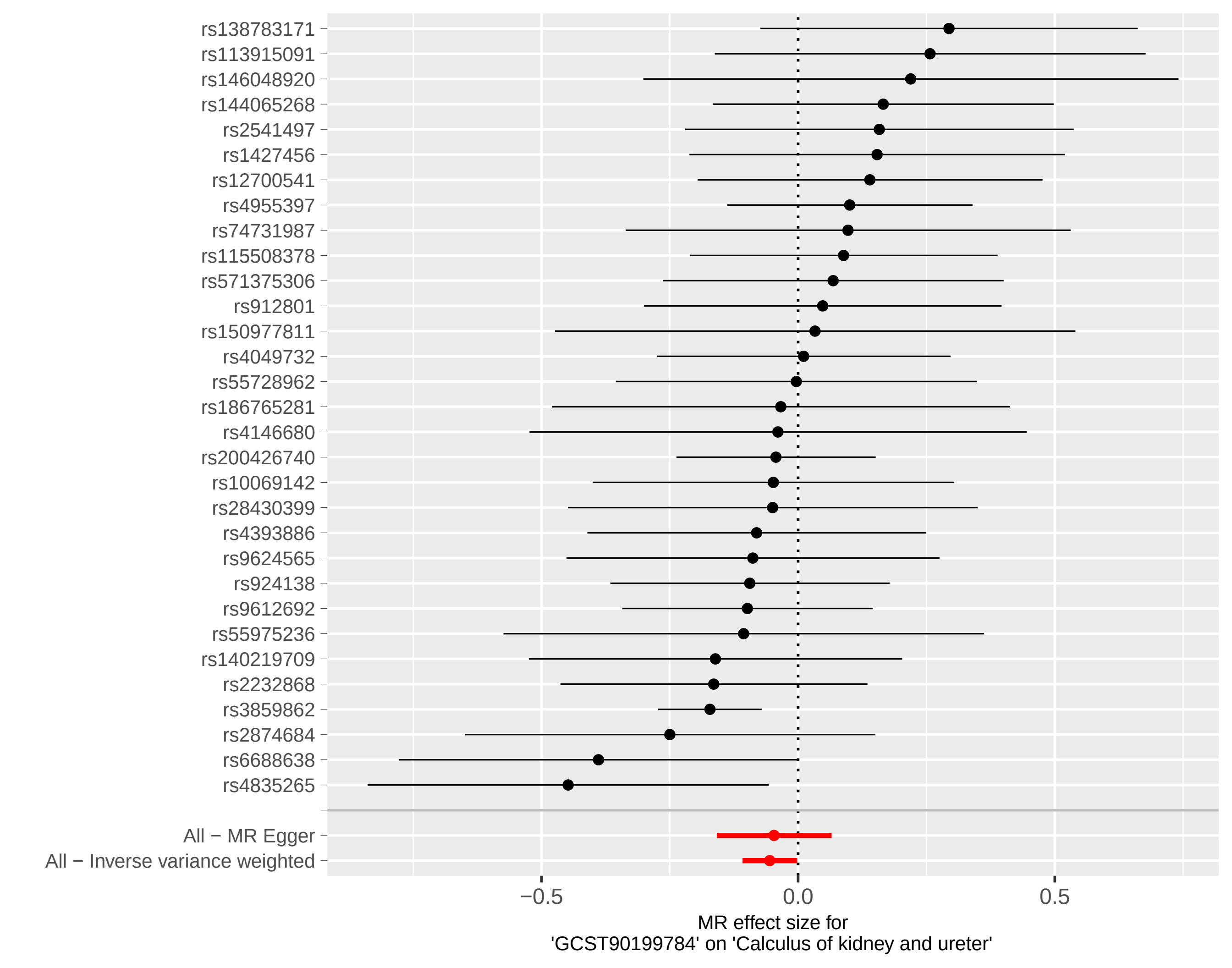
rs138783171
rs113915091
rs146048920
rs144065268
rs2541497
rs1427456
rs12700541
rs4955397
rs74731987
rs115508378
rs571375306
rs912801
rs150977811
rs4049732
rs55728962
rs186765281
rs4146680
rs200426740
rs10069142
rs28430399
rs4393886
rs9624565
rs924138
rs9612692
rs55975236
rs140219709
rs2232868
rs3859862
rs2874684
rs6688638
rs4835265
All − MR Egger
All − Inverse variance weighted
−0.5
0.0
0.5
MR effect size for
'GCST90199784' on 'Calculus of kidney and ureter'

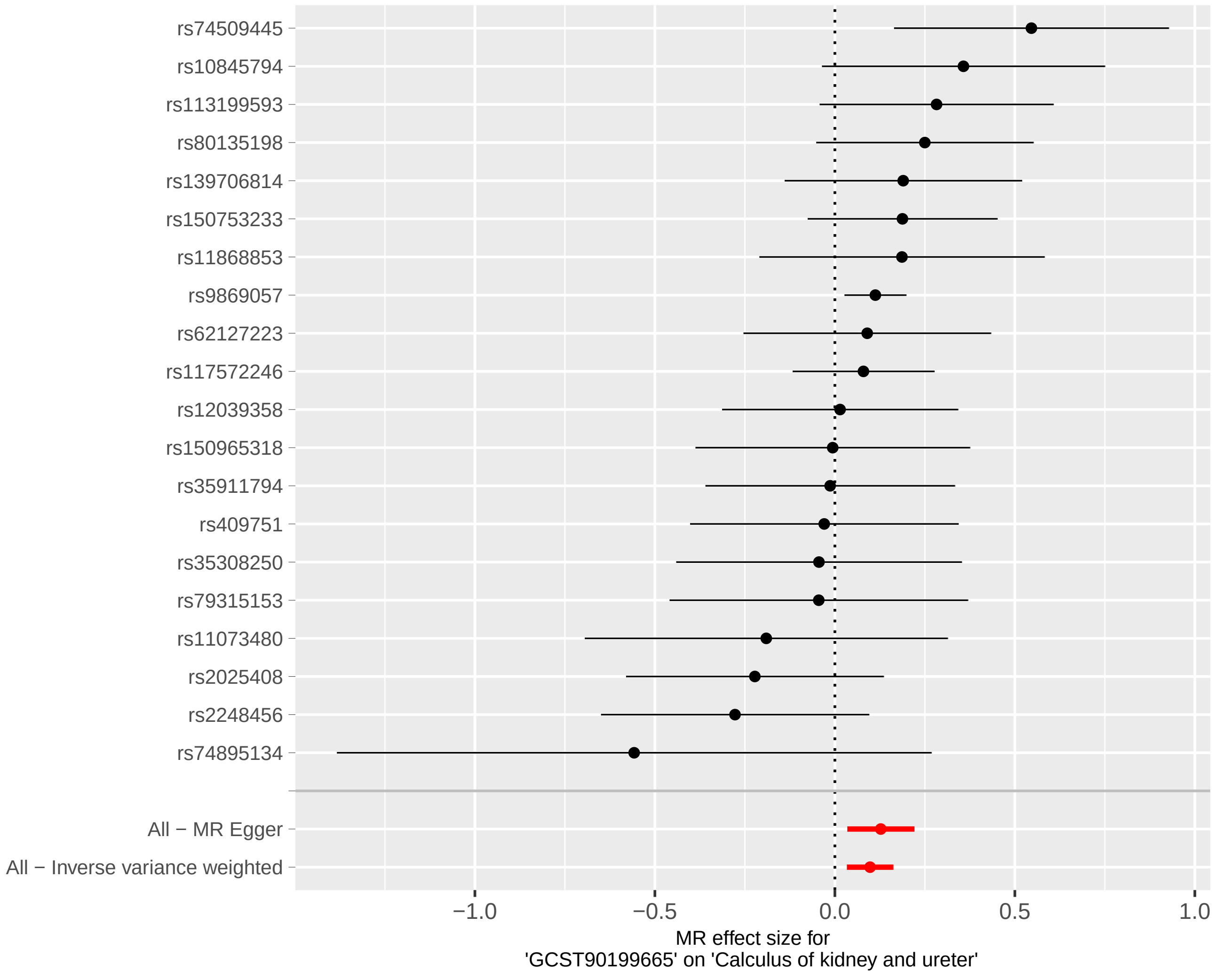
rs74509445
rs10845794
rs113199593
rs80135198
rs139706814
rs150753233
rs11868853
rs9869057
rs62127223
rs117572246
rs12039358
rs150965318
rs35911794
rs409751
rs35308250
rs79315153
rs11073480
rs2025408
rs2248456
rs74895134
All − MR Egger
All − Inverse variance weighted
−1.0
−0.5
0.0
0.5
1.0
MR effect size for
'GCST90199665' on 'Calculus of kidney and ureter'

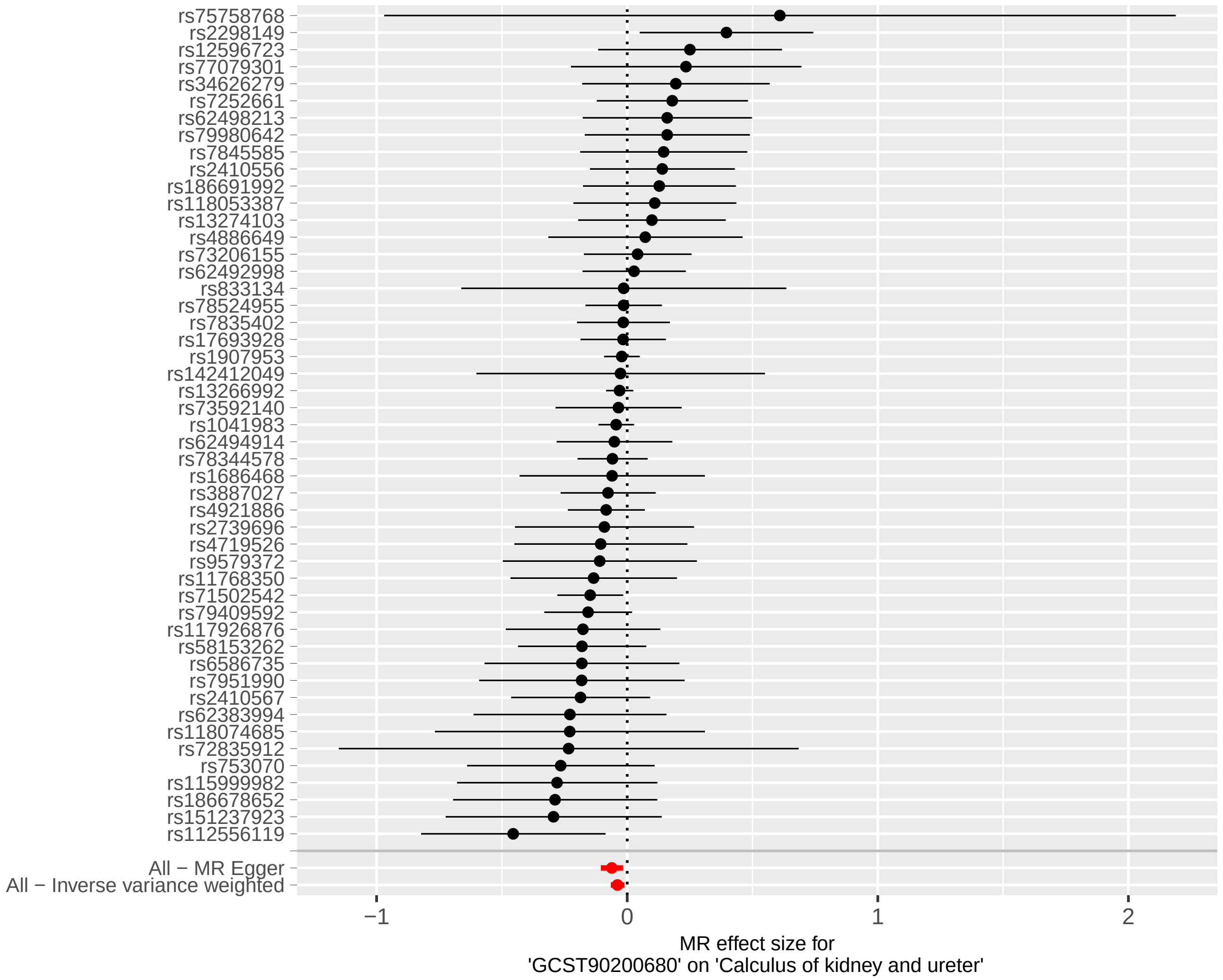
rs75758768
rs2298149
rs12596723
rs77079301
rs34626279
rs7252661
rs62498213
rs79980642
rs7845585
rs2410556
rs186691992
rs118053387
rs13274103
rs4886649
rs73206155
rs62492998
rs833134
rs78524955
rs7835402
rs17693928
rs1907953
rs142412049
rs13266992
rs73592140
rs1041983
rs62494914
rs78344578
rs1686468
rs3887027
rs4921886
rs2739696
rs4719526
rs9579372
rs11768350
rs71502542
rs79409592
rs117926876
rs58153262
rs6586735
rs7951990
rs2410567
rs62383994
rs118074685
rs72835912
rs753070
rs115999982
rs186678652
rs151237923
rs112556119
All − MR Egger
All − Inverse variance weighted
−1
0
1
2
MR effect size for
'GCST90200680' on 'Calculus of kidney and ureter'

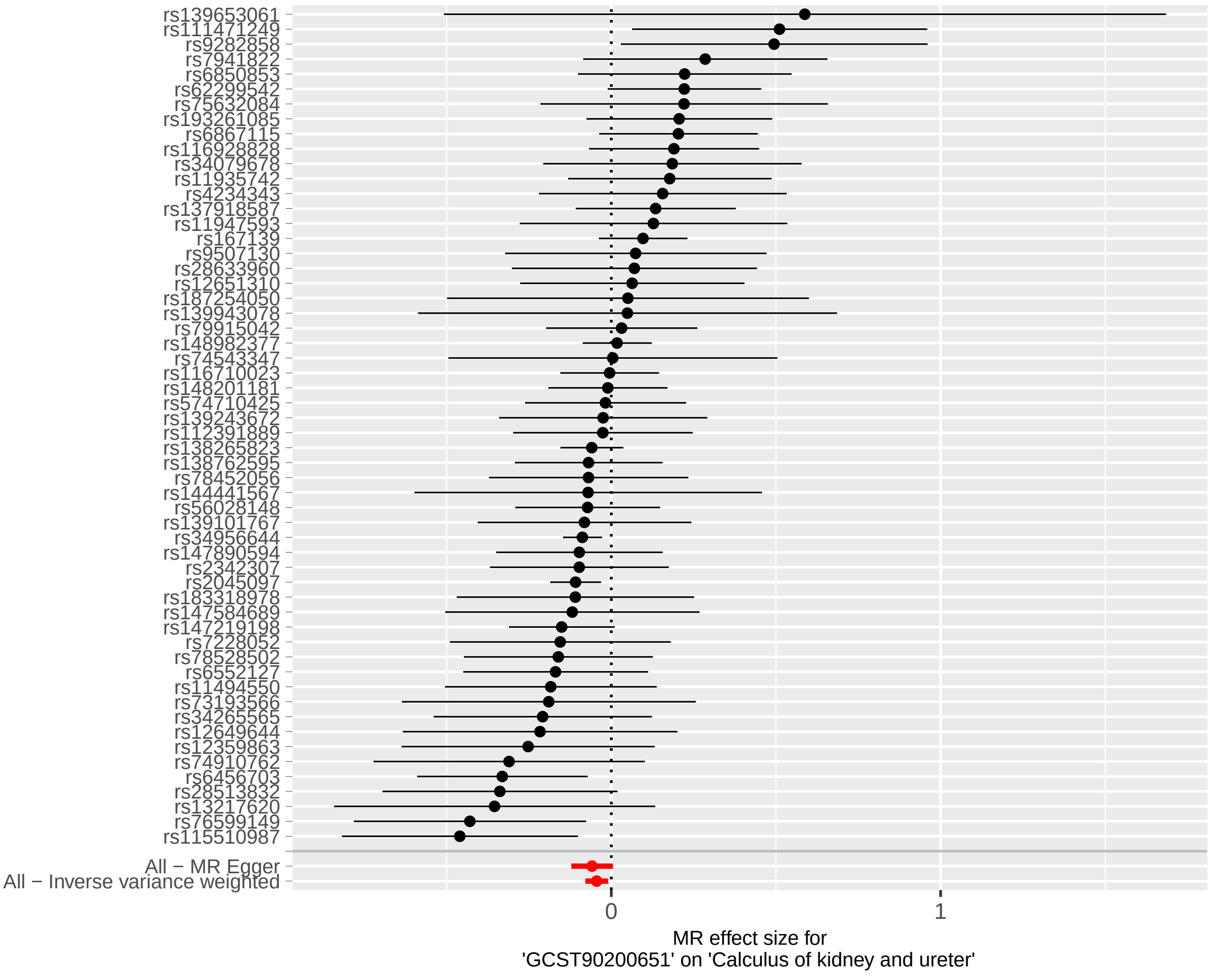

rs139653061
rs111471249
rs9282858
rs7941822
rs6850853
rs62299542
rs75632084
rs193261085
rs6867115
rs116928828
rs34079678
rs11935742
rs4234343
rs137918587
rs11947593
rs167139
rs9507130
rs28633960
rs12651310
rs187254050
rs139943078
rs79915042
rs148982377
rs74543347
rs116710023
rs148201181
rs574710425
rs139243672
rs112391889
rs138265823
rs138762595
rs78452056
rs144441567
rs56028148
rs139101767
rs34956644
rs147890594
rs2342307
rs2045097
rs183318978
rs147584689
rs147219198
rs7228052
rs78528502
rs6552127
rs11494550
rs73193566
rs34265565
rs12649644
rs12359863
rs74910762
rs6456703
rs28513832
rs13217620
rs76599149
rs115510987
All − MR Egger
All − Inverse variance weighted
0
1
MR effect size for
'GCST90200651' on 'Calculus of kidney and ureter'

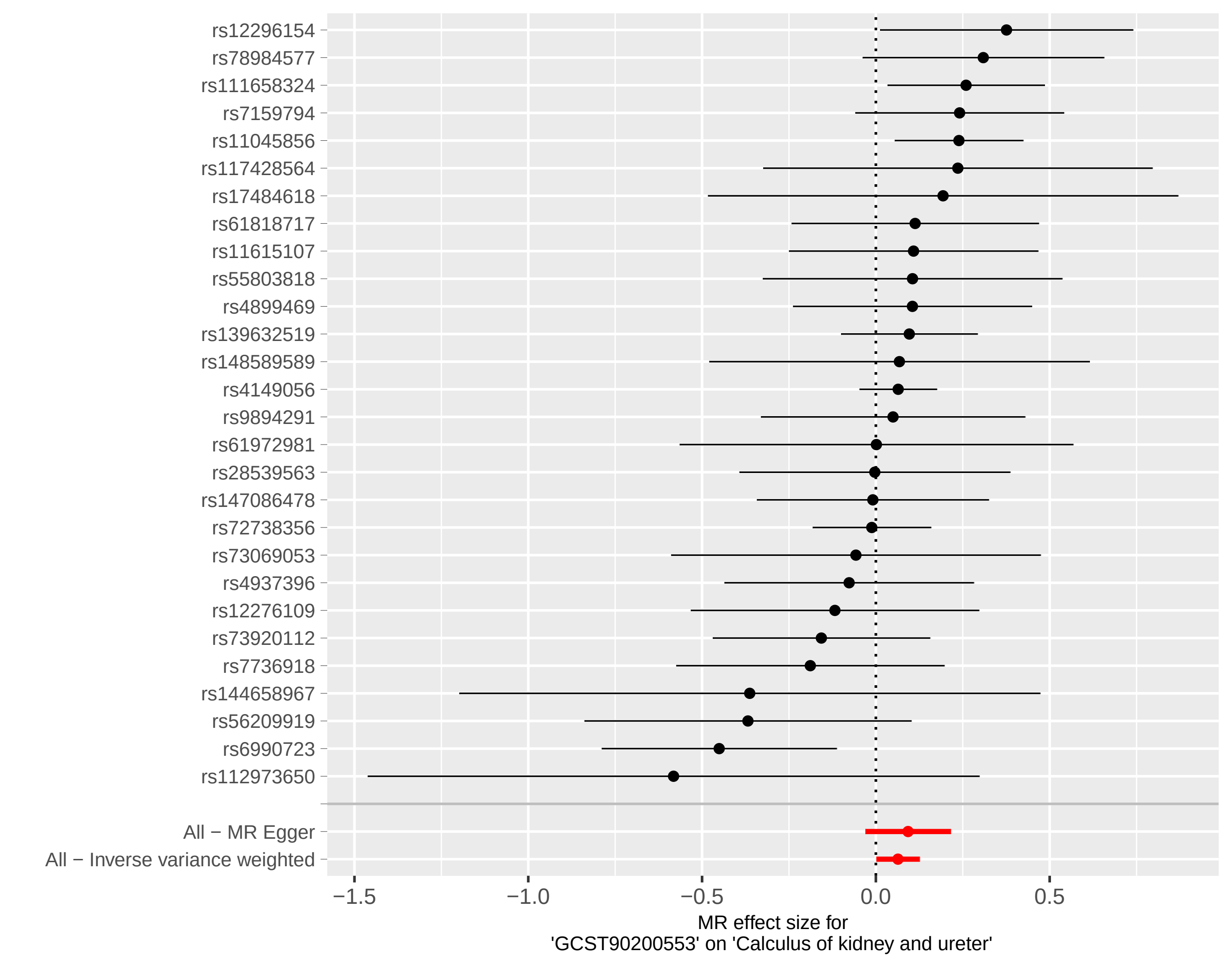
rs12296154
rs78984577
rs111658324
rs7159794
rs11045856
rs117428564
rs17484618
rs61818717
rs11615107
rs55803818
rs4899469
rs139632519
rs148589589
rs4149056
rs9894291
rs61972981
rs28539563
rs147086478
rs72738356
rs73069053
rs4937396
rs12276109
rs73920112
rs7736918
rs144658967
rs56209919
rs6990723
rs112973650
All − MR Egger
All − Inverse variance weighted
−1.5
−1.0
−0.5
0.0
0.5
MR effect size for
'GCST90200553' on 'Calculus of kidney and ureter'

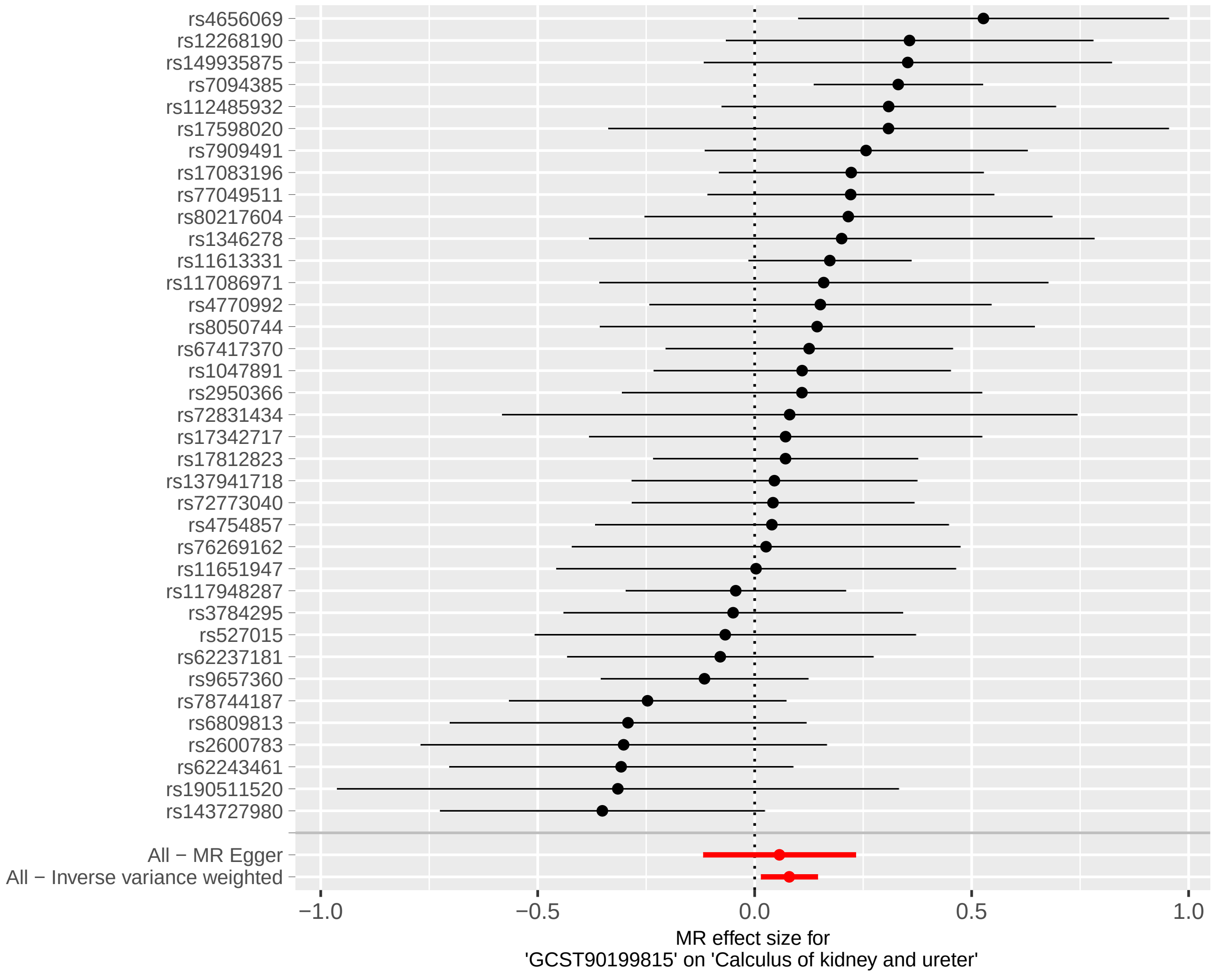
rs4656069
rs12268190
rs149935875
rs7094385
rs112485932
rs17598020
rs7909491
rs17083196
rs77049511
rs80217604
rs1346278
rs11613331
rs117086971
rs4770992
rs8050744
rs67417370
rs1047891
rs2950366
rs72831434
rs17342717
rs17812823
rs137941718
rs72773040
rs4754857
rs76269162
rs11651947
rs117948287
rs3784295
rs527015
rs62237181
rs9657360
rs78744187
rs6809813
rs2600783
rs62243461
rs190511520
rs143727980
All − MR Egger
All − Inverse variance weighted
−1.0
−0.5
0.0
0.5
1.0
MR effect size for
'GCST90199815' on 'Calculus of kidney and ureter'

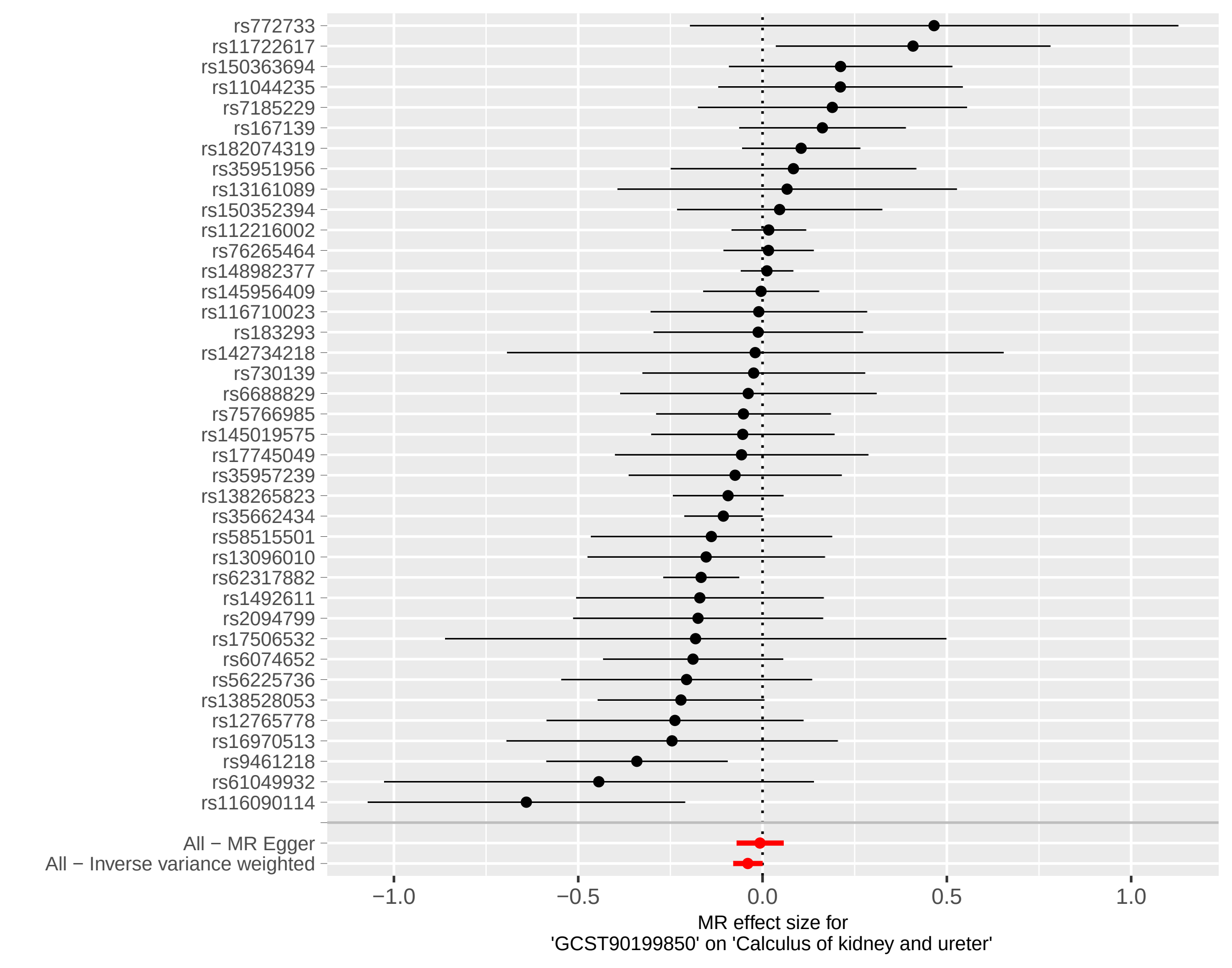

rs772733
rs11722617
rs150363694
rs11044235
rs7185229
rs167139
rs182074319
rs35951956
rs13161089
rs150352394
rs112216002
rs76265464
rs148982377
rs145956409
rs116710023
rs183293
rs142734218
rs730139
rs6688829
rs75766985
rs145019575
rs17745049
rs35957239
rs138265823
rs35662434
rs58515501
rs13096010
rs62317882
rs1492611
rs2094799
rs17506532
rs6074652
rs56225736
rs138528053
rs12765778
rs16970513
rs9461218
rs61049932
rs116090114
All − MR Egger
All − Inverse variance weighted
−1.0
−0.5
0.0
0.5
1.0
MR effect size for
'GCST90199850' on 'Calculus of kidney and ureter'

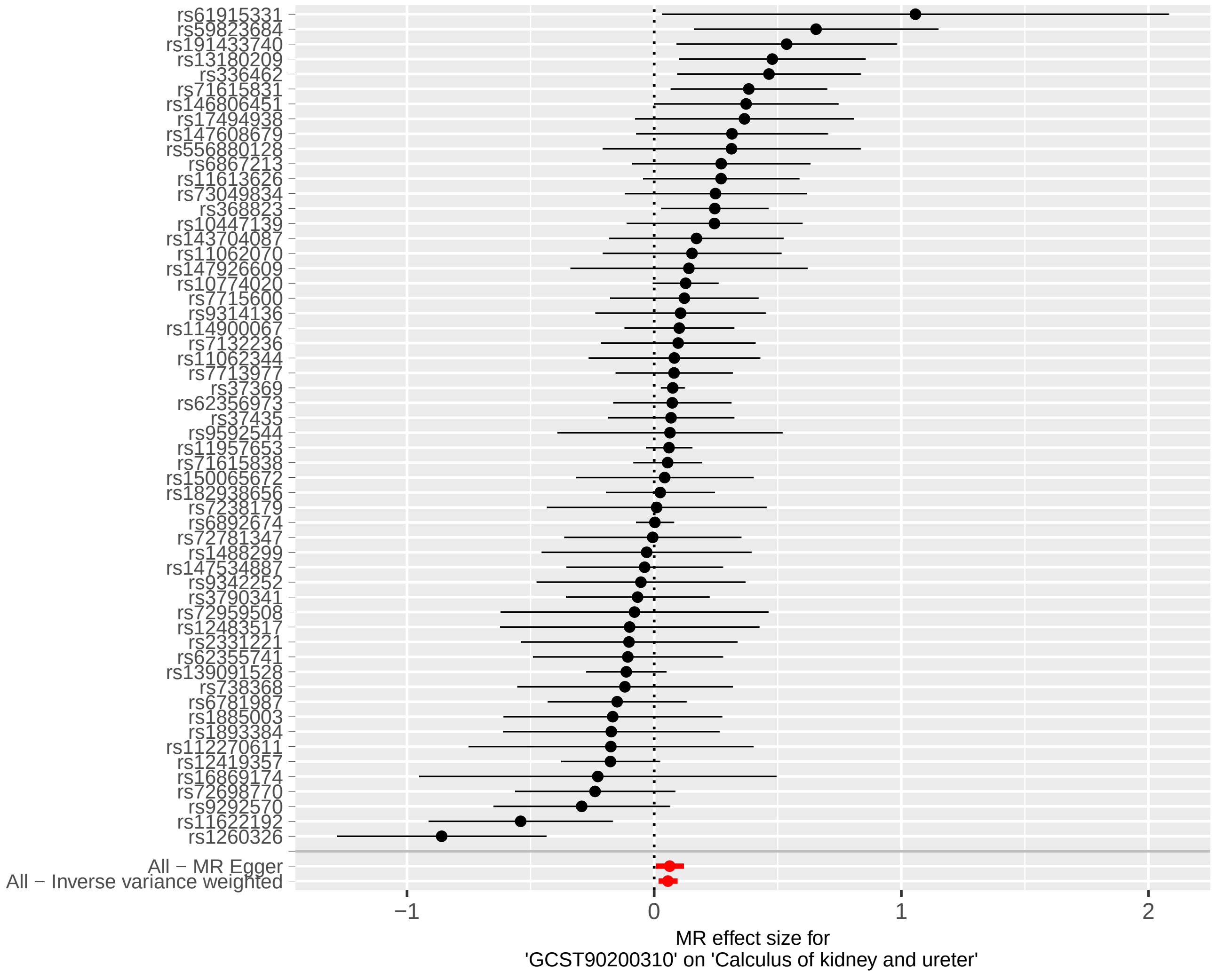
rs61915331
rs59823684
rs191433740
rs13180209
rs336462
rs71615831
rs146806451
rs17494938
rs147608679
rs556880128
rs6867213
rs11613626
rs73049834
rs368823
rs10447139
rs143704087
rs11062070
rs147926609
rs10774020
rs7715600
rs9314136
rs114900067
rs7132236
rs11062344
rs7713977
rs37369
rs62356973
rs37435
rs9592544
rs11957653
rs71615838
rs150065672
rs182938656
rs7238179
rs6892674
rs72781347
rs1488299
rs147534887
rs9342252
rs3790341
rs72959508
rs12483517
rs2331221
rs62355741
rs139091528
rs738368
rs6781987
rs1885003
rs1893384
rs112270611
rs12419357
rs16869174
rs72698770
rs9292570
rs11622192
rs1260326
All − MR Egger
All − Inverse variance weighted
−1
0
1
2
MR effect size for
'GCST90200310' on 'Calculus of kidney and ureter'

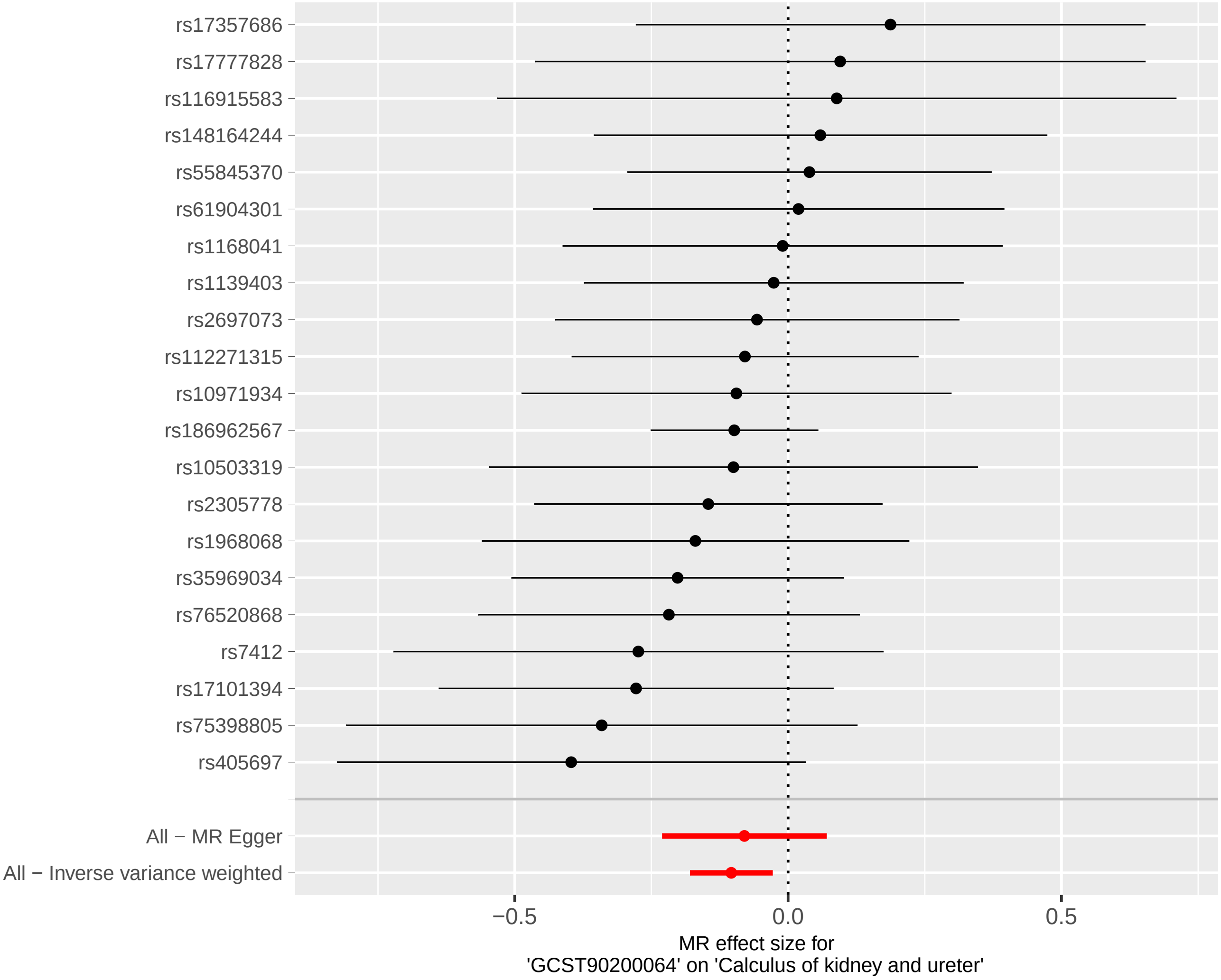
rs17357686
rs17777828
rs116915583
rs148164244
rs55845370
rs61904301
rs1168041
rs1139403
rs2697073
rs112271315
rs10971934
rs186962567
rs10503319
rs2305778
rs1968068
rs35969034
rs76520868
rs7412
rs17101394
rs75398805
rs405697
All − MR Egger
All − Inverse variance weighted
−0.5
0.0
0.5
MR effect size for
'GCST90200064' on 'Calculus of kidney and ureter'

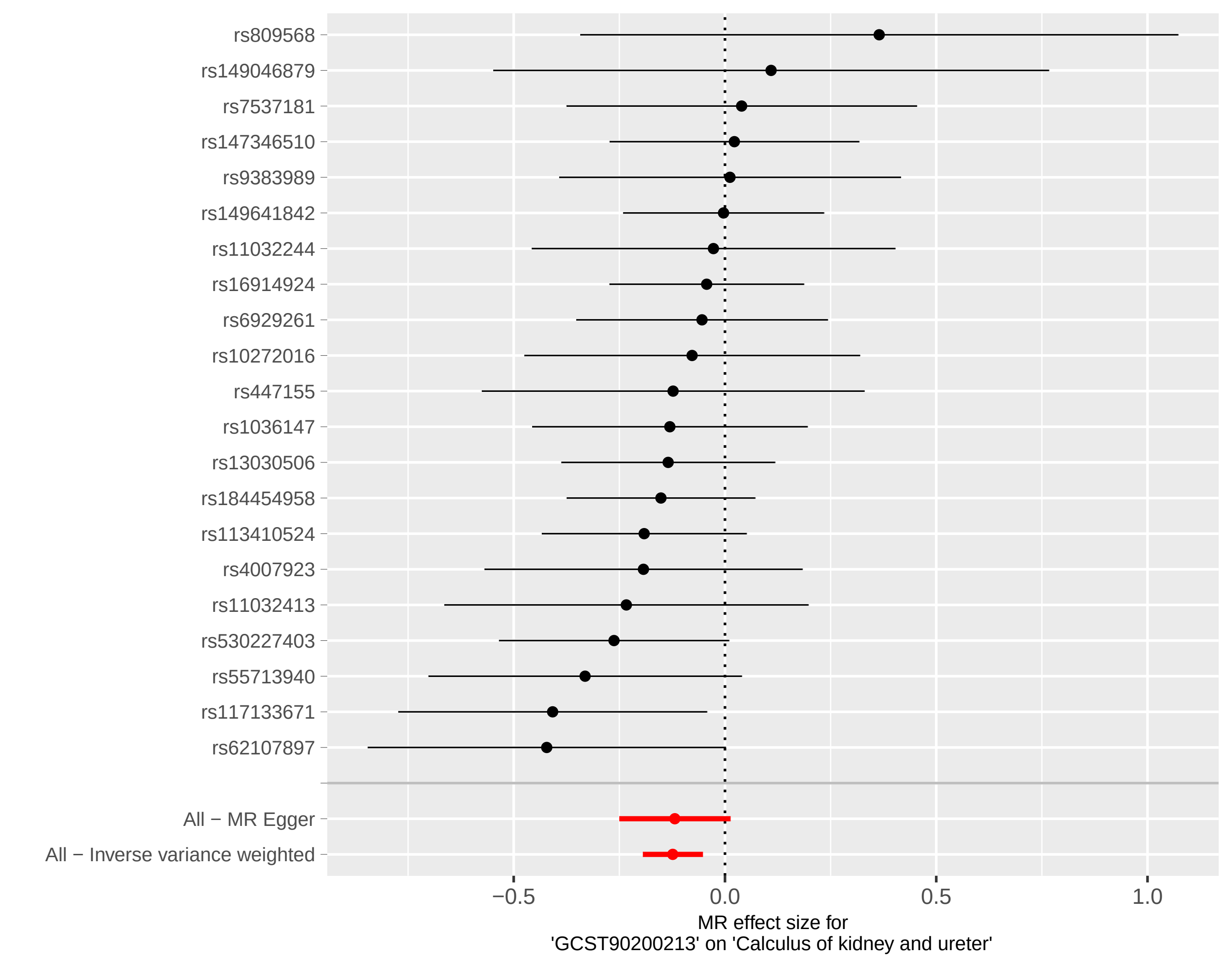

rs809568
rs149046879
rs7537181
rs147346510
rs9383989
rs149641842
rs11032244
rs16914924
rs6929261
rs10272016
rs447155
rs1036147
rs13030506
rs184454958
rs113410524
rs4007923
rs11032413
rs530227403
rs55713940
rs117133671
rs62107897
All − MR Egger
All − Inverse variance weighted
−0.5
0.0
0.5
1.0
MR effect size for
'GCST90200213' on 'Calculus of kidney and ureter'

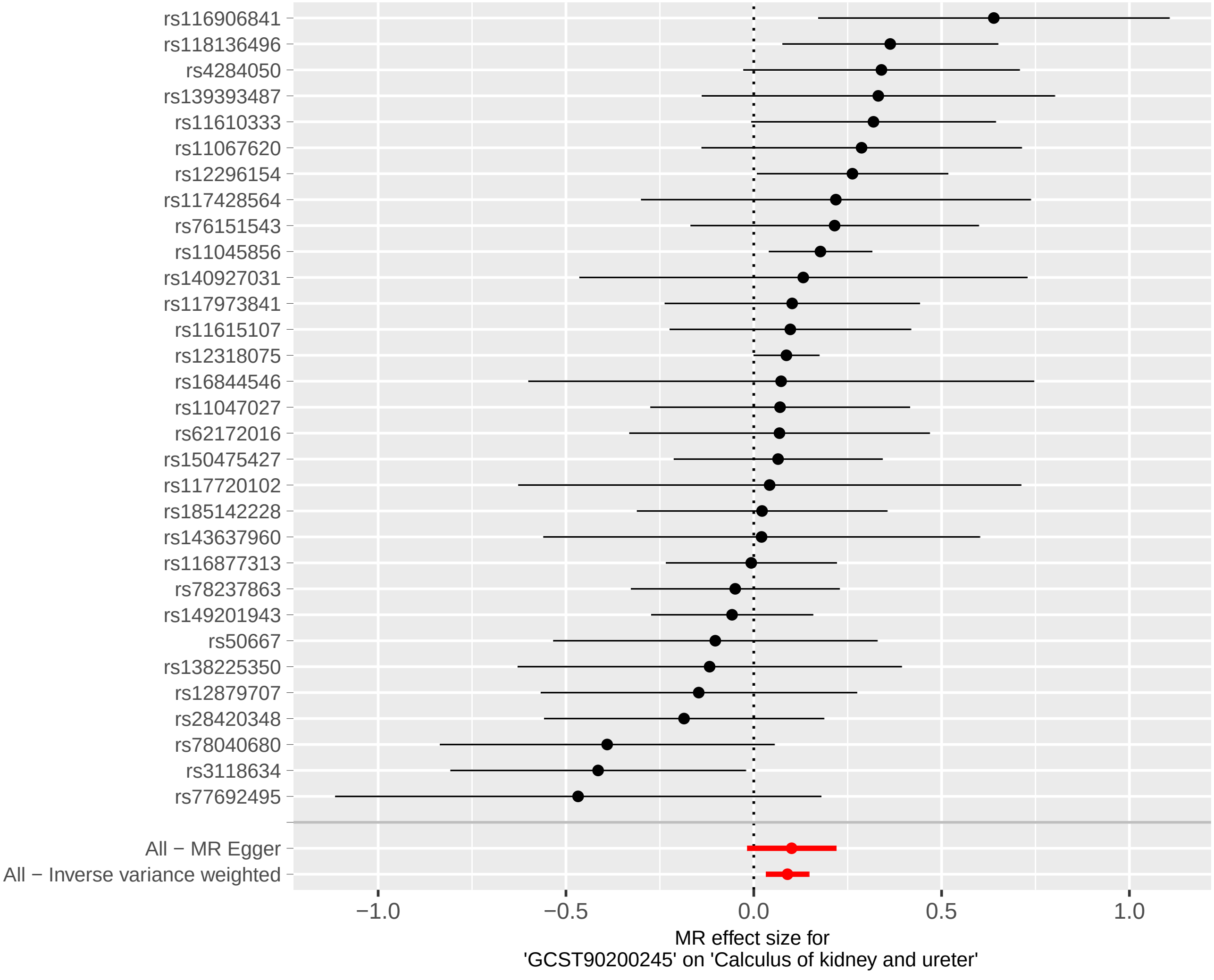
rs116906841
rs118136496
rs4284050
rs139393487
rs11610333
rs11067620
rs12296154
rs117428564
rs76151543
rs11045856
rs140927031
rs117973841
rs11615107
rs12318075
rs16844546
rs11047027
rs62172016
rs150475427
rs117720102
rs185142228
rs143637960
rs116877313
rs78237863
rs149201943
rs50667
rs138225350
rs12879707
rs28420348
rs78040680
rs3118634
rs77692495
All − MR Egger
All − Inverse variance weighted
−1.0
−0.5
0.0
0.5
1.0
MR effect size for
'GCST90200245' on 'Calculus of kidney and ureter'

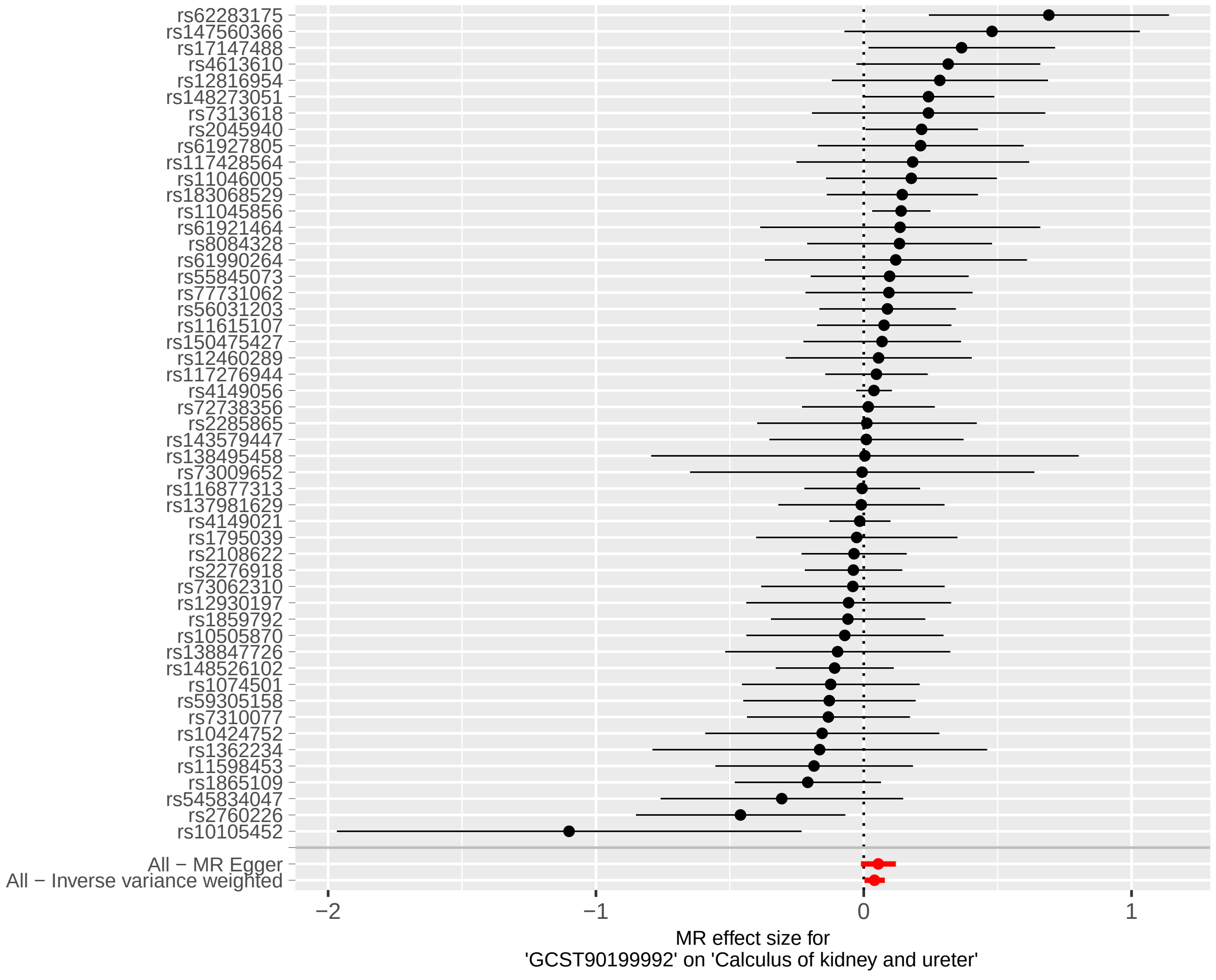
rs62283175
rs147560366
rs17147488
rs4613610
rs12816954
rs148273051
rs7313618
rs2045940
rs61927805
rs117428564
rs11046005
rs183068529
rs11045856
rs61921464
rs8084328
rs61990264
rs55845073
rs77731062
rs56031203
rs11615107
rs150475427
rs12460289
rs117276944
rs4149056
rs72738356
rs2285865
rs143579447
rs138495458
rs73009652
rs116877313
rs137981629
rs4149021
rs1795039
rs2108622
rs2276918
rs73062310
rs12930197
rs1859792
rs10505870
rs138847726
rs148526102
rs1074501
rs59305158
rs7310077
rs10424752
rs1362234
rs11598453
rs1865109
rs545834047
rs2760226
rs10105452
All − MR Egger
All − Inverse variance weighted
−2
−1
0
1
MR effect size for
'GCST90199992' on 'Calculus of kidney and ureter'

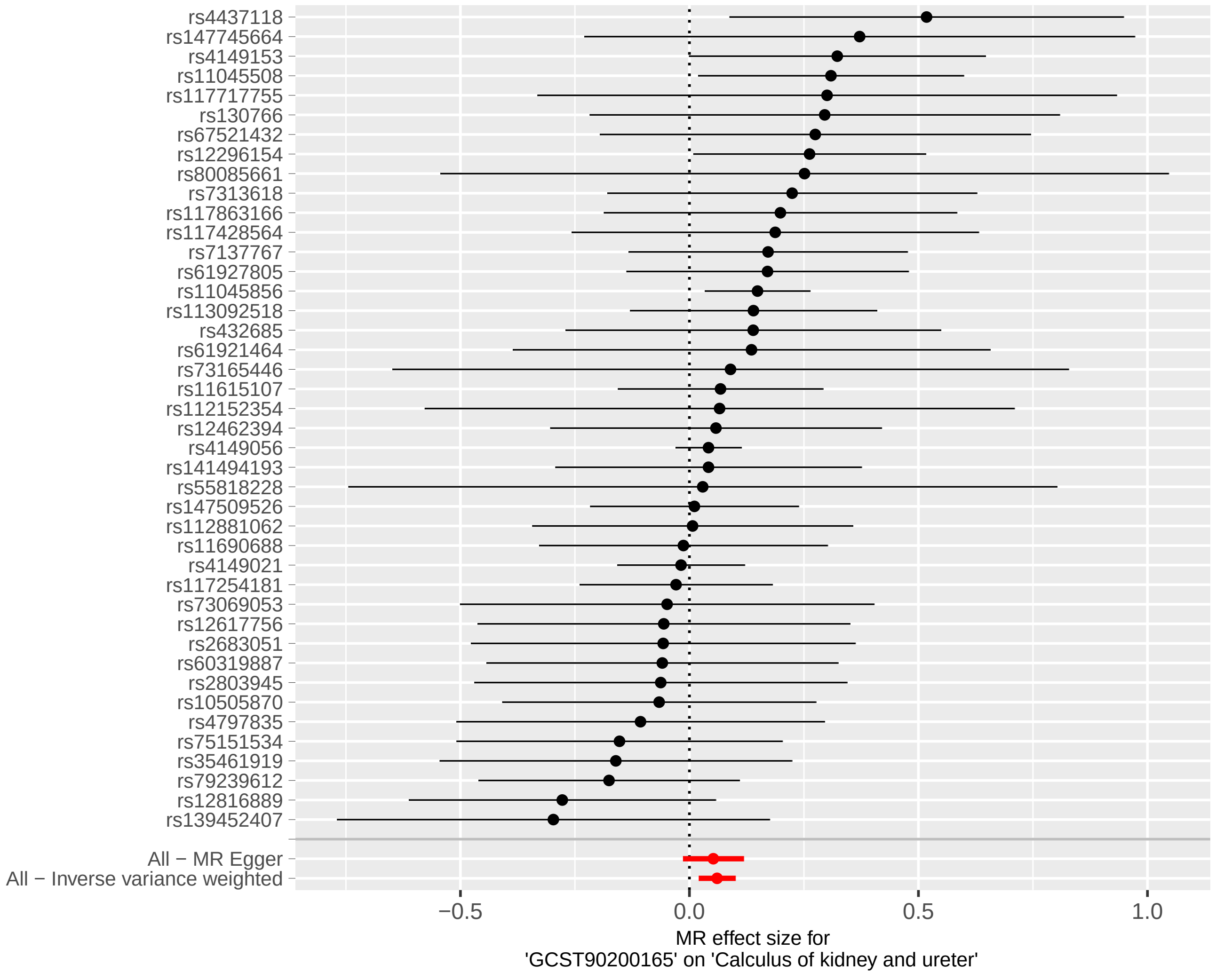
rs4437118
rs147745664
rs4149153
rs11045508
rs117717755
rs130766
rs67521432
rs12296154
rs80085661
rs7313618
rs117863166
rs117428564
rs7137767
rs61927805
rs11045856
rs113092518
rs432685
rs61921464
rs73165446
rs11615107
rs112152354
rs12462394
rs4149056
rs141494193
rs55818228
rs147509526
rs112881062
rs11690688
rs4149021
rs117254181
rs73069053
rs12617756
rs2683051
rs60319887
rs2803945
rs10505870
rs4797835
rs75151534
rs35461919
rs79239612
rs12816889
rs139452407
All − MR Egger
All − Inverse variance weighted
−0.5
0.0
0.5
1.0
MR effect size for
'GCST90200165' on 'Calculus of kidney and ureter'

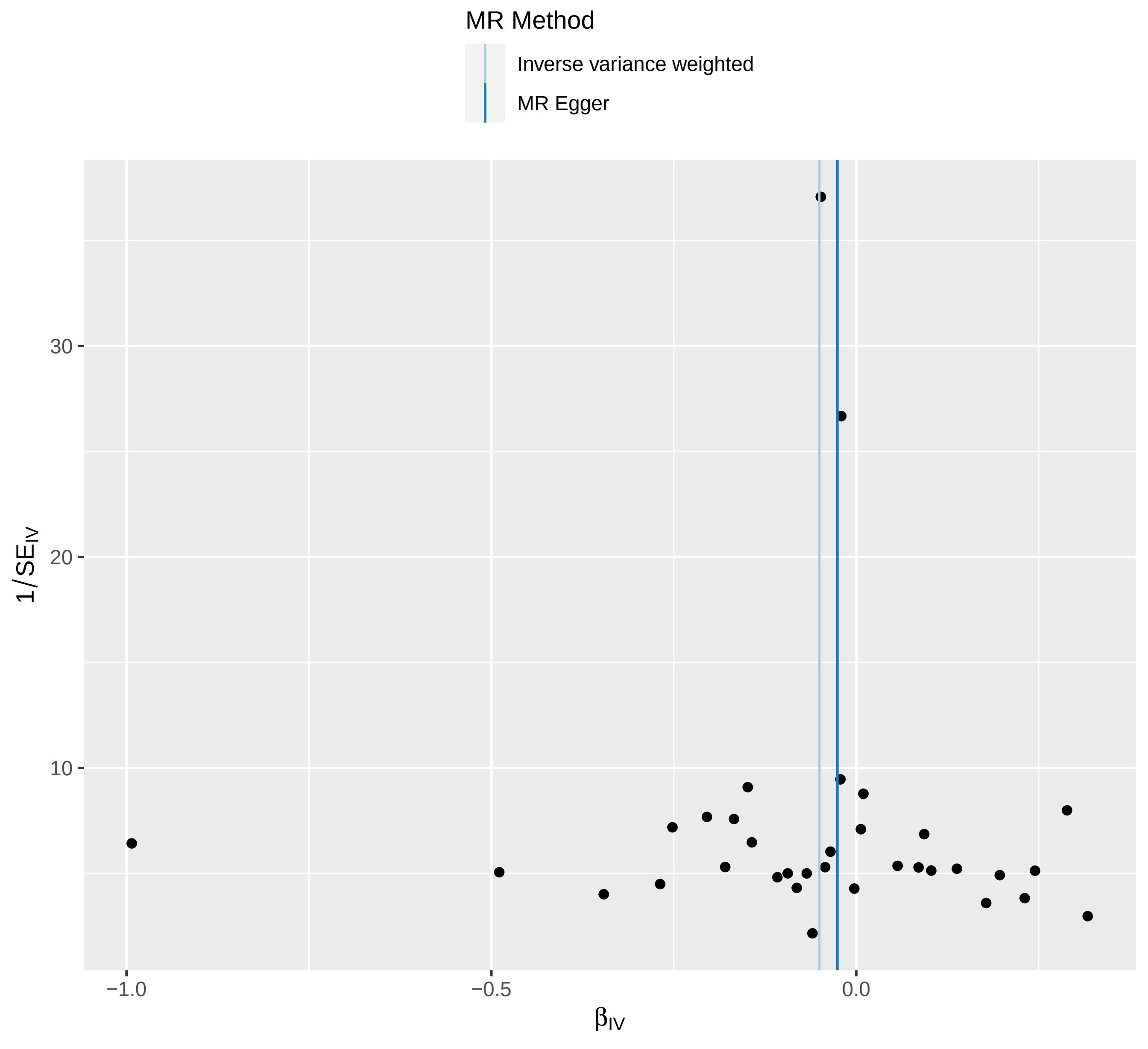
MR Method
Inverse variance weighted
MR Egger
30
20
10
1/SE$_{IV}$
−1.0
−0.5
0.0
β$_{IV}$

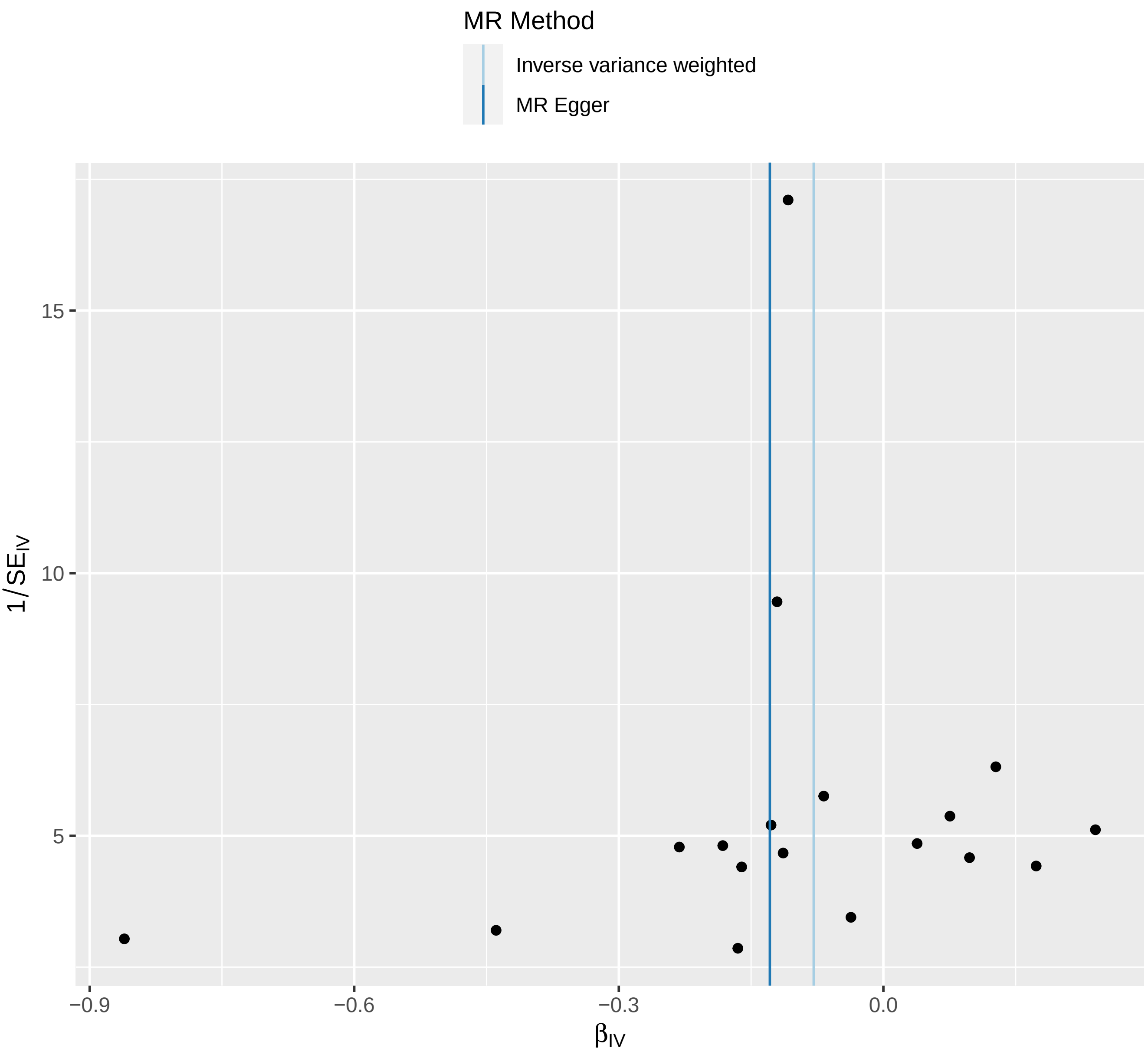
MR Method
Inverse variance weighted
MR Egger
1/SE$_{IV}$
15
10
5
−0.9
−0.6
−0.3
0.0
β$_{IV}$

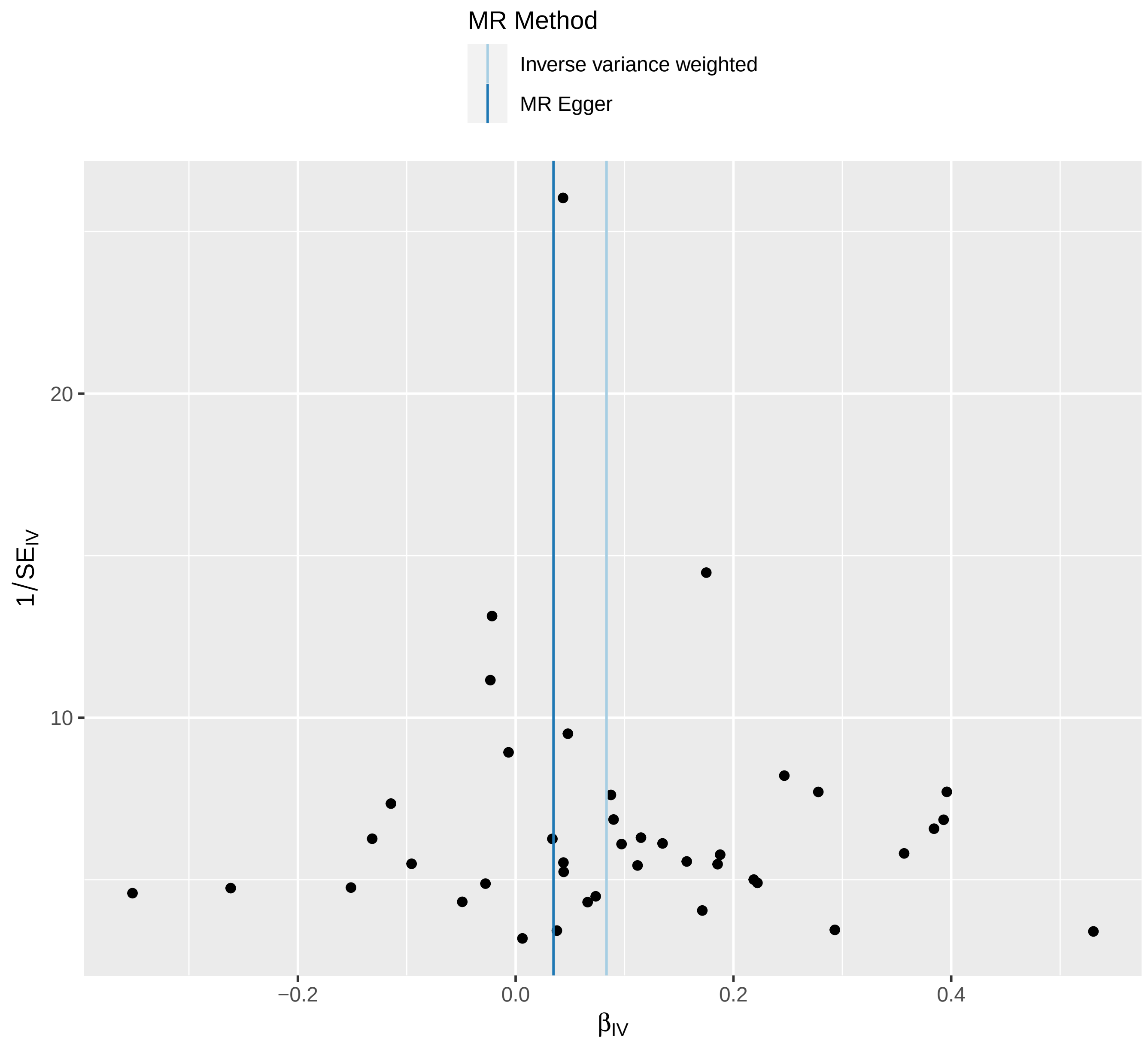
MR Method
Inverse variance weighted
MR Egger
20
10
$1/SE_{IV}$
−0.2
0.0
0.2
0.4
β_{IV}

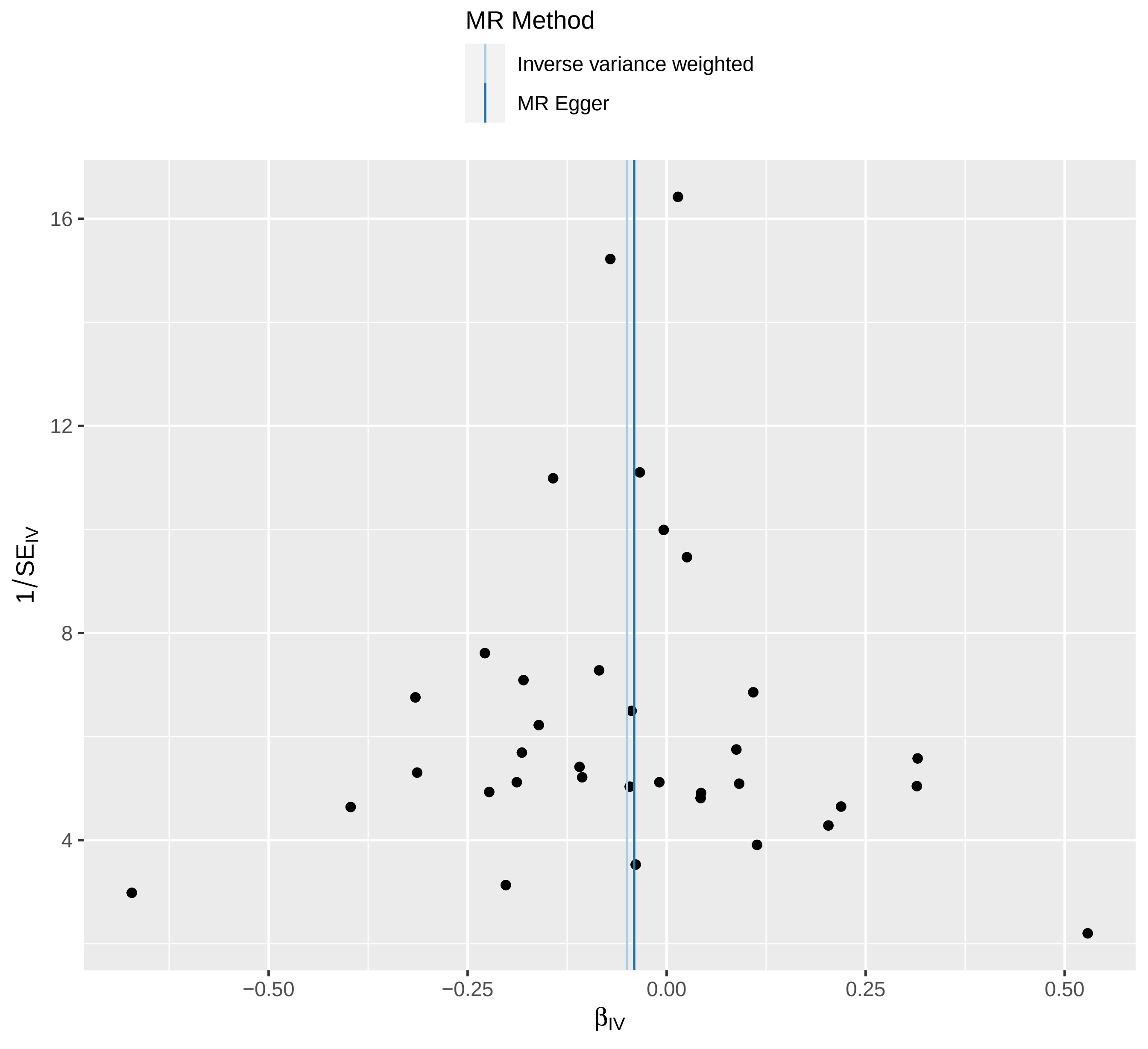

MR Method
Inverse variance weighted
MR Egger
16
12
8
4
$1/SE_{IV}$
−0.50
−0.25
0.00
0.25
0.50
β_{IV}

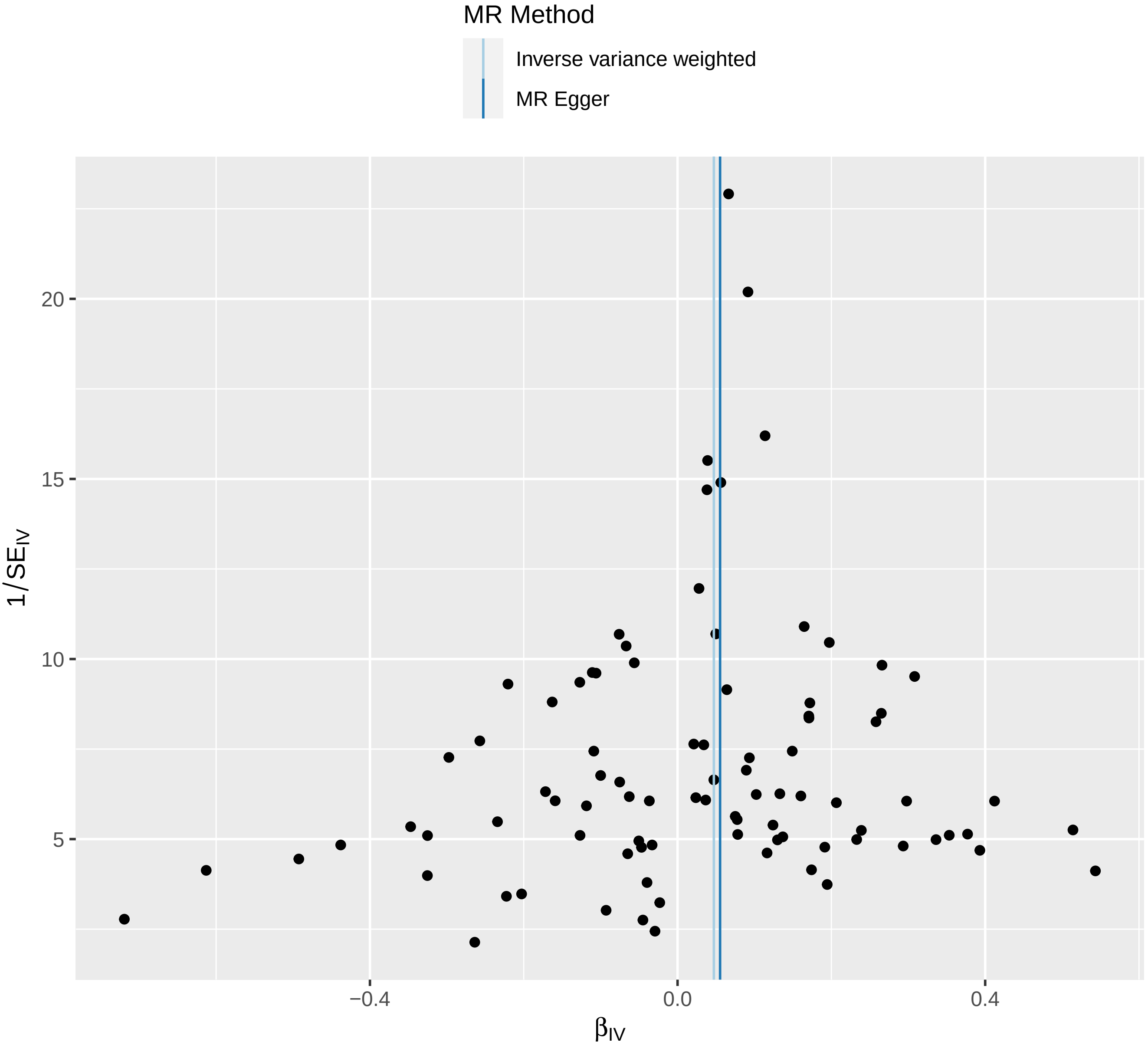
MR Method
Inverse variance weighted
MR Egger
20
15
10
5
1/SE_IV
−0.4
0.0
0.4
β_IV

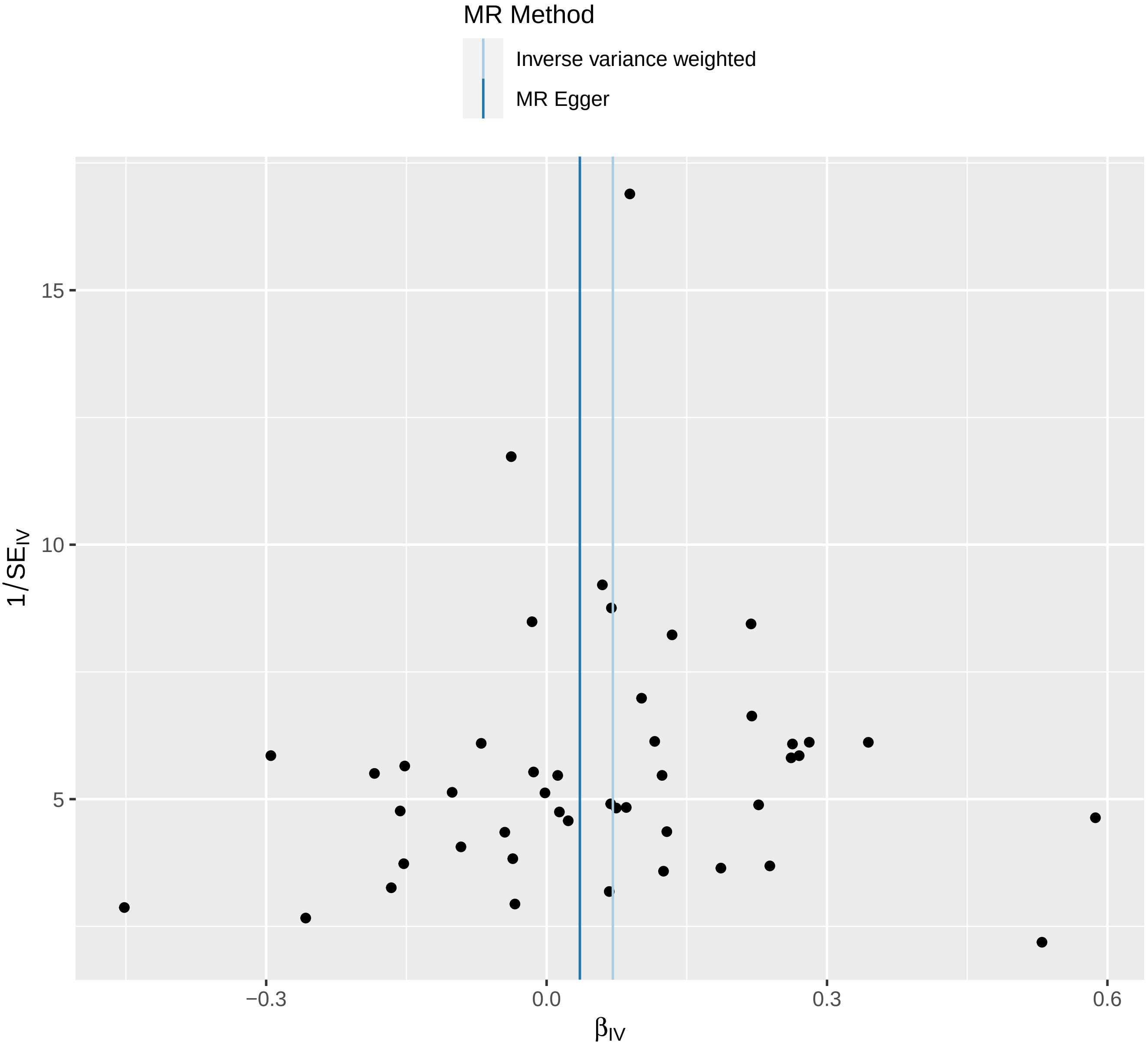
MR Method
Inverse variance weighted
MR Egger
15
10
5
$1/SE_{IV}$
−0.3
0.0
0.3
0.6
β_{IV}

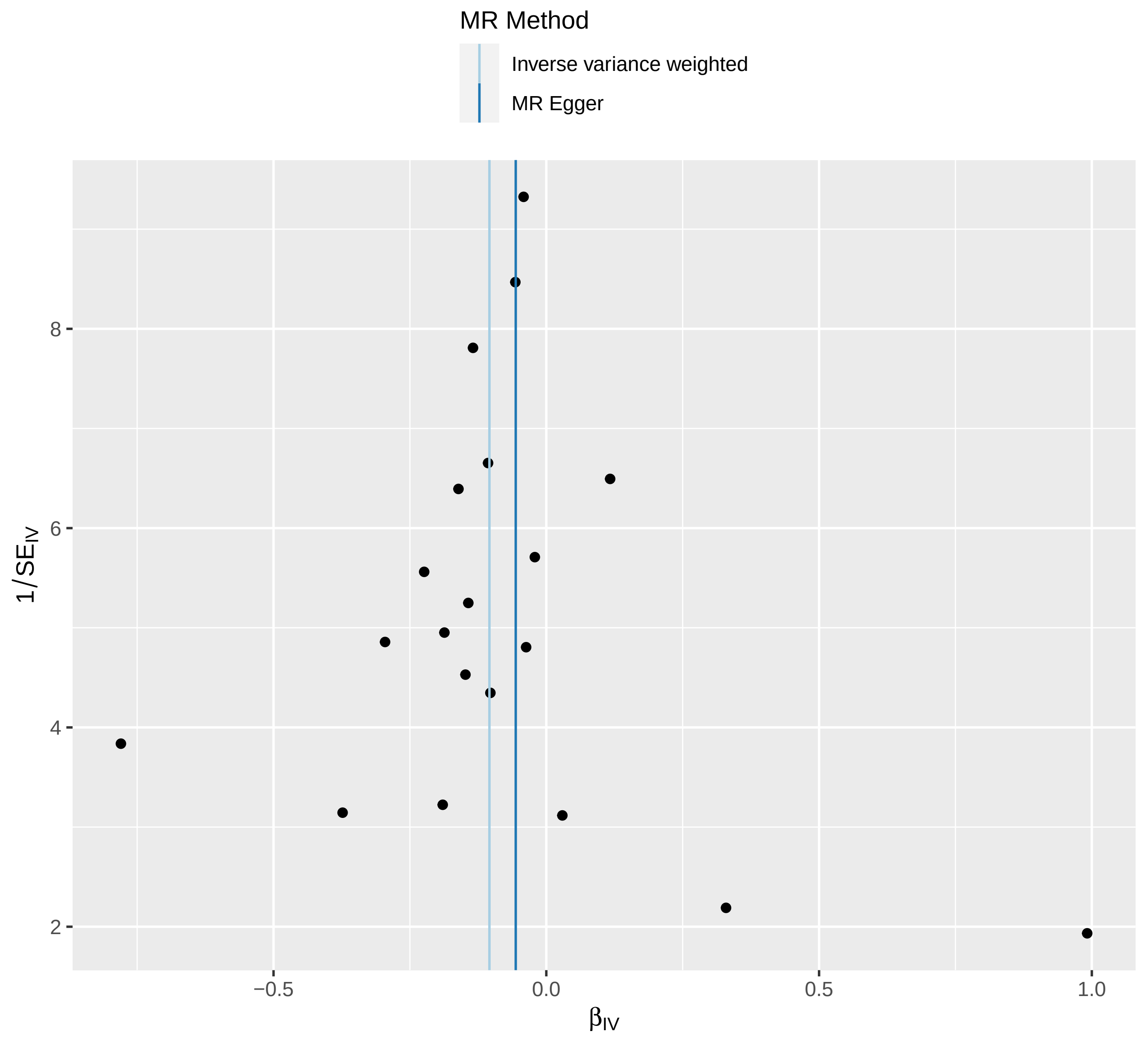

MR Method
Inverse variance weighted
MR Egger
8
6
4
2
$1/SE_{IV}$
−0.5
0.0
0.5
1.0
β_{IV}

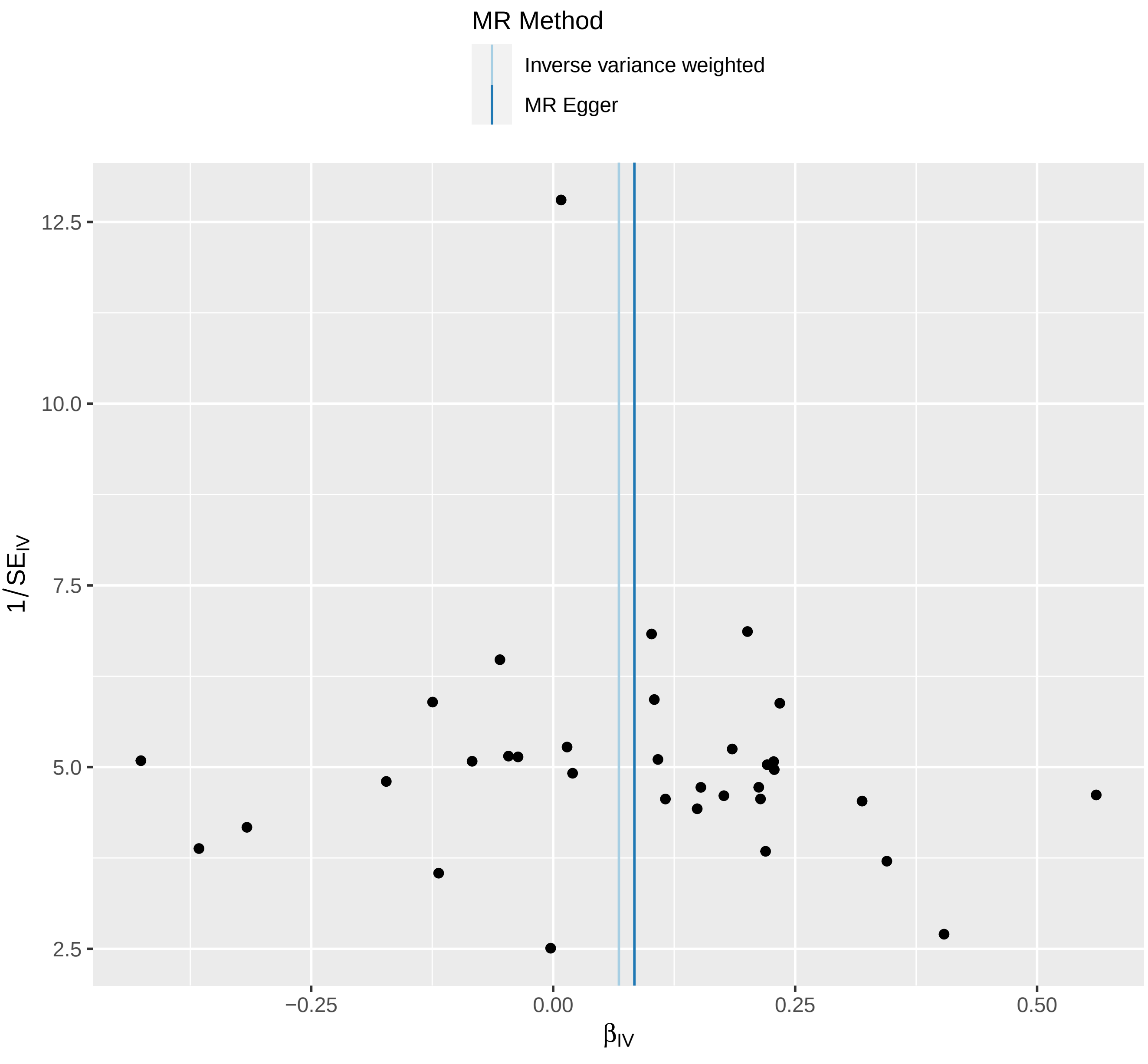
MR Method
Inverse variance weighted
MR Egger
12.5
10.0
7.5
5.0
2.5
$1/SE_{IV}$
−0.25
0.00
0.25
0.50
β_{IV}

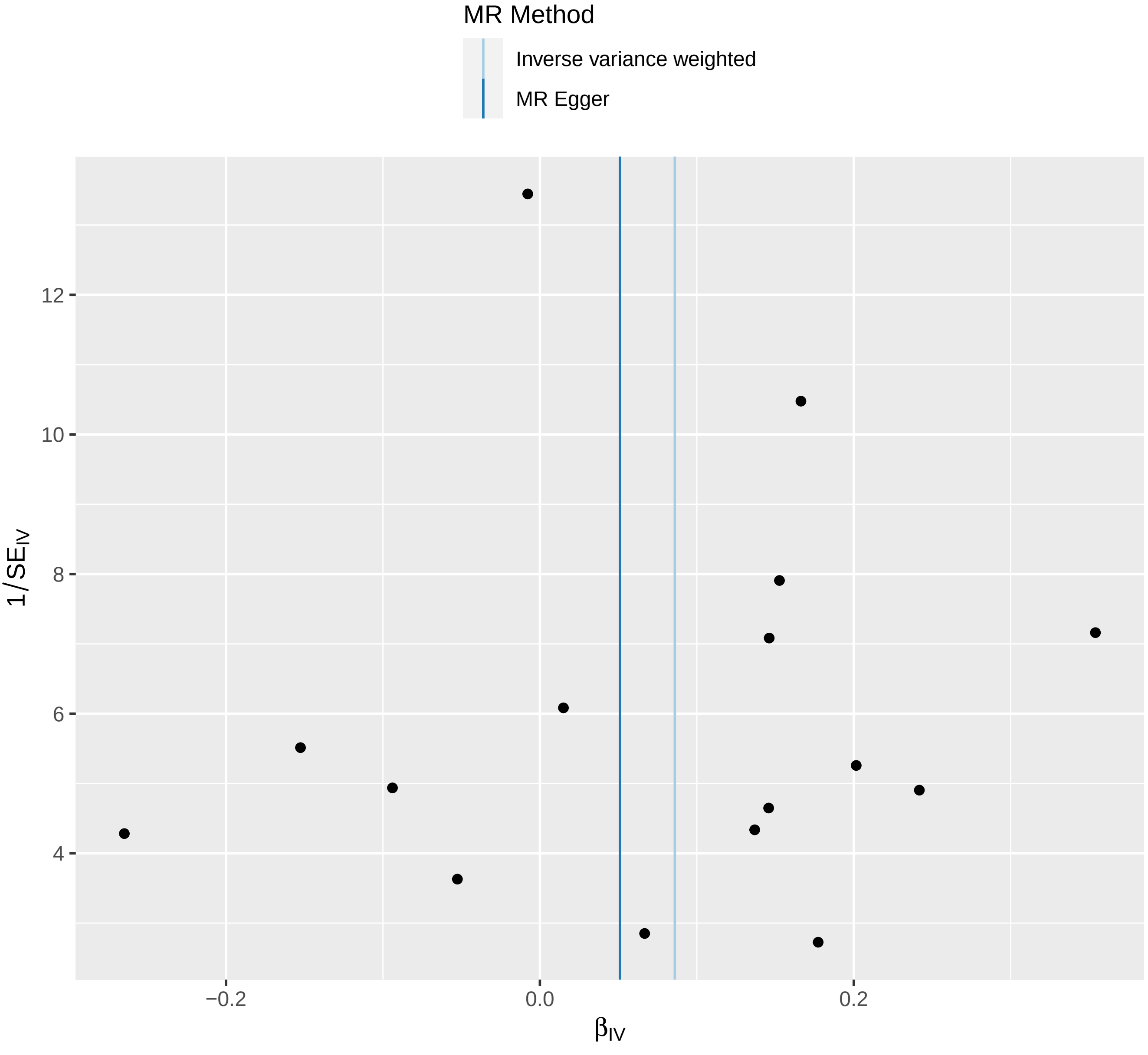

MR Method
Inverse variance weighted
MR Egger
12
10
8
6
4
−0.2
0.0
0.2
$1/SE_{IV}$
β_{IV}

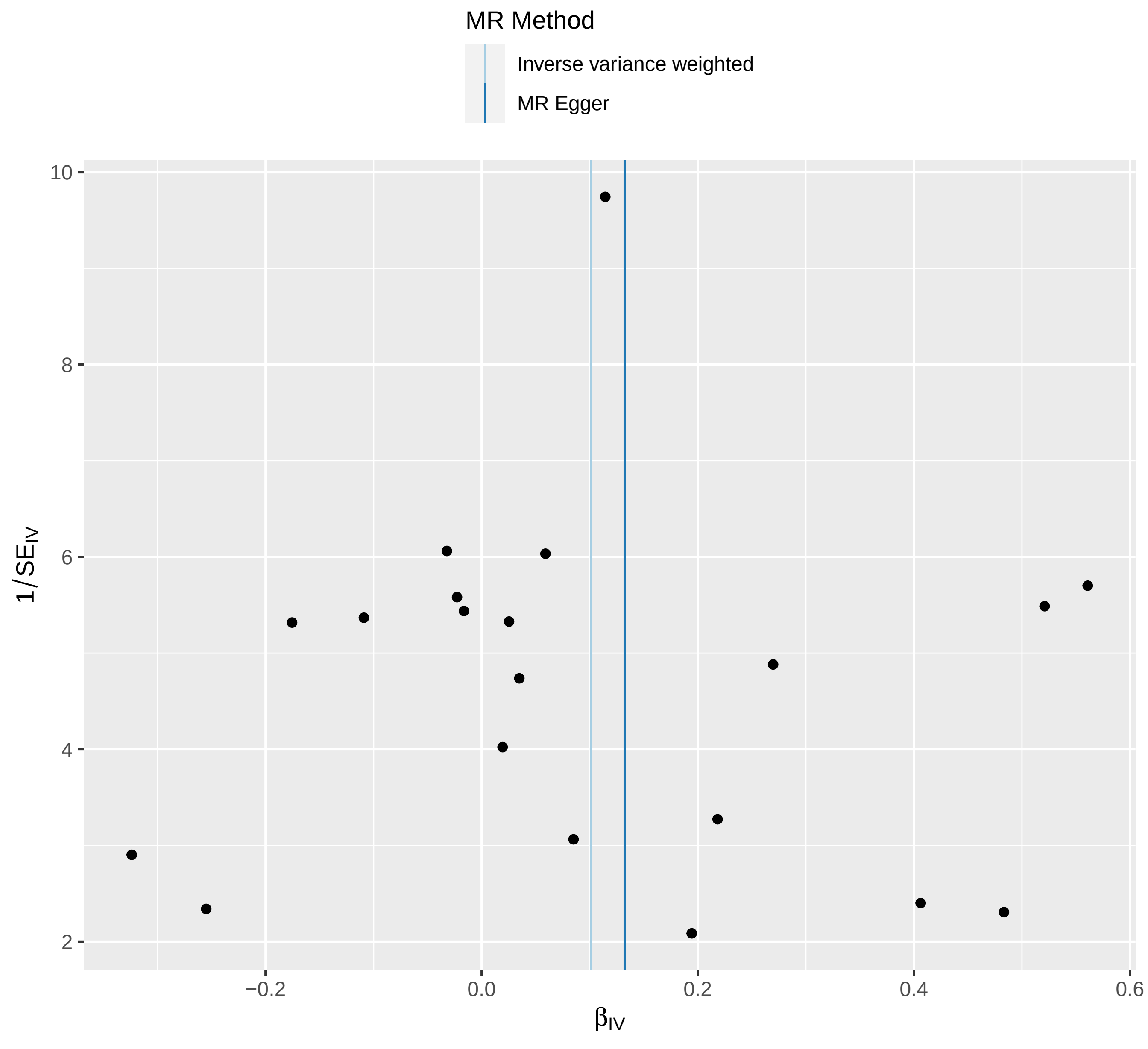

MR Method
Inverse variance weighted
MR Egger
10
8
6
4
2
$1/SE_{IV}$
−0.2
0.0
0.2
0.4
0.6
β_{IV}

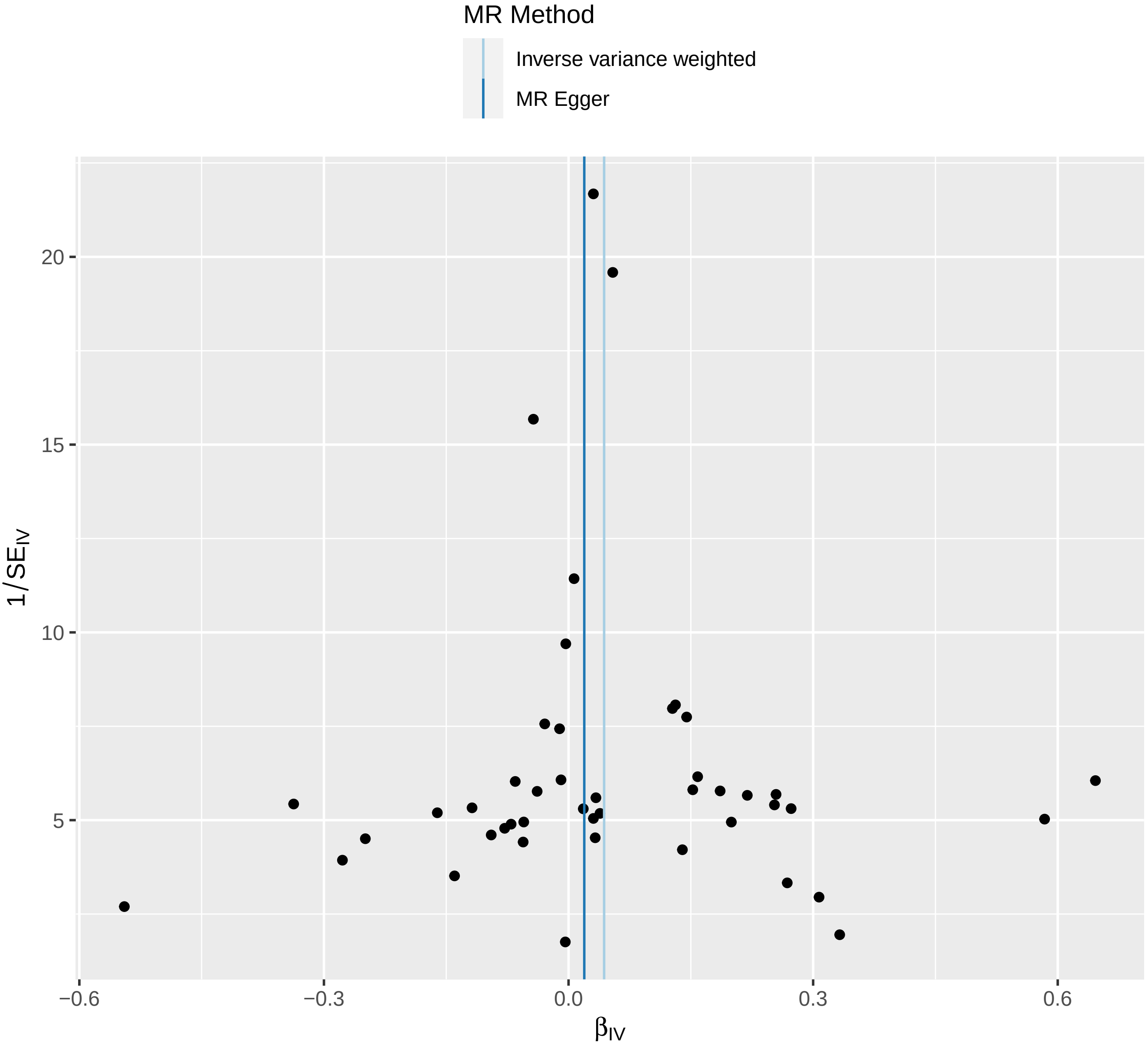
MR Method
Inverse variance weighted
MR Egger
20
15
10
5
$1/SE_{IV}$
−0.6
−0.3
0.0
0.3
0.6
β_{IV}

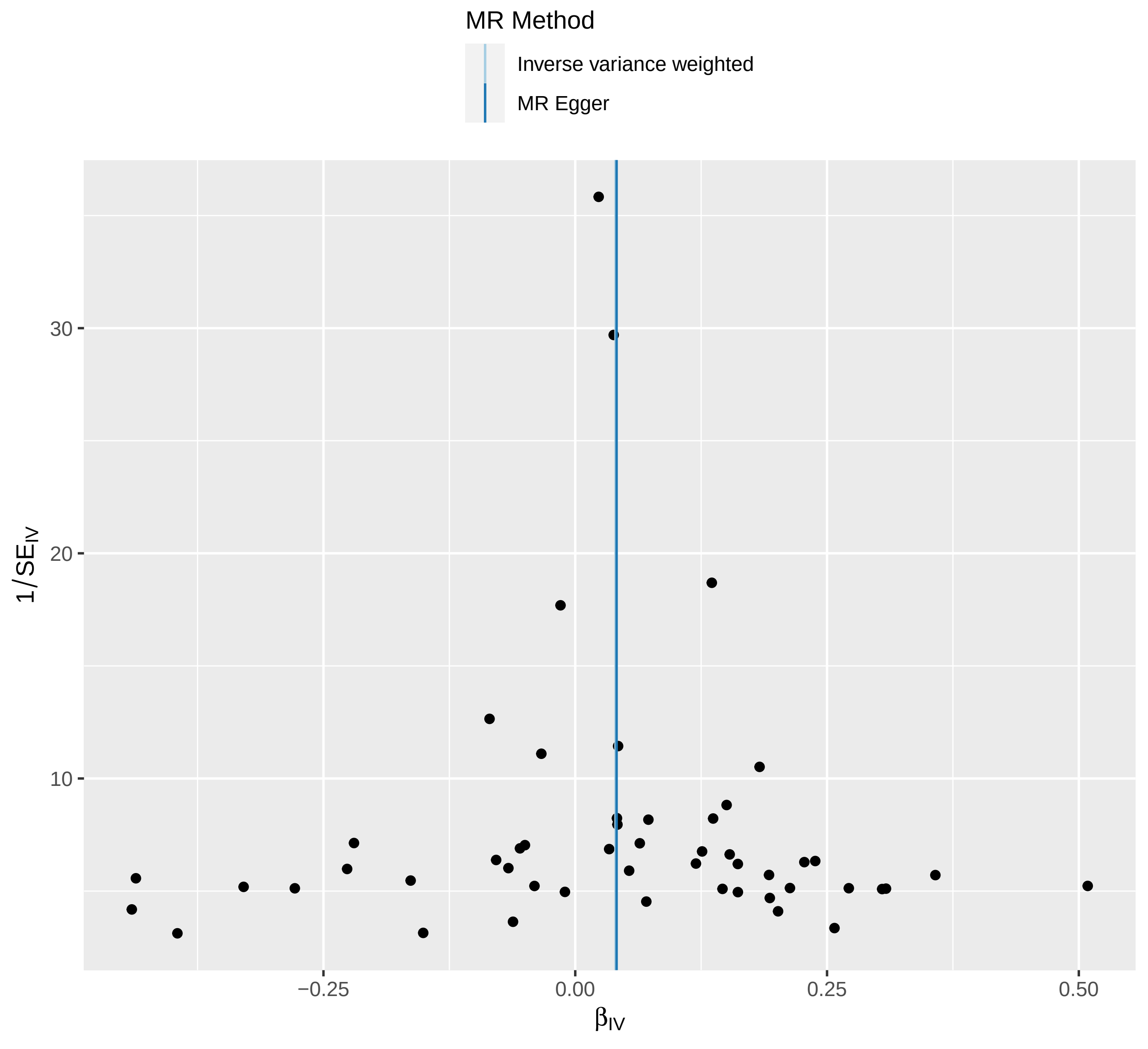

MR Method
Inverse variance weighted
MR Egger
30
20
10
1/SE_IV
−0.25
0.00
0.25
0.50
β_IV

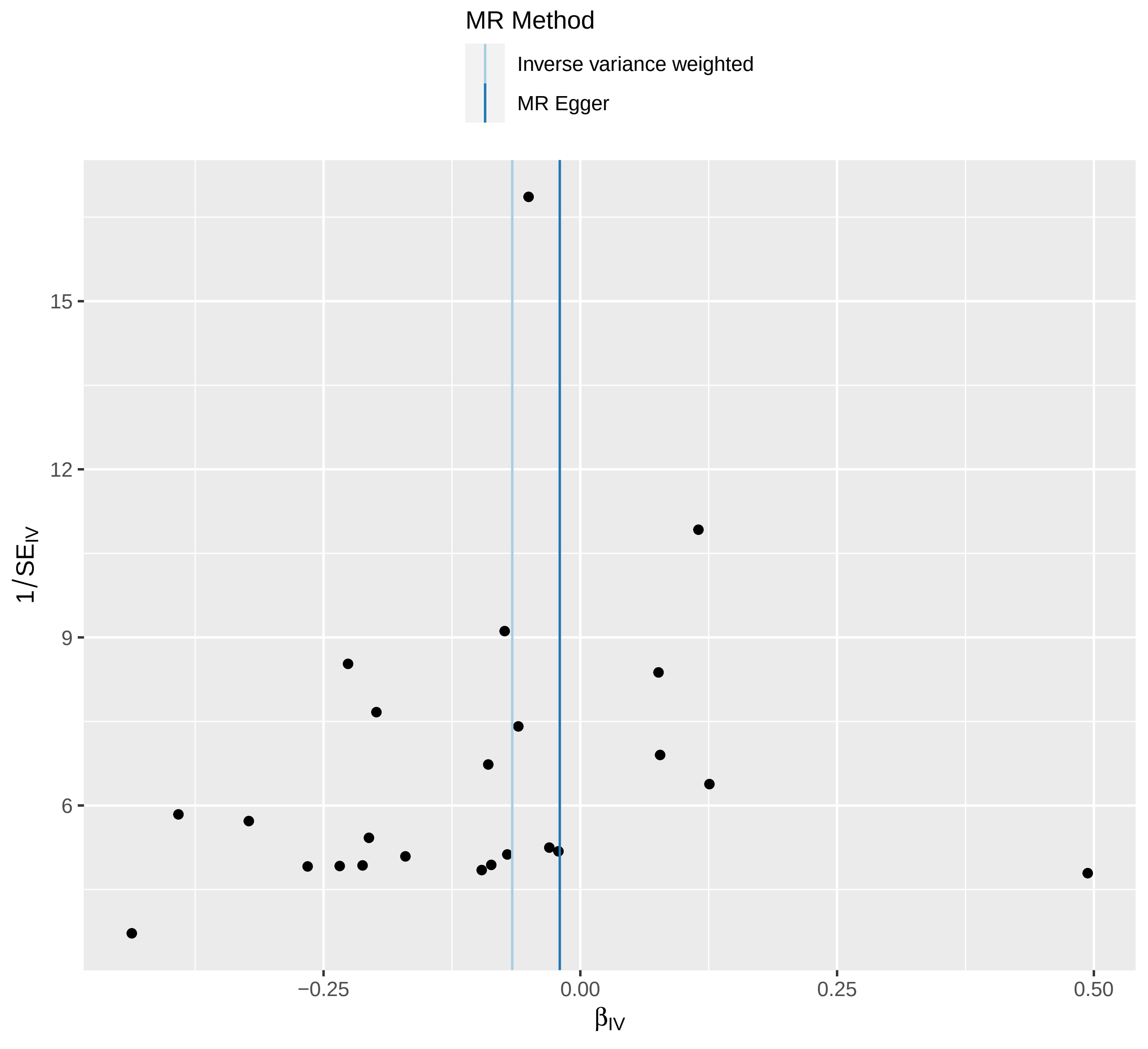
MR Method
Inverse variance weighted
MR Egger
15
12
9
6
1/SE_IV
−0.25
0.00
0.25
0.50
β_IV

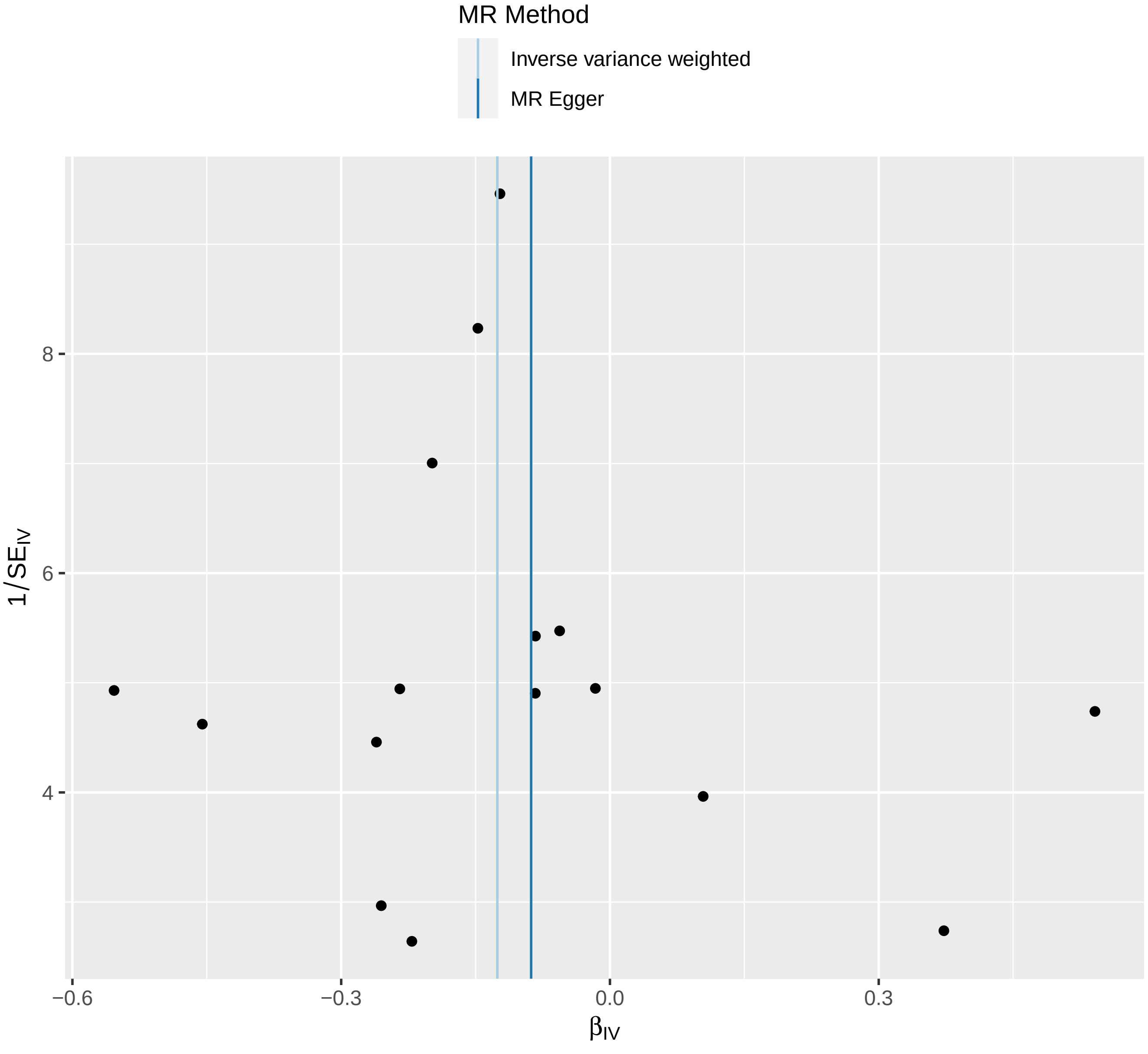

MR Method
Inverse variance weighted
MR Egger
8
6
4
1/SE_IV
−0.6
−0.3
0.0
0.3
β_IV

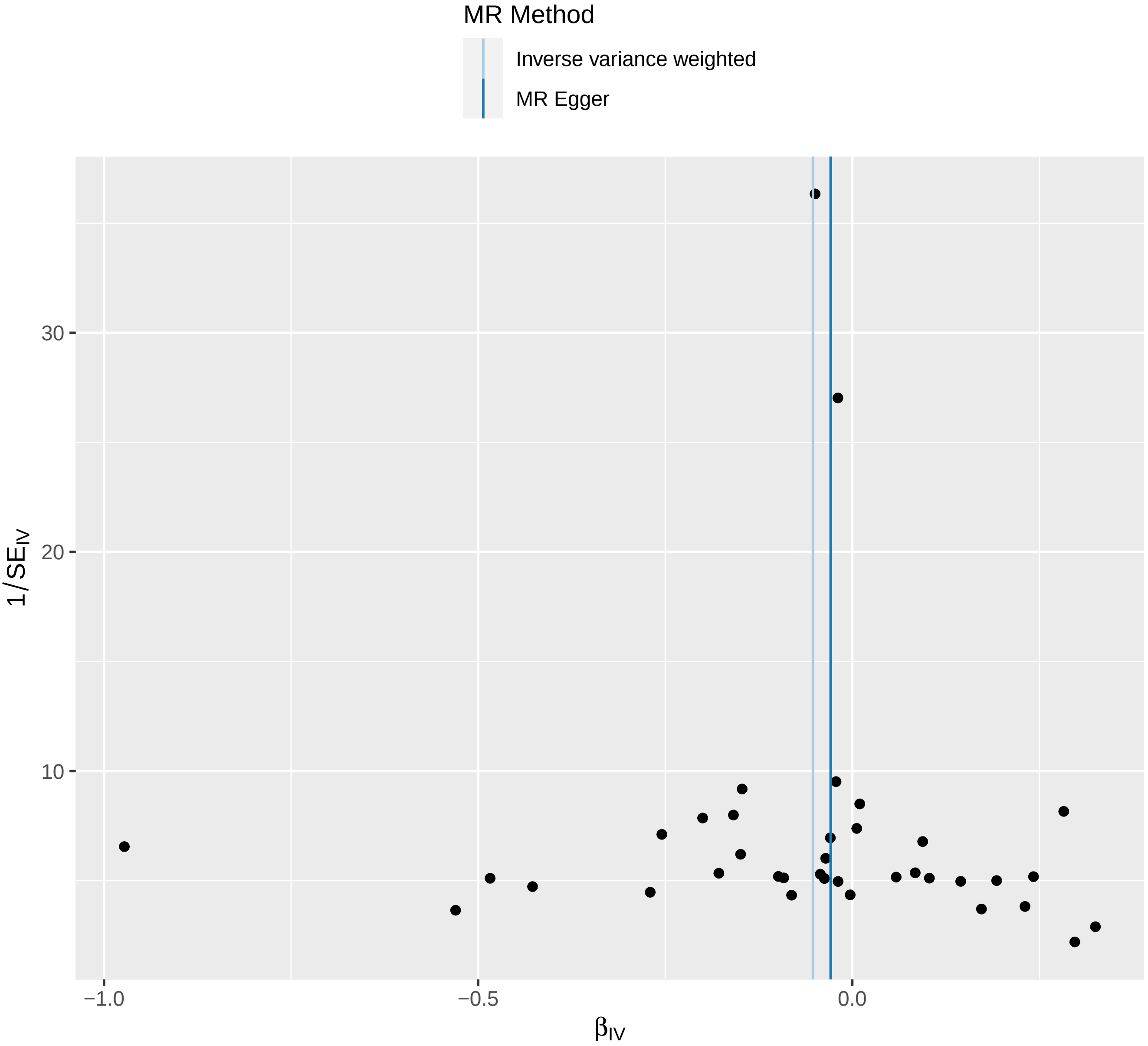

MR Method
Inverse variance weighted
MR Egger
30
20
10
1/SE_IV
−1.0
−0.5
0.0
β_IV

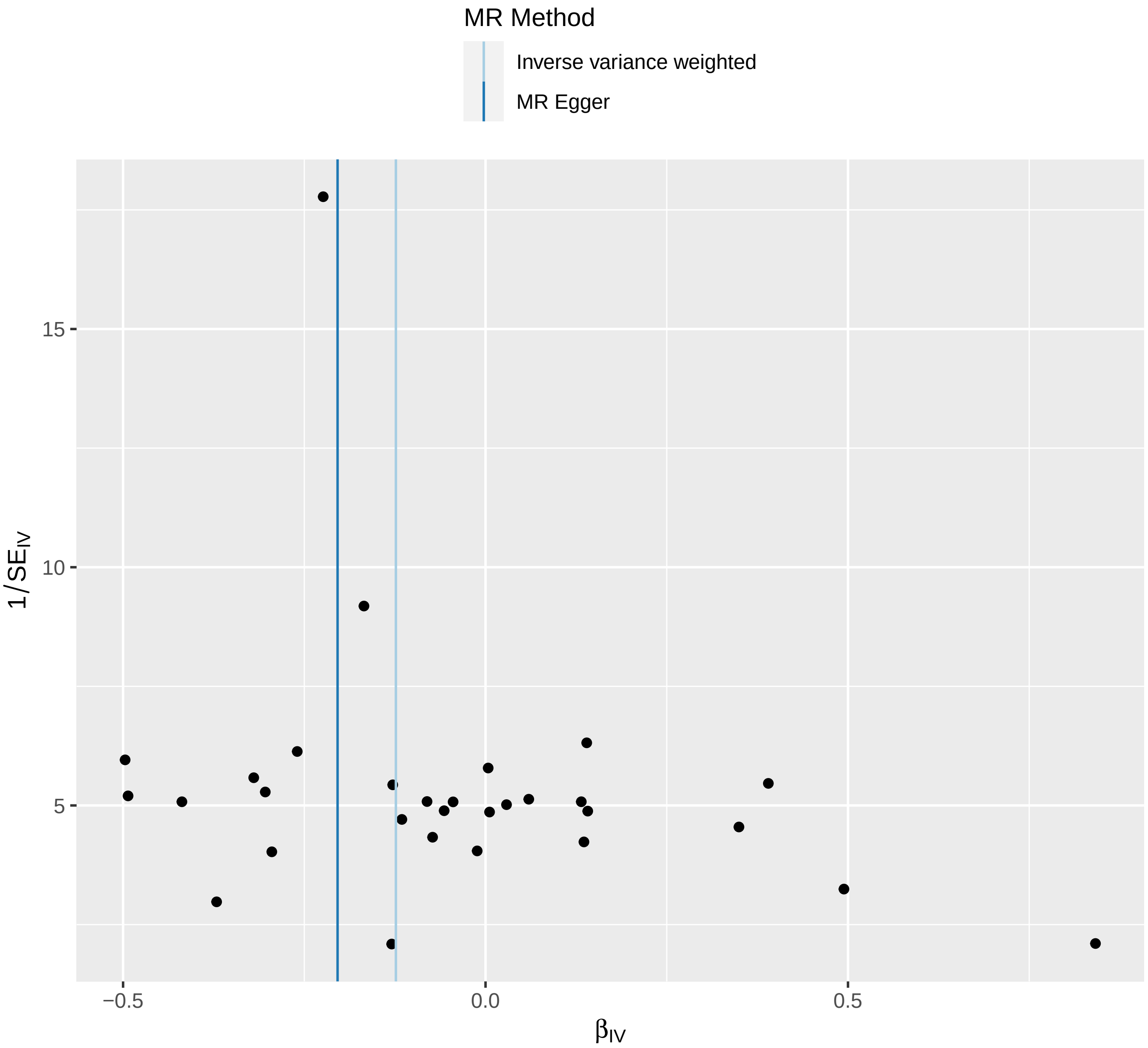

MR Method
Inverse variance weighted
MR Egger
15
10
5
−0.5
0.0
0.5
$1/SE_{IV}$
β_{IV}

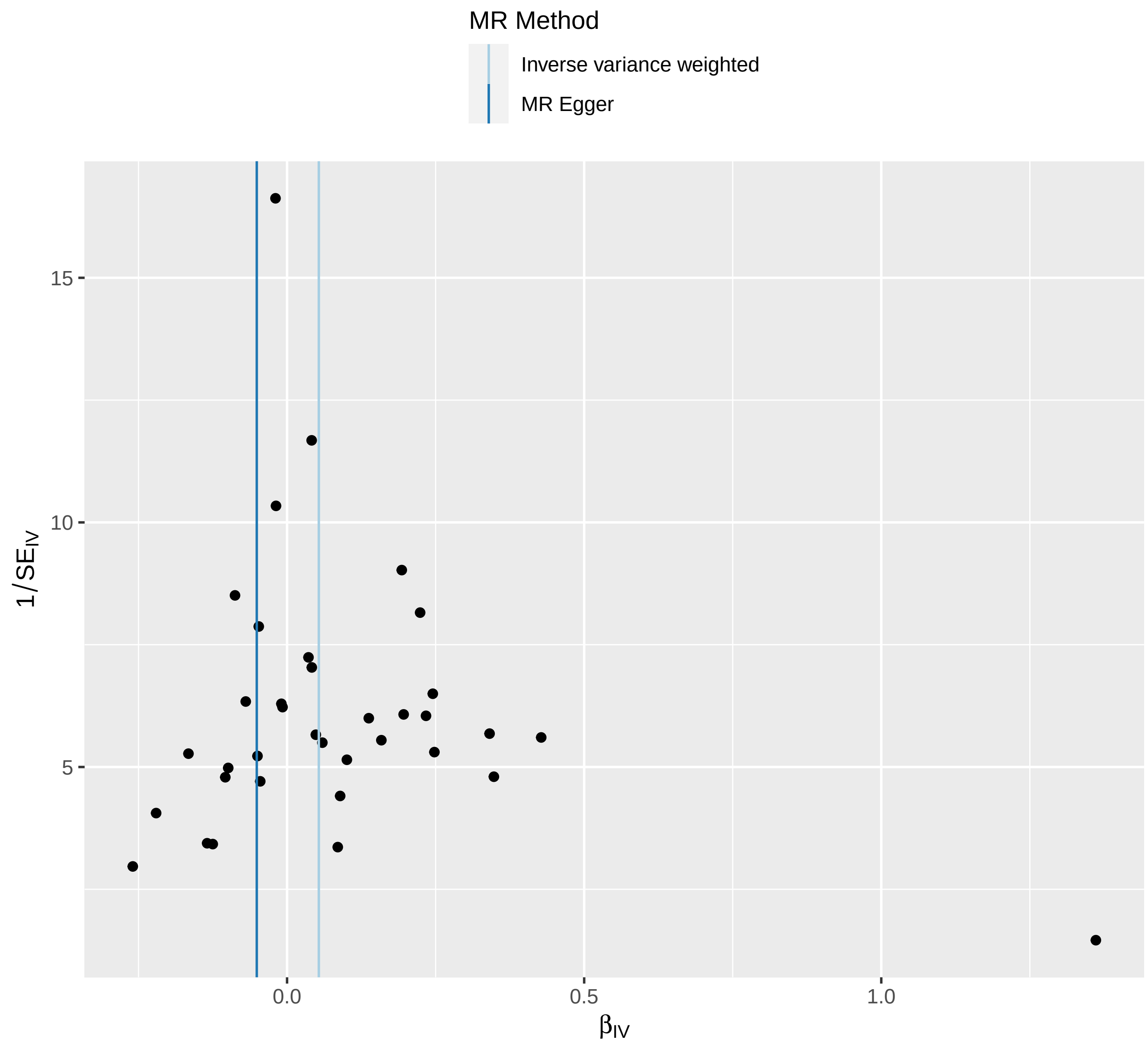
MR Method
Inverse variance weighted
MR Egger
15
10
5
1/SE_IV
0.0
0.5
1.0
β_IV

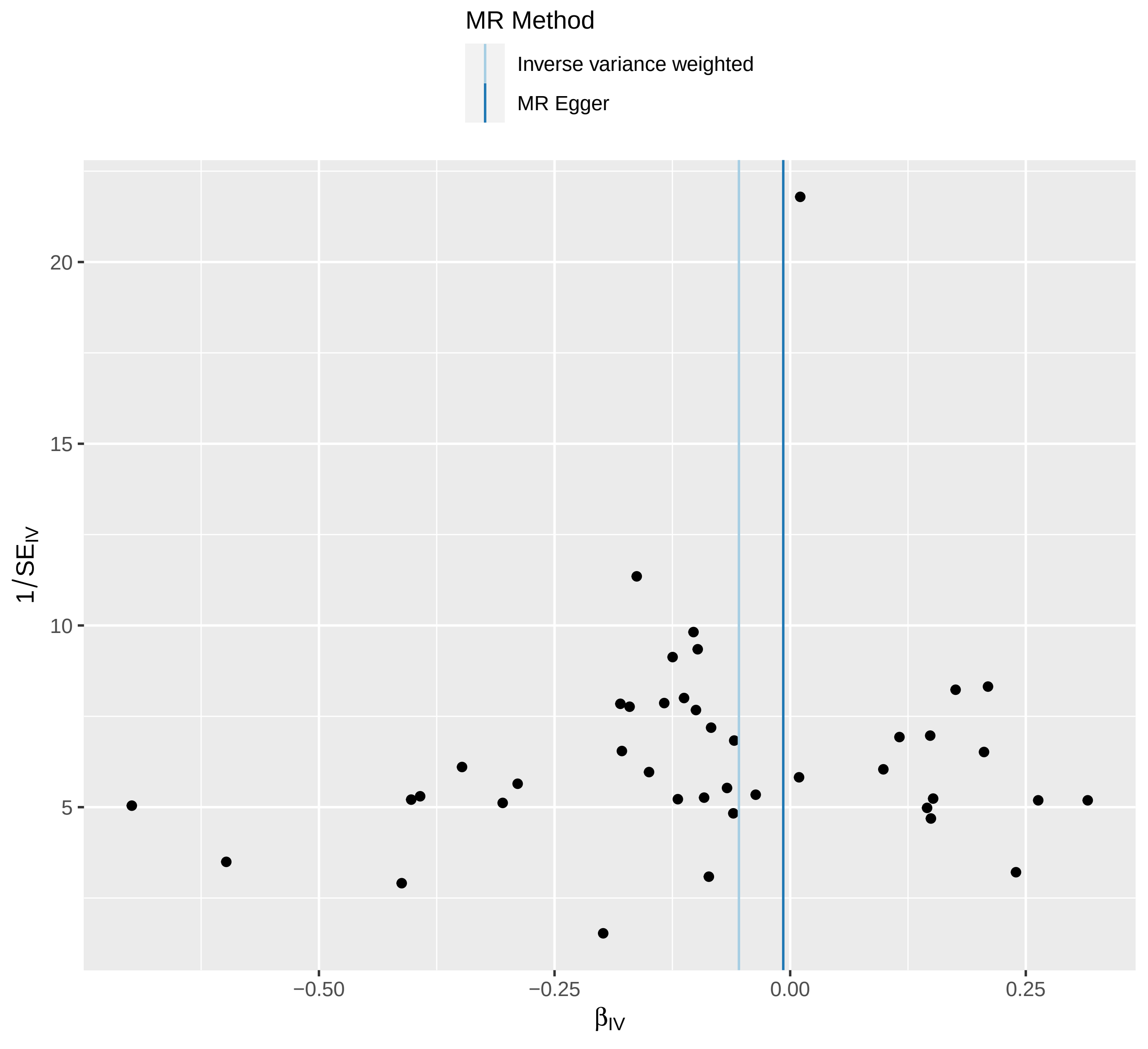

MR Method
Inverse variance weighted
MR Egger
20
15
10
5
$1/SE_{IV}$
−0.50
−0.25
0.00
0.25
β_{IV}

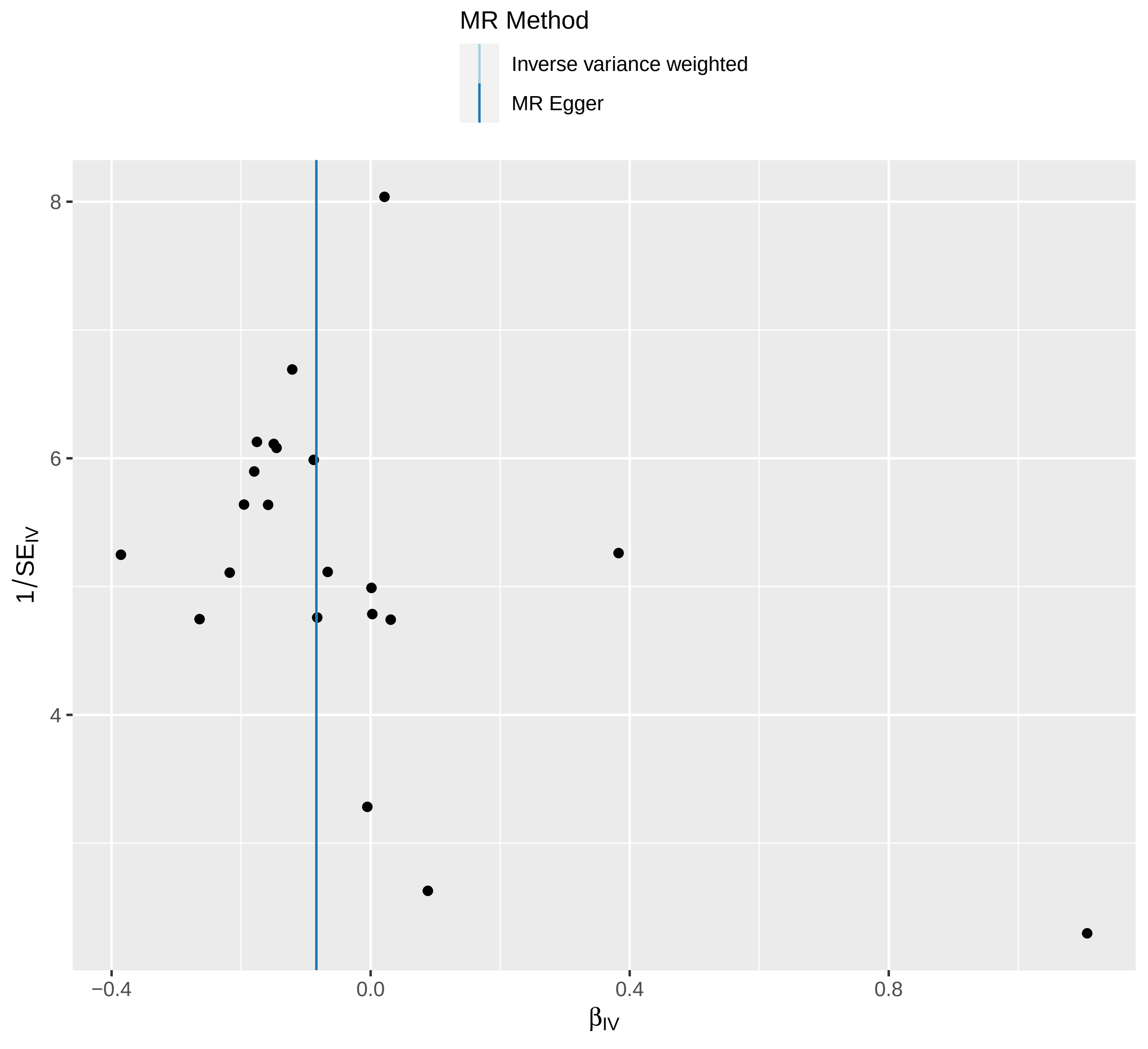
MR Method
Inverse variance weighted
MR Egger
8
6
4
1/SE$_{IV}$
−0.4
0.0
0.4
0.8
β$_{IV}$

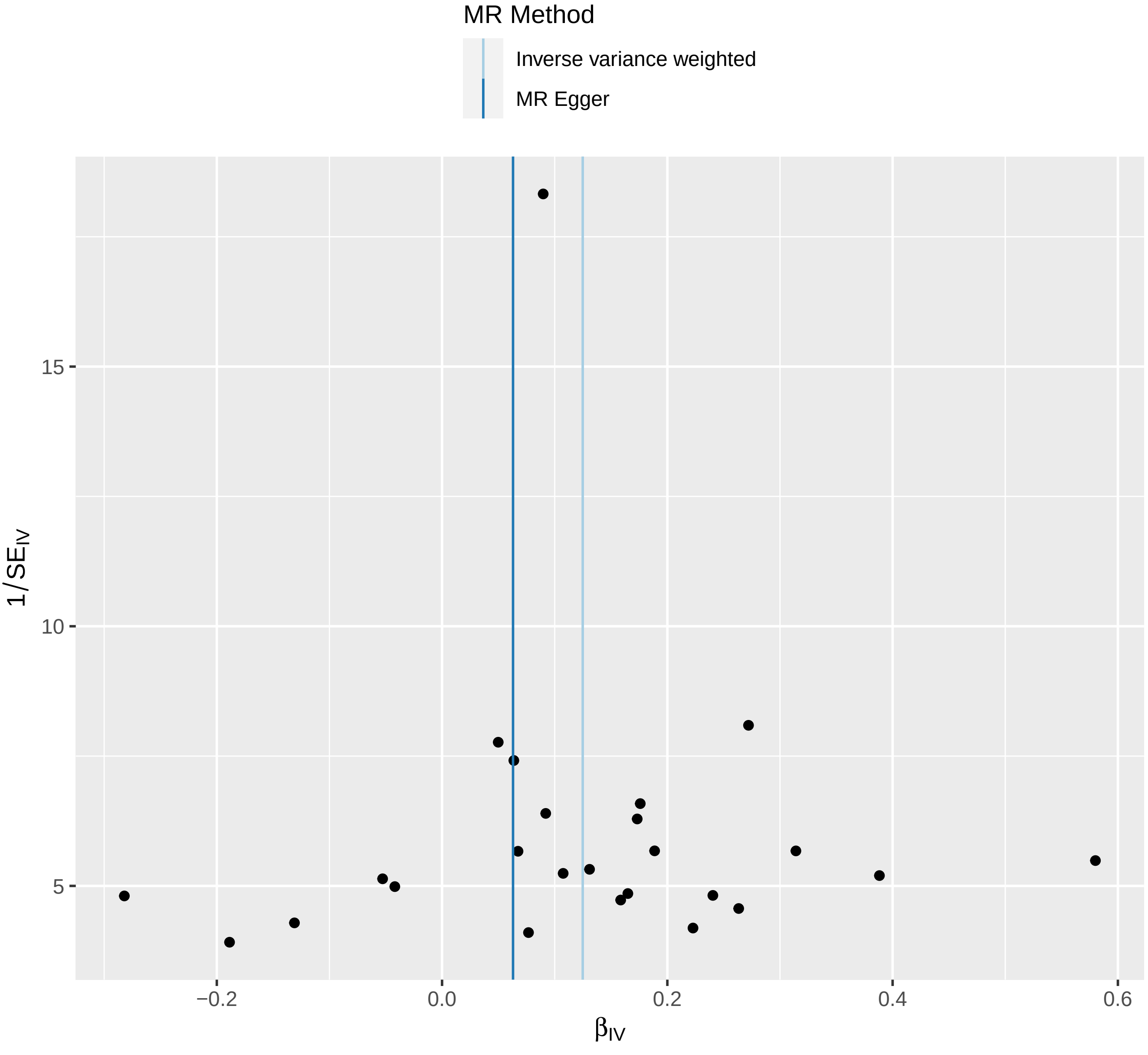
MR Method
Inverse variance weighted
MR Egger
15
10
5
1/SE_IV
−0.2
0.0
0.2
0.4
0.6
β_IV

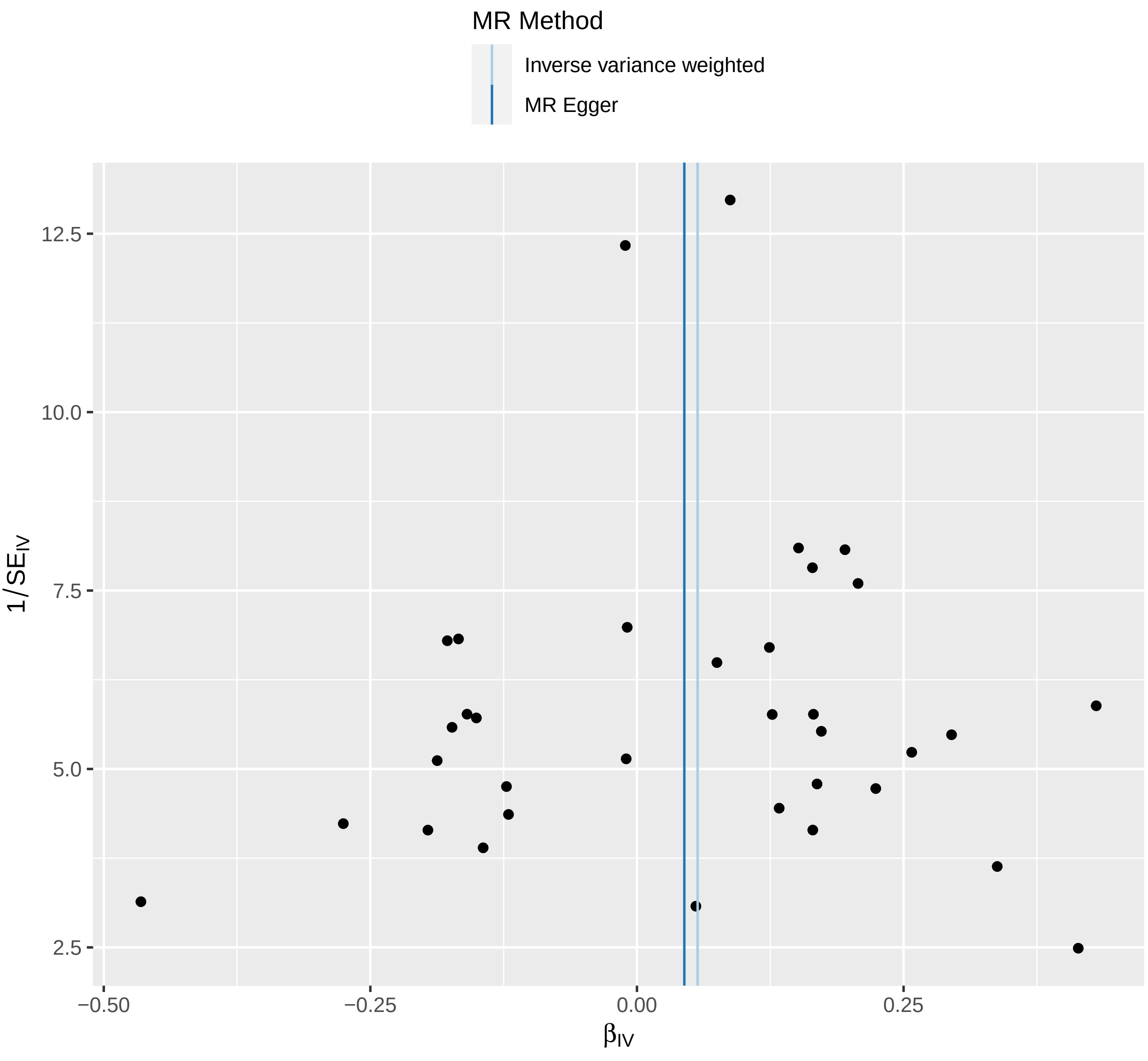

MR Method
Inverse variance weighted
MR Egger
12.5
10.0
7.5
5.0
2.5
1/SE_IV
−0.50
−0.25
0.00
0.25
β_IV

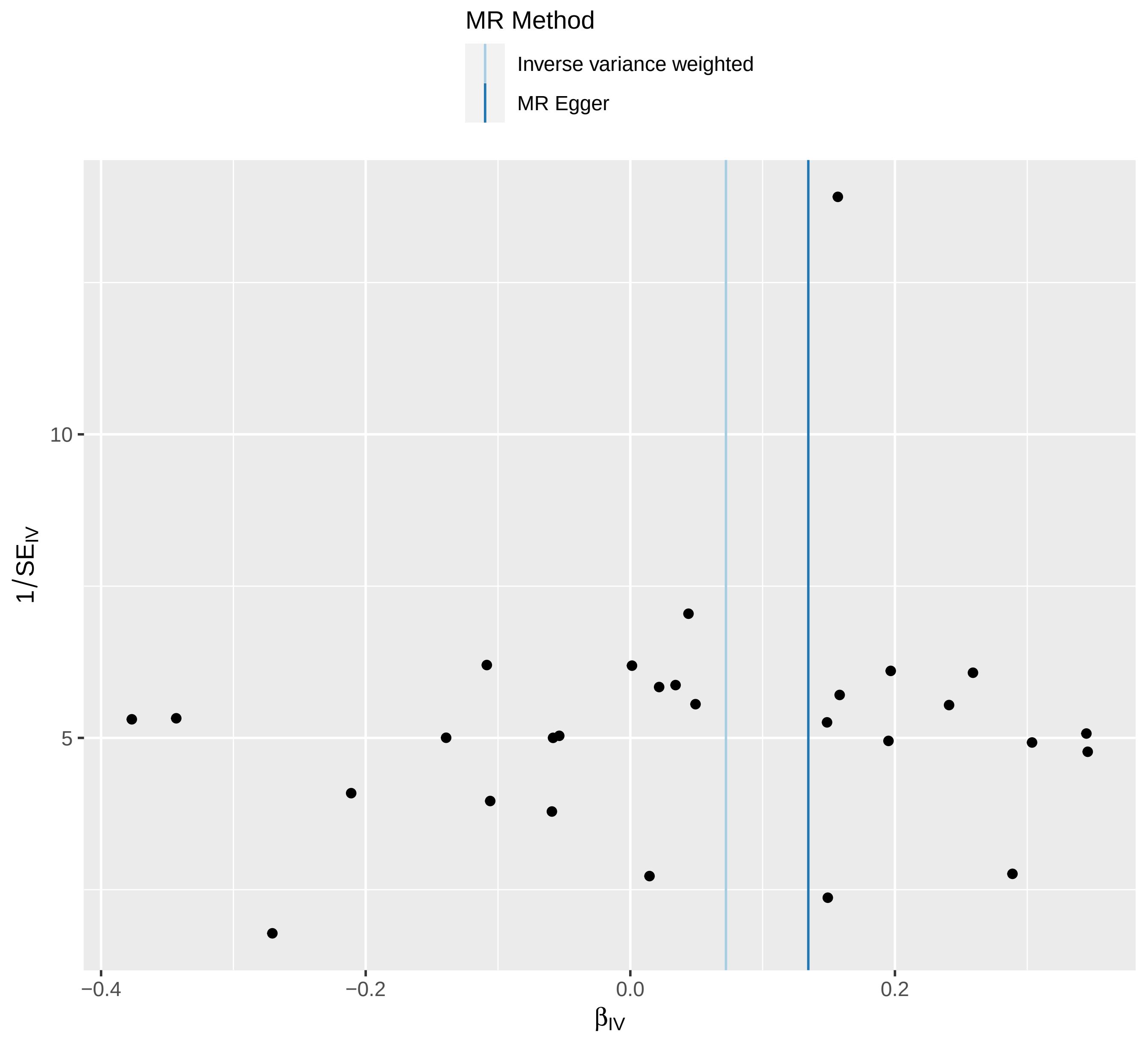

MR Method
Inverse variance weighted
MR Egger
10
5
1/SE_IV
−0.4
−0.2
0.0
0.2
β_IV

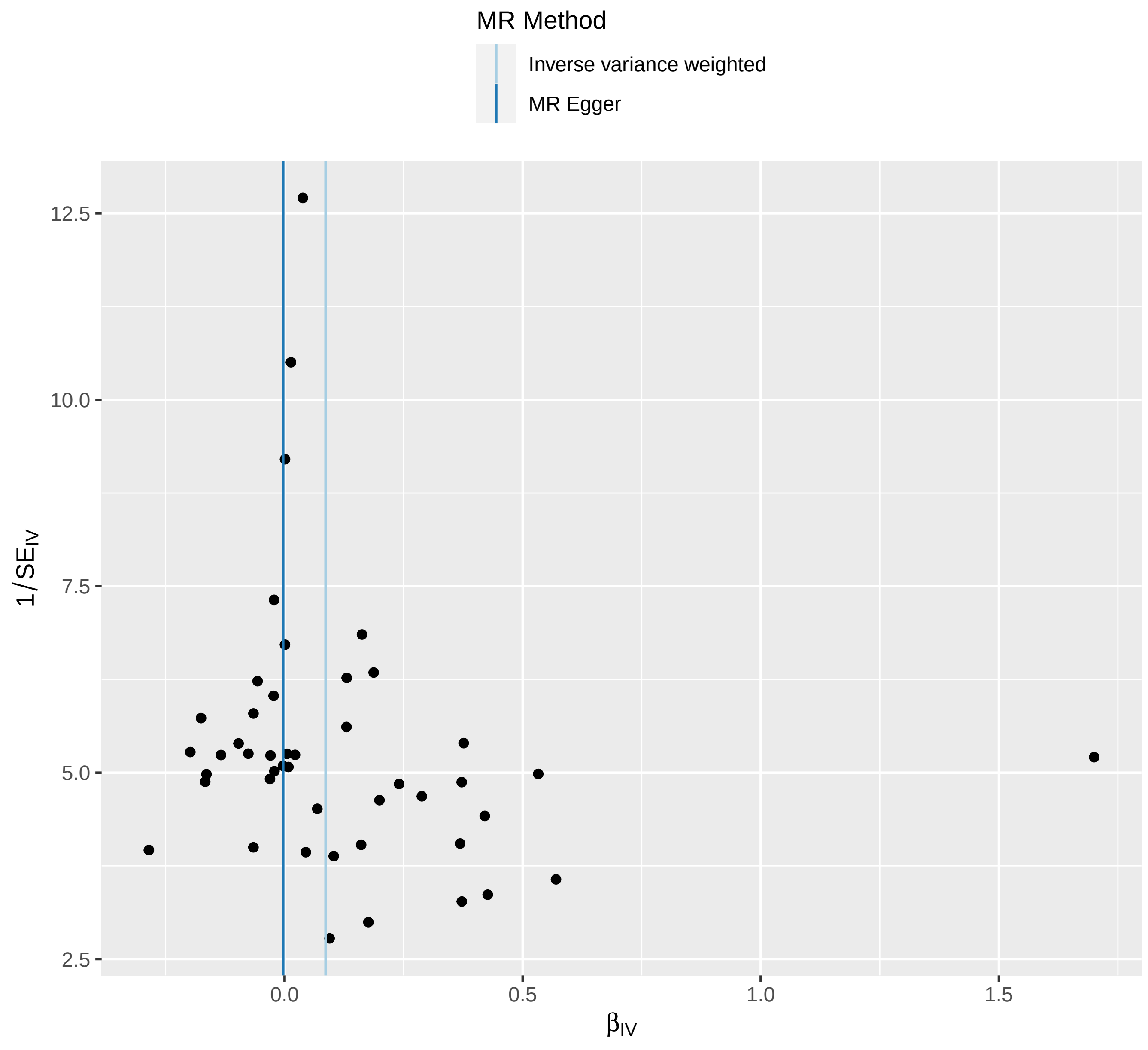
MR Method
Inverse variance weighted
MR Egger
12.5
10.0
7.5
5.0
2.5
1/SE_IV
0.0
0.5
1.0
1.5
β_IV

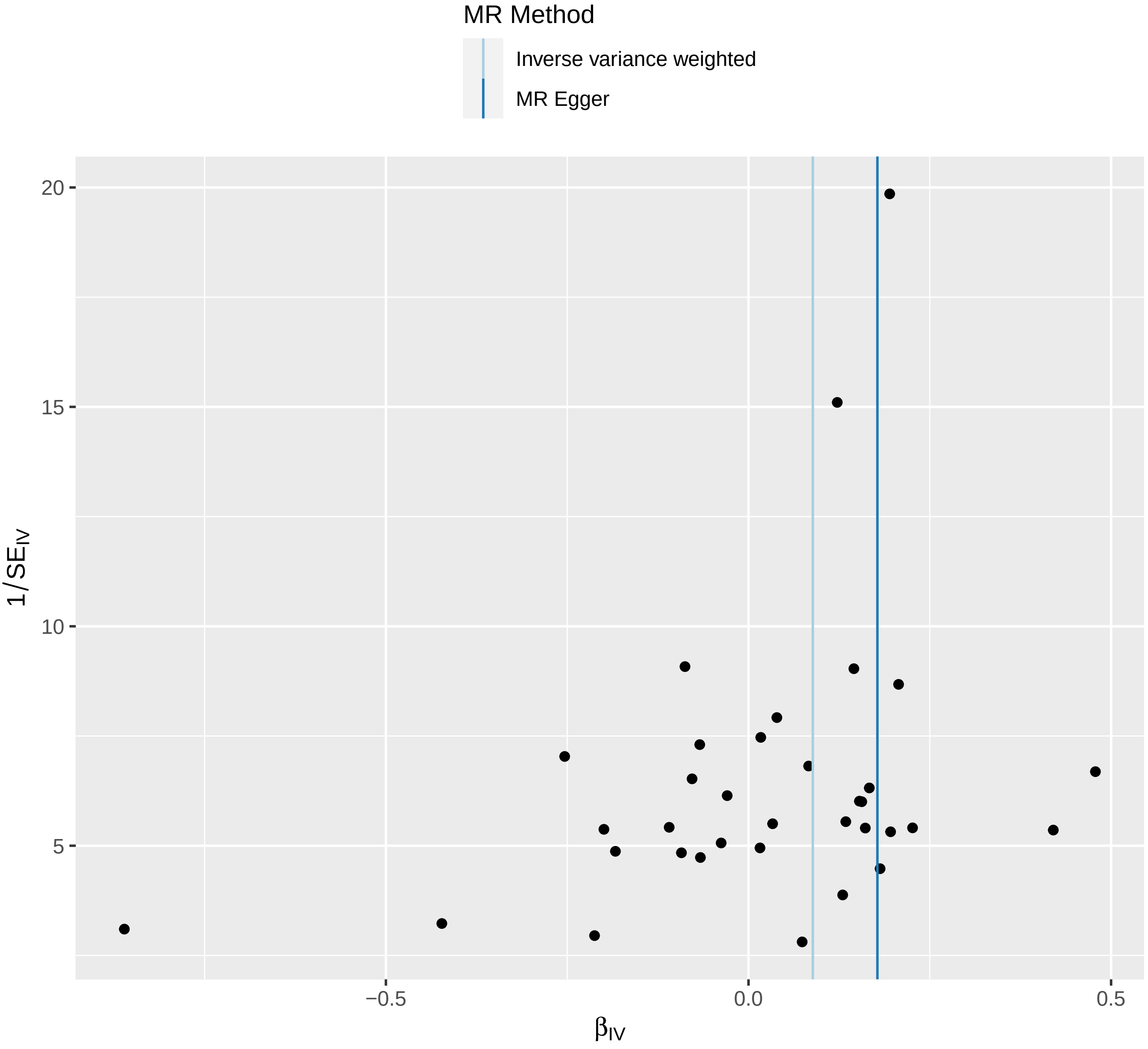

MR Method
Inverse variance weighted
MR Egger
20
15
10
5
1/SE$_{IV}$
−0.5
0.0
0.5
β$_{IV}$

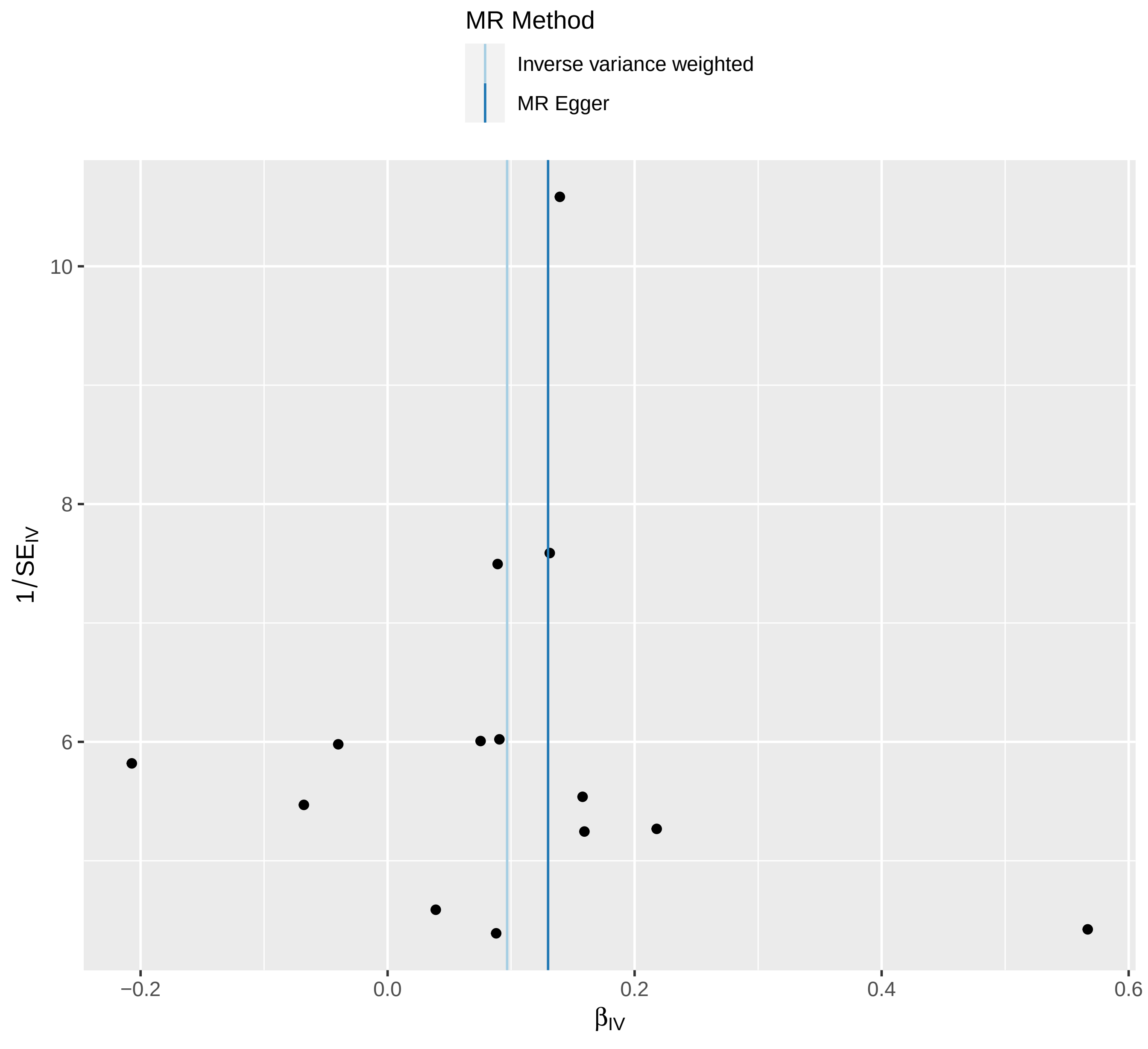

MR Method
Inverse variance weighted
MR Egger
10
8
6
1/SE_IV
−0.2
0.0
0.2
0.4
0.6
β_IV

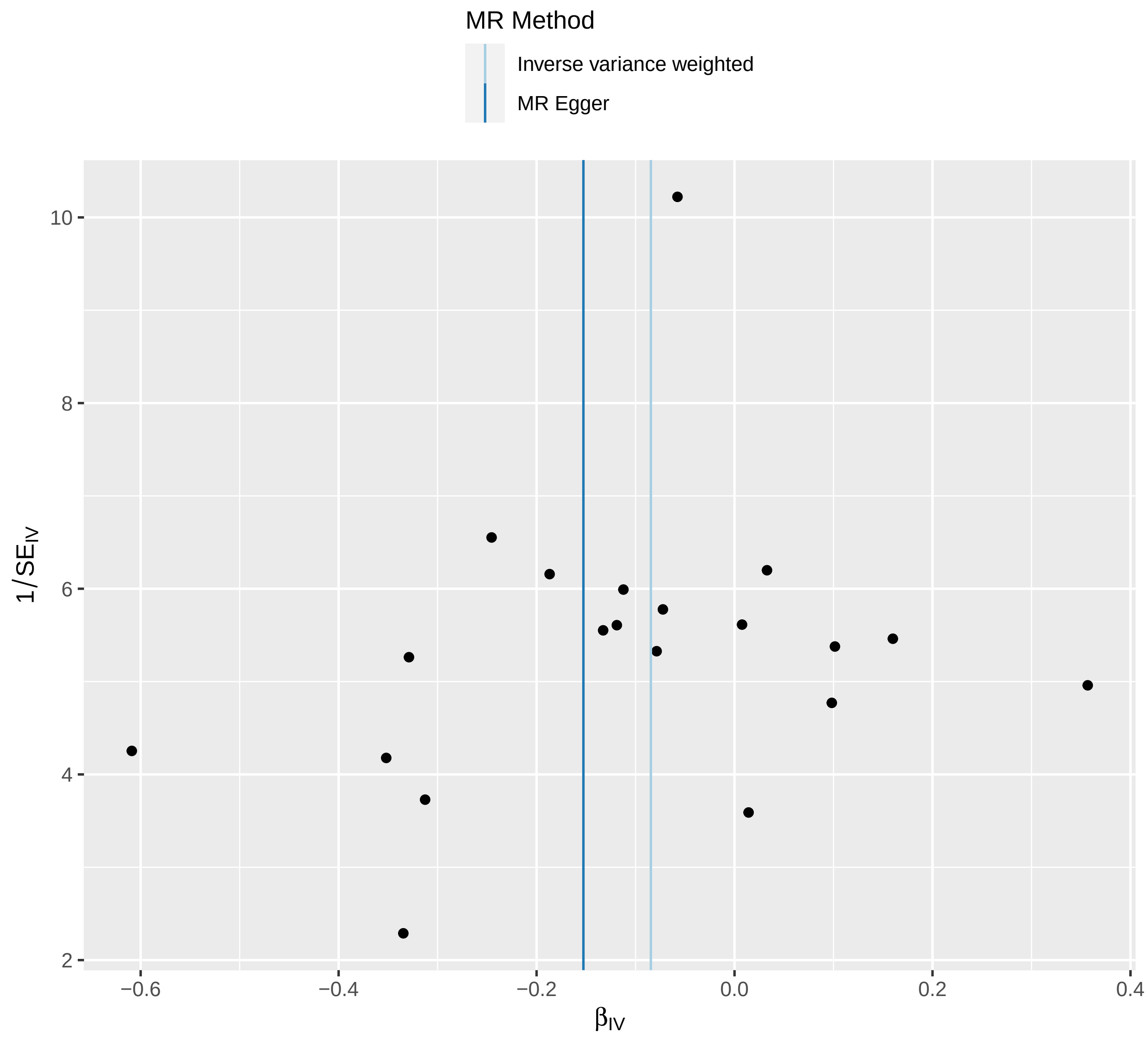

MR Method
Inverse variance weighted
MR Egger
10
8
6
4
2
−0.6
−0.4
−0.2
0.0
0.2
0.4
$1/SE_{IV}$
β_{IV}

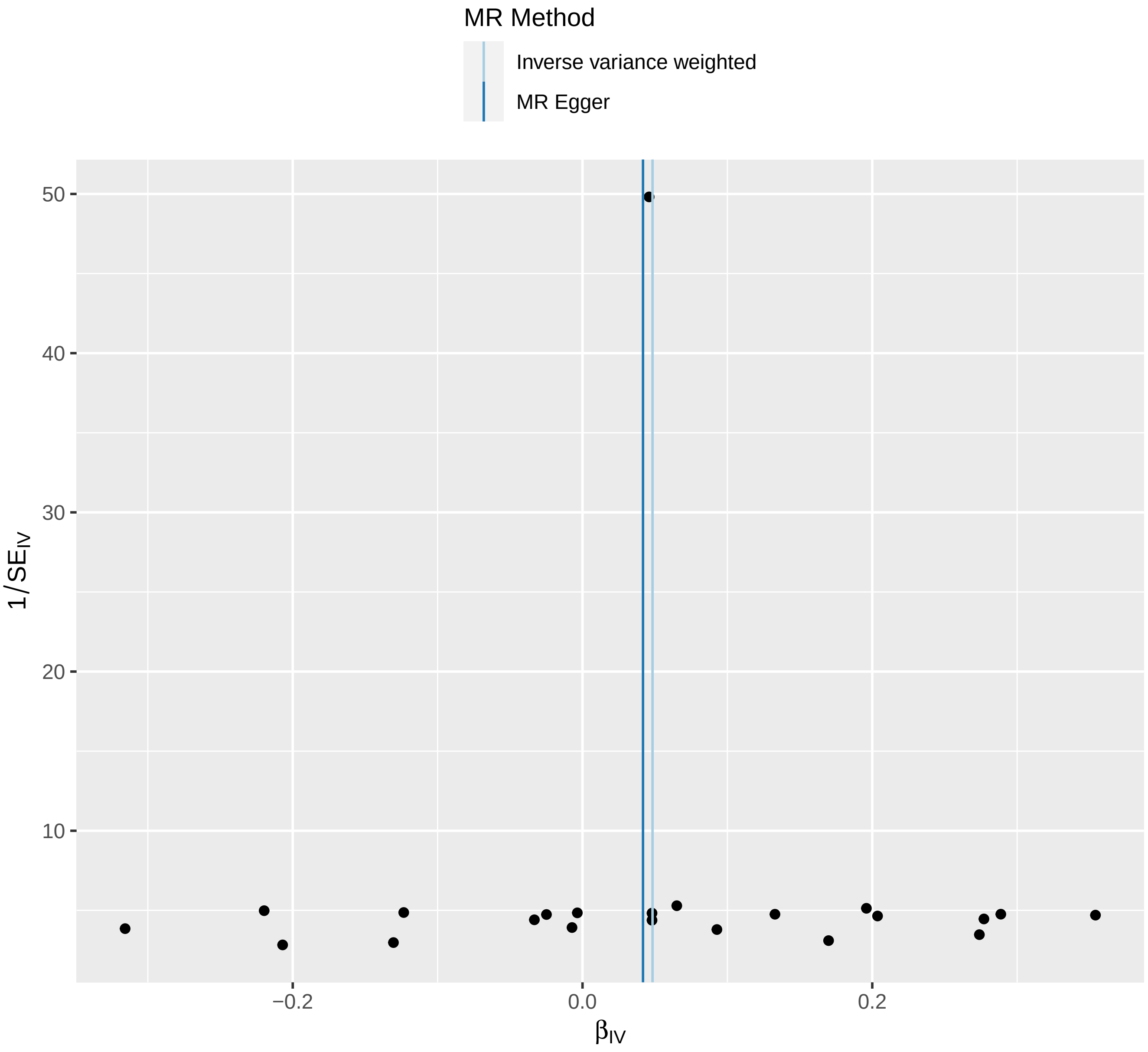
MR Method
Inverse variance weighted
MR Egger
50
40
30
20
10
1/SE$_{IV}$
−0.2
0.0
0.2
β$_{IV}$

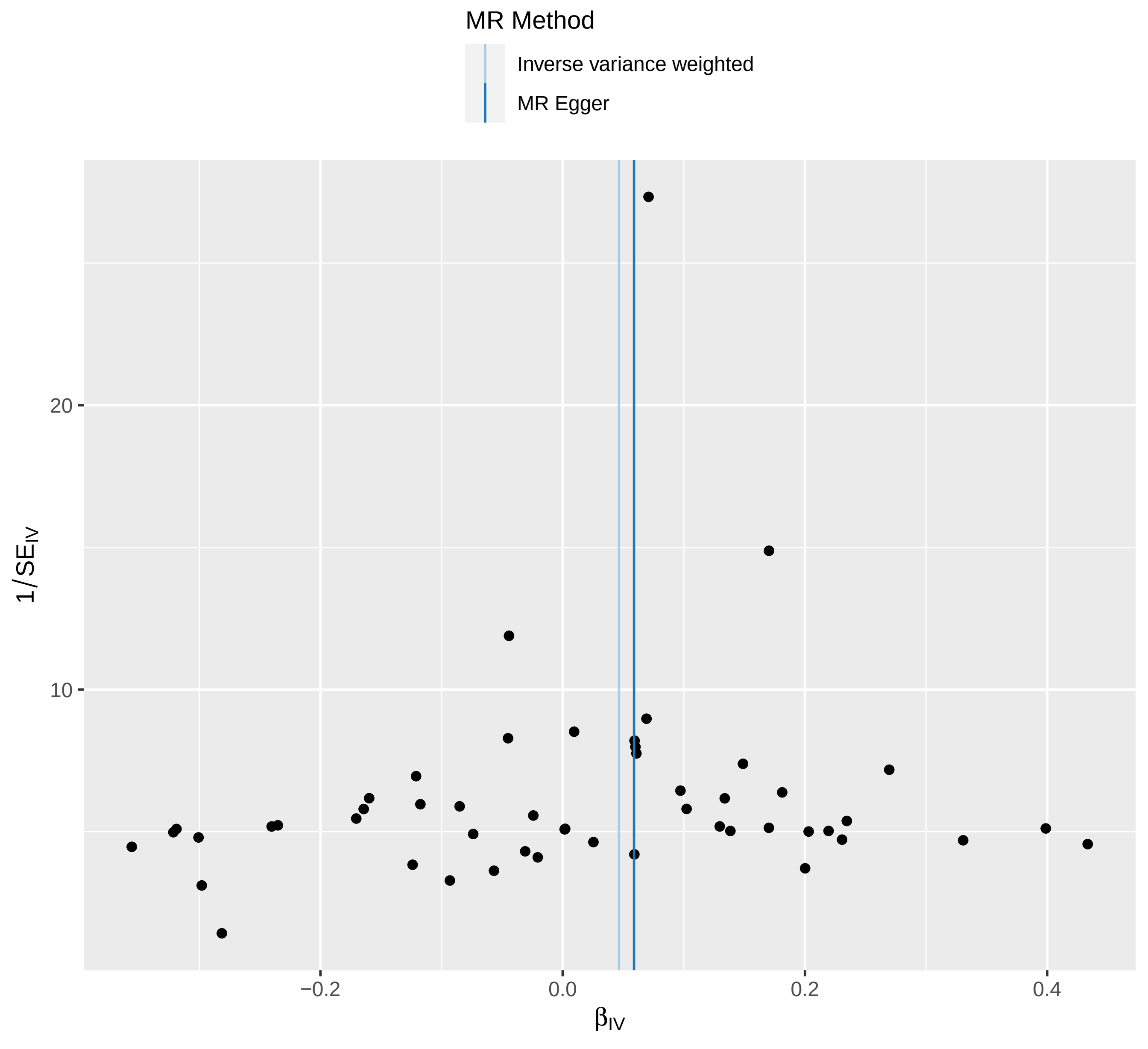
MR Method
Inverse variance weighted
MR Egger
20
10
−0.2
0.0
0.2
0.4
$1/SE_{IV}$
β_{IV}

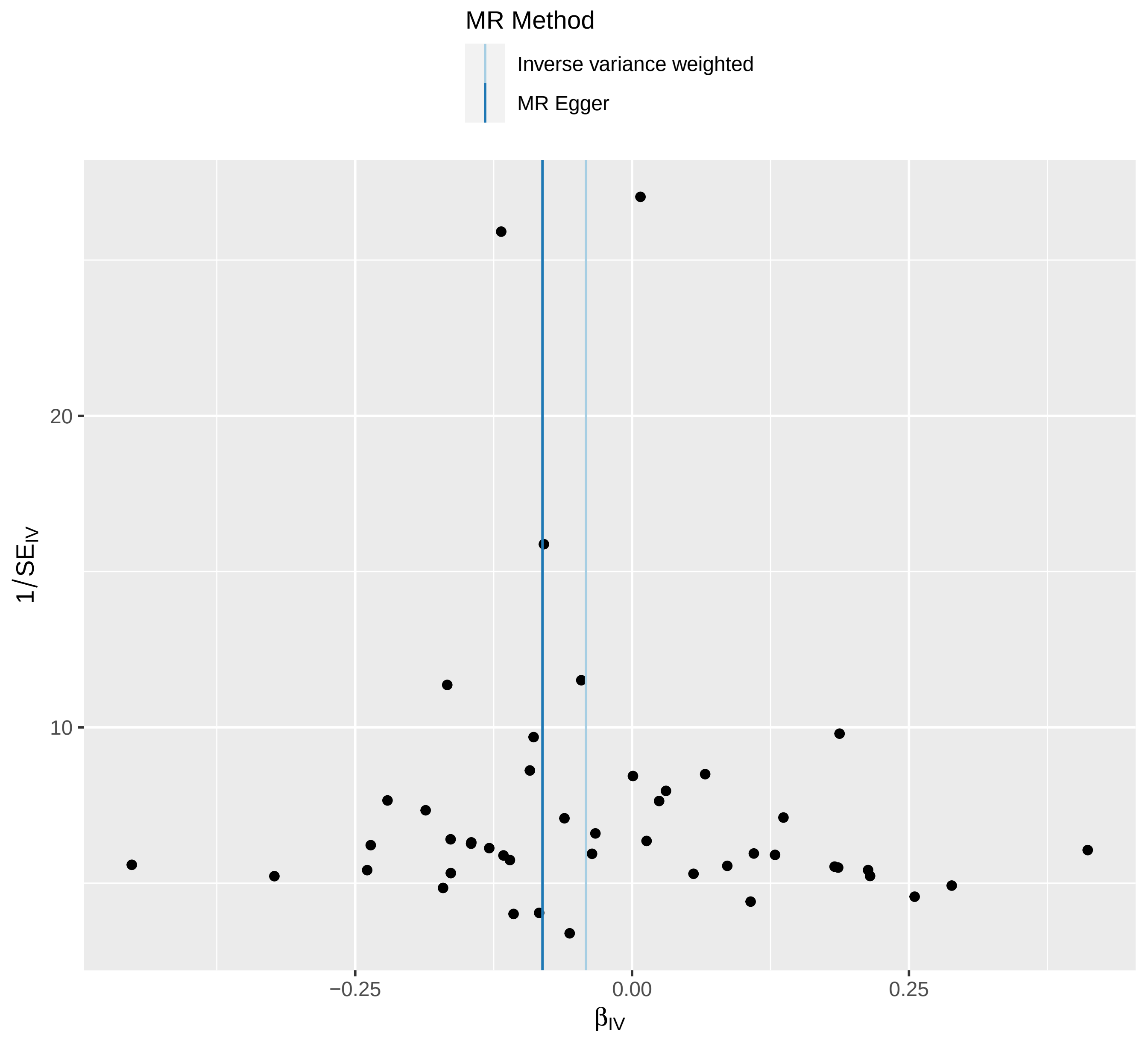
MR Method
Inverse variance weighted
MR Egger
20
10
1/SE_IV
−0.25
0.00
0.25
β_IV

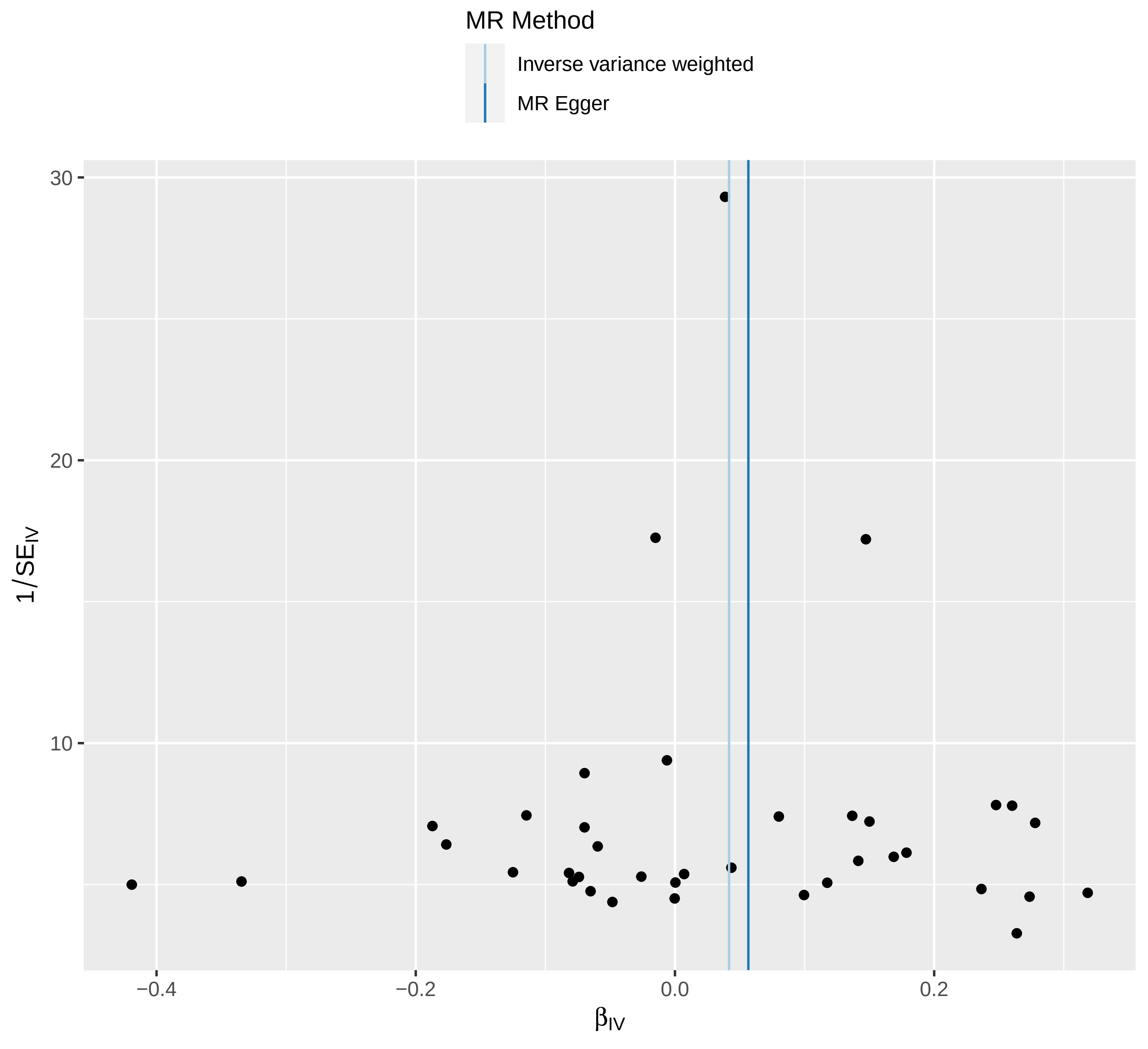
MR Method
Inverse variance weighted
MR Egger
30
20
10
1/SE_IV
−0.4
−0.2
0.0
0.2
β_IV

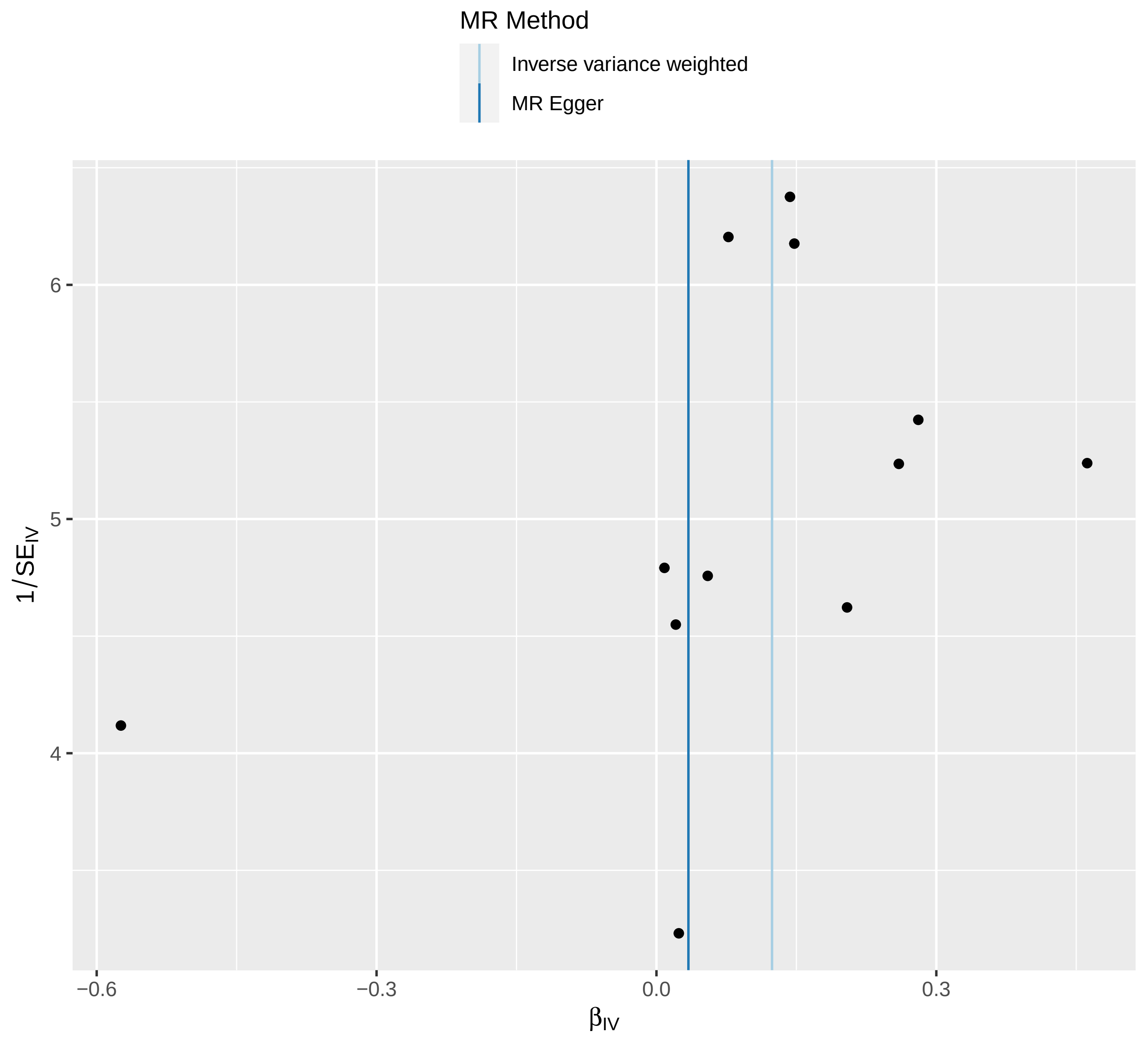

MR Method
Inverse variance weighted
MR Egger
6
5
4
$1/SE_{IV}$
−0.6
−0.3
0.0
0.3
β_{IV}

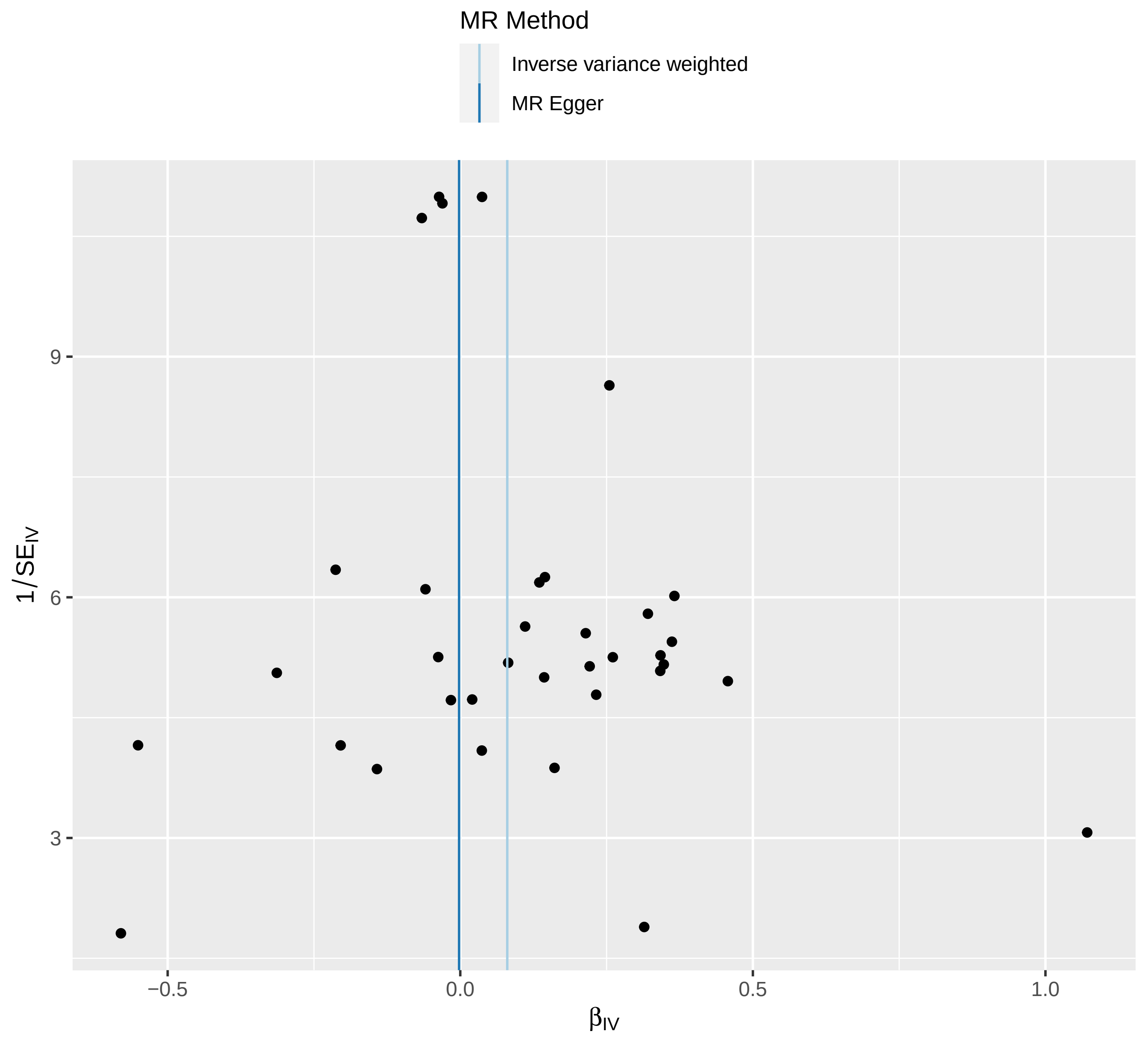
MR Method
Inverse variance weighted
MR Egger
1/SE_IV
9
6
3
β_IV
−0.5
0.0
0.5
1.0

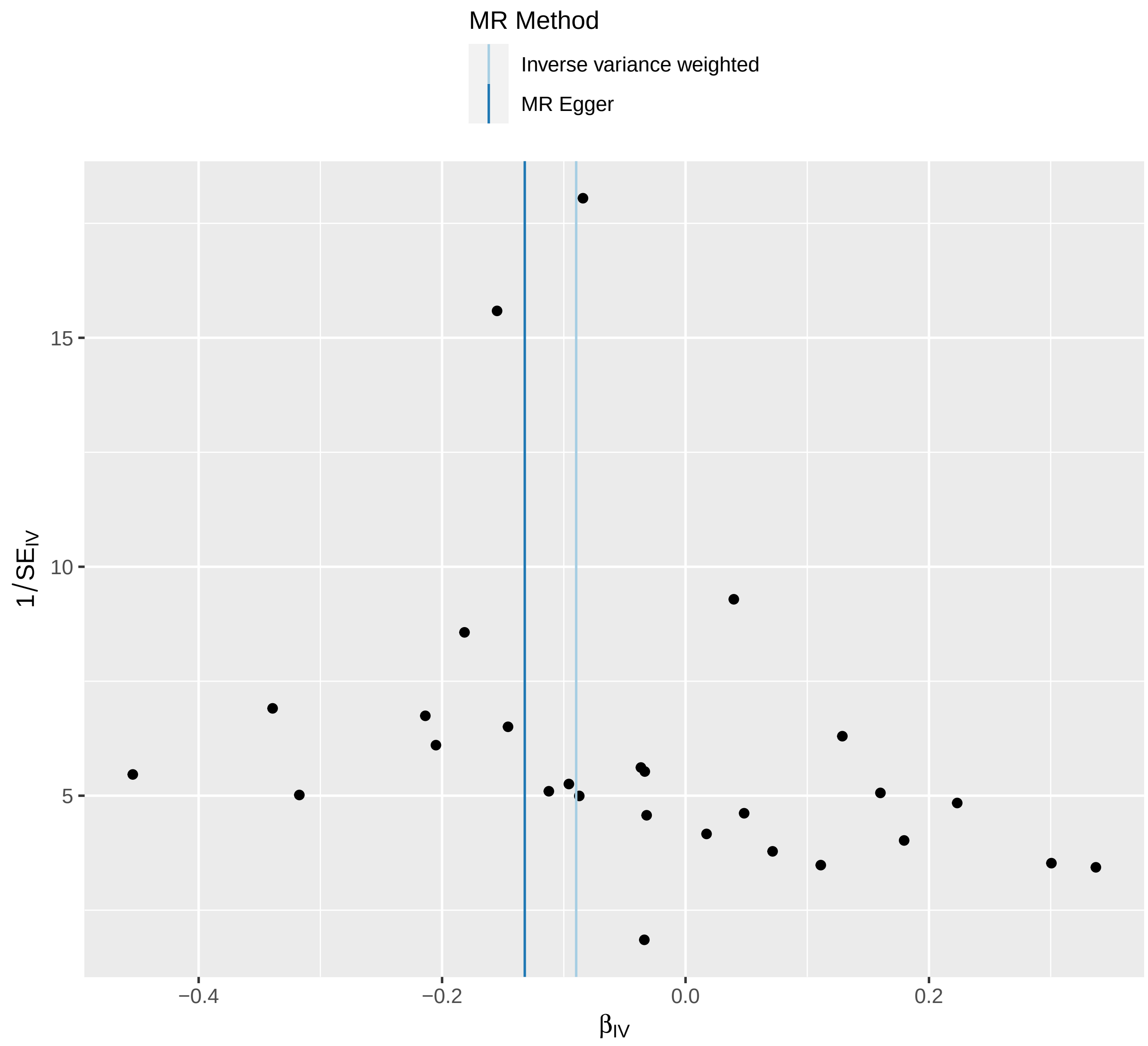
MR Method
Inverse variance weighted
MR Egger
15
10
5
$1/SE_{IV}$
−0.4
−0.2
0.0
0.2
β_{IV}

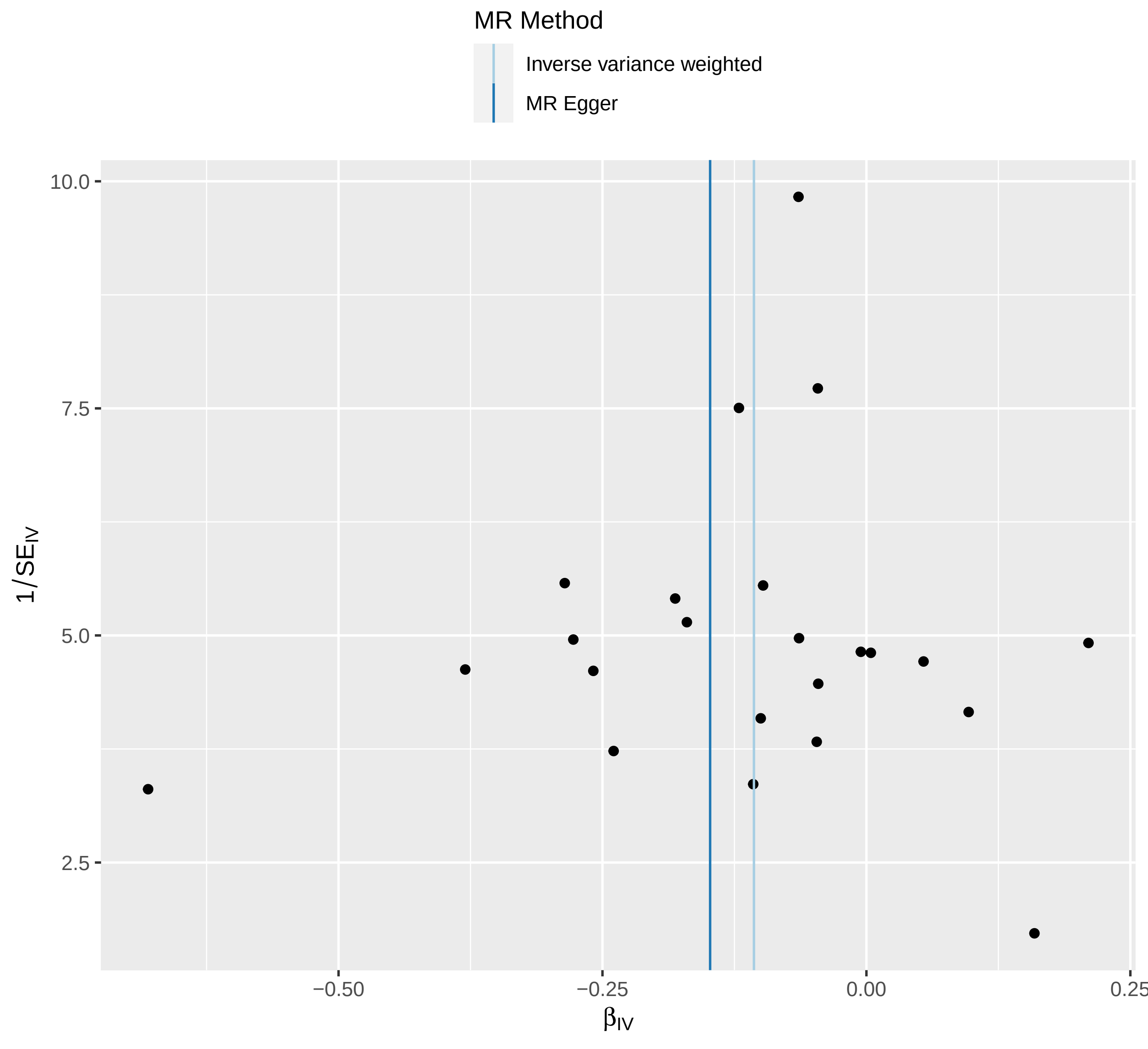
MR Method
Inverse variance weighted
MR Egger
10.0
7.5
5.0
2.5
$1/SE_{IV}$
−0.50
−0.25
0.00
0.25
β_{IV}

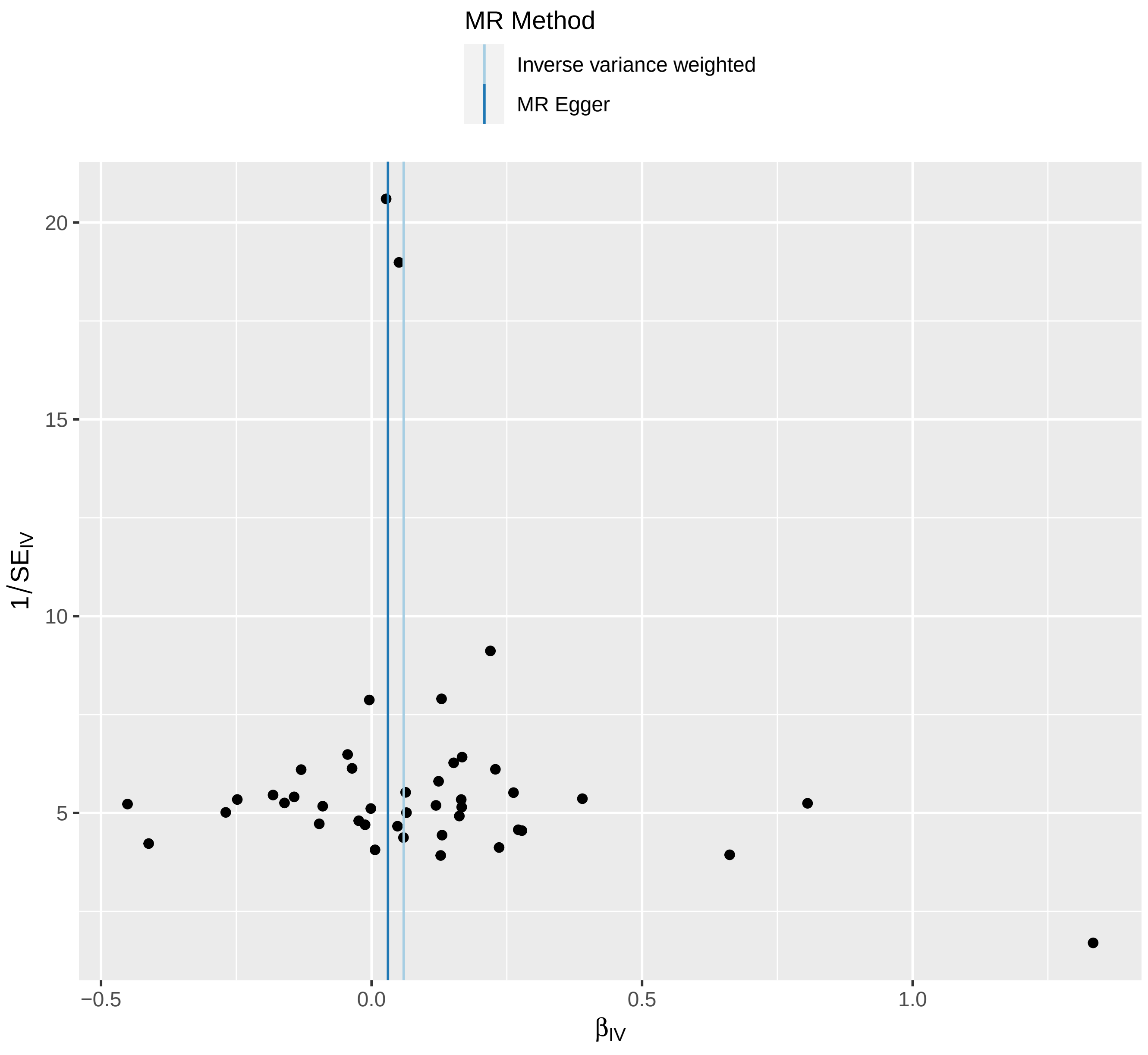
MR Method
Inverse variance weighted
MR Egger
20
15
10
5
$1/SE_{IV}$
−0.5
0.0
0.5
1.0
β_{IV}

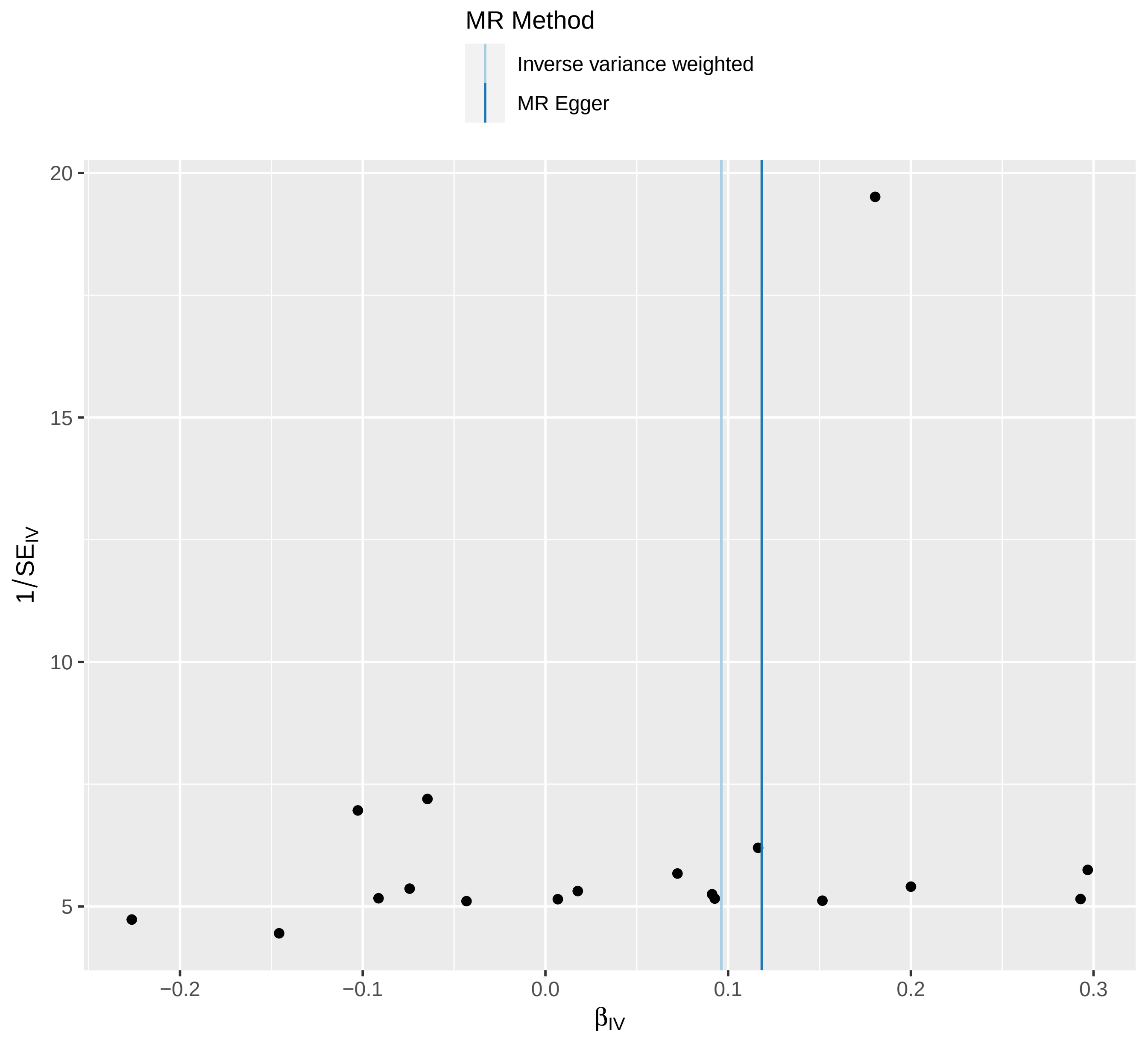

MR Method
Inverse variance weighted
MR Egger
20
15
10
5
$1/SE_{IV}$
−0.2
−0.1
0.0
0.1
0.2
0.3
β_{IV}

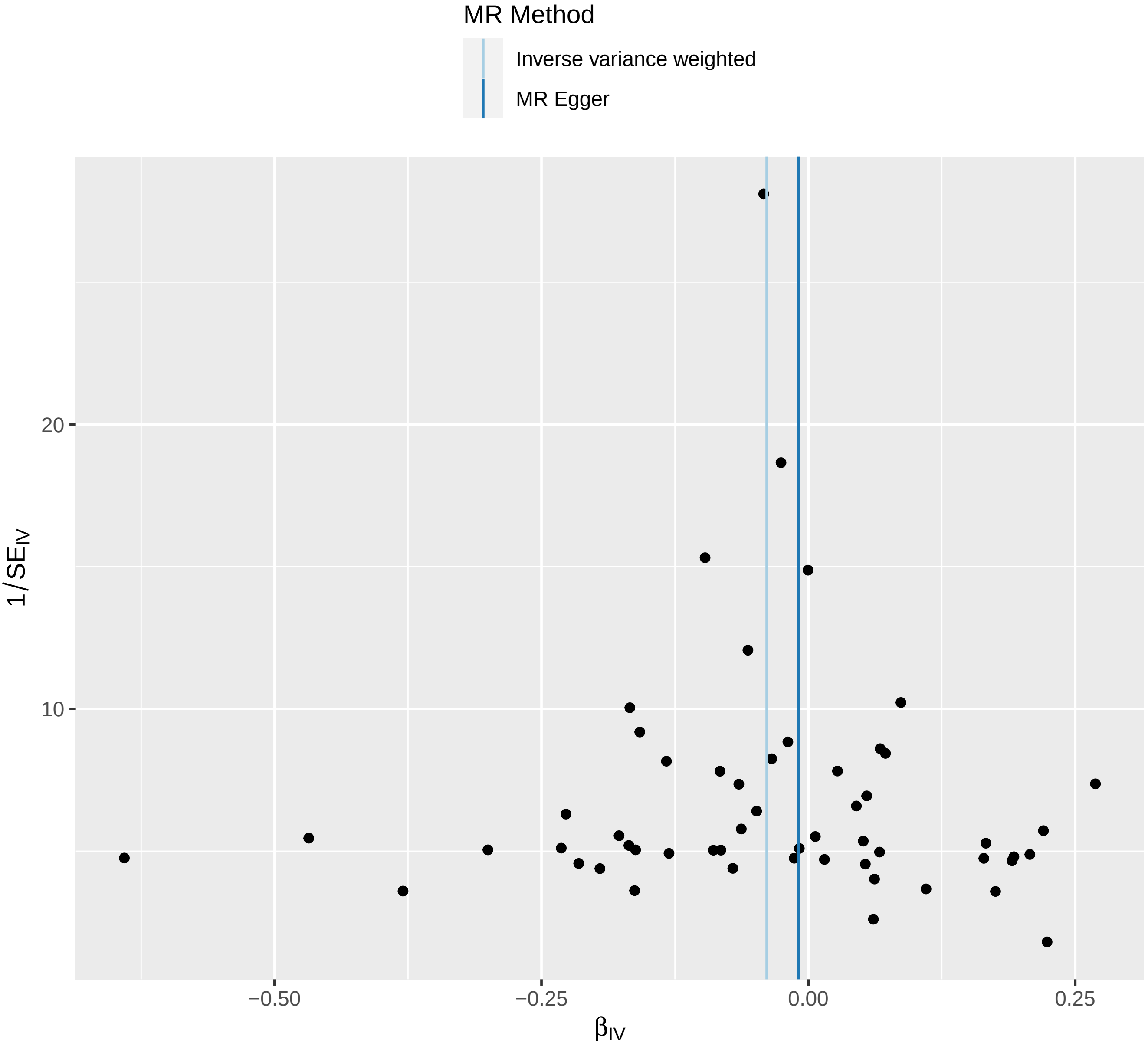

MR Method
Inverse variance weighted
MR Egger
20
10
1/SE$_{IV}$
−0.50
−0.25
0.00
0.25
β$_{IV}$

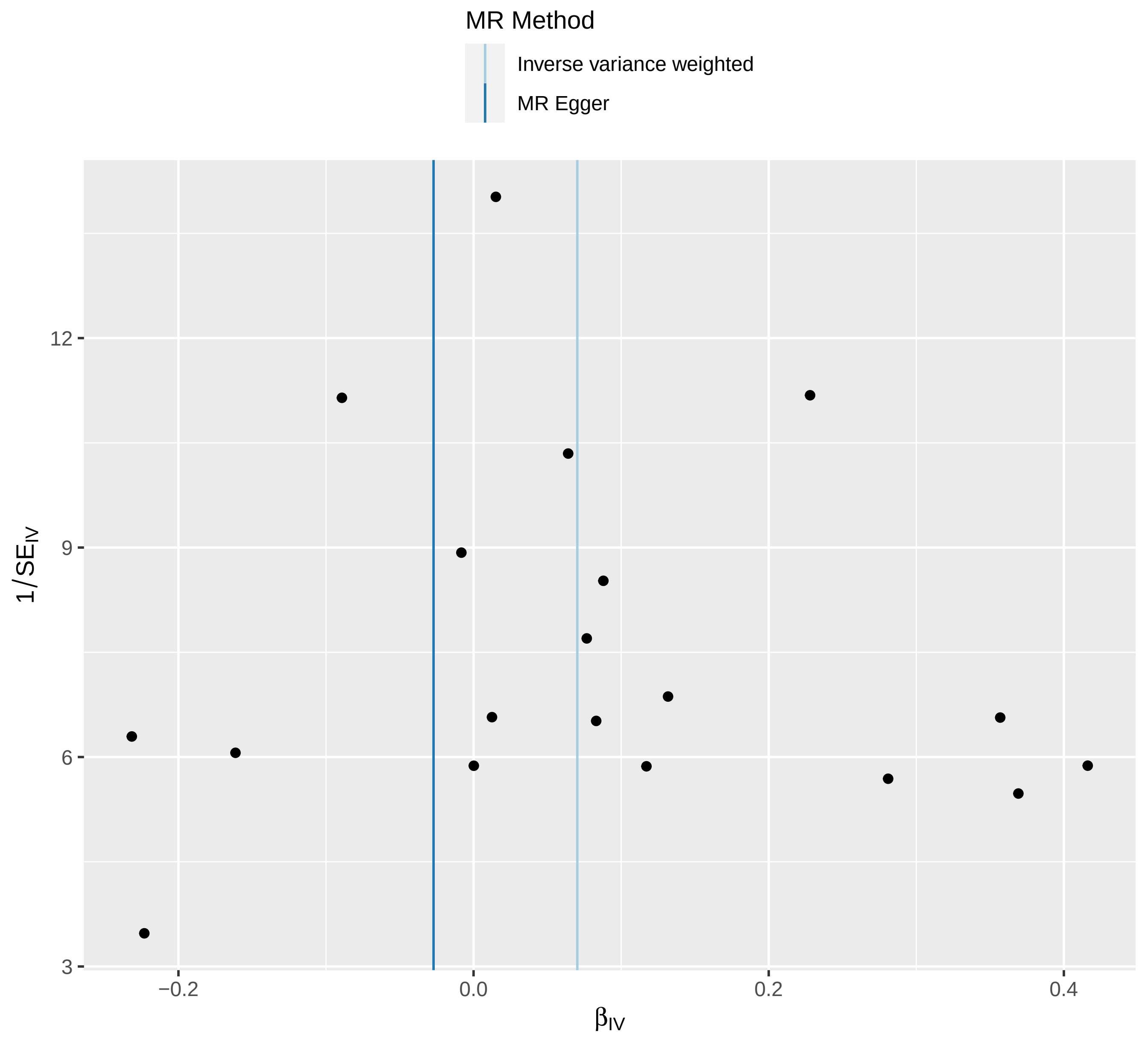

MR Method
Inverse variance weighted
MR Egger
12
9
6
3
1/SE$_{IV}$
−0.2
0.0
0.2
0.4
β$_{IV}$

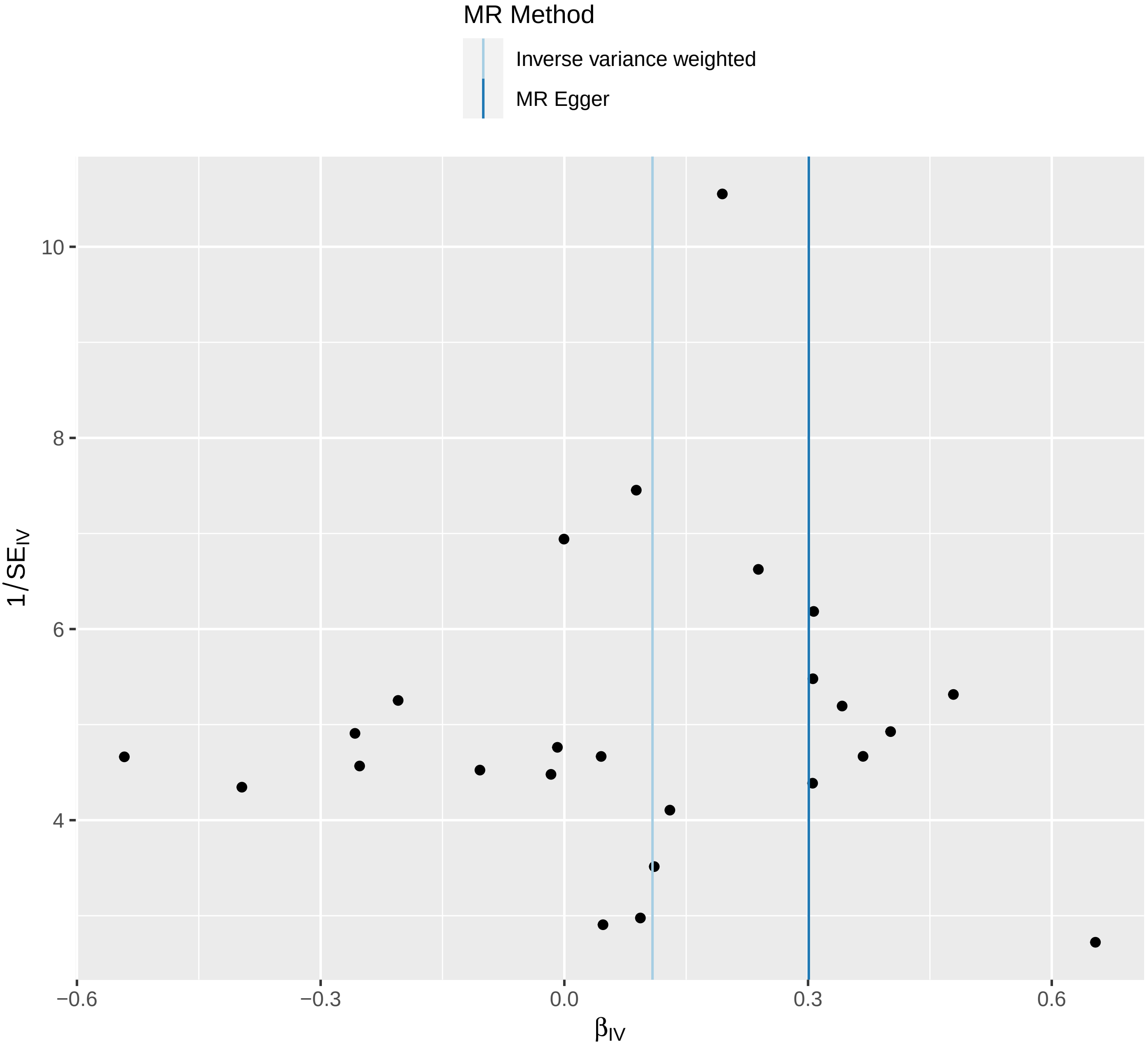
MR Method
Inverse variance weighted
MR Egger
10
8
6
4
1/SE$_{IV}$
−0.6
−0.3
0.0
0.3
0.6
β$_{IV}$

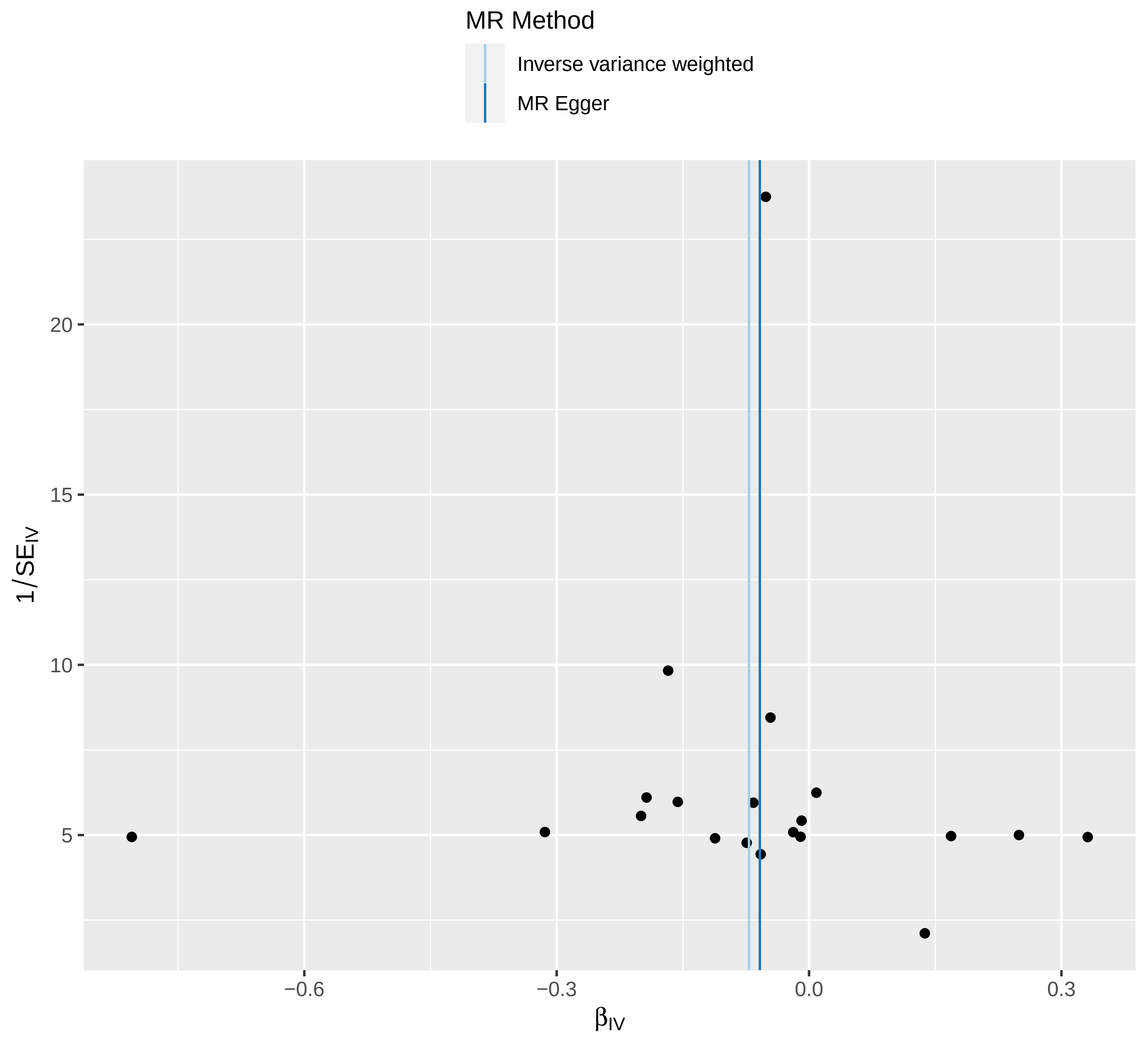

MR Method
Inverse variance weighted
MR Egger
20
15
10
5
1/SE_IV
−0.6
−0.3
0.0
0.3
β_IV

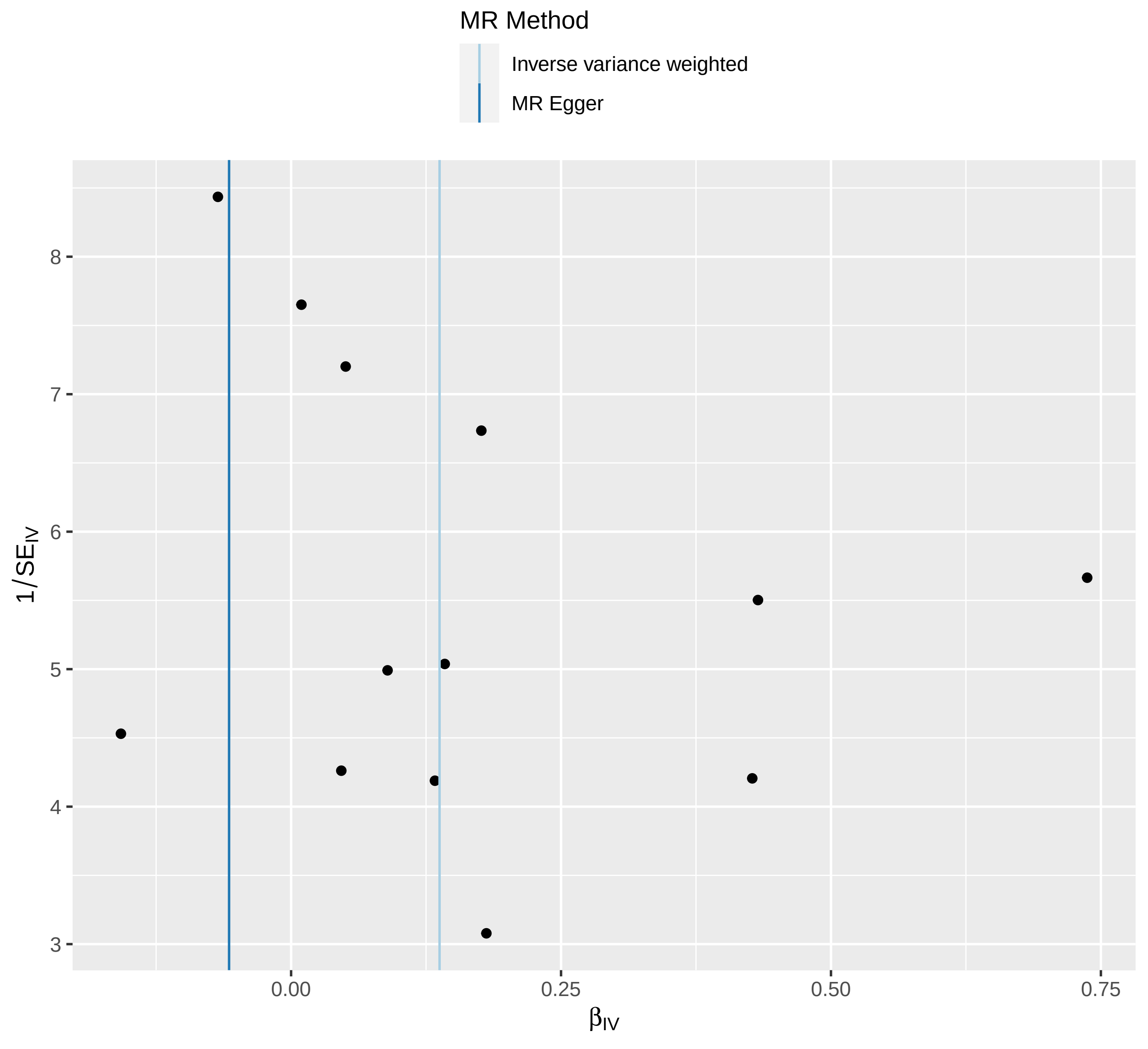

MR Method
Inverse variance weighted
MR Egger
1/SE_IV
8
7
6
5
4
3
0.00
0.25
0.50
0.75
β_IV

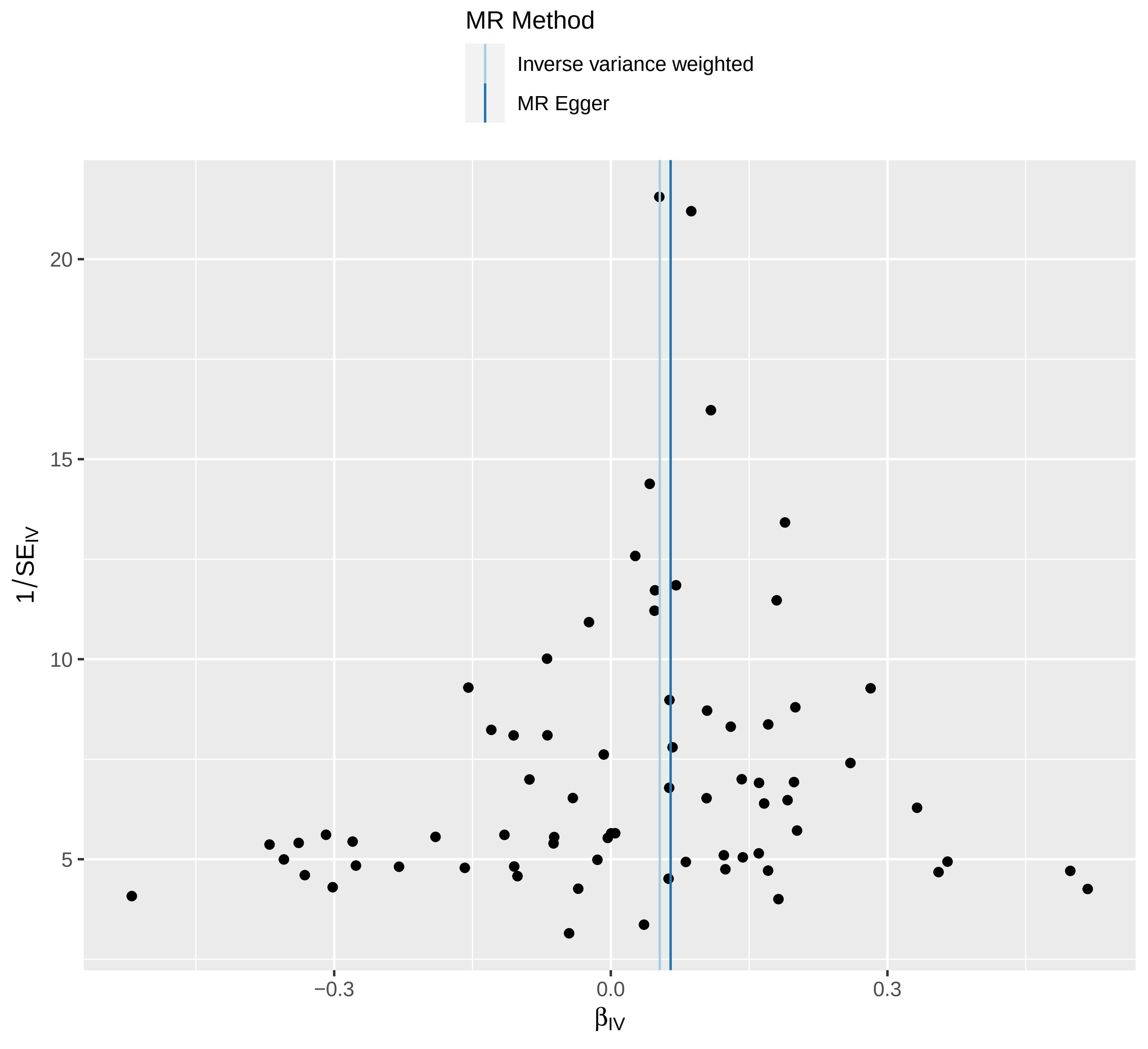
MR Method
Inverse variance weighted
MR Egger
20
15
10
5
$1/SE_{IV}$
−0.3
0.0
0.3
β_{IV}

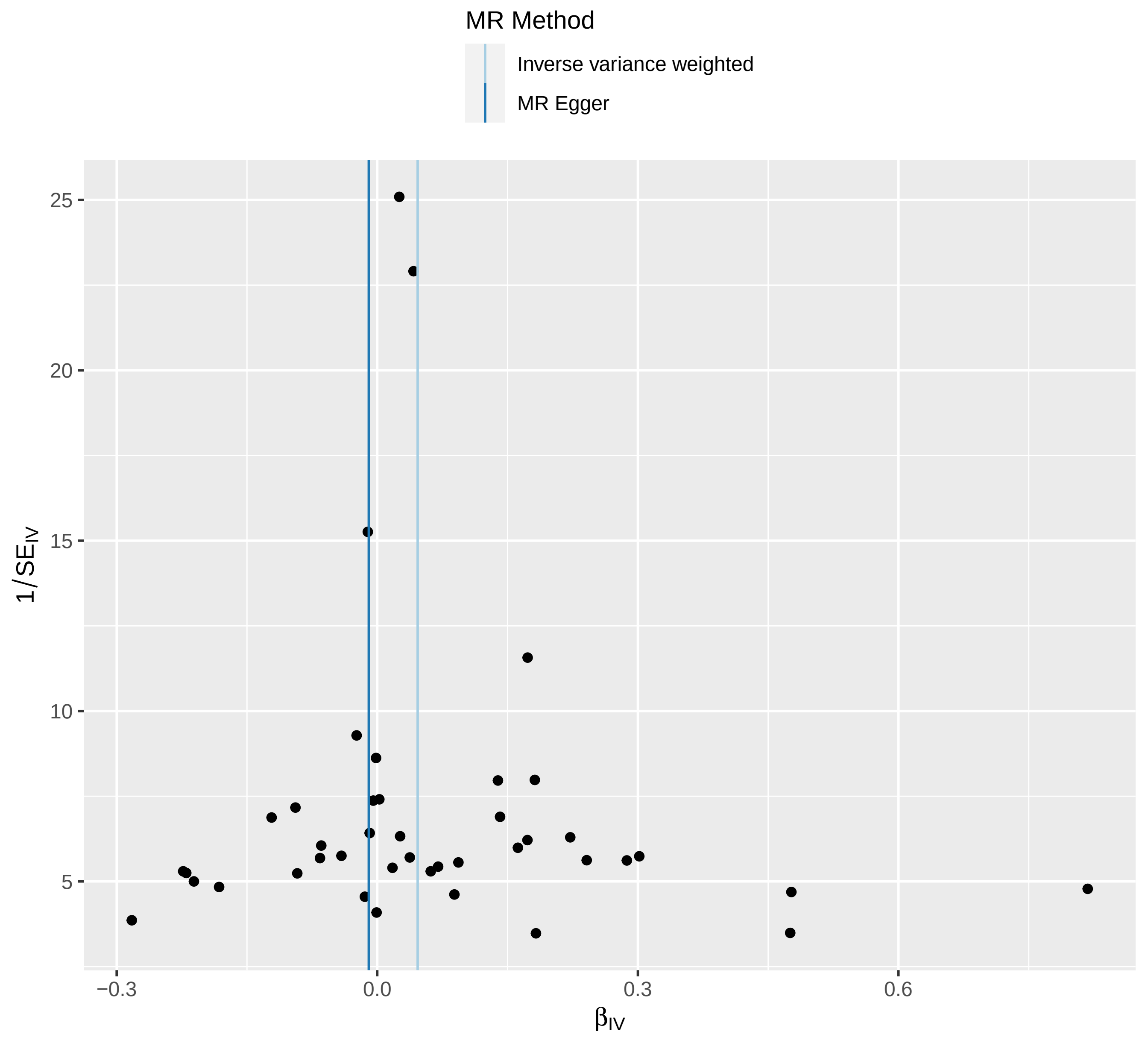

MR Method
Inverse variance weighted
MR Egger
1/SE_IV
25
20
15
10
5
−0.3
0.0
0.3
0.6
β_IV

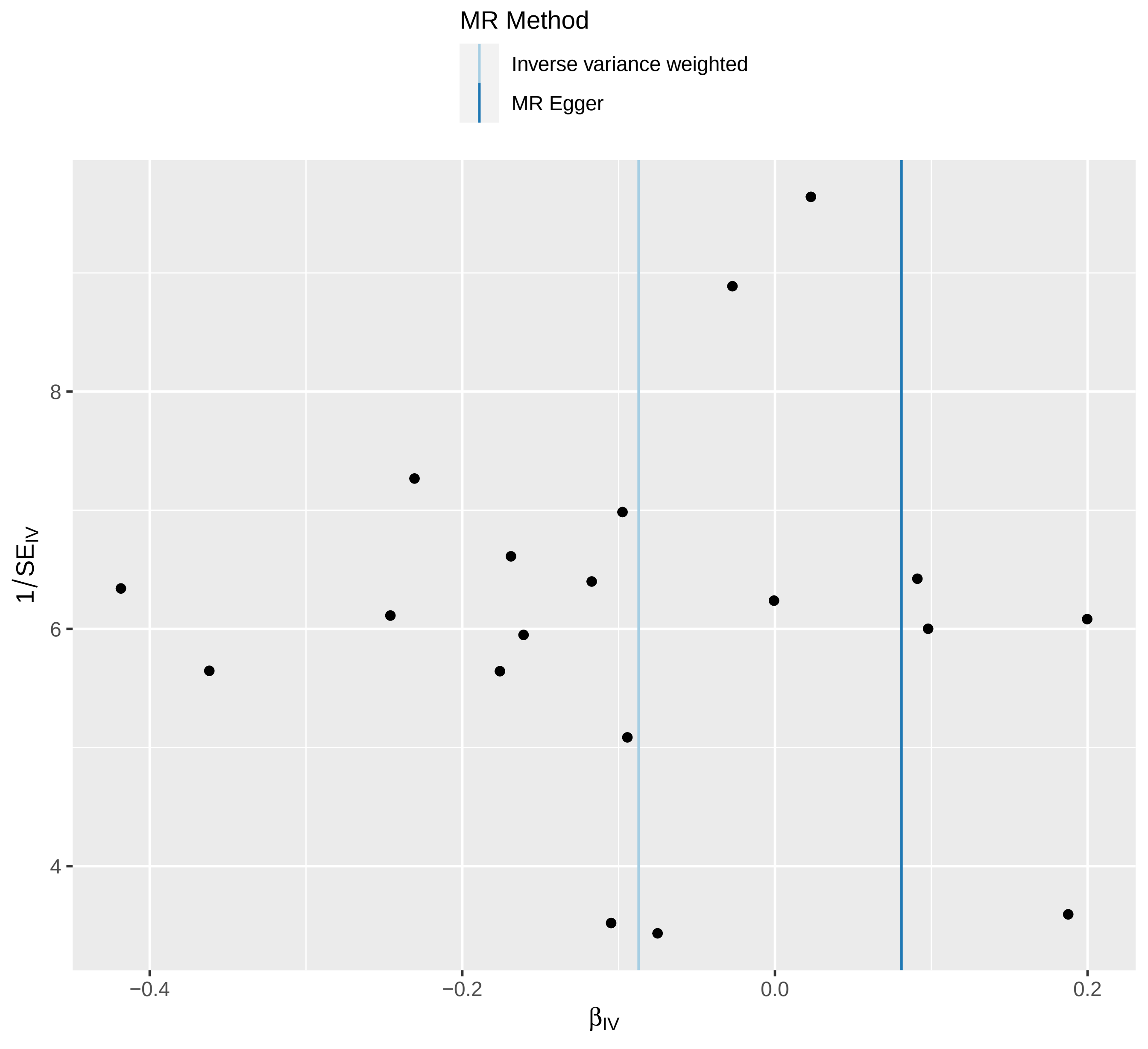

MR Method
Inverse variance weighted
MR Egger
8
6
4
1/SE_IV
−0.4
−0.2
0.0
0.2
β_IV

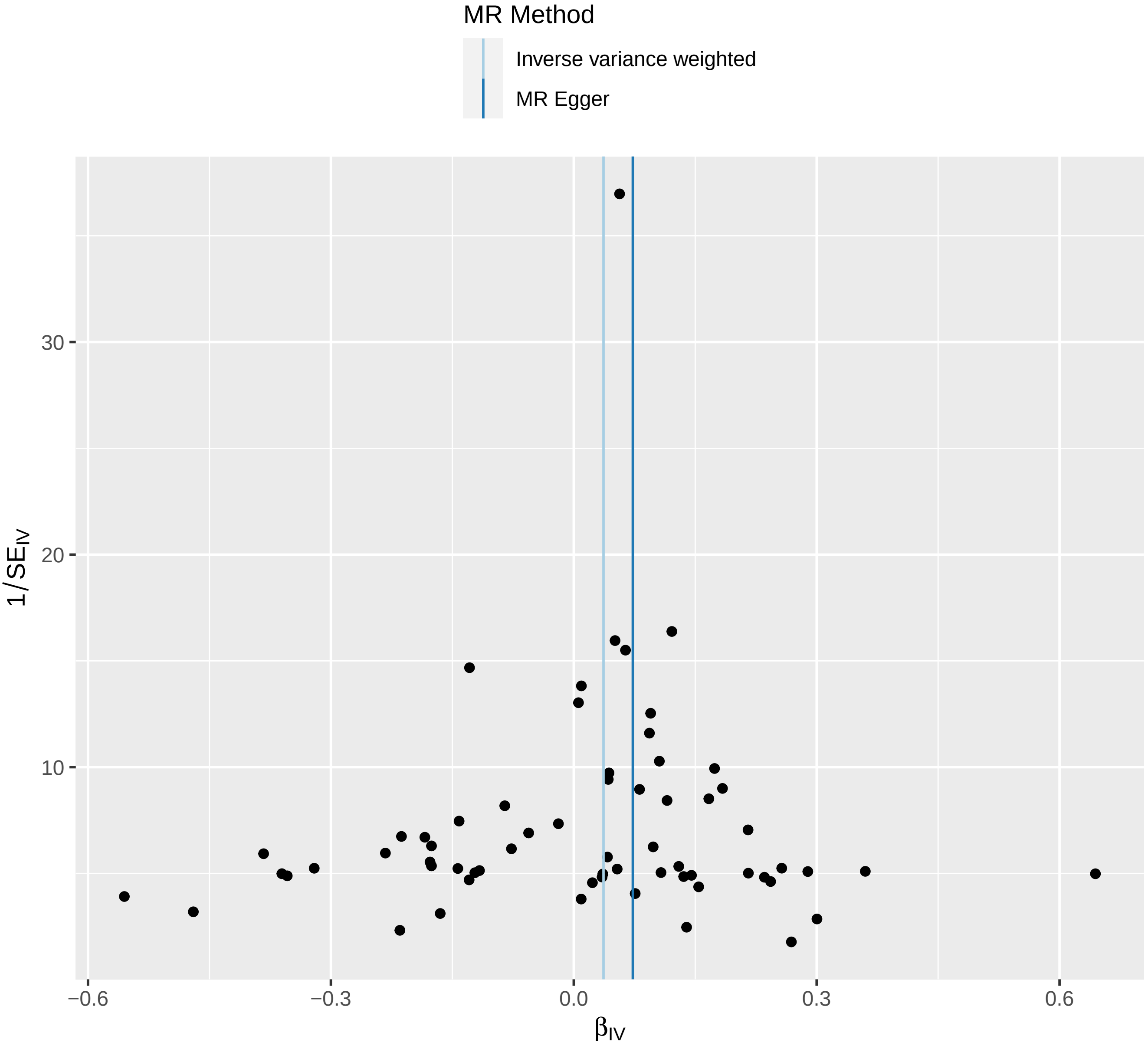
MR Method
Inverse variance weighted
MR Egger
30
20
10
1/SE_IV
−0.6
−0.3
0.0
0.3
0.6
β_IV

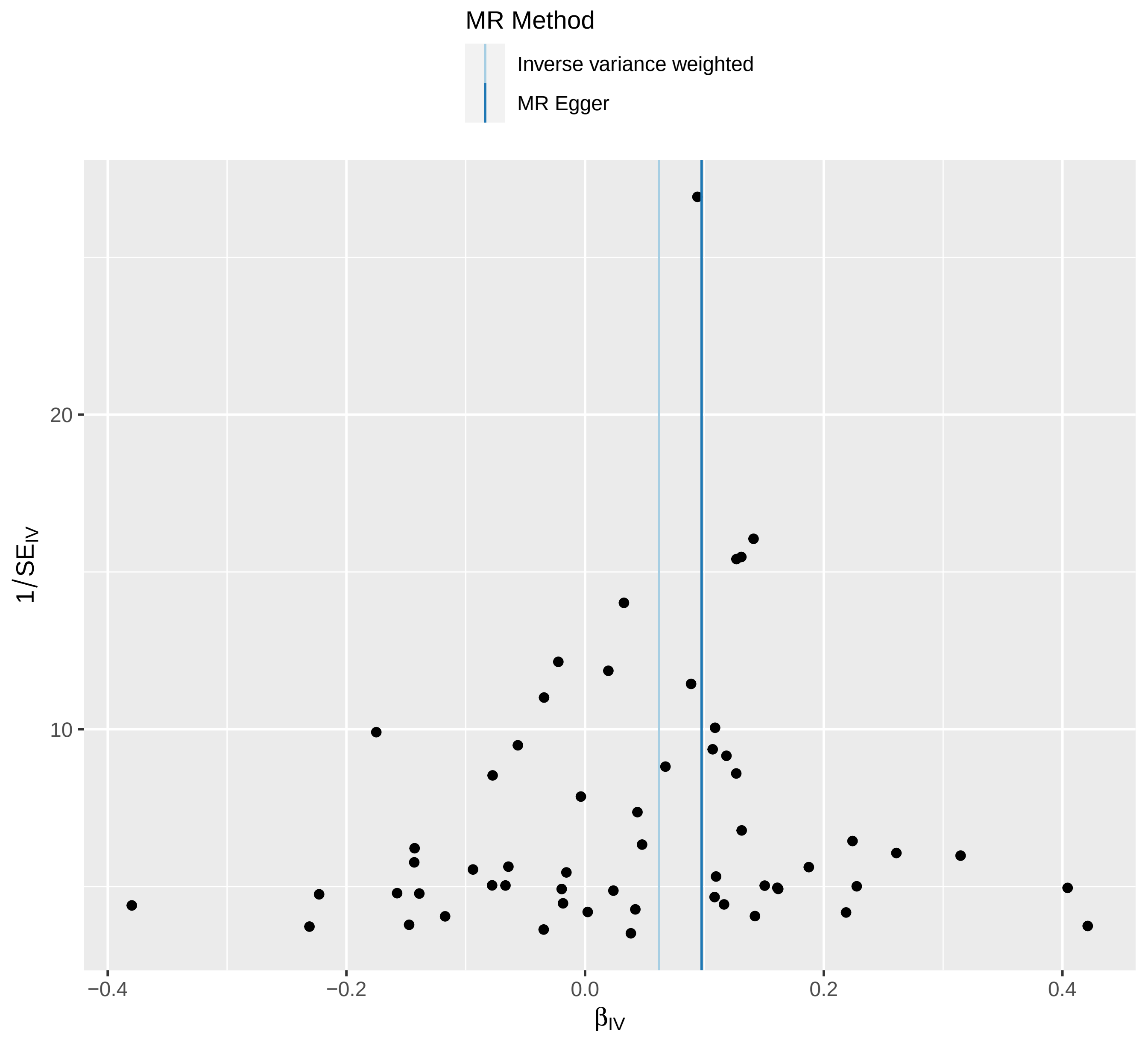

MR Method
Inverse variance weighted
MR Egger
20
10
1/SE_IV
−0.4
−0.2
0.0
0.2
0.4
β_IV

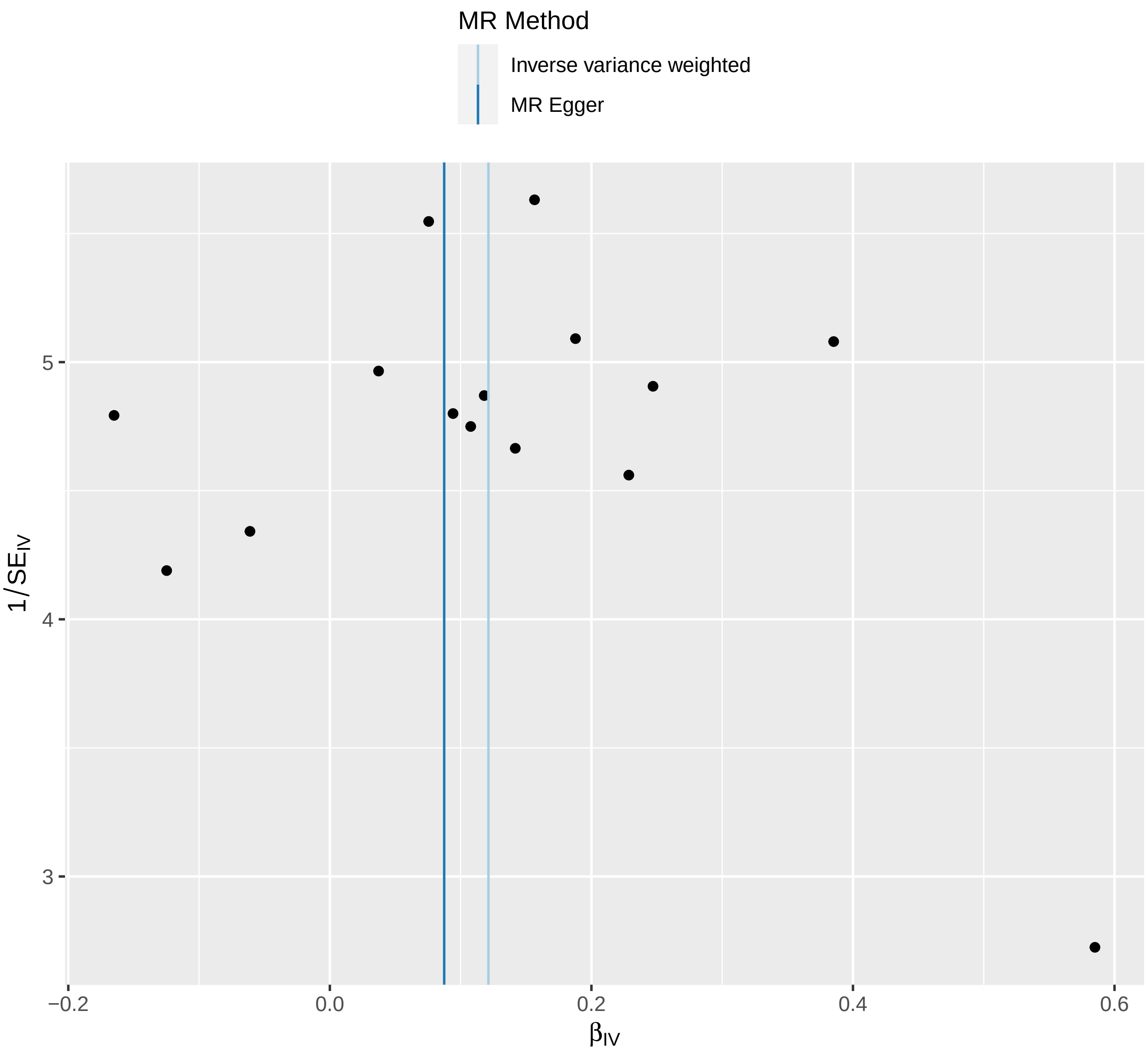

MR Method
Inverse variance weighted
MR Egger
5
4
3
1/SE$_{IV}$
−0.2
0.0
0.2
0.4
0.6
β$_{IV}$

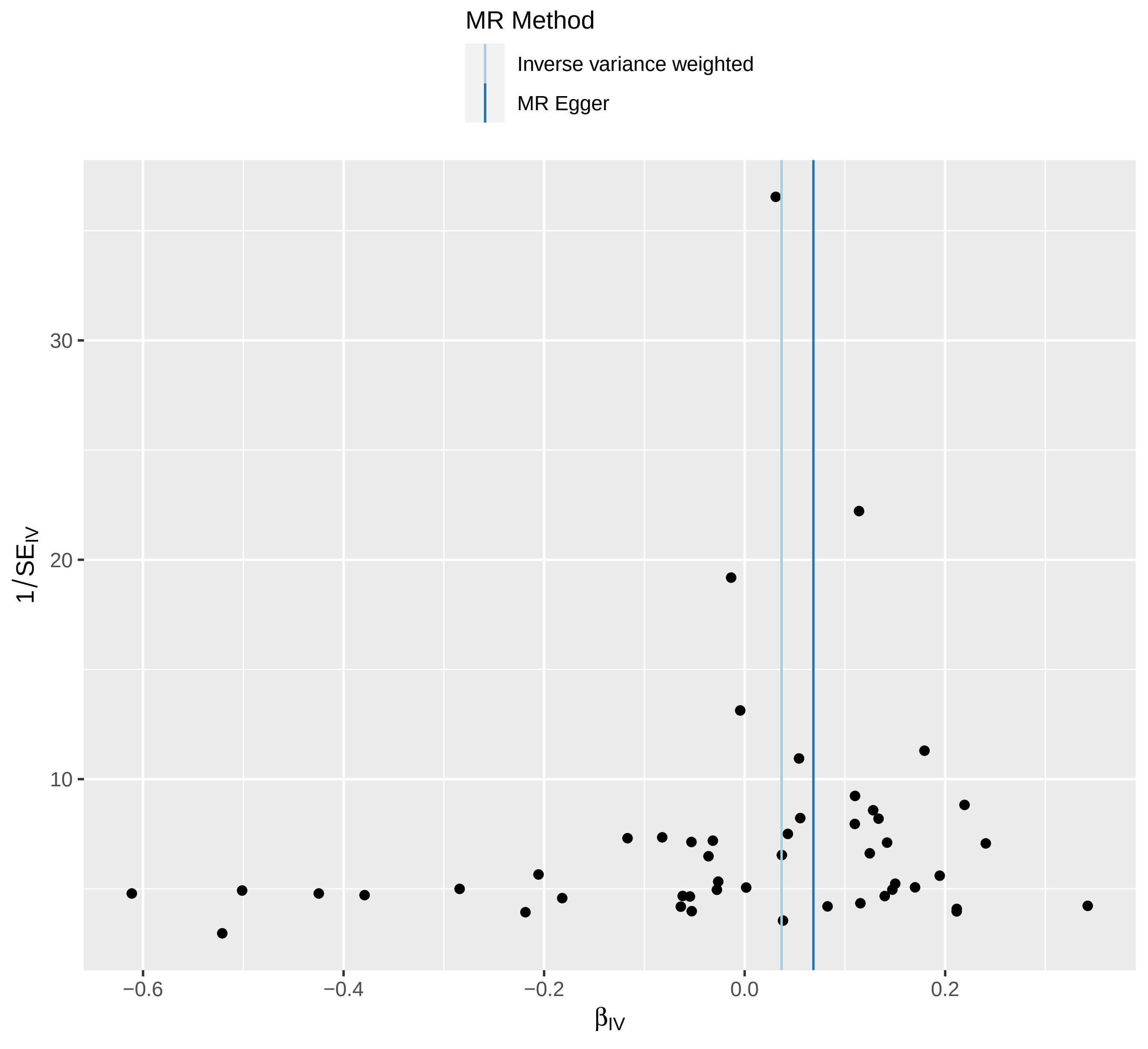
MR Method
Inverse variance weighted
MR Egger
30
20
10
1/SE$_{IV}$
−0.6
−0.4
−0.2
0.0
0.2
β_{IV}

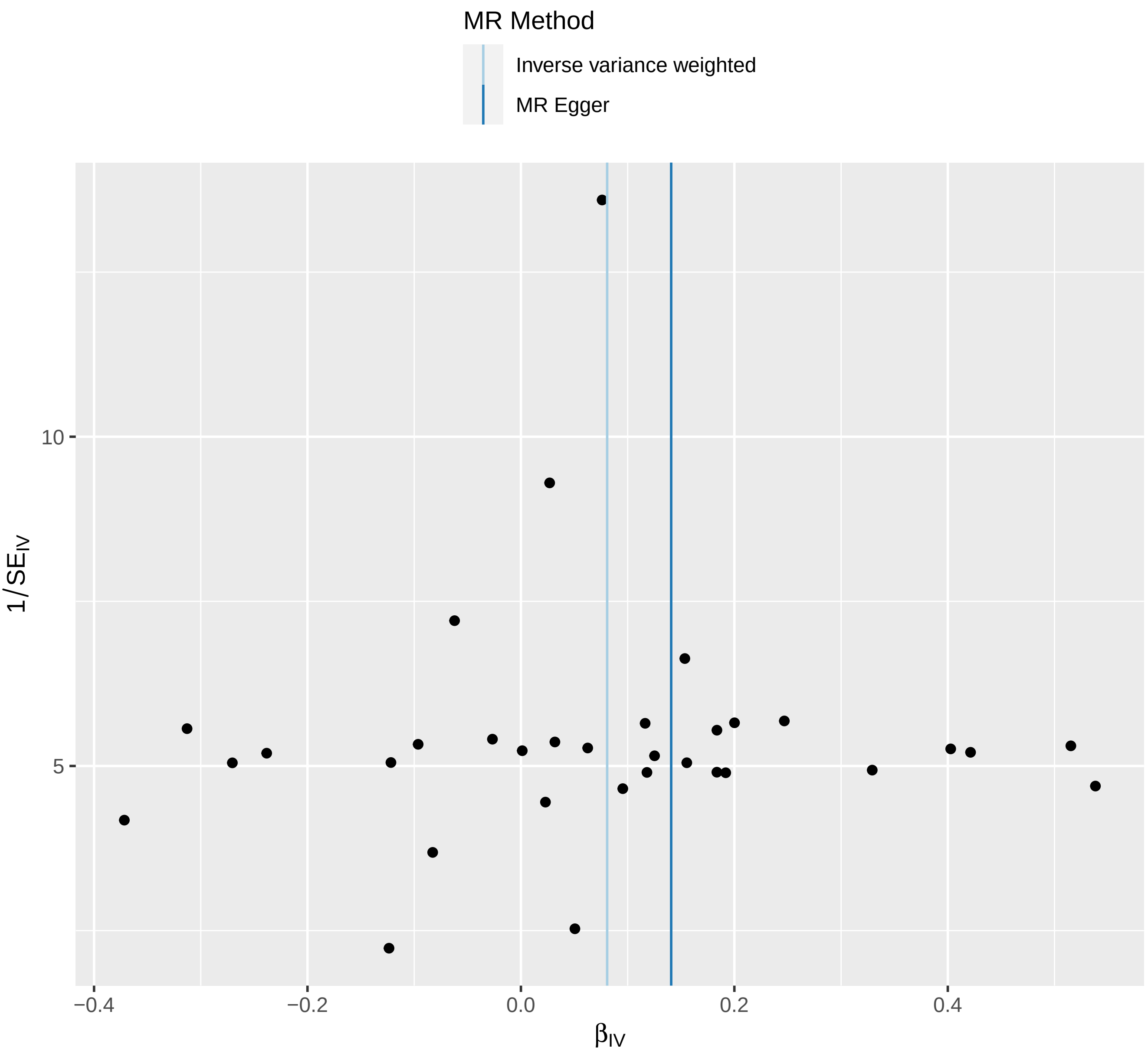

MR Method
Inverse variance weighted
MR Egger
10
5
1/SE_IV
−0.4
−0.2
0.0
0.2
0.4
β_IV

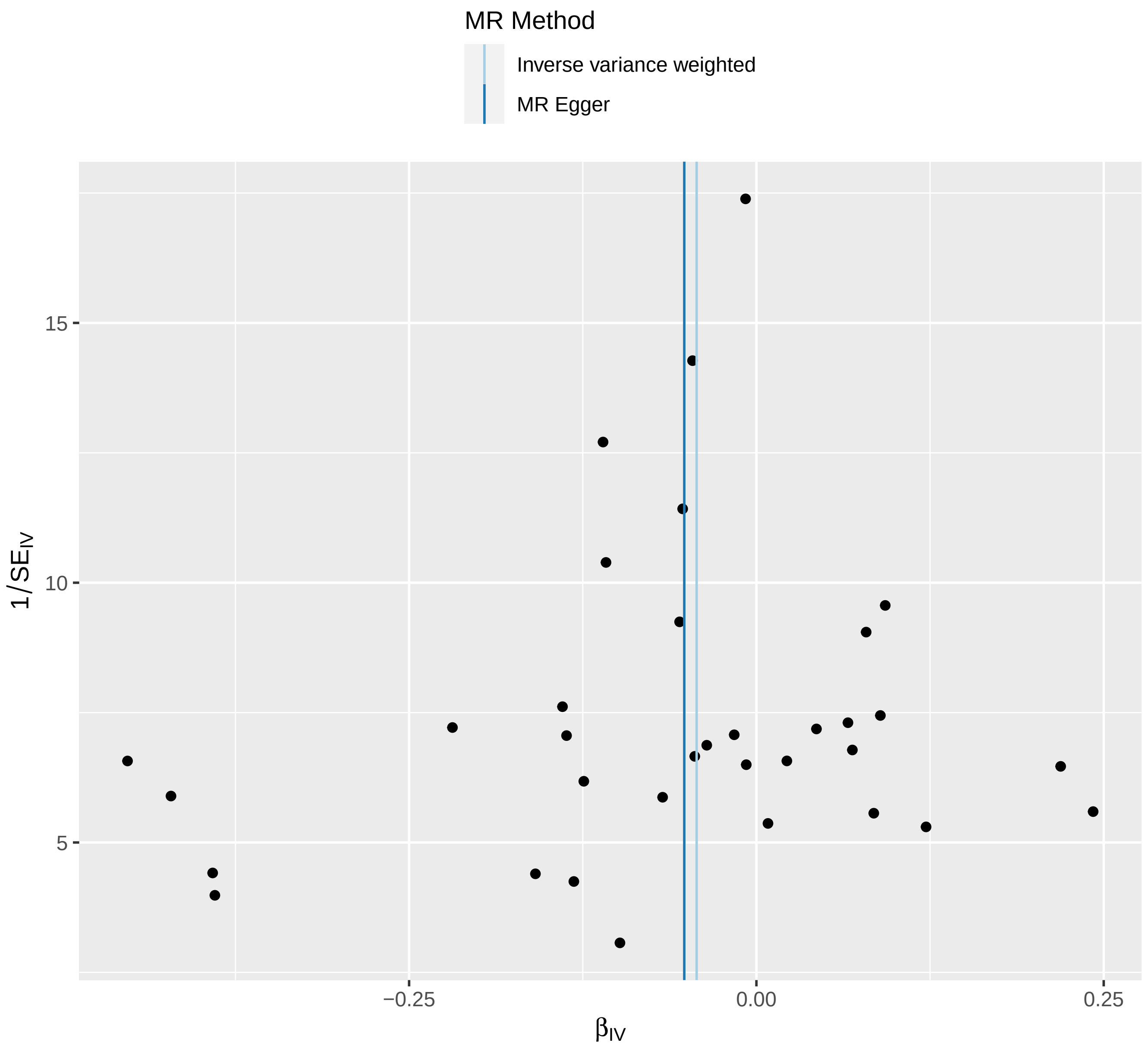

MR Method
Inverse variance weighted
MR Egger
15
10
5
1/SE_IV
−0.25
0.00
0.25
β_IV

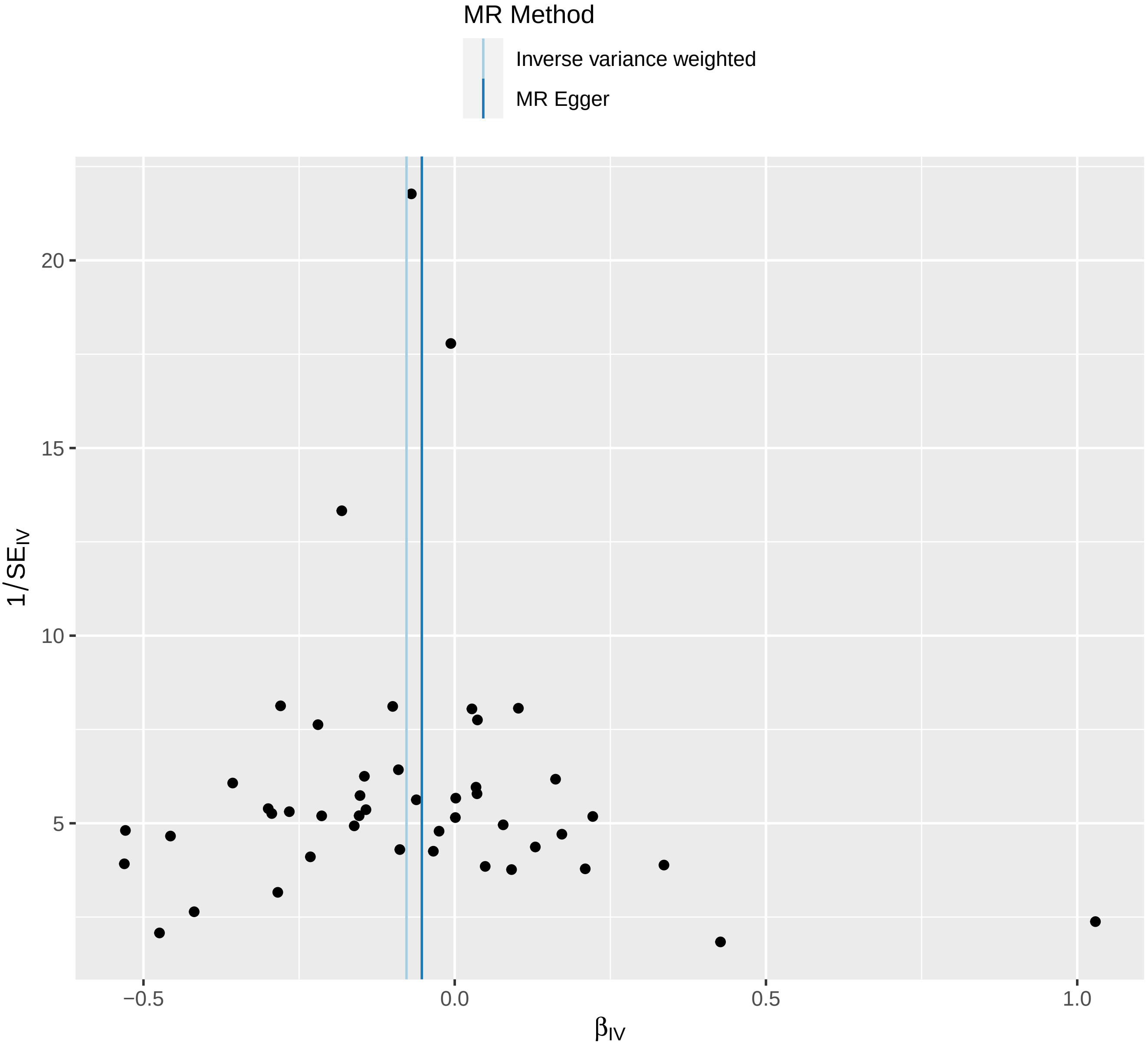
MR Method
Inverse variance weighted
MR Egger
20
15
10
5
1/SE$_{IV}$
−0.5
0.0
0.5
1.0
β$_{IV}$

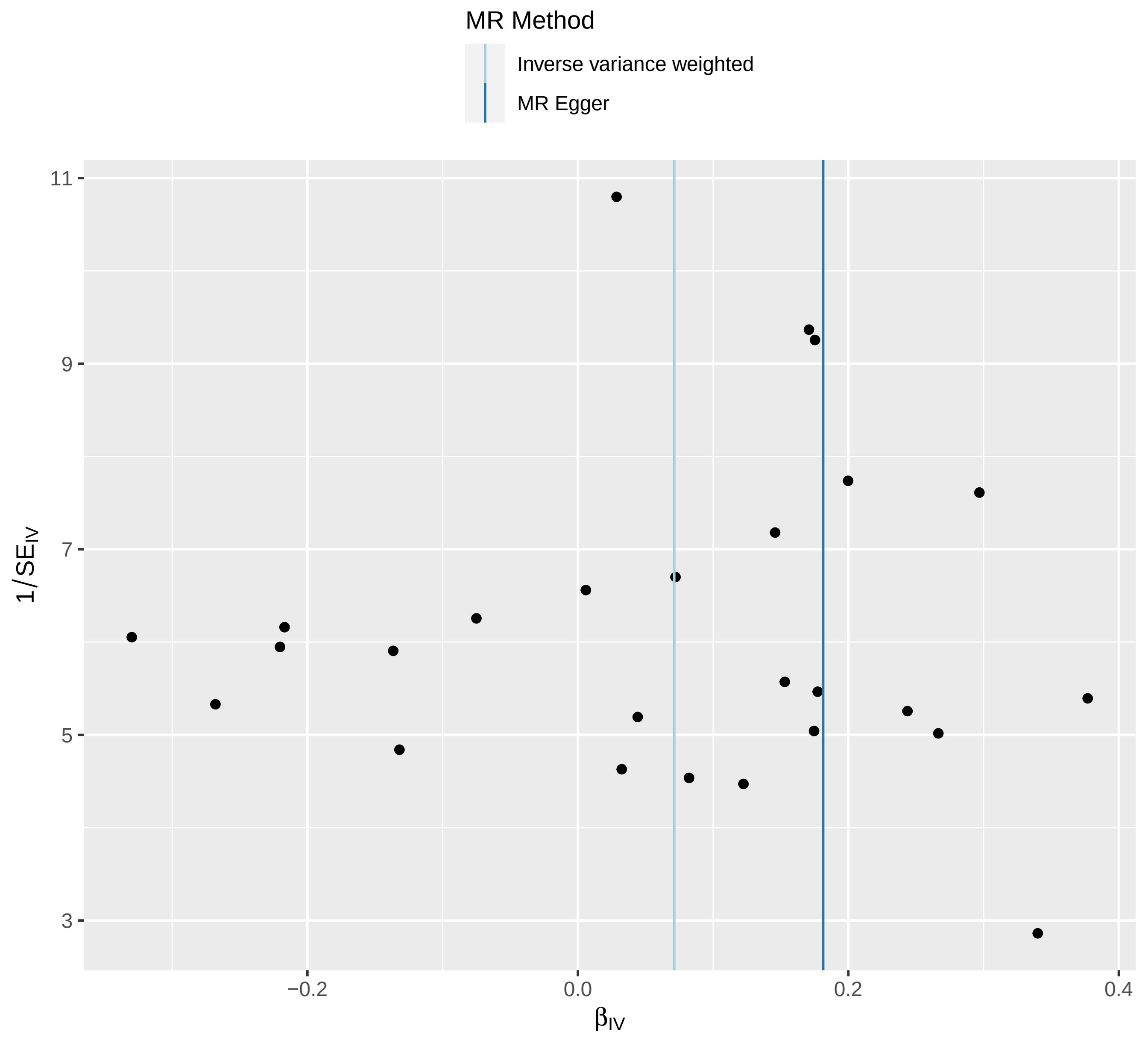
MR Method
Inverse variance weighted
MR Egger
11
9
7
5
3
$1/SE_{IV}$
−0.2
0.0
0.2
0.4
β_{IV}

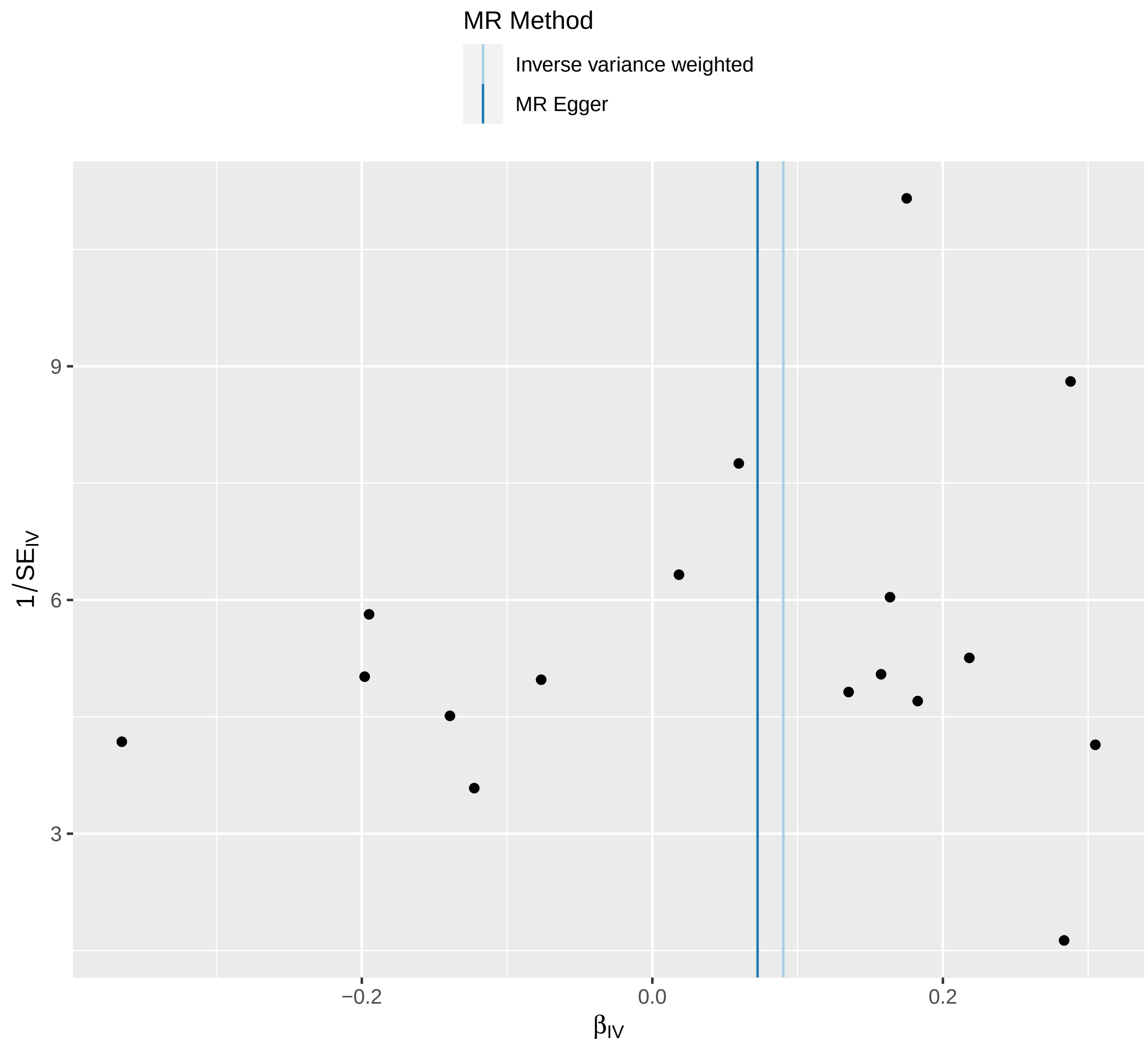

MR Method
Inverse variance weighted
MR Egger
9
6
3
$1/SE_{IV}$
−0.2
0.0
0.2
β_{IV}

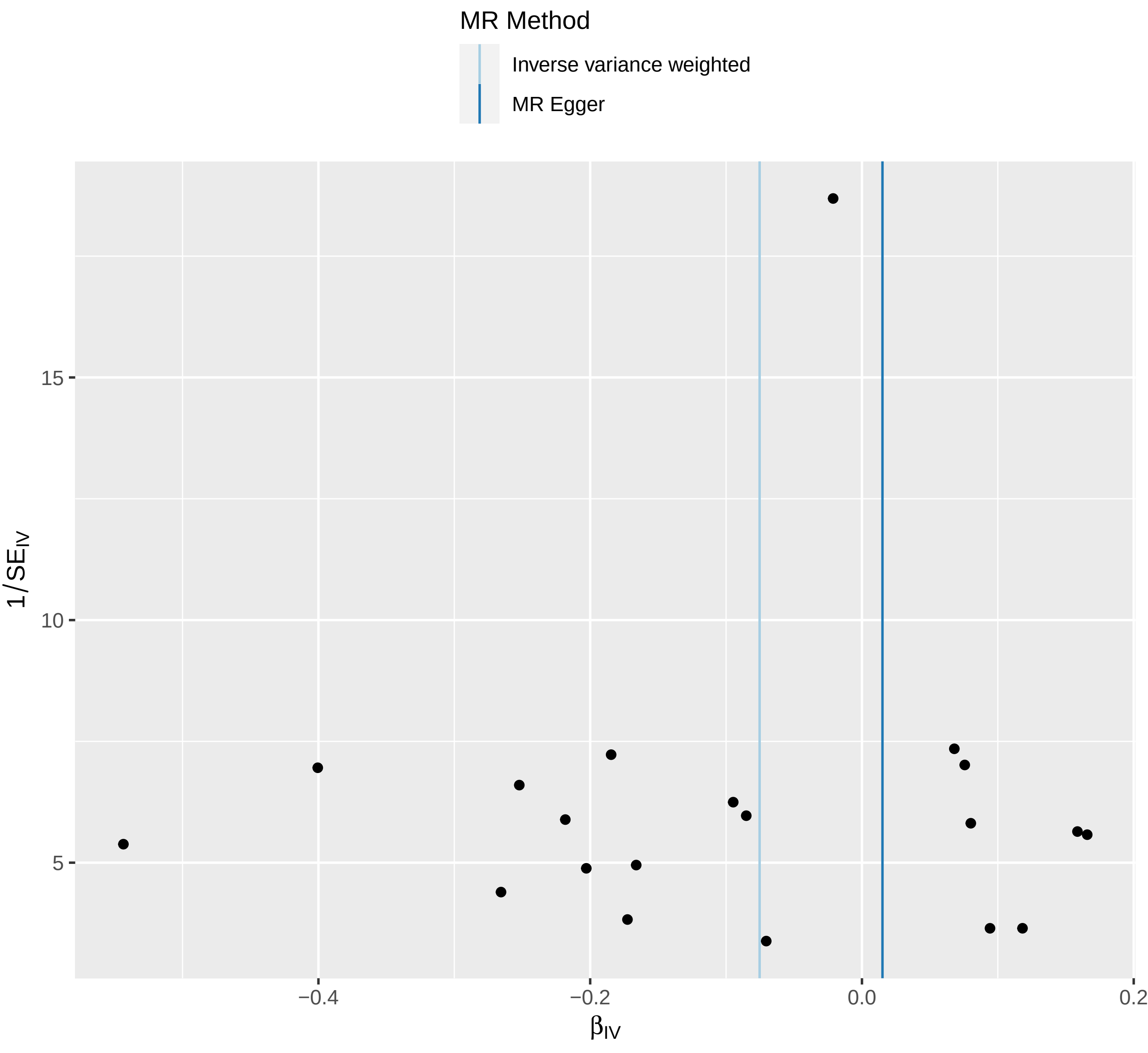
MR Method
Inverse variance weighted
MR Egger
15
10
5
1/SE_IV
−0.4
−0.2
0.0
0.2
β_IV

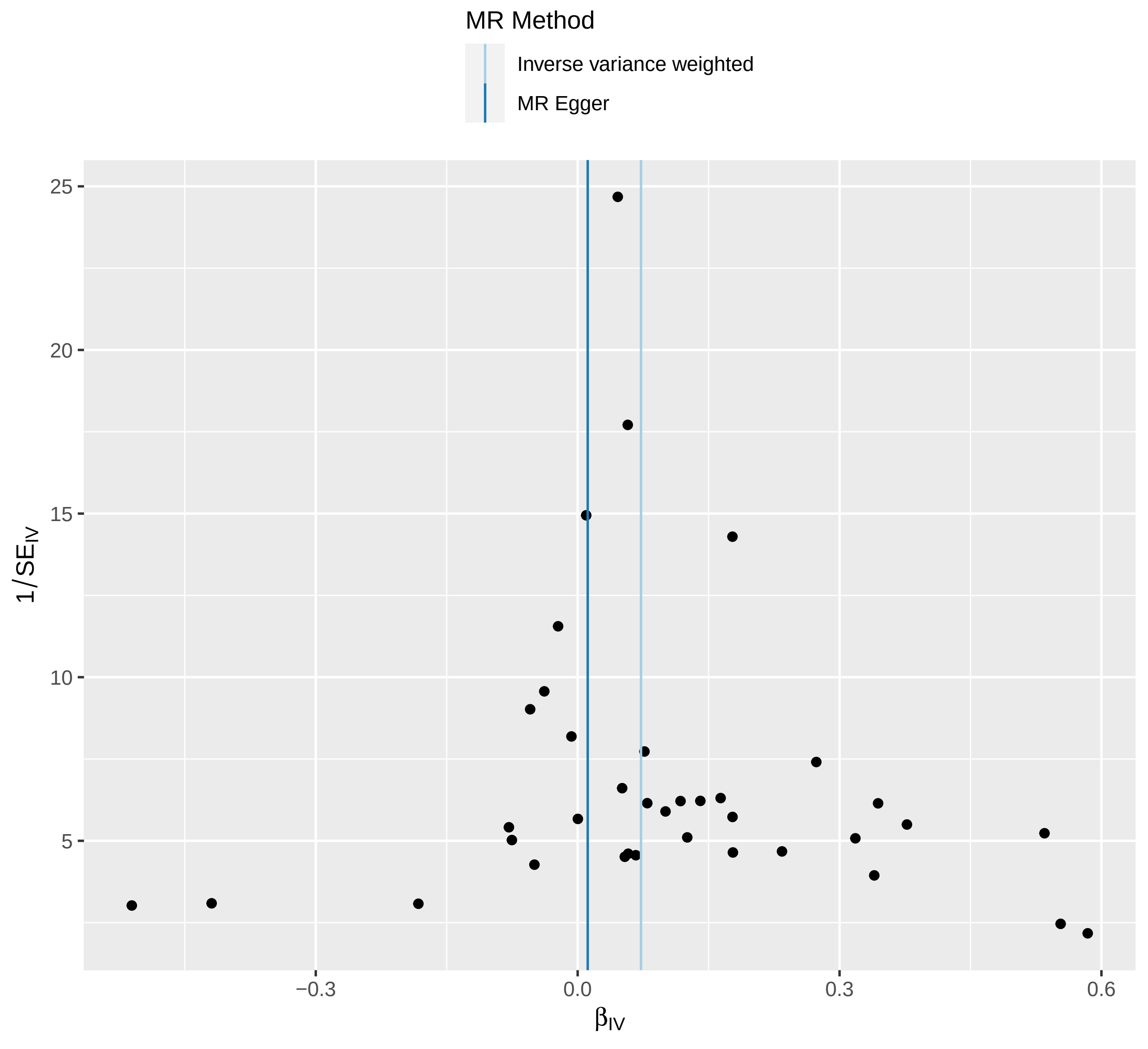
MR Method
Inverse variance weighted
MR Egger
25
20
15
10
5
$1/SE_{IV}$
−0.3
0.0
0.3
0.6
β_{IV}

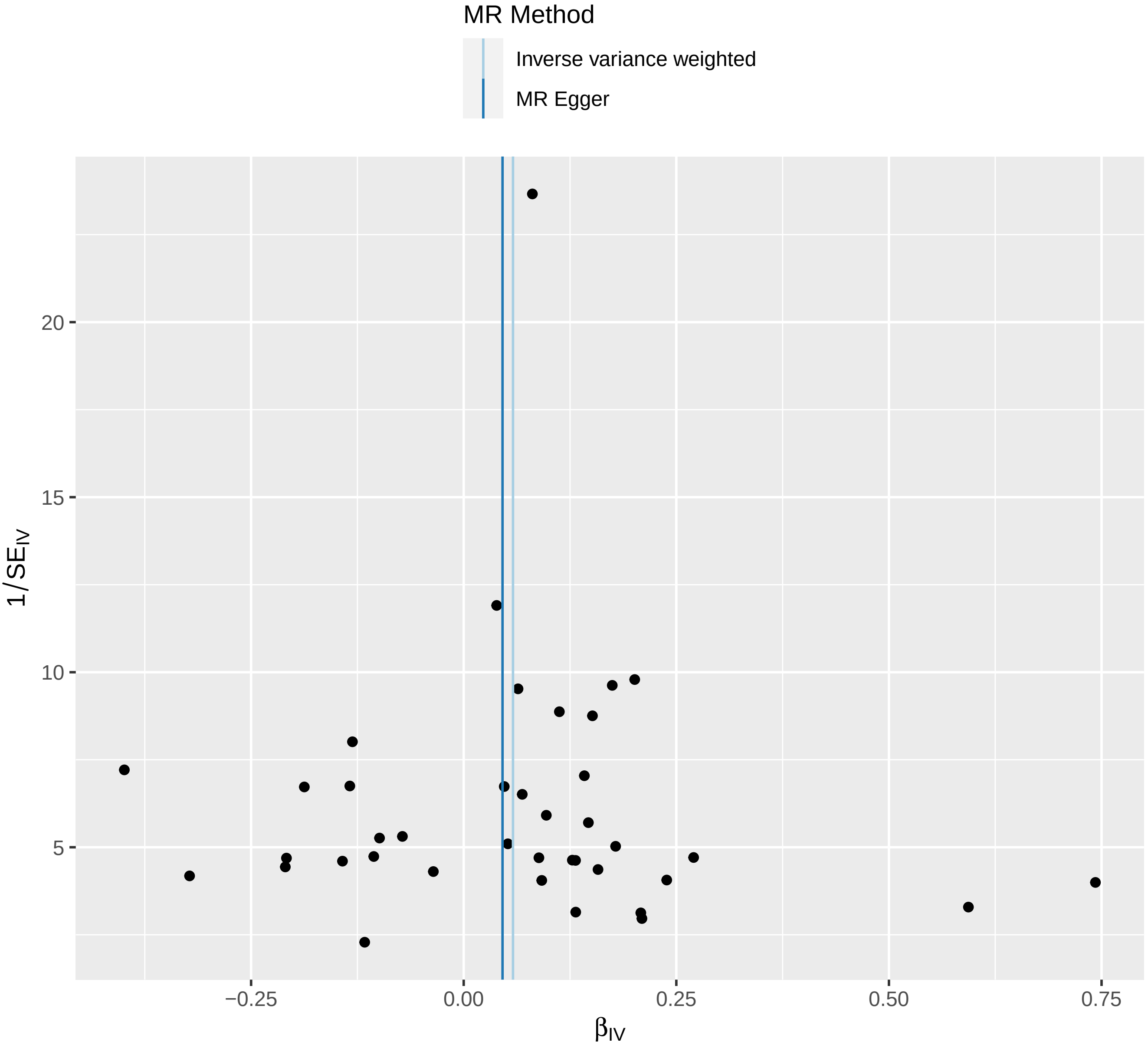
MR Method
Inverse variance weighted
MR Egger
20
15
10
5
1/SE_IV
−0.25
0.00
0.25
0.50
0.75
β_IV

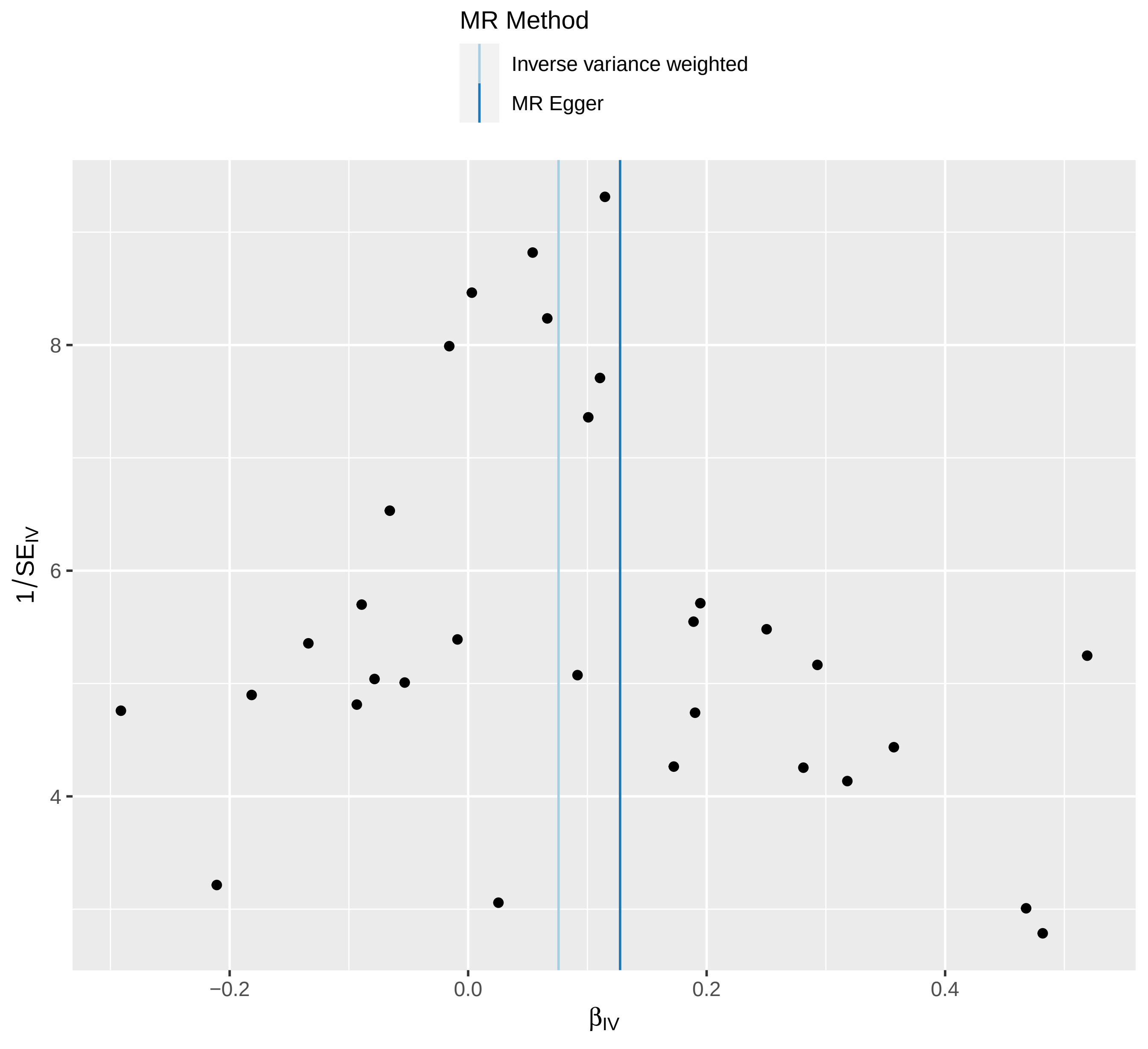

MR Method
Inverse variance weighted
MR Egger
1/SE_IV
8
6
4
−0.2
0.0
0.2
0.4
β_IV

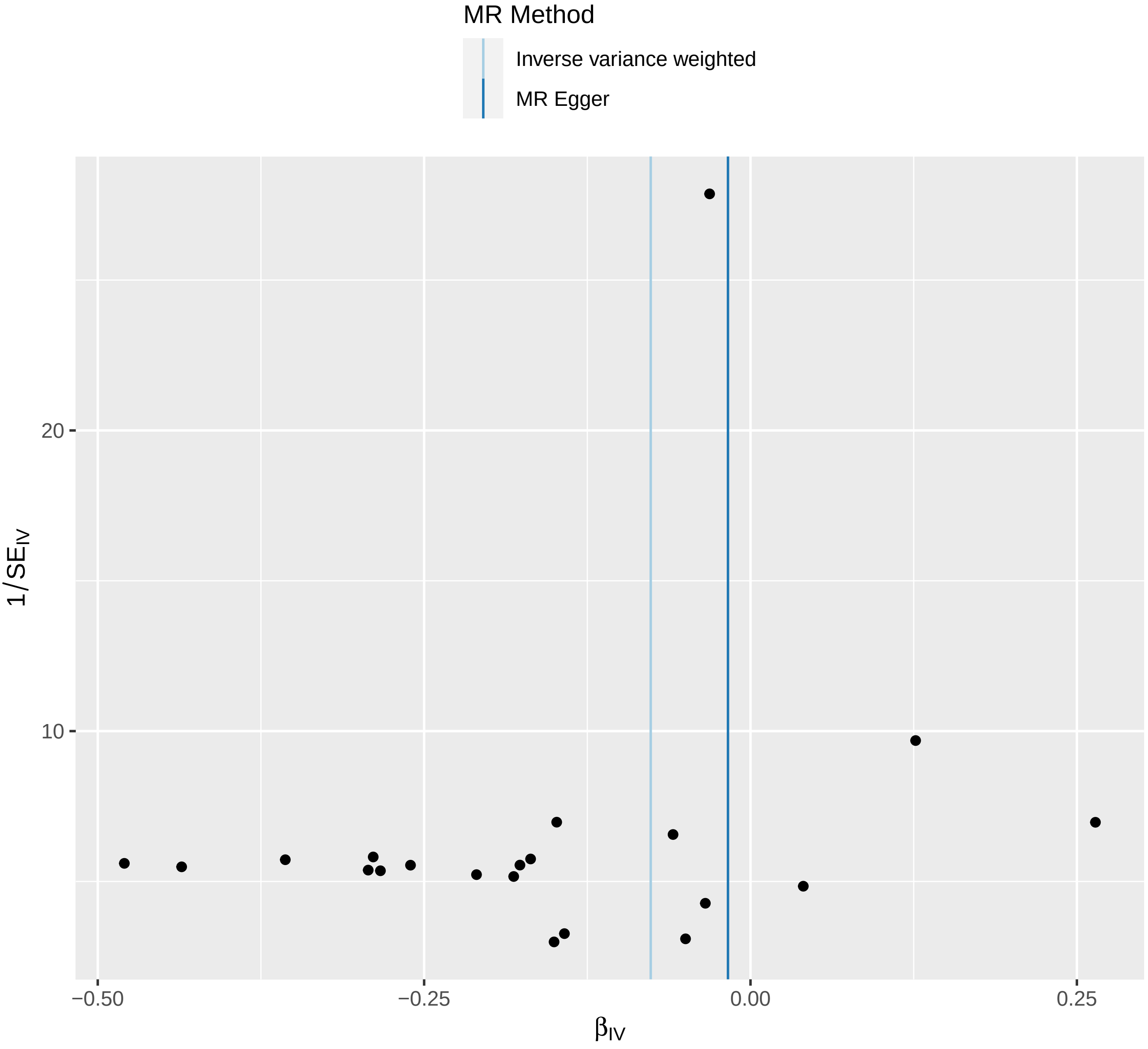
MR Method
Inverse variance weighted
MR Egger
20
10
$1/SE_{IV}$
−0.50
−0.25
0.00
0.25
β_{IV}

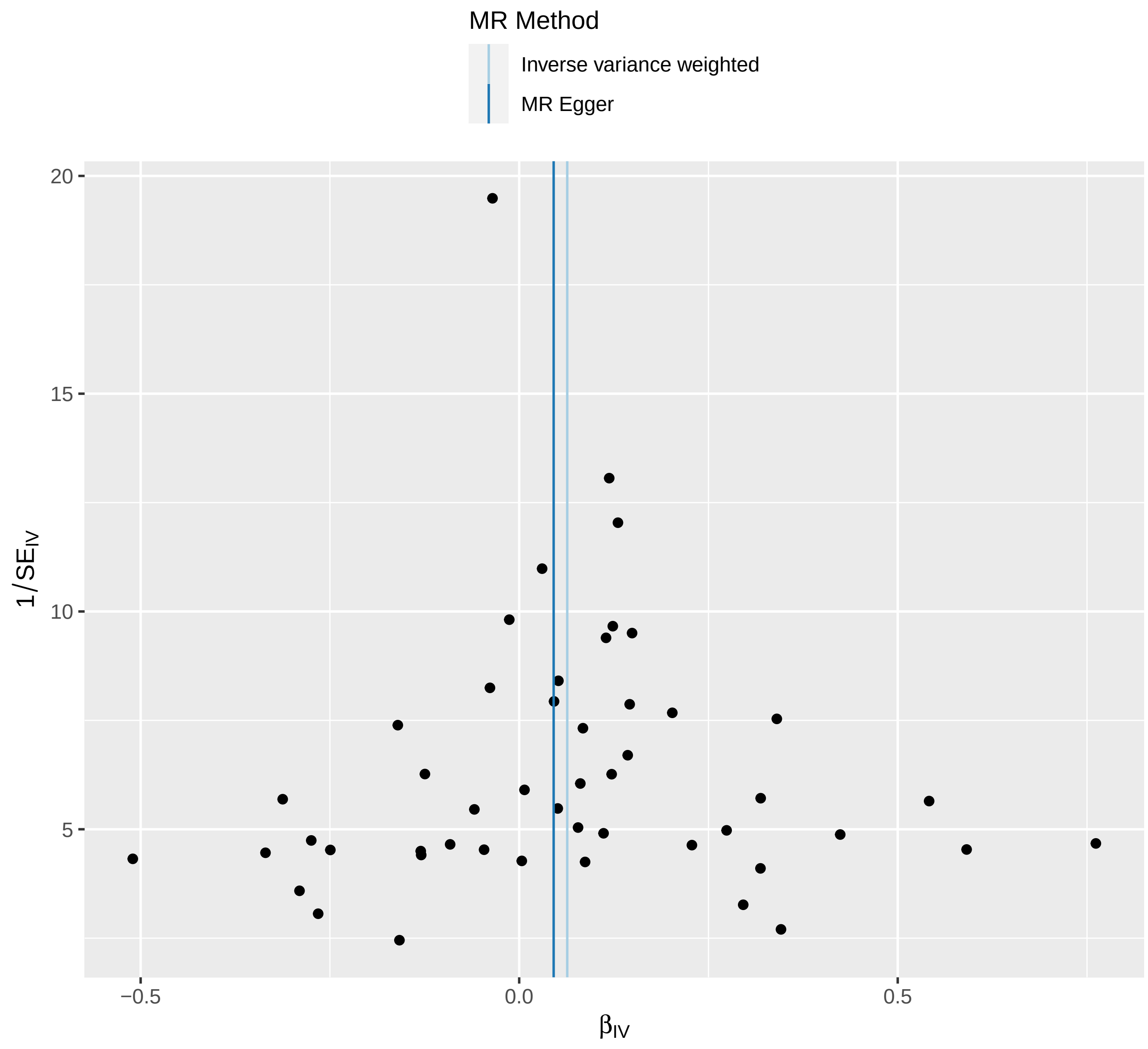

MR Method
Inverse variance weighted
MR Egger
20
15
10
5
1/SE$_{IV}$
−0.5
0.0
0.5
β$_{IV}$

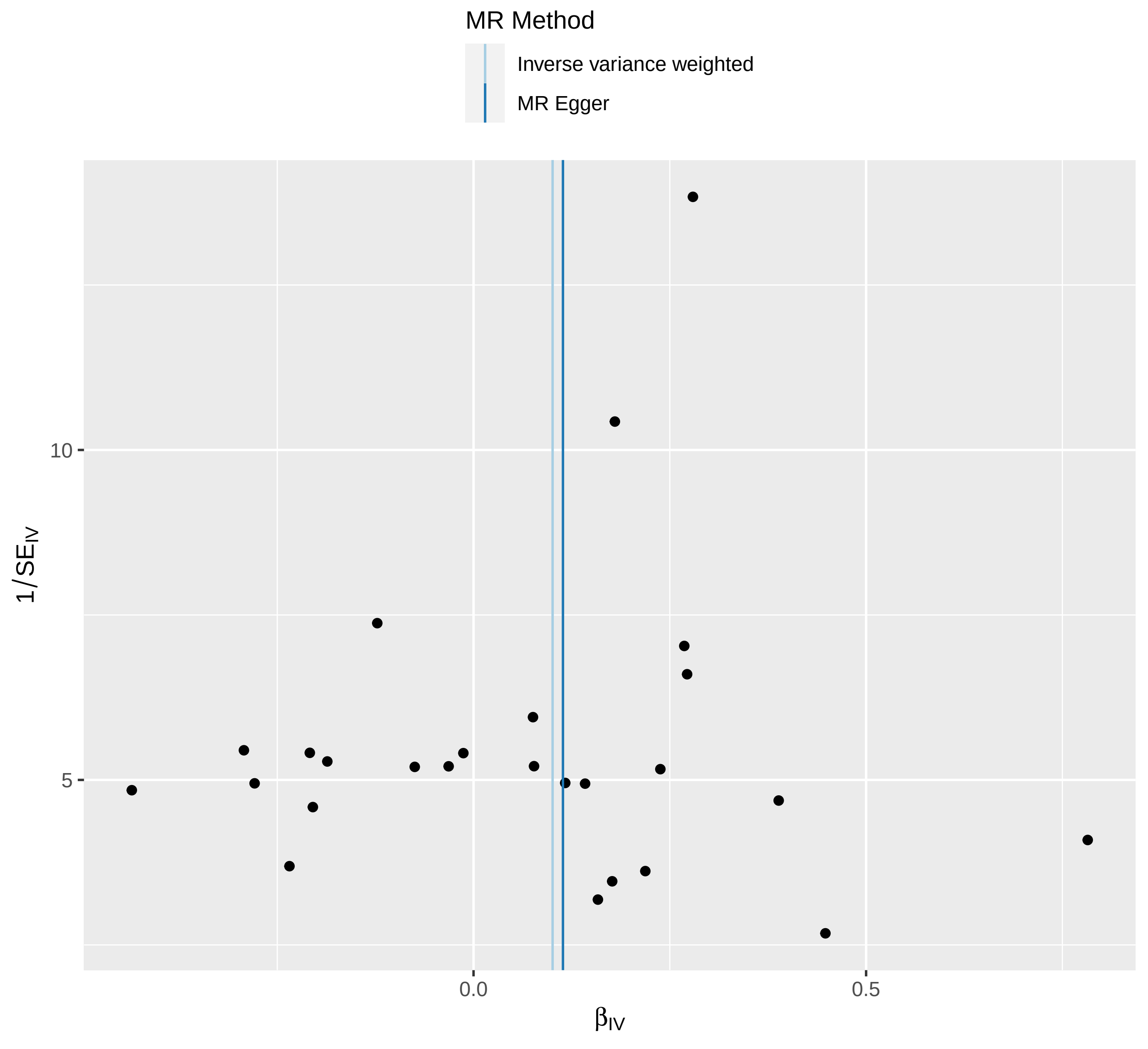
MR Method
Inverse variance weighted
MR Egger
10
5
$1/SE_{IV}$
0.0
0.5
β_{IV}

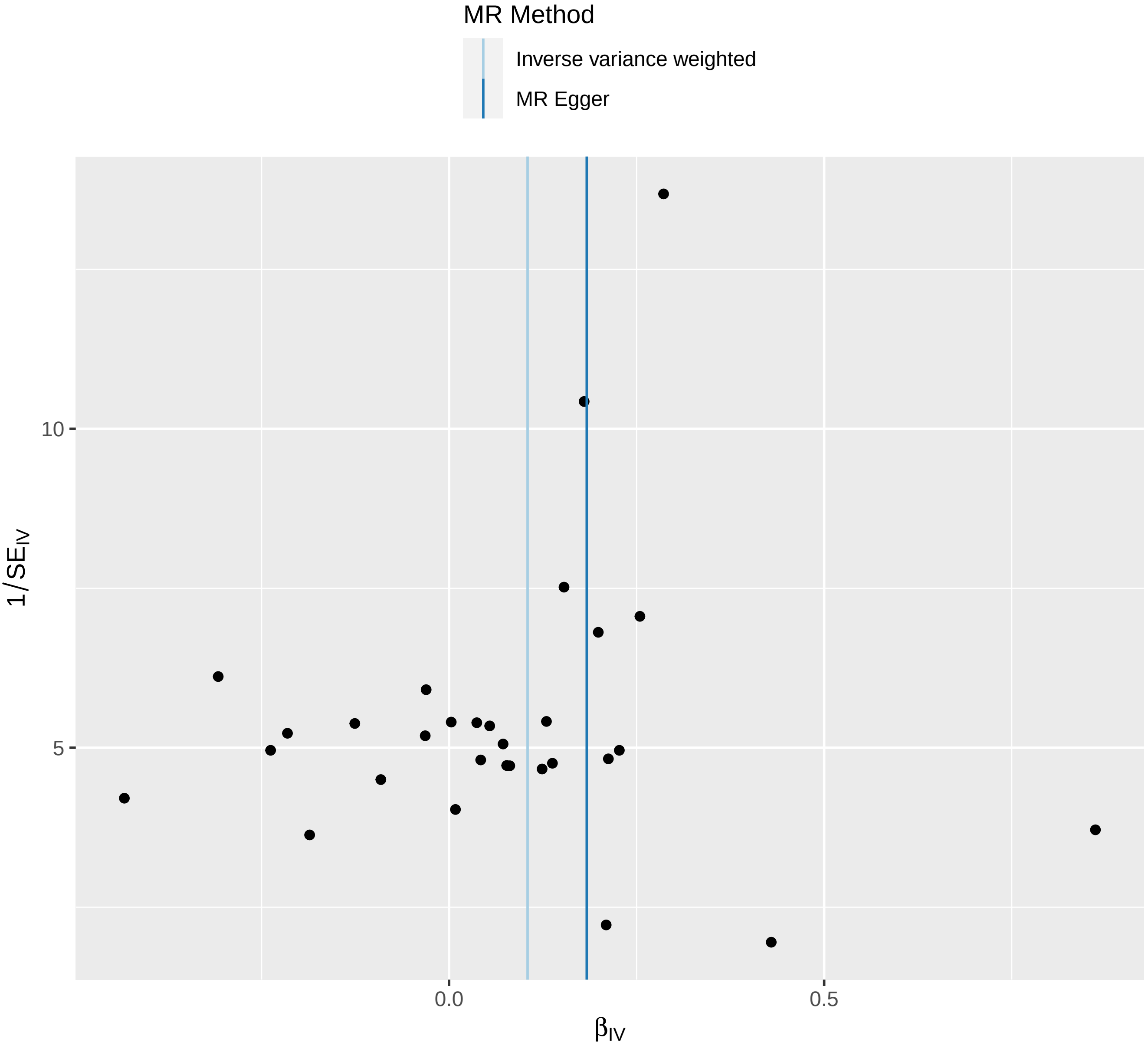
MR Method
Inverse variance weighted
MR Egger
1/SE_IV
10
5
0.0
0.5
β_IV

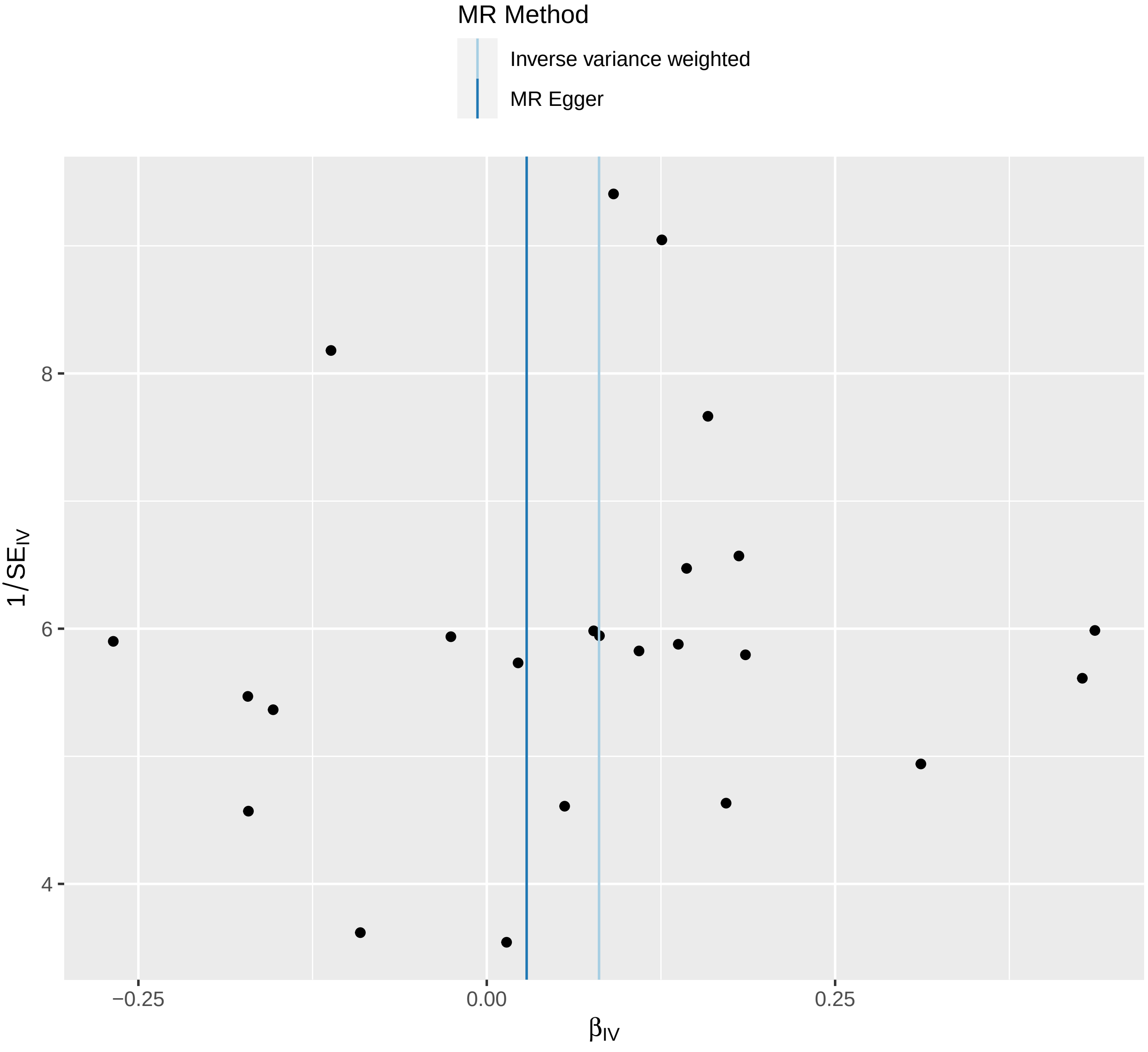
MR Method
Inverse variance weighted
MR Egger
8
6
4
1/SE$_{IV}$
−0.25
0.00
0.25
β$_{IV}$

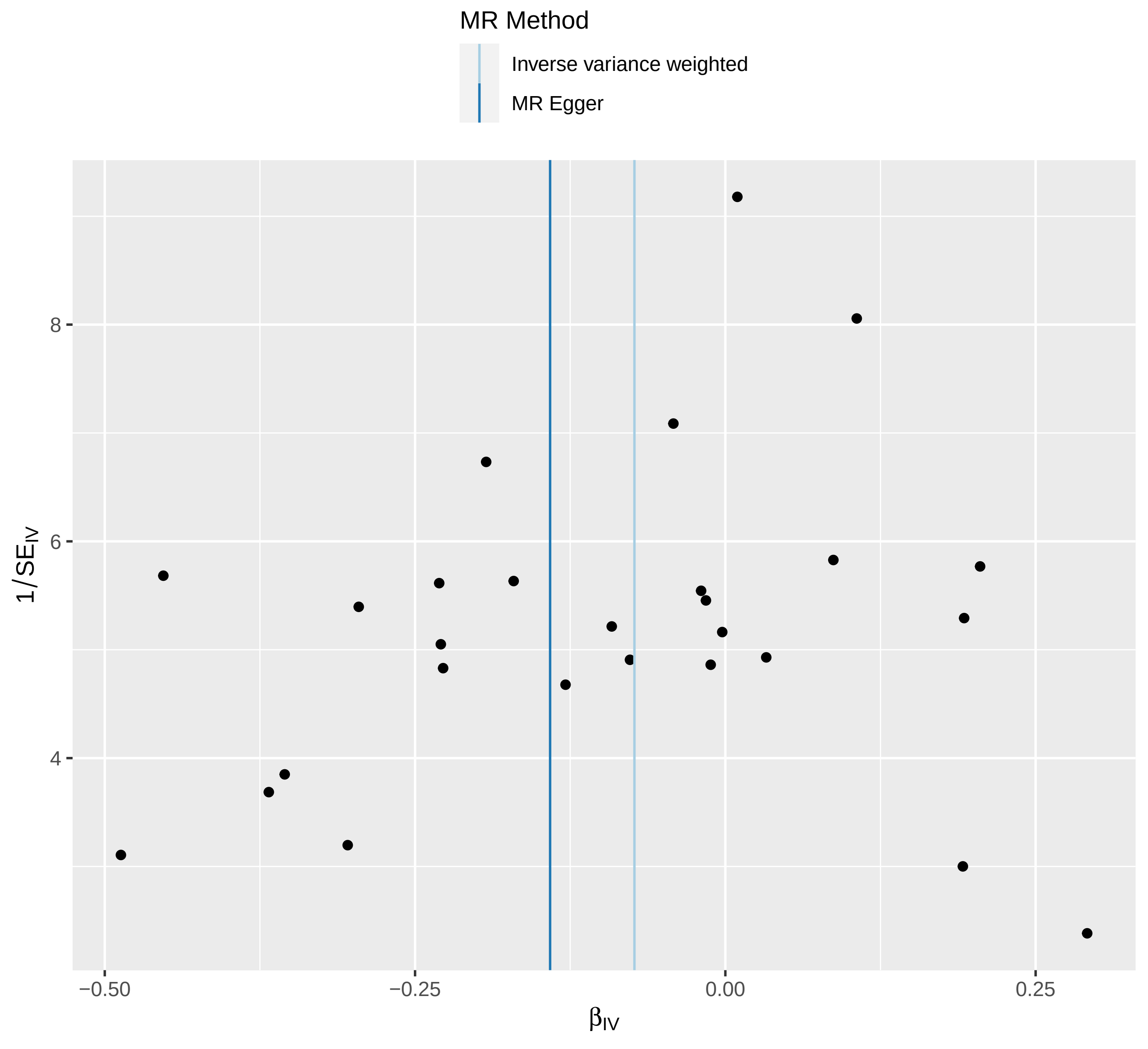

MR Method
Inverse variance weighted
MR Egger
8
6
4
$1/SE_{IV}$
−0.50
−0.25
0.00
0.25
β_{IV}

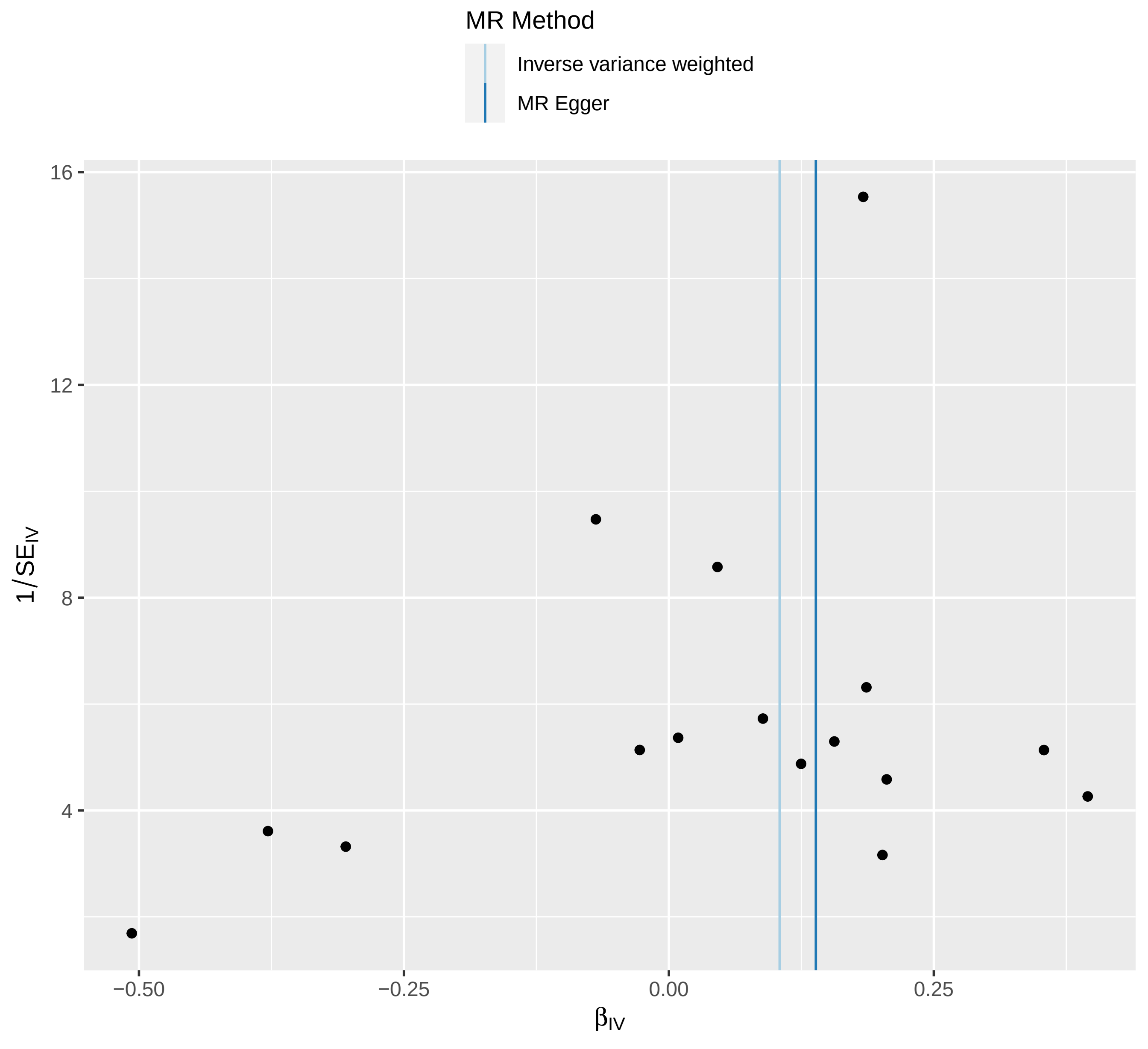

MR Method
Inverse variance weighted
MR Egger
16
12
8
4
1/SE$_{IV}$
−0.50
−0.25
0.00
0.25
β$_{IV}$

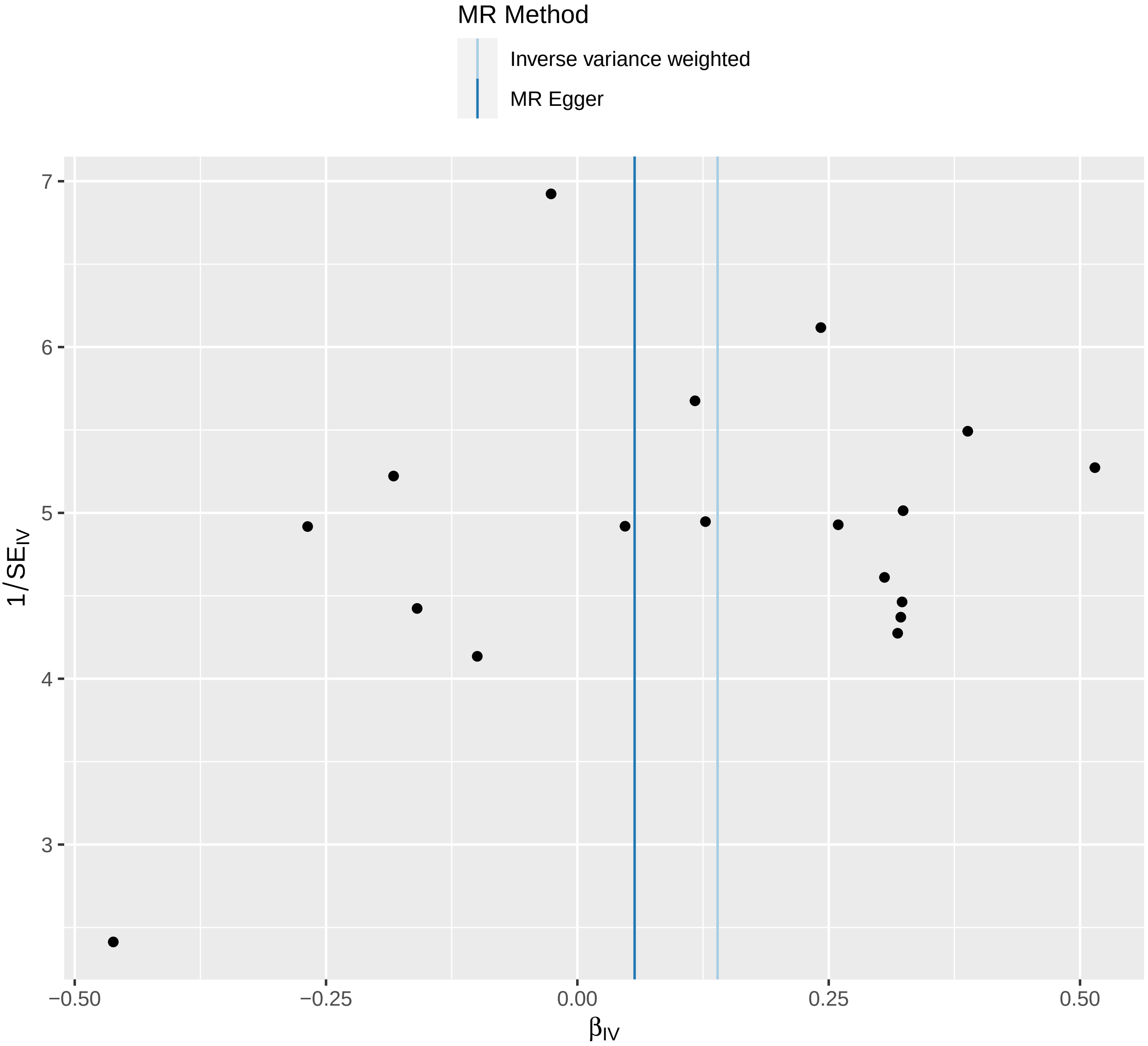

MR Method
Inverse variance weighted
MR Egger
7
6
5
4
3
1/SE_IV
−0.50
−0.25
0.00
0.25
0.50
β_IV

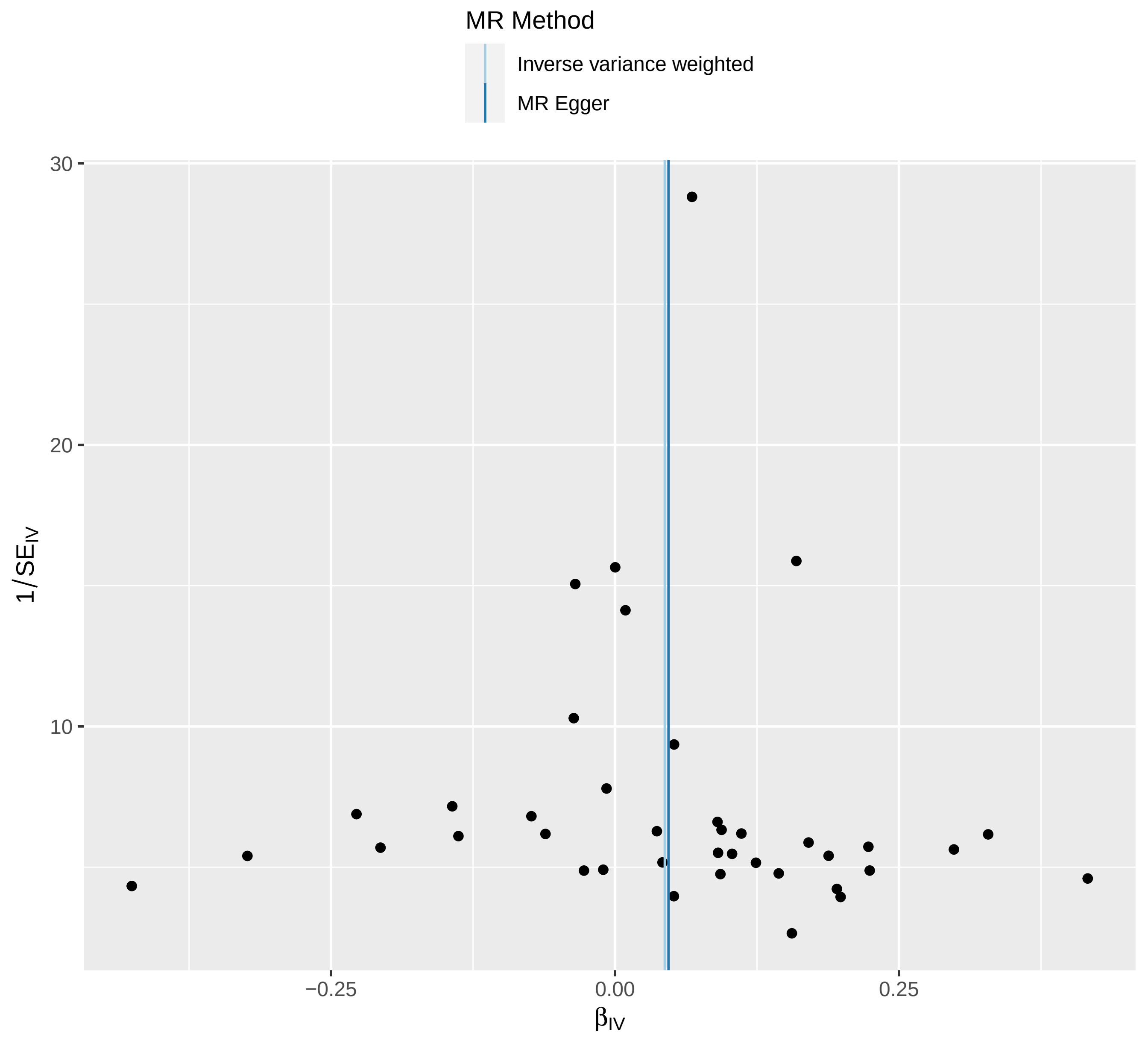
MR Method
Inverse variance weighted
MR Egger
30
20
10
$1/SE_{IV}$
−0.25
0.00
0.25
β_{IV}

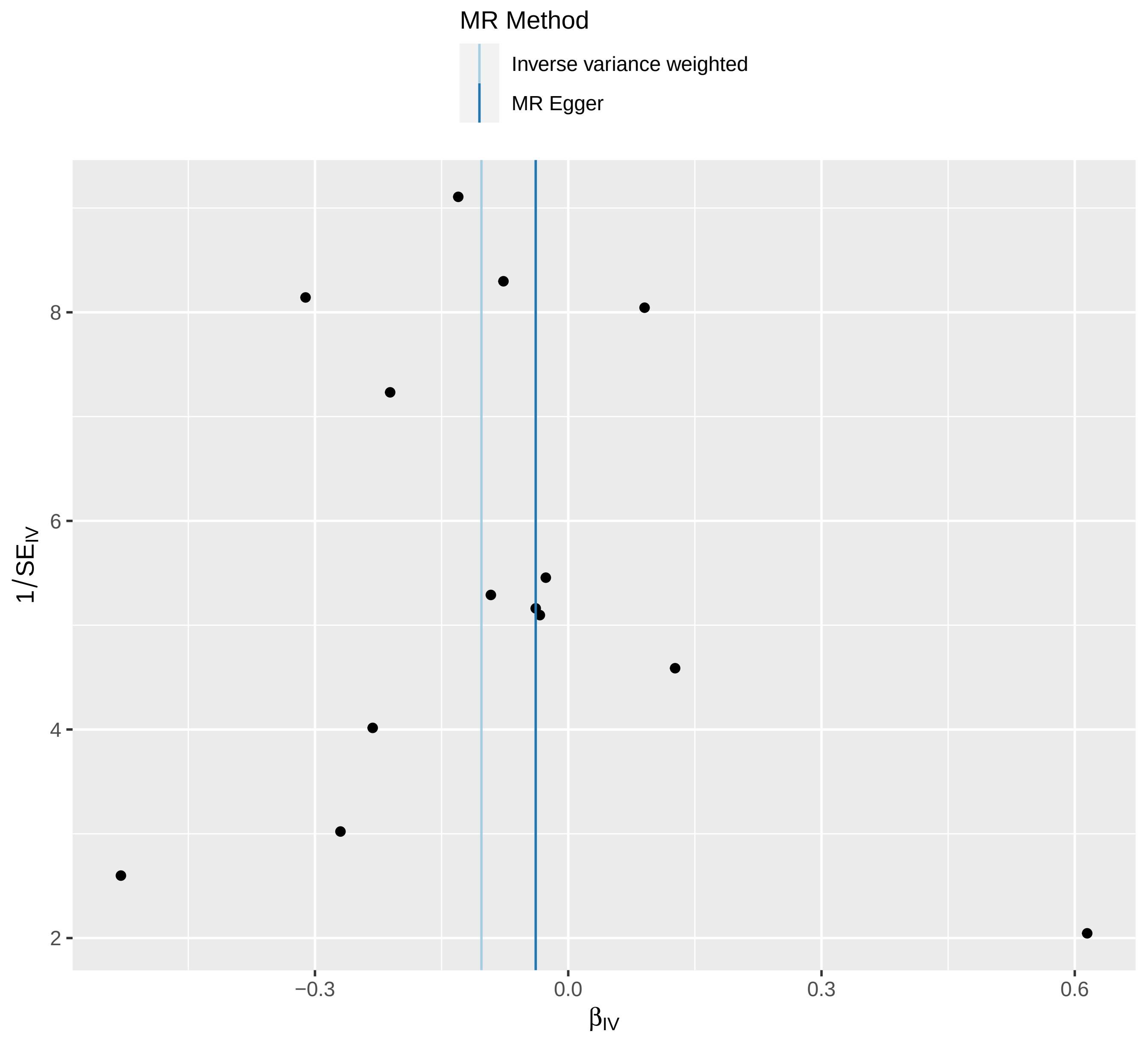
MR Method
Inverse variance weighted
MR Egger
1/SE_{IV}
8
6
4
2
−0.3
0.0
0.3
0.6
β_{IV}

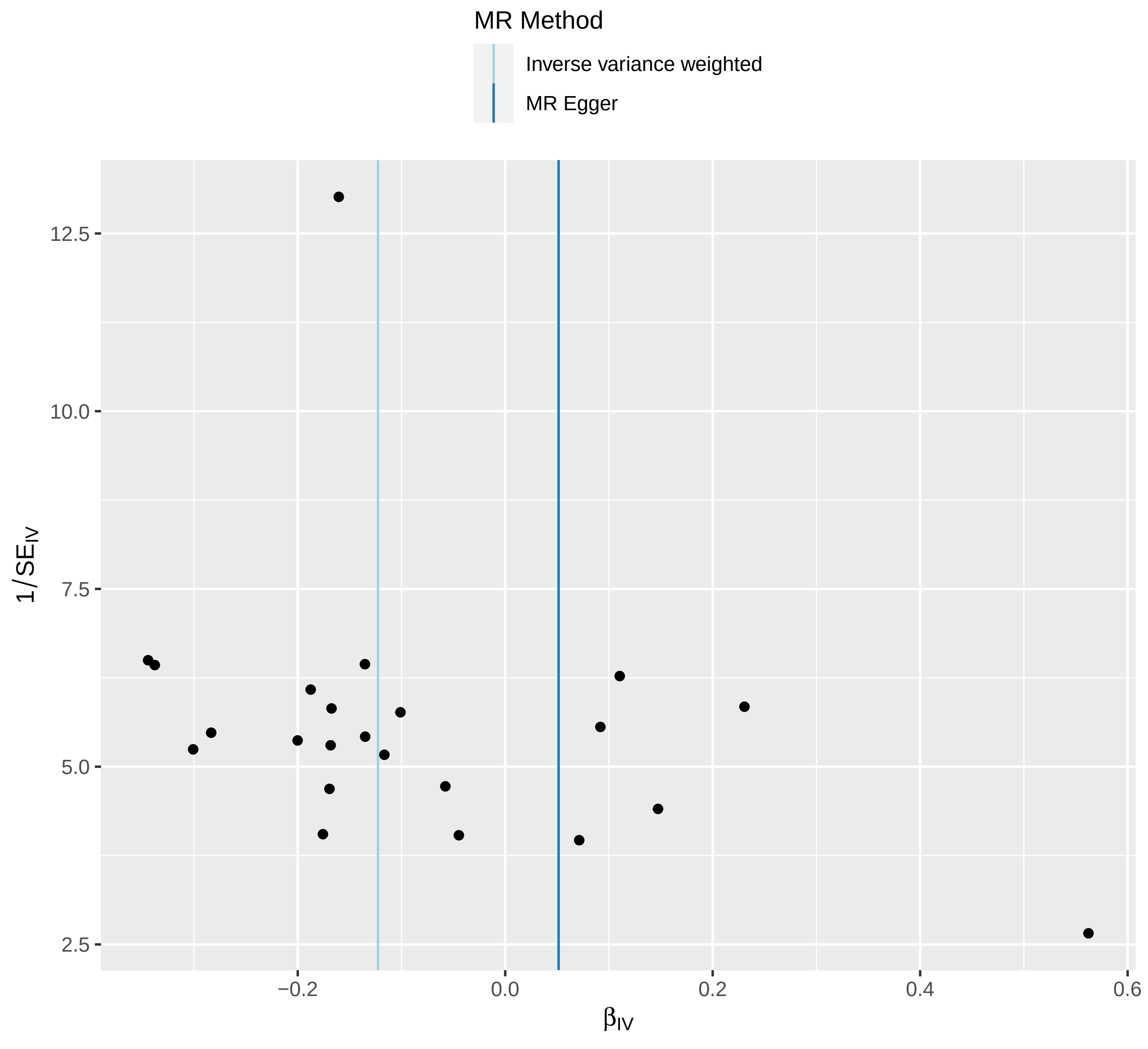

MR Method
Inverse variance weighted
MR Egger
12.5
10.0
7.5
5.0
2.5
1/SE_IV
−0.2
0.0
0.2
0.4
0.6
β_IV

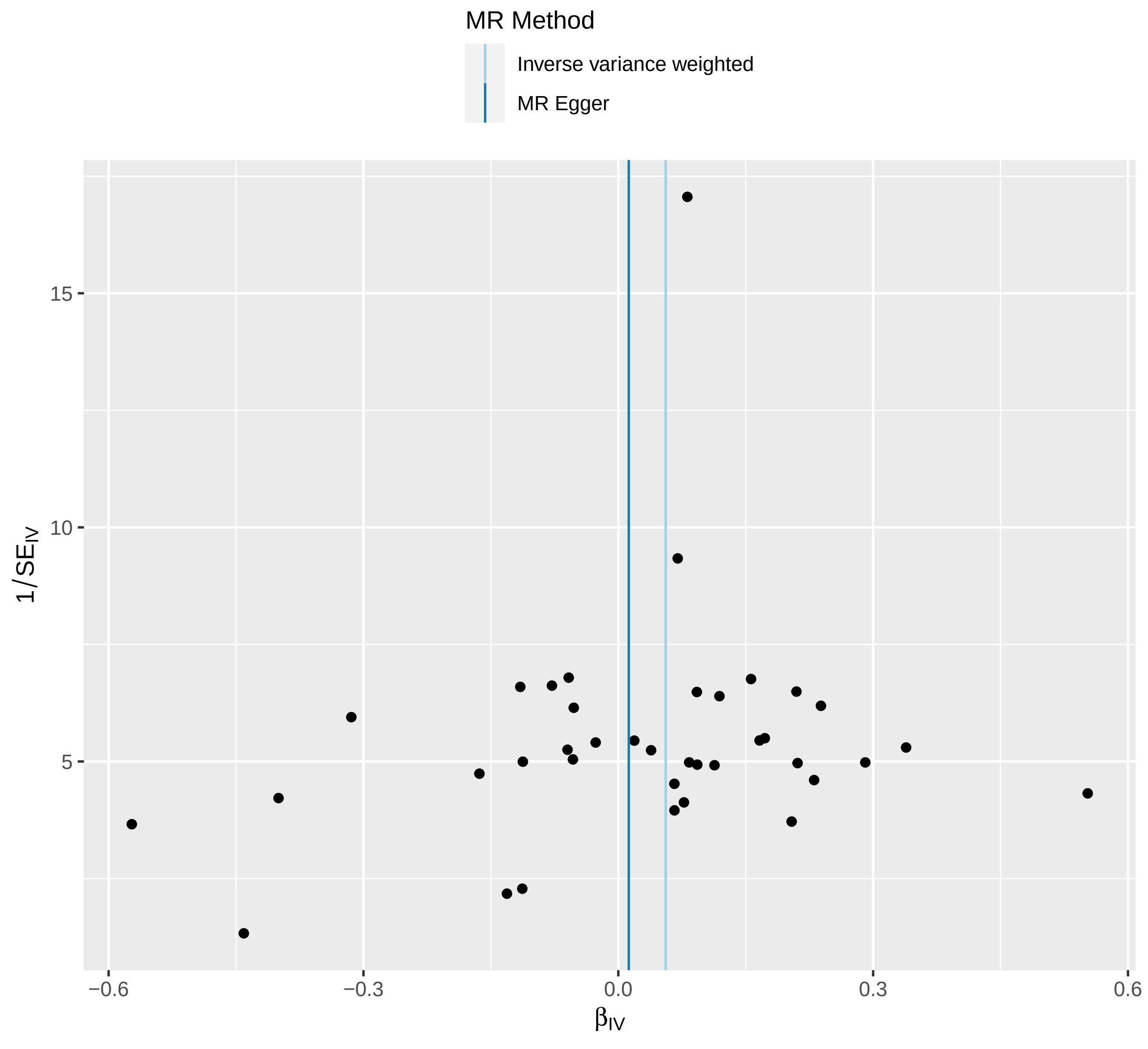

MR Method
Inverse variance weighted
MR Egger
15
10
5
1/SE_IV
−0.6
−0.3
0.0
0.3
0.6
β_IV

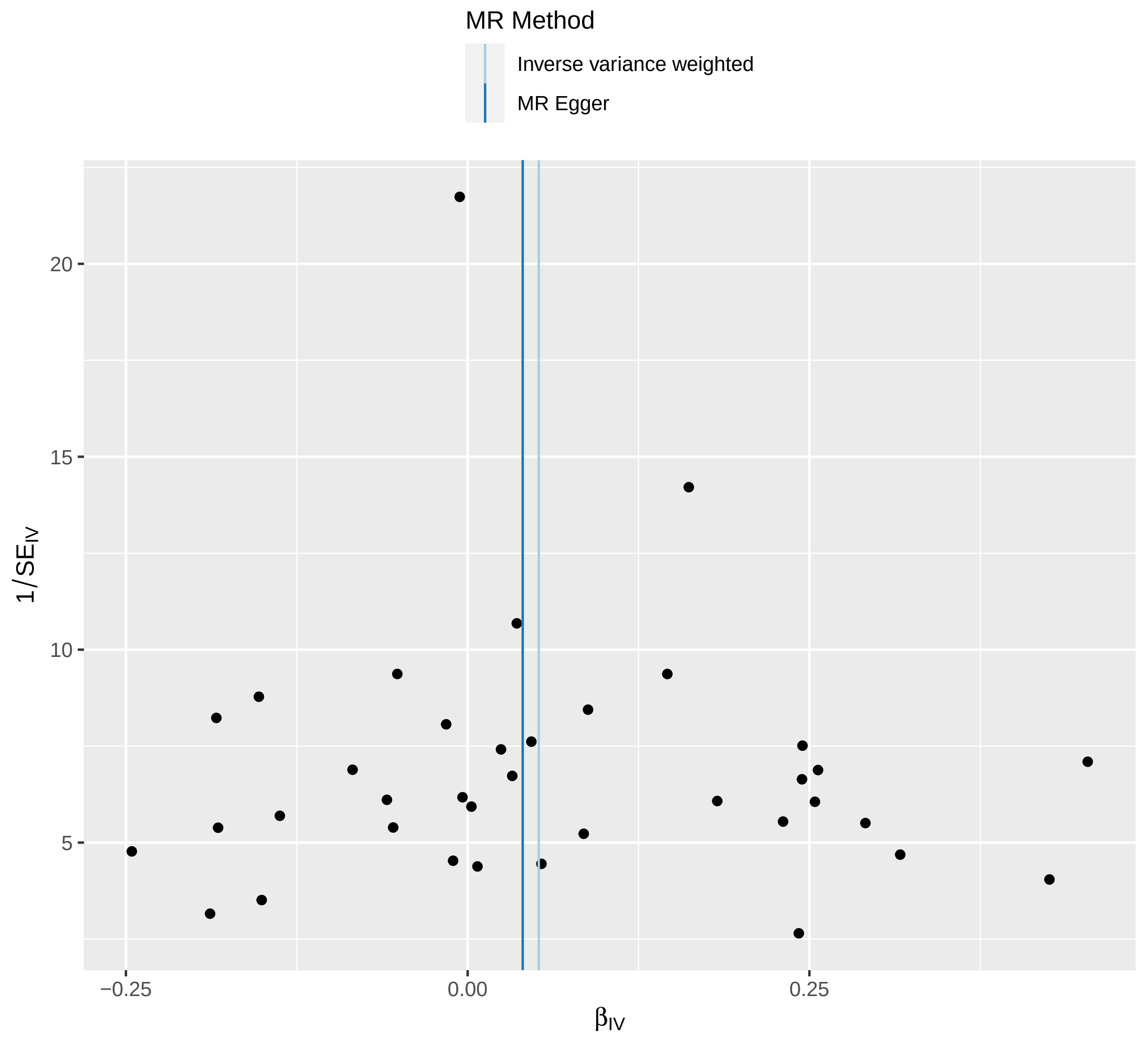
MR Method
Inverse variance weighted
MR Egger
20
15
10
5
−0.25
0.00
0.25
1/SE_IV
β_IV

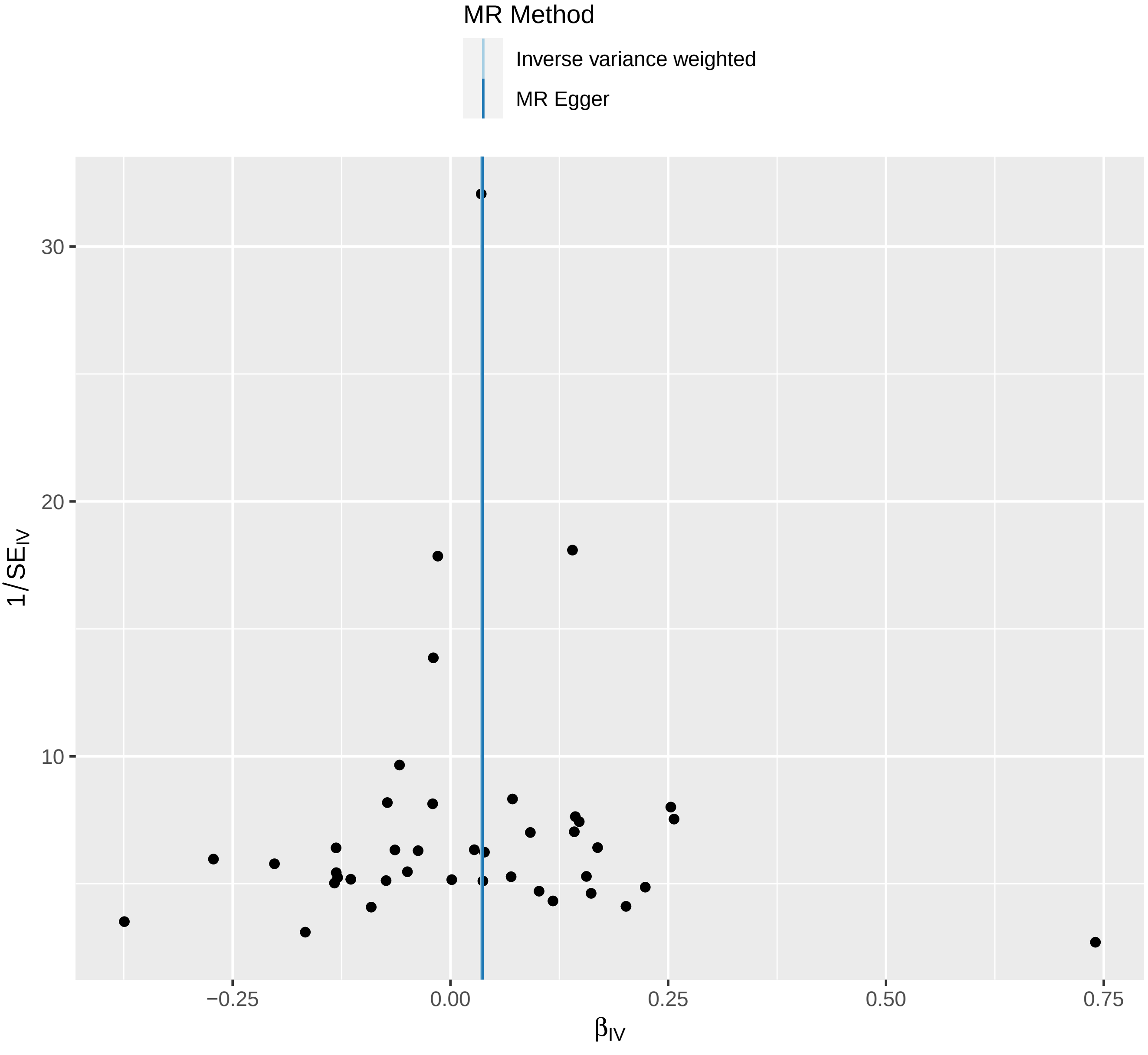

MR Method
Inverse variance weighted
MR Egger
30
20
10
1/SE_IV
−0.25
0.00
0.25
0.50
0.75
β_IV

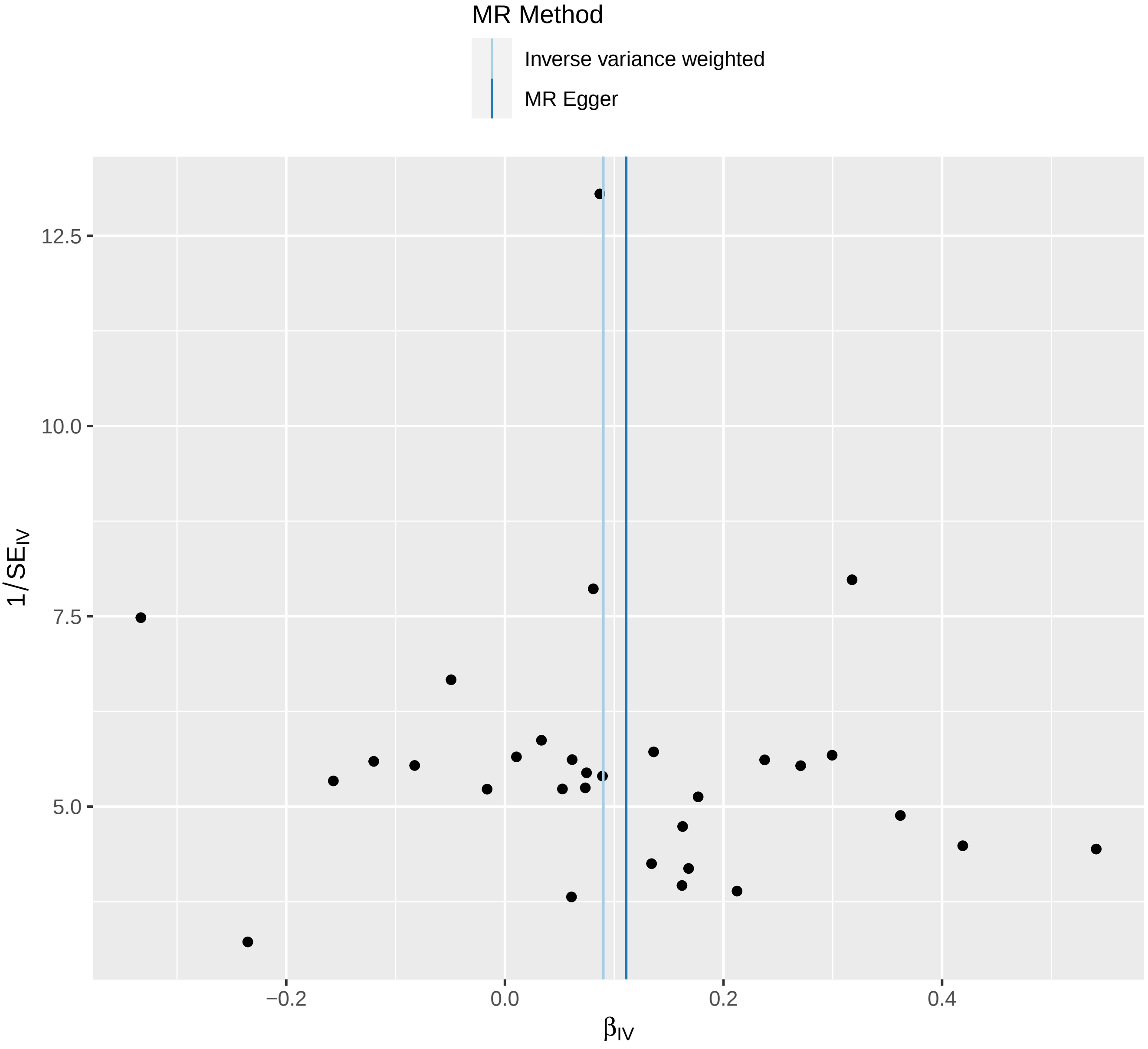
MR Method
Inverse variance weighted
MR Egger
12.5
10.0
7.5
5.0
−0.2
0.0
0.2
0.4
$1/SE_{IV}$
β_{IV}

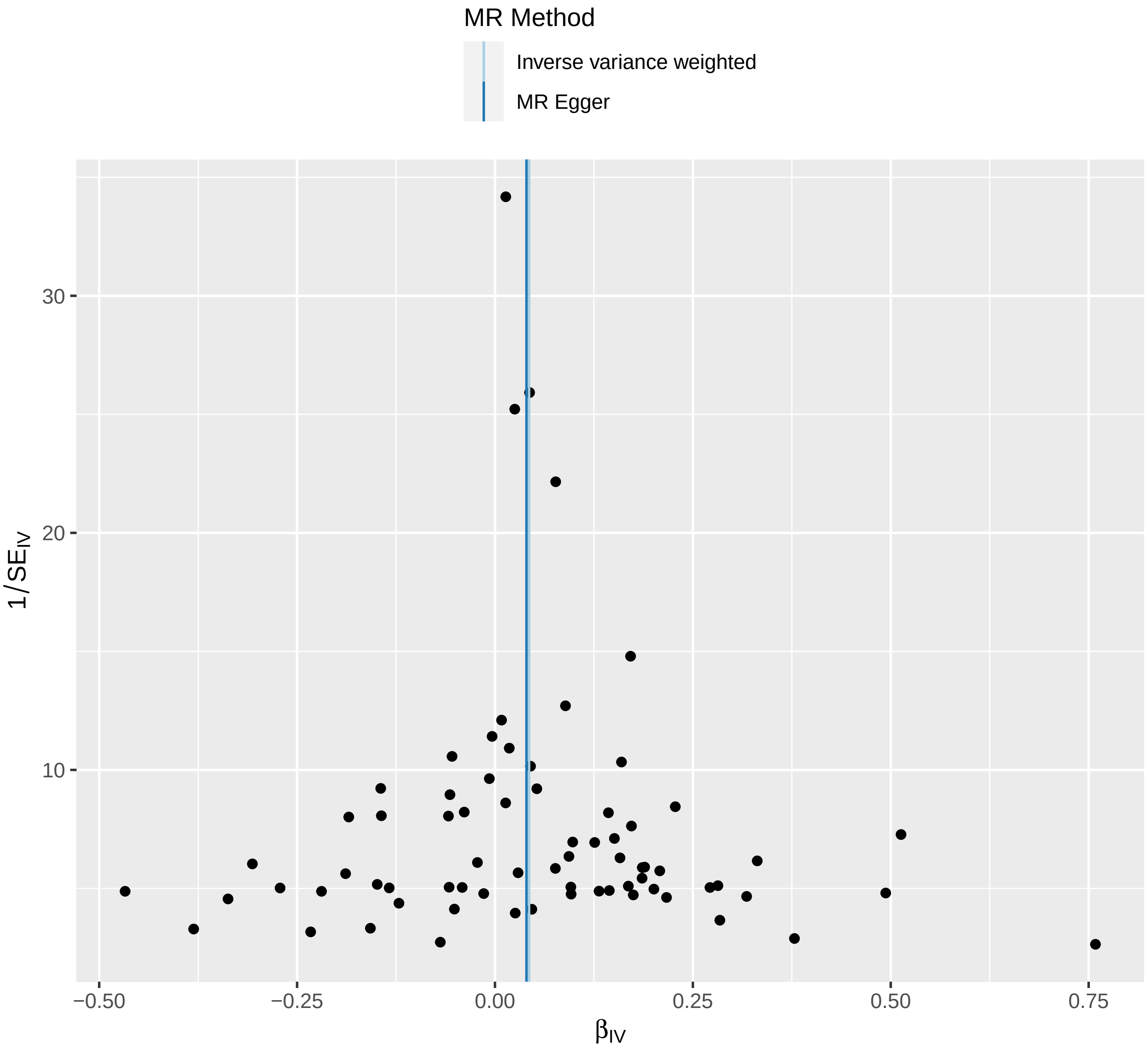

MR Method
Inverse variance weighted
MR Egger
30
20
10
1/SE$_{IV}$
−0.50
−0.25
0.00
0.25
0.50
0.75
β$_{IV}$

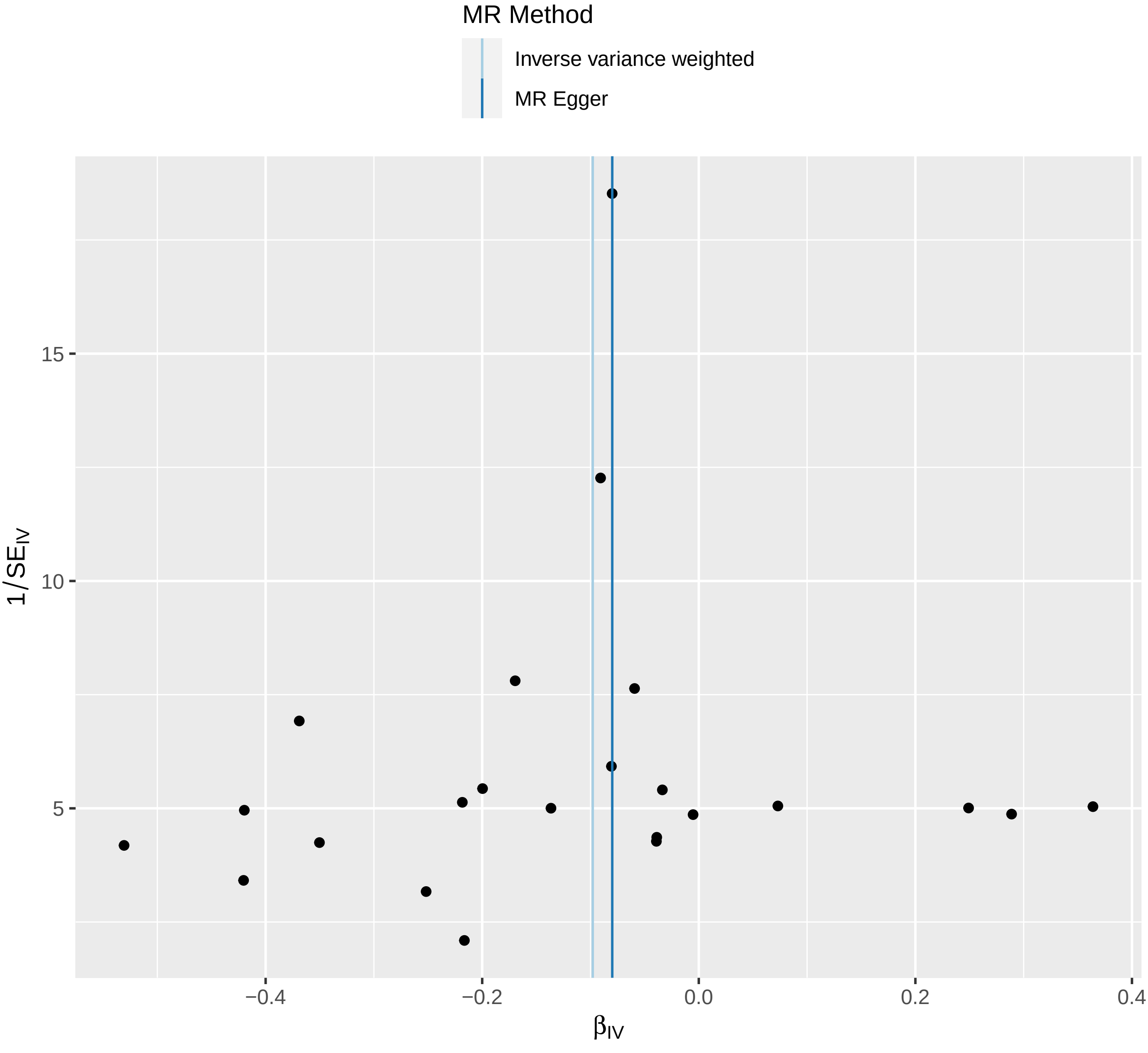
MR Method
Inverse variance weighted
MR Egger
15
10
5
$1/SE_{IV}$
−0.4
−0.2
0.0
0.2
0.4
β_{IV}

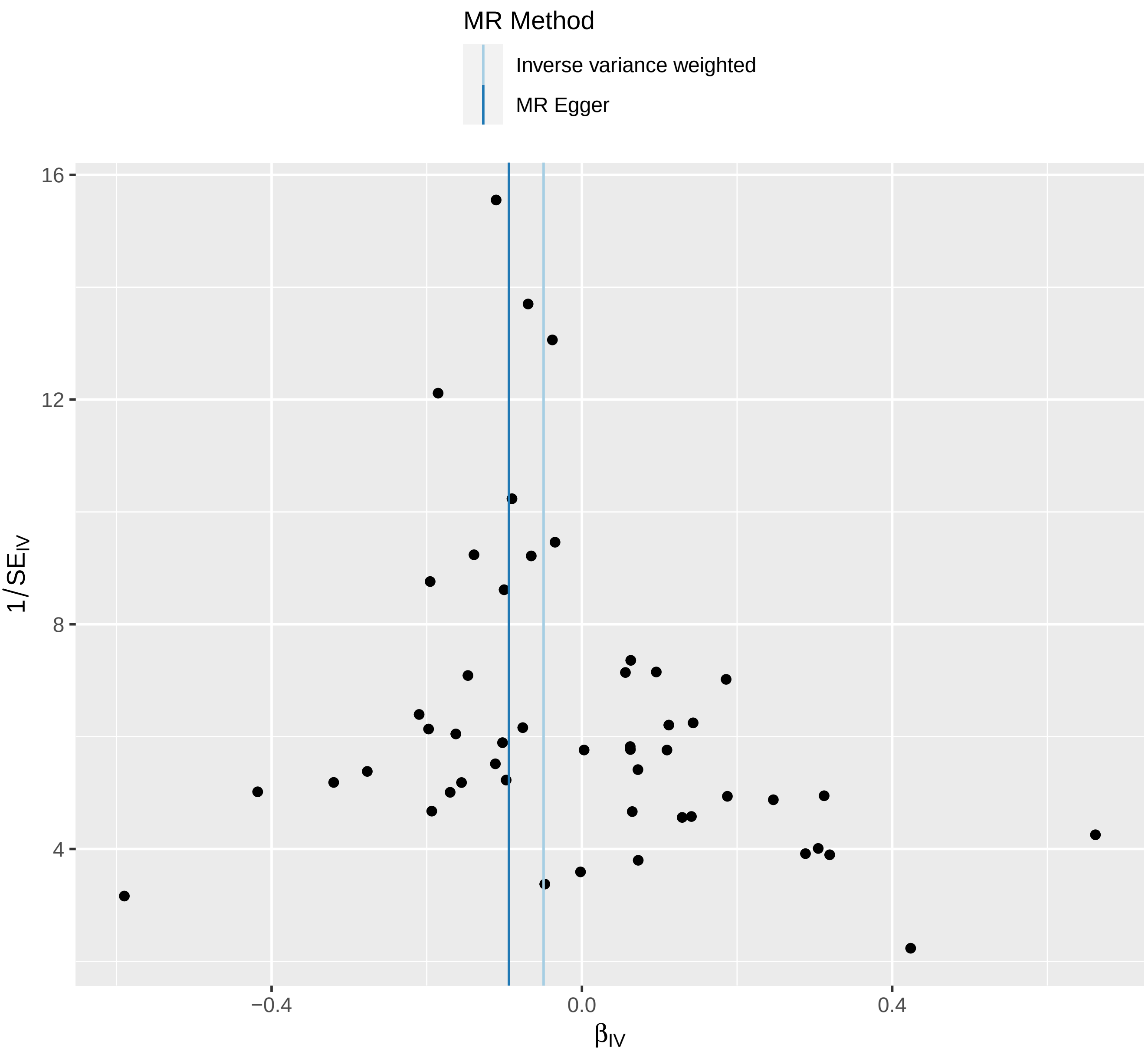
MR Method
Inverse variance weighted
MR Egger
16
12
8
4
−0.4
0.0
0.4
1/SE_IV
β_IV

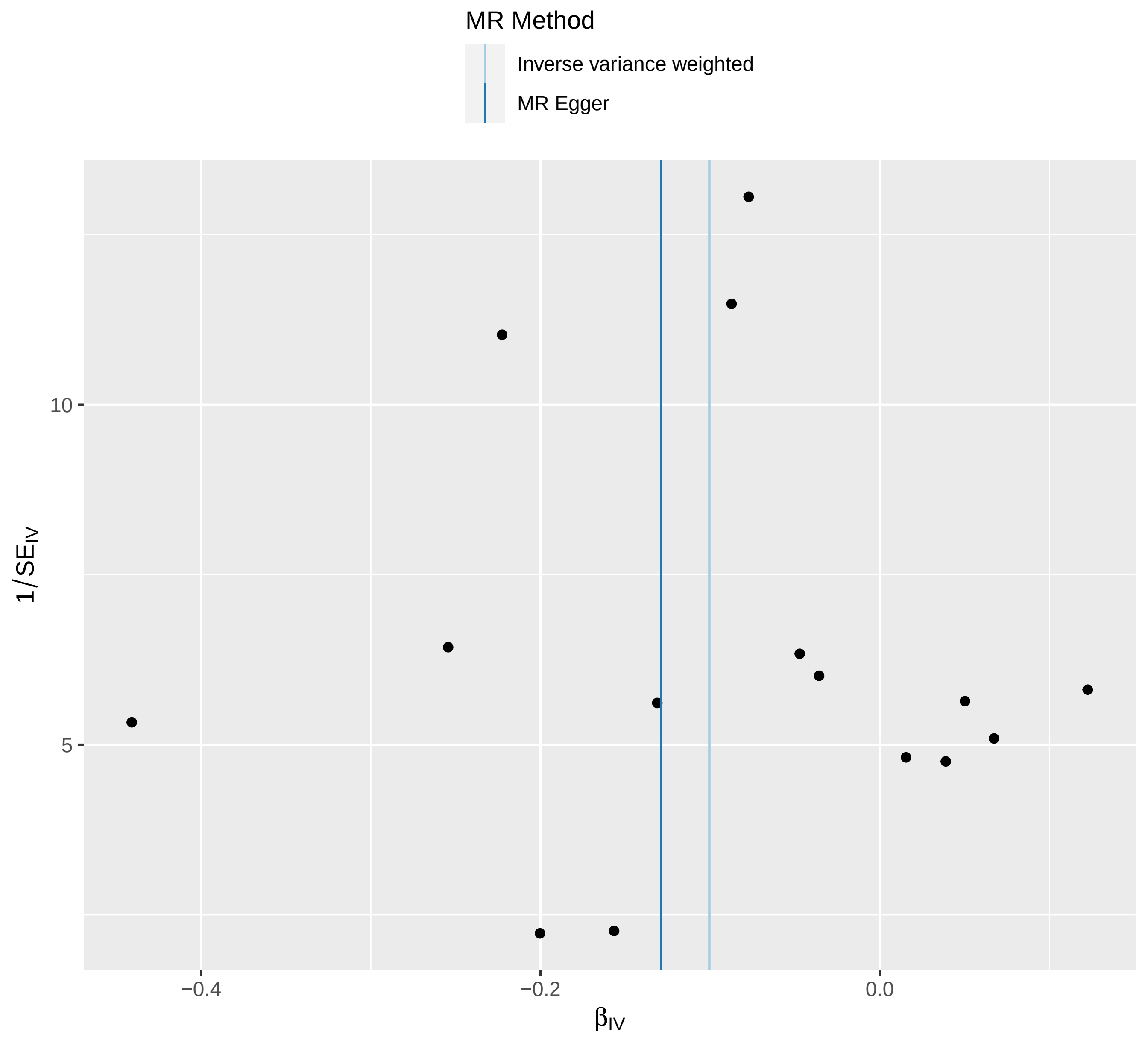

MR Method
Inverse variance weighted
MR Egger
10
5
$1/SE_{IV}$
−0.4
−0.2
0.0
β_{IV}

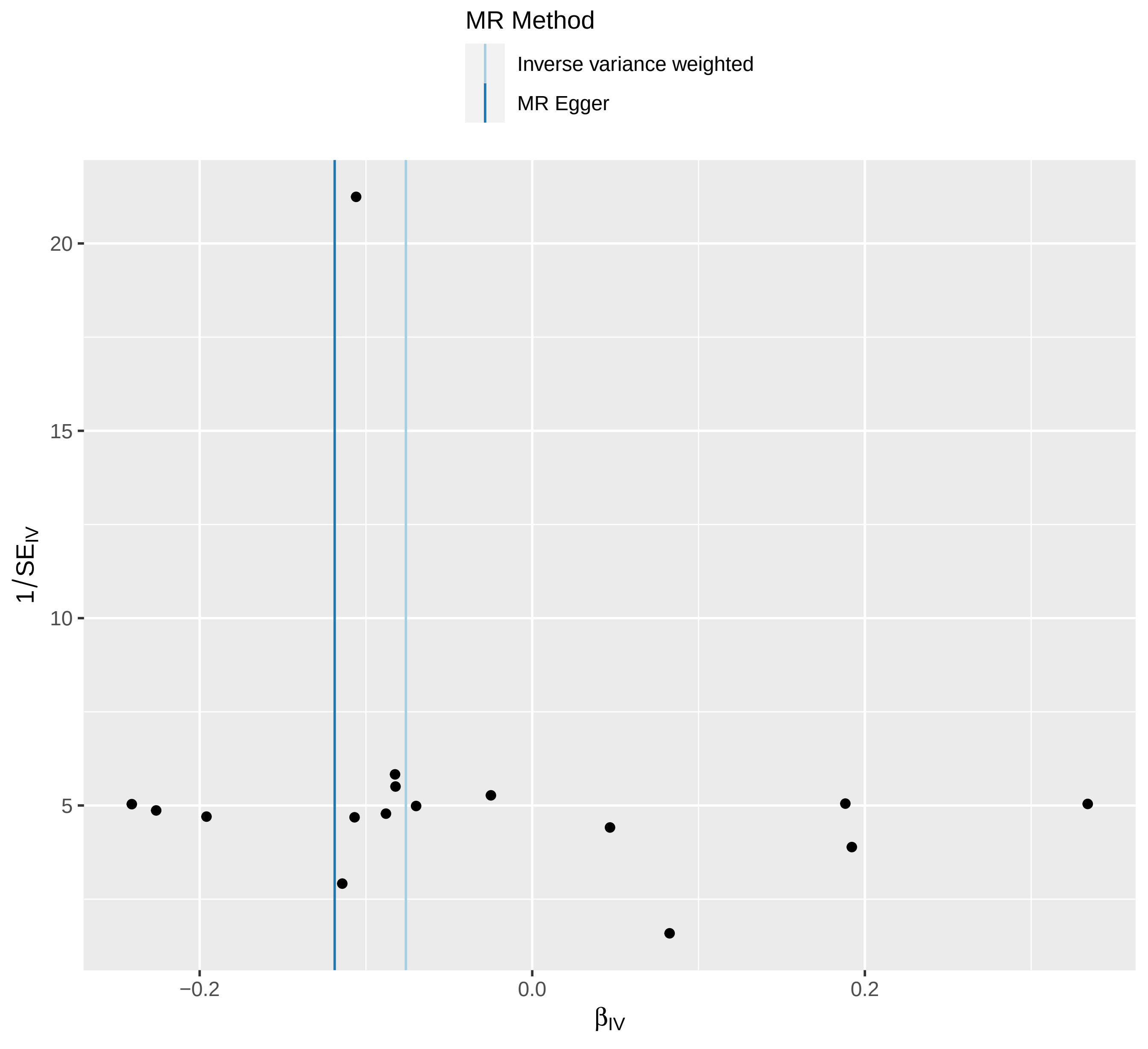
MR Method
Inverse variance weighted
MR Egger
20
15
10
5
1/SE_IV
−0.2
0.0
0.2
β_IV

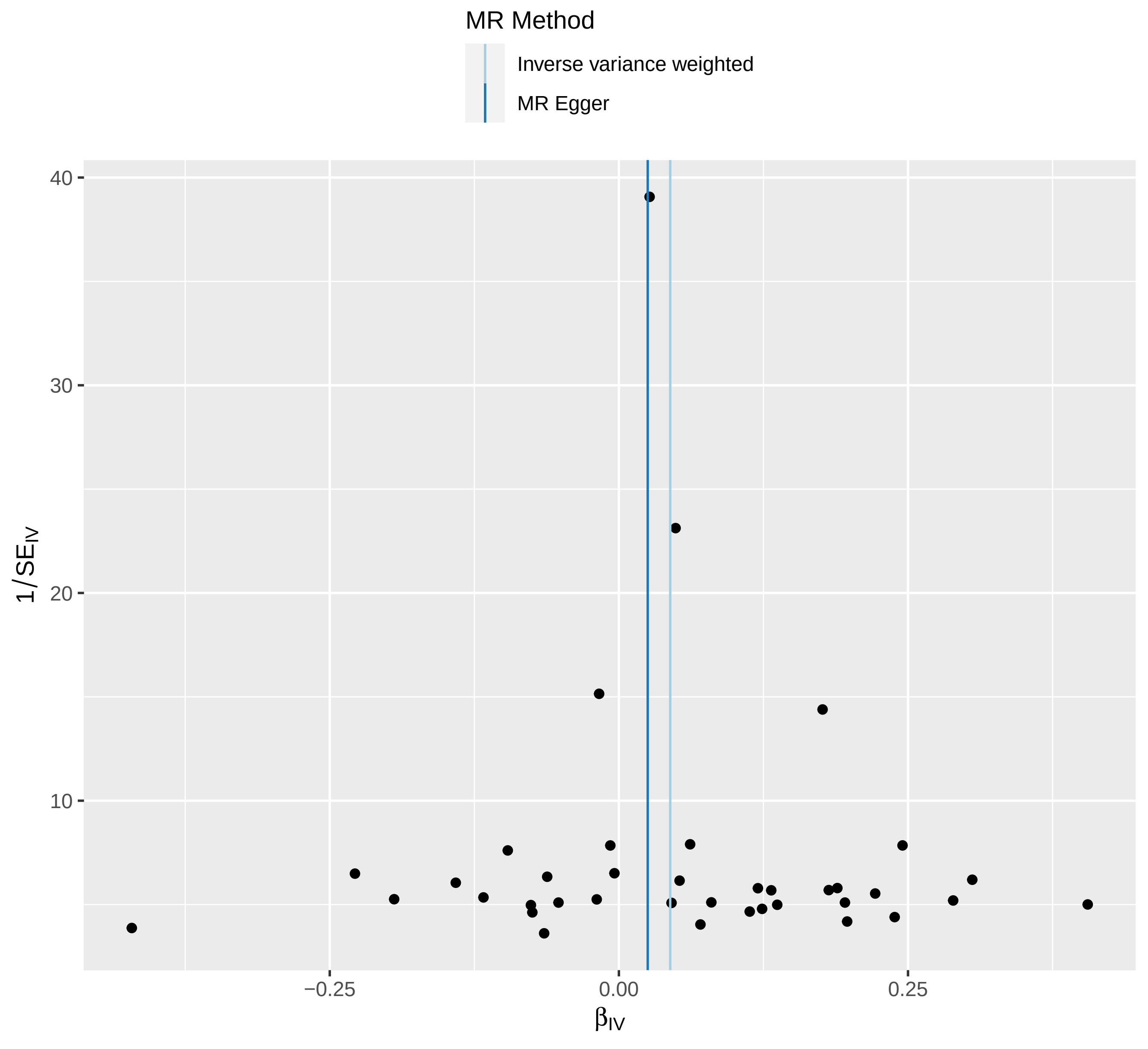

MR Method
Inverse variance weighted
MR Egger
40
30
20
10
$1/SE_{IV}$
−0.25
0.00
0.25
β_{IV}

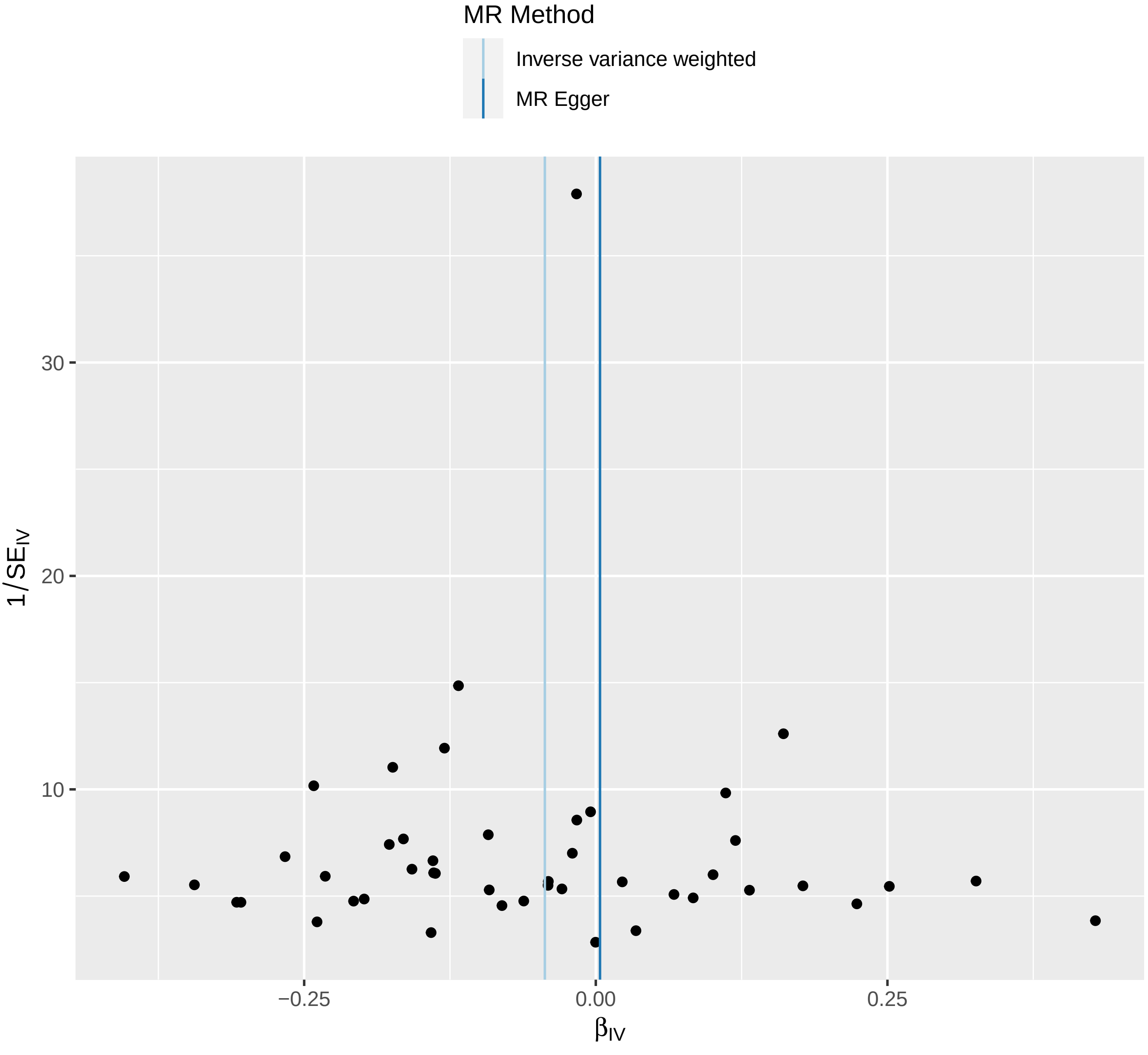

MR Method
Inverse variance weighted
MR Egger
30
20
10
−0.25
0.00
0.25
$1/SE_{IV}$
β_{IV}

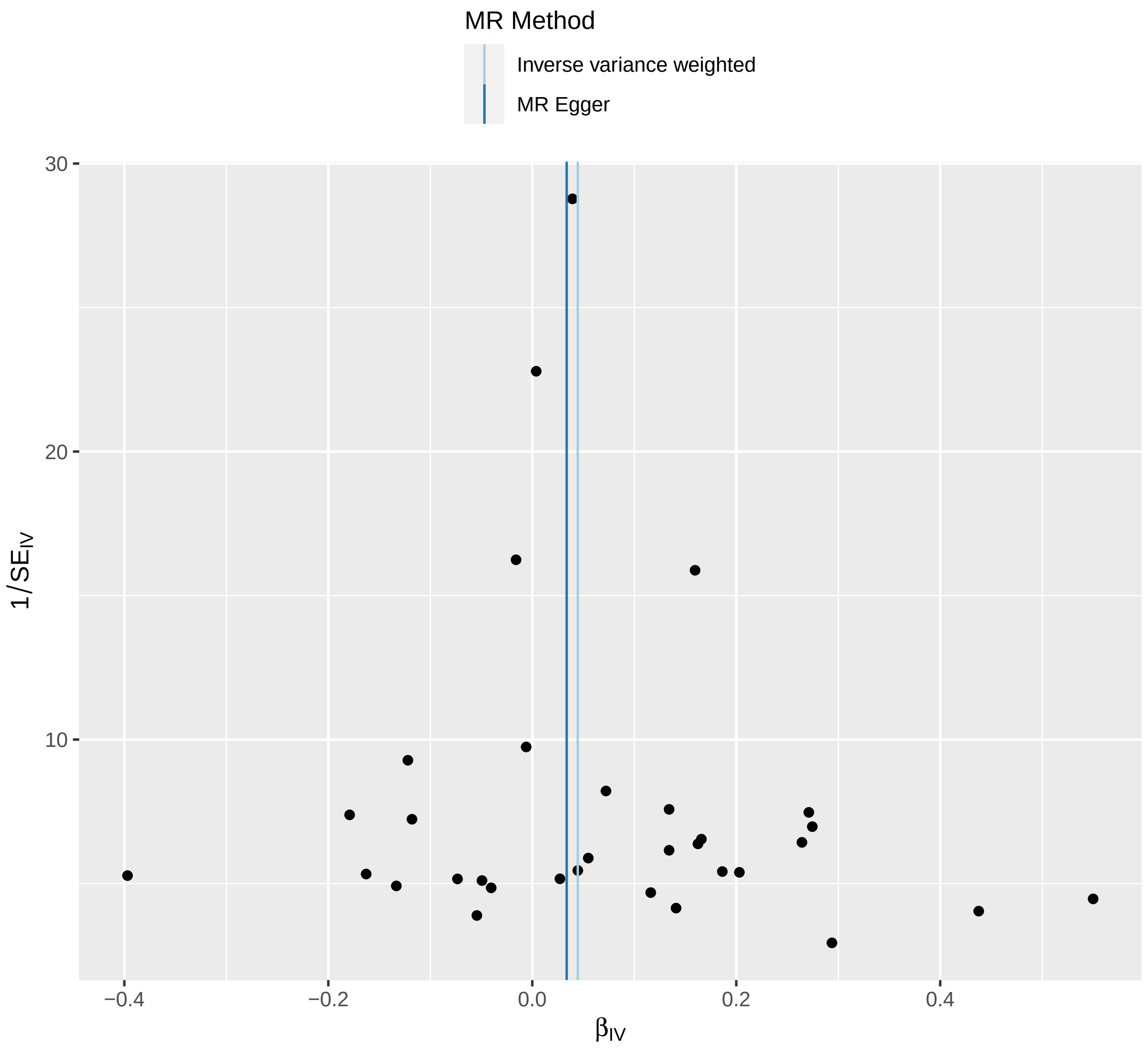
MR Method
Inverse variance weighted
MR Egger
30
20
10
1/SE_IV
−0.4
−0.2
0.0
0.2
0.4
β_IV

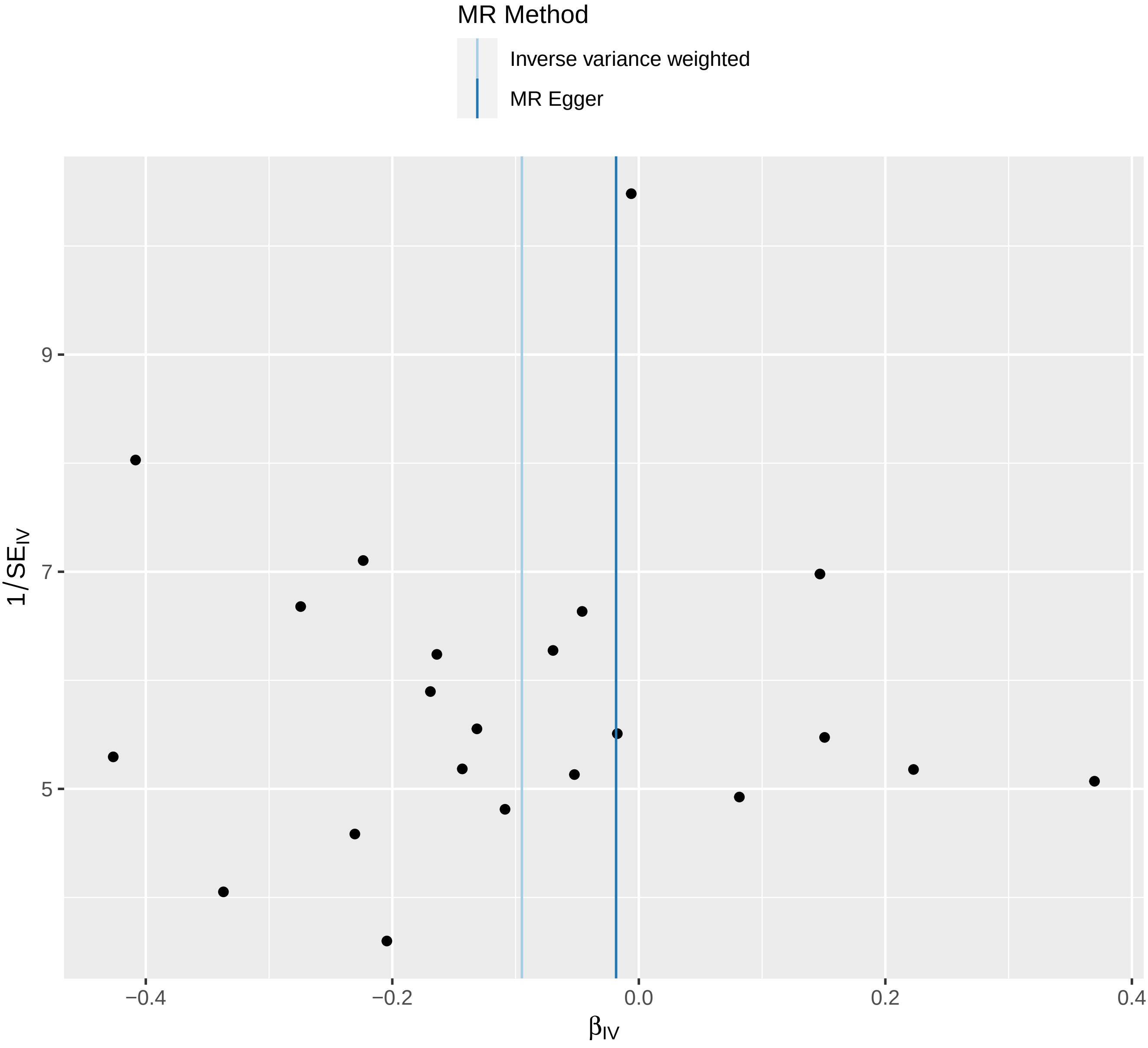

MR Method
Inverse variance weighted
MR Egger
9
7
5
1/SE_IV
−0.4
−0.2
0.0
0.2
0.4
β_IV

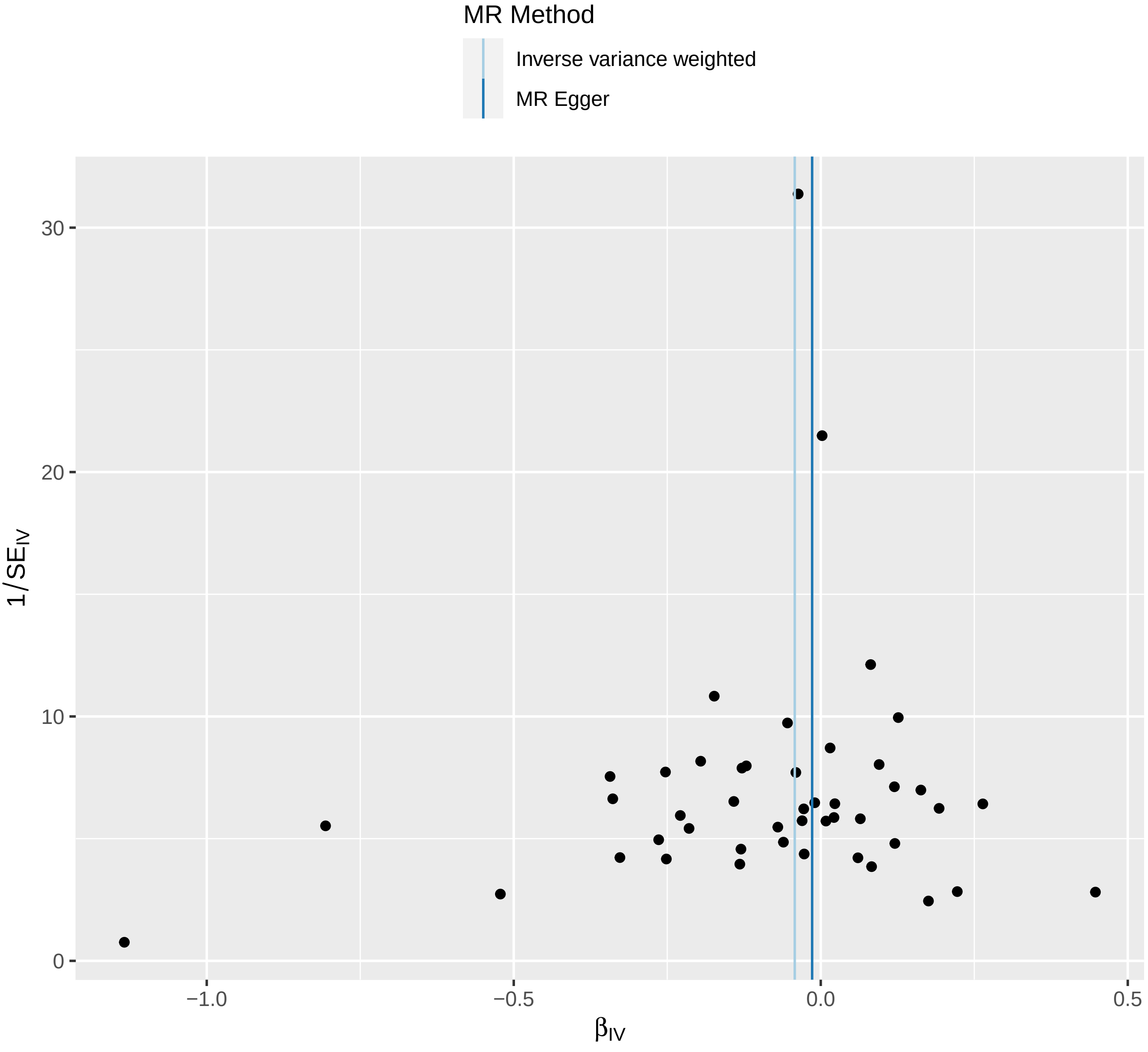
MR Method
Inverse variance weighted
MR Egger
30
20
10
0
$1/SE_{IV}$
−1.0
−0.5
0.0
0.5
β_{IV}

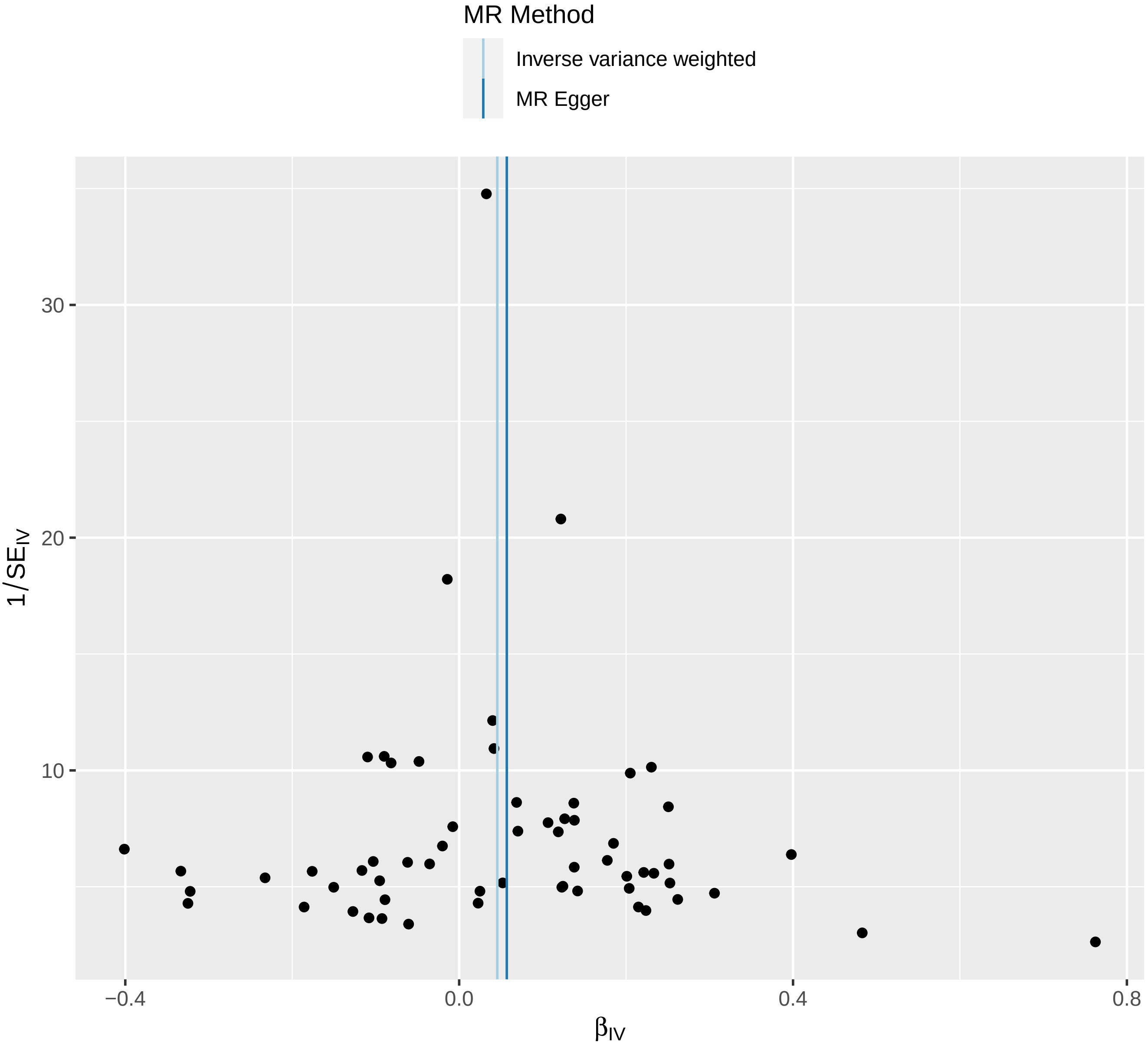
MR Method
Inverse variance weighted
MR Egger
30
20
10
1/SE$_{IV}$
−0.4
0.0
0.4
0.8
β$_{IV}$

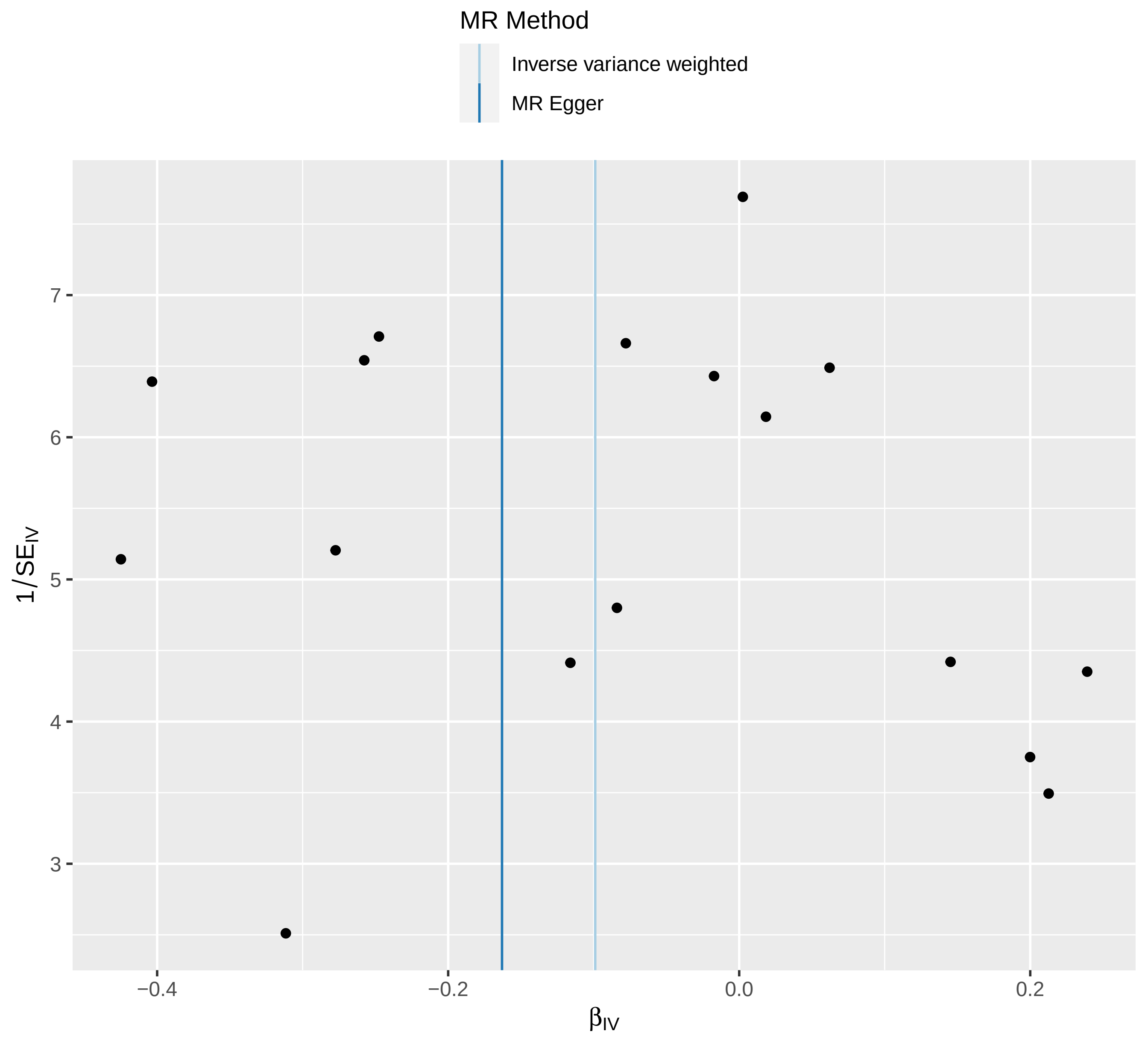
MR Method
Inverse variance weighted
MR Egger
7
6
5
4
3
$1/SE_{IV}$
−0.4
−0.2
0.0
0.2
β_{IV}

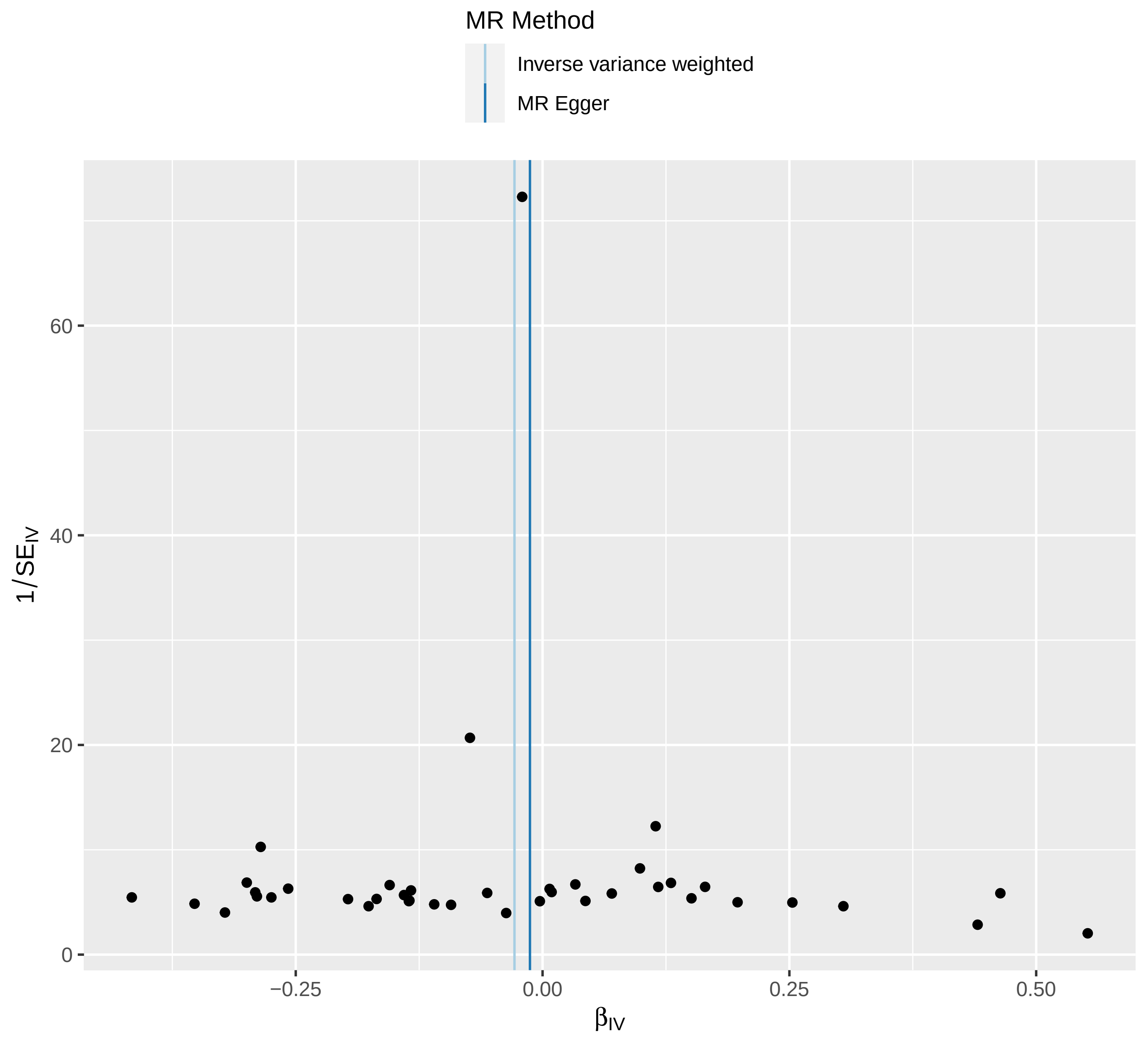

MR Method
Inverse variance weighted
MR Egger
1/SE_IV
60
40
20
0
−0.25
0.00
0.25
0.50
β_IV

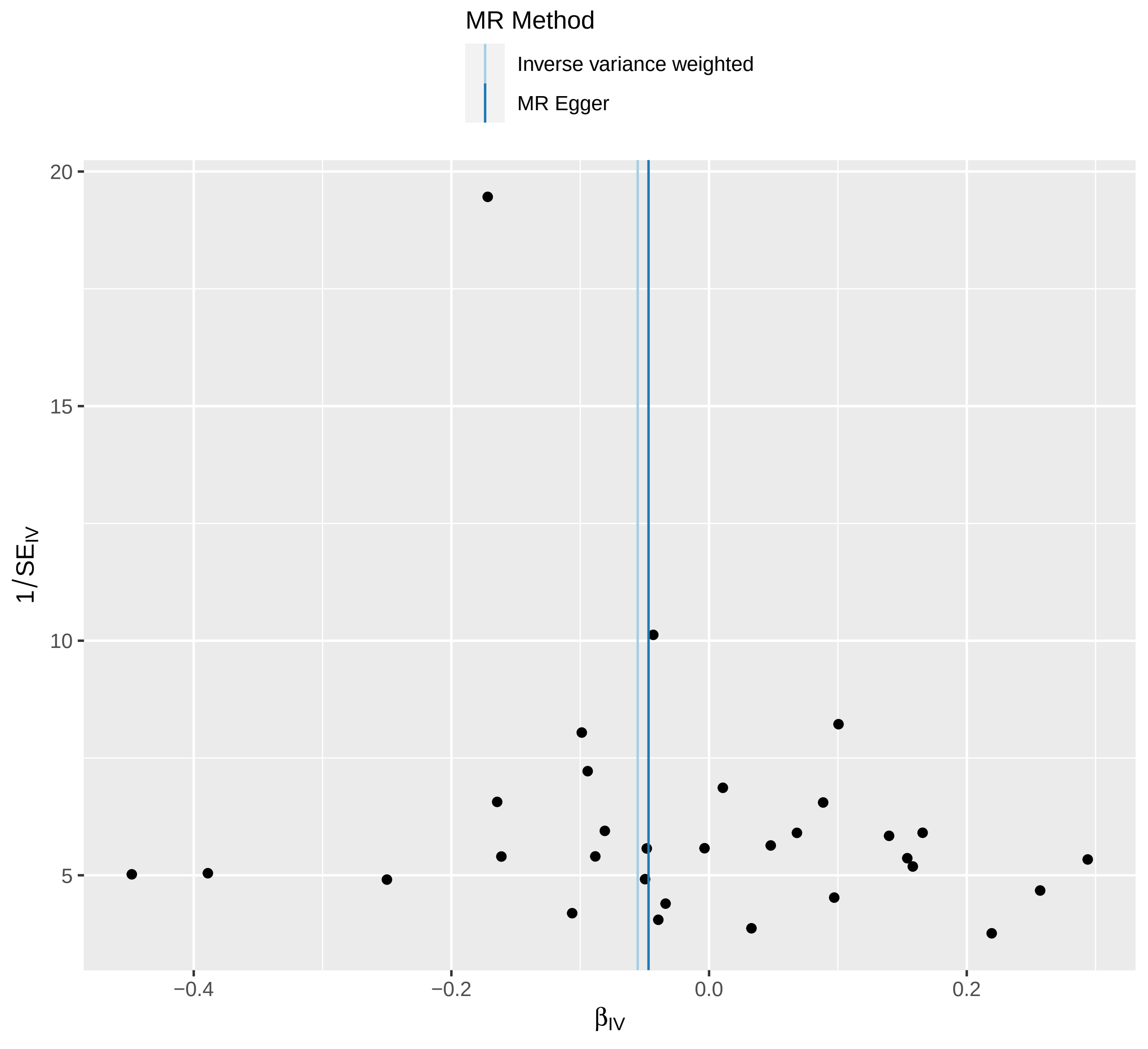

MR Method
Inverse variance weighted
MR Egger
1/SE_IV
20
15
10
5
−0.4
−0.2
0.0
0.2
β_IV

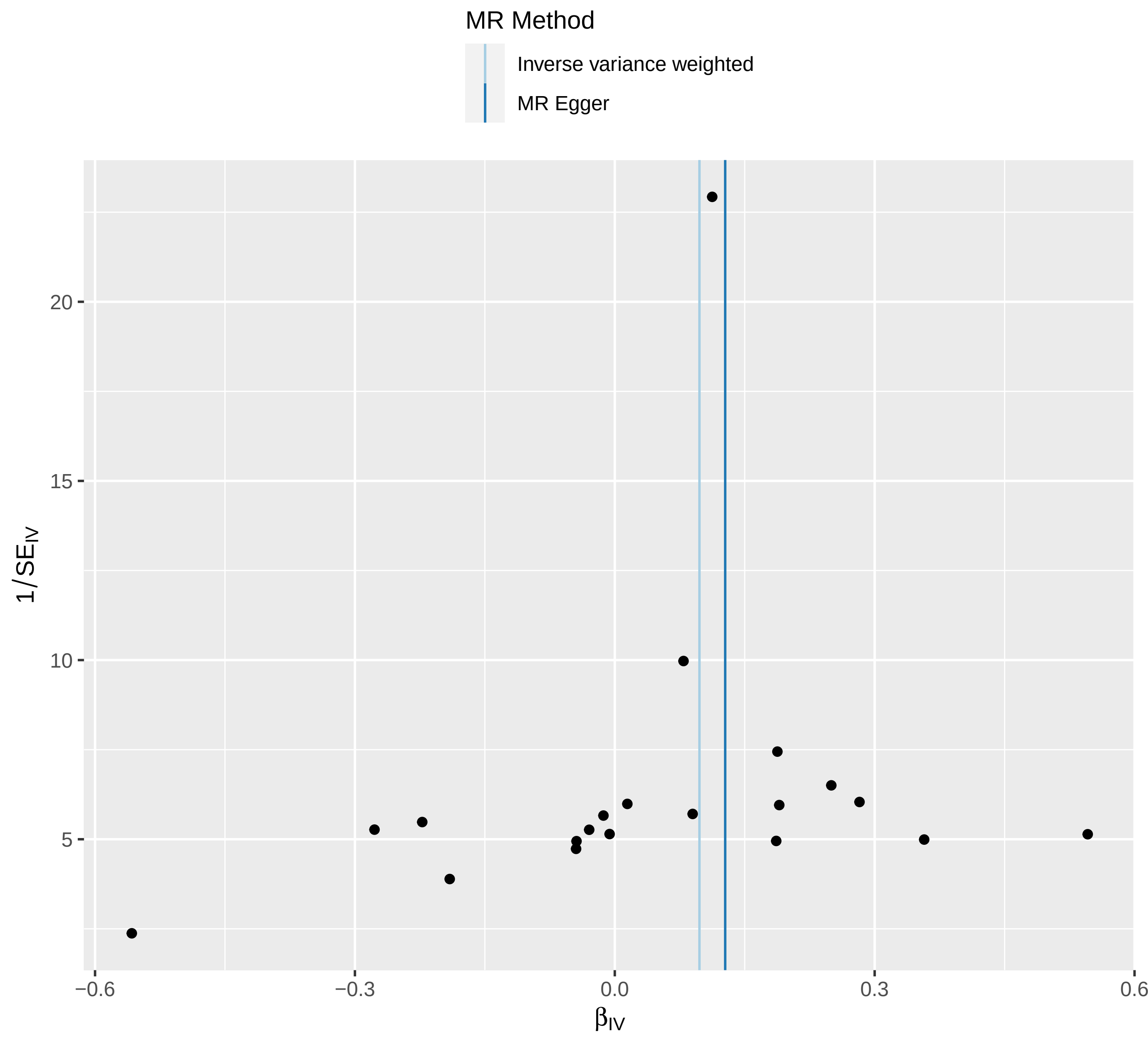
MR Method
Inverse variance weighted
MR Egger
20
15
10
5
−0.6
−0.3
0.0
0.3
0.6
$1/SE_{IV}$
β_{IV}

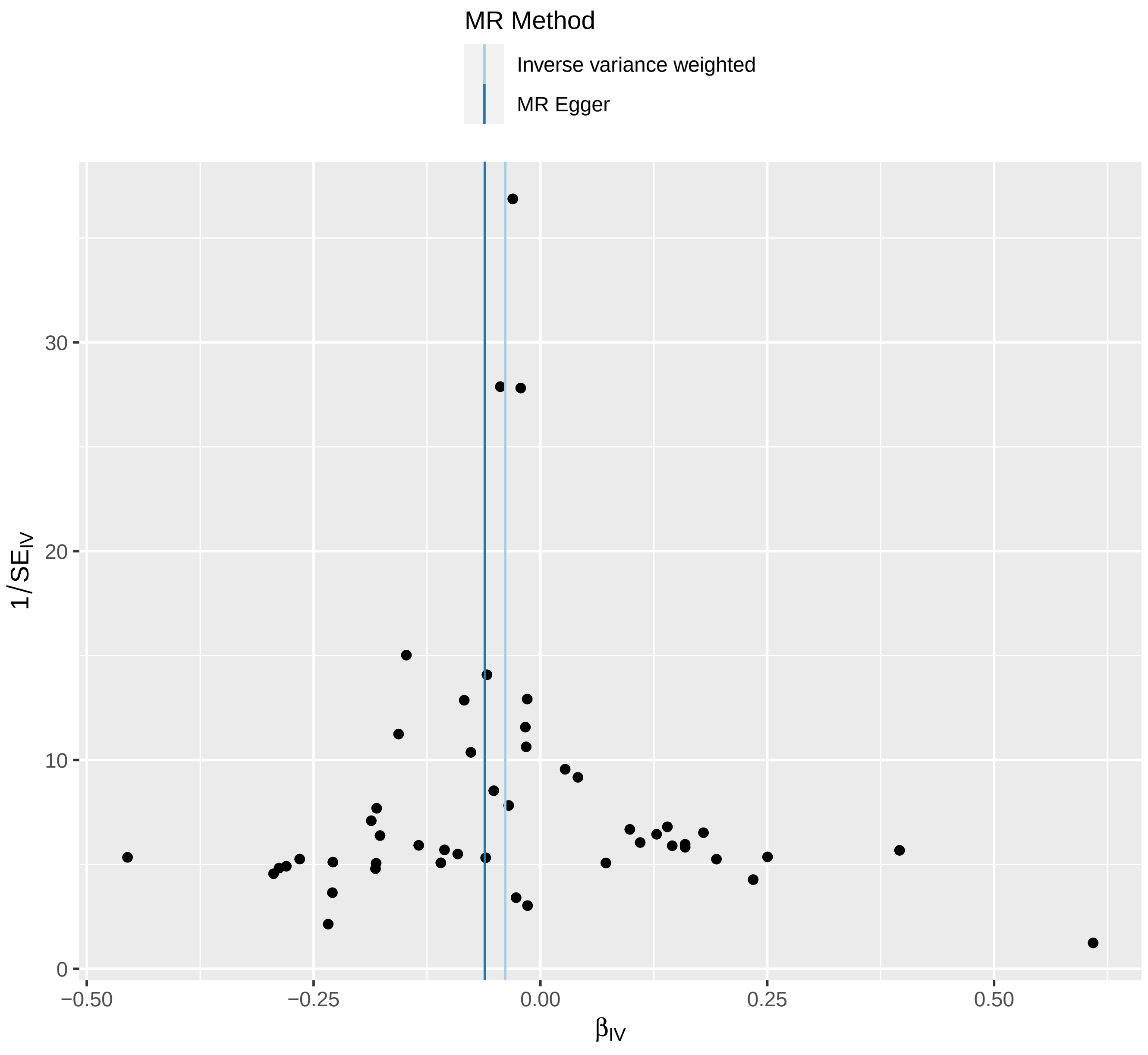

MR Method
Inverse variance weighted
MR Egger
30
20
10
0
−0.50
−0.25
0.00
0.25
0.50
1/SE_IV
β_IV

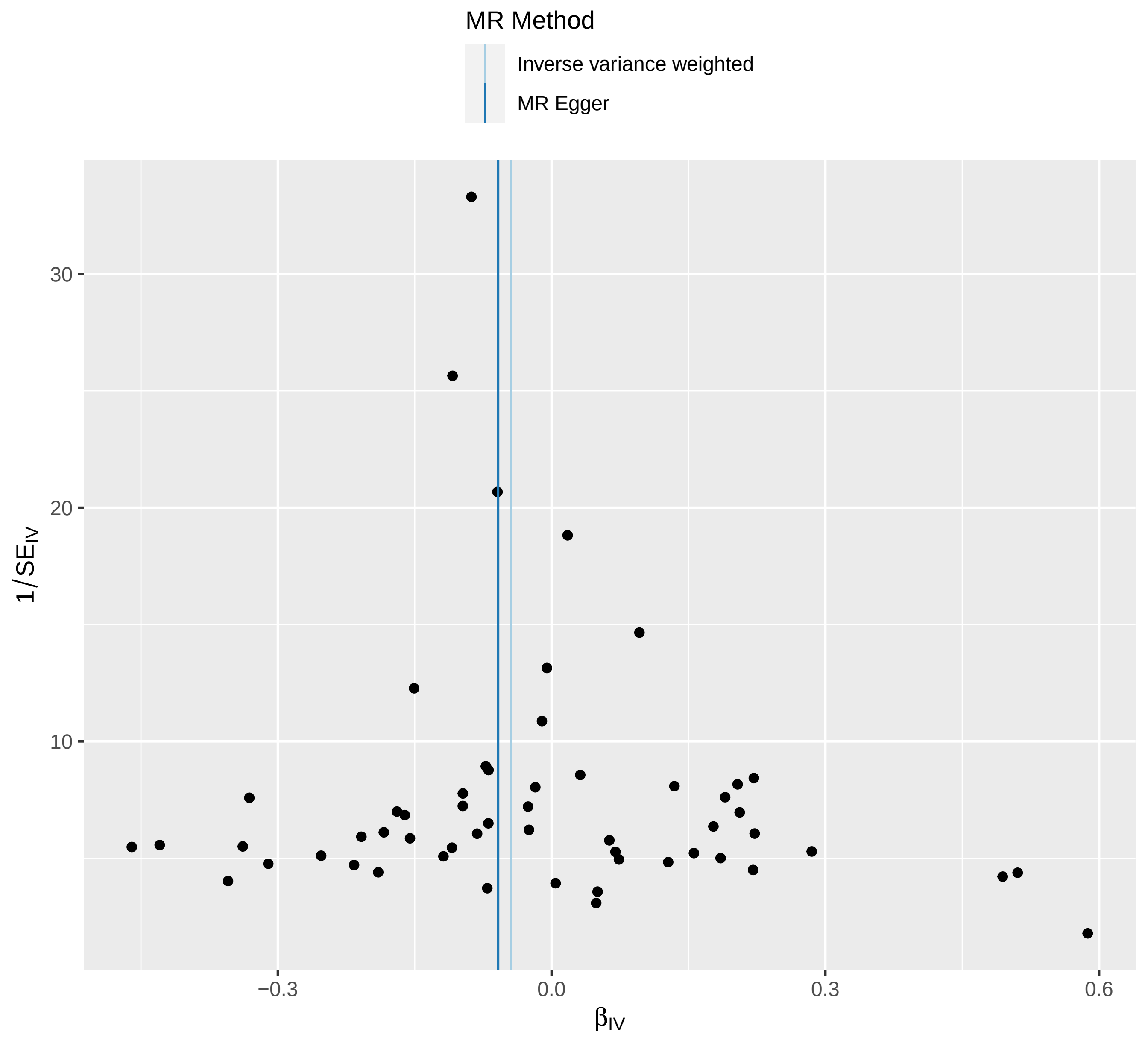
MR Method
Inverse variance weighted
MR Egger
30
20
10
−0.3
0.0
0.3
0.6
$1/SE_{IV}$
β_{IV}

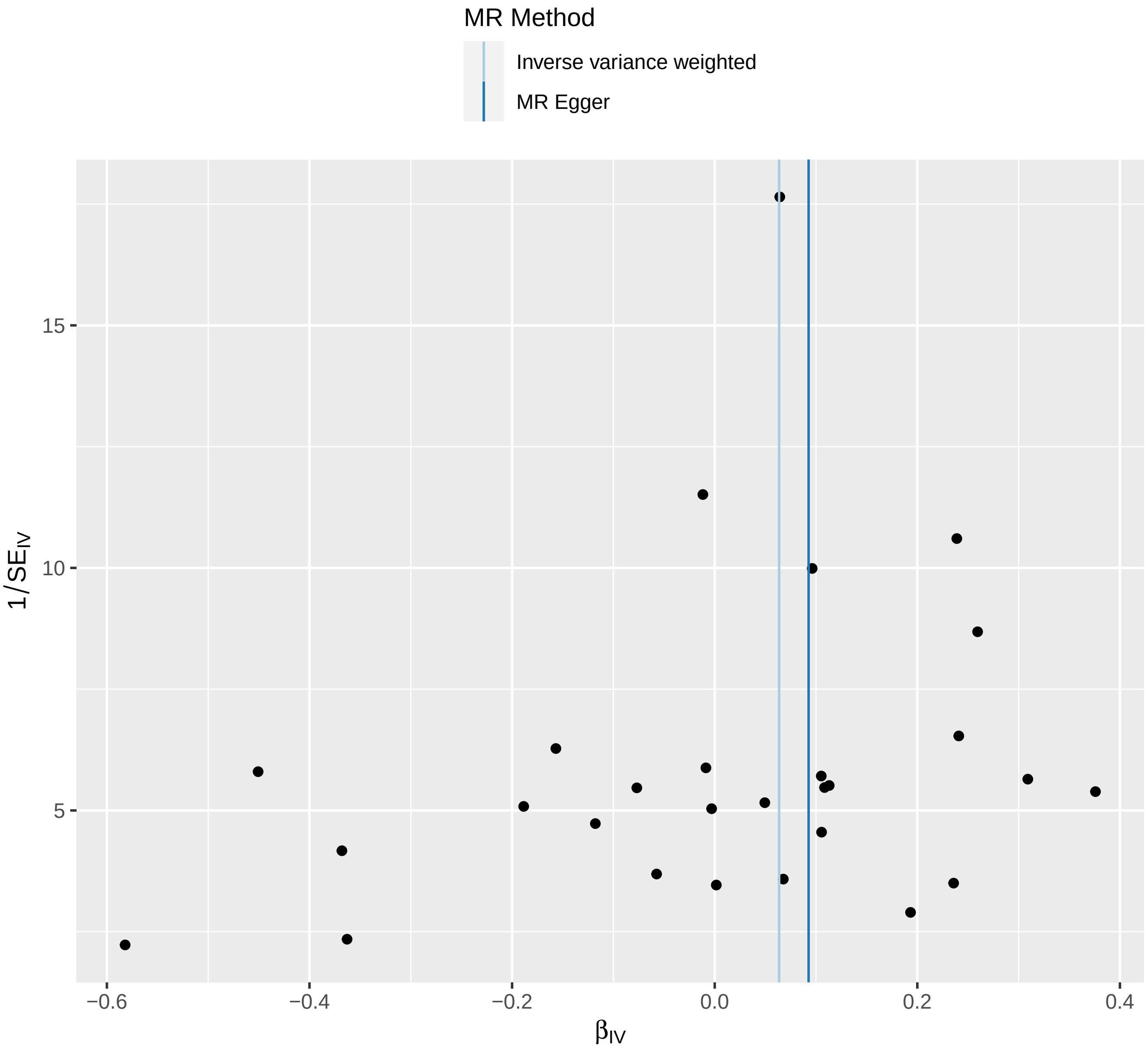
MR Method
Inverse variance weighted
MR Egger
1/SE_IV
15
10
5
−0.6
−0.4
−0.2
0.0
0.2
0.4
β_IV

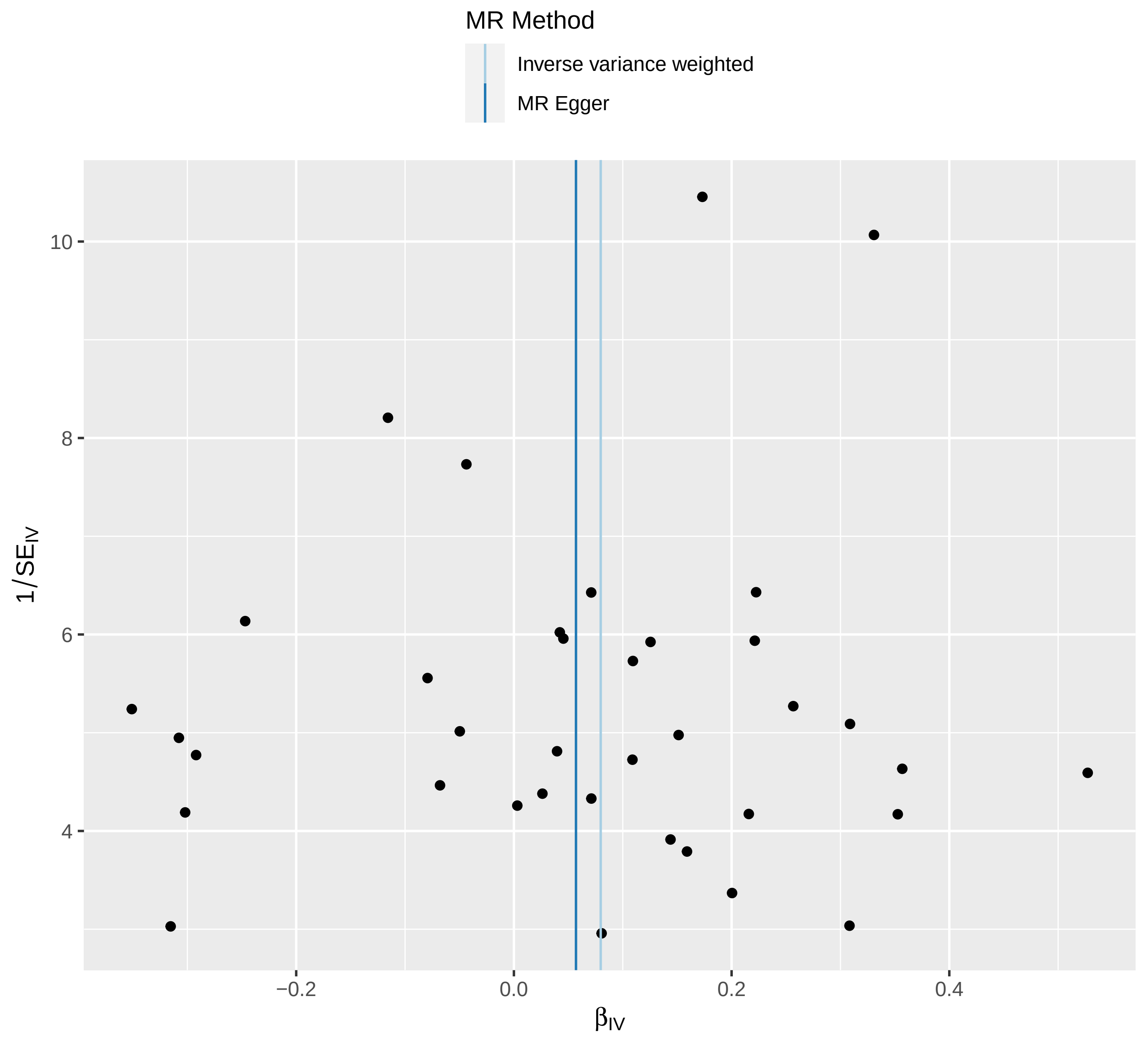
MR Method
Inverse variance weighted
MR Egger
10
8
6
4
1/SE_IV
−0.2
0.0
0.2
0.4
β_IV

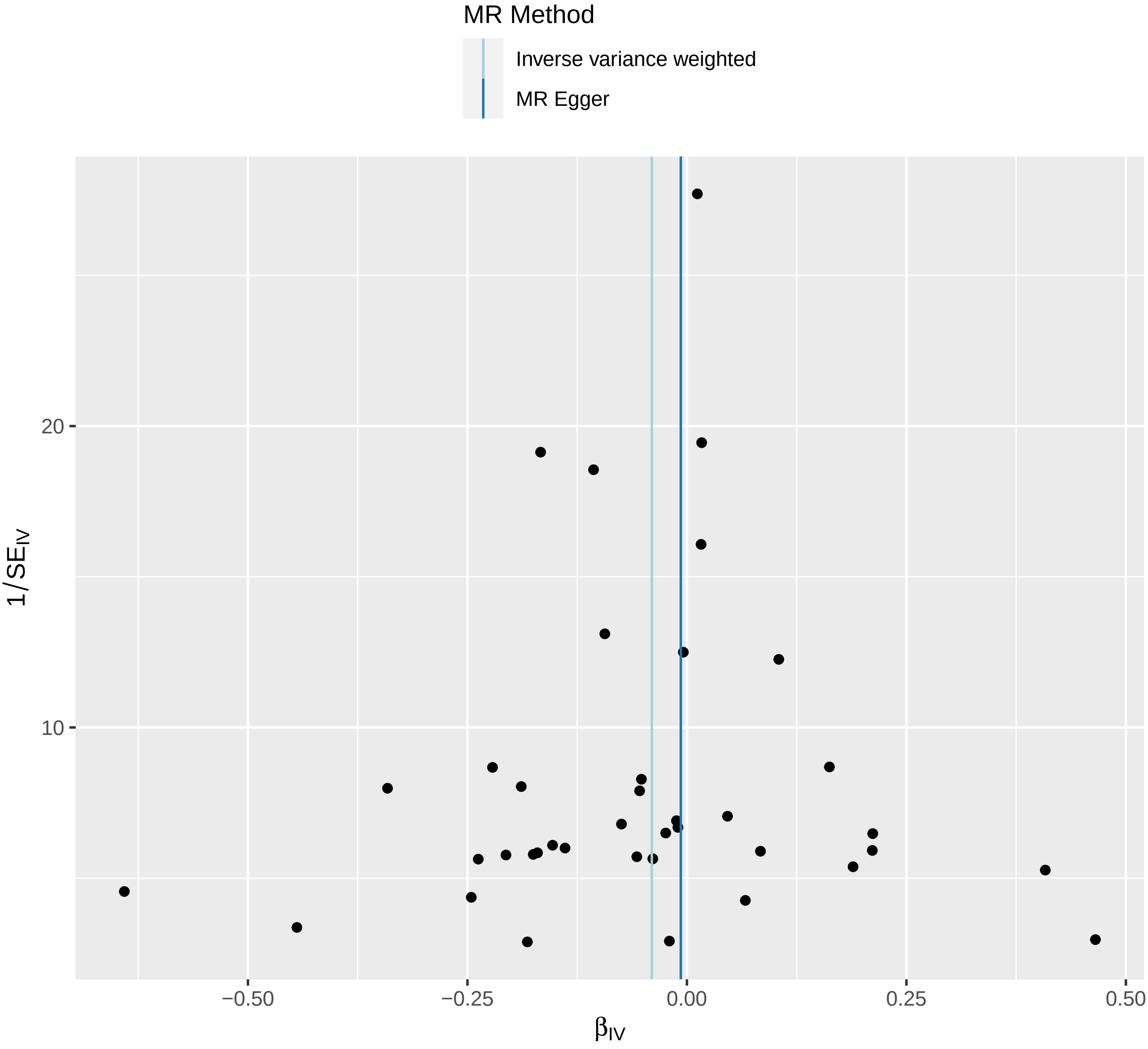

MR Method
Inverse variance weighted
MR Egger
20
10
$1/SE_{IV}$
−0.50
−0.25
0.00
0.25
0.50
β_{IV}

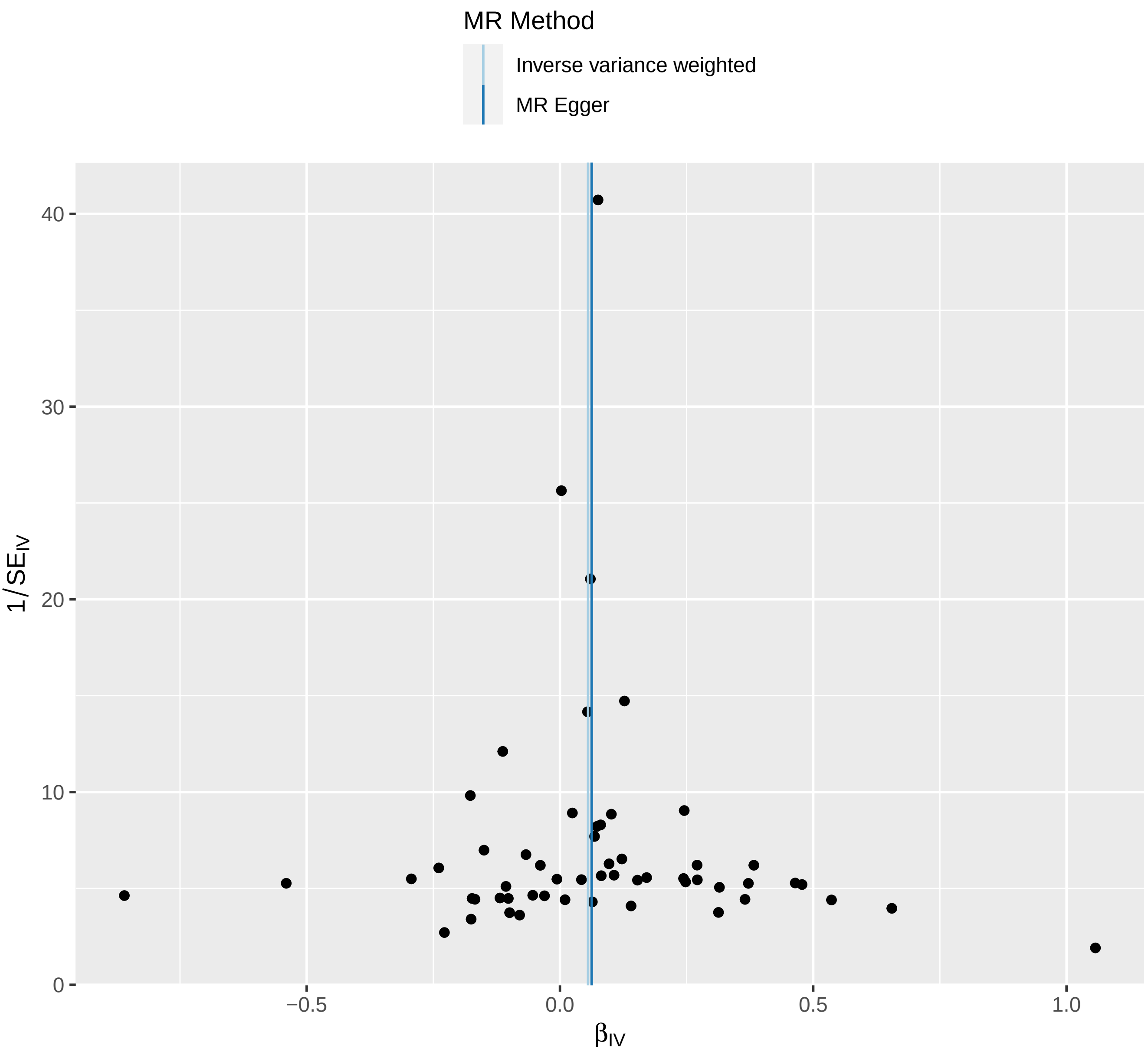
MR Method
Inverse variance weighted
MR Egger
1/SE_IV
40
30
20
10
0
−0.5
0.0
0.5
1.0
β_IV

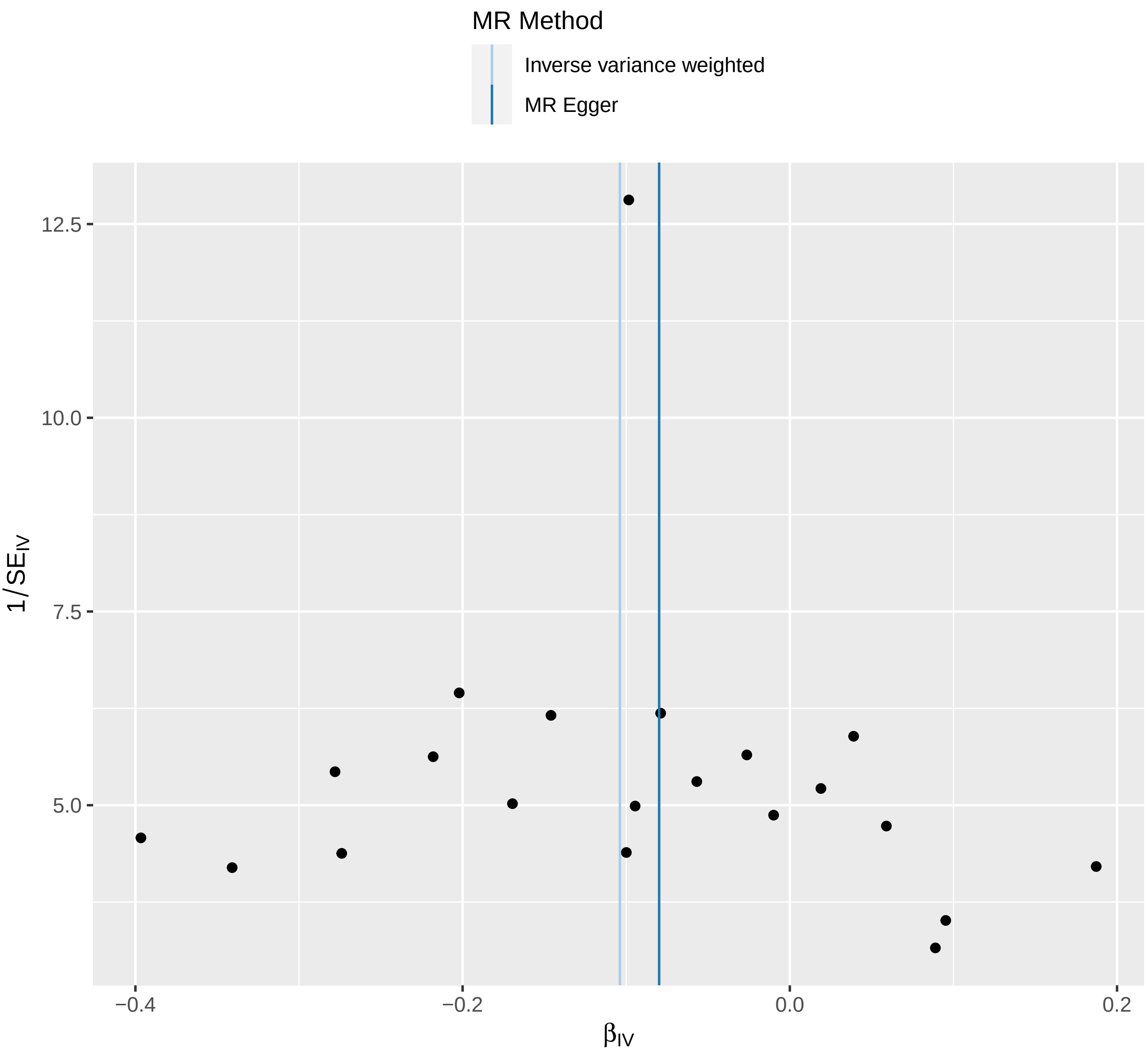

MR Method
Inverse variance weighted
MR Egger
12.5
10.0
7.5
5.0
1/SE_IV
−0.4
−0.2
0.0
0.2
β_IV

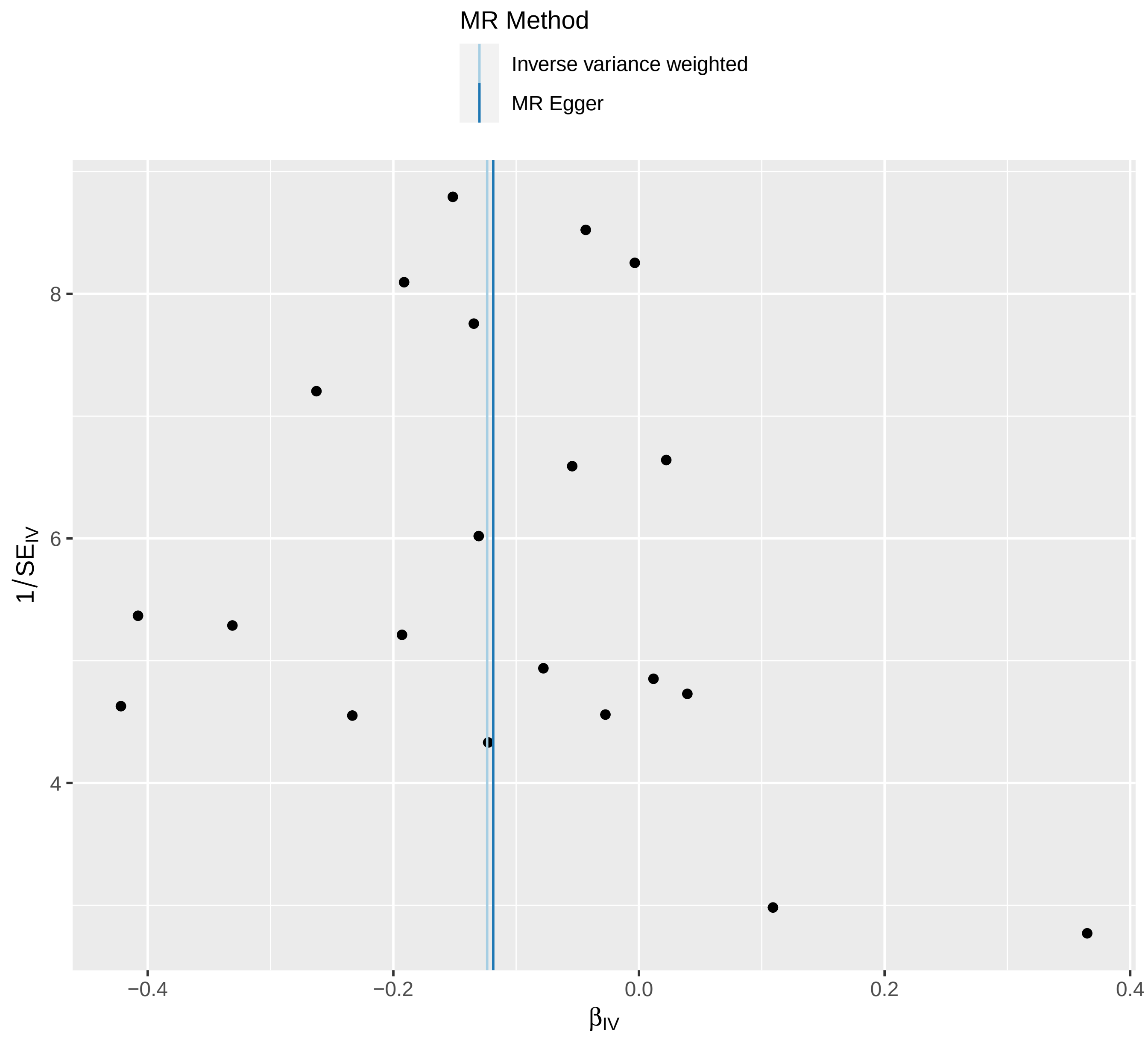

MR Method
Inverse variance weighted
MR Egger
8
6
4
$1/SE_{IV}$
−0.4
−0.2
0.0
0.2
0.4
β_{IV}

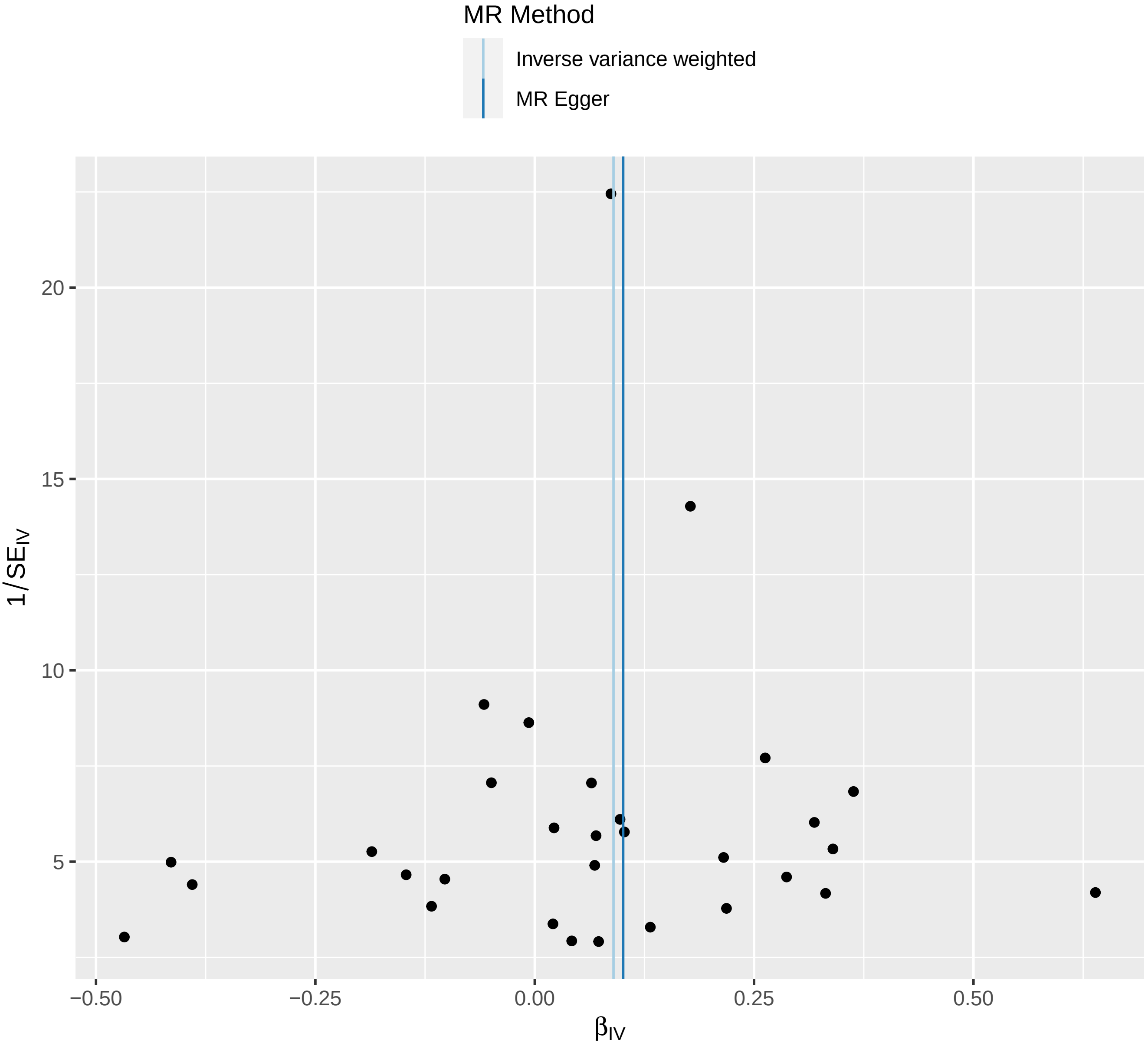
MR Method
Inverse variance weighted
MR Egger
1/SE_IV
20
15
10
5
−0.50
−0.25
0.00
0.25
0.50
β_IV

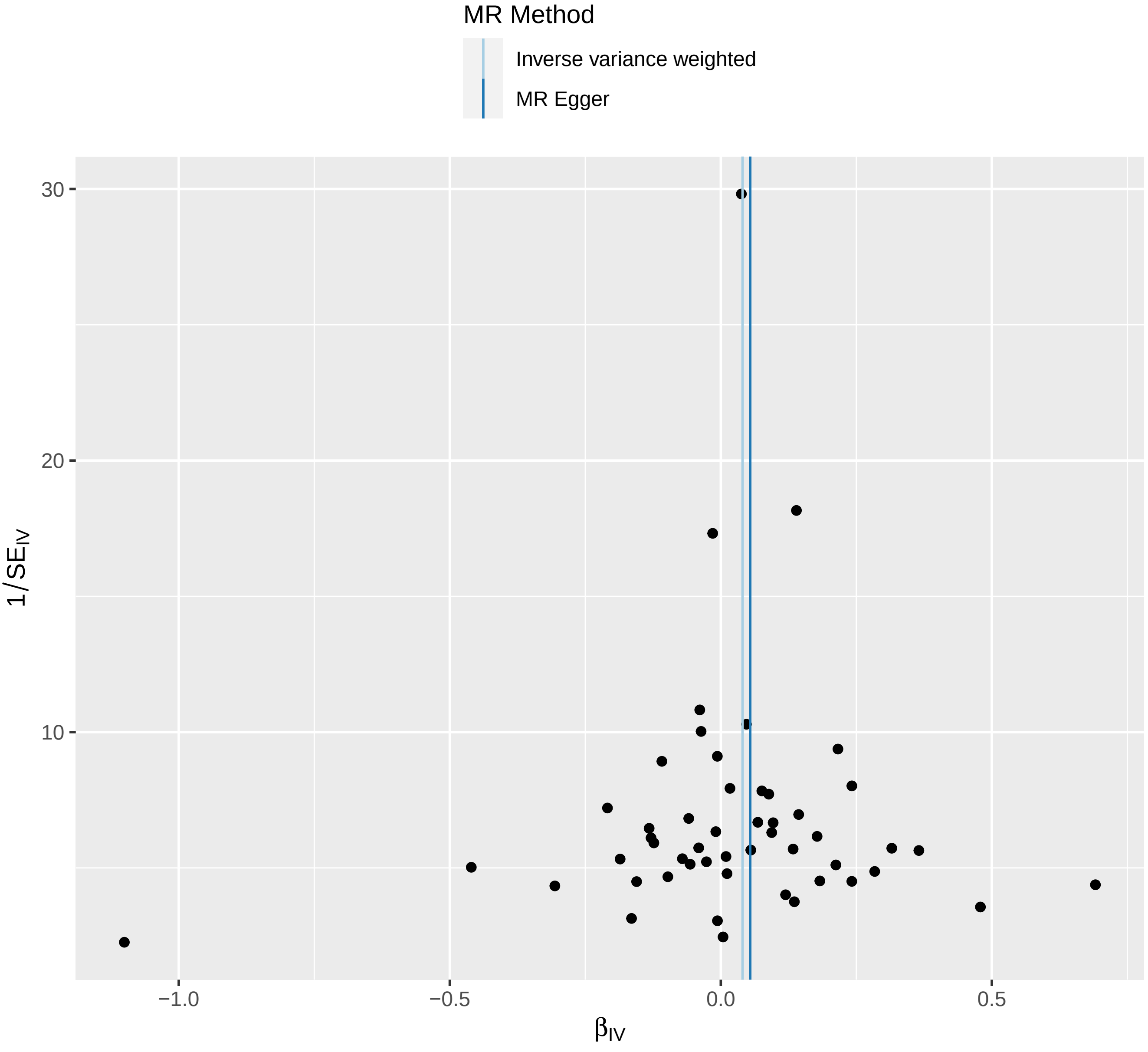
MR Method
Inverse variance weighted
MR Egger
30
20
10
1/SE$_{IV}$
−1.0
−0.5
0.0
0.5
β$_{IV}$

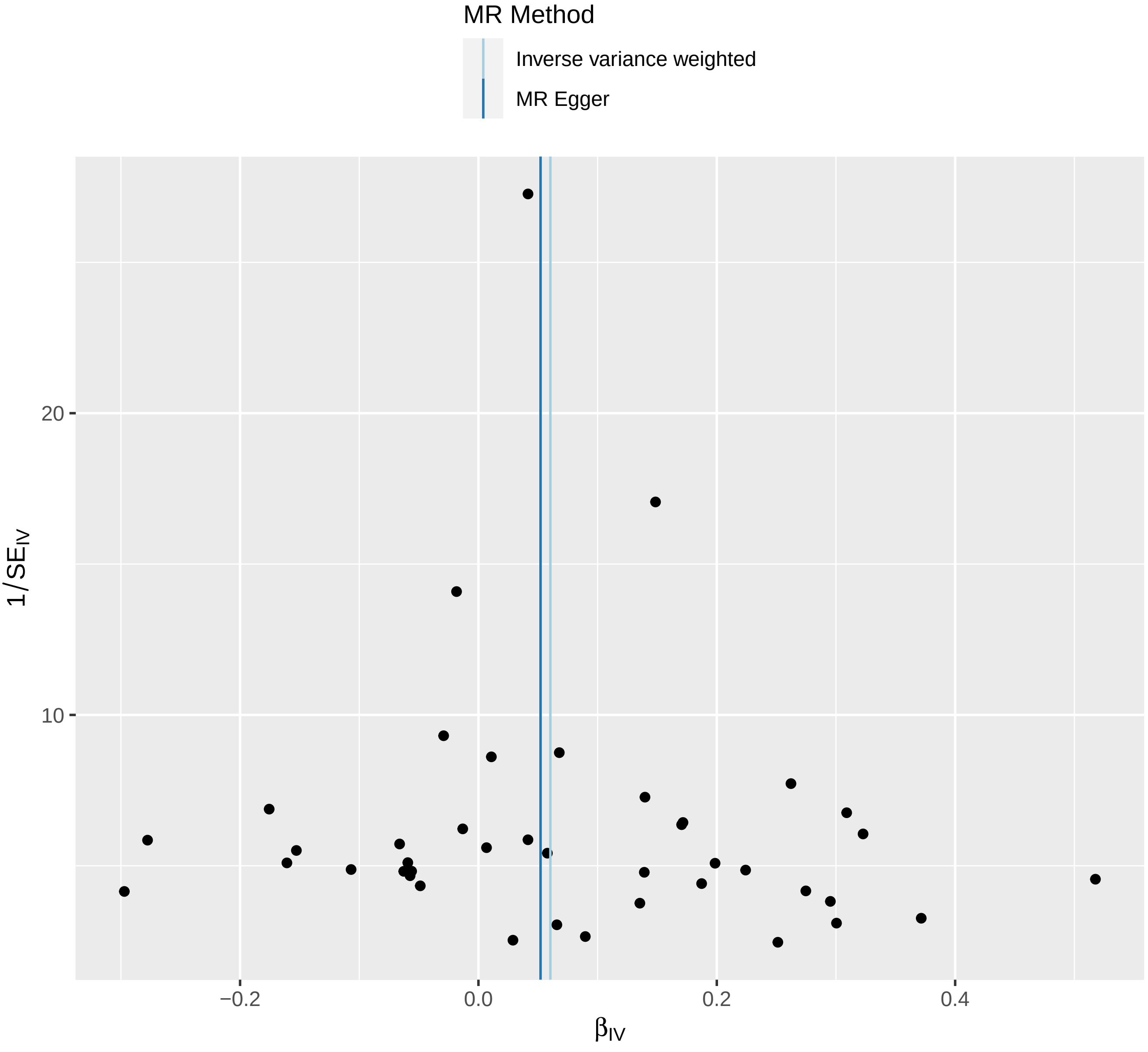
MR Method
Inverse variance weighted
MR Egger
20
10
−0.2
0.0
0.2
0.4
$1/SE_{IV}$
β_{IV}

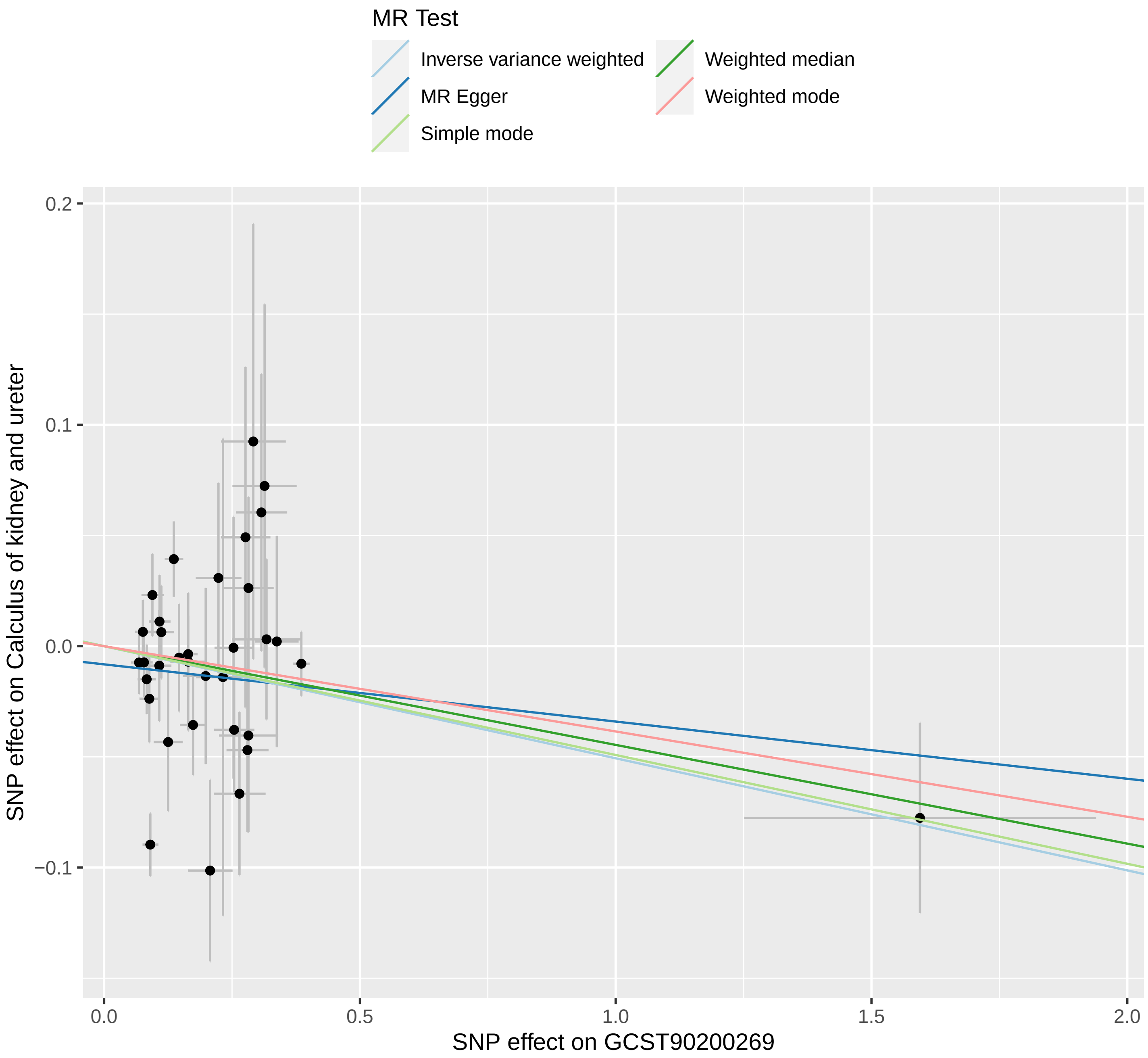
MR Test
Inverse variance weighted
MR Egger
Simple mode
Weighted median
Weighted mode
0.2
0.1
0.0
−0.1
SNP effect on Calculus of kidney and ureter
0.0
0.5
1.0
1.5
2.0
SNP effect on GCST90200269

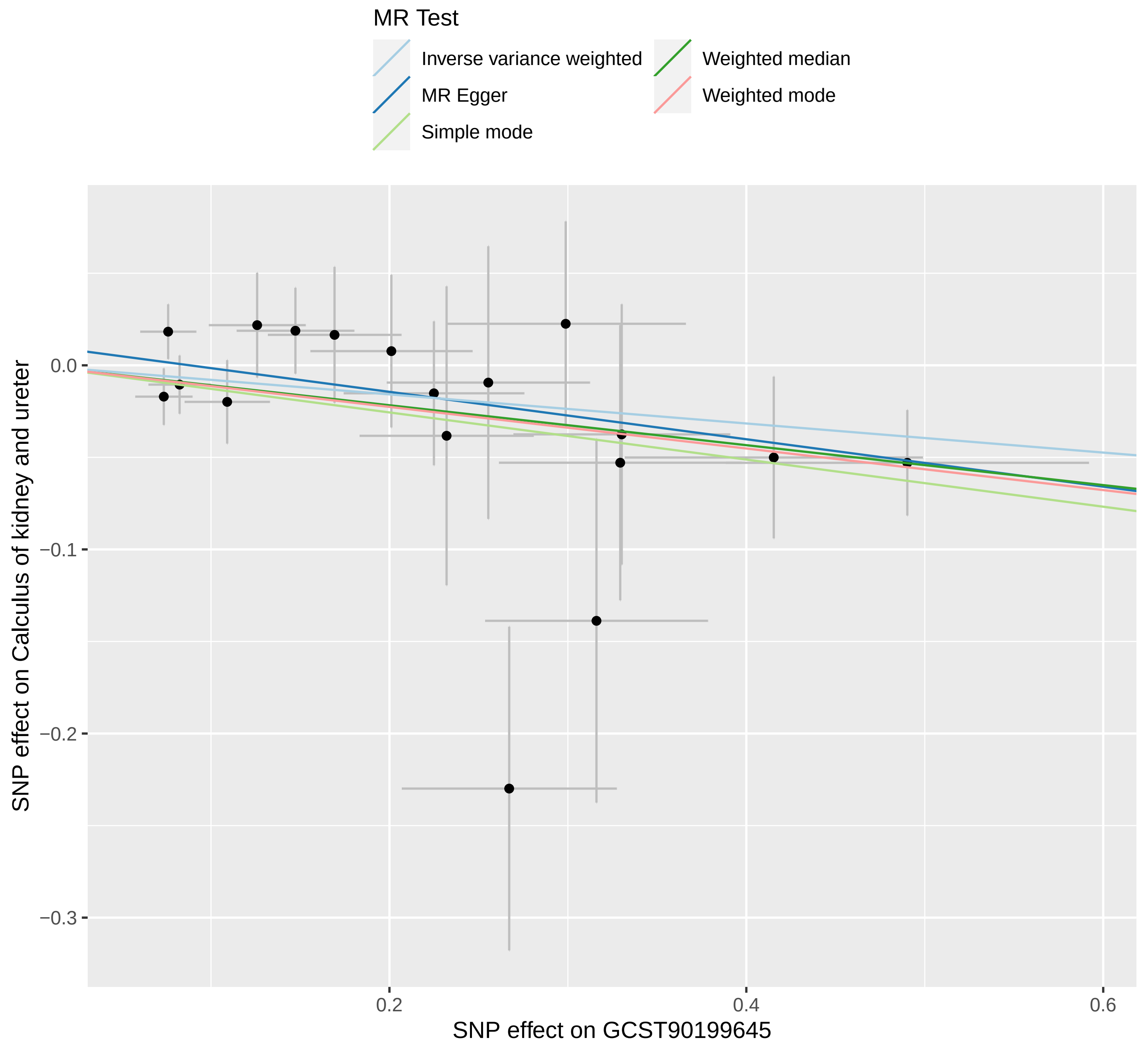
MR Test
Inverse variance weighted
MR Egger
Simple mode
Weighted median
Weighted mode
0.0
−0.1
−0.2
−0.3
0.2
0.4
0.6
SNP effect on Calculus of kidney and ureter
SNP effect on GCST90199645

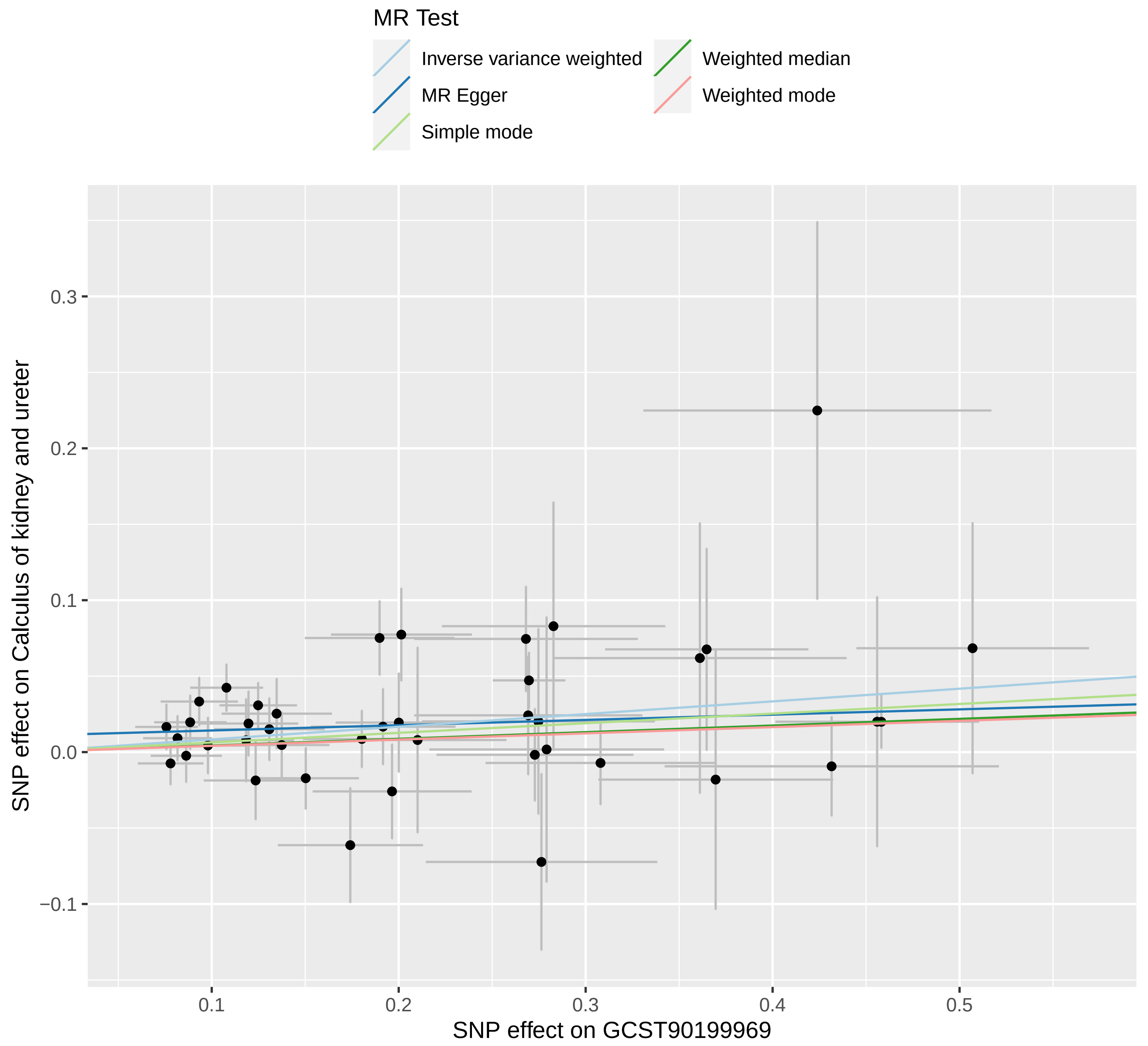
MR Test
Inverse variance weighted
MR Egger
Simple mode
Weighted median
Weighted mode
0.3
0.2
0.1
0.0
−0.1
0.1
0.2
0.3
0.4
0.5
SNP effect on Calculus of kidney and ureter
SNP effect on GCST90199969

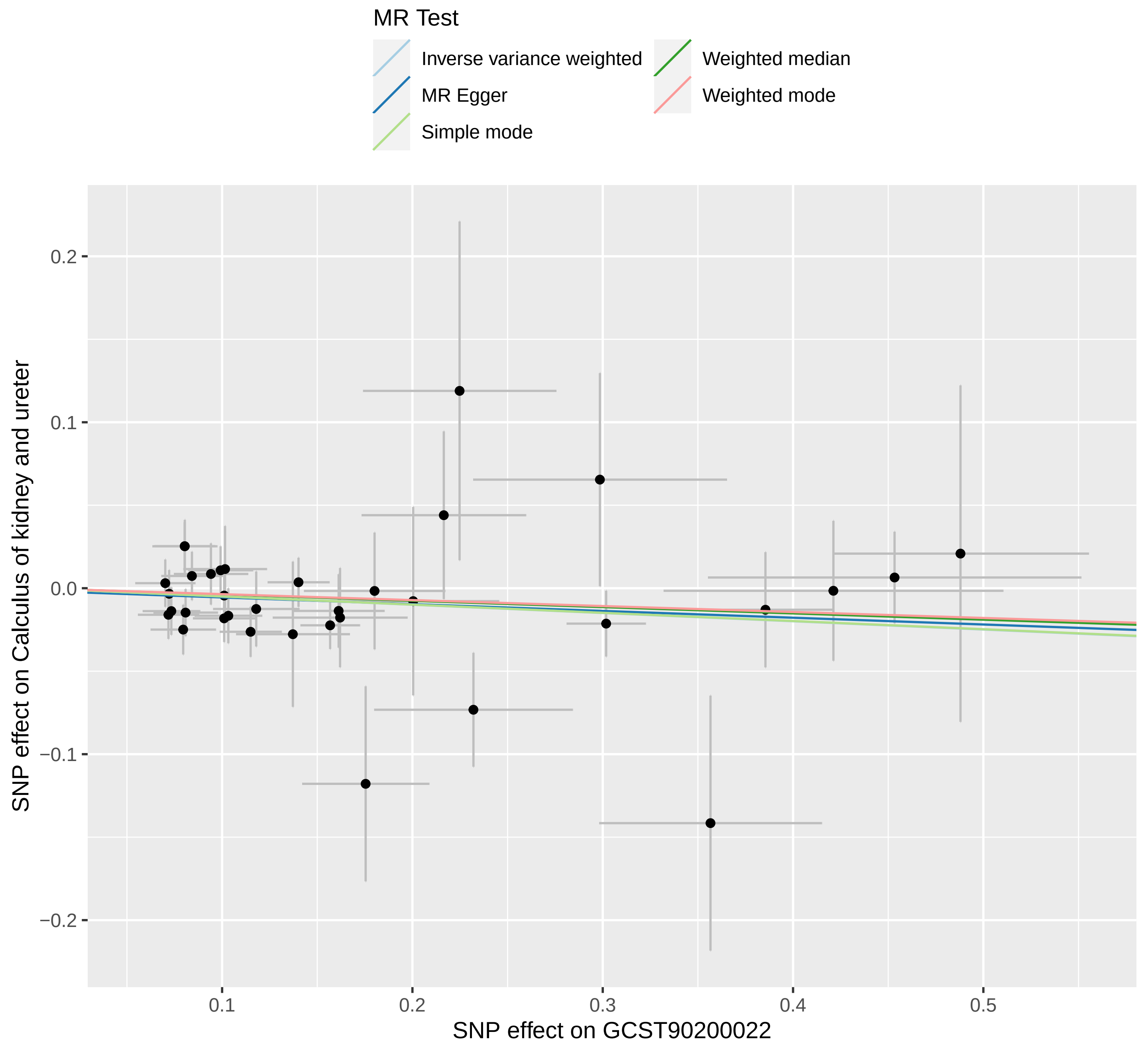
MR Test
Inverse variance weighted
MR Egger
Simple mode
Weighted median
Weighted mode
0.2
0.1
0.0
−0.1
−0.2
SNP effect on Calculus of kidney and ureter
0.1
0.2
0.3
0.4
0.5
SNP effect on GCST90200022

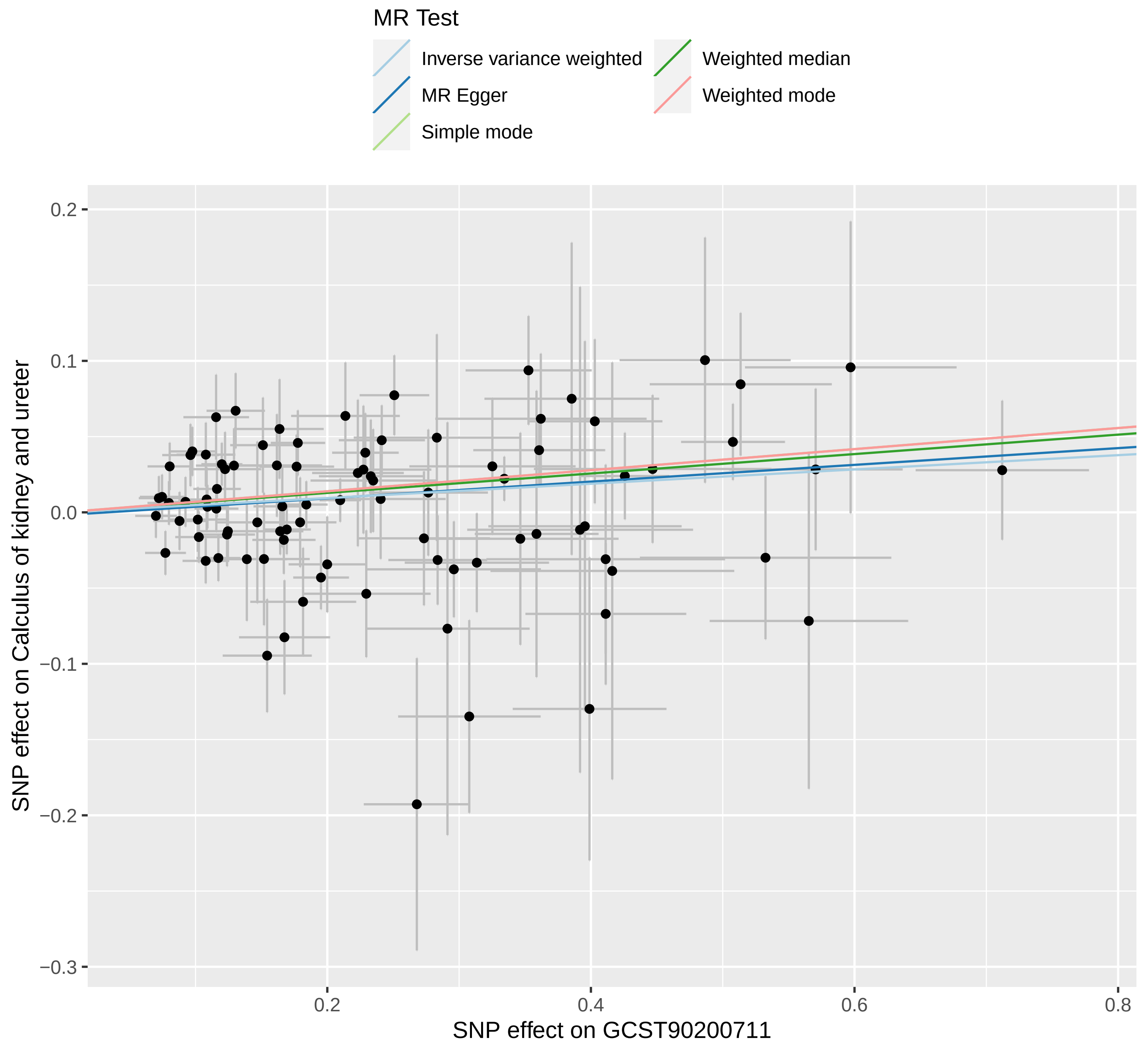
MR Test
Inverse variance weighted
MR Egger
Simple mode
Weighted median
Weighted mode
0.2
0.1
0.0
−0.1
−0.2
−0.3
0.2
0.4
0.6
0.8
SNP effect on Calculus of kidney and ureter
SNP effect on GCST90200711

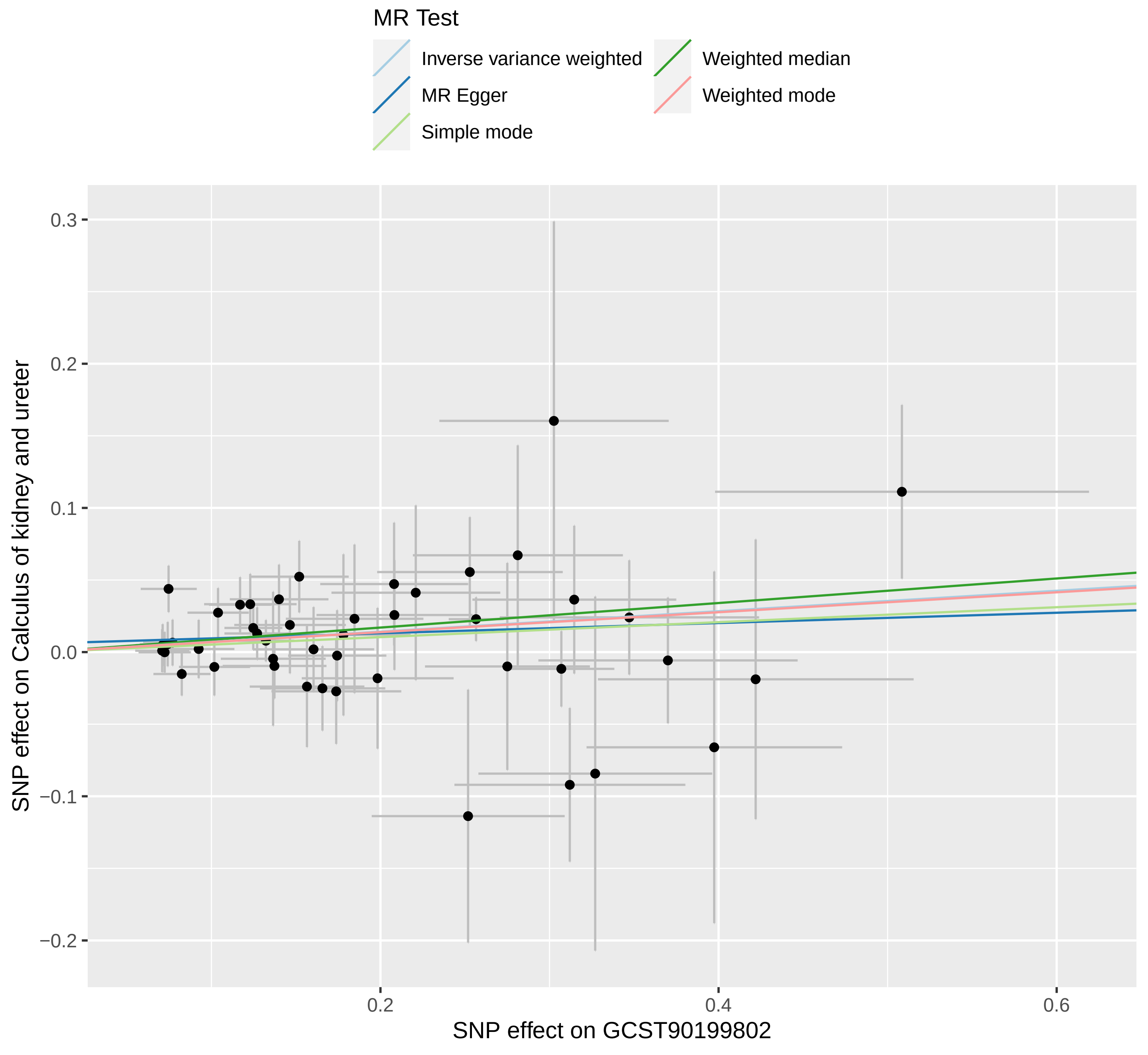

MR Test
Inverse variance weighted
MR Egger
Simple mode
Weighted median
Weighted mode
0.3
0.2
0.1
0.0
−0.1
−0.2
SNP effect on Calculus of kidney and ureter
0.2
0.4
0.6
SNP effect on GCST90199802

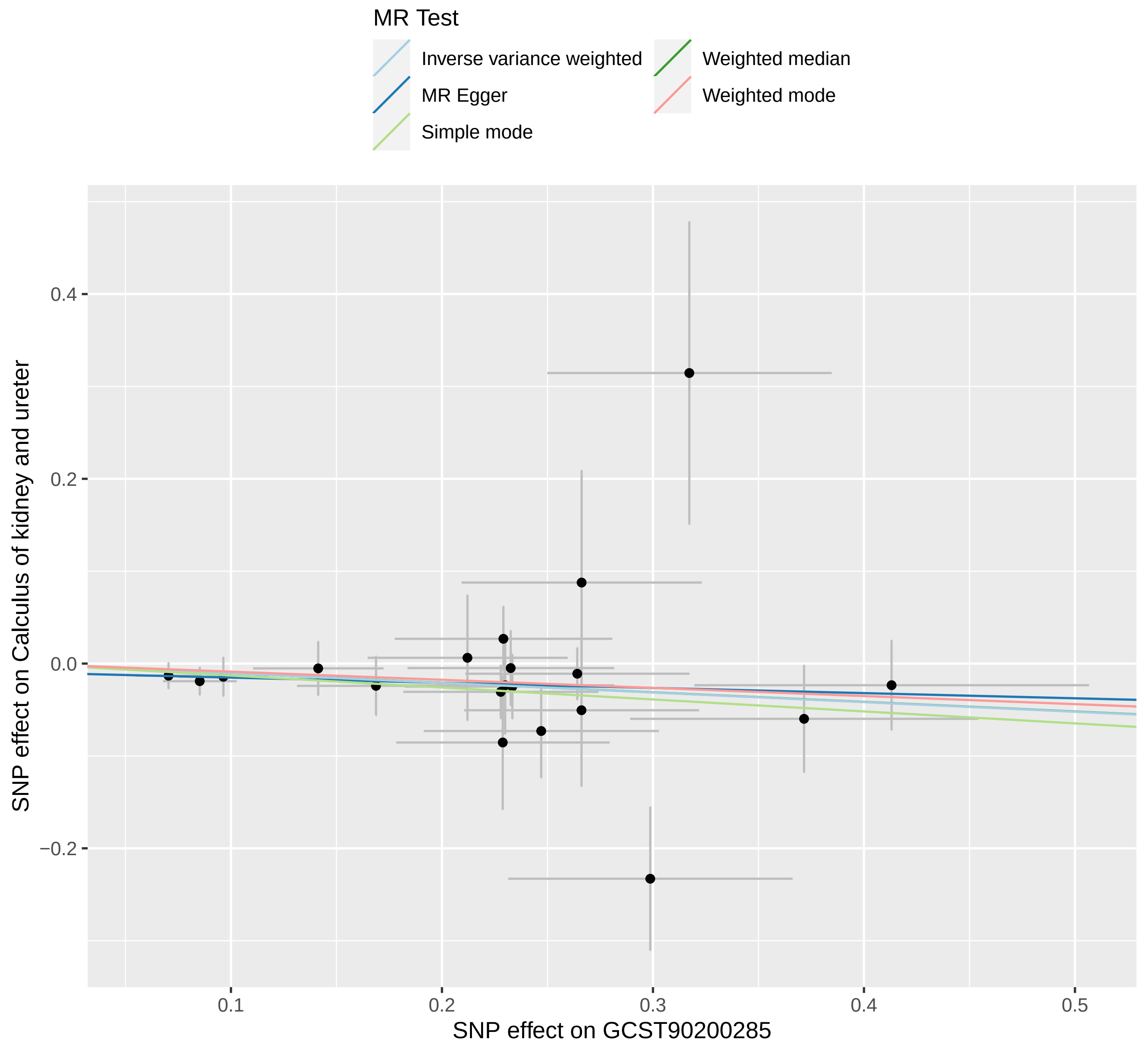

MR Test
Inverse variance weighted
MR Egger
Simple mode
Weighted median
Weighted mode
SNP effect on Calculus of kidney and ureter
0.4
0.2
0.0
−0.2
0.1
0.2
0.3
0.4
0.5
SNP effect on GCST90200285

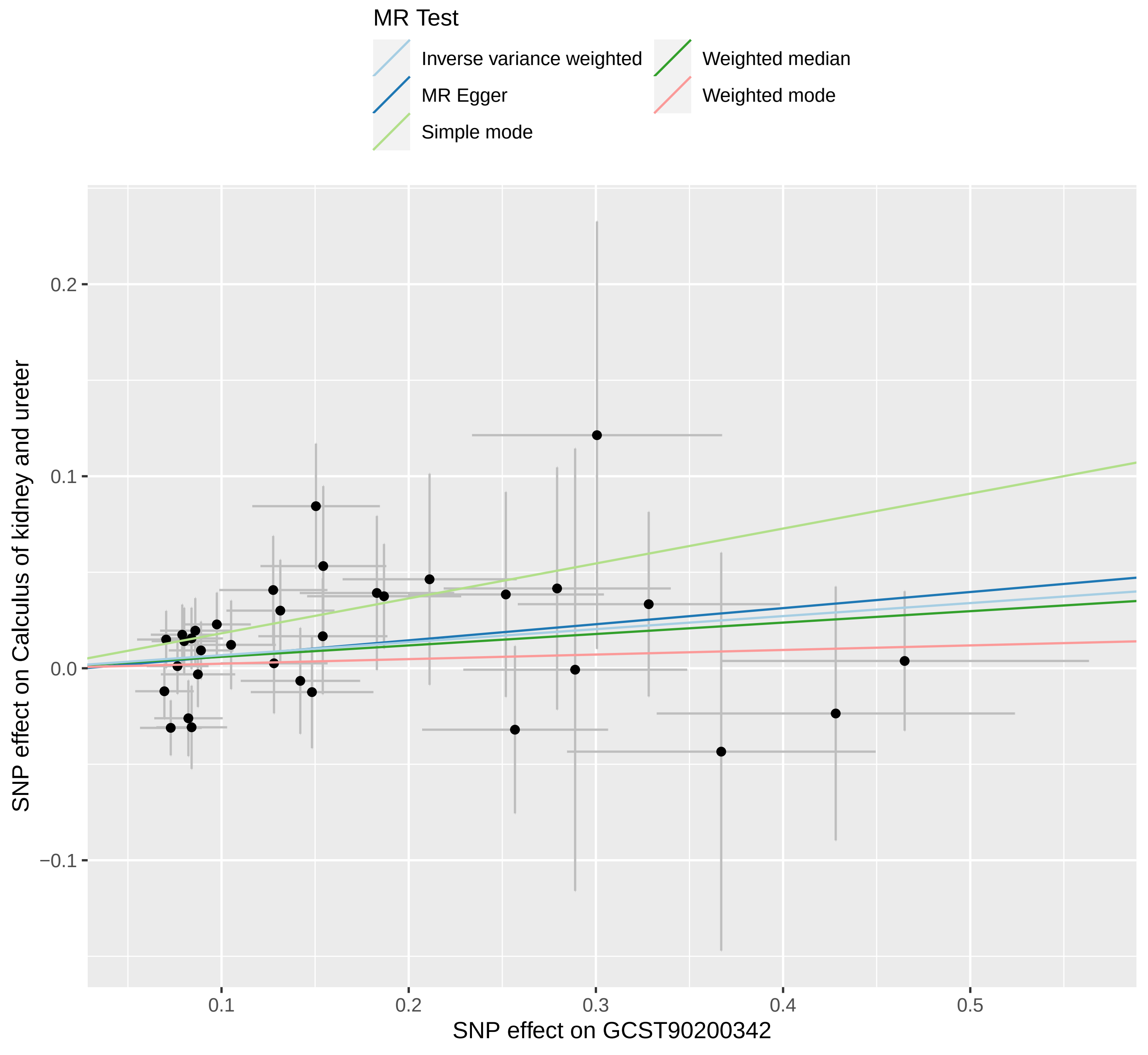
MR Test
Inverse variance weighted
MR Egger
Simple mode
Weighted median
Weighted mode
0.2
0.1
0.0
−0.1
SNP effect on Calculus of kidney and ureter
0.1
0.2
0.3
0.4
0.5
SNP effect on GCST90200342

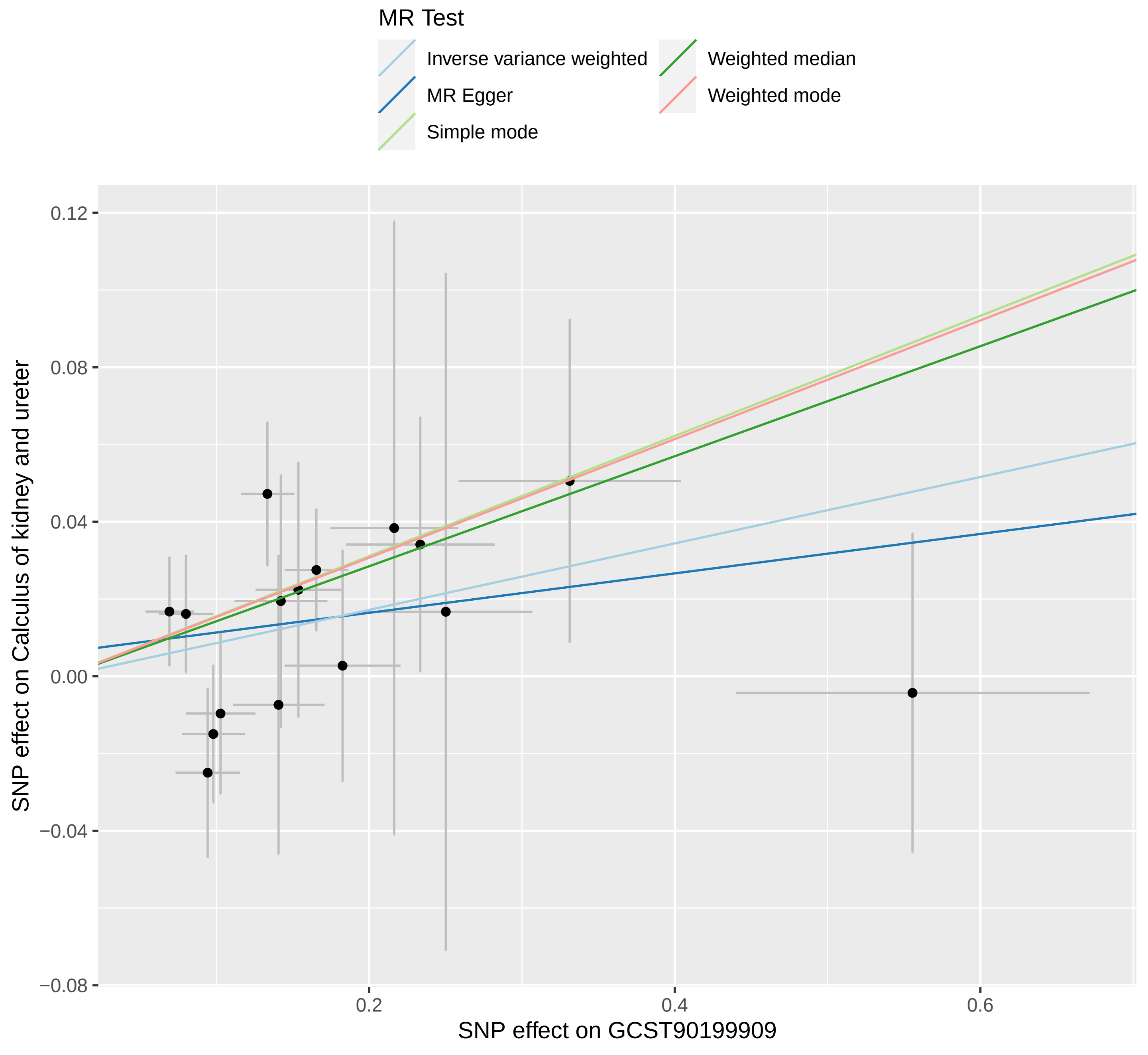

MR Test
Inverse variance weighted
MR Egger
Simple mode
Weighted median
Weighted mode
SNP effect on Calculus of kidney and ureter
0.12
0.08
0.04
0.00
−0.04
−0.08
0.2
0.4
0.6
SNP effect on GCST90199909

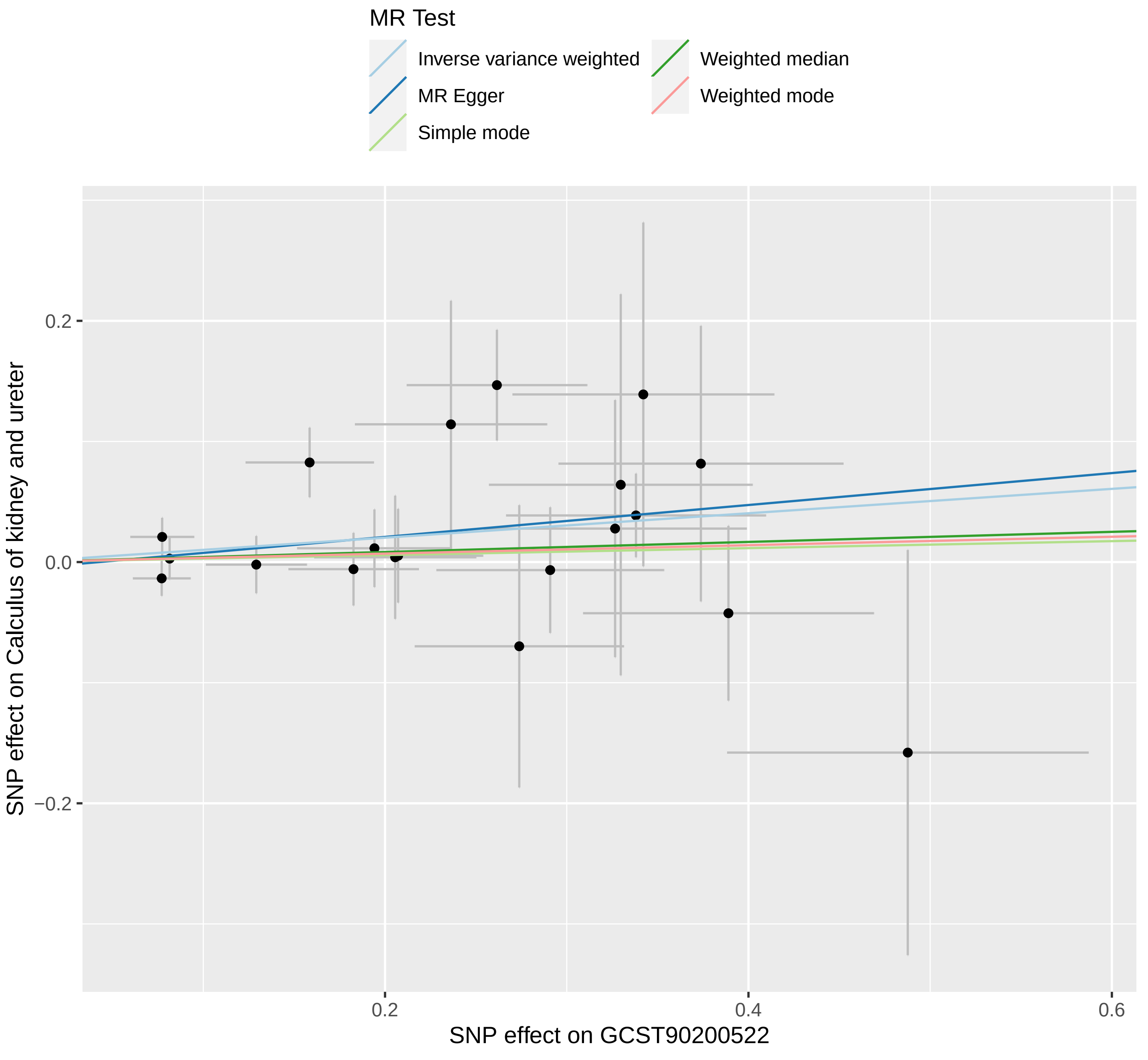

MR Test
Inverse variance weighted
MR Egger
Simple mode
Weighted median
Weighted mode
0.2
0.0
−0.2
0.2
0.4
0.6
SNP effect on Calculus of kidney and ureter
SNP effect on GCST90200522

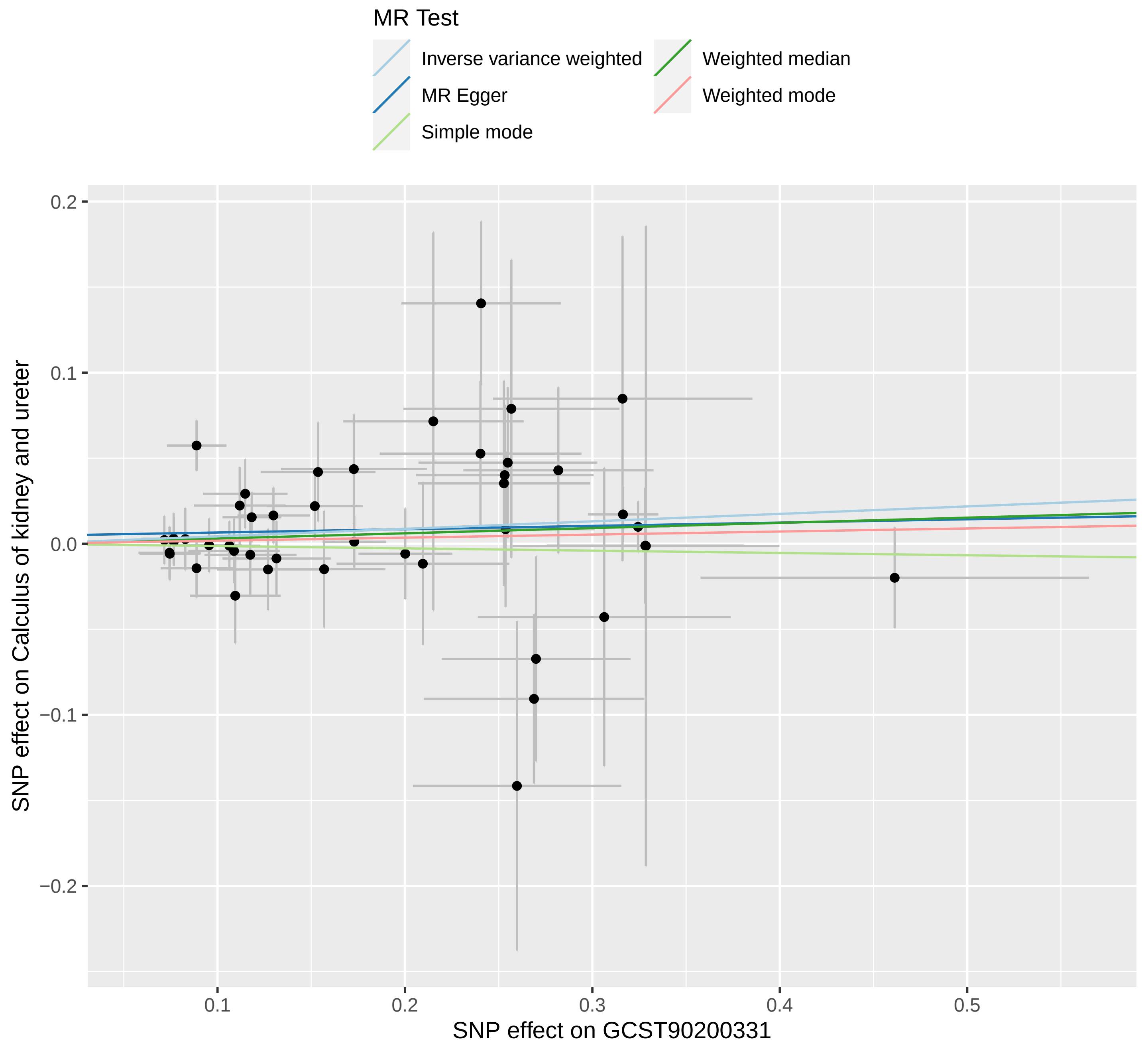

MR Test
Inverse variance weighted
MR Egger
Simple mode
Weighted median
Weighted mode
0.2
0.1
0.0
−0.1
−0.2
SNP effect on Calculus of kidney and ureter
0.1
0.2
0.3
0.4
0.5
SNP effect on GCST90200331

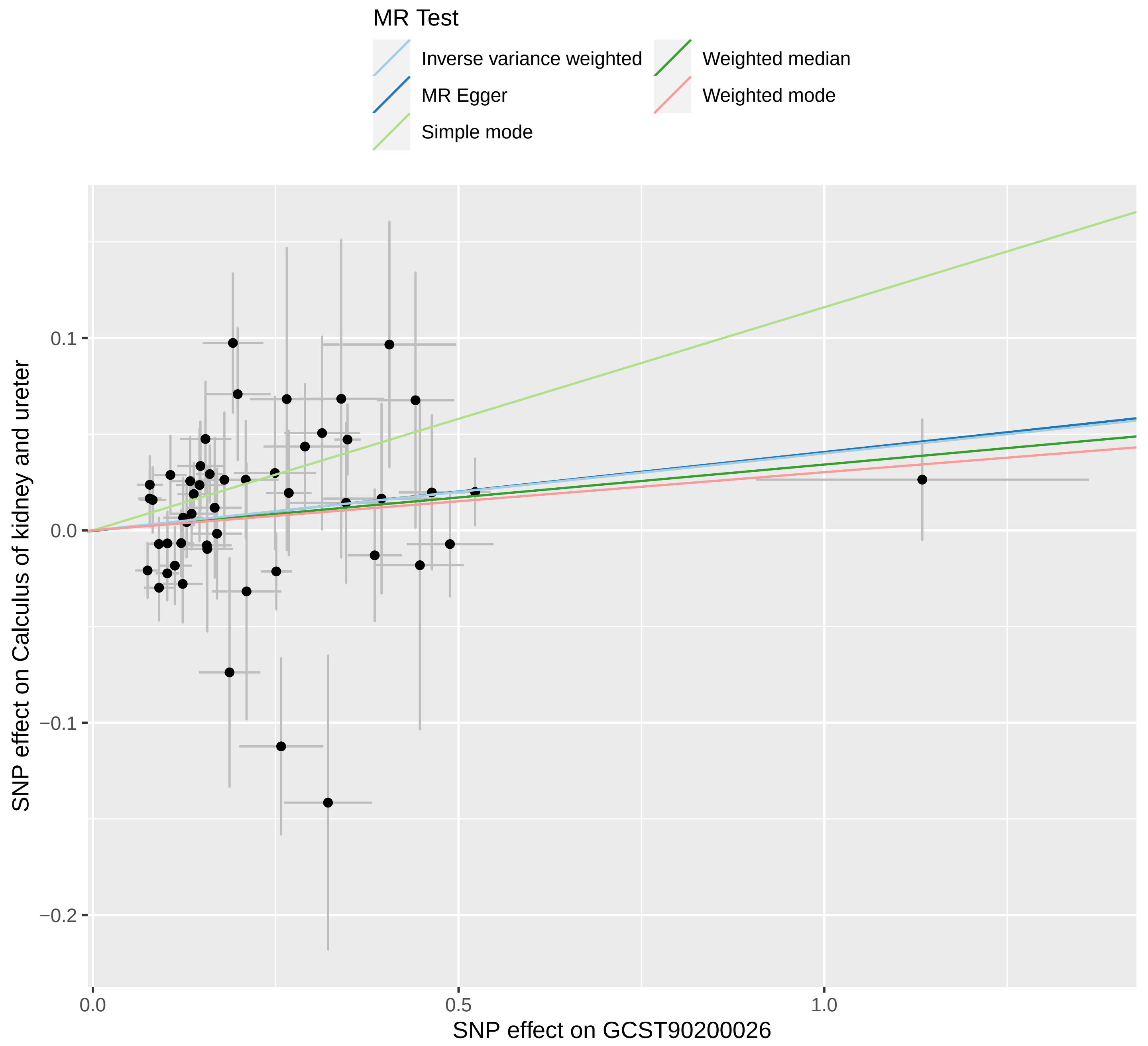

MR Test
Inverse variance weighted
MR Egger
Simple mode
Weighted median
Weighted mode
SNP effect on Calculus of kidney and ureter
0.1
0.0
−0.1
−0.2
0.0
0.5
1.0
SNP effect on GCST90200026

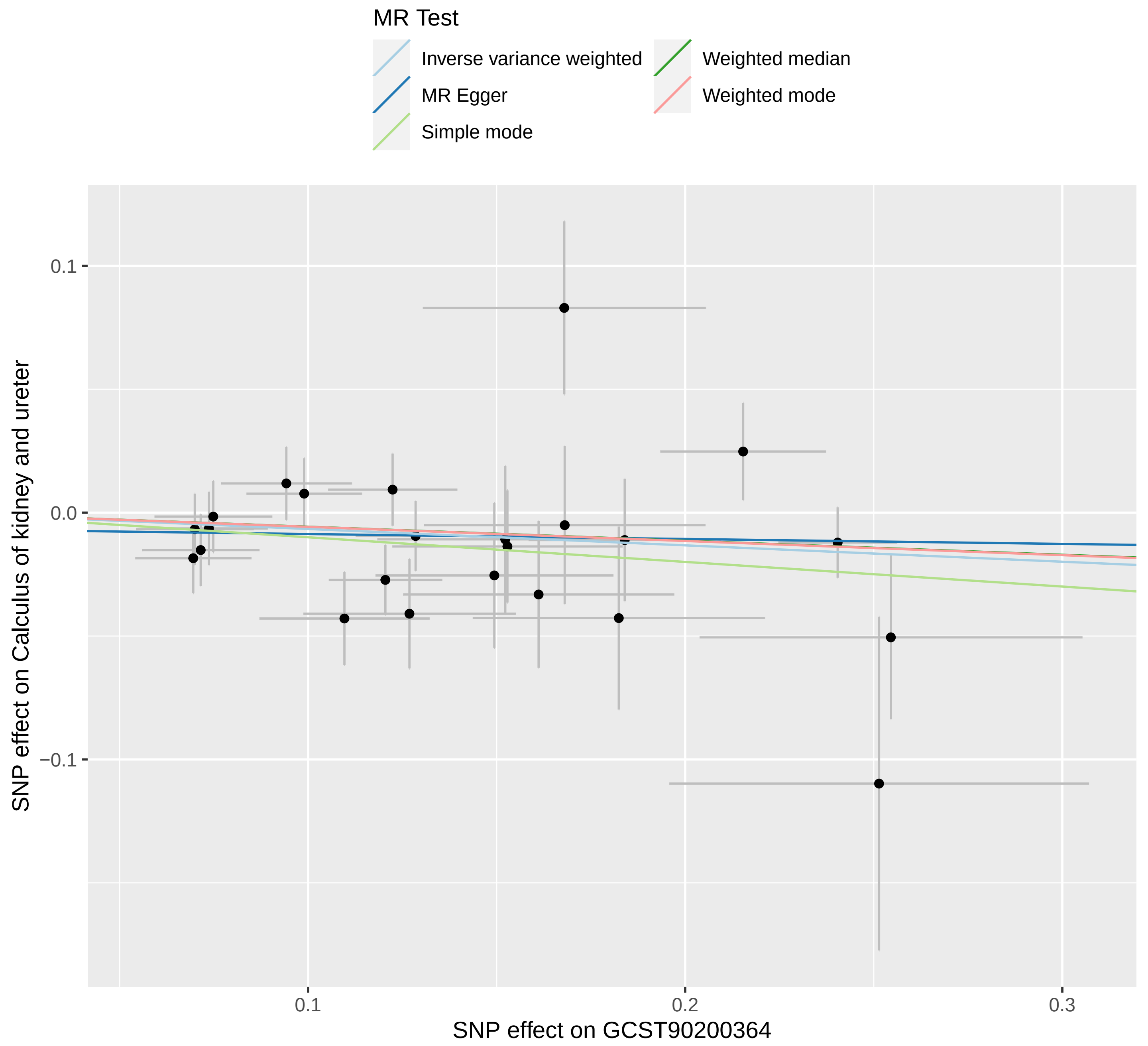
MR Test
Inverse variance weighted
MR Egger
Simple mode
Weighted median
Weighted mode
0.1
0.0
−0.1
SNP effect on Calculus of kidney and ureter
0.1
0.2
0.3
SNP effect on GCST90200364

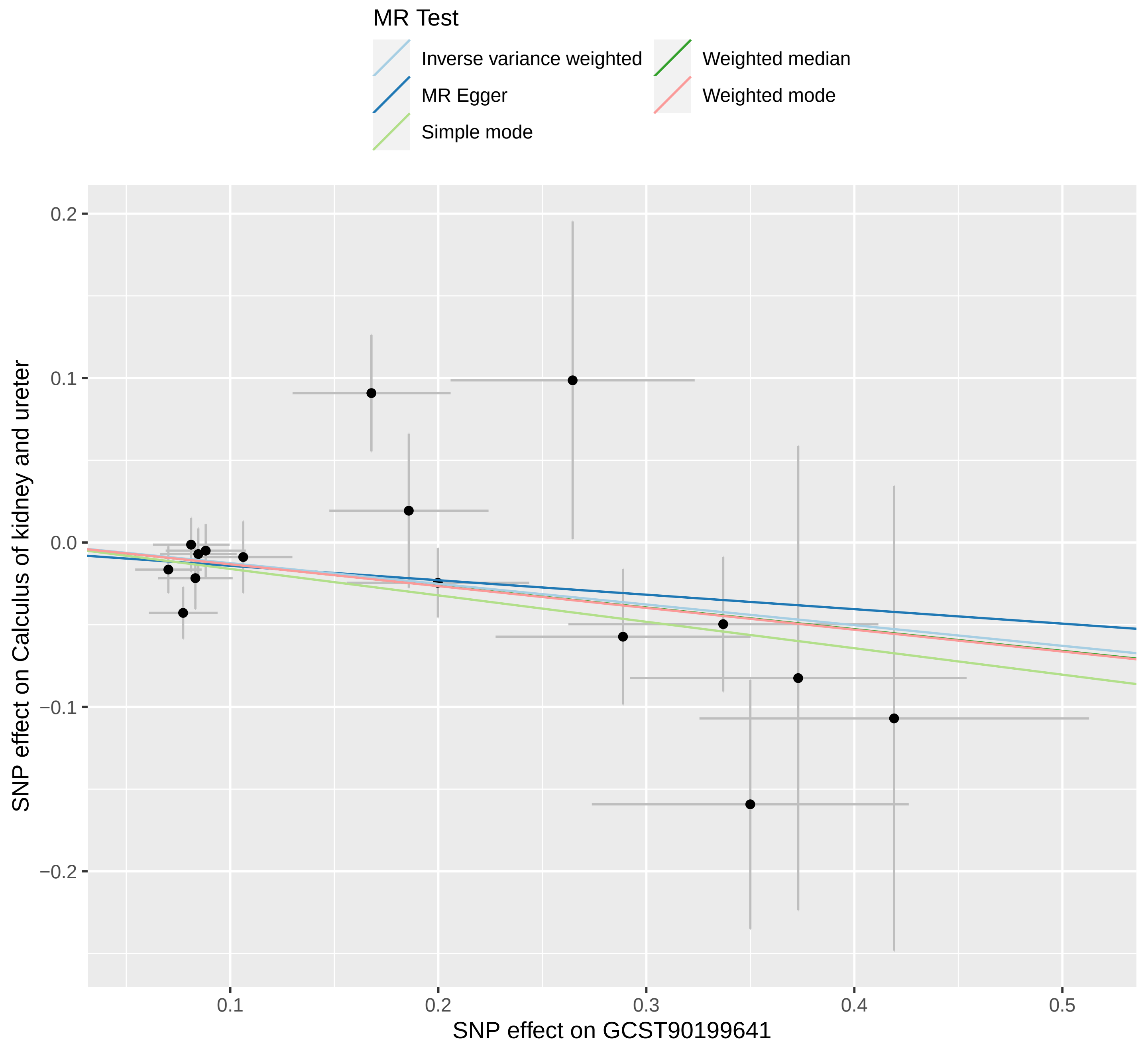
MR Test
Inverse variance weighted
MR Egger
Simple mode
Weighted median
Weighted mode
0.2
0.1
0.0
−0.1
−0.2
SNP effect on Calculus of kidney and ureter
0.1
0.2
0.3
0.4
0.5
SNP effect on GCST90199641

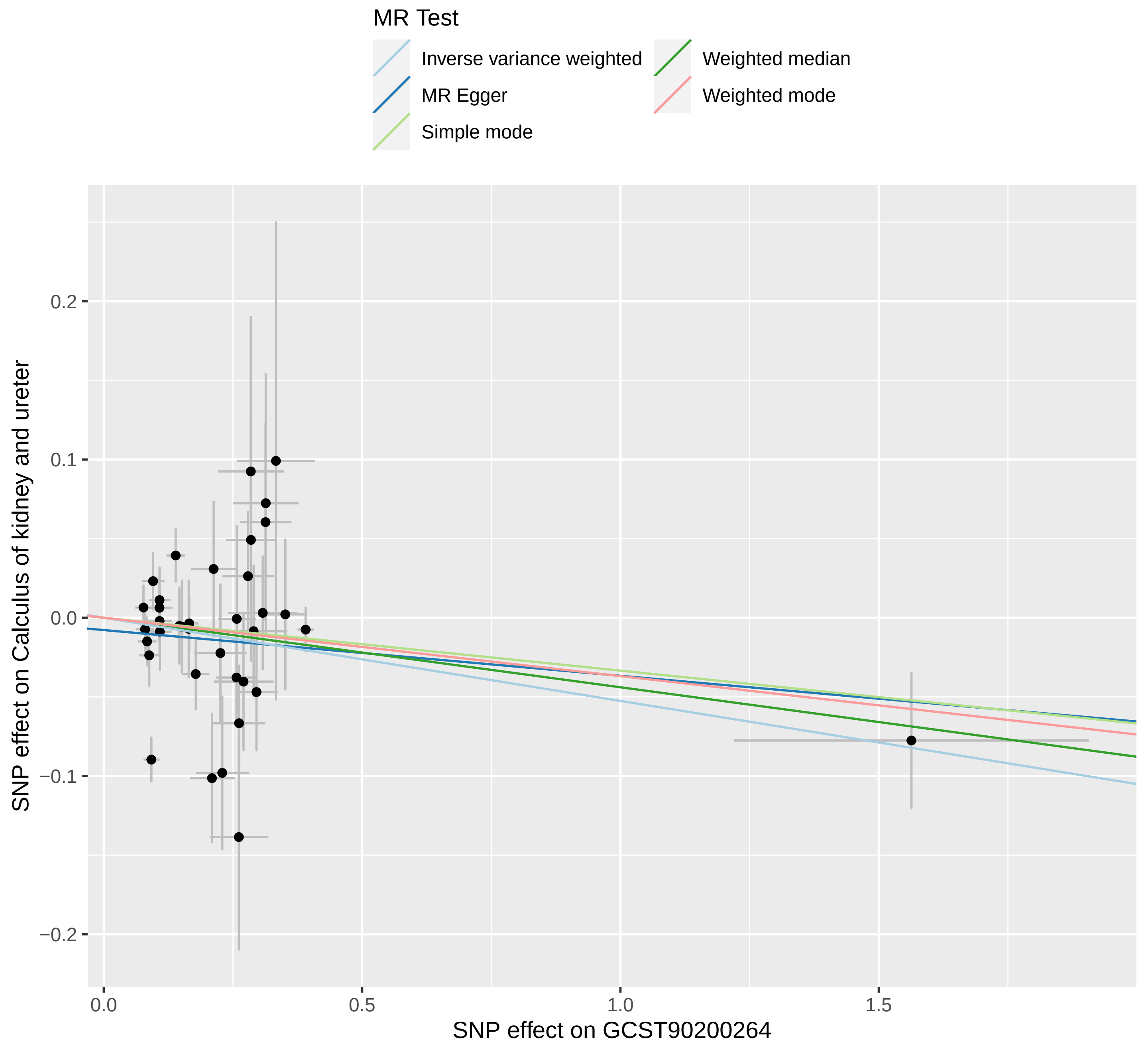
MR Test
Inverse variance weighted
MR Egger
Simple mode
Weighted median
Weighted mode
SNP effect on Calculus of kidney and ureter
0.2
0.1
0.0
−0.1
−0.2
0.0
0.5
1.0
1.5
SNP effect on GCST90200264

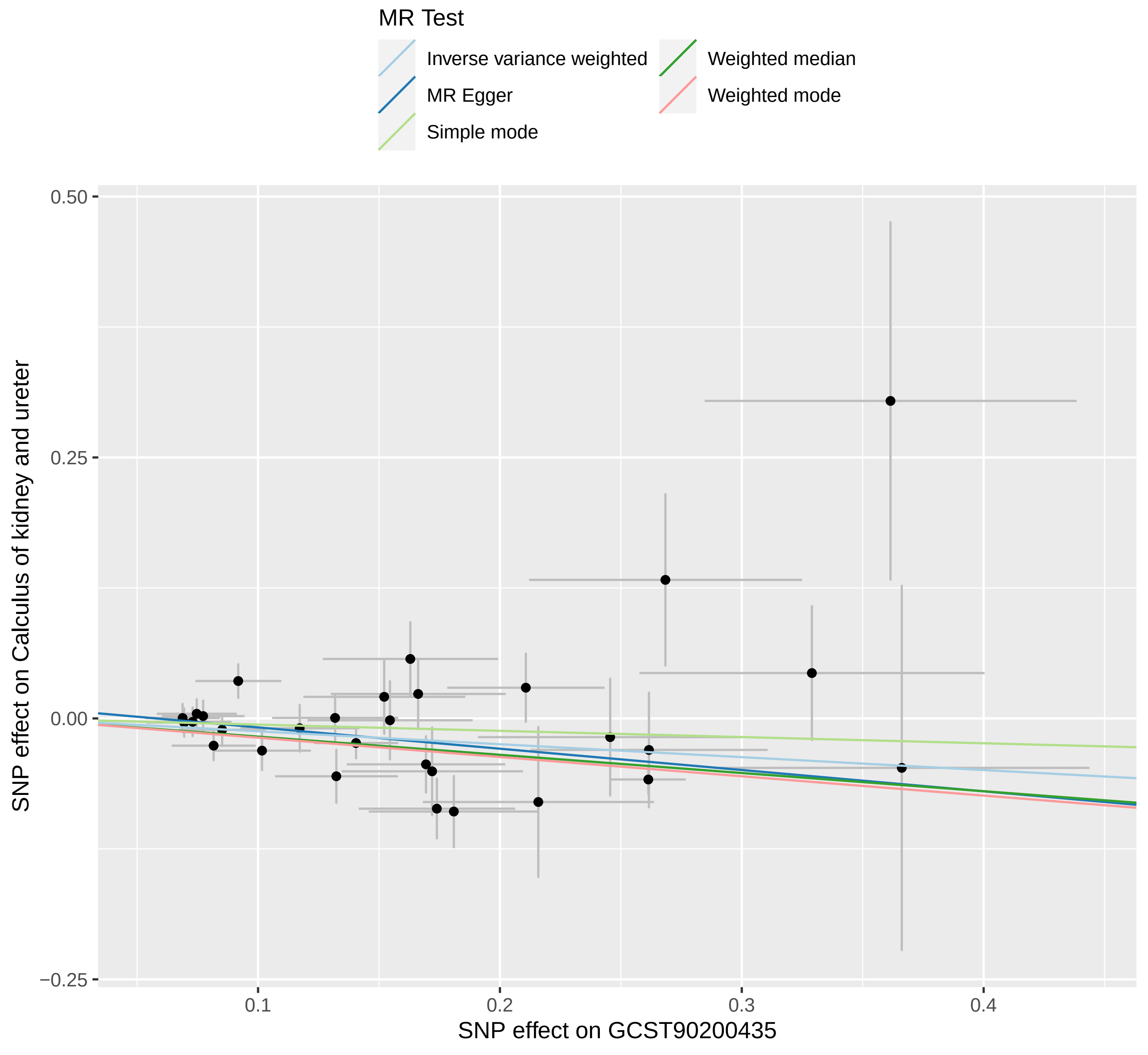
MR Test
Inverse variance weighted
MR Egger
Simple mode
Weighted median
Weighted mode
0.50
0.25
0.00
−0.25
0.1
0.2
0.3
0.4
SNP effect on GCST90200435
SNP effect on Calculus of kidney and ureter

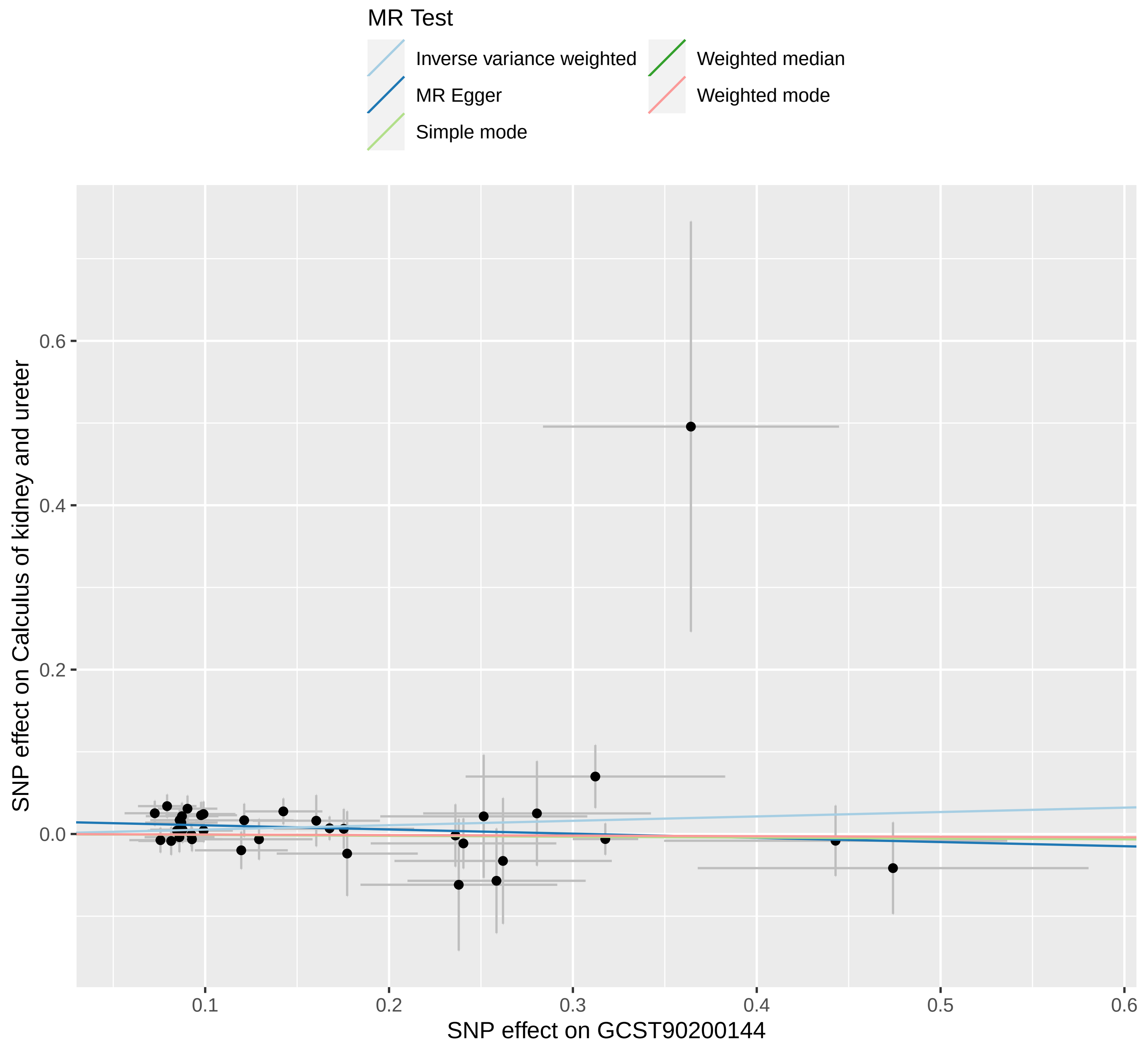

MR Test
Inverse variance weighted
MR Egger
Simple mode
Weighted median
Weighted mode
SNP effect on Calculus of kidney and ureter
0.6
0.4
0.2
0.0
0.1
0.2
0.3
0.4
0.5
0.6
SNP effect on GCST90200144

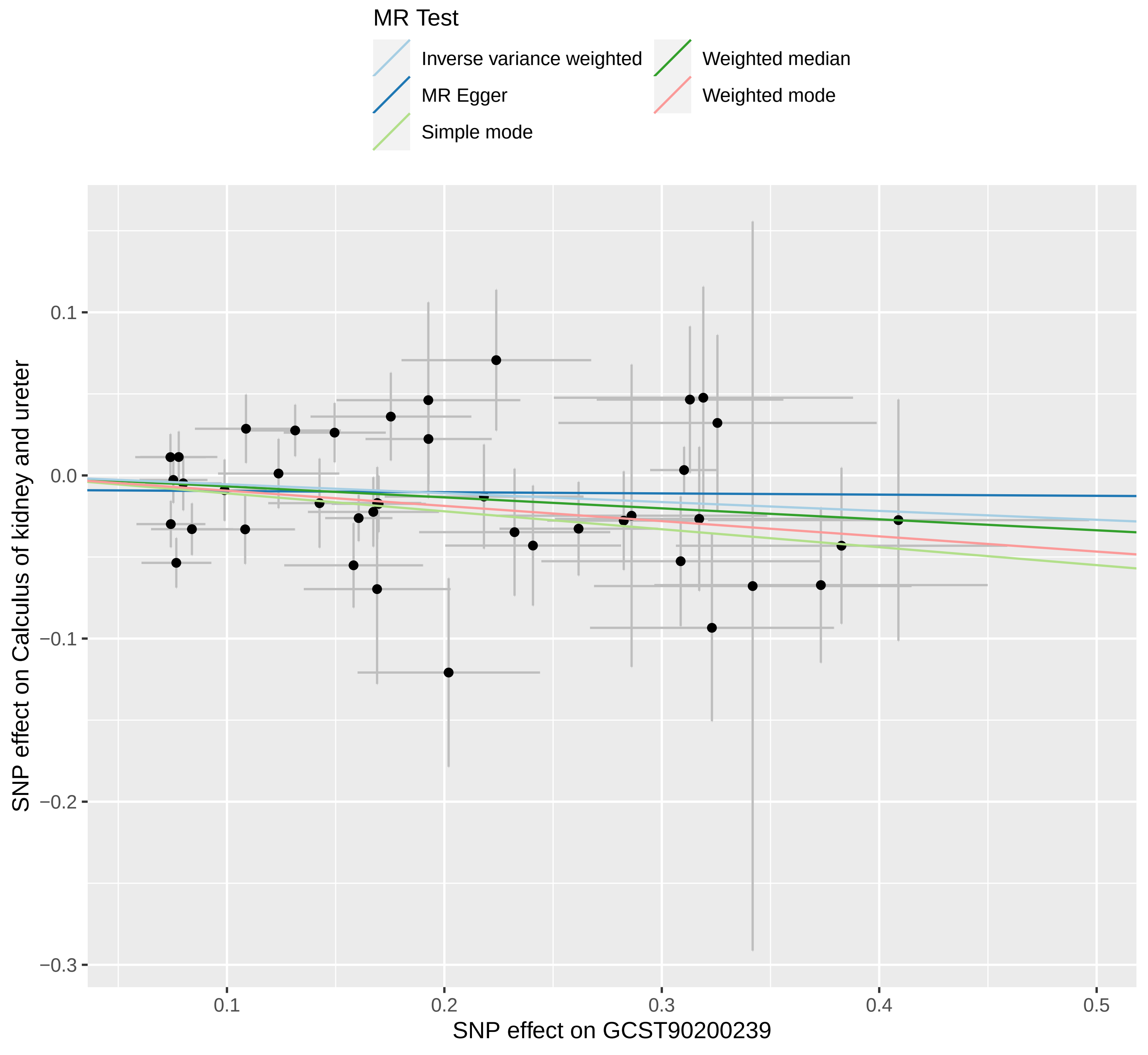

MR Test
Inverse variance weighted
MR Egger
Simple mode
Weighted median
Weighted mode
SNP effect on Calculus of kidney and ureter
0.1
0.0
−0.1
−0.2
−0.3
0.1
0.2
0.3
0.4
0.5
SNP effect on GCST90200239

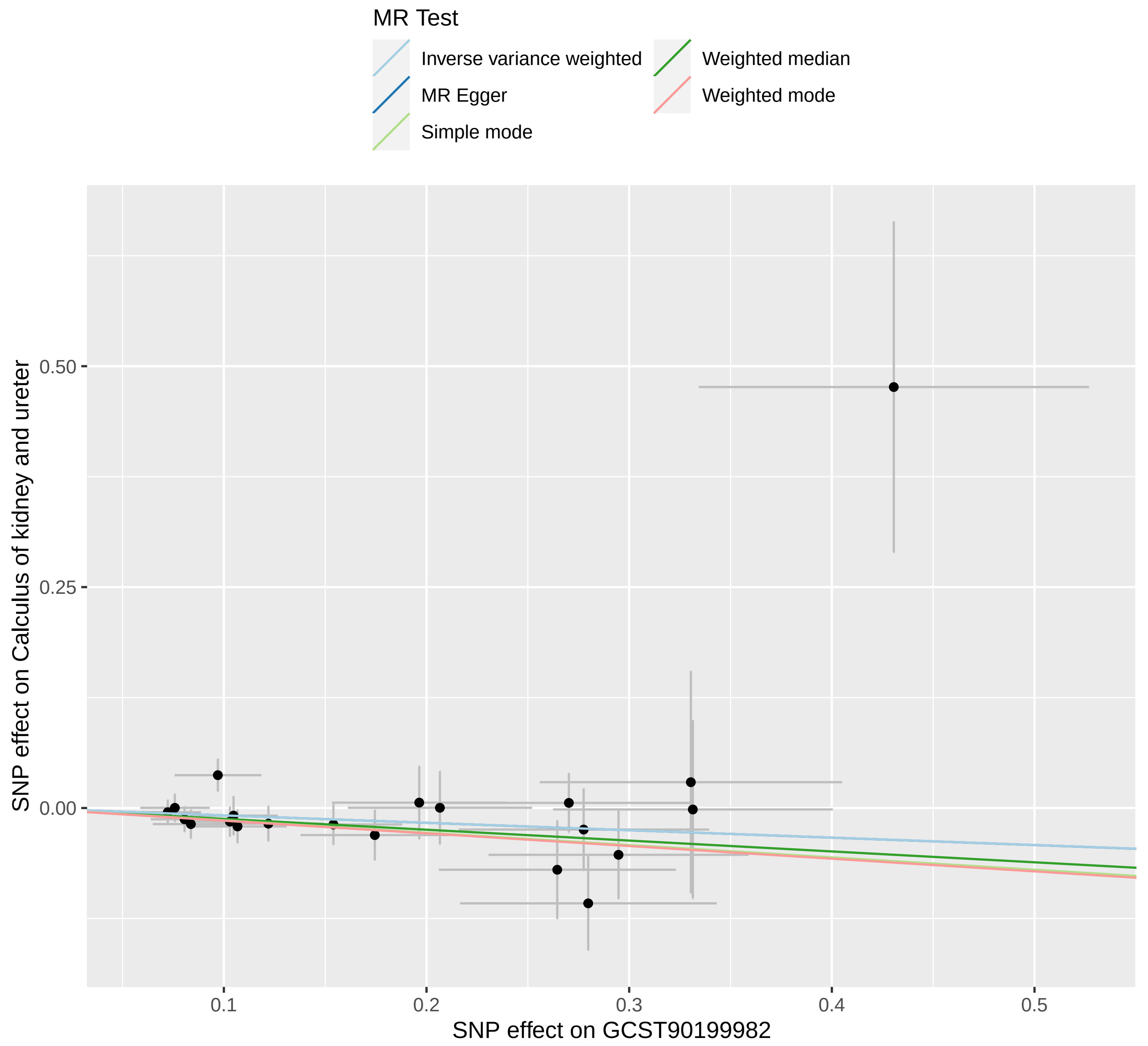

MR Test
Inverse variance weighted
MR Egger
Simple mode
Weighted median
Weighted mode
0.50
0.25
0.00
0.1
0.2
0.3
0.4
0.5
SNP effect on Calculus of kidney and ureter
SNP effect on GCST90199982

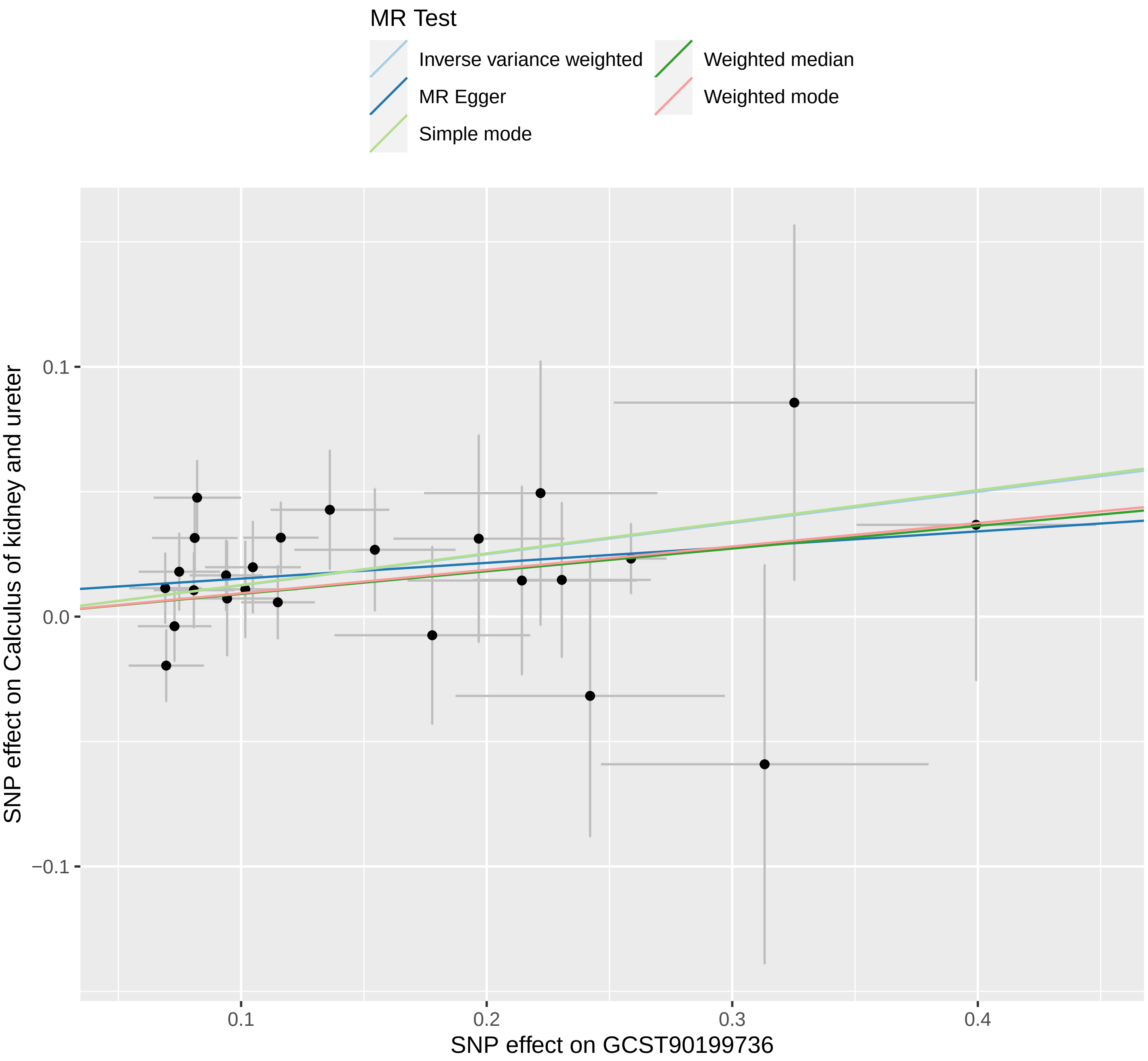
MR Test
Inverse variance weighted
MR Egger
Simple mode
Weighted median
Weighted mode
0.1
0.0
−0.1
0.1
0.2
0.3
0.4
SNP effect on Calculus of kidney and ureter
SNP effect on GCST90199736

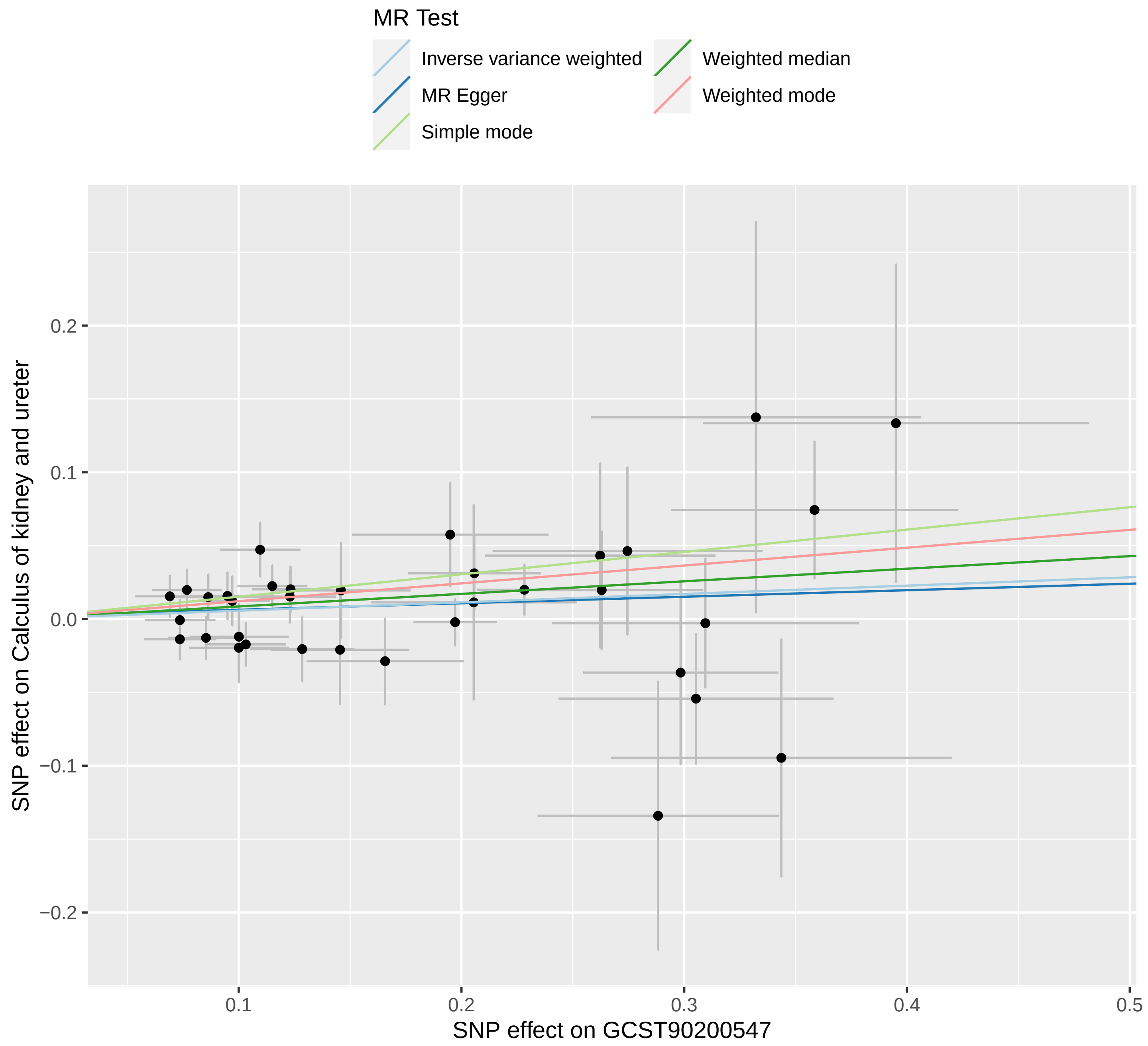

MR Test
Inverse variance weighted
MR Egger
Simple mode
Weighted median
Weighted mode
SNP effect on Calculus of kidney and ureter
0.2
0.1
0.0
−0.1
−0.2
0.1
0.2
0.3
0.4
0.5
SNP effect on GCST90200547

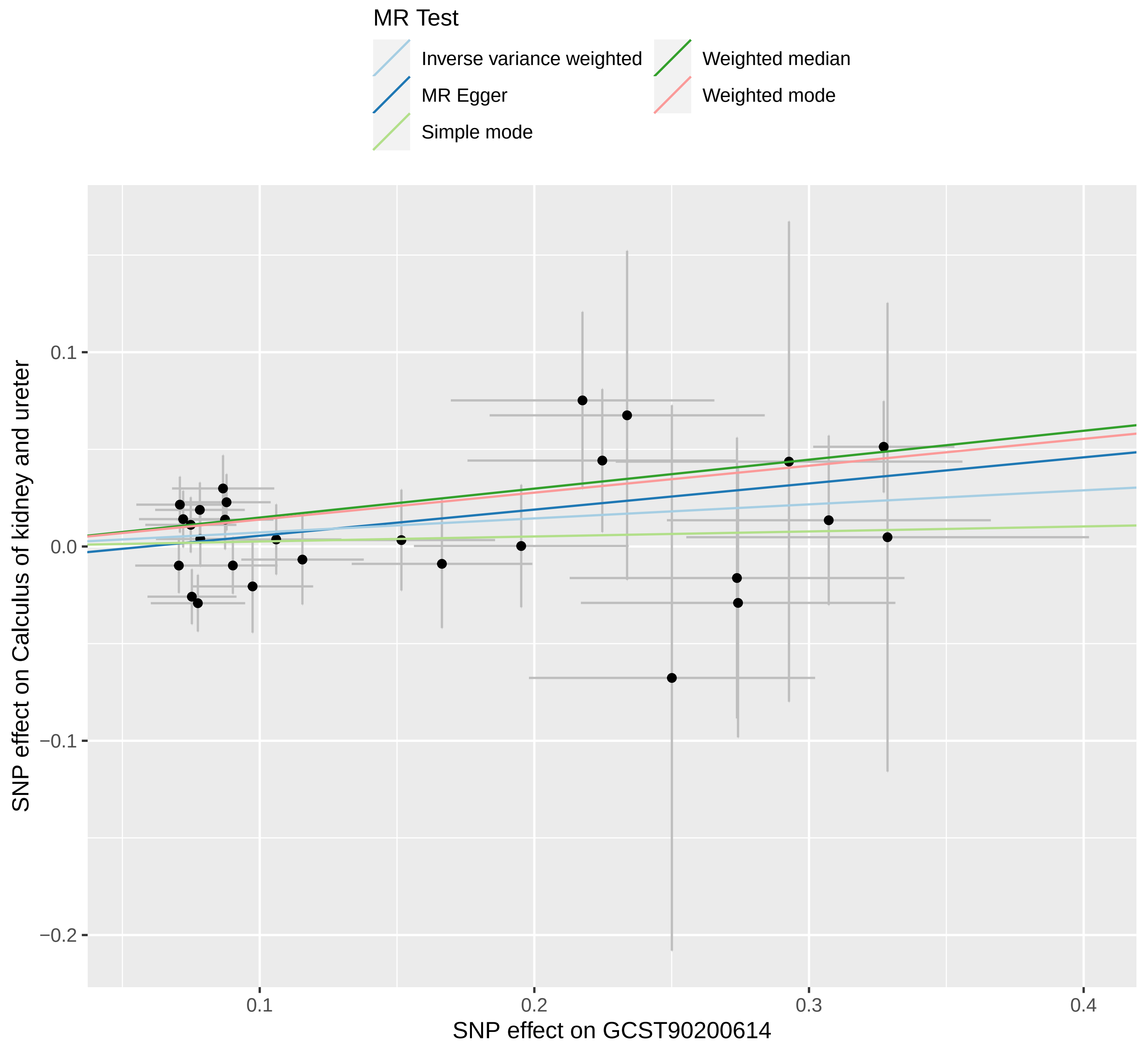

MR Test
Inverse variance weighted
MR Egger
Simple mode
Weighted median
Weighted mode
0.1
0.0
−0.1
−0.2
SNP effect on Calculus of kidney and ureter
0.1
0.2
0.3
0.4
SNP effect on GCST90200614

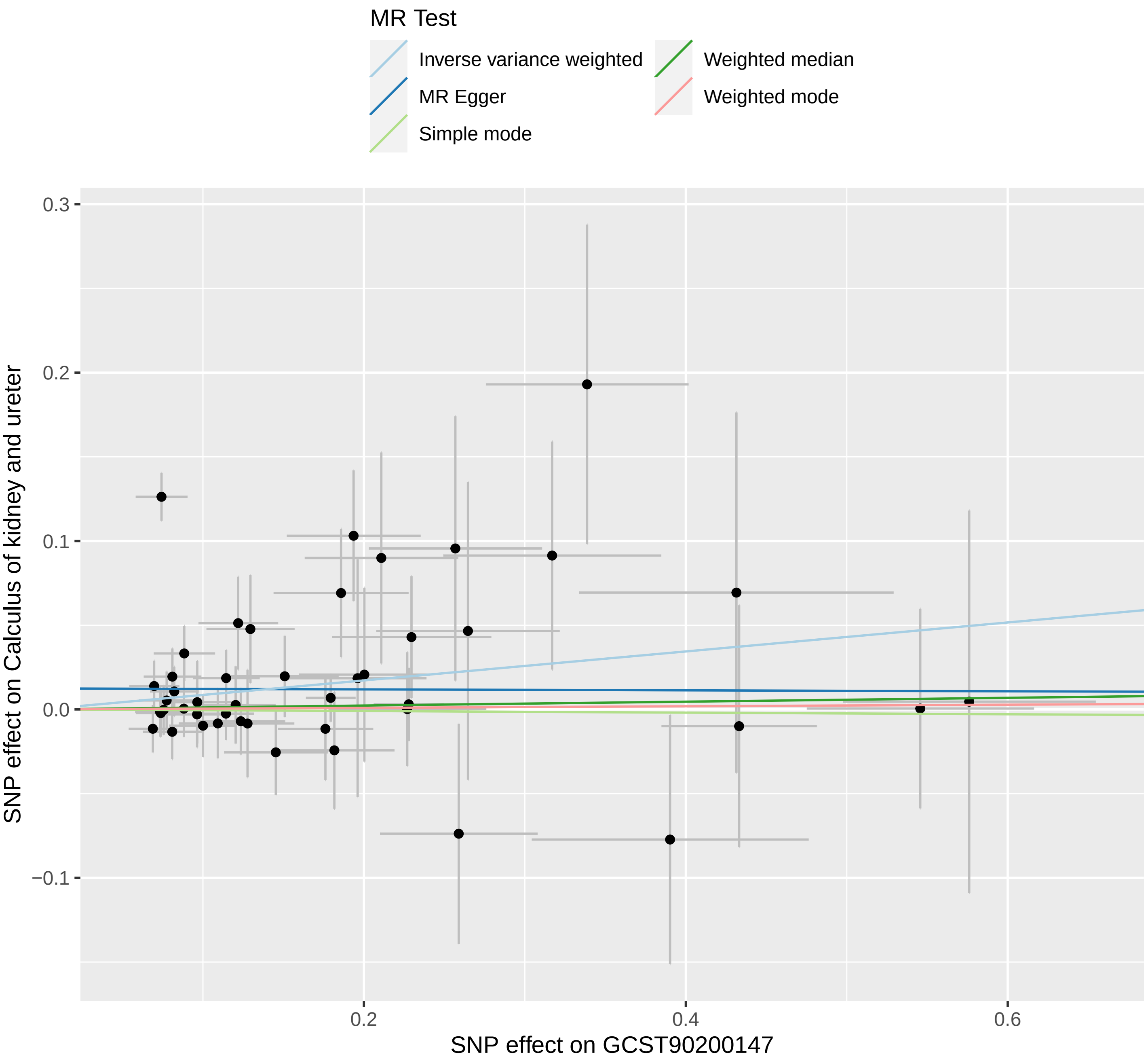
MR Test
Inverse variance weighted
MR Egger
Simple mode
Weighted median
Weighted mode
0.3
0.2
0.1
0.0
−0.1
SNP effect on Calculus of kidney and ureter
0.2
0.4
0.6
SNP effect on GCST90200147

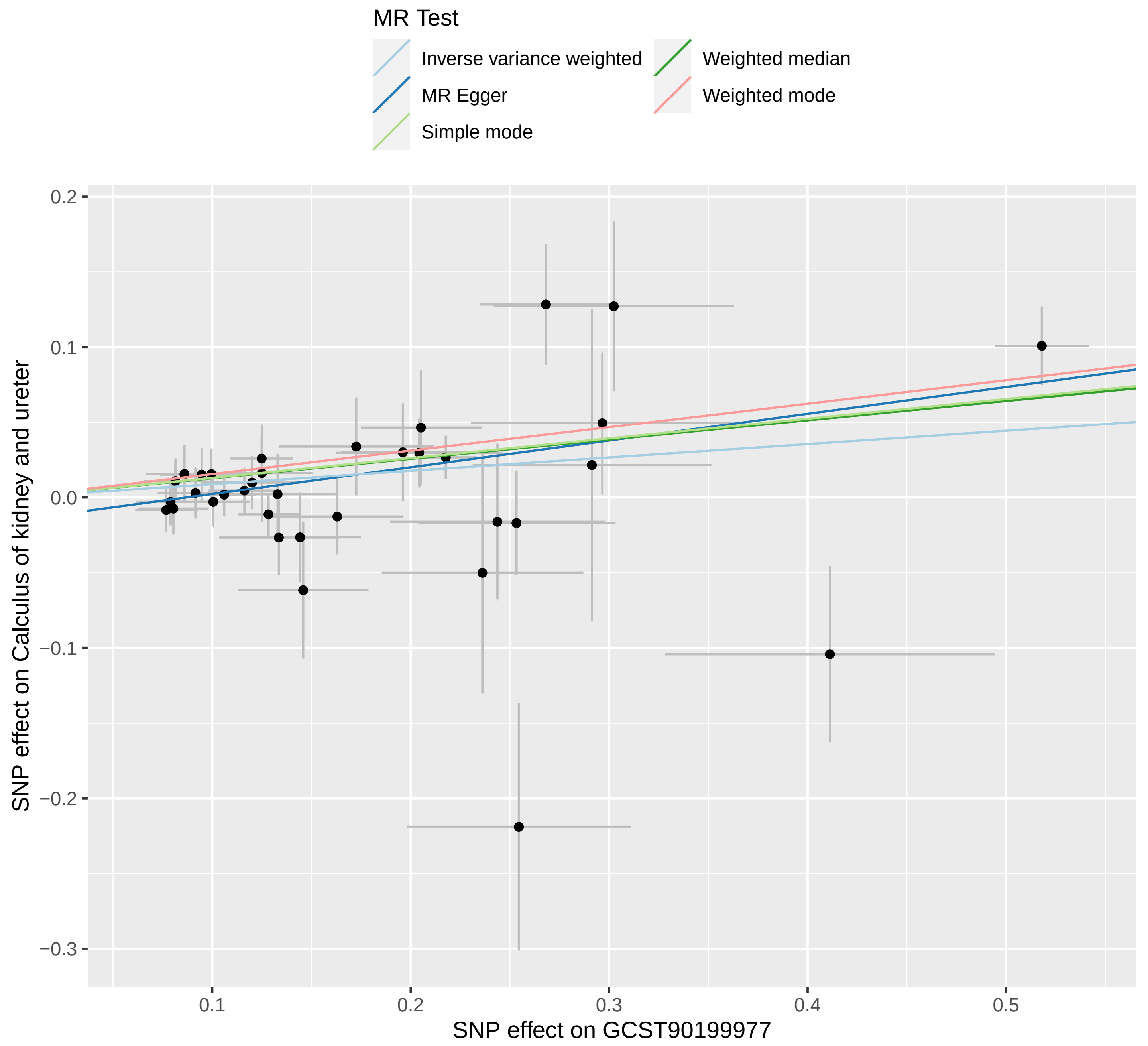

MR Test
Inverse variance weighted
MR Egger
Simple mode
Weighted median
Weighted mode
0.2
0.1
0.0
−0.1
−0.2
−0.3
0.1
0.2
0.3
0.4
0.5
SNP effect on Calculus of kidney and ureter
SNP effect on GCST90199977

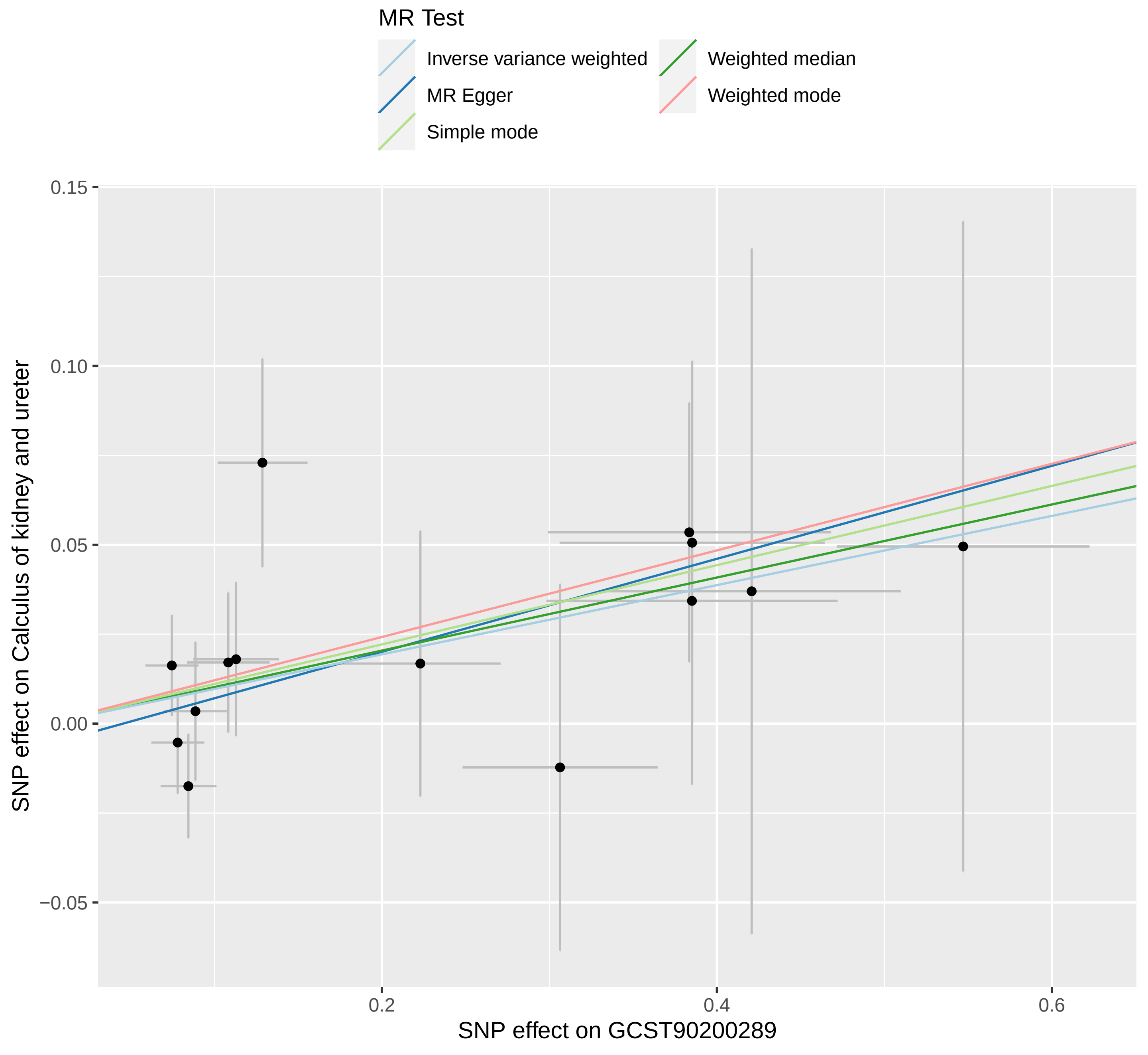
MR Test
Inverse variance weighted
MR Egger
Simple mode
Weighted median
Weighted mode
0.15
0.10
0.05
0.00
−0.05
0.2
0.4
0.6
SNP effect on Calculus of kidney and ureter
SNP effect on GCST90200289

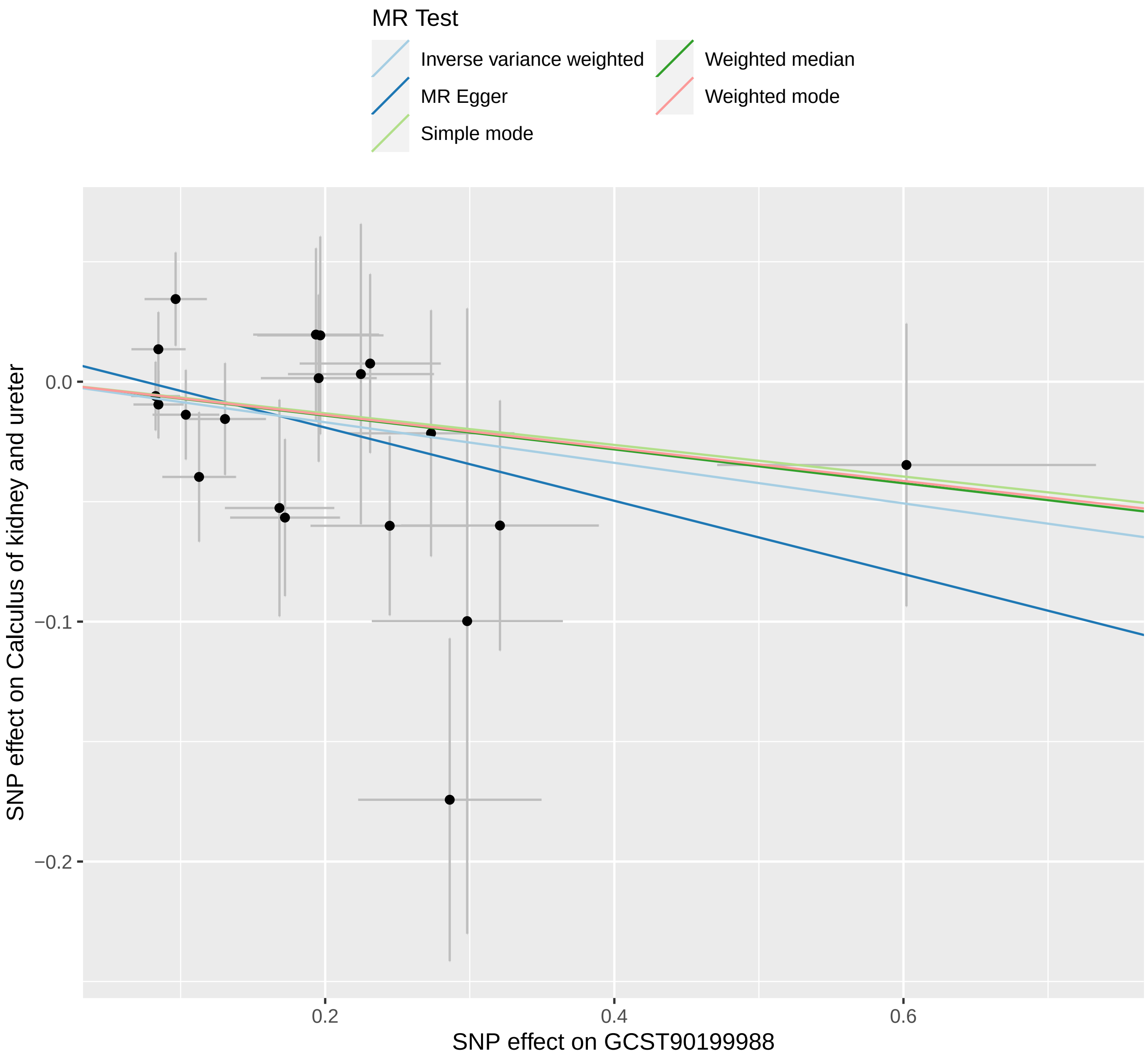
MR Test
Inverse variance weighted
MR Egger
Simple mode
Weighted median
Weighted mode
SNP effect on Calculus of kidney and ureter
0.0
−0.1
−0.2
0.2
0.4
0.6
SNP effect on GCST90199988

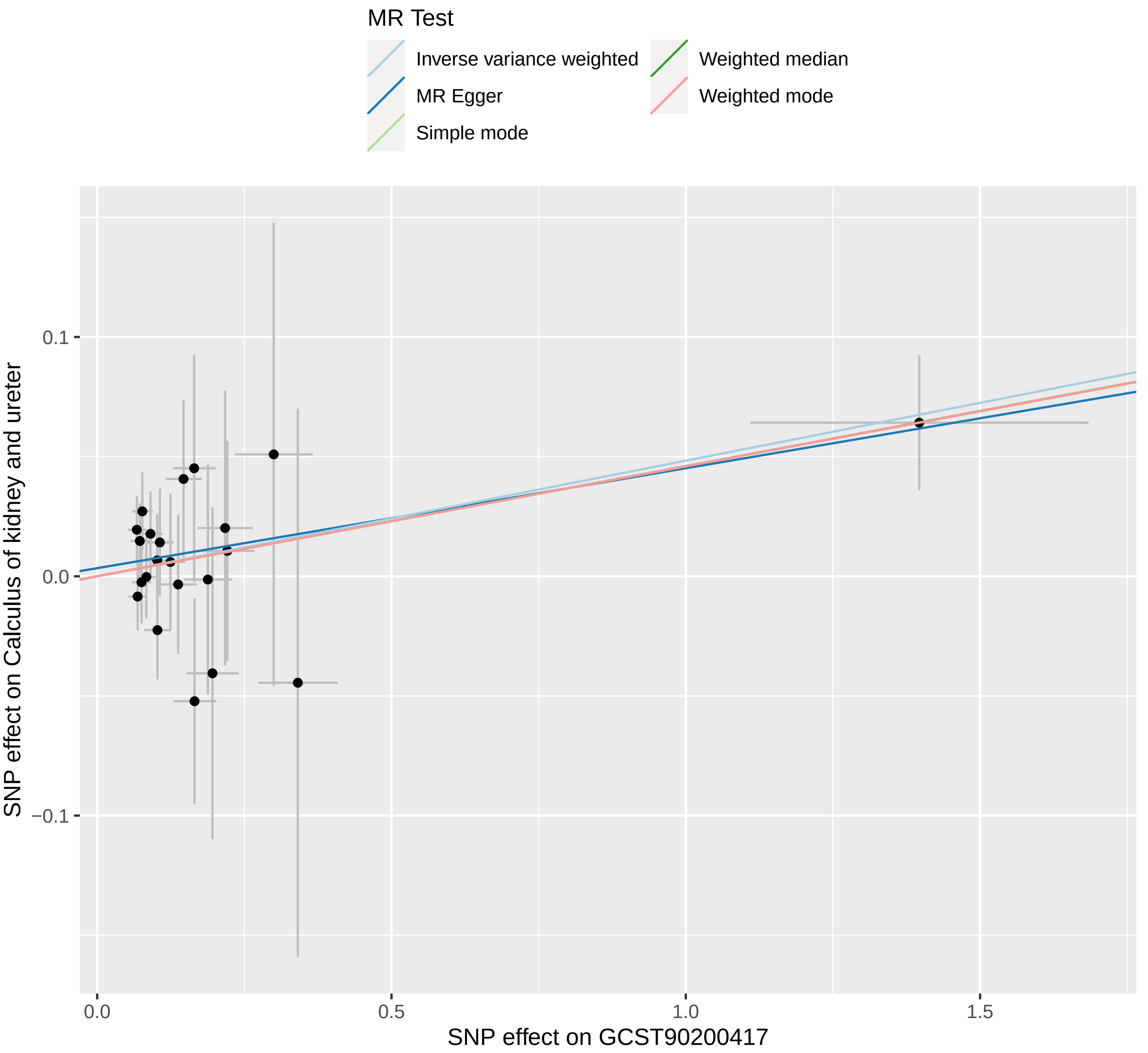

MR Test
Inverse variance weighted
MR Egger
Simple mode
Weighted median
Weighted mode
0.1
0.0
−0.1
0.0
0.5
1.0
1.5
SNP effect on Calculus of kidney and ureter
SNP effect on GCST90200417

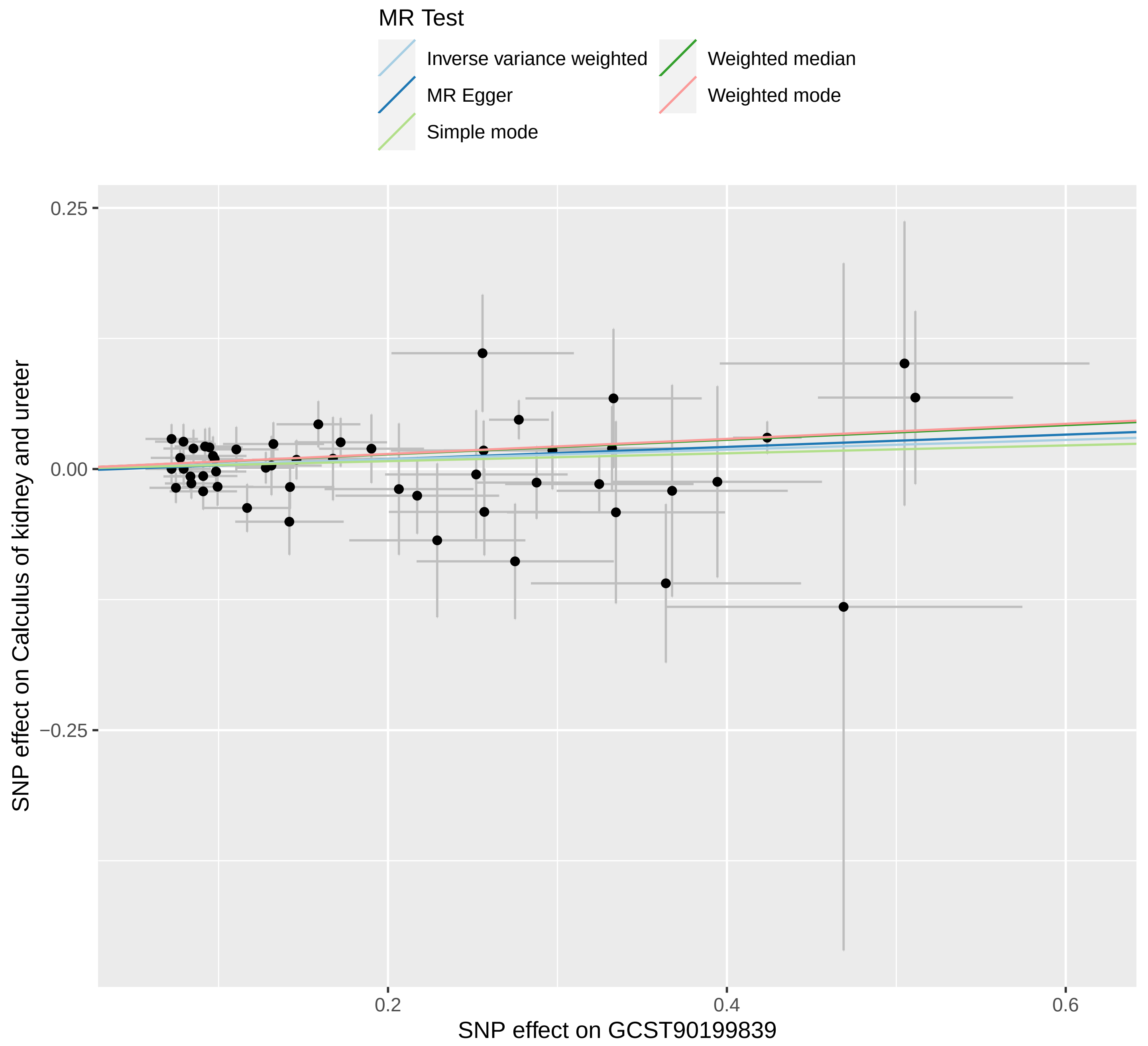
MR Test
Inverse variance weighted
MR Egger
Simple mode
Weighted median
Weighted mode
0.25
0.00
−0.25
0.2
0.4
0.6
SNP effect on Calculus of kidney and ureter
SNP effect on GCST90199839

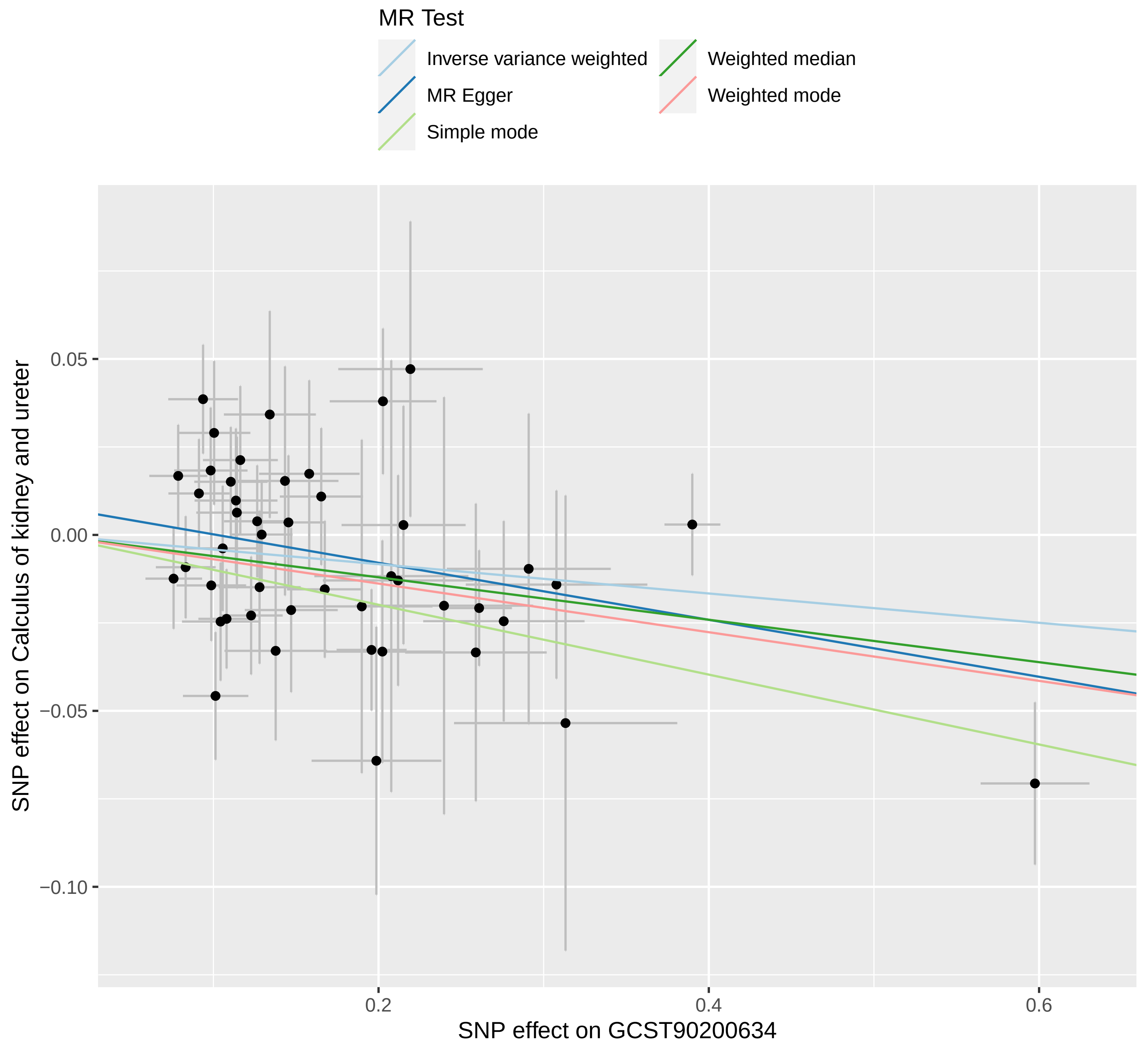
MR Test
Inverse variance weighted
MR Egger
Simple mode
Weighted median
Weighted mode
0.05
0.00
−0.05
−0.10
0.2
0.4
0.6
SNP effect on Calculus of kidney and ureter
SNP effect on GCST90200634

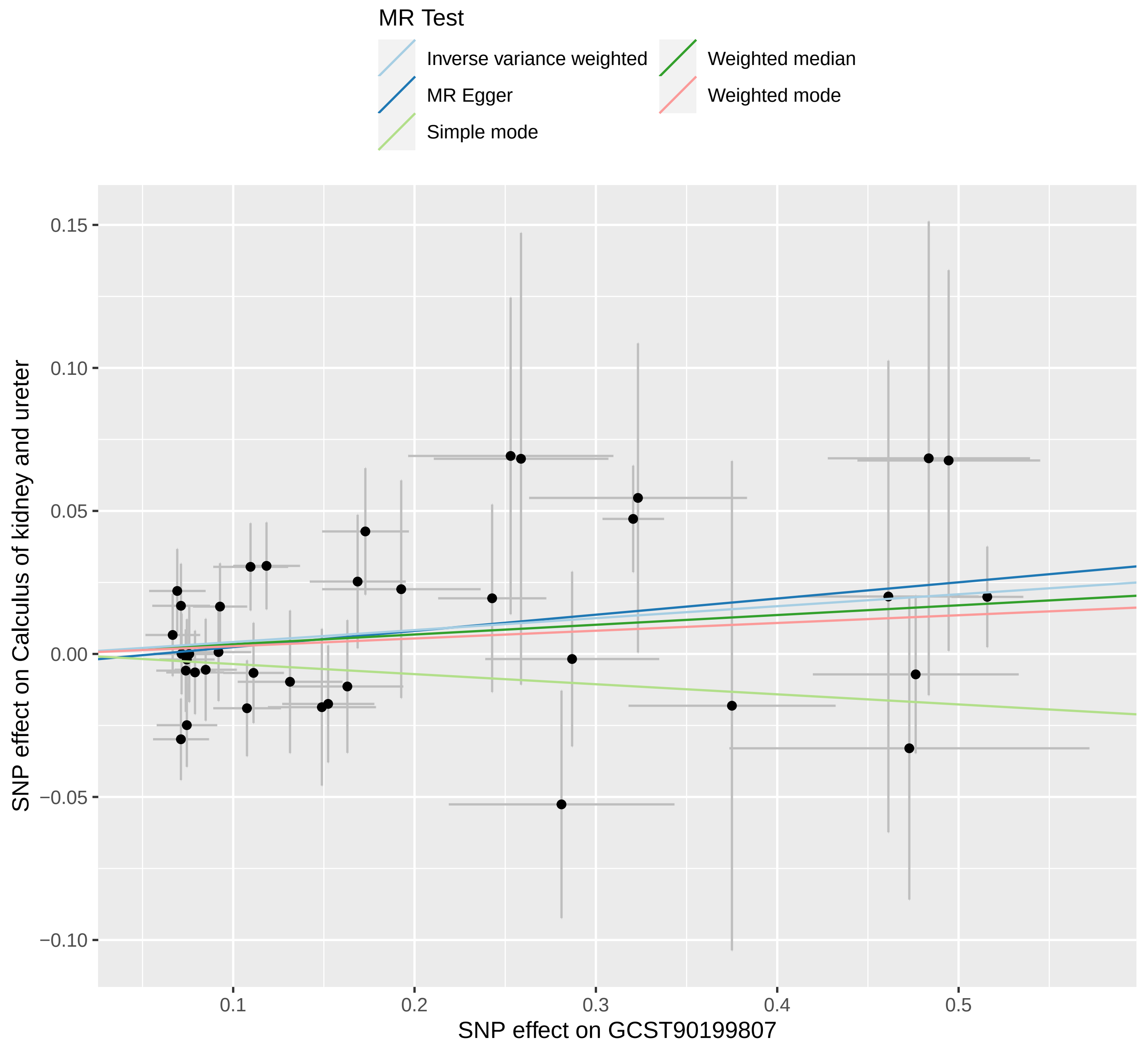

MR Test
Inverse variance weighted
MR Egger
Simple mode
Weighted median
Weighted mode
0.15
0.10
0.05
0.00
−0.05
−0.10
SNP effect on Calculus of kidney and ureter
0.1
0.2
0.3
0.4
0.5
SNP effect on GCST90199807

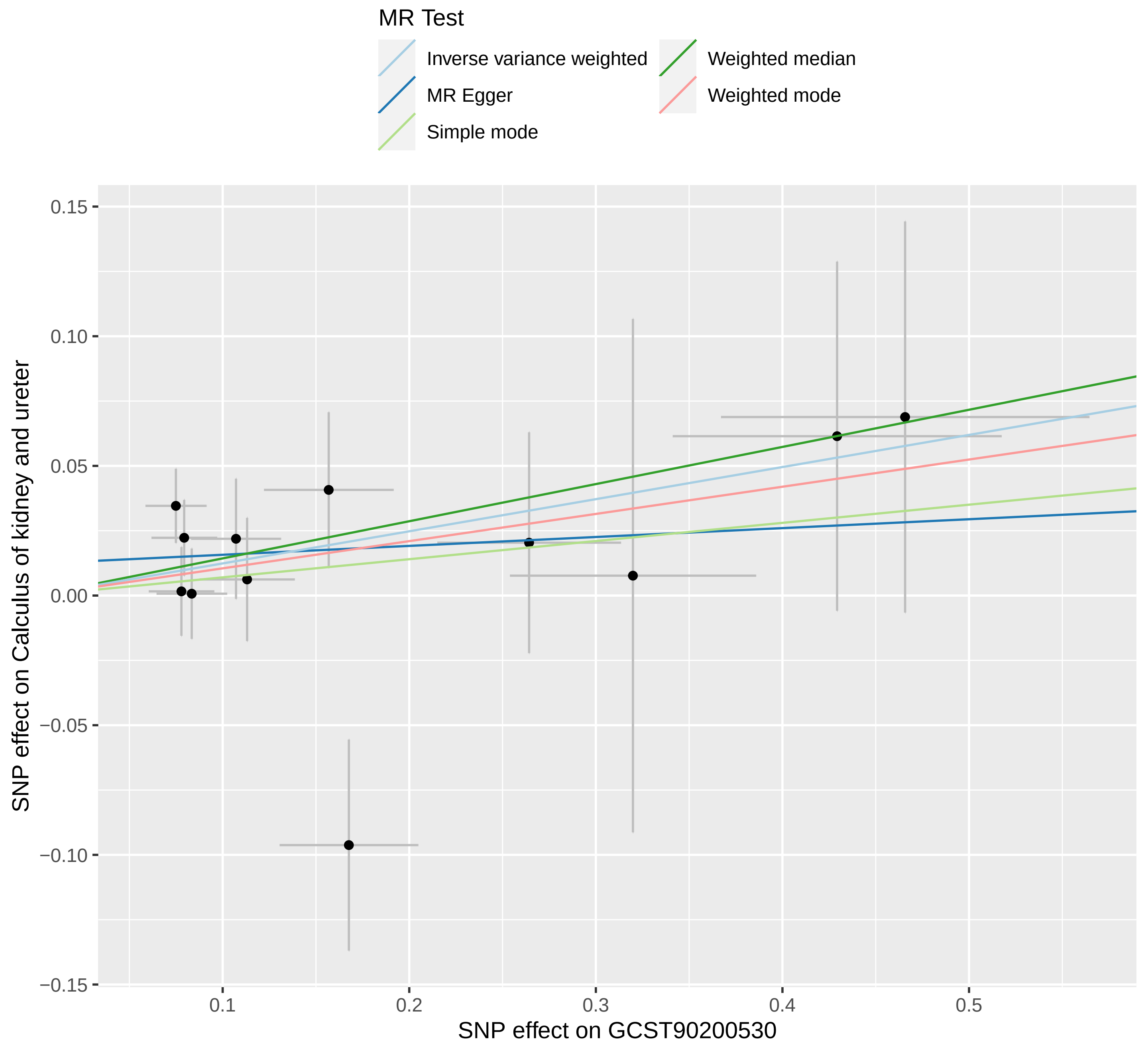
MR Test
Inverse variance weighted
MR Egger
Simple mode
Weighted median
Weighted mode
SNP effect on Calculus of kidney and ureter
SNP effect on GCST90200530
0.15
0.10
0.05
0.00
−0.05
−0.10
−0.15
0.1
0.2
0.3
0.4
0.5

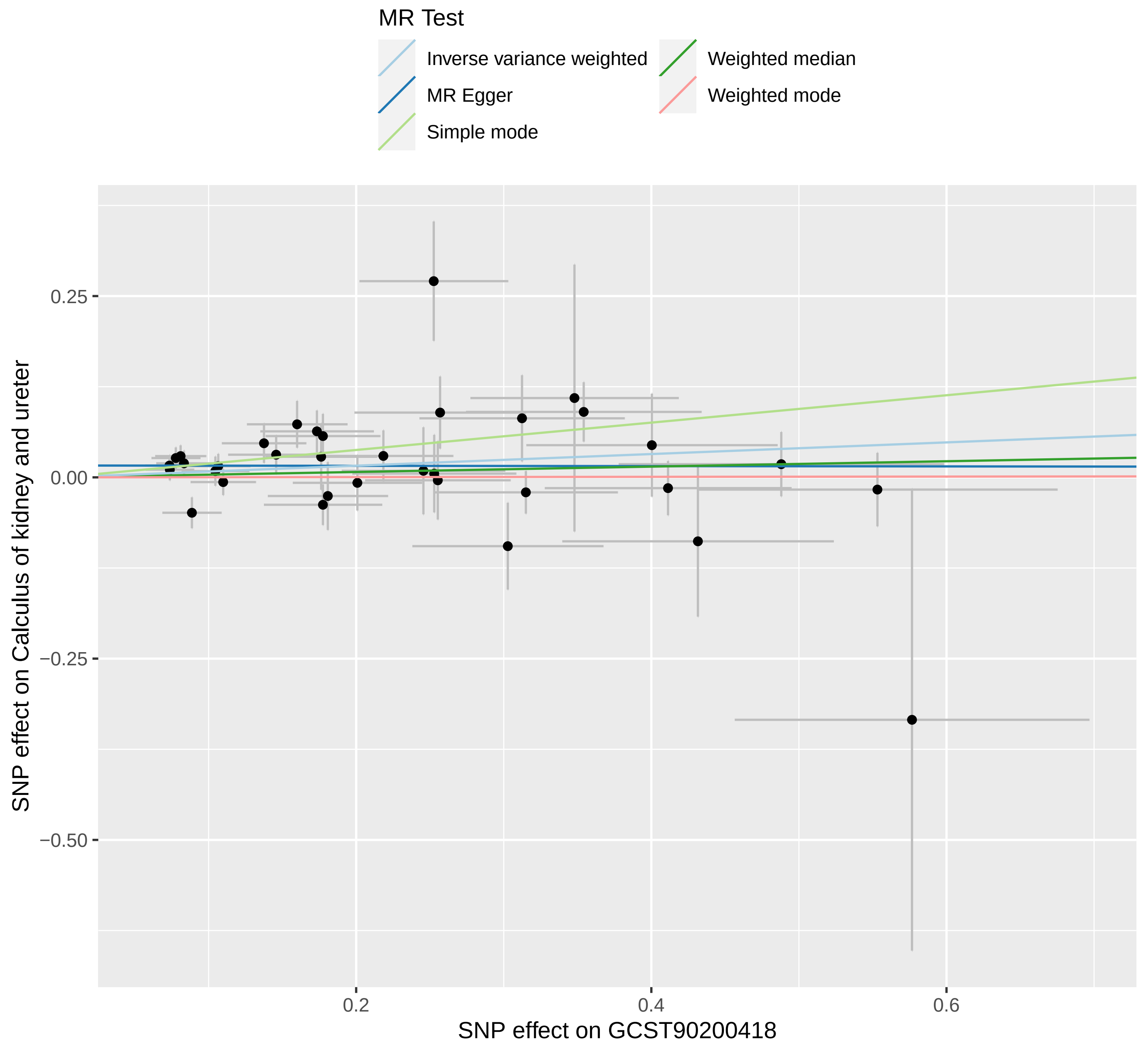
MR Test
Inverse variance weighted
MR Egger
Simple mode
Weighted median
Weighted mode
0.25
0.00
−0.25
−0.50
0.2
0.4
0.6
SNP effect on Calculus of kidney and ureter
SNP effect on GCST90200418

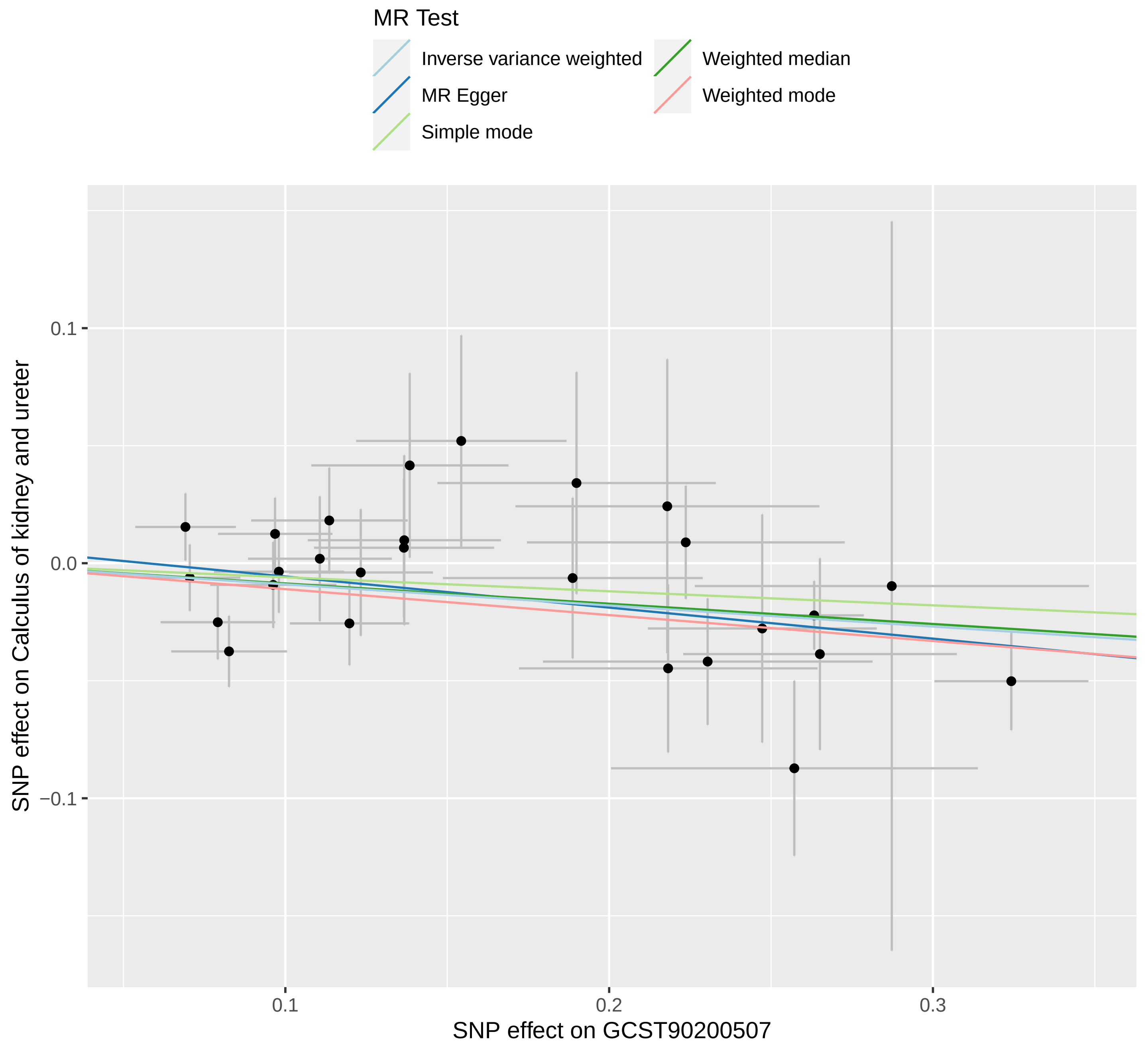
MR Test
Inverse variance weighted
MR Egger
Simple mode
Weighted median
Weighted mode
0.1
0.0
−0.1
SNP effect on Calculus of kidney and ureter
0.1
0.2
0.3
SNP effect on GCST90200507

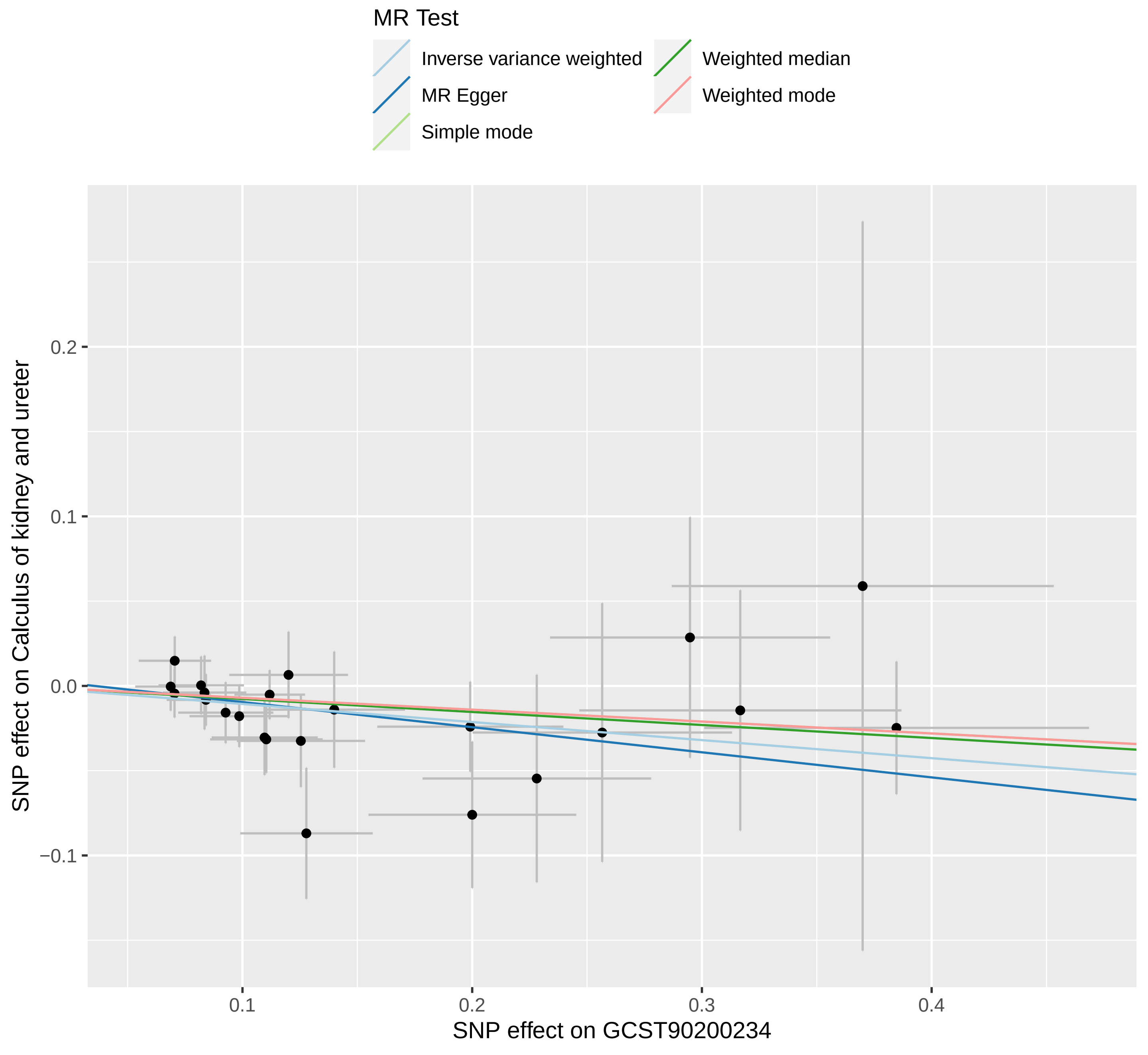

MR Test
Inverse variance weighted
MR Egger
Simple mode
Weighted median
Weighted mode
SNP effect on Calculus of kidney and ureter
0.2
0.1
0.0
−0.1
0.1
0.2
0.3
0.4
SNP effect on GCST90200234

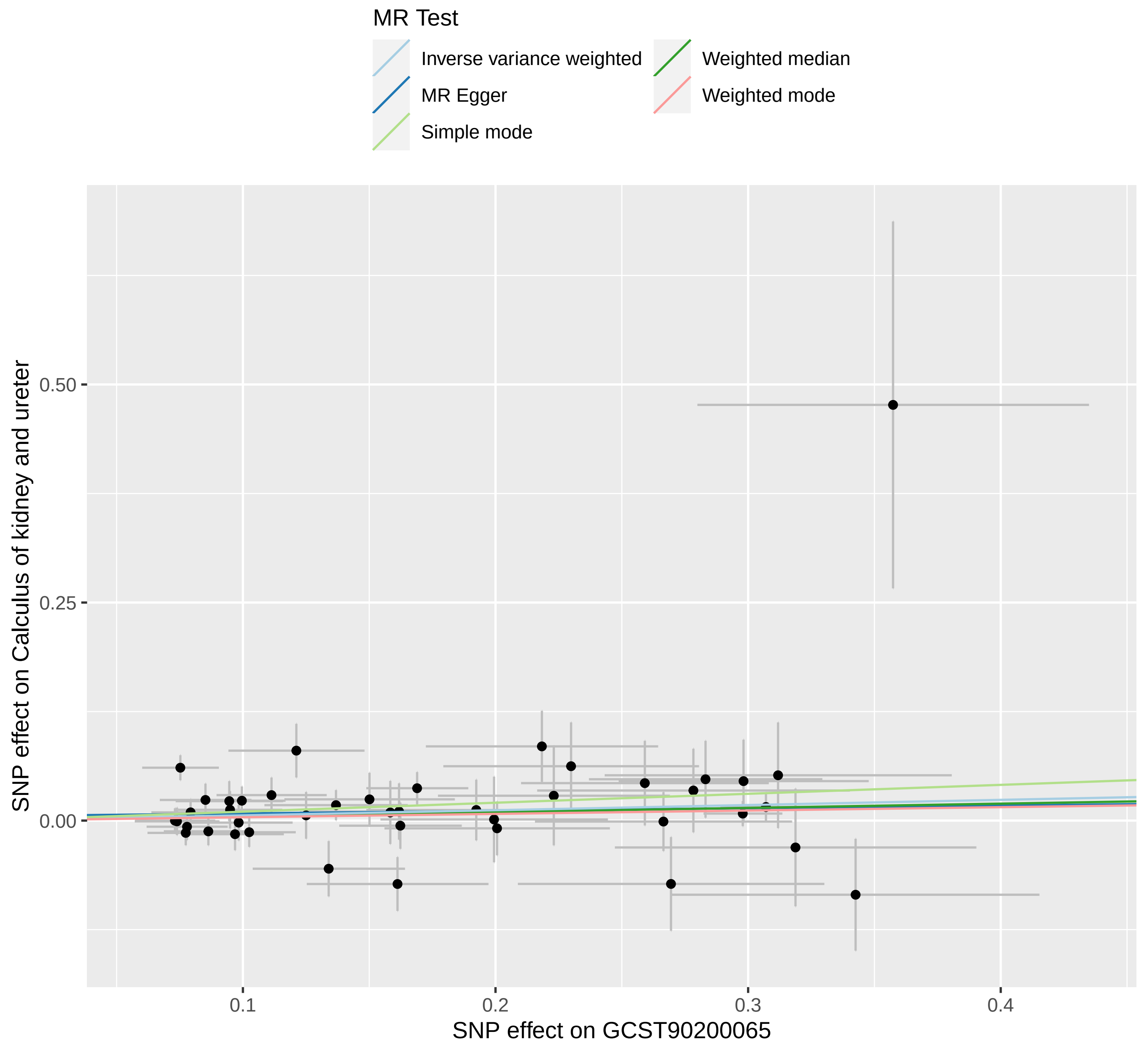
MR Test
Inverse variance weighted
MR Egger
Simple mode
Weighted median
Weighted mode
SNP effect on Calculus of kidney and ureter
0.50
0.25
0.00
0.1
0.2
0.3
0.4
SNP effect on GCST90200065

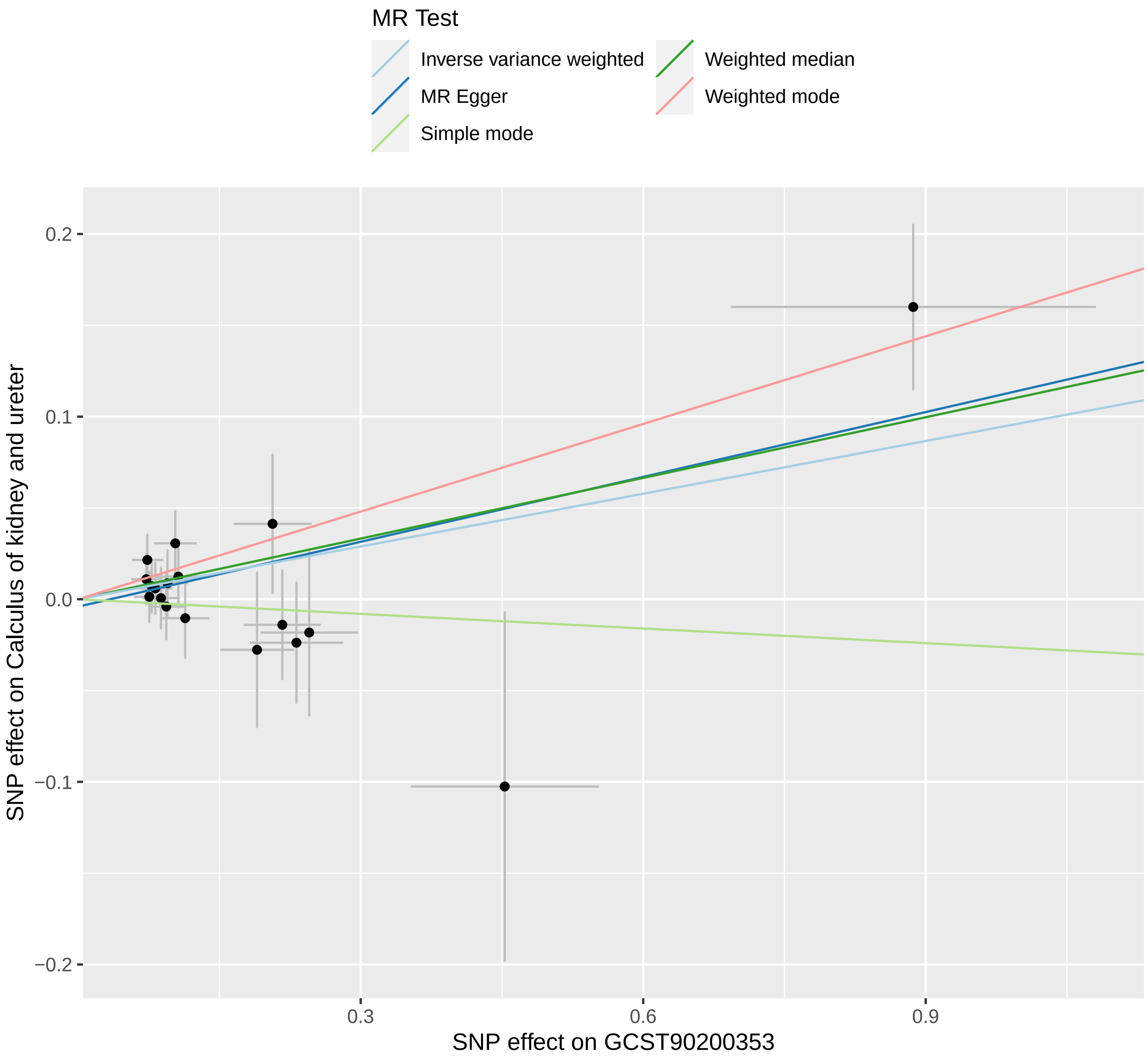

MR Test
Inverse variance weighted
MR Egger
Simple mode
Weighted median
Weighted mode
SNP effect on Calculus of kidney and ureter
0.2
0.1
0.0
−0.1
−0.2
0.3
0.6
0.9
SNP effect on GCST90200353

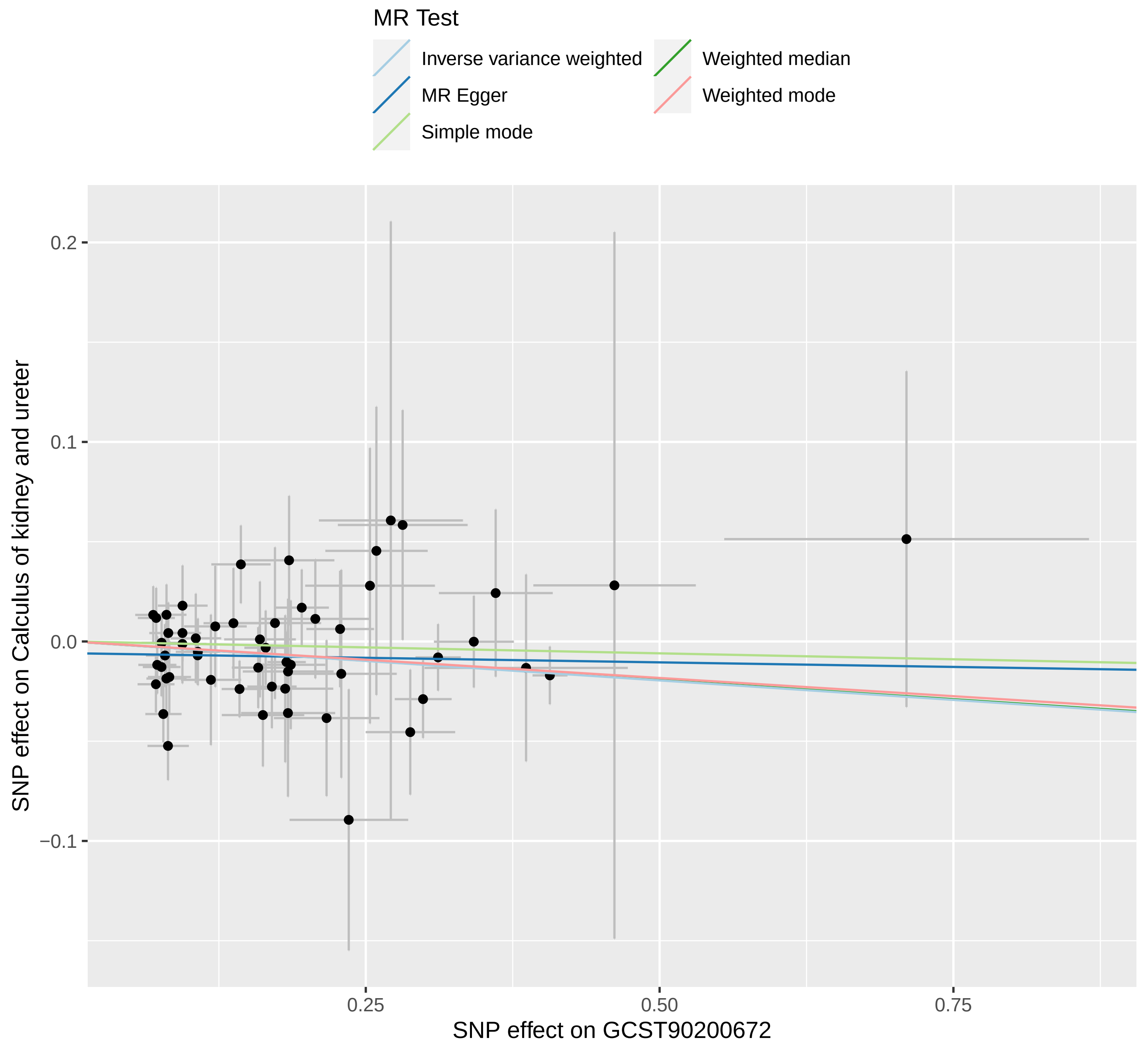
MR Test
Inverse variance weighted
MR Egger
Simple mode
Weighted median
Weighted mode
SNP effect on Calculus of kidney and ureter
SNP effect on GCST90200672
0.2
0.1
0.0
−0.1
0.25
0.50
0.75

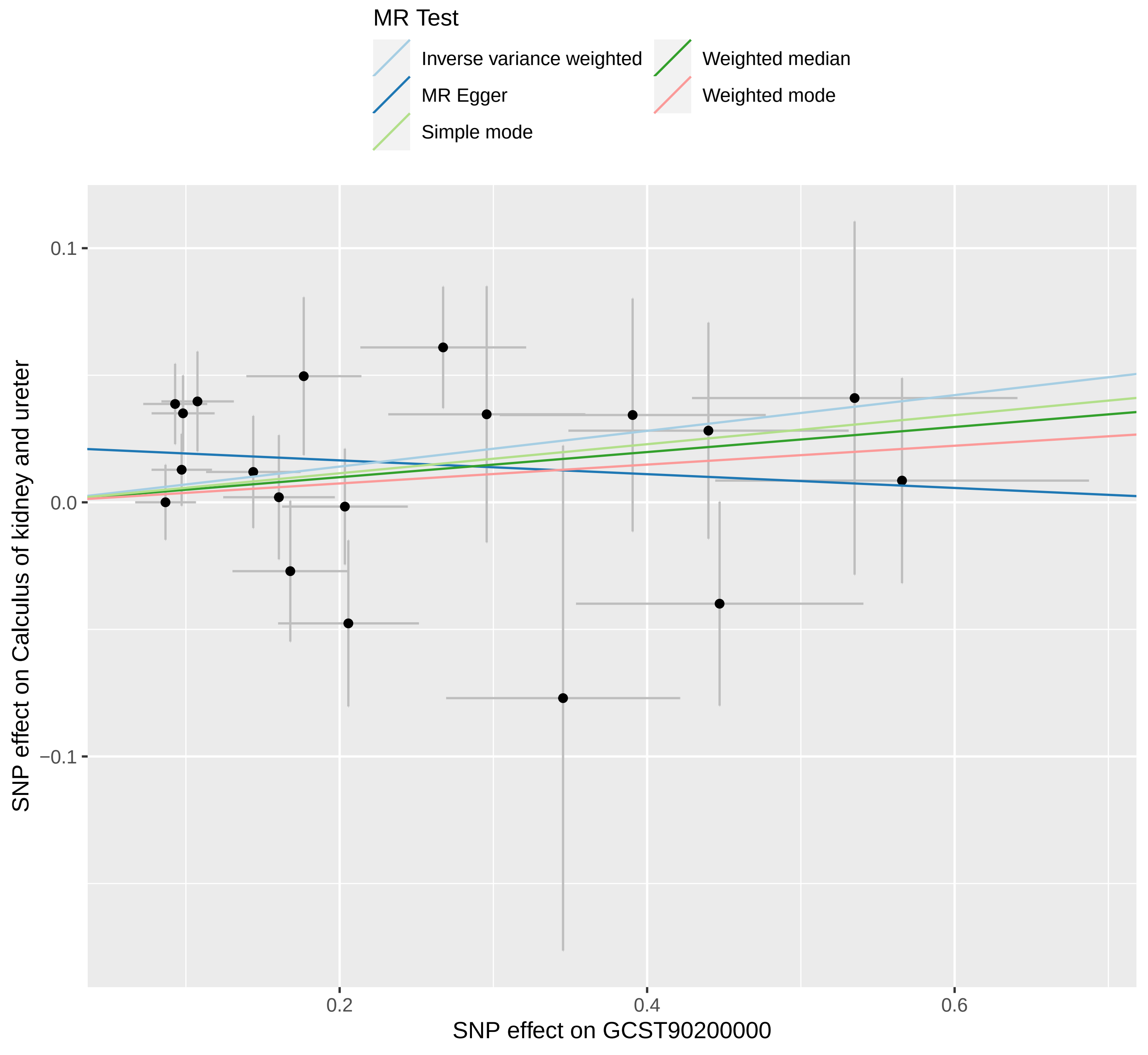

MR Test
Inverse variance weighted
MR Egger
Simple mode
Weighted median
Weighted mode
0.1
0.0
−0.1
0.2
0.4
0.6
SNP effect on GCST90200000
SNP effect on Calculus of kidney and ureter

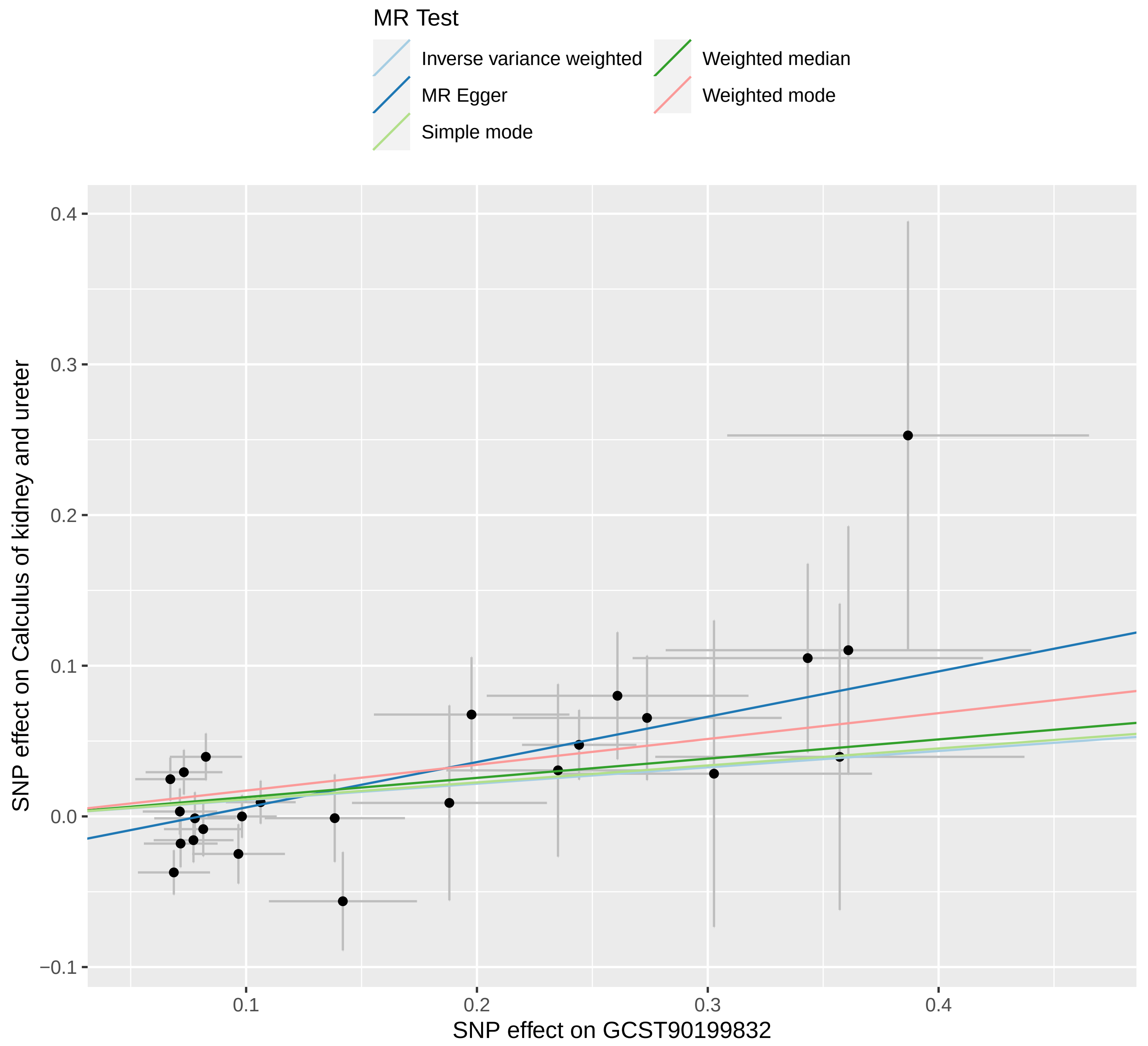

MR Test
Inverse variance weighted
MR Egger
Simple mode
Weighted median
Weighted mode
0.4
0.3
0.2
0.1
0.0
−0.1
0.1
0.2
0.3
0.4
SNP effect on Calculus of kidney and ureter
SNP effect on GCST90199832

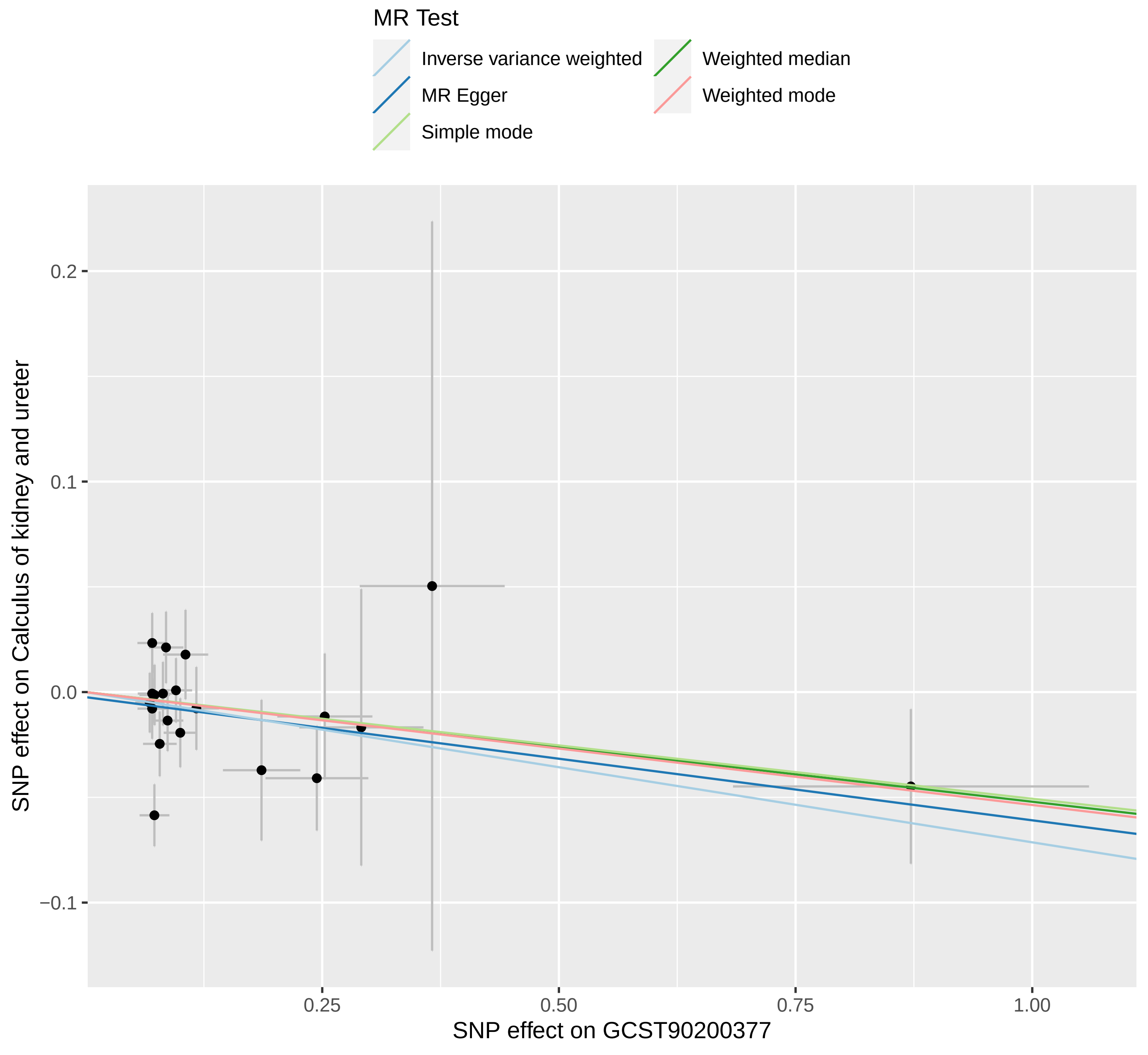

MR Test
Inverse variance weighted
MR Egger
Simple mode
Weighted median
Weighted mode
SNP effect on Calculus of kidney and ureter
0.2
0.1
0.0
−0.1
0.25
0.50
0.75
1.00
SNP effect on GCST90200377

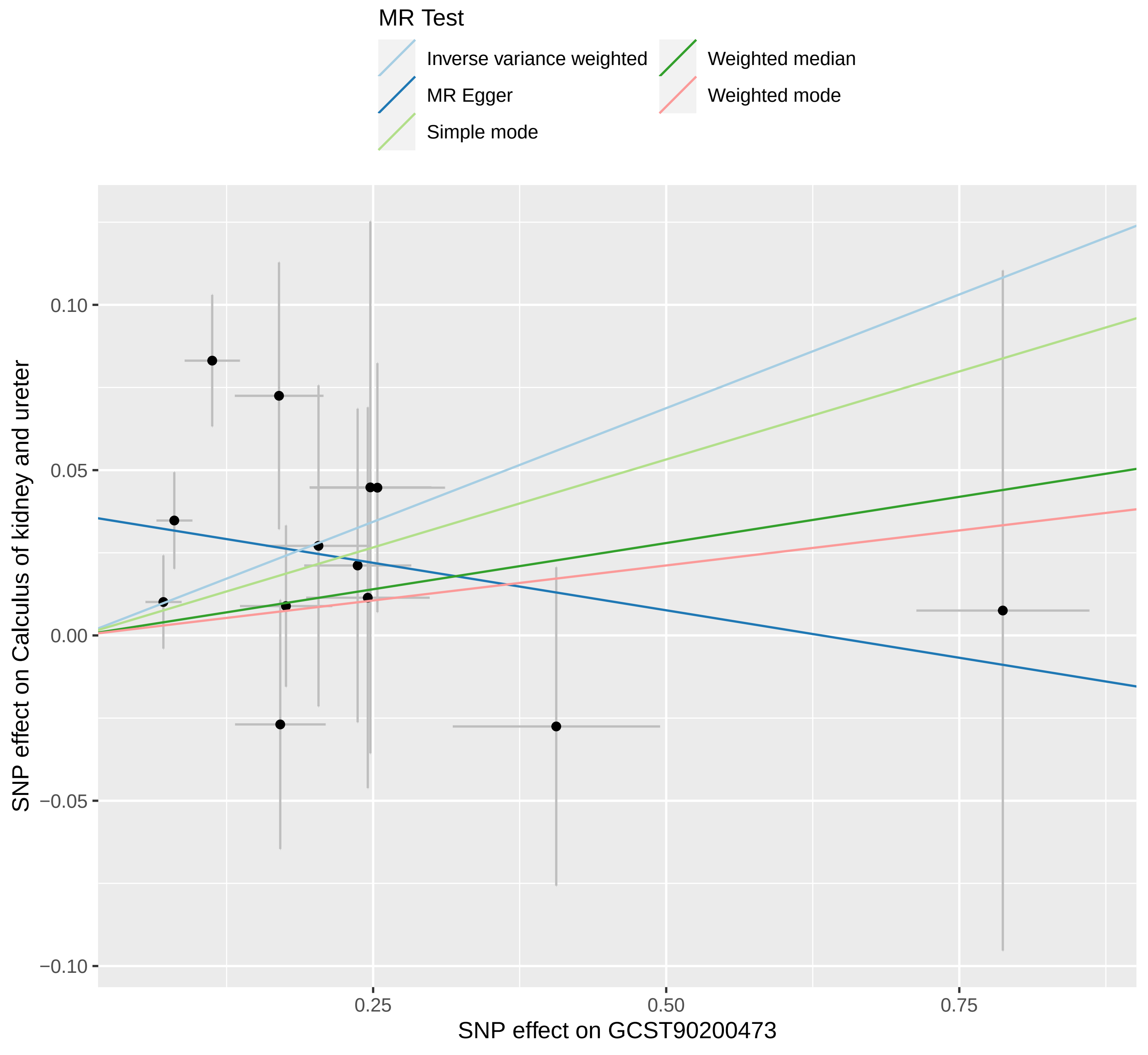
MR Test
Inverse variance weighted
MR Egger
Simple mode
Weighted median
Weighted mode
0.10
0.05
0.00
−0.05
−0.10
0.25
0.50
0.75
SNP effect on GCST90200473
SNP effect on Calculus of kidney and ureter

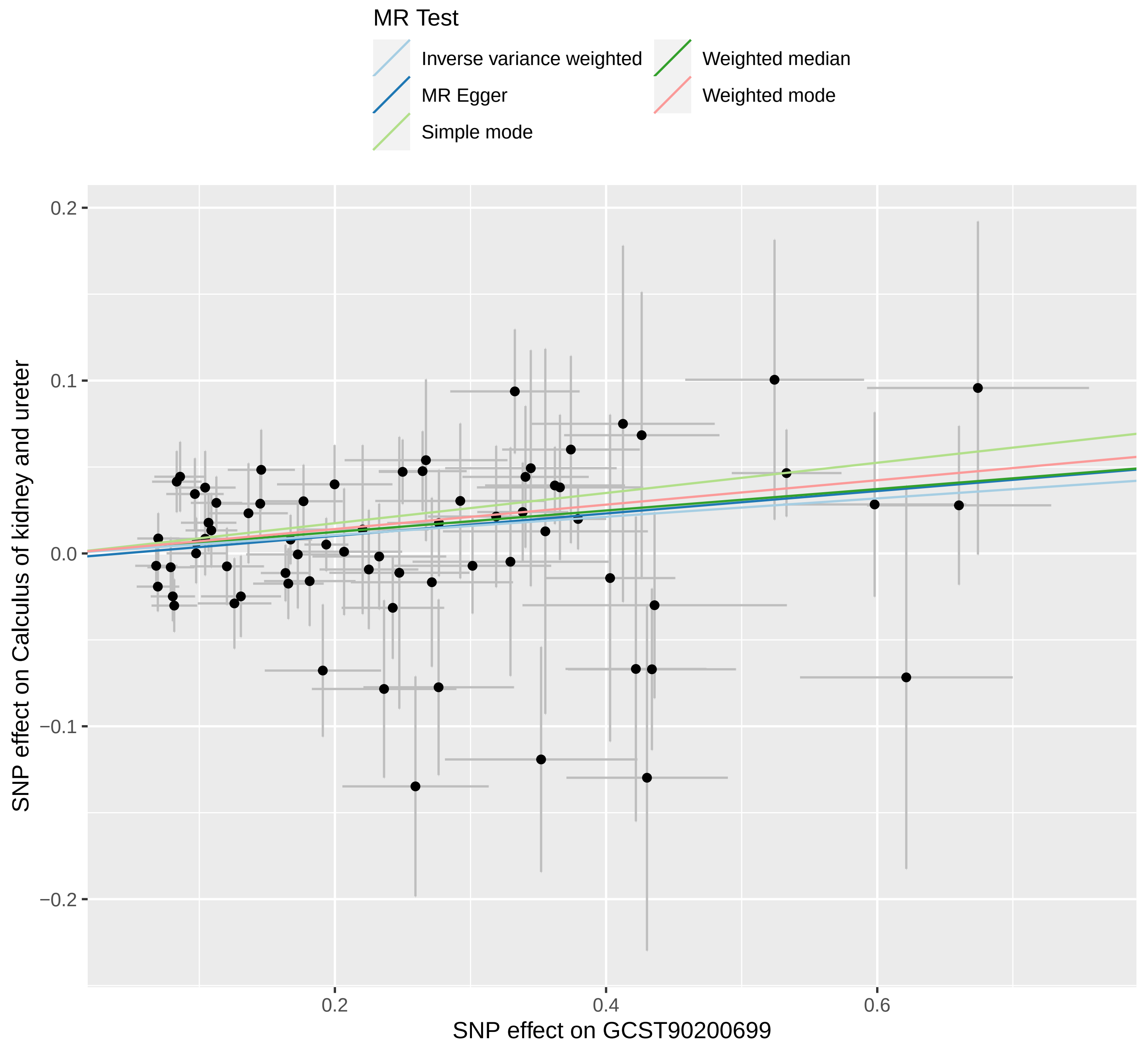

MR Test
Inverse variance weighted
MR Egger
Simple mode
Weighted median
Weighted mode
0.2
0.1
0.0
−0.1
−0.2
SNP effect on Calculus of kidney and ureter
0.2
0.4
0.6
SNP effect on GCST90200699

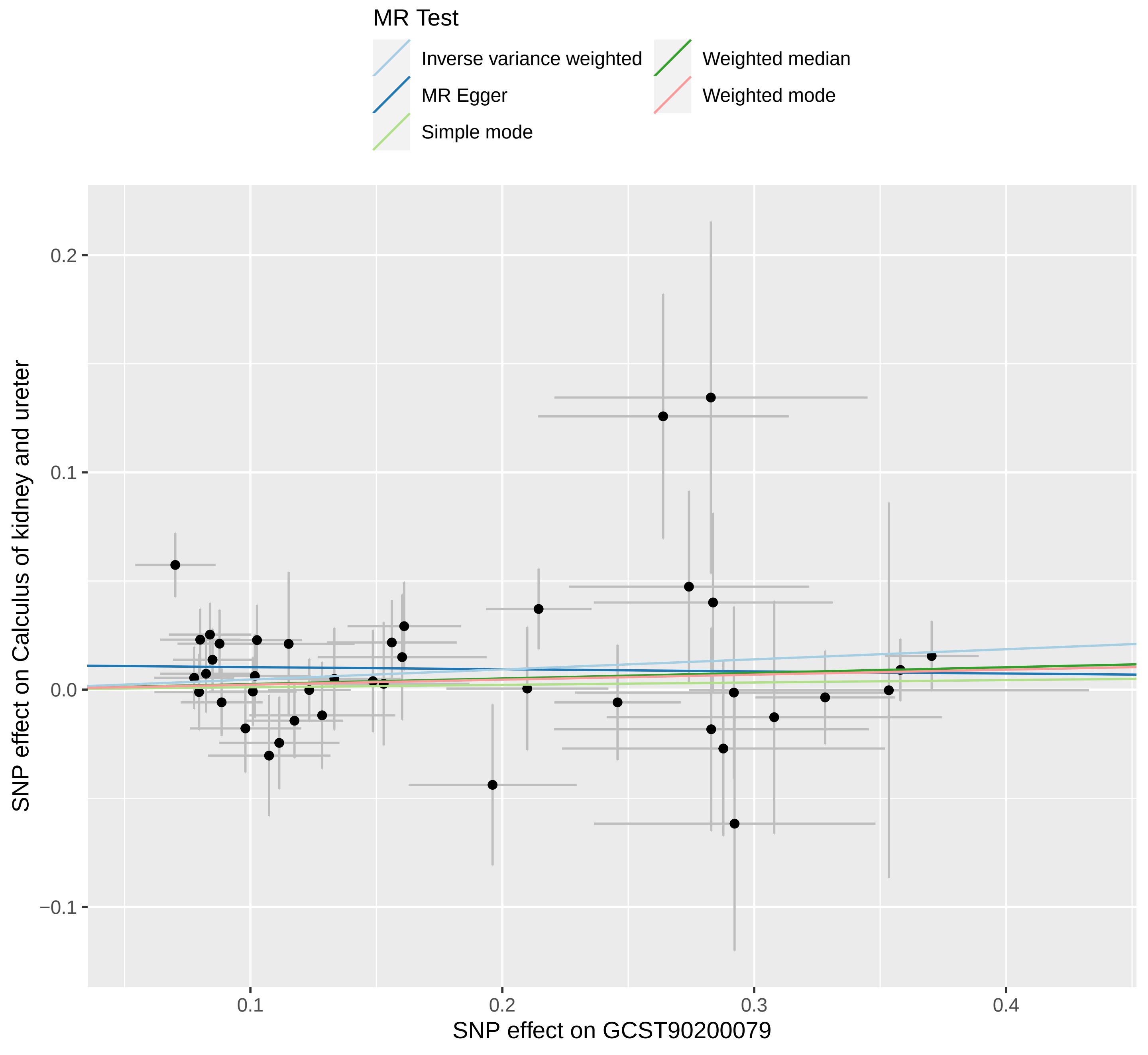

MR Test
Inverse variance weighted
MR Egger
Simple mode
Weighted median
Weighted mode
0.2
0.1
0.0
−0.1
0.1
0.2
0.3
0.4
SNP effect on Calculus of kidney and ureter
SNP effect on GCST90200079

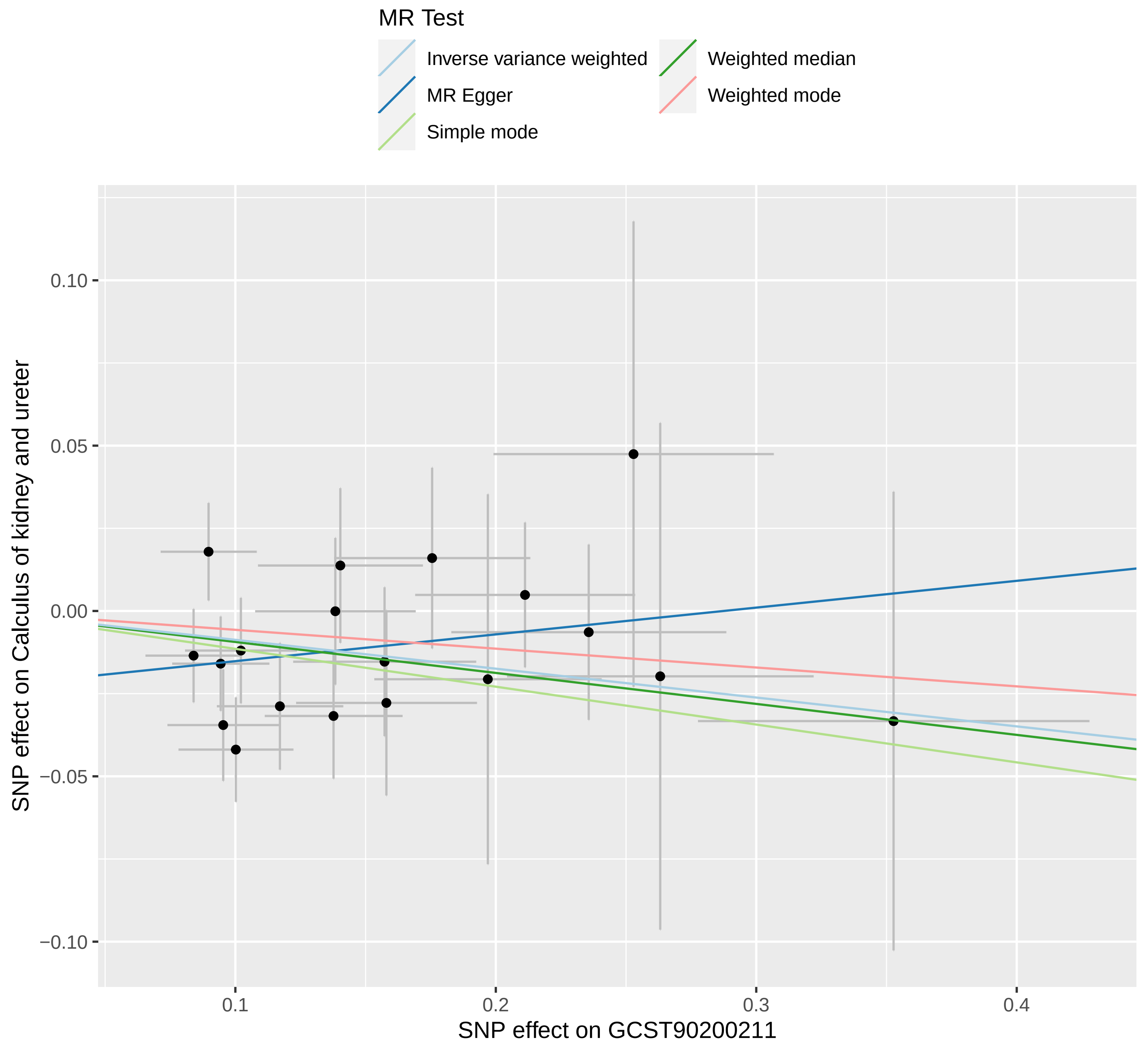
MR Test
Inverse variance weighted
MR Egger
Simple mode
Weighted median
Weighted mode
0.10
0.05
0.00
−0.05
−0.10
0.1
0.2
0.3
0.4
SNP effect on Calculus of kidney and ureter
SNP effect on GCST90200211

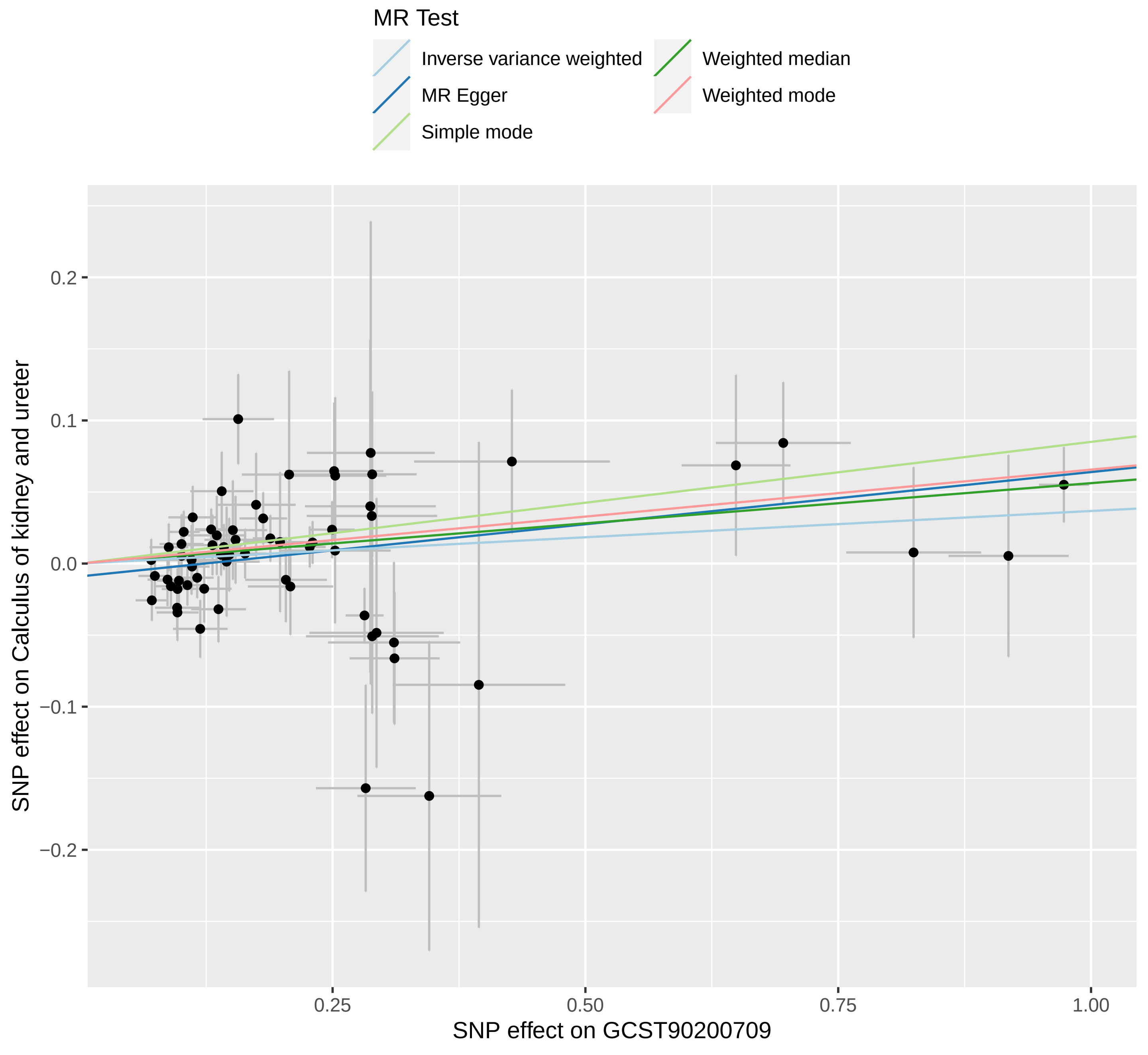

MR Test
Inverse variance weighted
MR Egger
Simple mode
Weighted median
Weighted mode
0.2
0.1
0.0
−0.1
−0.2
0.25
0.50
0.75
1.00
SNP effect on Calculus of kidney and ureter
SNP effect on GCST90200709

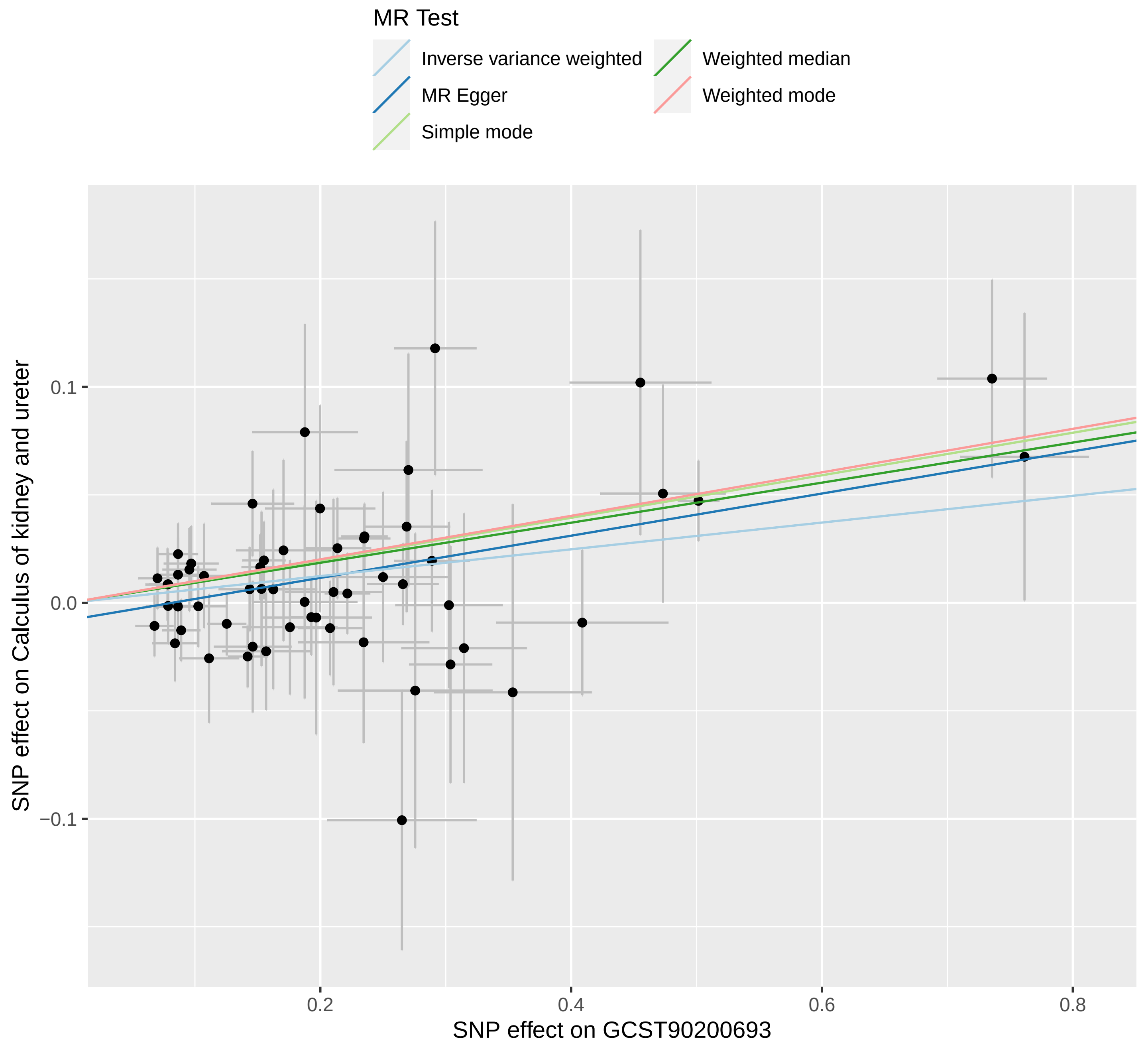
MR Test
Inverse variance weighted
MR Egger
Simple mode
Weighted median
Weighted mode
0.1
0.0
−0.1
0.2
0.4
0.6
0.8
SNP effect on Calculus of kidney and ureter
SNP effect on GCST90200693

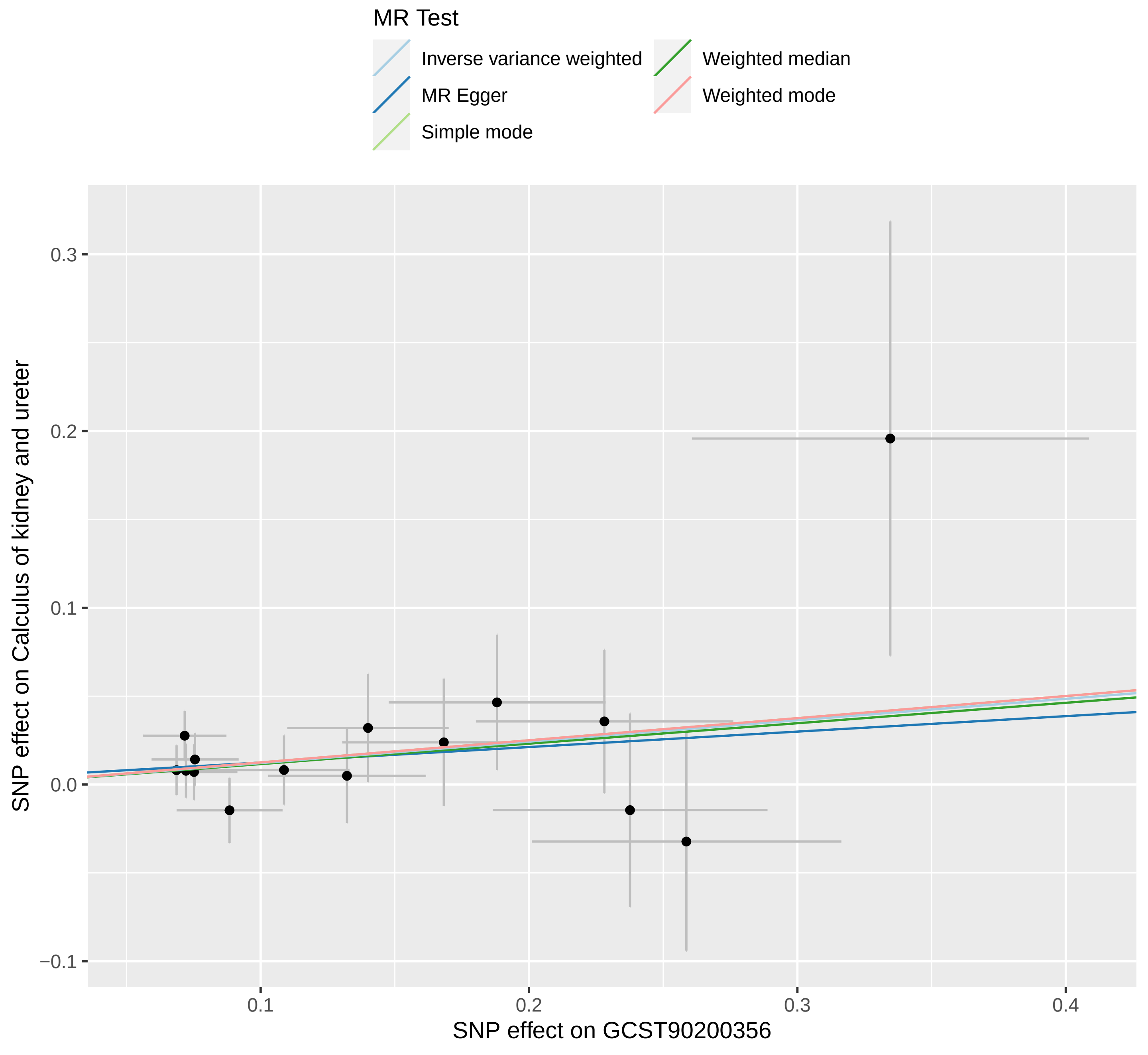
MR Test
Inverse variance weighted
MR Egger
Simple mode
Weighted median
Weighted mode
SNP effect on Calculus of kidney and ureter
0.3
0.2
0.1
0.0
−0.1
0.1
0.2
0.3
0.4
SNP effect on GCST90200356

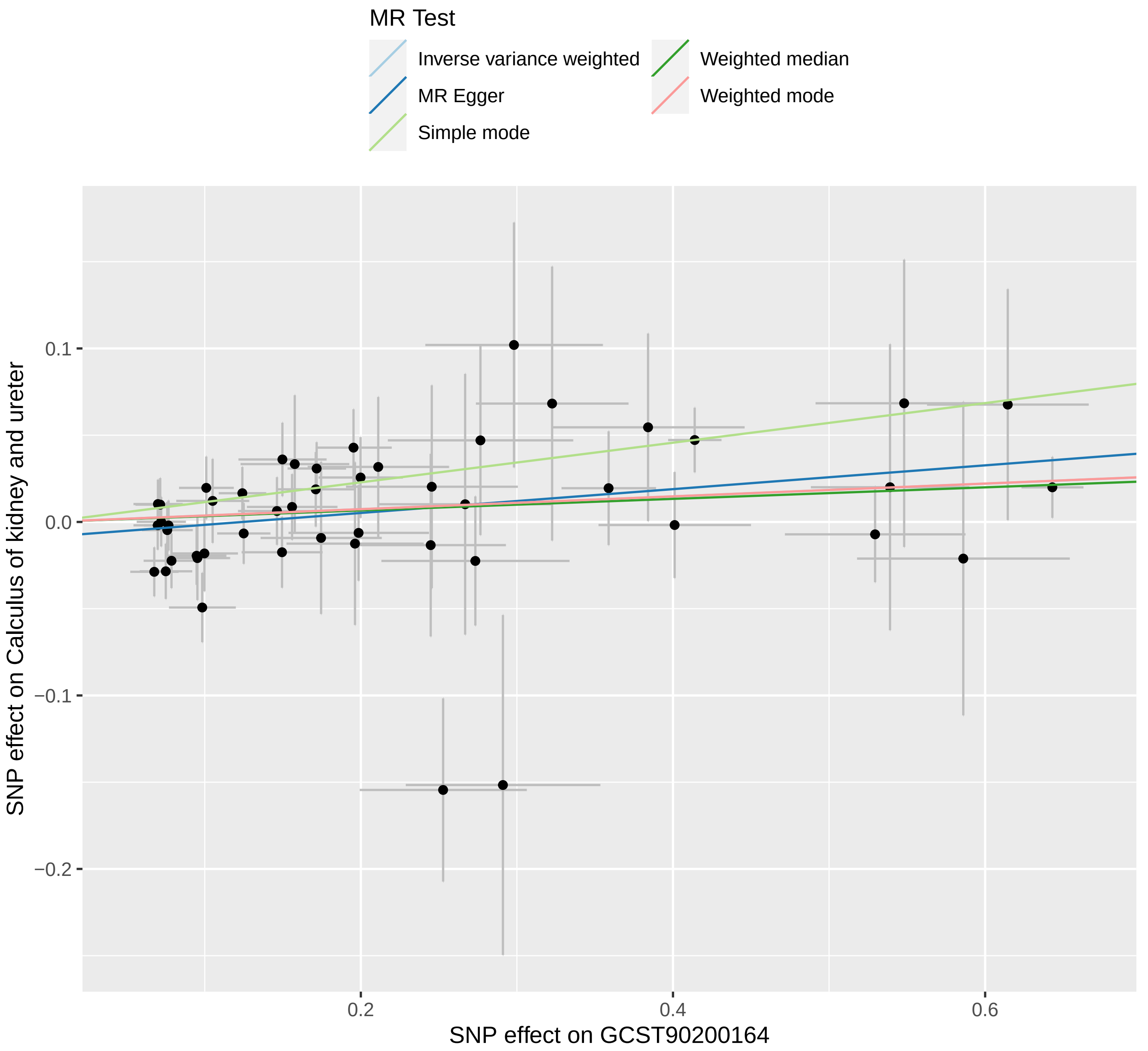

MR Test
Inverse variance weighted
MR Egger
Simple mode
Weighted median
Weighted mode
0.1
0.0
−0.1
−0.2
0.2
0.4
0.6
SNP effect on Calculus of kidney and ureter
SNP effect on GCST90200164

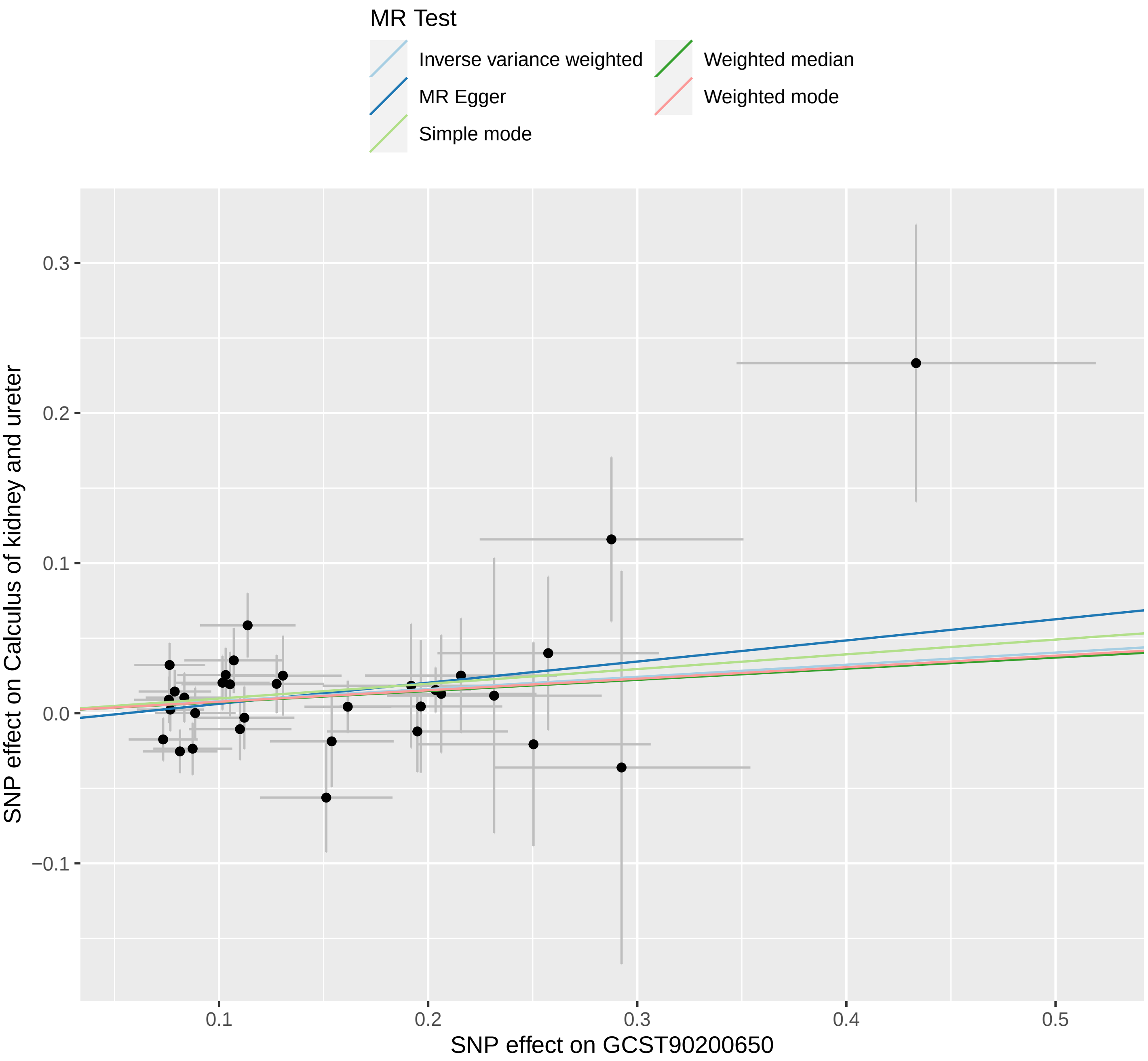

MR Test
Inverse variance weighted
MR Egger
Simple mode
Weighted median
Weighted mode
0.3
0.2
0.1
0.0
−0.1
0.1
0.2
0.3
0.4
0.5
SNP effect on Calculus of kidney and ureter
SNP effect on GCST90200650

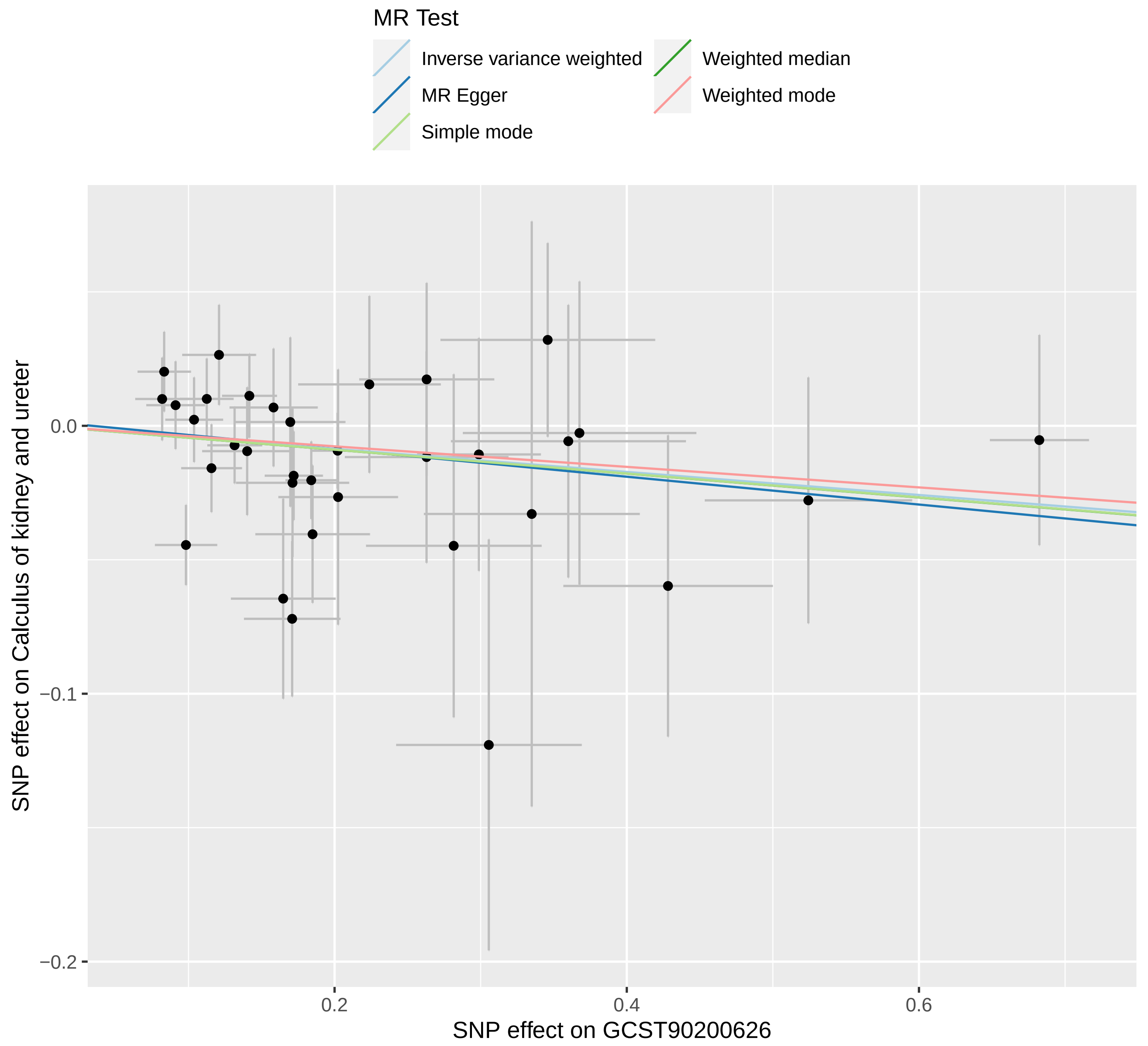

MR Test
Inverse variance weighted
MR Egger
Simple mode
Weighted median
Weighted mode
SNP effect on Calculus of kidney and ureter
0.0
−0.1
−0.2
0.2
0.4
0.6
SNP effect on GCST90200626

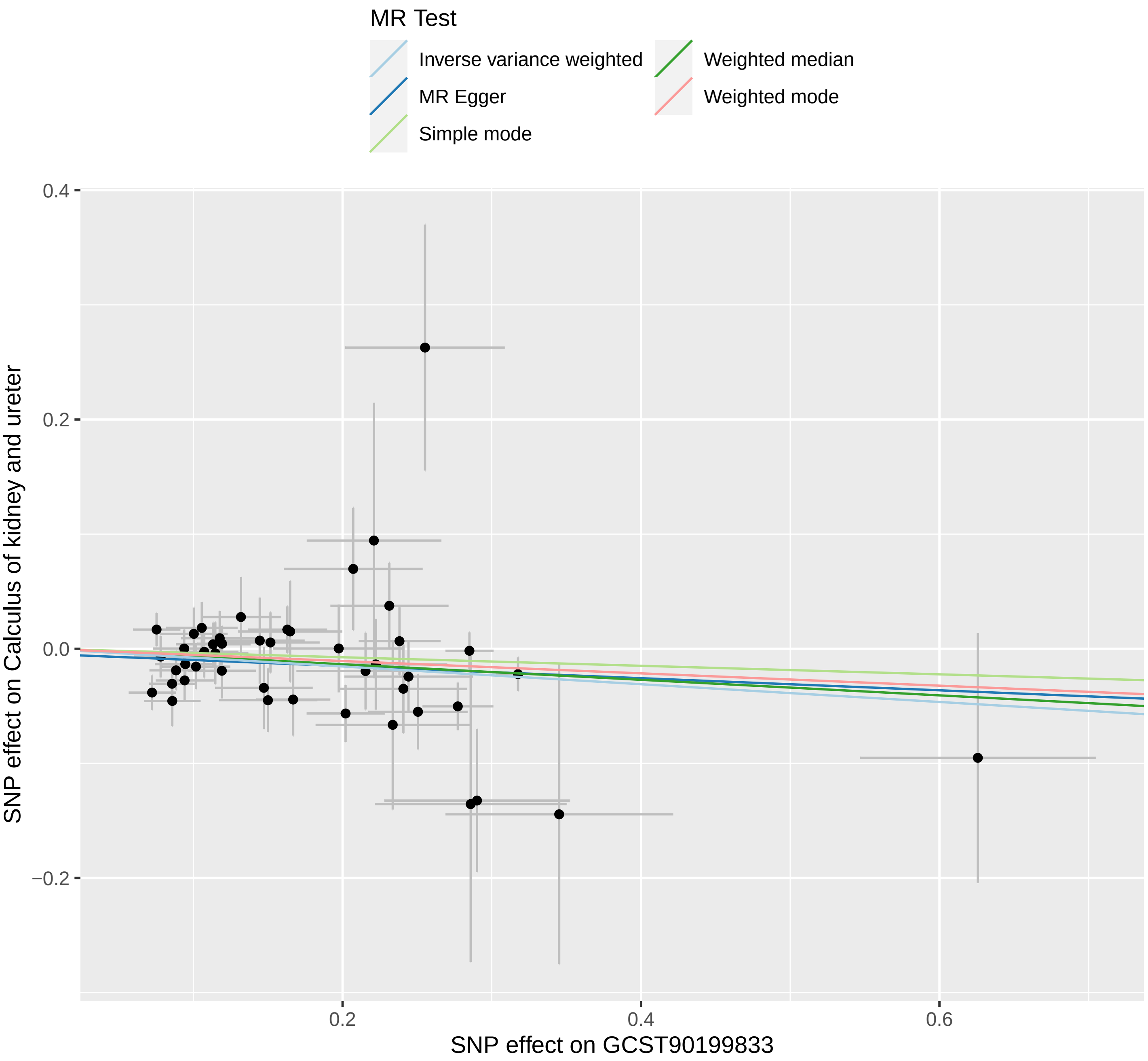

MR Test
Inverse variance weighted
MR Egger
Simple mode
Weighted median
Weighted mode
0.4
0.2
0.0
−0.2
SNP effect on Calculus of kidney and ureter
0.2
0.4
0.6
SNP effect on GCST90199833

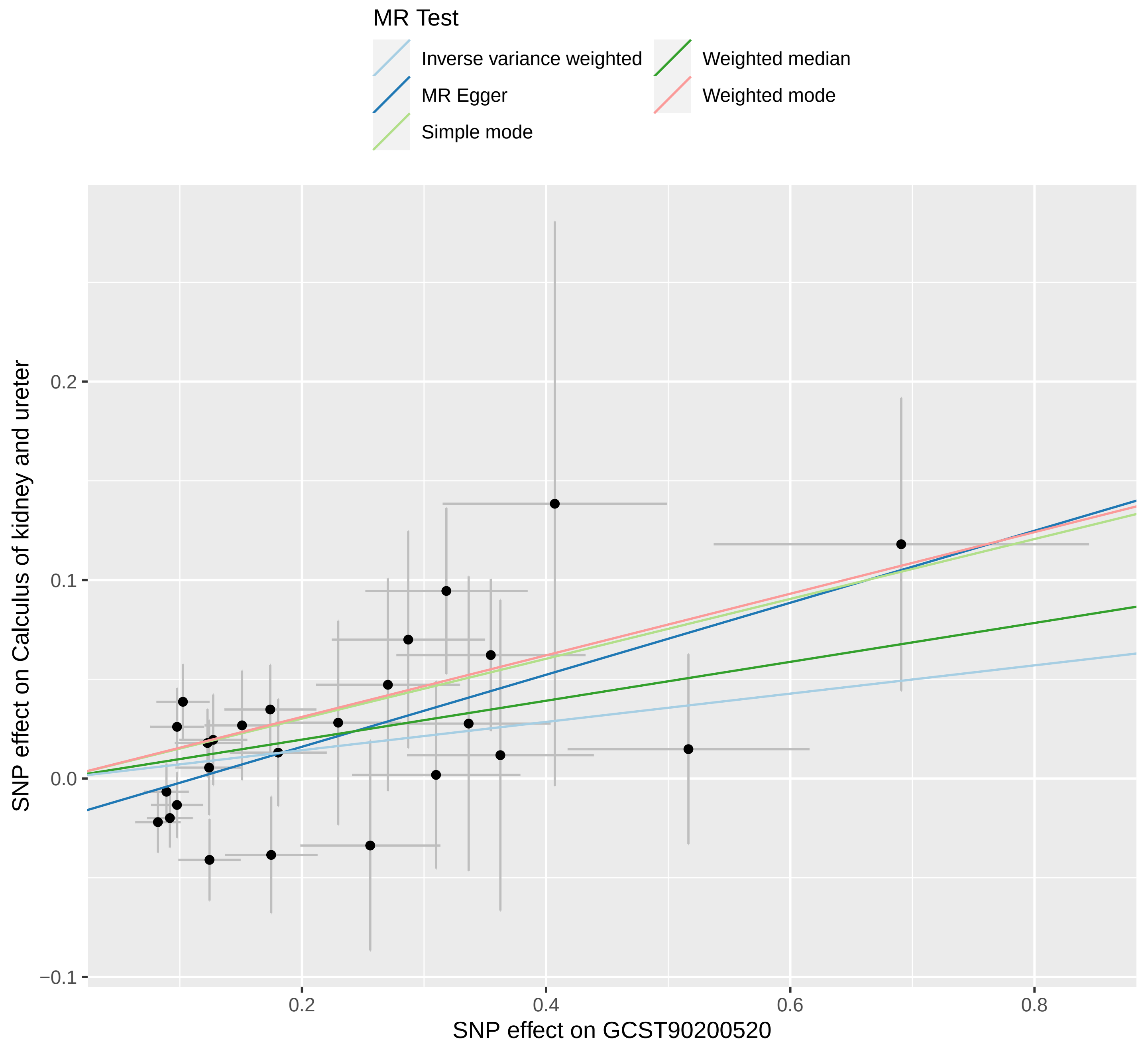
MR Test
Inverse variance weighted
MR Egger
Simple mode
Weighted median
Weighted mode
SNP effect on Calculus of kidney and ureter
0.2
0.1
0.0
−0.1
0.2
0.4
0.6
0.8
SNP effect on GCST90200520

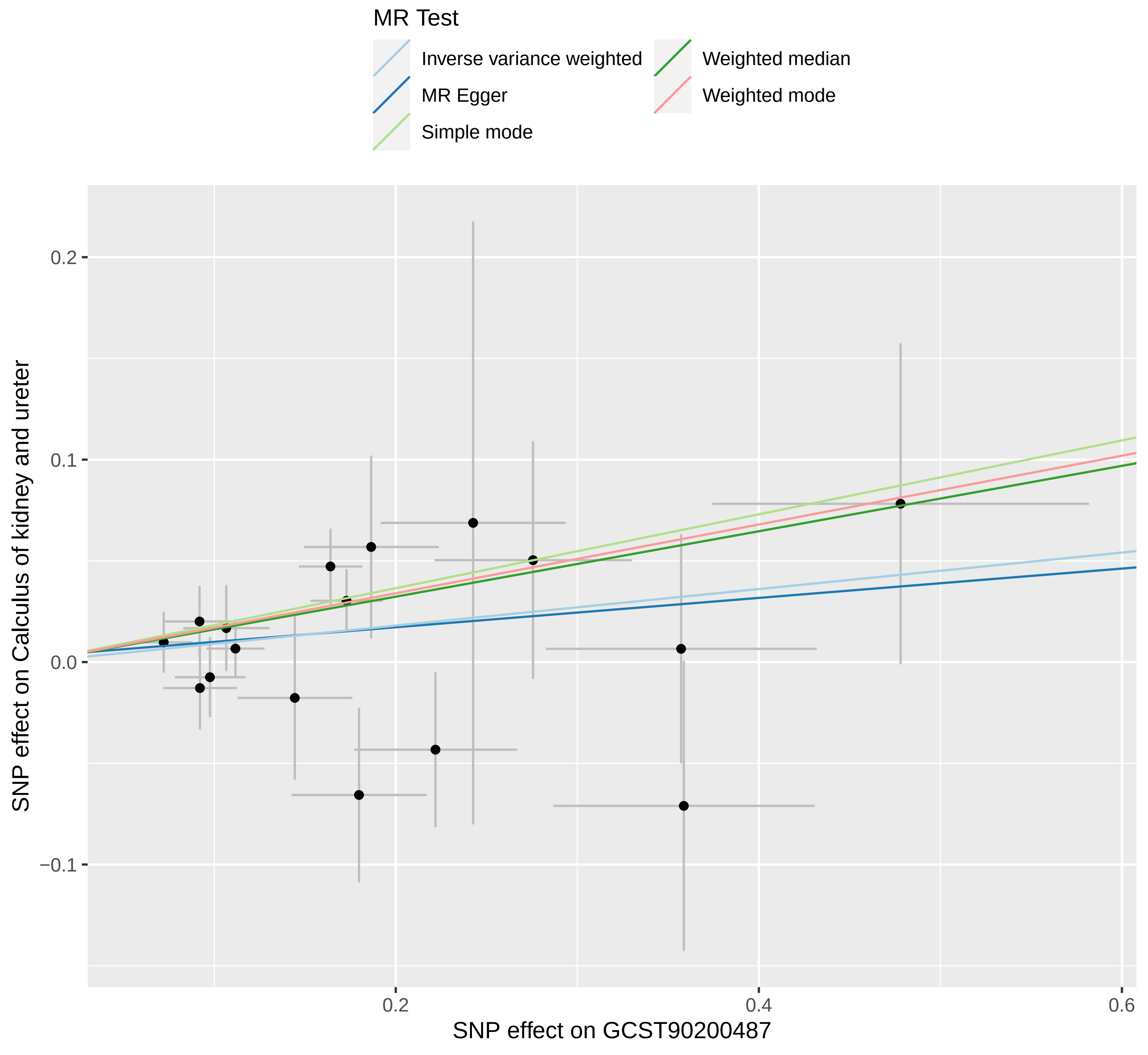

MR Test
Inverse variance weighted
MR Egger
Simple mode
Weighted median
Weighted mode
0.2
0.1
0.0
−0.1
SNP effect on Calculus of kidney and ureter
0.2
0.4
0.6
SNP effect on GCST90200487

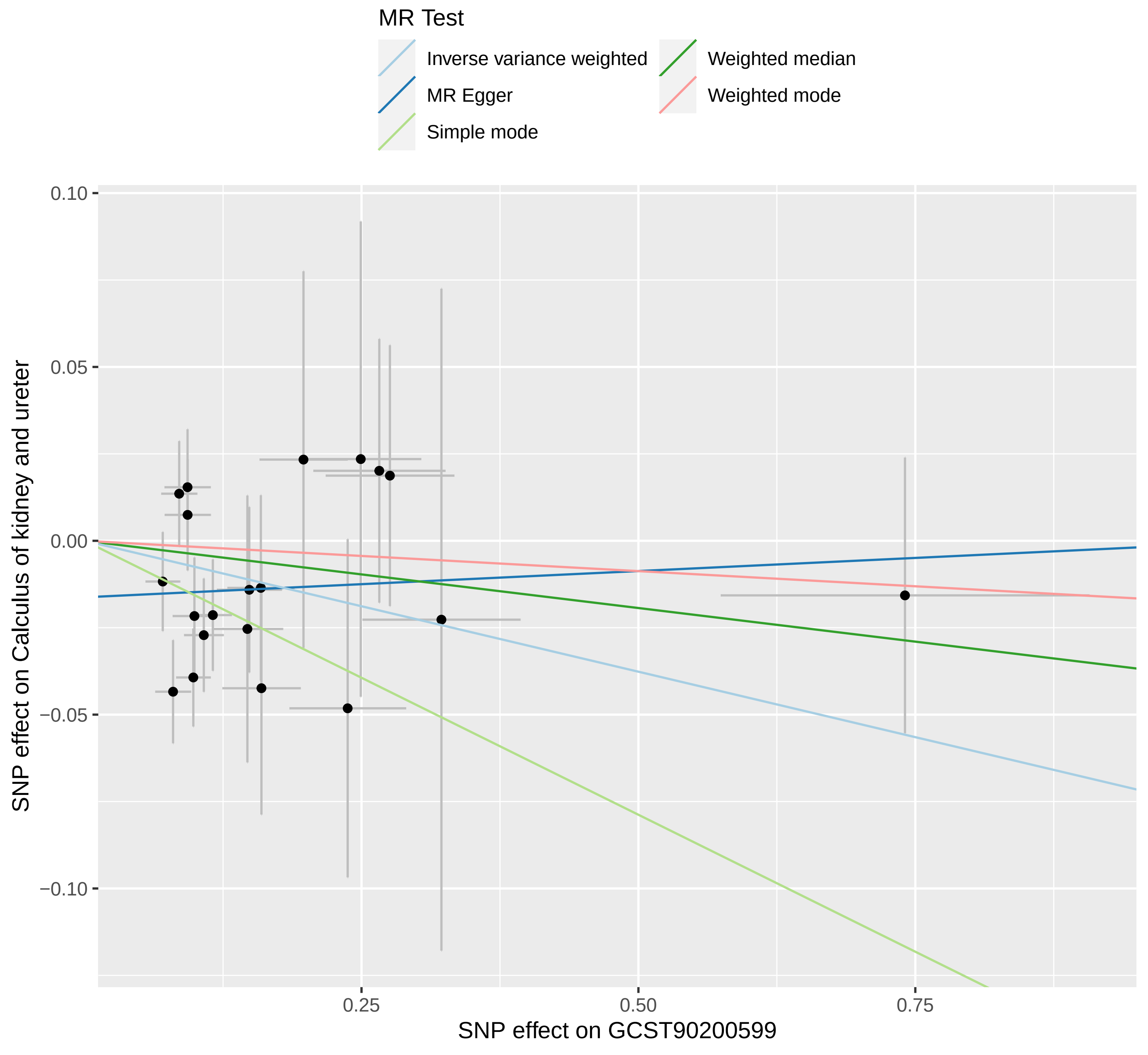
MR Test
Inverse variance weighted
MR Egger
Simple mode
Weighted median
Weighted mode
0.10
0.05
0.00
−0.05
−0.10
0.25
0.50
0.75
SNP effect on GCST90200599
SNP effect on Calculus of kidney and ureter

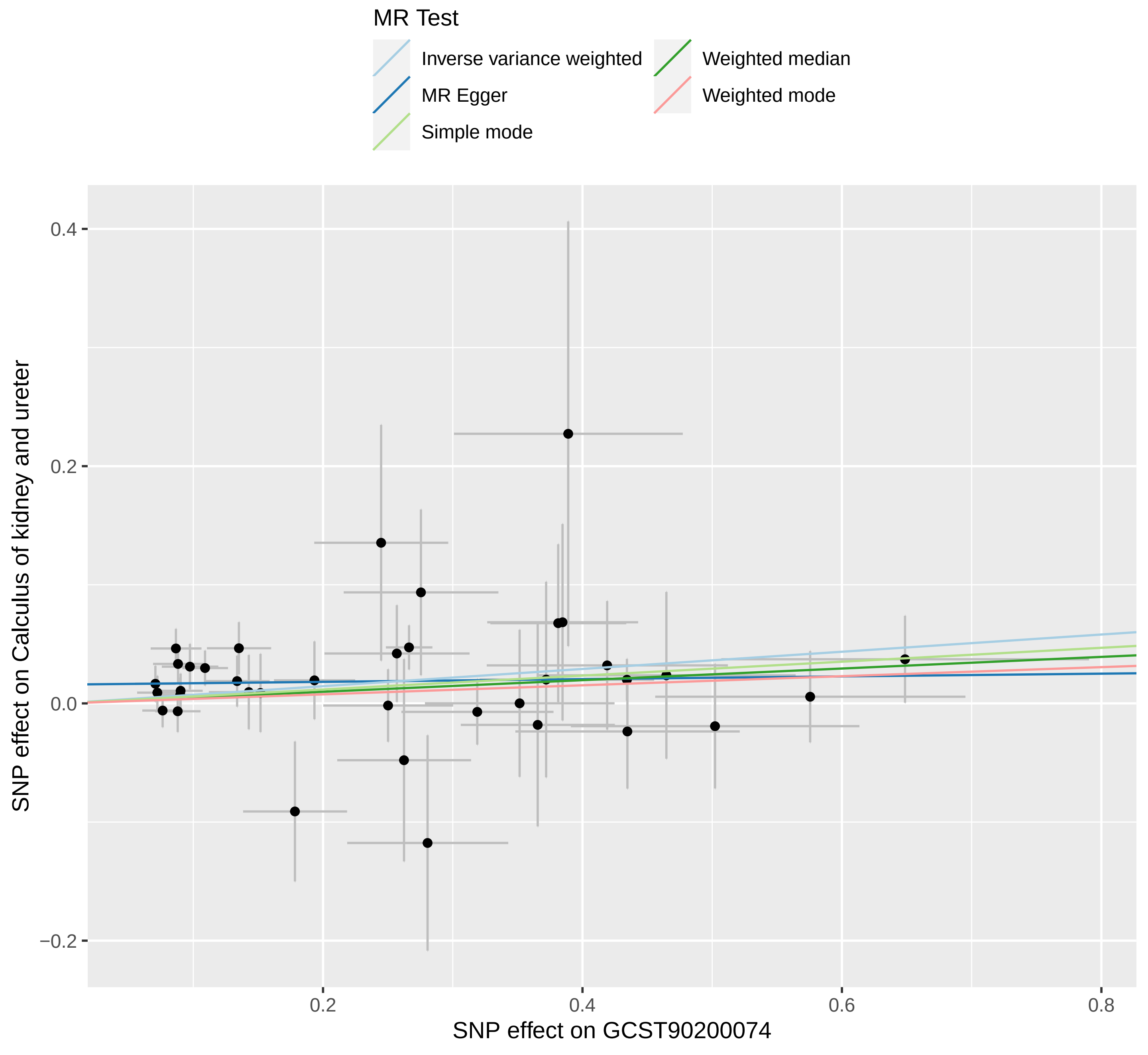

MR Test
Inverse variance weighted
MR Egger
Simple mode
Weighted median
Weighted mode
SNP effect on Calculus of kidney and ureter
0.4
0.2
0.0
−0.2
0.2
0.4
0.6
0.8
SNP effect on GCST90200074

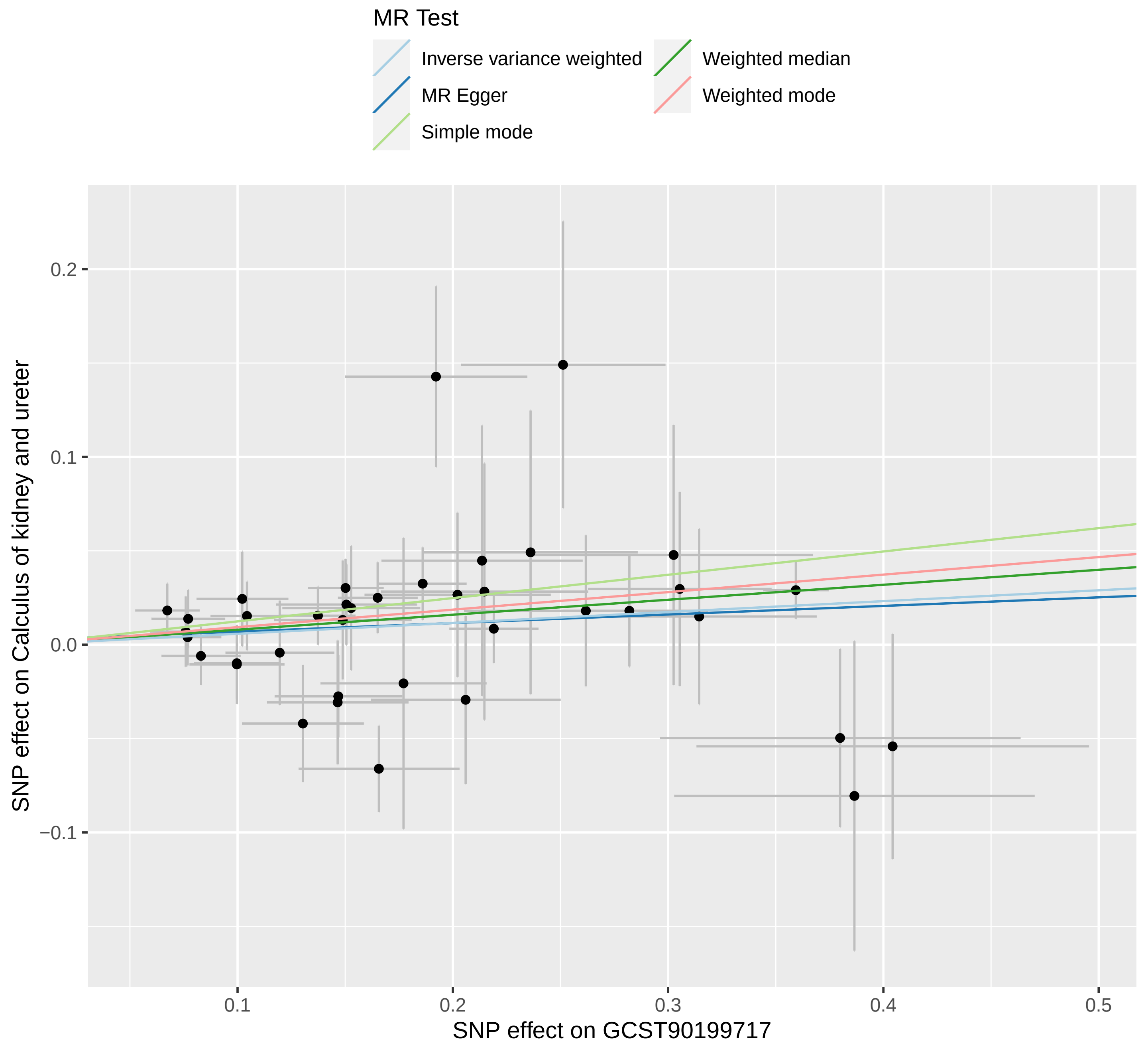

MR Test
Inverse variance weighted
MR Egger
Simple mode
Weighted median
Weighted mode
0.2
0.1
0.0
−0.1
SNP effect on Calculus of kidney and ureter
0.1
0.2
0.3
0.4
0.5
SNP effect on GCST90199717

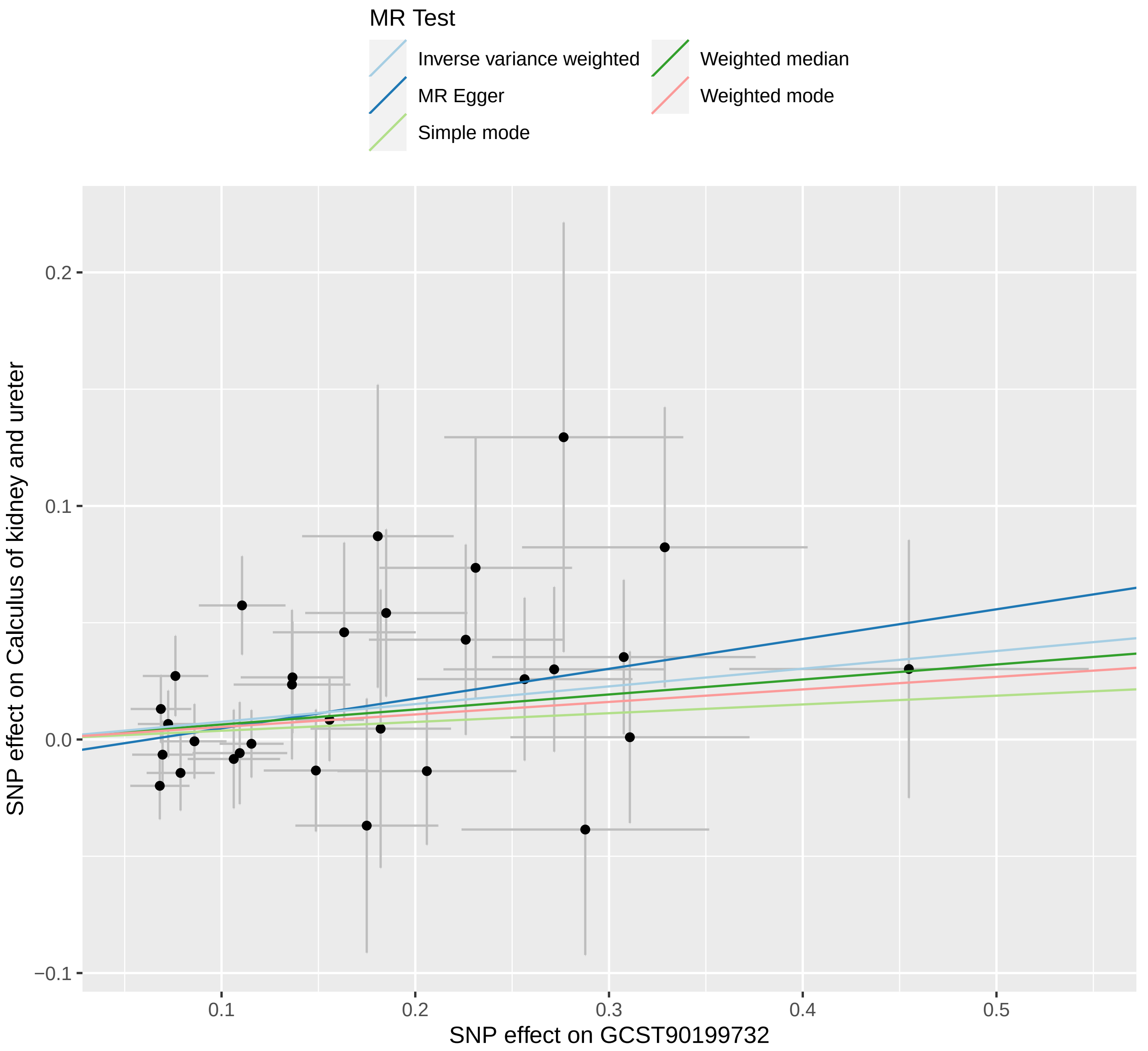

MR Test
Inverse variance weighted
MR Egger
Simple mode
Weighted median
Weighted mode
0.2
0.1
0.0
−0.1
0.1
0.2
0.3
0.4
0.5
SNP effect on GCST90199732
SNP effect on Calculus of kidney and ureter

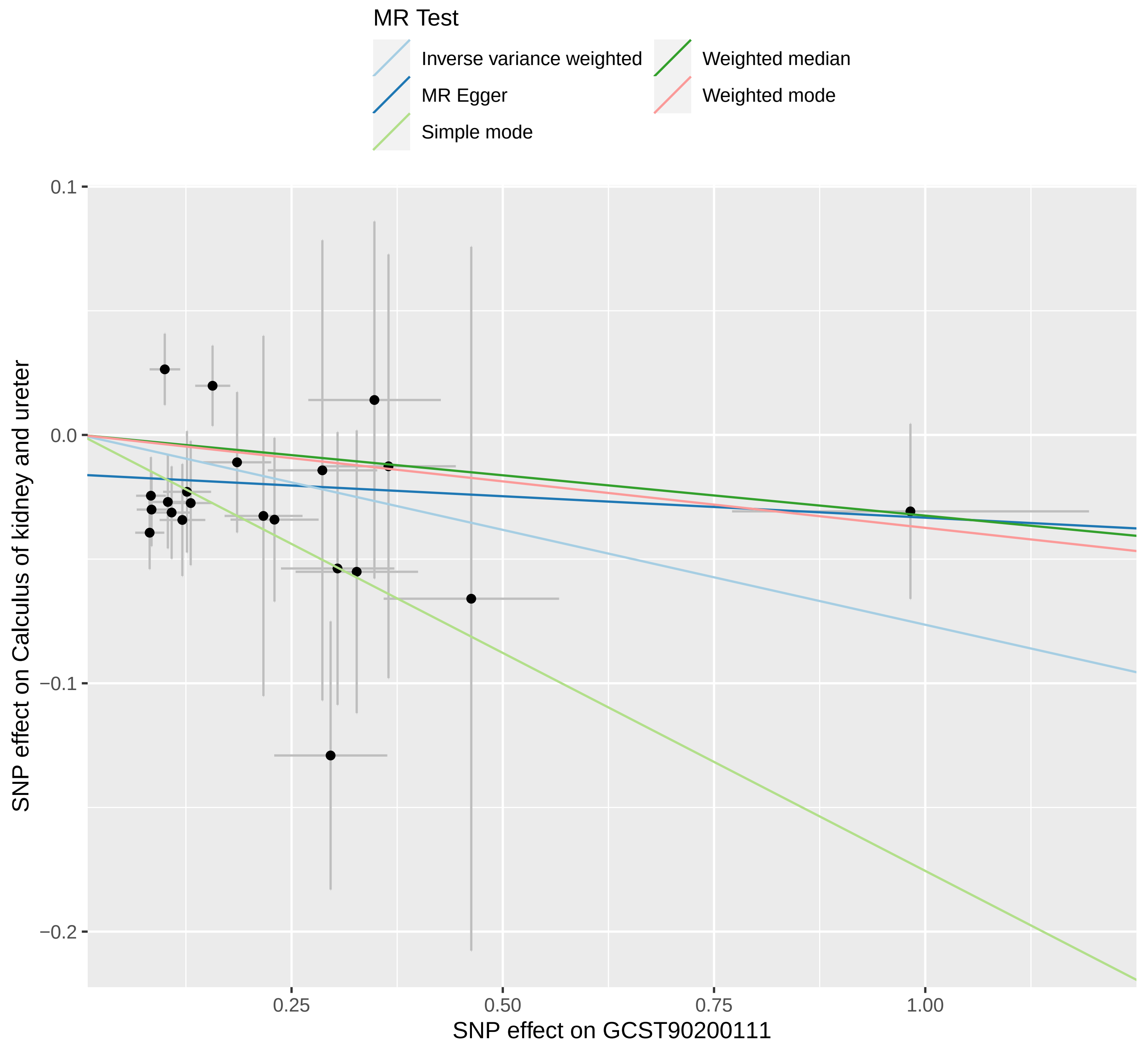
MR Test
Inverse variance weighted
MR Egger
Simple mode
Weighted median
Weighted mode
0.1
0.0
−0.1
−0.2
SNP effect on Calculus of kidney and ureter
0.25
0.50
0.75
1.00
SNP effect on GCST90200111

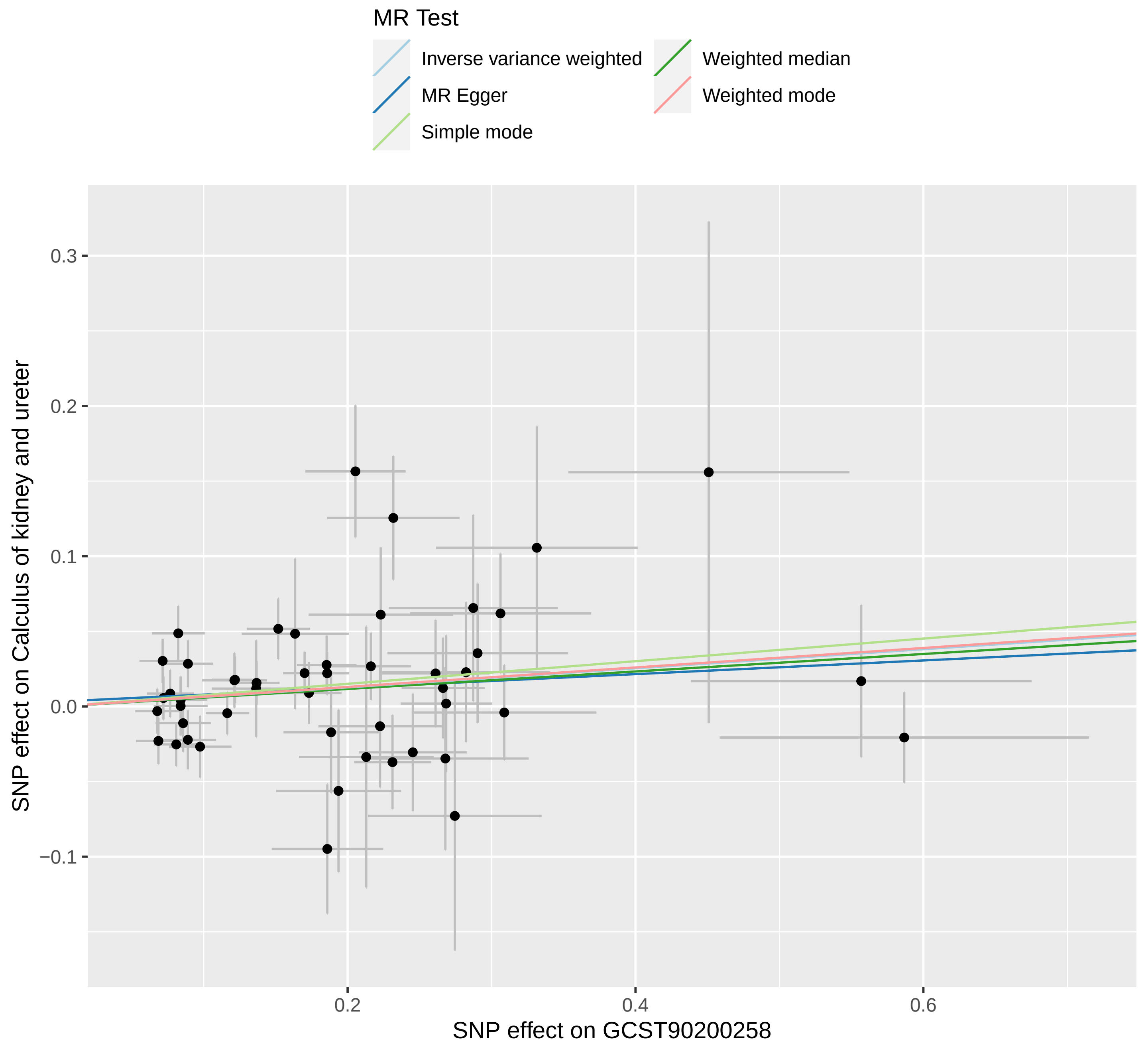

MR Test
Inverse variance weighted
MR Egger
Simple mode
Weighted median
Weighted mode
0.3
0.2
0.1
0.0
−0.1
0.2
0.4
0.6
SNP effect on Calculus of kidney and ureter
SNP effect on GCST90200258

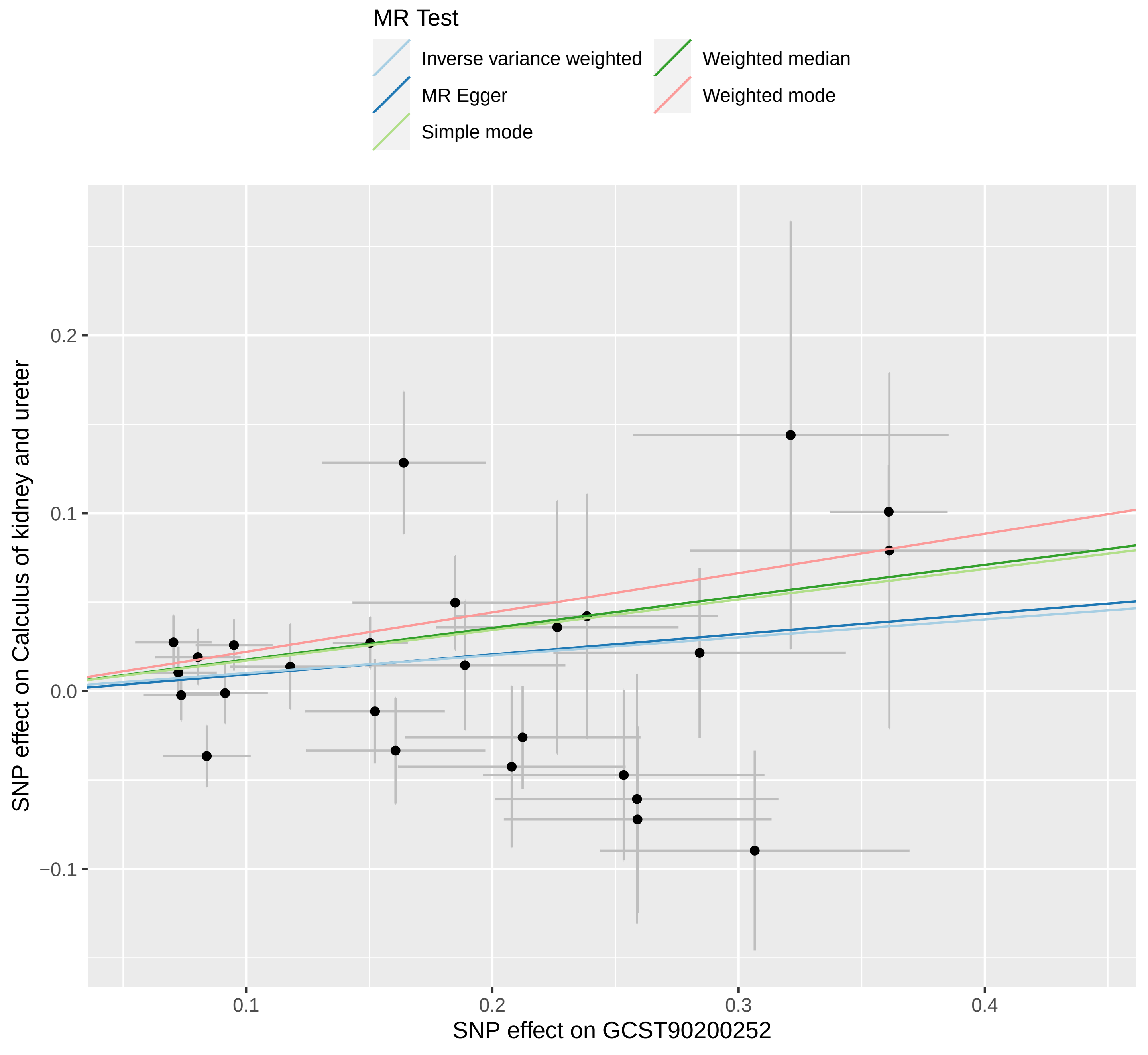
MR Test
Inverse variance weighted
MR Egger
Simple mode
Weighted median
Weighted mode
SNP effect on Calculus of kidney and ureter
0.2
0.1
0.0
−0.1
0.1
0.2
0.3
0.4
SNP effect on GCST90200252

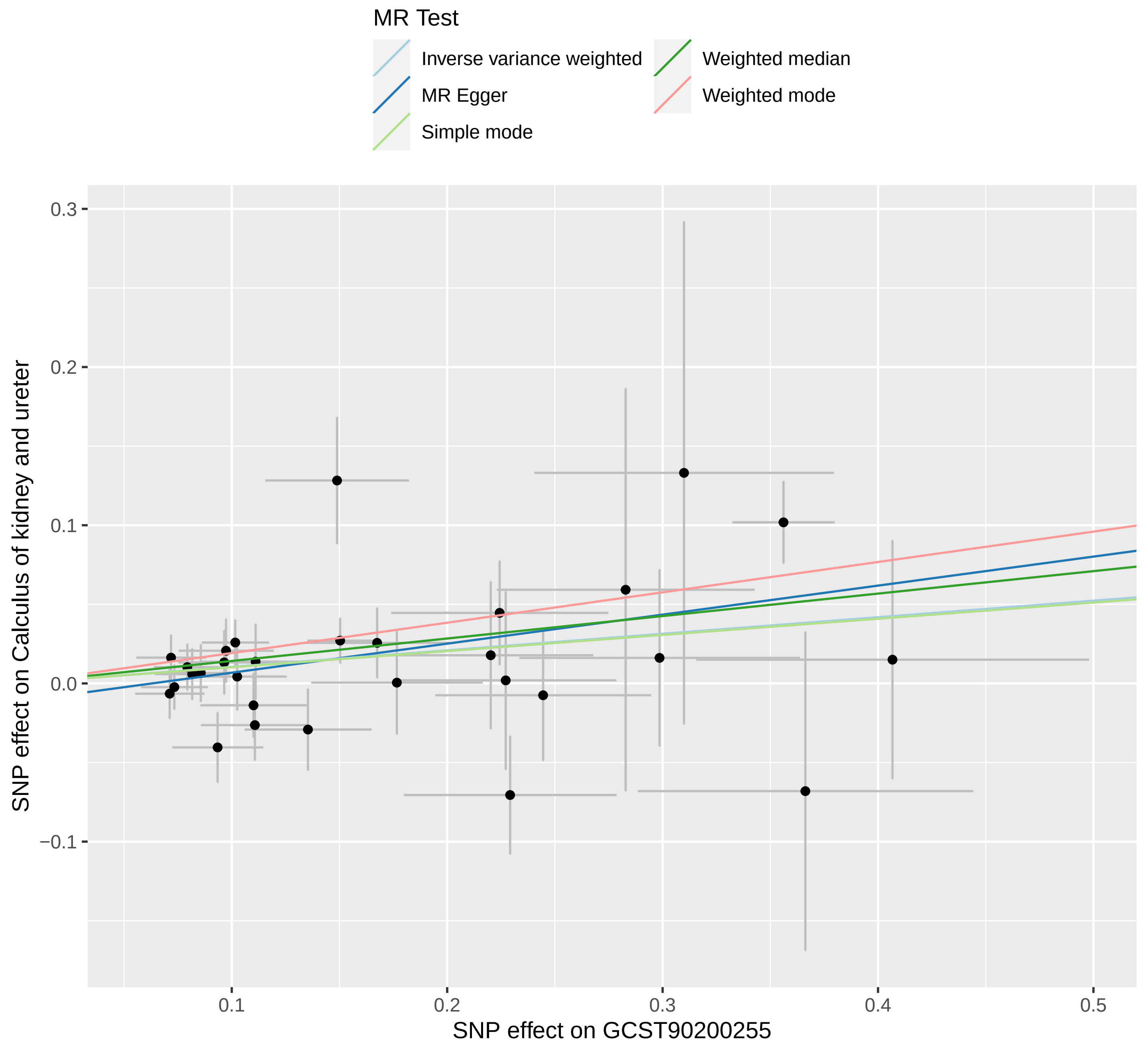

MR Test
Inverse variance weighted
MR Egger
Simple mode
Weighted median
Weighted mode
SNP effect on Calculus of kidney and ureter
0.3
0.2
0.1
0.0
−0.1
0.1
0.2
0.3
0.4
0.5
SNP effect on GCST90200255

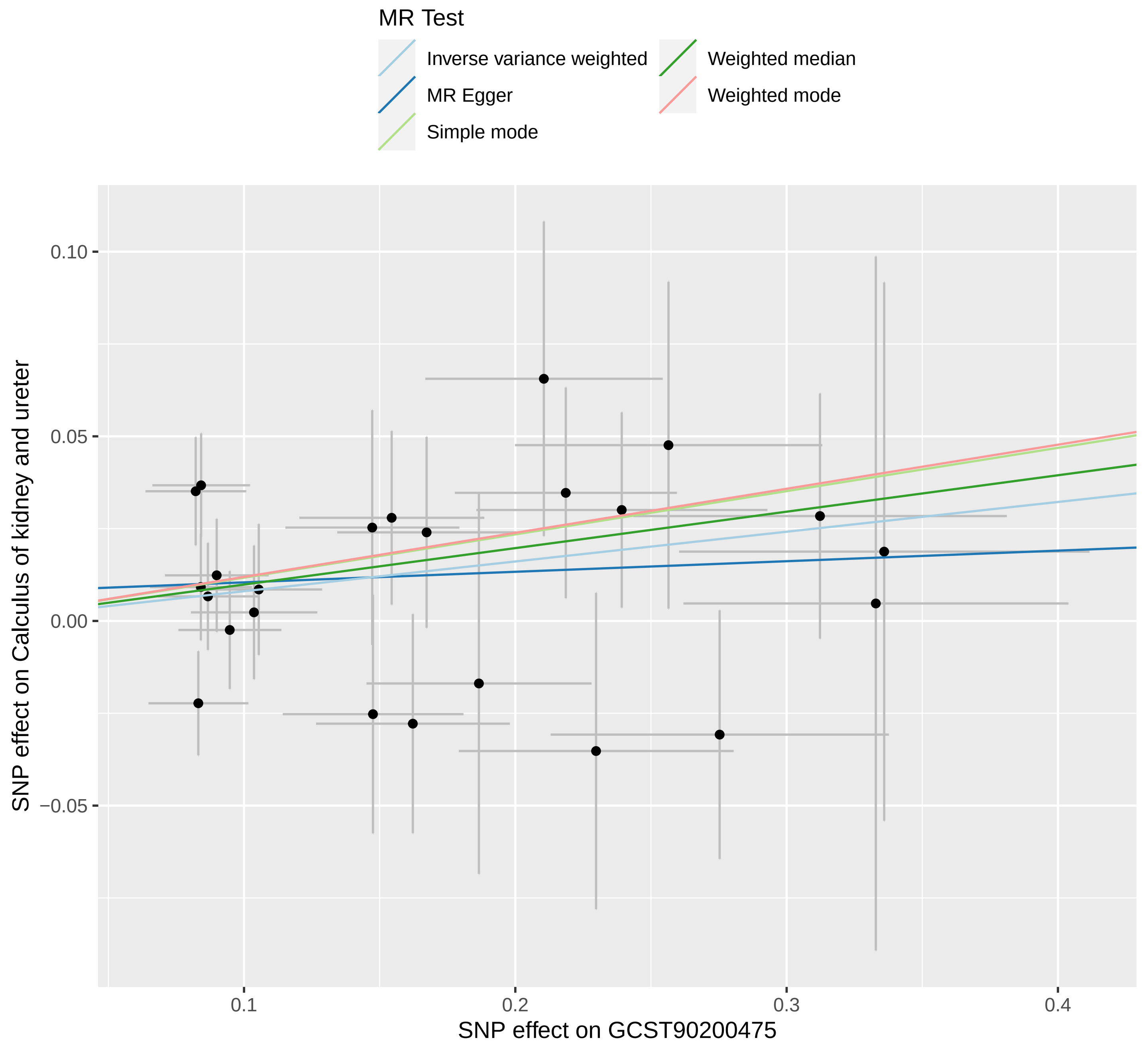

MR Test
Inverse variance weighted
MR Egger
Simple mode
Weighted median
Weighted mode
0.10
0.05
0.00
−0.05
SNP effect on Calculus of kidney and ureter
0.1
0.2
0.3
0.4
SNP effect on GCST90200475

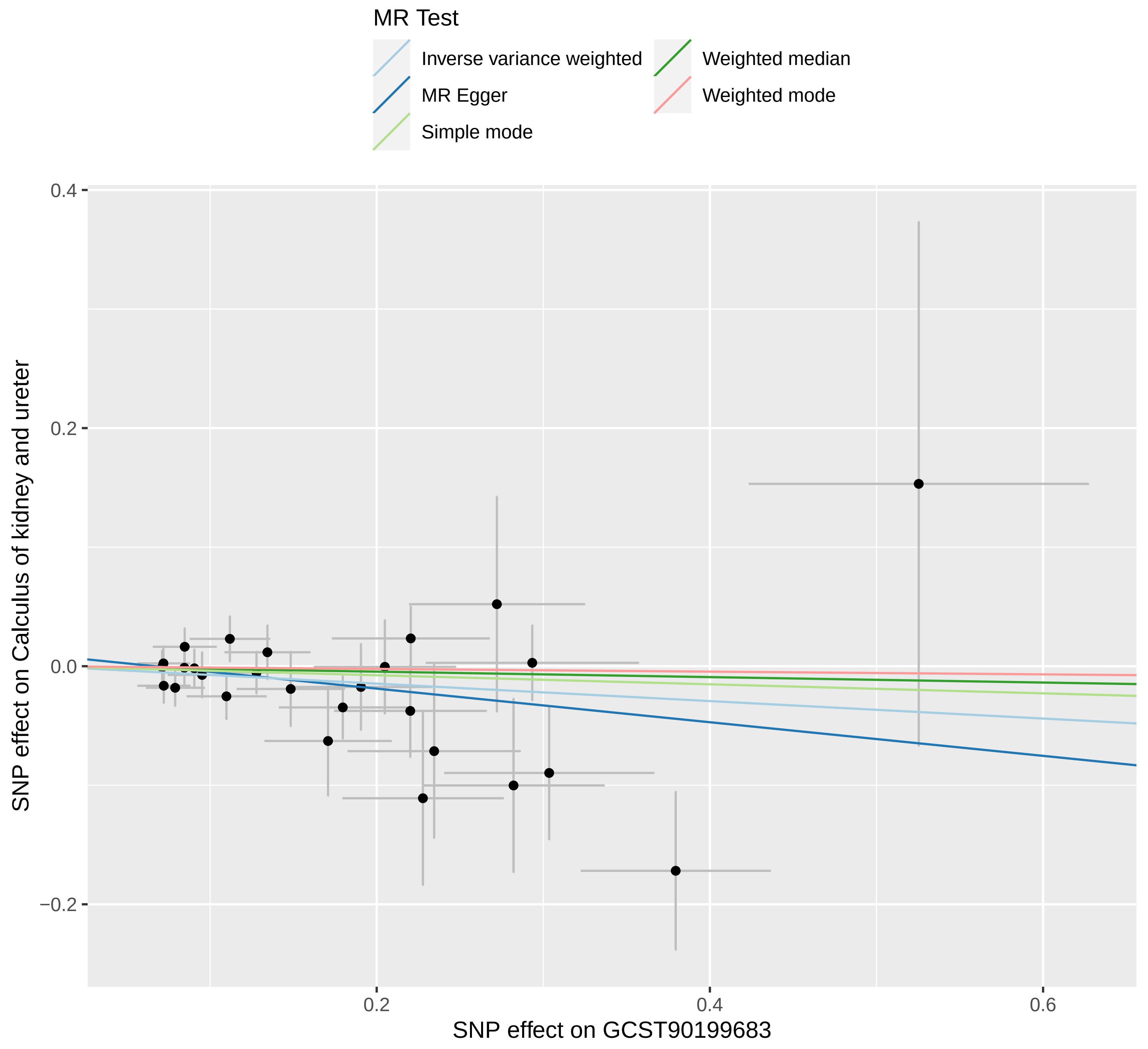
MR Test
Inverse variance weighted
MR Egger
Simple mode
Weighted median
Weighted mode
0.4
0.2
0.0
−0.2
0.2
0.4
0.6
SNP effect on GCST90199683
SNP effect on Calculus of kidney and ureter

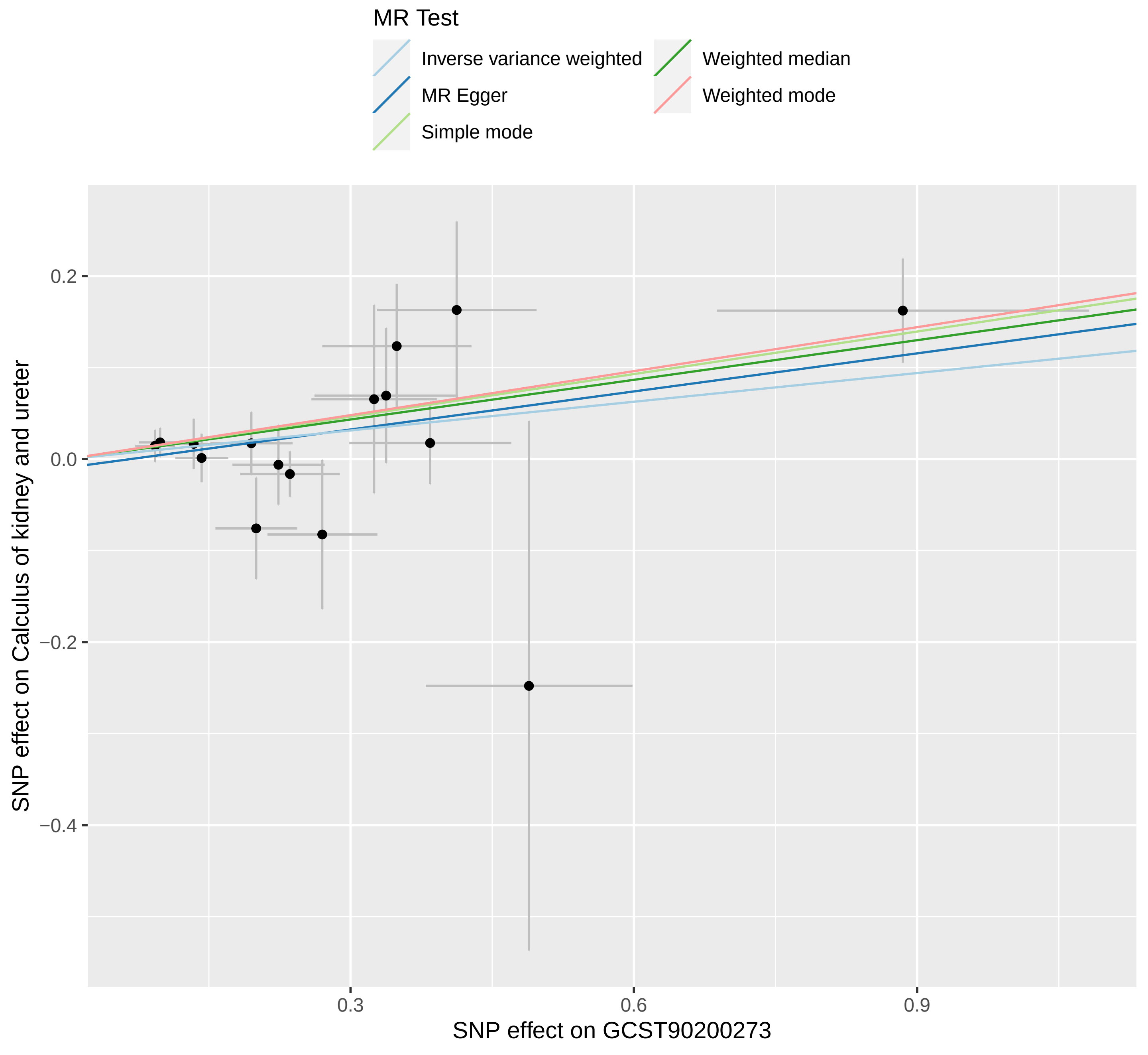
MR Test
Inverse variance weighted
MR Egger
Simple mode
Weighted median
Weighted mode
0.2
0.0
−0.2
−0.4
0.3
0.6
0.9
SNP effect on GCST90200273
SNP effect on Calculus of kidney and ureter

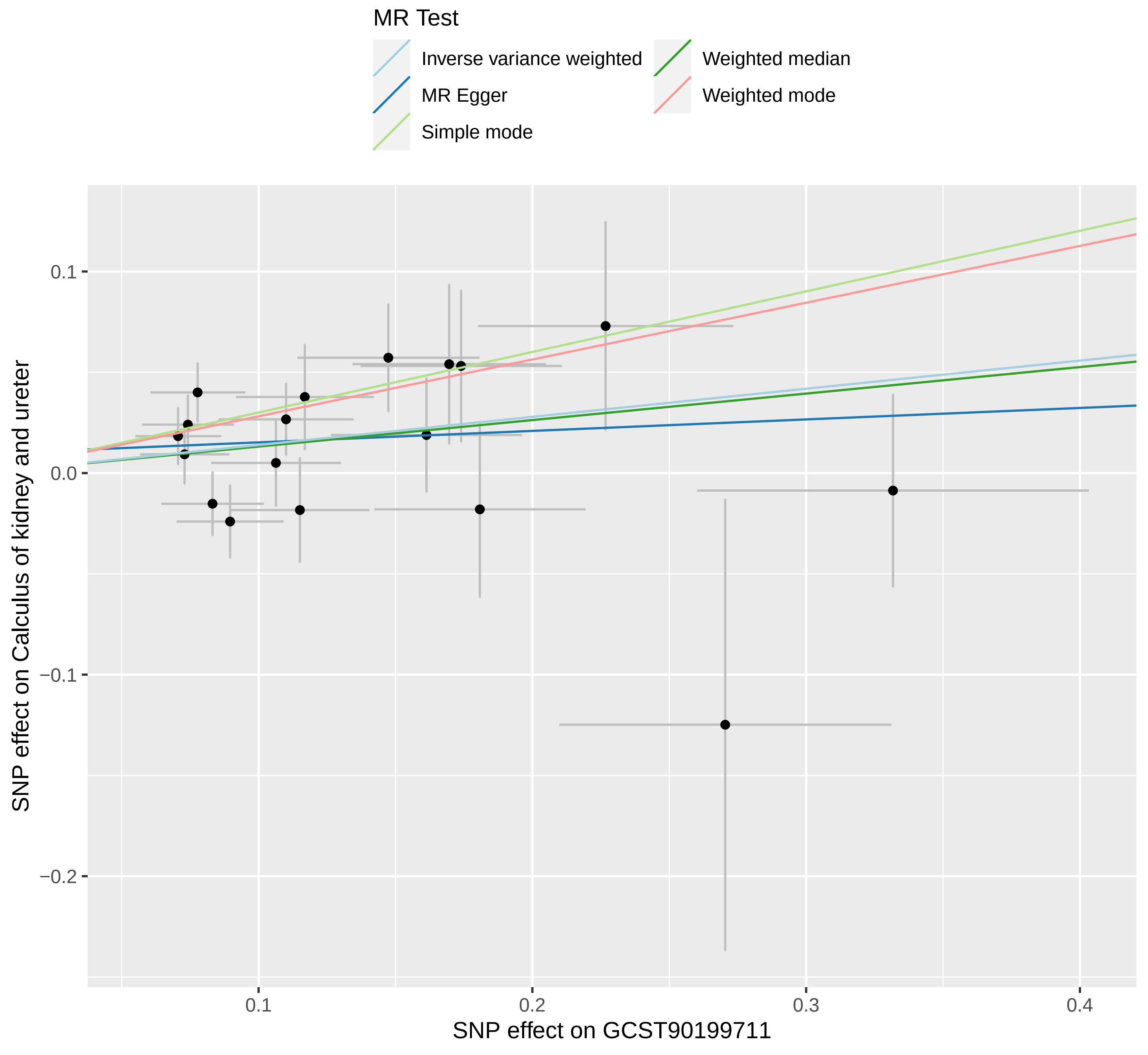
MR Test
Inverse variance weighted
MR Egger
Simple mode
Weighted median
Weighted mode
0.1
0.0
−0.1
−0.2
0.1
0.2
0.3
0.4
SNP effect on Calculus of kidney and ureter
SNP effect on GCST90199711

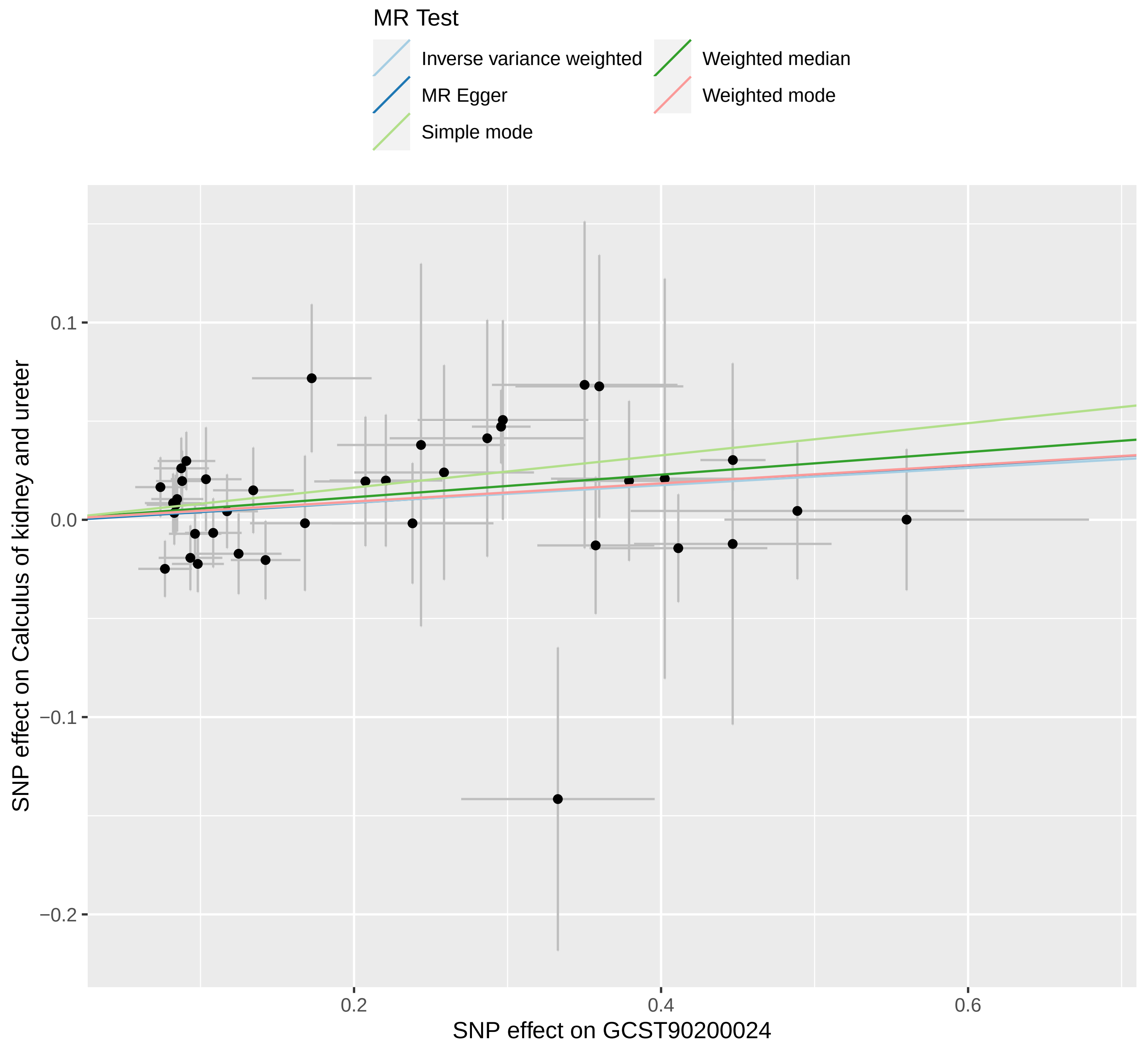
MR Test
Inverse variance weighted
MR Egger
Simple mode
Weighted median
Weighted mode
0.1
0.0
−0.1
−0.2
0.2
0.4
0.6
SNP effect on Calculus of kidney and ureter
SNP effect on GCST90200024

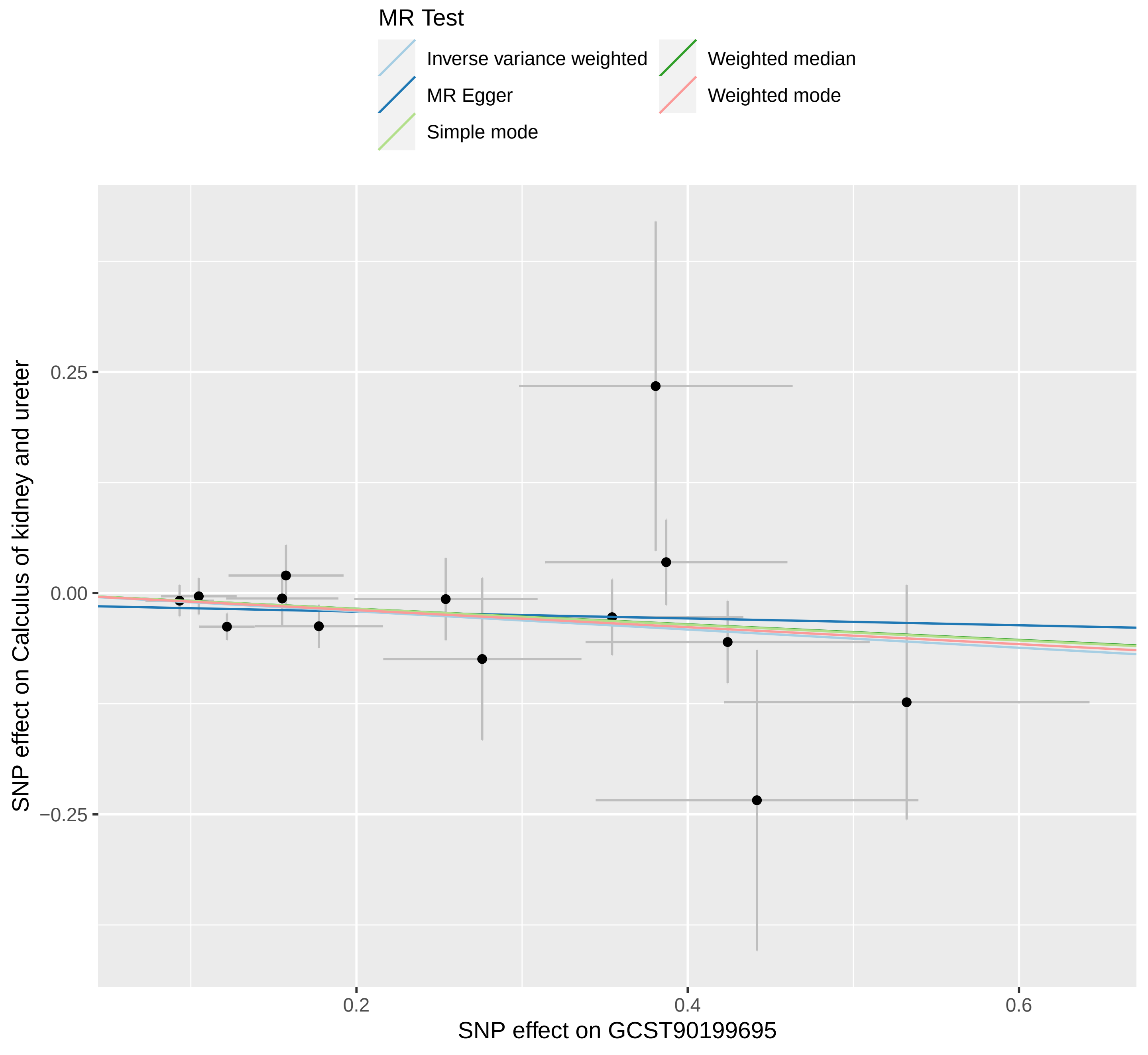

MR Test
Inverse variance weighted
MR Egger
Simple mode
Weighted median
Weighted mode
0.25
0.00
−0.25
0.2
0.4
0.6
SNP effect on Calculus of kidney and ureter
SNP effect on GCST90199695

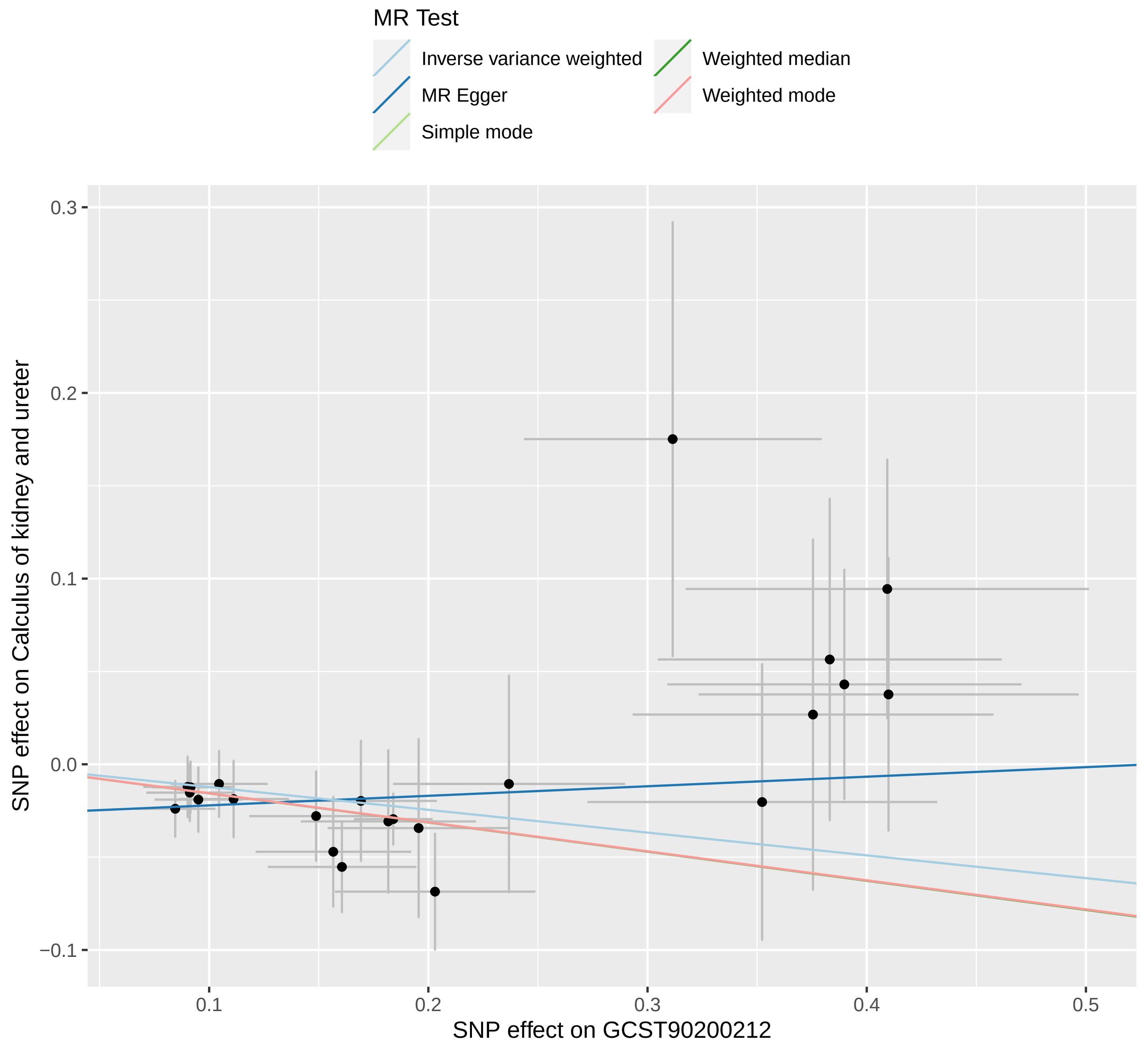
MR Test
Inverse variance weighted
MR Egger
Simple mode
Weighted median
Weighted mode
SNP effect on Calculus of kidney and ureter
SNP effect on GCST90200212
0.3
0.2
0.1
0.0
−0.1
0.1
0.2
0.3
0.4
0.5

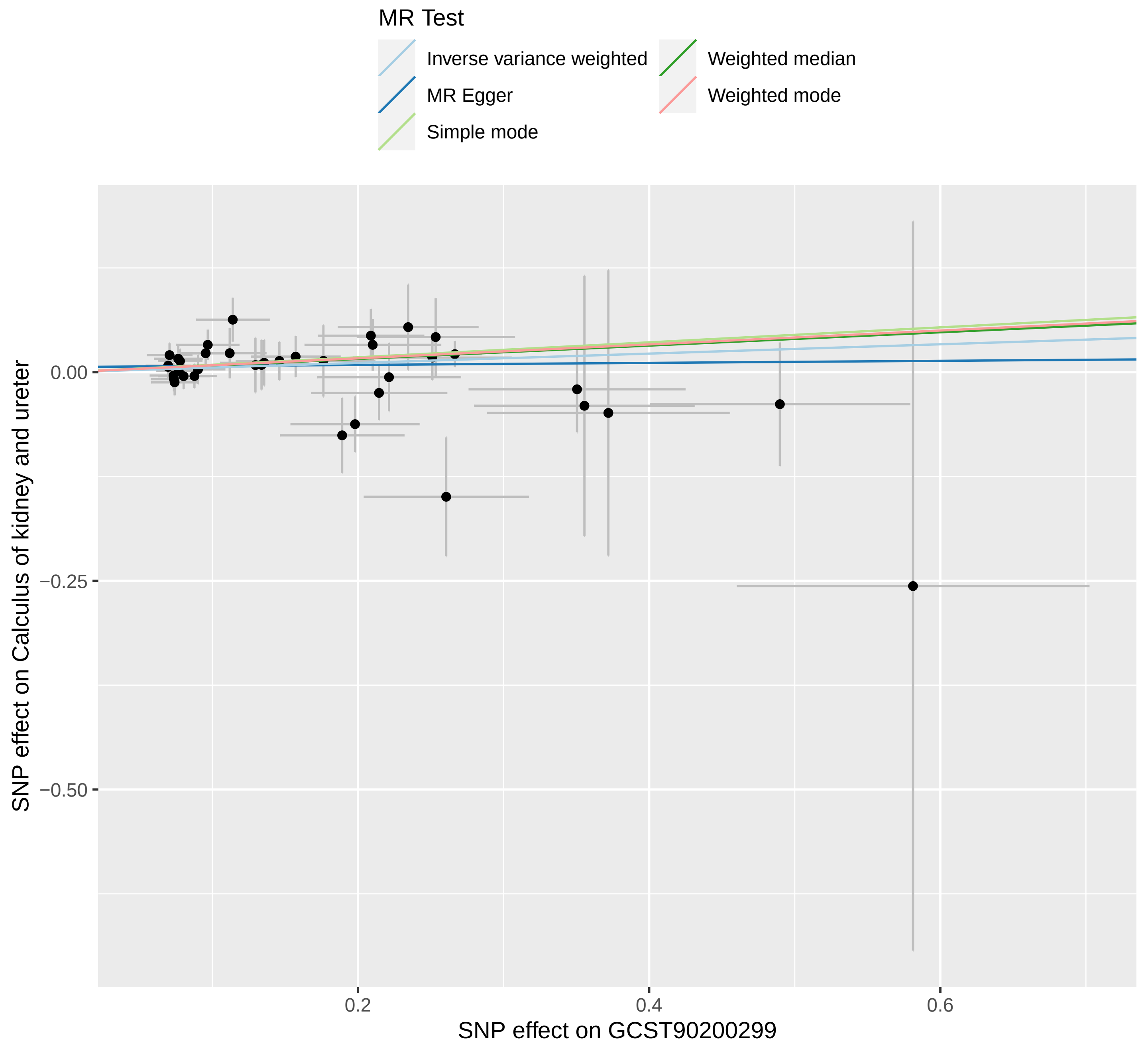

MR Test
Inverse variance weighted
MR Egger
Simple mode
Weighted median
Weighted mode
0.00
−0.25
−0.50
0.2
0.4
0.6
SNP effect on Calculus of kidney and ureter
SNP effect on GCST90200299

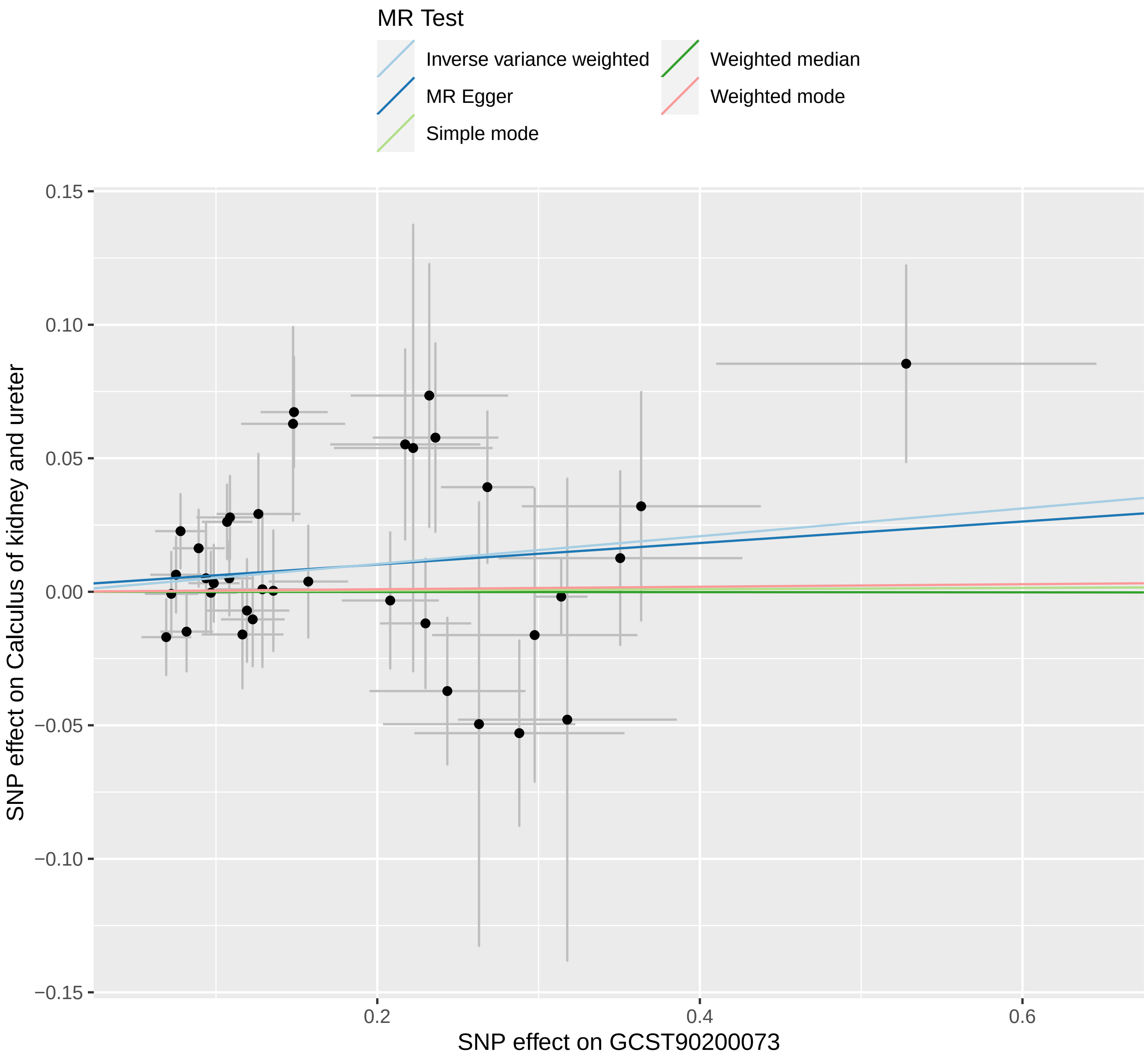
MR Test
Inverse variance weighted
MR Egger
Simple mode
Weighted median
Weighted mode
0.15
0.10
0.05
0.00
−0.05
−0.10
−0.15
0.2
0.4
0.6
SNP effect on Calculus of kidney and ureter
SNP effect on GCST90200073

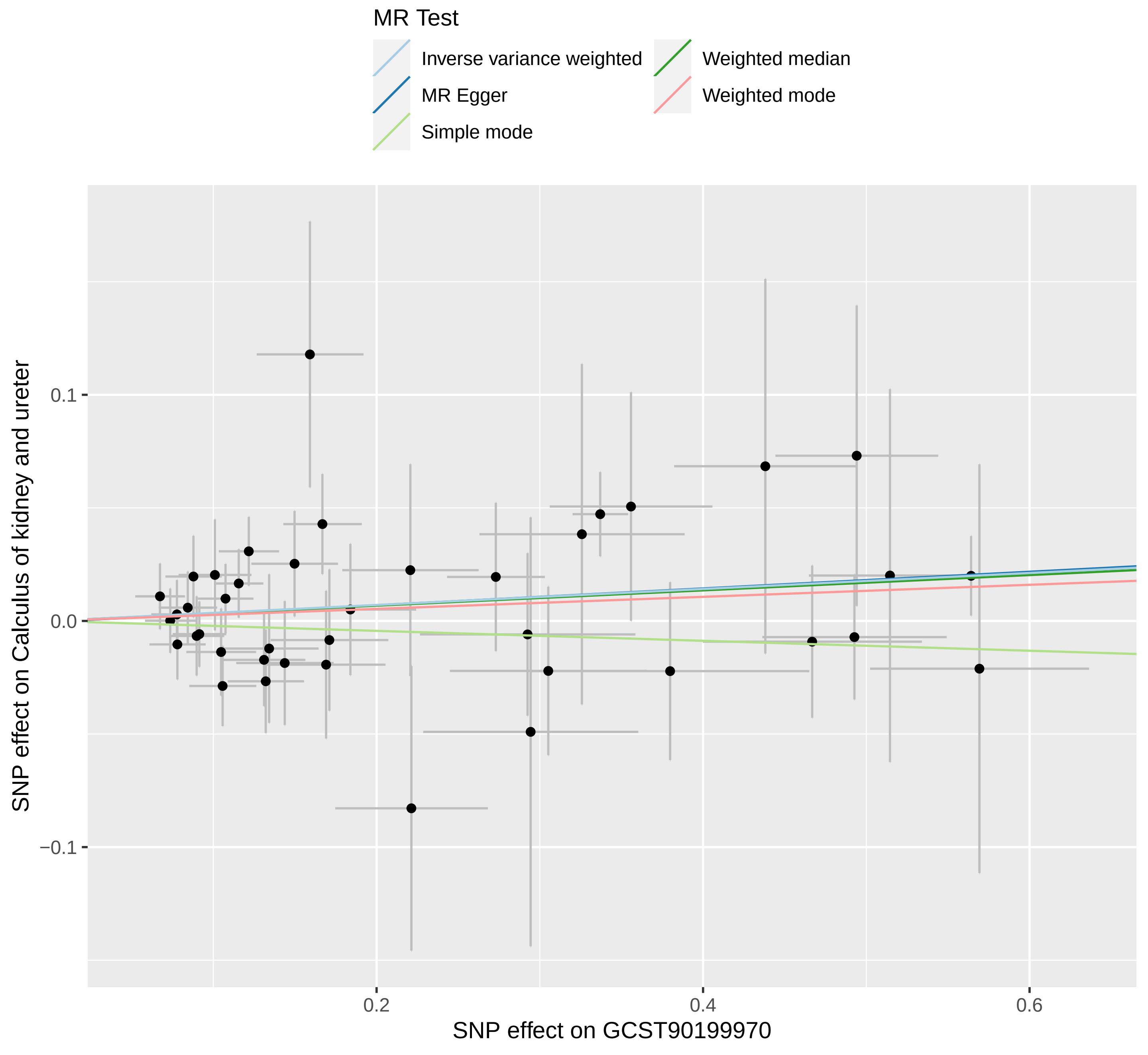
MR Test
Inverse variance weighted
MR Egger
Simple mode
Weighted median
Weighted mode
0.1
0.0
−0.1
0.2
0.4
0.6
SNP effect on Calculus of kidney and ureter
SNP effect on GCST90199970

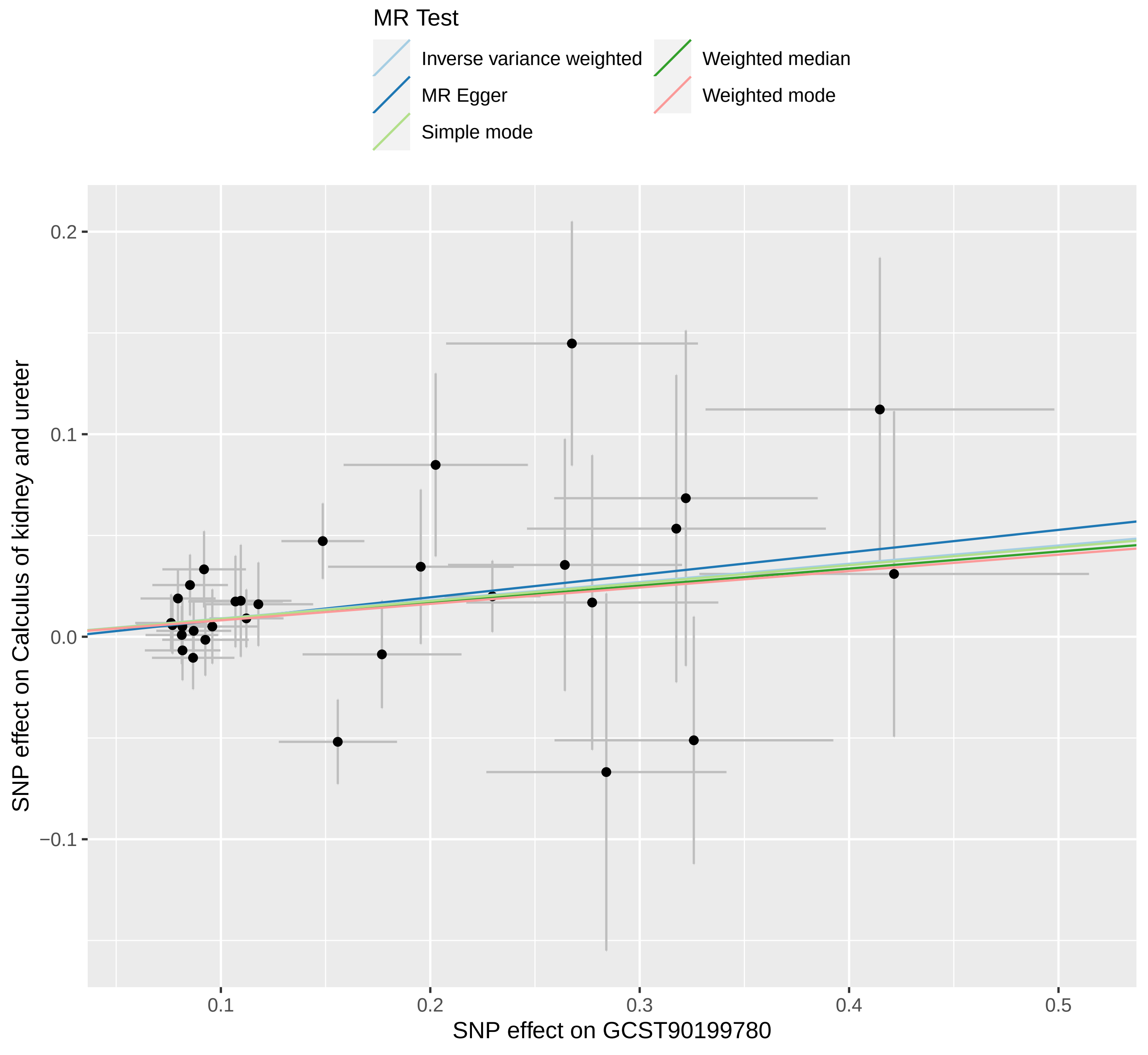
MR Test
Inverse variance weighted
MR Egger
Simple mode
Weighted median
Weighted mode
SNP effect on Calculus of kidney and ureter
0.2
0.1
0.0
−0.1
0.1
0.2
0.3
0.4
0.5
SNP effect on GCST90199780

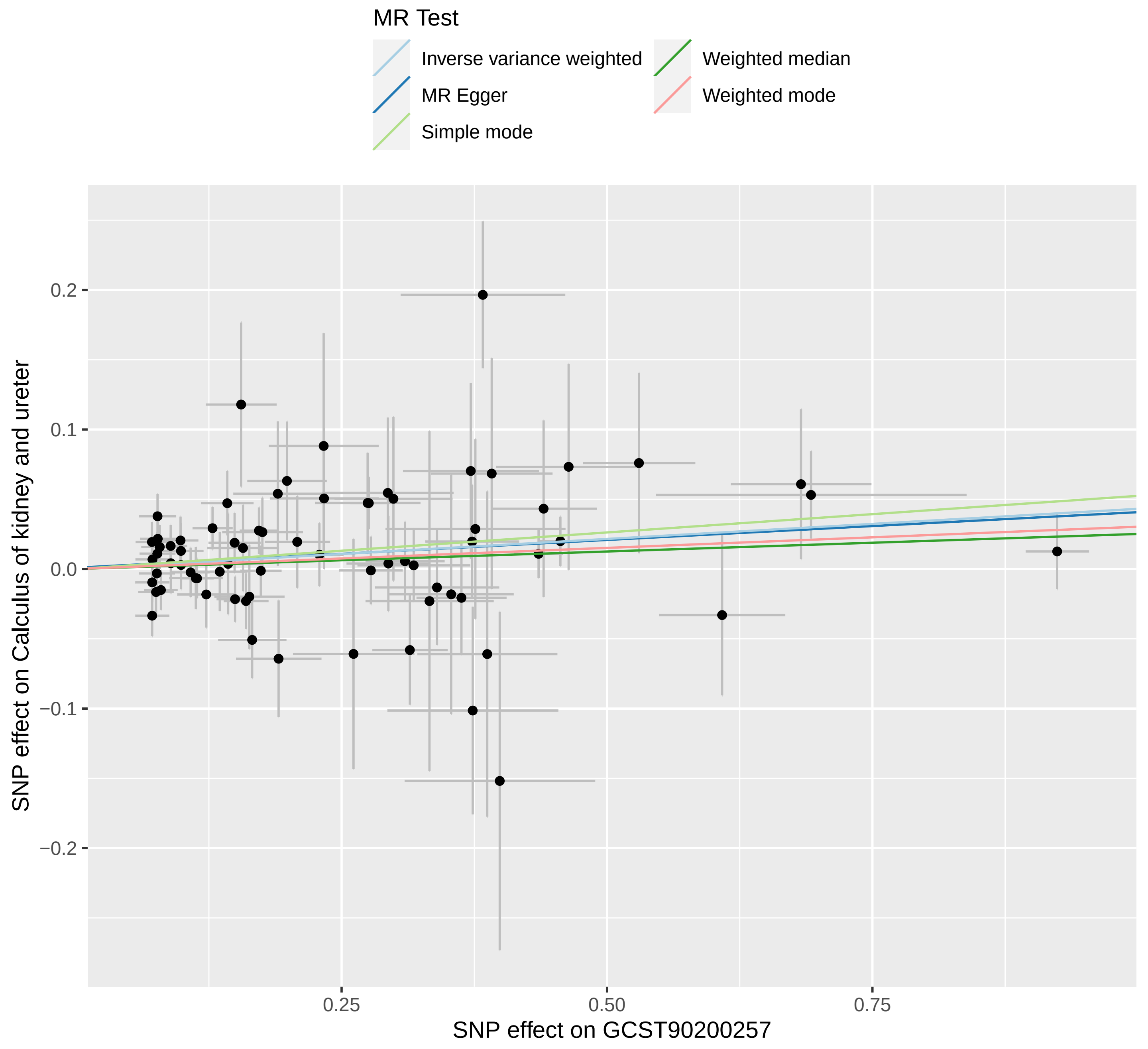

MR Test
Inverse variance weighted
MR Egger
Simple mode
Weighted median
Weighted mode
SNP effect on Calculus of kidney and ureter
0.2
0.1
0.0
−0.1
−0.2
0.25
0.50
0.75
SNP effect on GCST90200257

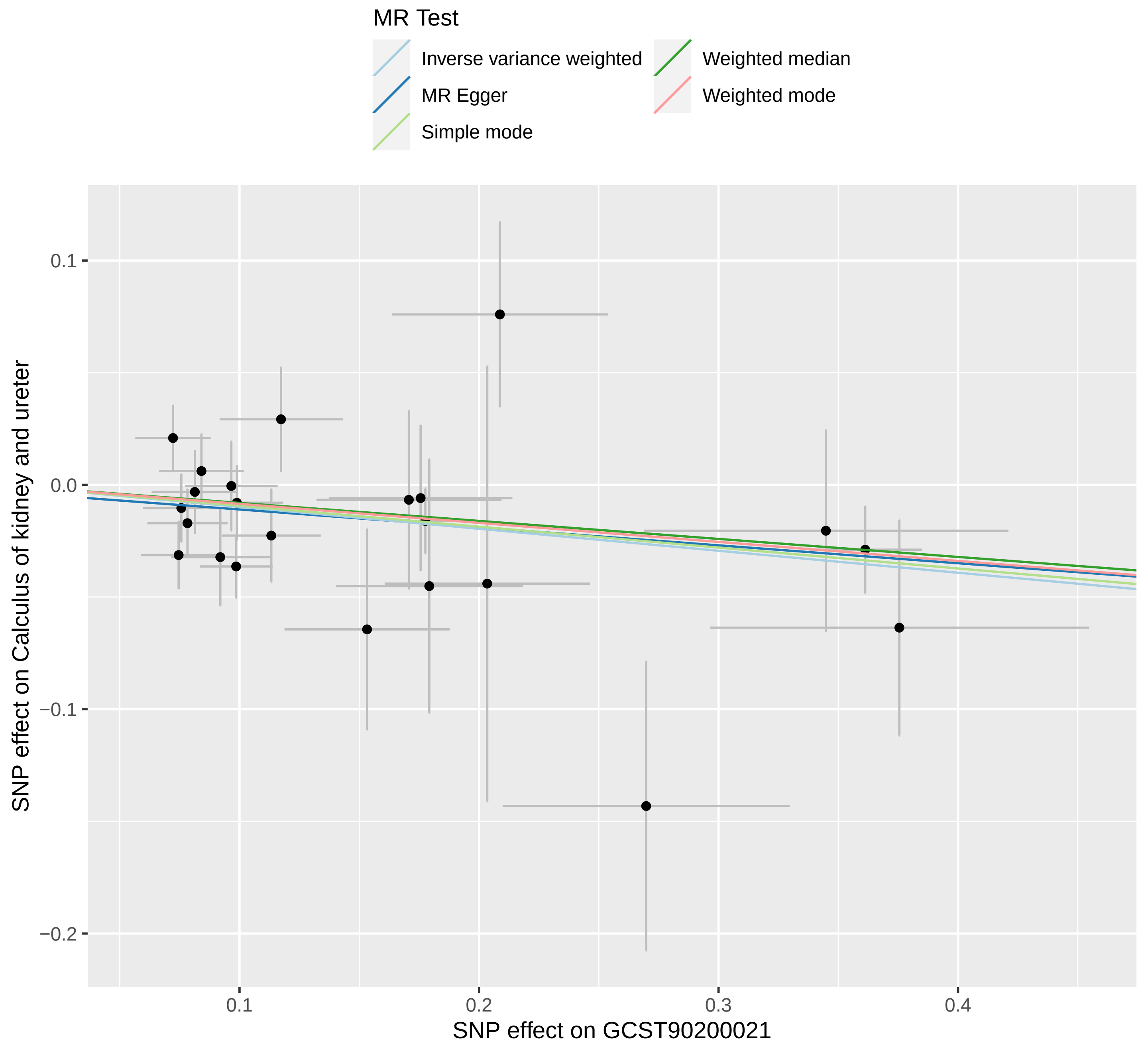

MR Test
Inverse variance weighted
MR Egger
Simple mode
Weighted median
Weighted mode
0.1
0.0
−0.1
−0.2
0.1
0.2
0.3
0.4
SNP effect on GCST90200021
SNP effect on Calculus of kidney and ureter

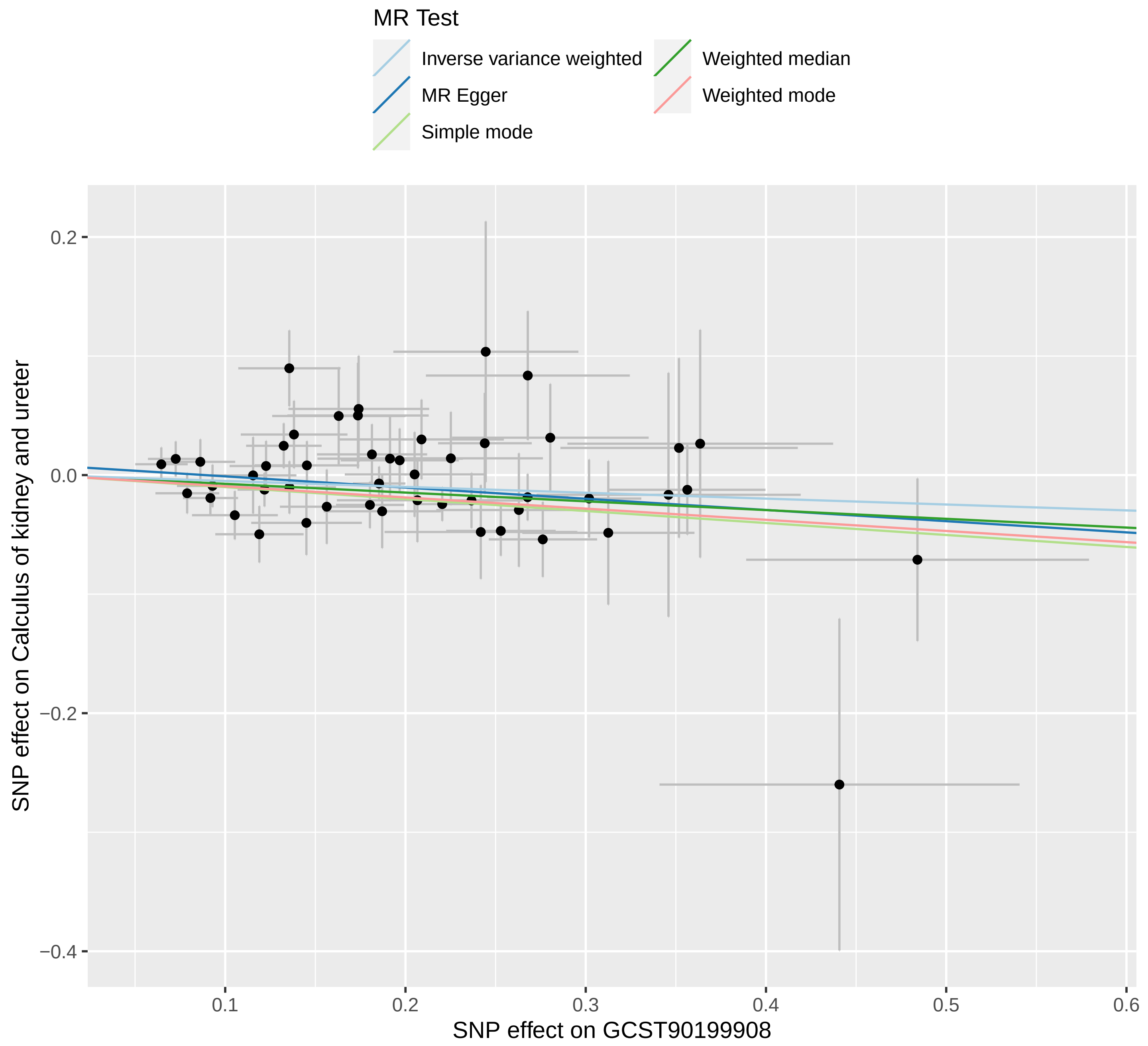

MR Test
Inverse variance weighted
MR Egger
Simple mode
Weighted median
Weighted mode
0.2
0.0
−0.2
−0.4
0.1
0.2
0.3
0.4
0.5
0.6
SNP effect on GCST90199908
SNP effect on Calculus of kidney and ureter

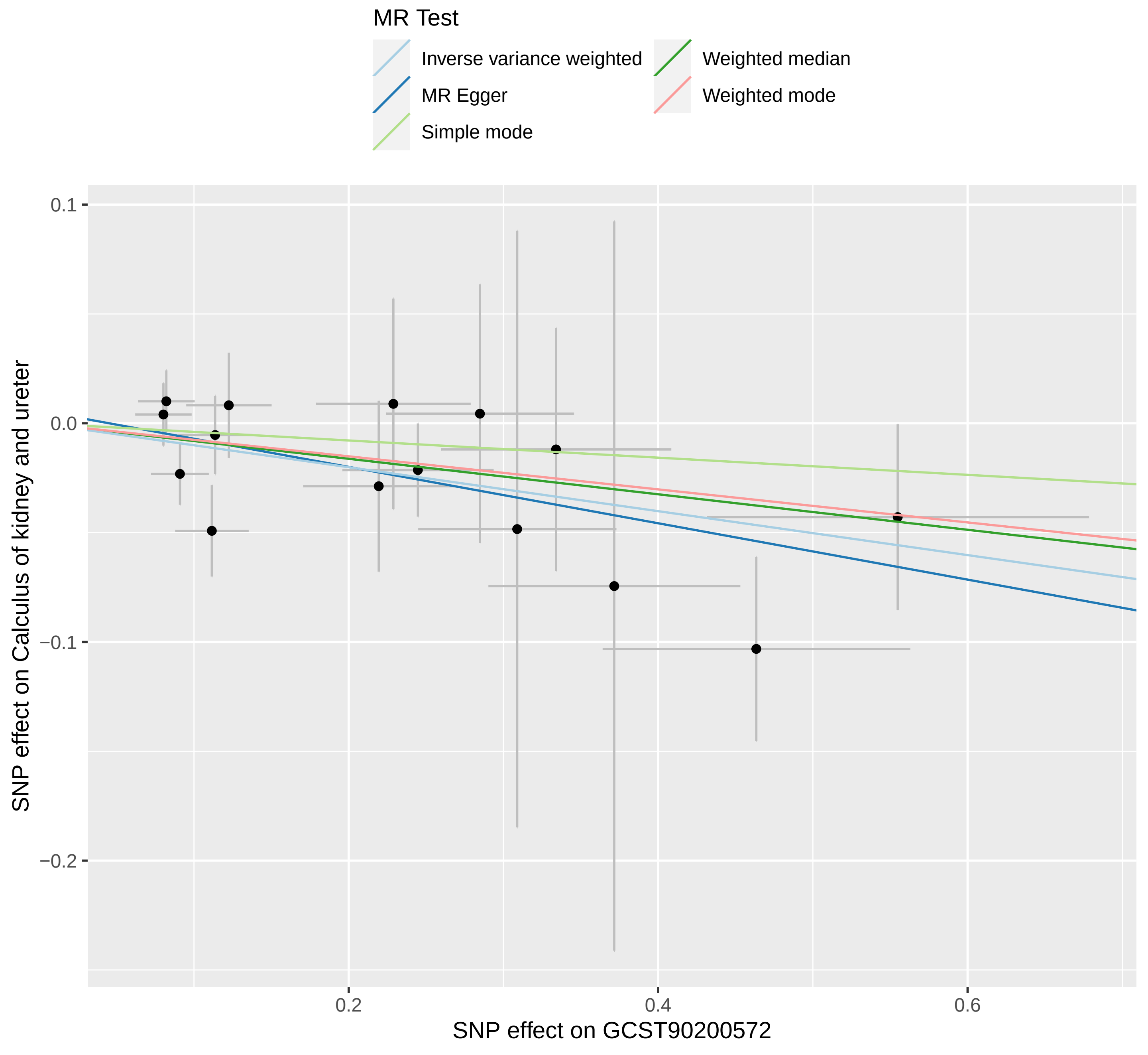

MR Test
Inverse variance weighted
MR Egger
Simple mode
Weighted median
Weighted mode
0.1
0.0
−0.1
−0.2
0.2
0.4
0.6
SNP effect on Calculus of kidney and ureter
SNP effect on GCST90200572

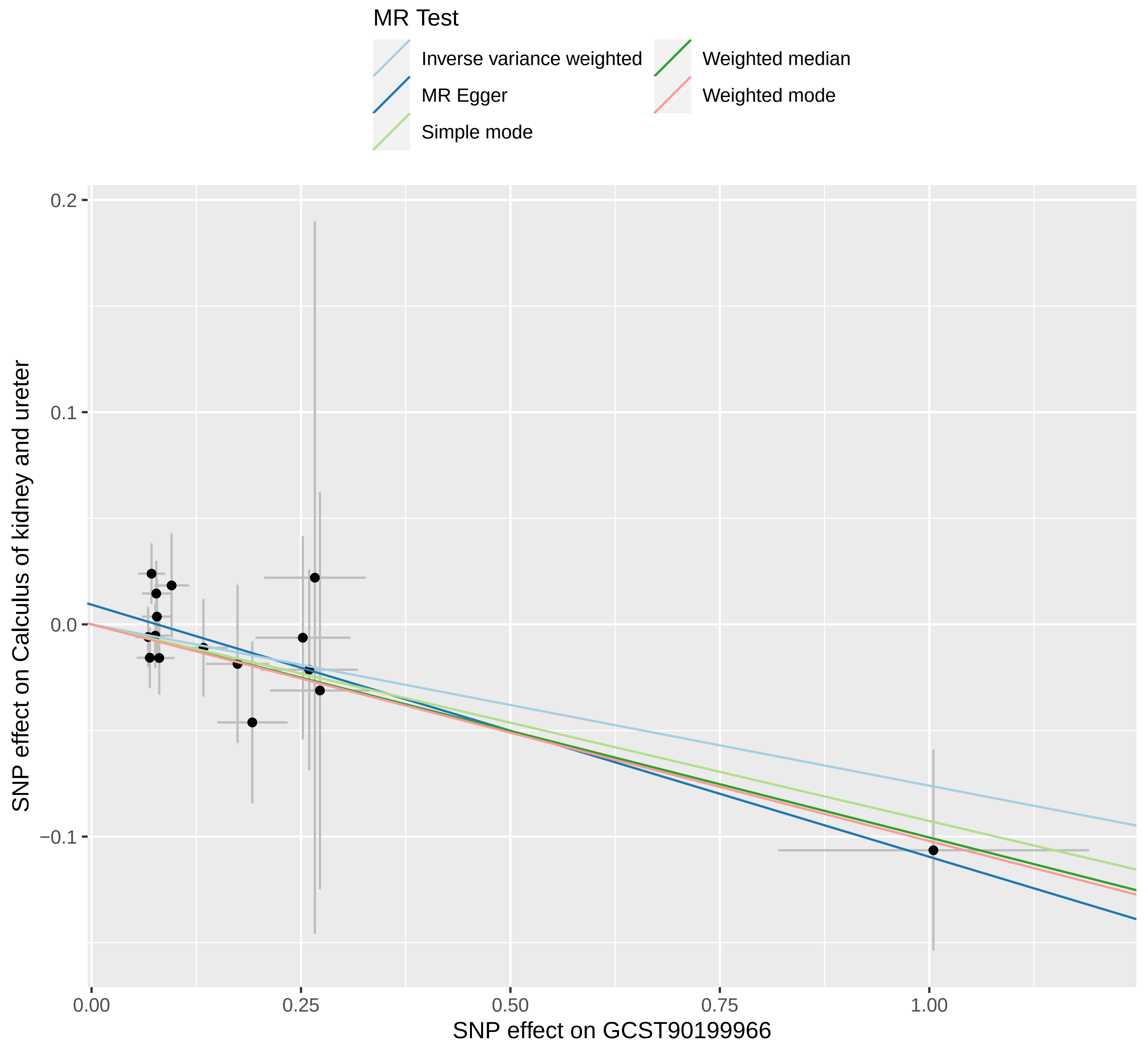
MR Test
Inverse variance weighted
MR Egger
Simple mode
Weighted median
Weighted mode
0.2
0.1
0.0
−0.1
0.00
0.25
0.50
0.75
1.00
SNP effect on GCST90199966
SNP effect on Calculus of kidney and ureter

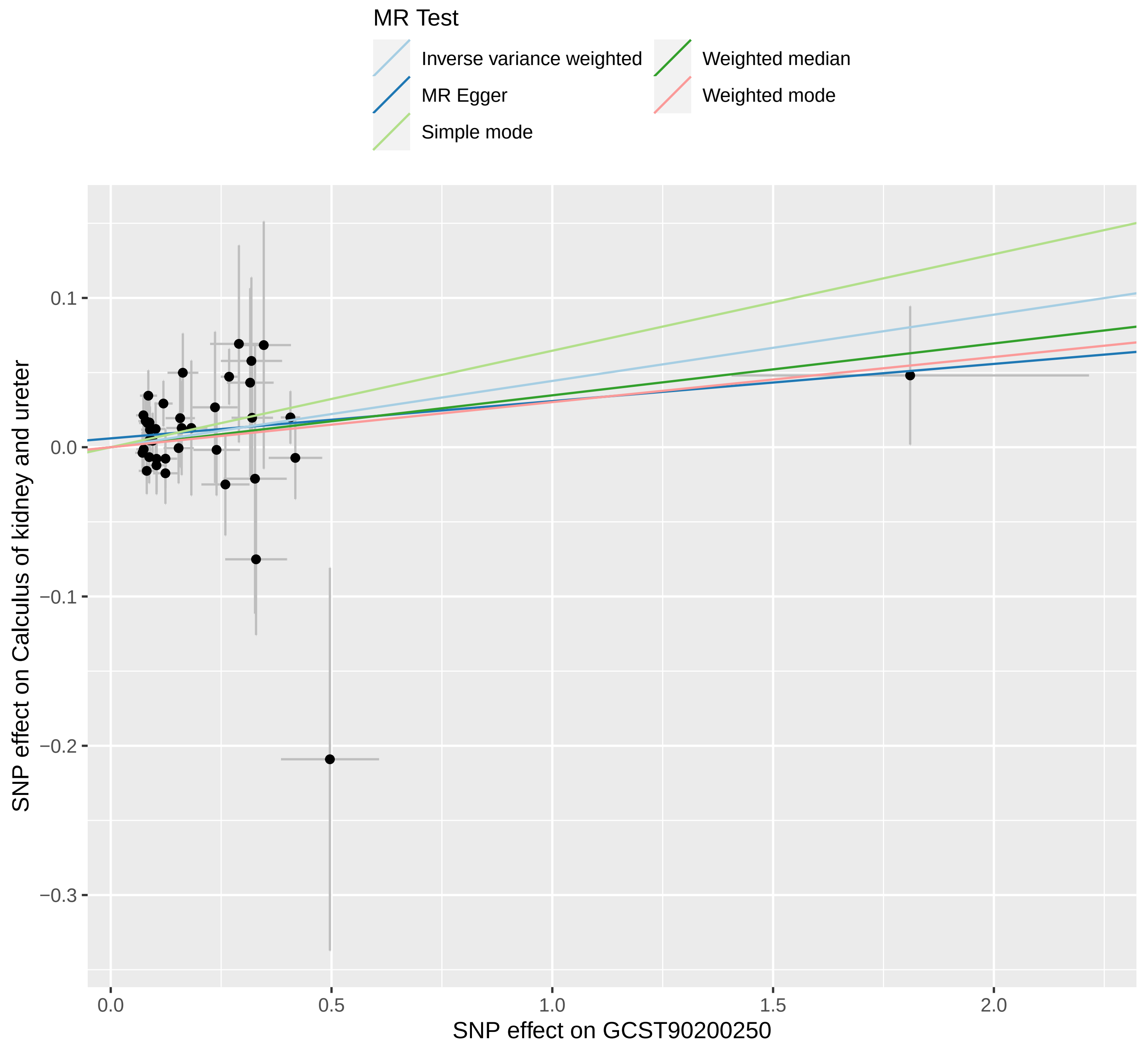
MR Test
Inverse variance weighted
MR Egger
Simple mode
Weighted median
Weighted mode
0.1
0.0
−0.1
−0.2
−0.3
0.0
0.5
1.0
1.5
2.0
SNP effect on GCST90200250
SNP effect on Calculus of kidney and ureter

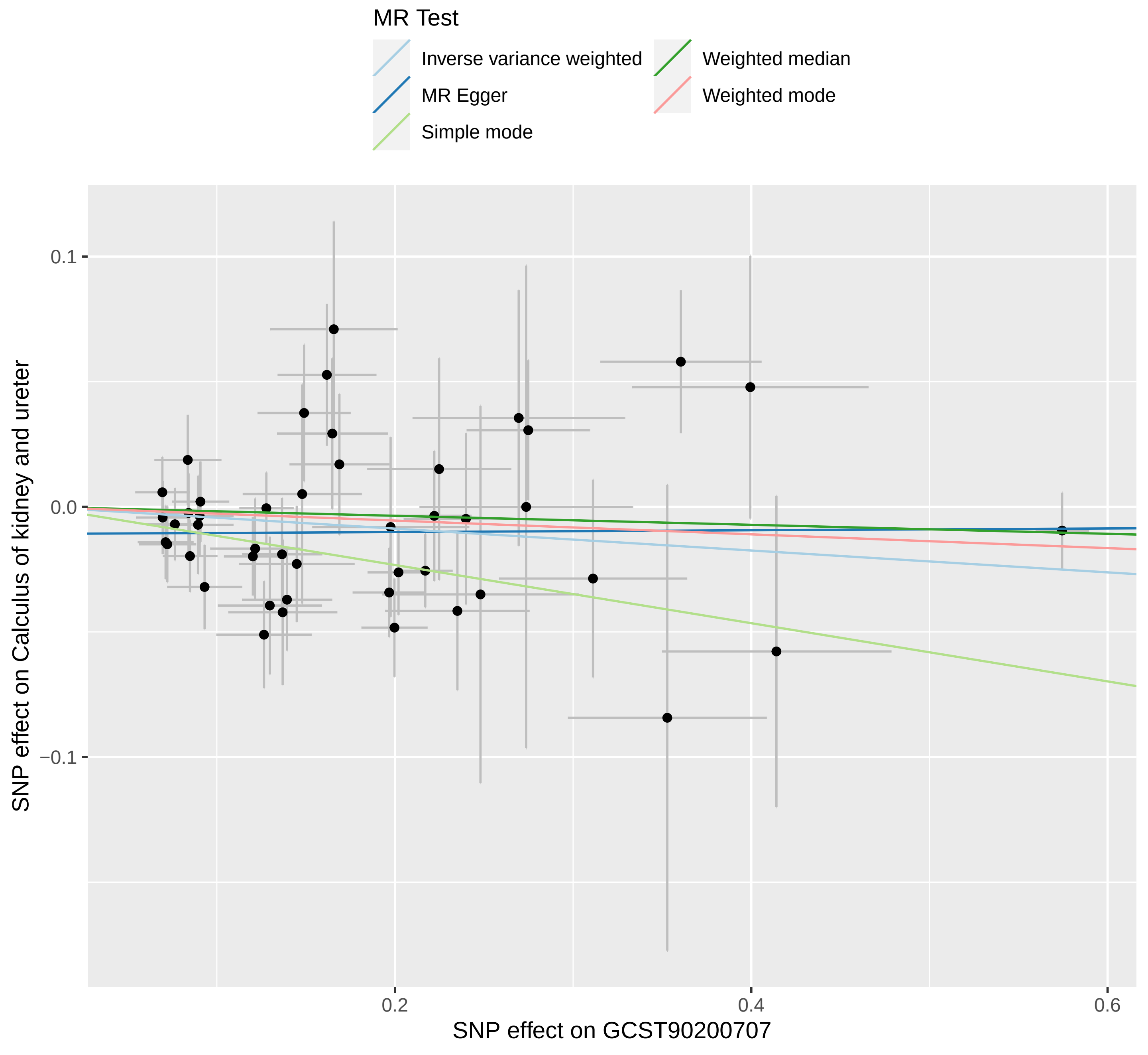
MR Test
Inverse variance weighted
MR Egger
Simple mode
Weighted median
Weighted mode
0.1
0.0
−0.1
0.2
0.4
0.6
SNP effect on Calculus of kidney and ureter
SNP effect on GCST90200707

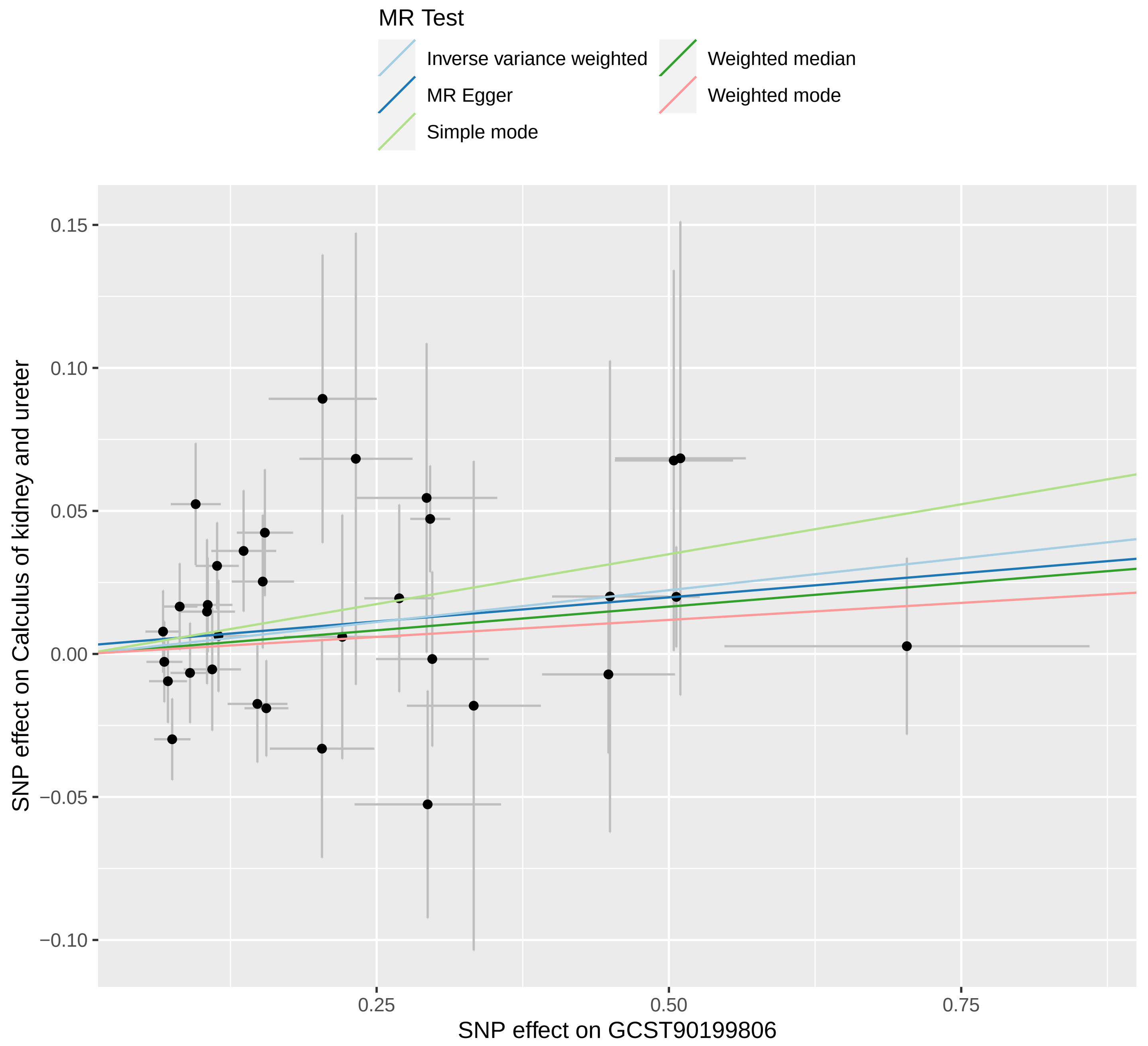
MR Test
Inverse variance weighted
MR Egger
Simple mode
Weighted median
Weighted mode
0.15
0.10
0.05
0.00
−0.05
−0.10
SNP effect on Calculus of kidney and ureter
0.25
0.50
0.75
SNP effect on GCST90199806

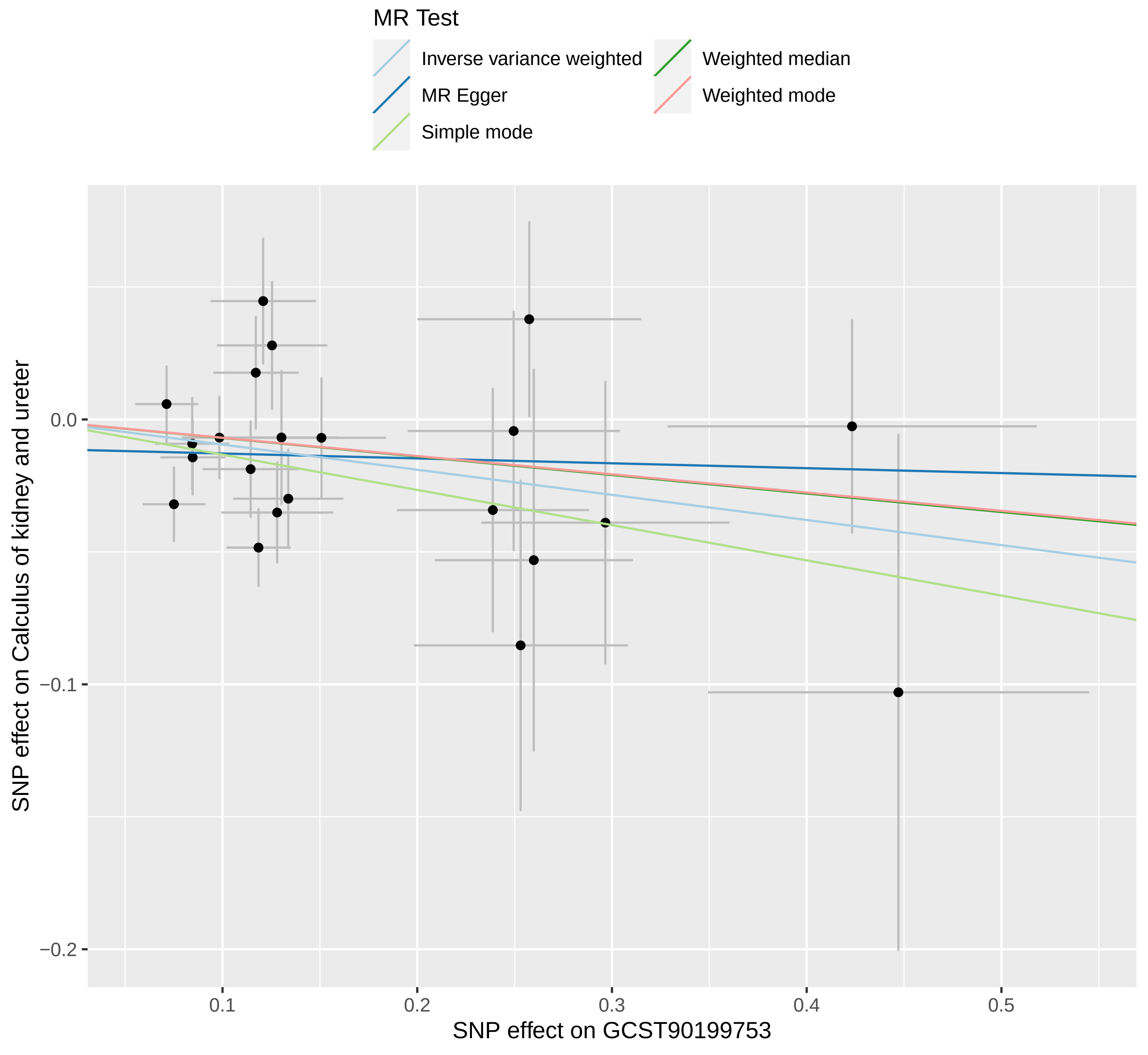
MR Test
Inverse variance weighted
MR Egger
Simple mode
Weighted median
Weighted mode
0.0
−0.1
−0.2
0.1
0.2
0.3
0.4
0.5
SNP effect on GCST90199753
SNP effect on Calculus of kidney and ureter

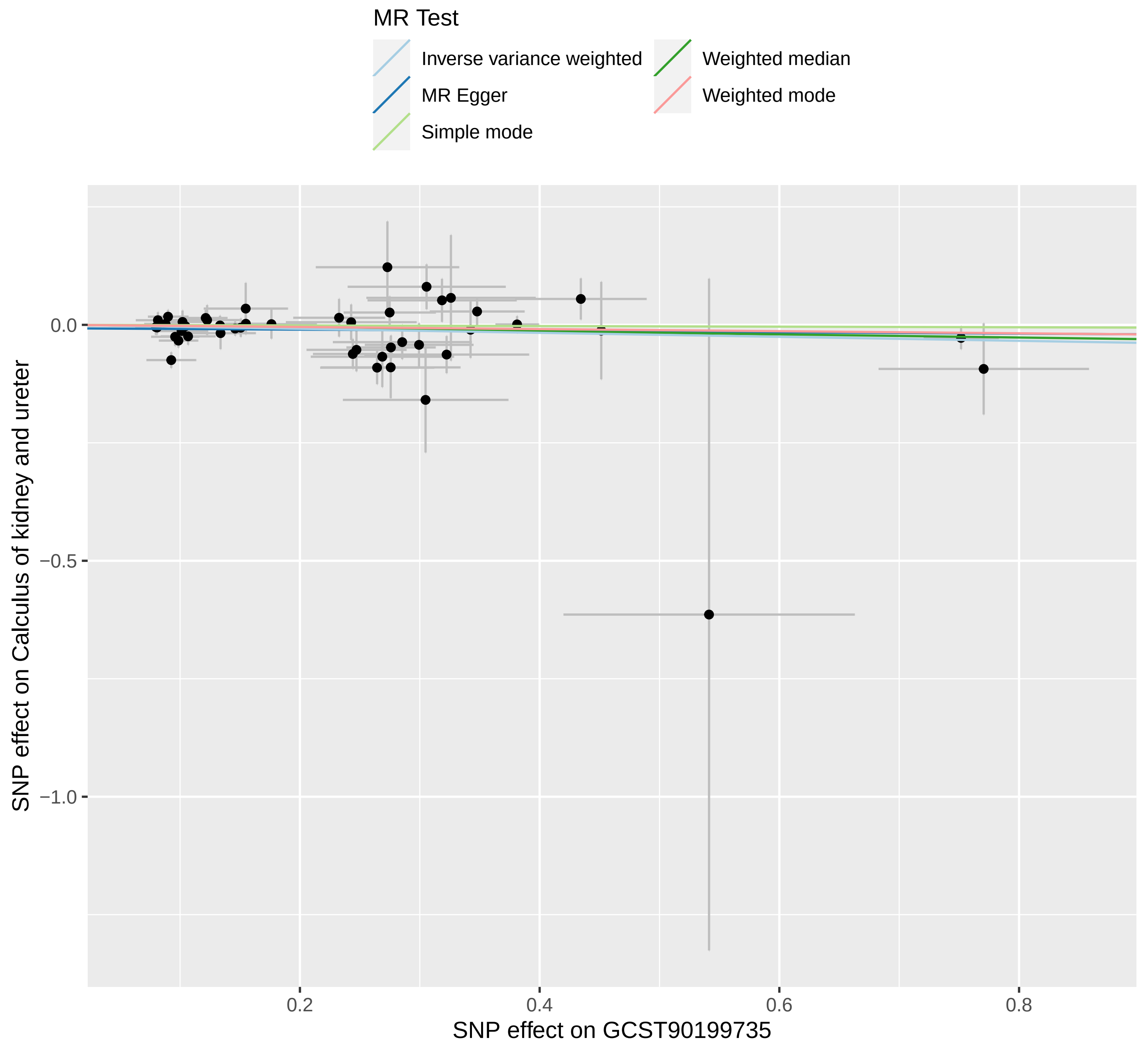
MR Test
Inverse variance weighted
MR Egger
Simple mode
Weighted median
Weighted mode
SNP effect on Calculus of kidney and ureter
0.0
−0.5
−1.0
0.2
0.4
0.6
0.8
SNP effect on GCST90199735

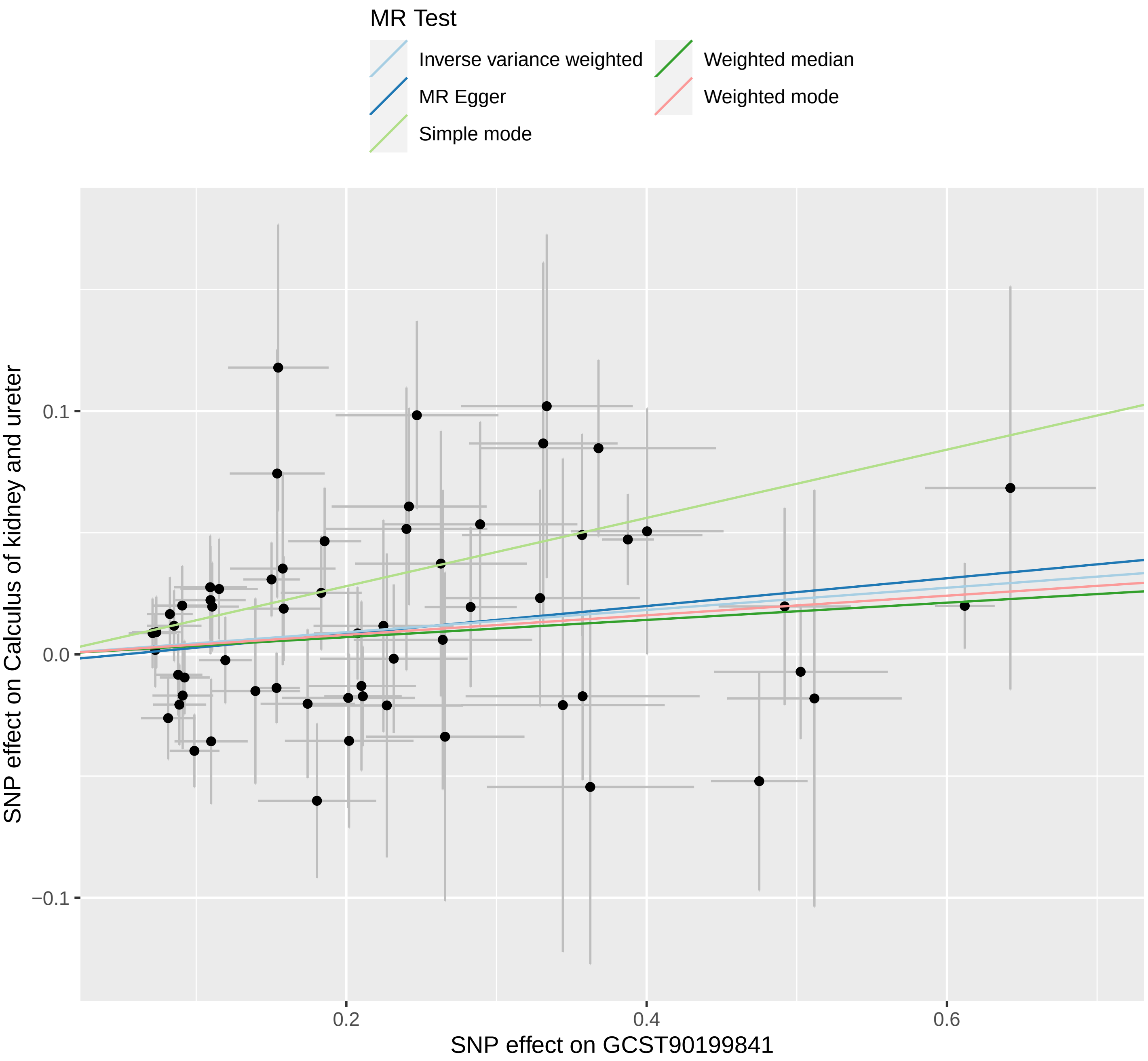
MR Test
Inverse variance weighted
MR Egger
Simple mode
Weighted median
Weighted mode
0.1
0.0
−0.1
0.2
0.4
0.6
SNP effect on Calculus of kidney and ureter
SNP effect on GCST90199841

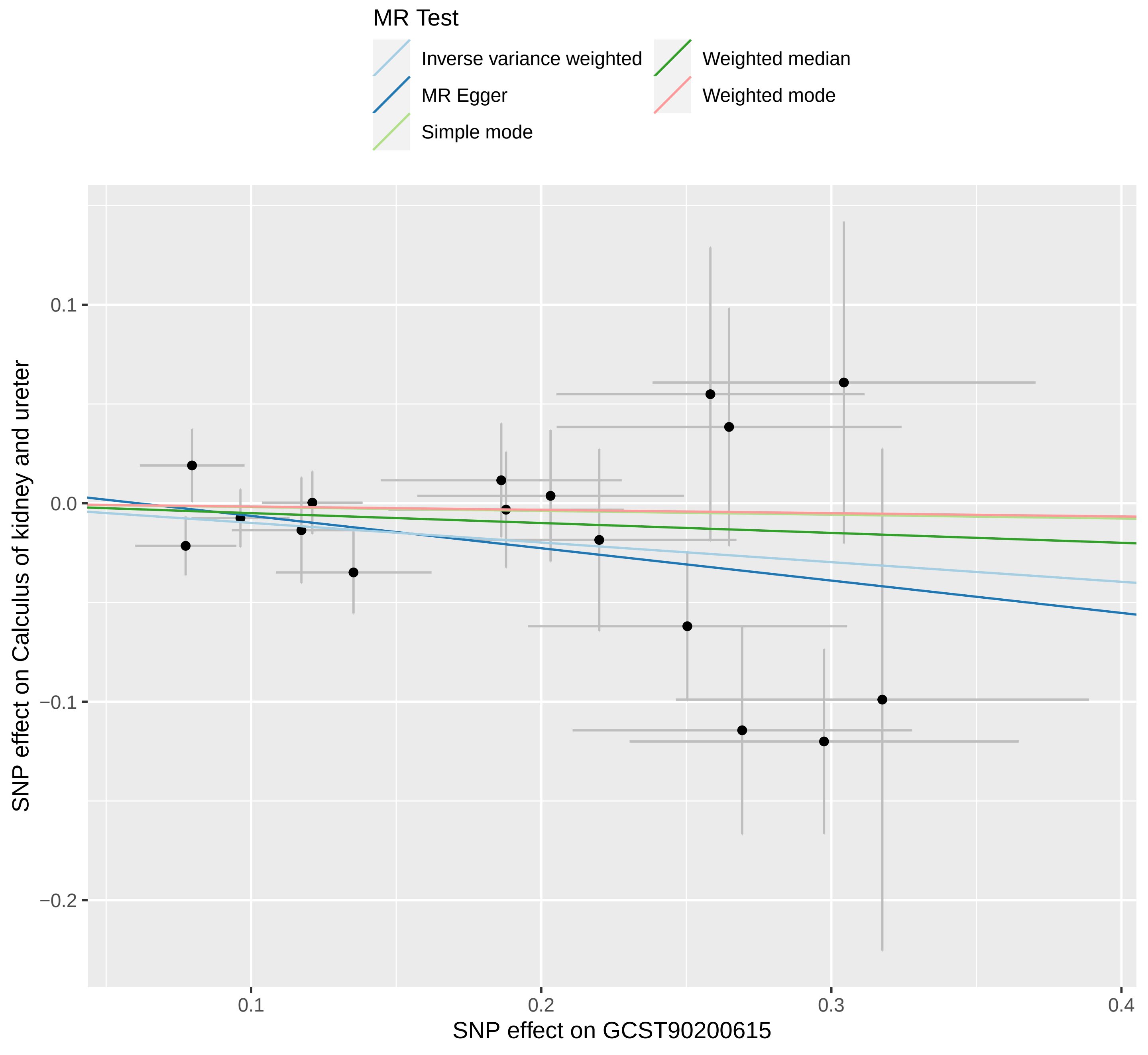
MR Test
Inverse variance weighted
MR Egger
Simple mode
Weighted median
Weighted mode
0.1
0.0
−0.1
−0.2
0.1
0.2
0.3
0.4
SNP effect on GCST90200615
SNP effect on Calculus of kidney and ureter

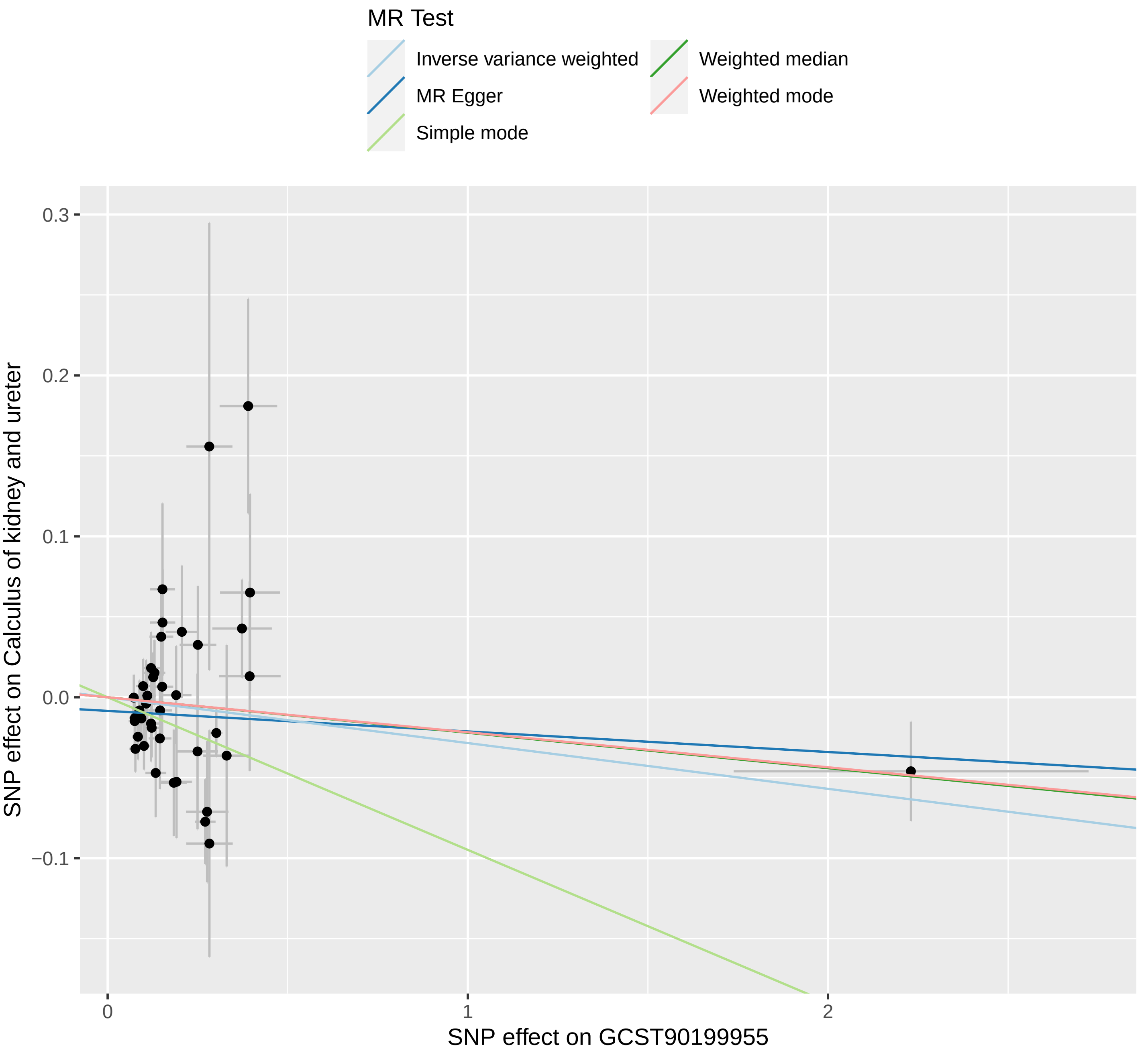

MR Test
Inverse variance weighted
MR Egger
Simple mode
Weighted median
Weighted mode
0.3
0.2
0.1
0.0
−0.1
0
1
2
SNP effect on Calculus of kidney and ureter
SNP effect on GCST90199955

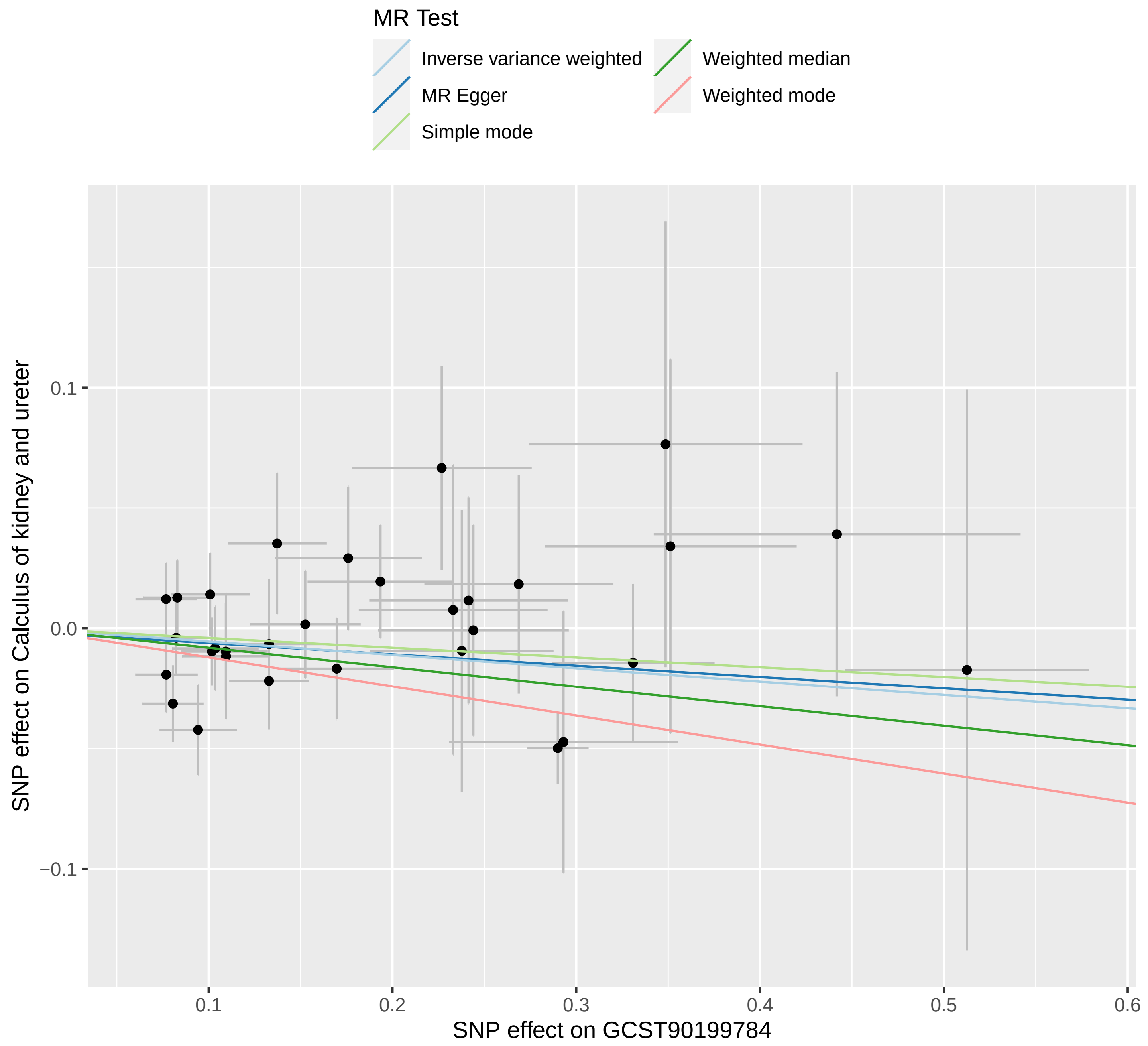
MR Test
Inverse variance weighted
MR Egger
Simple mode
Weighted median
Weighted mode
0.1
0.0
−0.1
0.1
0.2
0.3
0.4
0.5
0.6
SNP effect on GCST90199784
SNP effect on Calculus of kidney and ureter

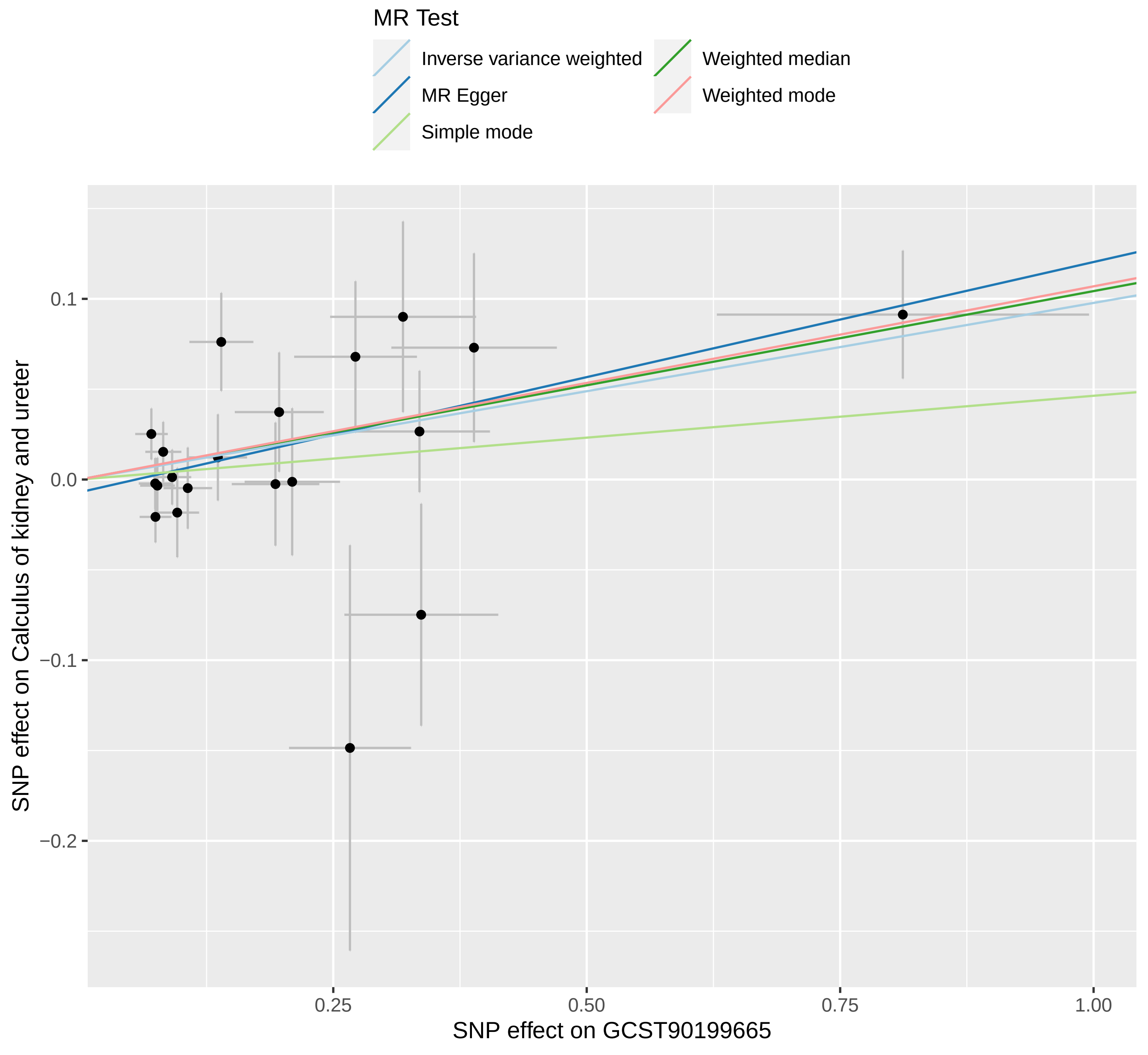

MR Test
Inverse variance weighted
MR Egger
Simple mode
Weighted median
Weighted mode
0.1
0.0
−0.1
−0.2
0.25
0.50
0.75
1.00
SNP effect on Calculus of kidney and ureter
SNP effect on GCST90199665

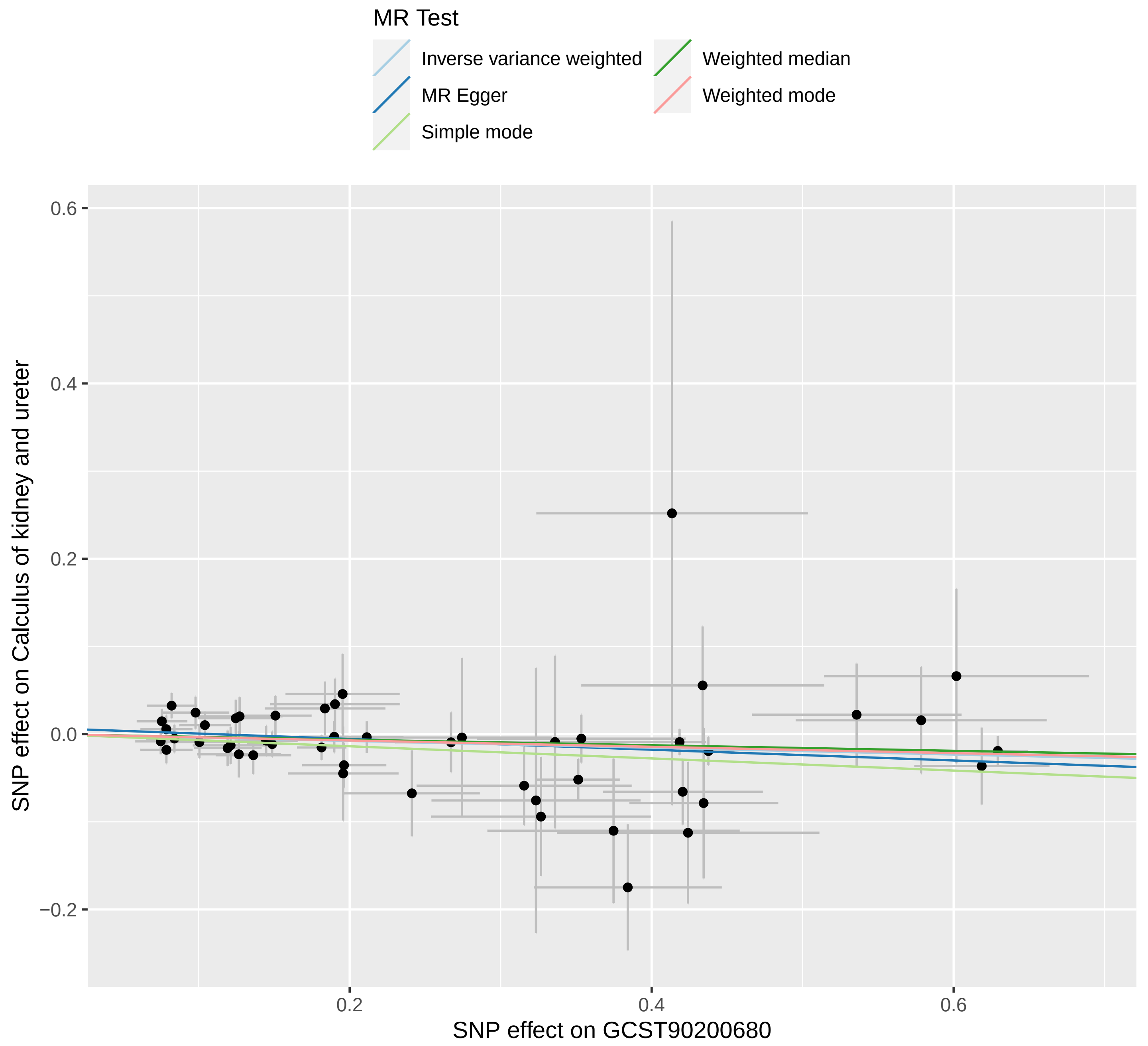
MR Test
Inverse variance weighted
MR Egger
Simple mode
Weighted median
Weighted mode
0.6
0.4
0.2
0.0
−0.2
0.2
0.4
0.6
SNP effect on Calculus of kidney and ureter
SNP effect on GCST90200680

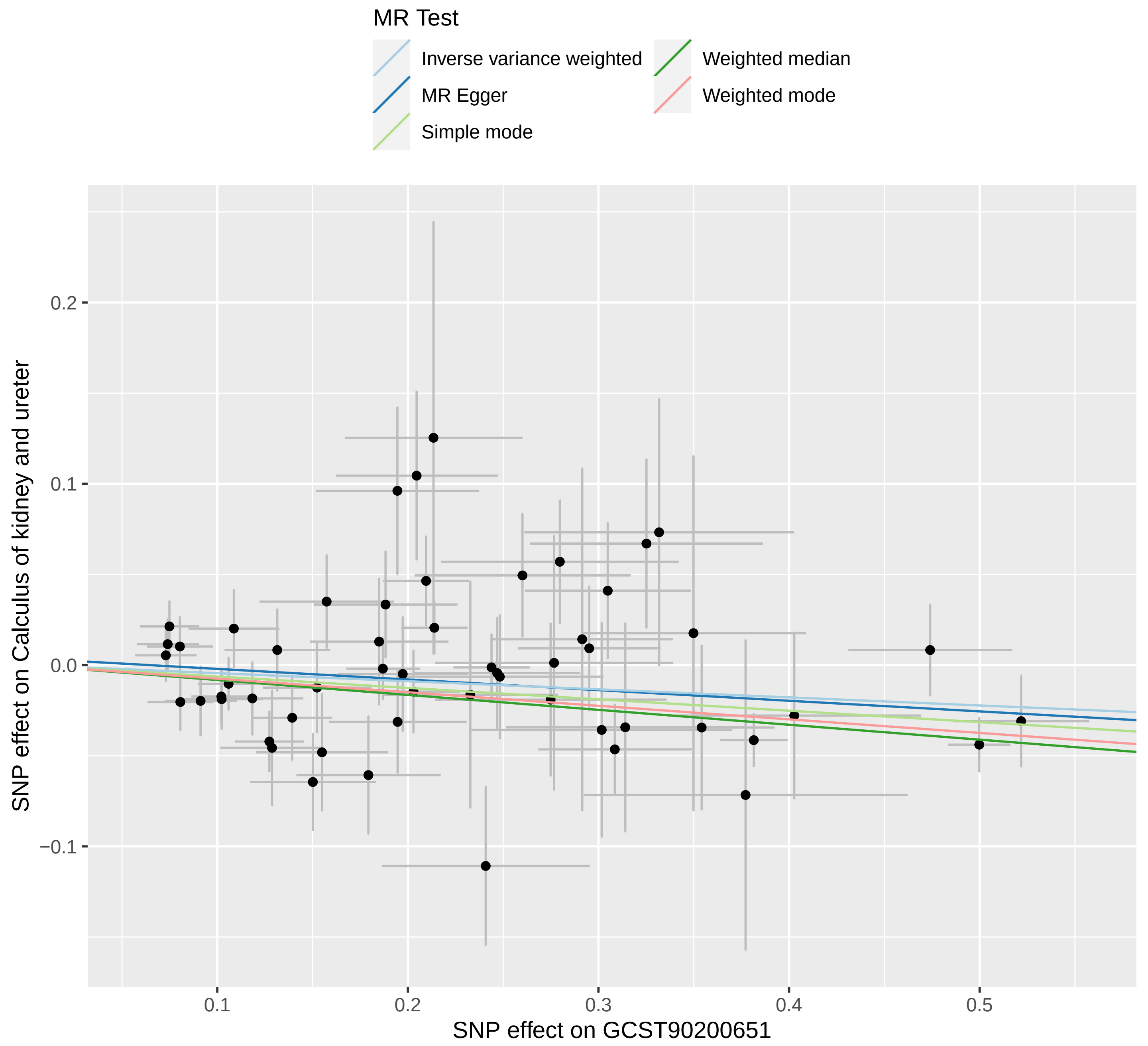

MR Test
Inverse variance weighted
MR Egger
Simple mode
Weighted median
Weighted mode
0.2
0.1
0.0
−0.1
0.1
0.2
0.3
0.4
0.5
SNP effect on Calculus of kidney and ureter
SNP effect on GCST90200651

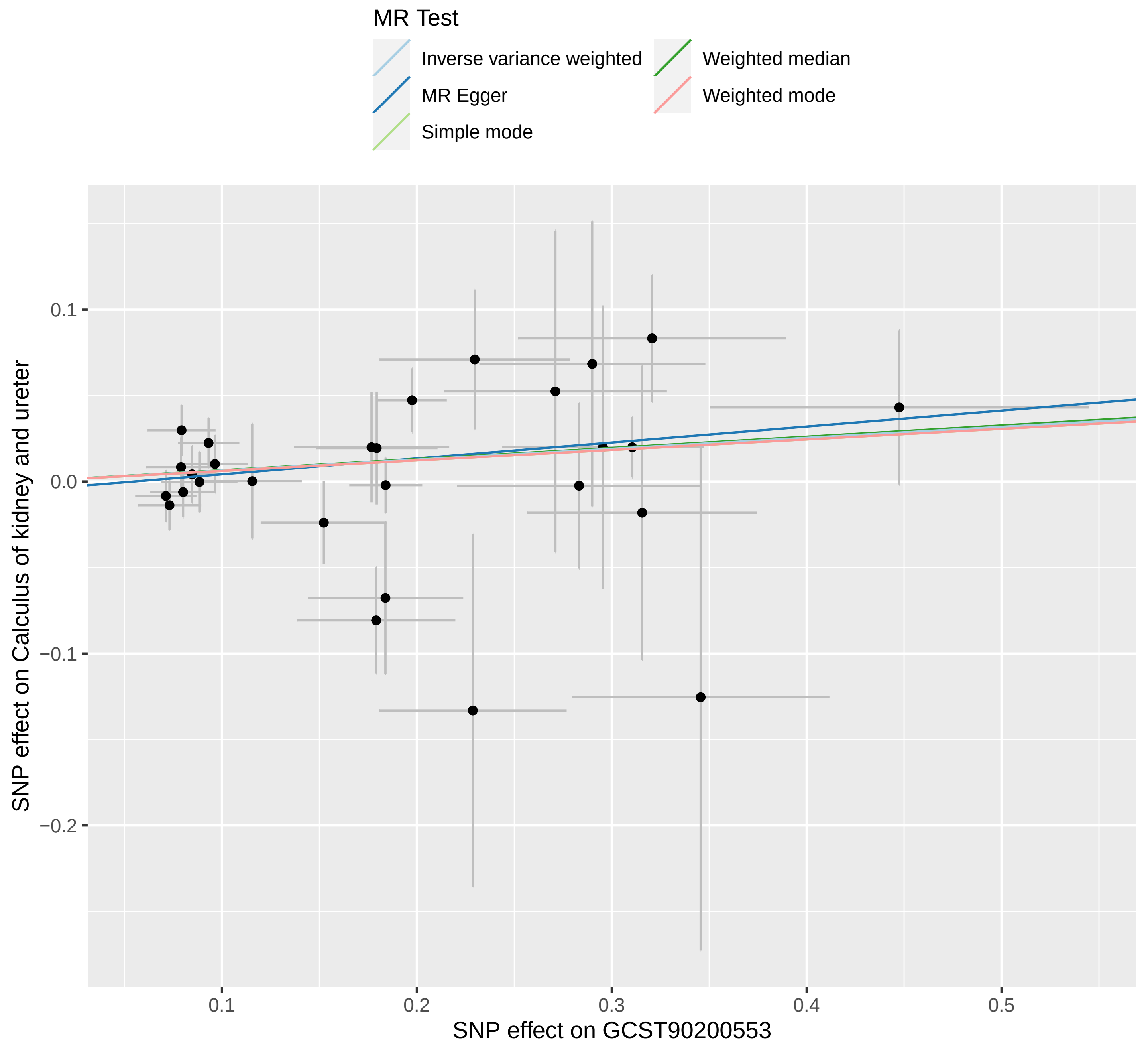

MR Test
Inverse variance weighted
MR Egger
Simple mode
Weighted median
Weighted mode
0.1
0.0
−0.1
−0.2
SNP effect on Calculus of kidney and ureter
0.1
0.2
0.3
0.4
0.5
SNP effect on GCST90200553

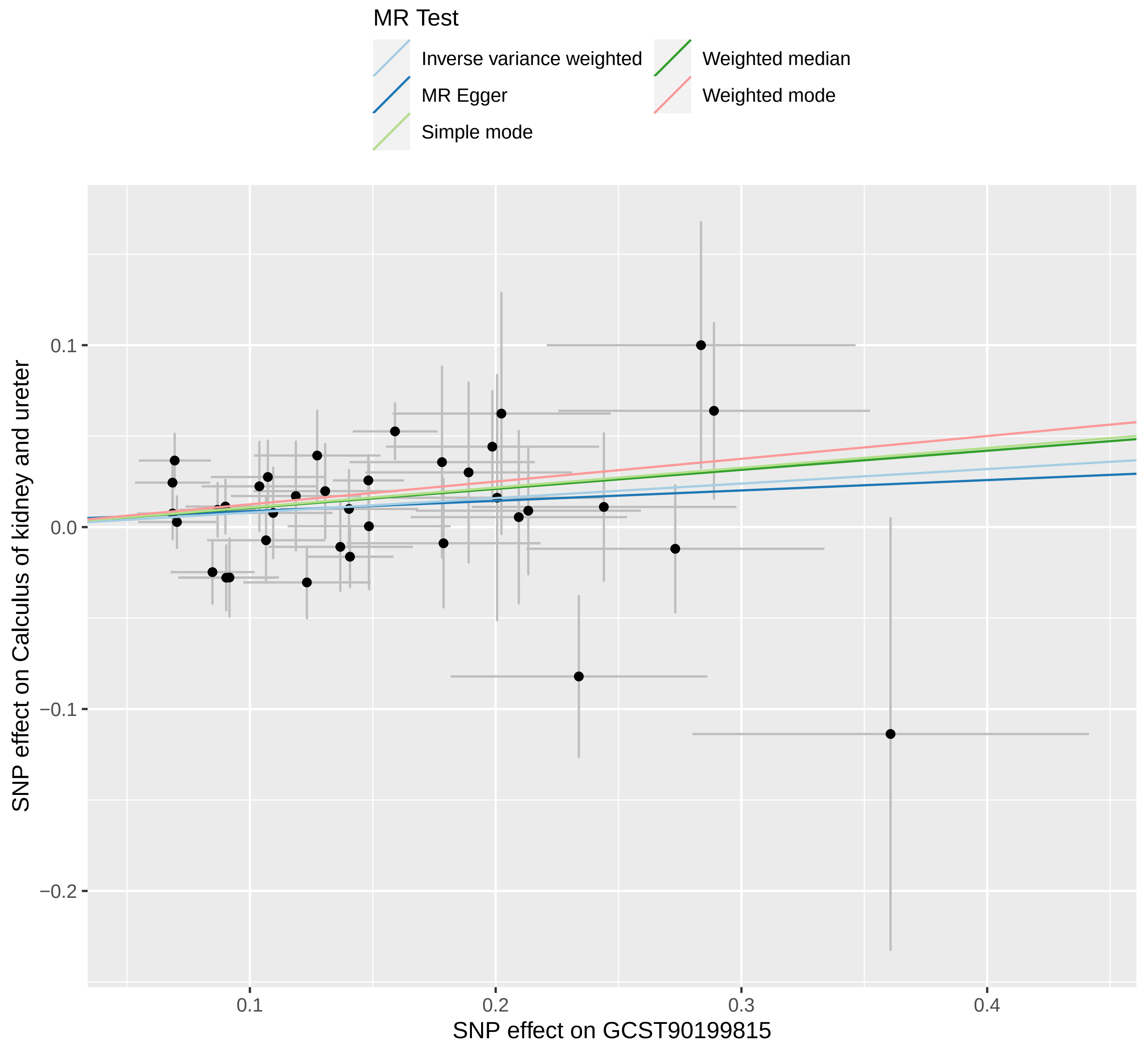

MR Test
Inverse variance weighted
MR Egger
Simple mode
Weighted median
Weighted mode
SNP effect on Calculus of kidney and ureter
0.1
0.0
−0.1
−0.2
0.1
0.2
0.3
0.4
SNP effect on GCST90199815

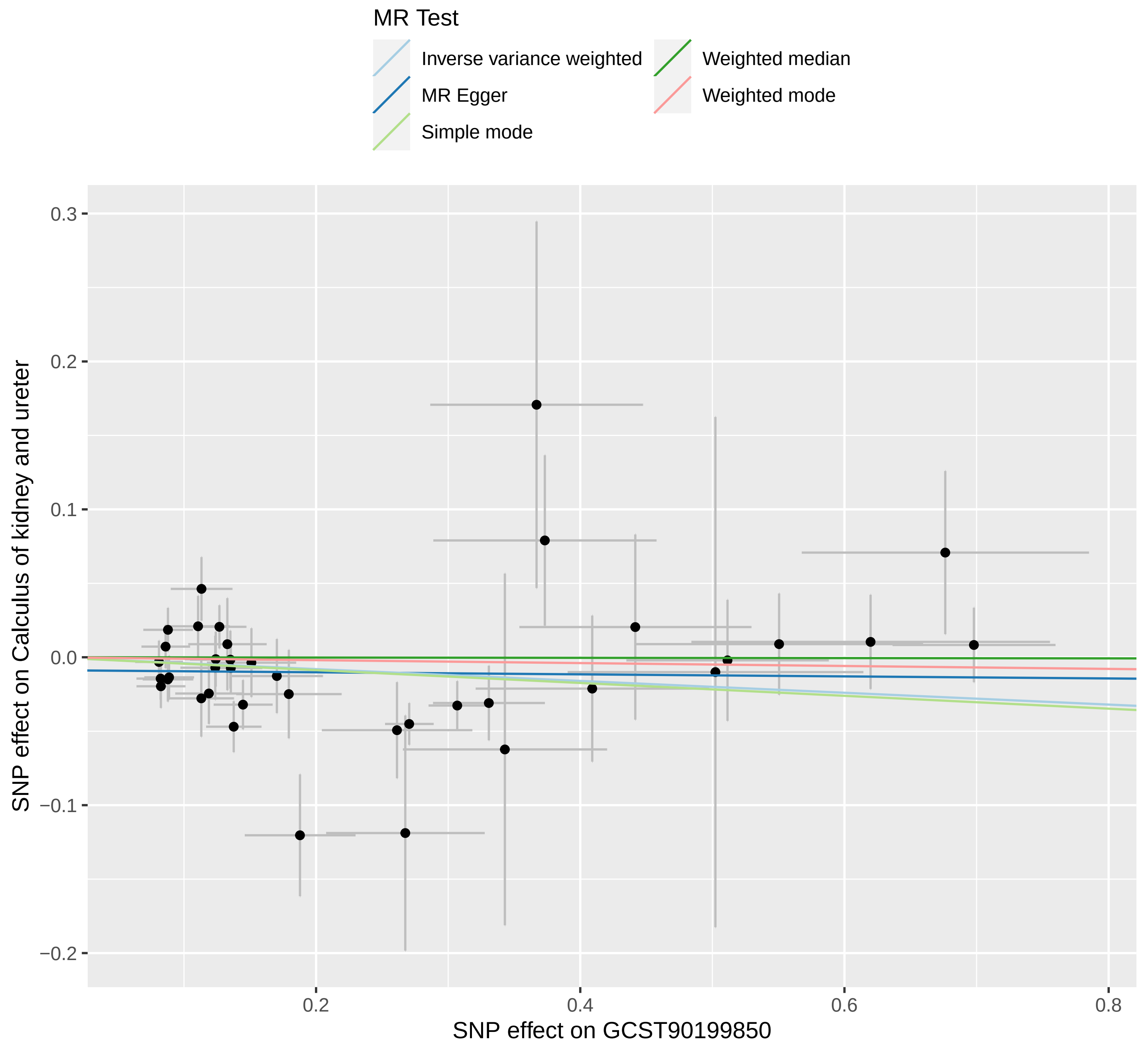

MR Test
Inverse variance weighted
MR Egger
Simple mode
Weighted median
Weighted mode
0.3
0.2
0.1
0.0
−0.1
−0.2
0.2
0.4
0.6
0.8
SNP effect on Calculus of kidney and ureter
SNP effect on GCST90199850

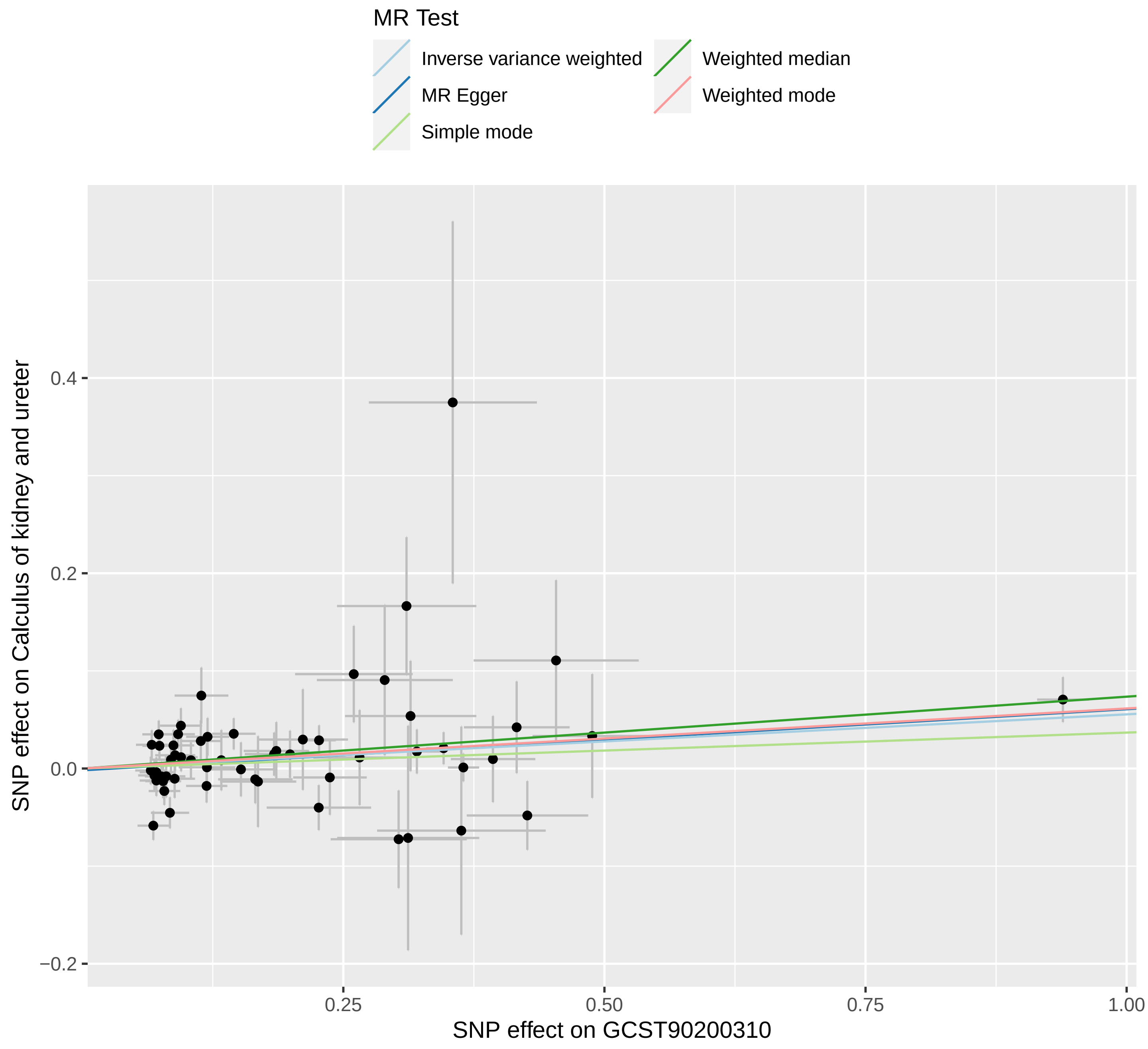

MR Test
Inverse variance weighted
MR Egger
Simple mode
Weighted median
Weighted mode
SNP effect on Calculus of kidney and ureter
0.4
0.2
0.0
−0.2
0.25
0.50
0.75
1.00
SNP effect on GCST90200310

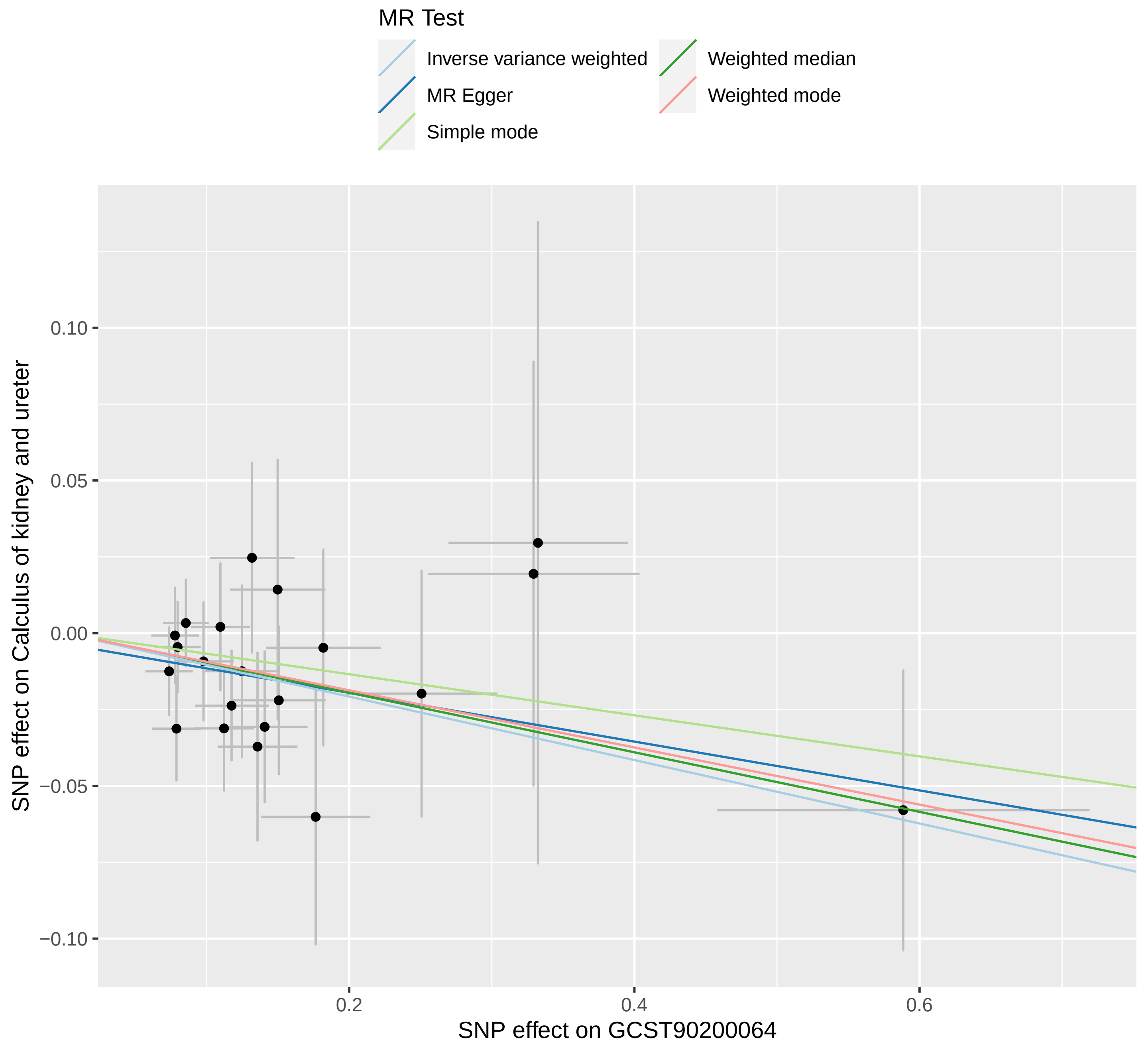

MR Test
Inverse variance weighted
MR Egger
Simple mode
Weighted median
Weighted mode
0.10
0.05
0.00
−0.05
−0.10
SNP effect on Calculus of kidney and ureter
0.2
0.4
0.6
SNP effect on GCST90200064

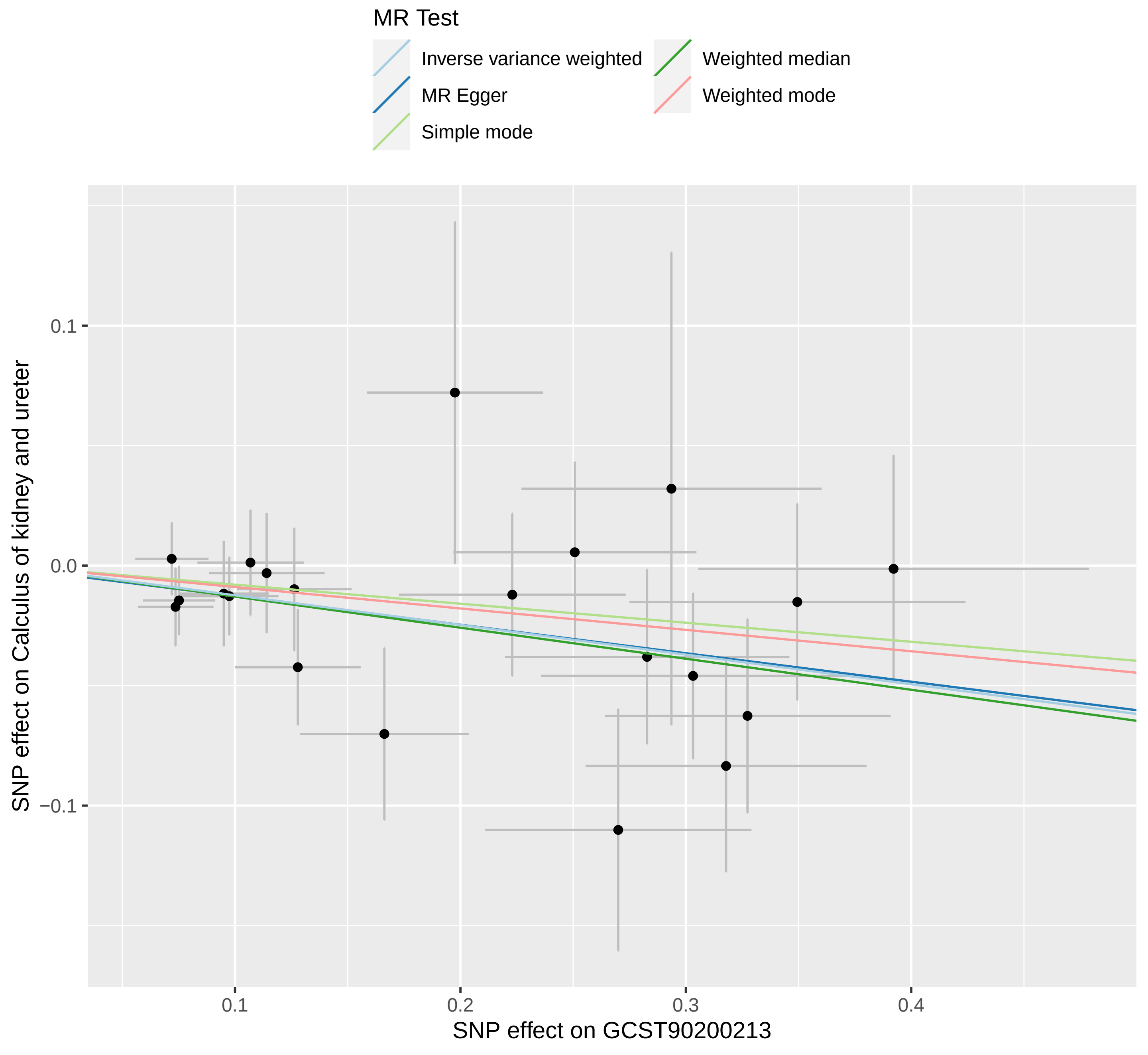
MR Test
Inverse variance weighted
MR Egger
Simple mode
Weighted median
Weighted mode
SNP effect on Calculus of kidney and ureter
0.1
0.0
−0.1
0.1
0.2
0.3
0.4
SNP effect on GCST90200213

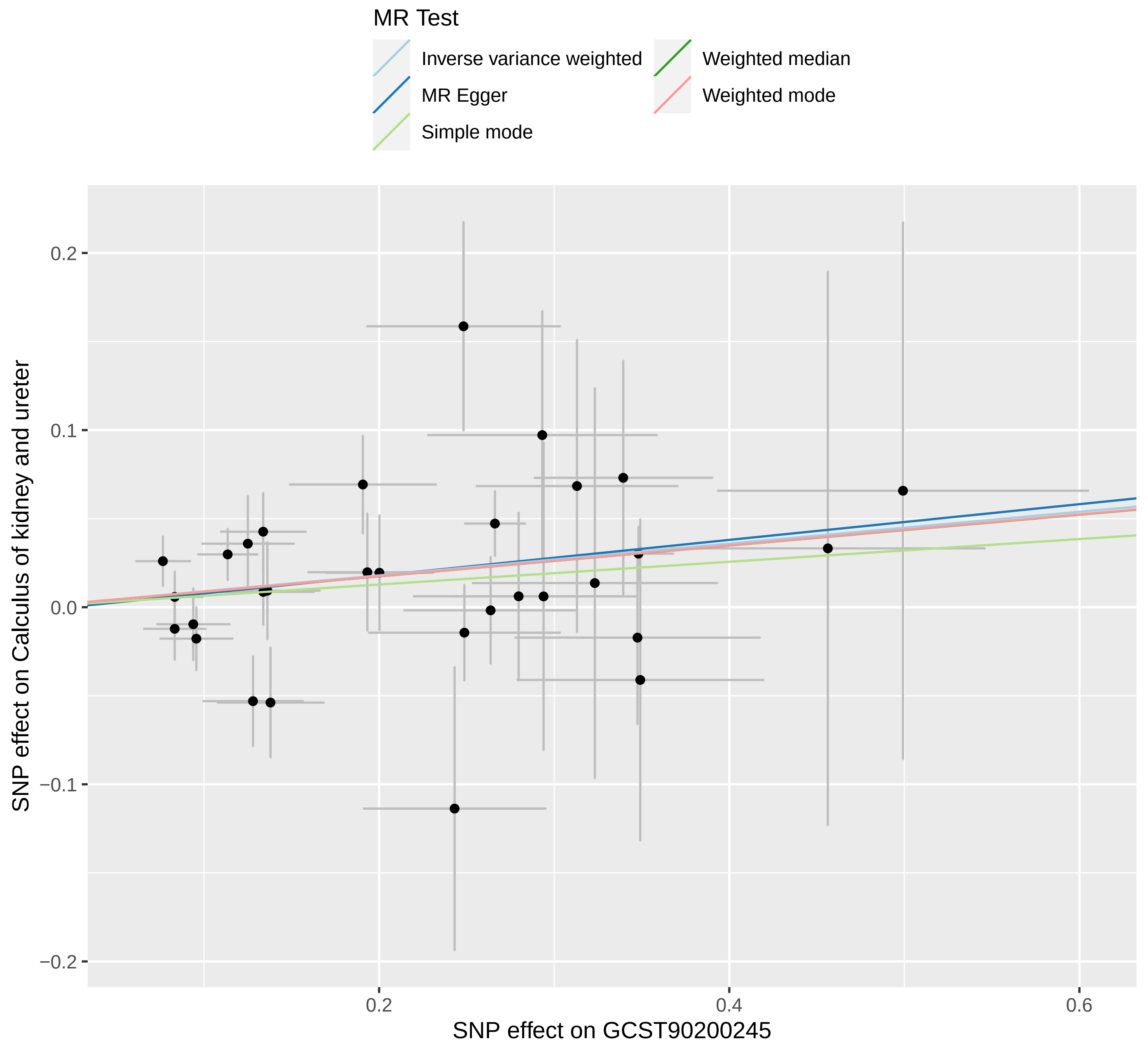

MR Test
Inverse variance weighted
MR Egger
Simple mode
Weighted median
Weighted mode
SNP effect on Calculus of kidney and ureter
0.2
0.1
0.0
−0.1
−0.2
0.2
0.4
0.6
SNP effect on GCST90200245

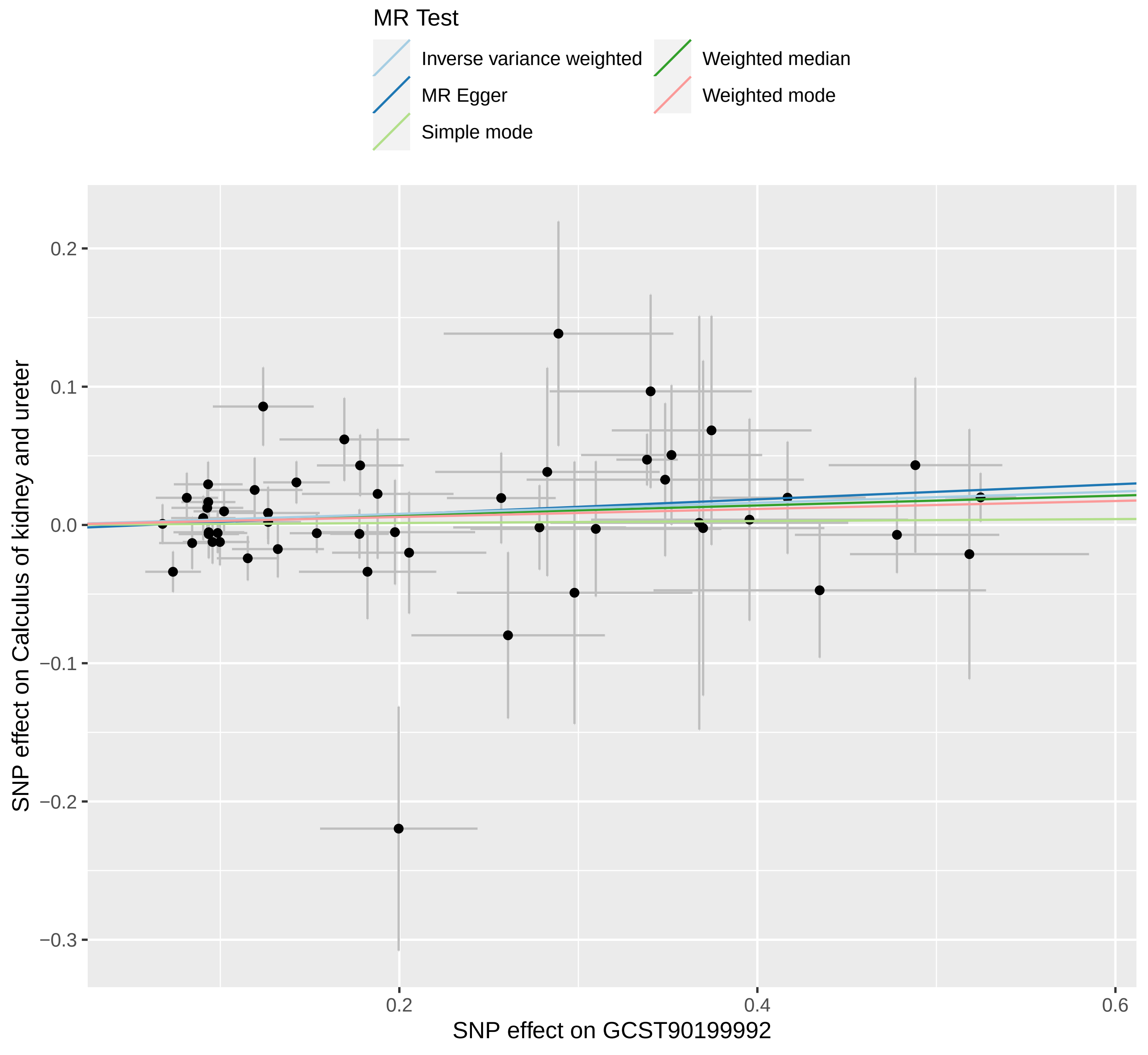

MR Test
Inverse variance weighted
MR Egger
Simple mode
Weighted median
Weighted mode
0.2
0.1
0.0
−0.1
−0.2
−0.3
SNP effect on Calculus of kidney and ureter
0.2
0.4
0.6
SNP effect on GCST90199992

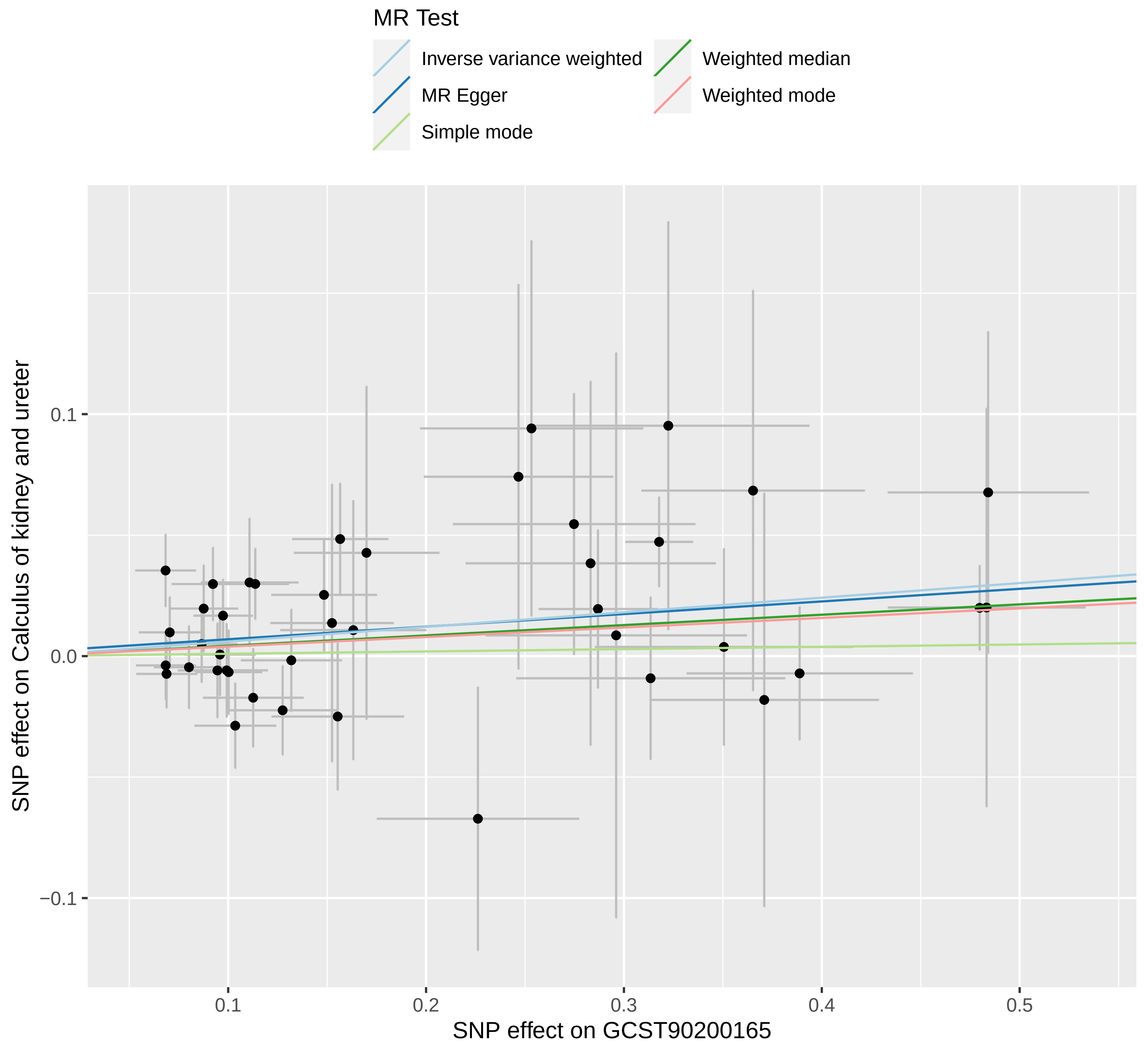
MR Test
Inverse variance weighted
MR Egger
Simple mode
Weighted median
Weighted mode
0.1
0.0
−0.1
0.1
0.2
0.3
0.4
0.5
SNP effect on Calculus of kidney and ureter
SNP effect on GCST90200165

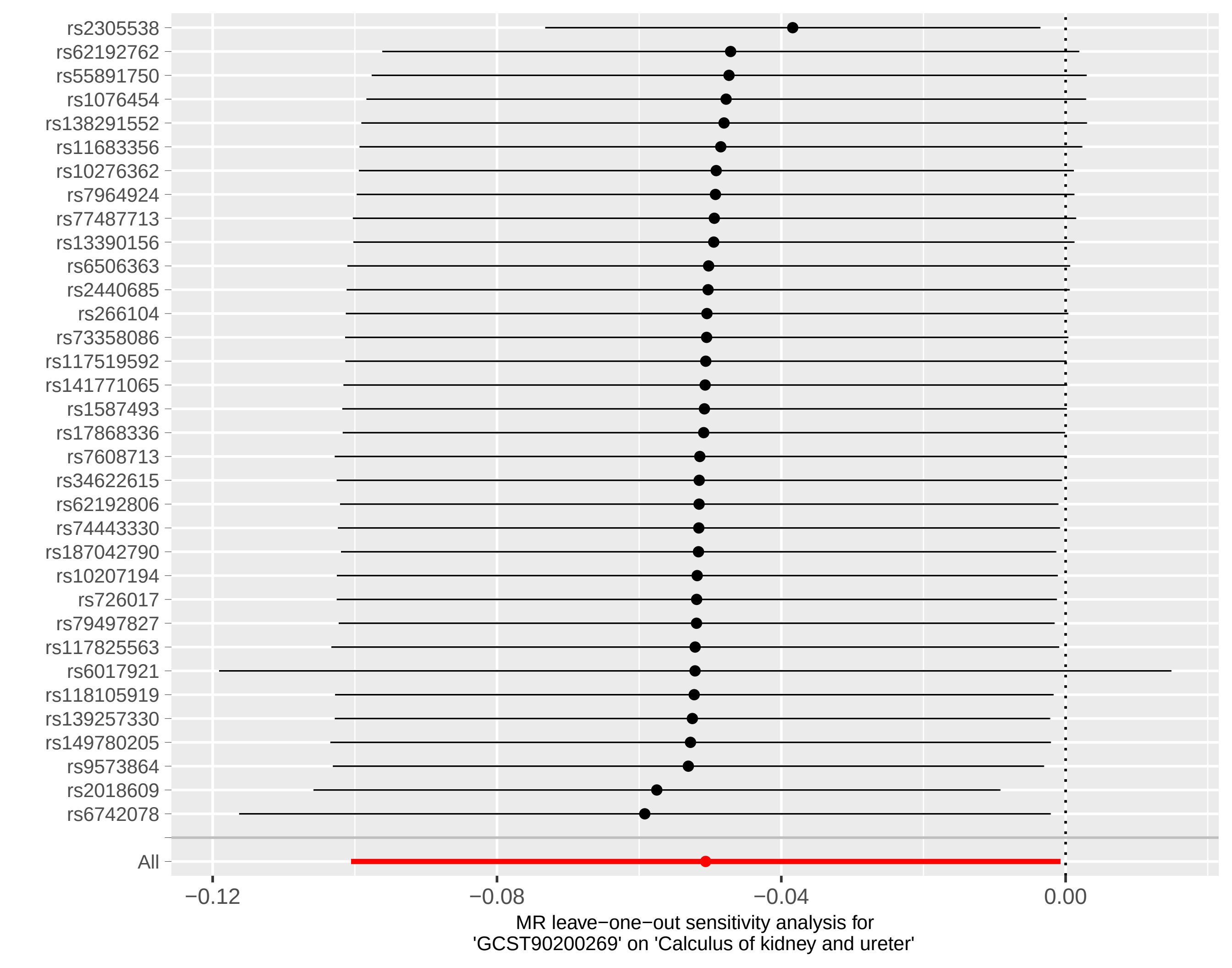
rs2305538
rs62192762
rs55891750
rs1076454
rs138291552
rs11683356
rs10276362
rs7964924
rs77487713
rs13390156
rs6506363
rs2440685
rs266104
rs73358086
rs117519592
rs141771065
rs1587493
rs17868336
rs7608713
rs34622615
rs62192806
rs74443330
rs187042790
rs10207194
rs726017
rs79497827
rs117825563
rs6017921
rs118105919
rs139257330
rs149780205
rs9573864
rs2018609
rs6742078
All
−0.12
−0.08
−0.04
0.00
MR leave−one−out sensitivity analysis for
'GCST90200269' on 'Calculus of kidney and ureter'

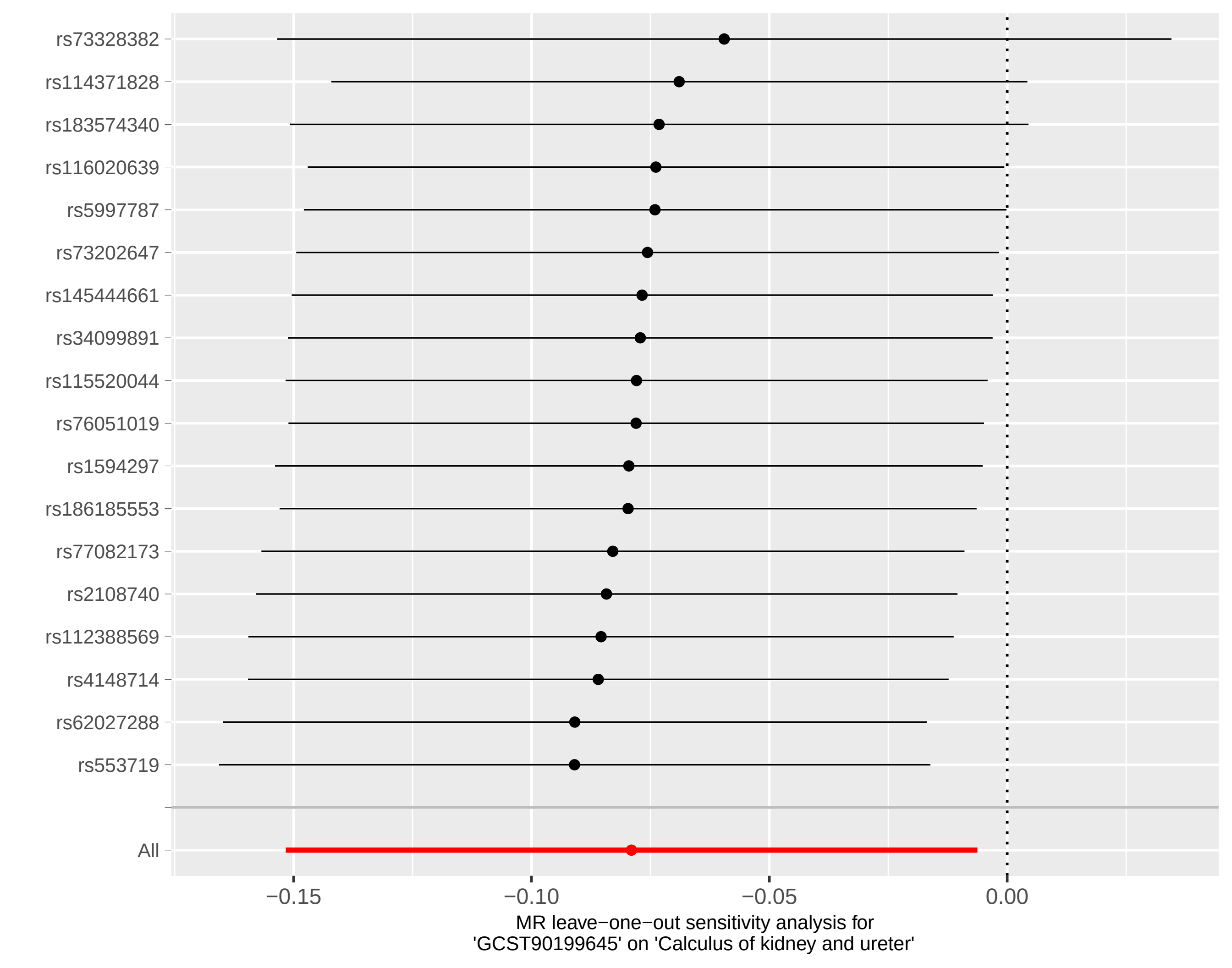

rs73328382
rs114371828
rs183574340
rs116020639
rs5997787
rs73202647
rs145444661
rs34099891
rs115520044
rs76051019
rs1594297
rs186185553
rs77082173
rs2108740
rs112388569
rs4148714
rs62027288
rs553719
All
−0.15
−0.10
−0.05
0.00
MR leave−one−out sensitivity analysis for
'GCST90199645' on 'Calculus of kidney and ureter'

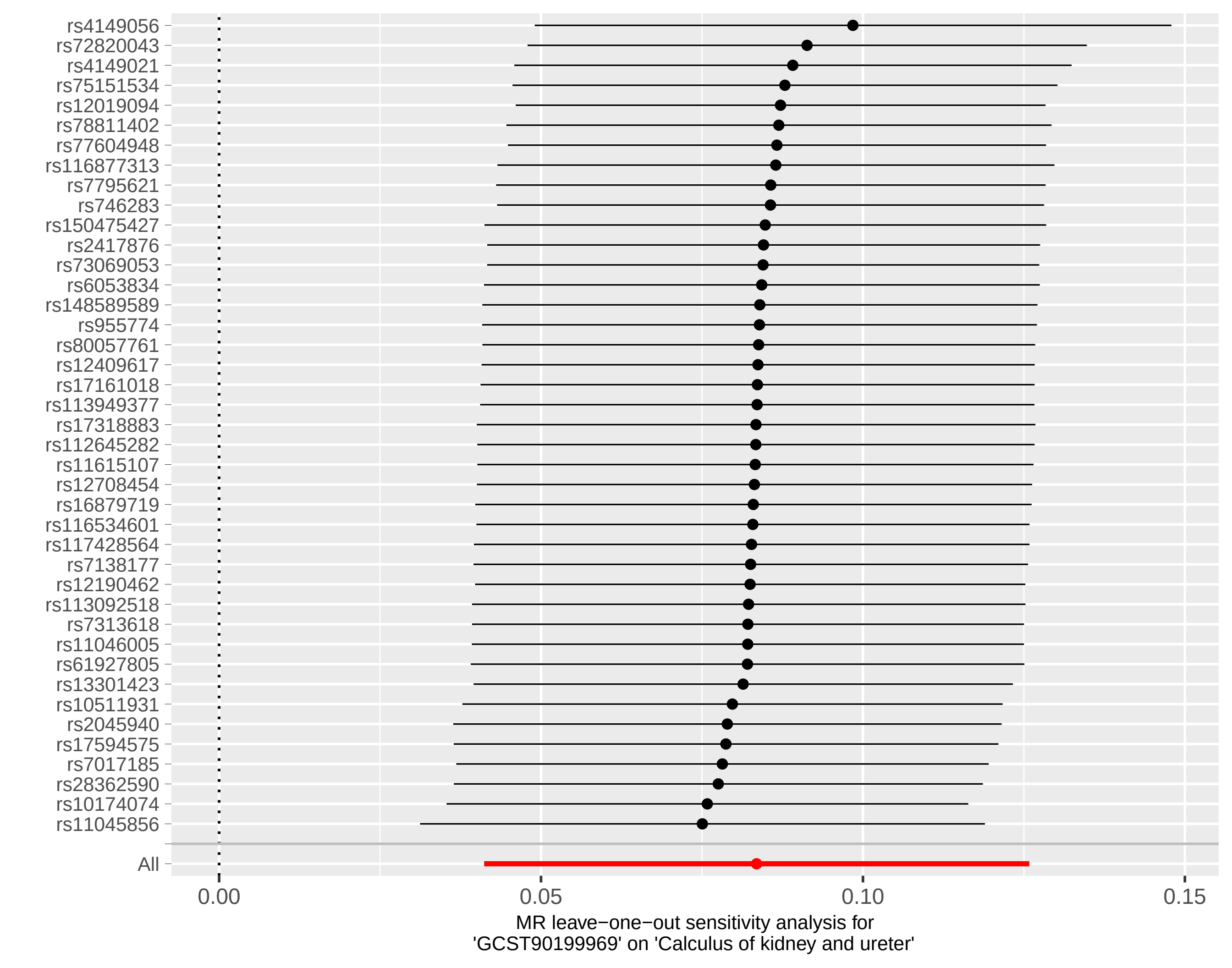

rs4149056
rs72820043
rs4149021
rs75151534
rs12019094
rs78811402
rs77604948
rs116877313
rs7795621
rs746283
rs150475427
rs2417876
rs73069053
rs6053834
rs148589589
rs955774
rs80057761
rs12409617
rs17161018
rs113949377
rs17318883
rs112645282
rs11615107
rs12708454
rs16879719
rs116534601
rs117428564
rs7138177
rs12190462
rs113092518
rs7313618
rs11046005
rs61927805
rs13301423
rs10511931
rs2045940
rs17594575
rs7017185
rs28362590
rs10174074
rs11045856
All
0.00
0.05
0.10
0.15
MR leave−one−out sensitivity analysis for
'GCST90199969' on 'Calculus of kidney and ureter'

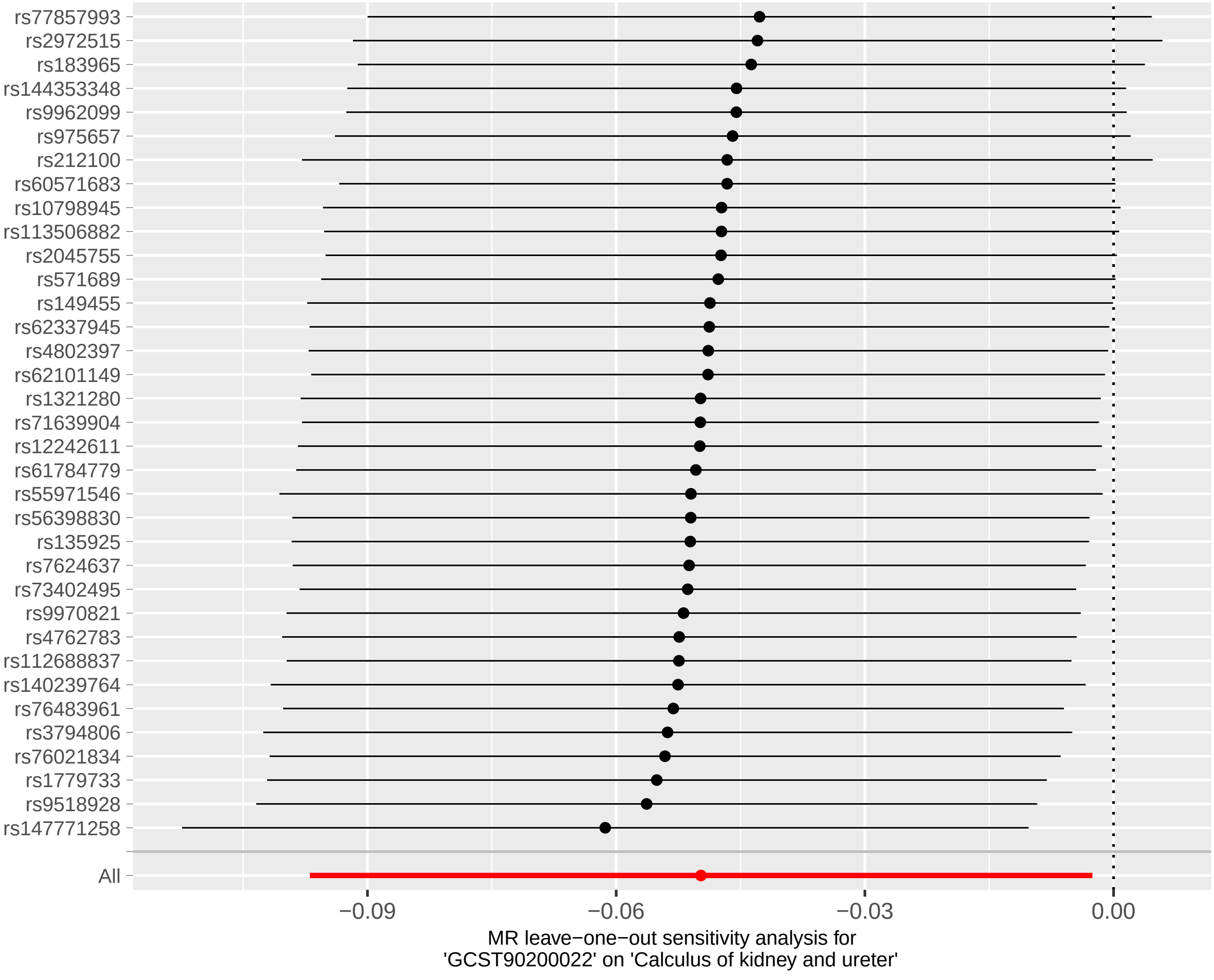
rs77857993
rs2972515
rs183965
rs144353348
rs9962099
rs975657
rs212100
rs60571683
rs10798945
rs113506882
rs2045755
rs571689
rs149455
rs62337945
rs4802397
rs62101149
rs1321280
rs71639904
rs12242611
rs61784779
rs55971546
rs56398830
rs135925
rs7624637
rs73402495
rs9970821
rs4762783
rs112688837
rs140239764
rs76483961
rs3794806
rs76021834
rs1779733
rs9518928
rs147771258
All
−0.09
−0.06
−0.03
0.00
MR leave−one−out sensitivity analysis for
'GCST90200022' on 'Calculus of kidney and ureter'

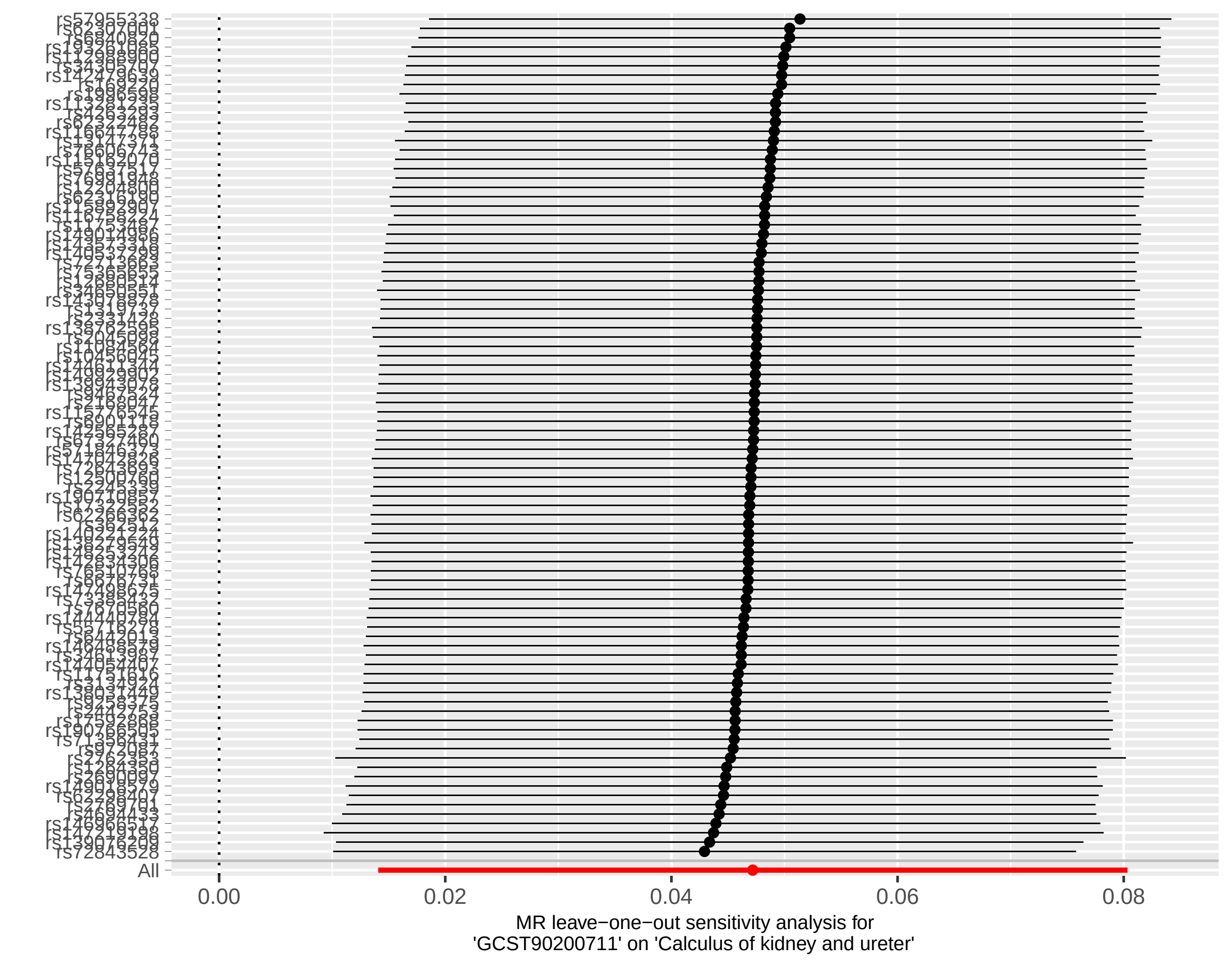
rs57955338
rs72843528
All
0.00
0.02
0.04
0.06
0.08
MR leave−one−out sensitivity analysis for
'GCST90200711' on 'Calculus of kidney and ureter'

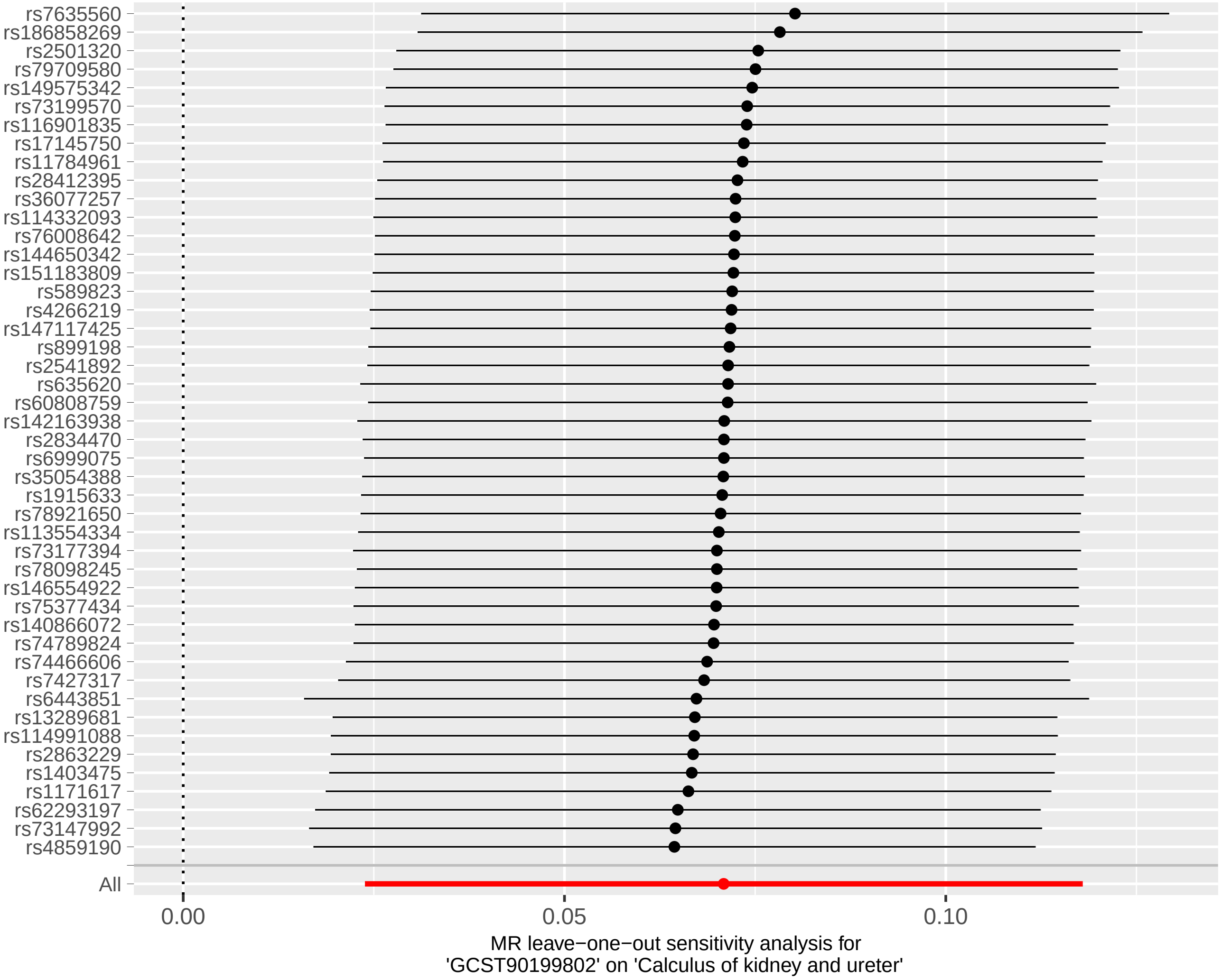
rs7635560
rs186858269
rs2501320
rs79709580
rs149575342
rs73199570
rs116901835
rs17145750
rs11784961
rs28412395
rs36077257
rs114332093
rs76008642
rs144650342
rs151183809
rs589823
rs4266219
rs147117425
rs899198
rs2541892
rs635620
rs60808759
rs142163938
rs2834470
rs6999075
rs35054388
rs1915633
rs78921650
rs113554334
rs73177394
rs78098245
rs146554922
rs75377434
rs140866072
rs74789824
rs74466606
rs7427317
rs6443851
rs13289681
rs114991088
rs2863229
rs1403475
rs1171617
rs62293197
rs73147992
rs4859190
All
0.00
0.05
0.10
MR leave−one−out sensitivity analysis for
'GCST90199802' on 'Calculus of kidney and ureter'

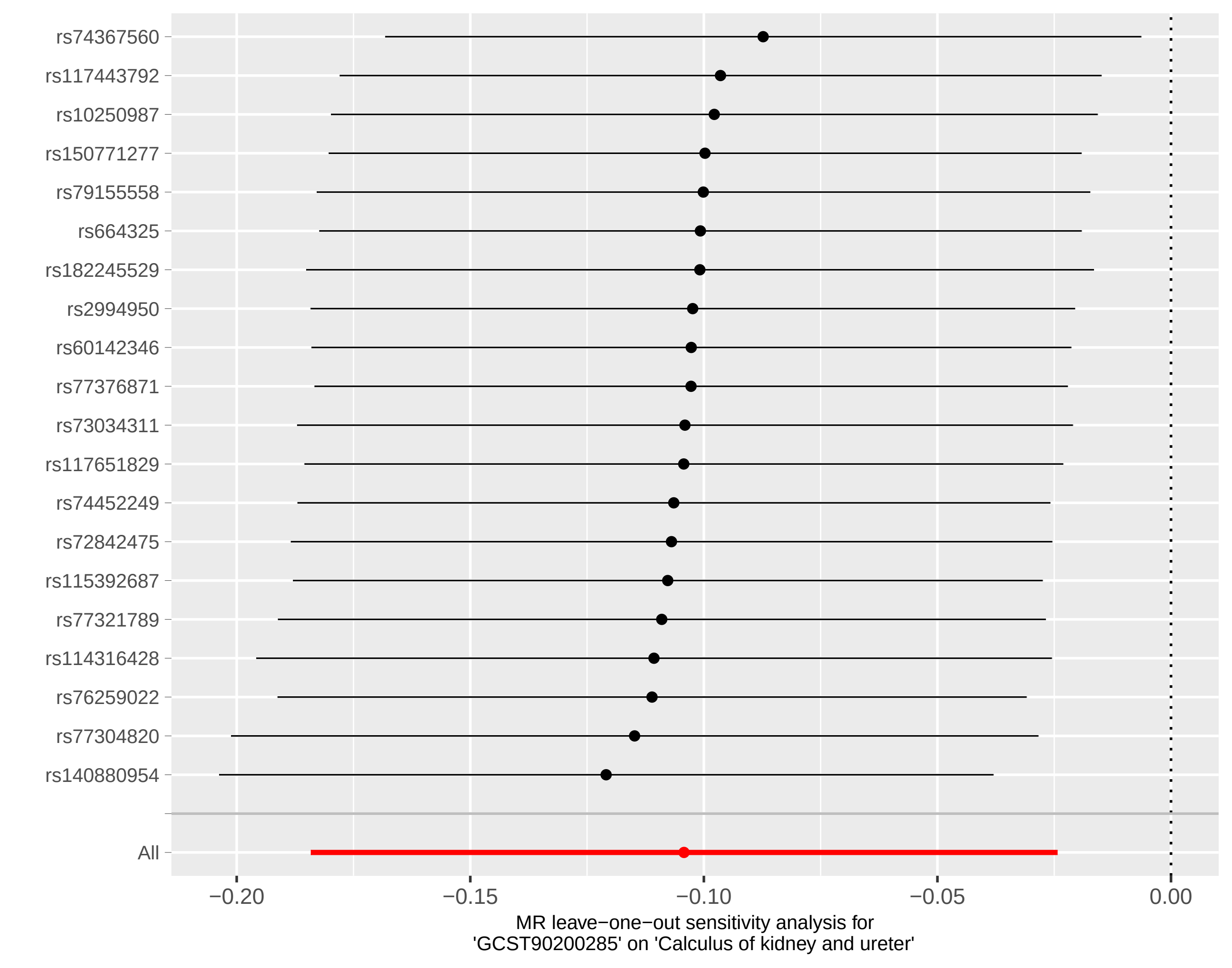

rs74367560
rs117443792
rs10250987
rs150771277
rs79155558
rs664325
rs182245529
rs2994950
rs60142346
rs77376871
rs73034311
rs117651829
rs74452249
rs72842475
rs115392687
rs77321789
rs114316428
rs76259022
rs77304820
rs140880954
All
−0.20
−0.15
−0.10
−0.05
0.00
MR leave−one−out sensitivity analysis for
'GCST90200285' on 'Calculus of kidney and ureter'

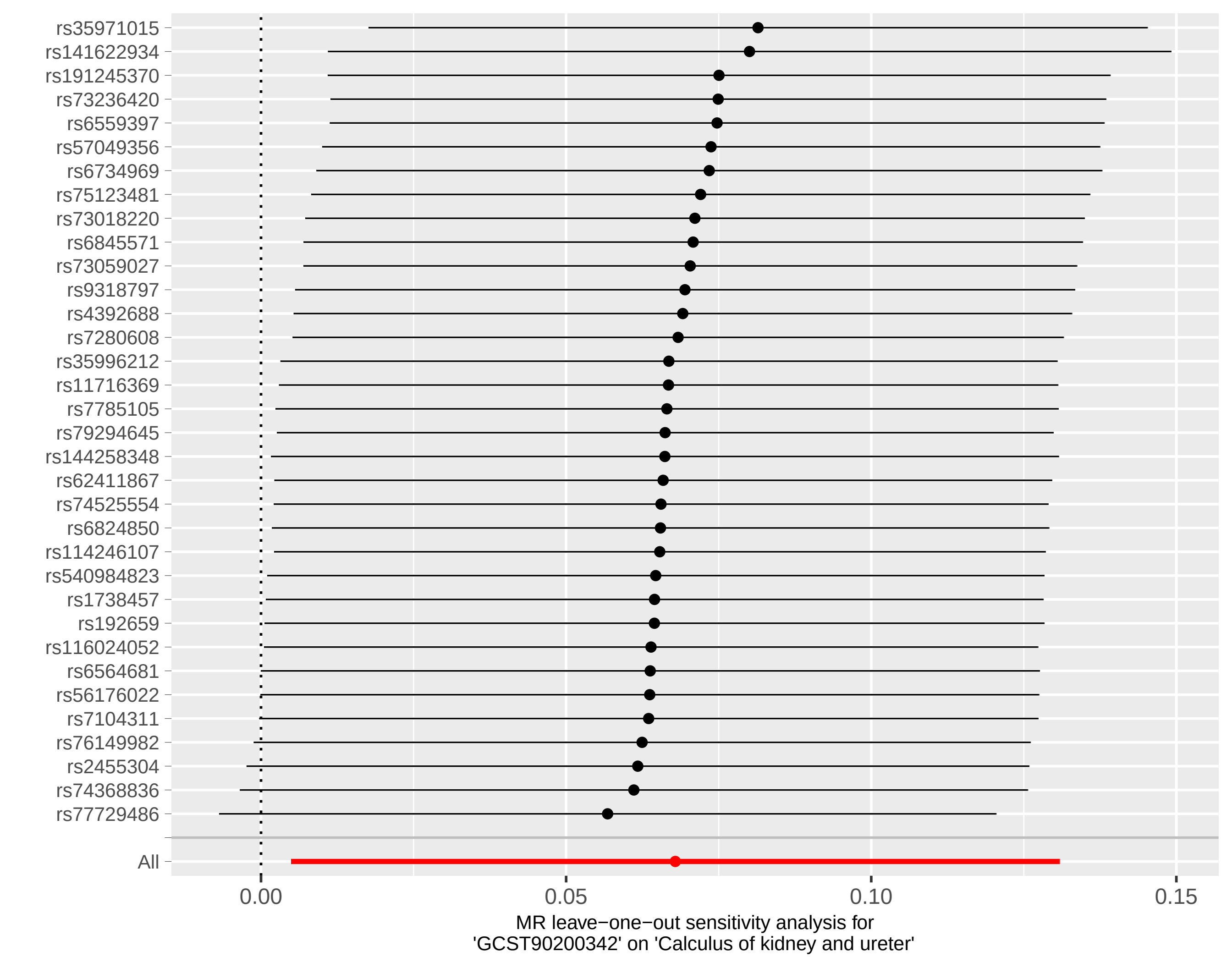

rs35971015
rs141622934
rs191245370
rs73236420
rs6559397
rs57049356
rs6734969
rs75123481
rs73018220
rs6845571
rs73059027
rs9318797
rs4392688
rs7280608
rs35996212
rs11716369
rs7785105
rs79294645
rs144258348
rs62411867
rs74525554
rs6824850
rs114246107
rs540984823
rs1738457
rs192659
rs116024052
rs6564681
rs56176022
rs7104311
rs76149982
rs2455304
rs74368836
rs77729486
All
0.00
0.05
0.10
0.15
MR leave−one−out sensitivity analysis for
'GCST90200342' on 'Calculus of kidney and ureter'

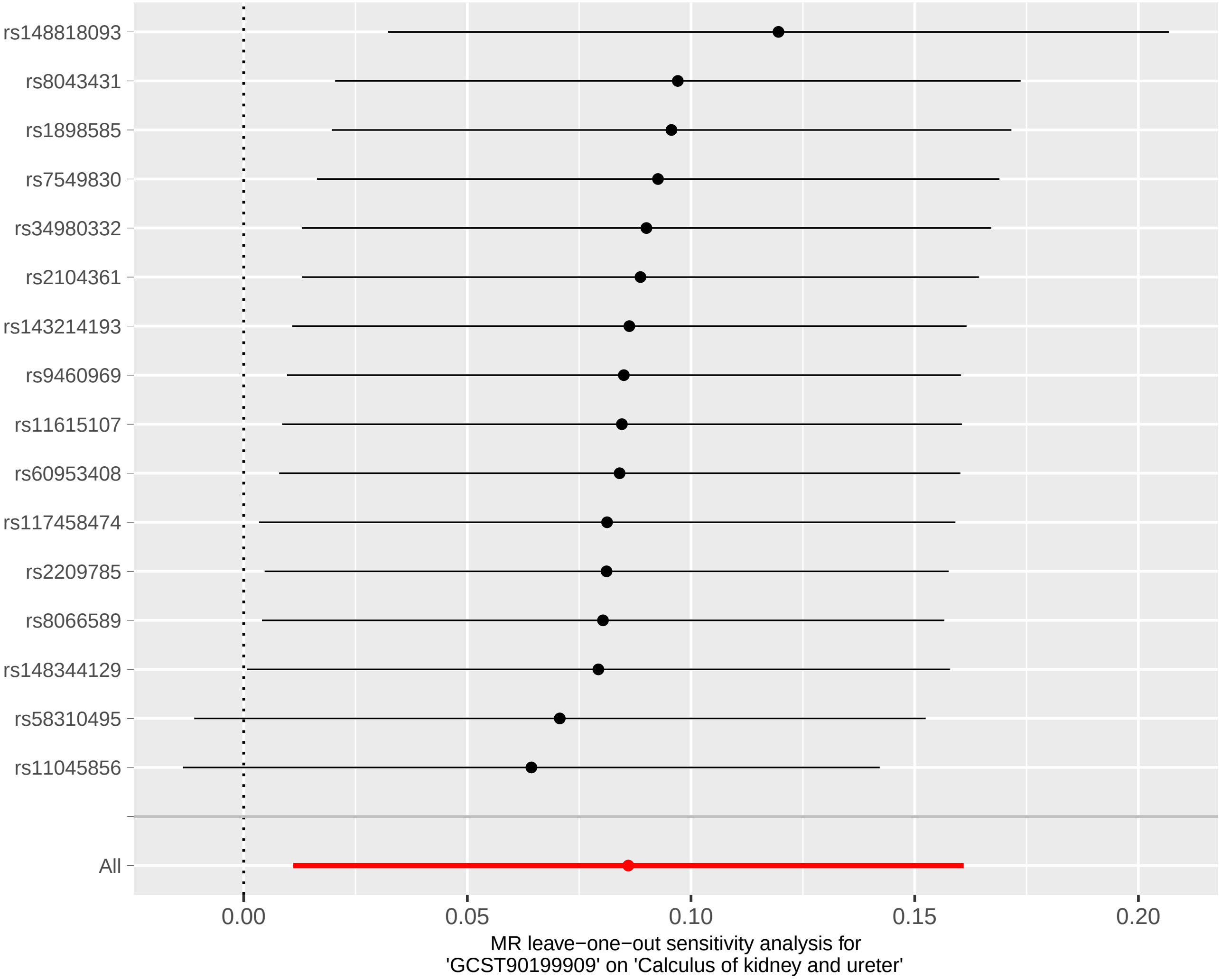
rs148818093
rs8043431
rs1898585
rs7549830
rs34980332
rs2104361
rs143214193
rs9460969
rs11615107
rs60953408
rs117458474
rs2209785
rs8066589
rs148344129
rs58310495
rs11045856
All
0.00
0.05
0.10
0.15
0.20
MR leave−one−out sensitivity analysis for
'GCST90199909' on 'Calculus of kidney and ureter'

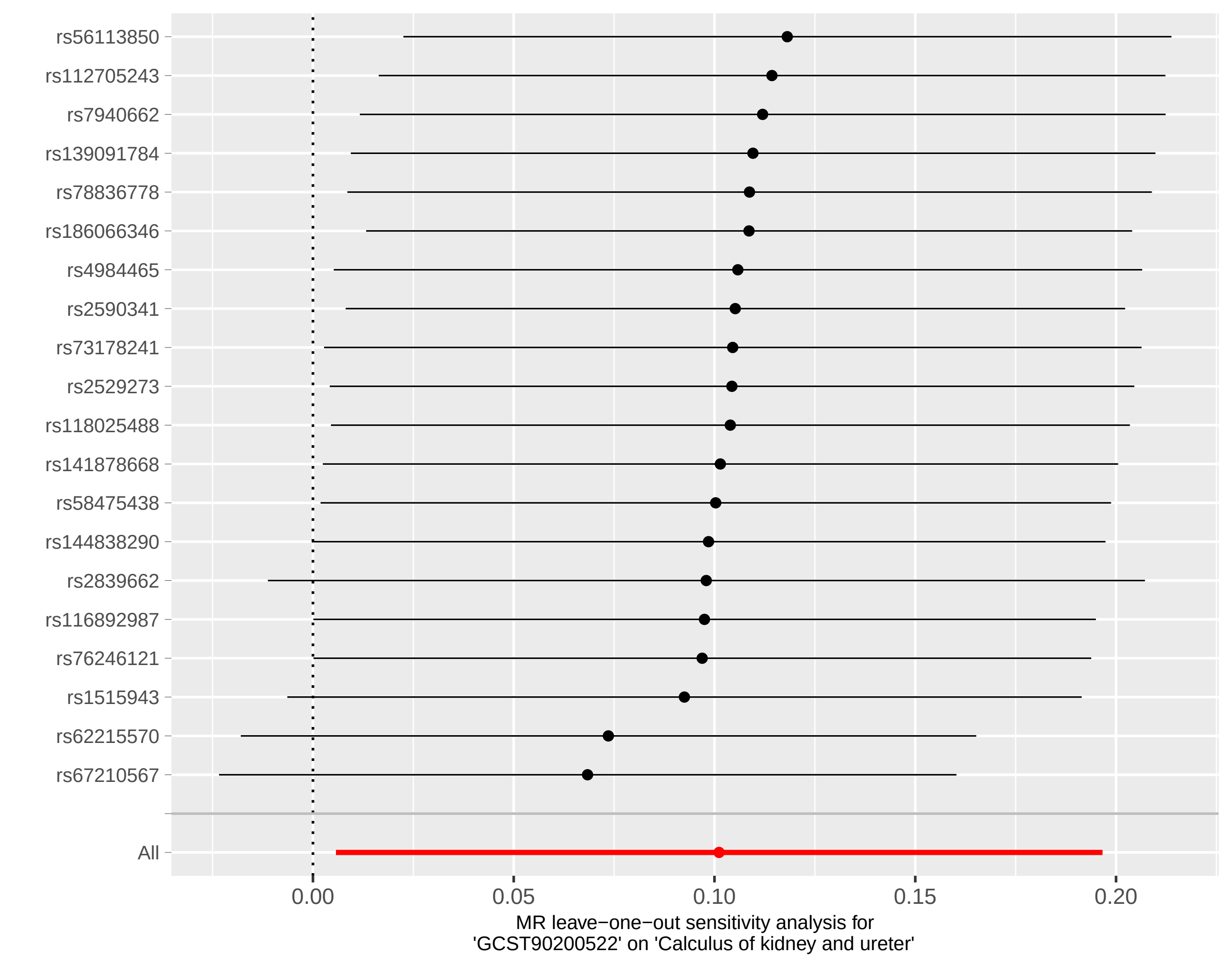
rs56113850
rs112705243
rs7940662
rs139091784
rs78836778
rs186066346
rs4984465
rs2590341
rs73178241
rs2529273
rs118025488
rs141878668
rs58475438
rs144838290
rs2839662
rs116892987
rs76246121
rs1515943
rs62215570
rs67210567
All
0.00
0.05
0.10
0.15
0.20
MR leave−one−out sensitivity analysis for
'GCST90200522' on 'Calculus of kidney and ureter'

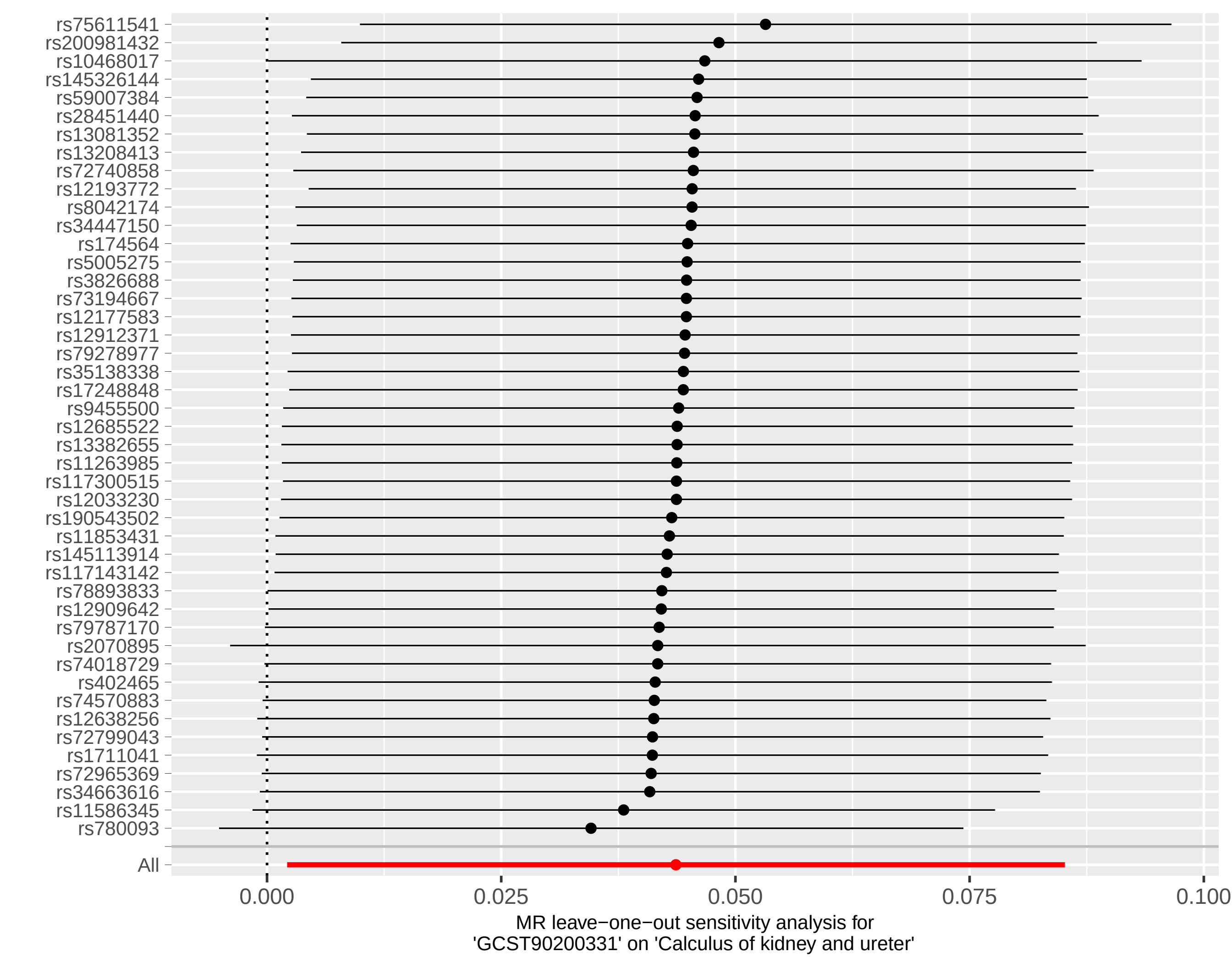
rs75611541
rs200981432
rs10468017
rs145326144
rs59007384
rs28451440
rs13081352
rs13208413
rs72740858
rs12193772
rs8042174
rs34447150
rs174564
rs5005275
rs3826688
rs73194667
rs12177583
rs12912371
rs79278977
rs35138338
rs17248848
rs9455500
rs12685522
rs13382655
rs11263985
rs117300515
rs12033230
rs190543502
rs11853431
rs145113914
rs117143142
rs78893833
rs12909642
rs79787170
rs2070895
rs74018729
rs402465
rs74570883
rs12638256
rs72799043
rs1711041
rs72965369
rs34663616
rs11586345
rs780093
All
0.000
0.025
0.050
0.075
0.100
MR leave−one−out sensitivity analysis for
'GCST90200331' on 'Calculus of kidney and ureter'

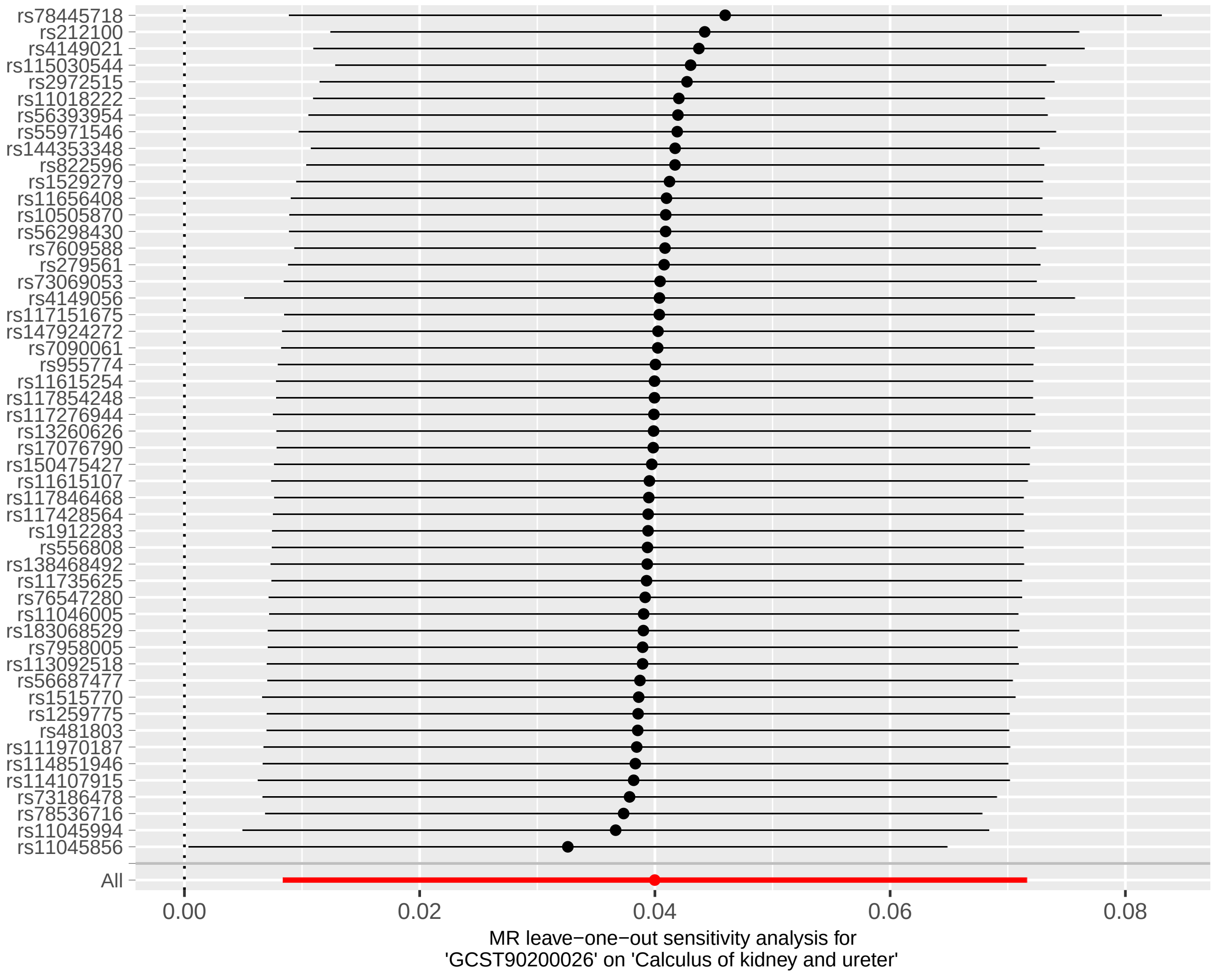
rs78445718
rs212100
rs4149021
rs115030544
rs2972515
rs11018222
rs56393954
rs55971546
rs144353348
rs822596
rs1529279
rs11656408
rs10505870
rs56298430
rs7609588
rs279561
rs73069053
rs4149056
rs117151675
rs147924272
rs7090061
rs955774
rs11615254
rs117854248
rs117276944
rs13260626
rs17076790
rs150475427
rs11615107
rs117846468
rs117428564
rs1912283
rs556808
rs138468492
rs11735625
rs76547280
rs11046005
rs183068529
rs7958005
rs113092518
rs56687477
rs1515770
rs1259775
rs481803
rs111970187
rs114851946
rs114107915
rs73186478
rs78536716
rs11045994
rs11045856
All
0.00
0.02
0.04
0.06
0.08
MR leave−one−out sensitivity analysis for
'GCST90200026' on 'Calculus of kidney and ureter'

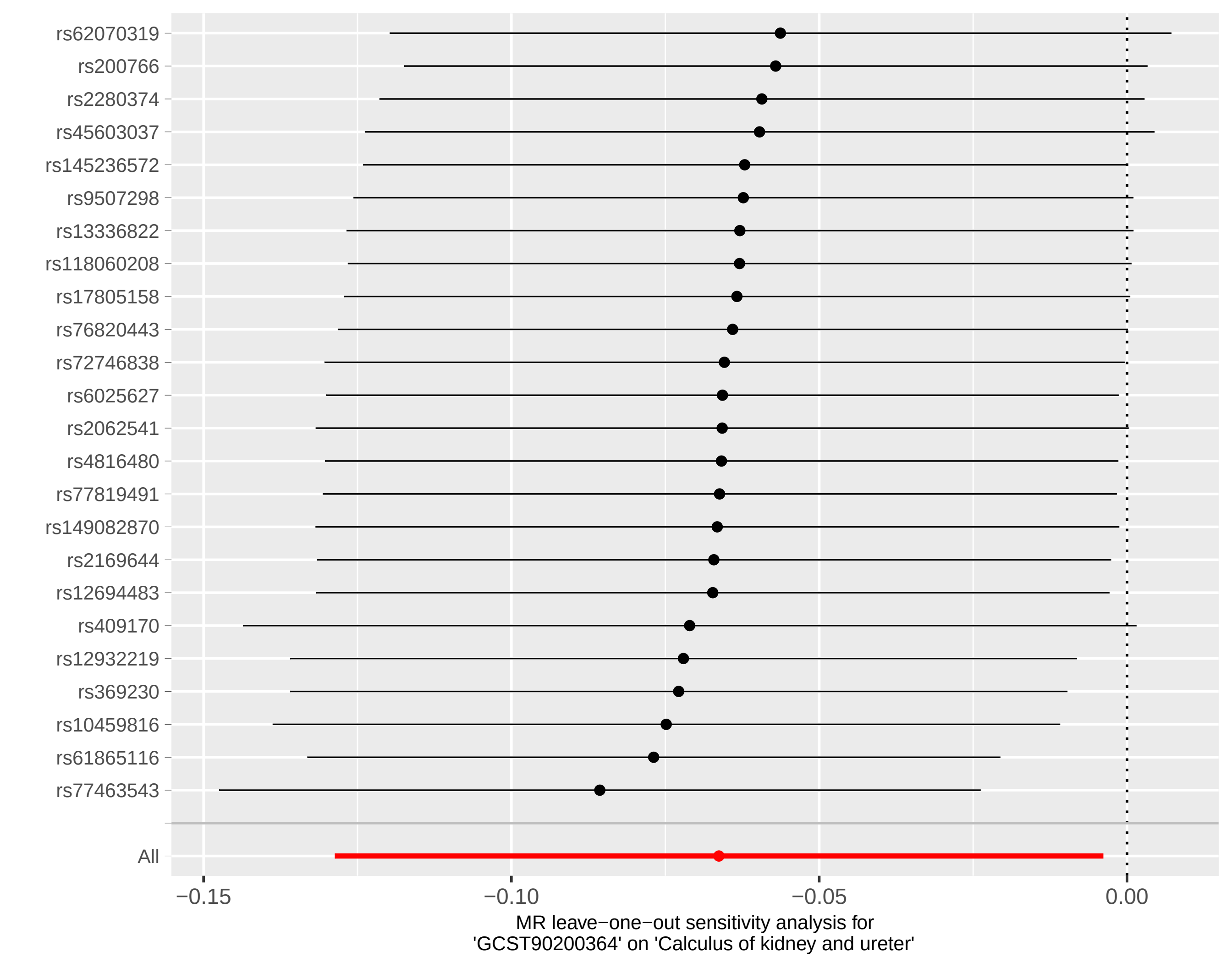

rs62070319
rs200766
rs2280374
rs45603037
rs145236572
rs9507298
rs13336822
rs118060208
rs17805158
rs76820443
rs72746838
rs6025627
rs2062541
rs4816480
rs77819491
rs149082870
rs2169644
rs12694483
rs409170
rs12932219
rs369230
rs10459816
rs61865116
rs77463543
All
−0.15
−0.10
−0.05
0.00
MR leave−one−out sensitivity analysis for
'GCST90200364' on 'Calculus of kidney and ureter'

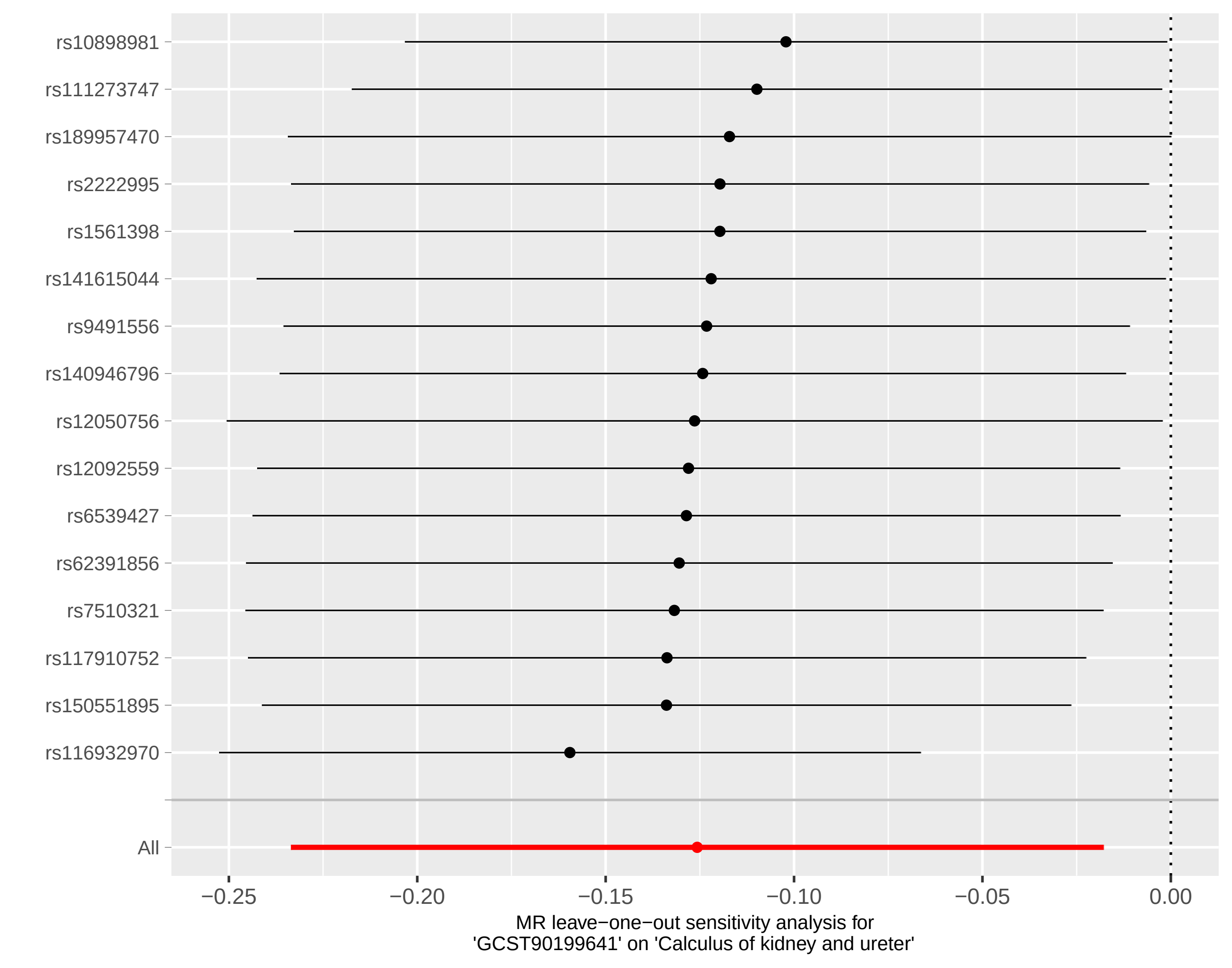
rs10898981
rs111273747
rs189957470
rs2222995
rs1561398
rs141615044
rs9491556
rs140946796
rs12050756
rs12092559
rs6539427
rs62391856
rs7510321
rs117910752
rs150551895
rs116932970
All
−0.25
−0.20
−0.15
−0.10
−0.05
0.00
MR leave−one−out sensitivity analysis for
'GCST90199641' on 'Calculus of kidney and ureter'

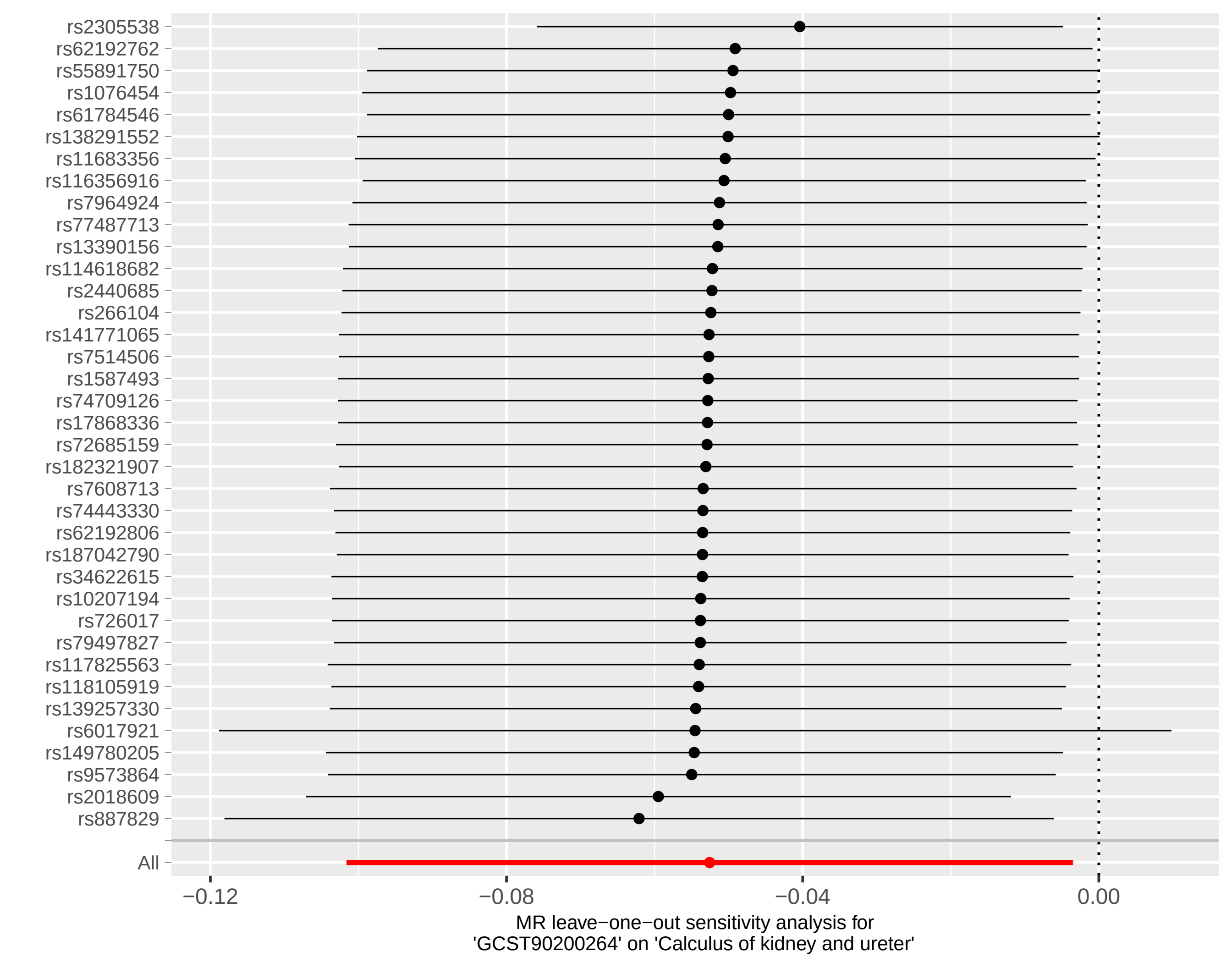
rs2305538
rs62192762
rs55891750
rs1076454
rs61784546
rs138291552
rs11683356
rs116356916
rs7964924
rs77487713
rs13390156
rs114618682
rs2440685
rs266104
rs141771065
rs7514506
rs1587493
rs74709126
rs17868336
rs72685159
rs182321907
rs7608713
rs74443330
rs62192806
rs187042790
rs34622615
rs10207194
rs726017
rs79497827
rs117825563
rs118105919
rs139257330
rs6017921
rs149780205
rs9573864
rs2018609
rs887829
All
−0.12
−0.08
−0.04
0.00
MR leave−one−out sensitivity analysis for
'GCST90200264' on 'Calculus of kidney and ureter'

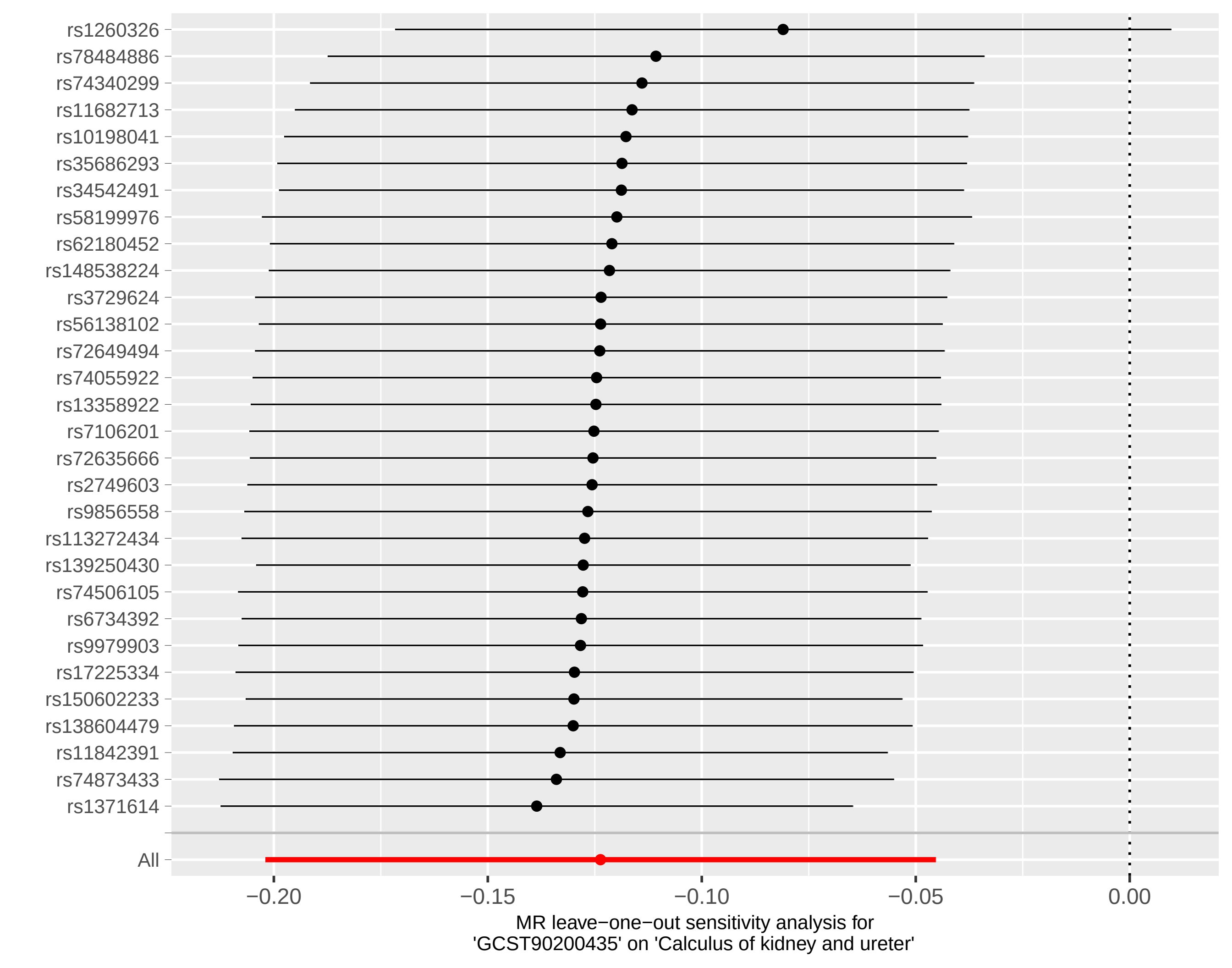
rs1260326
rs78484886
rs74340299
rs11682713
rs10198041
rs35686293
rs34542491
rs58199976
rs62180452
rs148538224
rs3729624
rs56138102
rs72649494
rs74055922
rs13358922
rs7106201
rs72635666
rs2749603
rs9856558
rs113272434
rs139250430
rs74506105
rs6734392
rs9979903
rs17225334
rs150602233
rs138604479
rs11842391
rs74873433
rs1371614
All
−0.20
−0.15
−0.10
−0.05
0.00
MR leave−one−out sensitivity analysis for
'GCST90200435' on 'Calculus of kidney and ureter'

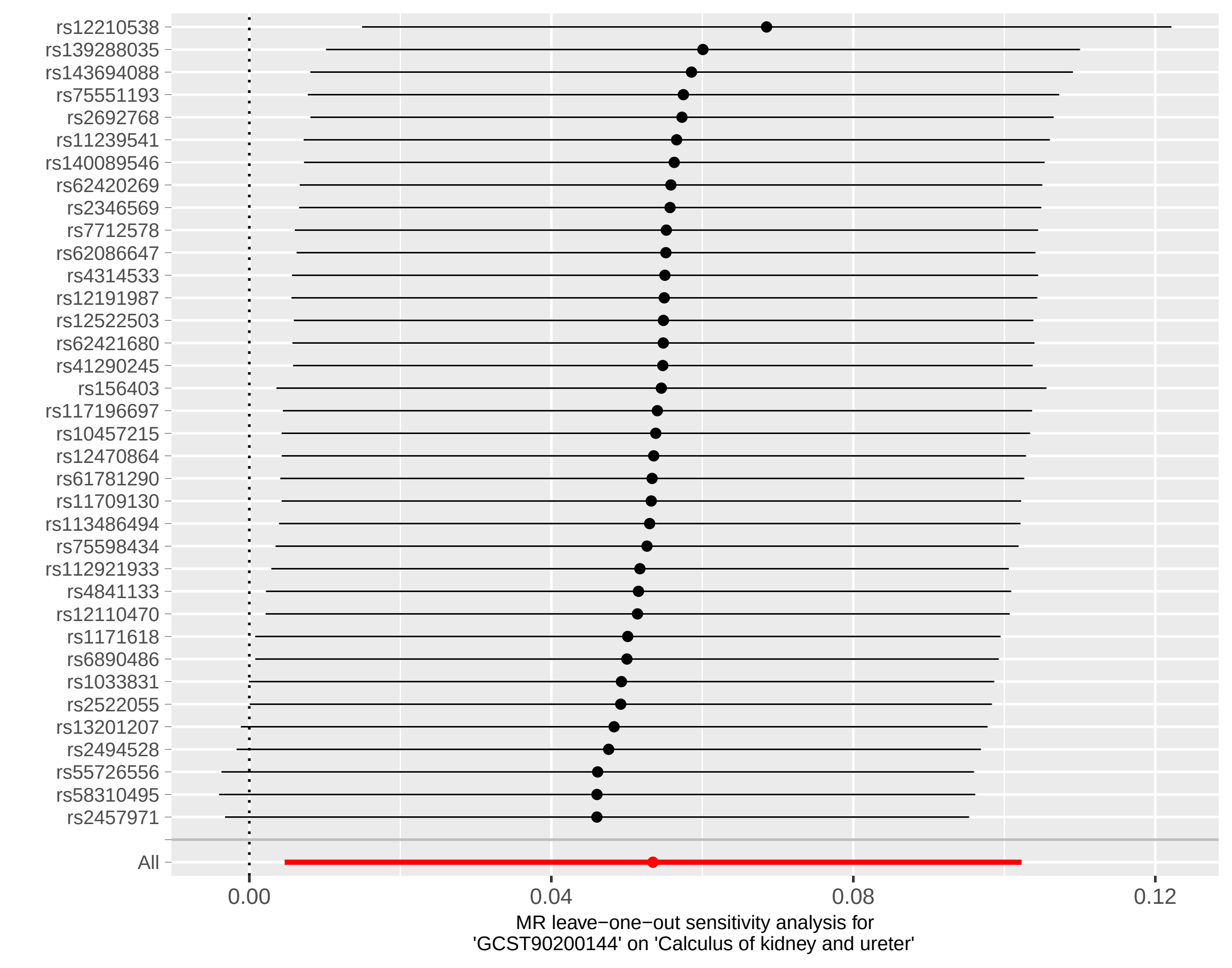
rs12210538
rs139288035
rs143694088
rs75551193
rs2692768
rs11239541
rs140089546
rs62420269
rs2346569
rs7712578
rs62086647
rs4314533
rs12191987
rs12522503
rs62421680
rs41290245
rs156403
rs117196697
rs10457215
rs12470864
rs61781290
rs11709130
rs113486494
rs75598434
rs112921933
rs4841133
rs12110470
rs1171618
rs6890486
rs1033831
rs2522055
rs13201207
rs2494528
rs55726556
rs58310495
rs2457971
All
0.00
0.04
0.08
0.12
MR leave−one−out sensitivity analysis for
'GCST90200144' on 'Calculus of kidney and ureter'

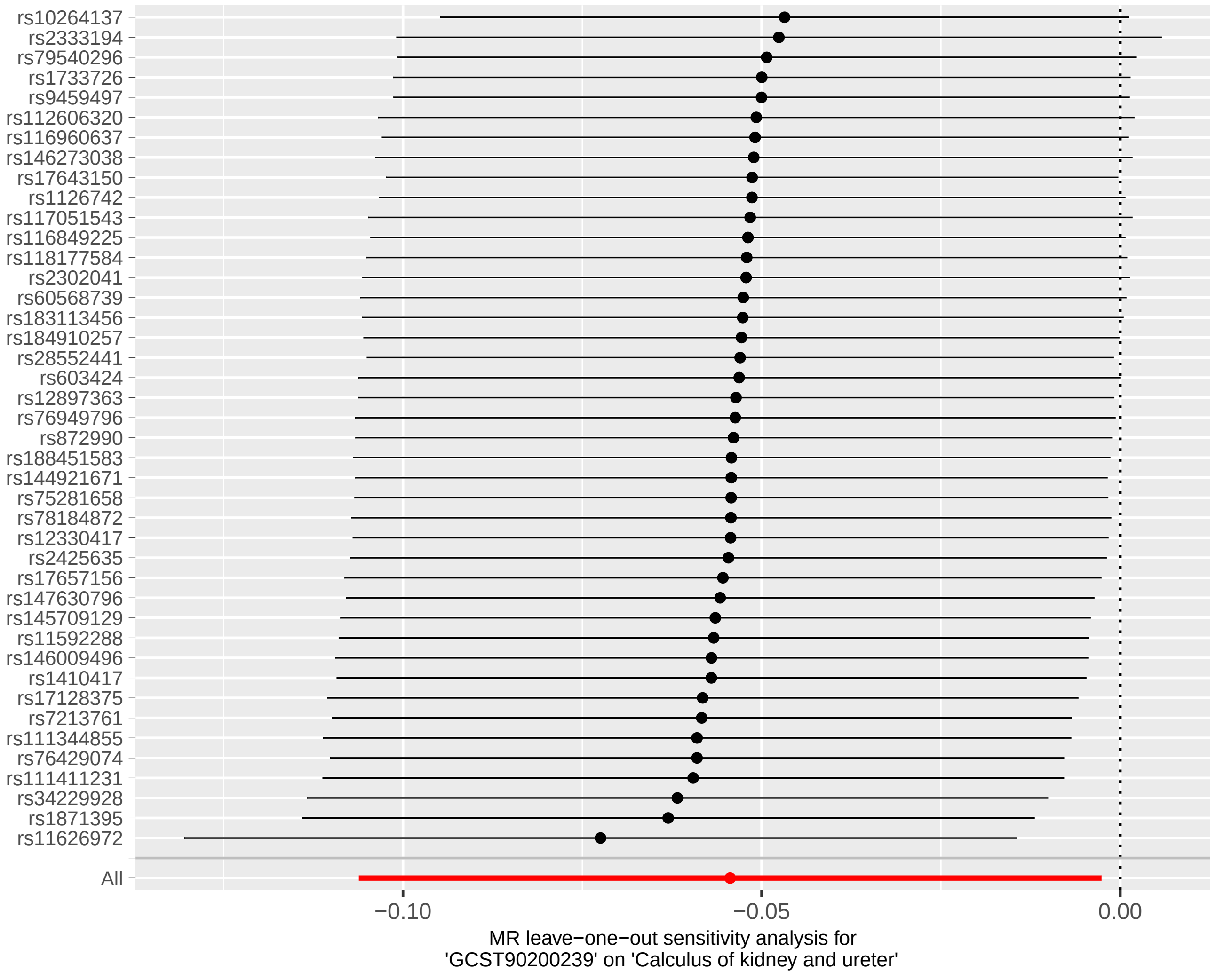
rs10264137
rs2333194
rs79540296
rs1733726
rs9459497
rs112606320
rs116960637
rs146273038
rs17643150
rs1126742
rs117051543
rs116849225
rs118177584
rs2302041
rs60568739
rs183113456
rs184910257
rs28552441
rs603424
rs12897363
rs76949796
rs872990
rs188451583
rs144921671
rs75281658
rs78184872
rs12330417
rs2425635
rs17657156
rs147630796
rs145709129
rs11592288
rs146009496
rs1410417
rs17128375
rs7213761
rs111344855
rs76429074
rs111411231
rs34229928
rs1871395
rs11626972
All
−0.10
−0.05
0.00
MR leave−one−out sensitivity analysis for
'GCST90200239' on 'Calculus of kidney and ureter'

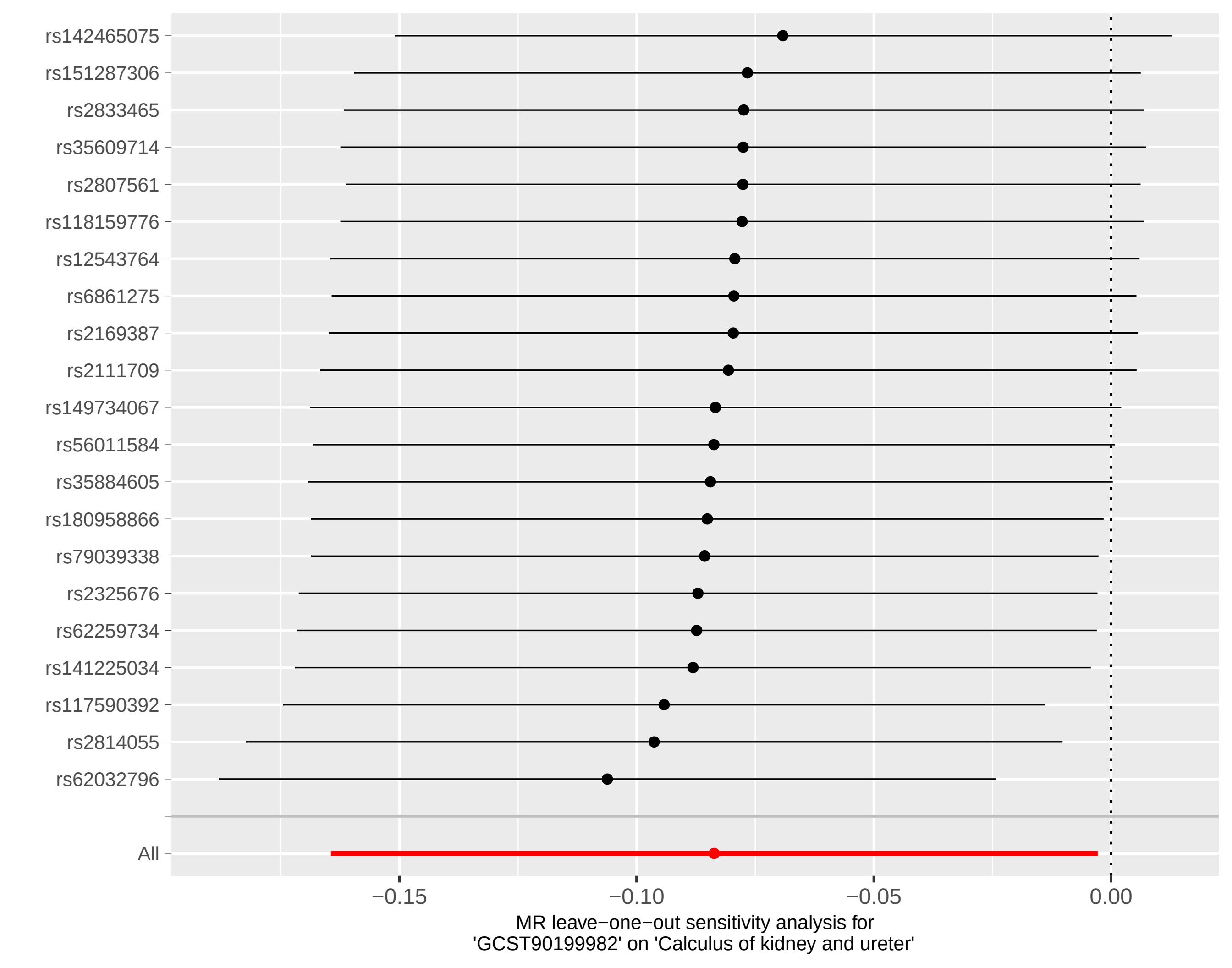
rs142465075
rs151287306
rs2833465
rs35609714
rs2807561
rs118159776
rs12543764
rs6861275
rs2169387
rs2111709
rs149734067
rs56011584
rs35884605
rs180958866
rs79039338
rs2325676
rs62259734
rs141225034
rs117590392
rs2814055
rs62032796
All
−0.15
−0.10
−0.05
0.00
MR leave−one−out sensitivity analysis for
'GCST90199982' on 'Calculus of kidney and ureter'

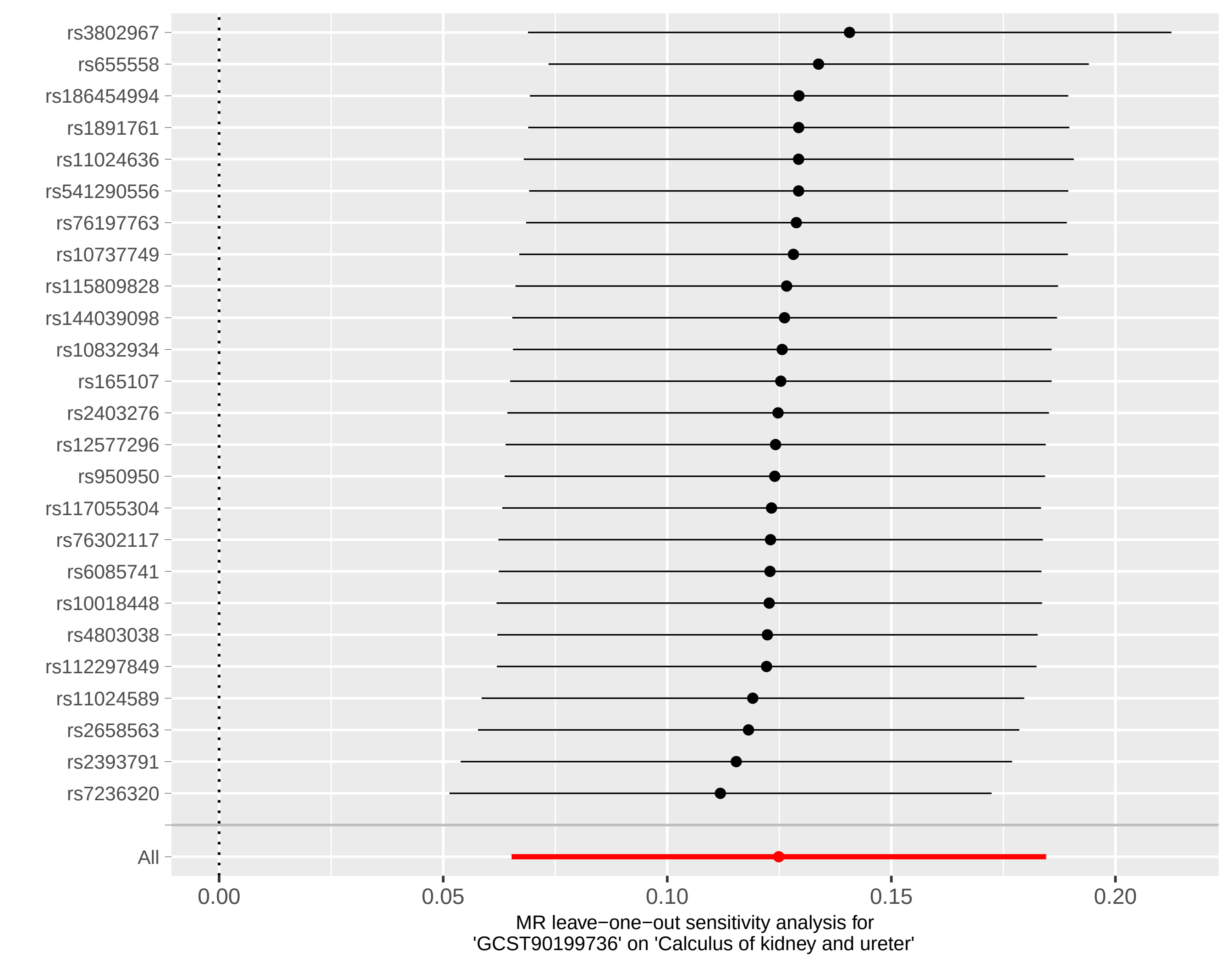
rs3802967
rs655558
rs186454994
rs1891761
rs11024636
rs541290556
rs76197763
rs10737749
rs115809828
rs144039098
rs10832934
rs165107
rs2403276
rs12577296
rs950950
rs117055304
rs76302117
rs6085741
rs10018448
rs4803038
rs112297849
rs11024589
rs2658563
rs2393791
rs7236320
All
0.00
0.05
0.10
0.15
0.20
MR leave−one−out sensitivity analysis for
'GCST90199736' on 'Calculus of kidney and ureter'

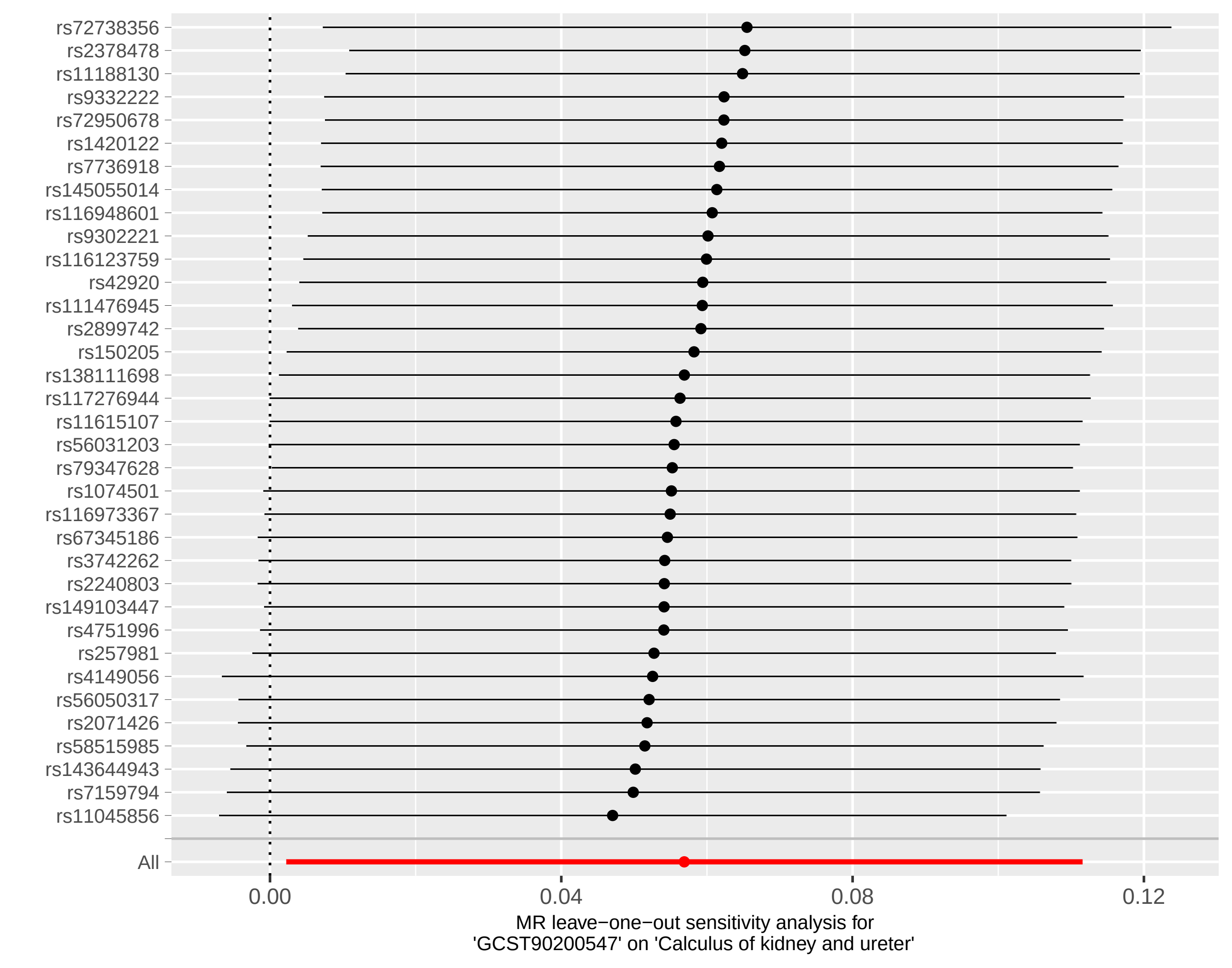

rs72738356
rs2378478
rs11188130
rs9332222
rs72950678
rs1420122
rs7736918
rs145055014
rs116948601
rs9302221
rs116123759
rs42920
rs111476945
rs2899742
rs150205
rs138111698
rs117276944
rs11615107
rs56031203
rs79347628
rs1074501
rs116973367
rs67345186
rs3742262
rs2240803
rs149103447
rs4751996
rs257981
rs4149056
rs56050317
rs2071426
rs58515985
rs143644943
rs7159794
rs11045856
All
0.00
0.04
0.08
0.12
MR leave−one−out sensitivity analysis for
'GCST90200547' on 'Calculus of kidney and ureter'

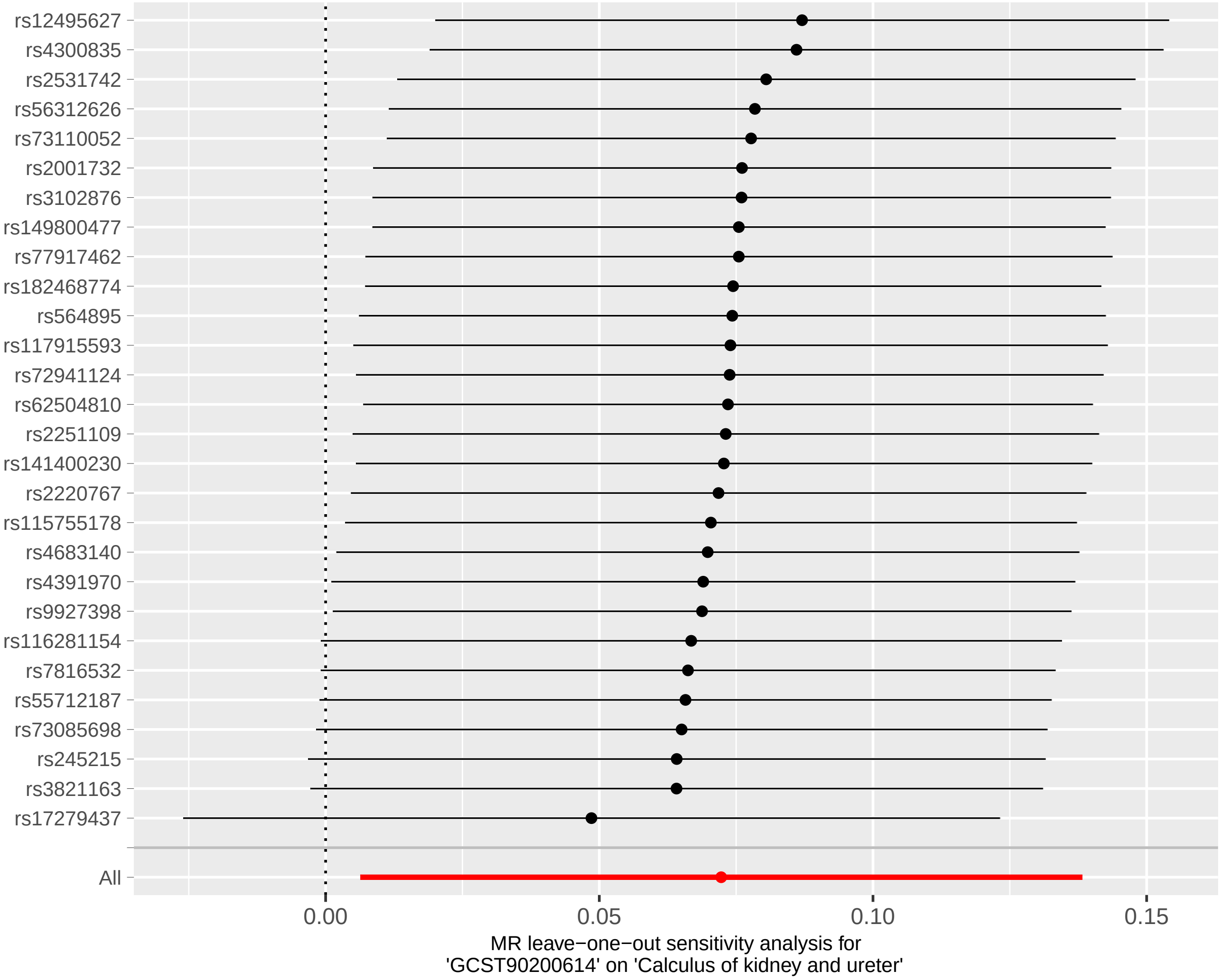
rs12495627
rs4300835
rs2531742
rs56312626
rs73110052
rs2001732
rs3102876
rs149800477
rs77917462
rs182468774
rs564895
rs117915593
rs72941124
rs62504810
rs2251109
rs141400230
rs2220767
rs115755178
rs4683140
rs4391970
rs9927398
rs116281154
rs7816532
rs55712187
rs73085698
rs245215
rs3821163
rs17279437
All
0.00
0.05
0.10
0.15
MR leave−one−out sensitivity analysis for
'GCST90200614' on 'Calculus of kidney and ureter'

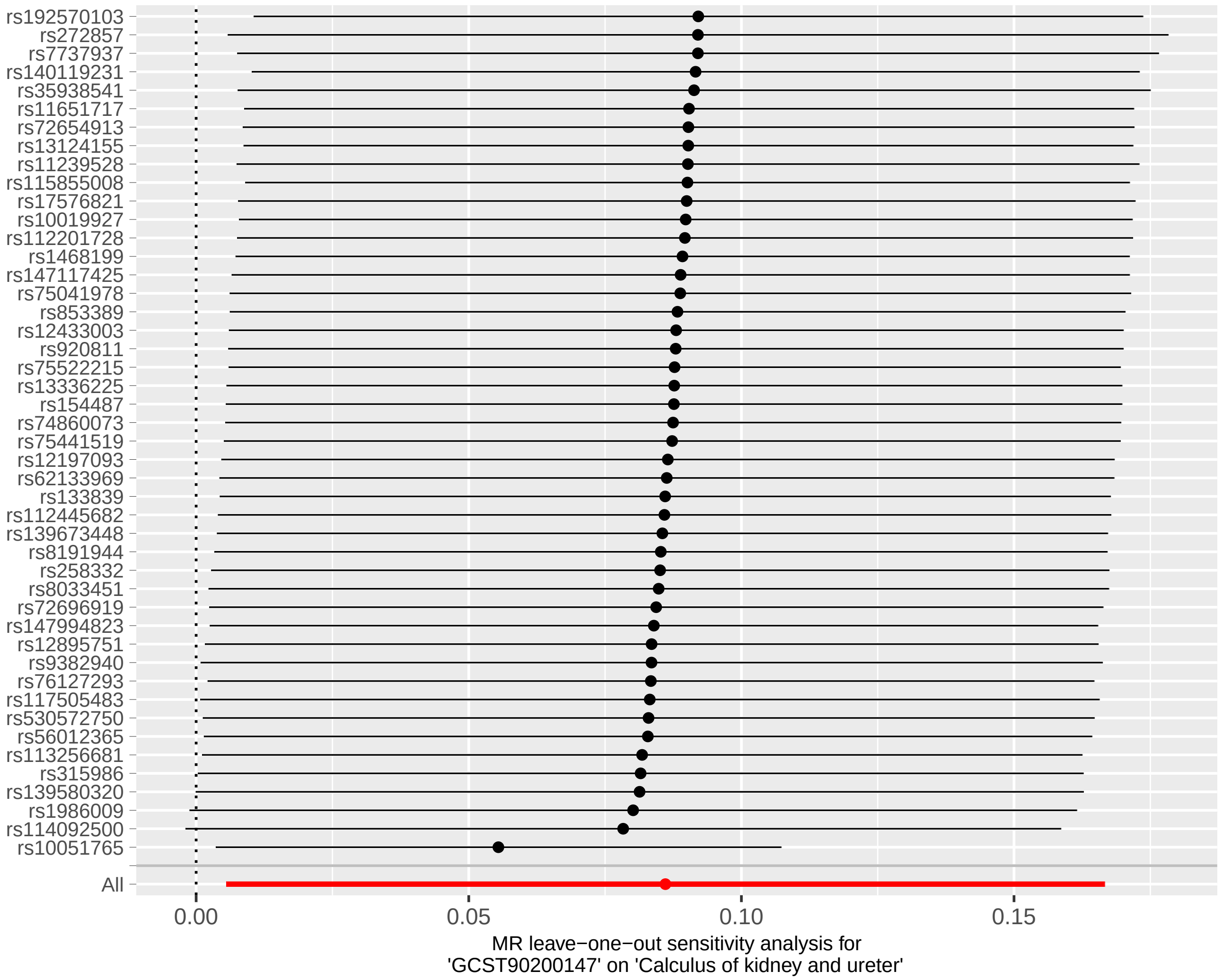
rs192570103
rs272857
rs7737937
rs140119231
rs35938541
rs11651717
rs72654913
rs13124155
rs11239528
rs115855008
rs17576821
rs10019927
rs112201728
rs1468199
rs147117425
rs75041978
rs853389
rs12433003
rs920811
rs75522215
rs13336225
rs154487
rs74860073
rs75441519
rs12197093
rs62133969
rs133839
rs112445682
rs139673448
rs8191944
rs258332
rs8033451
rs72696919
rs147994823
rs12895751
rs9382940
rs76127293
rs117505483
rs530572750
rs56012365
rs113256681
rs315986
rs139580320
rs1986009
rs114092500
rs10051765
All
0.00
0.05
0.10
0.15
MR leave−one−out sensitivity analysis for
'GCST90200147' on 'Calculus of kidney and ureter'

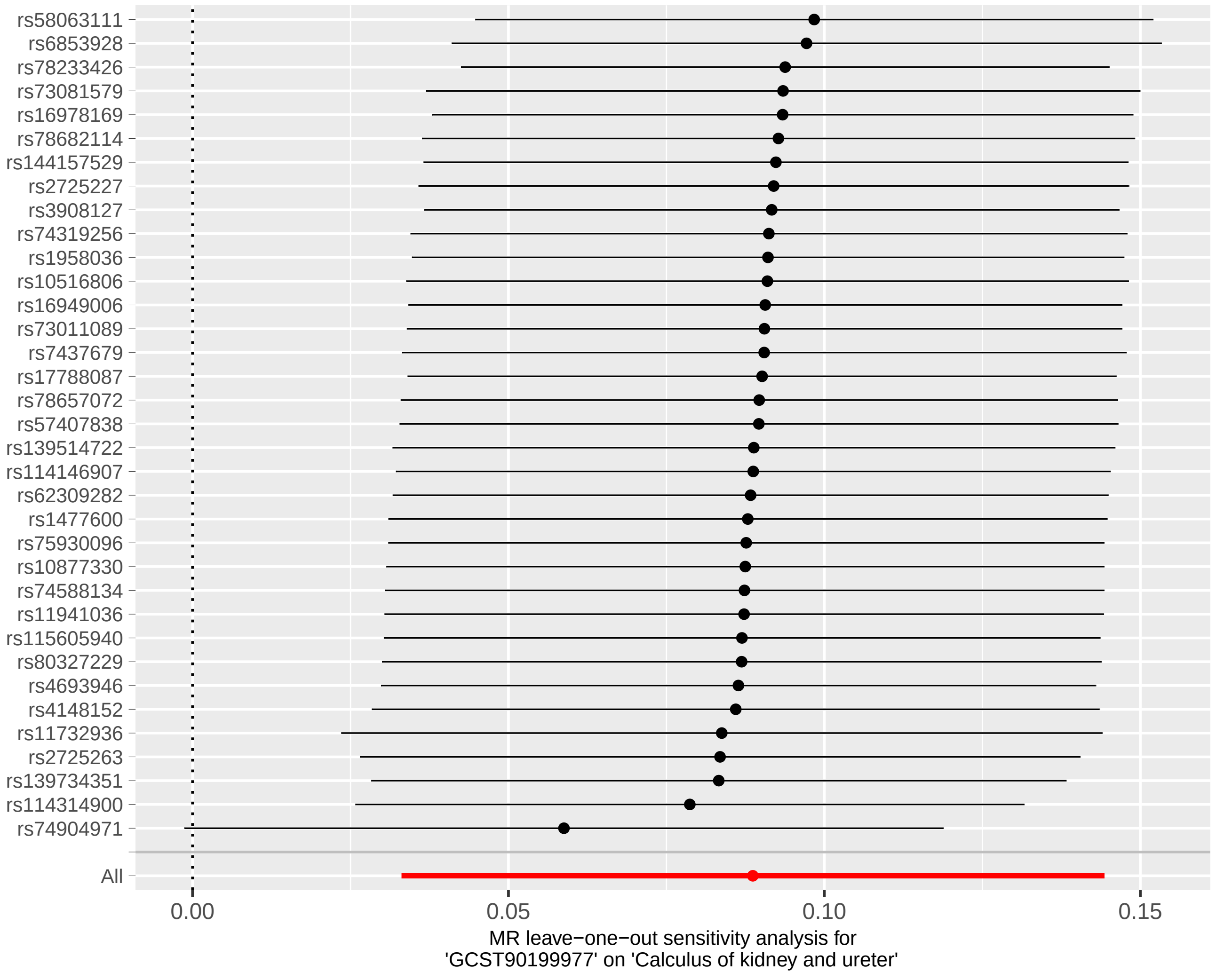
rs58063111
rs6853928
rs78233426
rs73081579
rs16978169
rs78682114
rs144157529
rs2725227
rs3908127
rs74319256
rs1958036
rs10516806
rs16949006
rs73011089
rs7437679
rs17788087
rs78657072
rs57407838
rs139514722
rs114146907
rs62309282
rs1477600
rs75930096
rs10877330
rs74588134
rs11941036
rs115605940
rs80327229
rs4693946
rs4148152
rs11732936
rs2725263
rs139734351
rs114314900
rs74904971
All
0.00
0.05
0.10
0.15
MR leave−one−out sensitivity analysis for
'GCST90199977' on 'Calculus of kidney and ureter'

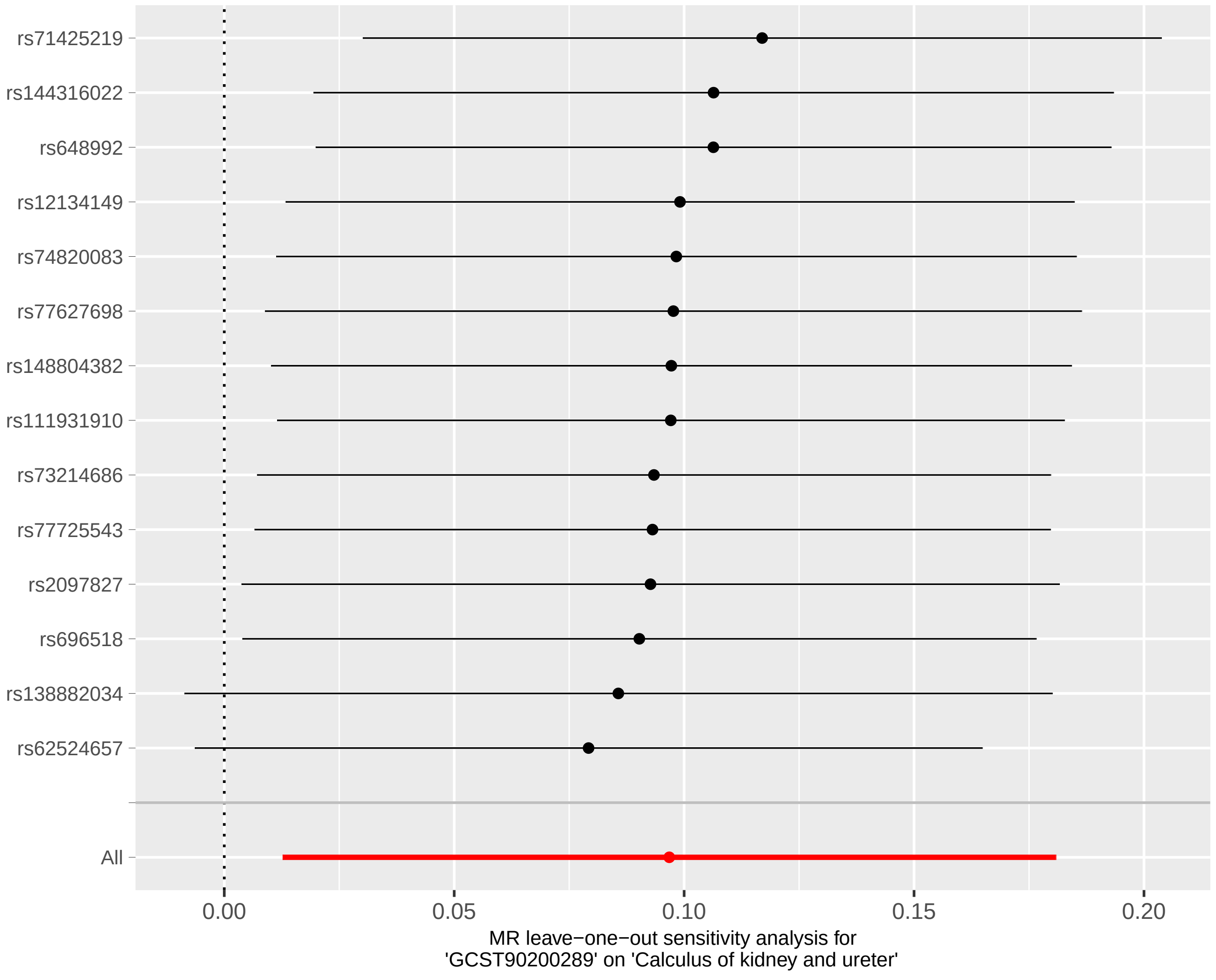
rs71425219
rs144316022
rs648992
rs12134149
rs74820083
rs77627698
rs148804382
rs111931910
rs73214686
rs77725543
rs2097827
rs696518
rs138882034
rs62524657
All
0.00
0.05
0.10
0.15
0.20
MR leave−one−out sensitivity analysis for
'GCST90200289' on 'Calculus of kidney and ureter'

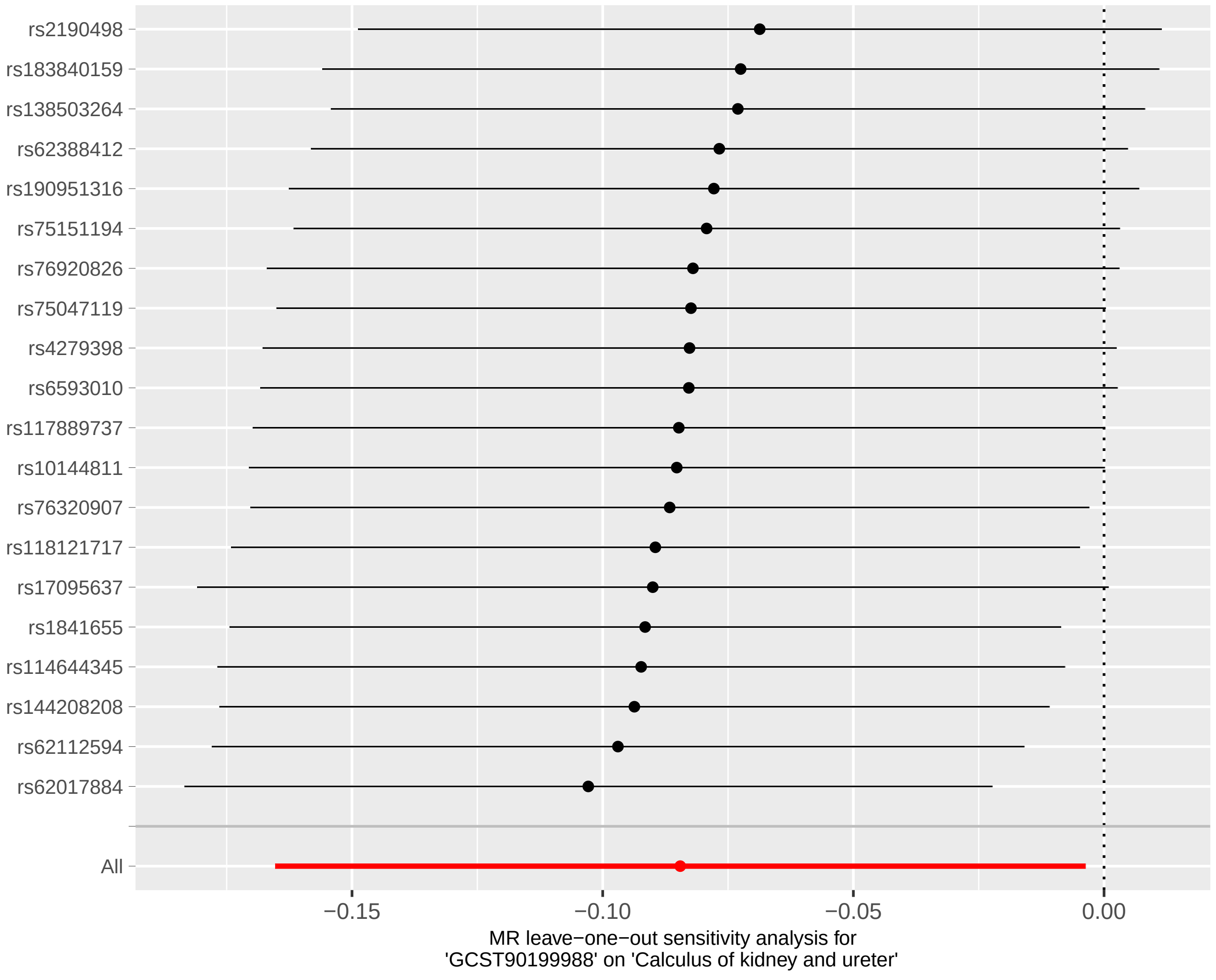
rs2190498
rs183840159
rs138503264
rs62388412
rs190951316
rs75151194
rs76920826
rs75047119
rs4279398
rs6593010
rs117889737
rs10144811
rs76320907
rs118121717
rs17095637
rs1841655
rs114644345
rs144208208
rs62112594
rs62017884
All
−0.15
−0.10
−0.05
0.00
MR leave−one−out sensitivity analysis for
'GCST90199988' on 'Calculus of kidney and ureter'

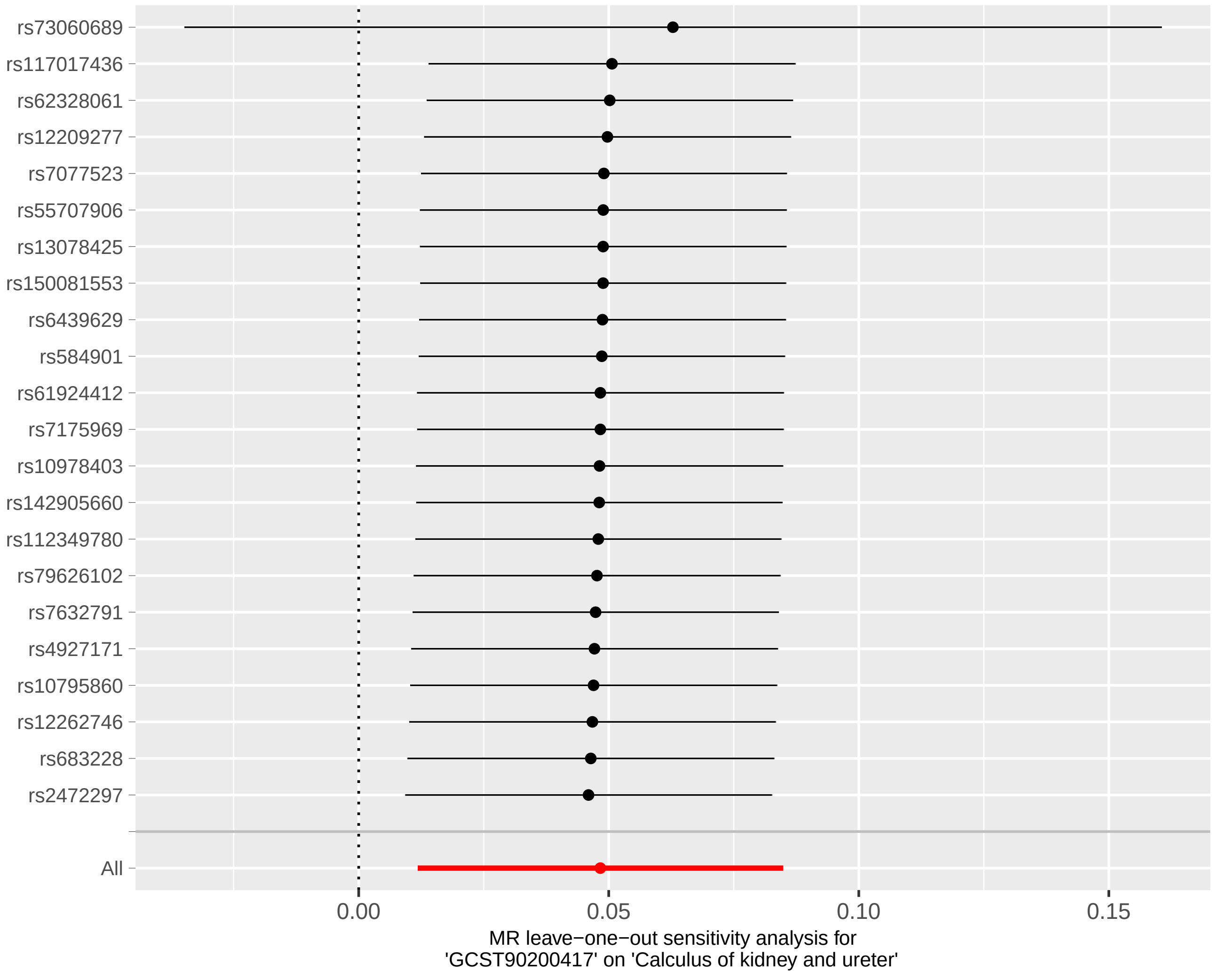
rs73060689
rs117017436
rs62328061
rs12209277
rs7077523
rs55707906
rs13078425
rs150081553
rs6439629
rs584901
rs61924412
rs7175969
rs10978403
rs142905660
rs112349780
rs79626102
rs7632791
rs4927171
rs10795860
rs12262746
rs683228
rs2472297
All
0.00
0.05
0.10
0.15
MR leave−one−out sensitivity analysis for
'GCST90200417' on 'Calculus of kidney and ureter'

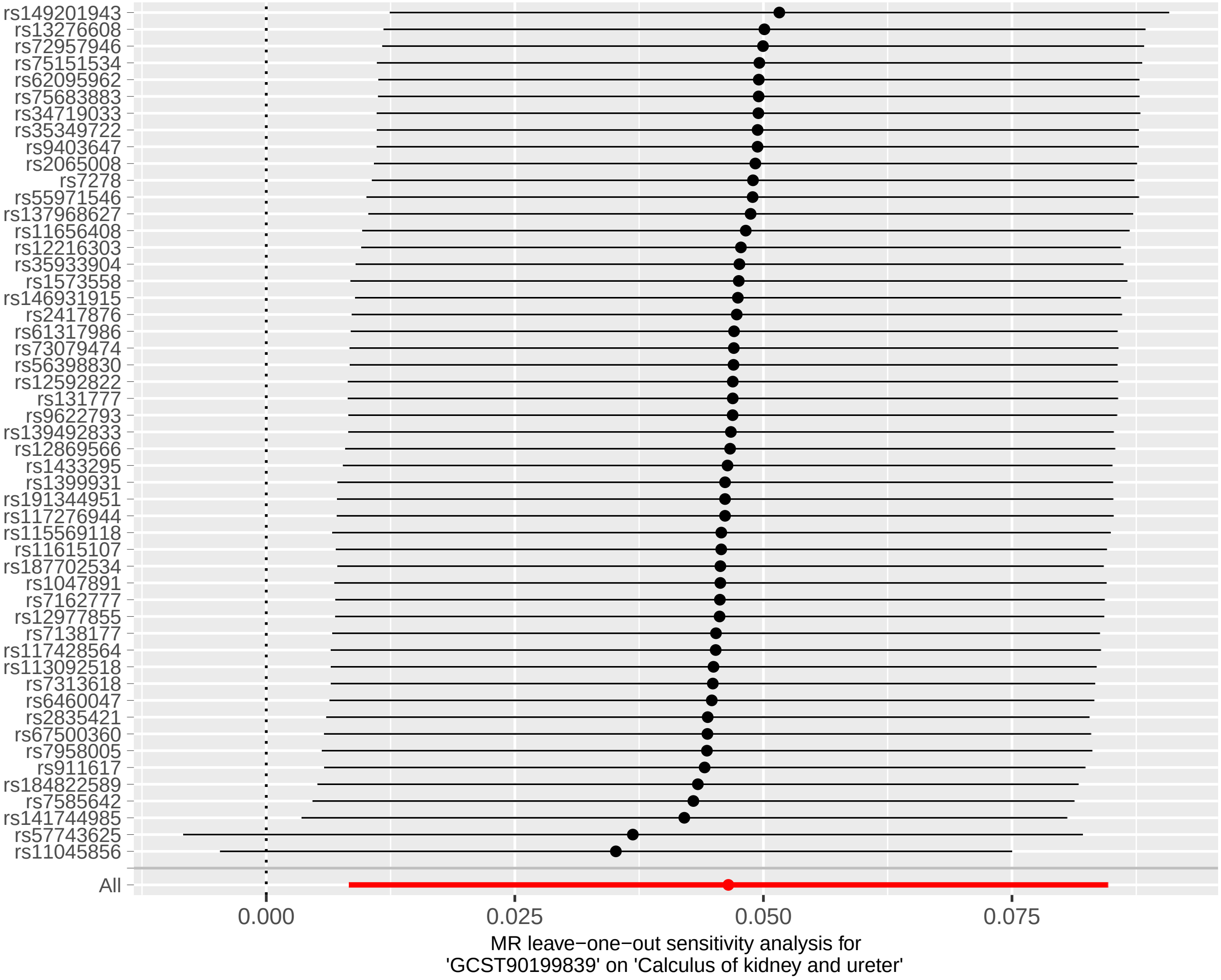
rs149201943
rs13276608
rs72957946
rs75151534
rs62095962
rs75683883
rs34719033
rs35349722
rs9403647
rs2065008
rs7278
rs55971546
rs137968627
rs11656408
rs12216303
rs35933904
rs1573558
rs146931915
rs2417876
rs61317986
rs73079474
rs56398830
rs12592822
rs131777
rs9622793
rs139492833
rs12869566
rs1433295
rs1399931
rs191344951
rs117276944
rs115569118
rs11615107
rs187702534
rs1047891
rs7162777
rs12977855
rs7138177
rs117428564
rs113092518
rs7313618
rs6460047
rs2835421
rs67500360
rs7958005
rs911617
rs184822589
rs7585642
rs141744985
rs57743625
rs11045856
All
0.000
0.025
0.050
0.075
MR leave−one−out sensitivity analysis for
'GCST90199839' on 'Calculus of kidney and ureter'

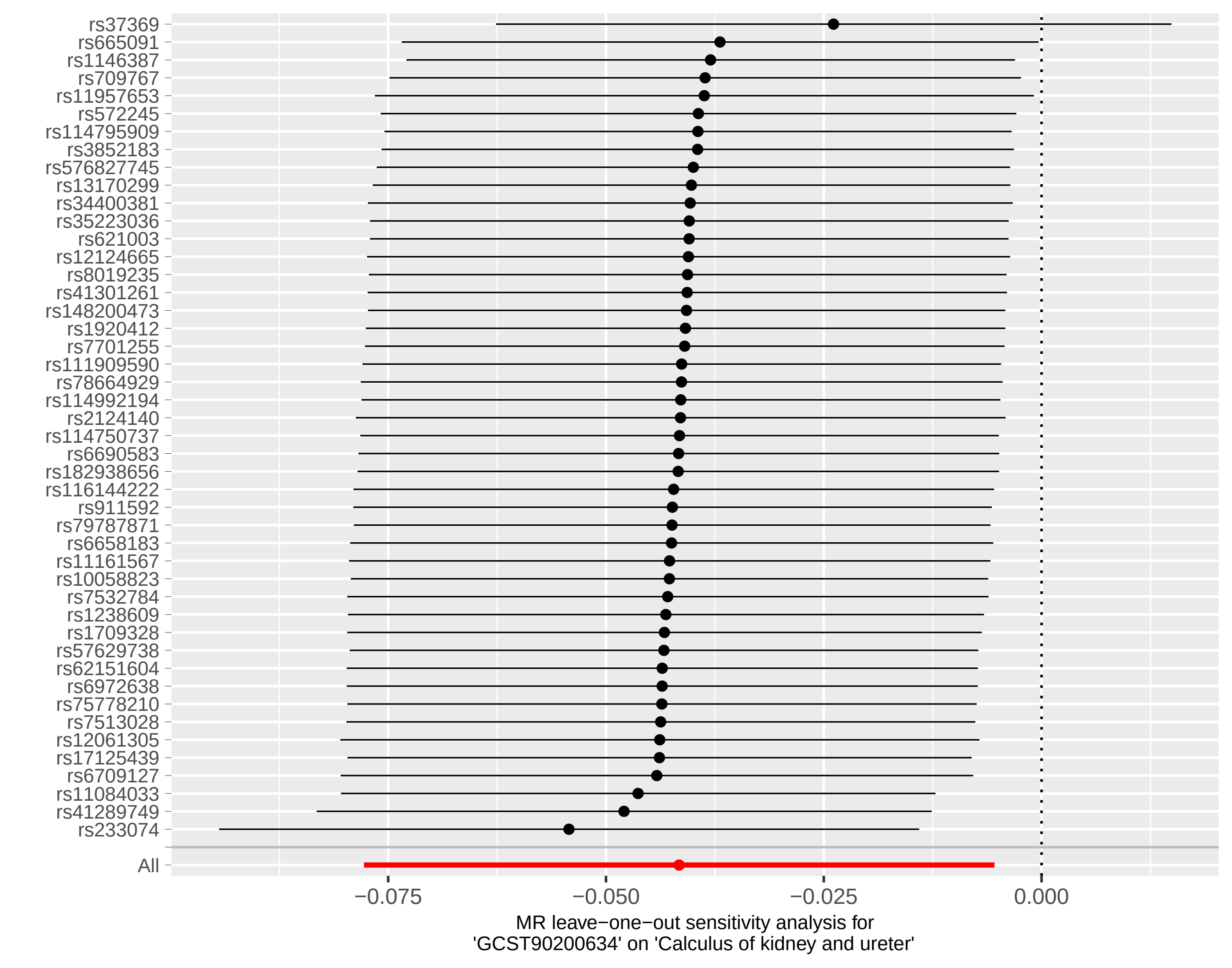
rs37369
rs665091
rs1146387
rs709767
rs11957653
rs572245
rs114795909
rs3852183
rs576827745
rs13170299
rs34400381
rs35223036
rs621003
rs12124665
rs8019235
rs41301261
rs148200473
rs1920412
rs7701255
rs111909590
rs78664929
rs114992194
rs2124140
rs114750737
rs6690583
rs182938656
rs116144222
rs911592
rs79787871
rs6658183
rs11161567
rs10058823
rs7532784
rs1238609
rs1709328
rs57629738
rs62151604
rs6972638
rs75778210
rs7513028
rs12061305
rs17125439
rs6709127
rs11084033
rs41289749
rs233074
All
−0.075
−0.050
−0.025
0.000
MR leave−one−out sensitivity analysis for
'GCST90200634' on 'Calculus of kidney and ureter'

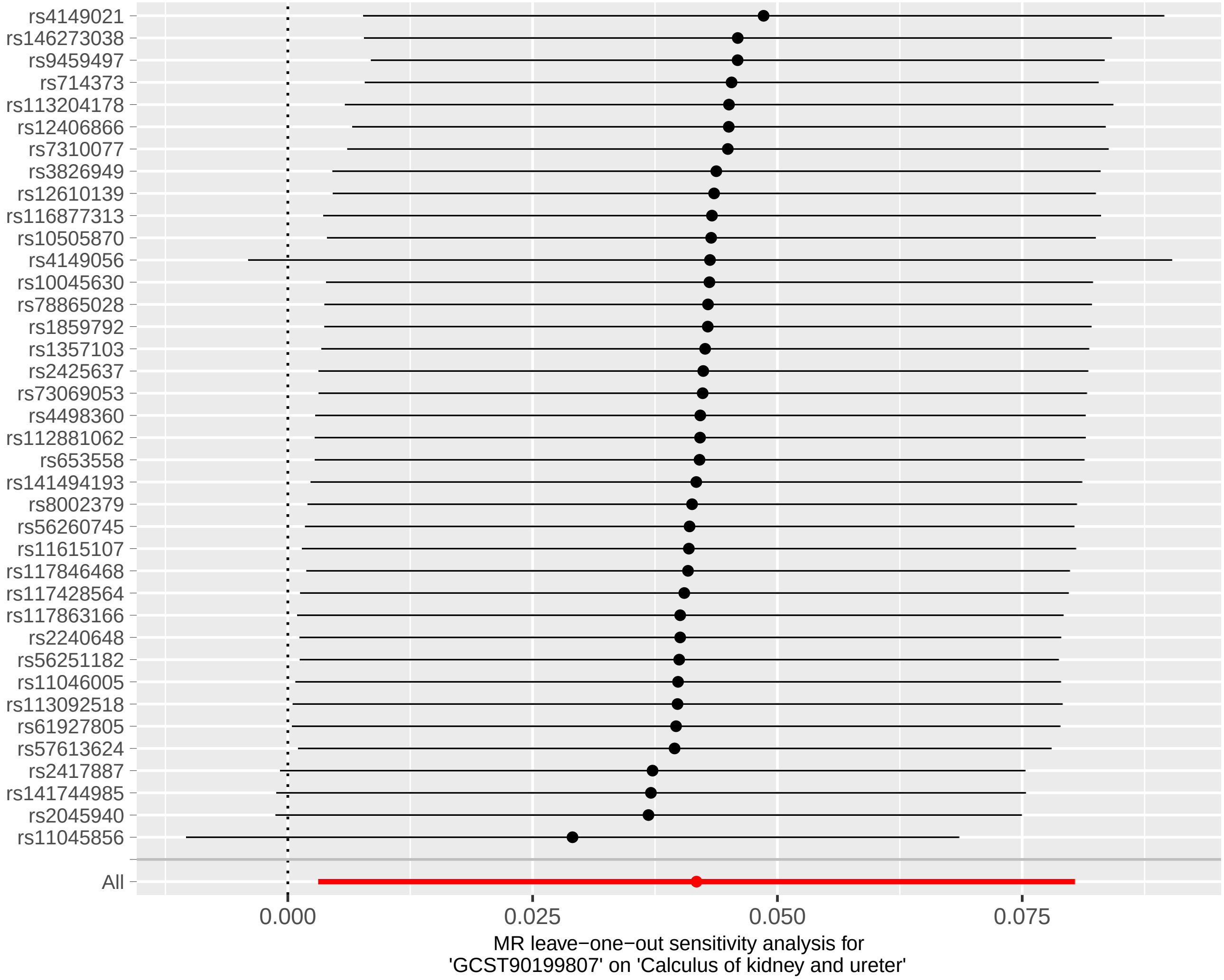
rs4149021
rs146273038
rs9459497
rs714373
rs113204178
rs12406866
rs7310077
rs3826949
rs12610139
rs116877313
rs10505870
rs4149056
rs10045630
rs78865028
rs1859792
rs1357103
rs2425637
rs73069053
rs4498360
rs112881062
rs653558
rs141494193
rs8002379
rs56260745
rs11615107
rs117846468
rs117428564
rs117863166
rs2240648
rs56251182
rs11046005
rs113092518
rs61927805
rs57613624
rs2417887
rs141744985
rs2045940
rs11045856
All
0.000
0.025
0.050
0.075
MR leave−one−out sensitivity analysis for
'GCST90199807' on 'Calculus of kidney and ureter'

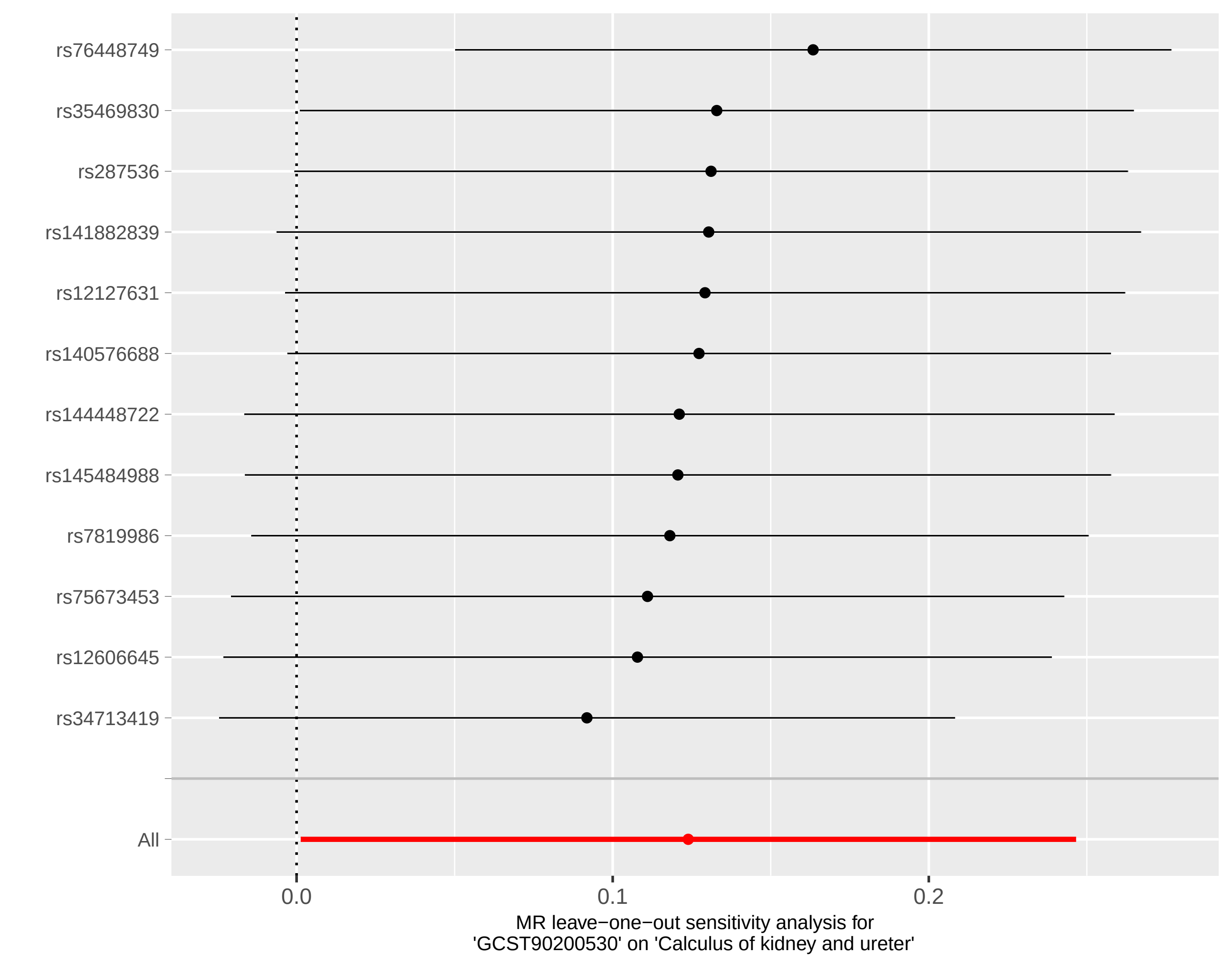
rs76448749
rs35469830
rs287536
rs141882839
rs12127631
rs140576688
rs144448722
rs145484988
rs7819986
rs75673453
rs12606645
rs34713419
All
0.0
0.1
0.2
MR leave−one−out sensitivity analysis for
'GCST90200530' on 'Calculus of kidney and ureter'

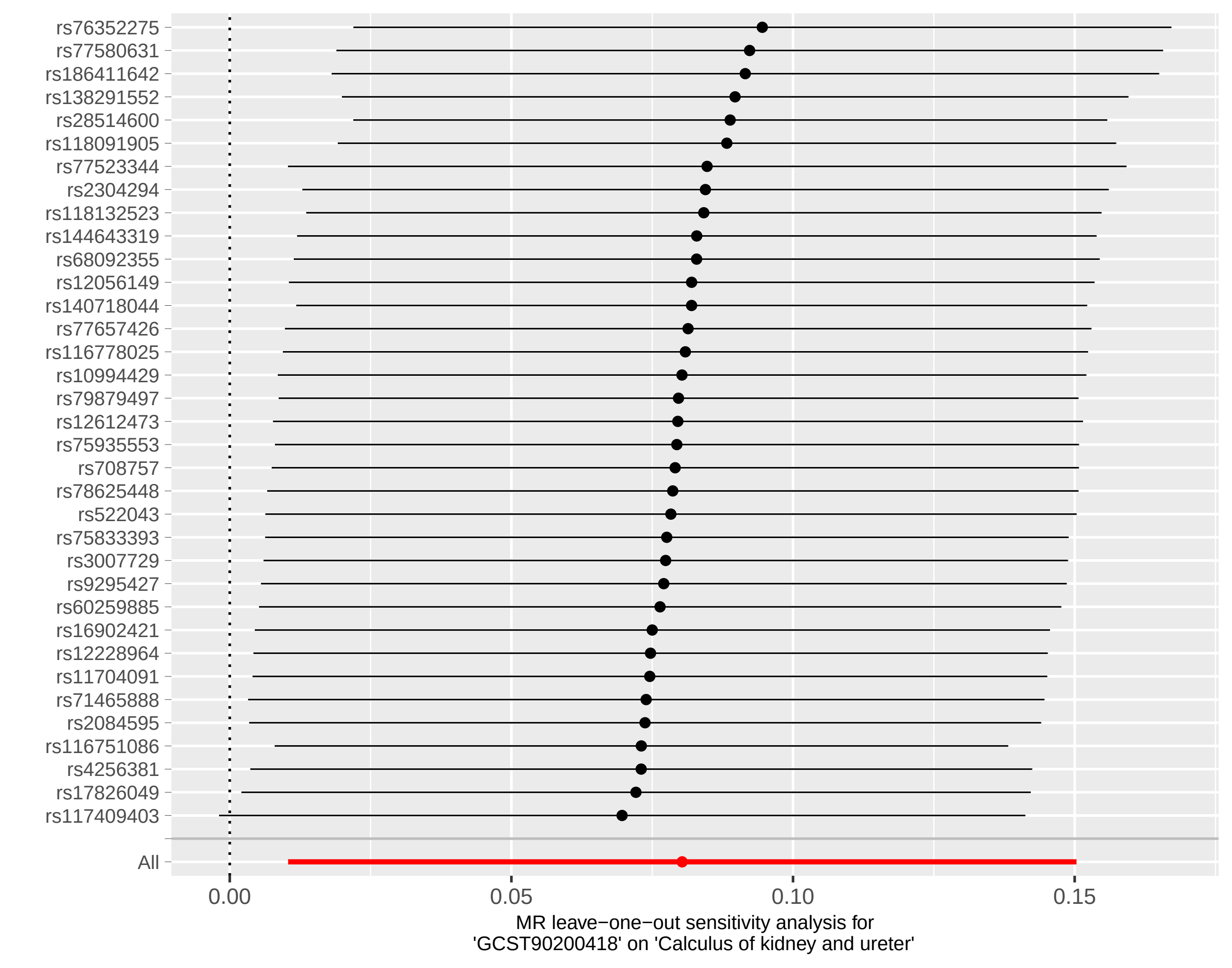
rs76352275
rs77580631
rs186411642
rs138291552
rs28514600
rs118091905
rs77523344
rs2304294
rs118132523
rs144643319
rs68092355
rs12056149
rs140718044
rs77657426
rs116778025
rs10994429
rs79879497
rs12612473
rs75935553
rs708757
rs78625448
rs522043
rs75833393
rs3007729
rs9295427
rs60259885
rs16902421
rs12228964
rs11704091
rs71465888
rs2084595
rs116751086
rs4256381
rs17826049
rs117409403
All
0.00
0.05
0.10
0.15
MR leave−one−out sensitivity analysis for
'GCST90200418' on 'Calculus of kidney and ureter'

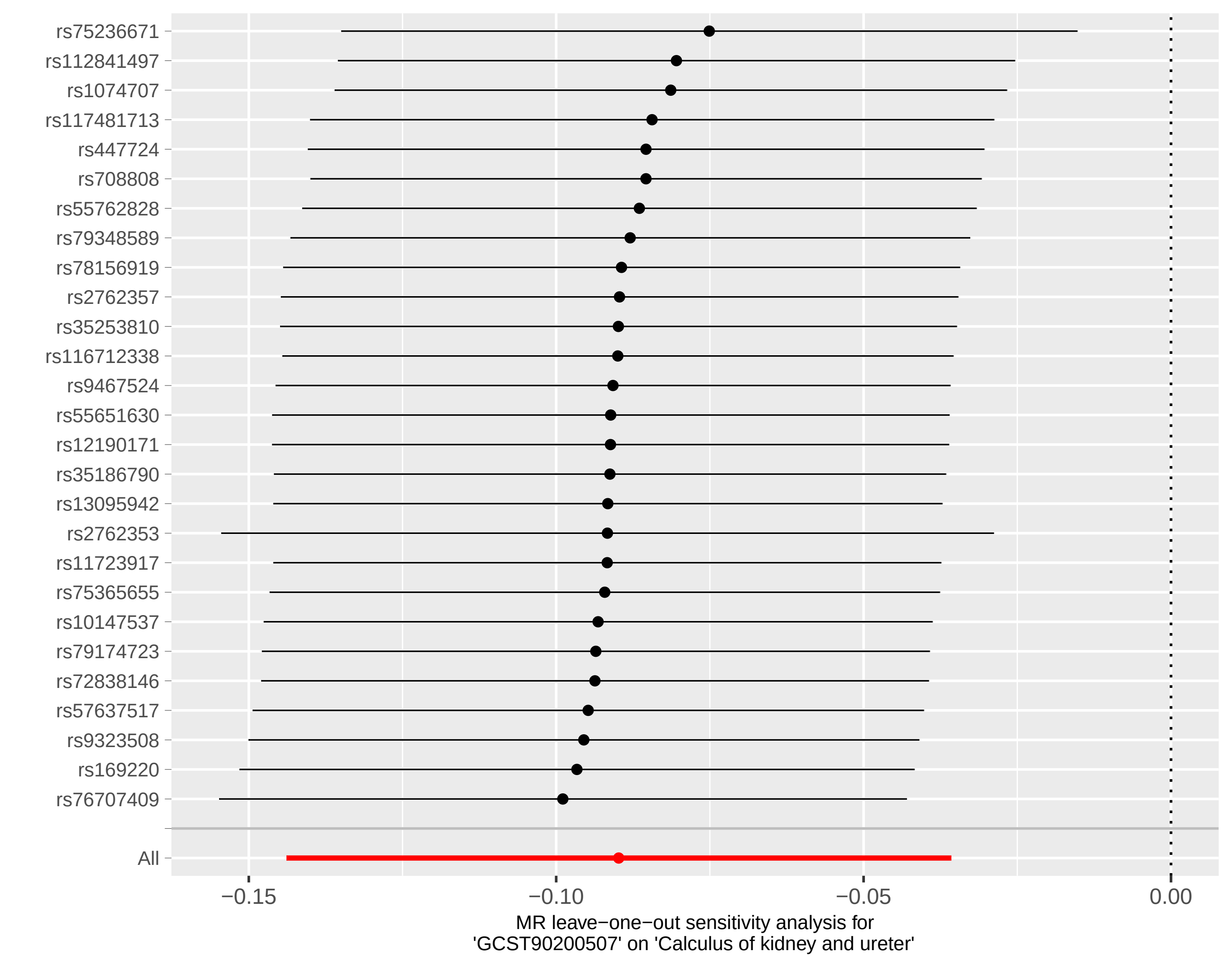

rs75236671
rs112841497
rs1074707
rs117481713
rs447724
rs708808
rs55762828
rs79348589
rs78156919
rs2762357
rs35253810
rs116712338
rs9467524
rs55651630
rs12190171
rs35186790
rs13095942
rs2762353
rs11723917
rs75365655
rs10147537
rs79174723
rs72838146
rs57637517
rs9323508
rs169220
rs76707409
All
−0.15
−0.10
−0.05
0.00
MR leave−one−out sensitivity analysis for
'GCST90200507' on 'Calculus of kidney and ureter'

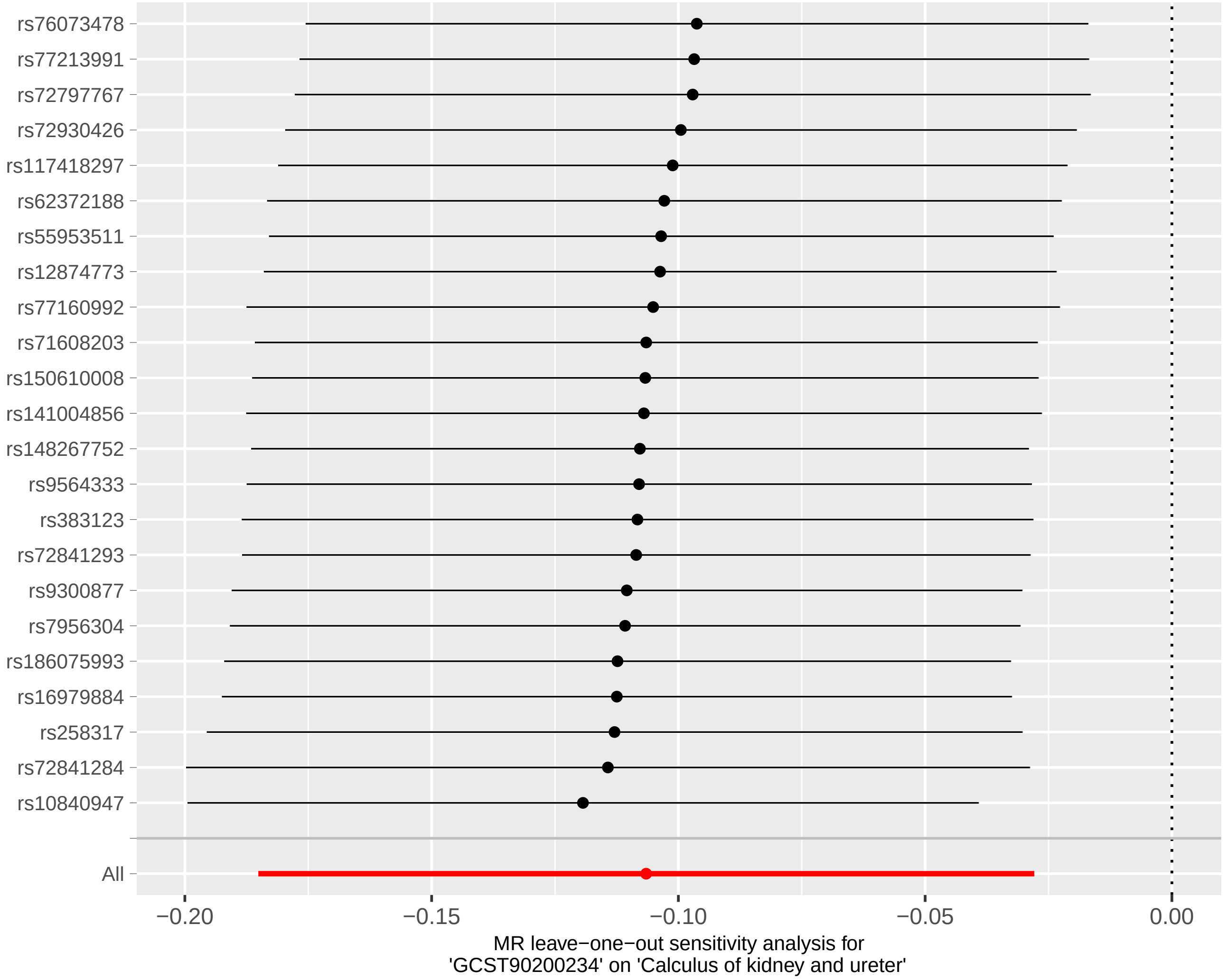
rs76073478
rs77213991
rs72797767
rs72930426
rs117418297
rs62372188
rs55953511
rs12874773
rs77160992
rs71608203
rs150610008
rs141004856
rs148267752
rs9564333
rs383123
rs72841293
rs9300877
rs7956304
rs186075993
rs16979884
rs258317
rs72841284
rs10840947
All
−0.20
−0.15
−0.10
−0.05
0.00
MR leave−one−out sensitivity analysis for
'GCST90200234' on 'Calculus of kidney and ureter'

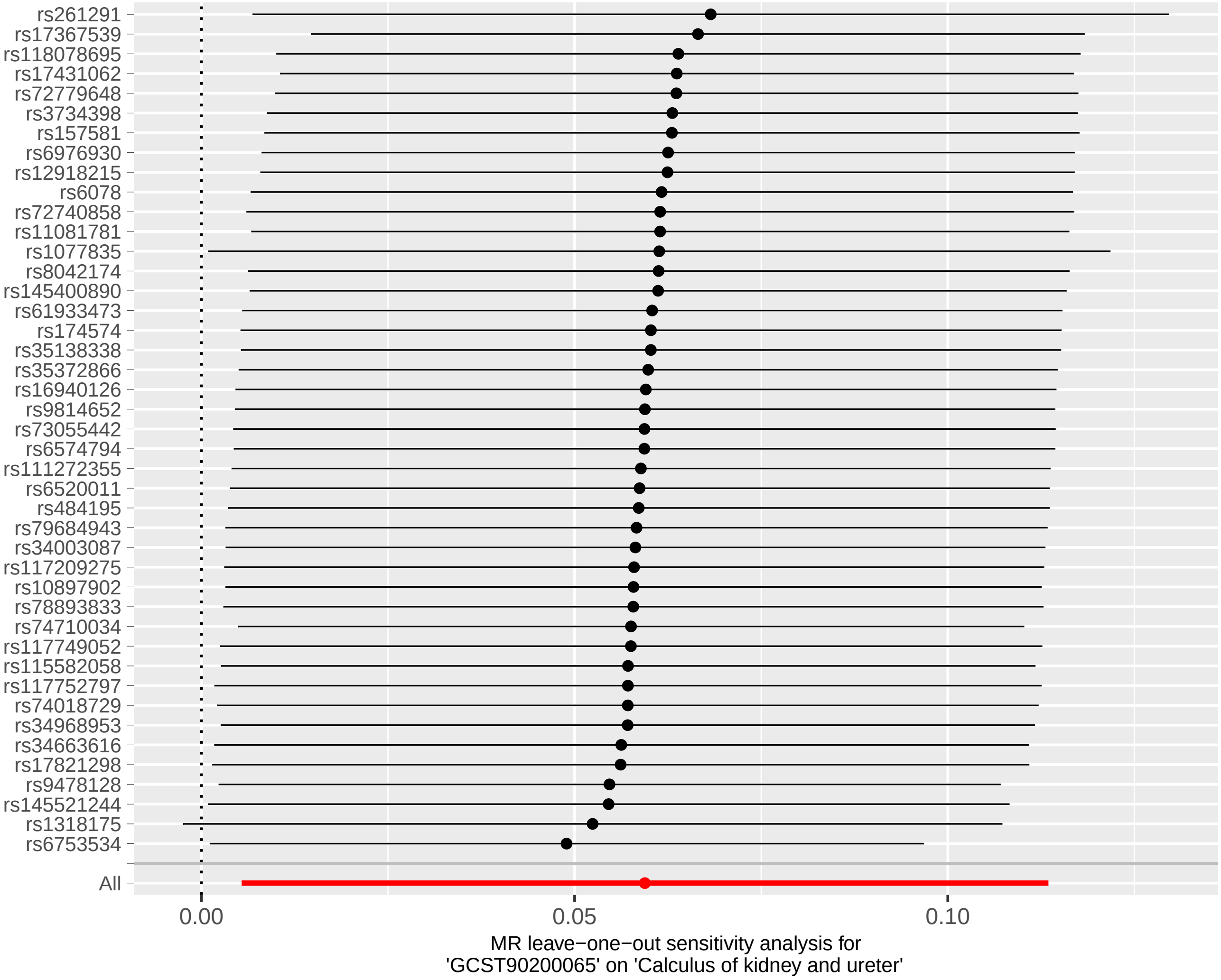

rs261291
rs17367539
rs118078695
rs17431062
rs72779648
rs3734398
rs157581
rs6976930
rs12918215
rs6078
rs72740858
rs11081781
rs1077835
rs8042174
rs145400890
rs61933473
rs174574
rs35138338
rs35372866
rs16940126
rs9814652
rs73055442
rs6574794
rs111272355
rs6520011
rs484195
rs79684943
rs34003087
rs117209275
rs10897902
rs78893833
rs74710034
rs117749052
rs115582058
rs117752797
rs74018729
rs34968953
rs34663616
rs17821298
rs9478128
rs145521244
rs1318175
rs6753534
All
0.00
0.05
0.10
MR leave−one−out sensitivity analysis for
'GCST90200065' on 'Calculus of kidney and ureter'

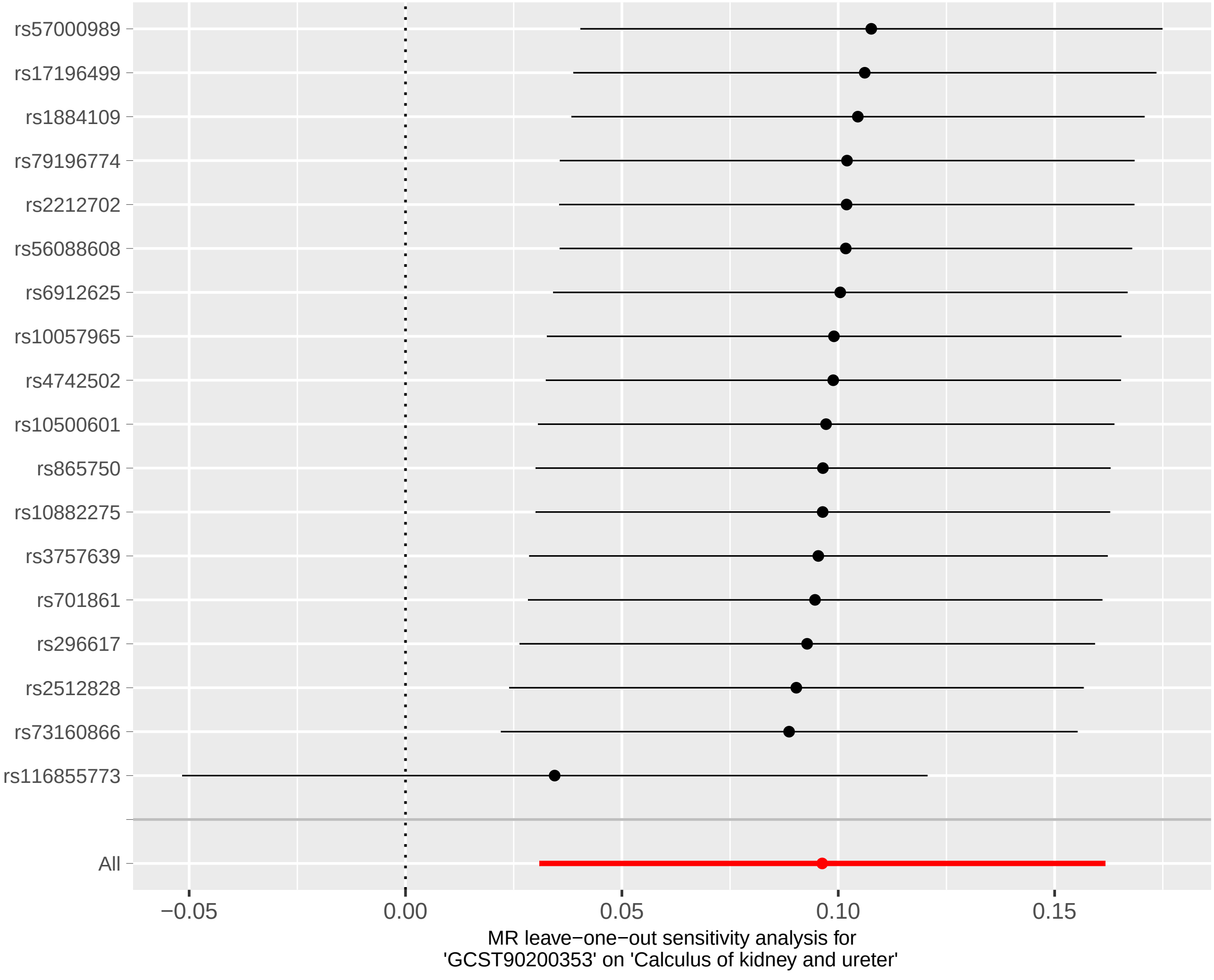
rs57000989
rs17196499
rs1884109
rs79196774
rs2212702
rs56088608
rs6912625
rs10057965
rs4742502
rs10500601
rs865750
rs10882275
rs3757639
rs701861
rs296617
rs2512828
rs73160866
rs116855773
All
−0.05
0.00
0.05
0.10
0.15
MR leave−one−out sensitivity analysis for
'GCST90200353' on 'Calculus of kidney and ureter'

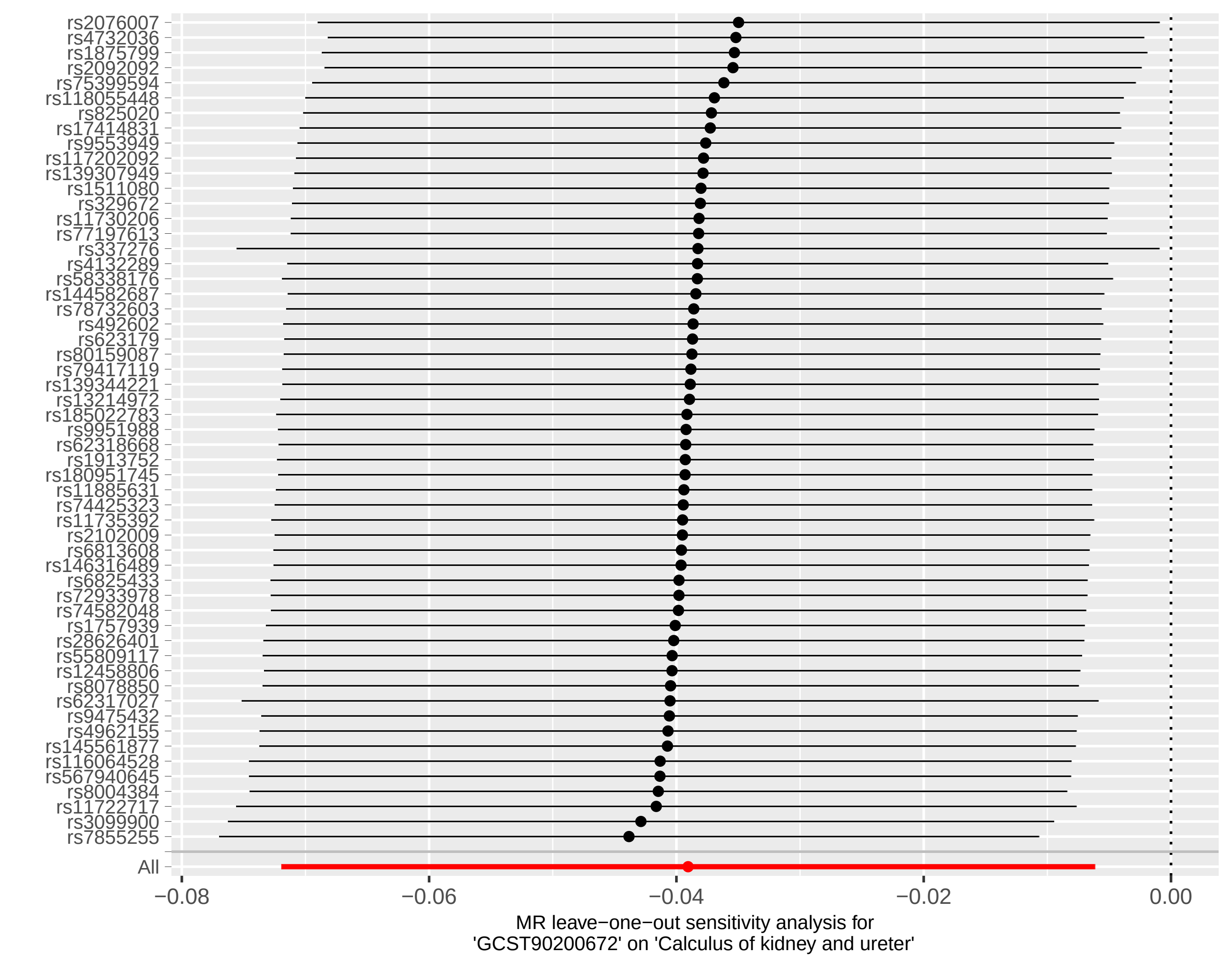
rs2076007
rs4732036
rs1875799
rs2092092
rs75399594
rs118055448
rs825020
rs17414831
rs9553949
rs117202092
rs139307949
rs1511080
rs329672
rs11730206
rs77197613
rs337276
rs4132289
rs58338176
rs144582687
rs78732603
rs492602
rs623179
rs80159087
rs79417119
rs139344221
rs13214972
rs185022783
rs9951988
rs62318668
rs1913752
rs180951745
rs11885631
rs74425323
rs11735392
rs2102009
rs6813608
rs146316489
rs6825433
rs72933978
rs74582048
rs1757939
rs28626401
rs55809117
rs12458806
rs8078850
rs62317027
rs9475432
rs4962155
rs145561877
rs116064528
rs567940645
rs8004384
rs11722717
rs3099900
rs7855255
All
−0.08
−0.06
−0.04
−0.02
0.00
MR leave−one−out sensitivity analysis for
'GCST90200672' on 'Calculus of kidney and ureter'

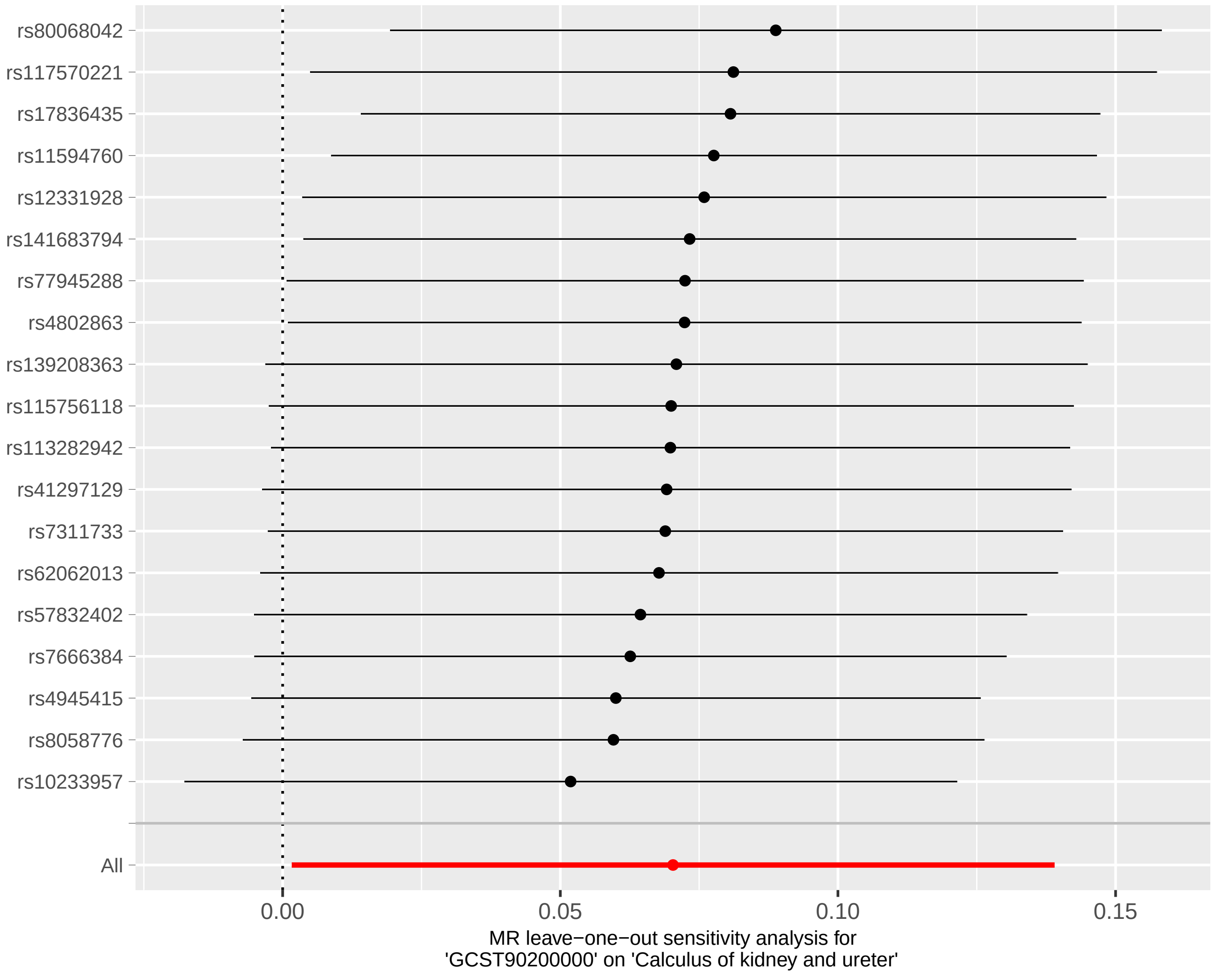

rs80068042
rs117570221
rs17836435
rs11594760
rs12331928
rs141683794
rs77945288
rs4802863
rs139208363
rs115756118
rs113282942
rs41297129
rs7311733
rs62062013
rs57832402
rs7666384
rs4945415
rs8058776
rs10233957
All
0.00
0.05
0.10
0.15
MR leave−one−out sensitivity analysis for
'GCST90200000' on 'Calculus of kidney and ureter'

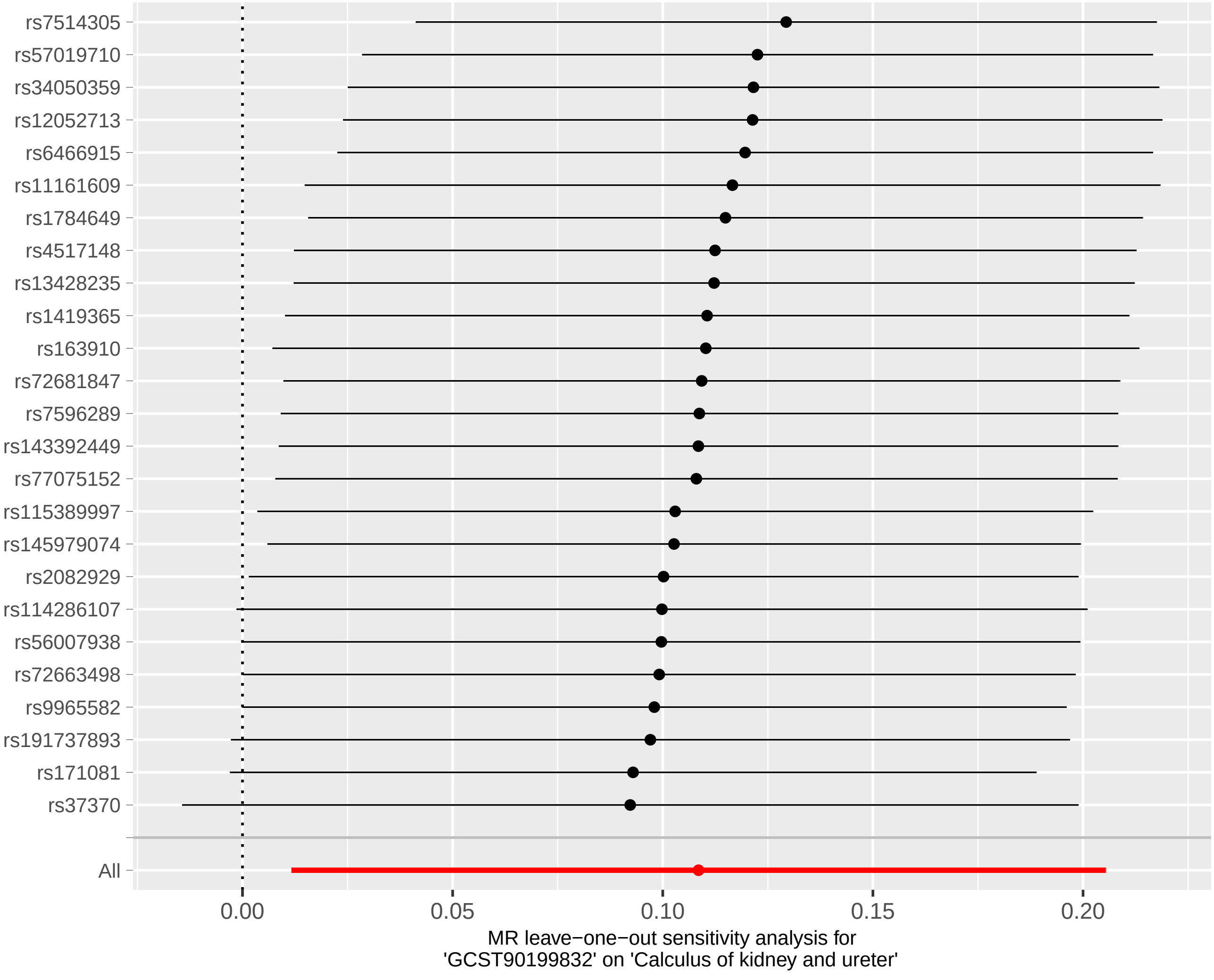
rs7514305
rs57019710
rs34050359
rs12052713
rs6466915
rs11161609
rs1784649
rs4517148
rs13428235
rs1419365
rs163910
rs72681847
rs7596289
rs143392449
rs77075152
rs115389997
rs145979074
rs2082929
rs114286107
rs56007938
rs72663498
rs9965582
rs191737893
rs171081
rs37370
All
0.00
0.05
0.10
0.15
0.20
MR leave−one−out sensitivity analysis for
'GCST90199832' on 'Calculus of kidney and ureter'

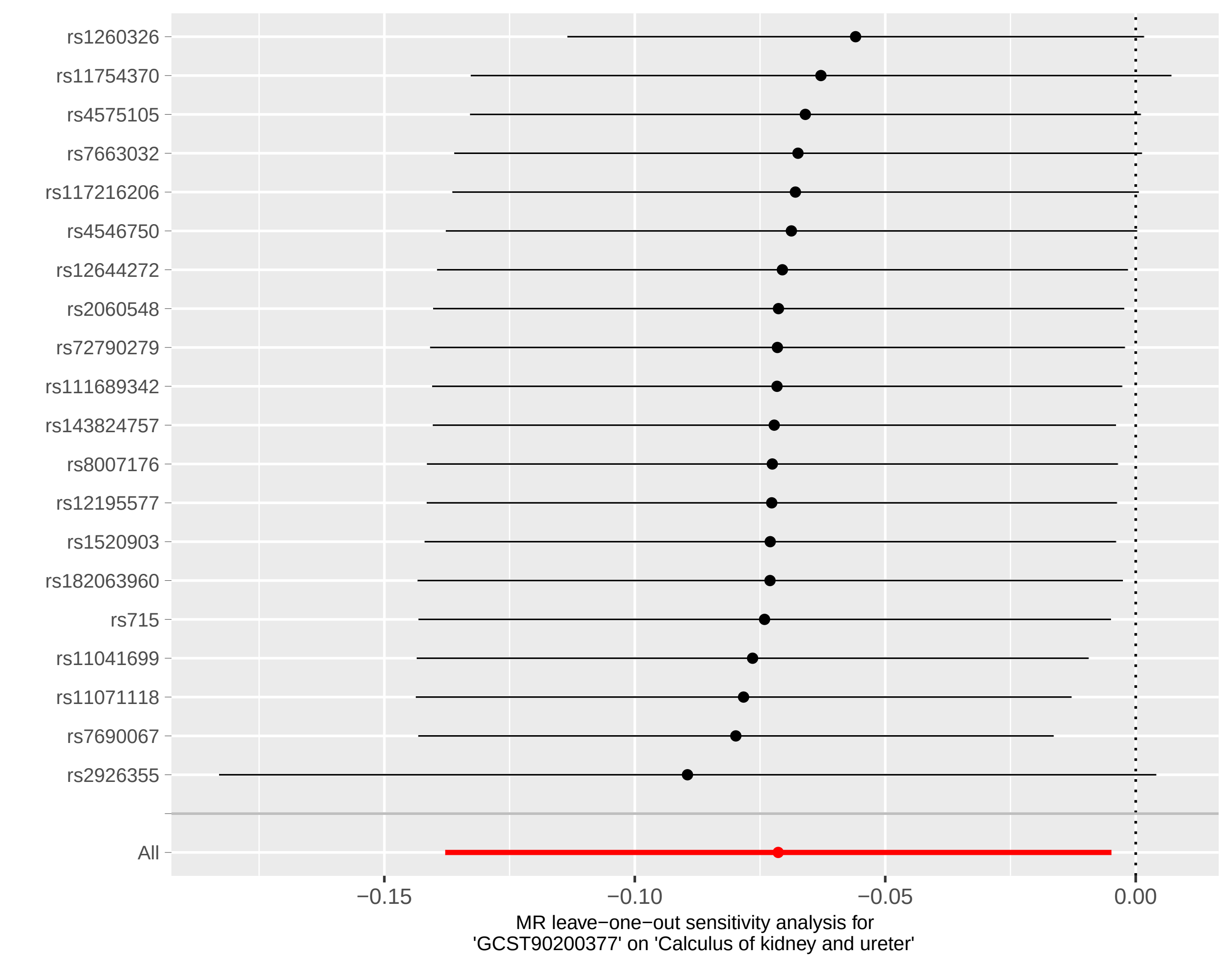
rs1260326
rs11754370
rs4575105
rs7663032
rs117216206
rs4546750
rs12644272
rs2060548
rs72790279
rs111689342
rs143824757
rs8007176
rs12195577
rs1520903
rs182063960
rs715
rs11041699
rs11071118
rs7690067
rs2926355
All
−0.15
−0.10
−0.05
0.00
MR leave−one−out sensitivity analysis for
'GCST90200377' on 'Calculus of kidney and ureter'

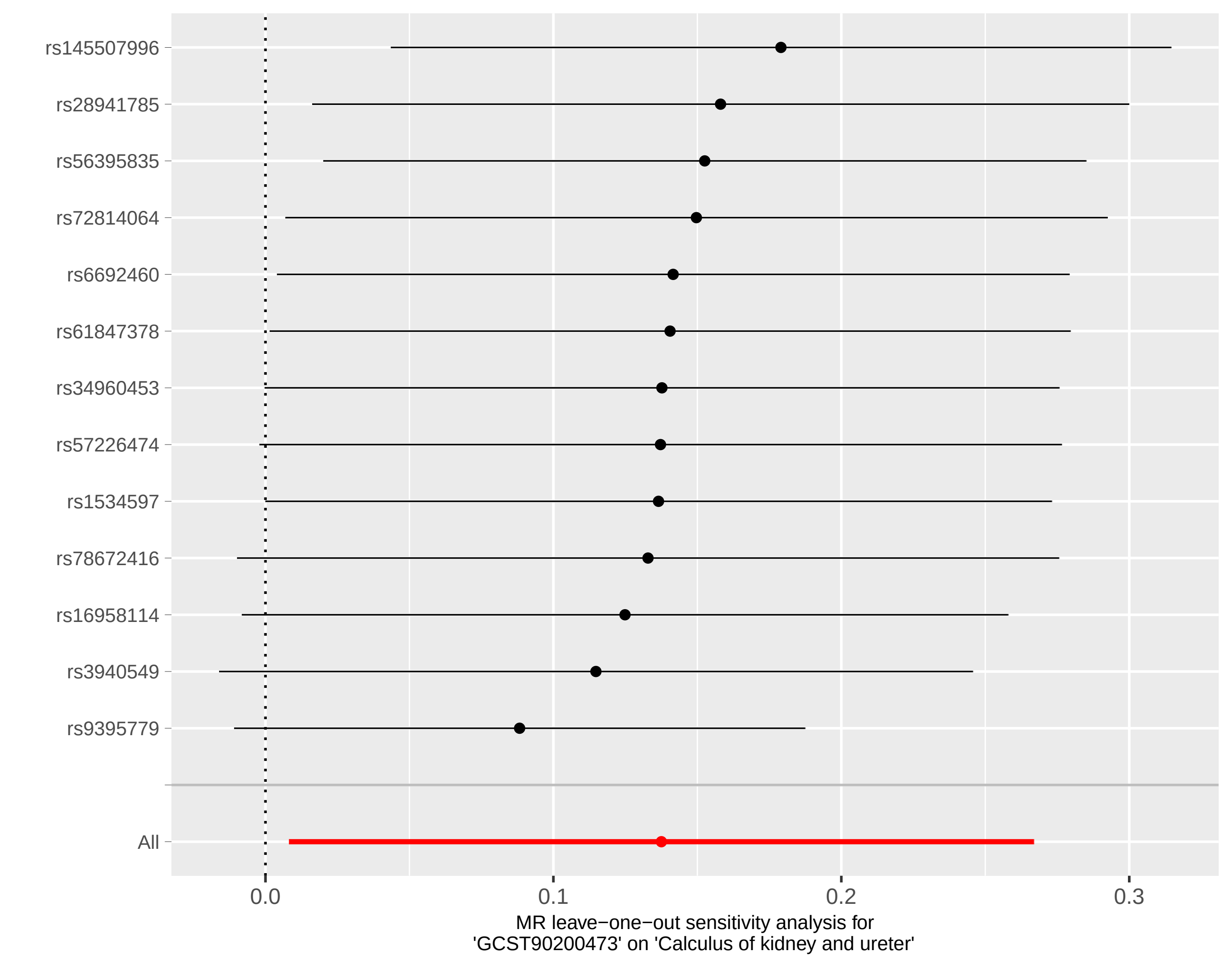
rs145507996
rs28941785
rs56395835
rs72814064
rs6692460
rs61847378
rs34960453
rs57226474
rs1534597
rs78672416
rs16958114
rs3940549
rs9395779
All
0.0
0.1
0.2
0.3
MR leave−one−out sensitivity analysis for
'GCST90200473' on 'Calculus of kidney and ureter'

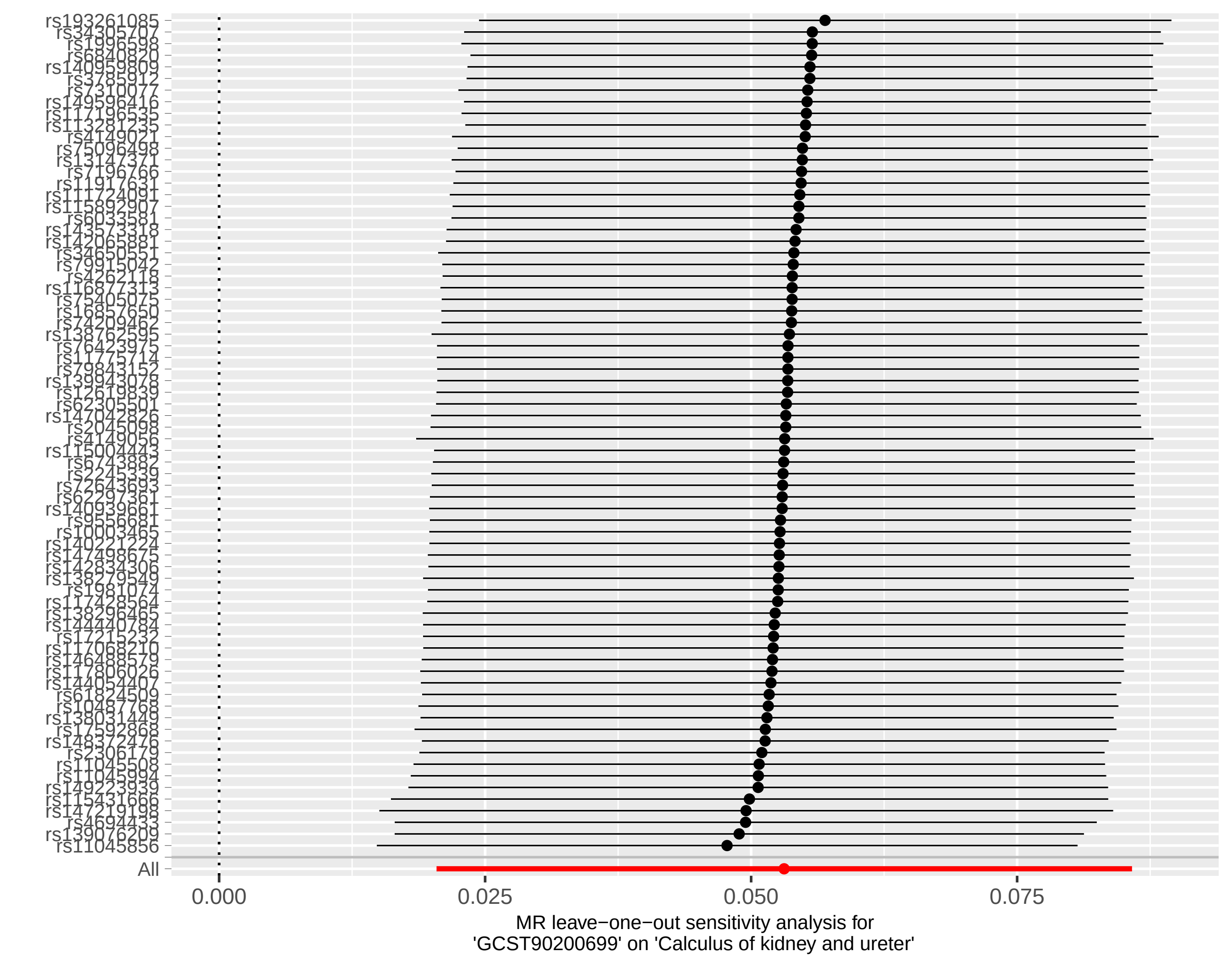
rs193261085
rs34305707
rs1996598
rs6840820
rs140959809
rs3785912
rs7310077
rs149596416
rs117196535
rs113281235
rs4149021
rs75096498
rs13147371
rs7196766
rs11917631
rs111724091
rs115892907
rs6033581
rs143573318
rs142065881
rs34650551
rs79915042
rs4262118
rs116877313
rs75405075
rs16857650
rs74209462
rs138762595
rs76423975
rs11775714
rs79843152
rs139943078
rs12619839
rs62305501
rs147042826
rs2045098
rs4149056
rs115004443
rs6743882
rs2245339
rs72643693
rs62297361
rs140939661
rs9556681
rs10003465
rs140221224
rs147498675
rs142834306
rs138279549
rs1981074
rs117428564
rs138296465
rs144440784
rs17215232
rs117068210
rs146488579
rs117806026
rs144054407
rs61824509
rs10487768
rs138031449
rs17592868
rs148372476
rs2306179
rs11045508
rs11045994
rs149223939
rs115431666
rs147219198
rs4694433
rs139076209
rs11045856
All
0.000
0.025
0.050
0.075
MR leave−one−out sensitivity analysis for
'GCST90200699' on 'Calculus of kidney and ureter'

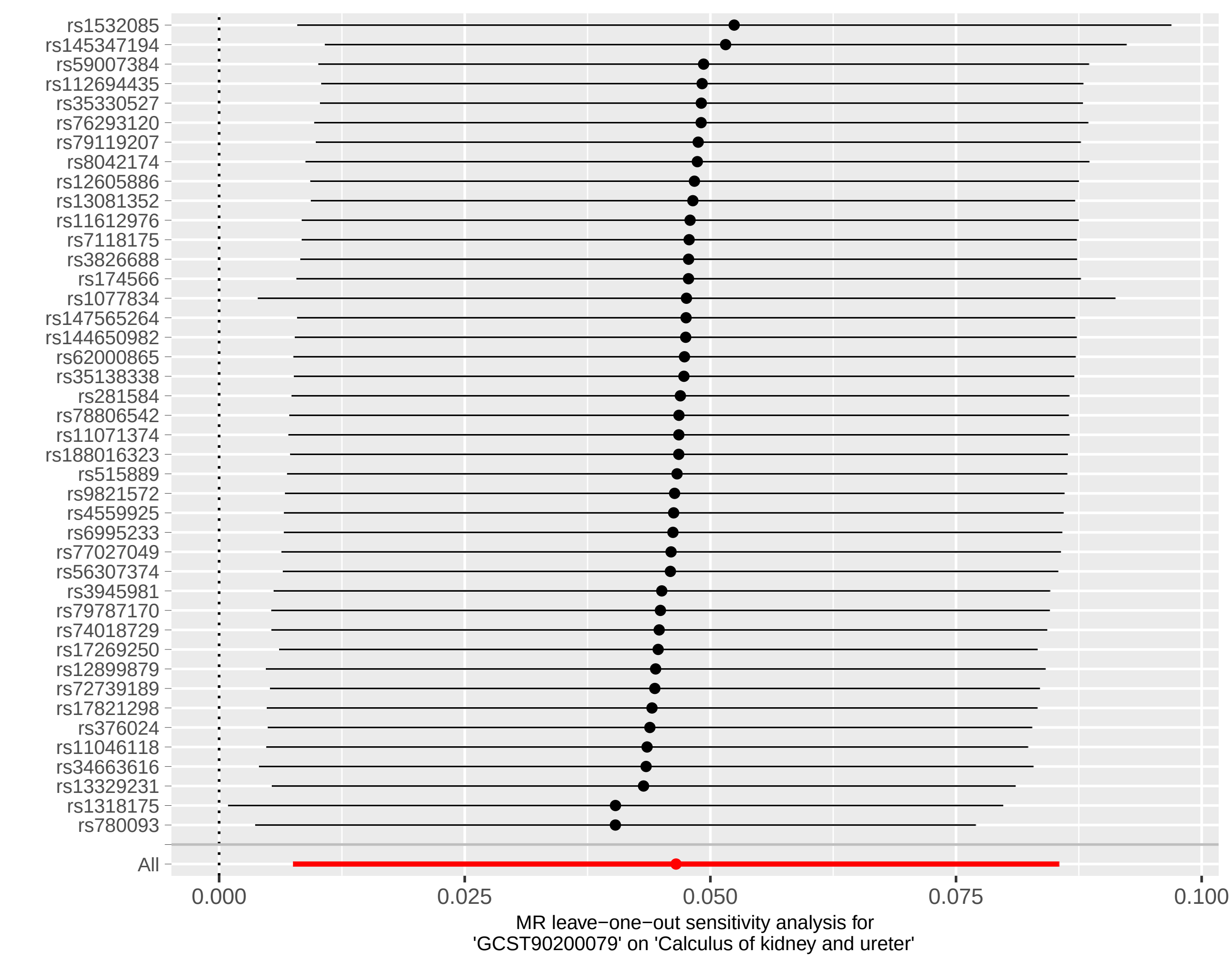
rs1532085
rs145347194
rs59007384
rs112694435
rs35330527
rs76293120
rs79119207
rs8042174
rs12605886
rs13081352
rs11612976
rs7118175
rs3826688
rs174566
rs1077834
rs147565264
rs144650982
rs62000865
rs35138338
rs281584
rs78806542
rs11071374
rs188016323
rs515889
rs9821572
rs4559925
rs6995233
rs77027049
rs56307374
rs3945981
rs79787170
rs74018729
rs17269250
rs12899879
rs72739189
rs17821298
rs376024
rs11046118
rs34663616
rs13329231
rs1318175
rs780093
All
0.000
0.025
0.050
0.075
0.100
MR leave−one−out sensitivity analysis for
'GCST90200079' on 'Calculus of kidney and ureter'

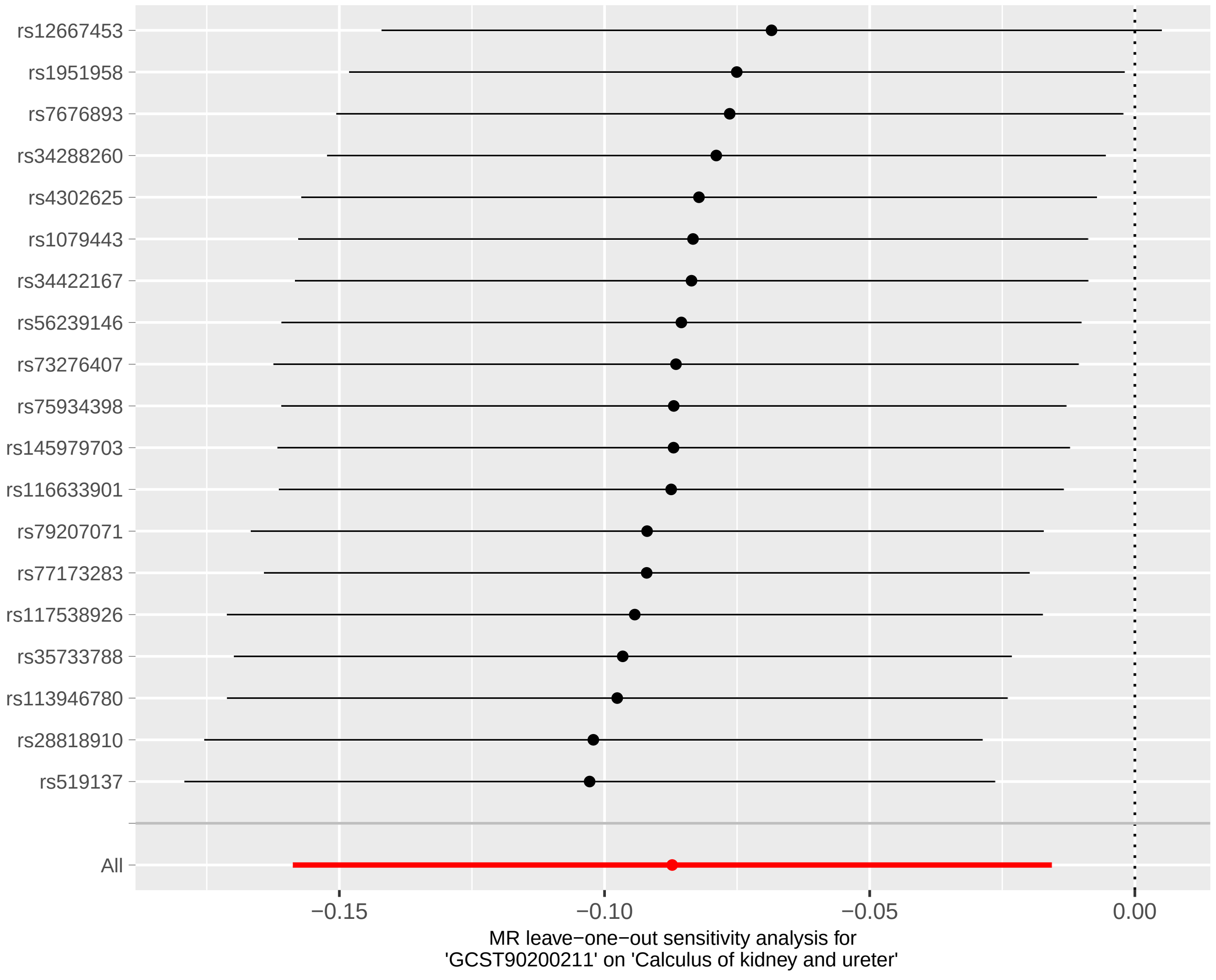
rs12667453
rs1951958
rs7676893
rs34288260
rs4302625
rs1079443
rs34422167
rs56239146
rs73276407
rs75934398
rs145979703
rs116633901
rs79207071
rs77173283
rs117538926
rs35733788
rs113946780
rs28818910
rs519137
All
−0.15
−0.10
−0.05
0.00
MR leave−one−out sensitivity analysis for
'GCST90200211' on 'Calculus of kidney and ureter'

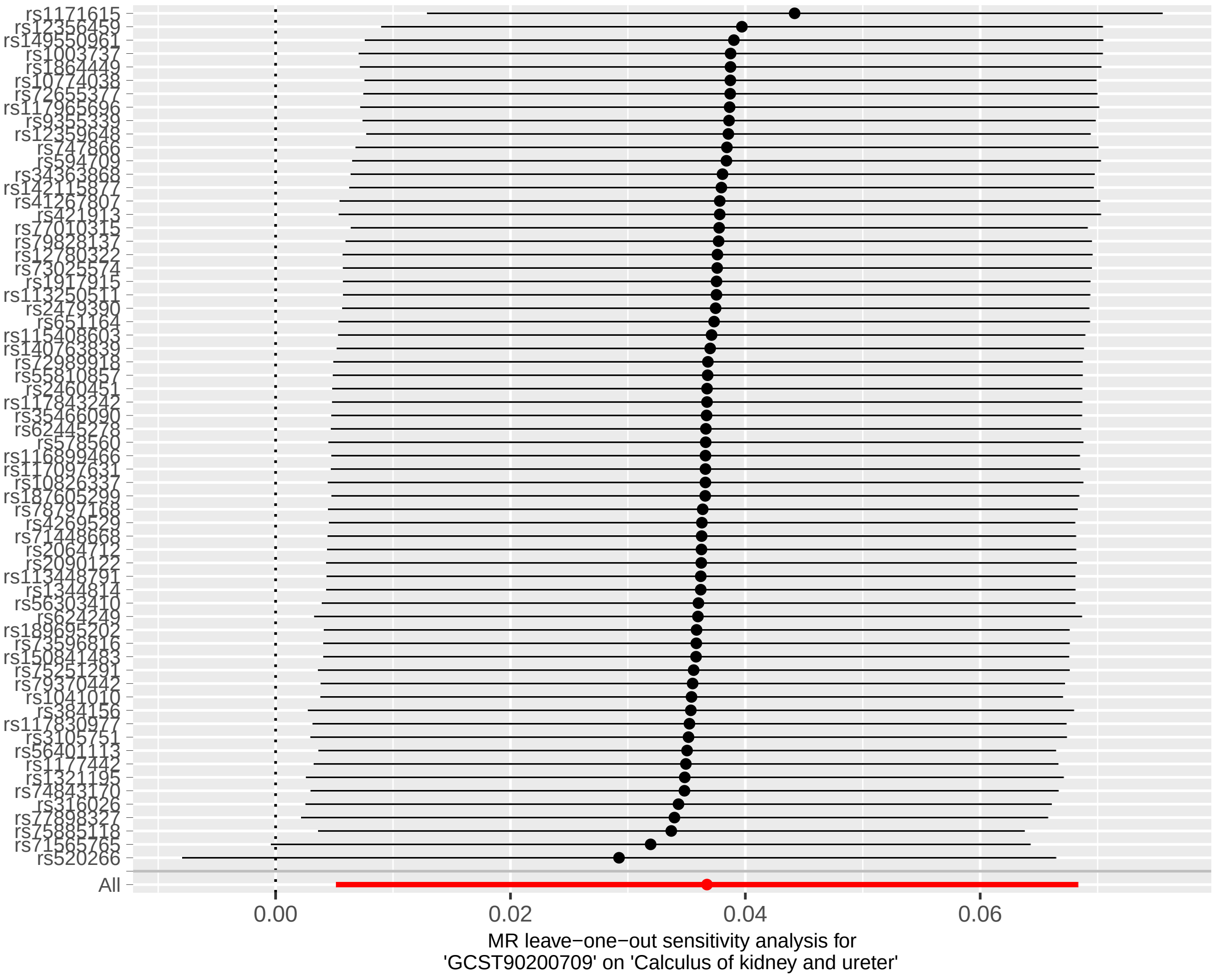
rs1171615
rs12356459
rs149550961
rs1003737
rs1864449
rs10774038
rs72655377
rs117965696
rs9355339
rs12359648
rs747866
rs594709
rs34363868
rs142115877
rs41267807
rs421913
rs77010315
rs79828137
rs12780322
rs73025574
rs1917915
rs113250511
rs2479390
rs651164
rs115408603
rs140763839
rs72989918
rs55810857
rs2460451
rs117843242
rs35466090
rs62445278
rs578560
rs116899466
rs117097631
rs10826337
rs187605299
rs78797168
rs4269529
rs71448668
rs2064712
rs2090122
rs113448791
rs1344814
rs56303410
rs624249
rs189695202
rs73596816
rs150841483
rs75251291
rs79370442
rs1041010
rs384156
rs117830977
rs3105751
rs56401113
rs1177442
rs1321195
rs74843170
rs316026
rs77898327
rs75885118
rs71565765
rs520266
All
0.00
0.02
0.04
0.06
MR leave−one−out sensitivity analysis for
'GCST90200709' on 'Calculus of kidney and ureter'

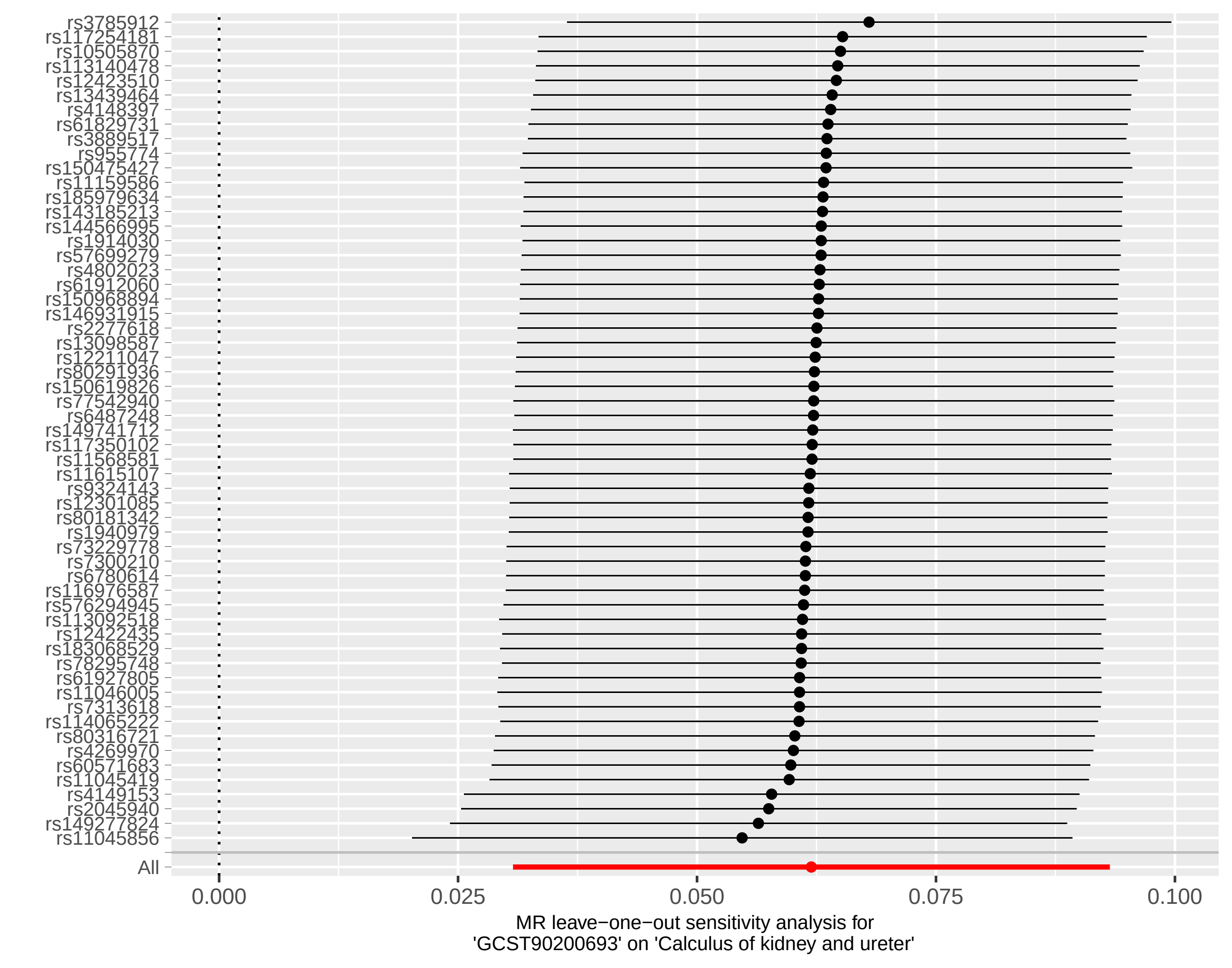
rs3785912
rs117254181
rs10505870
rs113140478
rs12423510
rs13439464
rs4148397
rs61829731
rs3889517
rs955774
rs150475427
rs11159586
rs185979634
rs143185213
rs144566995
rs1914030
rs57699279
rs4802023
rs61912060
rs150968894
rs146931915
rs2277618
rs13098587
rs12211047
rs80291936
rs150619826
rs77542940
rs6487248
rs149741712
rs117350102
rs11568581
rs11615107
rs9324143
rs12301085
rs80181342
rs1940979
rs73229778
rs7300210
rs6780614
rs116976587
rs576294945
rs113092518
rs12422435
rs183068529
rs78295748
rs61927805
rs11046005
rs7313618
rs114065222
rs80316721
rs4269970
rs60571683
rs11045419
rs4149153
rs2045940
rs149277824
rs11045856
All
0.000
0.025
0.050
0.075
0.100
MR leave−one−out sensitivity analysis for
'GCST90200693' on 'Calculus of kidney and ureter'

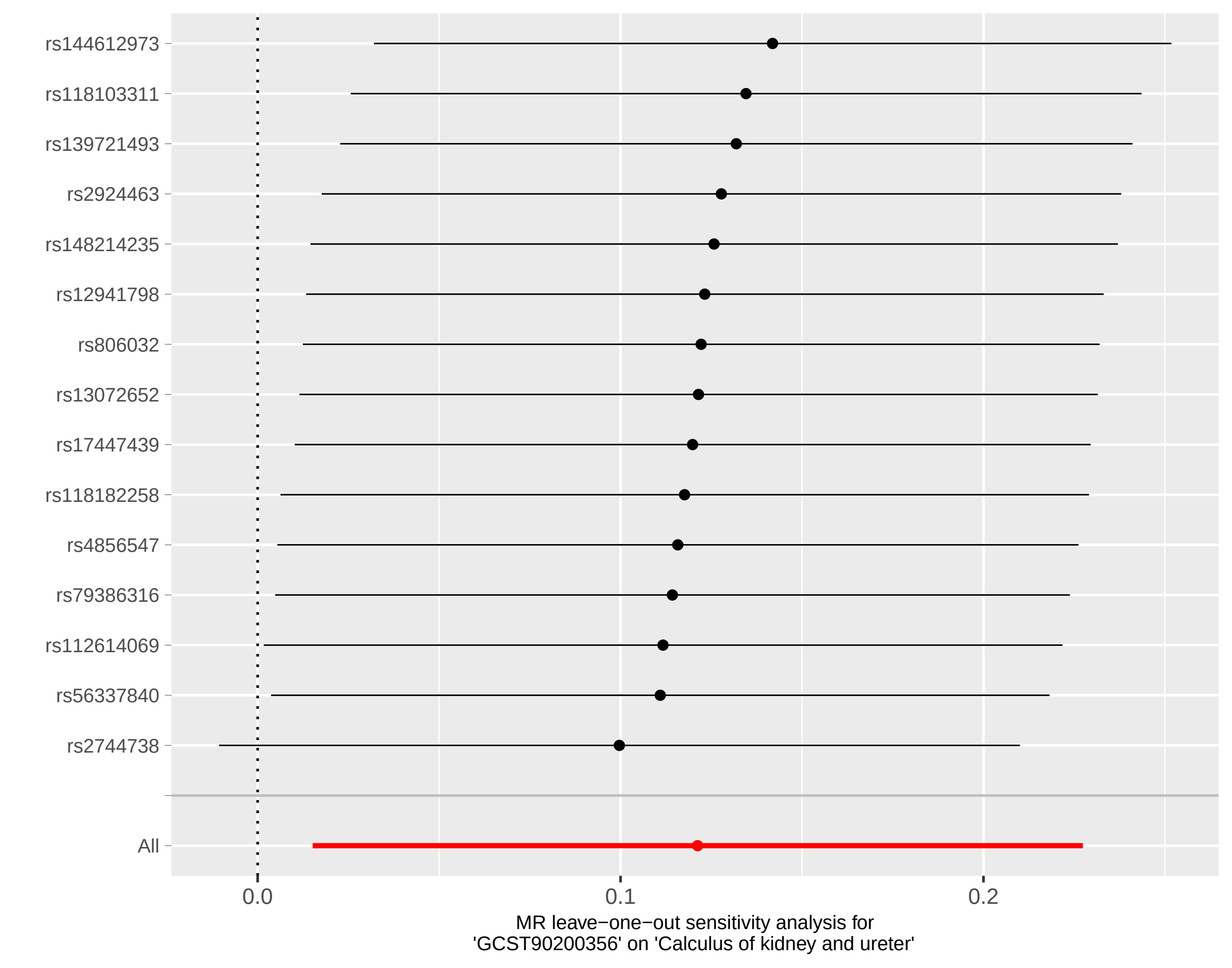
rs144612973
rs118103311
rs139721493
rs2924463
rs148214235
rs12941798
rs806032
rs13072652
rs17447439
rs118182258
rs4856547
rs79386316
rs112614069
rs56337840
rs2744738
All
0.0
0.1
0.2
MR leave−one−out sensitivity analysis for
'GCST90200356' on 'Calculus of kidney and ureter'

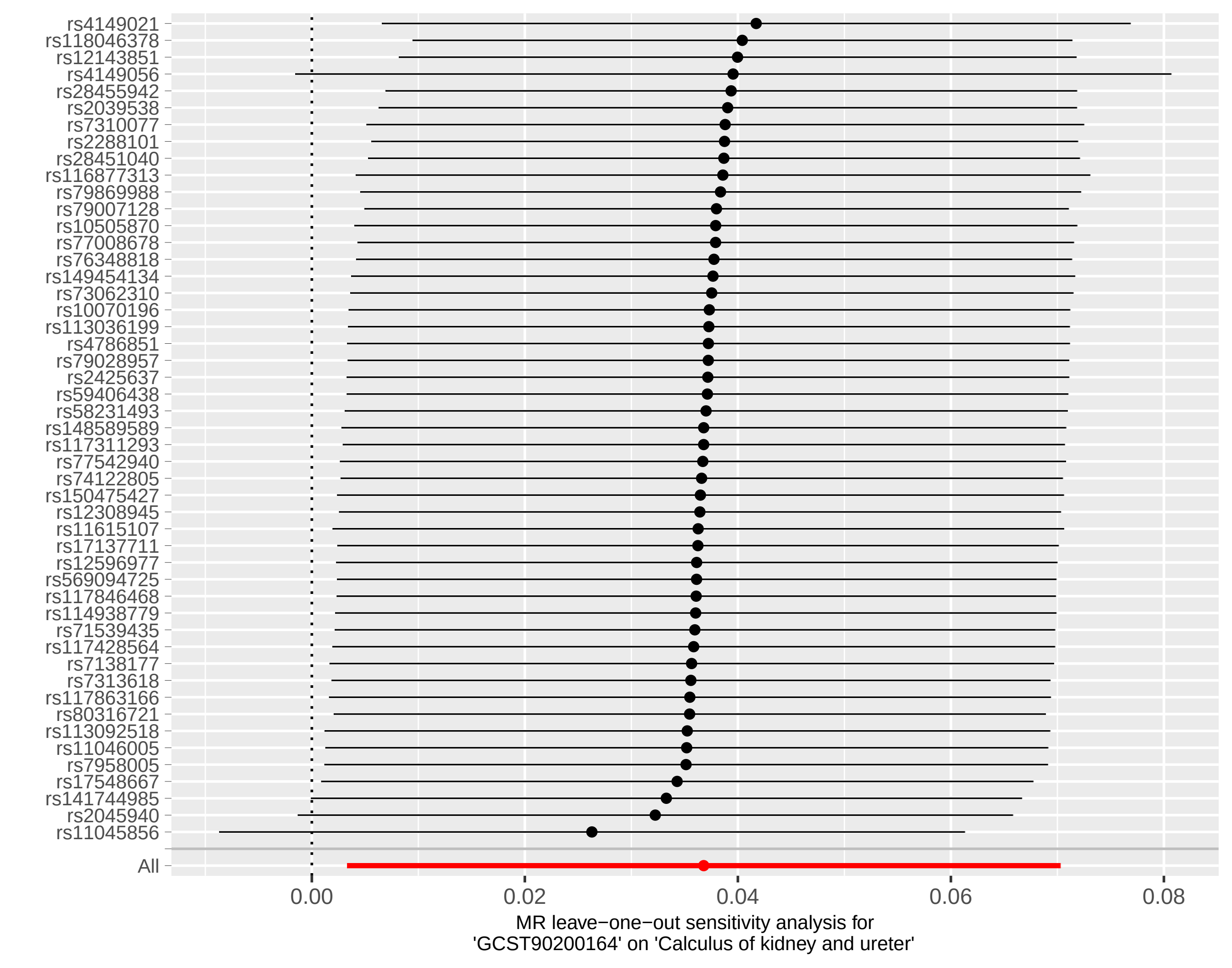
rs4149021
rs118046378
rs12143851
rs4149056
rs28455942
rs2039538
rs7310077
rs2288101
rs28451040
rs116877313
rs79869988
rs79007128
rs10505870
rs77008678
rs76348818
rs149454134
rs73062310
rs10070196
rs113036199
rs4786851
rs79028957
rs2425637
rs59406438
rs58231493
rs148589589
rs117311293
rs77542940
rs74122805
rs150475427
rs12308945
rs11615107
rs17137711
rs12596977
rs569094725
rs117846468
rs114938779
rs71539435
rs117428564
rs7138177
rs7313618
rs117863166
rs80316721
rs113092518
rs11046005
rs7958005
rs17548667
rs141744985
rs2045940
rs11045856
All
0.00
0.02
0.04
0.06
0.08
MR leave−one−out sensitivity analysis for
'GCST90200164' on 'Calculus of kidney and ureter'

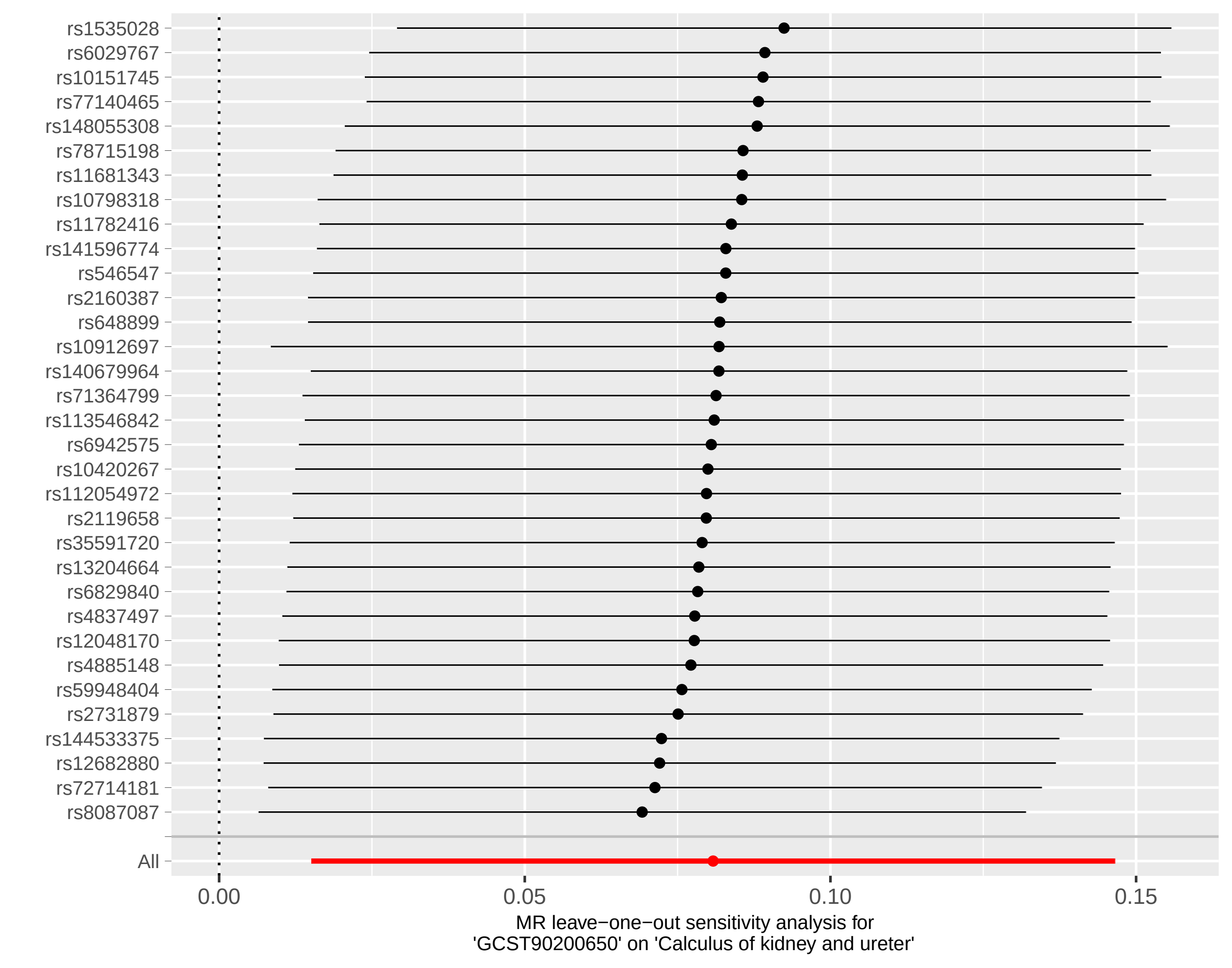
rs1535028
rs6029767
rs10151745
rs77140465
rs148055308
rs78715198
rs11681343
rs10798318
rs11782416
rs141596774
rs546547
rs2160387
rs648899
rs10912697
rs140679964
rs71364799
rs113546842
rs6942575
rs10420267
rs112054972
rs2119658
rs35591720
rs13204664
rs6829840
rs4837497
rs12048170
rs4885148
rs59948404
rs2731879
rs144533375
rs12682880
rs72714181
rs8087087
All
0.00
0.05
0.10
0.15
MR leave−one−out sensitivity analysis for
'GCST90200650' on 'Calculus of kidney and ureter'

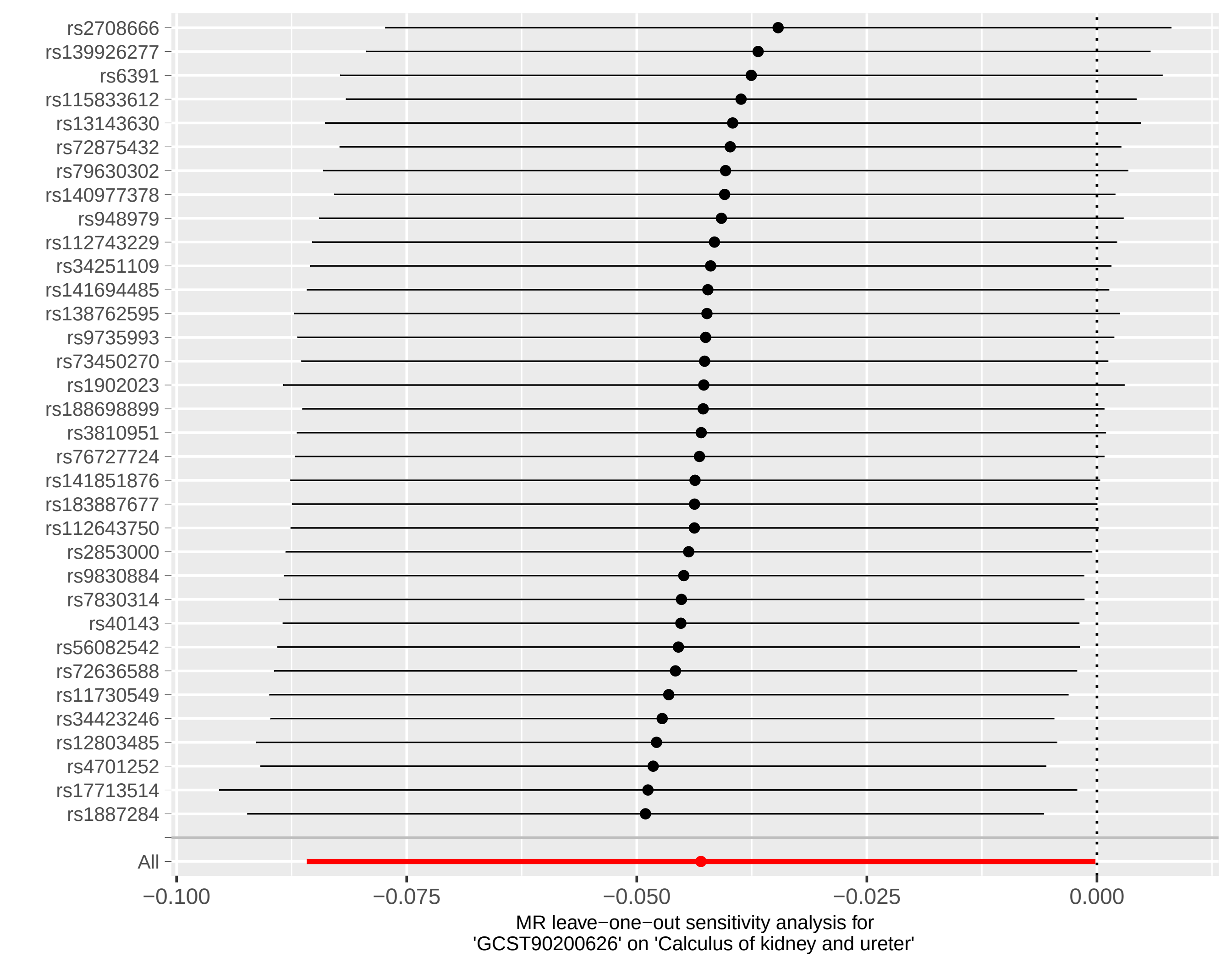
rs2708666
rs139926277
rs6391
rs115833612
rs13143630
rs72875432
rs79630302
rs140977378
rs948979
rs112743229
rs34251109
rs141694485
rs138762595
rs9735993
rs73450270
rs1902023
rs188698899
rs3810951
rs76727724
rs141851876
rs183887677
rs112643750
rs2853000
rs9830884
rs7830314
rs40143
rs56082542
rs72636588
rs11730549
rs34423246
rs12803485
rs4701252
rs17713514
rs1887284
All
−0.100
−0.075
−0.050
−0.025
0.000
MR leave−one−out sensitivity analysis for
'GCST90200626' on 'Calculus of kidney and ureter'

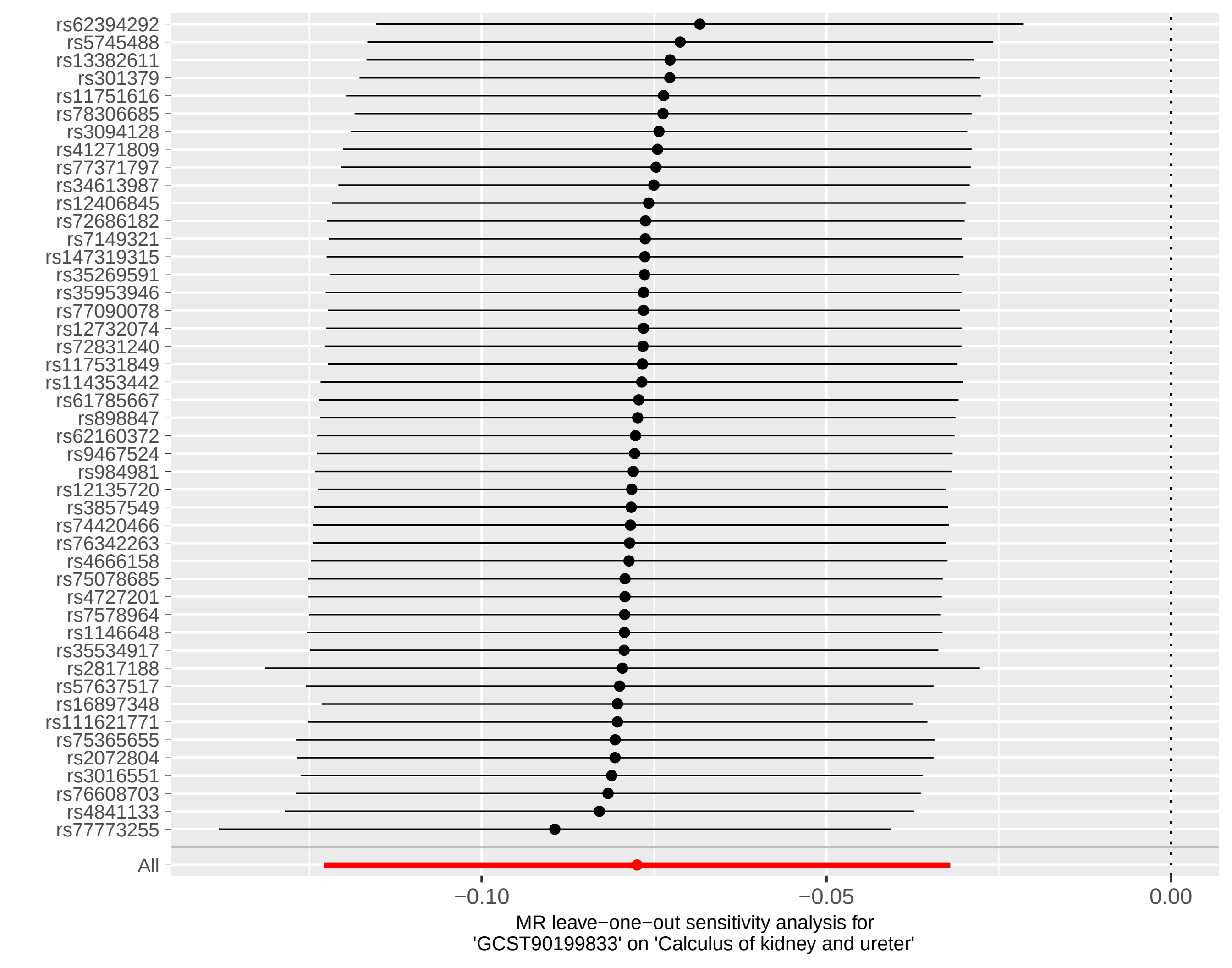
rs62394292
rs5745488
rs13382611
rs301379
rs11751616
rs78306685
rs3094128
rs41271809
rs77371797
rs34613987
rs12406845
rs72686182
rs7149321
rs147319315
rs35269591
rs35953946
rs77090078
rs12732074
rs72831240
rs117531849
rs114353442
rs61785667
rs898847
rs62160372
rs9467524
rs984981
rs12135720
rs3857549
rs74420466
rs76342263
rs4666158
rs75078685
rs4727201
rs7578964
rs1146648
rs35534917
rs2817188
rs57637517
rs16897348
rs111621771
rs75365655
rs2072804
rs3016551
rs76608703
rs4841133
rs77773255
All
−0.10
−0.05
0.00
MR leave−one−out sensitivity analysis for
'GCST90199833' on 'Calculus of kidney and ureter'

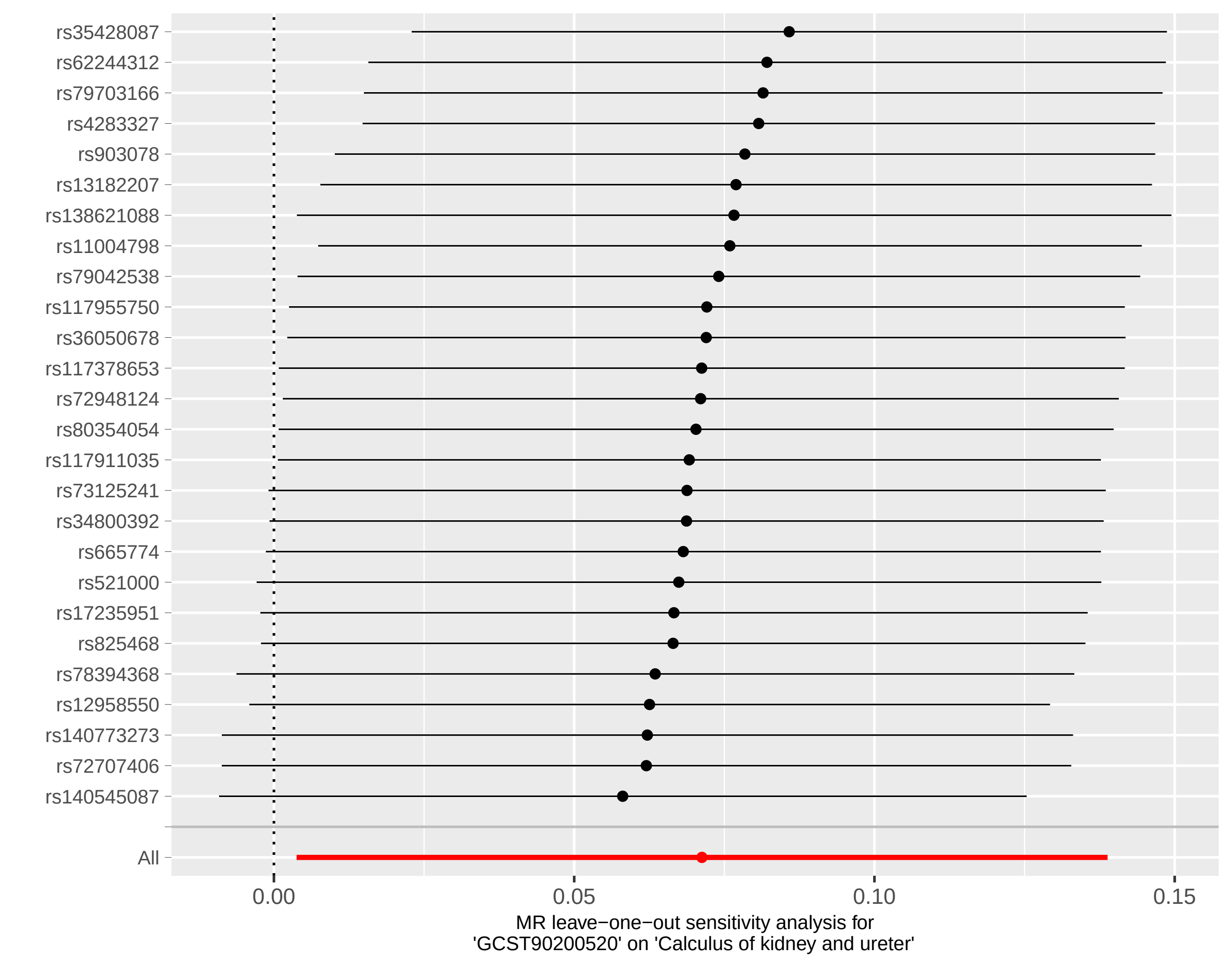
rs35428087
rs62244312
rs79703166
rs4283327
rs903078
rs13182207
rs138621088
rs11004798
rs79042538
rs117955750
rs36050678
rs117378653
rs72948124
rs80354054
rs117911035
rs73125241
rs34800392
rs665774
rs521000
rs17235951
rs825468
rs78394368
rs12958550
rs140773273
rs72707406
rs140545087
All
0.00
0.05
0.10
0.15
MR leave−one−out sensitivity analysis for
'GCST90200520' on 'Calculus of kidney and ureter'

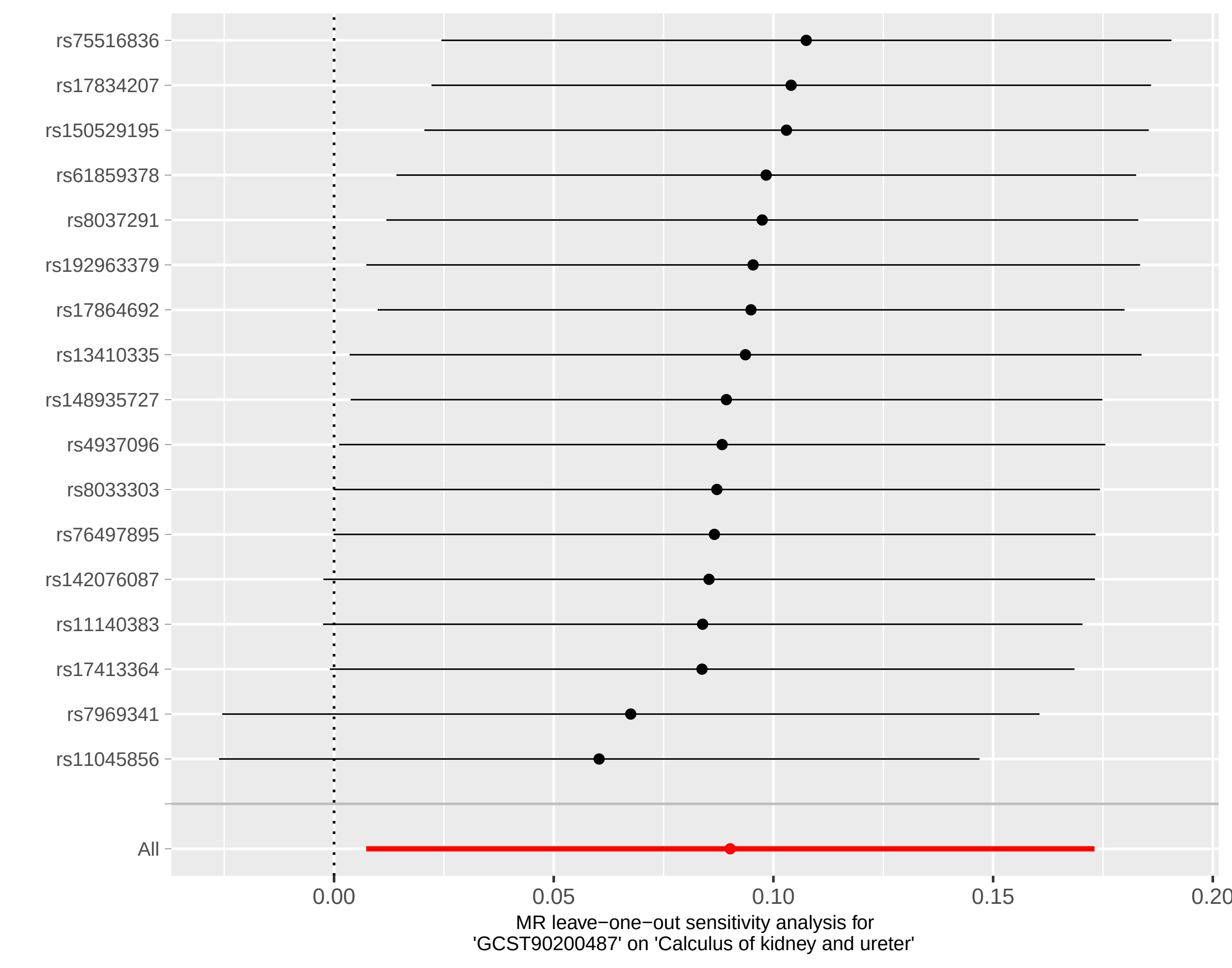
rs75516836
rs17834207
rs150529195
rs61859378
rs8037291
rs192963379
rs17864692
rs13410335
rs148935727
rs4937096
rs8033303
rs76497895
rs142076087
rs11140383
rs17413364
rs7969341
rs11045856
All
0.00
0.05
0.10
0.15
0.20
MR leave−one−out sensitivity analysis for
'GCST90200487' on 'Calculus of kidney and ureter'

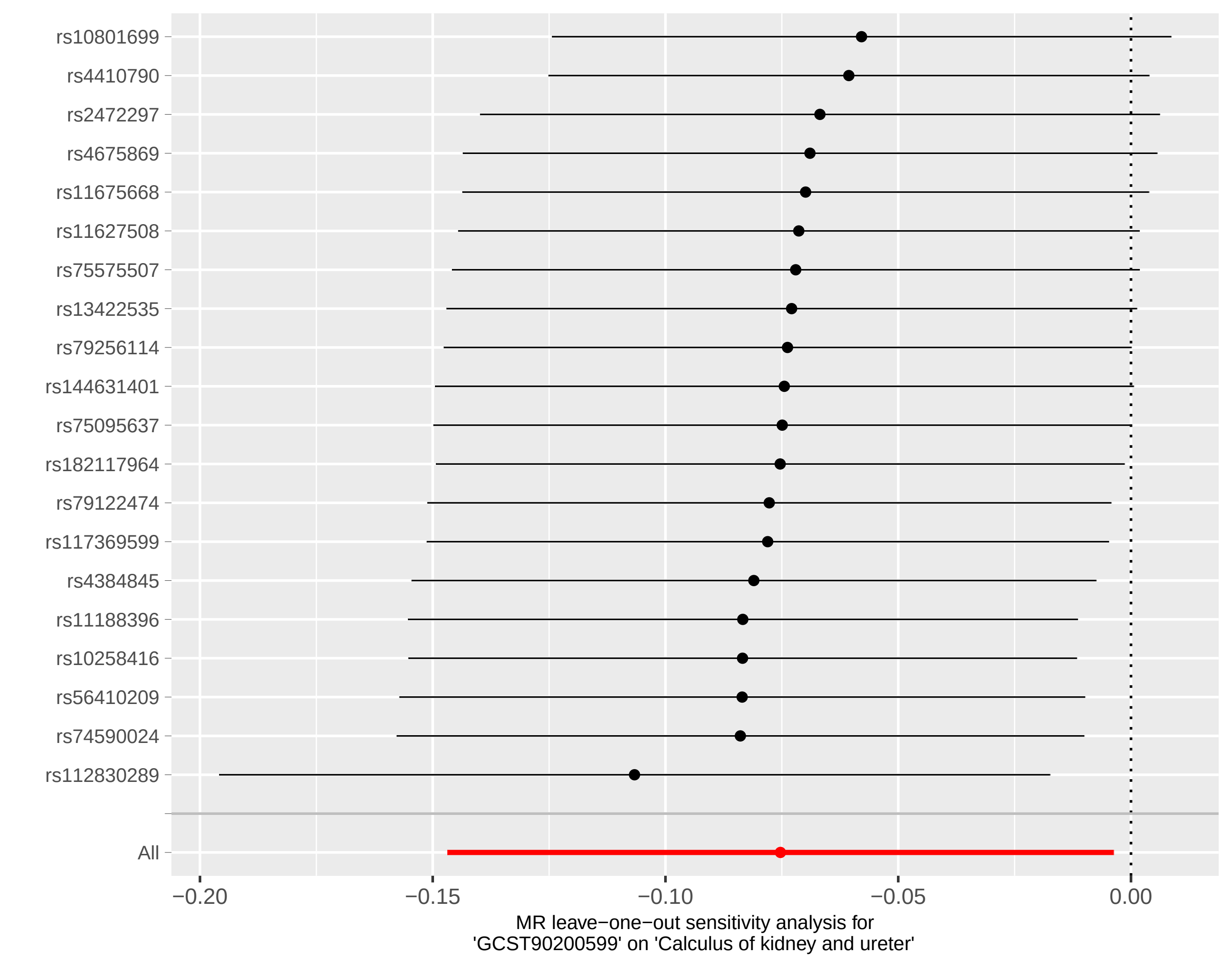
rs10801699
rs4410790
rs2472297
rs4675869
rs11675668
rs11627508
rs75575507
rs13422535
rs79256114
rs144631401
rs75095637
rs182117964
rs79122474
rs117369599
rs4384845
rs11188396
rs10258416
rs56410209
rs74590024
rs112830289
All
−0.20
−0.15
−0.10
−0.05
0.00
MR leave−one−out sensitivity analysis for
'GCST90200599' on 'Calculus of kidney and ureter'

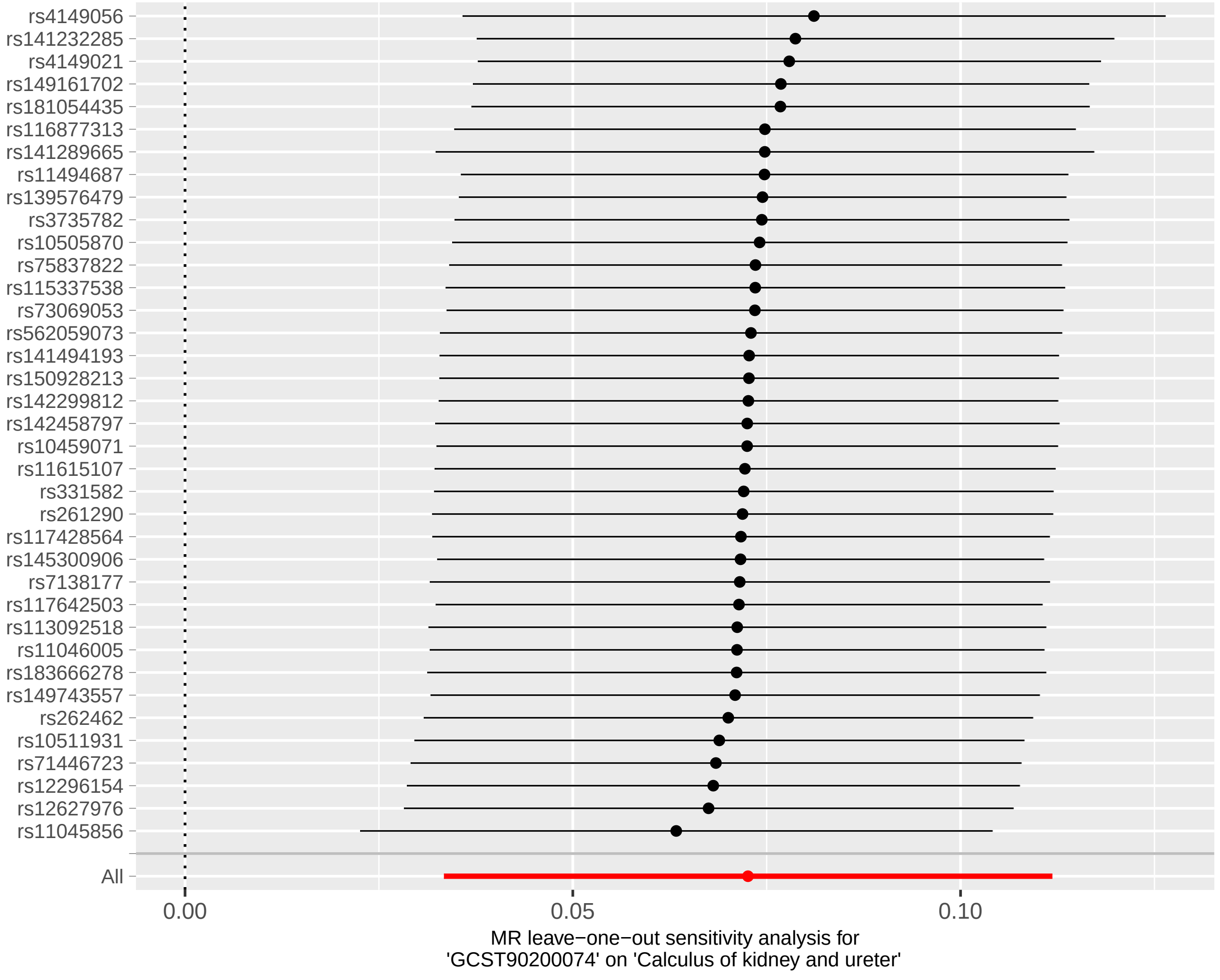
rs4149056
rs141232285
rs4149021
rs149161702
rs181054435
rs116877313
rs141289665
rs11494687
rs139576479
rs3735782
rs10505870
rs75837822
rs115337538
rs73069053
rs562059073
rs141494193
rs150928213
rs142299812
rs142458797
rs10459071
rs11615107
rs331582
rs261290
rs117428564
rs145300906
rs7138177
rs117642503
rs113092518
rs11046005
rs183666278
rs149743557
rs262462
rs10511931
rs71446723
rs12296154
rs12627976
rs11045856
All
0.00
0.05
0.10
MR leave−one−out sensitivity analysis for
'GCST90200074' on 'Calculus of kidney and ureter'

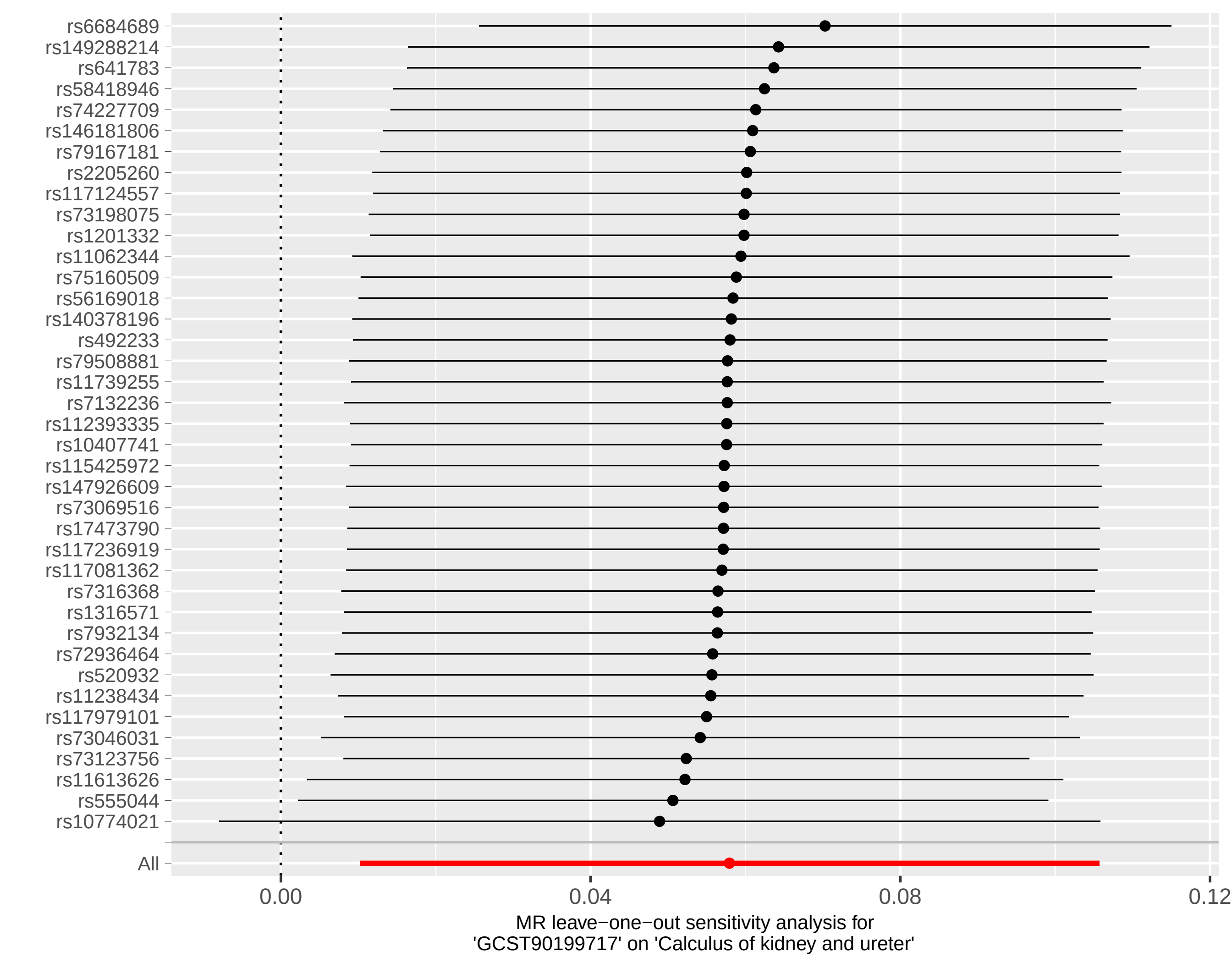

rs6684689
rs149288214
rs641783
rs58418946
rs74227709
rs146181806
rs79167181
rs2205260
rs117124557
rs73198075
rs1201332
rs11062344
rs75160509
rs56169018
rs140378196
rs492233
rs79508881
rs11739255
rs7132236
rs112393335
rs10407741
rs115425972
rs147926609
rs73069516
rs17473790
rs117236919
rs117081362
rs7316368
rs1316571
rs7932134
rs72936464
rs520932
rs11238434
rs117979101
rs73046031
rs73123756
rs11613626
rs555044
rs10774021
All
0.00
0.04
0.08
0.12
MR leave−one−out sensitivity analysis for
'GCST90199717' on 'Calculus of kidney and ureter'

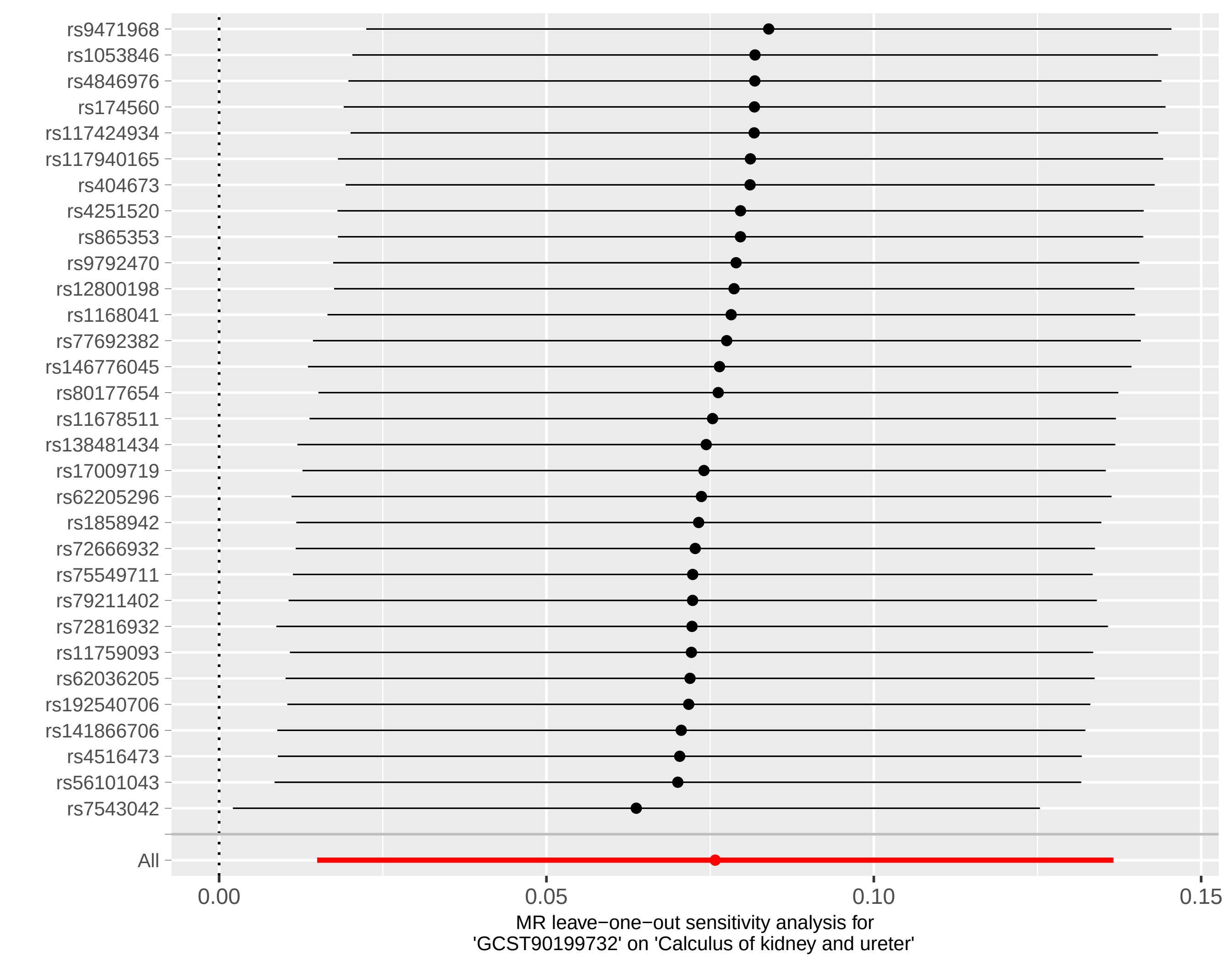

rs9471968
rs1053846
rs4846976
rs174560
rs117424934
rs117940165
rs404673
rs4251520
rs865353
rs9792470
rs12800198
rs1168041
rs77692382
rs146776045
rs80177654
rs11678511
rs138481434
rs17009719
rs62205296
rs1858942
rs72666932
rs75549711
rs79211402
rs72816932
rs11759093
rs62036205
rs192540706
rs141866706
rs4516473
rs56101043
rs7543042
All
0.00
0.05
0.10
0.15
MR leave−one−out sensitivity analysis for
'GCST90199732' on 'Calculus of kidney and ureter'

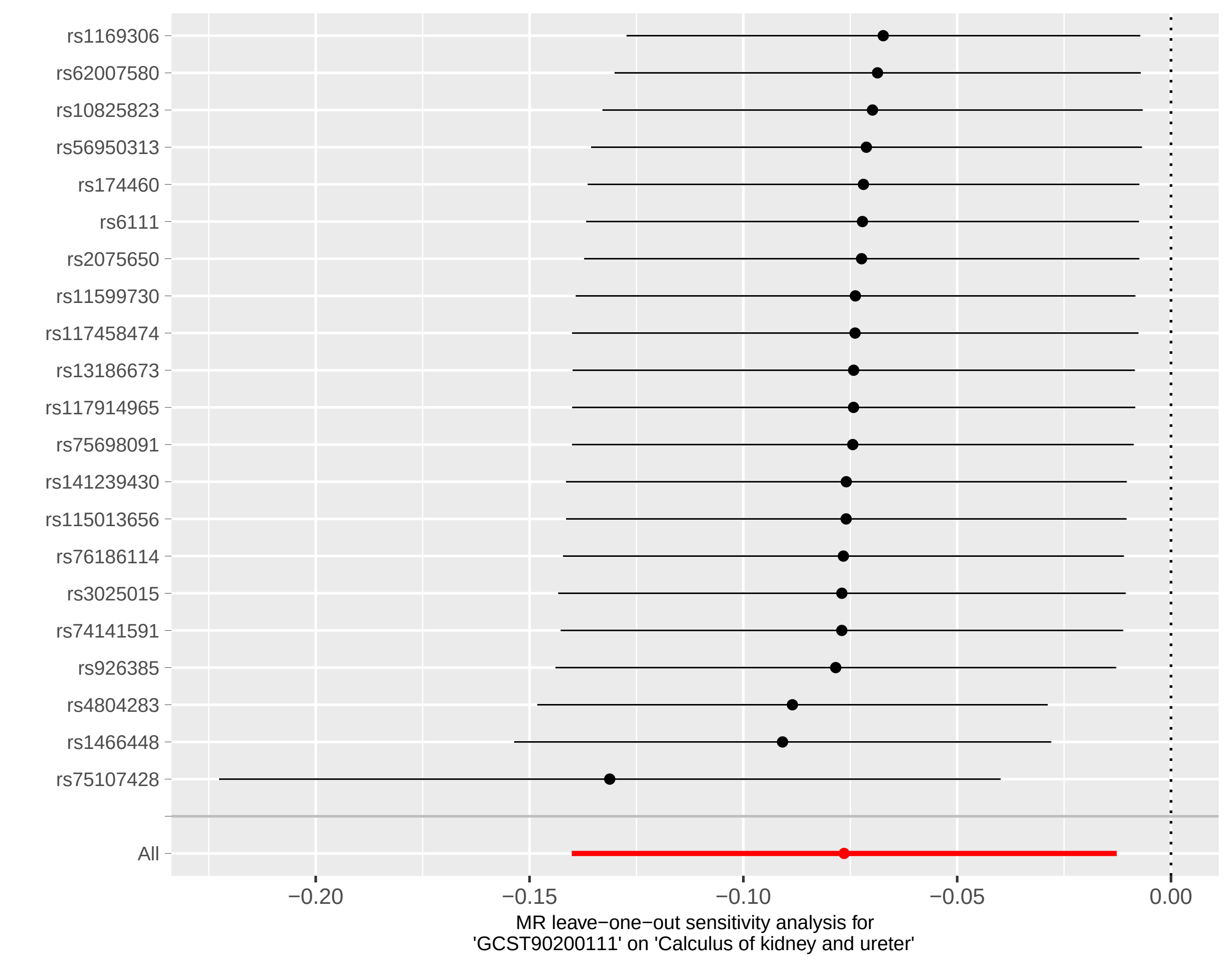

rs1169306
rs62007580
rs10825823
rs56950313
rs174460
rs6111
rs2075650
rs11599730
rs117458474
rs13186673
rs117914965
rs75698091
rs141239430
rs115013656
rs76186114
rs3025015
rs74141591
rs926385
rs4804283
rs1466448
rs75107428
All
−0.20
−0.15
−0.10
−0.05
0.00
MR leave−one−out sensitivity analysis for
'GCST90200111' on 'Calculus of kidney and ureter'

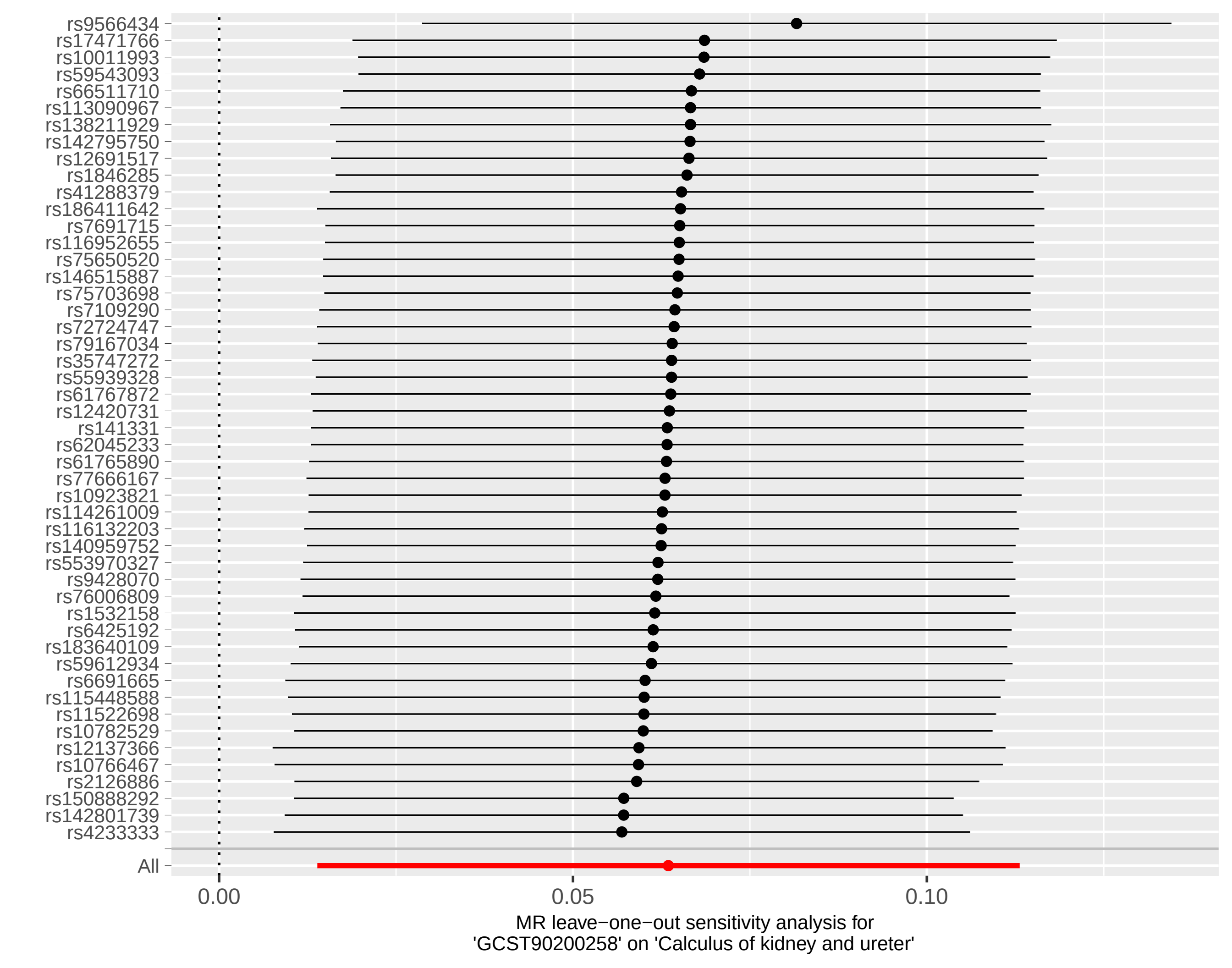
rs9566434
rs17471766
rs10011993
rs59543093
rs66511710
rs113090967
rs138211929
rs142795750
rs12691517
rs1846285
rs41288379
rs186411642
rs7691715
rs116952655
rs75650520
rs146515887
rs75703698
rs7109290
rs72724747
rs79167034
rs35747272
rs55939328
rs61767872
rs12420731
rs141331
rs62045233
rs61765890
rs77666167
rs10923821
rs114261009
rs116132203
rs140959752
rs553970327
rs9428070
rs76006809
rs1532158
rs6425192
rs183640109
rs59612934
rs6691665
rs115448588
rs11522698
rs10782529
rs12137366
rs10766467
rs2126886
rs150888292
rs142801739
rs4233333
All
0.00
0.05
0.10
MR leave−one−out sensitivity analysis for
'GCST90200258' on 'Calculus of kidney and ureter'

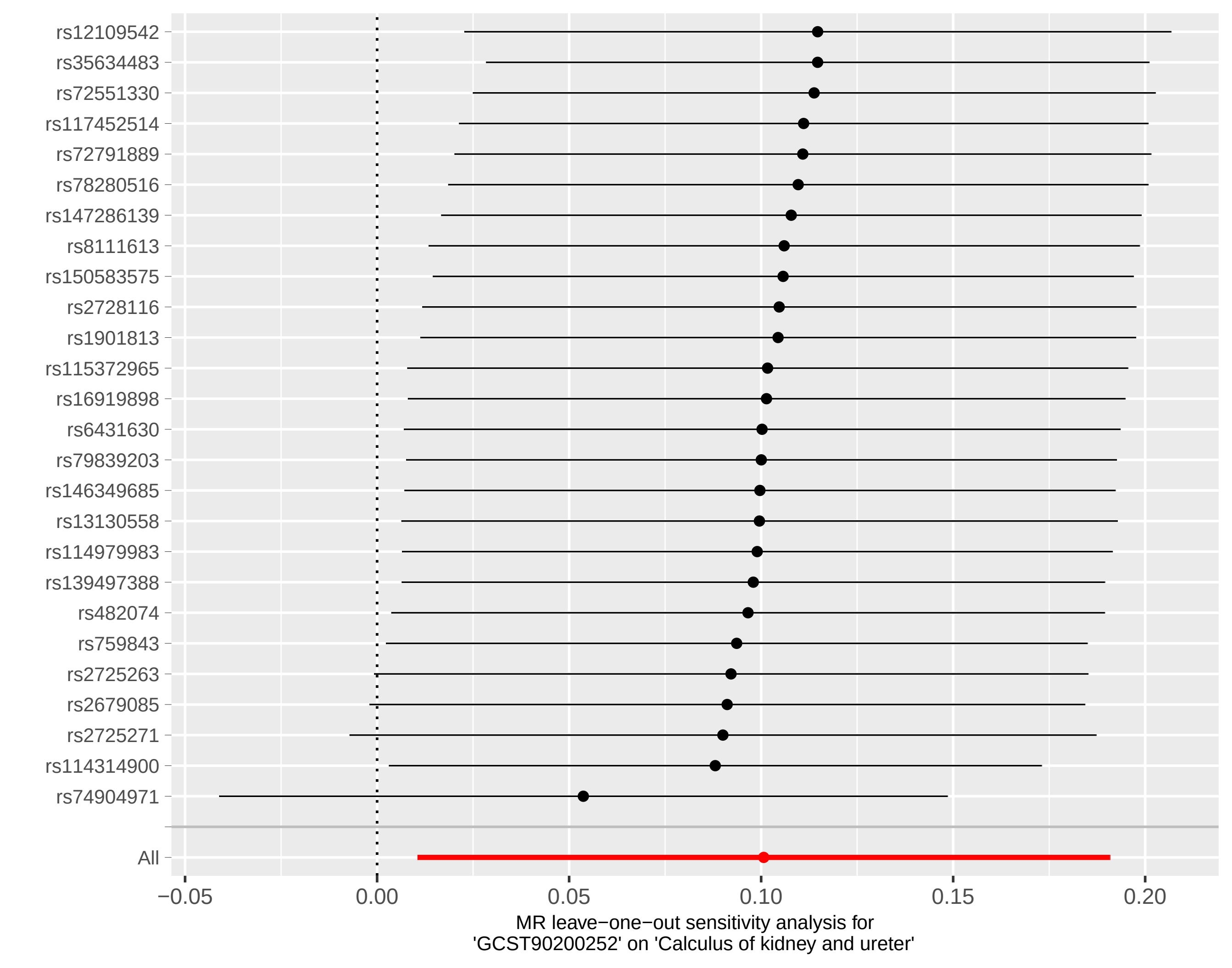
rs12109542
rs35634483
rs72551330
rs117452514
rs72791889
rs78280516
rs147286139
rs8111613
rs150583575
rs2728116
rs1901813
rs115372965
rs16919898
rs6431630
rs79839203
rs146349685
rs13130558
rs114979983
rs139497388
rs482074
rs759843
rs2725263
rs2679085
rs2725271
rs114314900
rs74904971
All
−0.05
0.00
0.05
0.10
0.15
0.20
MR leave−one−out sensitivity analysis for
'GCST90200252' on 'Calculus of kidney and ureter'

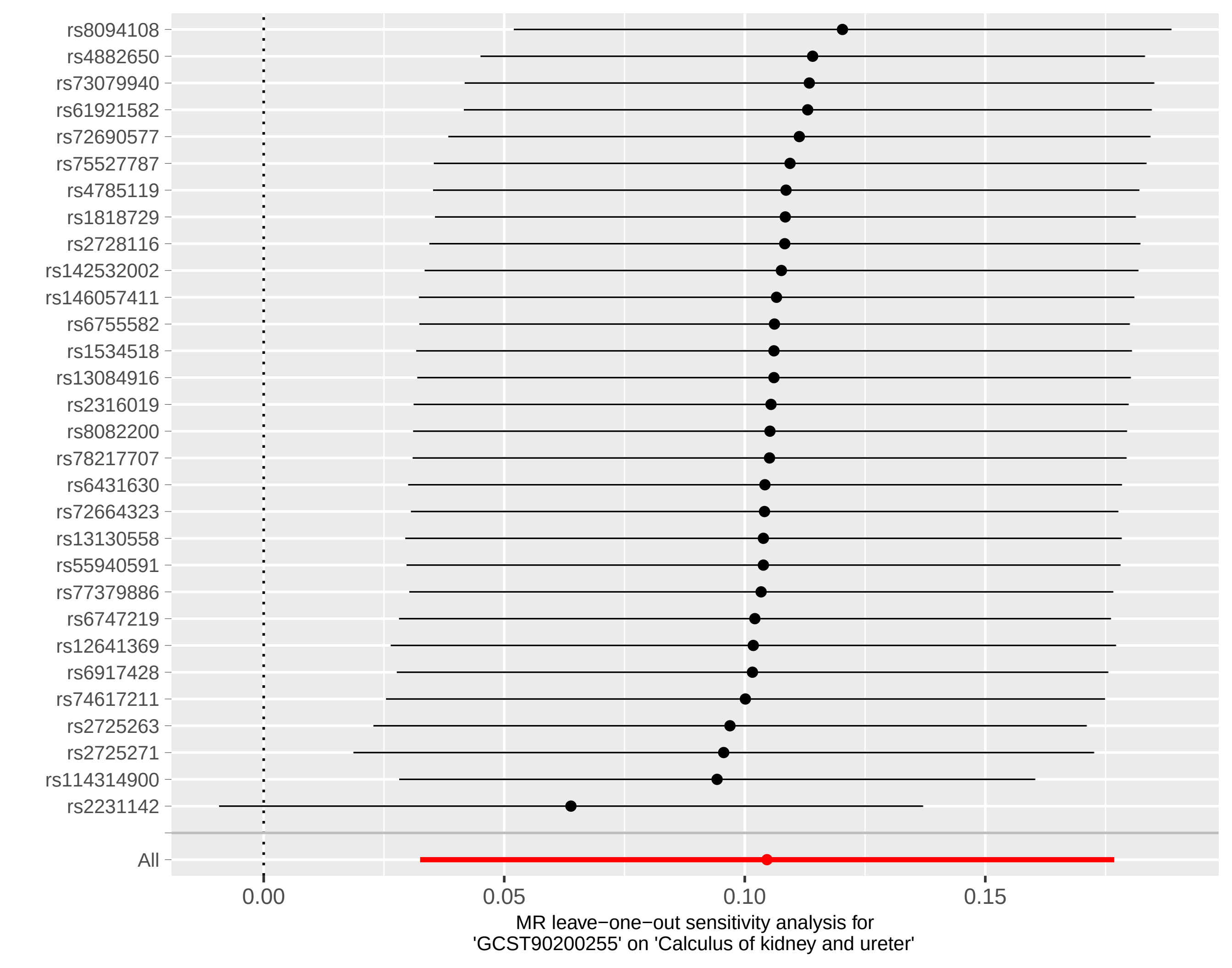
rs8094108
rs4882650
rs73079940
rs61921582
rs72690577
rs75527787
rs4785119
rs1818729
rs2728116
rs142532002
rs146057411
rs6755582
rs1534518
rs13084916
rs2316019
rs8082200
rs78217707
rs6431630
rs72664323
rs13130558
rs55940591
rs77379886
rs6747219
rs12641369
rs6917428
rs74617211
rs2725263
rs2725271
rs114314900
rs2231142
All
0.00
0.05
0.10
0.15
MR leave−one−out sensitivity analysis for
'GCST90200255' on 'Calculus of kidney and ureter'

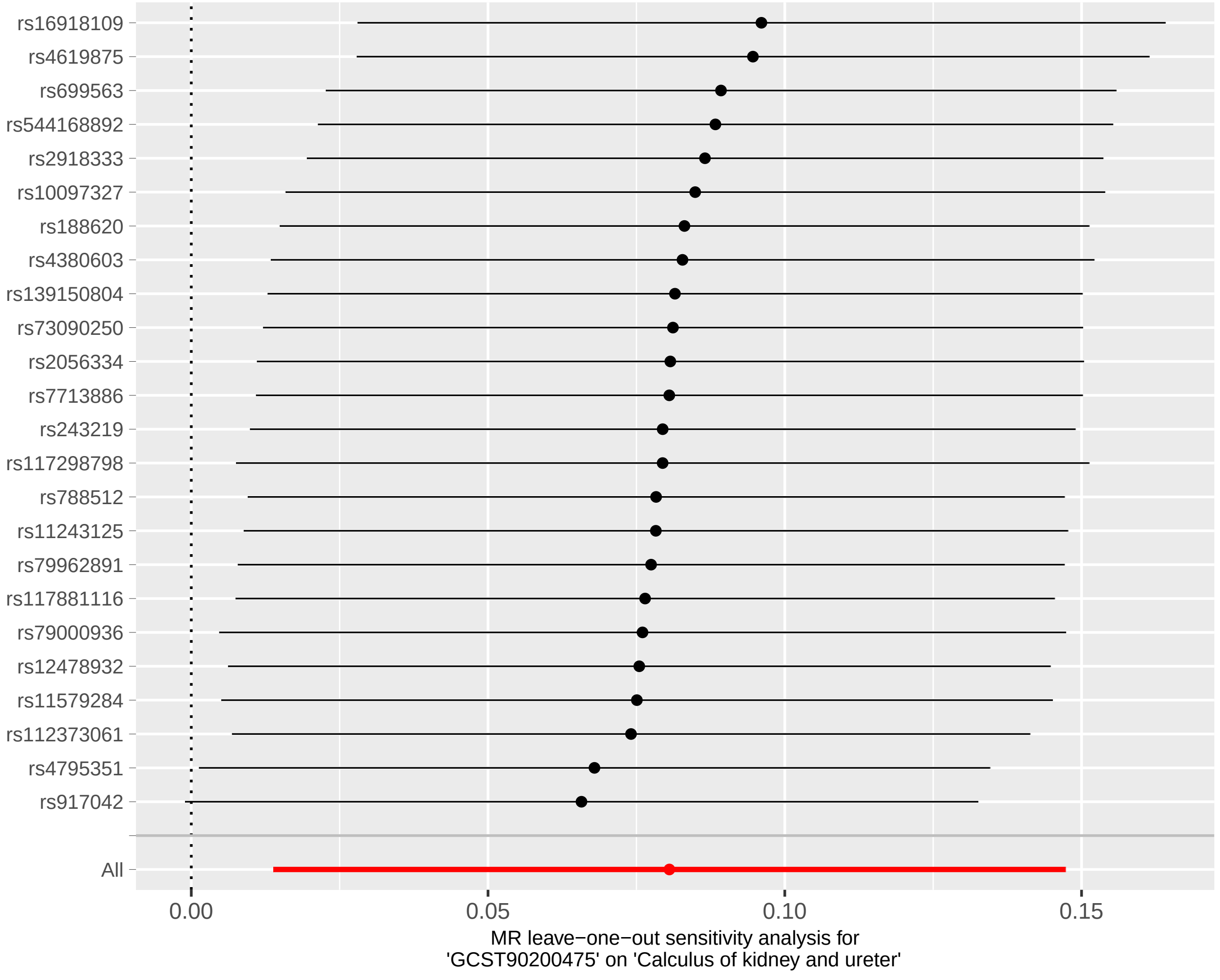
rs16918109
rs4619875
rs699563
rs544168892
rs2918333
rs10097327
rs188620
rs4380603
rs139150804
rs73090250
rs2056334
rs7713886
rs243219
rs117298798
rs788512
rs11243125
rs79962891
rs117881116
rs79000936
rs12478932
rs11579284
rs112373061
rs4795351
rs917042
All
0.00
0.05
0.10
0.15
MR leave−one−out sensitivity analysis for
'GCST90200475' on 'Calculus of kidney and ureter'

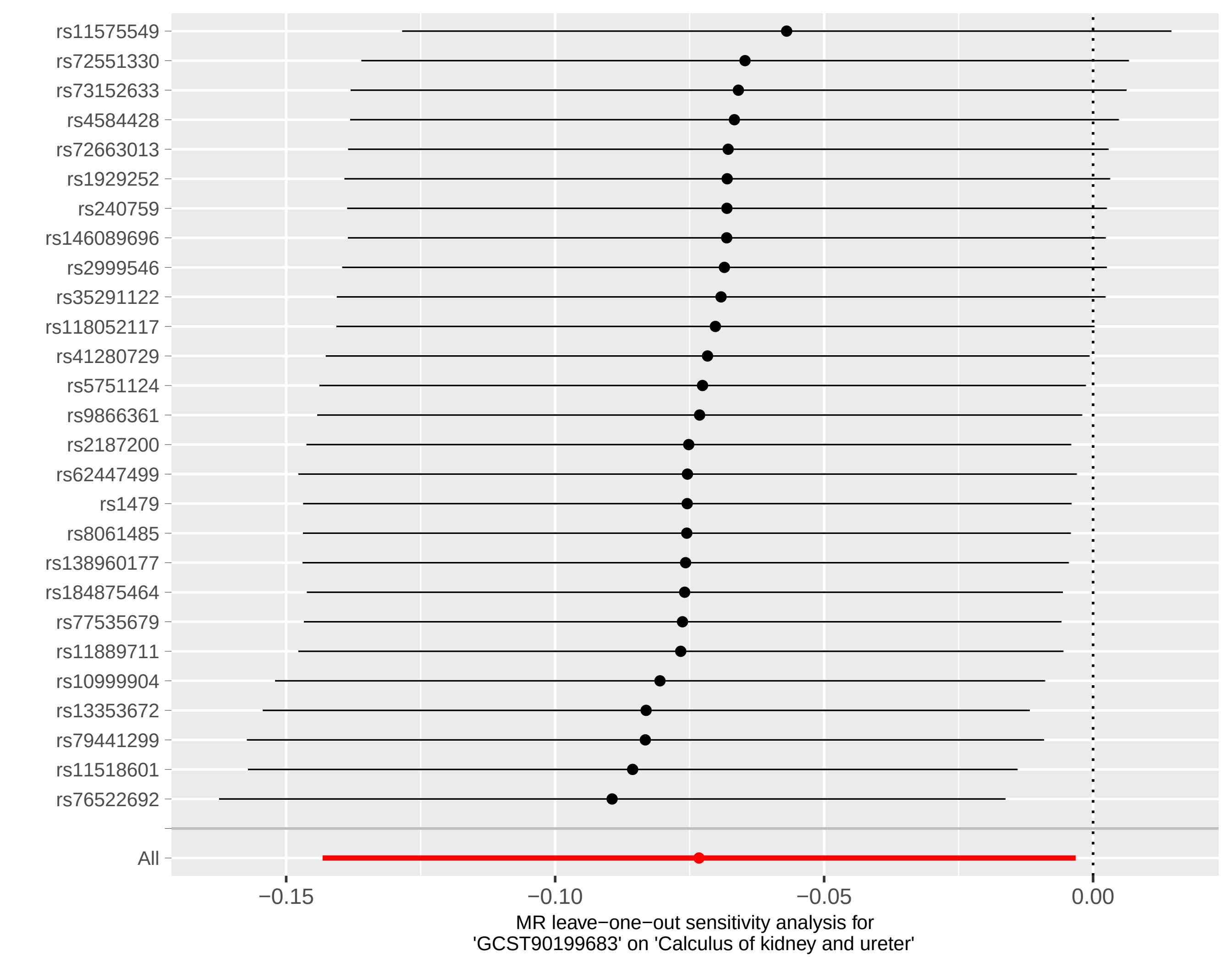

rs11575549
rs72551330
rs73152633
rs4584428
rs72663013
rs1929252
rs240759
rs146089696
rs2999546
rs35291122
rs118052117
rs41280729
rs5751124
rs9866361
rs2187200
rs62447499
rs1479
rs8061485
rs138960177
rs184875464
rs77535679
rs11889711
rs10999904
rs13353672
rs79441299
rs11518601
rs76522692
All
−0.15
−0.10
−0.05
0.00
MR leave−one−out sensitivity analysis for
'GCST90199683' on 'Calculus of kidney and ureter'

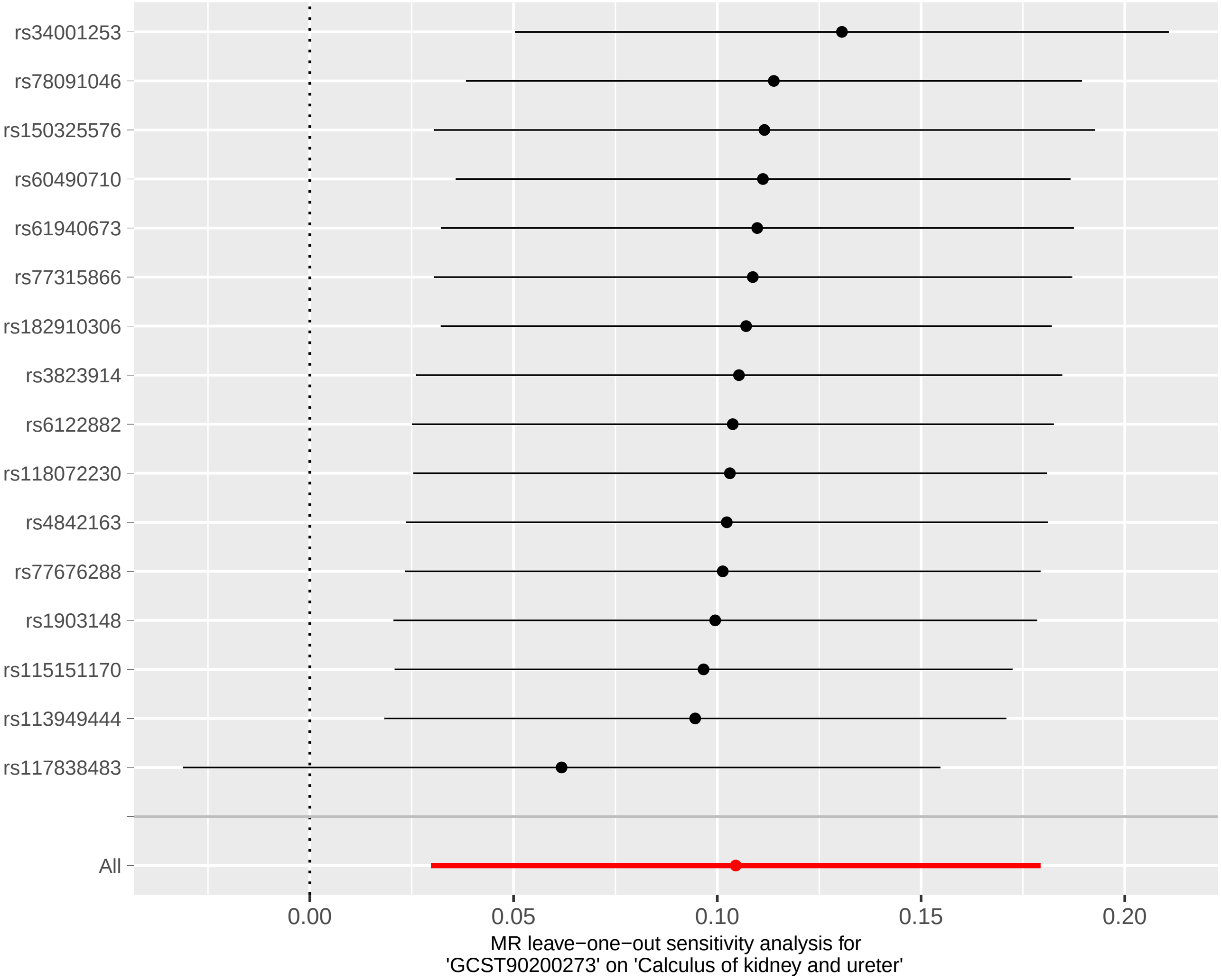

rs34001253
rs78091046
rs150325576
rs60490710
rs61940673
rs77315866
rs182910306
rs3823914
rs6122882
rs118072230
rs4842163
rs77676288
rs1903148
rs115151170
rs113949444
rs117838483
All
0.00
0.05
0.10
0.15
0.20
MR leave−one−out sensitivity analysis for
'GCST90200273' on 'Calculus of kidney and ureter'

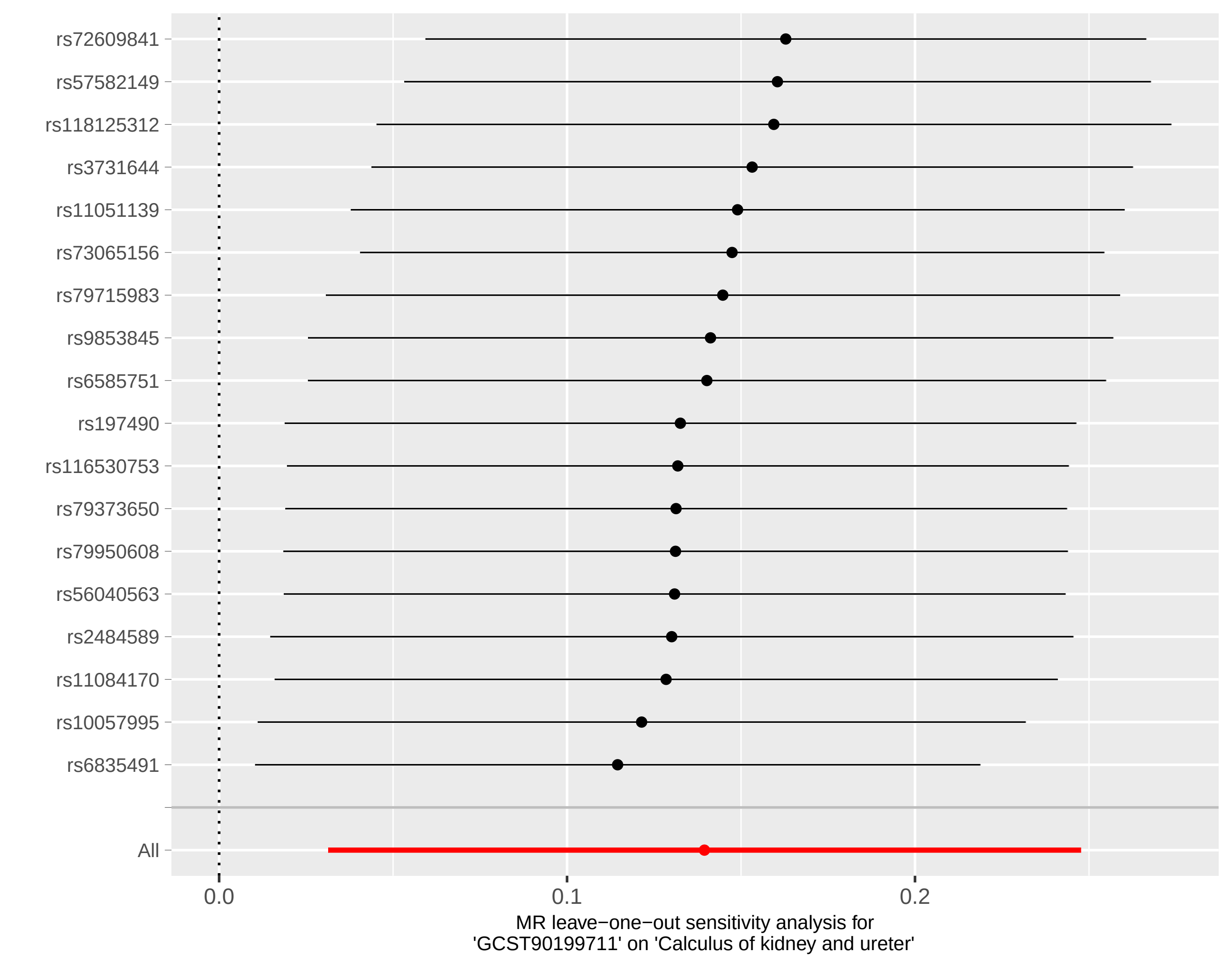
rs72609841
rs57582149
rs118125312
rs3731644
rs11051139
rs73065156
rs79715983
rs9853845
rs6585751
rs197490
rs116530753
rs79373650
rs79950608
rs56040563
rs2484589
rs11084170
rs10057995
rs6835491
All
0.0
0.1
0.2
MR leave−one−out sensitivity analysis for
'GCST90199711' on 'Calculus of kidney and ureter'

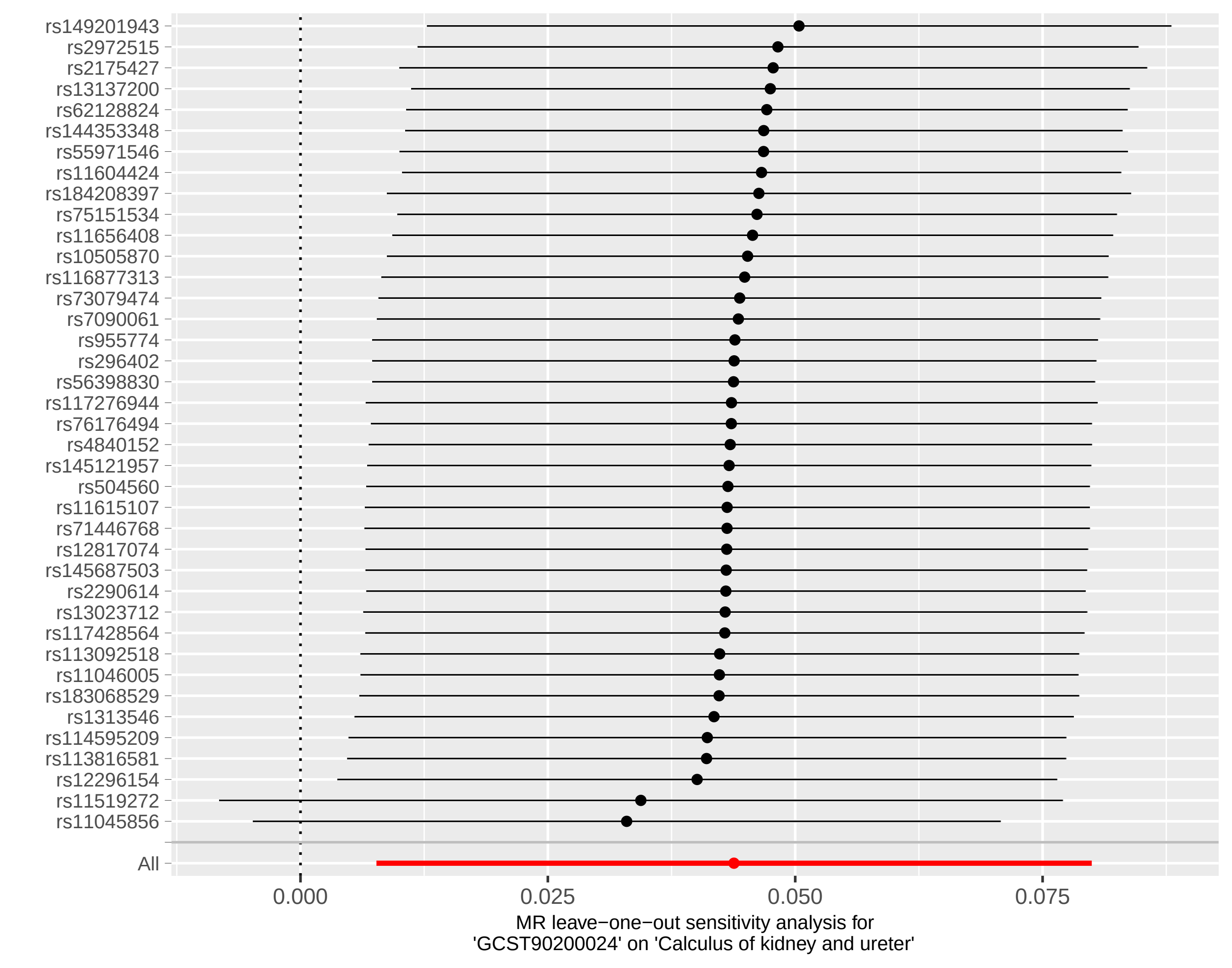
rs149201943
rs2972515
rs2175427
rs13137200
rs62128824
rs144353348
rs55971546
rs11604424
rs184208397
rs75151534
rs11656408
rs10505870
rs116877313
rs73079474
rs7090061
rs955774
rs296402
rs56398830
rs117276944
rs76176494
rs4840152
rs145121957
rs504560
rs11615107
rs71446768
rs12817074
rs145687503
rs2290614
rs13023712
rs117428564
rs113092518
rs11046005
rs183068529
rs1313546
rs114595209
rs113816581
rs12296154
rs11519272
rs11045856
All
0.000
0.025
0.050
0.075
MR leave−one−out sensitivity analysis for
'GCST90200024' on 'Calculus of kidney and ureter'

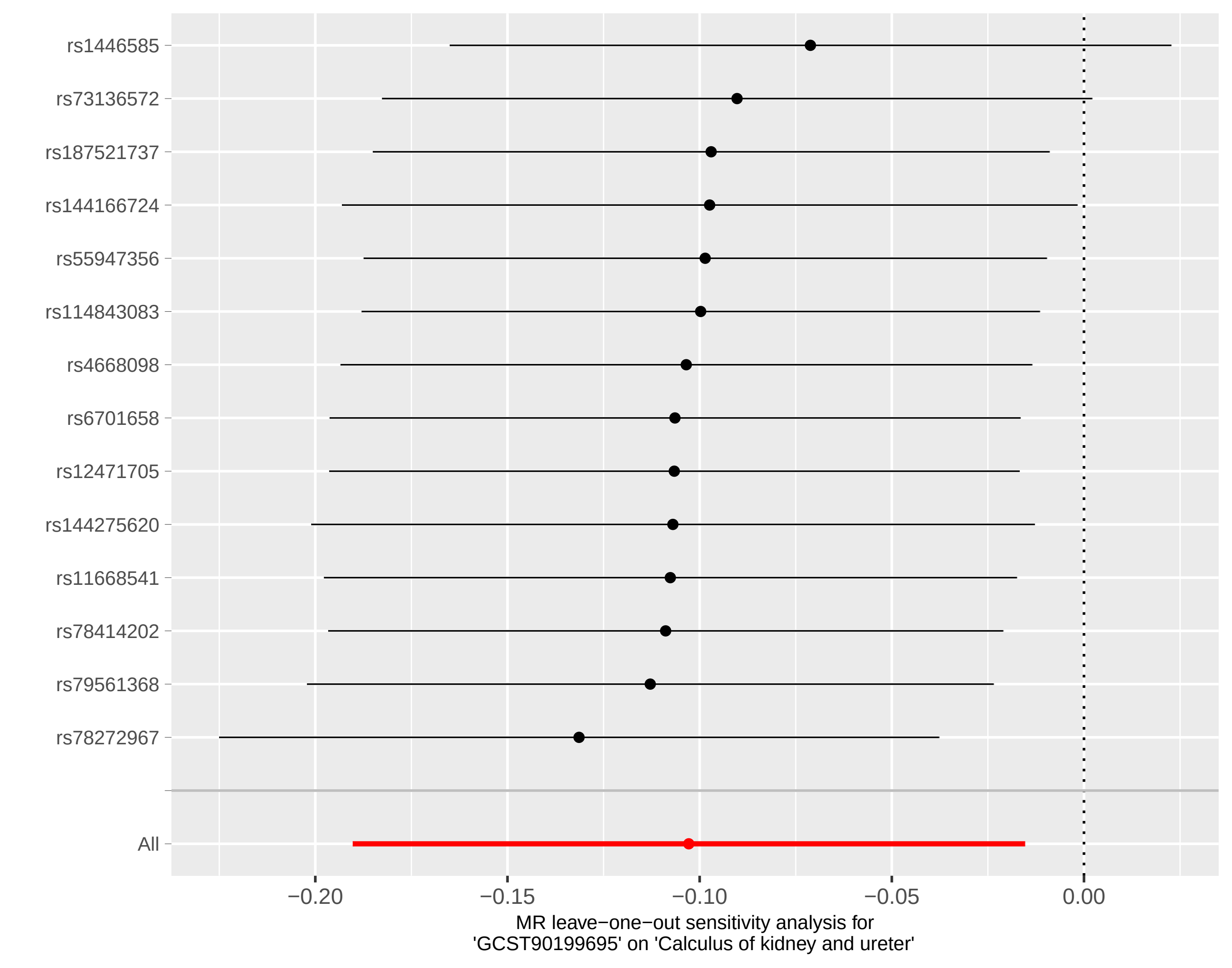
rs1446585
rs73136572
rs187521737
rs144166724
rs55947356
rs114843083
rs4668098
rs6701658
rs12471705
rs144275620
rs11668541
rs78414202
rs79561368
rs78272967
All
−0.20
−0.15
−0.10
−0.05
0.00
MR leave−one−out sensitivity analysis for
'GCST90199695' on 'Calculus of kidney and ureter'

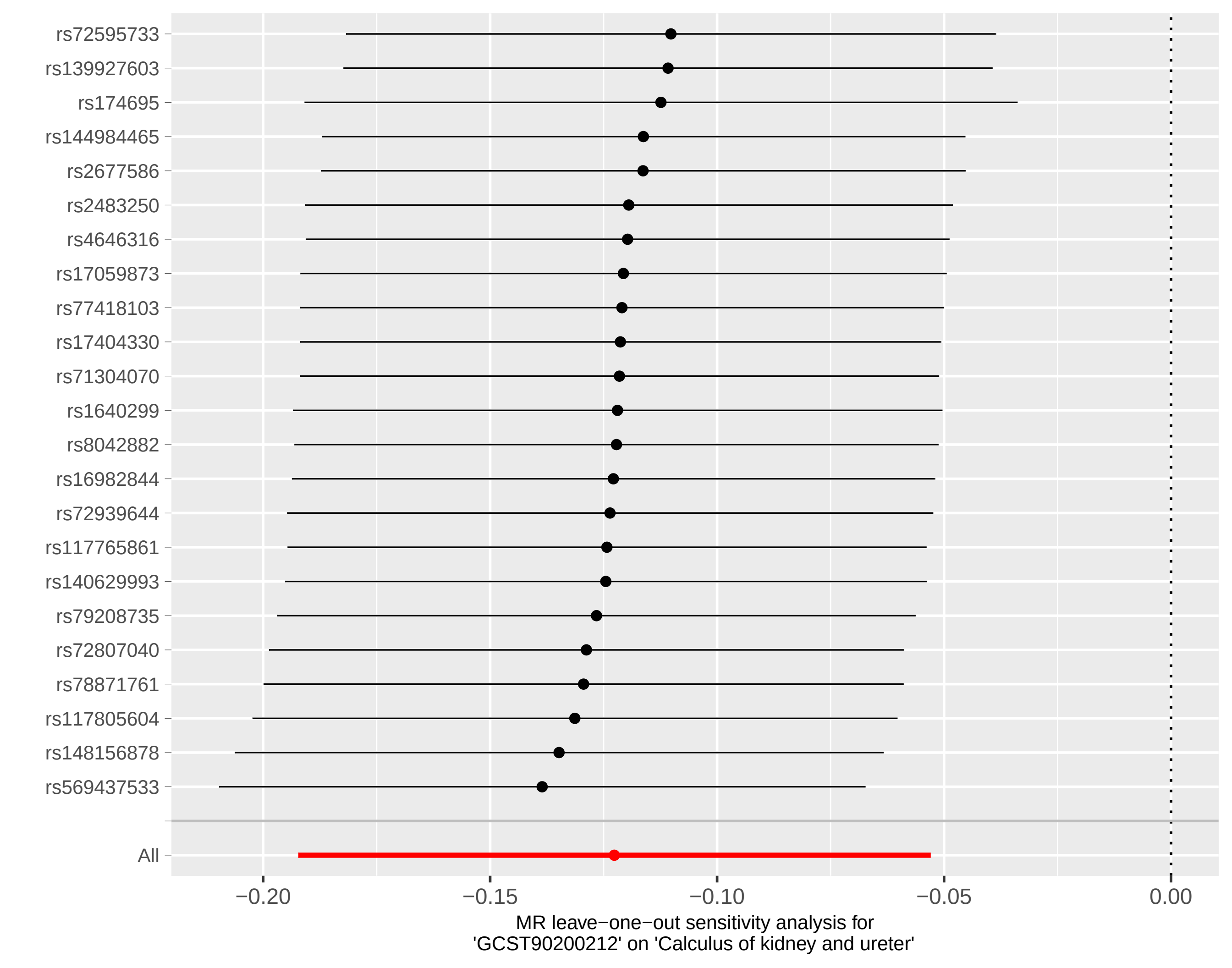

rs72595733
rs139927603
rs174695
rs144984465
rs2677586
rs2483250
rs4646316
rs17059873
rs77418103
rs17404330
rs71304070
rs1640299
rs8042882
rs16982844
rs72939644
rs117765861
rs140629993
rs79208735
rs72807040
rs78871761
rs117805604
rs148156878
rs569437533
All
−0.20
−0.15
−0.10
−0.05
0.00
MR leave−one−out sensitivity analysis for
'GCST90200212' on 'Calculus of kidney and ureter'

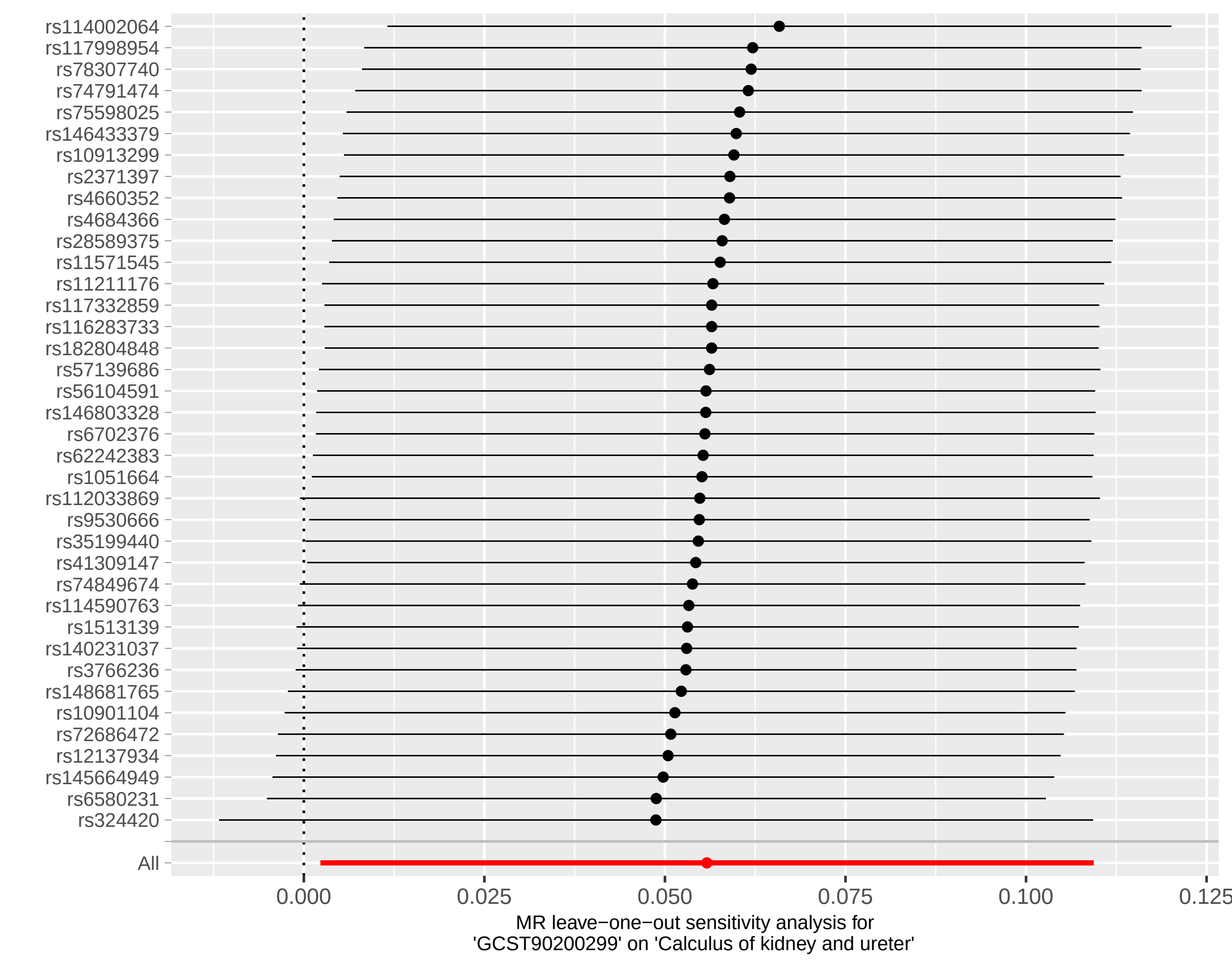
rs114002064
rs117998954
rs78307740
rs74791474
rs75598025
rs146433379
rs10913299
rs2371397
rs4660352
rs4684366
rs28589375
rs11571545
rs11211176
rs117332859
rs116283733
rs182804848
rs57139686
rs56104591
rs146803328
rs6702376
rs62242383
rs1051664
rs112033869
rs9530666
rs35199440
rs41309147
rs74849674
rs114590763
rs1513139
rs140231037
rs3766236
rs148681765
rs10901104
rs72686472
rs12137934
rs145664949
rs6580231
rs324420
All
0.000
0.025
0.050
0.075
0.100
0.125
MR leave−one−out sensitivity analysis for
'GCST90200299' on 'Calculus of kidney and ureter'

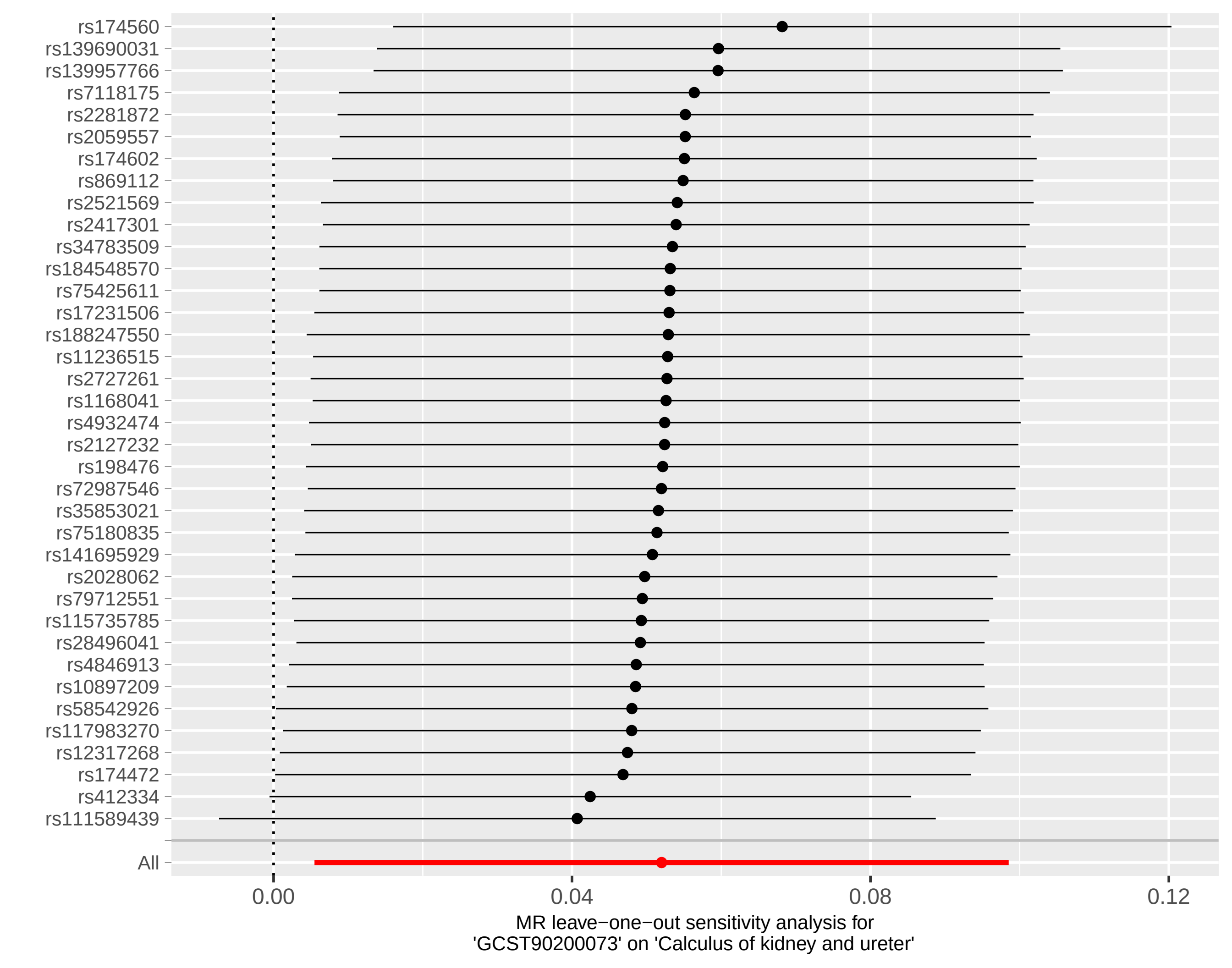
rs174560
rs139690031
rs139957766
rs7118175
rs2281872
rs2059557
rs174602
rs869112
rs2521569
rs2417301
rs34783509
rs184548570
rs75425611
rs17231506
rs188247550
rs11236515
rs2727261
rs1168041
rs4932474
rs2127232
rs198476
rs72987546
rs35853021
rs75180835
rs141695929
rs2028062
rs79712551
rs115735785
rs28496041
rs4846913
rs10897209
rs58542926
rs117983270
rs12317268
rs174472
rs412334
rs111589439
All
0.00
0.04
0.08
0.12
MR leave−one−out sensitivity analysis for
'GCST90200073' on 'Calculus of kidney and ureter'

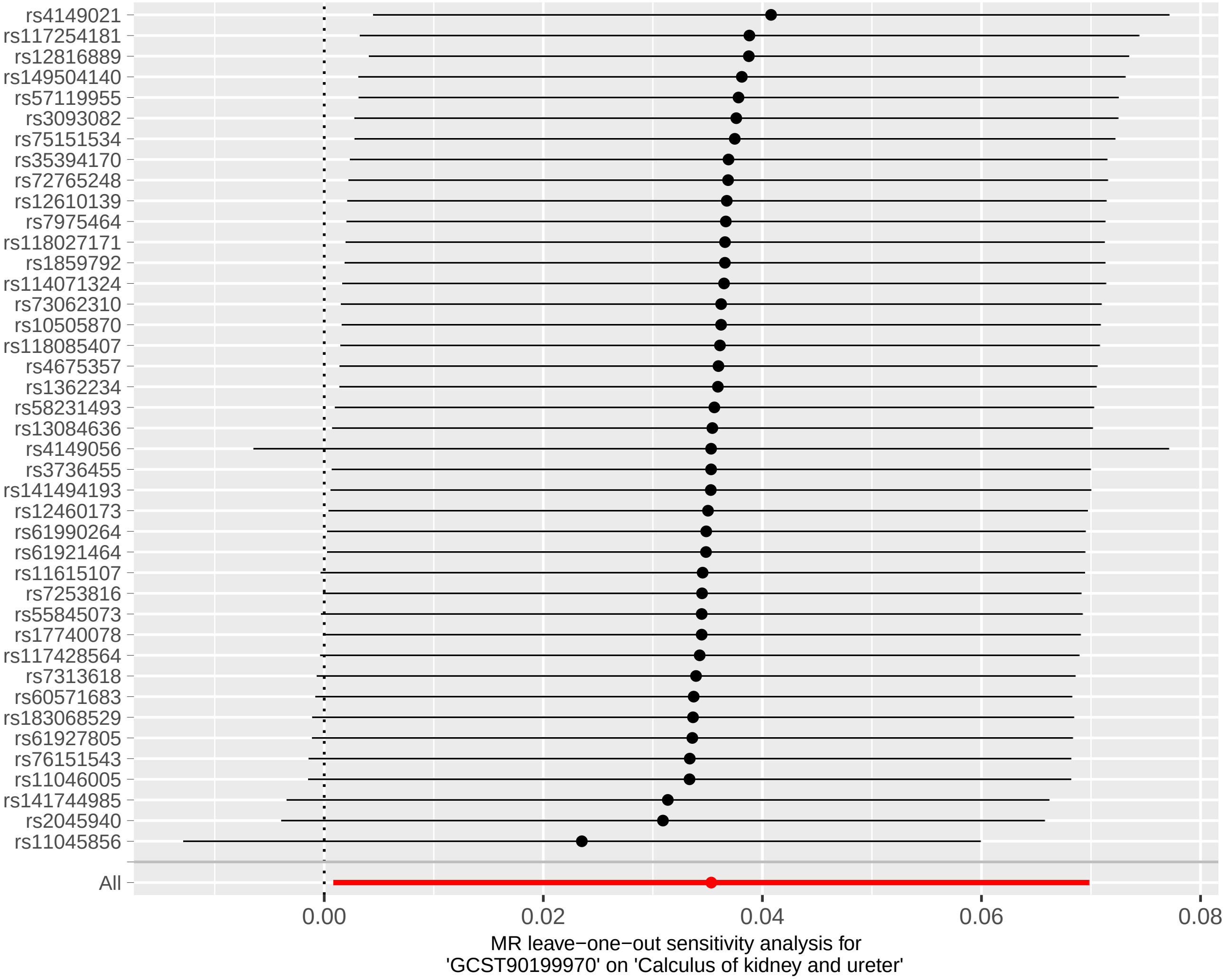

rs4149021
rs117254181
rs12816889
rs149504140
rs57119955
rs3093082
rs75151534
rs35394170
rs72765248
rs12610139
rs7975464
rs118027171
rs1859792
rs114071324
rs73062310
rs10505870
rs118085407
rs4675357
rs1362234
rs58231493
rs13084636
rs4149056
rs3736455
rs141494193
rs12460173
rs61990264
rs61921464
rs11615107
rs7253816
rs55845073
rs17740078
rs117428564
rs7313618
rs60571683
rs183068529
rs61927805
rs76151543
rs11046005
rs141744985
rs2045940
rs11045856
All
0.00
0.02
0.04
0.06
0.08
MR leave−one−out sensitivity analysis for
'GCST90199970' on 'Calculus of kidney and ureter'

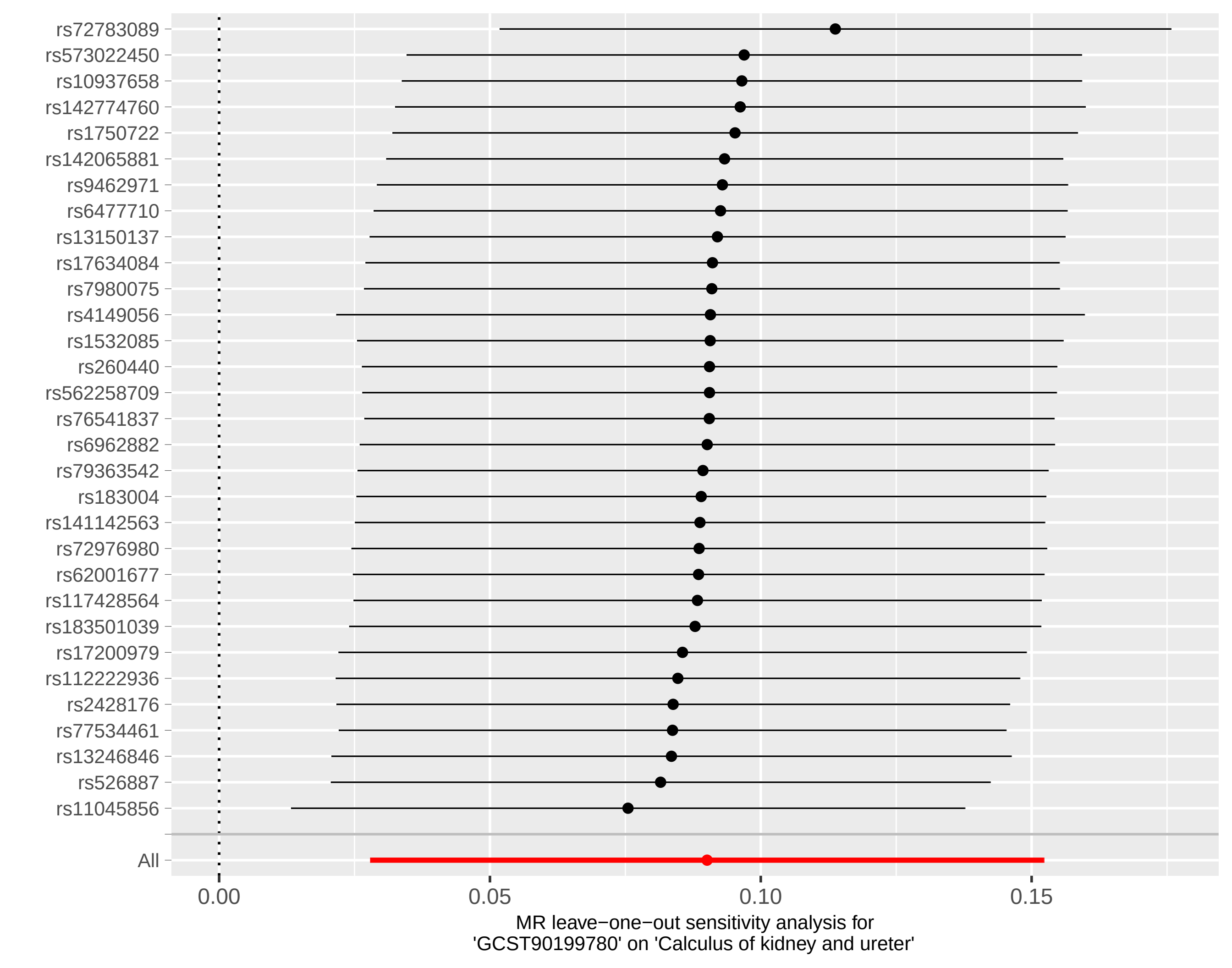
rs72783089
rs573022450
rs10937658
rs142774760
rs1750722
rs142065881
rs9462971
rs6477710
rs13150137
rs17634084
rs7980075
rs4149056
rs1532085
rs260440
rs562258709
rs76541837
rs6962882
rs79363542
rs183004
rs141142563
rs72976980
rs62001677
rs117428564
rs183501039
rs17200979
rs112222936
rs2428176
rs77534461
rs13246846
rs526887
rs11045856
All
0.00
0.05
0.10
0.15
MR leave−one−out sensitivity analysis for
'GCST90199780' on 'Calculus of kidney and ureter'

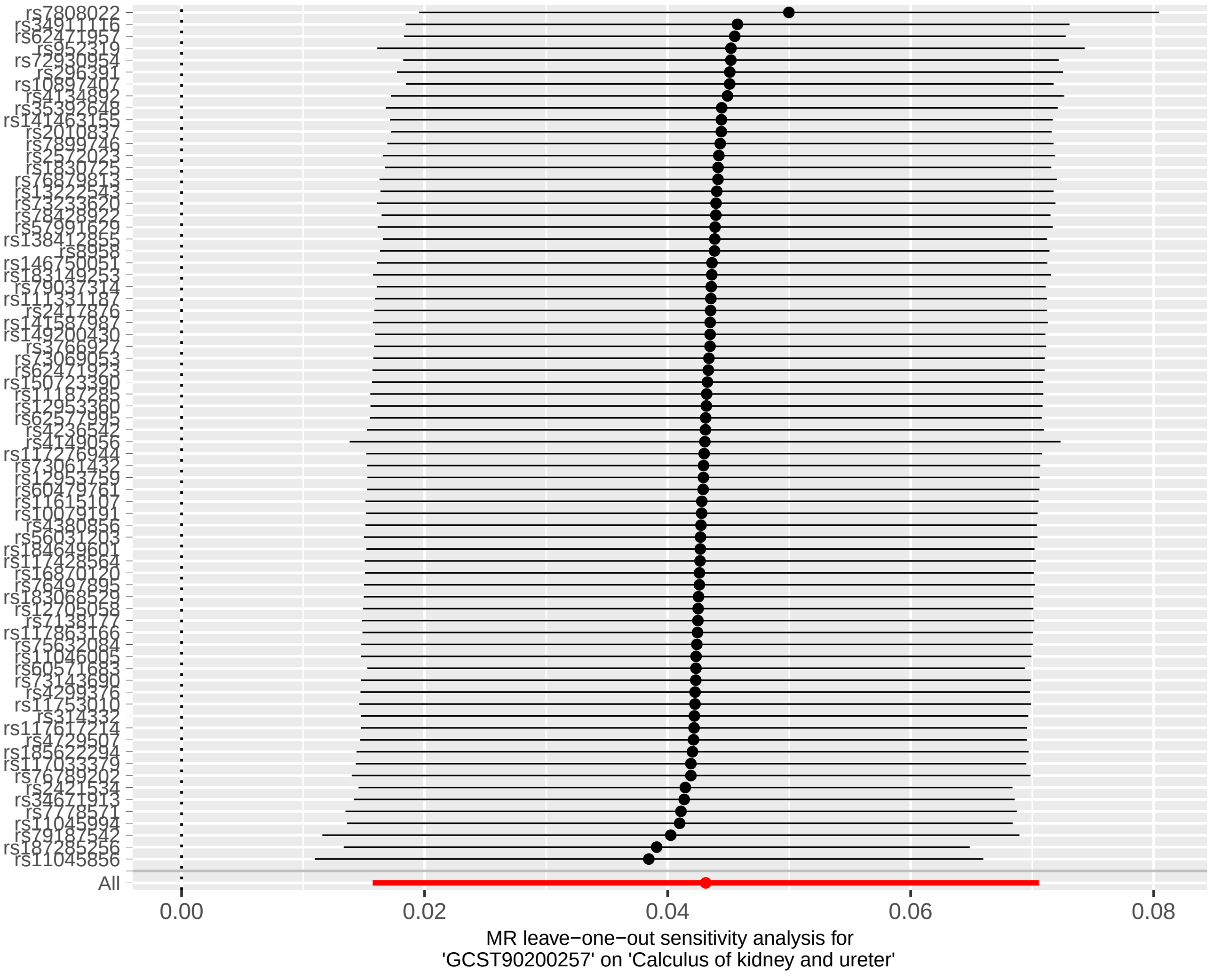
rs7808022
rs34911116
rs62471957
rs952319
rs72930954
rs296391
rs10897407
rs4134892
rs35392648
rs141463155
rs2010837
rs7899746
rs2572023
rs1830725
rs76879813
rs13222543
rs73233620
rs78428922
rs57991629
rs138412855
rs8958
rs146750051
rs183149253
rs79037314
rs111331187
rs2417876
rs141587987
rs149200430
rs3766927
rs73069053
rs62471923
rs150723390
rs11187285
rs12953360
rs62577995
rs4236542
rs4149056
rs117276944
rs73061432
rs12953759
rs60479761
rs11615107
rs10079191
rs4380856
rs56031203
rs184649601
rs117428564
rs16870120
rs76497895
rs183068529
rs12705058
rs7138177
rs117863166
rs75632084
rs11046005
rs60571683
rs73143690
rs4299376
rs11753010
rs314332
rs117617214
rs4729507
rs185622294
rs117033379
rs76789202
rs2421534
rs34671913
rs7778571
rs11045994
rs79187542
rs187285256
rs11045856
All
0.00
0.02
0.04
0.06
0.08
MR leave−one−out sensitivity analysis for
'GCST90200257' on 'Calculus of kidney and ureter'

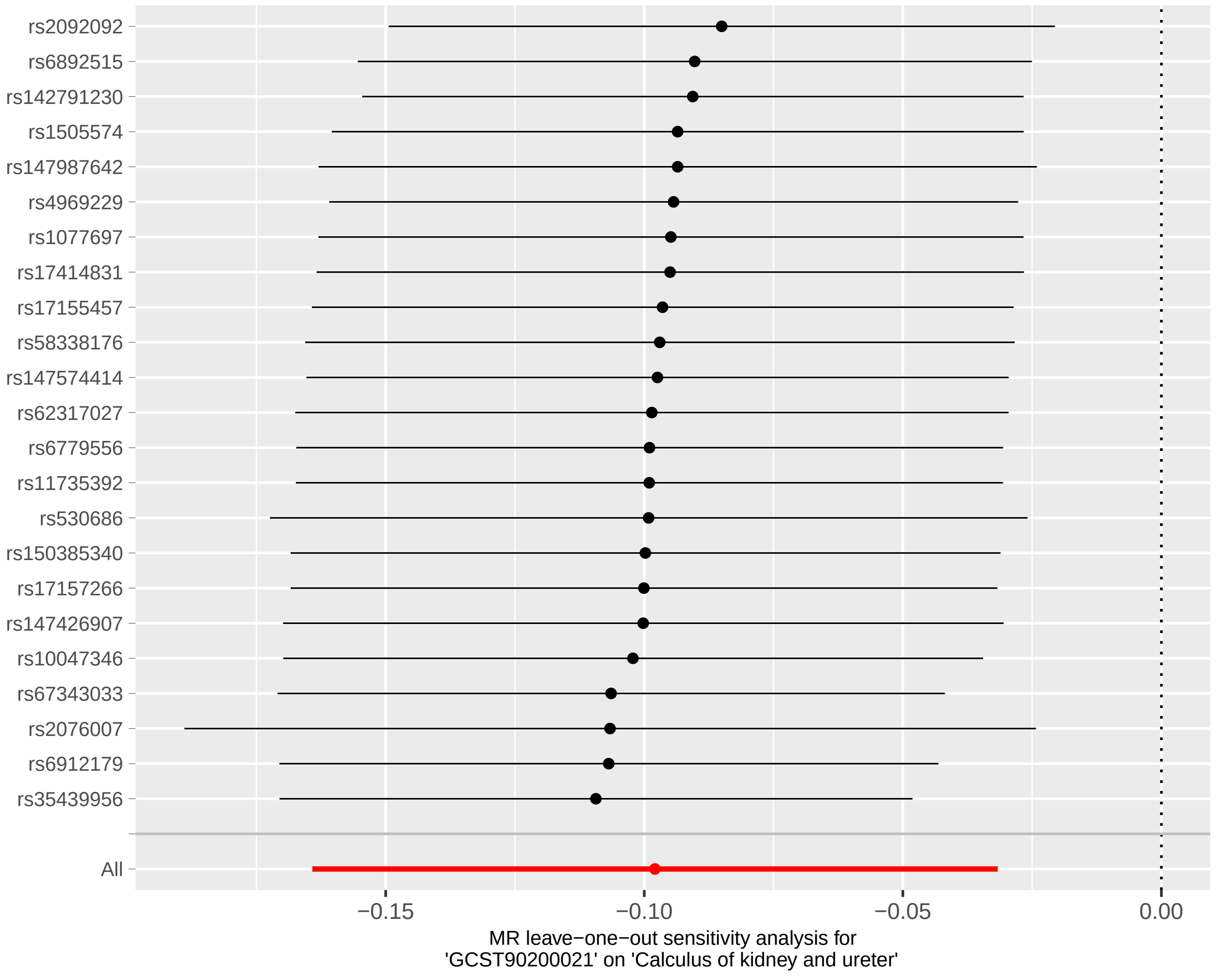
rs2092092
rs6892515
rs142791230
rs1505574
rs147987642
rs4969229
rs1077697
rs17414831
rs17155457
rs58338176
rs147574414
rs62317027
rs6779556
rs11735392
rs530686
rs150385340
rs17157266
rs147426907
rs10047346
rs67343033
rs2076007
rs6912179
rs35439956
All
−0.15
−0.10
−0.05
0.00
MR leave−one−out sensitivity analysis for
'GCST90200021' on 'Calculus of kidney and ureter'

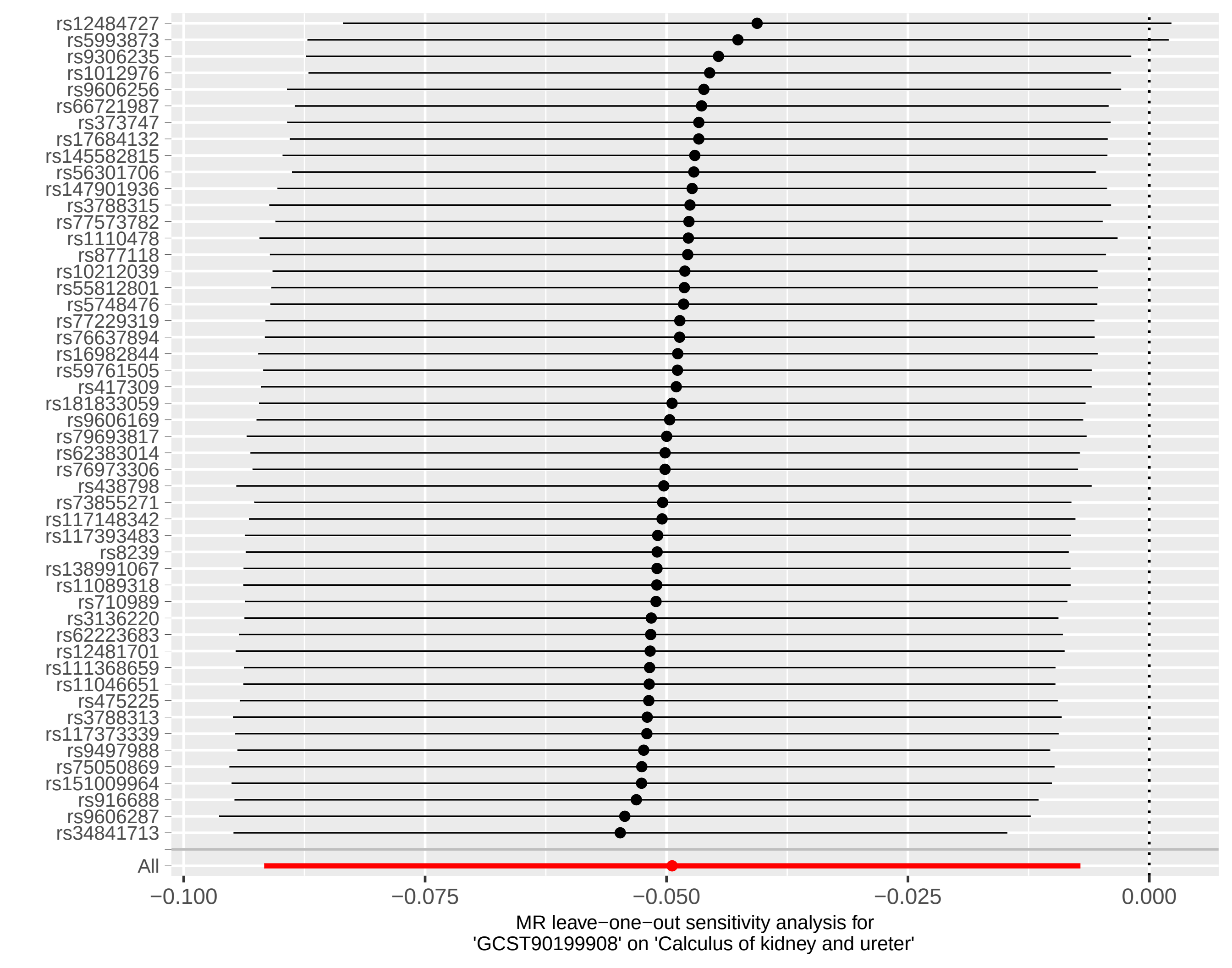
rs12484727
rs5993873
rs9306235
rs1012976
rs9606256
rs66721987
rs373747
rs17684132
rs145582815
rs56301706
rs147901936
rs3788315
rs77573782
rs1110478
rs877118
rs10212039
rs55812801
rs5748476
rs77229319
rs76637894
rs16982844
rs59761505
rs417309
rs181833059
rs9606169
rs79693817
rs62383014
rs76973306
rs438798
rs73855271
rs117148342
rs117393483
rs8239
rs138991067
rs11089318
rs710989
rs3136220
rs62223683
rs12481701
rs111368659
rs11046651
rs475225
rs3788313
rs117373339
rs9497988
rs75050869
rs151009964
rs916688
rs9606287
rs34841713
All
−0.100
−0.075
−0.050
−0.025
0.000
MR leave−one−out sensitivity analysis for
'GCST90199908' on 'Calculus of kidney and ureter'

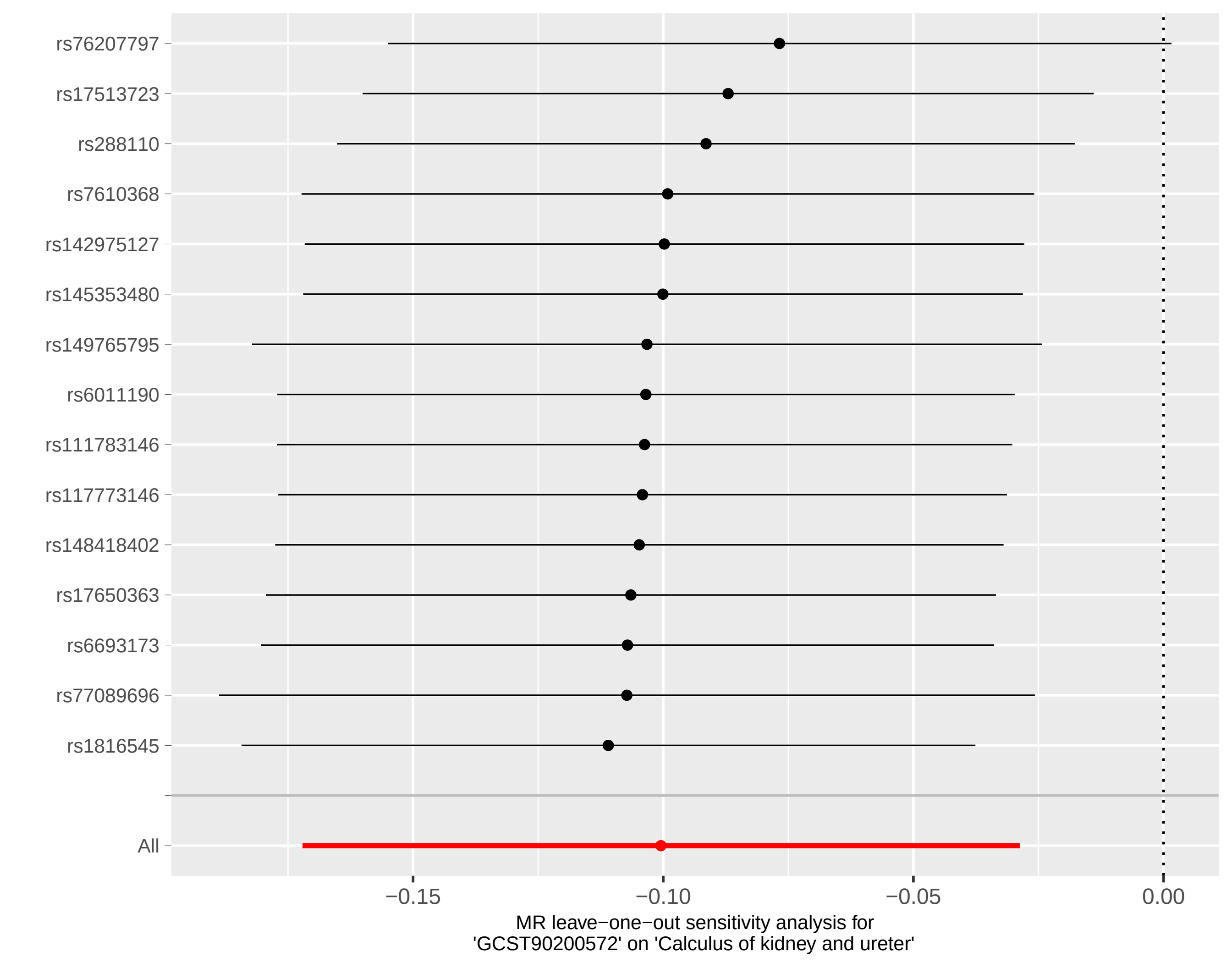
rs76207797
rs17513723
rs288110
rs7610368
rs142975127
rs145353480
rs149765795
rs6011190
rs111783146
rs117773146
rs148418402
rs17650363
rs6693173
rs77089696
rs1816545
All
−0.15
−0.10
−0.05
0.00
MR leave−one−out sensitivity analysis for
'GCST90200572' on 'Calculus of kidney and ureter'

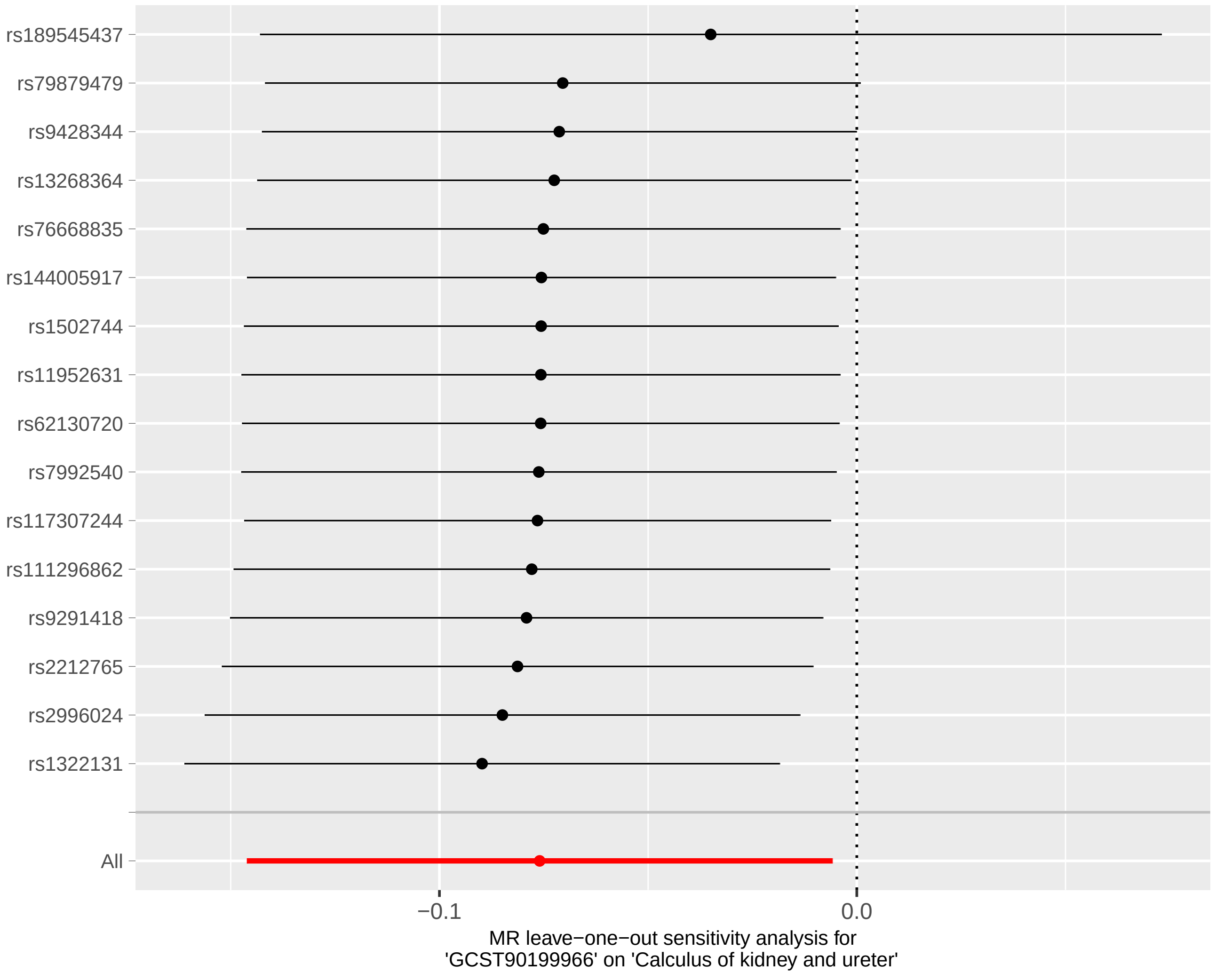
rs189545437
rs79879479
rs9428344
rs13268364
rs76668835
rs144005917
rs1502744
rs11952631
rs62130720
rs7992540
rs117307244
rs111296862
rs9291418
rs2212765
rs2996024
rs1322131
All
−0.1
0.0
MR leave−one−out sensitivity analysis for
'GCST90199966' on 'Calculus of kidney and ureter'

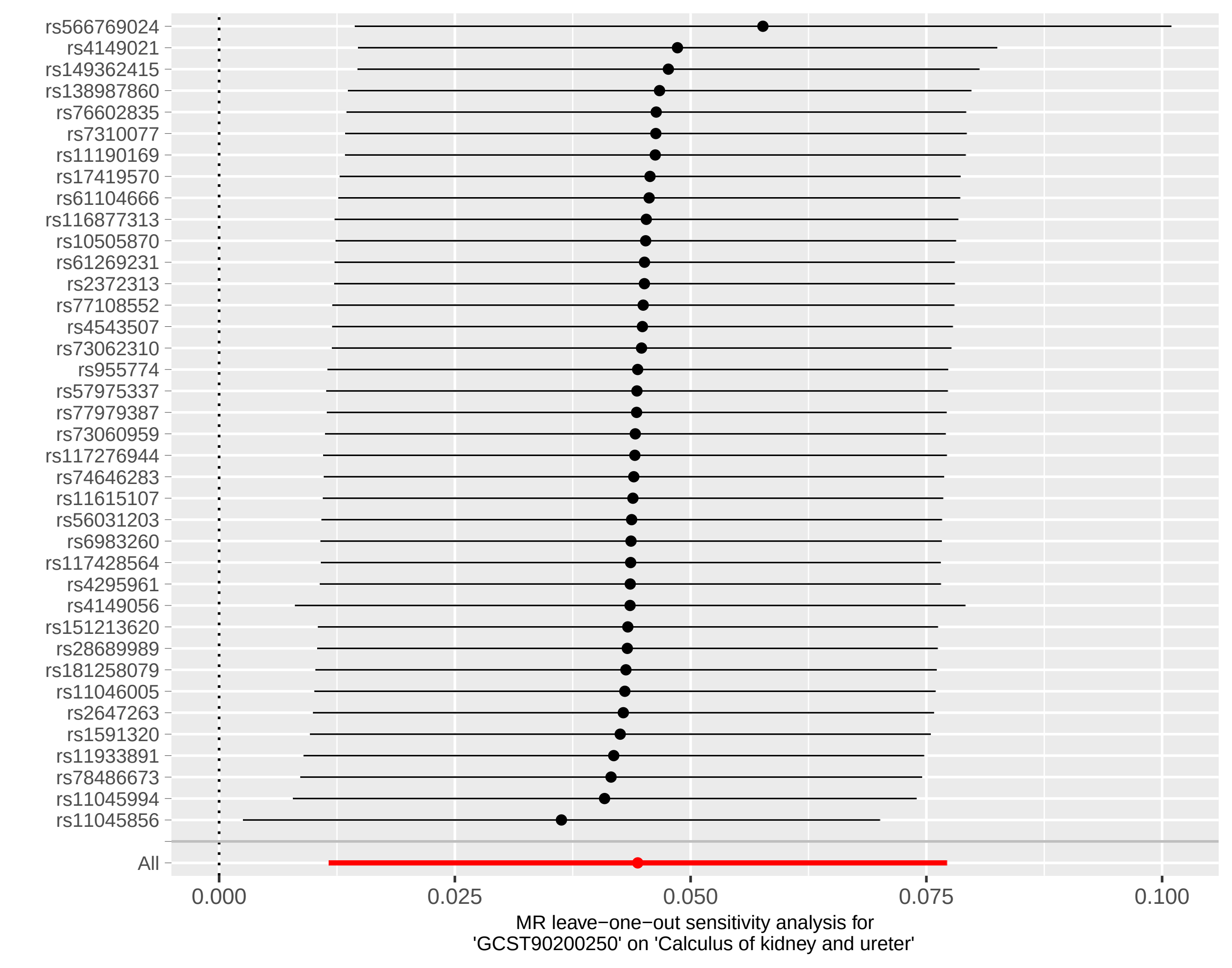
rs566769024
rs4149021
rs149362415
rs138987860
rs76602835
rs7310077
rs11190169
rs17419570
rs61104666
rs116877313
rs10505870
rs61269231
rs2372313
rs77108552
rs4543507
rs73062310
rs955774
rs57975337
rs77979387
rs73060959
rs117276944
rs74646283
rs11615107
rs56031203
rs6983260
rs117428564
rs4295961
rs4149056
rs151213620
rs28689989
rs181258079
rs11046005
rs2647263
rs1591320
rs11933891
rs78486673
rs11045994
rs11045856
All
0.000
0.025
0.050
0.075
0.100
MR leave−one−out sensitivity analysis for
'GCST90200250' on 'Calculus of kidney and ureter'

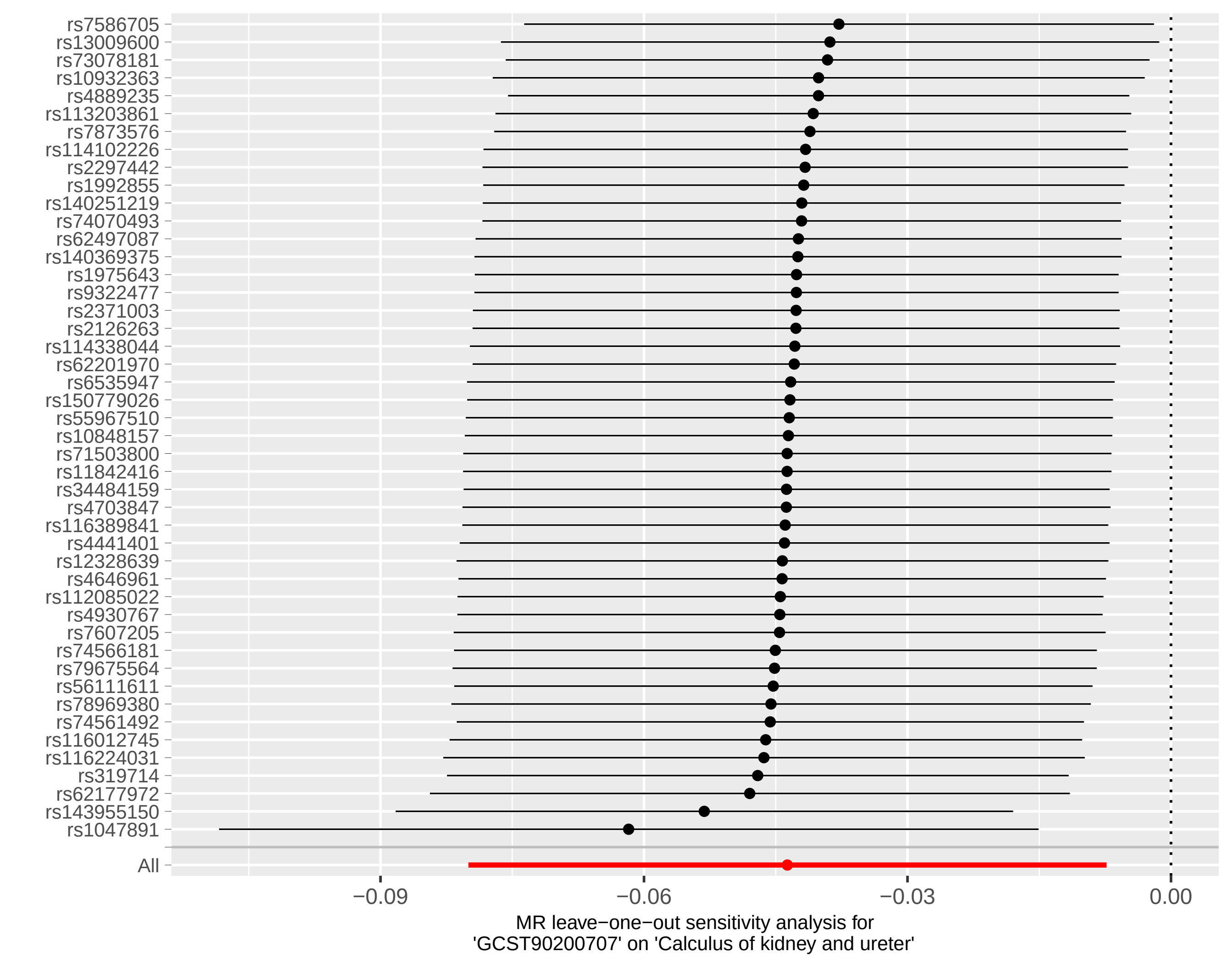

rs7586705
rs13009600
rs73078181
rs10932363
rs4889235
rs113203861
rs7873576
rs114102226
rs2297442
rs1992855
rs140251219
rs74070493
rs62497087
rs140369375
rs1975643
rs9322477
rs2371003
rs2126263
rs114338044
rs62201970
rs6535947
rs150779026
rs55967510
rs10848157
rs71503800
rs11842416
rs34484159
rs4703847
rs116389841
rs4441401
rs12328639
rs4646961
rs112085022
rs4930767
rs7607205
rs74566181
rs79675564
rs56111611
rs78969380
rs74561492
rs116012745
rs116224031
rs319714
rs62177972
rs143955150
rs1047891
All
−0.09
−0.06
−0.03
0.00
MR leave−one−out sensitivity analysis for
'GCST90200707' on 'Calculus of kidney and ureter'

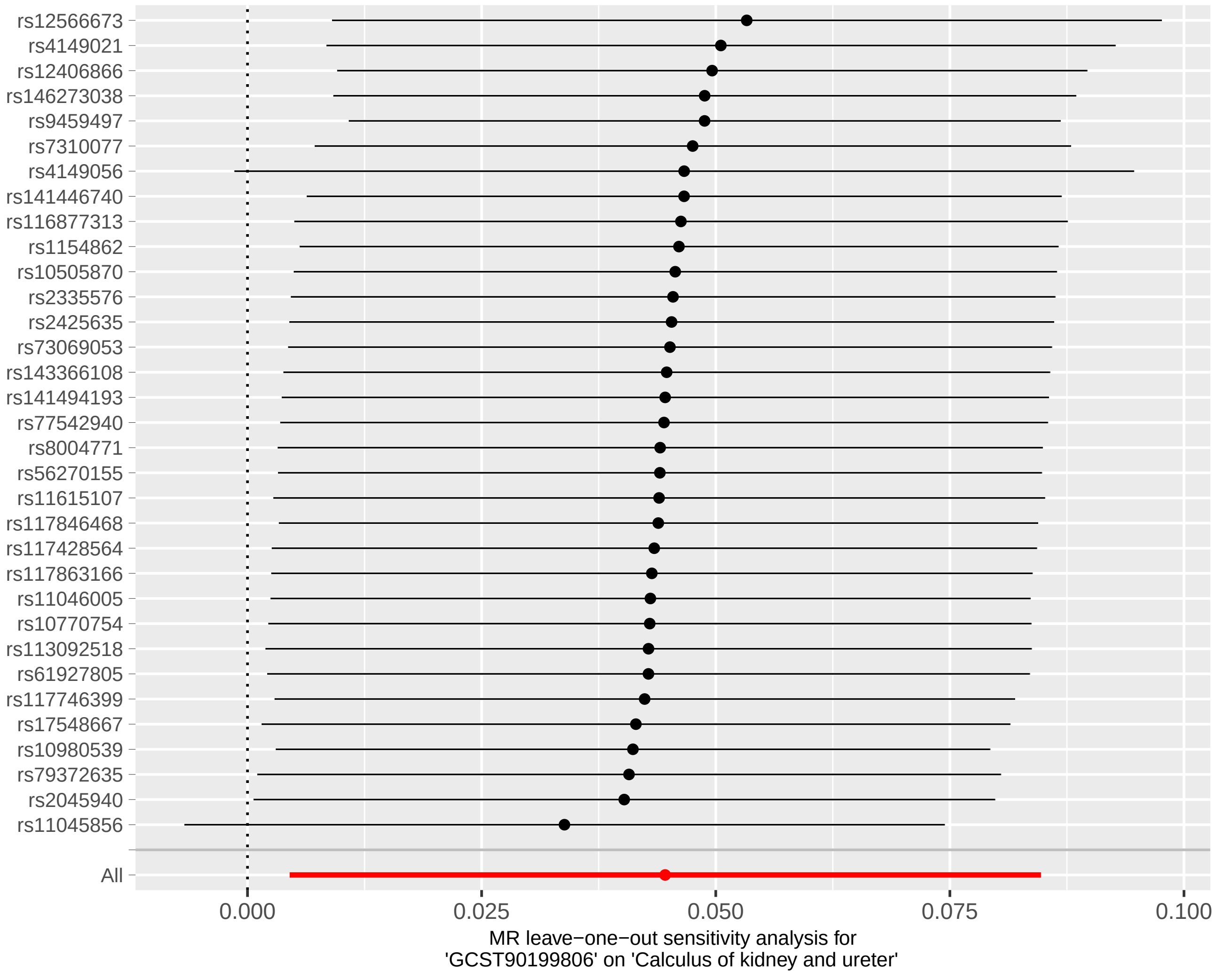
rs12566673
rs4149021
rs12406866
rs146273038
rs9459497
rs7310077
rs4149056
rs141446740
rs116877313
rs1154862
rs10505870
rs2335576
rs2425635
rs73069053
rs143366108
rs141494193
rs77542940
rs8004771
rs56270155
rs11615107
rs117846468
rs117428564
rs117863166
rs11046005
rs10770754
rs113092518
rs61927805
rs117746399
rs17548667
rs10980539
rs79372635
rs2045940
rs11045856
All
0.000
0.025
0.050
0.075
0.100
MR leave−one−out sensitivity analysis for
'GCST90199806' on 'Calculus of kidney and ureter'

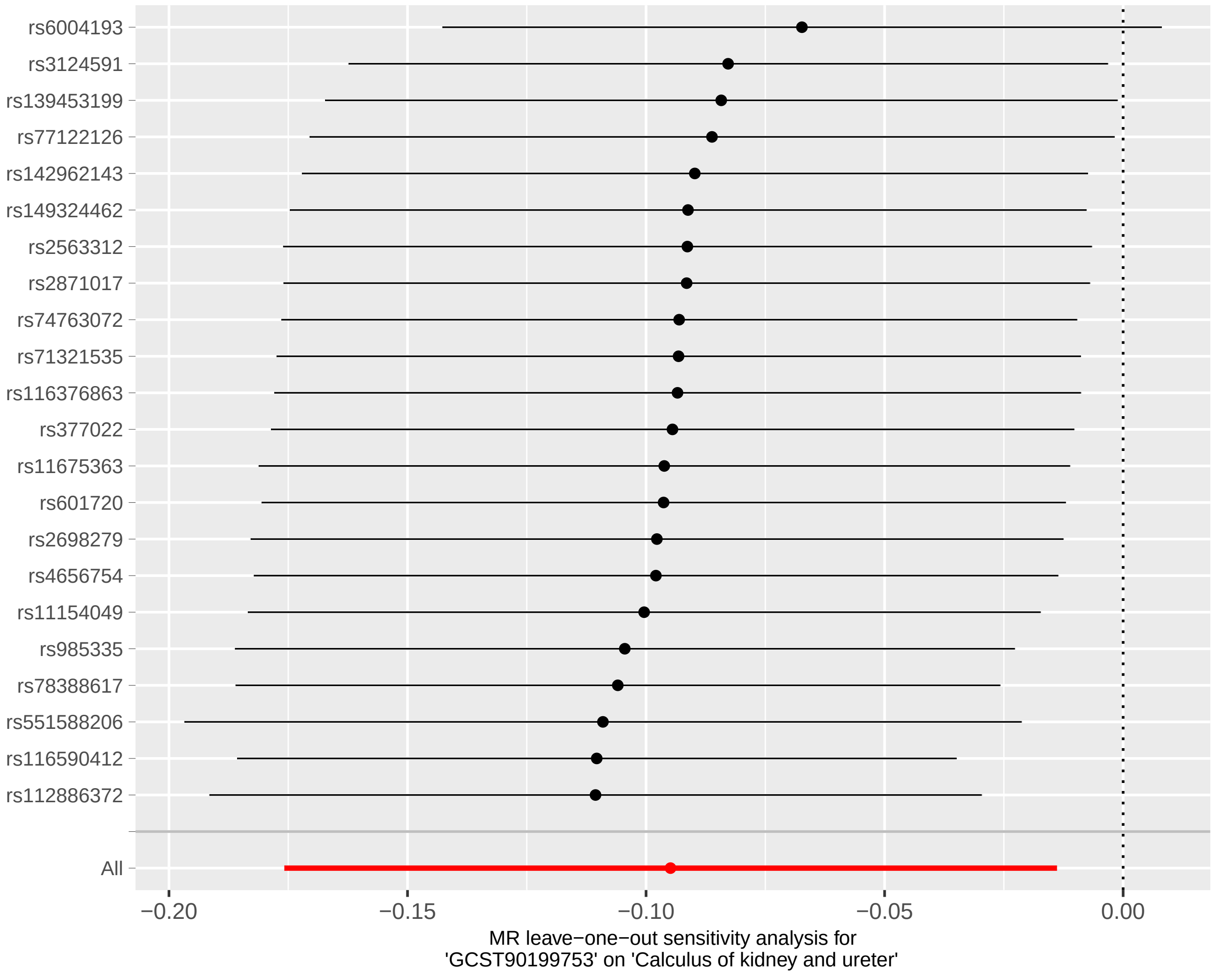

rs6004193
rs3124591
rs139453199
rs77122126
rs142962143
rs149324462
rs2563312
rs2871017
rs74763072
rs71321535
rs116376863
rs377022
rs11675363
rs601720
rs2698279
rs4656754
rs11154049
rs985335
rs78388617
rs551588206
rs116590412
rs112886372
All
−0.20
−0.15
−0.10
−0.05
0.00
MR leave−one−out sensitivity analysis for
'GCST90199753' on 'Calculus of kidney and ureter'

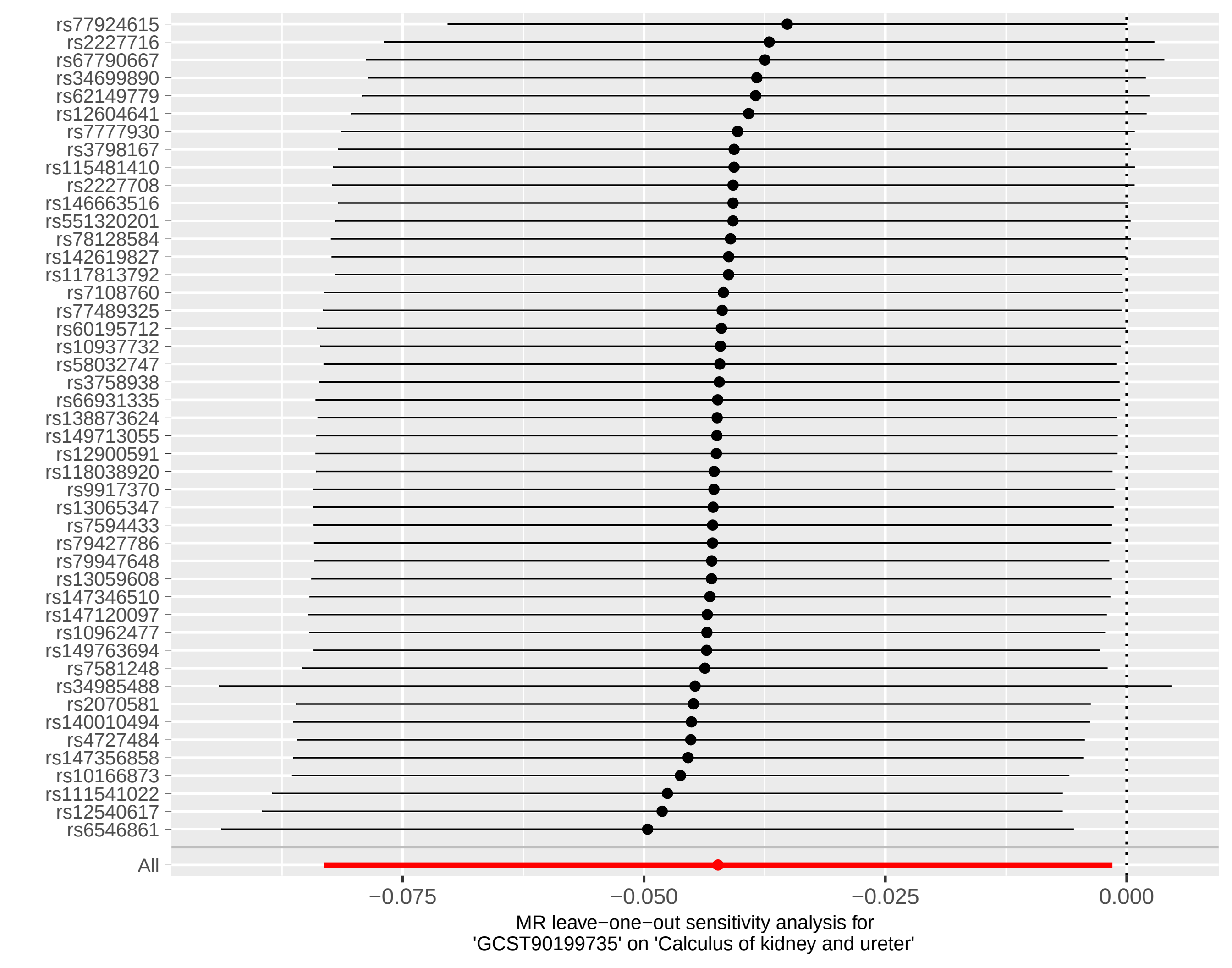
rs77924615
rs2227716
rs67790667
rs34699890
rs62149779
rs12604641
rs7777930
rs3798167
rs115481410
rs2227708
rs146663516
rs551320201
rs78128584
rs142619827
rs117813792
rs7108760
rs77489325
rs60195712
rs10937732
rs58032747
rs3758938
rs66931335
rs138873624
rs149713055
rs12900591
rs118038920
rs9917370
rs13065347
rs7594433
rs79427786
rs79947648
rs13059608
rs147346510
rs147120097
rs10962477
rs149763694
rs7581248
rs34985488
rs2070581
rs140010494
rs4727484
rs147356858
rs10166873
rs111541022
rs12540617
rs6546861
All
−0.075
−0.050
−0.025
0.000
MR leave−one−out sensitivity analysis for
'GCST90199735' on 'Calculus of kidney and ureter'

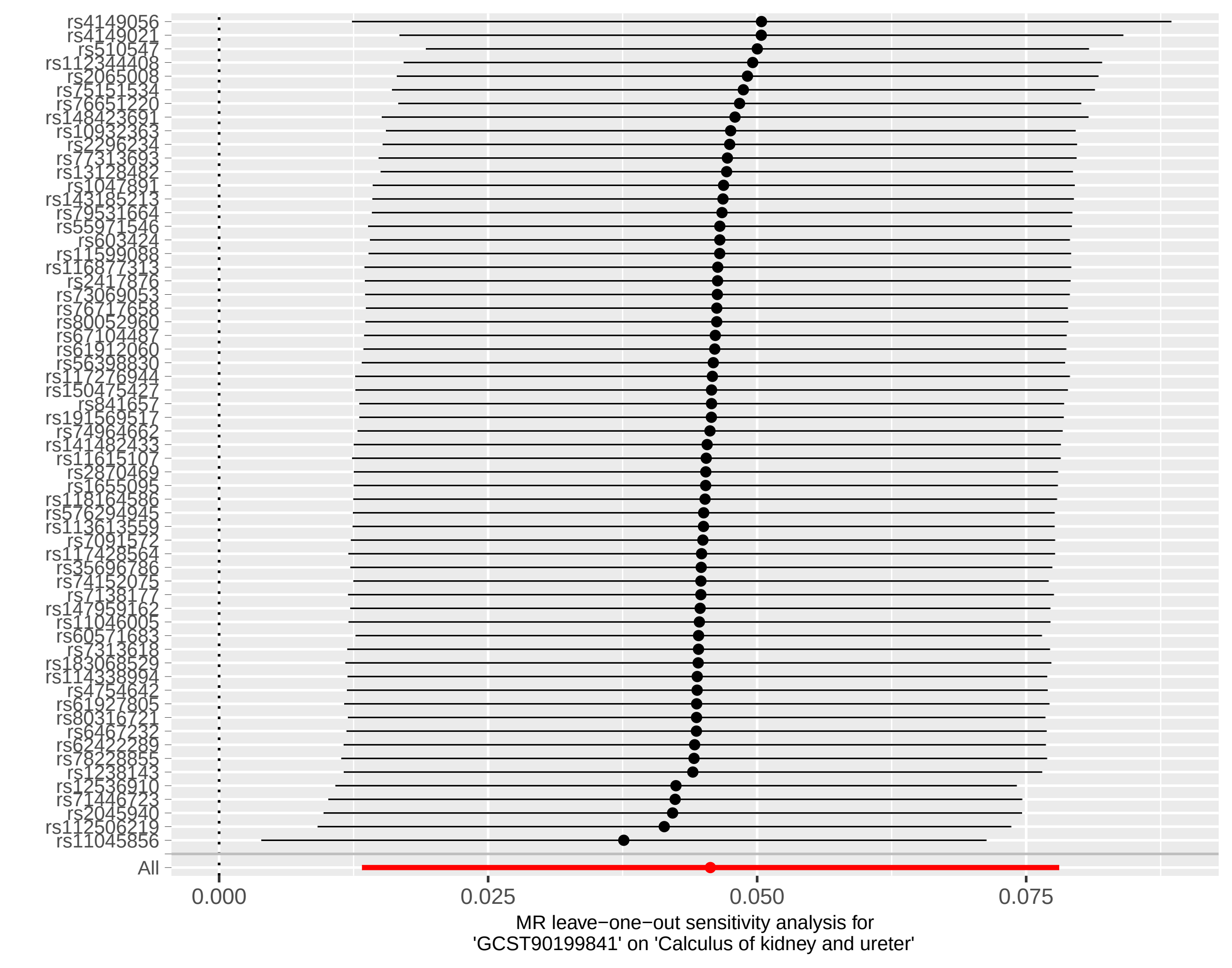
rs4149056
rs4149021
rs510547
rs112344408
rs2065008
rs75151534
rs76651220
rs148423691
rs10932363
rs2296234
rs77313693
rs13128482
rs1047891
rs143185213
rs79531664
rs55971546
rs603424
rs11599088
rs116877313
rs2417876
rs73069053
rs76717658
rs80052960
rs67104487
rs61912060
rs56398830
rs117276944
rs150475427
rs841657
rs191569517
rs74964662
rs141482433
rs11615107
rs2870469
rs1655095
rs118164586
rs576294945
rs113613559
rs7091572
rs117428564
rs35696786
rs74152075
rs7138177
rs147959162
rs11046005
rs60571683
rs7313618
rs183068529
rs114338994
rs4754642
rs61927805
rs80316721
rs6467232
rs62422289
rs78228855
rs1238143
rs12536910
rs71446723
rs2045940
rs112506219
rs11045856
All
0.000
0.025
0.050
0.075
MR leave−one−out sensitivity analysis for
'GCST90199841' on 'Calculus of kidney and ureter'

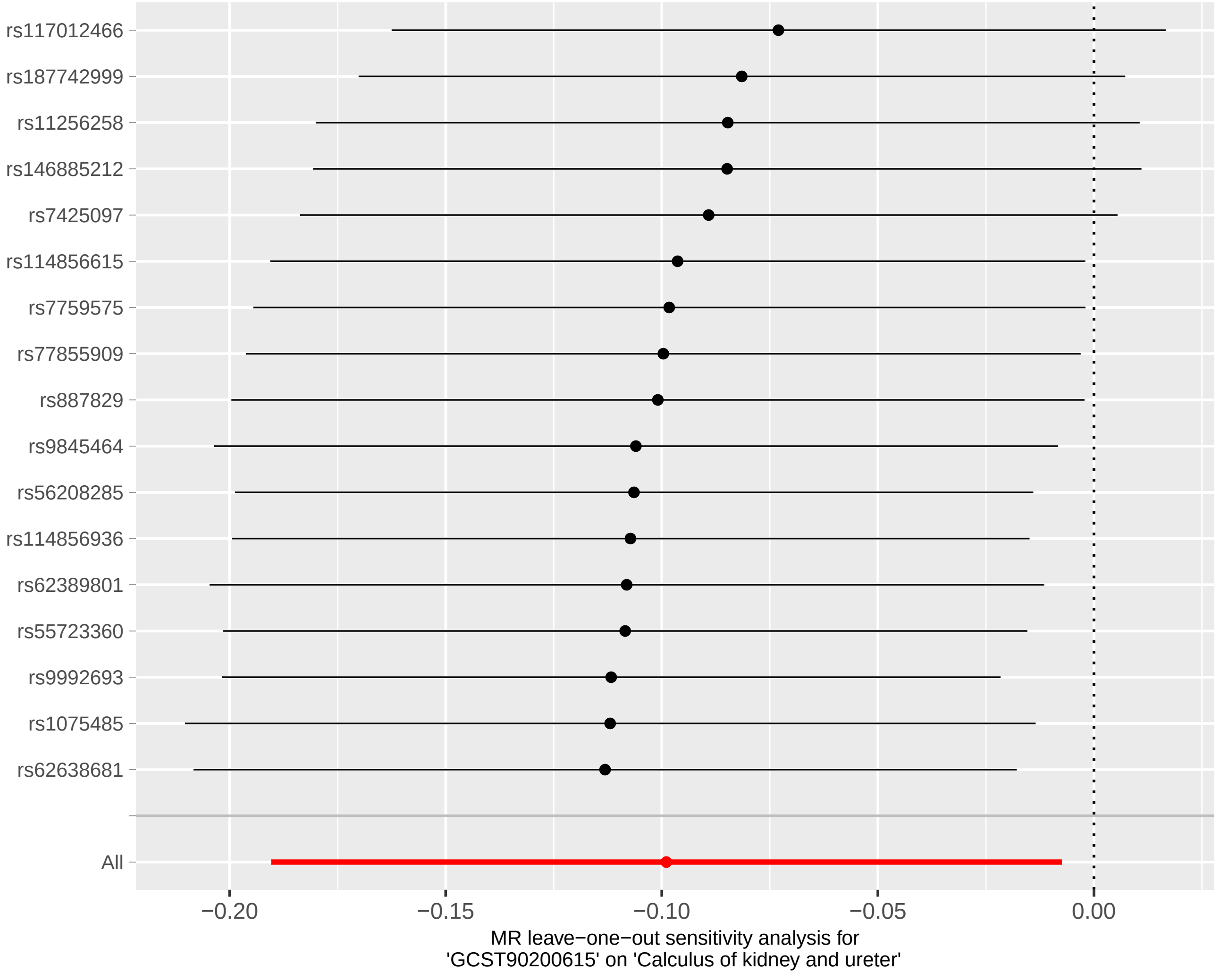
rs117012466
rs187742999
rs11256258
rs146885212
rs7425097
rs114856615
rs7759575
rs77855909
rs887829
rs9845464
rs56208285
rs114856936
rs62389801
rs55723360
rs9992693
rs1075485
rs62638681
All
−0.20
−0.15
−0.10
−0.05
0.00
MR leave−one−out sensitivity analysis for
'GCST90200615' on 'Calculus of kidney and ureter'

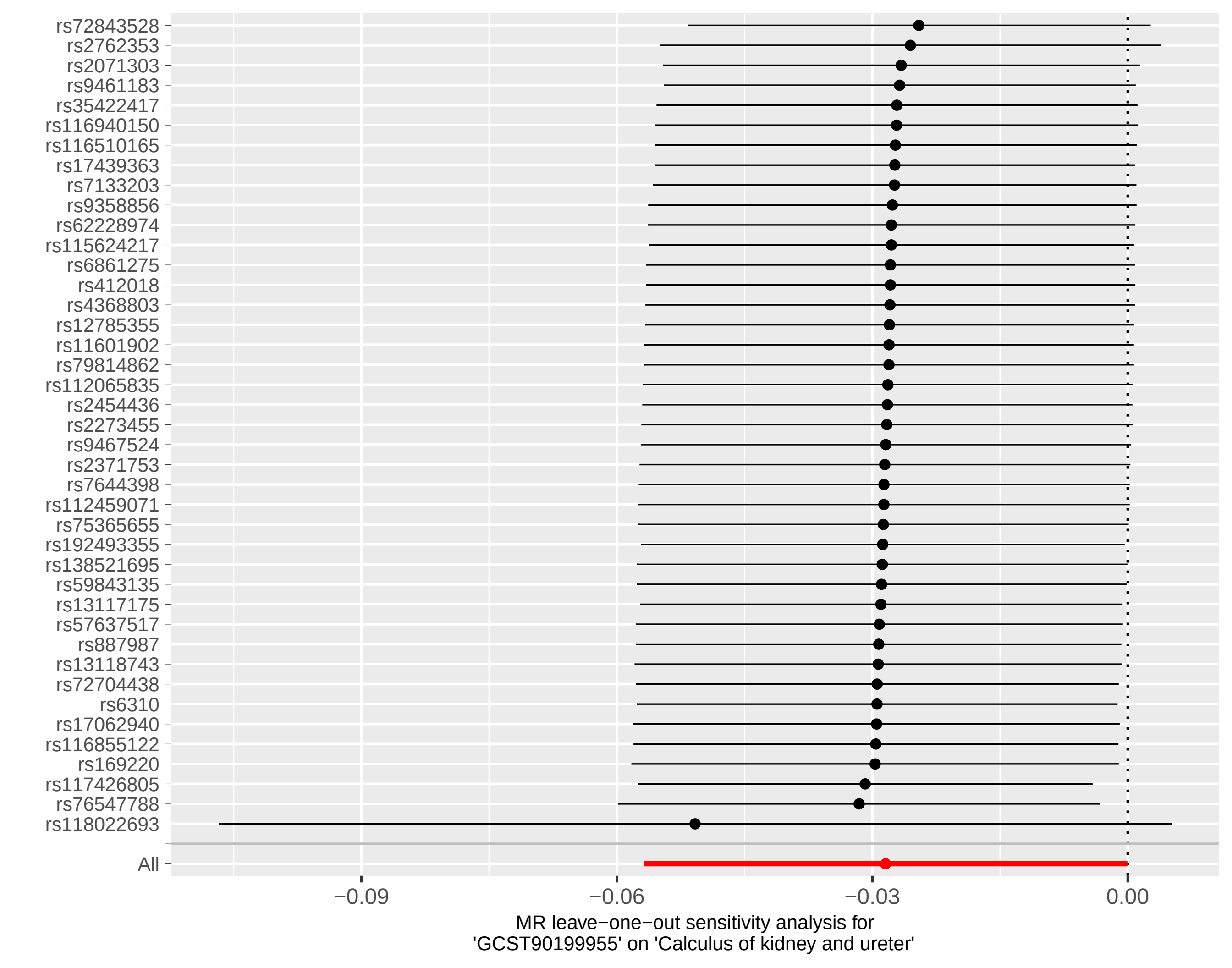
rs72843528
rs2762353
rs2071303
rs9461183
rs35422417
rs116940150
rs116510165
rs17439363
rs7133203
rs9358856
rs62228974
rs115624217
rs6861275
rs412018
rs4368803
rs12785355
rs11601902
rs79814862
rs112065835
rs2454436
rs2273455
rs9467524
rs2371753
rs7644398
rs112459071
rs75365655
rs192493355
rs138521695
rs59843135
rs13117175
rs57637517
rs887987
rs13118743
rs72704438
rs6310
rs17062940
rs116855122
rs169220
rs117426805
rs76547788
rs118022693
All
−0.09
−0.06
−0.03
0.00
MR leave−one−out sensitivity analysis for
'GCST90199955' on 'Calculus of kidney and ureter'

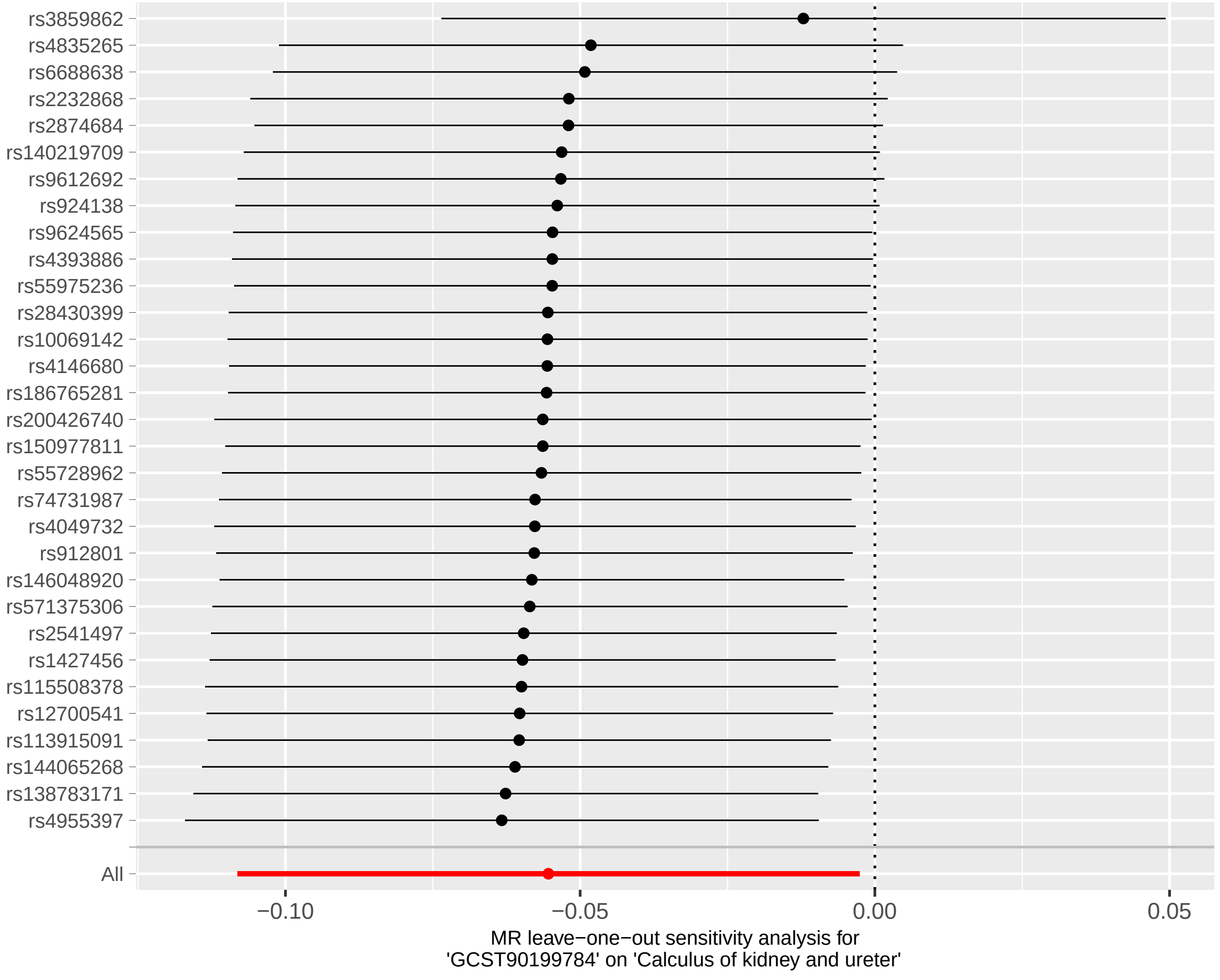
rs3859862
rs4835265
rs6688638
rs2232868
rs2874684
rs140219709
rs9612692
rs924138
rs9624565
rs4393886
rs55975236
rs28430399
rs10069142
rs4146680
rs186765281
rs200426740
rs150977811
rs55728962
rs74731987
rs4049732
rs912801
rs146048920
rs571375306
rs2541497
rs1427456
rs115508378
rs12700541
rs113915091
rs144065268
rs138783171
rs4955397
All
−0.10
−0.05
0.00
0.05
MR leave−one−out sensitivity analysis for
'GCST90199784' on 'Calculus of kidney and ureter'

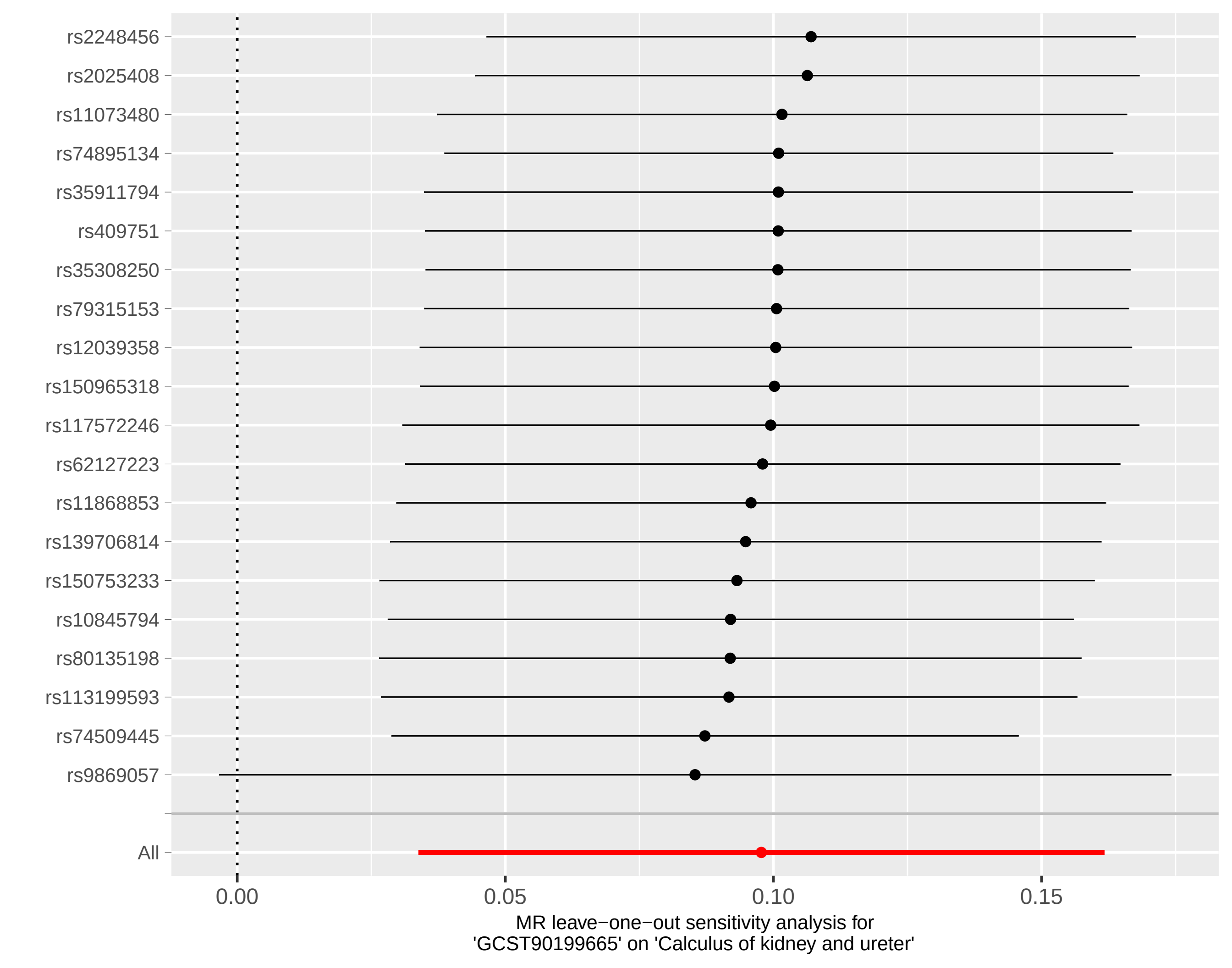
rs2248456
rs2025408
rs11073480
rs74895134
rs35911794
rs409751
rs35308250
rs79315153
rs12039358
rs150965318
rs117572246
rs62127223
rs11868853
rs139706814
rs150753233
rs10845794
rs80135198
rs113199593
rs74509445
rs9869057
All
0.00
0.05
0.10
0.15
MR leave−one−out sensitivity analysis for
'GCST90199665' on 'Calculus of kidney and ureter'

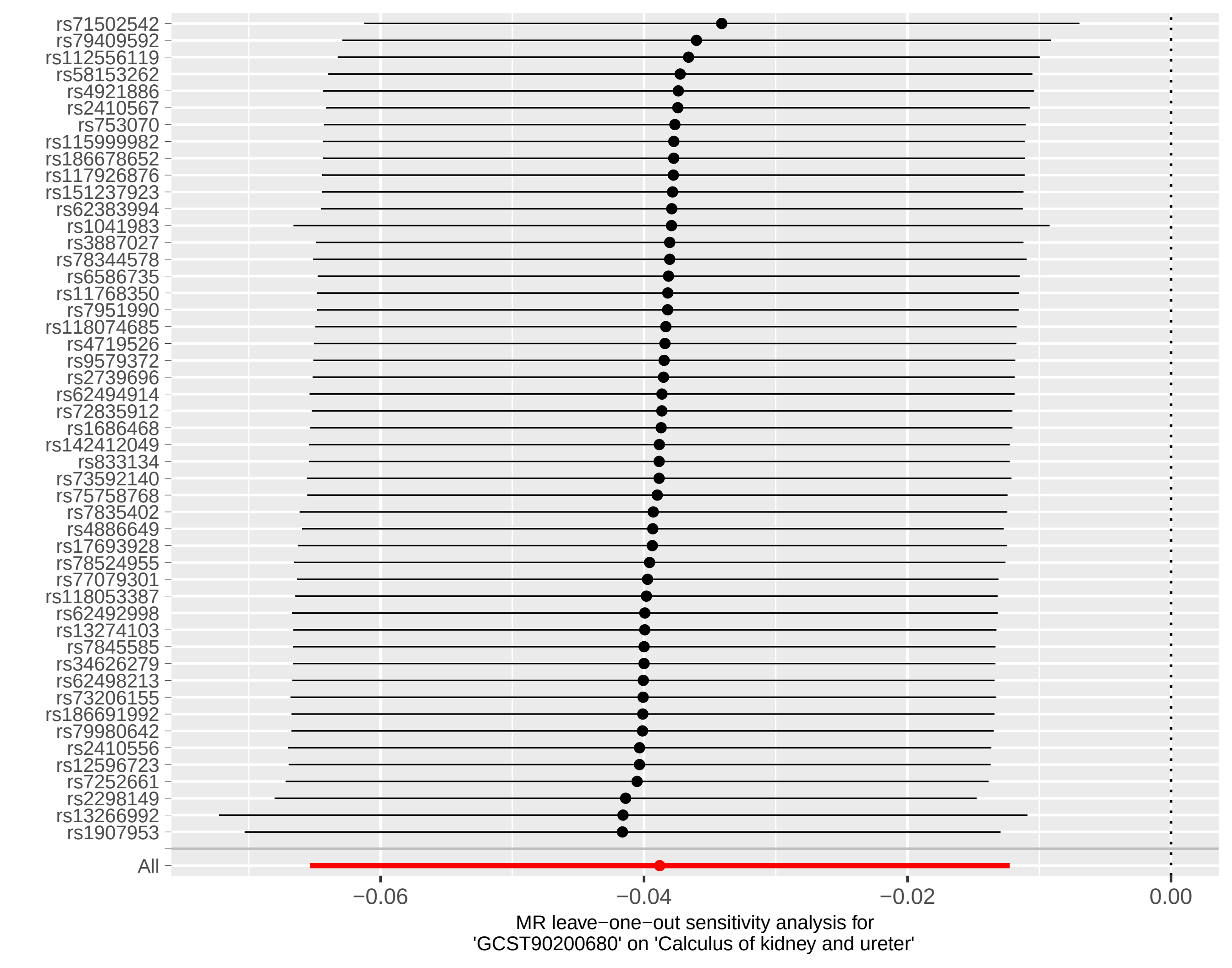
rs71502542
rs79409592
rs112556119
rs58153262
rs4921886
rs2410567
rs753070
rs115999982
rs186678652
rs117926876
rs151237923
rs62383994
rs1041983
rs3887027
rs78344578
rs6586735
rs11768350
rs7951990
rs118074685
rs4719526
rs9579372
rs2739696
rs62494914
rs72835912
rs1686468
rs142412049
rs833134
rs73592140
rs75758768
rs7835402
rs4886649
rs17693928
rs78524955
rs77079301
rs118053387
rs62492998
rs13274103
rs7845585
rs34626279
rs62498213
rs73206155
rs186691992
rs79980642
rs2410556
rs12596723
rs7252661
rs2298149
rs13266992
rs1907953
All
−0.06
−0.04
−0.02
0.00
MR leave−one−out sensitivity analysis for
'GCST90200680' on 'Calculus of kidney and ureter'

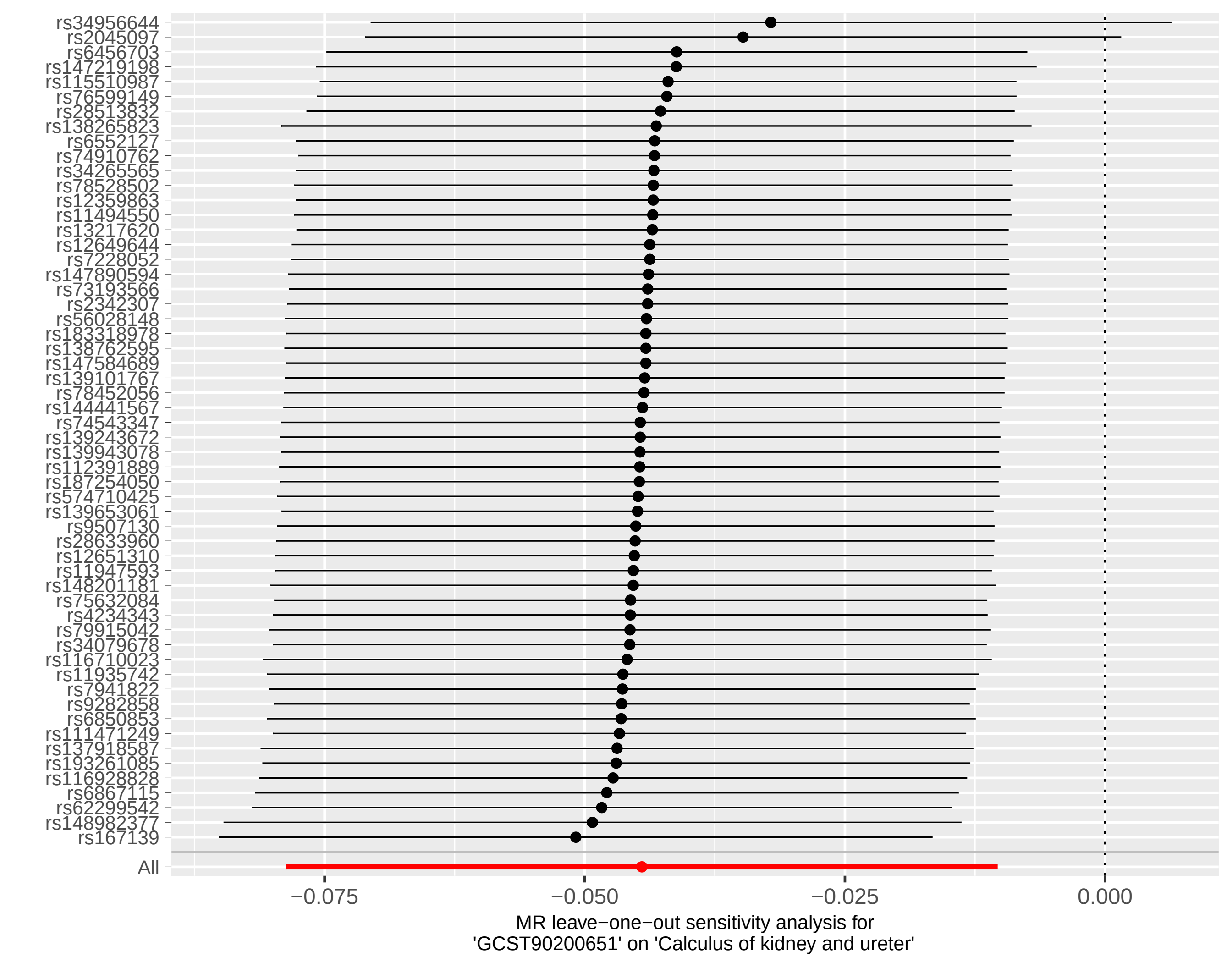
rs34956644
rs2045097
rs6456703
rs147219198
rs115510987
rs76599149
rs28513832
rs138265823
rs6552127
rs74910762
rs34265565
rs78528502
rs12359863
rs11494550
rs13217620
rs12649644
rs7228052
rs147890594
rs73193566
rs2342307
rs56028148
rs183318978
rs138762595
rs147584689
rs139101767
rs78452056
rs144441567
rs74543347
rs139243672
rs139943078
rs112391889
rs187254050
rs574710425
rs139653061
rs9507130
rs28633960
rs12651310
rs11947593
rs148201181
rs75632084
rs4234343
rs79915042
rs34079678
rs116710023
rs11935742
rs7941822
rs9282858
rs6850853
rs111471249
rs137918587
rs193261085
rs116928828
rs6867115
rs62299542
rs148982377
rs167139
All
−0.075
−0.050
−0.025
0.000
MR leave−one−out sensitivity analysis for
'GCST90200651' on 'Calculus of kidney and ureter'

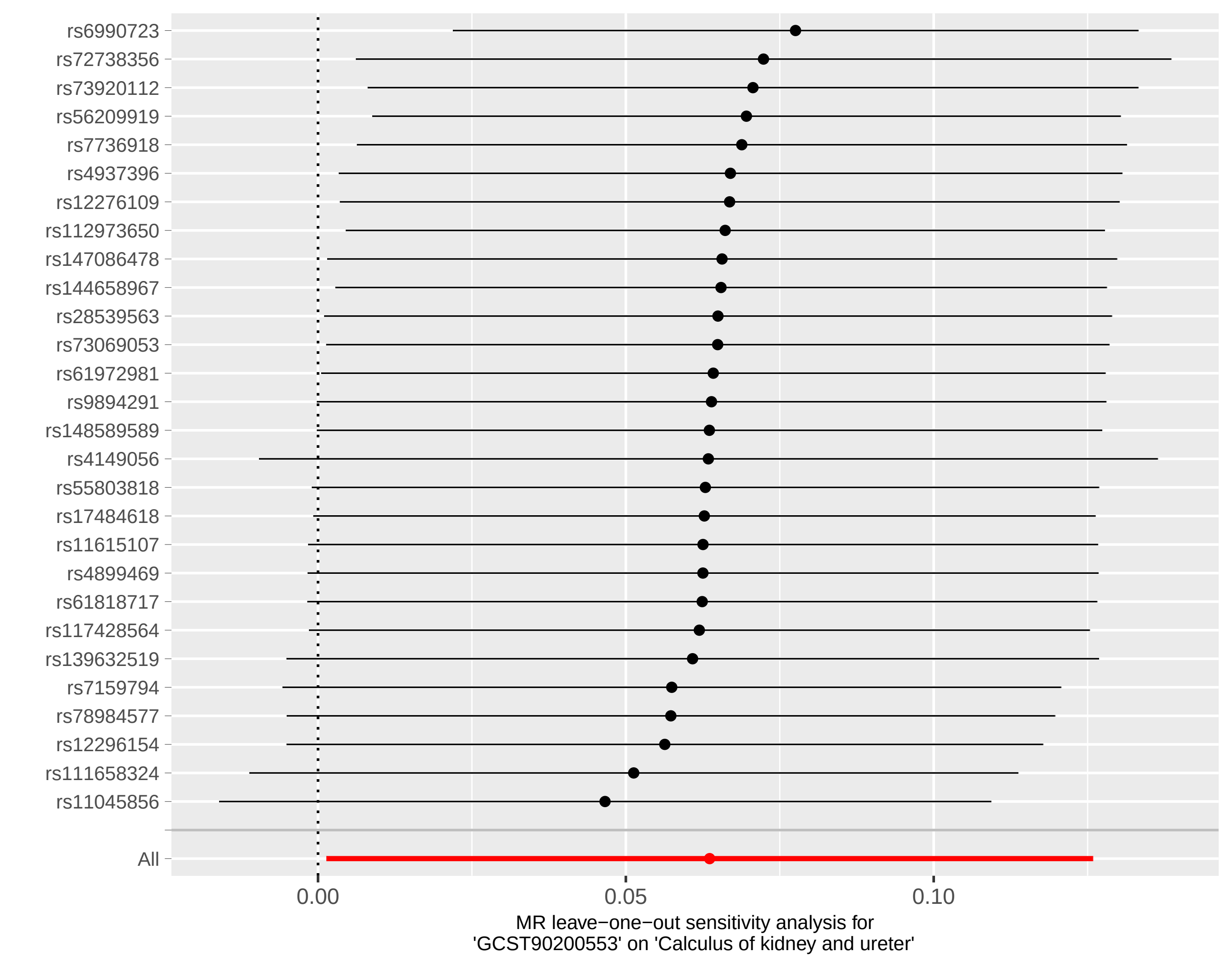
rs6990723
rs72738356
rs73920112
rs56209919
rs7736918
rs4937396
rs12276109
rs112973650
rs147086478
rs144658967
rs28539563
rs73069053
rs61972981
rs9894291
rs148589589
rs4149056
rs55803818
rs17484618
rs11615107
rs4899469
rs61818717
rs117428564
rs139632519
rs7159794
rs78984577
rs12296154
rs111658324
rs11045856
All
0.00
0.05
0.10
MR leave−one−out sensitivity analysis for
'GCST90200553' on 'Calculus of kidney and ureter'

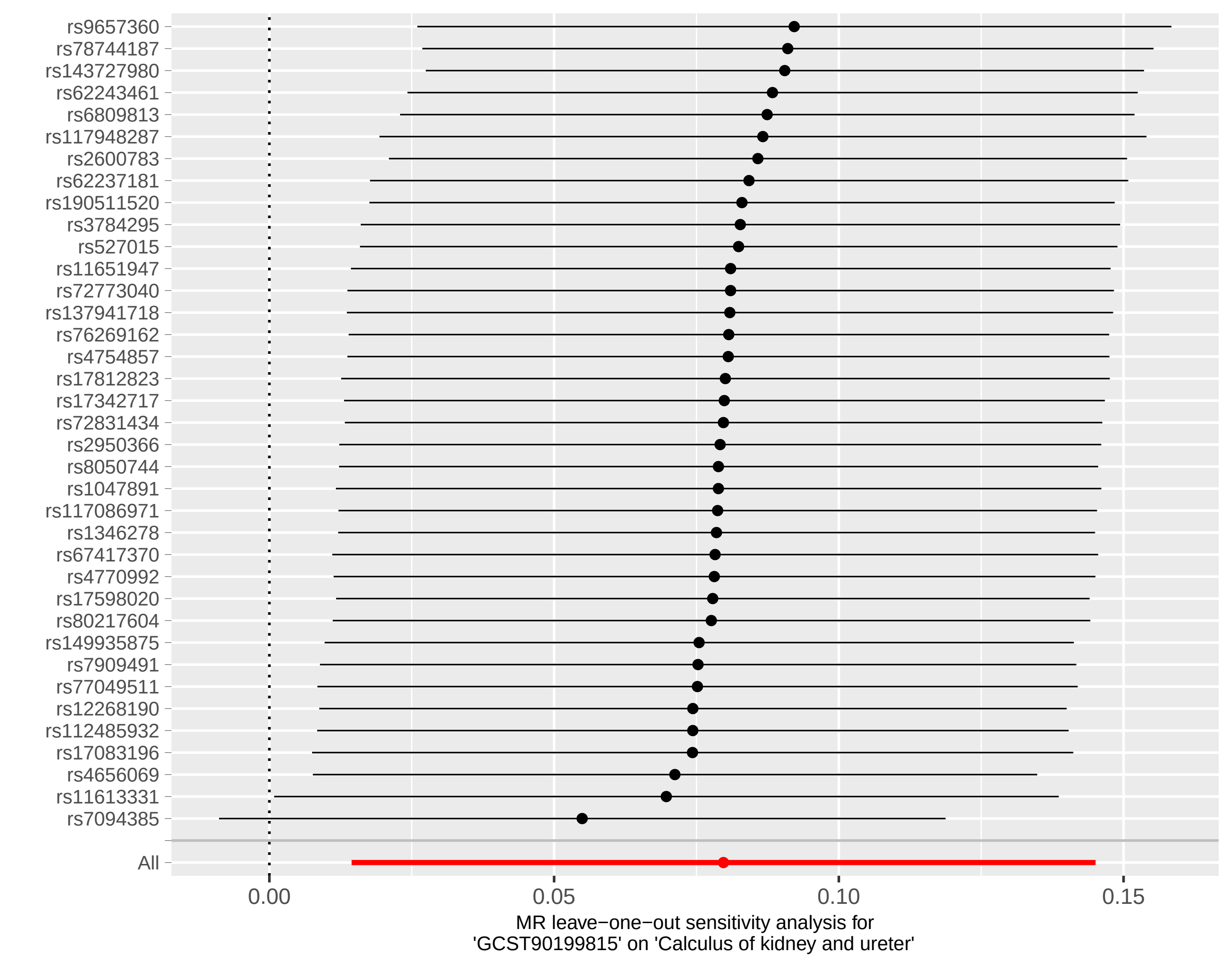
rs9657360
rs78744187
rs143727980
rs62243461
rs6809813
rs117948287
rs2600783
rs62237181
rs190511520
rs3784295
rs527015
rs11651947
rs72773040
rs137941718
rs76269162
rs4754857
rs17812823
rs17342717
rs72831434
rs2950366
rs8050744
rs1047891
rs117086971
rs1346278
rs67417370
rs4770992
rs17598020
rs80217604
rs149935875
rs7909491
rs77049511
rs12268190
rs112485932
rs17083196
rs4656069
rs11613331
rs7094385
All
0.00
0.05
0.10
0.15
MR leave−one−out sensitivity analysis for
'GCST90199815' on 'Calculus of kidney and ureter'

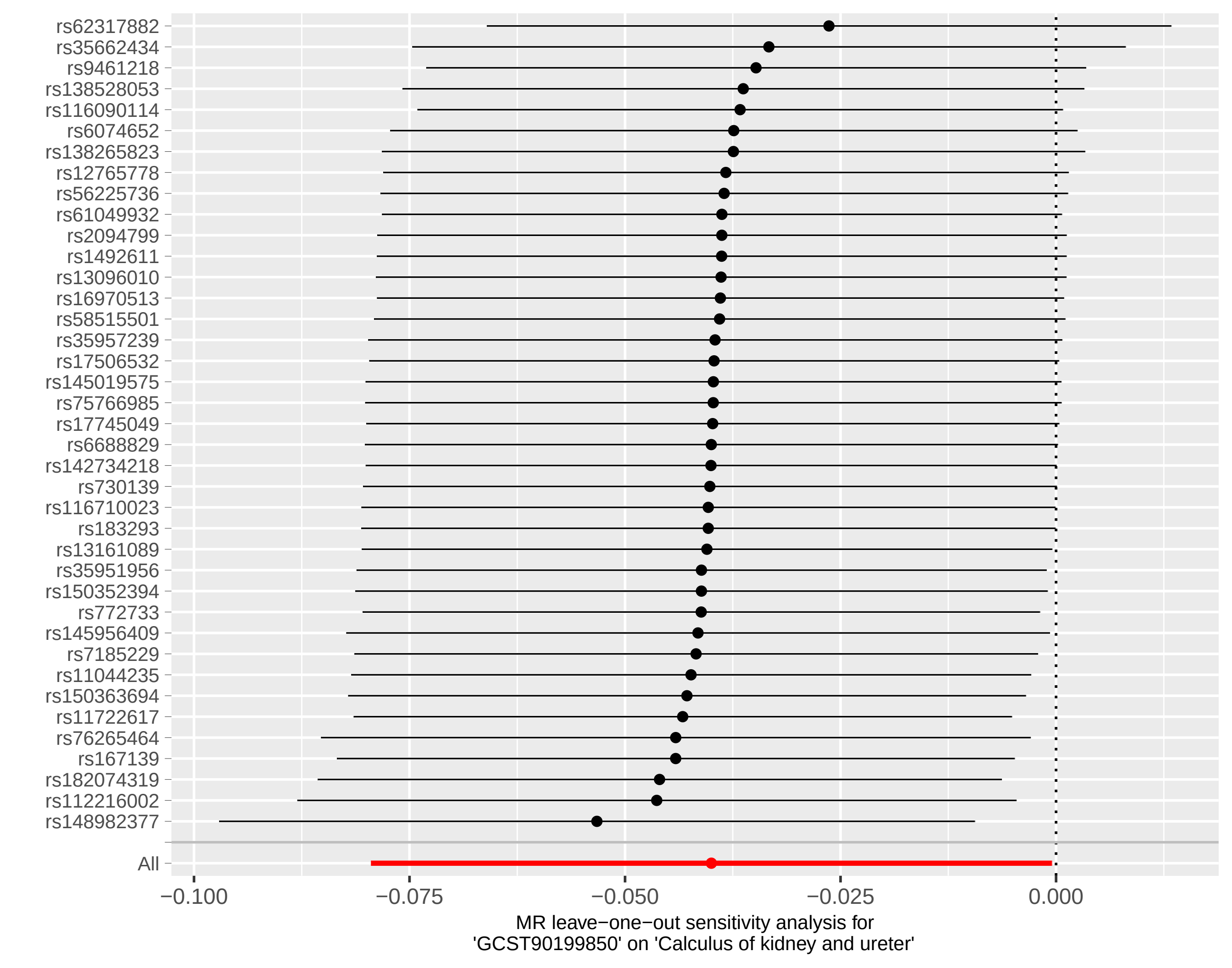
rs62317882
rs35662434
rs9461218
rs138528053
rs116090114
rs6074652
rs138265823
rs12765778
rs56225736
rs61049932
rs2094799
rs1492611
rs13096010
rs16970513
rs58515501
rs35957239
rs17506532
rs145019575
rs75766985
rs17745049
rs6688829
rs142734218
rs730139
rs116710023
rs183293
rs13161089
rs35951956
rs150352394
rs772733
rs145956409
rs7185229
rs11044235
rs150363694
rs11722617
rs76265464
rs167139
rs182074319
rs112216002
rs148982377
All
−0.100
−0.075
−0.050
−0.025
0.000
MR leave−one−out sensitivity analysis for
'GCST90199850' on 'Calculus of kidney and ureter'

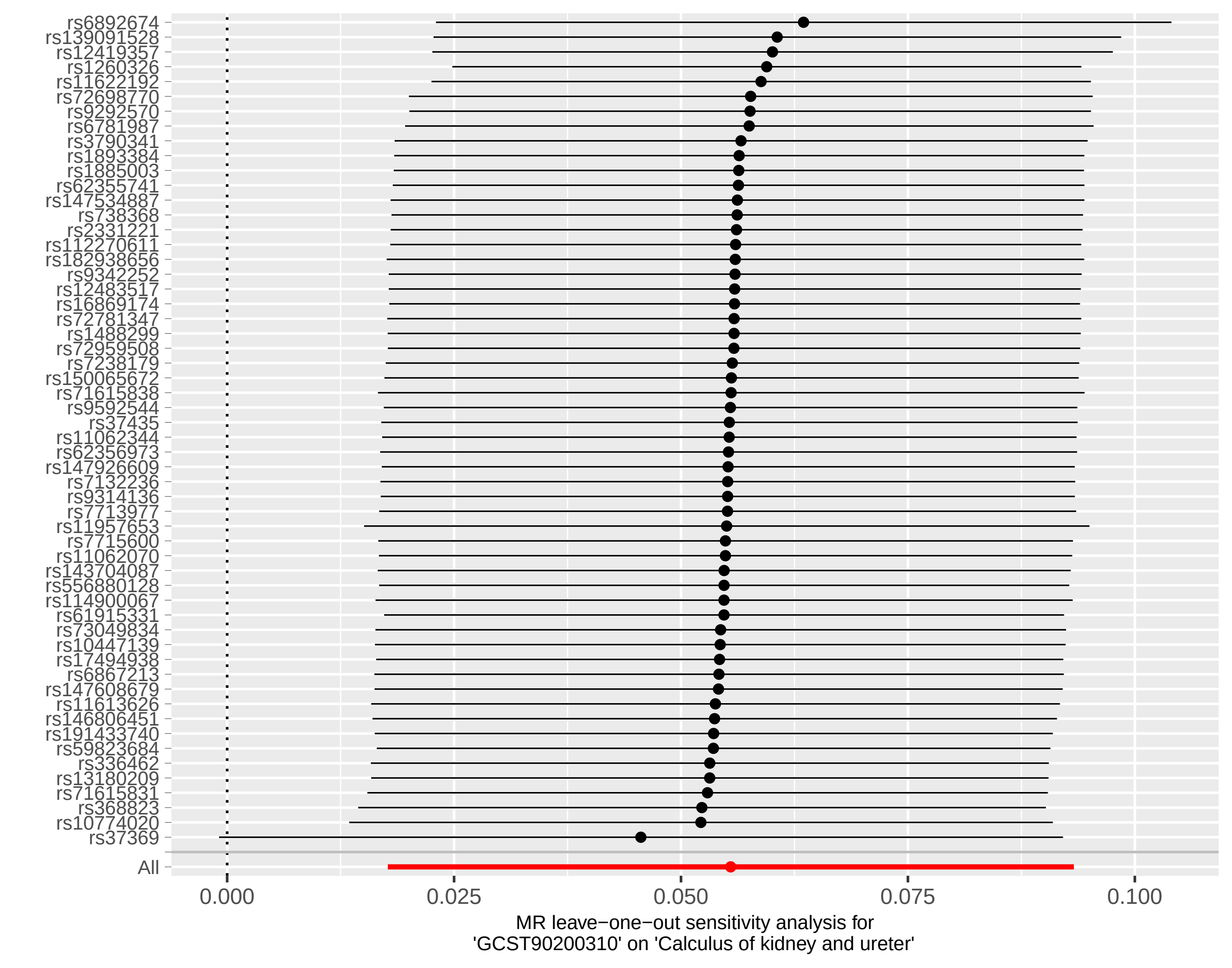
rs6892674
rs139091528
rs12419357
rs1260326
rs11622192
rs72698770
rs9292570
rs6781987
rs3790341
rs1893384
rs1885003
rs62355741
rs147534887
rs738368
rs2331221
rs112270611
rs182938656
rs9342252
rs12483517
rs16869174
rs72781347
rs1488299
rs72959508
rs7238179
rs150065672
rs71615838
rs9592544
rs37435
rs11062344
rs62356973
rs147926609
rs7132236
rs9314136
rs7713977
rs11957653
rs7715600
rs11062070
rs143704087
rs556880128
rs114900067
rs61915331
rs73049834
rs10447139
rs17494938
rs6867213
rs147608679
rs11613626
rs146806451
rs191433740
rs59823684
rs336462
rs13180209
rs71615831
rs368823
rs10774020
rs37369
All
0.000
0.025
0.050
0.075
0.100
MR leave−one−out sensitivity analysis for
'GCST90200310' on 'Calculus of kidney and ureter'

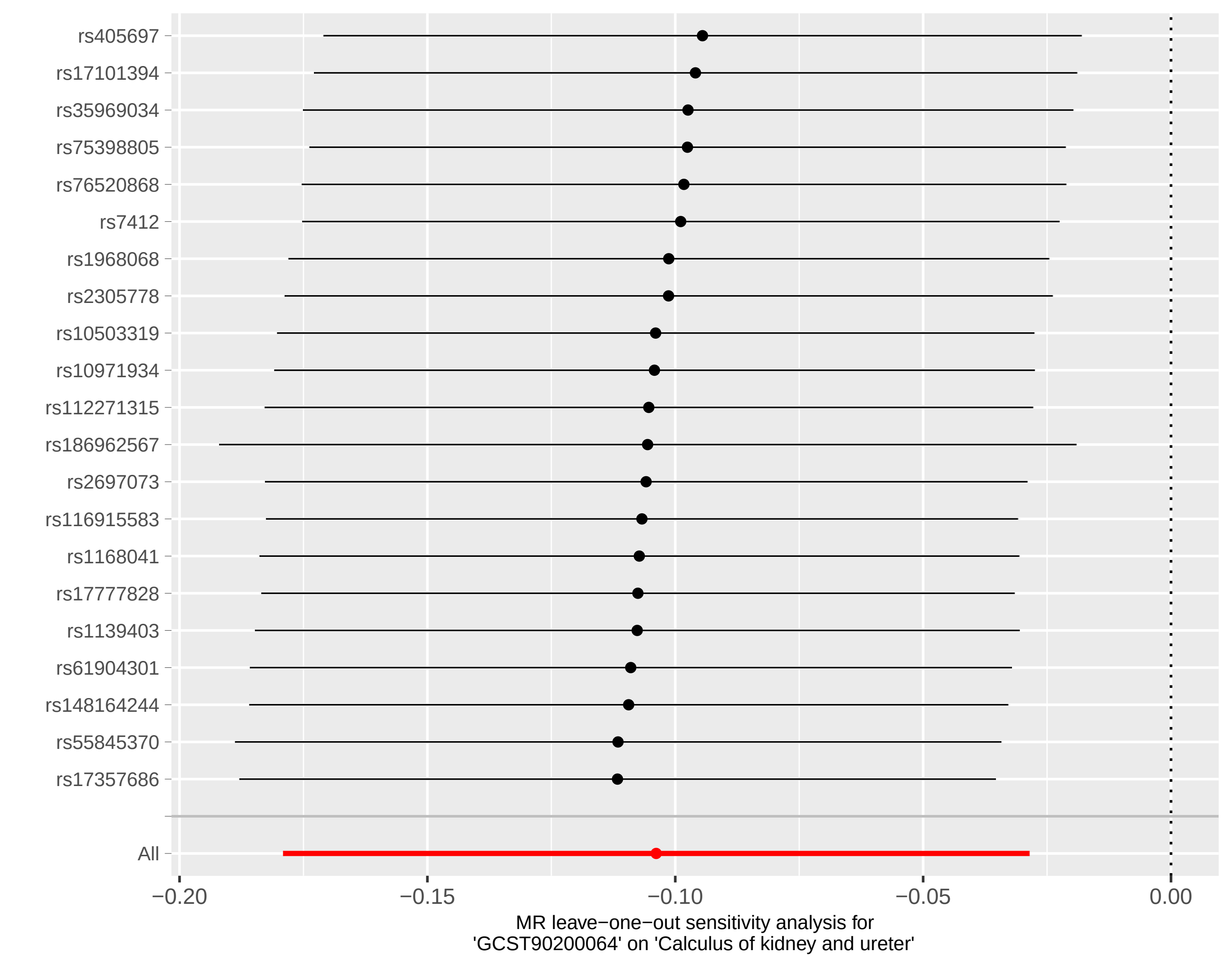

rs405697
rs17101394
rs35969034
rs75398805
rs76520868
rs7412
rs1968068
rs2305778
rs10503319
rs10971934
rs112271315
rs186962567
rs2697073
rs116915583
rs1168041
rs17777828
rs1139403
rs61904301
rs148164244
rs55845370
rs17357686
All
−0.20
−0.15
−0.10
−0.05
0.00
MR leave−one−out sensitivity analysis for
'GCST90200064' on 'Calculus of kidney and ureter'

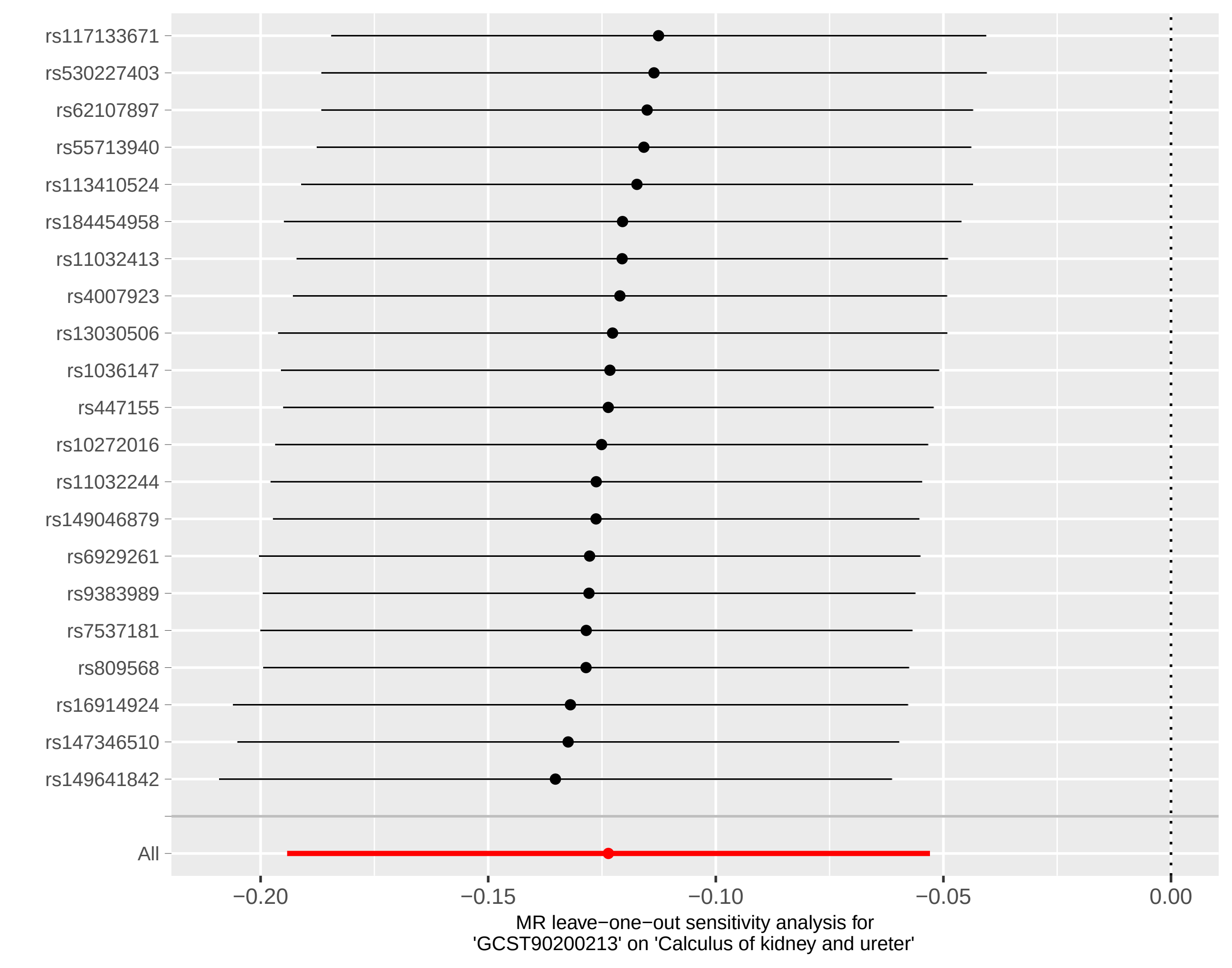
rs117133671
rs530227403
rs62107897
rs55713940
rs113410524
rs184454958
rs11032413
rs4007923
rs13030506
rs1036147
rs447155
rs10272016
rs11032244
rs149046879
rs6929261
rs9383989
rs7537181
rs809568
rs16914924
rs147346510
rs149641842
All
−0.20
−0.15
−0.10
−0.05
0.00
MR leave−one−out sensitivity analysis for
'GCST90200213' on 'Calculus of kidney and ureter'

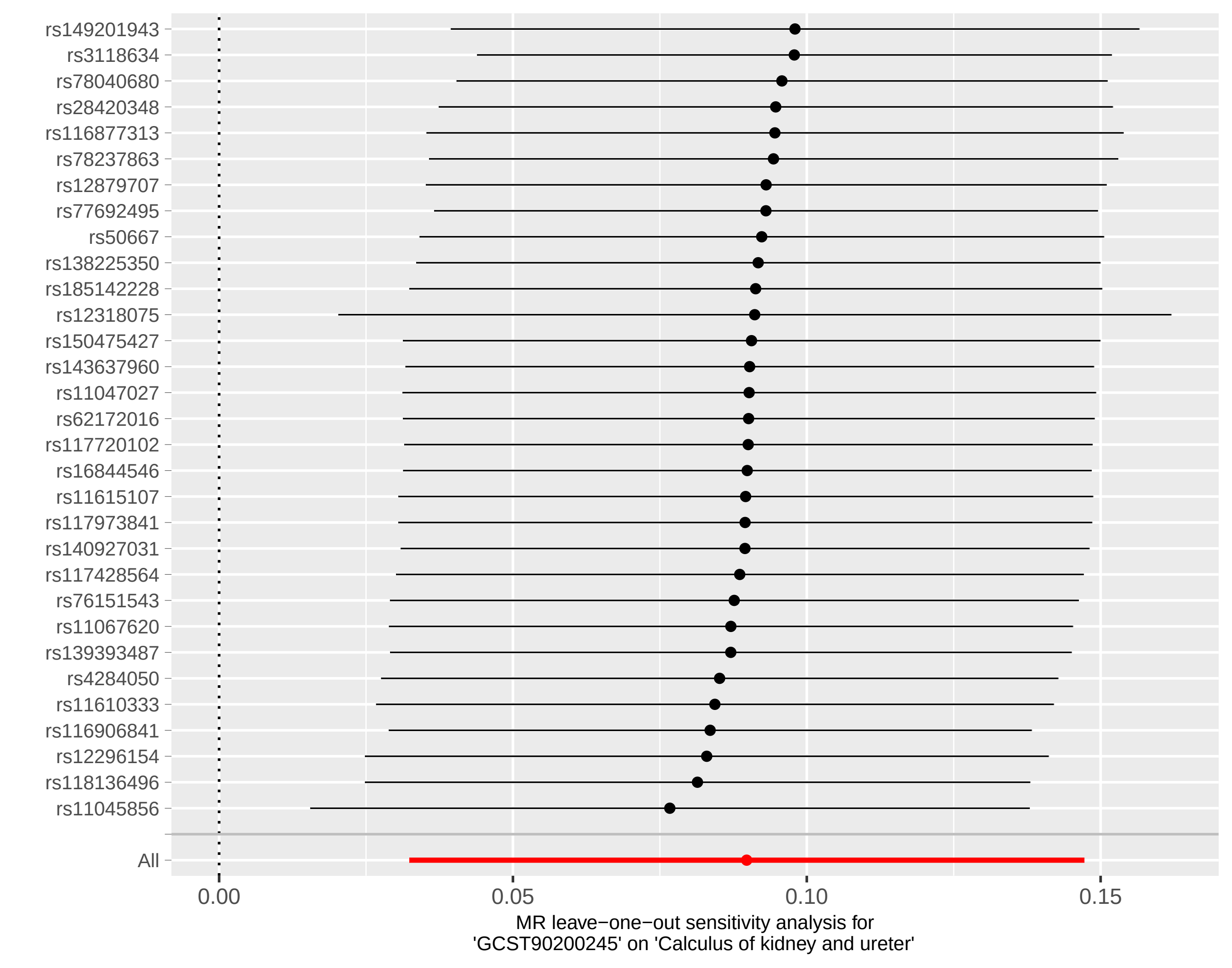
rs149201943
rs3118634
rs78040680
rs28420348
rs116877313
rs78237863
rs12879707
rs77692495
rs50667
rs138225350
rs185142228
rs12318075
rs150475427
rs143637960
rs11047027
rs62172016
rs117720102
rs16844546
rs11615107
rs117973841
rs140927031
rs117428564
rs76151543
rs11067620
rs139393487
rs4284050
rs11610333
rs116906841
rs12296154
rs118136496
rs11045856
All
0.00
0.05
0.10
0.15
MR leave−one−out sensitivity analysis for
'GCST90200245' on 'Calculus of kidney and ureter'

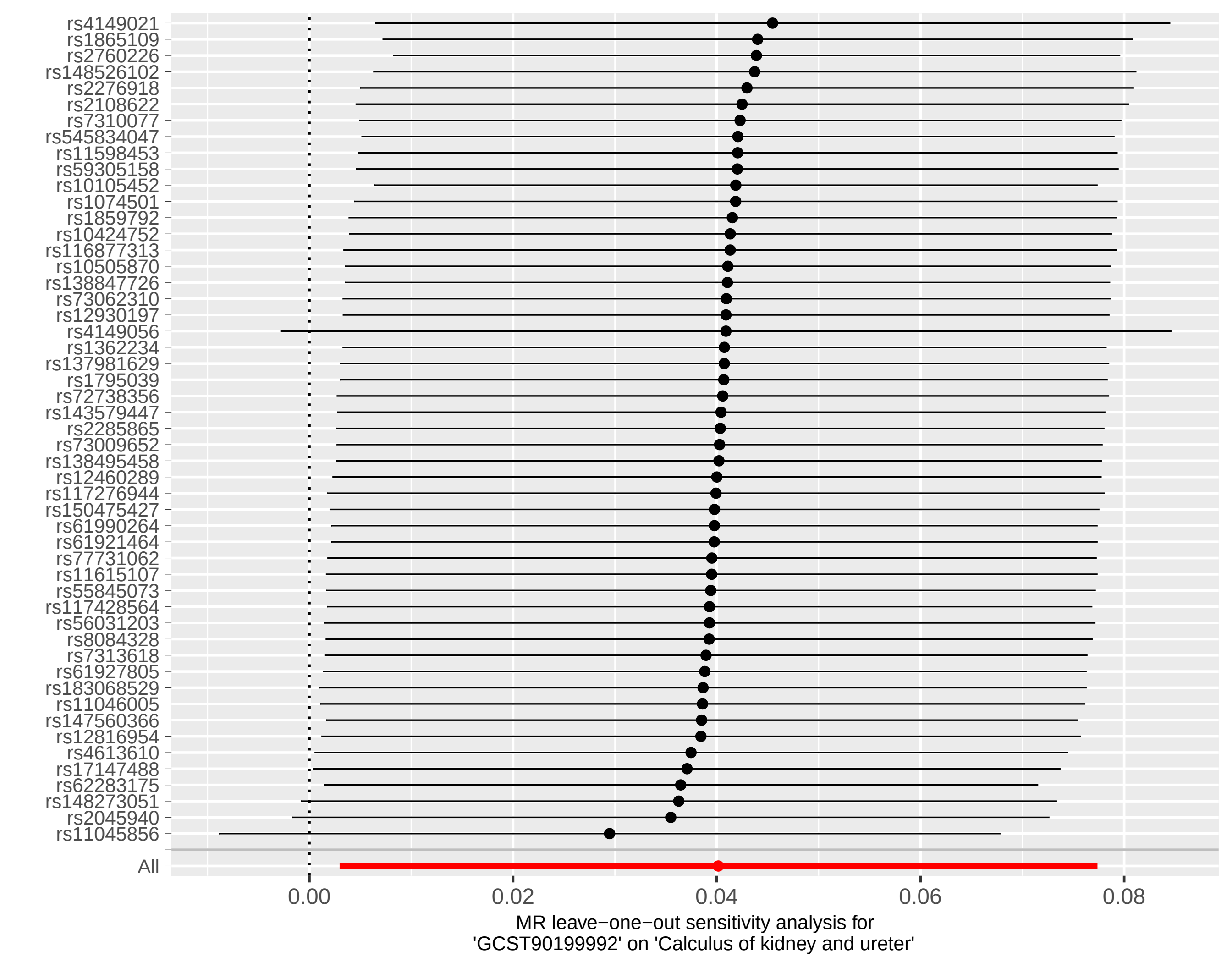

rs4149021
rs1865109
rs2760226
rs148526102
rs2276918
rs2108622
rs7310077
rs545834047
rs11598453
rs59305158
rs10105452
rs1074501
rs1859792
rs10424752
rs116877313
rs10505870
rs138847726
rs73062310
rs12930197
rs4149056
rs1362234
rs137981629
rs1795039
rs72738356
rs143579447
rs2285865
rs73009652
rs138495458
rs12460289
rs117276944
rs150475427
rs61990264
rs61921464
rs77731062
rs11615107
rs55845073
rs117428564
rs56031203
rs8084328
rs7313618
rs61927805
rs183068529
rs11046005
rs147560366
rs12816954
rs4613610
rs17147488
rs62283175
rs148273051
rs2045940
rs11045856
All
0.00
0.02
0.04
0.06
0.08
MR leave−one−out sensitivity analysis for
'GCST90199992' on 'Calculus of kidney and ureter'

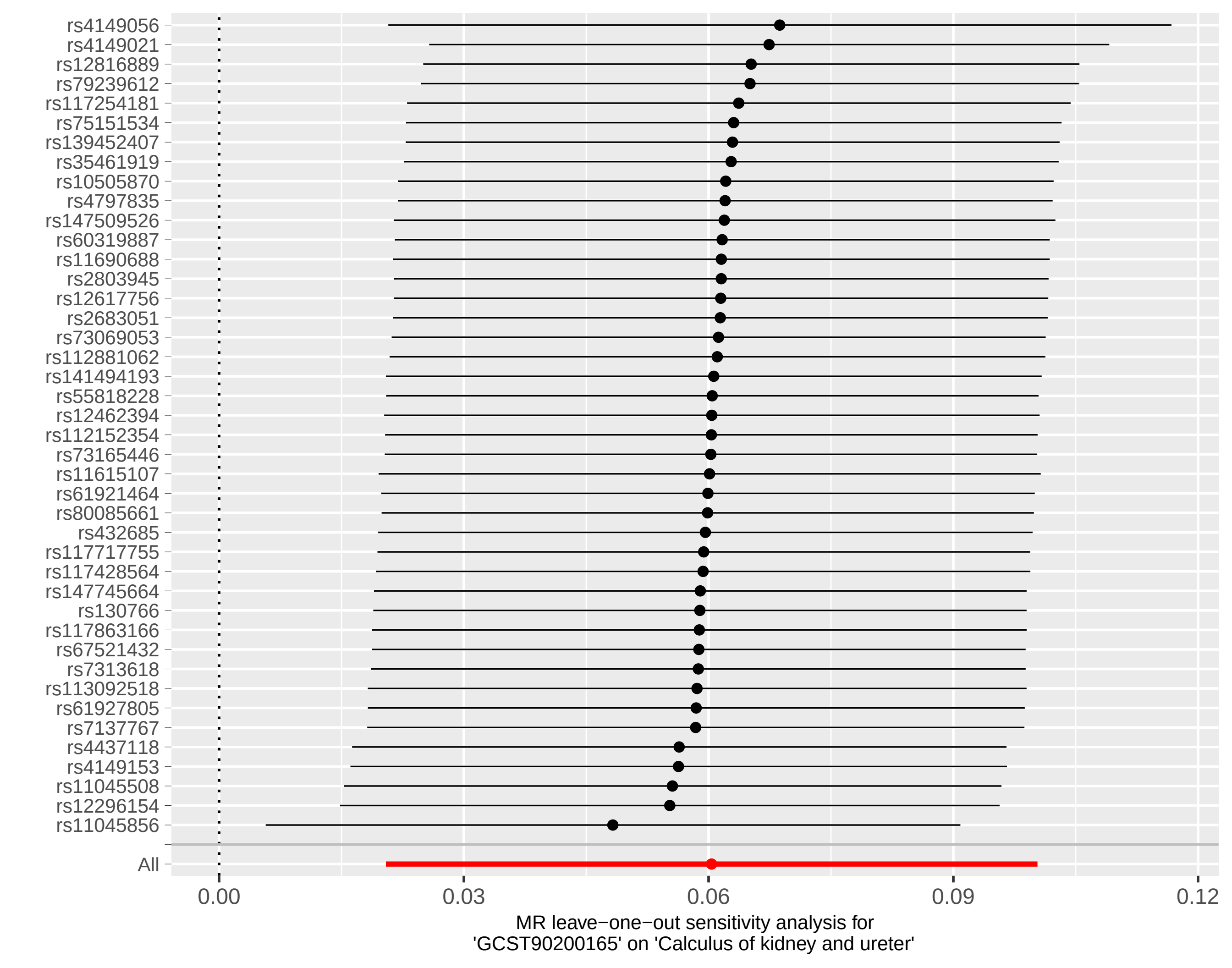

rs4149056
rs4149021
rs12816889
rs79239612
rs117254181
rs75151534
rs139452407
rs35461919
rs10505870
rs4797835
rs147509526
rs60319887
rs11690688
rs2803945
rs12617756
rs2683051
rs73069053
rs112881062
rs141494193
rs55818228
rs12462394
rs112152354
rs73165446
rs11615107
rs61921464
rs80085661
rs432685
rs117717755
rs117428564
rs147745664
rs130766
rs117863166
rs67521432
rs7313618
rs113092518
rs61927805
rs7137767
rs4437118
rs4149153
rs11045508
rs12296154
rs11045856
All
0.00
0.03
0.06
0.09
0.12
MR leave−one−out sensitivity analysis for
'GCST90200165' on 'Calculus of kidney and ureter'

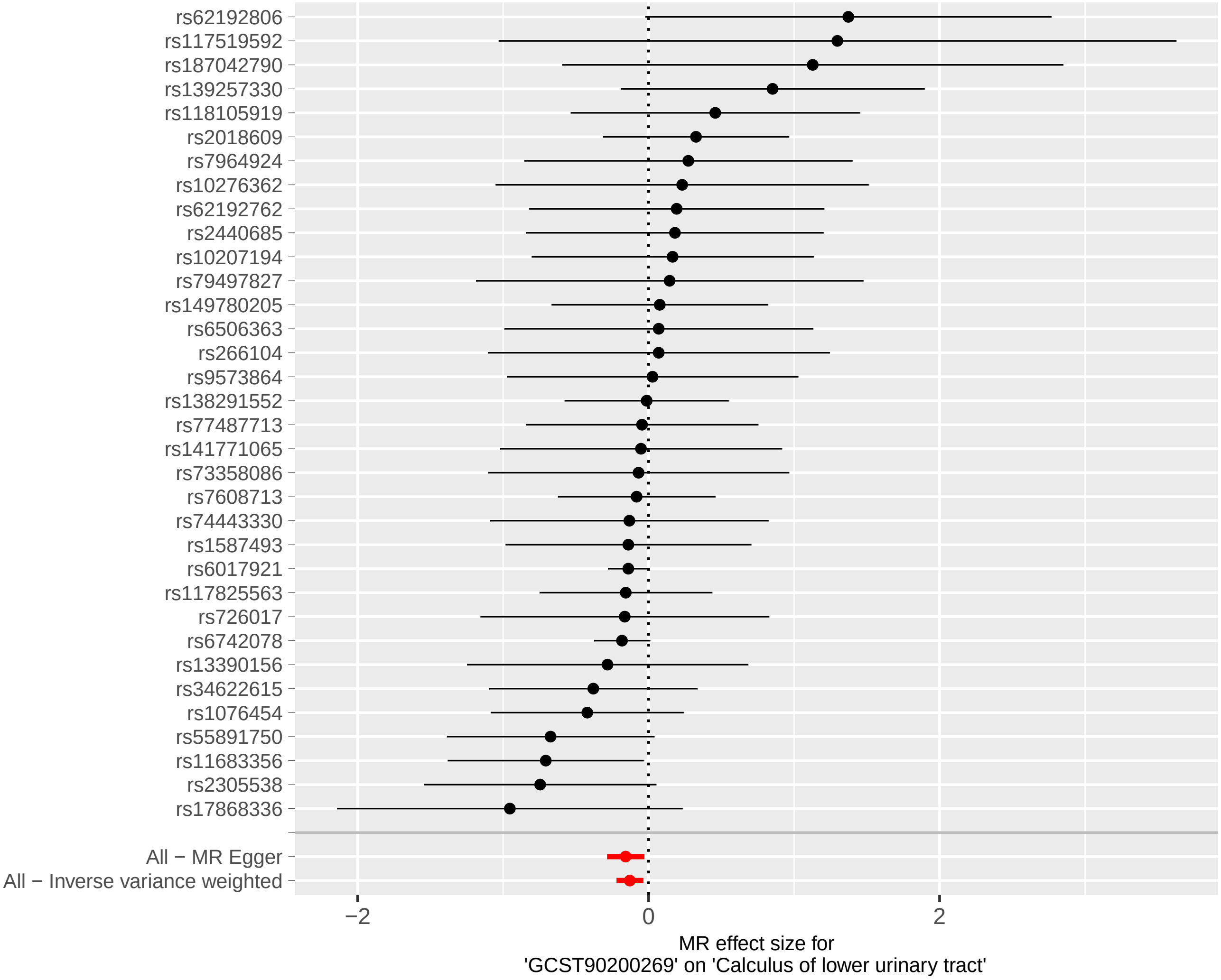
rs62192806
rs117519592
rs187042790
rs139257330
rs118105919
rs2018609
rs7964924
rs10276362
rs62192762
rs2440685
rs10207194
rs79497827
rs149780205
rs6506363
rs266104
rs9573864
rs138291552
rs77487713
rs141771065
rs73358086
rs7608713
rs74443330
rs1587493
rs6017921
rs117825563
rs726017
rs6742078
rs13390156
rs34622615
rs1076454
rs55891750
rs11683356
rs2305538
rs17868336
All − MR Egger
All − Inverse variance weighted
−2
0
2
MR effect size for
'GCST90200269' on 'Calculus of lower urinary tract'

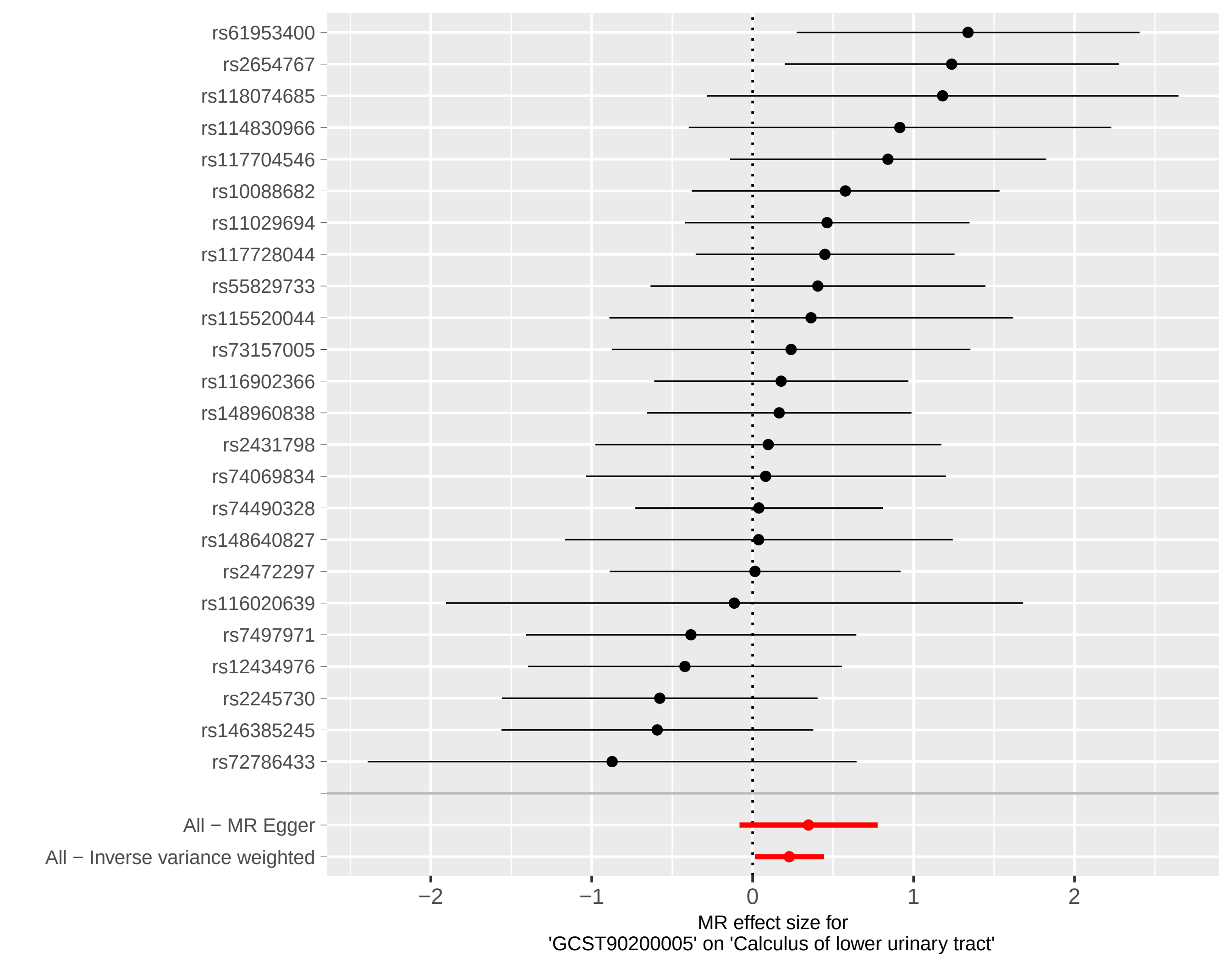
rs61953400
rs2654767
rs118074685
rs114830966
rs117704546
rs10088682
rs11029694
rs117728044
rs55829733
rs115520044
rs73157005
rs116902366
rs148960838
rs2431798
rs74069834
rs74490328
rs148640827
rs2472297
rs116020639
rs7497971
rs12434976
rs2245730
rs146385245
rs72786433
All − MR Egger
All − Inverse variance weighted
−2
−1
0
1
2
MR effect size for
'GCST90200005' on 'Calculus of lower urinary tract'

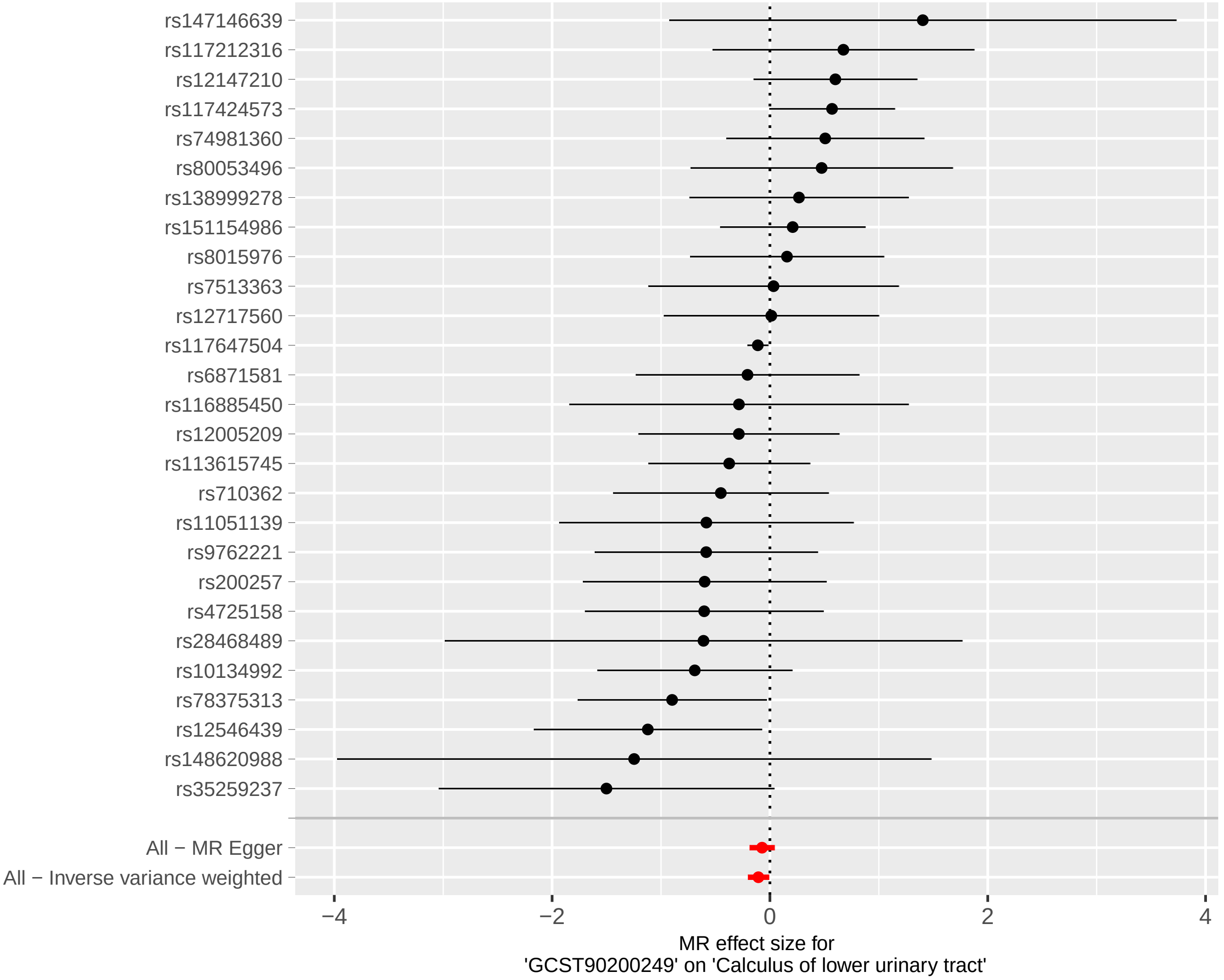
rs147146639
rs117212316
rs12147210
rs117424573
rs74981360
rs80053496
rs138999278
rs151154986
rs8015976
rs7513363
rs12717560
rs117647504
rs6871581
rs116885450
rs12005209
rs113615745
rs710362
rs11051139
rs9762221
rs200257
rs4725158
rs28468489
rs10134992
rs78375313
rs12546439
rs148620988
rs35259237
All − MR Egger
All − Inverse variance weighted
−4
−2
0
2
4
MR effect size for
'GCST90200249' on 'Calculus of lower urinary tract'

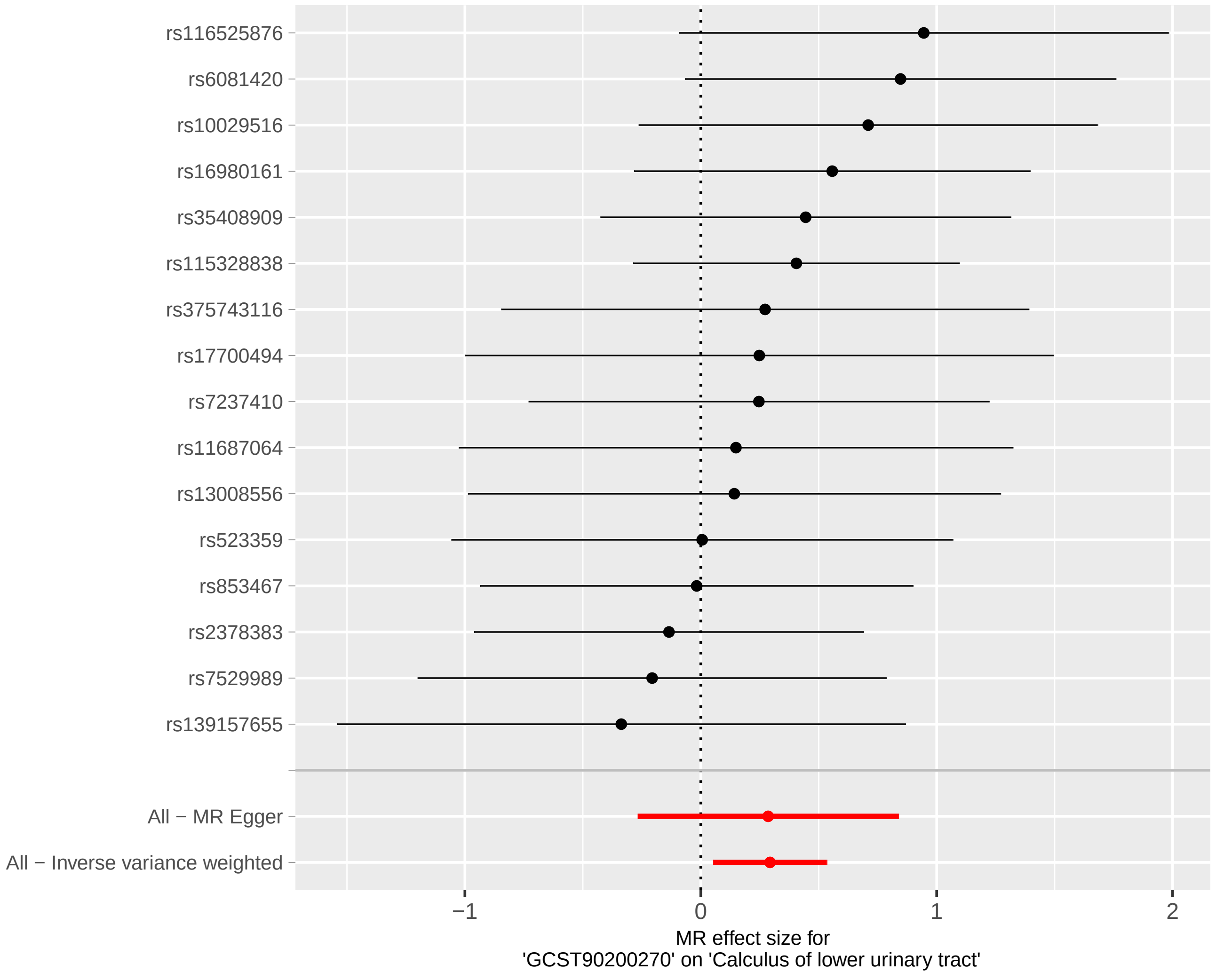
rs116525876
rs6081420
rs10029516
rs16980161
rs35408909
rs115328838
rs375743116
rs17700494
rs7237410
rs11687064
rs13008556
rs523359
rs853467
rs2378383
rs7529989
rs139157655
All − MR Egger
All − Inverse variance weighted
−1
0
1
2
MR effect size for
'GCST90200270' on 'Calculus of lower urinary tract'

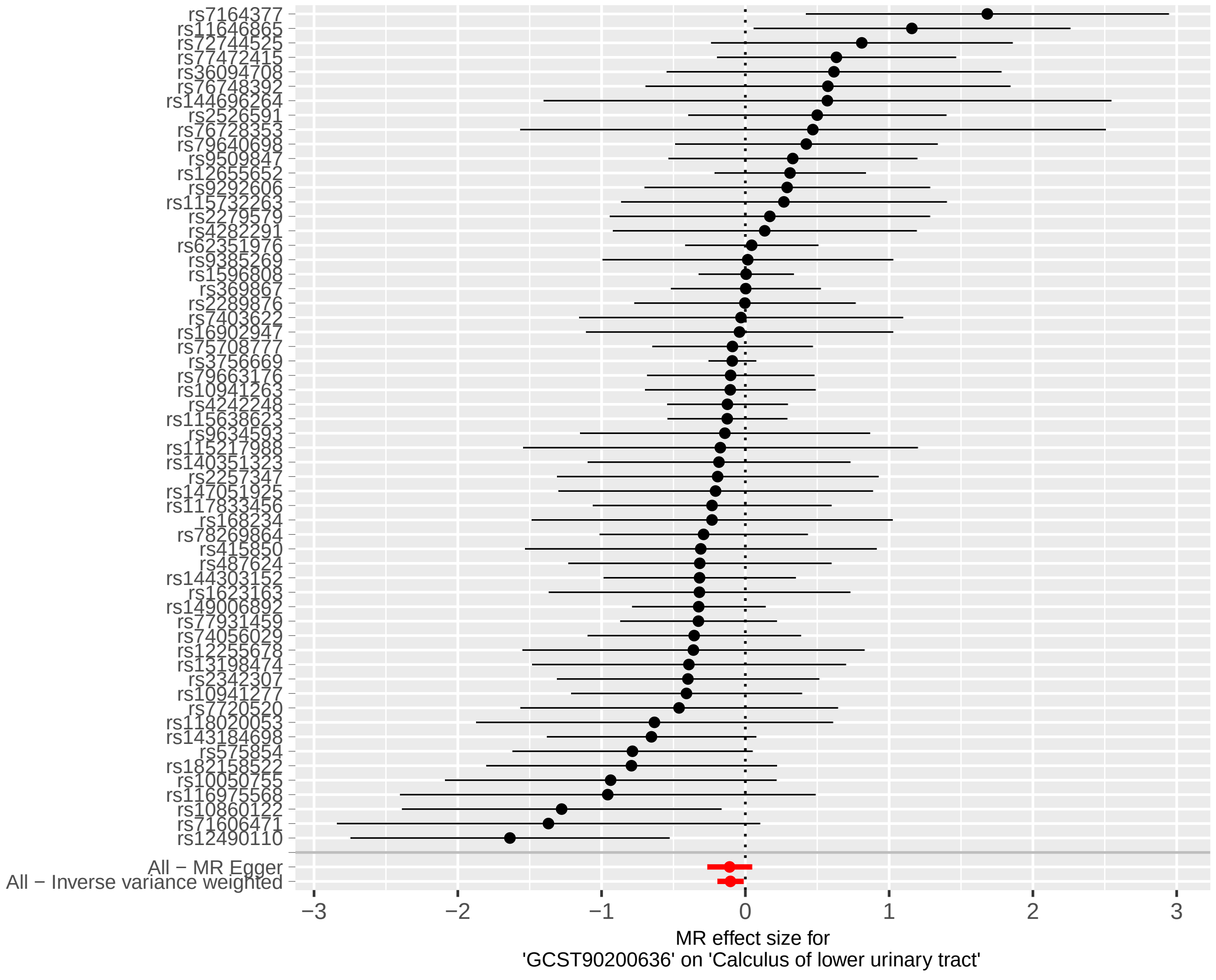
rs7164377
rs11646865
rs72744525
rs77472415
rs36094708
rs76748392
rs144696264
rs2526591
rs76728353
rs79640698
rs9509847
rs12655652
rs9292606
rs115732263
rs2279579
rs4282291
rs62351976
rs9385269
rs1596808
rs369867
rs2289876
rs7403622
rs16902947
rs75708777
rs3756669
rs79663176
rs10941263
rs4242248
rs115638623
rs9634593
rs115217988
rs140351323
rs2257347
rs147051925
rs117833456
rs168234
rs78269864
rs415850
rs487624
rs144303152
rs1623163
rs149006892
rs77931459
rs74056029
rs12255678
rs13198474
rs2342307
rs10941277
rs7720520
rs118020053
rs143184698
rs575854
rs182158522
rs10050755
rs116975568
rs10860122
rs71606471
rs12490110
All − MR Egger
All − Inverse variance weighted
−3
−2
−1
0
1
2
3
MR effect size for
'GCST90200636' on 'Calculus of lower urinary tract'

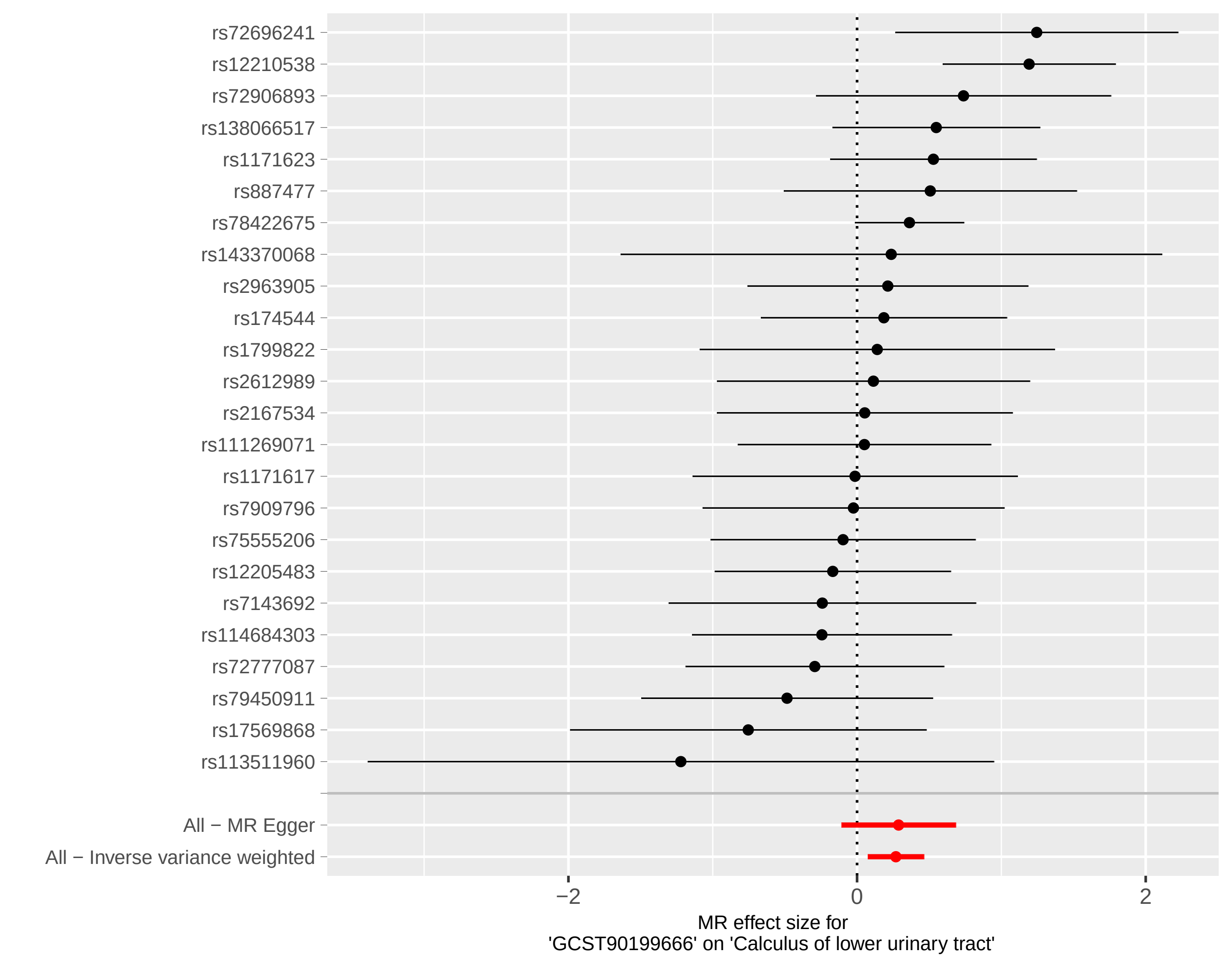
rs72696241
rs12210538
rs72906893
rs138066517
rs1171623
rs887477
rs78422675
rs143370068
rs2963905
rs174544
rs1799822
rs2612989
rs2167534
rs111269071
rs1171617
rs7909796
rs75555206
rs12205483
rs7143692
rs114684303
rs72777087
rs79450911
rs17569868
rs113511960
All − MR Egger
All − Inverse variance weighted
−2
0
2
MR effect size for
'GCST90199666' on 'Calculus of lower urinary tract'

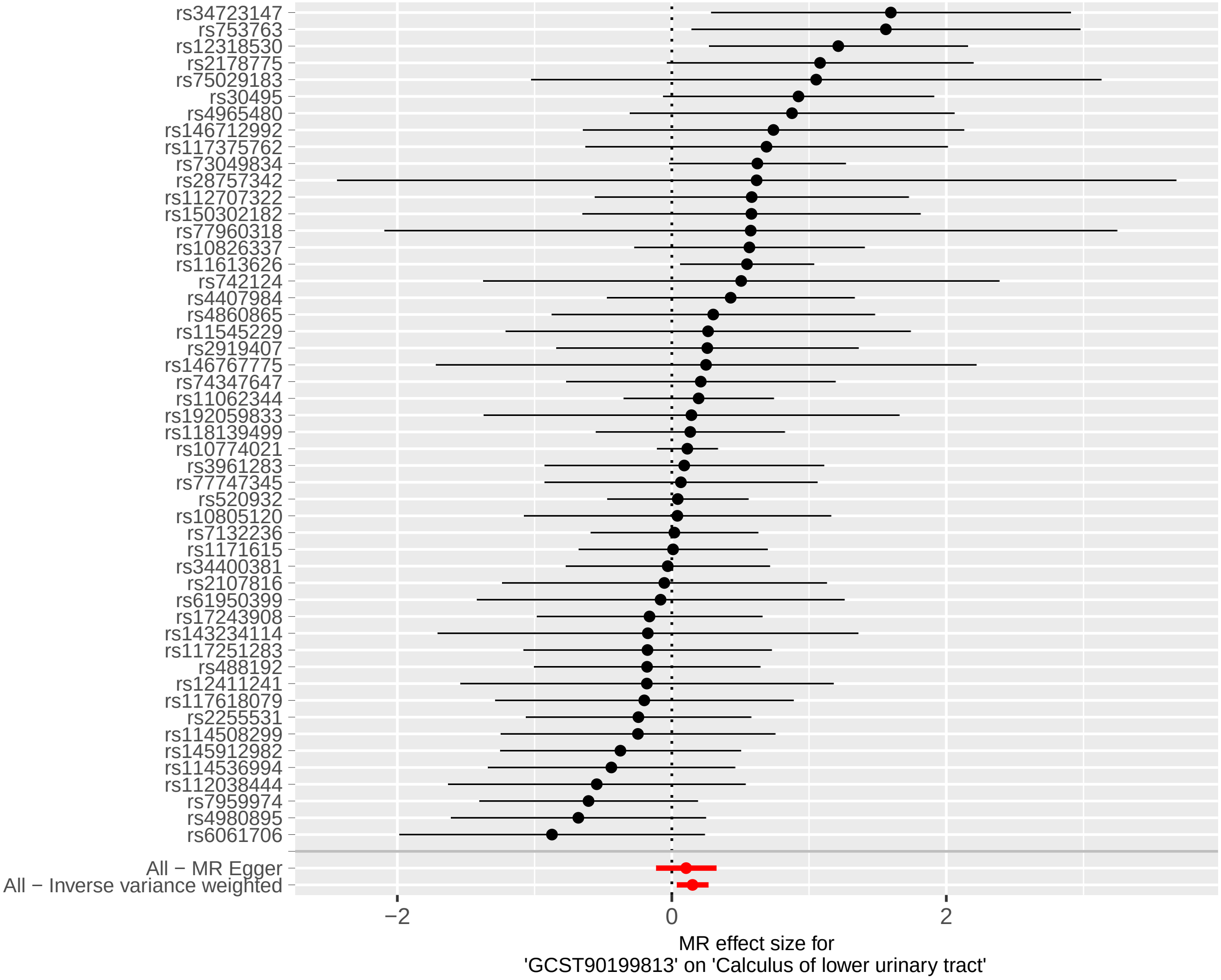
rs34723147
rs753763
rs12318530
rs2178775
rs75029183
rs30495
rs4965480
rs146712992
rs117375762
rs73049834
rs28757342
rs112707322
rs150302182
rs77960318
rs10826337
rs11613626
rs742124
rs4407984
rs4860865
rs11545229
rs2919407
rs146767775
rs74347647
rs11062344
rs192059833
rs118139499
rs10774021
rs3961283
rs77747345
rs520932
rs10805120
rs7132236
rs1171615
rs34400381
rs2107816
rs61950399
rs17243908
rs143234114
rs117251283
rs488192
rs12411241
rs117618079
rs2255531
rs114508299
rs145912982
rs114536994
rs112038444
rs7959974
rs4980895
rs6061706
All − MR Egger
All − Inverse variance weighted
−2
0
2
MR effect size for
'GCST90199813' on 'Calculus of lower urinary tract'

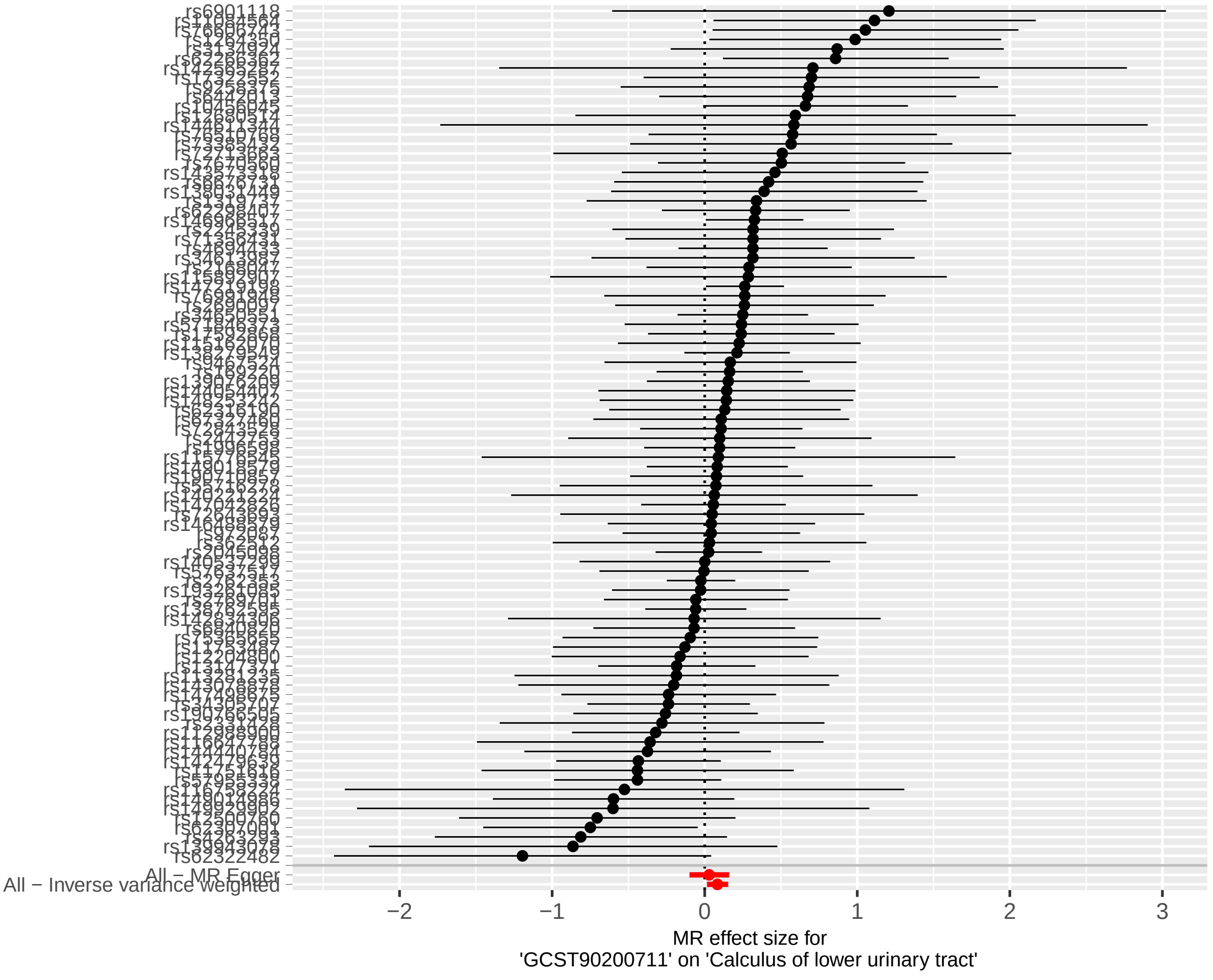

rs6901118
rs62322482
All − MR Egger
All − Inverse variance weighted
−2
−1
0
1
2
3
MR effect size for
'GCST90200711' on 'Calculus of lower urinary tract'

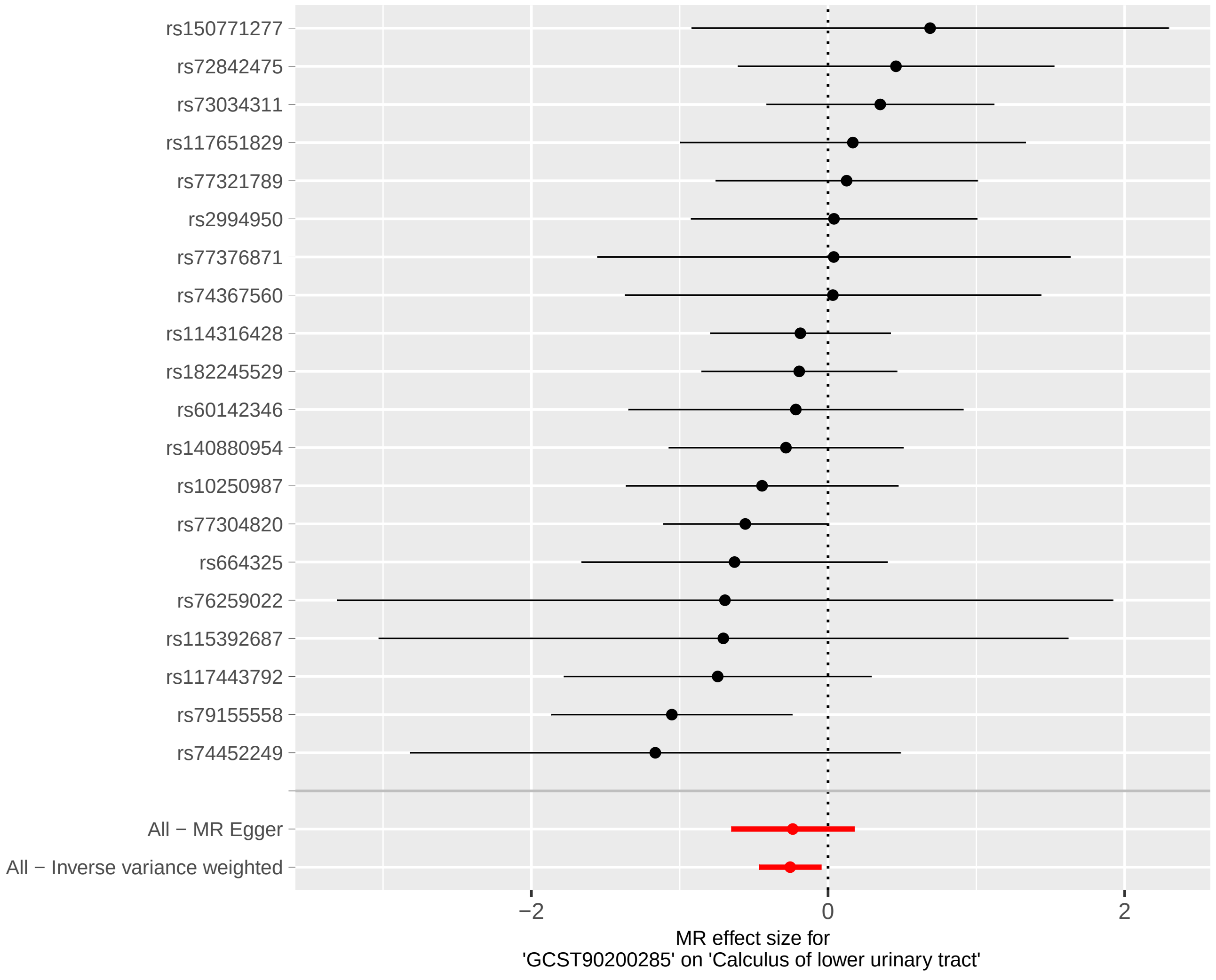

rs150771277
rs72842475
rs73034311
rs117651829
rs77321789
rs2994950
rs77376871
rs74367560
rs114316428
rs182245529
rs60142346
rs140880954
rs10250987
rs77304820
rs664325
rs76259022
rs115392687
rs117443792
rs79155558
rs74452249
All − MR Egger
All − Inverse variance weighted
−2
0
2
MR effect size for
'GCST90200285' on 'Calculus of lower urinary tract'

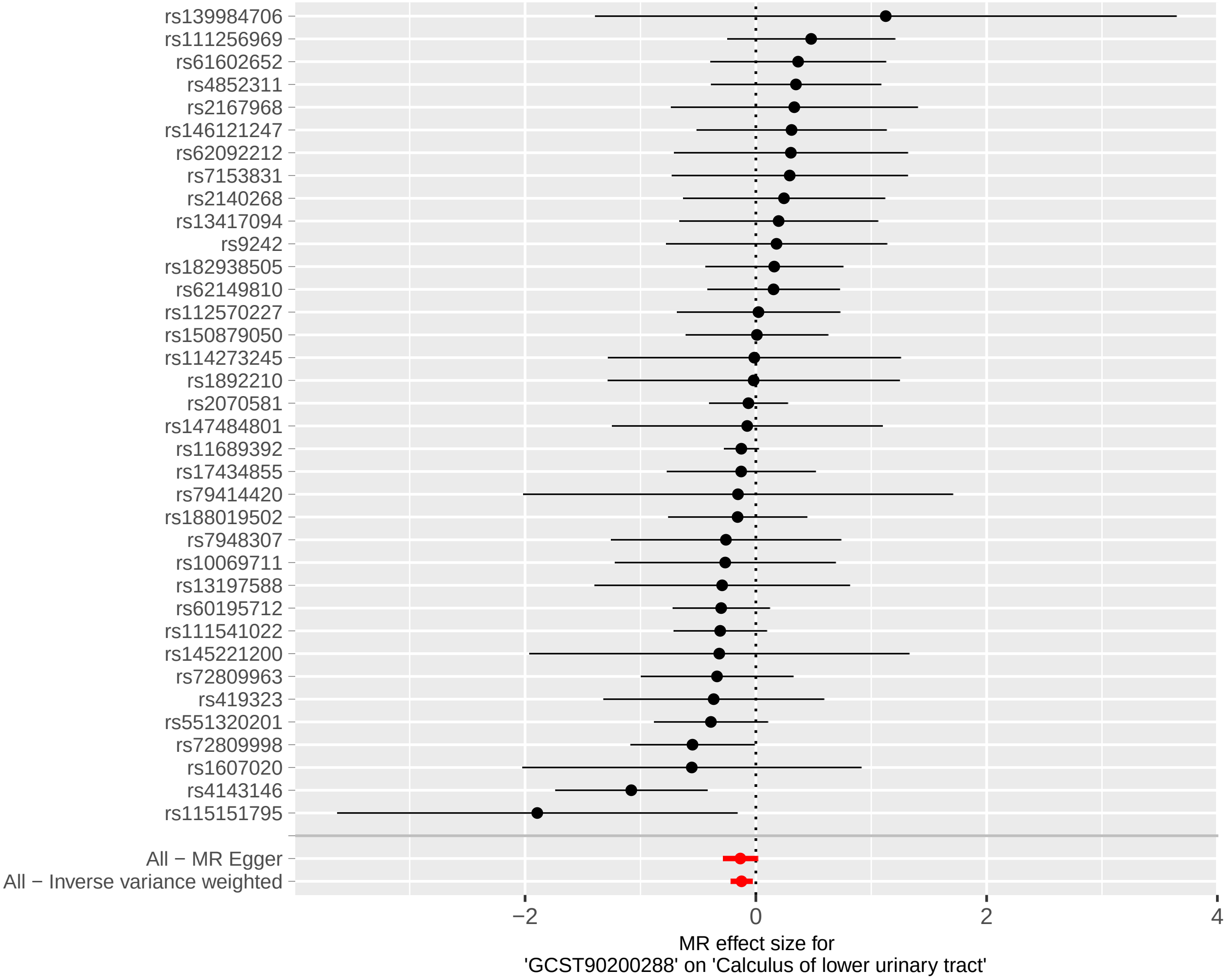

rs139984706
rs111256969
rs61602652
rs4852311
rs2167968
rs146121247
rs62092212
rs7153831
rs2140268
rs13417094
rs9242
rs182938505
rs62149810
rs112570227
rs150879050
rs114273245
rs1892210
rs2070581
rs147484801
rs11689392
rs17434855
rs79414420
rs188019502
rs7948307
rs10069711
rs13197588
rs60195712
rs111541022
rs145221200
rs72809963
rs419323
rs551320201
rs72809998
rs1607020
rs4143146
rs115151795
All − MR Egger
All − Inverse variance weighted
−2
0
2
4
MR effect size for
'GCST90200288' on 'Calculus of lower urinary tract'

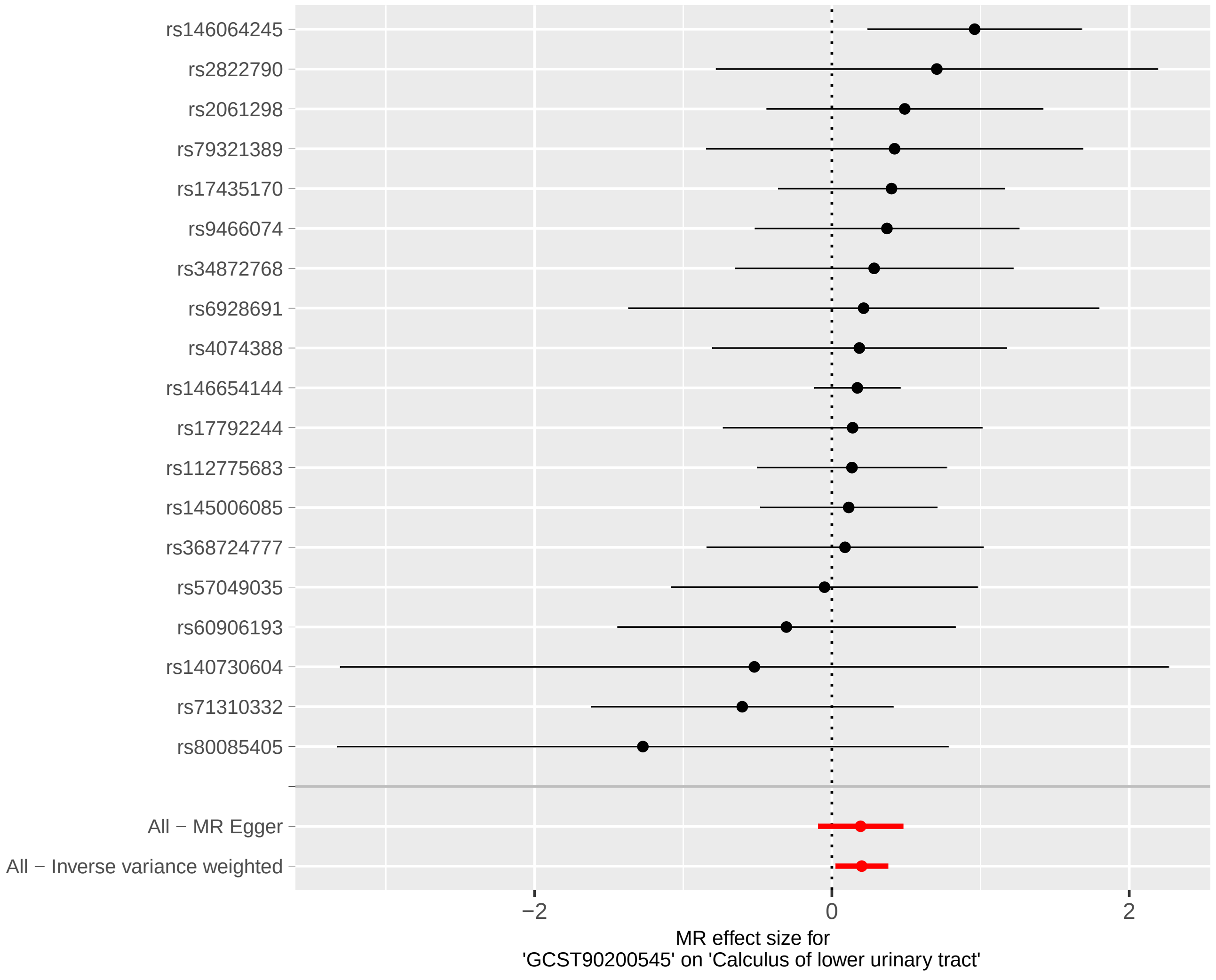
rs146064245
rs2822790
rs2061298
rs79321389
rs17435170
rs9466074
rs34872768
rs6928691
rs4074388
rs146654144
rs17792244
rs112775683
rs145006085
rs368724777
rs57049035
rs60906193
rs140730604
rs71310332
rs80085405
All − MR Egger
All − Inverse variance weighted
−2
0
2
MR effect size for
'GCST90200545' on 'Calculus of lower urinary tract'

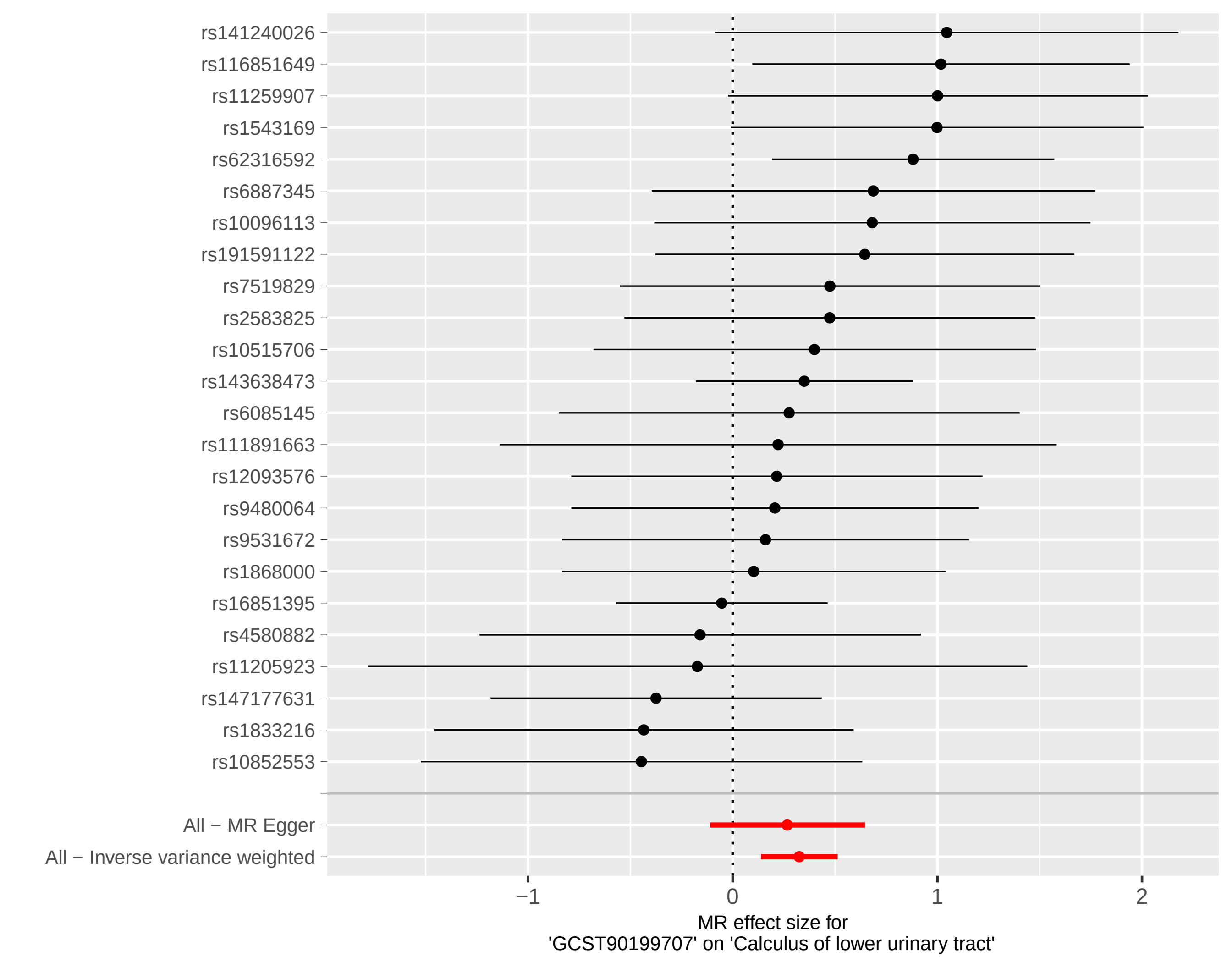

rs141240026
rs116851649
rs11259907
rs1543169
rs62316592
rs6887345
rs10096113
rs191591122
rs7519829
rs2583825
rs10515706
rs143638473
rs6085145
rs111891663
rs12093576
rs9480064
rs9531672
rs1868000
rs16851395
rs4580882
rs11205923
rs147177631
rs1833216
rs10852553
All − MR Egger
All − Inverse variance weighted
−1
0
1
2
MR effect size for
'GCST90199707' on 'Calculus of lower urinary tract'

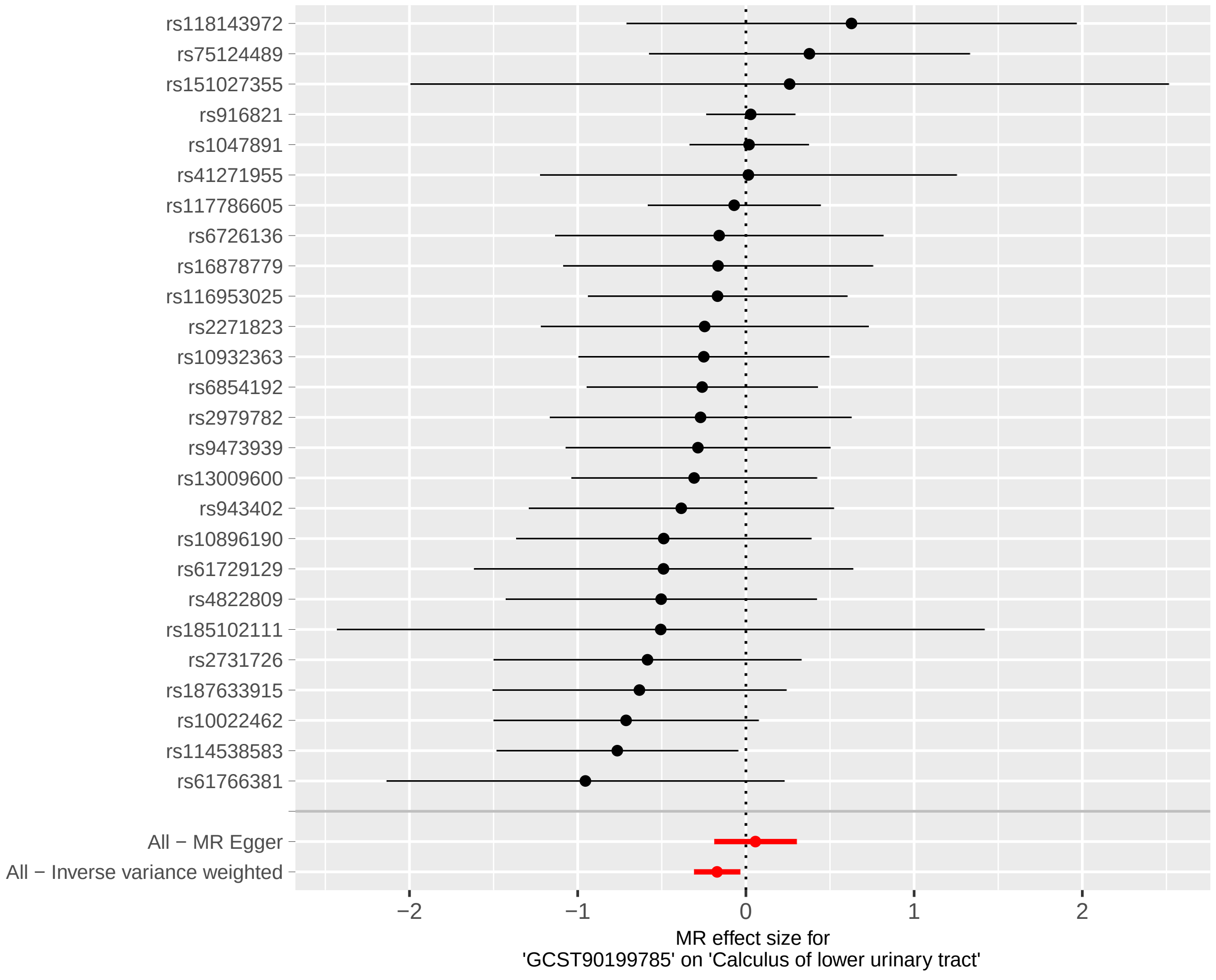
rs118143972
rs75124489
rs151027355
rs916821
rs1047891
rs41271955
rs117786605
rs6726136
rs16878779
rs116953025
rs2271823
rs10932363
rs6854192
rs2979782
rs9473939
rs13009600
rs943402
rs10896190
rs61729129
rs4822809
rs185102111
rs2731726
rs187633915
rs10022462
rs114538583
rs61766381
All − MR Egger
All − Inverse variance weighted
−2
−1
0
1
2
MR effect size for
'GCST90199785' on 'Calculus of lower urinary tract'

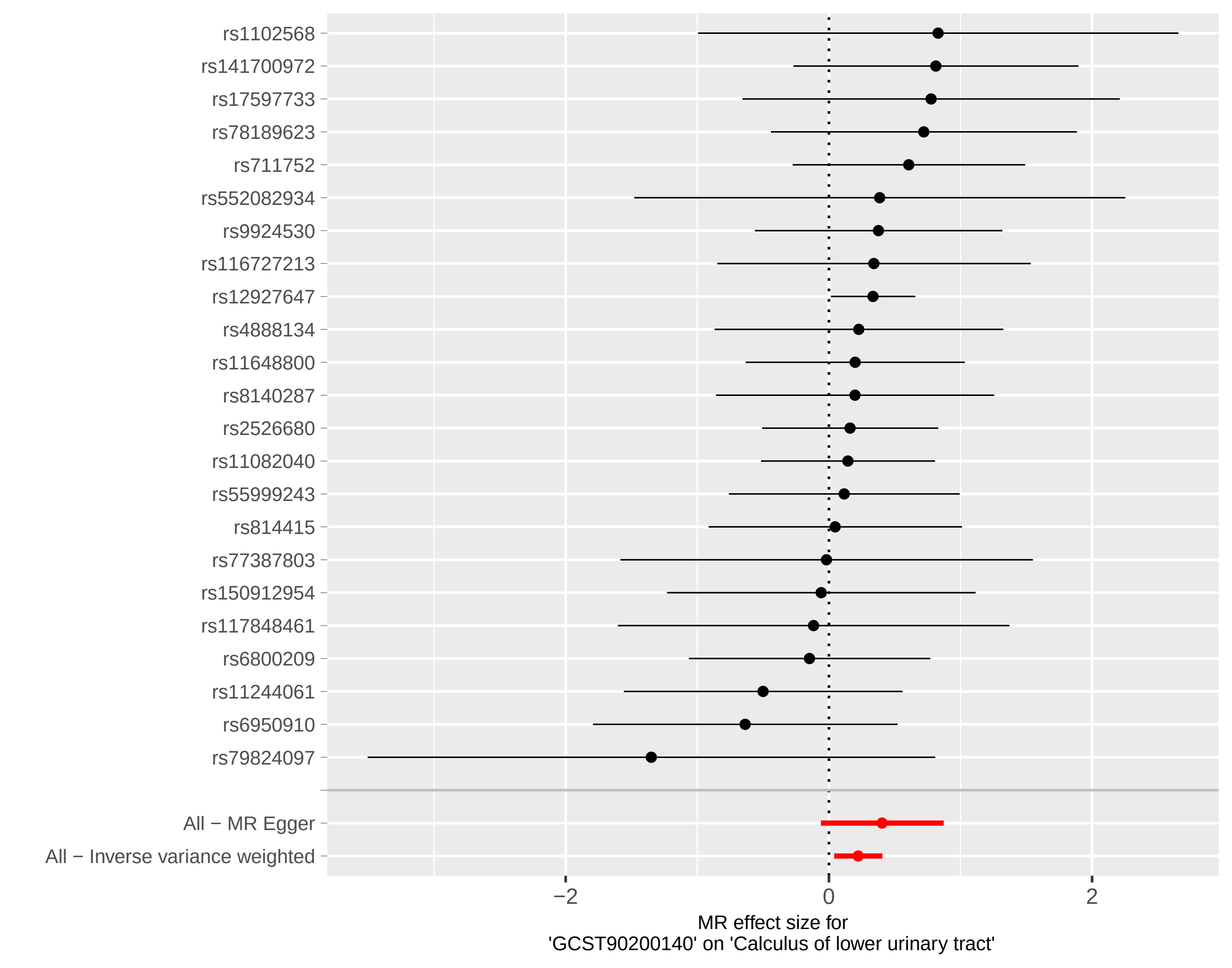

rs1102568
rs141700972
rs17597733
rs78189623
rs711752
rs552082934
rs9924530
rs116727213
rs12927647
rs4888134
rs11648800
rs8140287
rs2526680
rs11082040
rs55999243
rs814415
rs77387803
rs150912954
rs117848461
rs6800209
rs11244061
rs6950910
rs79824097
All − MR Egger
All − Inverse variance weighted
−2
0
2
MR effect size for
'GCST90200140' on 'Calculus of lower urinary tract'

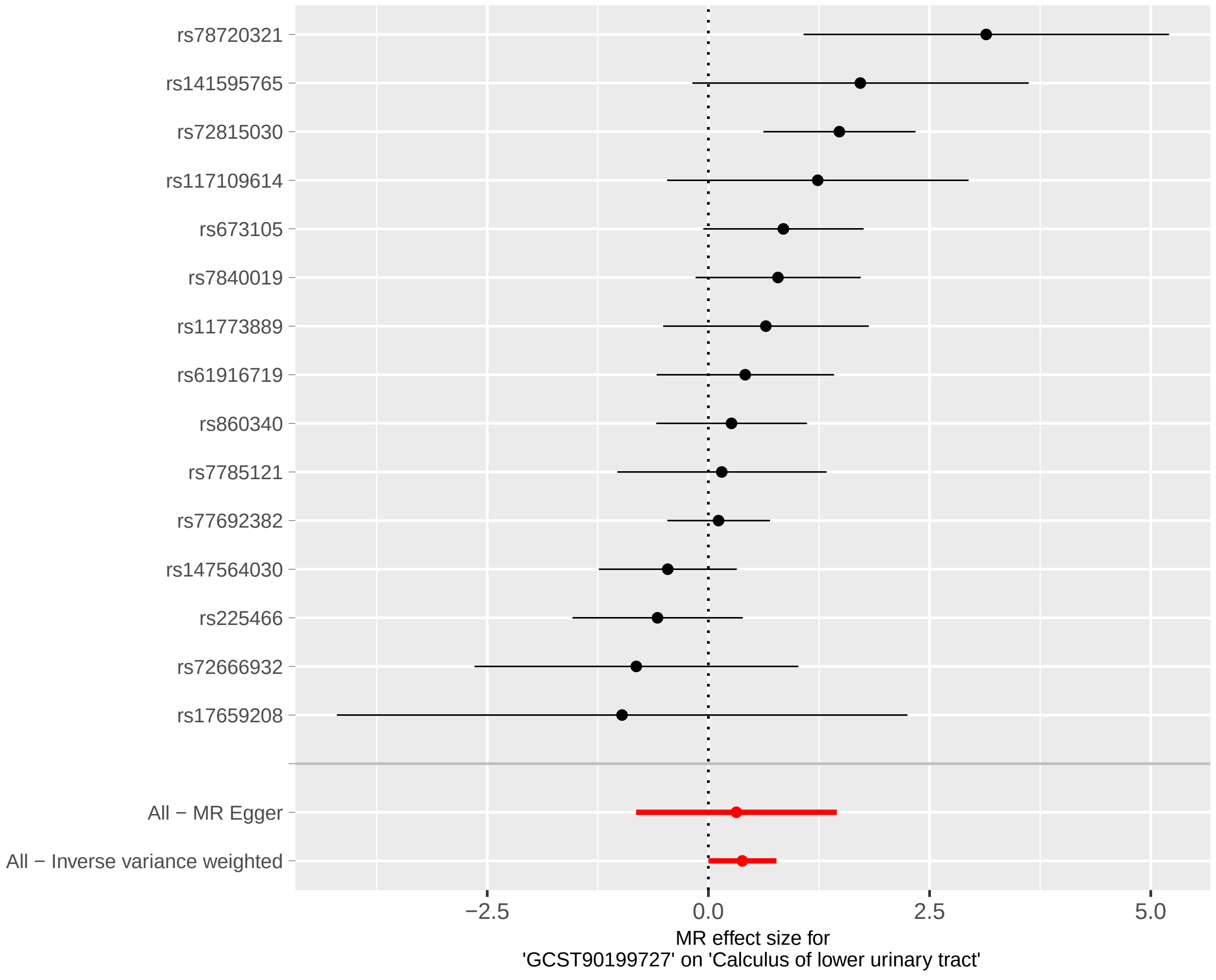

rs78720321
rs141595765
rs72815030
rs117109614
rs673105
rs7840019
rs11773889
rs61916719
rs860340
rs7785121
rs77692382
rs147564030
rs225466
rs72666932
rs17659208
All − MR Egger
All − Inverse variance weighted
−2.5
0.0
2.5
5.0
MR effect size for
'GCST90199727' on 'Calculus of lower urinary tract'

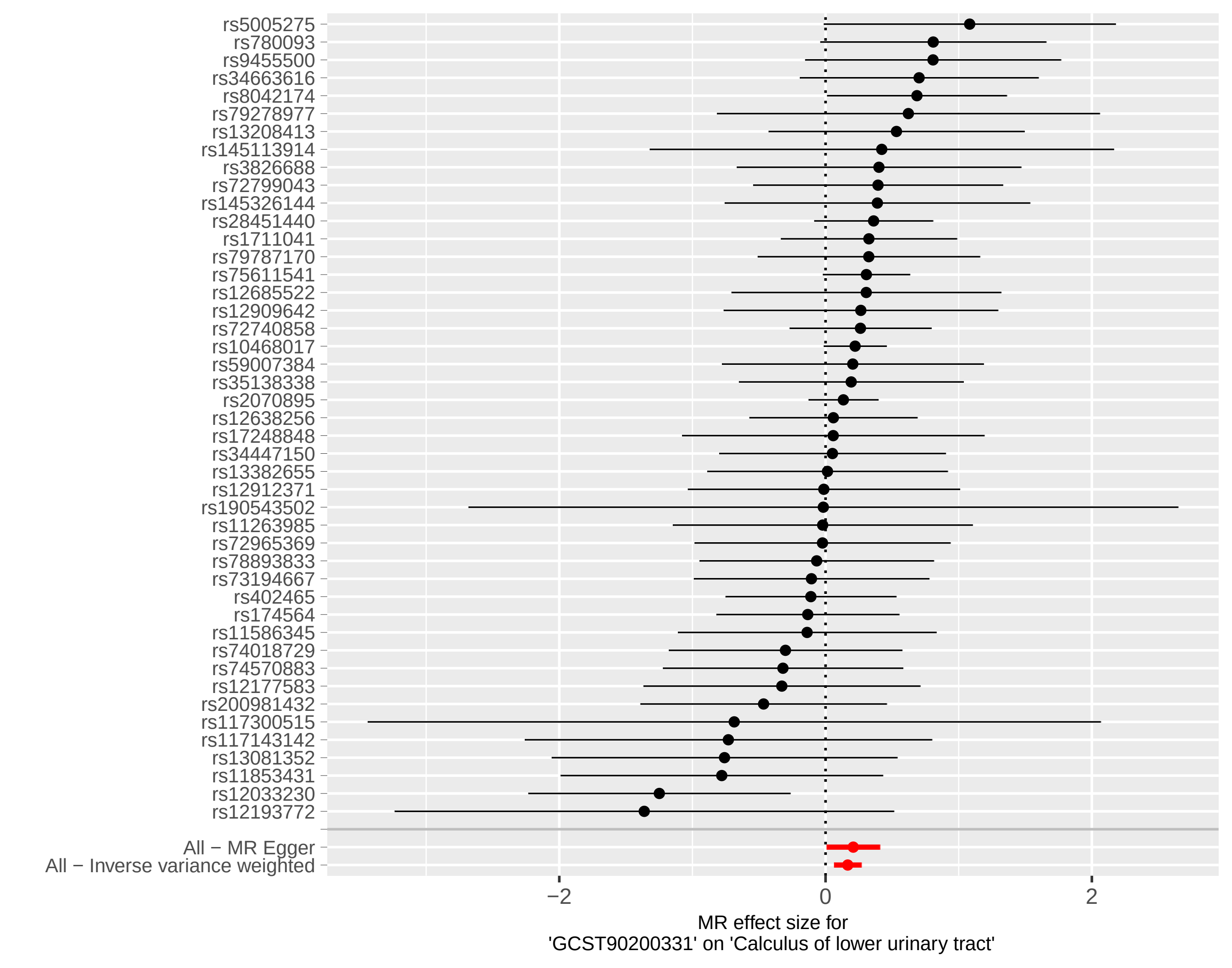

rs5005275
rs780093
rs9455500
rs34663616
rs8042174
rs79278977
rs13208413
rs145113914
rs3826688
rs72799043
rs145326144
rs28451440
rs1711041
rs79787170
rs75611541
rs12685522
rs12909642
rs72740858
rs10468017
rs59007384
rs35138338
rs2070895
rs12638256
rs17248848
rs34447150
rs13382655
rs12912371
rs190543502
rs11263985
rs72965369
rs78893833
rs73194667
rs402465
rs174564
rs11586345
rs74018729
rs74570883
rs12177583
rs200981432
rs117300515
rs117143142
rs13081352
rs11853431
rs12033230
rs12193772
All − MR Egger
All − Inverse variance weighted
−2
0
2
MR effect size for
'GCST90200331' on 'Calculus of lower urinary tract'

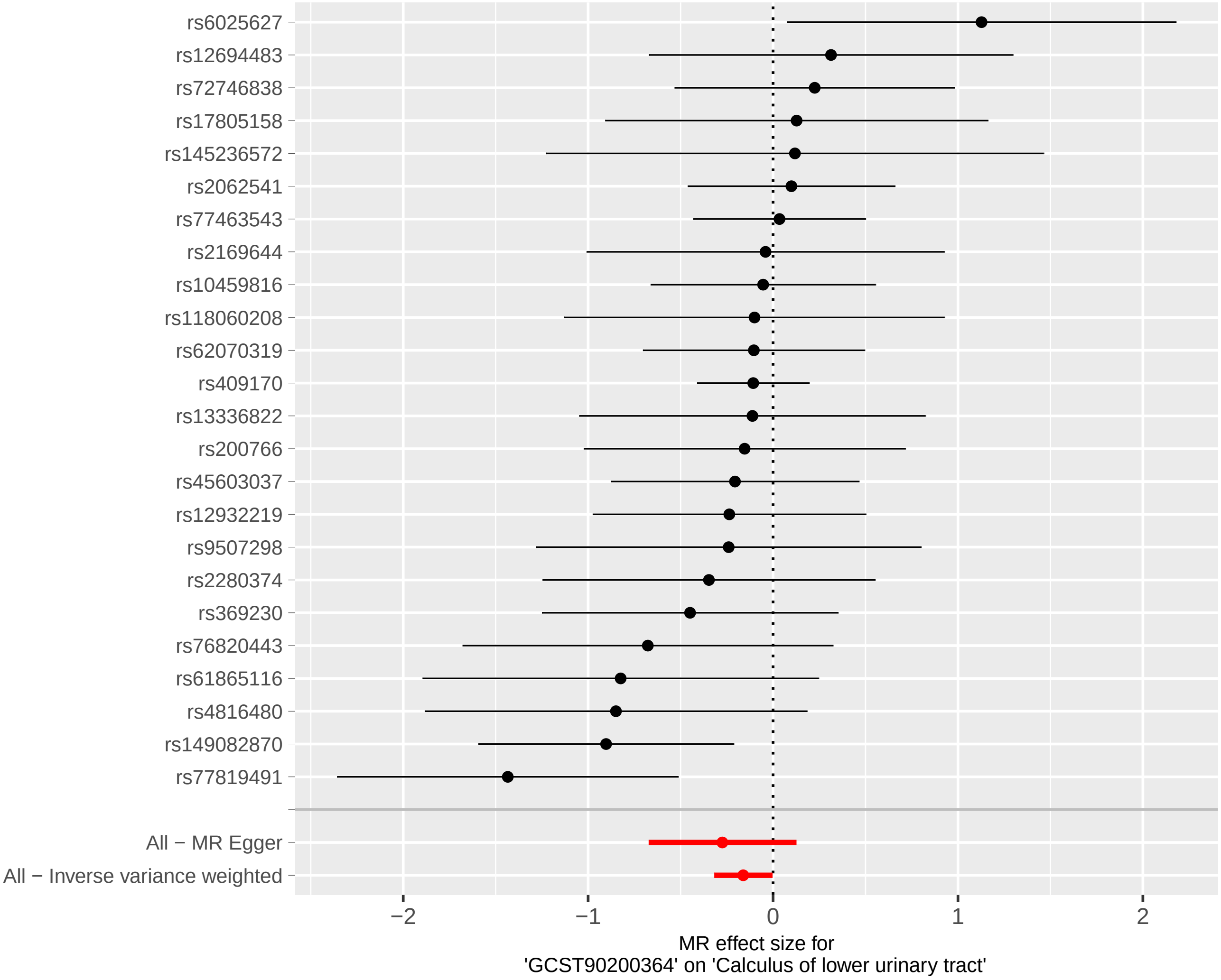
rs6025627
rs12694483
rs72746838
rs17805158
rs145236572
rs2062541
rs77463543
rs2169644
rs10459816
rs118060208
rs62070319
rs409170
rs13336822
rs200766
rs45603037
rs12932219
rs9507298
rs2280374
rs369230
rs76820443
rs61865116
rs4816480
rs149082870
rs77819491
All − MR Egger
All − Inverse variance weighted
−2
−1
0
1
2
MR effect size for
'GCST90200364' on 'Calculus of lower urinary tract'

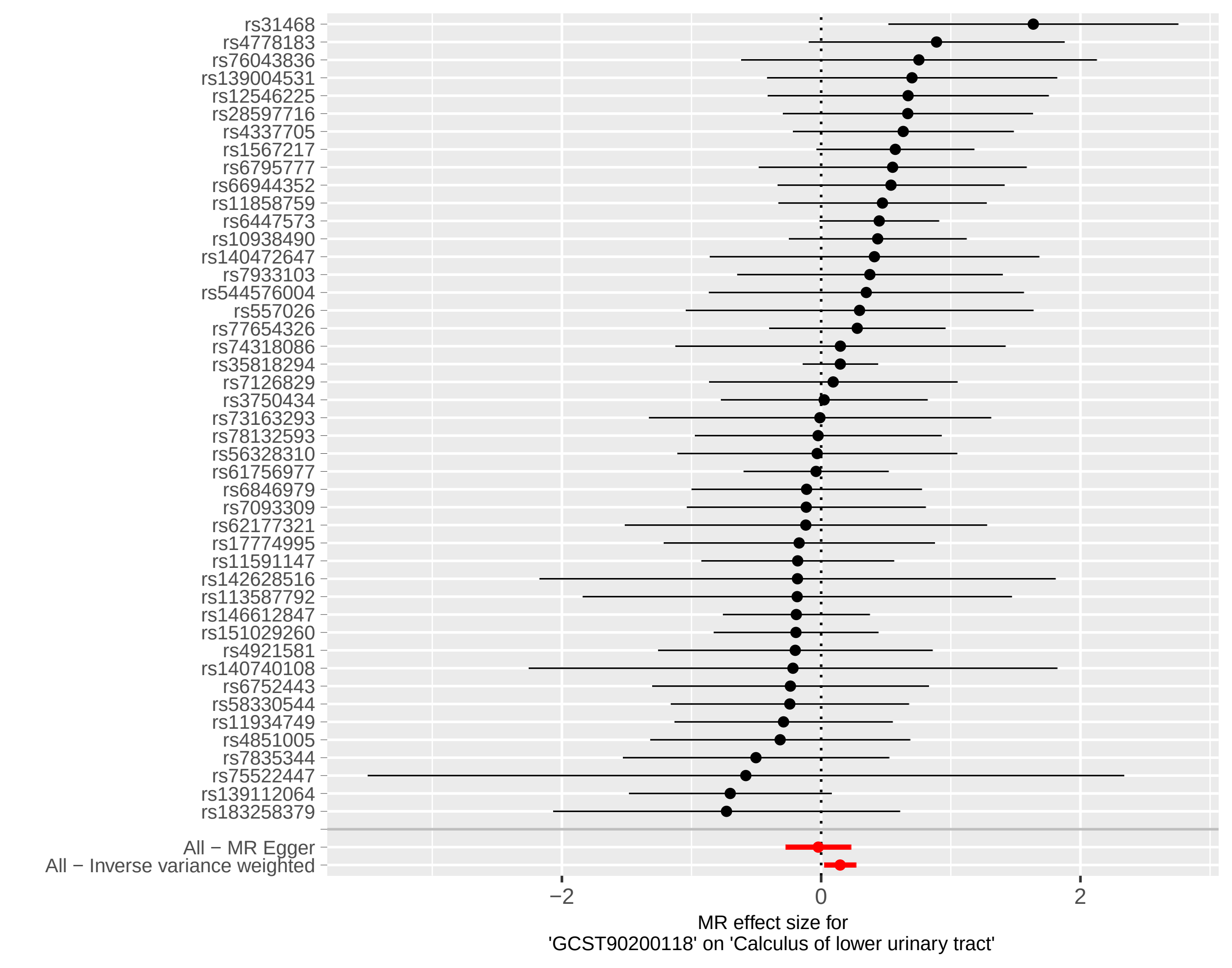
rs31468
rs4778183
rs76043836
rs139004531
rs12546225
rs28597716
rs4337705
rs1567217
rs6795777
rs66944352
rs11858759
rs6447573
rs10938490
rs140472647
rs7933103
rs544576004
rs557026
rs77654326
rs74318086
rs35818294
rs7126829
rs3750434
rs73163293
rs78132593
rs56328310
rs61756977
rs6846979
rs7093309
rs62177321
rs17774995
rs11591147
rs142628516
rs113587792
rs146612847
rs151029260
rs4921581
rs140740108
rs6752443
rs58330544
rs11934749
rs4851005
rs7835344
rs75522447
rs139112064
rs183258379
All − MR Egger
All − Inverse variance weighted
−2
0
2
MR effect size for
'GCST90200118' on 'Calculus of lower urinary tract'

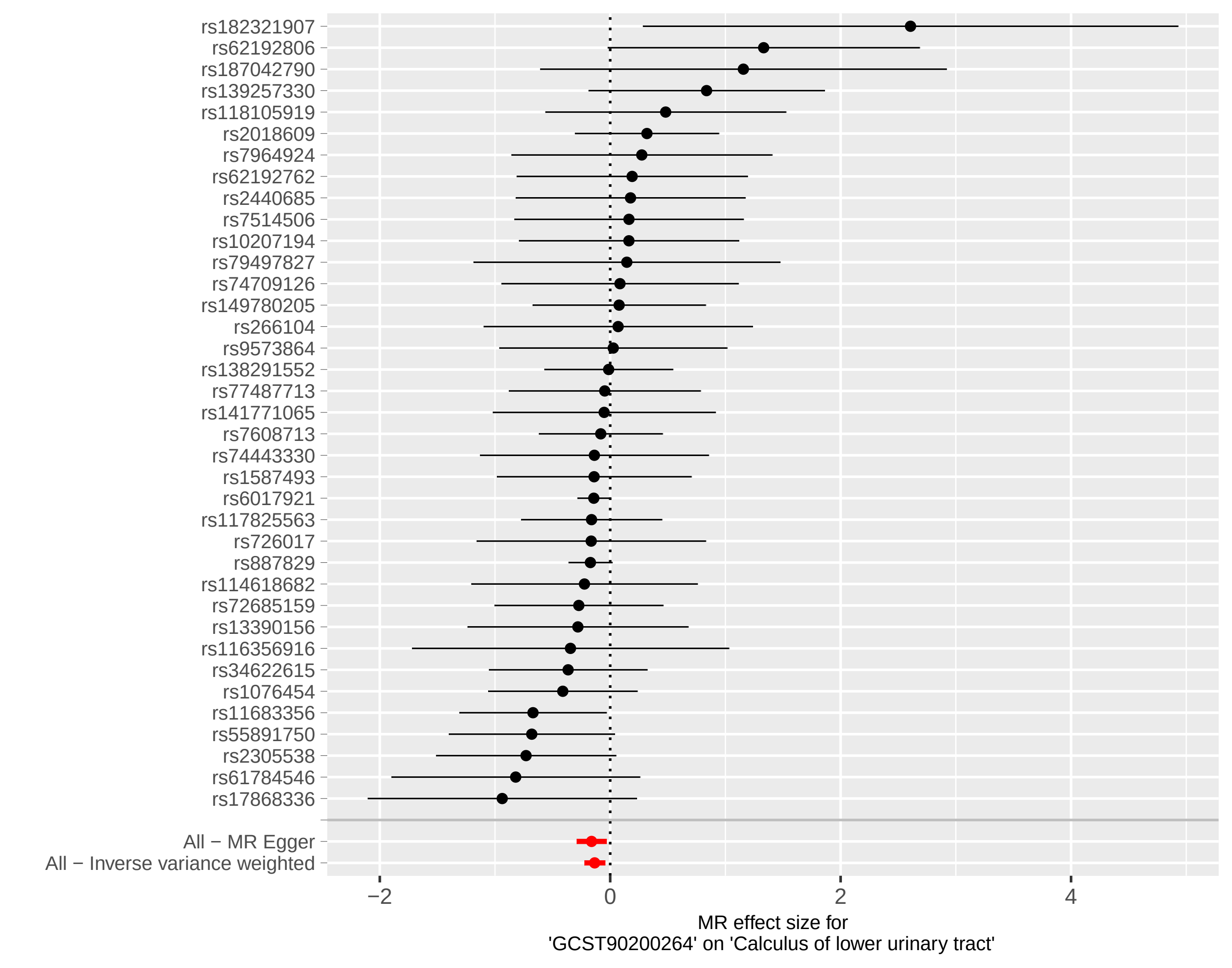

rs182321907
rs62192806
rs187042790
rs139257330
rs118105919
rs2018609
rs7964924
rs62192762
rs2440685
rs7514506
rs10207194
rs79497827
rs74709126
rs149780205
rs266104
rs9573864
rs138291552
rs77487713
rs141771065
rs7608713
rs74443330
rs1587493
rs6017921
rs117825563
rs726017
rs887829
rs114618682
rs72685159
rs13390156
rs116356916
rs34622615
rs1076454
rs11683356
rs55891750
rs2305538
rs61784546
rs17868336
All − MR Egger
All − Inverse variance weighted
−2
0
2
4
MR effect size for
'GCST90200264' on 'Calculus of lower urinary tract'

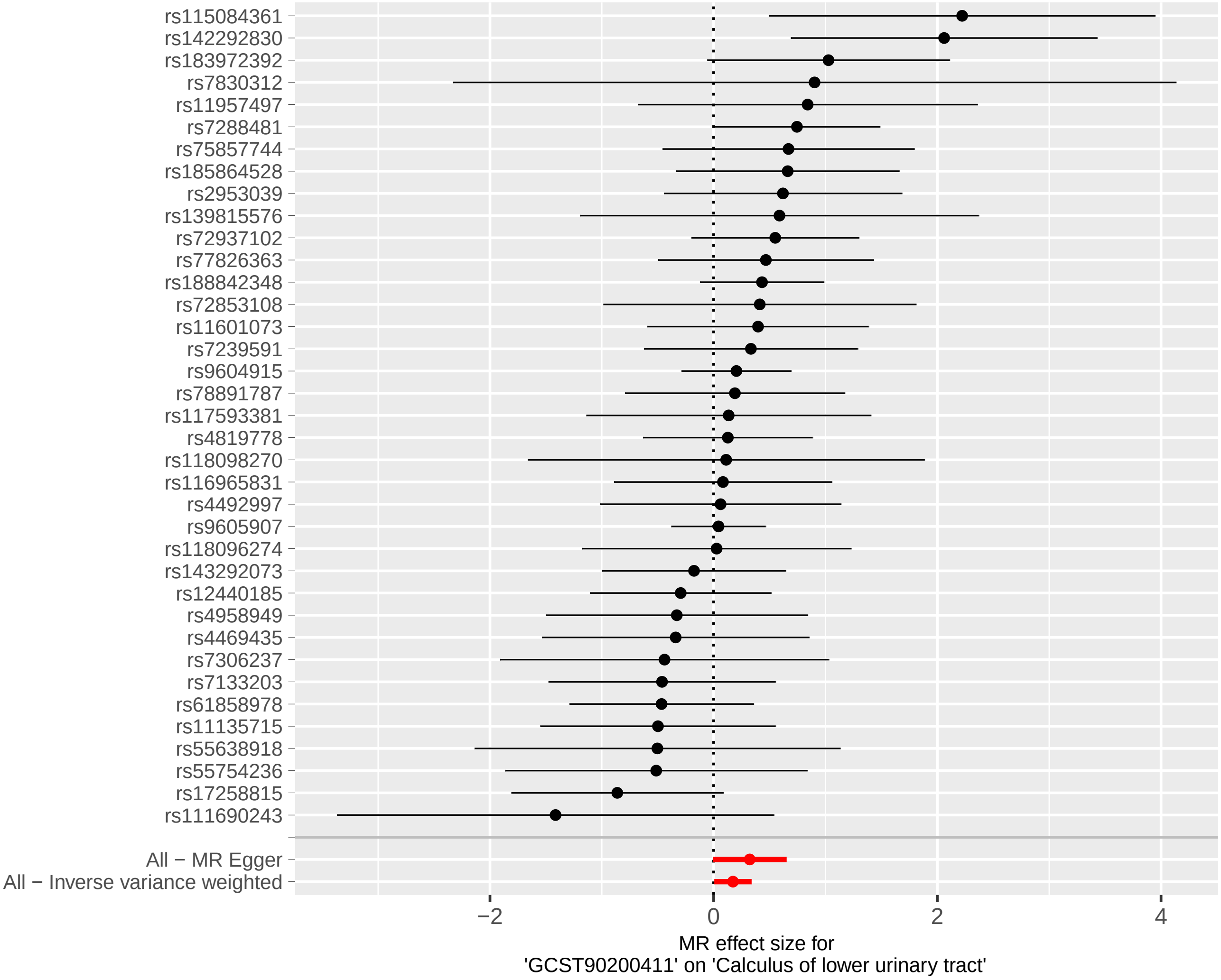
rs115084361
rs142292830
rs183972392
rs7830312
rs11957497
rs7288481
rs75857744
rs185864528
rs2953039
rs139815576
rs72937102
rs77826363
rs188842348
rs72853108
rs11601073
rs7239591
rs9604915
rs78891787
rs117593381
rs4819778
rs118098270
rs116965831
rs4492997
rs9605907
rs118096274
rs143292073
rs12440185
rs4958949
rs4469435
rs7306237
rs7133203
rs61858978
rs11135715
rs55638918
rs55754236
rs17258815
rs111690243
All − MR Egger
All − Inverse variance weighted
−2
0
2
4
MR effect size for
'GCST90200411' on 'Calculus of lower urinary tract'

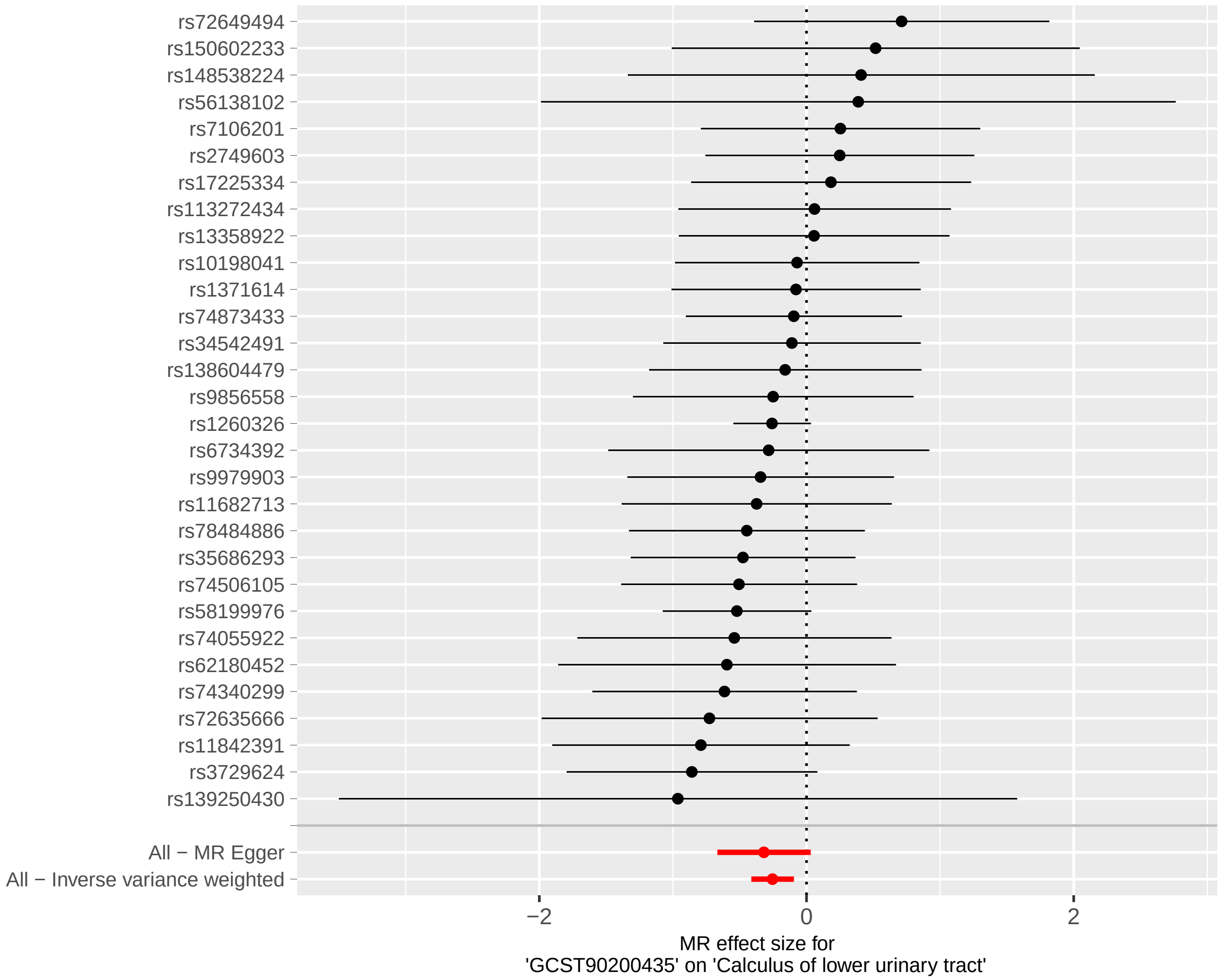

rs72649494
rs150602233
rs148538224
rs56138102
rs7106201
rs2749603
rs17225334
rs113272434
rs13358922
rs10198041
rs1371614
rs74873433
rs34542491
rs138604479
rs9856558
rs1260326
rs6734392
rs9979903
rs11682713
rs78484886
rs35686293
rs74506105
rs58199976
rs74055922
rs62180452
rs74340299
rs72635666
rs11842391
rs3729624
rs139250430
All − MR Egger
All − Inverse variance weighted
−2
0
2
MR effect size for
'GCST90200435' on 'Calculus of lower urinary tract'

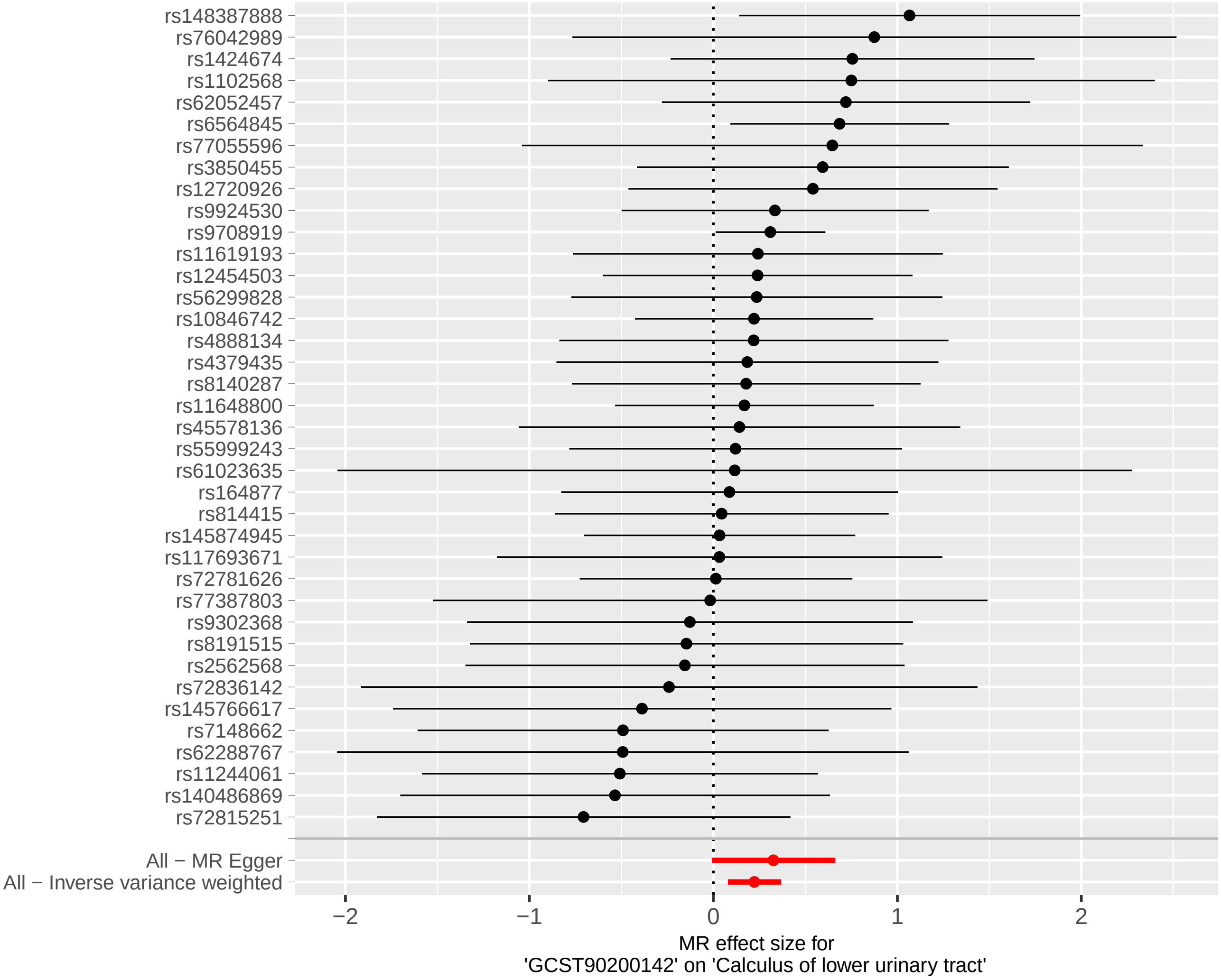

rs148387888
rs76042989
rs1424674
rs1102568
rs62052457
rs6564845
rs77055596
rs3850455
rs12720926
rs9924530
rs9708919
rs11619193
rs12454503
rs56299828
rs10846742
rs4888134
rs4379435
rs8140287
rs11648800
rs45578136
rs55999243
rs61023635
rs164877
rs814415
rs145874945
rs117693671
rs72781626
rs77387803
rs9302368
rs8191515
rs2562568
rs72836142
rs145766617
rs7148662
rs62288767
rs11244061
rs140486869
rs72815251
All − MR Egger
All − Inverse variance weighted
−2
−1
0
1
2
MR effect size for
'GCST90200142' on 'Calculus of lower urinary tract'

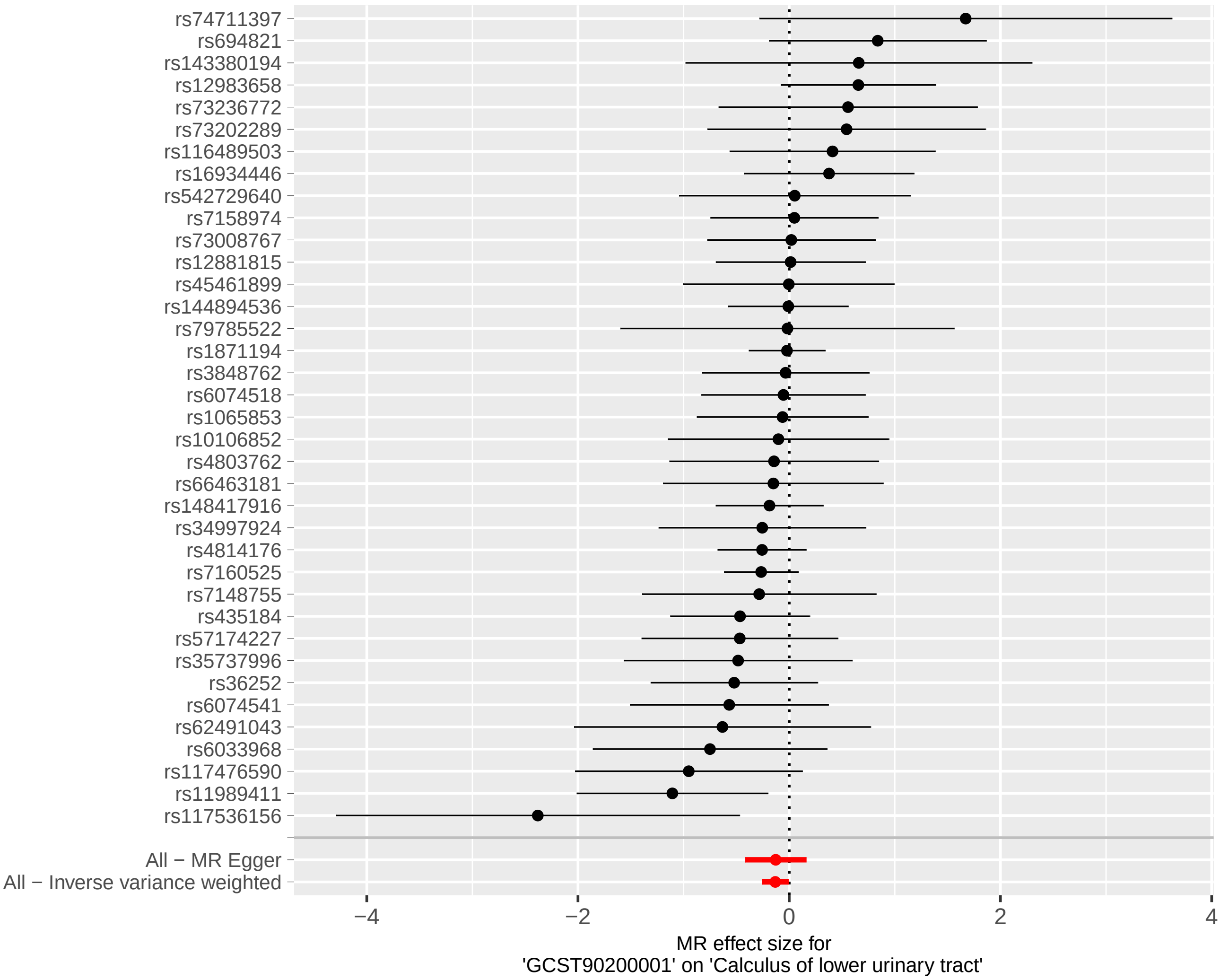
rs74711397
rs694821
rs143380194
rs12983658
rs73236772
rs73202289
rs116489503
rs16934446
rs542729640
rs7158974
rs73008767
rs12881815
rs45461899
rs144894536
rs79785522
rs1871194
rs3848762
rs6074518
rs1065853
rs10106852
rs4803762
rs66463181
rs148417916
rs34997924
rs4814176
rs7160525
rs7148755
rs435184
rs57174227
rs35737996
rs36252
rs6074541
rs62491043
rs6033968
rs117476590
rs11989411
rs117536156
All − MR Egger
All − Inverse variance weighted
−4
−2
0
2
4
MR effect size for
'GCST90200001' on 'Calculus of lower urinary tract'

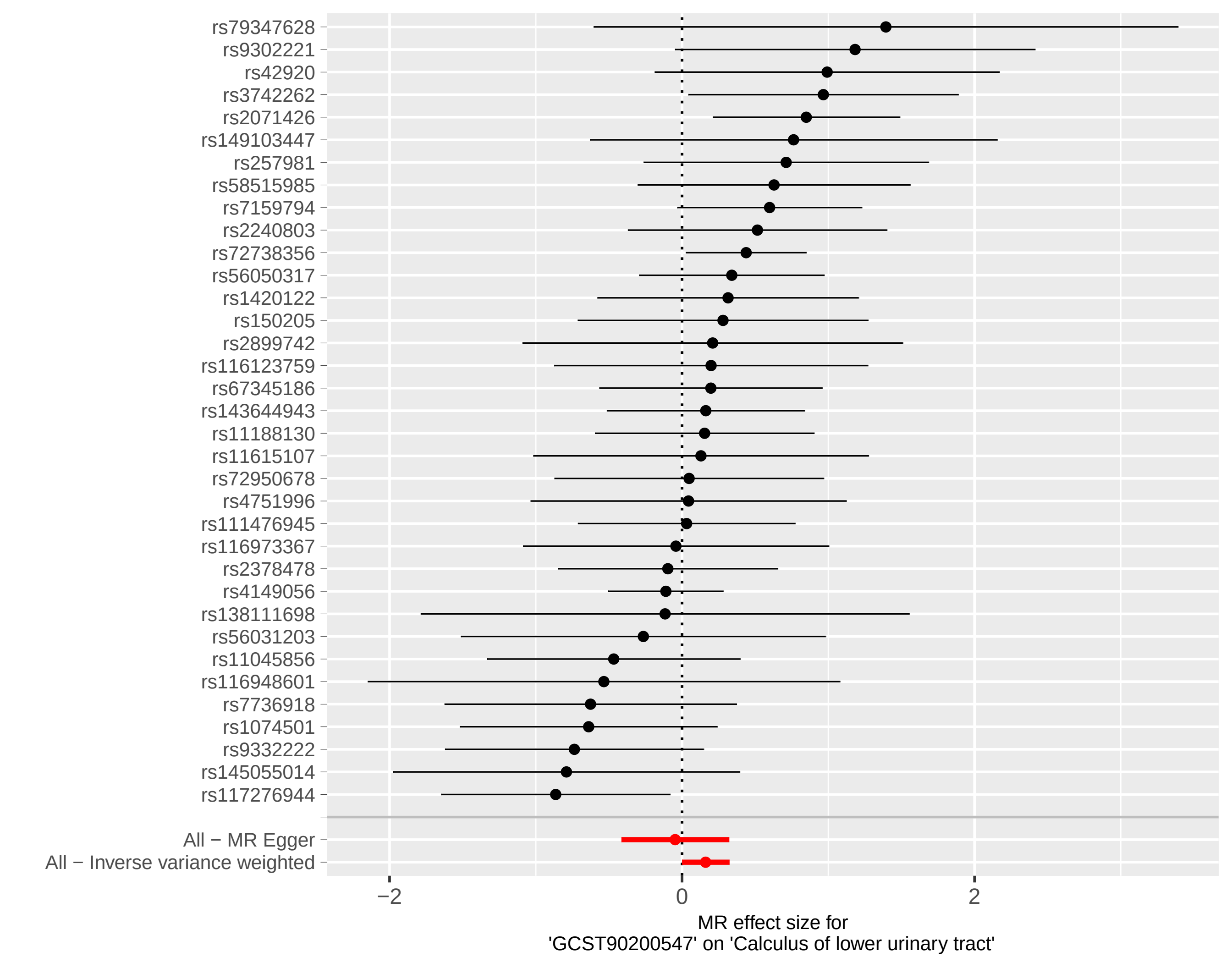
rs79347628
rs9302221
rs42920
rs3742262
rs2071426
rs149103447
rs257981
rs58515985
rs7159794
rs2240803
rs72738356
rs56050317
rs1420122
rs150205
rs2899742
rs116123759
rs67345186
rs143644943
rs11188130
rs11615107
rs72950678
rs4751996
rs111476945
rs116973367
rs2378478
rs4149056
rs138111698
rs56031203
rs11045856
rs116948601
rs7736918
rs1074501
rs9332222
rs145055014
rs117276944
All − MR Egger
All − Inverse variance weighted
−2
0
2
MR effect size for
'GCST90200547' on 'Calculus of lower urinary tract'

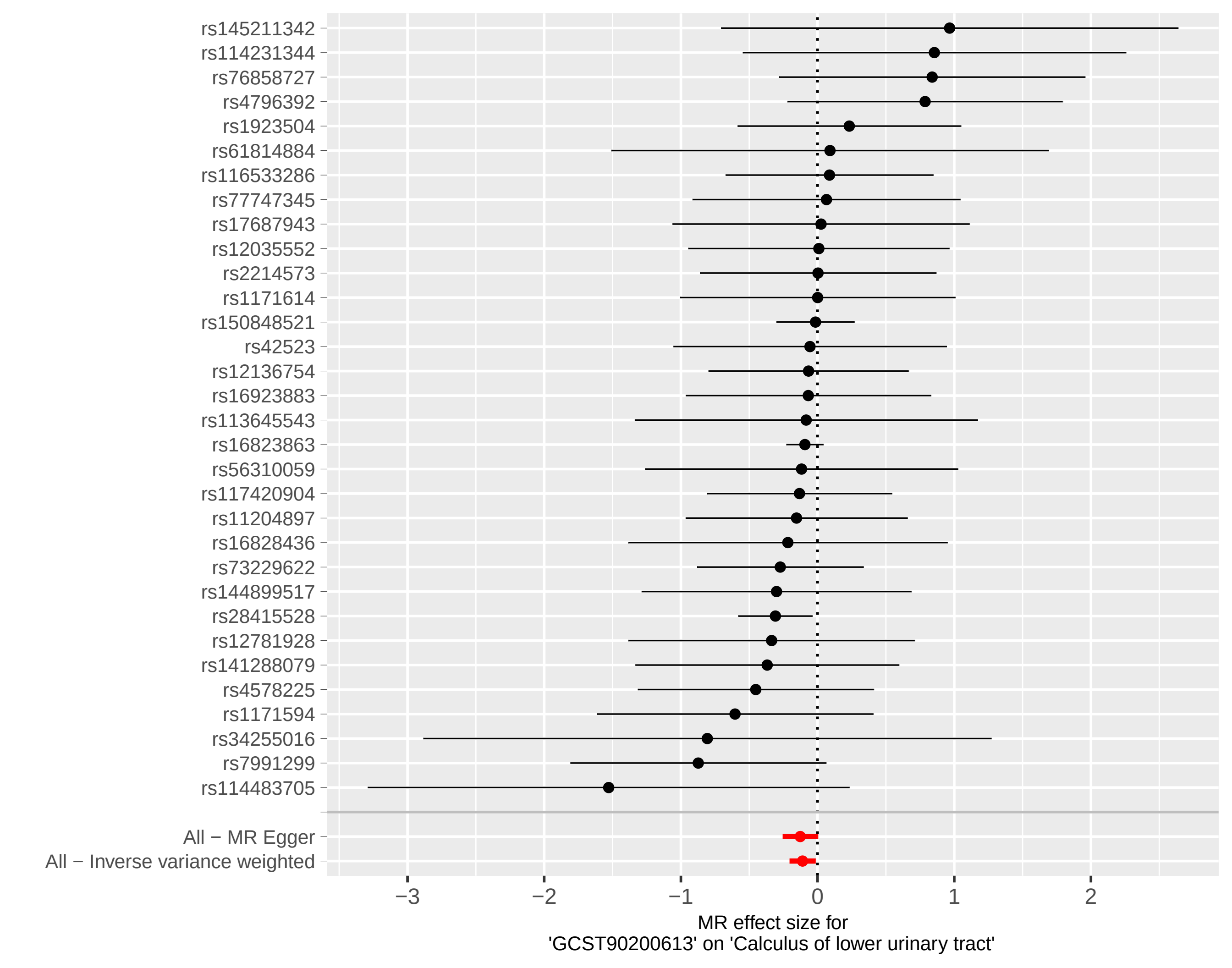
rs145211342
rs114231344
rs76858727
rs4796392
rs1923504
rs61814884
rs116533286
rs77747345
rs17687943
rs12035552
rs2214573
rs1171614
rs150848521
rs42523
rs12136754
rs16923883
rs113645543
rs16823863
rs56310059
rs117420904
rs11204897
rs16828436
rs73229622
rs144899517
rs28415528
rs12781928
rs141288079
rs4578225
rs1171594
rs34255016
rs7991299
rs114483705
All − MR Egger
All − Inverse variance weighted
−3
−2
−1
0
1
2
MR effect size for
'GCST90200613' on 'Calculus of lower urinary tract'

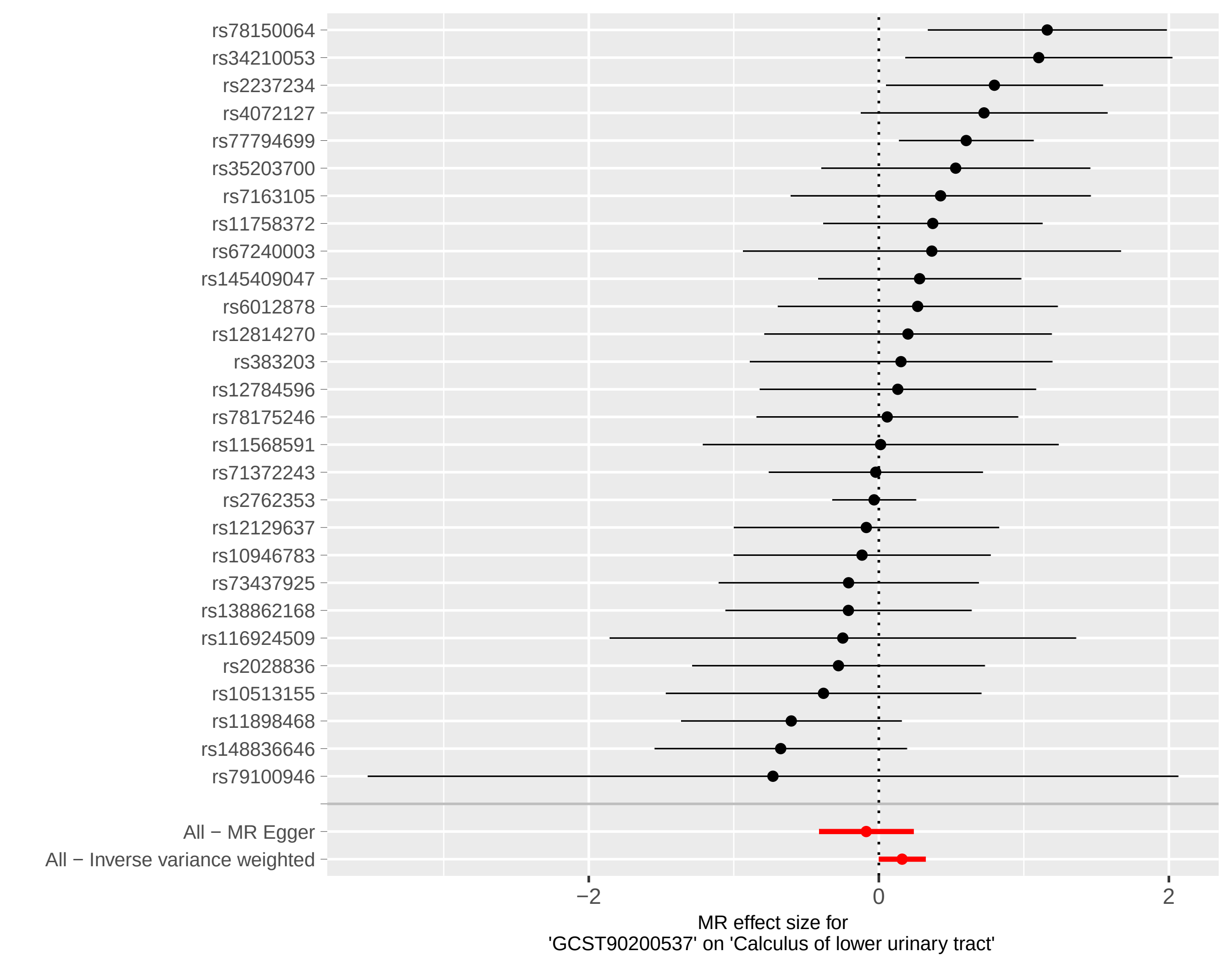
rs78150064
rs34210053
rs2237234
rs4072127
rs77794699
rs35203700
rs7163105
rs11758372
rs67240003
rs145409047
rs6012878
rs12814270
rs383203
rs12784596
rs78175246
rs11568591
rs71372243
rs2762353
rs12129637
rs10946783
rs73437925
rs138862168
rs116924509
rs2028836
rs10513155
rs11898468
rs148836646
rs79100946
All − MR Egger
All − Inverse variance weighted
−2
0
2
MR effect size for
'GCST90200537' on 'Calculus of lower urinary tract'

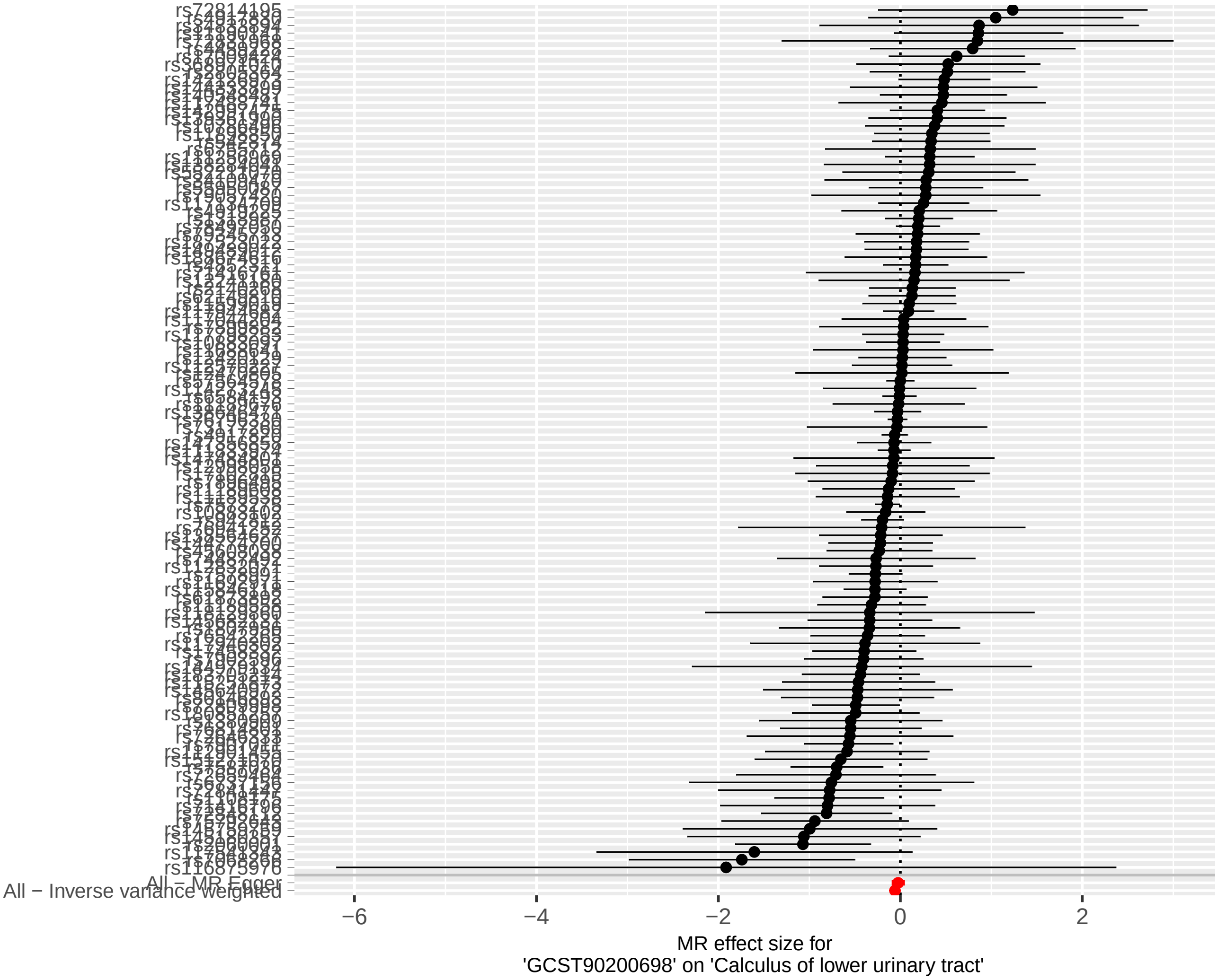

rs72814195
rs116875970
All − MR Egger
All − Inverse variance weighted
−6
−4
−2
0
2
MR effect size for
'GCST90200698' on 'Calculus of lower urinary tract'

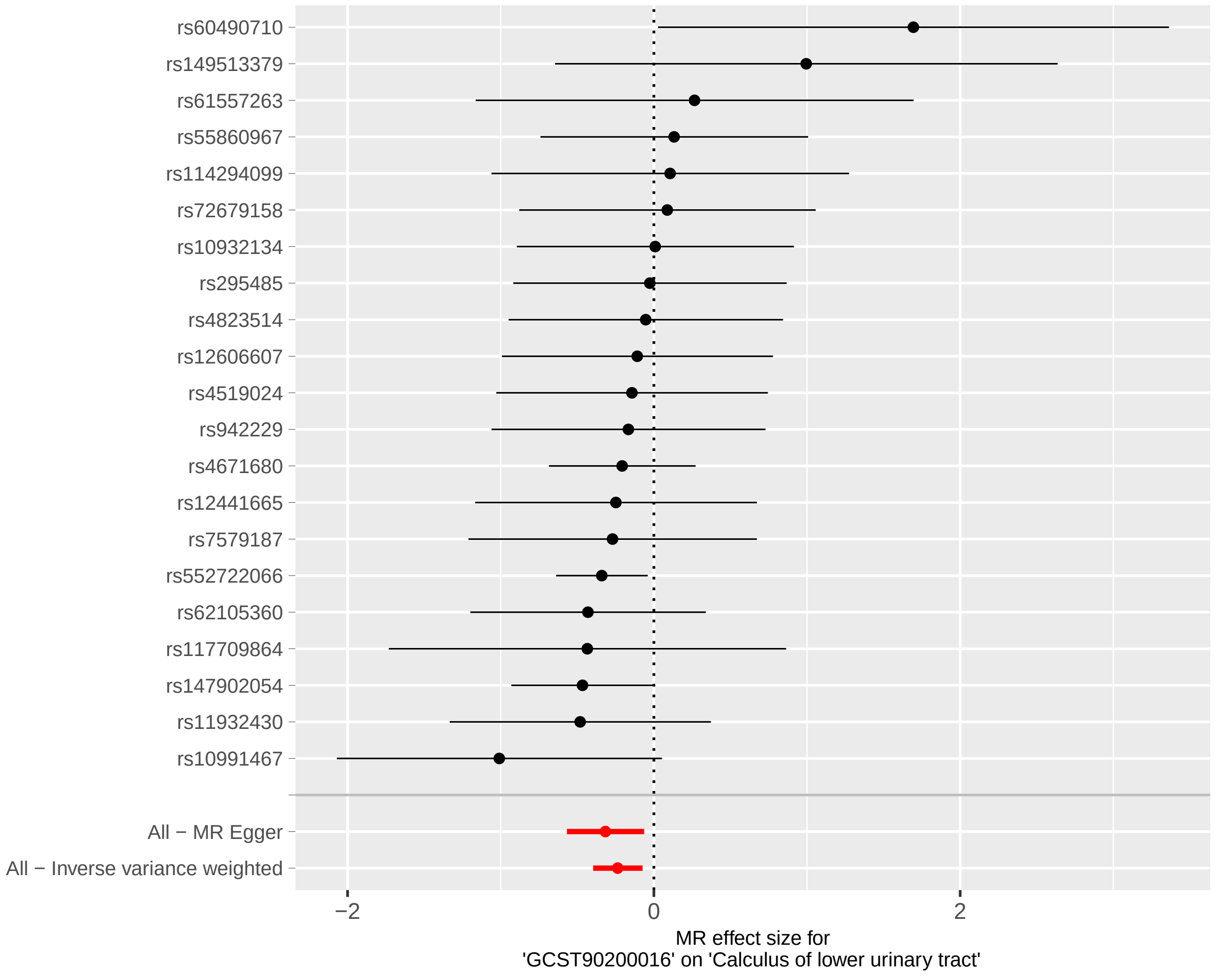
rs60490710
rs149513379
rs61557263
rs55860967
rs114294099
rs72679158
rs10932134
rs295485
rs4823514
rs12606607
rs4519024
rs942229
rs4671680
rs12441665
rs7579187
rs552722066
rs62105360
rs117709864
rs147902054
rs11932430
rs10991467
All − MR Egger
All − Inverse variance weighted
−2
0
2
MR effect size for
'GCST90200016' on 'Calculus of lower urinary tract'

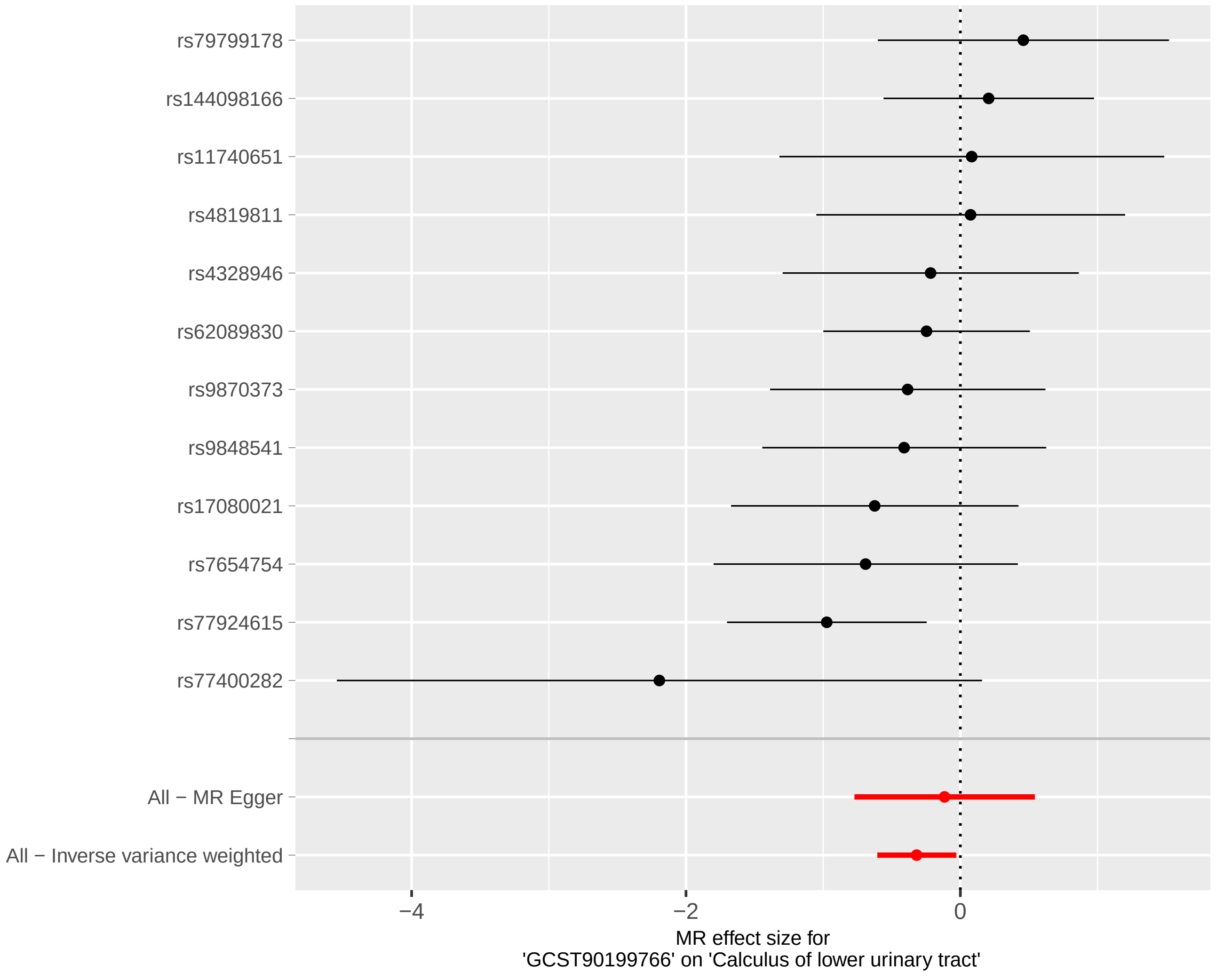
rs79799178
rs144098166
rs11740651
rs4819811
rs4328946
rs62089830
rs9870373
rs9848541
rs17080021
rs7654754
rs77924615
rs77400282
All − MR Egger
All − Inverse variance weighted
−4
−2
0
MR effect size for
'GCST90199766' on 'Calculus of lower urinary tract'

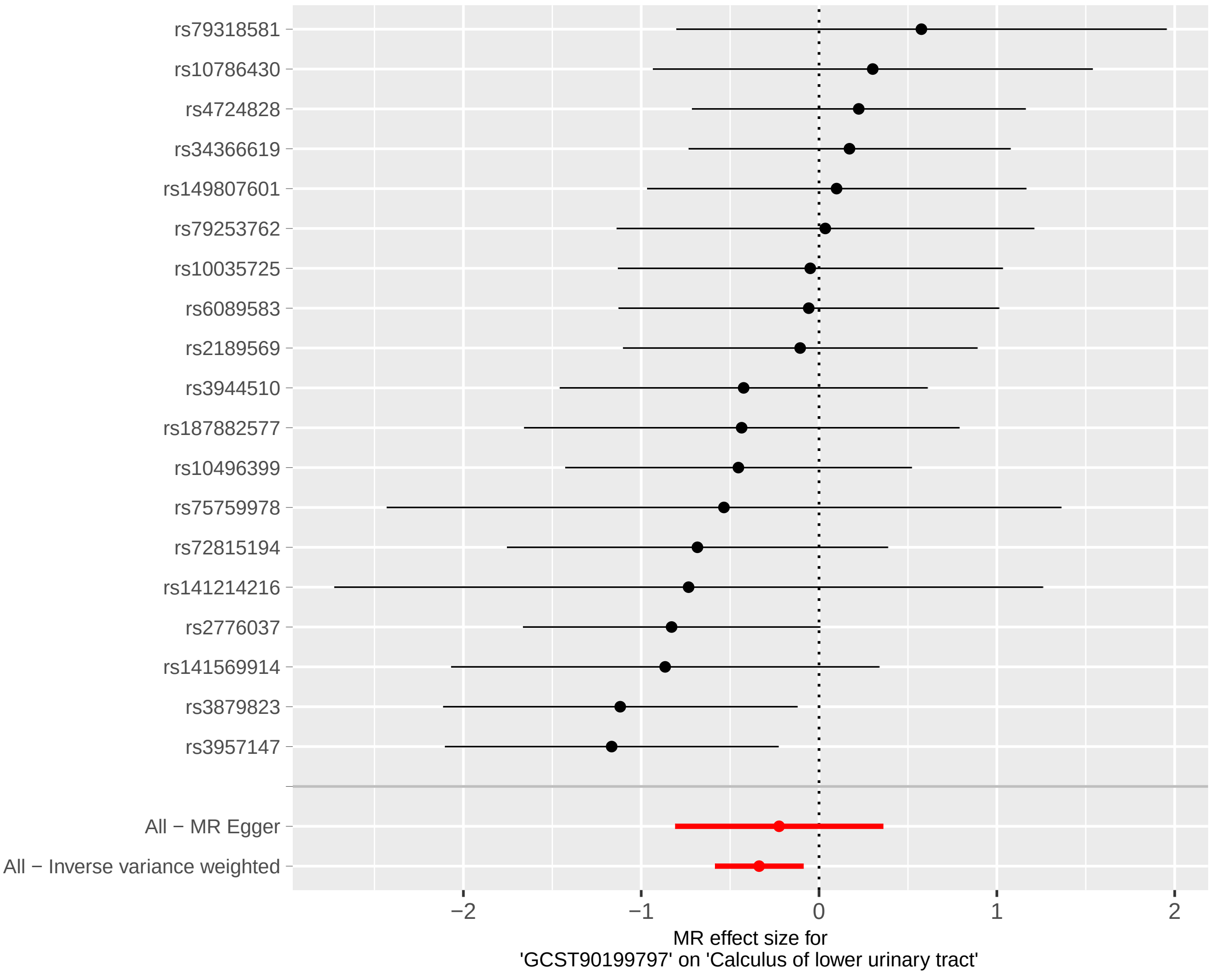
rs79318581
rs10786430
rs4724828
rs34366619
rs149807601
rs79253762
rs10035725
rs6089583
rs2189569
rs3944510
rs187882577
rs10496399
rs75759978
rs72815194
rs141214216
rs2776037
rs141569914
rs3879823
rs3957147
All − MR Egger
All − Inverse variance weighted
−2
−1
0
1
2
MR effect size for
'GCST90199797' on 'Calculus of lower urinary tract'

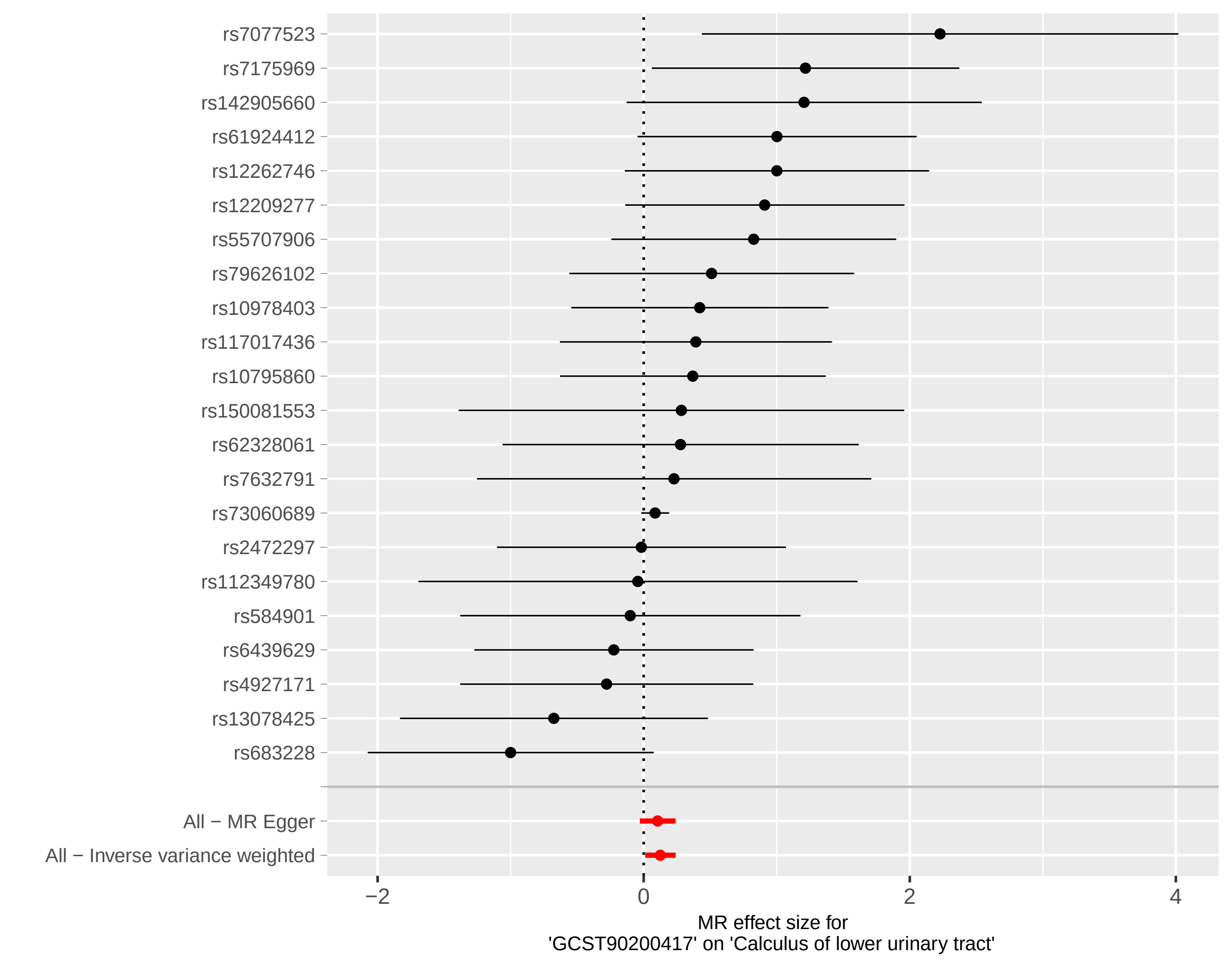
rs7077523
rs7175969
rs142905660
rs61924412
rs12262746
rs12209277
rs55707906
rs79626102
rs10978403
rs117017436
rs10795860
rs150081553
rs62328061
rs7632791
rs73060689
rs2472297
rs112349780
rs584901
rs6439629
rs4927171
rs13078425
rs683228
All − MR Egger
All − Inverse variance weighted
−2
0
2
4
MR effect size for
'GCST90200417' on 'Calculus of lower urinary tract'

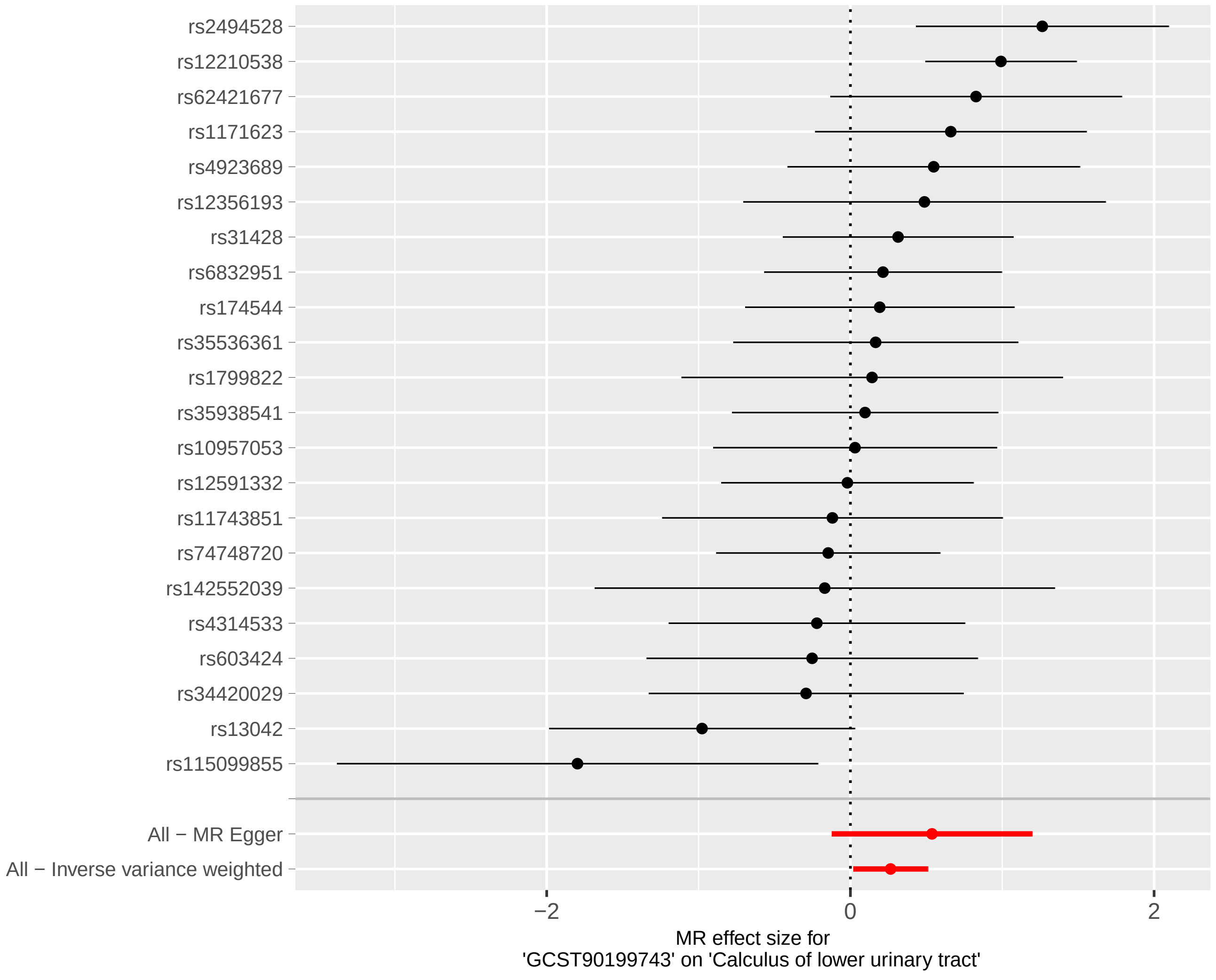
rs2494528
rs12210538
rs62421677
rs1171623
rs4923689
rs12356193
rs31428
rs6832951
rs174544
rs35536361
rs1799822
rs35938541
rs10957053
rs12591332
rs11743851
rs74748720
rs142552039
rs4314533
rs603424
rs34420029
rs13042
rs115099855
All − MR Egger
All − Inverse variance weighted
−2
0
2
MR effect size for
'GCST90199743' on 'Calculus of lower urinary tract'

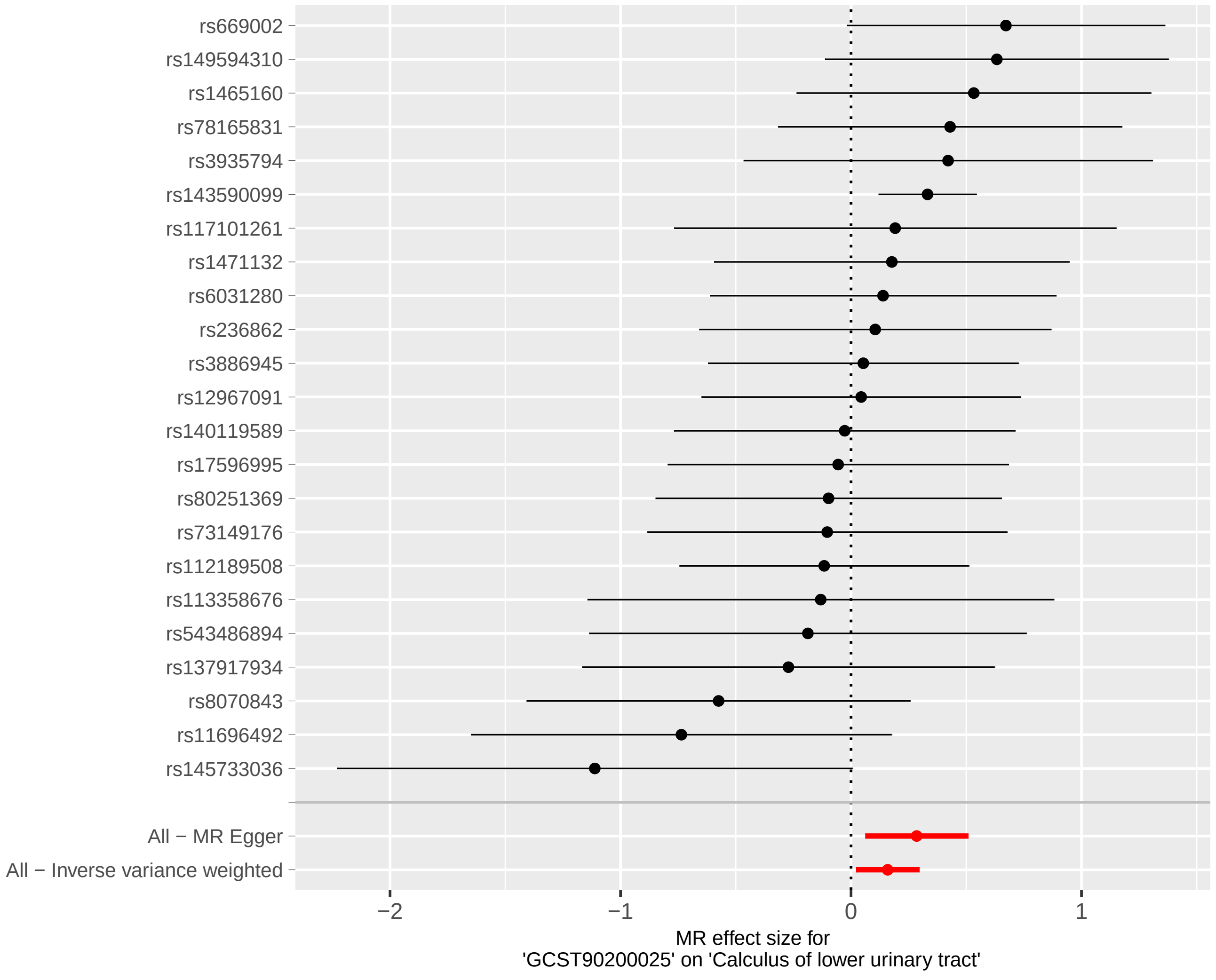

rs669002
rs149594310
rs1465160
rs78165831
rs3935794
rs143590099
rs117101261
rs1471132
rs6031280
rs236862
rs3886945
rs12967091
rs140119589
rs17596995
rs80251369
rs73149176
rs112189508
rs113358676
rs543486894
rs137917934
rs8070843
rs11696492
rs145733036
All − MR Egger
All − Inverse variance weighted
−2
−1
0
1
MR effect size for
'GCST90200025' on 'Calculus of lower urinary tract'

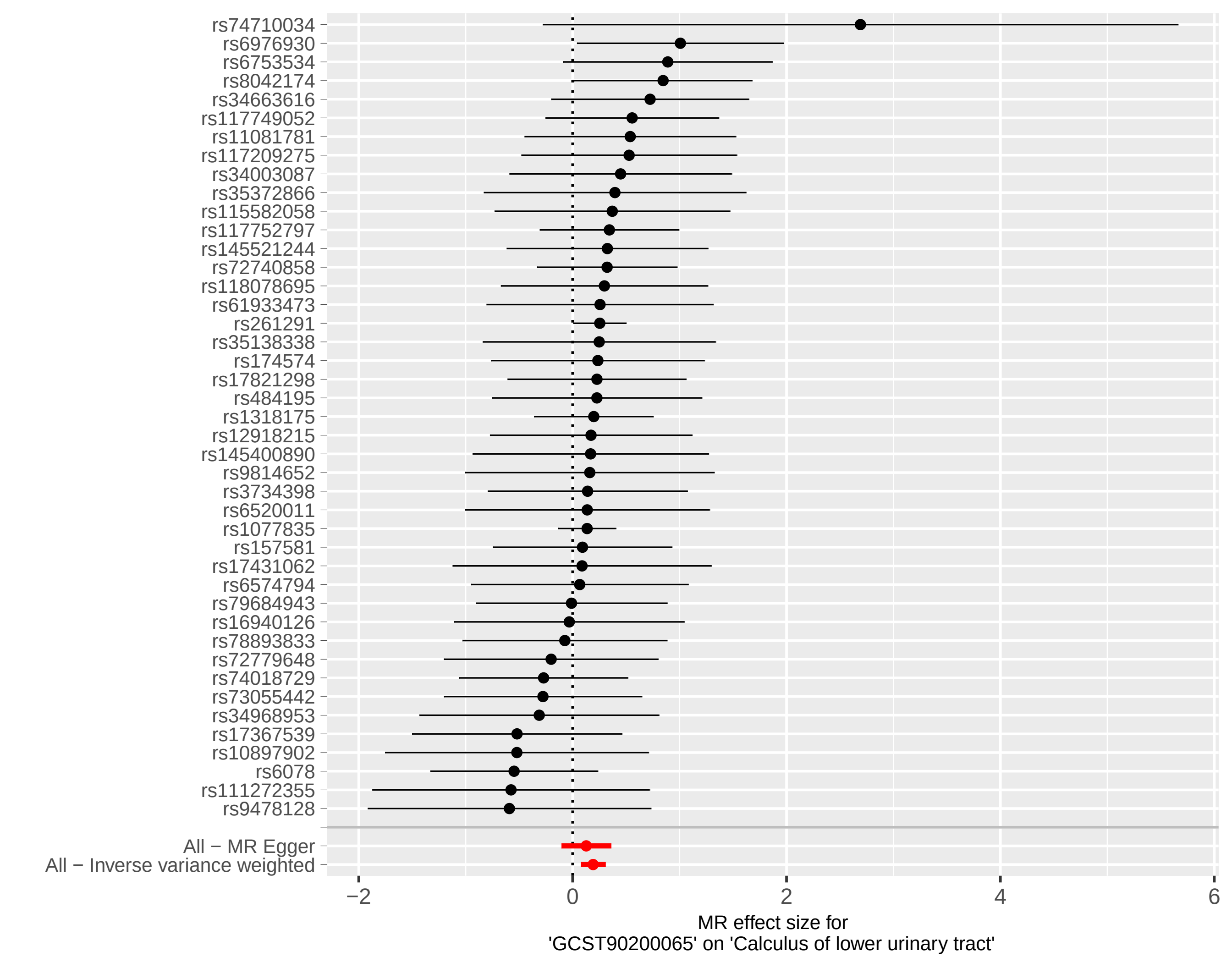
rs74710034
rs6976930
rs6753534
rs8042174
rs34663616
rs117749052
rs11081781
rs117209275
rs34003087
rs35372866
rs115582058
rs117752797
rs145521244
rs72740858
rs118078695
rs61933473
rs261291
rs35138338
rs174574
rs17821298
rs484195
rs1318175
rs12918215
rs145400890
rs9814652
rs3734398
rs6520011
rs1077835
rs157581
rs17431062
rs6574794
rs79684943
rs16940126
rs78893833
rs72779648
rs74018729
rs73055442
rs34968953
rs17367539
rs10897902
rs6078
rs111272355
rs9478128
All − MR Egger
All − Inverse variance weighted
−2
0
2
4
6
MR effect size for
'GCST90200065' on 'Calculus of lower urinary tract'

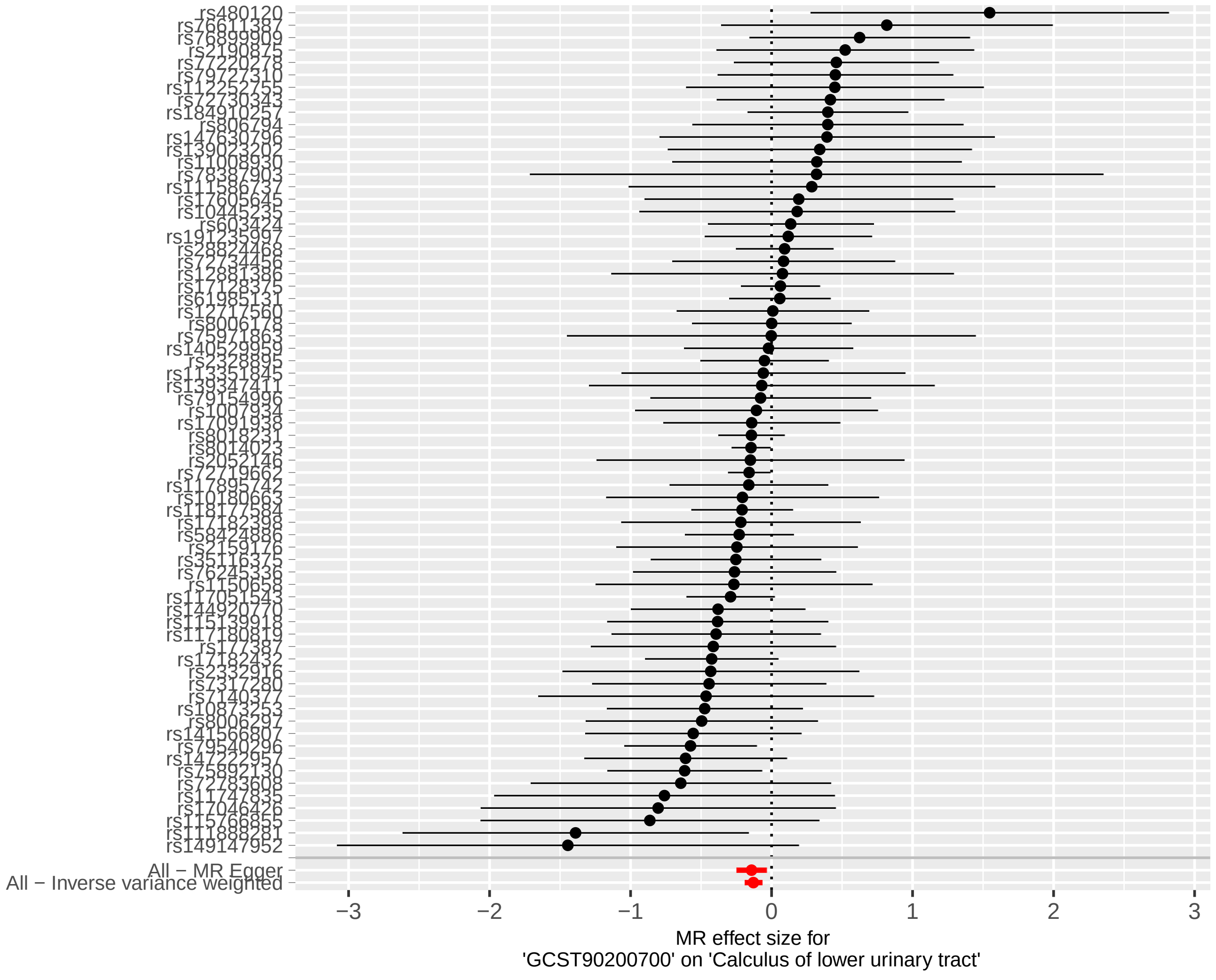
rs480120
rs76611387
rs76899909
rs2190875
rs77220278
rs79727310
rs112252755
rs72730343
rs184910257
rs806794
rs147630796
rs139023202
rs11008930
rs78387903
rs111586737
rs17605645
rs10445235
rs603424
rs191235997
rs28824468
rs72734456
rs12881386
rs17128375
rs61985131
rs12717560
rs8006178
rs75971863
rs140529959
rs2328895
rs113351845
rs139347411
rs79154996
rs1007934
rs17091938
rs8018231
rs8014023
rs2052146
rs72719662
rs117895742
rs10180663
rs118177584
rs17182398
rs58424886
rs2159176
rs35116375
rs76245336
rs1150658
rs117051543
rs144920770
rs115139918
rs117180819
rs177387
rs17182432
rs2332916
rs7317280
rs7140377
rs10873253
rs8006297
rs141566807
rs79540296
rs147222957
rs75892130
rs72783608
rs11747835
rs17046426
rs115766855
rs111888281
rs149147952
All − MR Egger
All − Inverse variance weighted
−3
−2
−1
0
1
2
3
MR effect size for
'GCST90200700' on 'Calculus of lower urinary tract'

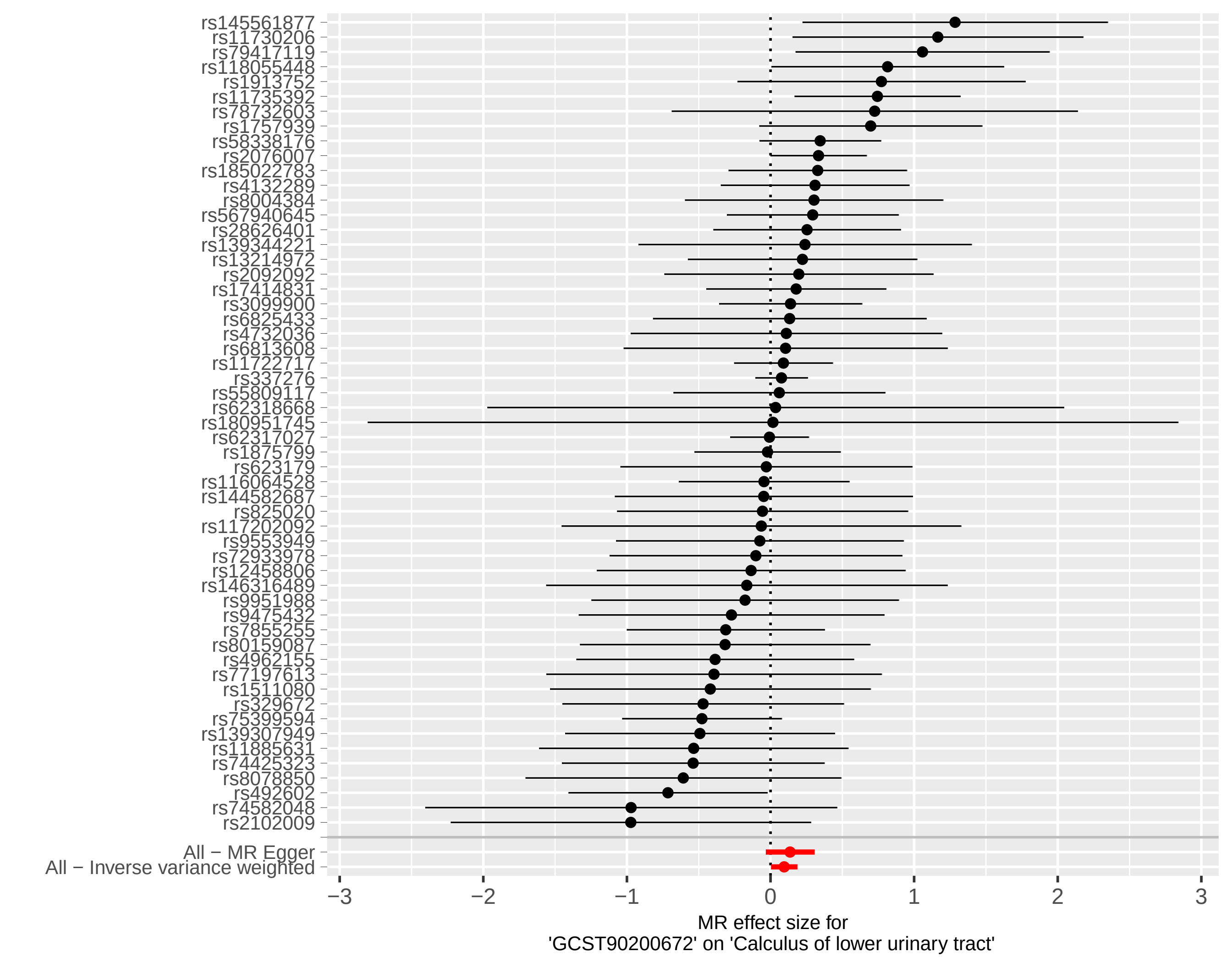

rs145561877
rs11730206
rs79417119
rs118055448
rs1913752
rs11735392
rs78732603
rs1757939
rs58338176
rs2076007
rs185022783
rs4132289
rs8004384
rs567940645
rs28626401
rs139344221
rs13214972
rs2092092
rs17414831
rs3099900
rs6825433
rs4732036
rs6813608
rs11722717
rs337276
rs55809117
rs62318668
rs180951745
rs62317027
rs1875799
rs623179
rs116064528
rs144582687
rs825020
rs117202092
rs9553949
rs72933978
rs12458806
rs146316489
rs9951988
rs9475432
rs7855255
rs80159087
rs4962155
rs77197613
rs1511080
rs329672
rs75399594
rs139307949
rs11885631
rs74425323
rs8078850
rs492602
rs74582048
rs2102009
All − MR Egger
All − Inverse variance weighted
−3
−2
−1
0
1
2
3
MR effect size for
'GCST90200672' on 'Calculus of lower urinary tract'

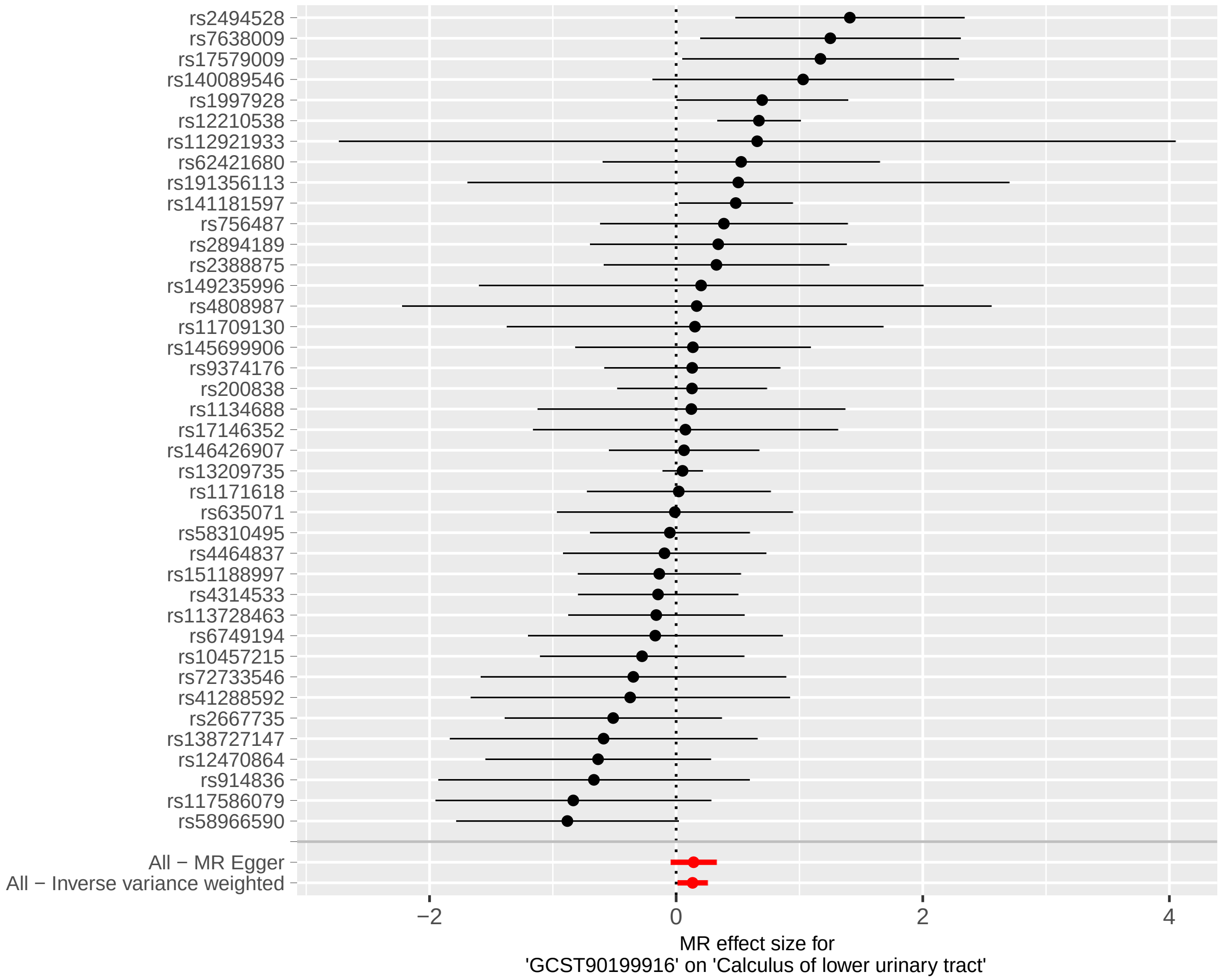
rs2494528
rs7638009
rs17579009
rs140089546
rs1997928
rs12210538
rs112921933
rs62421680
rs191356113
rs141181597
rs756487
rs2894189
rs2388875
rs149235996
rs4808987
rs11709130
rs145699906
rs9374176
rs200838
rs1134688
rs17146352
rs146426907
rs13209735
rs1171618
rs635071
rs58310495
rs4464837
rs151188997
rs4314533
rs113728463
rs6749194
rs10457215
rs72733546
rs41288592
rs2667735
rs138727147
rs12470864
rs914836
rs117586079
rs58966590
All − MR Egger
All − Inverse variance weighted
−2
0
2
4
MR effect size for
'GCST90199916' on 'Calculus of lower urinary tract'

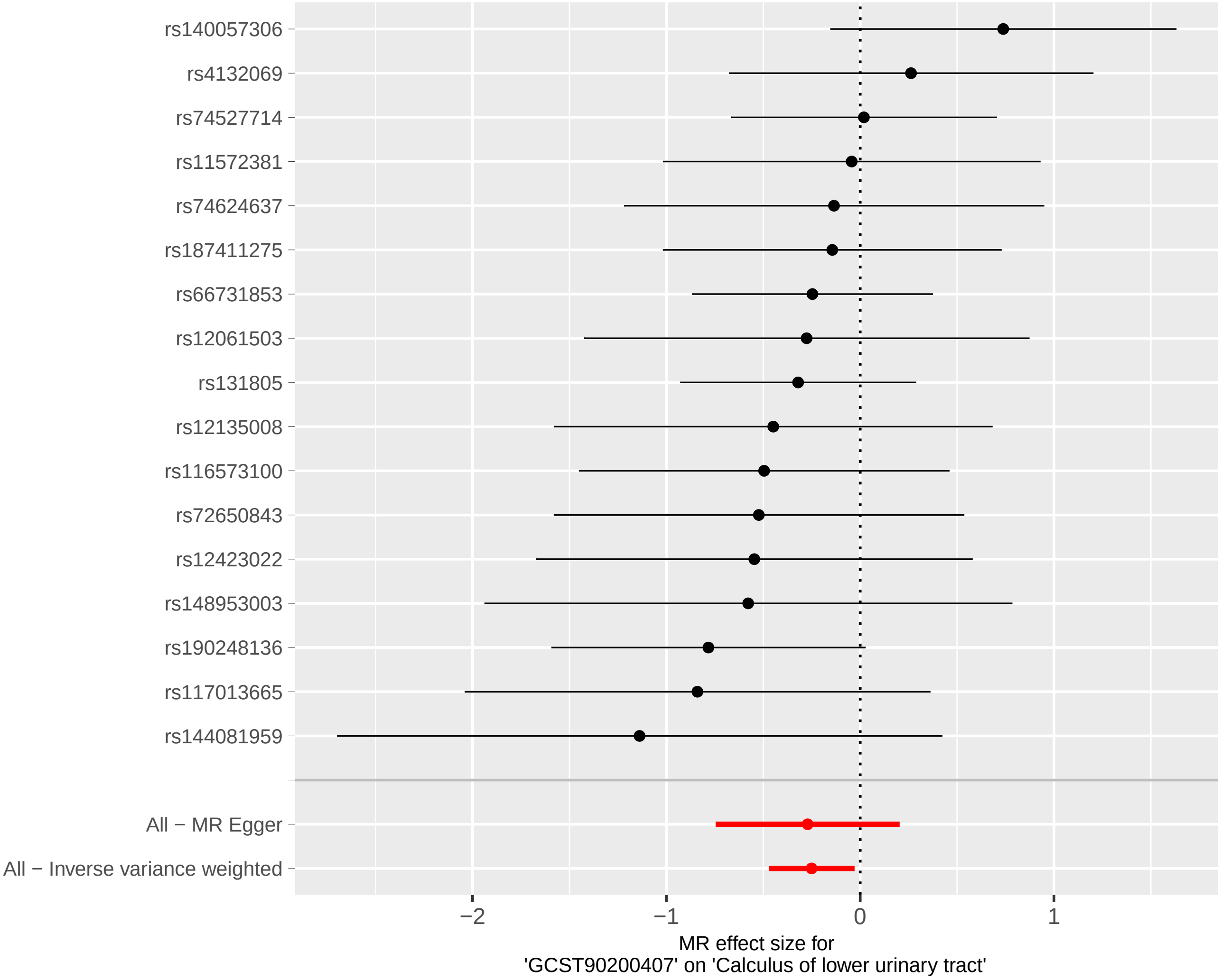
rs140057306
rs4132069
rs74527714
rs11572381
rs74624637
rs187411275
rs66731853
rs12061503
rs131805
rs12135008
rs116573100
rs72650843
rs12423022
rs148953003
rs190248136
rs117013665
rs144081959
All − MR Egger
All − Inverse variance weighted
−2
−1
0
1
MR effect size for
'GCST90200407' on 'Calculus of lower urinary tract'

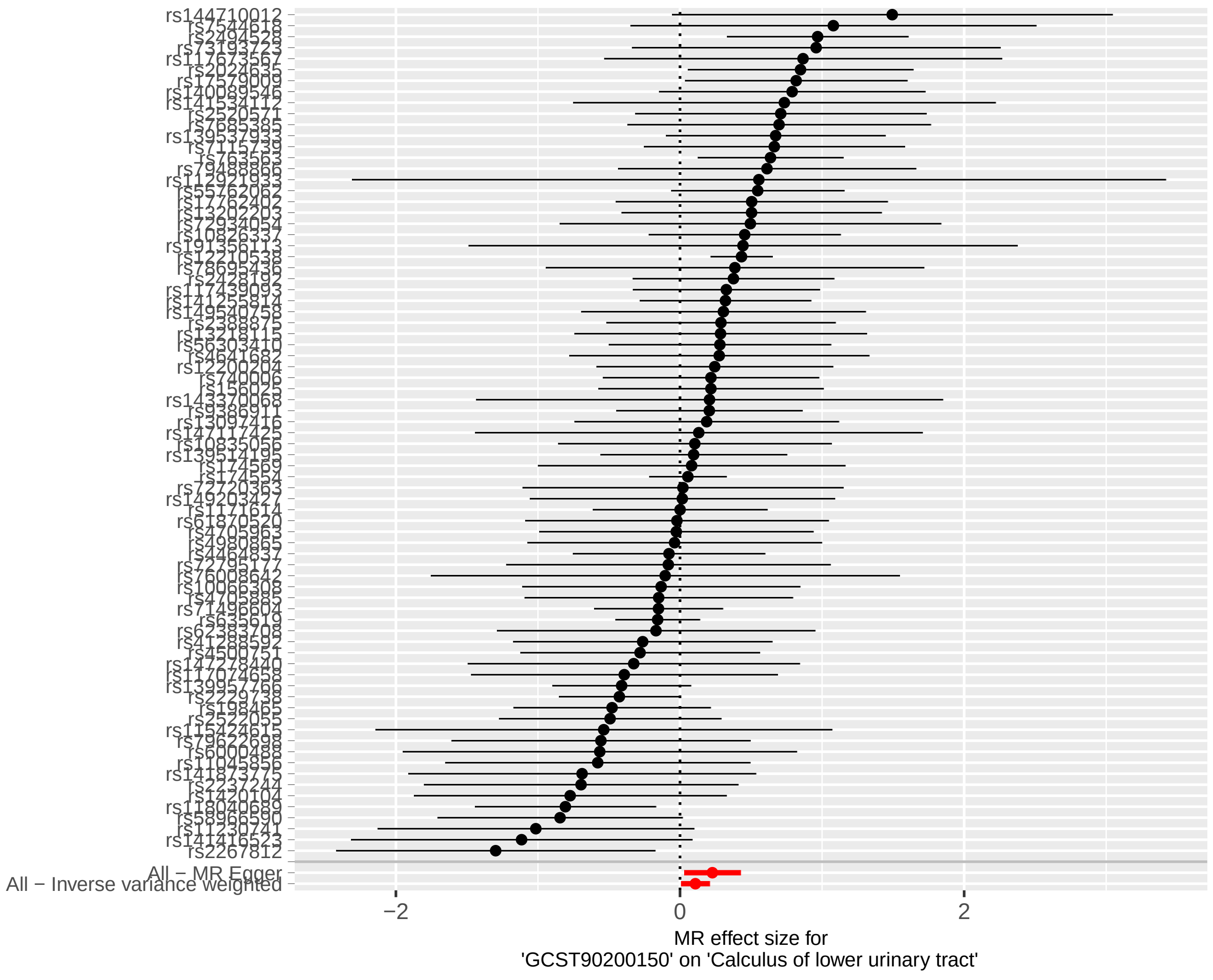

All − MR Egger
All − Inverse variance weighted
−2
0
2
MR effect size for
'GCST90200150' on 'Calculus of lower urinary tract'

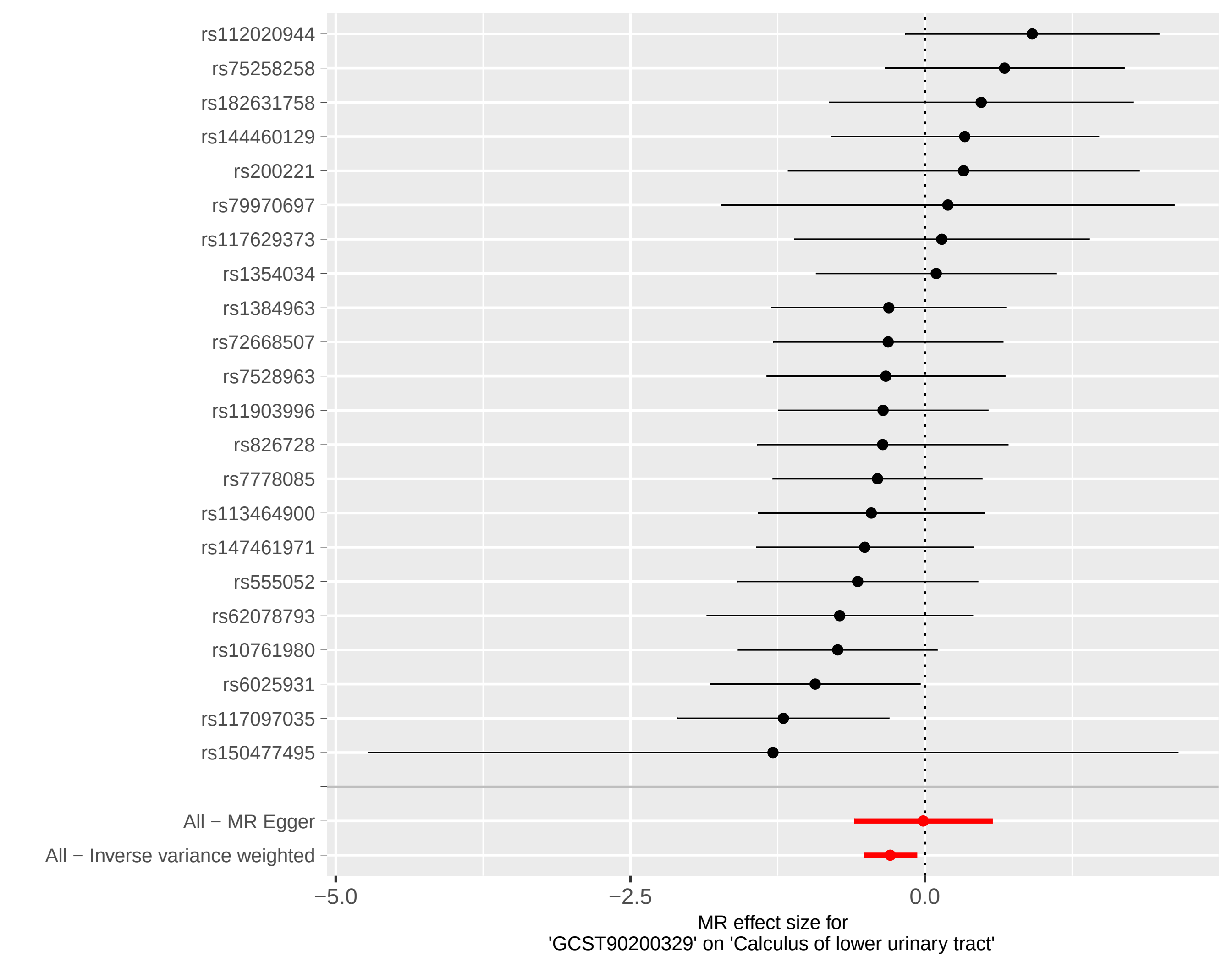
rs112020944
rs75258258
rs182631758
rs144460129
rs200221
rs79970697
rs117629373
rs1354034
rs1384963
rs72668507
rs7528963
rs11903996
rs826728
rs7778085
rs113464900
rs147461971
rs555052
rs62078793
rs10761980
rs6025931
rs117097035
rs150477495
All − MR Egger
All − Inverse variance weighted
−5.0
−2.5
0.0
MR effect size for
'GCST90200329' on 'Calculus of lower urinary tract'

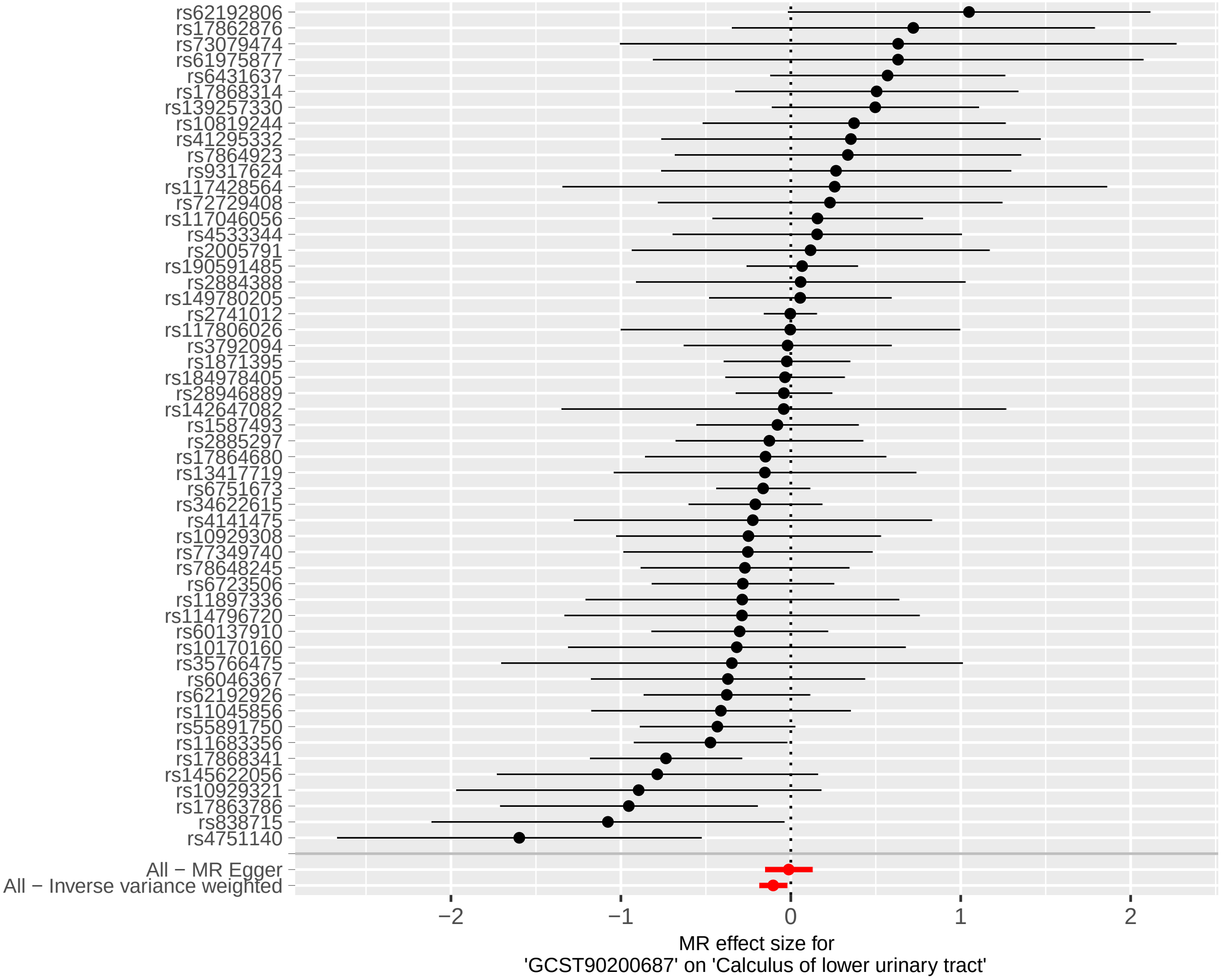

rs62192806
rs17862876
rs73079474
rs61975877
rs6431637
rs17868314
rs139257330
rs10819244
rs41295332
rs7864923
rs9317624
rs117428564
rs72729408
rs117046056
rs4533344
rs2005791
rs190591485
rs2884388
rs149780205
rs2741012
rs117806026
rs3792094
rs1871395
rs184978405
rs28946889
rs142647082
rs1587493
rs2885297
rs17864680
rs13417719
rs6751673
rs34622615
rs4141475
rs10929308
rs77349740
rs78648245
rs6723506
rs11897336
rs114796720
rs60137910
rs10170160
rs35766475
rs6046367
rs62192926
rs11045856
rs55891750
rs11683356
rs17868341
rs145622056
rs10929321
rs17863786
rs838715
rs4751140
All − MR Egger
All − Inverse variance weighted
−2
−1
0
1
2
MR effect size for
'GCST90200687' on 'Calculus of lower urinary tract'

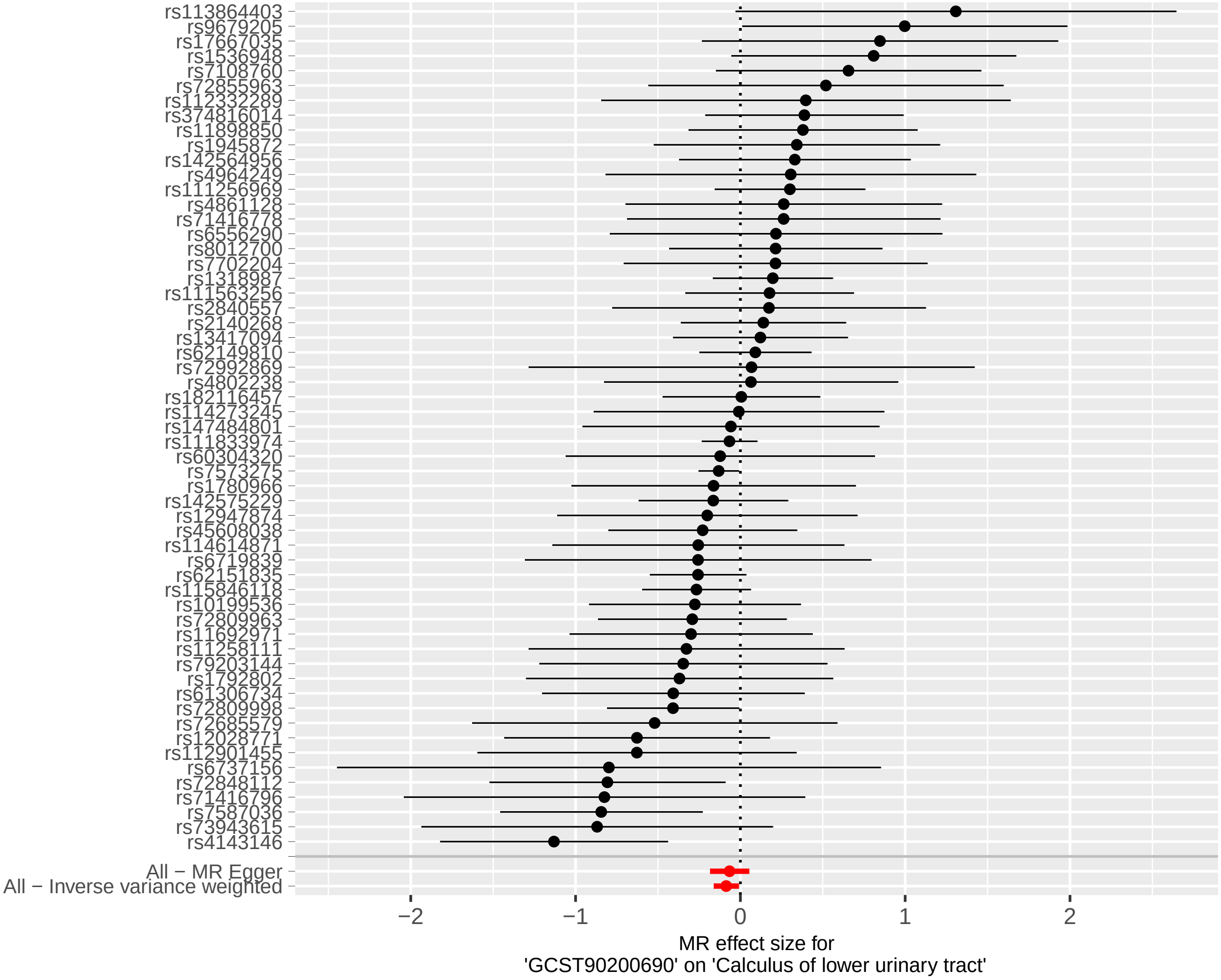

rs113864403
rs9679205
rs17667035
rs1536948
rs7108760
rs72855963
rs112332289
rs374816014
rs11898850
rs1945872
rs142564956
rs4964249
rs111256969
rs4861128
rs71416778
rs6556290
rs8012700
rs7702204
rs1318987
rs111563256
rs2840557
rs2140268
rs13417094
rs62149810
rs72992869
rs4802238
rs182116457
rs114273245
rs147484801
rs111833974
rs60304320
rs7573275
rs1780966
rs142575229
rs12947874
rs45608038
rs114614871
rs6719839
rs62151835
rs115846118
rs10199536
rs72809963
rs11692971
rs11258111
rs79203144
rs1792802
rs61306734
rs72809998
rs72685579
rs12028771
rs112901455
rs6737156
rs72848112
rs71416796
rs7587036
rs73943615
rs4143146
All − MR Egger
All − Inverse variance weighted
−2
−1
0
1
2
MR effect size for
'GCST90200690' on 'Calculus of lower urinary tract'

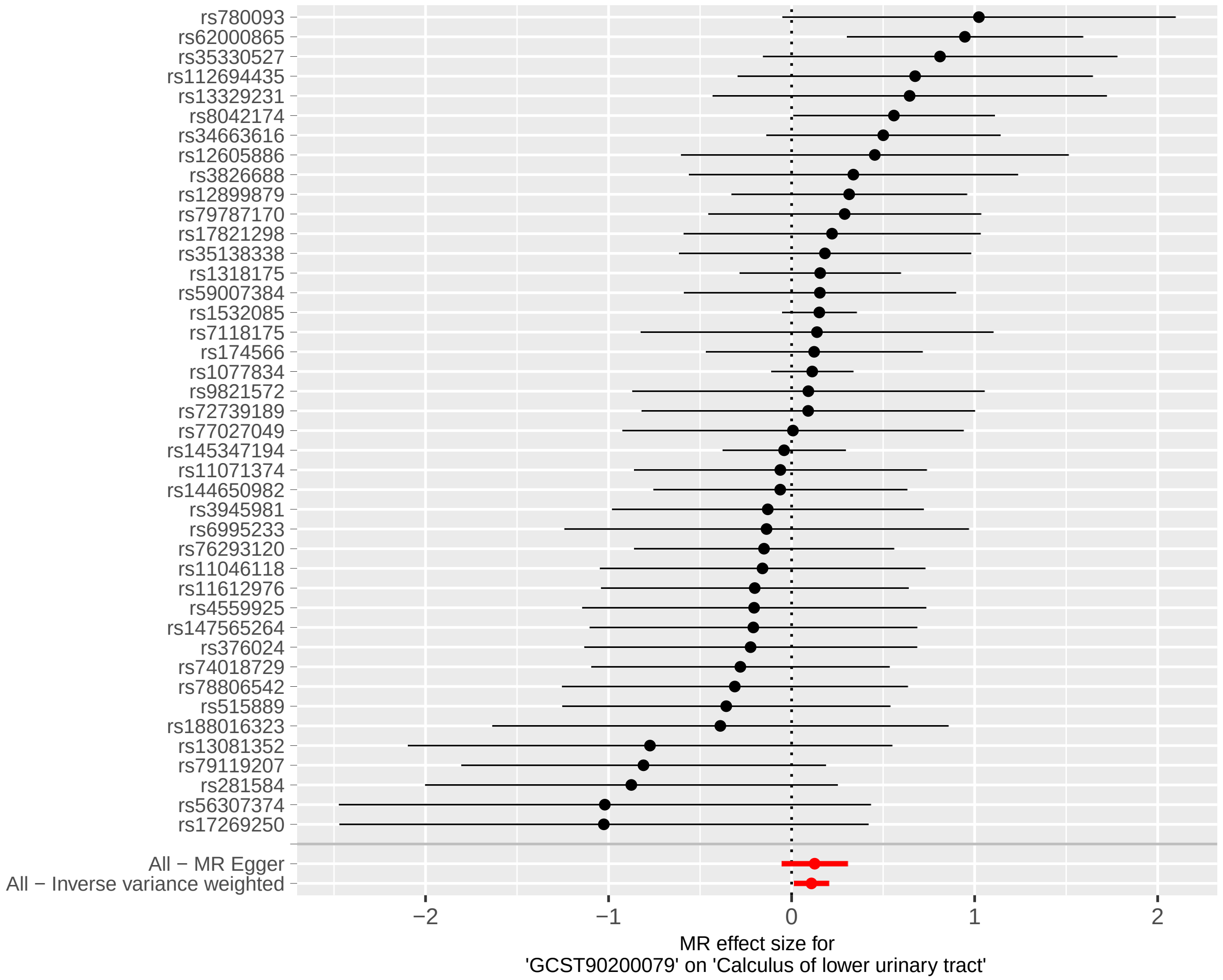

rs780093
rs62000865
rs35330527
rs112694435
rs13329231
rs8042174
rs34663616
rs12605886
rs3826688
rs12899879
rs79787170
rs17821298
rs35138338
rs1318175
rs59007384
rs1532085
rs7118175
rs174566
rs1077834
rs9821572
rs72739189
rs77027049
rs145347194
rs11071374
rs144650982
rs3945981
rs6995233
rs76293120
rs11046118
rs11612976
rs4559925
rs147565264
rs376024
rs74018729
rs78806542
rs515889
rs188016323
rs13081352
rs79119207
rs281584
rs56307374
rs17269250
All − MR Egger
All − Inverse variance weighted
−2
−1
0
1
2
MR effect size for
'GCST90200079' on 'Calculus of lower urinary tract'

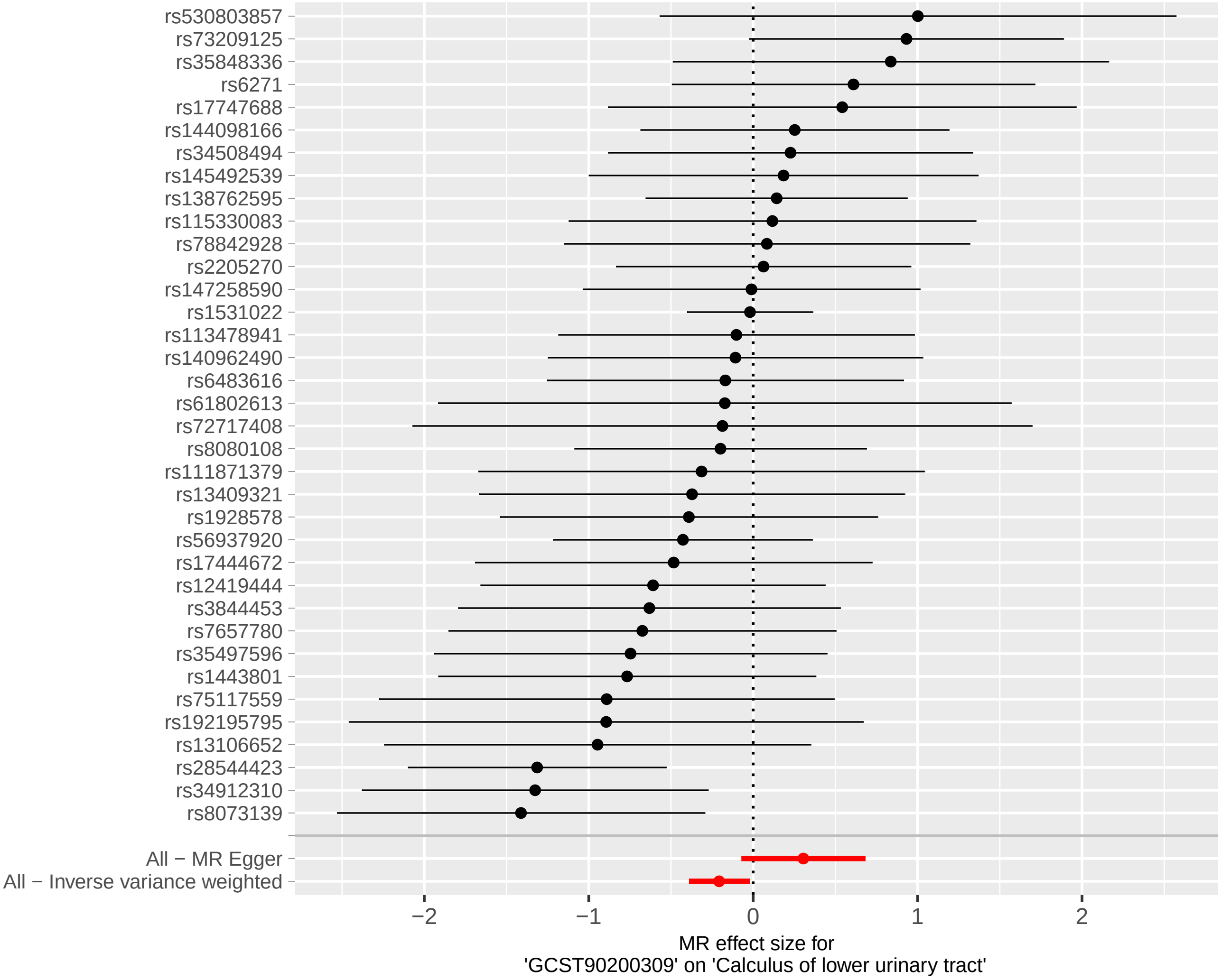

rs530803857
rs73209125
rs35848336
rs6271
rs17747688
rs144098166
rs34508494
rs145492539
rs138762595
rs115330083
rs78842928
rs2205270
rs147258590
rs1531022
rs113478941
rs140962490
rs6483616
rs61802613
rs72717408
rs8080108
rs111871379
rs13409321
rs1928578
rs56937920
rs17444672
rs12419444
rs3844453
rs7657780
rs35497596
rs1443801
rs75117559
rs192195795
rs13106652
rs28544423
rs34912310
rs8073139
All − MR Egger
All − Inverse variance weighted
−2
−1
0
1
2
MR effect size for
'GCST90200309' on 'Calculus of lower urinary tract'

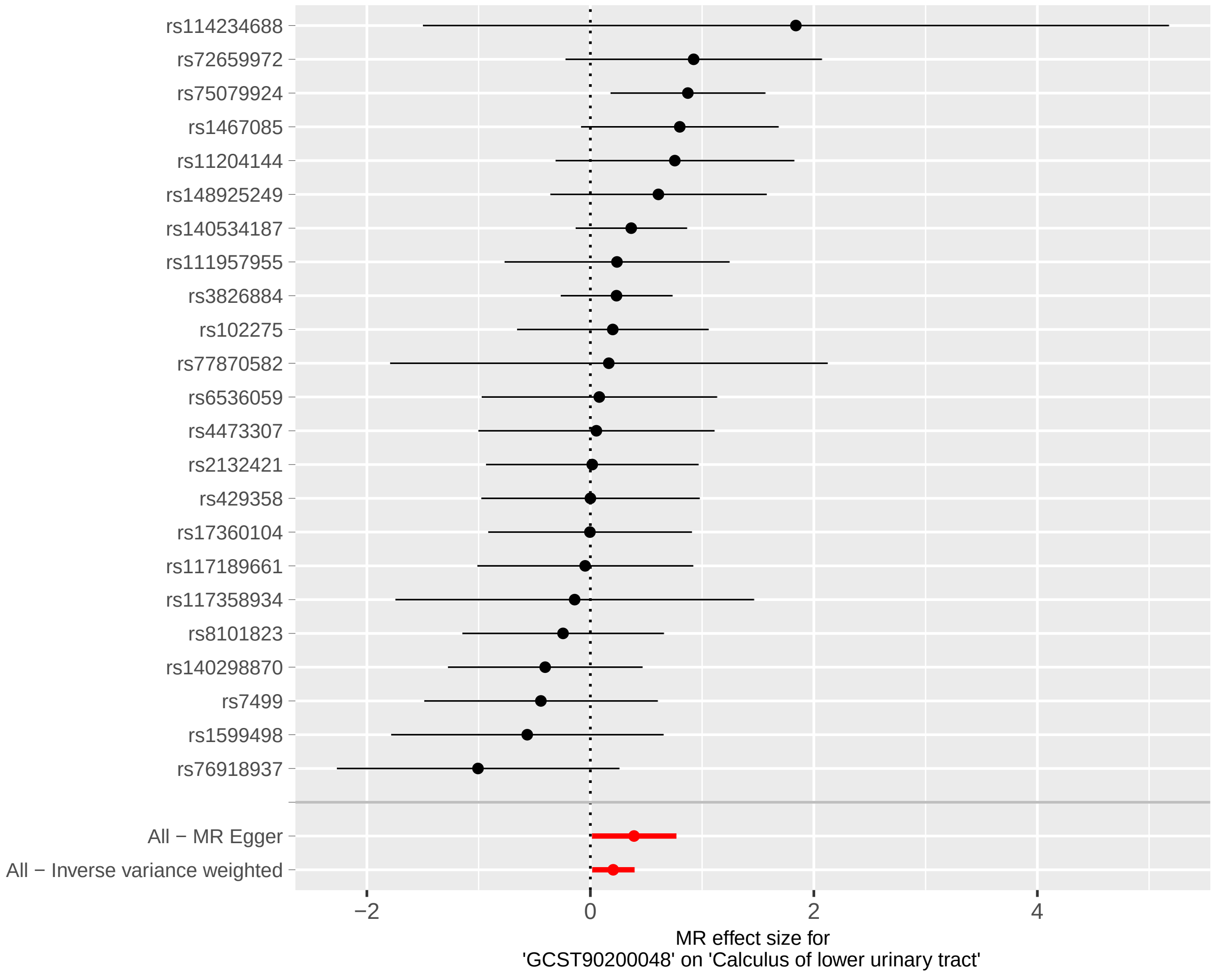

rs114234688
rs72659972
rs75079924
rs1467085
rs11204144
rs148925249
rs140534187
rs111957955
rs3826884
rs102275
rs77870582
rs6536059
rs4473307
rs2132421
rs429358
rs17360104
rs117189661
rs117358934
rs8101823
rs140298870
rs7499
rs1599498
rs76918937
All − MR Egger
All − Inverse variance weighted
−2
0
2
4
MR effect size for
'GCST90200048' on 'Calculus of lower urinary tract'

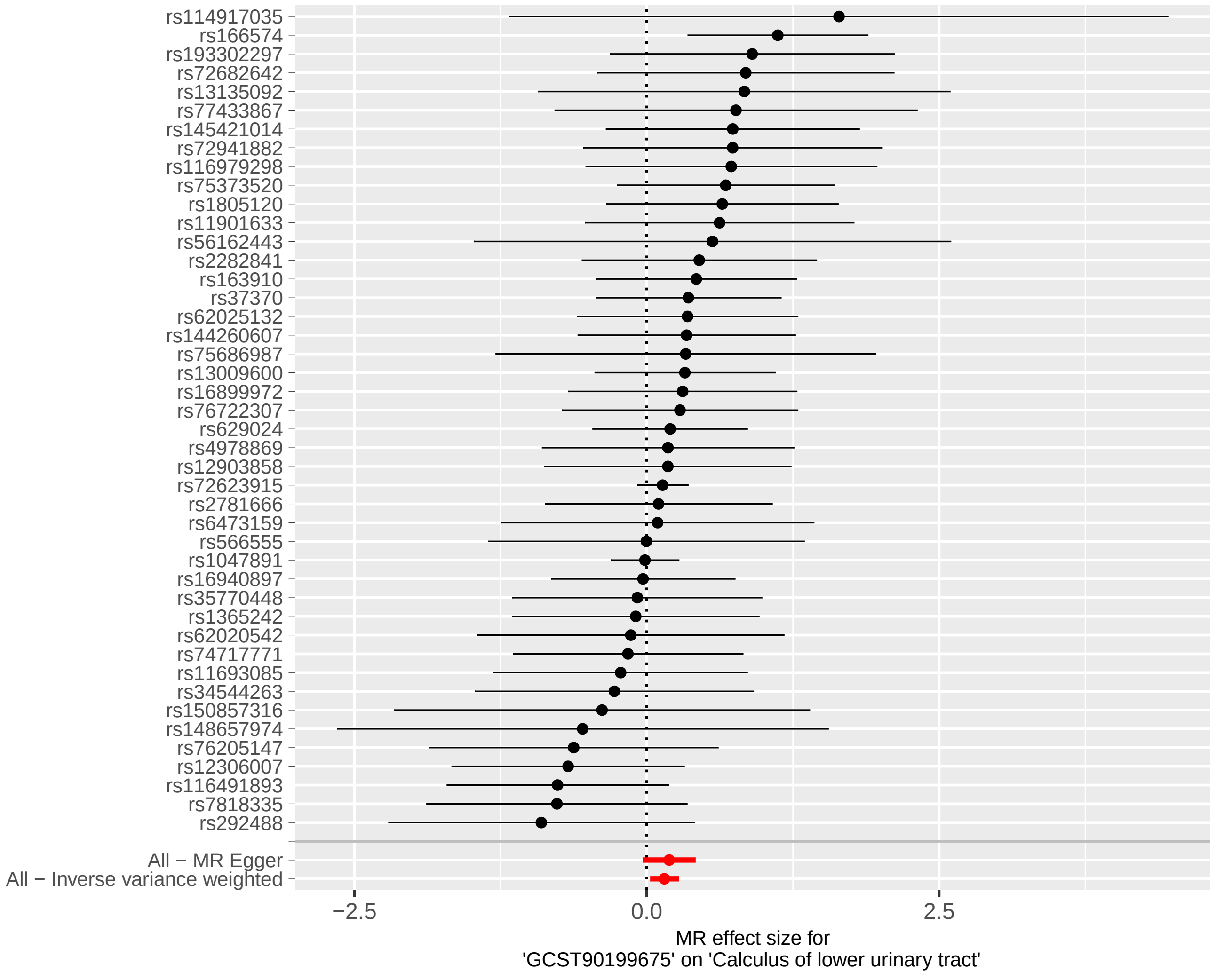
rs114917035
rs166574
rs193302297
rs72682642
rs13135092
rs77433867
rs145421014
rs72941882
rs116979298
rs75373520
rs1805120
rs11901633
rs56162443
rs2282841
rs163910
rs37370
rs62025132
rs144260607
rs75686987
rs13009600
rs16899972
rs76722307
rs629024
rs4978869
rs12903858
rs72623915
rs2781666
rs6473159
rs566555
rs1047891
rs16940897
rs35770448
rs1365242
rs62020542
rs74717771
rs11693085
rs34544263
rs150857316
rs148657974
rs76205147
rs12306007
rs116491893
rs7818335
rs292488
All − MR Egger
All − Inverse variance weighted
−2.5
0.0
2.5
MR effect size for
'GCST90199675' on 'Calculus of lower urinary tract'

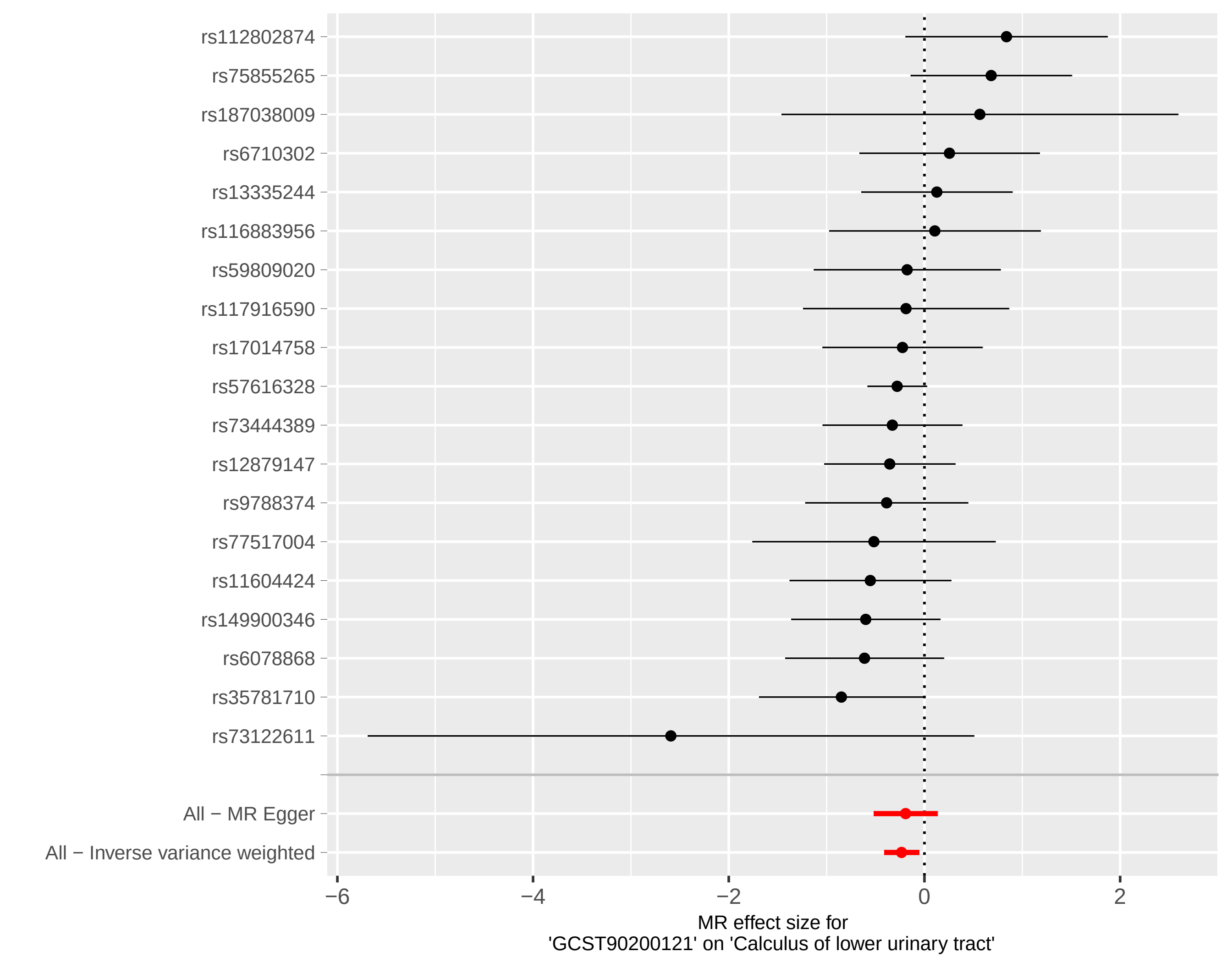

rs112802874
rs75855265
rs187038009
rs6710302
rs13335244
rs116883956
rs59809020
rs117916590
rs17014758
rs57616328
rs73444389
rs12879147
rs9788374
rs77517004
rs11604424
rs149900346
rs6078868
rs35781710
rs73122611
All − MR Egger
All − Inverse variance weighted
−6
−4
−2
0
2
MR effect size for
'GCST90200121' on 'Calculus of lower urinary tract'

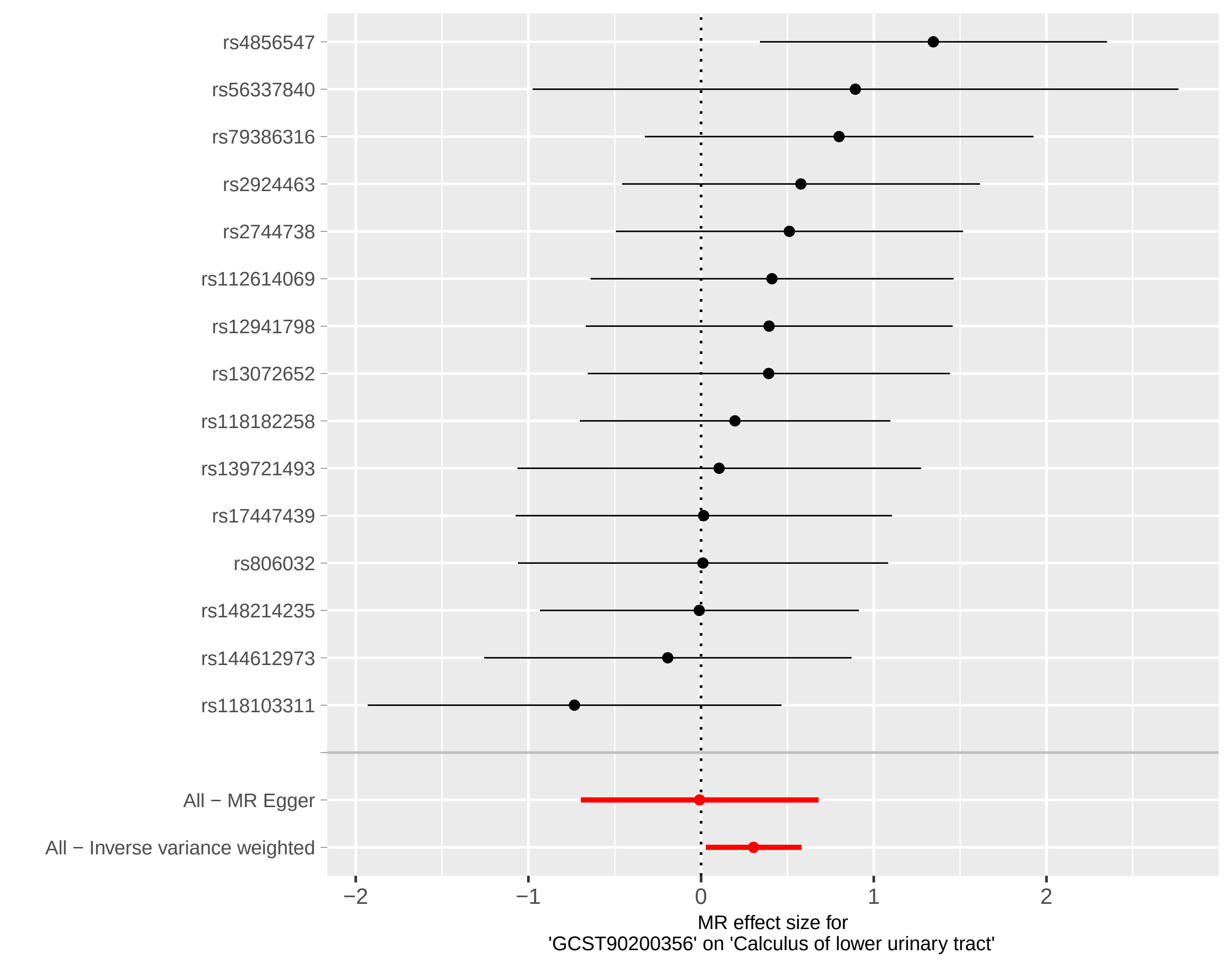
rs4856547
rs56337840
rs79386316
rs2924463
rs2744738
rs112614069
rs12941798
rs13072652
rs118182258
rs139721493
rs17447439
rs806032
rs148214235
rs144612973
rs118103311
All − MR Egger
All − Inverse variance weighted
−2
−1
0
1
2
MR effect size for
'GCST90200356' on 'Calculus of lower urinary tract'

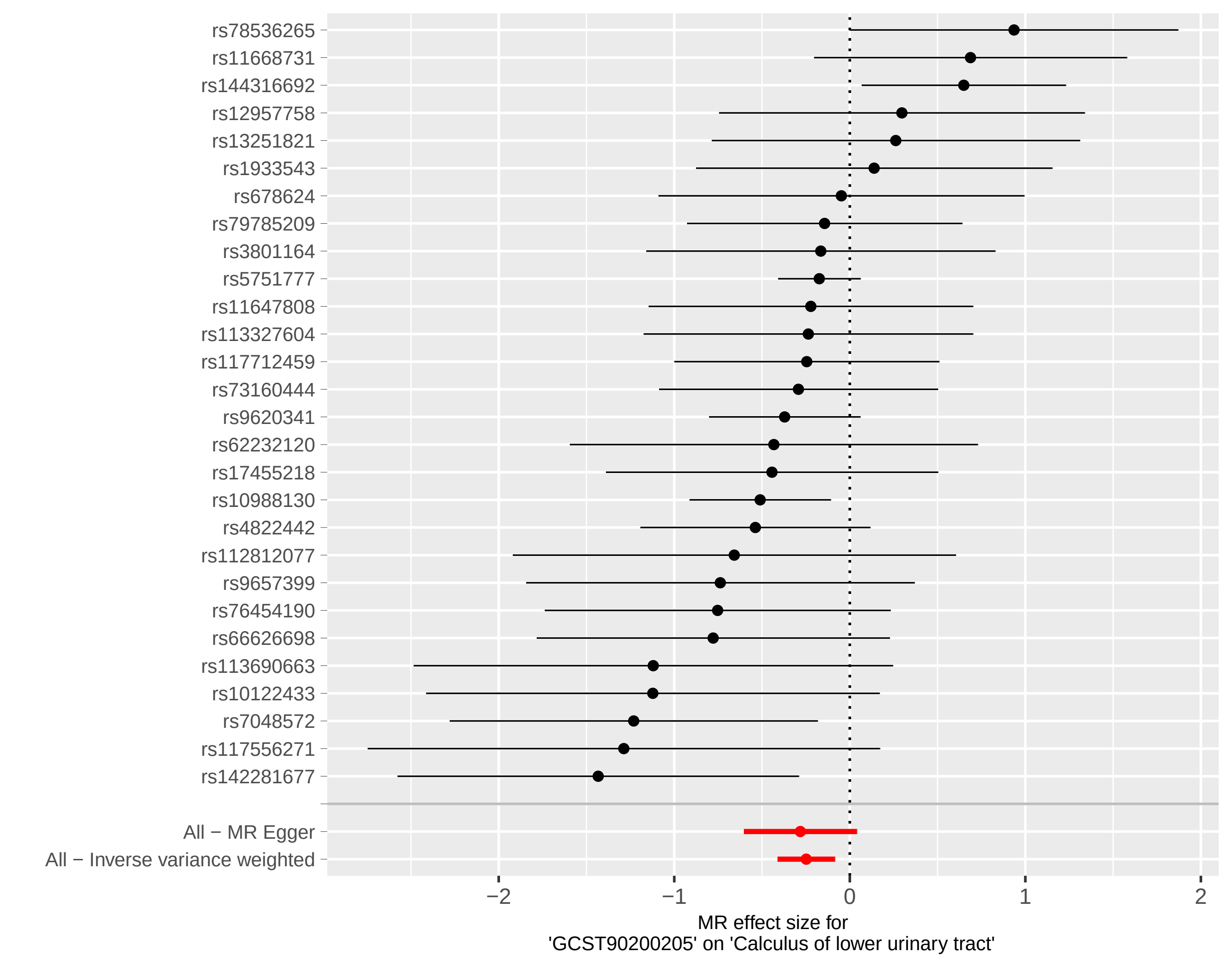
rs78536265
rs11668731
rs144316692
rs12957758
rs13251821
rs1933543
rs678624
rs79785209
rs3801164
rs5751777
rs11647808
rs113327604
rs117712459
rs73160444
rs9620341
rs62232120
rs17455218
rs10988130
rs4822442
rs112812077
rs9657399
rs76454190
rs66626698
rs113690663
rs10122433
rs7048572
rs117556271
rs142281677
All − MR Egger
All − Inverse variance weighted
−2
−1
0
1
2
MR effect size for
'GCST90200205' on 'Calculus of lower urinary tract'

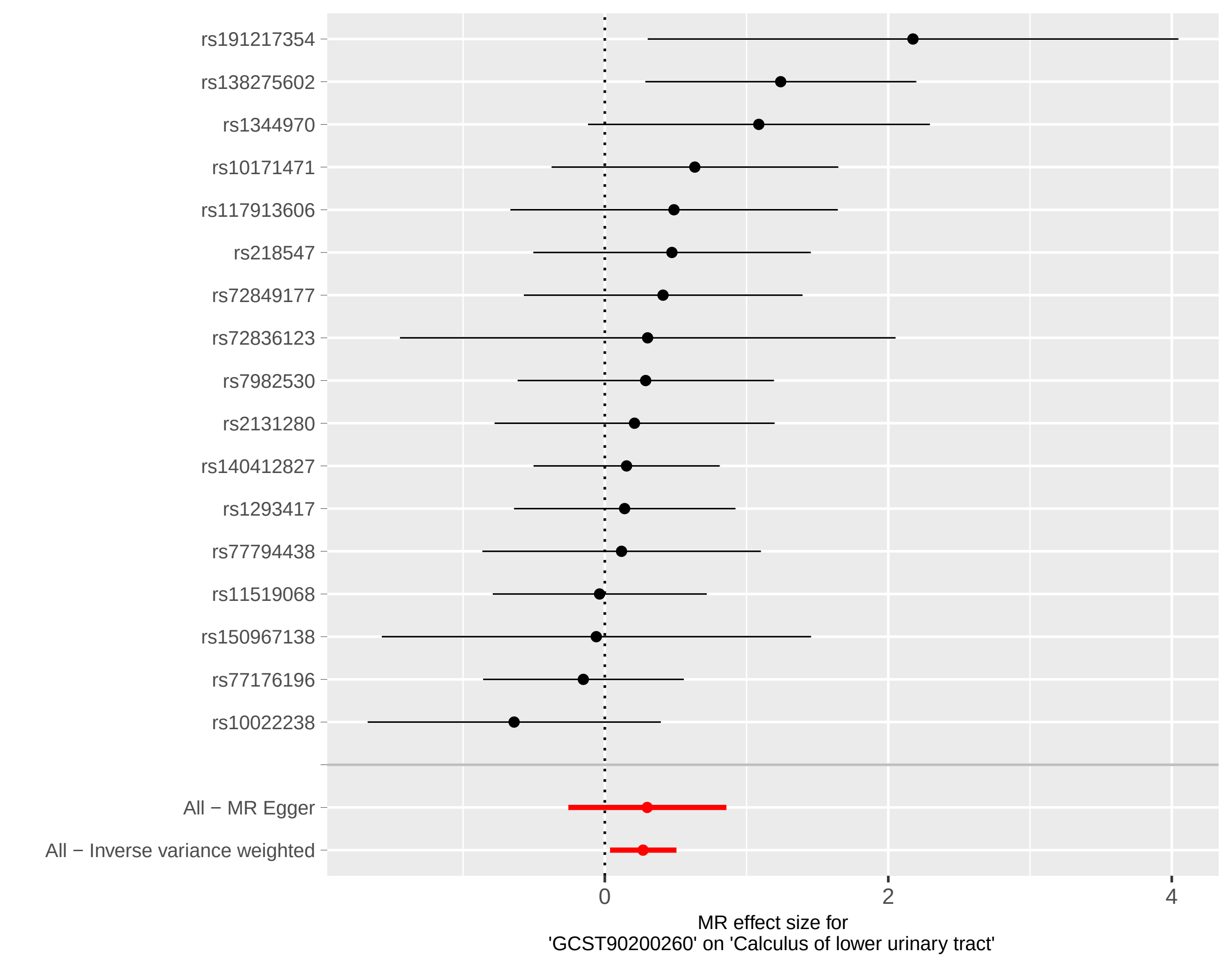
rs191217354
rs138275602
rs1344970
rs10171471
rs117913606
rs218547
rs72849177
rs72836123
rs7982530
rs2131280
rs140412827
rs1293417
rs77794438
rs11519068
rs150967138
rs77176196
rs10022238
All − MR Egger
All − Inverse variance weighted
0
2
4
MR effect size for
'GCST90200260' on 'Calculus of lower urinary tract'

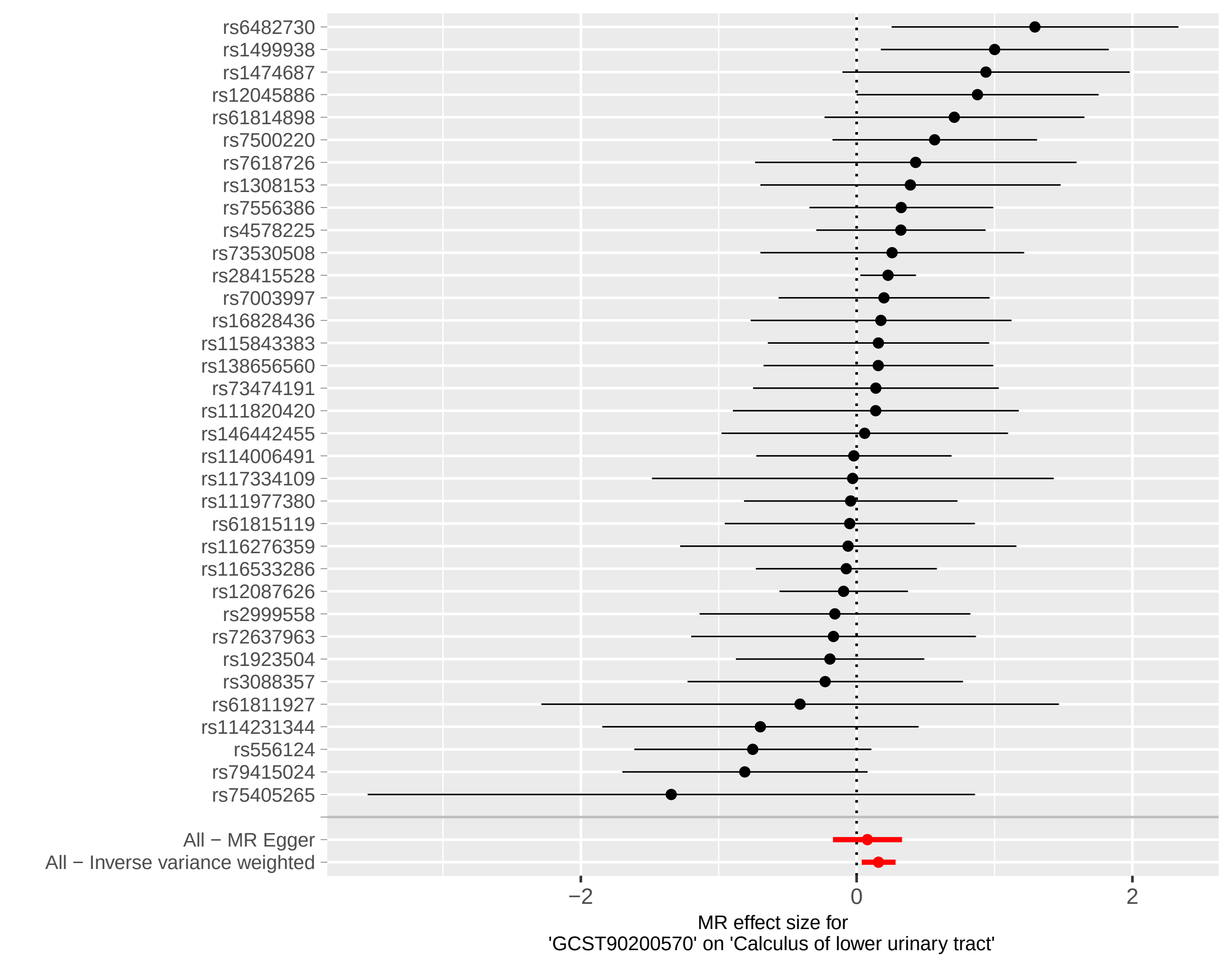
rs6482730
rs1499938
rs1474687
rs12045886
rs61814898
rs7500220
rs7618726
rs1308153
rs7556386
rs4578225
rs73530508
rs28415528
rs7003997
rs16828436
rs115843383
rs138656560
rs73474191
rs111820420
rs146442455
rs114006491
rs117334109
rs111977380
rs61815119
rs116276359
rs116533286
rs12087626
rs2999558
rs72637963
rs1923504
rs3088357
rs61811927
rs114231344
rs556124
rs79415024
rs75405265
All − MR Egger
All − Inverse variance weighted
−2
0
2
MR effect size for
'GCST90200570' on 'Calculus of lower urinary tract'

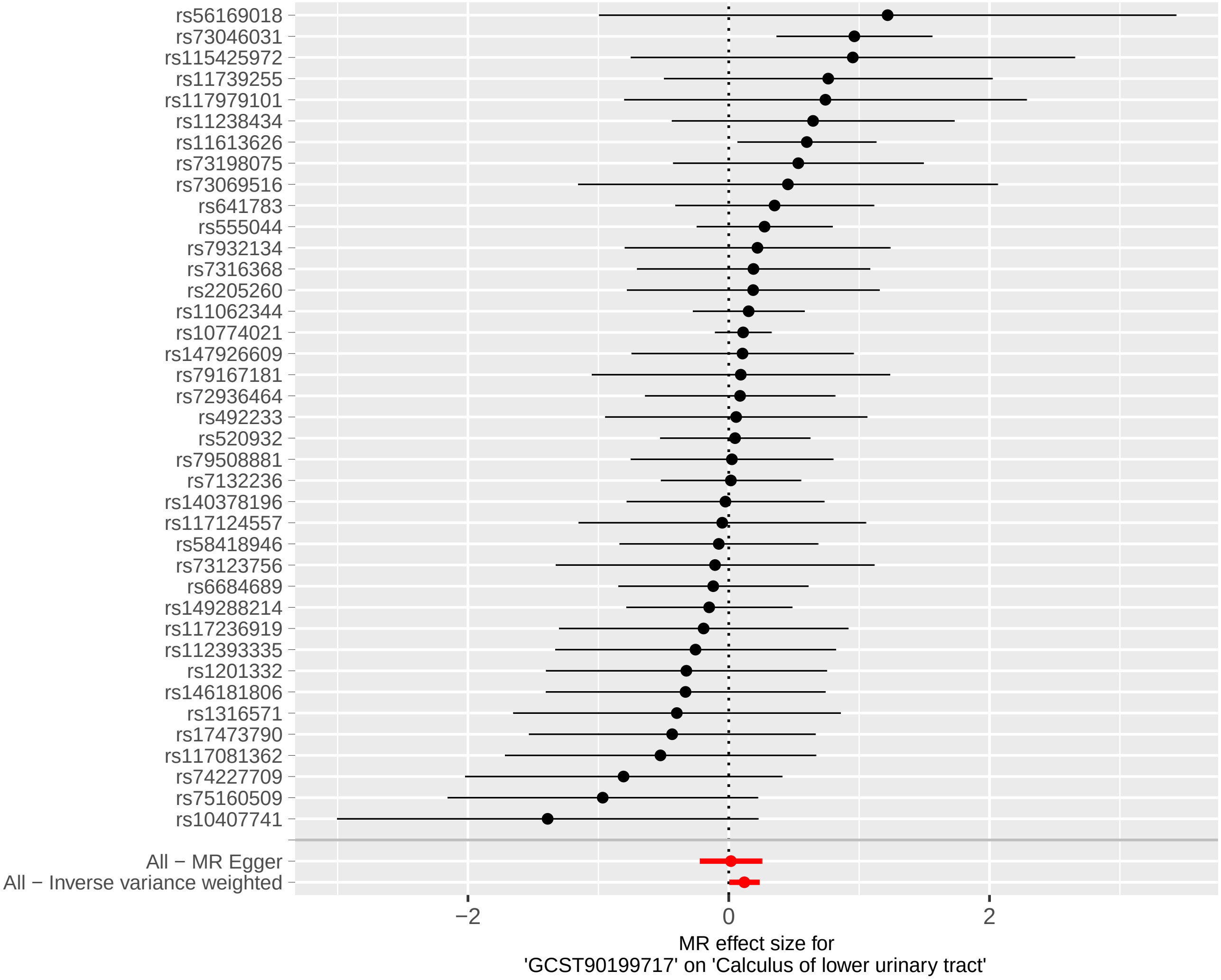
rs56169018
rs73046031
rs115425972
rs11739255
rs117979101
rs11238434
rs11613626
rs73198075
rs73069516
rs641783
rs555044
rs7932134
rs7316368
rs2205260
rs11062344
rs10774021
rs147926609
rs79167181
rs72936464
rs492233
rs520932
rs79508881
rs7132236
rs140378196
rs117124557
rs58418946
rs73123756
rs6684689
rs149288214
rs117236919
rs112393335
rs1201332
rs146181806
rs1316571
rs17473790
rs117081362
rs74227709
rs75160509
rs10407741
All − MR Egger
All − Inverse variance weighted
−2
0
2
MR effect size for
'GCST90199717' on 'Calculus of lower urinary tract'

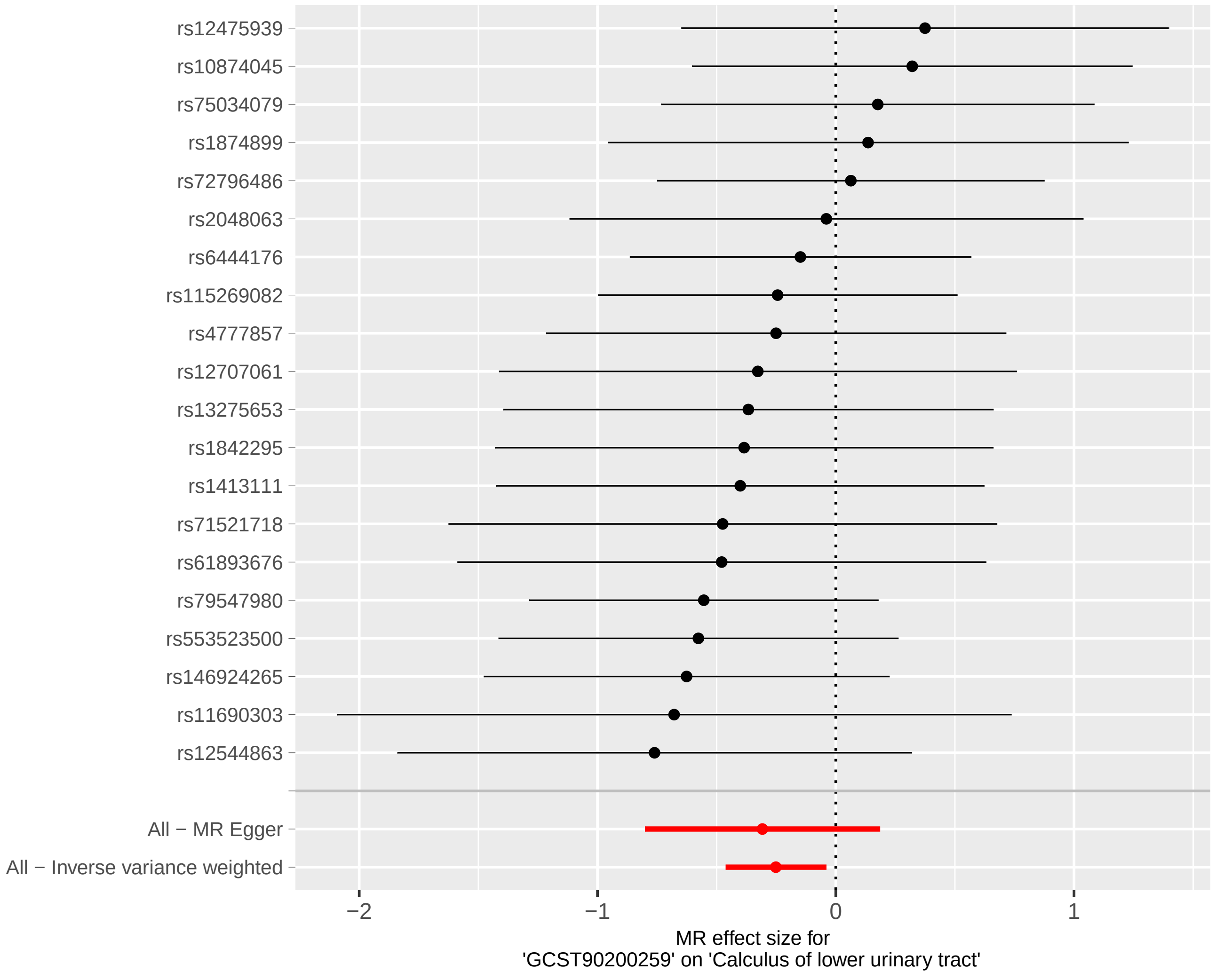

rs12475939
rs10874045
rs75034079
rs1874899
rs72796486
rs2048063
rs6444176
rs115269082
rs4777857
rs12707061
rs13275653
rs1842295
rs1413111
rs71521718
rs61893676
rs79547980
rs553523500
rs146924265
rs11690303
rs12544863
All − MR Egger
All − Inverse variance weighted
−2
−1
0
1
MR effect size for
'GCST90200259' on 'Calculus of lower urinary tract'

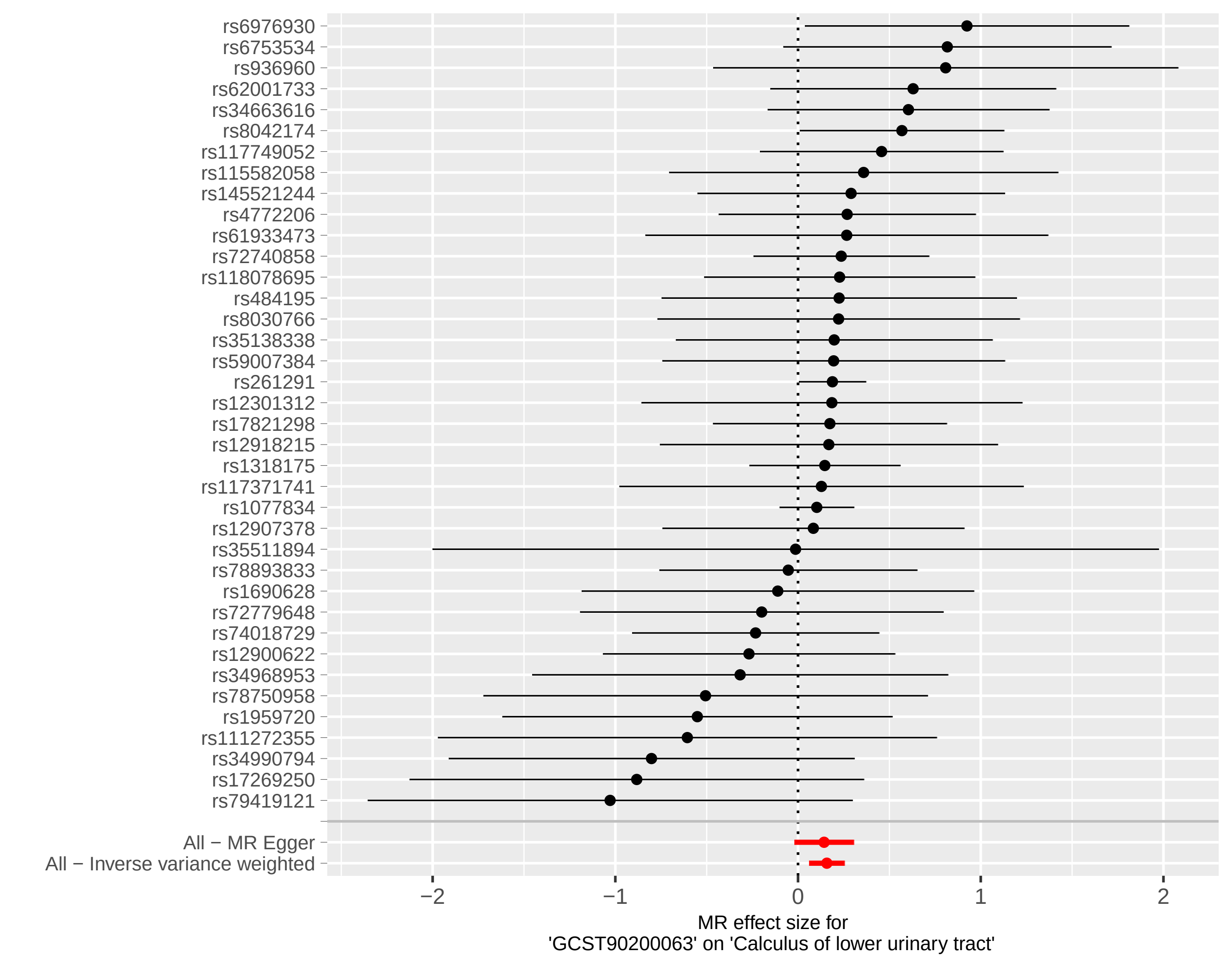
rs6976930
rs6753534
rs936960
rs62001733
rs34663616
rs8042174
rs117749052
rs115582058
rs145521244
rs4772206
rs61933473
rs72740858
rs118078695
rs484195
rs8030766
rs35138338
rs59007384
rs261291
rs12301312
rs17821298
rs12918215
rs1318175
rs117371741
rs1077834
rs12907378
rs35511894
rs78893833
rs1690628
rs72779648
rs74018729
rs12900622
rs34968953
rs78750958
rs1959720
rs111272355
rs34990794
rs17269250
rs79419121
All − MR Egger
All − Inverse variance weighted
−2
−1
0
1
2
MR effect size for
'GCST90200063' on 'Calculus of lower urinary tract'

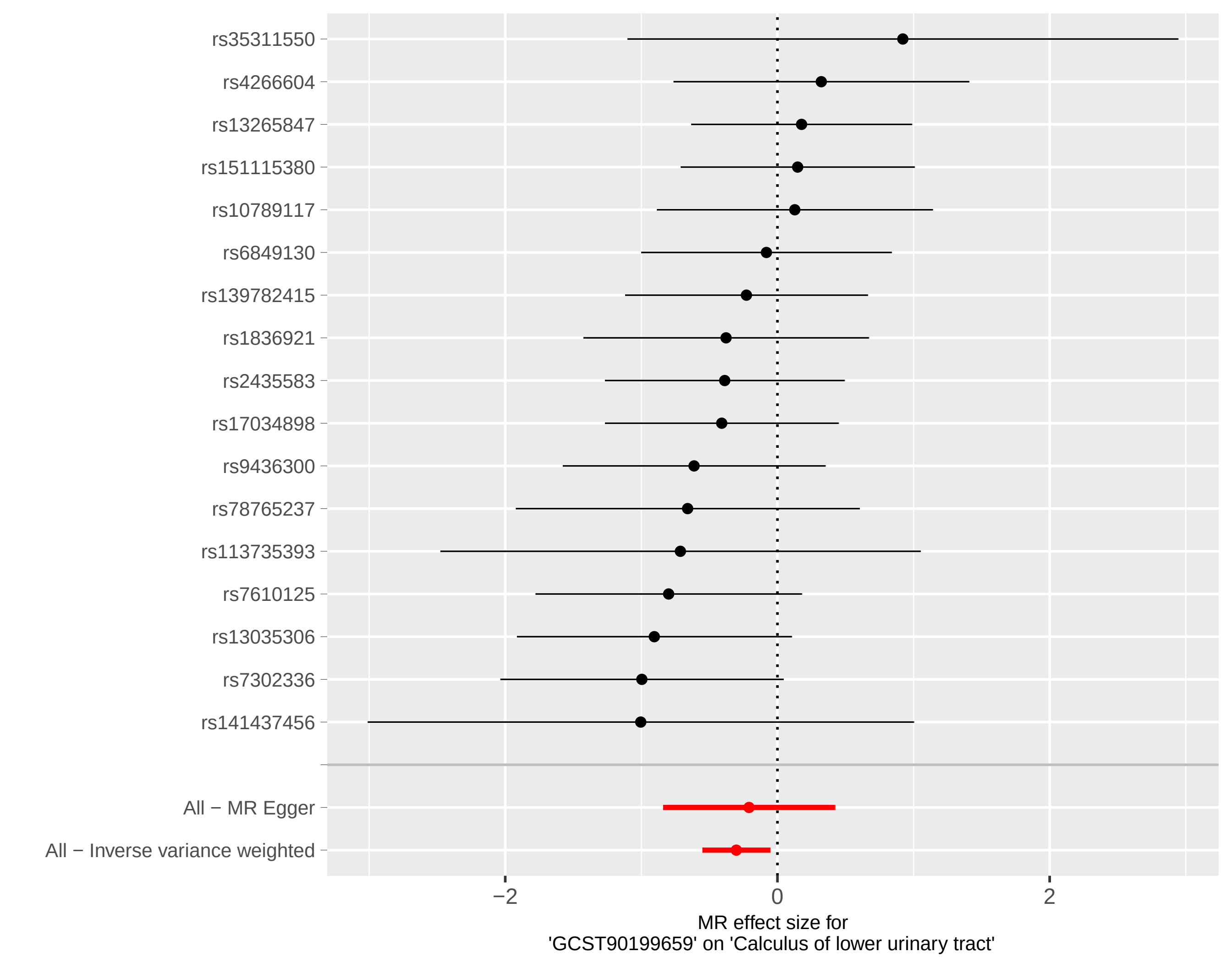
rs35311550
rs4266604
rs13265847
rs151115380
rs10789117
rs6849130
rs139782415
rs1836921
rs2435583
rs17034898
rs9436300
rs78765237
rs113735393
rs7610125
rs13035306
rs7302336
rs141437456
All − MR Egger
All − Inverse variance weighted
−2
0
2
MR effect size for
'GCST90199659' on 'Calculus of lower urinary tract'

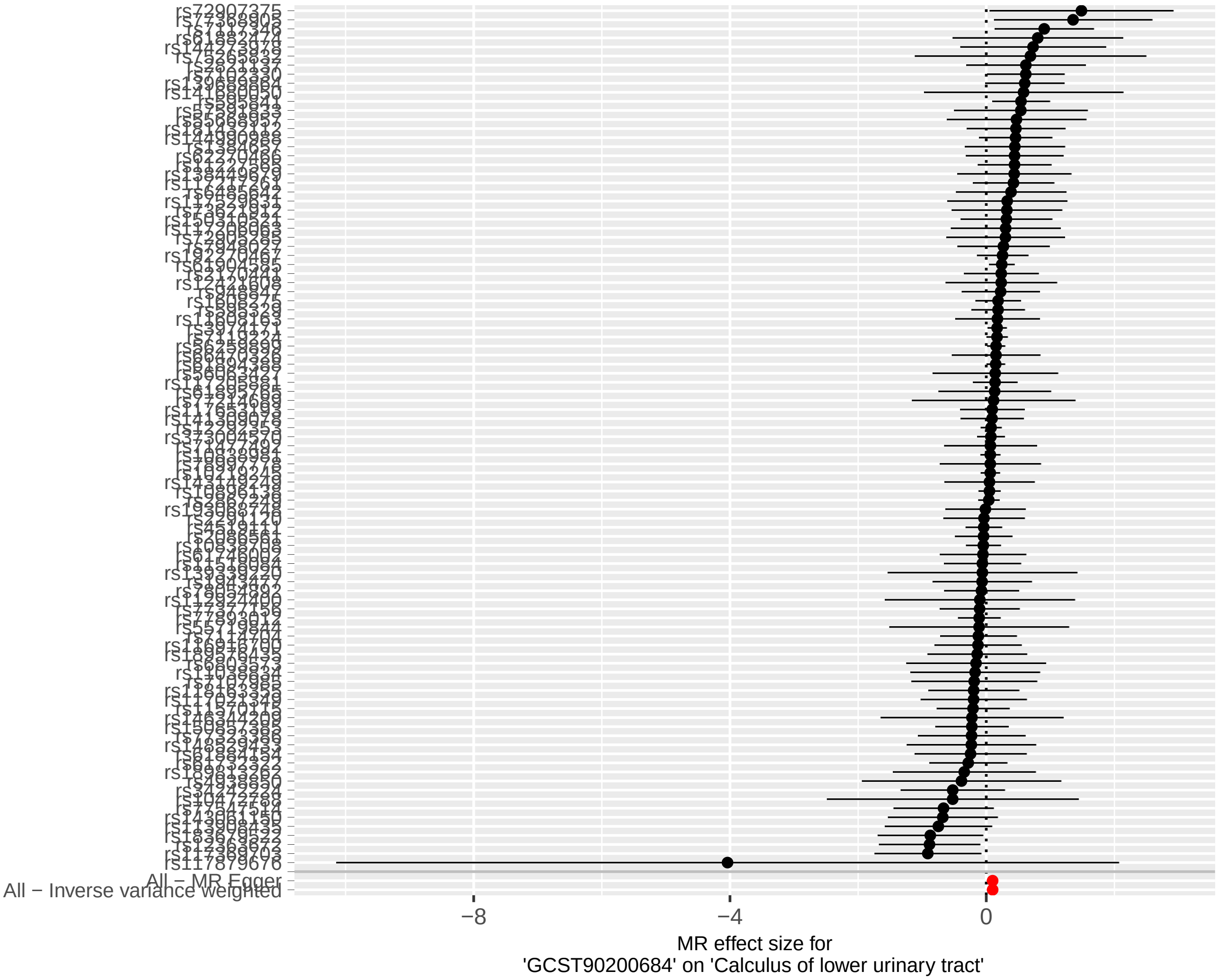

rs72907375
rs117879676
All − MR Egger
All − Inverse variance weighted
−8
−4
0
MR effect size for
'GCST90200684' on 'Calculus of lower urinary tract'

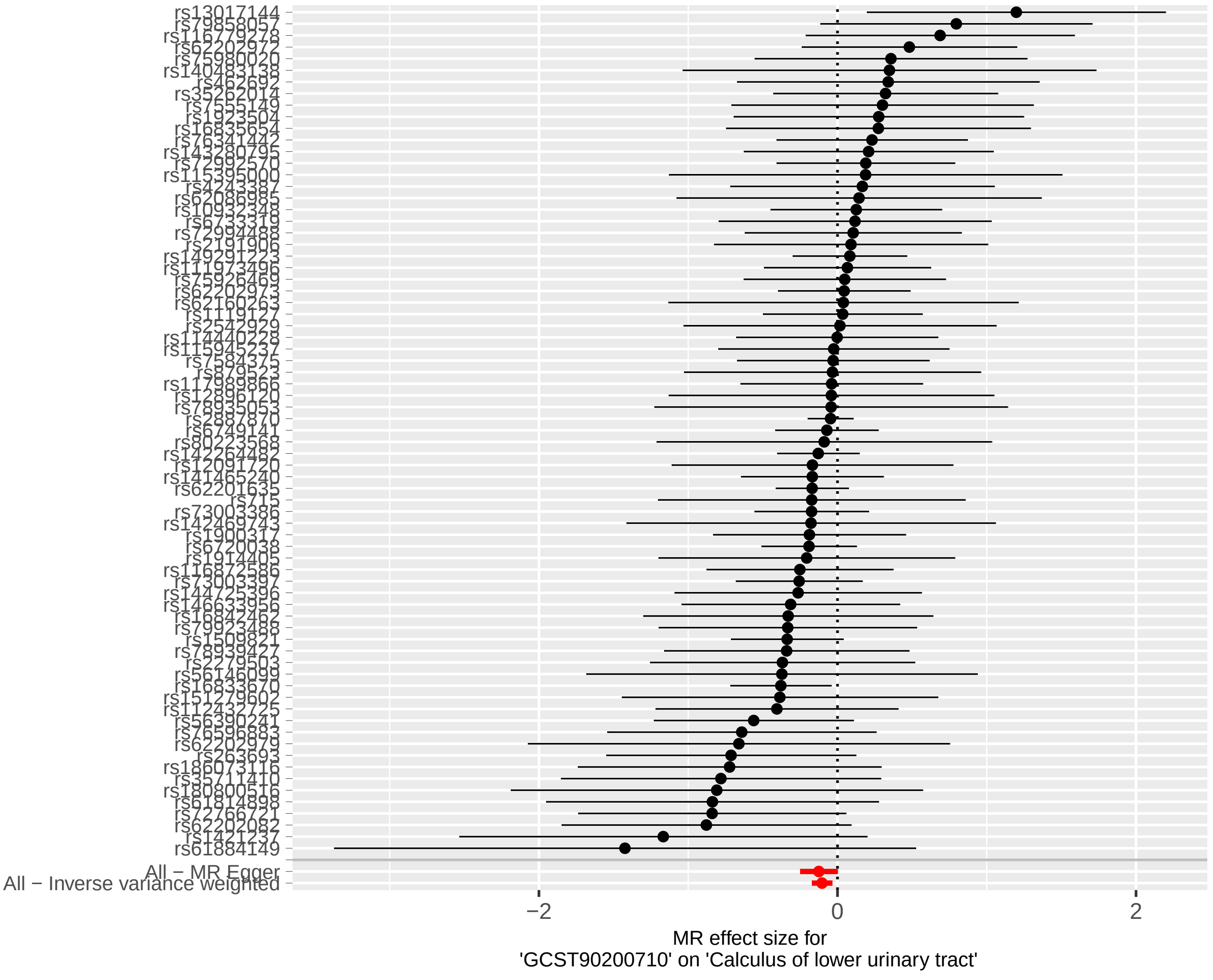
rs13017144
rs79858057
rs116779278
rs62202972
rs75980020
rs140483138
rs462692
rs35262014
rs7555149
rs1923504
rs16835654
rs76341442
rs143280795
rs72992570
rs115395000
rs4243387
rs62086985
rs10932348
rs6733319
rs72994488
rs2191906
rs149291223
rs111973496
rs75926469
rs62202973
rs62160263
rs1119127
rs2542929
rs114440228
rs115945237
rs7584375
rs879523
rs117989866
rs12896120
rs78935053
rs2887870
rs6749141
rs80223568
rs142264482
rs12091720
rs141465240
rs62201635
rs715
rs73003386
rs142469743
rs1900317
rs6720038
rs1914405
rs116872586
rs73003397
rs144725396
rs146633956
rs16842462
rs79923488
rs1509821
rs78939427
rs2279503
rs56146099
rs16833670
rs151279602
rs112432725
rs56390241
rs76596883
rs62202979
rs263693
rs186073116
rs35711410
rs180800516
rs61814898
rs72766721
rs62202082
rs1421237
rs61884149
All − MR Egger
All − Inverse variance weighted
−2
0
2
MR effect size for
'GCST90200710' on 'Calculus of lower urinary tract'

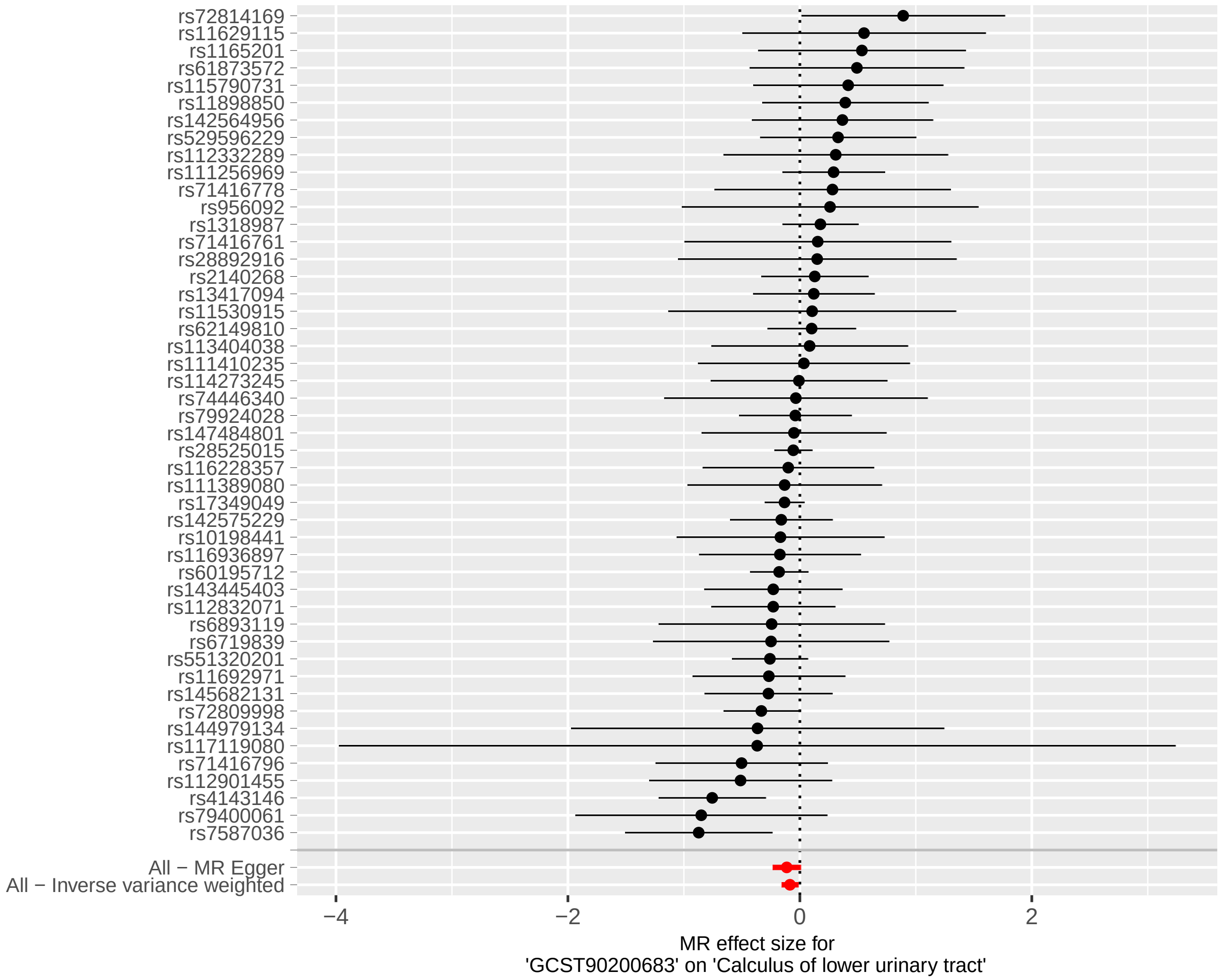

rs72814169
rs11629115
rs1165201
rs61873572
rs115790731
rs11898850
rs142564956
rs529596229
rs112332289
rs111256969
rs71416778
rs956092
rs1318987
rs71416761
rs28892916
rs2140268
rs13417094
rs11530915
rs62149810
rs113404038
rs111410235
rs114273245
rs74446340
rs79924028
rs147484801
rs28525015
rs116228357
rs111389080
rs17349049
rs142575229
rs10198441
rs116936897
rs60195712
rs143445403
rs112832071
rs6893119
rs6719839
rs551320201
rs11692971
rs145682131
rs72809998
rs144979134
rs117119080
rs71416796
rs112901455
rs4143146
rs79400061
rs7587036
All − MR Egger
All − Inverse variance weighted
−4
−2
0
2
MR effect size for
'GCST90200683' on 'Calculus of lower urinary tract'

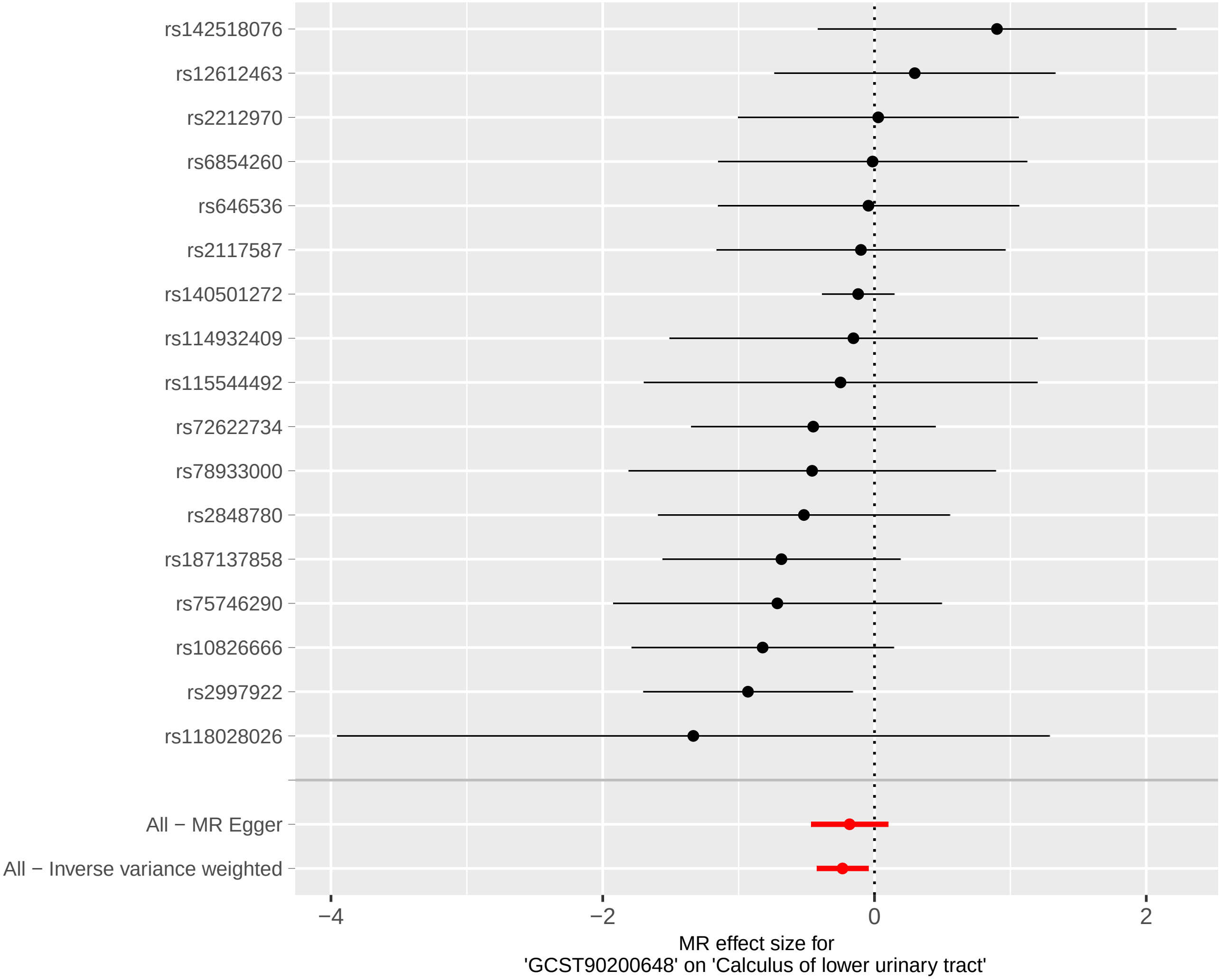
rs142518076
rs12612463
rs2212970
rs6854260
rs646536
rs2117587
rs140501272
rs114932409
rs115544492
rs72622734
rs78933000
rs2848780
rs187137858
rs75746290
rs10826666
rs2997922
rs118028026
All − MR Egger
All − Inverse variance weighted
−4
−2
0
2
MR effect size for
'GCST90200648' on 'Calculus of lower urinary tract'

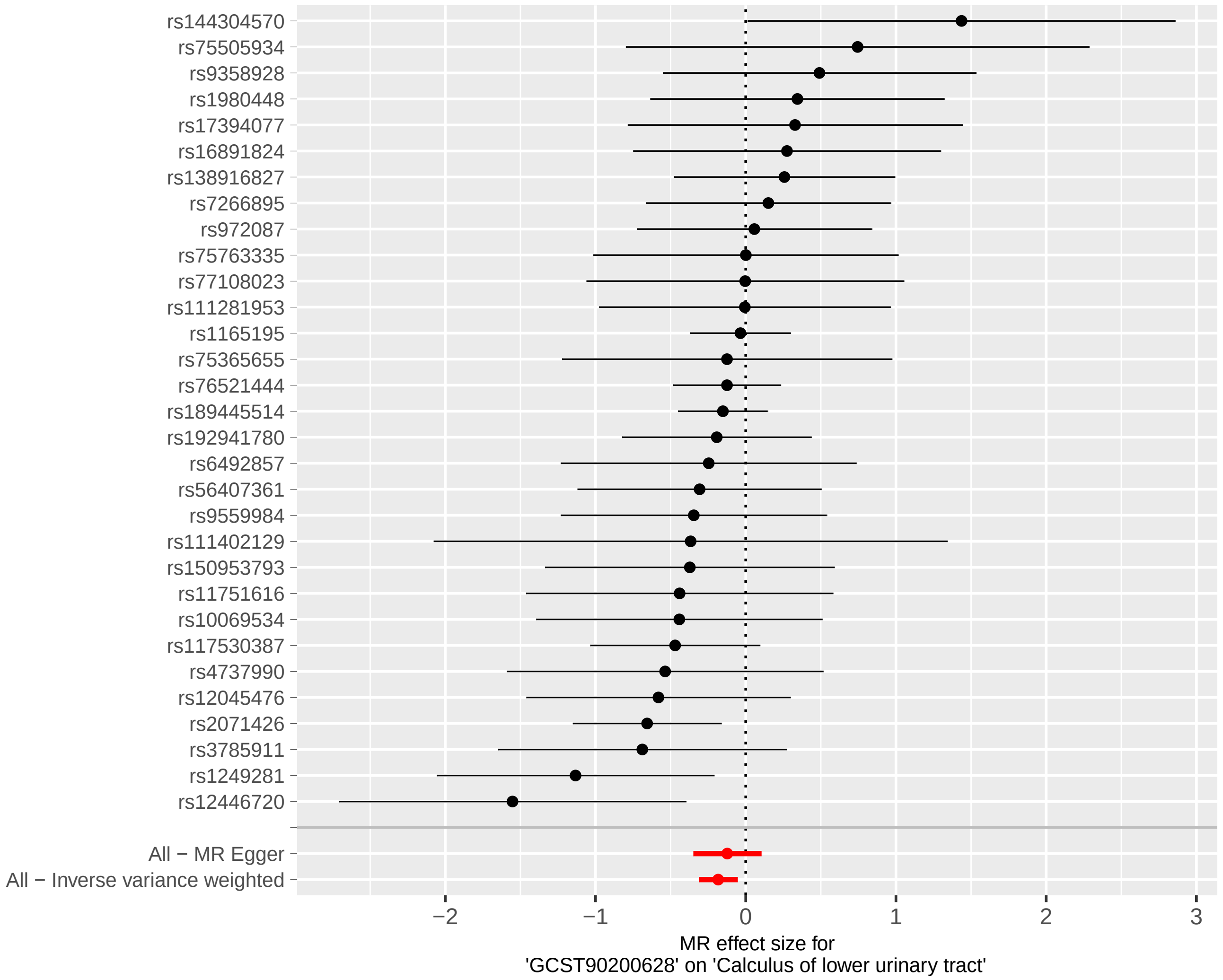
rs144304570
rs75505934
rs9358928
rs1980448
rs17394077
rs16891824
rs138916827
rs7266895
rs972087
rs75763335
rs77108023
rs111281953
rs1165195
rs75365655
rs76521444
rs189445514
rs192941780
rs6492857
rs56407361
rs9559984
rs111402129
rs150953793
rs11751616
rs10069534
rs117530387
rs4737990
rs12045476
rs2071426
rs3785911
rs1249281
rs12446720
All − MR Egger
All − Inverse variance weighted
−2
−1
0
1
2
3
MR effect size for
'GCST90200628' on 'Calculus of lower urinary tract'

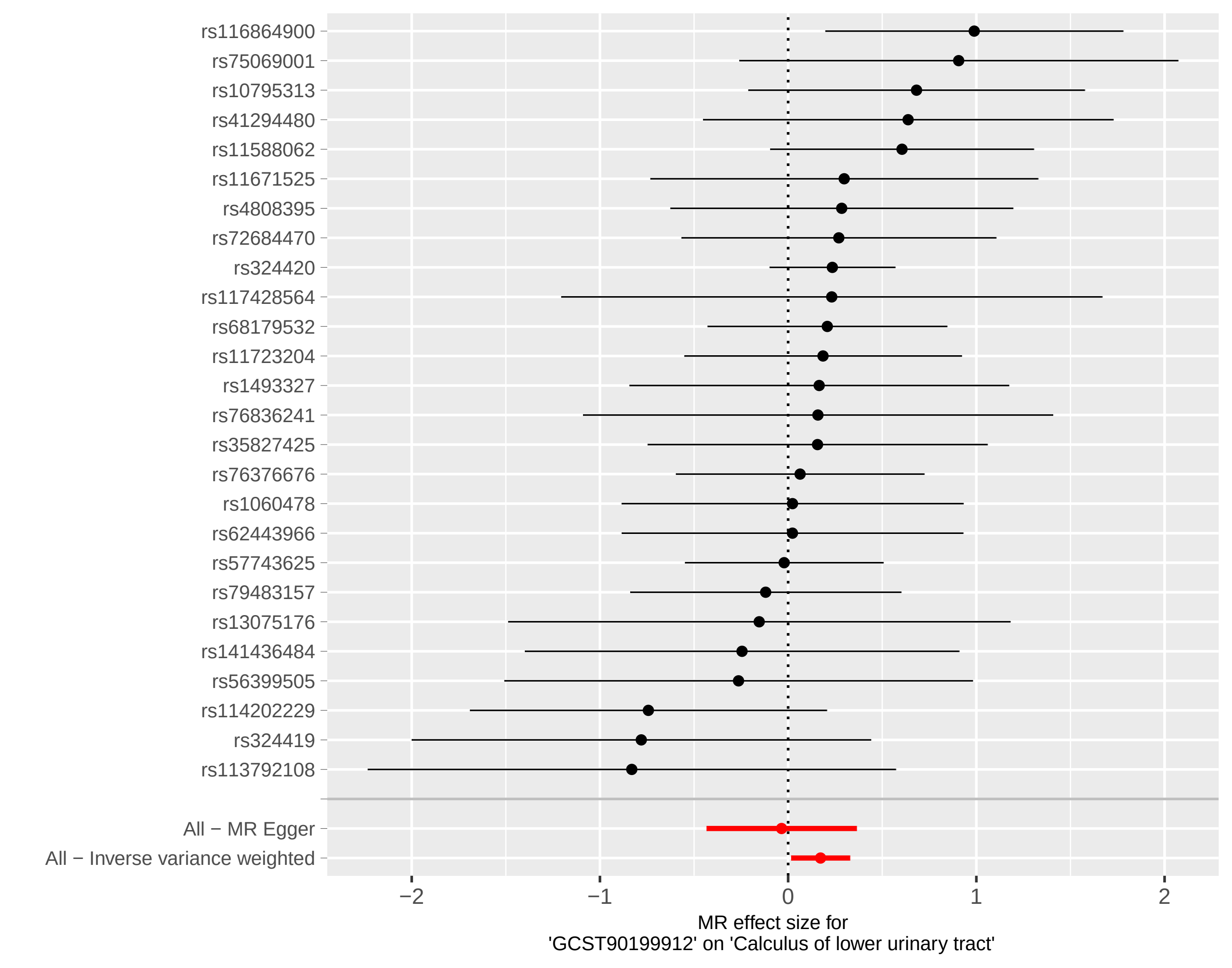
rs116864900
rs75069001
rs10795313
rs41294480
rs11588062
rs11671525
rs4808395
rs72684470
rs324420
rs117428564
rs68179532
rs11723204
rs1493327
rs76836241
rs35827425
rs76376676
rs1060478
rs62443966
rs57743625
rs79483157
rs13075176
rs141436484
rs56399505
rs114202229
rs324419
rs113792108
All − MR Egger
All − Inverse variance weighted
−2
−1
0
1
2
MR effect size for
'GCST90199912' on 'Calculus of lower urinary tract'

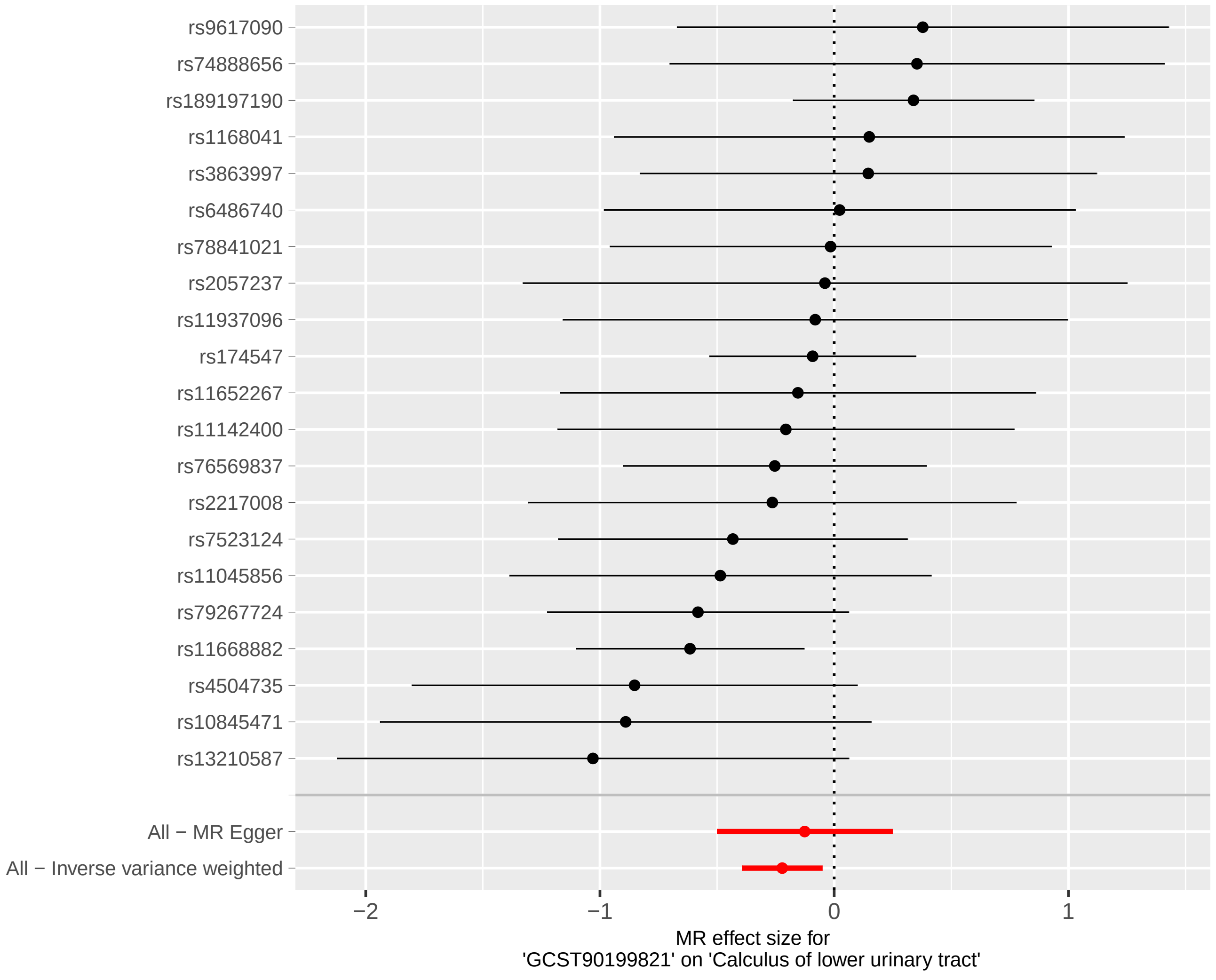
rs9617090
rs74888656
rs189197190
rs1168041
rs3863997
rs6486740
rs78841021
rs2057237
rs11937096
rs174547
rs11652267
rs11142400
rs76569837
rs2217008
rs7523124
rs11045856
rs79267724
rs11668882
rs4504735
rs10845471
rs13210587
All − MR Egger
All − Inverse variance weighted
−2
−1
0
1
MR effect size for
'GCST90199821' on 'Calculus of lower urinary tract'

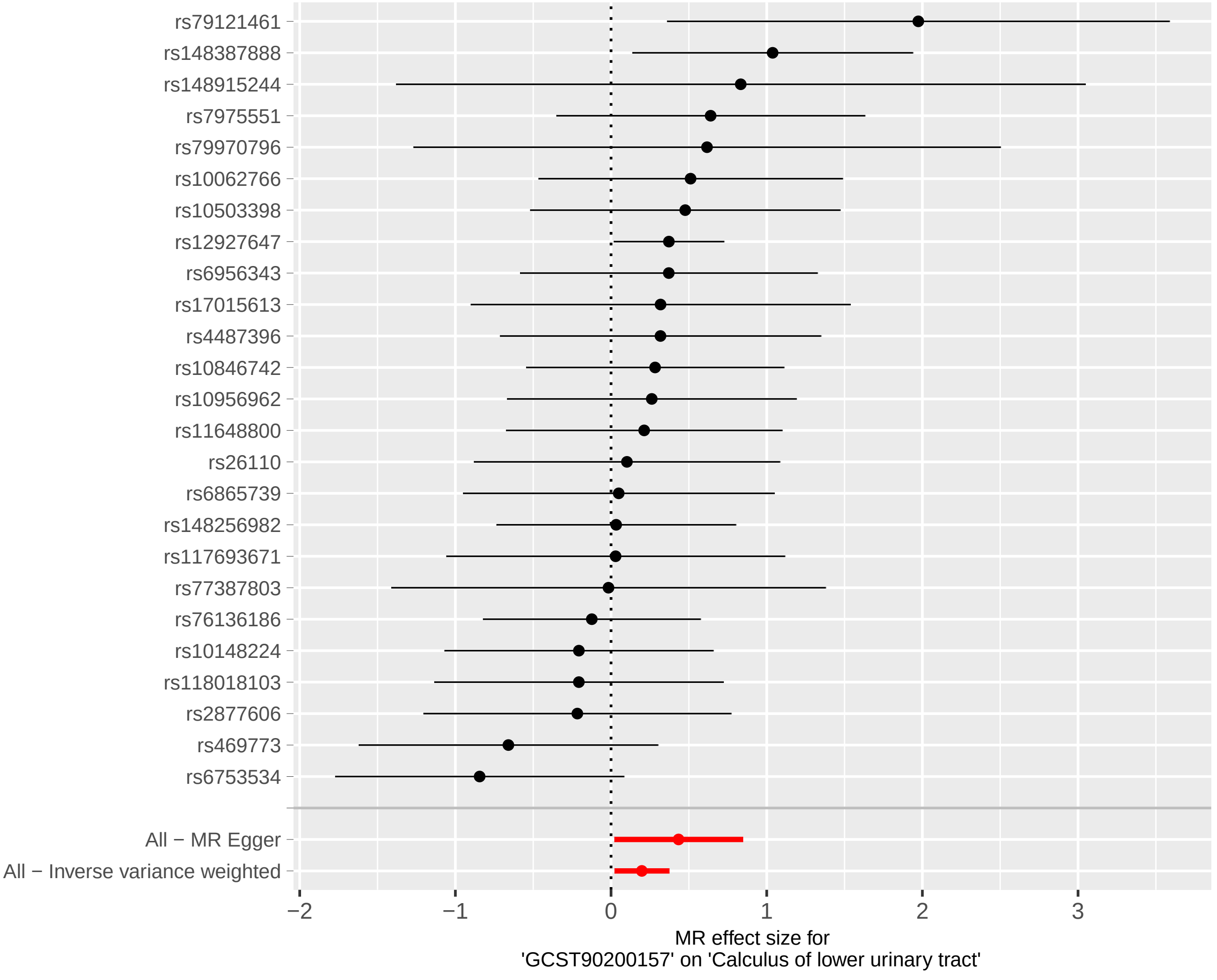
rs79121461
rs148387888
rs148915244
rs7975551
rs79970796
rs10062766
rs10503398
rs12927647
rs6956343
rs17015613
rs4487396
rs10846742
rs10956962
rs11648800
rs26110
rs6865739
rs148256982
rs117693671
rs77387803
rs76136186
rs10148224
rs118018103
rs2877606
rs469773
rs6753534
All − MR Egger
All − Inverse variance weighted
−2
−1
0
1
2
3
MR effect size for
'GCST90200157' on 'Calculus of lower urinary tract'

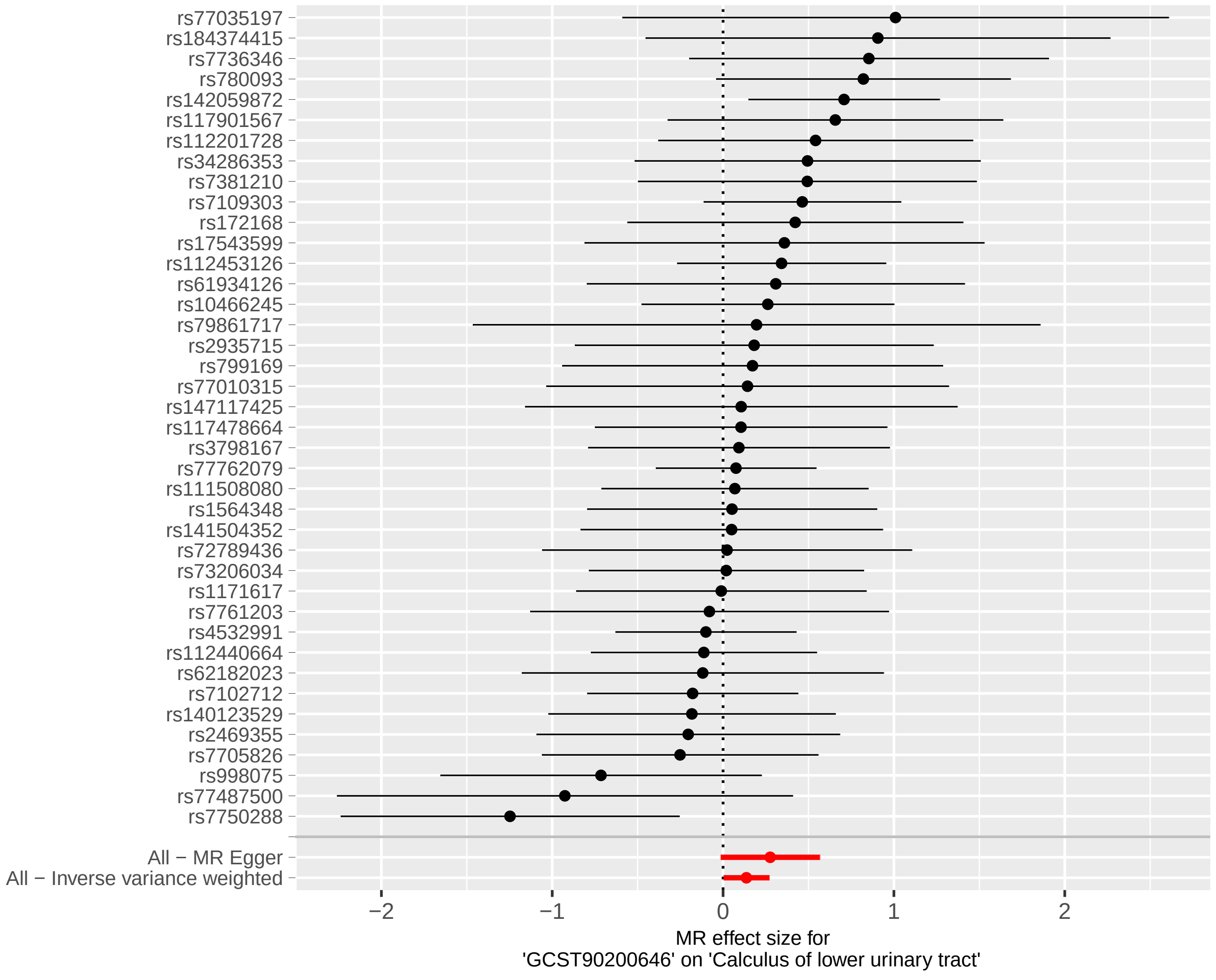
rs77035197
rs184374415
rs7736346
rs780093
rs142059872
rs117901567
rs112201728
rs34286353
rs7381210
rs7109303
rs172168
rs17543599
rs112453126
rs61934126
rs10466245
rs79861717
rs2935715
rs799169
rs77010315
rs147117425
rs117478664
rs3798167
rs77762079
rs111508080
rs1564348
rs141504352
rs72789436
rs73206034
rs1171617
rs7761203
rs4532991
rs112440664
rs62182023
rs7102712
rs140123529
rs2469355
rs7705826
rs998075
rs77487500
rs7750288
All − MR Egger
All − Inverse variance weighted
−2
−1
0
1
2
MR effect size for
'GCST90200646' on 'Calculus of lower urinary tract'

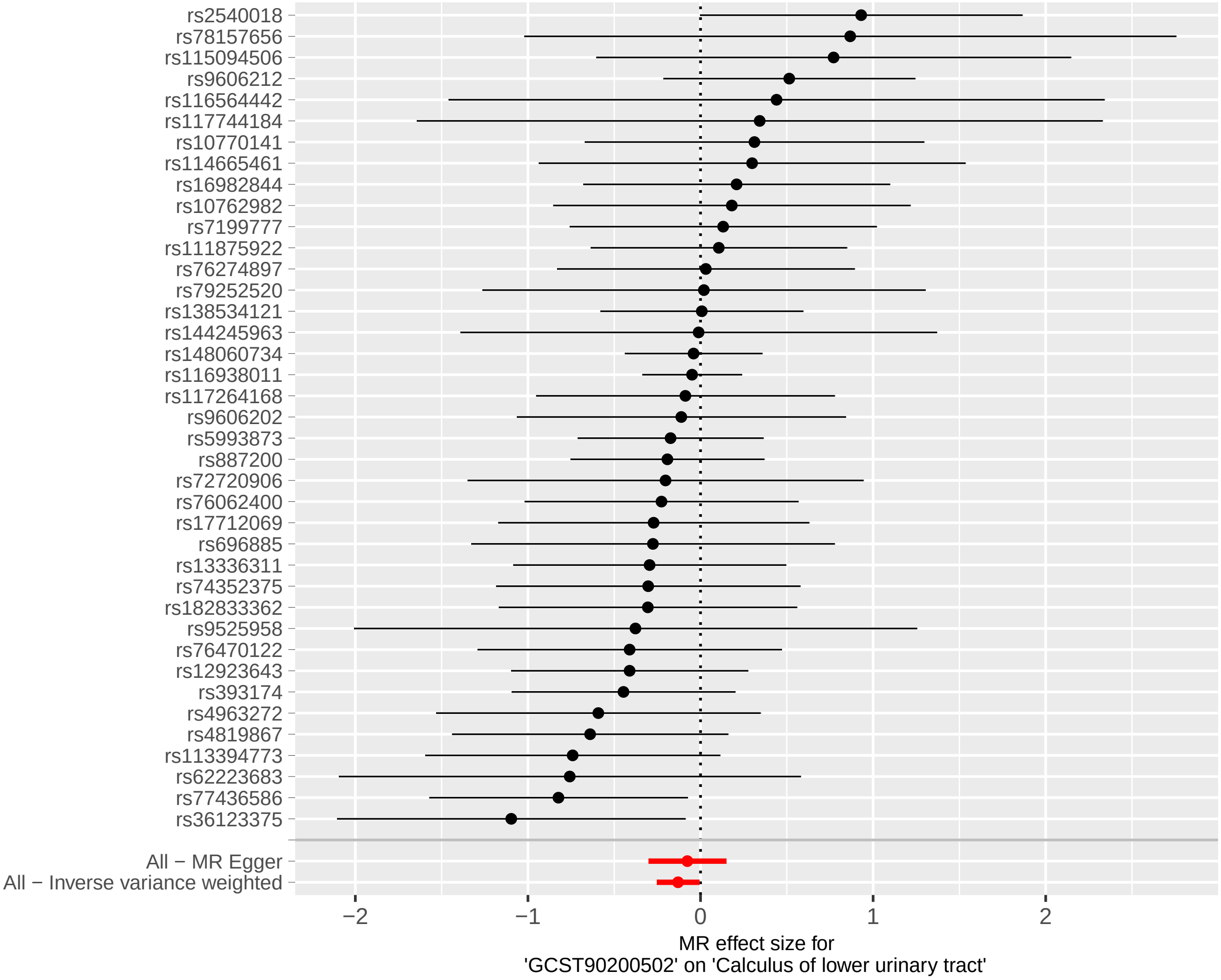
rs2540018
rs78157656
rs115094506
rs9606212
rs116564442
rs117744184
rs10770141
rs114665461
rs16982844
rs10762982
rs7199777
rs111875922
rs76274897
rs79252520
rs138534121
rs144245963
rs148060734
rs116938011
rs117264168
rs9606202
rs5993873
rs887200
rs72720906
rs76062400
rs17712069
rs696885
rs13336311
rs74352375
rs182833362
rs9525958
rs76470122
rs12923643
rs393174
rs4963272
rs4819867
rs113394773
rs62223683
rs77436586
rs36123375
All − MR Egger
All − Inverse variance weighted
−2
−1
0
1
2
MR effect size for
'GCST90200502' on 'Calculus of lower urinary tract'

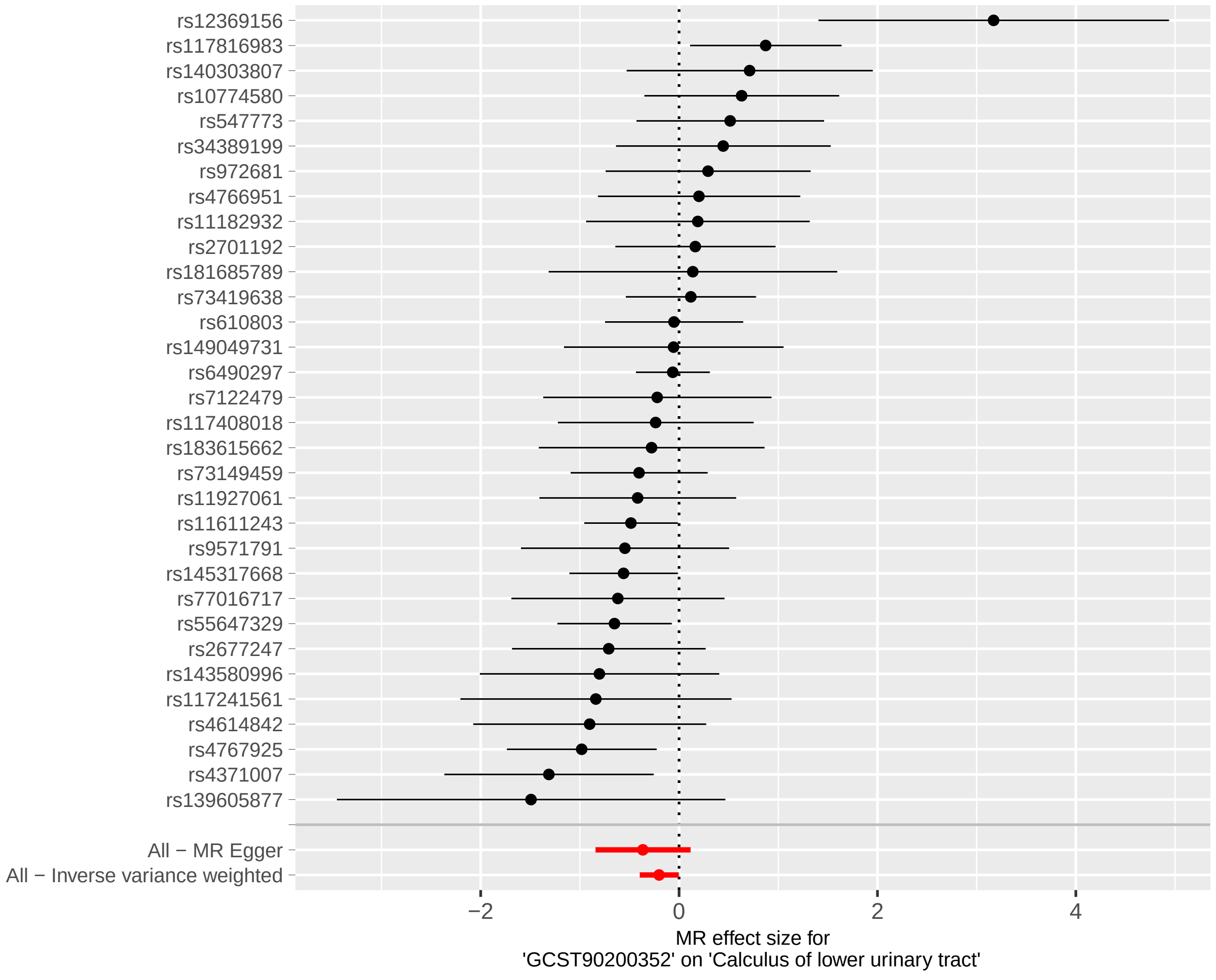
rs12369156
rs117816983
rs140303807
rs10774580
rs547773
rs34389199
rs972681
rs4766951
rs11182932
rs2701192
rs181685789
rs73419638
rs610803
rs149049731
rs6490297
rs7122479
rs117408018
rs183615662
rs73149459
rs11927061
rs11611243
rs9571791
rs145317668
rs77016717
rs55647329
rs2677247
rs143580996
rs117241561
rs4614842
rs4767925
rs4371007
rs139605877
All − MR Egger
All − Inverse variance weighted
−2
0
2
4
MR effect size for
'GCST90200352' on 'Calculus of lower urinary tract'

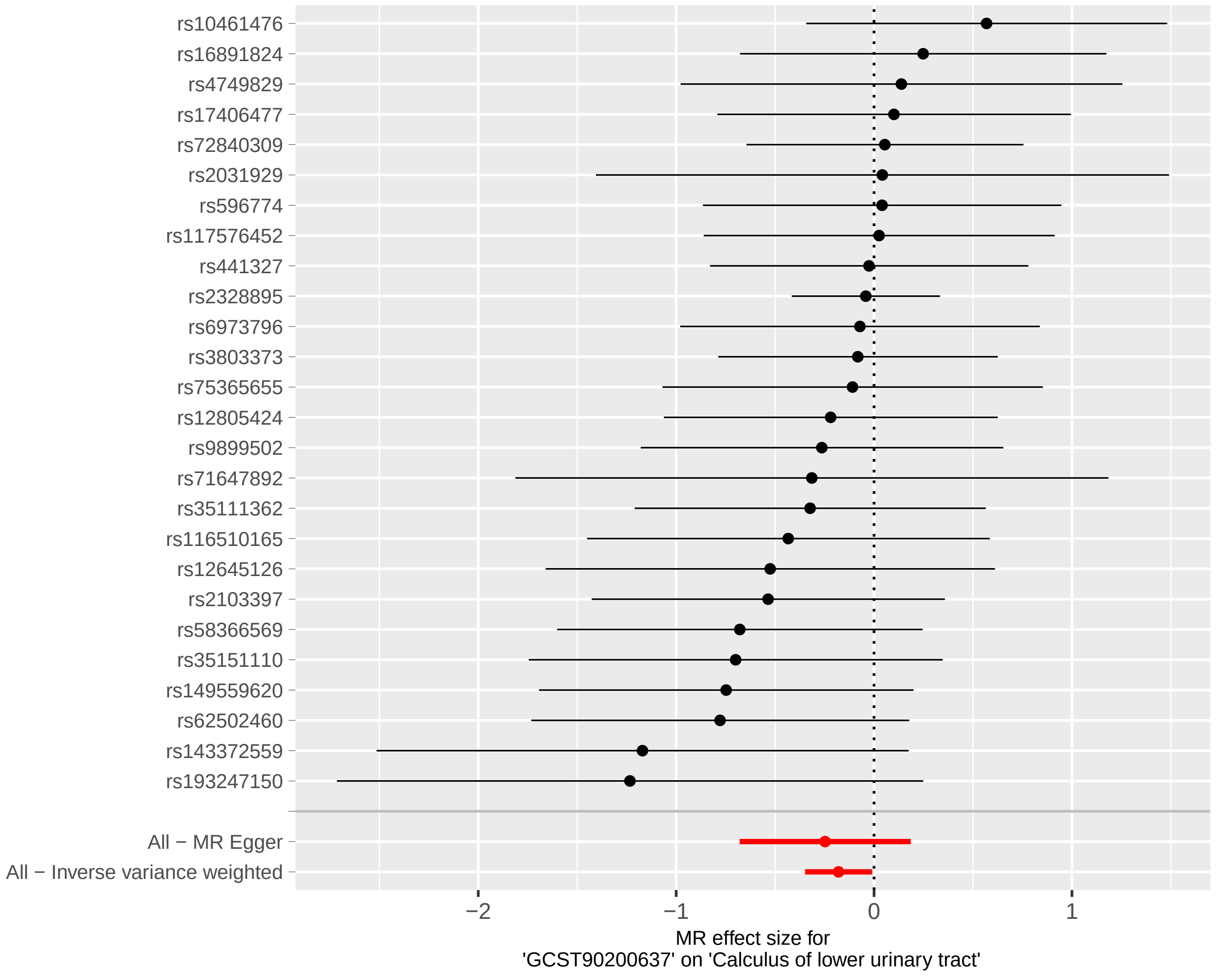
rs10461476
rs16891824
rs4749829
rs17406477
rs72840309
rs2031929
rs596774
rs117576452
rs441327
rs2328895
rs6973796
rs3803373
rs75365655
rs12805424
rs9899502
rs71647892
rs35111362
rs116510165
rs12645126
rs2103397
rs58366569
rs35151110
rs149559620
rs62502460
rs143372559
rs193247150
All − MR Egger
All − Inverse variance weighted
−2
−1
0
1
MR effect size for
'GCST90200637' on 'Calculus of lower urinary tract'

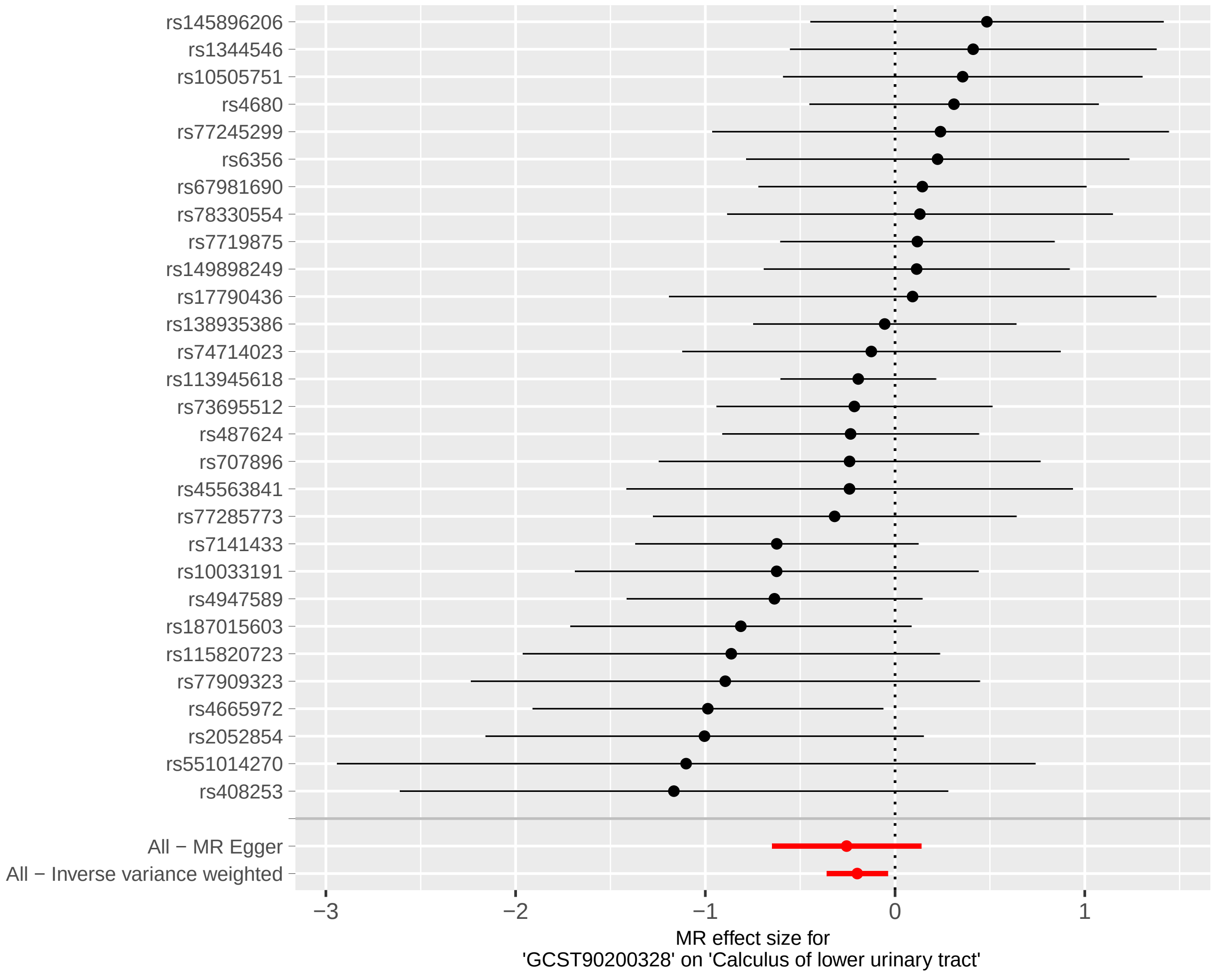
rs145896206
rs1344546
rs10505751
rs4680
rs77245299
rs6356
rs67981690
rs78330554
rs7719875
rs149898249
rs17790436
rs138935386
rs74714023
rs113945618
rs73695512
rs487624
rs707896
rs45563841
rs77285773
rs7141433
rs10033191
rs4947589
rs187015603
rs115820723
rs77909323
rs4665972
rs2052854
rs551014270
rs408253
All − MR Egger
All − Inverse variance weighted
−3
−2
−1
0
1
MR effect size for
'GCST90200328' on 'Calculus of lower urinary tract'

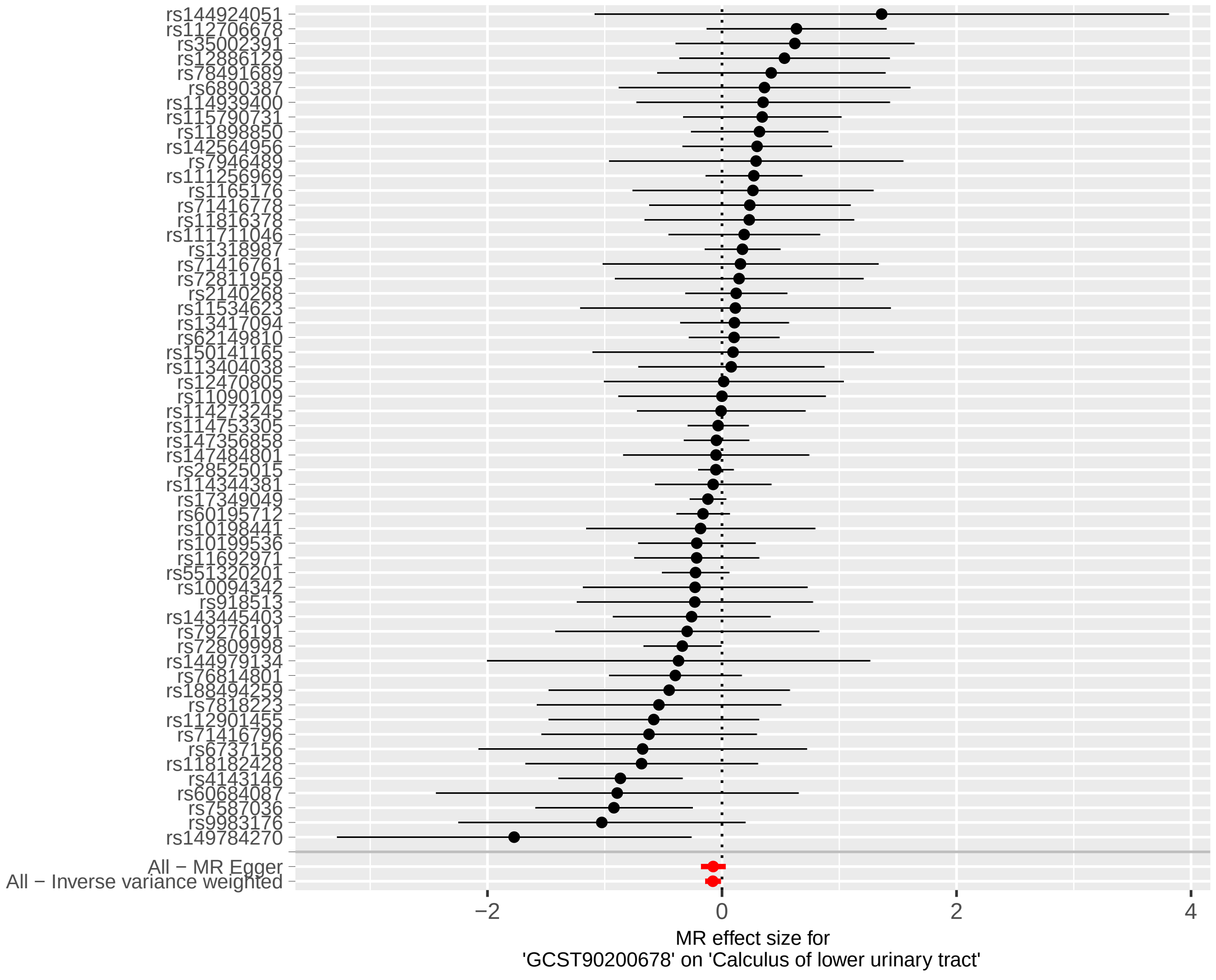
rs144924051
rs112706678
rs35002391
rs12886129
rs78491689
rs6890387
rs114939400
rs115790731
rs11898850
rs142564956
rs7946489
rs111256969
rs1165176
rs71416778
rs11816378
rs111711046
rs1318987
rs71416761
rs72811959
rs2140268
rs11534623
rs13417094
rs62149810
rs150141165
rs113404038
rs12470805
rs11090109
rs114273245
rs114753305
rs147356858
rs147484801
rs28525015
rs114344381
rs17349049
rs60195712
rs10198441
rs10199536
rs11692971
rs551320201
rs10094342
rs918513
rs143445403
rs79276191
rs72809998
rs144979134
rs76814801
rs188494259
rs7818223
rs112901455
rs71416796
rs6737156
rs118182428
rs4143146
rs60684087
rs7587036
rs9983176
rs149784270
All − MR Egger
All − Inverse variance weighted
−2
0
2
4
MR effect size for
'GCST90200678' on 'Calculus of lower urinary tract'

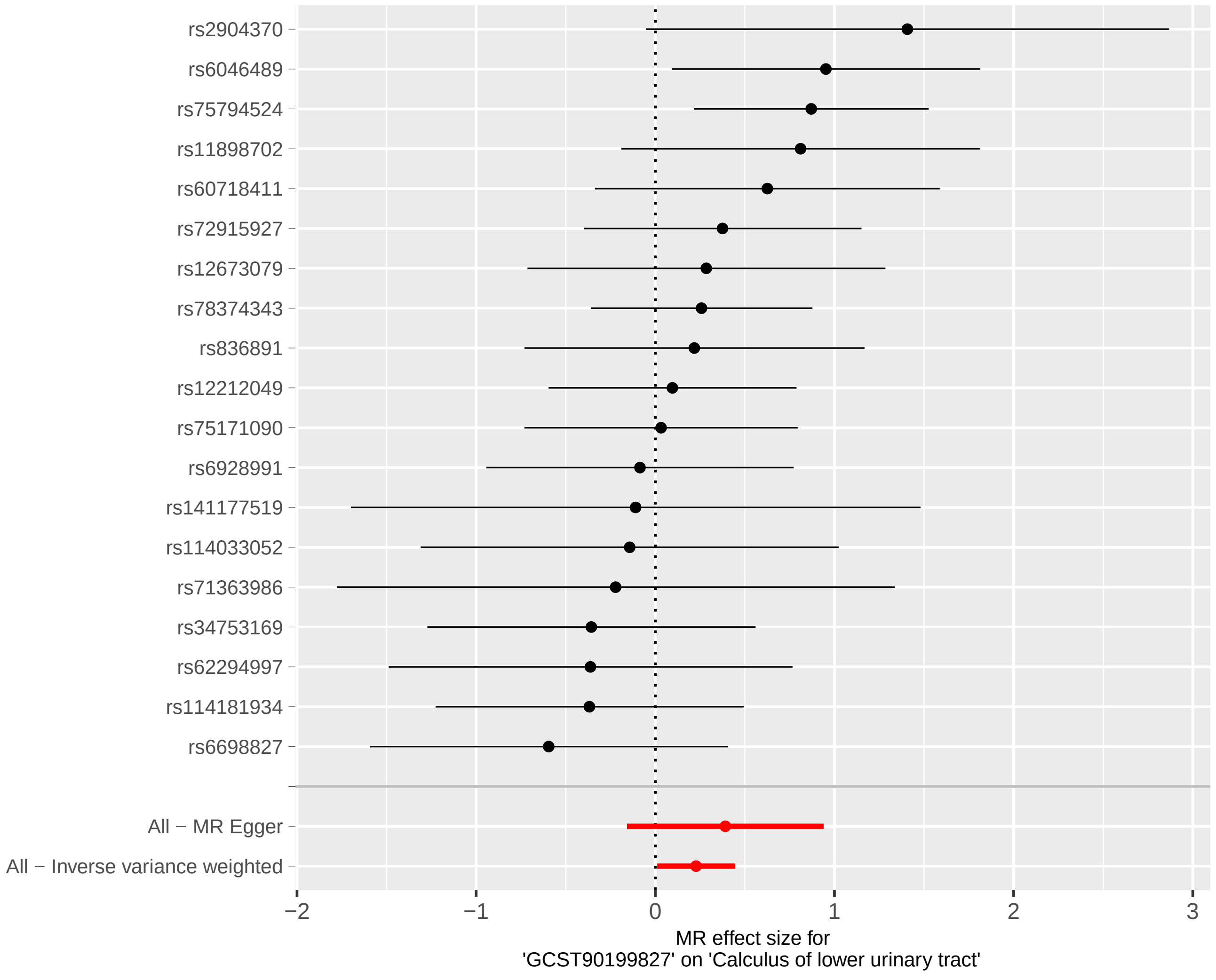
rs2904370
rs6046489
rs75794524
rs11898702
rs60718411
rs72915927
rs12673079
rs78374343
rs836891
rs12212049
rs75171090
rs6928991
rs141177519
rs114033052
rs71363986
rs34753169
rs62294997
rs114181934
rs6698827
All − MR Egger
All − Inverse variance weighted
−2
−1
0
1
2
3
MR effect size for
'GCST90199827' on 'Calculus of lower urinary tract'

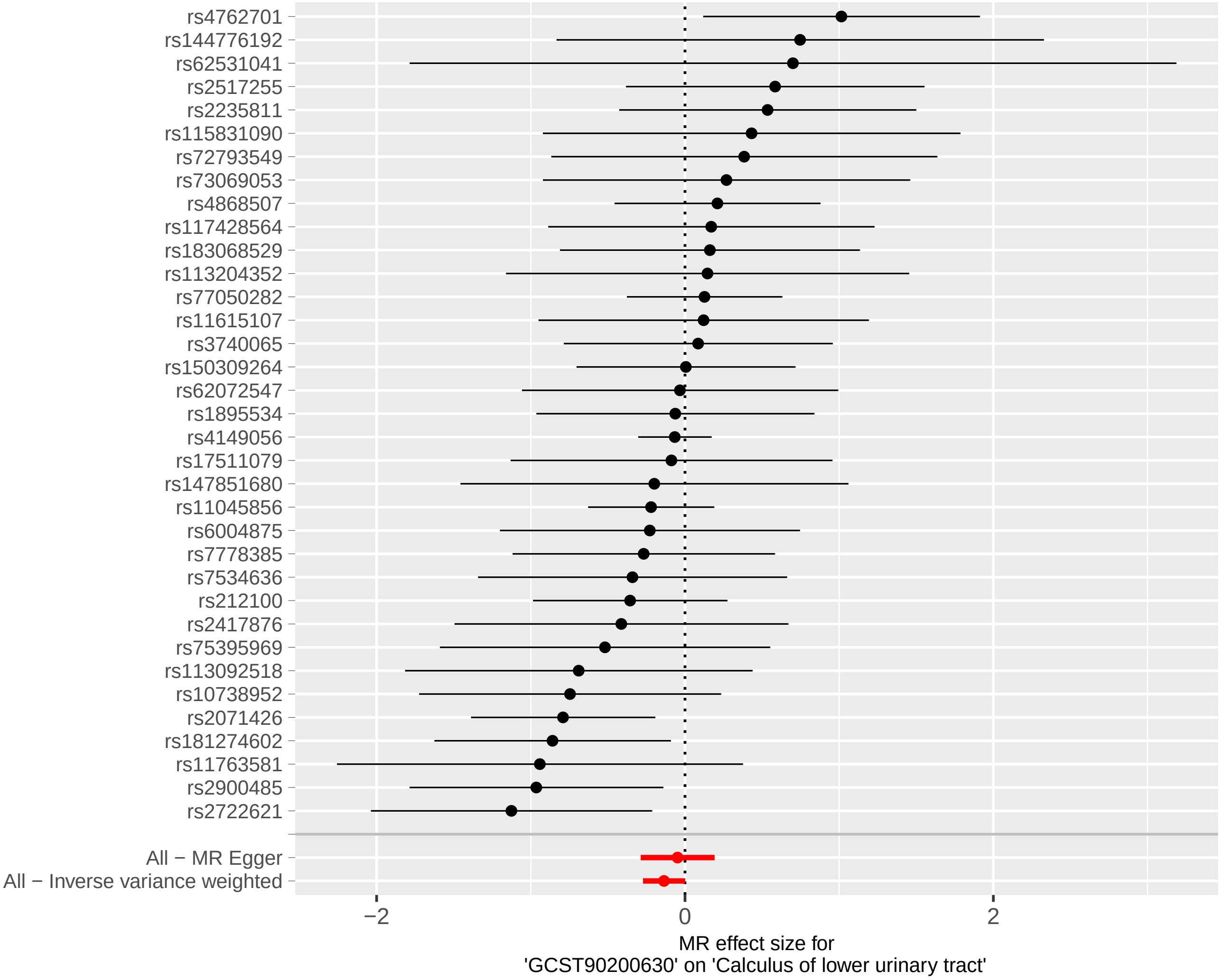
rs4762701
rs144776192
rs62531041
rs2517255
rs2235811
rs115831090
rs72793549
rs73069053
rs4868507
rs117428564
rs183068529
rs113204352
rs77050282
rs11615107
rs3740065
rs150309264
rs62072547
rs1895534
rs4149056
rs17511079
rs147851680
rs11045856
rs6004875
rs7778385
rs7534636
rs212100
rs2417876
rs75395969
rs113092518
rs10738952
rs2071426
rs181274602
rs11763581
rs2900485
rs2722621
All − MR Egger
All − Inverse variance weighted
−2
0
2
MR effect size for
'GCST90200630' on 'Calculus of lower urinary tract'

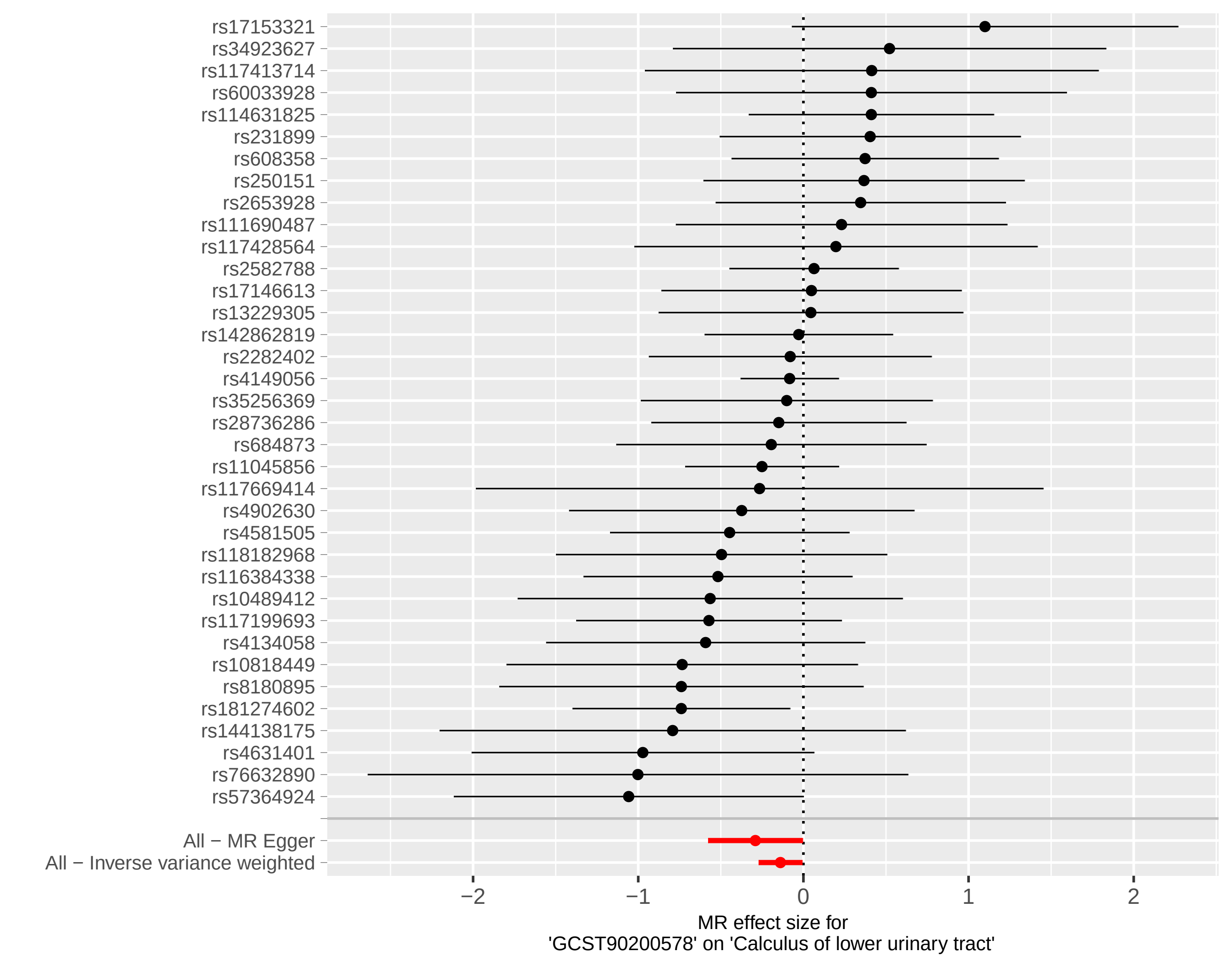

rs17153321
rs34923627
rs117413714
rs60033928
rs114631825
rs231899
rs608358
rs250151
rs2653928
rs111690487
rs117428564
rs2582788
rs17146613
rs13229305
rs142862819
rs2282402
rs4149056
rs35256369
rs28736286
rs684873
rs11045856
rs117669414
rs4902630
rs4581505
rs118182968
rs116384338
rs10489412
rs117199693
rs4134058
rs10818449
rs8180895
rs181274602
rs144138175
rs4631401
rs76632890
rs57364924
All − MR Egger
All − Inverse variance weighted
−2
−1
0
1
2
MR effect size for
'GCST90200578' on 'Calculus of lower urinary tract'

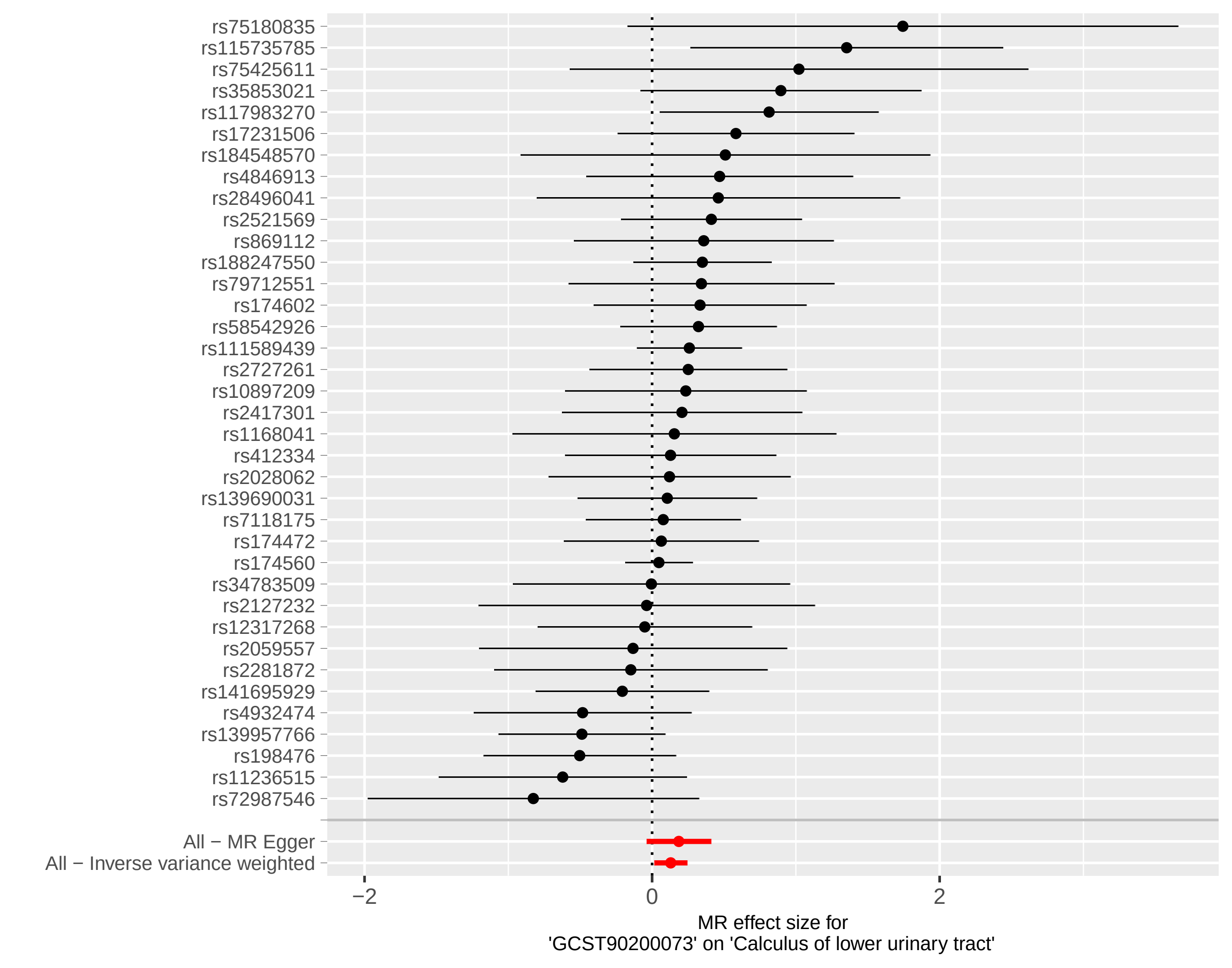
rs75180835
rs115735785
rs75425611
rs35853021
rs117983270
rs17231506
rs184548570
rs4846913
rs28496041
rs2521569
rs869112
rs188247550
rs79712551
rs174602
rs58542926
rs111589439
rs2727261
rs10897209
rs2417301
rs1168041
rs412334
rs2028062
rs139690031
rs7118175
rs174472
rs174560
rs34783509
rs2127232
rs12317268
rs2059557
rs2281872
rs141695929
rs4932474
rs139957766
rs198476
rs11236515
rs72987546
All − MR Egger
All − Inverse variance weighted
−2
0
2
MR effect size for
'GCST90200073' on 'Calculus of lower urinary tract'

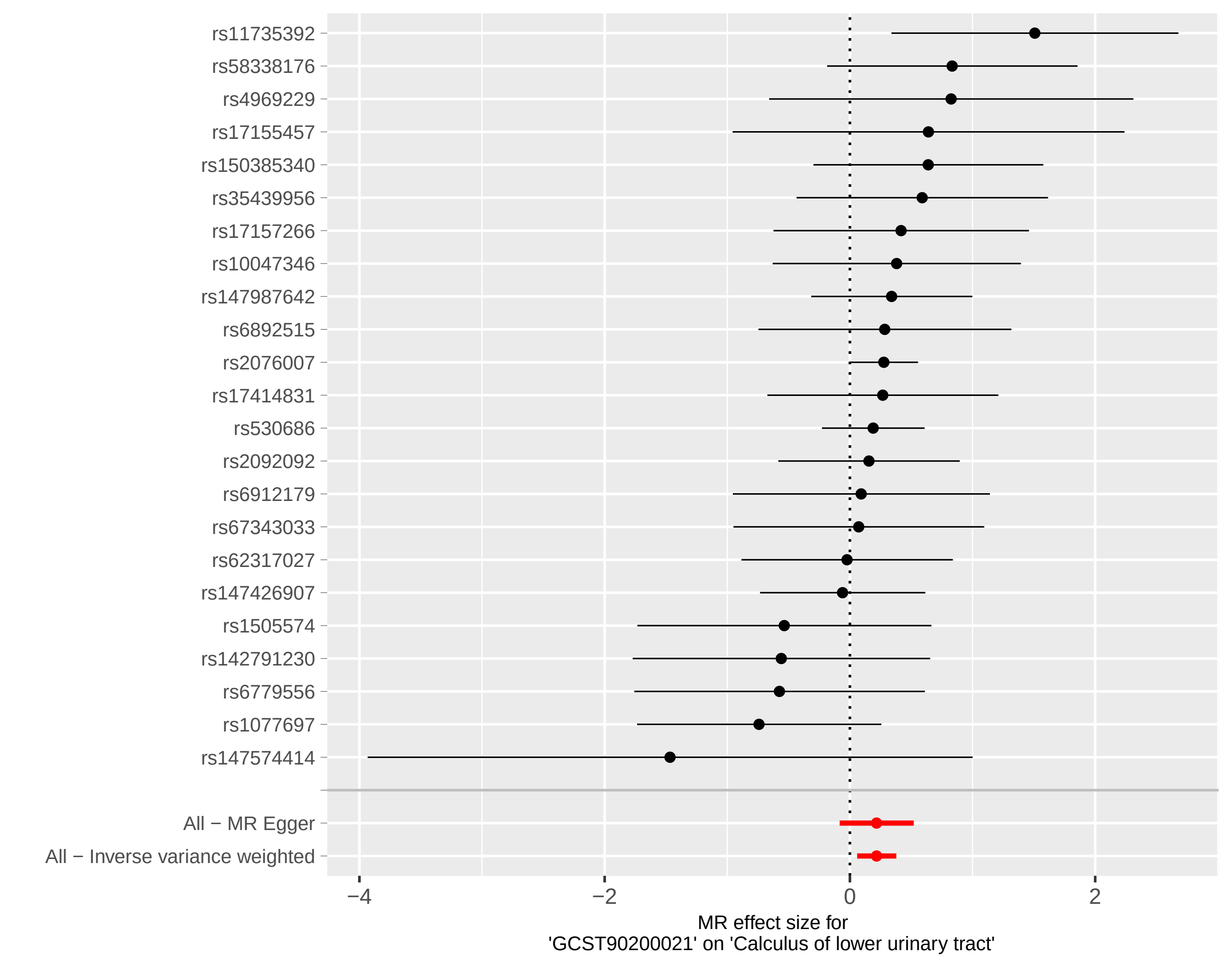
rs11735392
rs58338176
rs4969229
rs17155457
rs150385340
rs35439956
rs17157266
rs10047346
rs147987642
rs6892515
rs2076007
rs17414831
rs530686
rs2092092
rs6912179
rs67343033
rs62317027
rs147426907
rs1505574
rs142791230
rs6779556
rs1077697
rs147574414
All − MR Egger
All − Inverse variance weighted
−4
−2
0
2
MR effect size for
'GCST90200021' on 'Calculus of lower urinary tract'

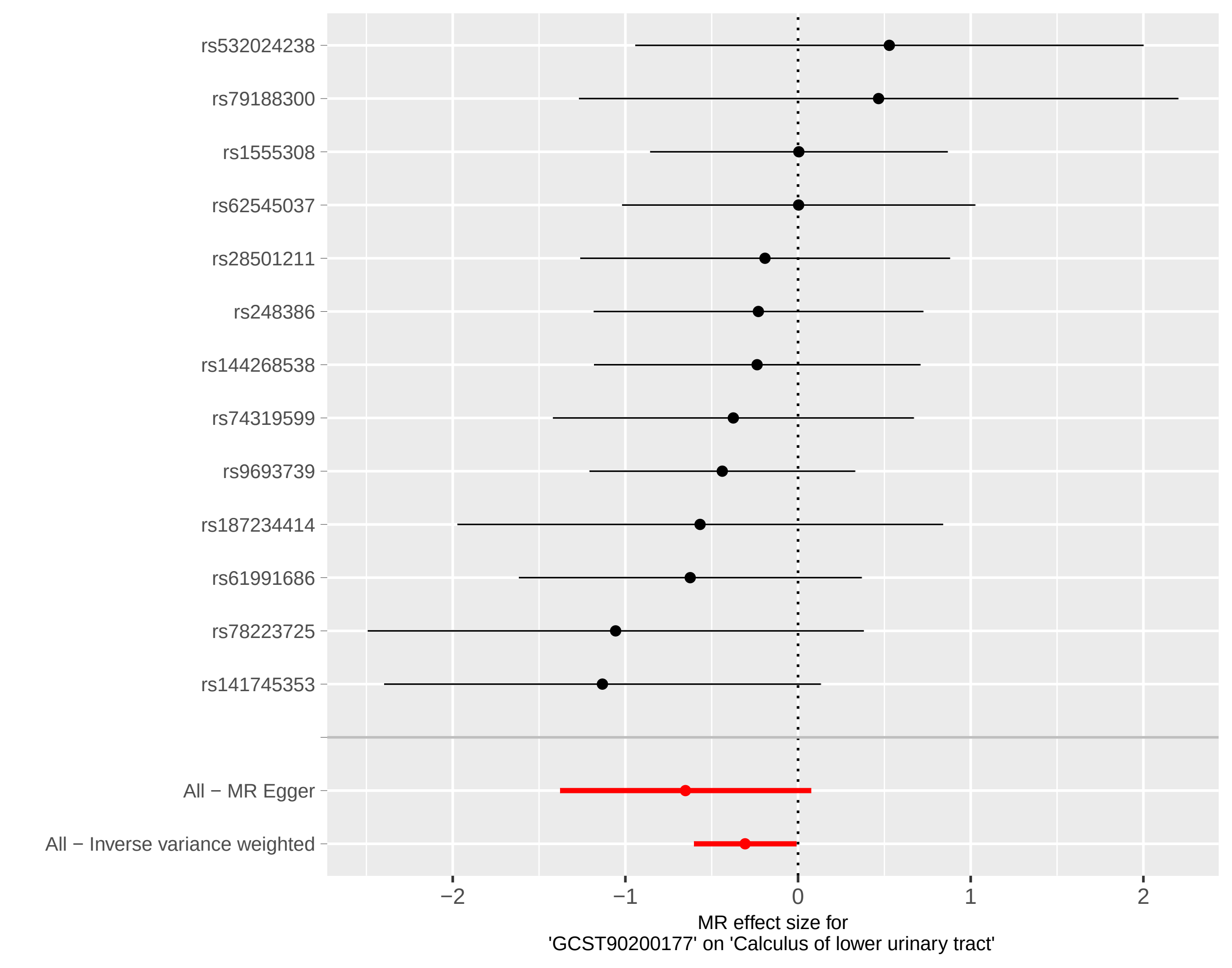
rs532024238
rs79188300
rs1555308
rs62545037
rs28501211
rs248386
rs144268538
rs74319599
rs9693739
rs187234414
rs61991686
rs78223725
rs141745353
All − MR Egger
All − Inverse variance weighted
−2
−1
0
1
2
MR effect size for
'GCST90200177' on 'Calculus of lower urinary tract'

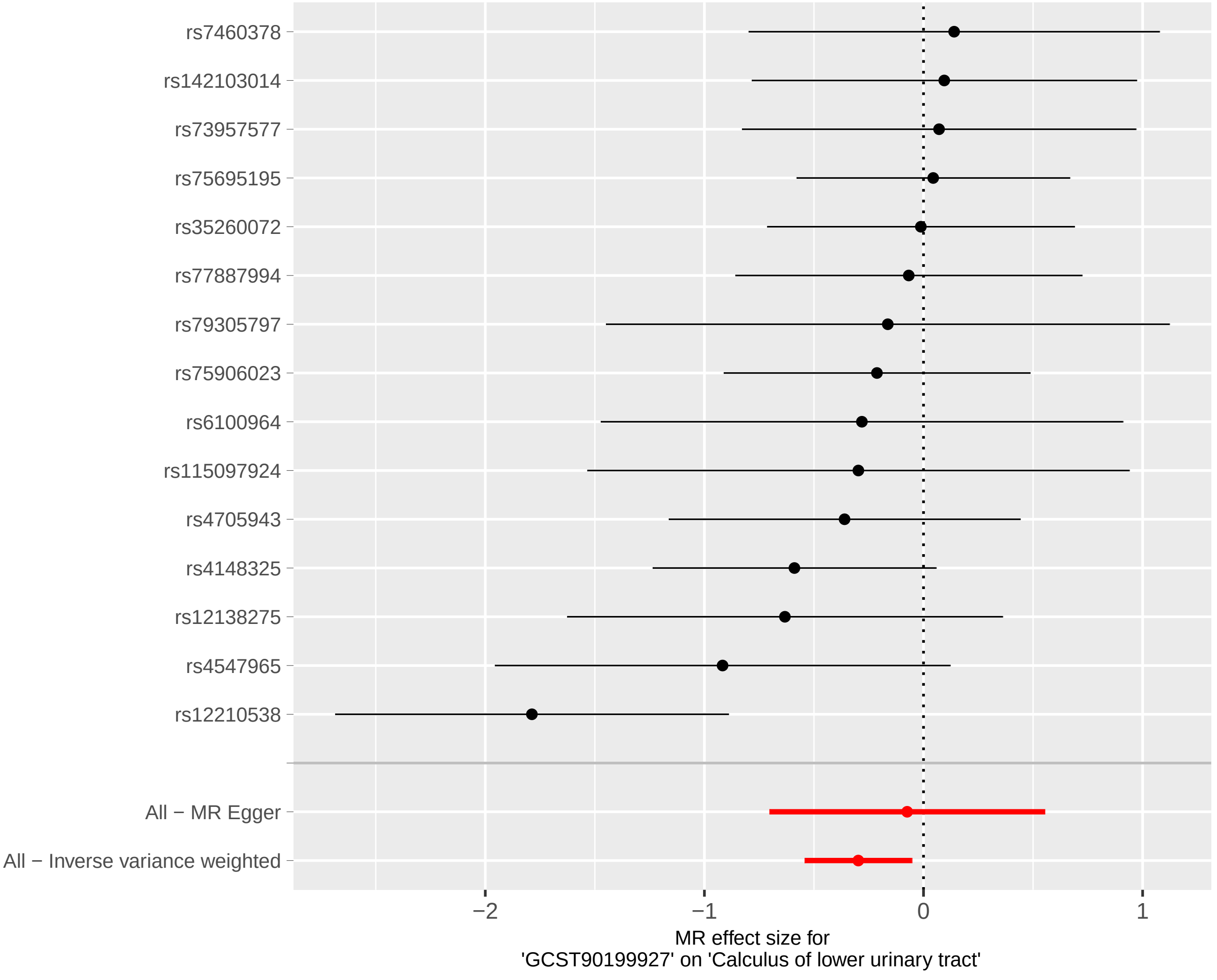
rs7460378
rs142103014
rs73957577
rs75695195
rs35260072
rs77887994
rs79305797
rs75906023
rs6100964
rs115097924
rs4705943
rs4148325
rs12138275
rs4547965
rs12210538
All − MR Egger
All − Inverse variance weighted
−2
−1
0
1
MR effect size for
'GCST90199927' on 'Calculus of lower urinary tract'

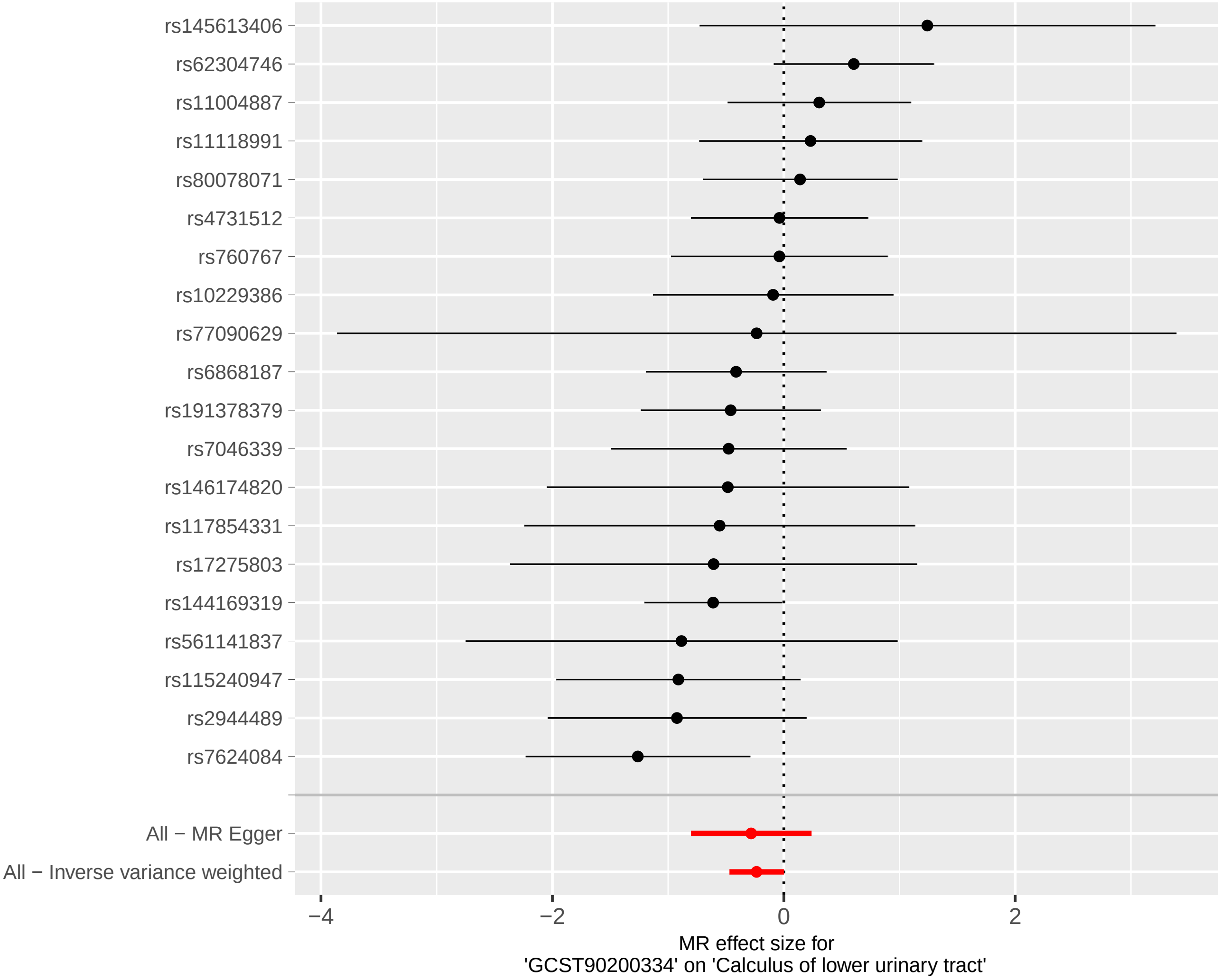
rs145613406
rs62304746
rs11004887
rs11118991
rs80078071
rs4731512
rs760767
rs10229386
rs77090629
rs6868187
rs191378379
rs7046339
rs146174820
rs117854331
rs17275803
rs144169319
rs561141837
rs115240947
rs2944489
rs7624084
All − MR Egger
All − Inverse variance weighted
−4
−2
0
2
MR effect size for
'GCST90200334' on 'Calculus of lower urinary tract'

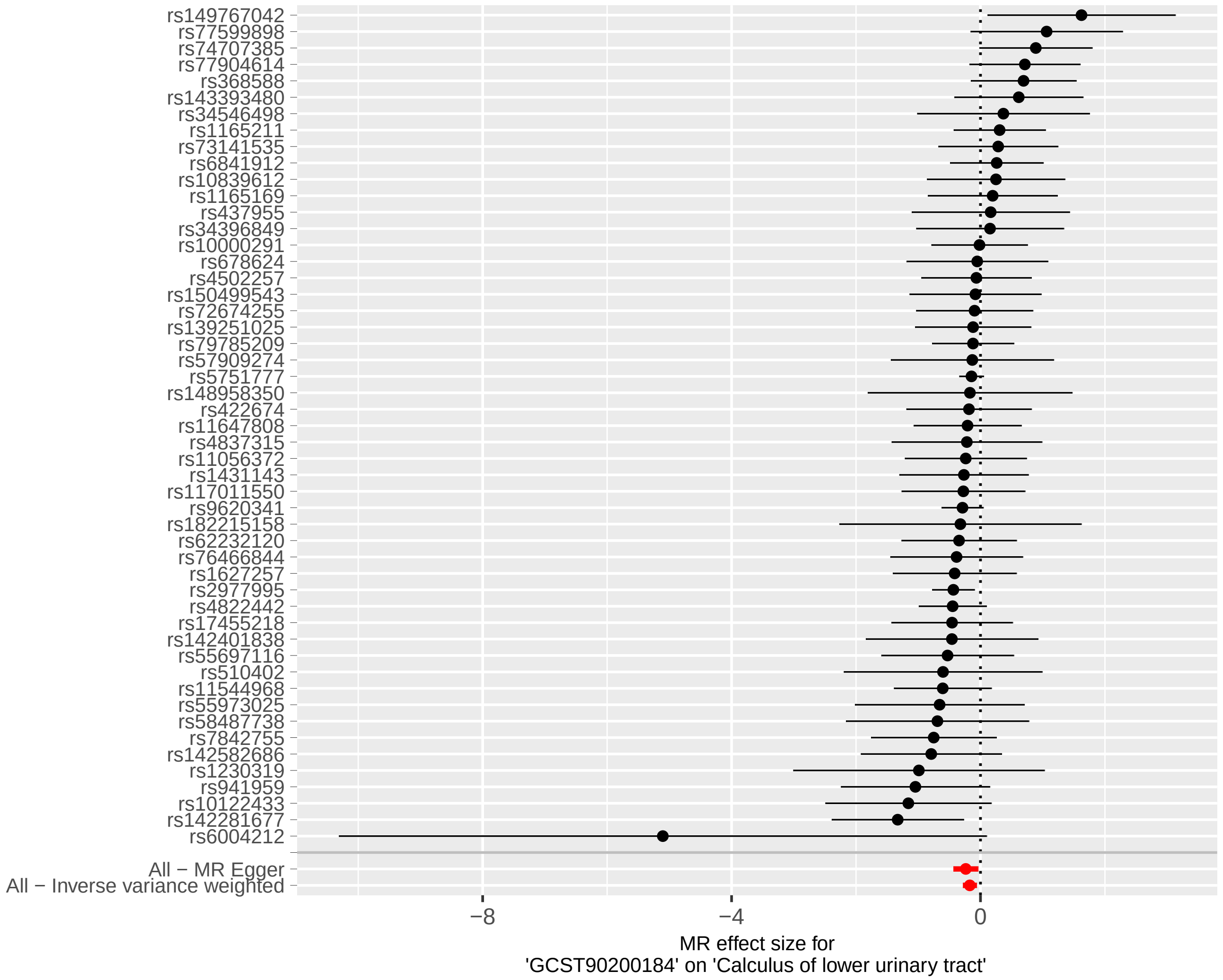

rs149767042
rs77599898
rs74707385
rs77904614
rs368588
rs143393480
rs34546498
rs1165211
rs73141535
rs6841912
rs10839612
rs1165169
rs437955
rs34396849
rs10000291
rs678624
rs4502257
rs150499543
rs72674255
rs139251025
rs79785209
rs57909274
rs5751777
rs148958350
rs422674
rs11647808
rs4837315
rs11056372
rs1431143
rs117011550
rs9620341
rs182215158
rs62232120
rs76466844
rs1627257
rs2977995
rs4822442
rs17455218
rs142401838
rs55697116
rs510402
rs11544968
rs55973025
rs58487738
rs7842755
rs142582686
rs1230319
rs941959
rs10122433
rs142281677
rs6004212
All − MR Egger
All − Inverse variance weighted
−8
−4
0
MR effect size for
'GCST90200184' on 'Calculus of lower urinary tract'

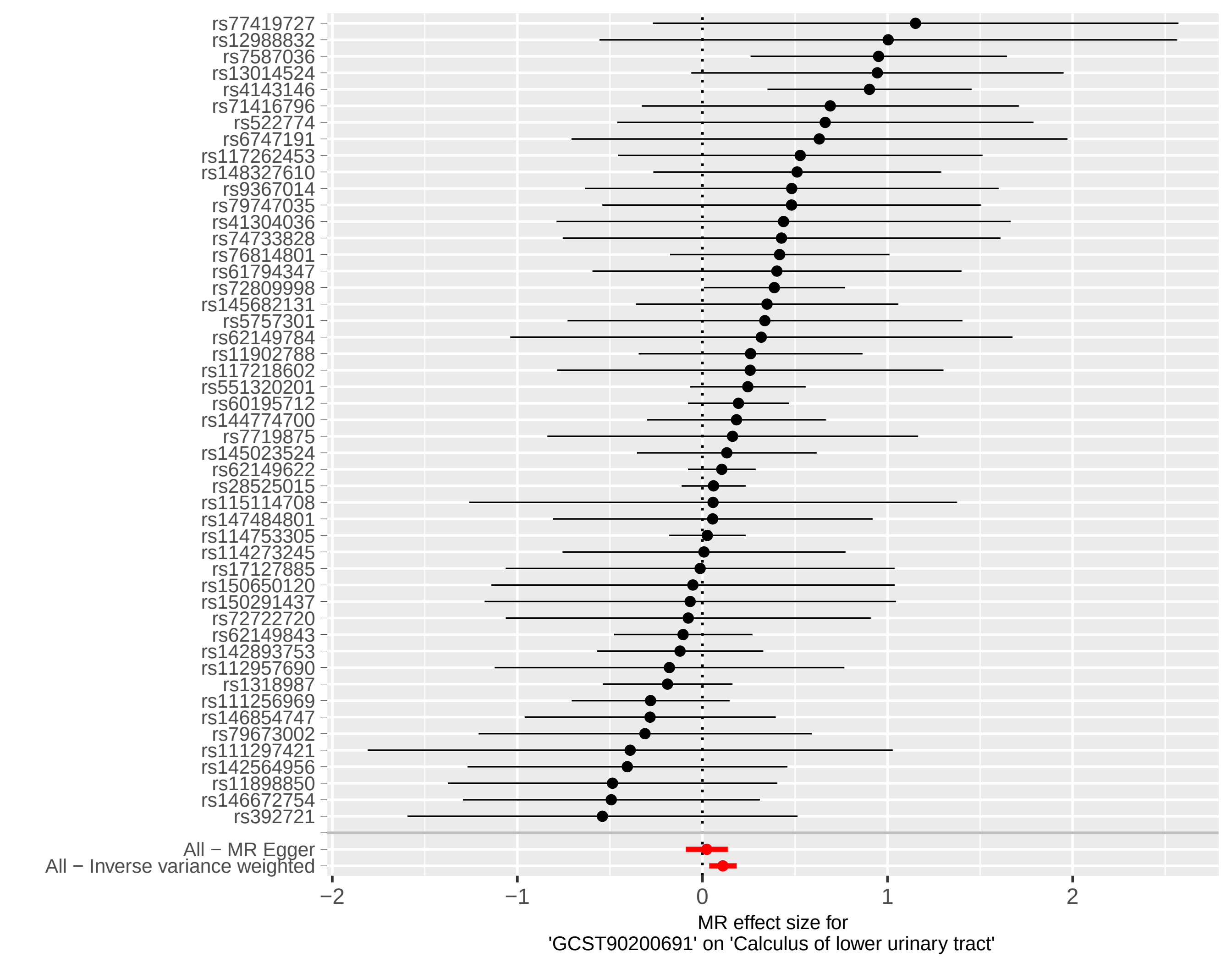
rs77419727
rs12988832
rs7587036
rs13014524
rs4143146
rs71416796
rs522774
rs6747191
rs117262453
rs148327610
rs9367014
rs79747035
rs41304036
rs74733828
rs76814801
rs61794347
rs72809998
rs145682131
rs5757301
rs62149784
rs11902788
rs117218602
rs551320201
rs60195712
rs144774700
rs7719875
rs145023524
rs62149622
rs28525015
rs115114708
rs147484801
rs114753305
rs114273245
rs17127885
rs150650120
rs150291437
rs72722720
rs62149843
rs142893753
rs112957690
rs1318987
rs111256969
rs146854747
rs79673002
rs111297421
rs142564956
rs11898850
rs146672754
rs392721
All − MR Egger
All − Inverse variance weighted
−2
−1
0
1
2
MR effect size for
'GCST90200691' on 'Calculus of lower urinary tract'

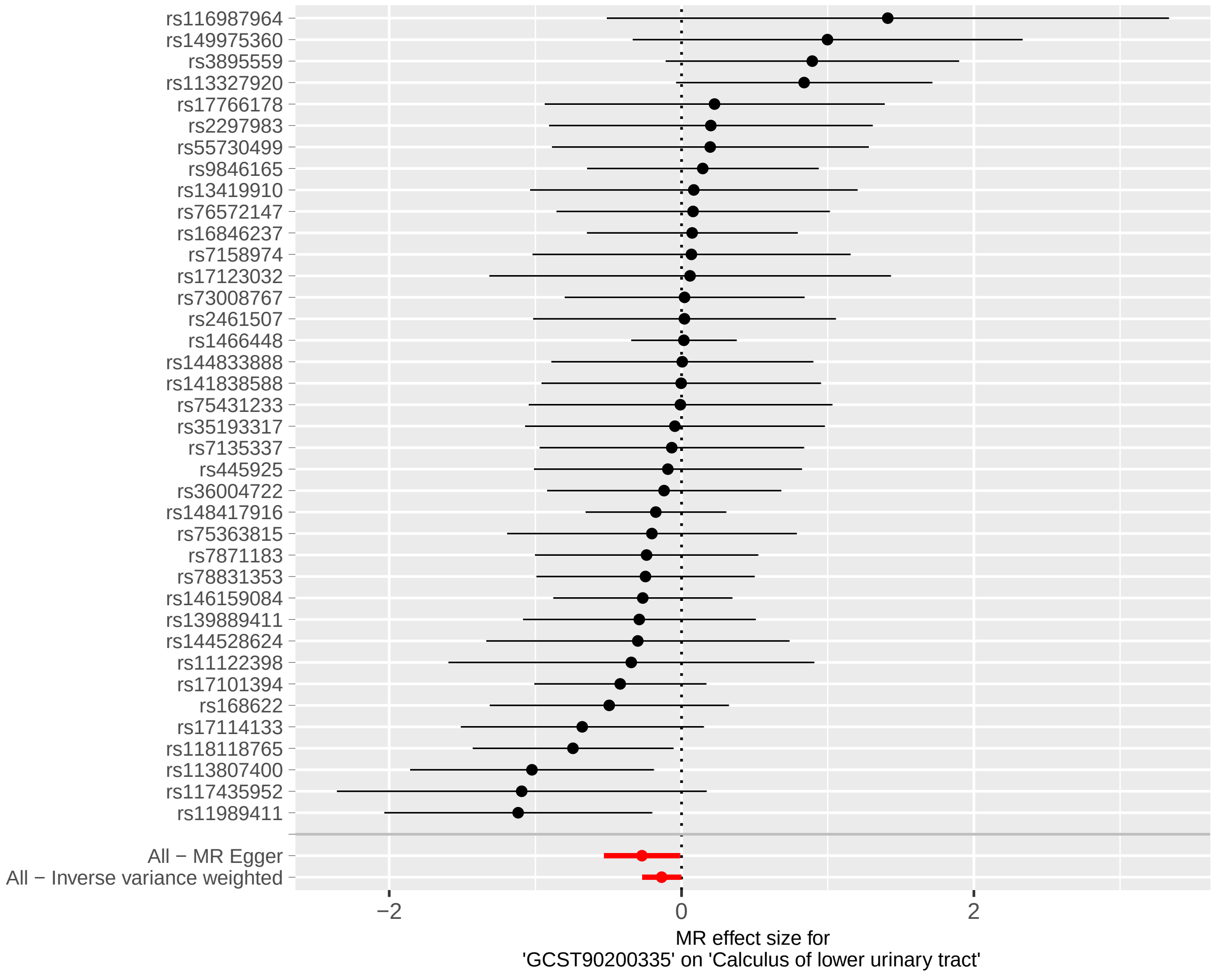

rs116987964
rs149975360
rs3895559
rs113327920
rs17766178
rs2297983
rs55730499
rs9846165
rs13419910
rs76572147
rs16846237
rs7158974
rs17123032
rs73008767
rs2461507
rs1466448
rs144833888
rs141838588
rs75431233
rs35193317
rs7135337
rs445925
rs36004722
rs148417916
rs75363815
rs7871183
rs78831353
rs146159084
rs139889411
rs144528624
rs11122398
rs17101394
rs168622
rs17114133
rs118118765
rs113807400
rs117435952
rs11989411
All − MR Egger
All − Inverse variance weighted
−2
0
2
MR effect size for
'GCST90200335' on 'Calculus of lower urinary tract'

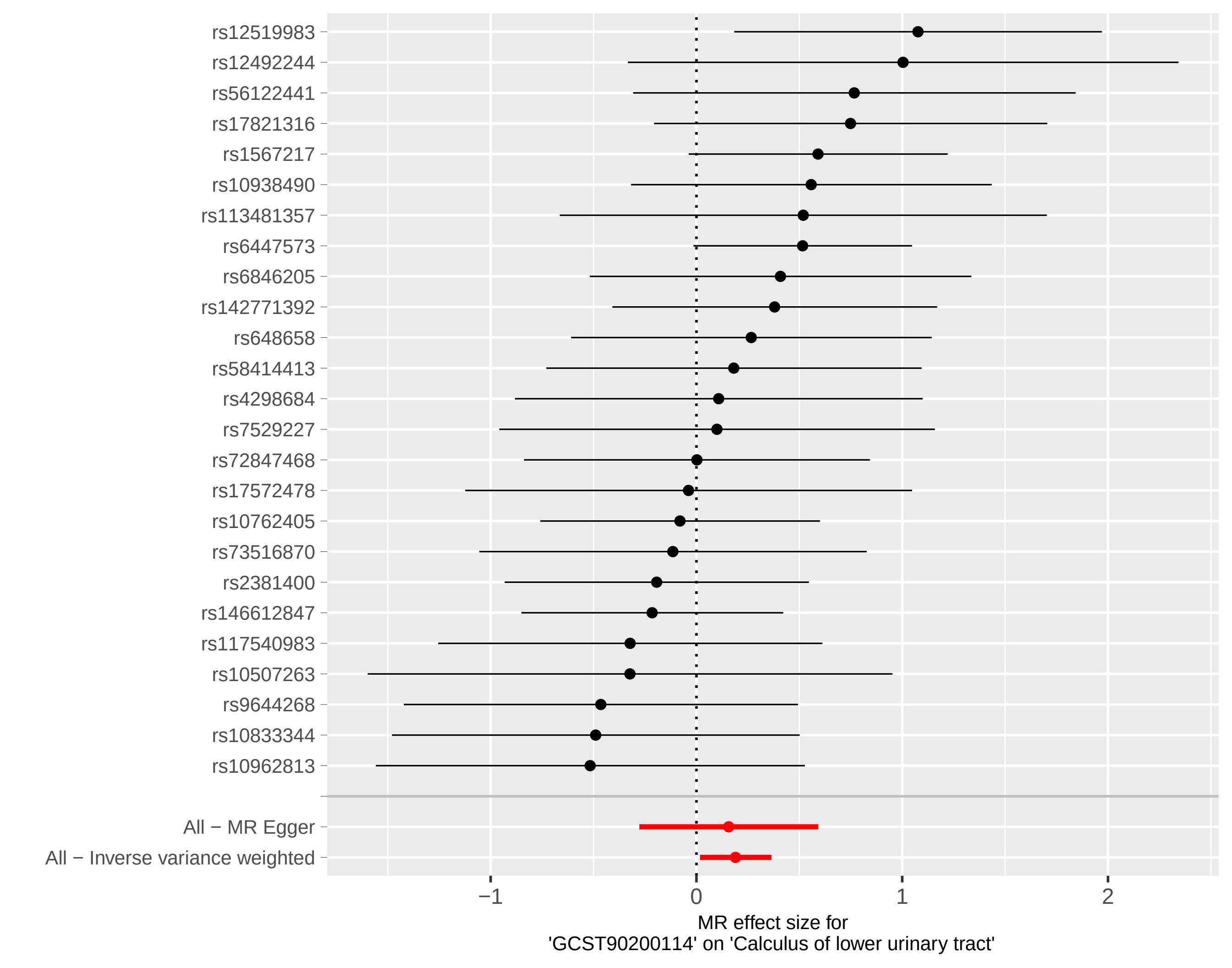

rs12519983
rs12492244
rs56122441
rs17821316
rs1567217
rs10938490
rs113481357
rs6447573
rs6846205
rs142771392
rs648658
rs58414413
rs4298684
rs7529227
rs72847468
rs17572478
rs10762405
rs73516870
rs2381400
rs146612847
rs117540983
rs10507263
rs9644268
rs10833344
rs10962813
All − MR Egger
All − Inverse variance weighted
−1
0
1
2
MR effect size for
'GCST90200114' on 'Calculus of lower urinary tract'

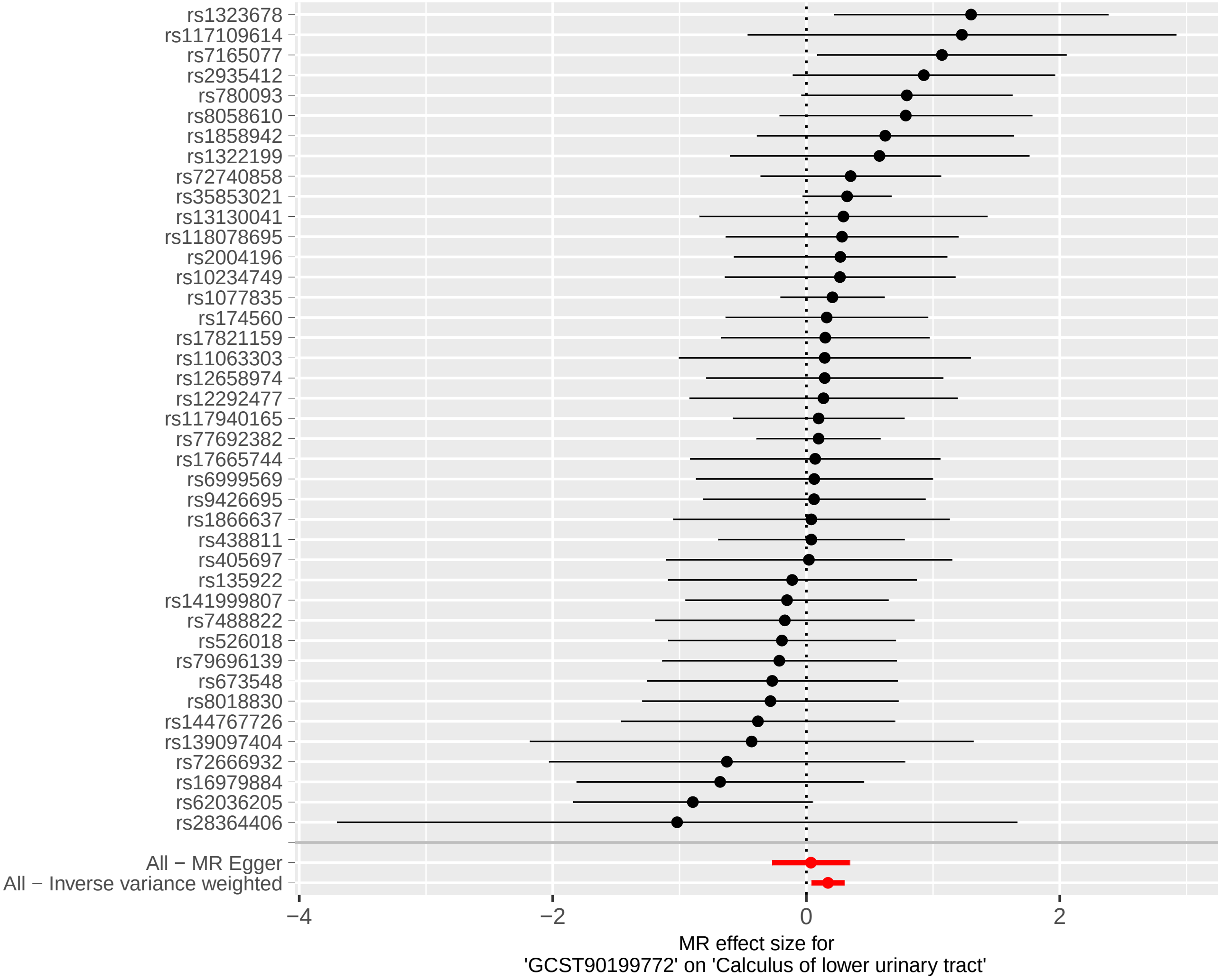

rs1323678
rs117109614
rs7165077
rs2935412
rs780093
rs8058610
rs1858942
rs1322199
rs72740858
rs35853021
rs13130041
rs118078695
rs2004196
rs10234749
rs1077835
rs174560
rs17821159
rs11063303
rs12658974
rs12292477
rs117940165
rs77692382
rs17665744
rs6999569
rs9426695
rs1866637
rs438811
rs405697
rs135922
rs141999807
rs7488822
rs526018
rs79696139
rs673548
rs8018830
rs144767726
rs139097404
rs72666932
rs16979884
rs62036205
rs28364406
All − MR Egger
All − Inverse variance weighted
−4
−2
0
2
MR effect size for
'GCST90199772' on 'Calculus of lower urinary tract'

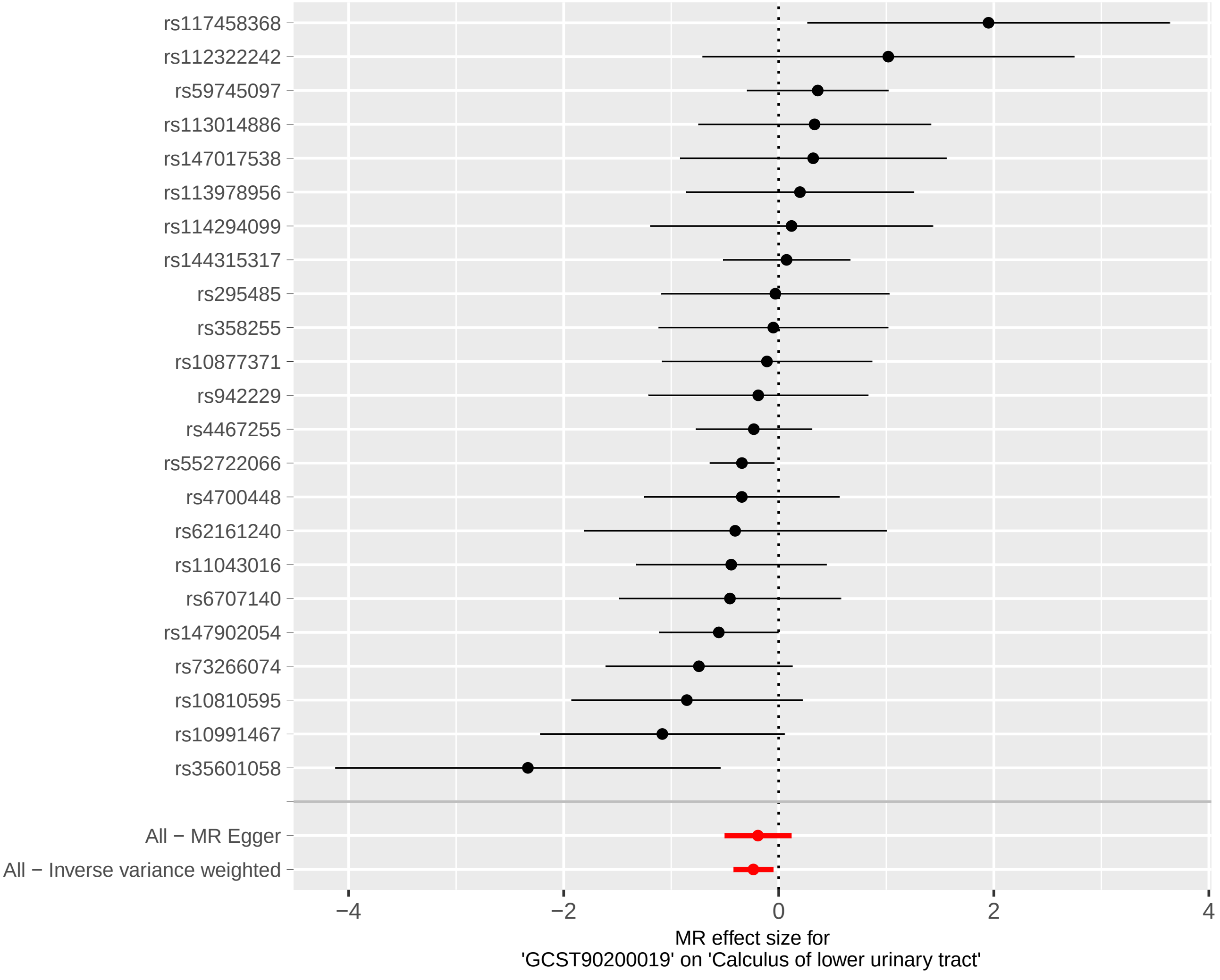
rs117458368
rs112322242
rs59745097
rs113014886
rs147017538
rs113978956
rs114294099
rs144315317
rs295485
rs358255
rs10877371
rs942229
rs4467255
rs552722066
rs4700448
rs62161240
rs11043016
rs6707140
rs147902054
rs73266074
rs10810595
rs10991467
rs35601058
All − MR Egger
All − Inverse variance weighted
−4
−2
0
2
4
MR effect size for
'GCST90200019' on 'Calculus of lower urinary tract'

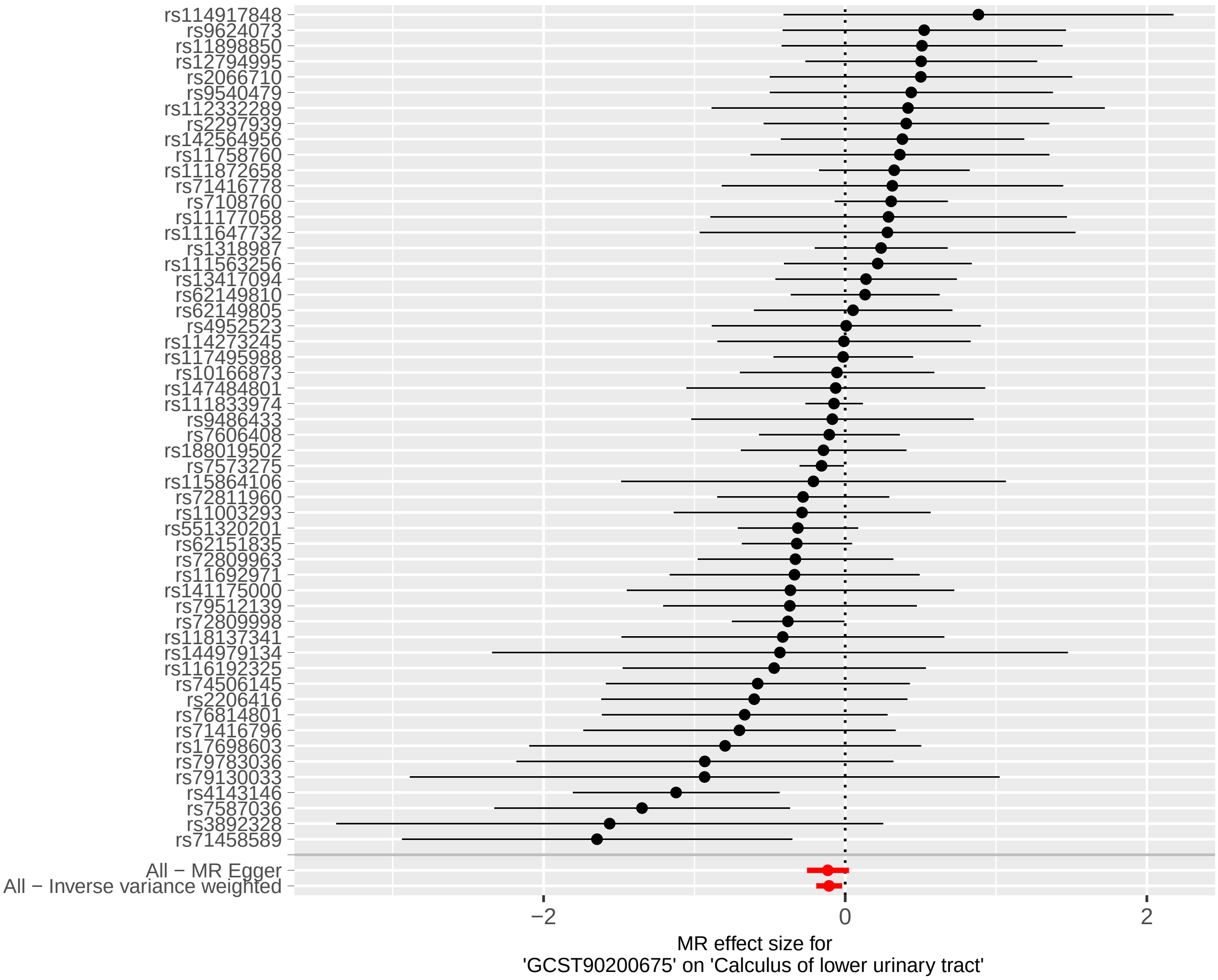
rs114917848
rs9624073
rs11898850
rs12794995
rs2066710
rs9540479
rs112332289
rs2297939
rs142564956
rs11758760
rs111872658
rs71416778
rs7108760
rs11177058
rs111647732
rs1318987
rs111563256
rs13417094
rs62149810
rs62149805
rs4952523
rs114273245
rs117495988
rs10166873
rs147484801
rs111833974
rs9486433
rs7606408
rs188019502
rs7573275
rs115864106
rs72811960
rs11003293
rs551320201
rs62151835
rs72809963
rs11692971
rs141175000
rs79512139
rs72809998
rs118137341
rs144979134
rs116192325
rs74506145
rs2206416
rs76814801
rs71416796
rs17698603
rs79783036
rs79130033
rs4143146
rs7587036
rs3892328
rs71458589
All − MR Egger
All − Inverse variance weighted
−2
0
2
MR effect size for
'GCST90200675' on 'Calculus of lower urinary tract'

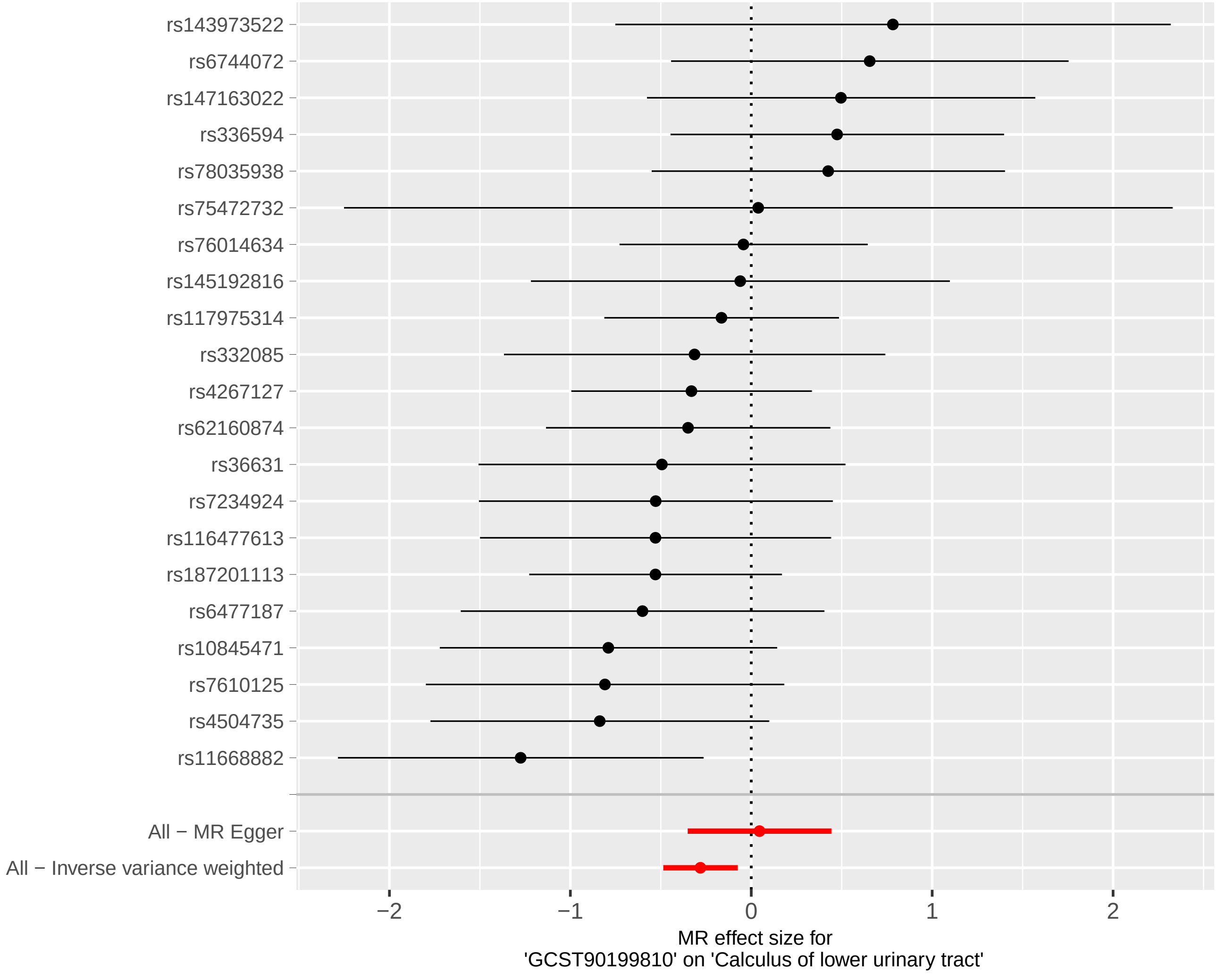

rs143973522
rs6744072
rs147163022
rs336594
rs78035938
rs75472732
rs76014634
rs145192816
rs117975314
rs332085
rs4267127
rs62160874
rs36631
rs7234924
rs116477613
rs187201113
rs6477187
rs10845471
rs7610125
rs4504735
rs11668882
All − MR Egger
All − Inverse variance weighted
−2
−1
0
1
2
MR effect size for
'GCST90199810' on 'Calculus of lower urinary tract'

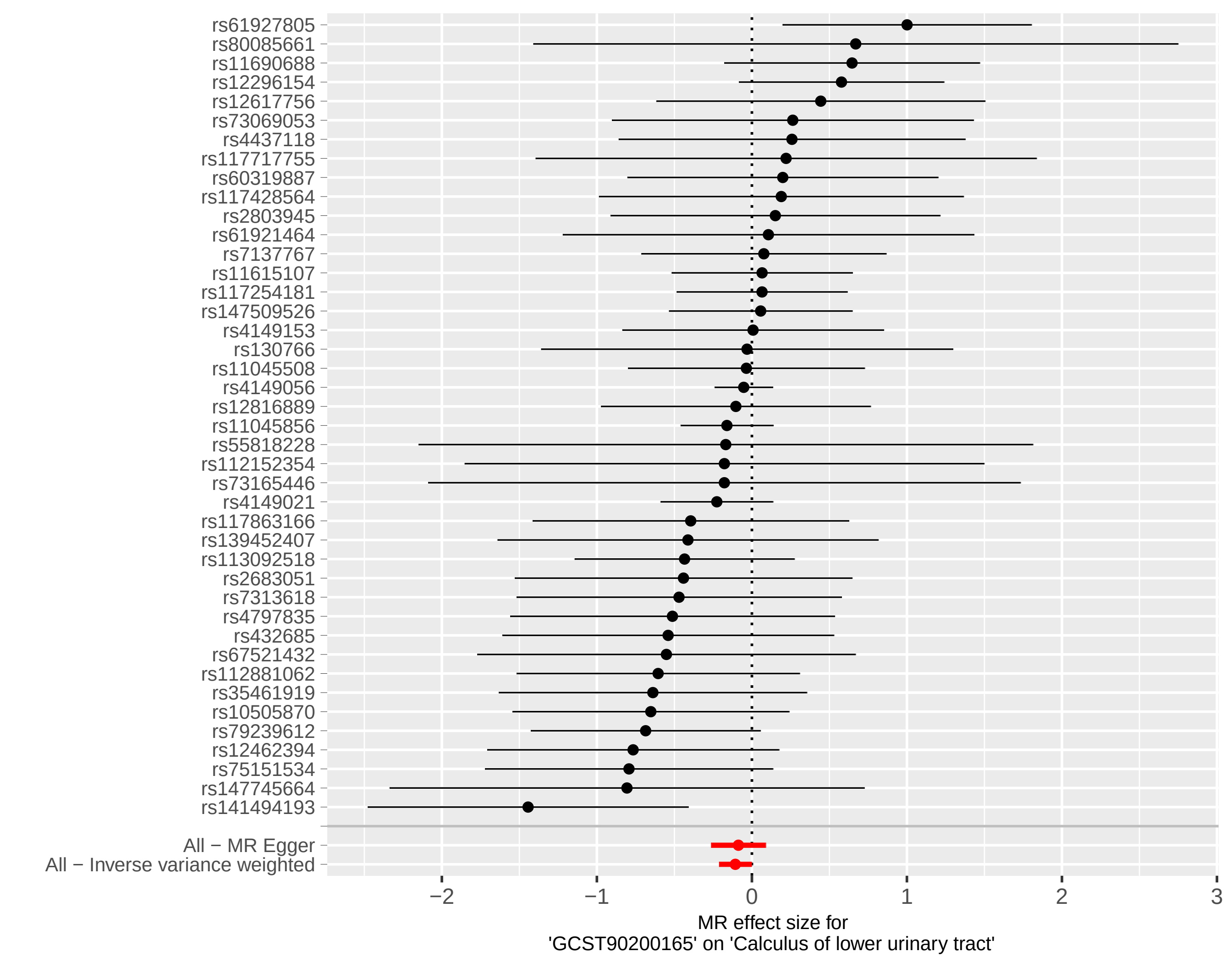
rs61927805
rs80085661
rs11690688
rs12296154
rs12617756
rs73069053
rs4437118
rs117717755
rs60319887
rs117428564
rs2803945
rs61921464
rs7137767
rs11615107
rs117254181
rs147509526
rs4149153
rs130766
rs11045508
rs4149056
rs12816889
rs11045856
rs55818228
rs112152354
rs73165446
rs4149021
rs117863166
rs139452407
rs113092518
rs2683051
rs7313618
rs4797835
rs432685
rs67521432
rs112881062
rs35461919
rs10505870
rs79239612
rs12462394
rs75151534
rs147745664
rs141494193
All − MR Egger
All − Inverse variance weighted
−2
−1
0
1
2
3
MR effect size for
'GCST90200165' on 'Calculus of lower urinary tract'

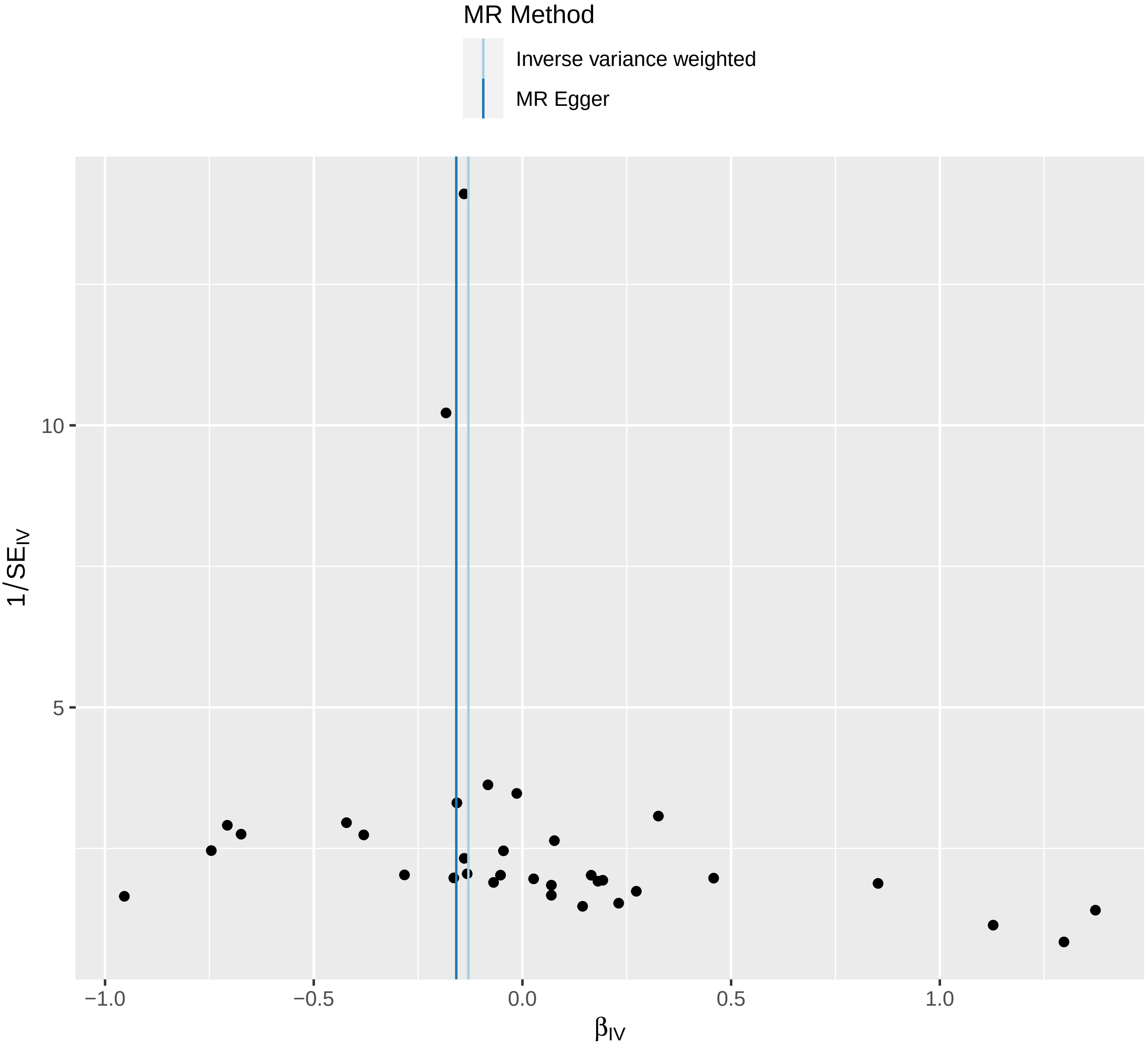
MR Method
Inverse variance weighted
MR Egger
1/SE_IV
10
5
−1.0
−0.5
0.0
0.5
1.0
β_IV

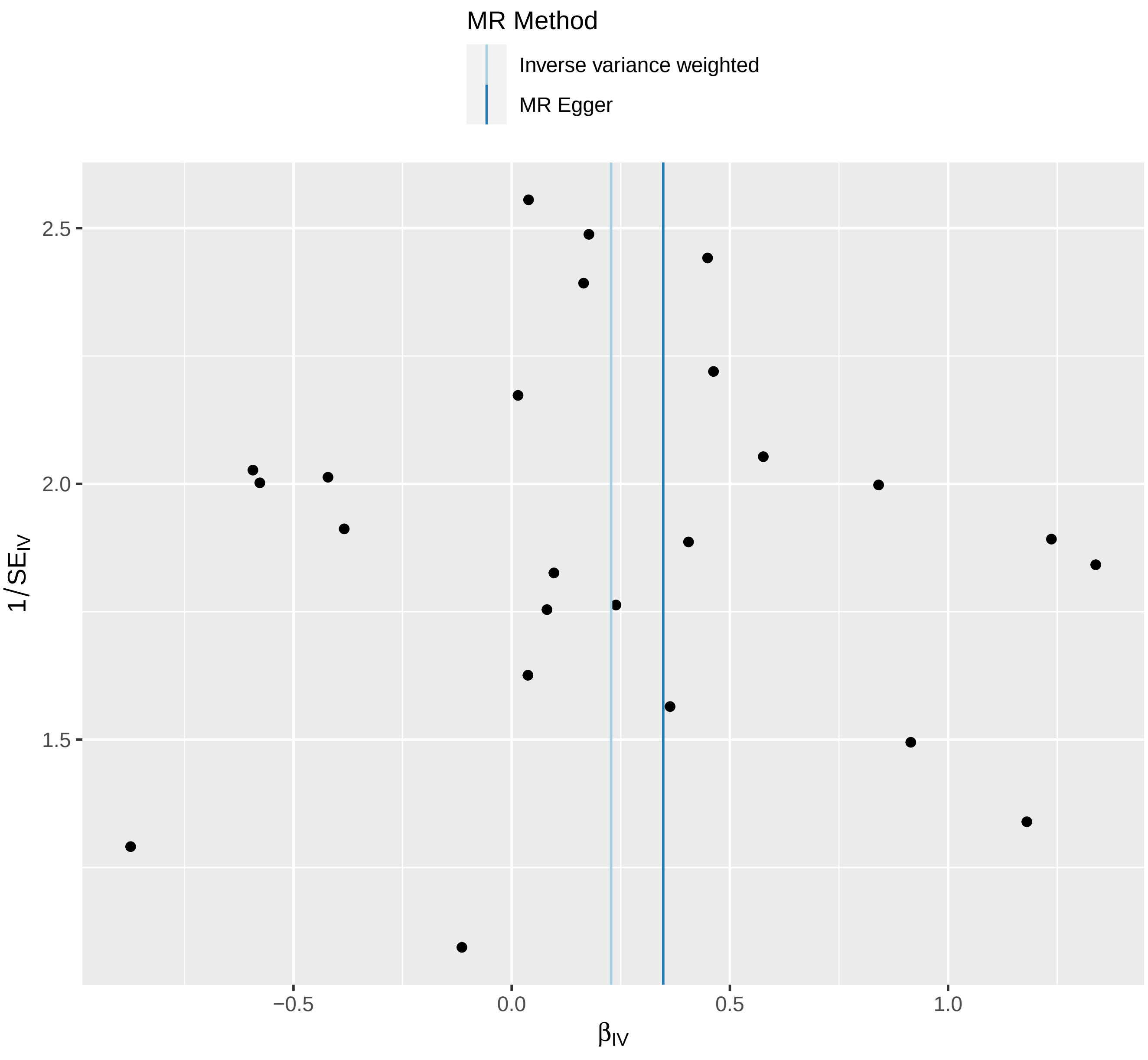
MR Method
Inverse variance weighted
MR Egger
2.5
2.0
1.5
1/SE$_{IV}$
−0.5
0.0
0.5
1.0
β$_{IV}$

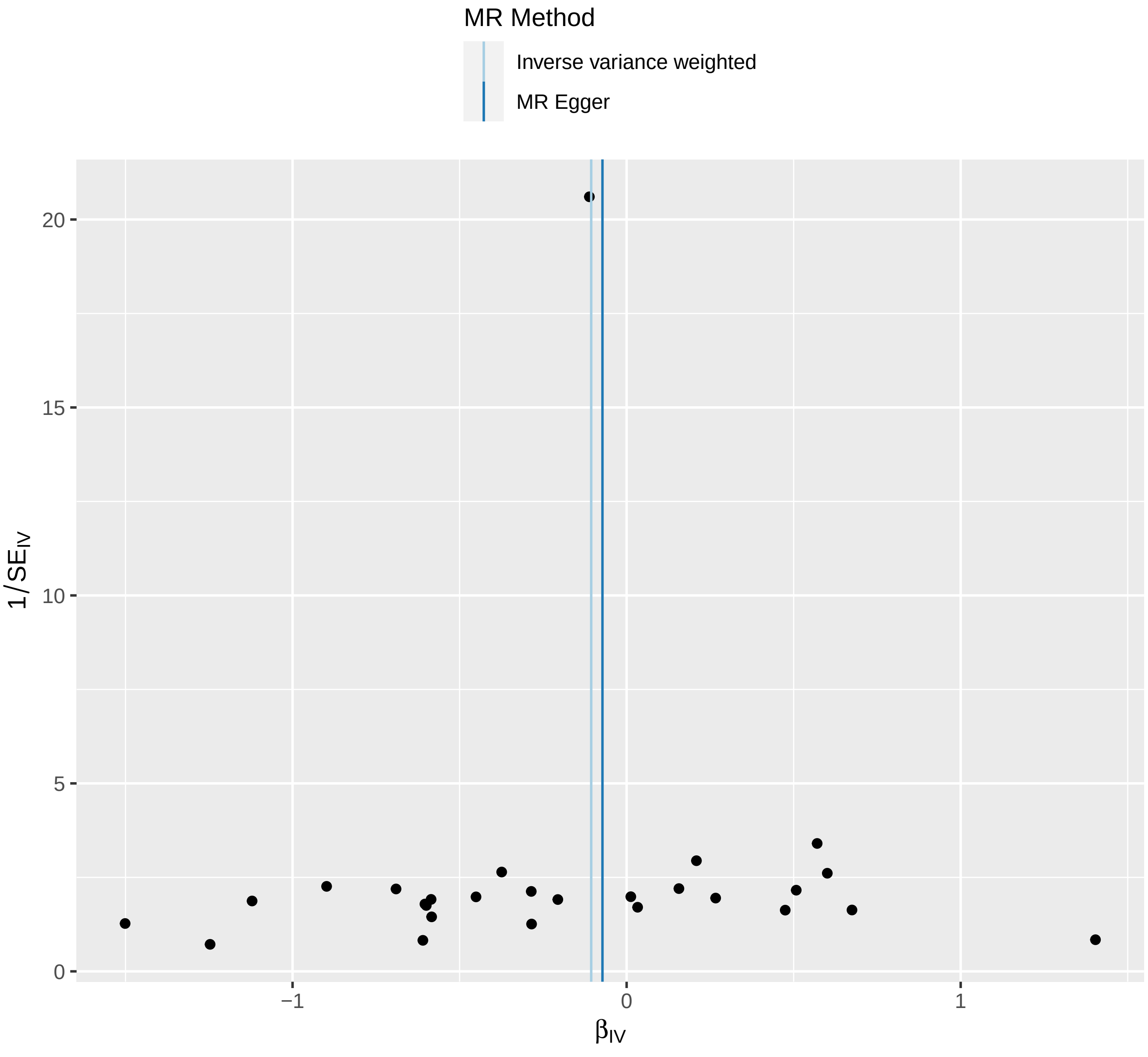

MR Method
Inverse variance weighted
MR Egger
20
15
10
5
0
−1
0
1
$1/SE_{IV}$
β_{IV}

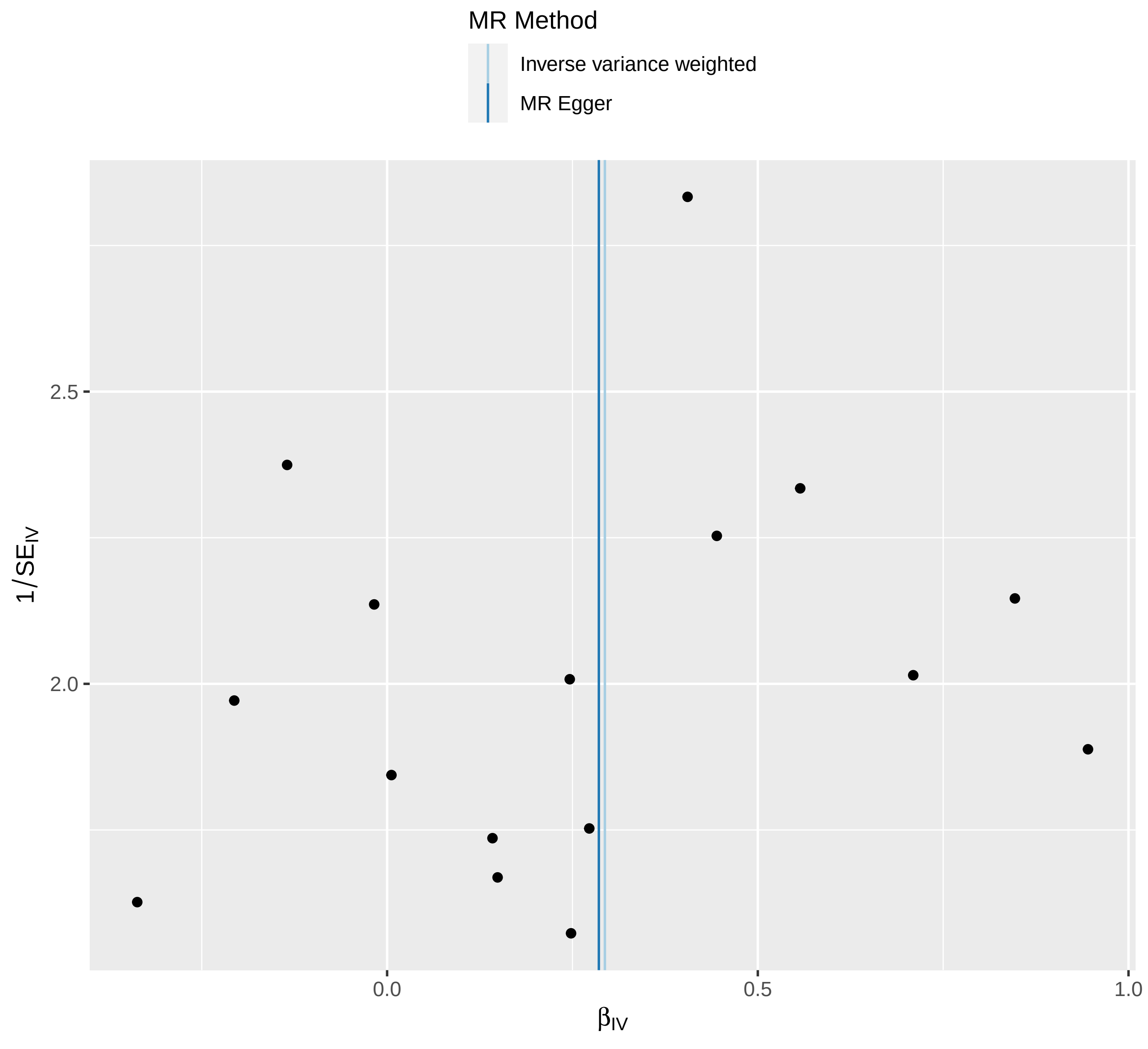
MR Method
Inverse variance weighted
MR Egger
2.5
2.0
$1/SE_{IV}$
0.0
0.5
1.0
β_{IV}

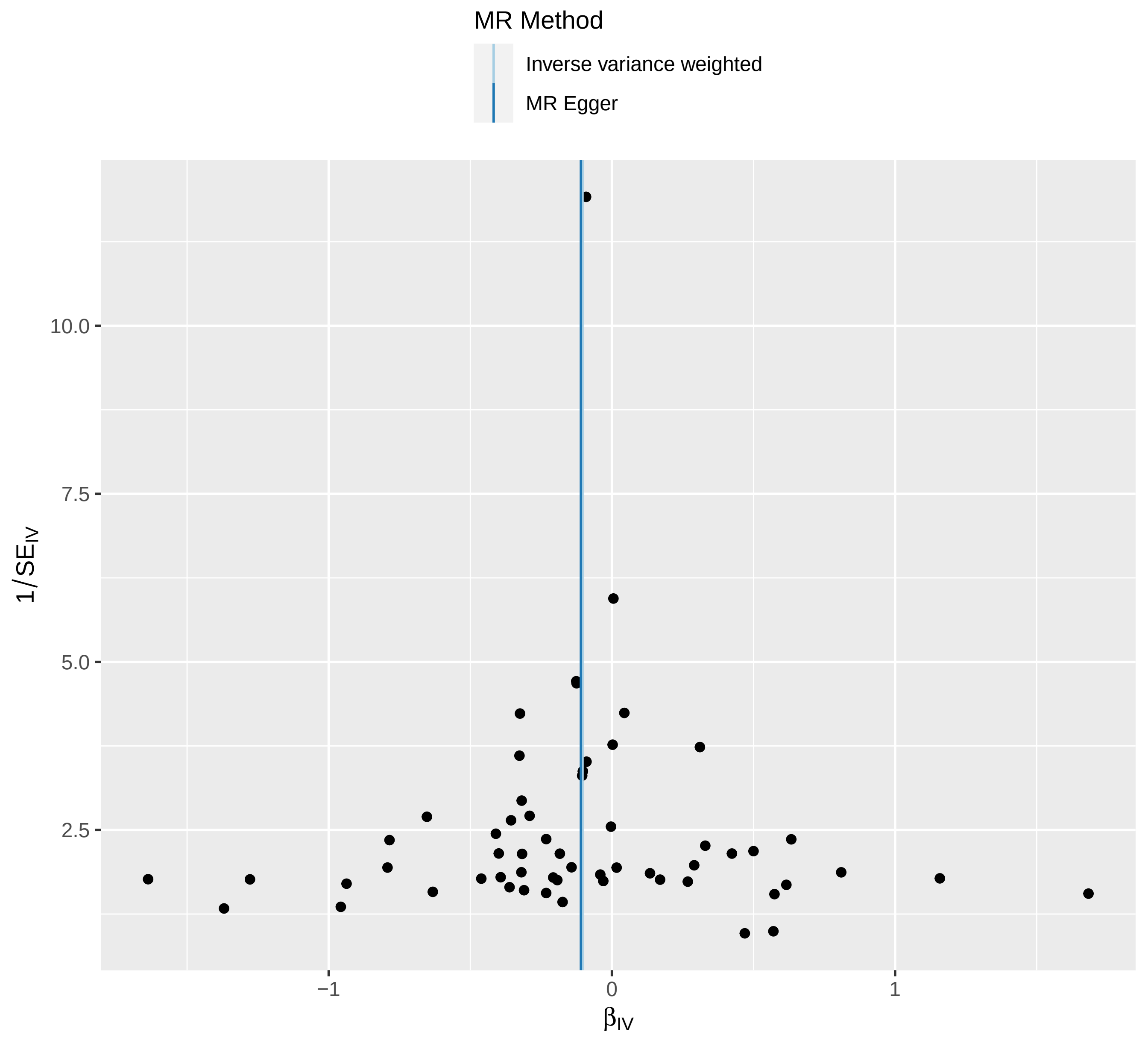

MR Method
Inverse variance weighted
MR Egger
10.0
7.5
5.0
2.5
1/SE_IV
−1
0
1
β_IV

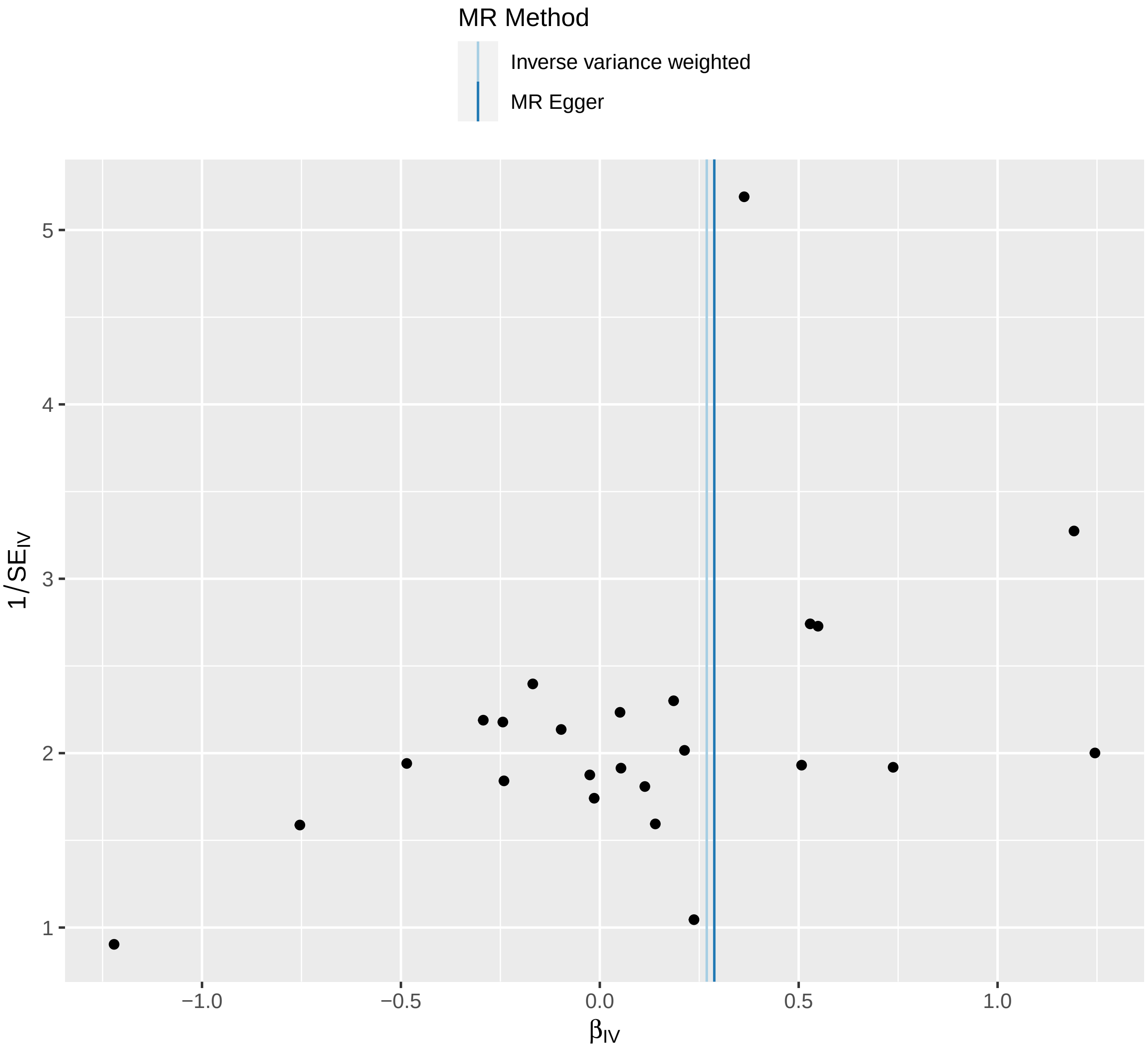

MR Method
Inverse variance weighted
MR Egger
$1/SE_{IV}$
5
4
3
2
1
−1.0
−0.5
0.0
0.5
1.0
β_{IV}

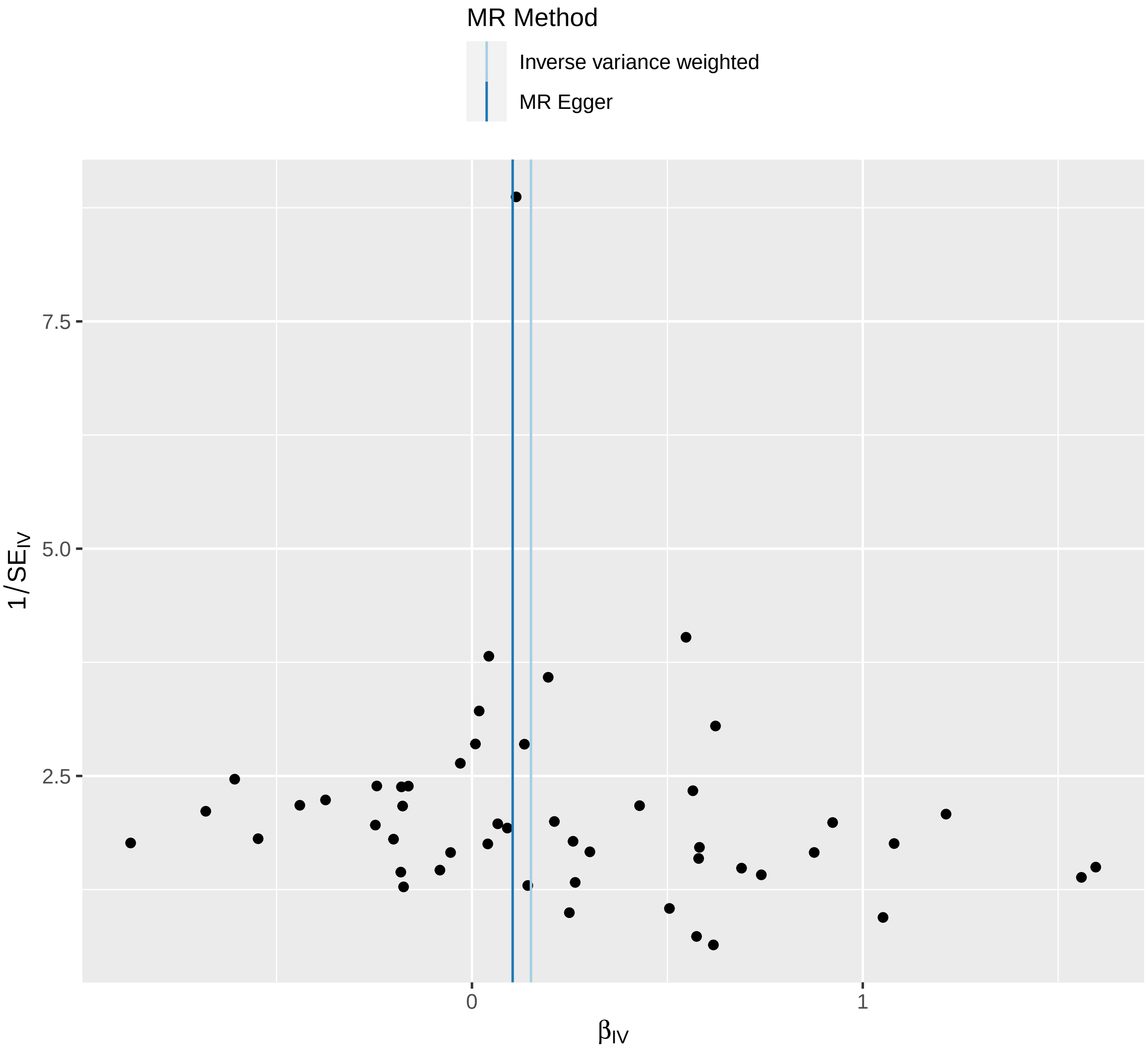
MR Method
Inverse variance weighted
MR Egger
7.5
5.0
2.5
1/SE_IV
0
1
β_IV

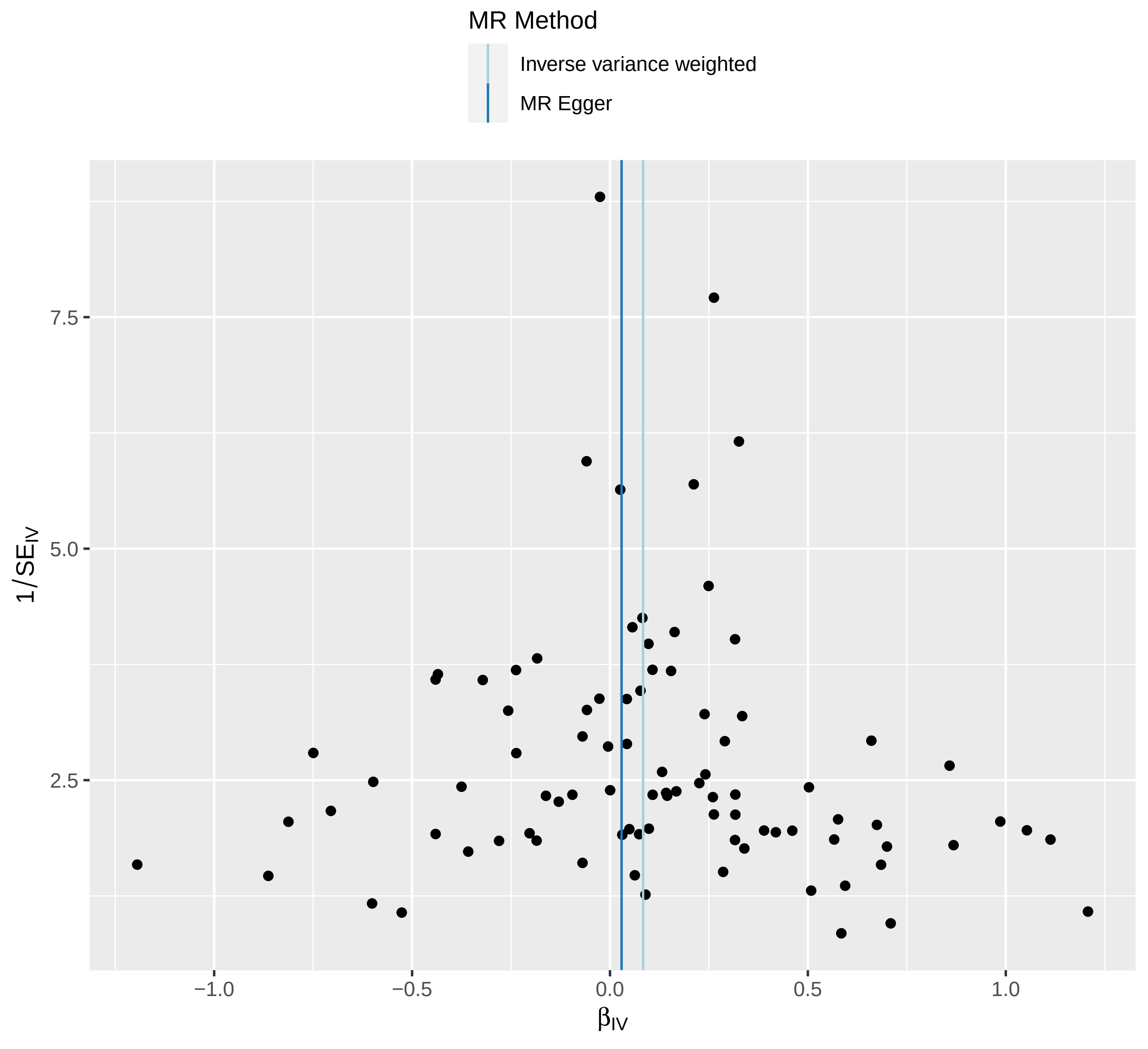

MR Method
Inverse variance weighted
MR Egger
7.5
5.0
2.5
1/SE_IV
−1.0
−0.5
0.0
0.5
1.0
β_IV

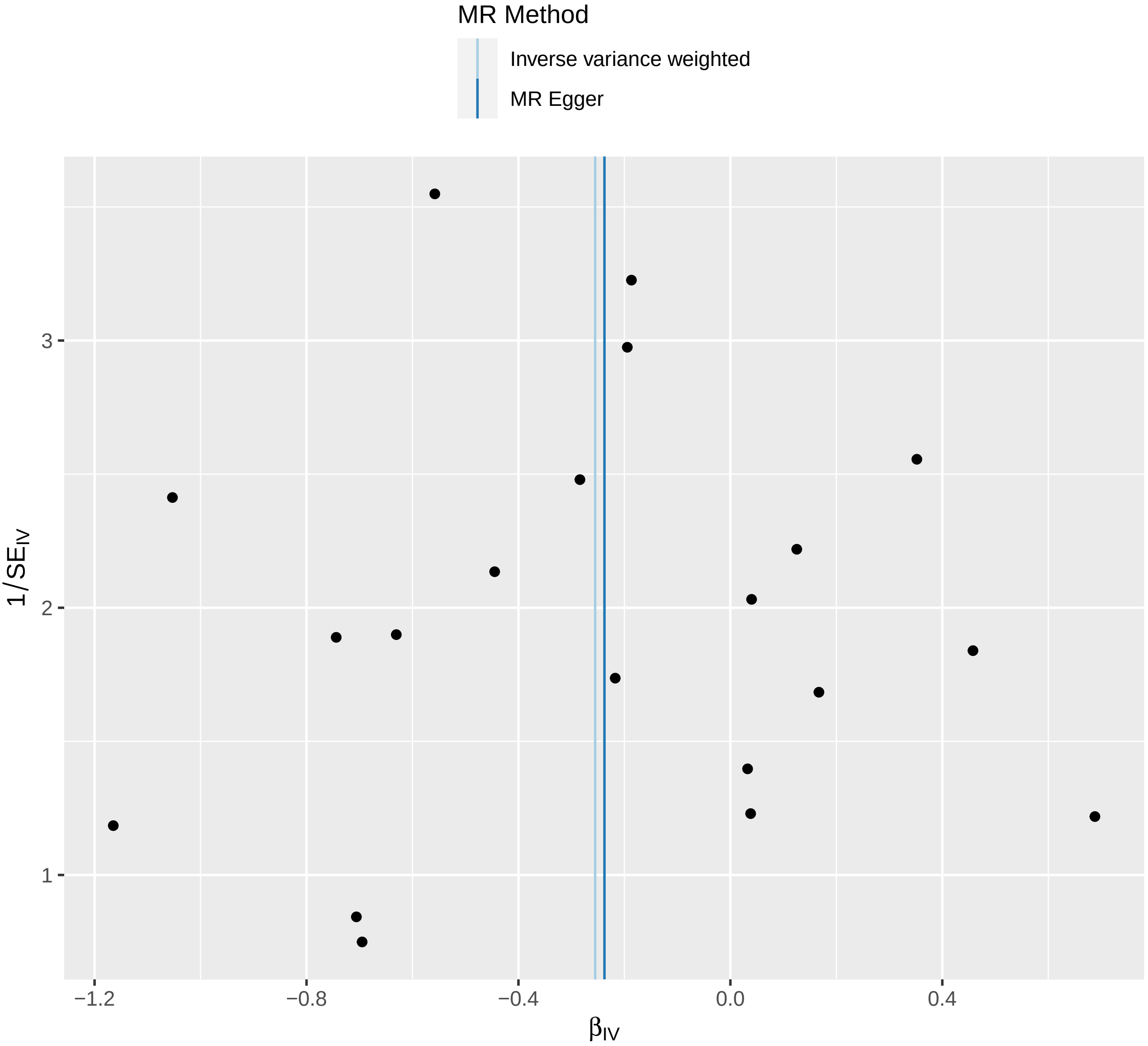
MR Method
Inverse variance weighted
MR Egger
1/SE_IV
3
2
1
−1.2
−0.8
−0.4
0.0
0.4
β_IV

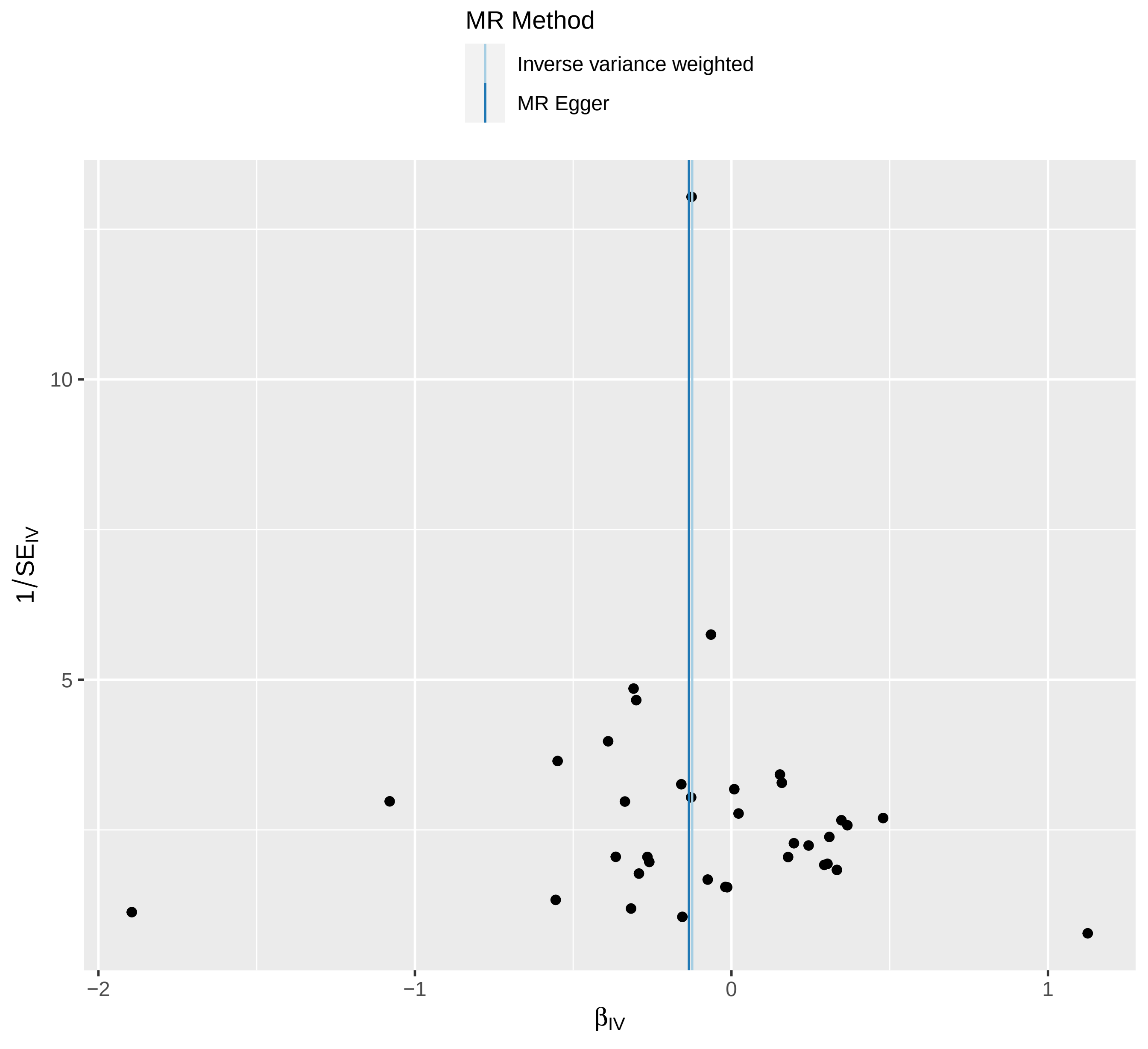
MR Method
Inverse variance weighted
MR Egger
10
5
1/SE_IV
−2
−1
0
1
β_IV

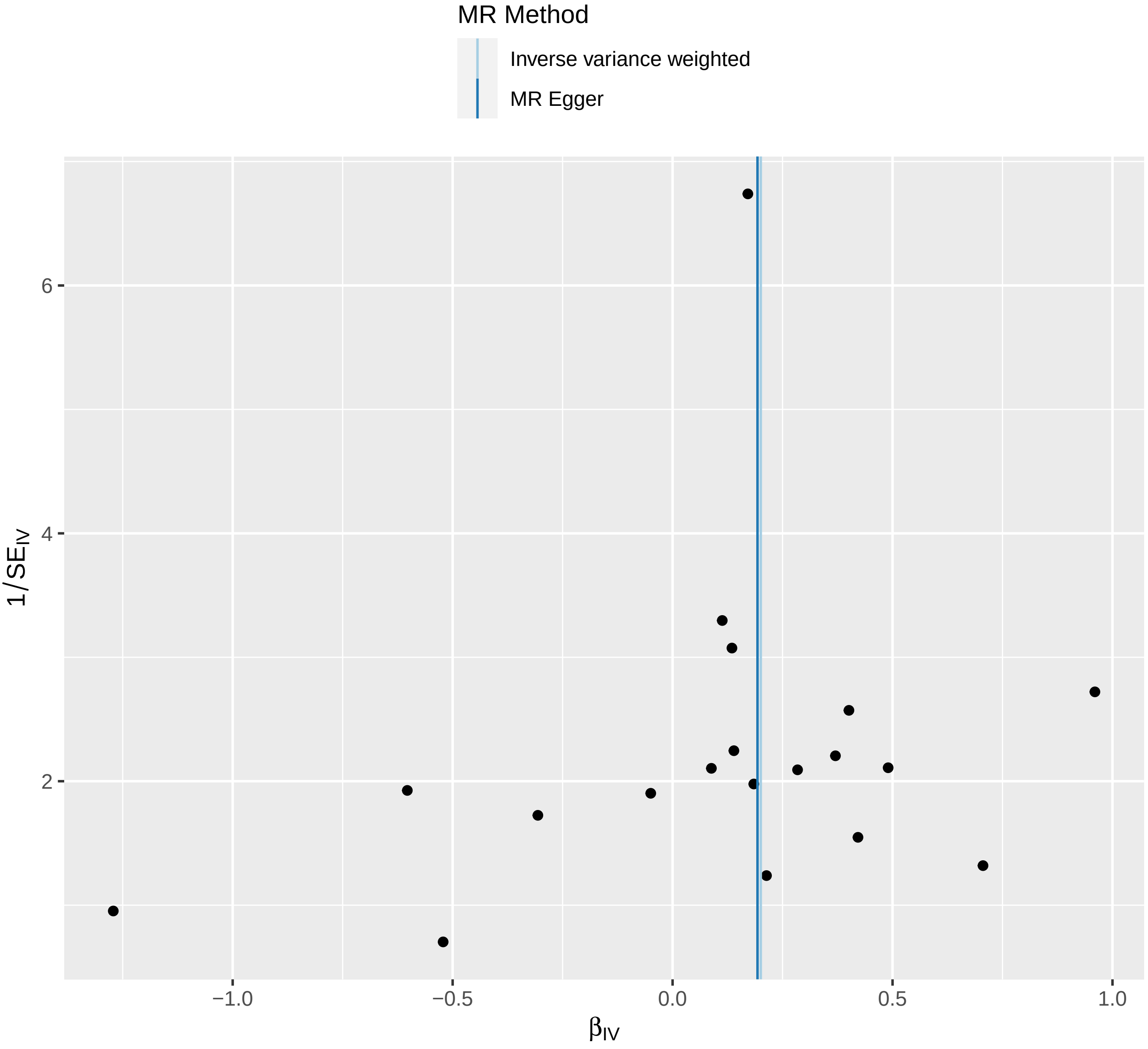
MR Method
Inverse variance weighted
MR Egger
6
4
2
$1/SE_{IV}$
−1.0
−0.5
0.0
0.5
1.0
β_{IV}

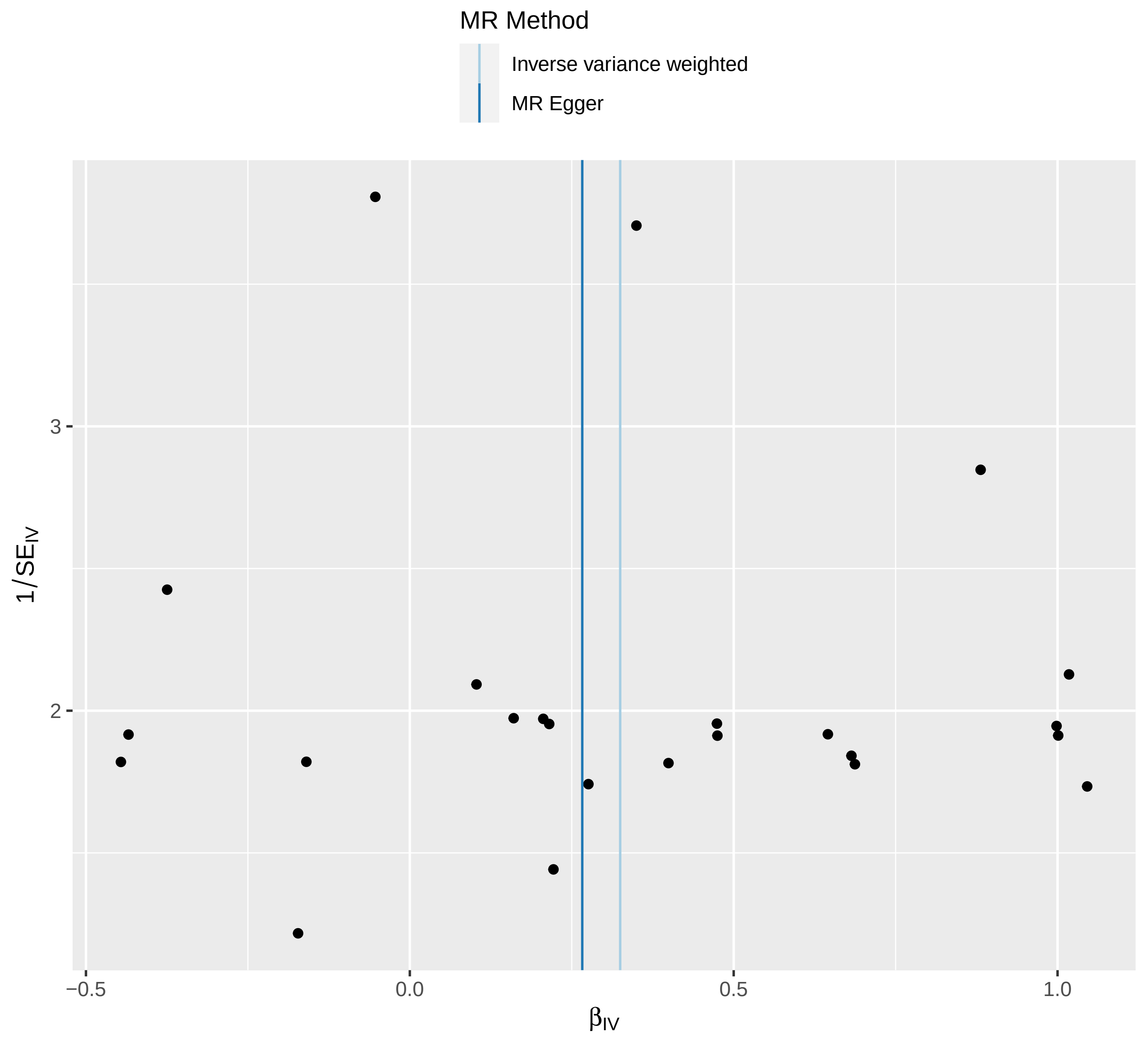
MR Method
Inverse variance weighted
MR Egger
3
2
1/SE$_{IV}$
−0.5
0.0
0.5
1.0
β$_{IV}$

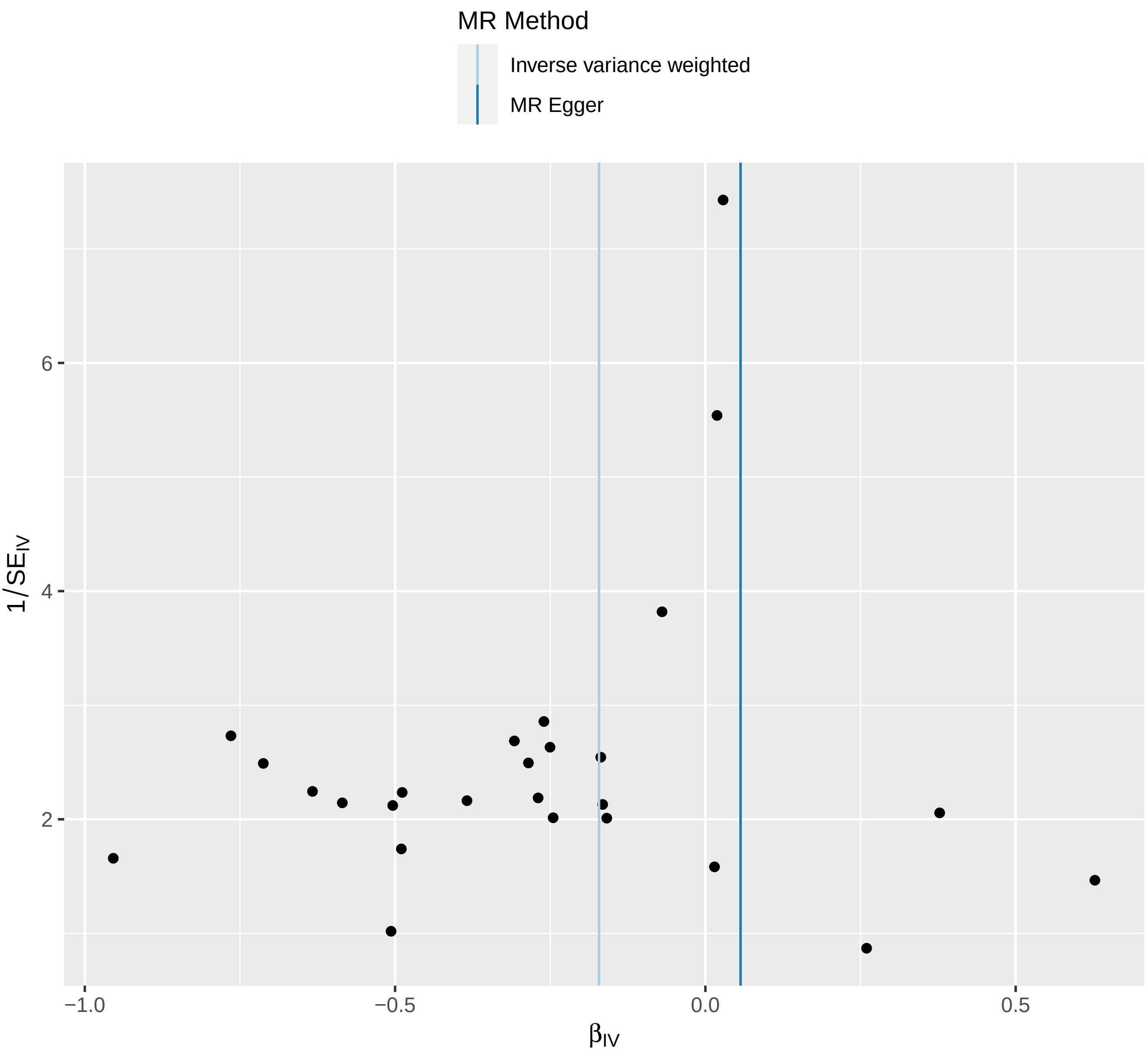
MR Method
Inverse variance weighted
MR Egger
6
4
2
1/SE_IV
−1.0
−0.5
0.0
0.5
β_IV

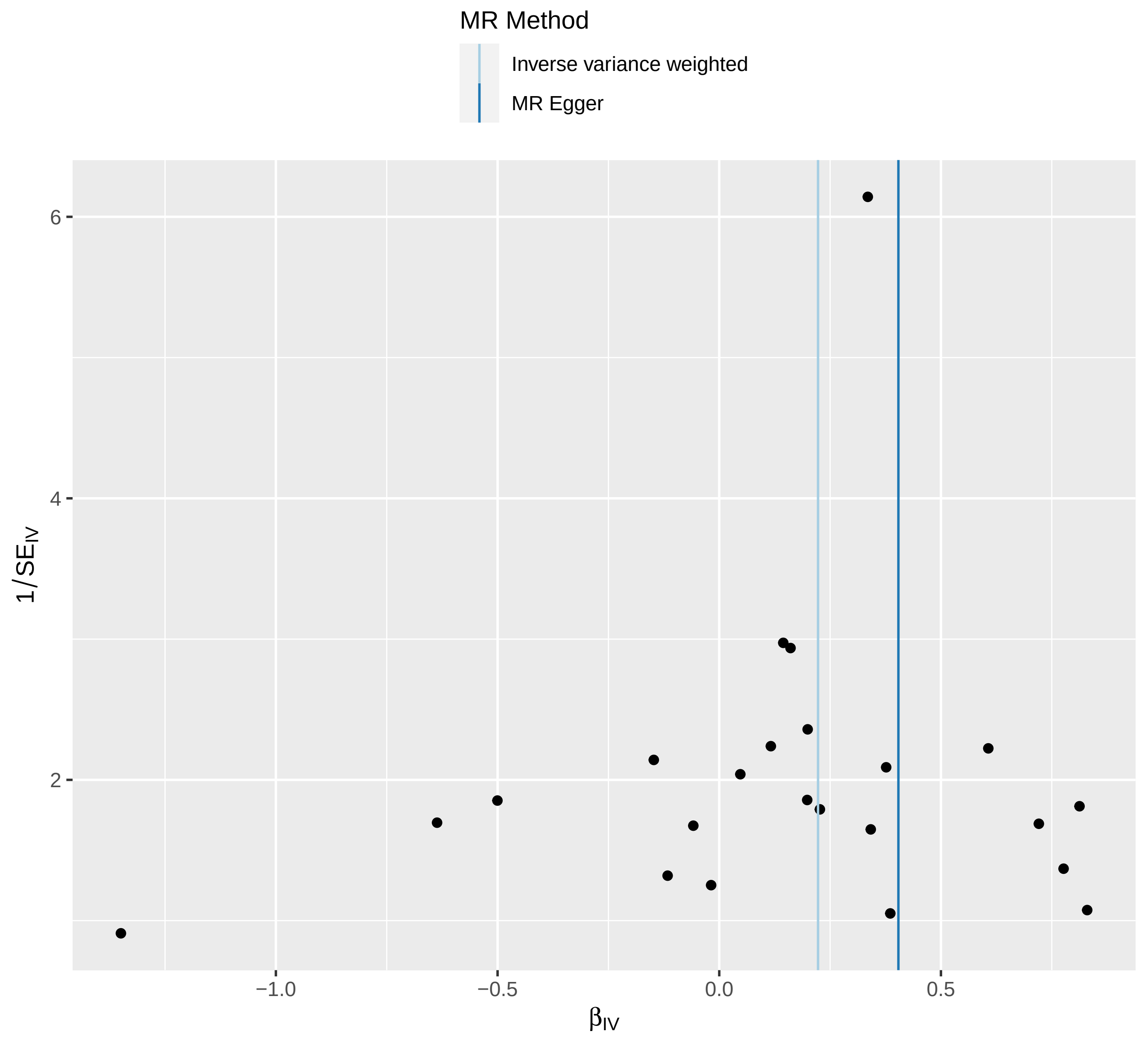

MR Method
Inverse variance weighted
MR Egger
6
4
2
1/SE_IV
−1.0
−0.5
0.0
0.5
β_IV

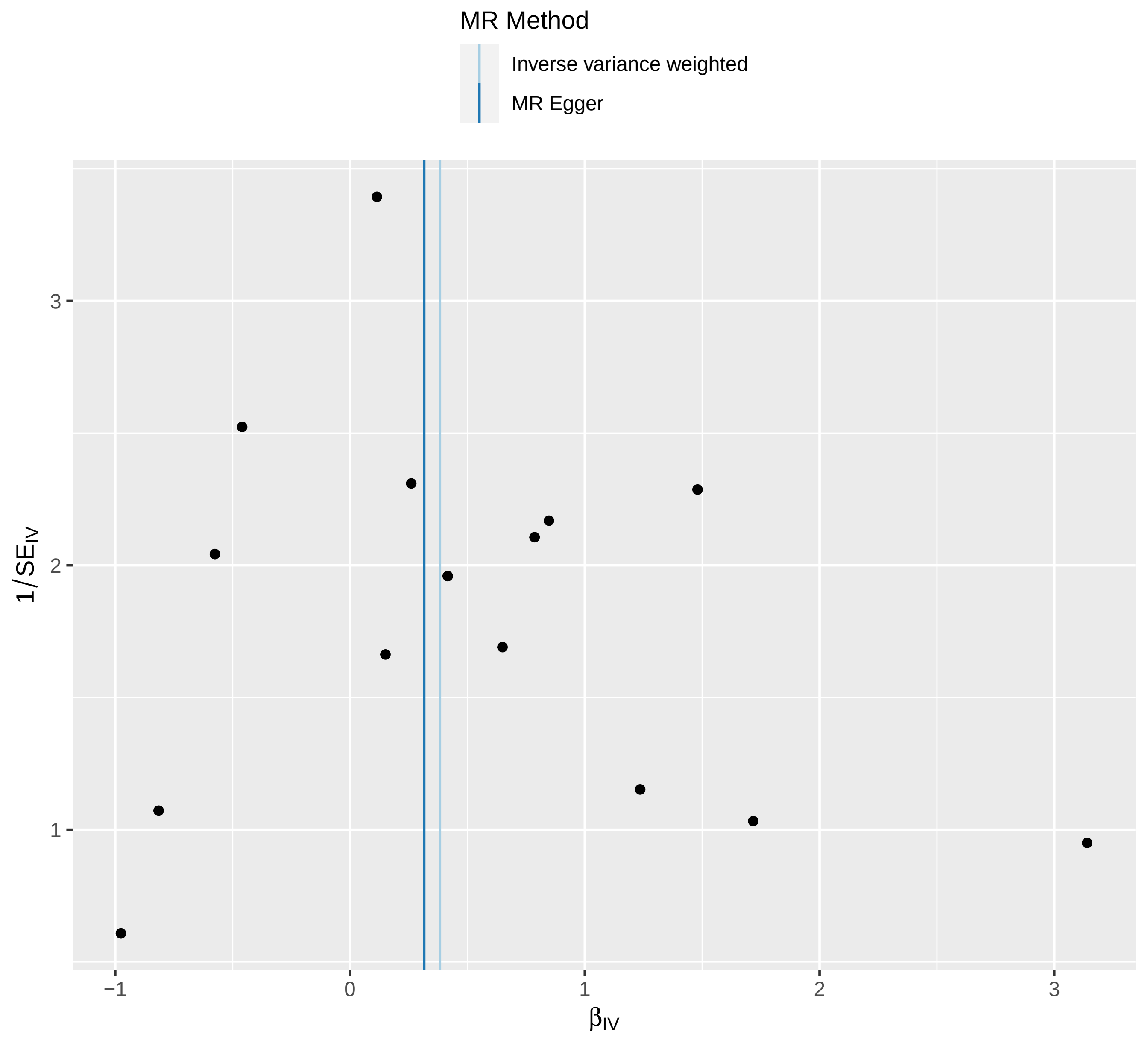
MR Method
Inverse variance weighted
MR Egger
1/SE_{IV}
β_{IV}
3
2
1
−1
0
1
2
3

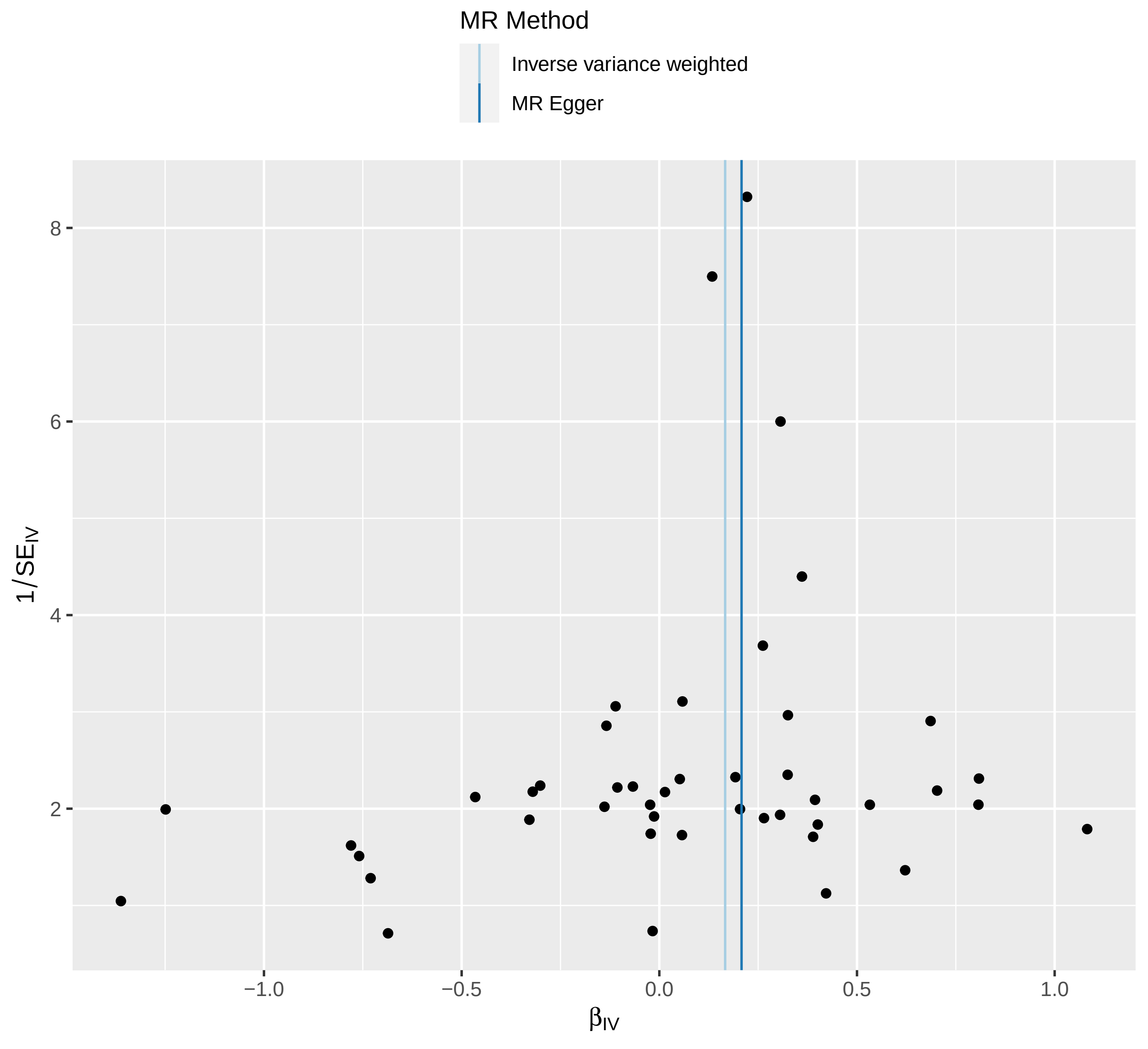
MR Method
Inverse variance weighted
MR Egger
8
6
4
2
1/SE_IV
−1.0
−0.5
0.0
0.5
1.0
β_IV

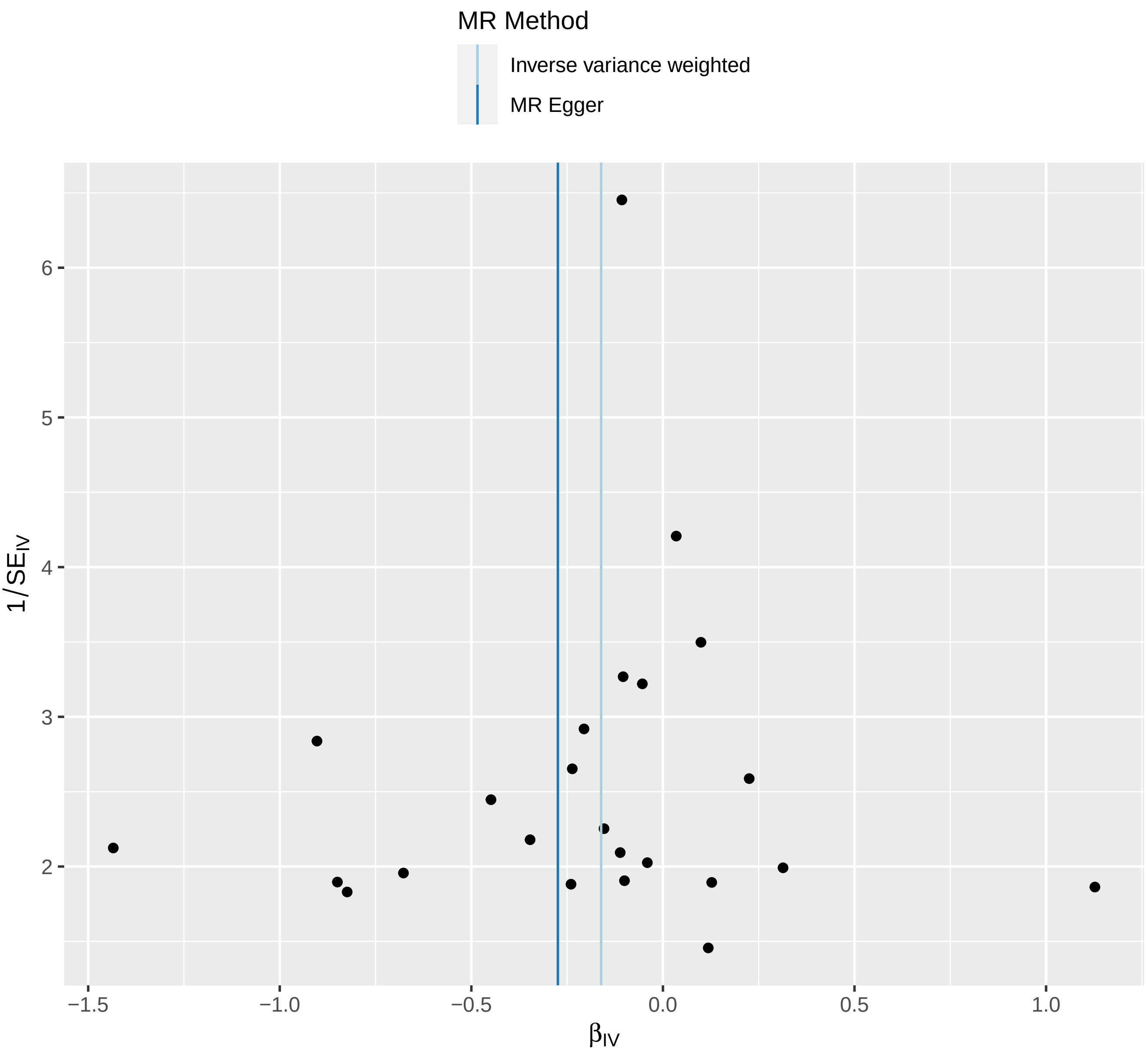
MR Method
Inverse variance weighted
MR Egger
1/SE$_{IV}$
β$_{IV}$
6
5
4
3
2
−1.5
−1.0
−0.5
0.0
0.5
1.0

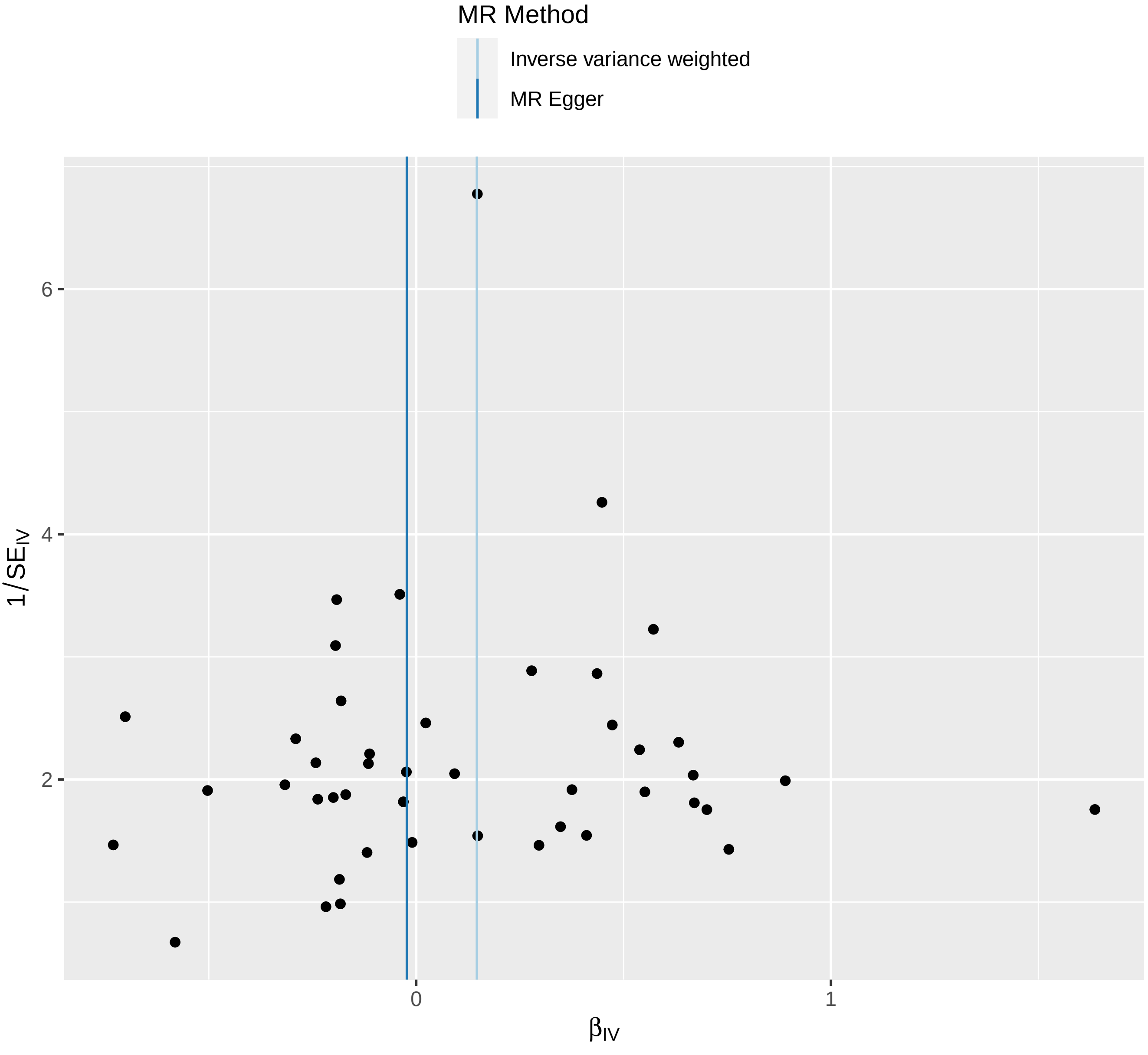
MR Method
Inverse variance weighted
MR Egger
6
4
2
0
1
1/SE_IV
β_IV

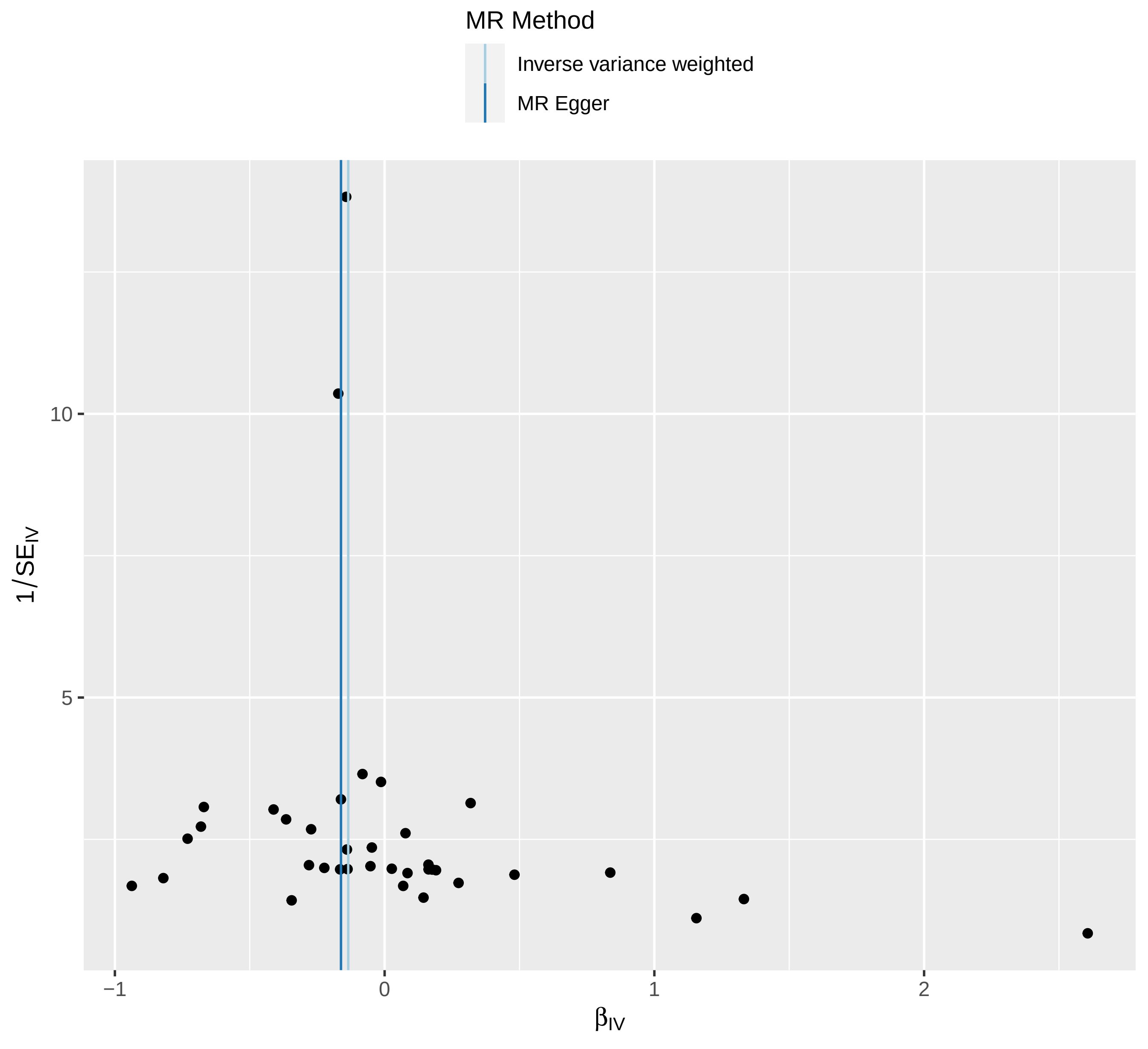
MR Method
Inverse variance weighted
MR Egger
1/SE_IV
10
5
−1
0
1
2
β_IV

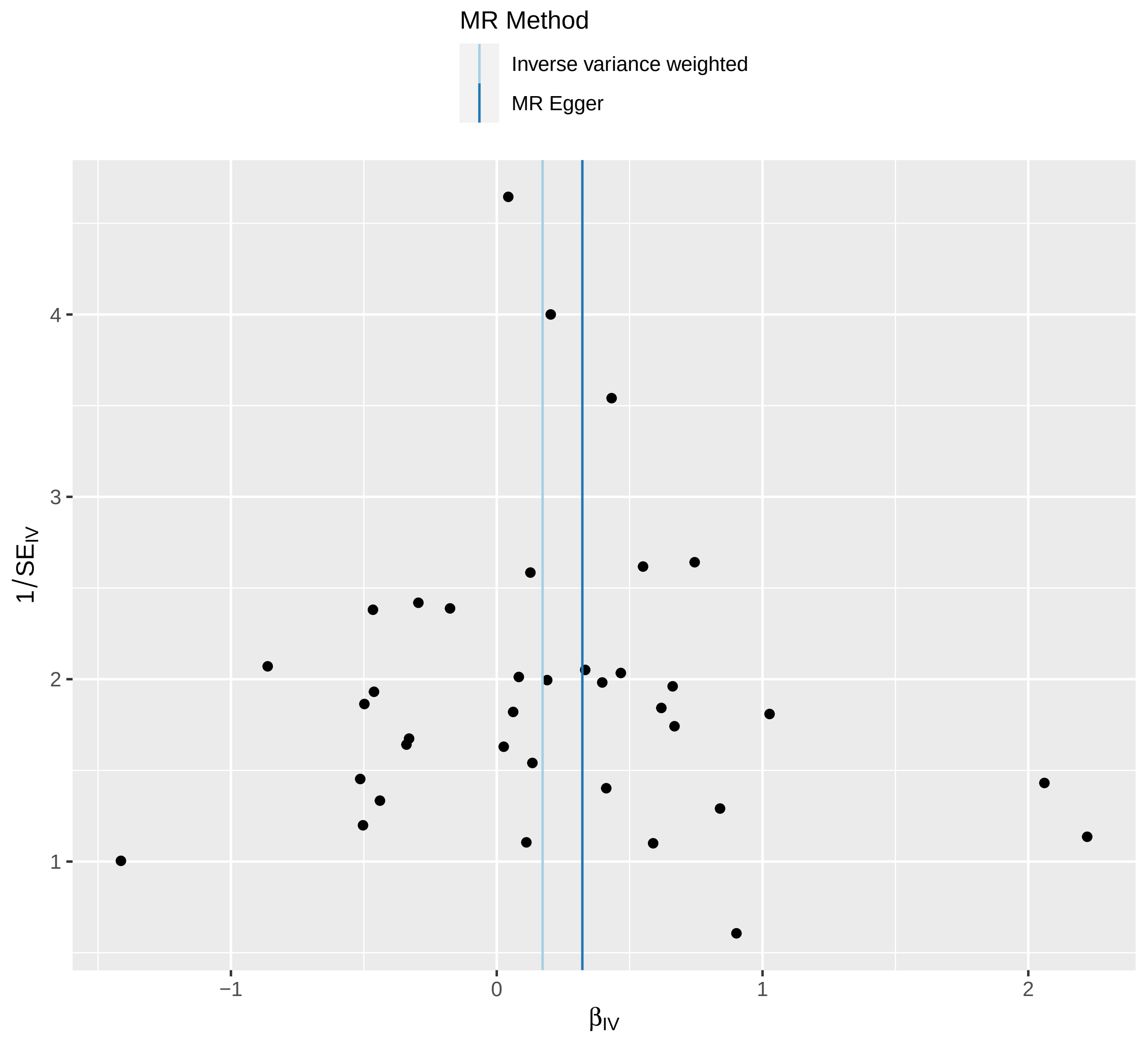
MR Method
Inverse variance weighted
MR Egger
$1/SE_{IV}$
β_{IV}
4
3
2
1
−1
0
1
2

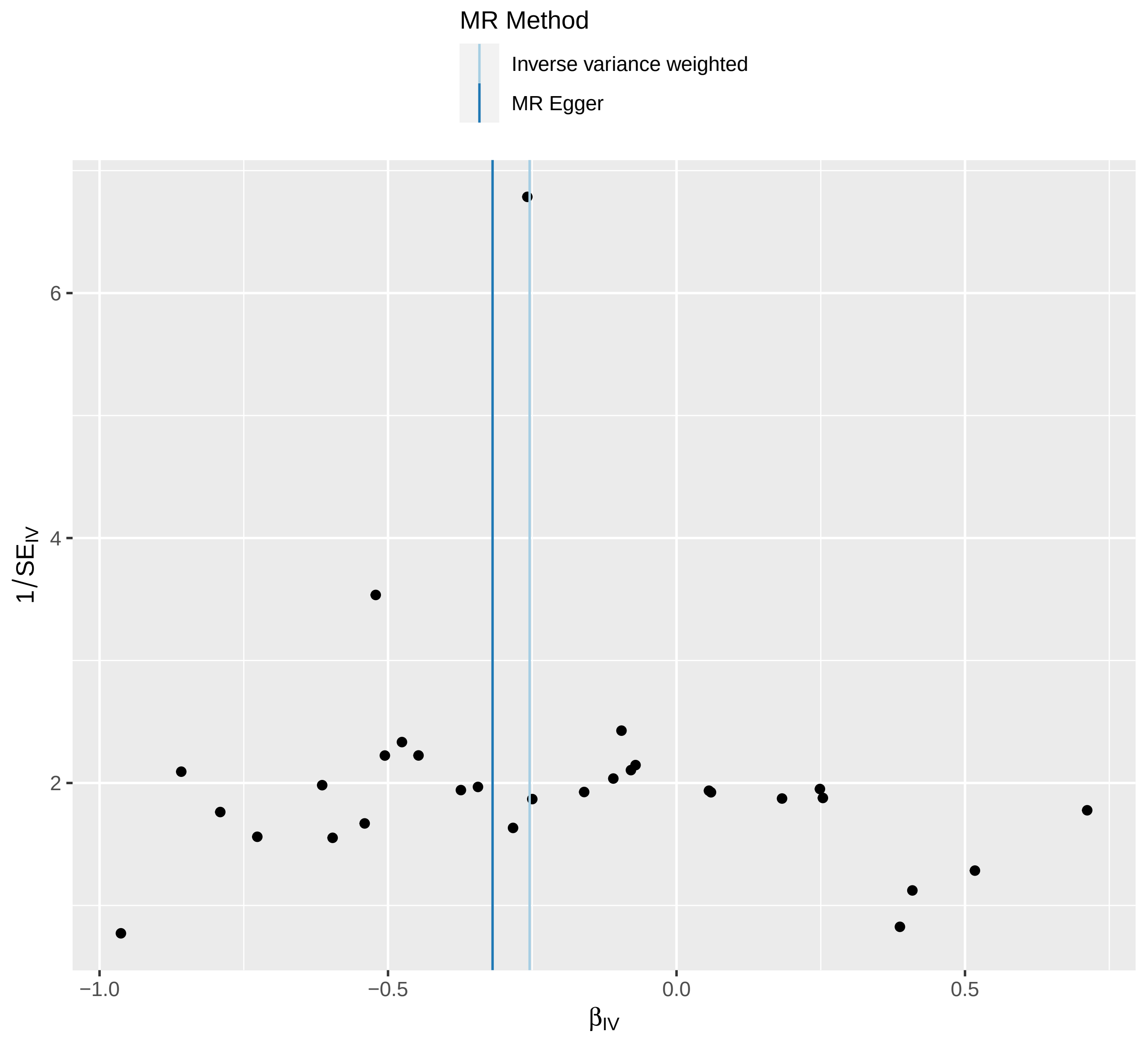

MR Method
Inverse variance weighted
MR Egger
6
4
2
1/SE_IV
−1.0
−0.5
0.0
0.5
β_IV

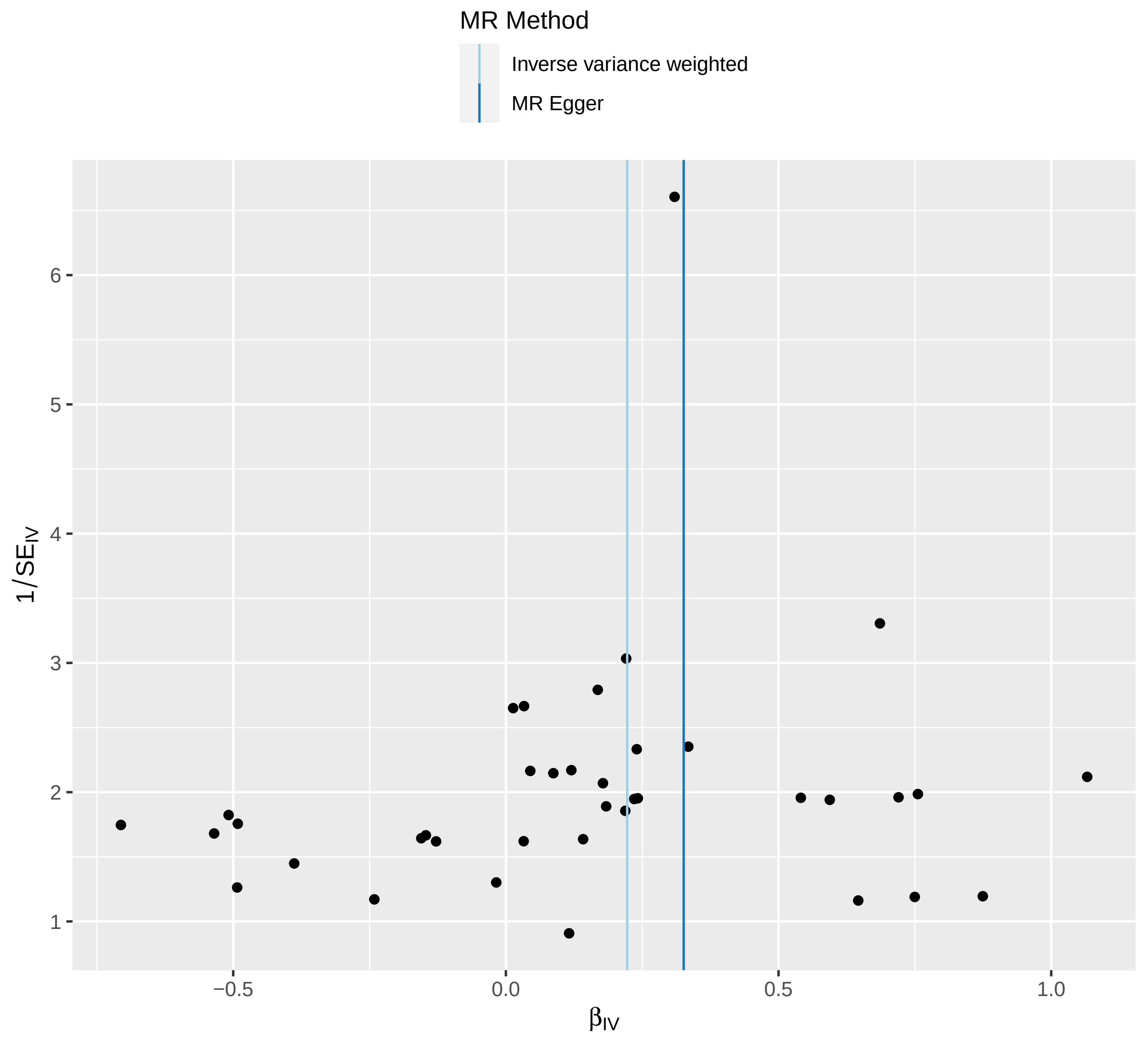

MR Method
Inverse variance weighted
MR Egger
1/SE_{IV}
β_{IV}
6
5
4
3
2
1
−0.5
0.0
0.5
1.0

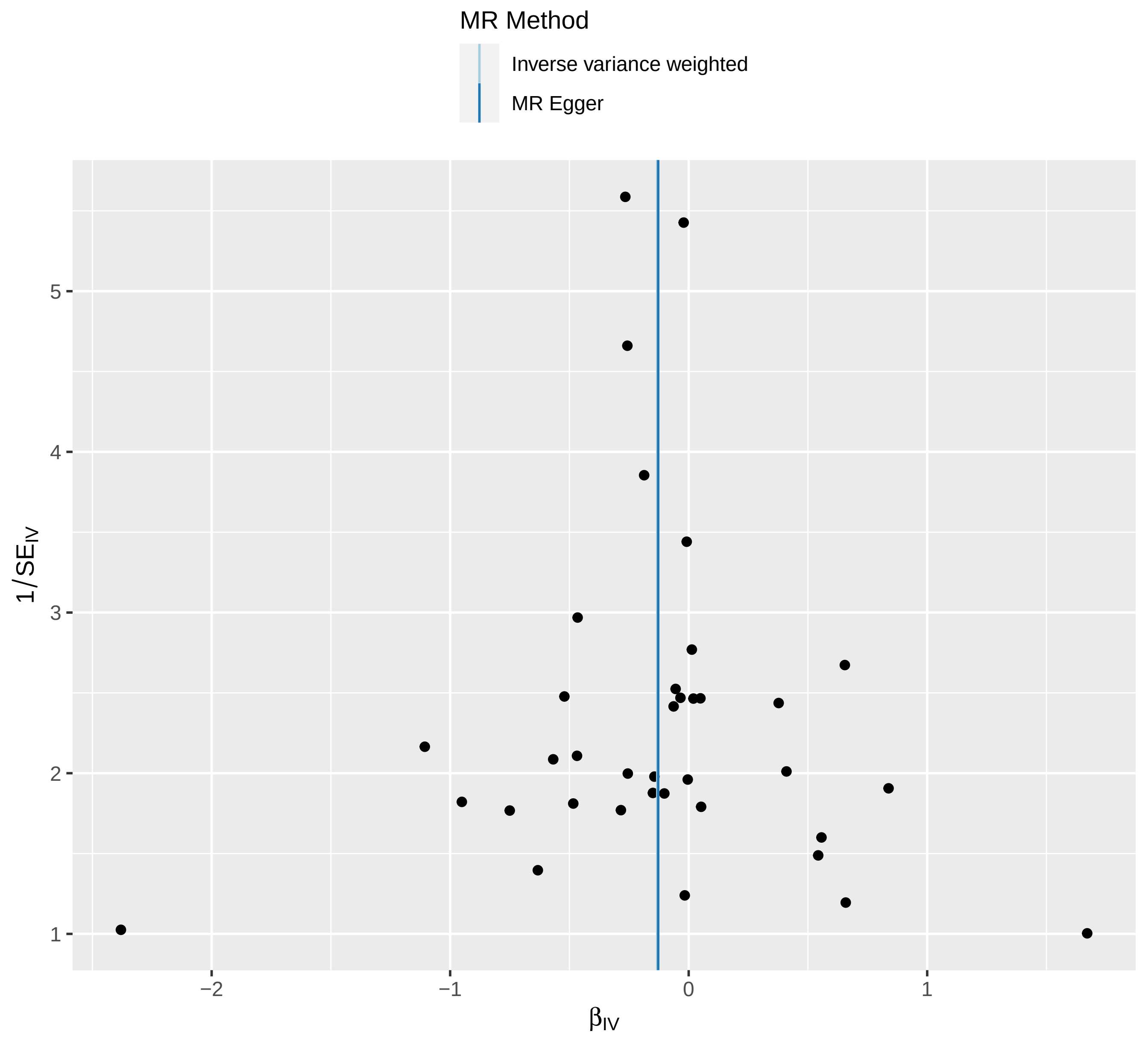

MR Method
Inverse variance weighted
MR Egger
5
4
3
2
1
1/SE$_{IV}$
−2
−1
0
1
β$_{IV}$

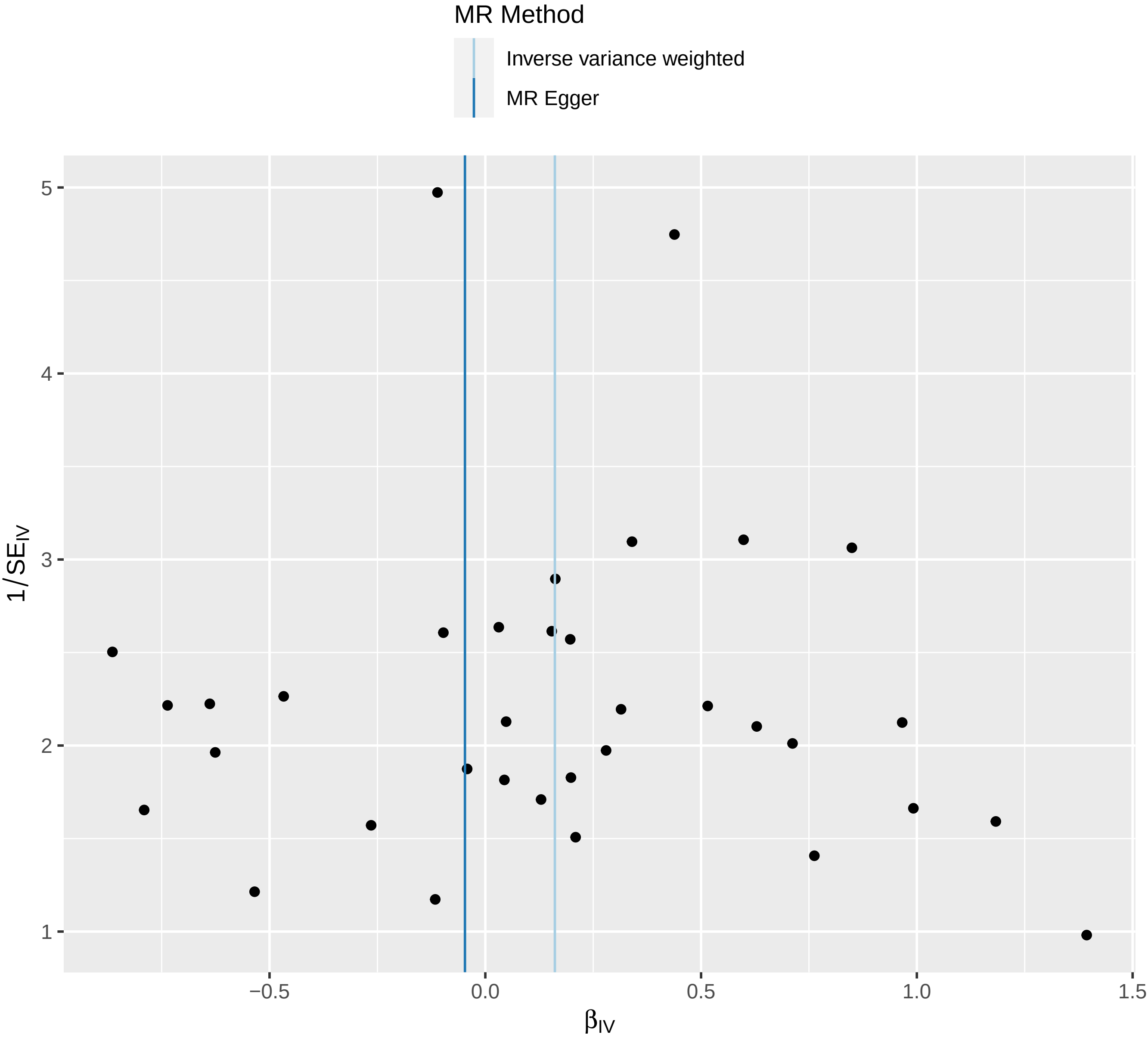
MR Method
Inverse variance weighted
MR Egger
5
4
3
2
1
1/SE_IV
−0.5
0.0
0.5
1.0
1.5
β_IV

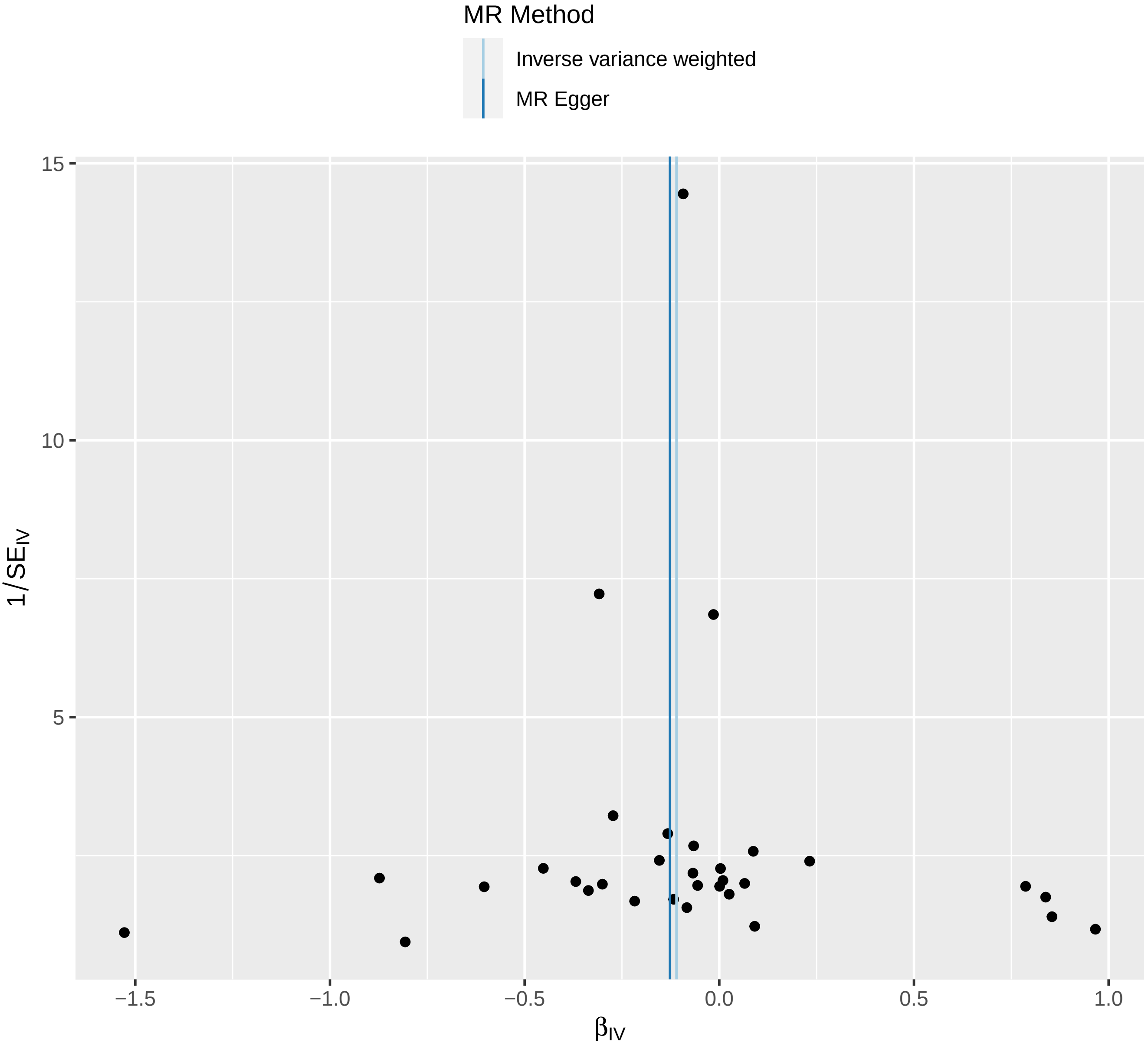
MR Method
Inverse variance weighted
MR Egger
15
10
5
$1/SE_{IV}$
−1.5
−1.0
−0.5
0.0
0.5
1.0
β_{IV}

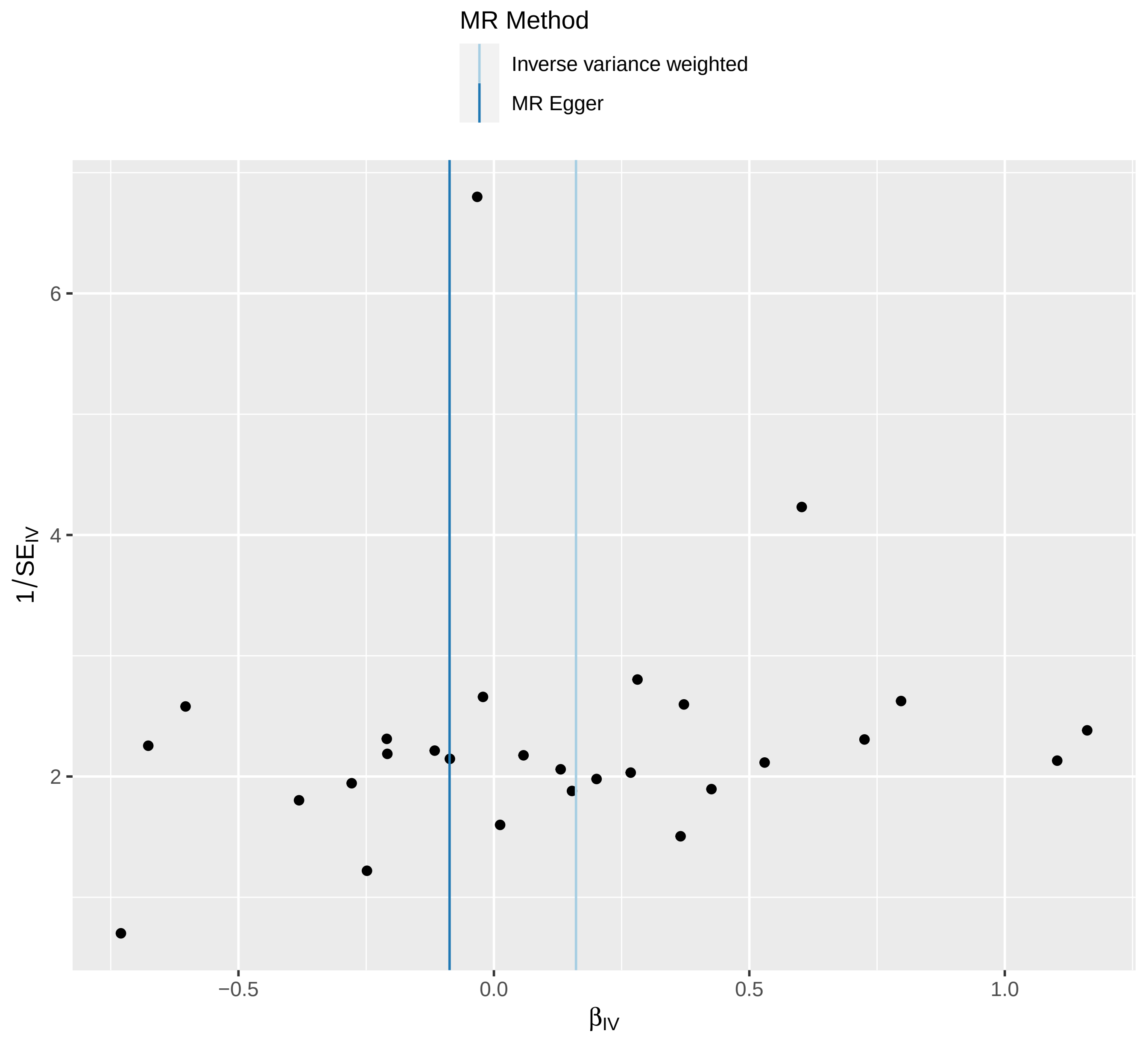

MR Method
Inverse variance weighted
MR Egger
6
4
2
$1/SE_{IV}$
−0.5
0.0
0.5
1.0
β_{IV}

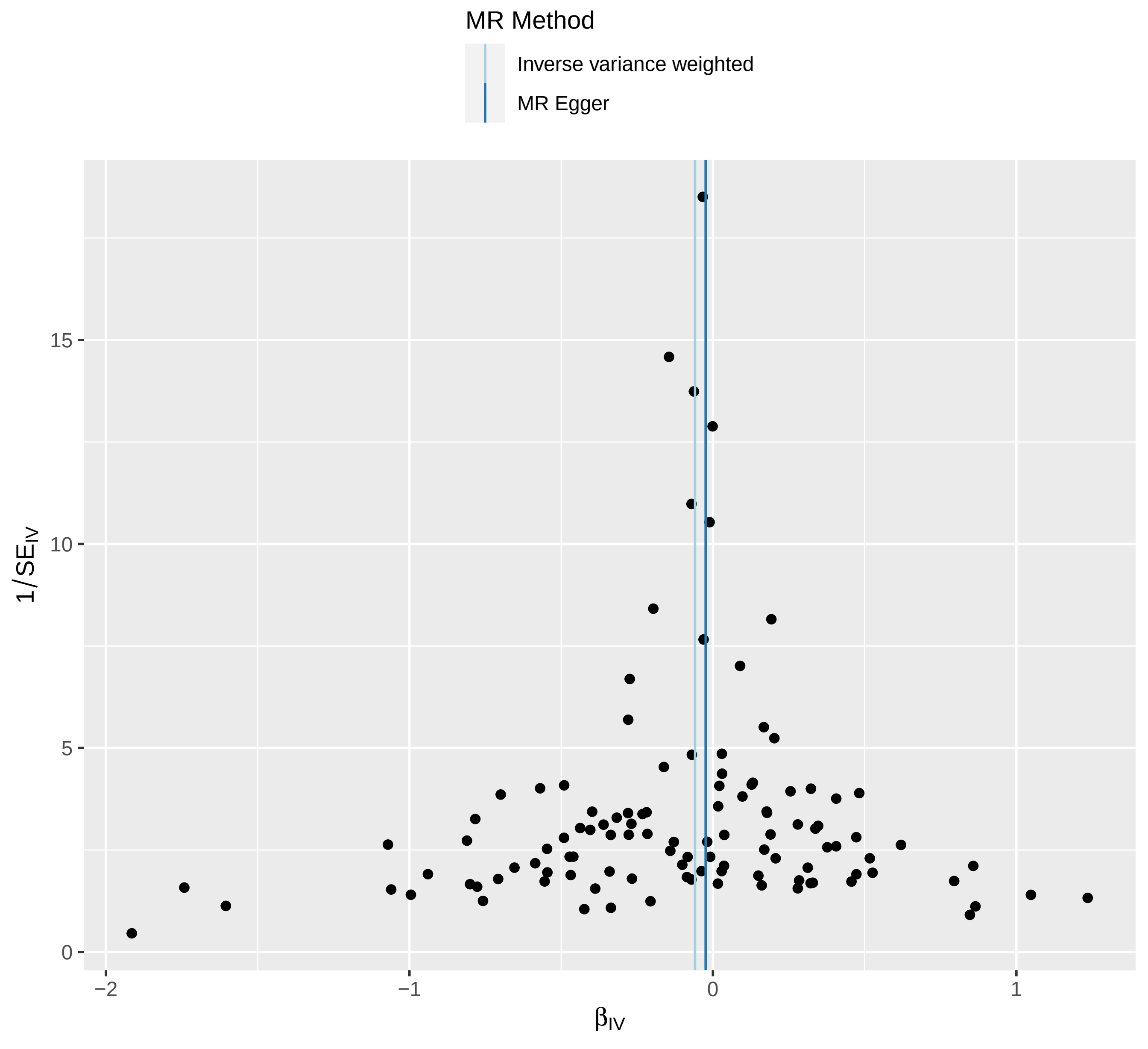

MR Method
Inverse variance weighted
MR Egger
15
10
5
0
1/SE_IV
−2
−1
0
1
β_IV

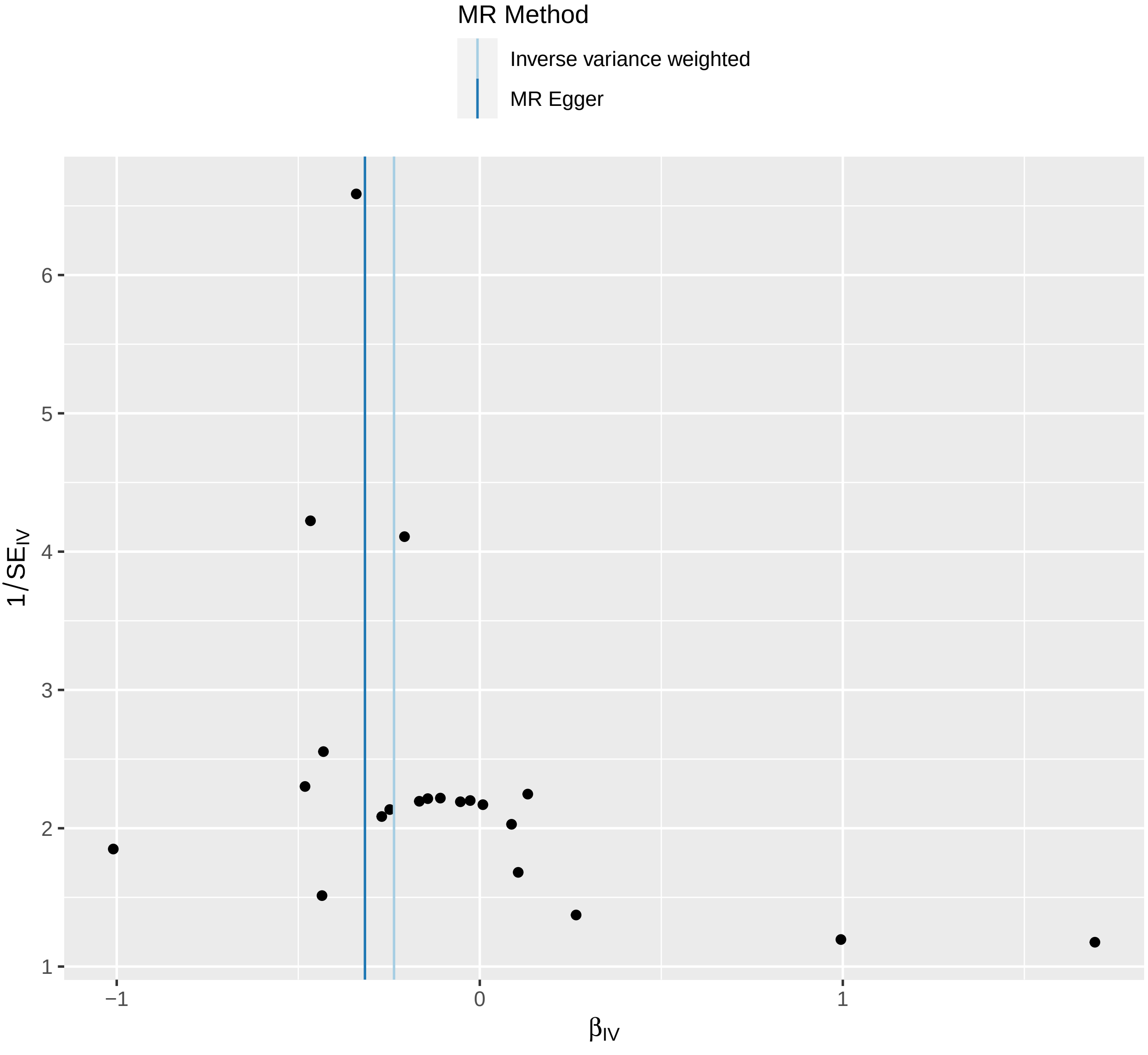
MR Method
Inverse variance weighted
MR Egger
1/SE_IV
6
5
4
3
2
1
−1
0
1
β_IV

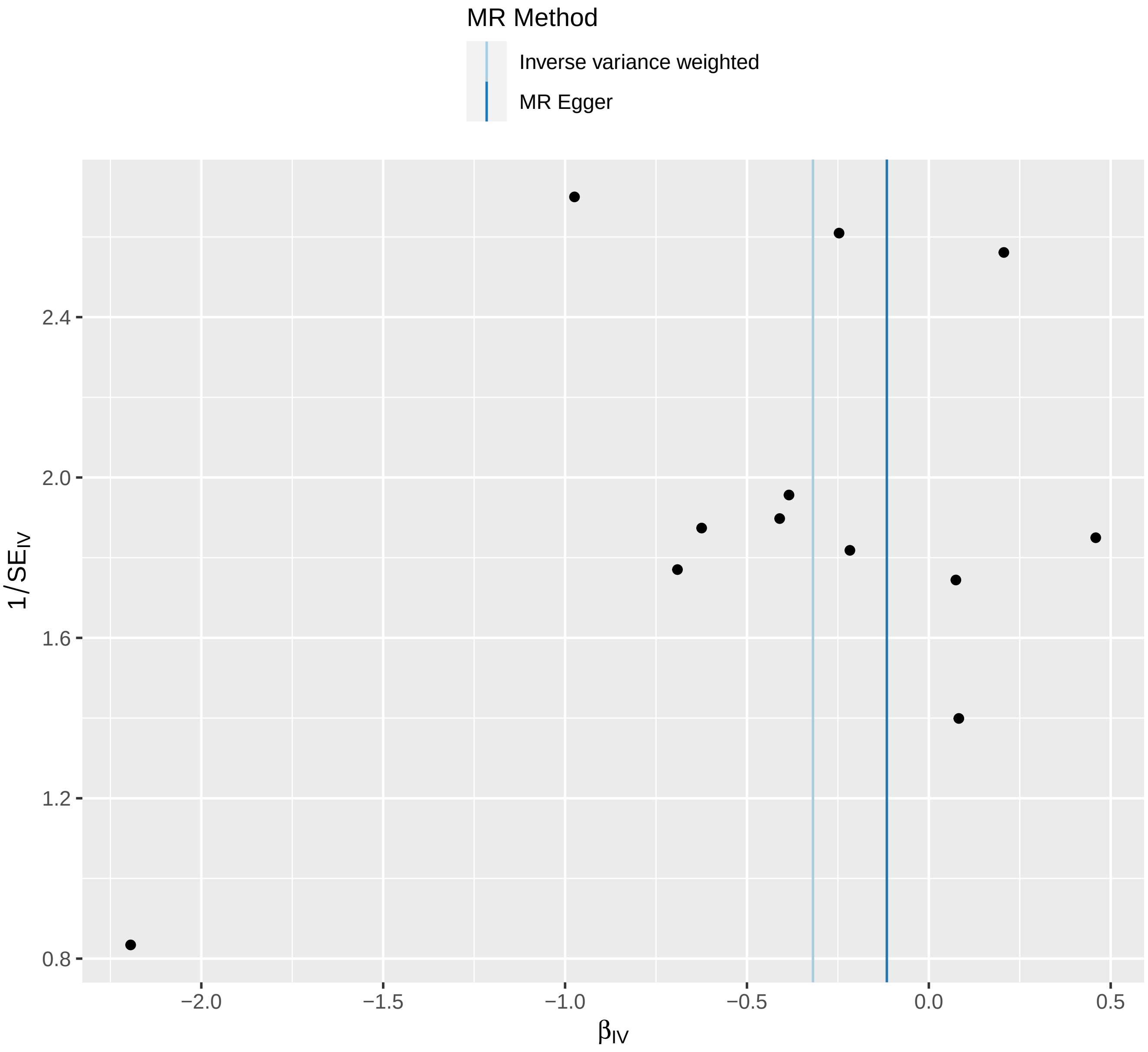
MR Method
Inverse variance weighted
MR Egger
2.4
2.0
1.6
1.2
0.8
1/SE_IV
−2.0
−1.5
−1.0
−0.5
0.0
0.5
β_IV

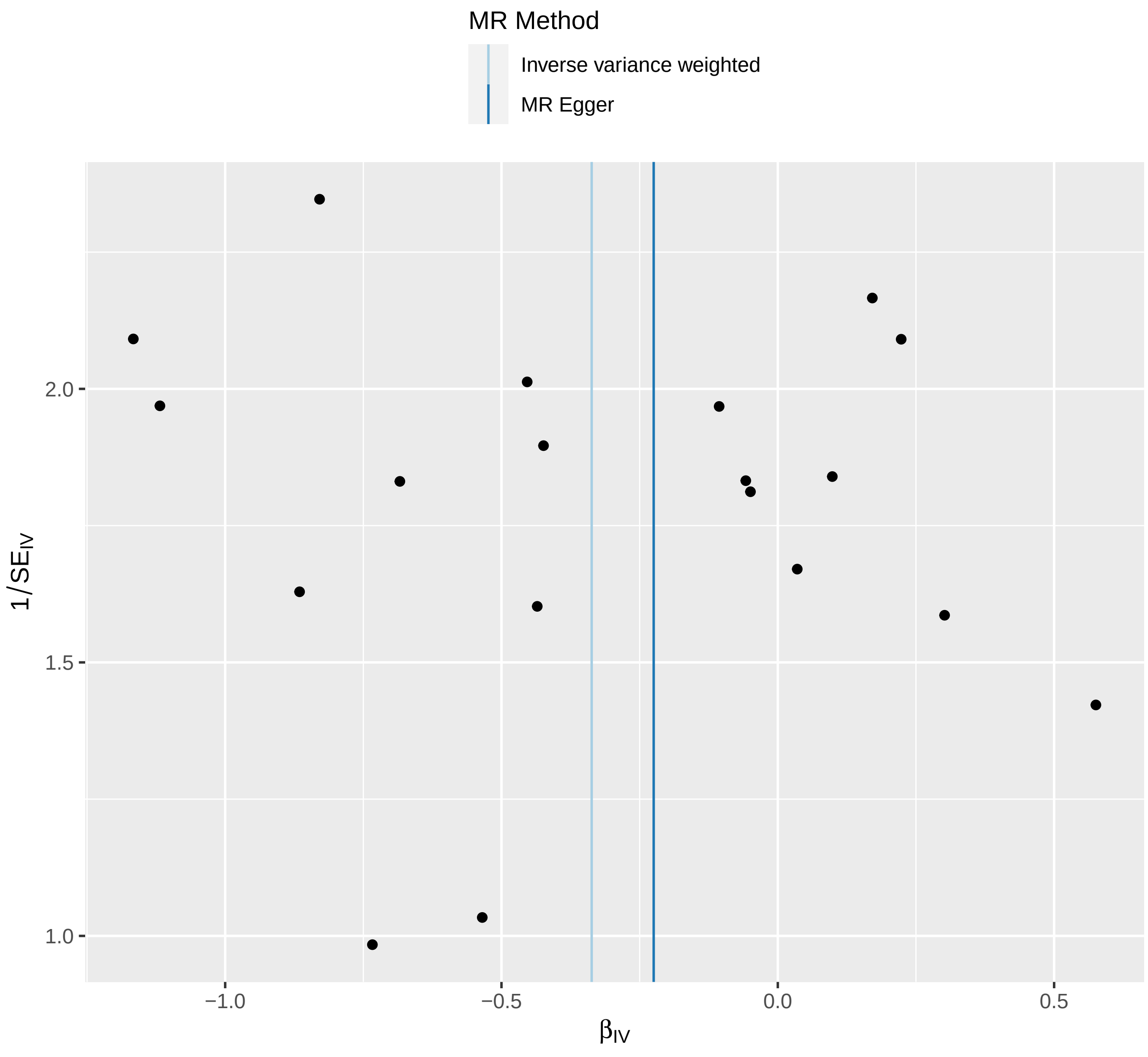

MR Method
Inverse variance weighted
MR Egger
2.0
1.5
1.0
1/SE$_{IV}$
−1.0
−0.5
0.0
0.5
β$_{IV}$

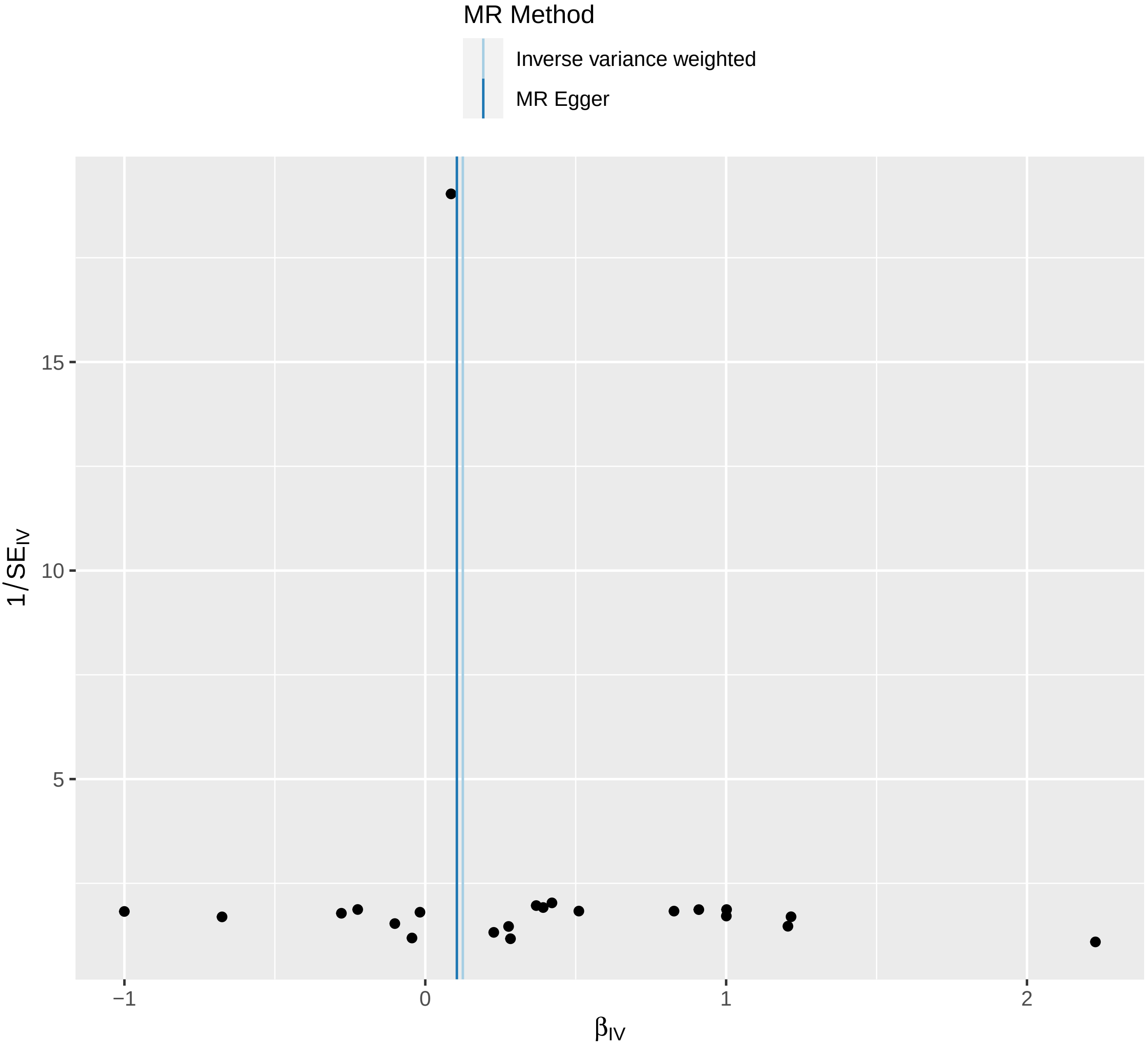

MR Method
Inverse variance weighted
MR Egger
15
10
5
1/SE$_{IV}$
−1
0
1
2
β$_{IV}$

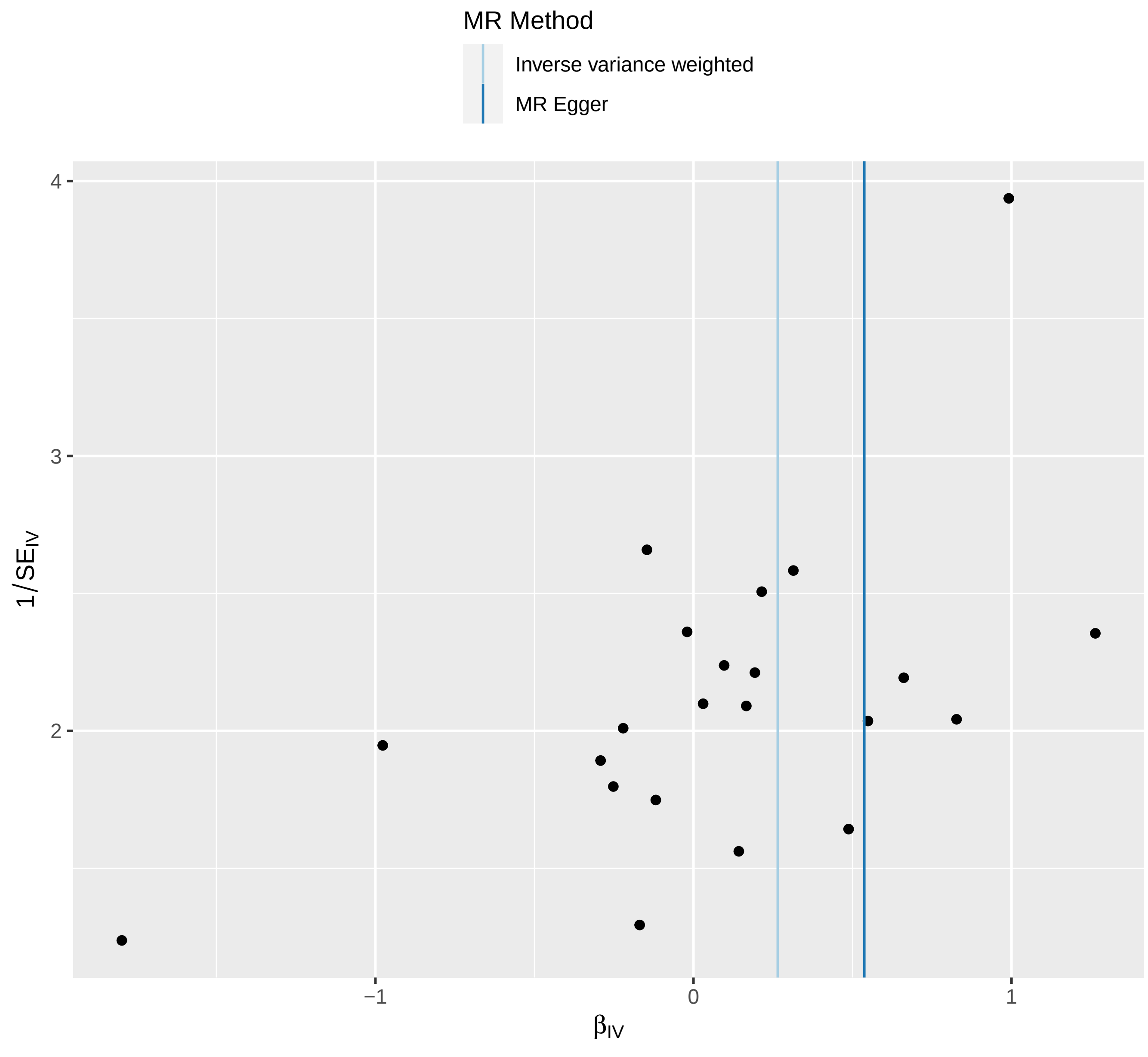

MR Method
Inverse variance weighted
MR Egger
4
3
2
1/SE$_{IV}$
−1
0
1
β$_{IV}$

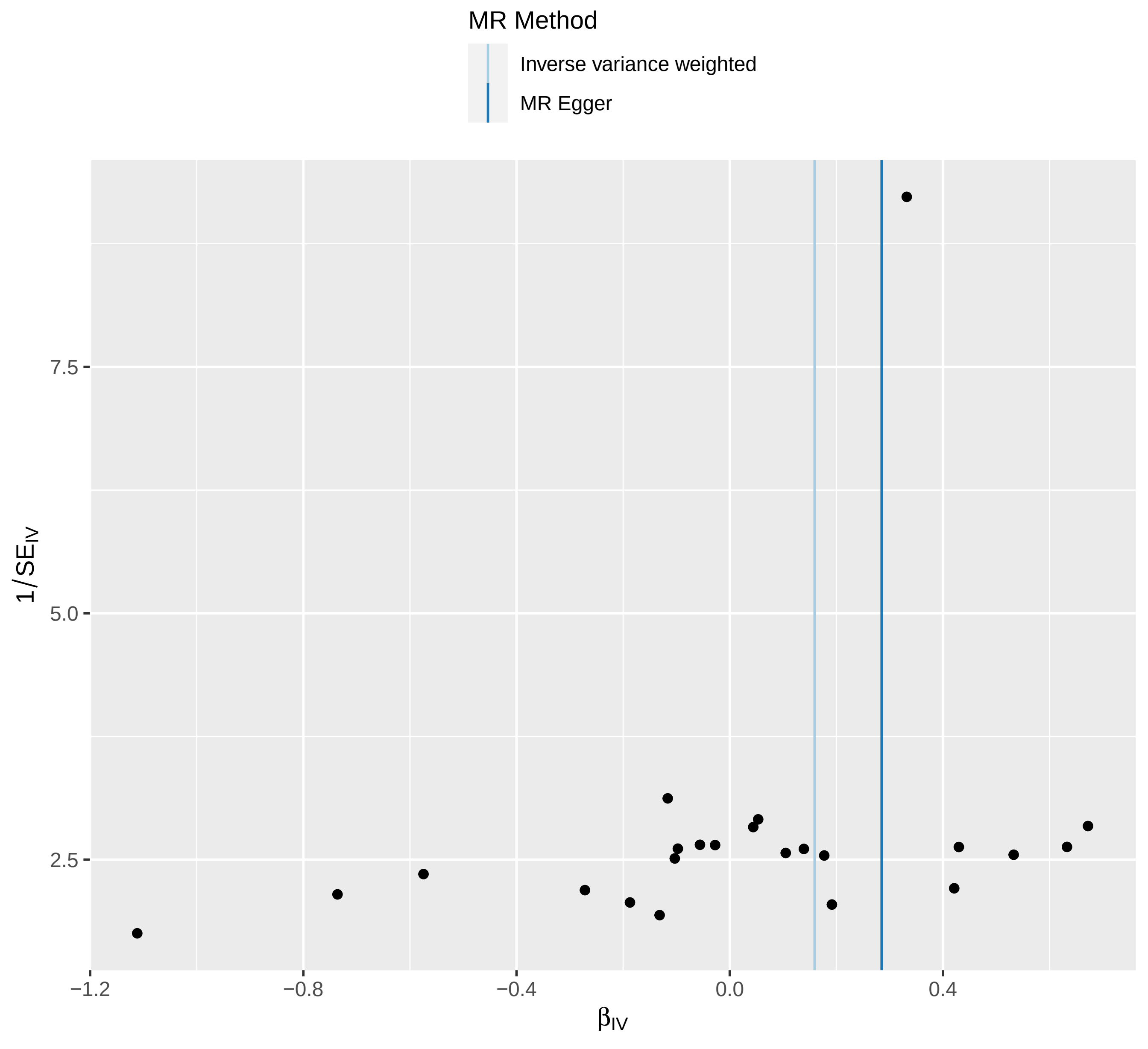
MR Method
Inverse variance weighted
MR Egger
7.5
5.0
2.5
1/SE$_{IV}$
−1.2
−0.8
−0.4
0.0
0.4
β$_{IV}$

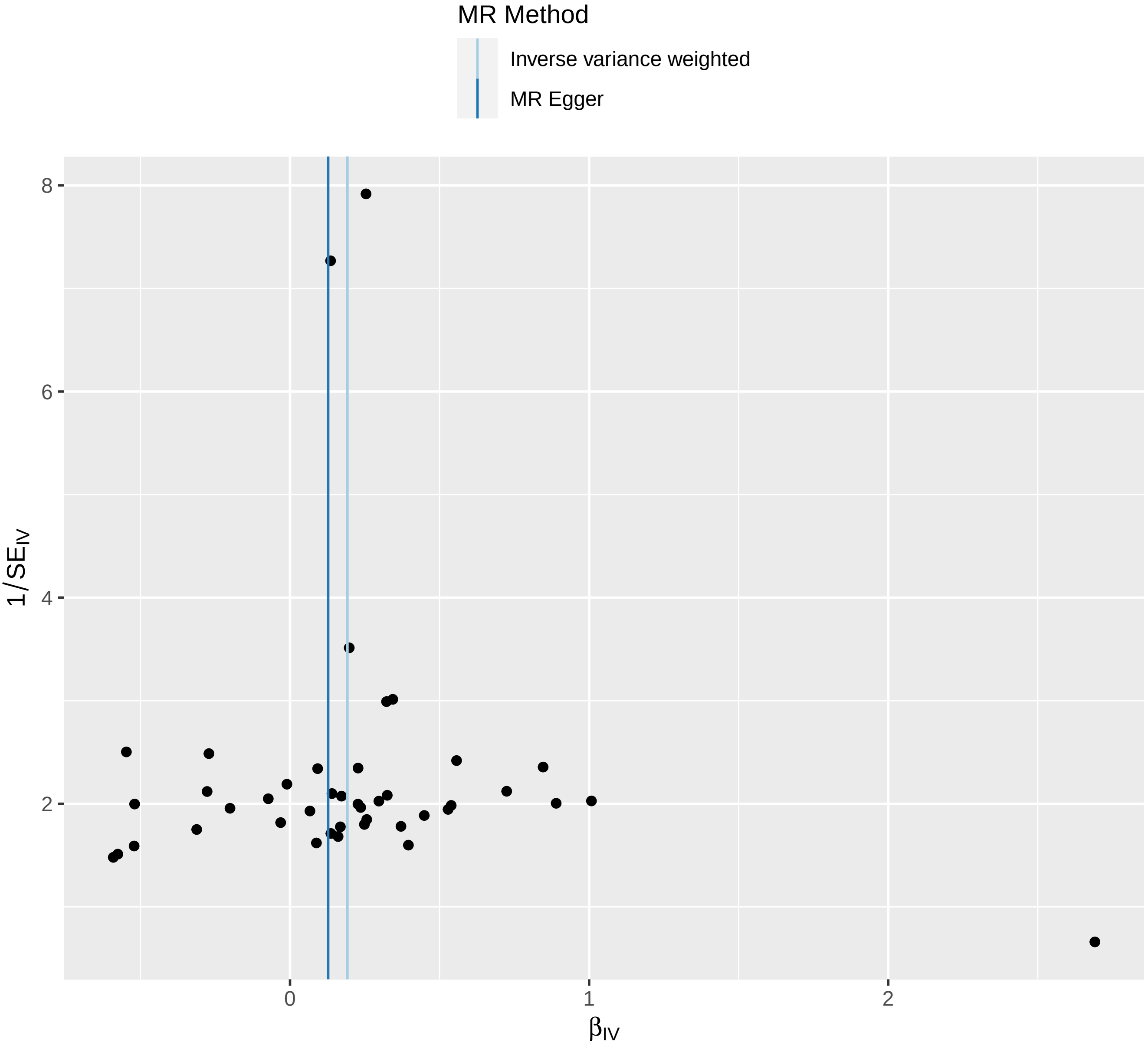
MR Method
Inverse variance weighted
MR Egger
8
6
4
2
1/SE$_{IV}$
0
1
2
β$_{IV}$

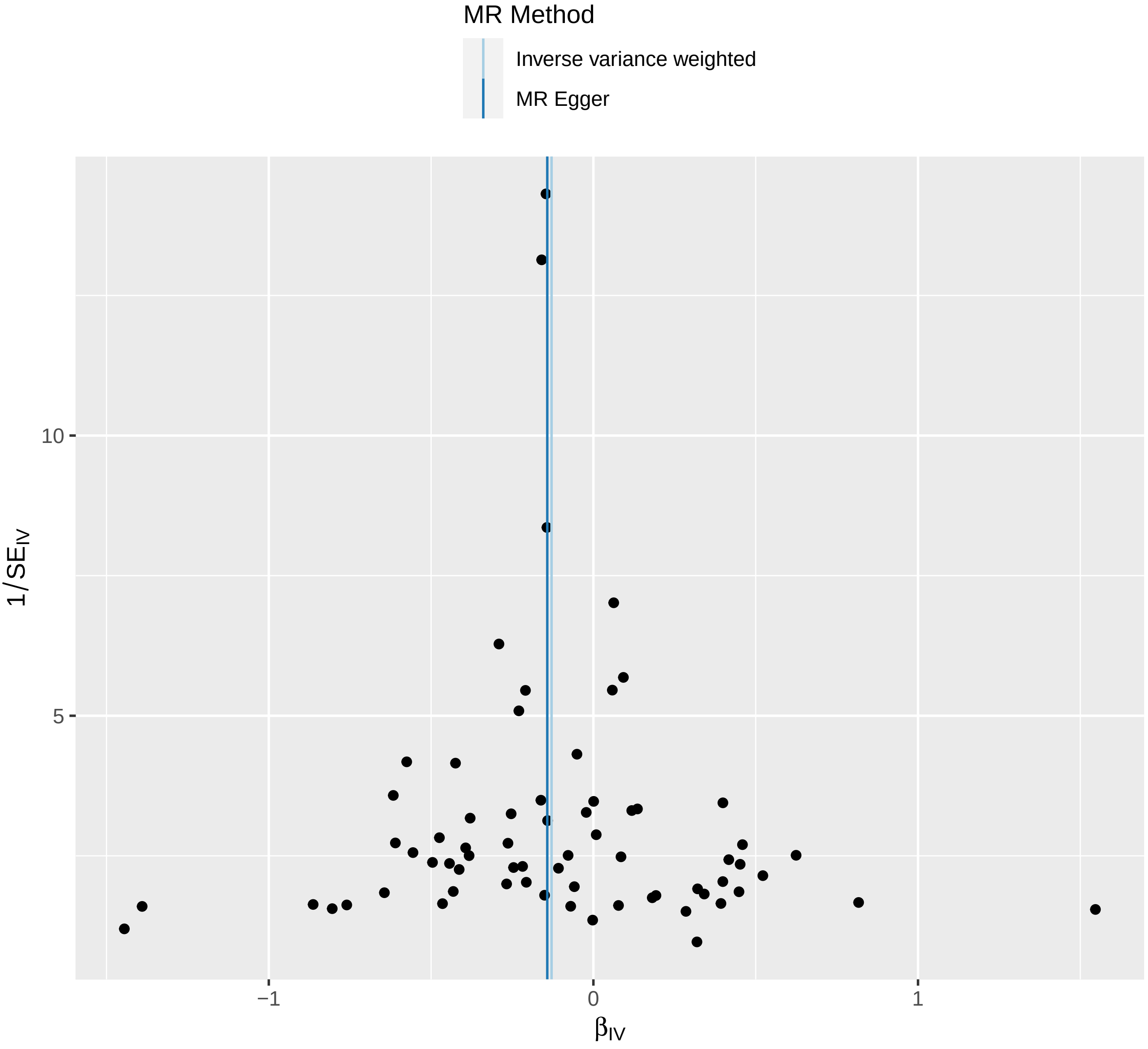

MR Method
Inverse variance weighted
MR Egger
10
5
1/SE_IV
−1
0
1
β_IV

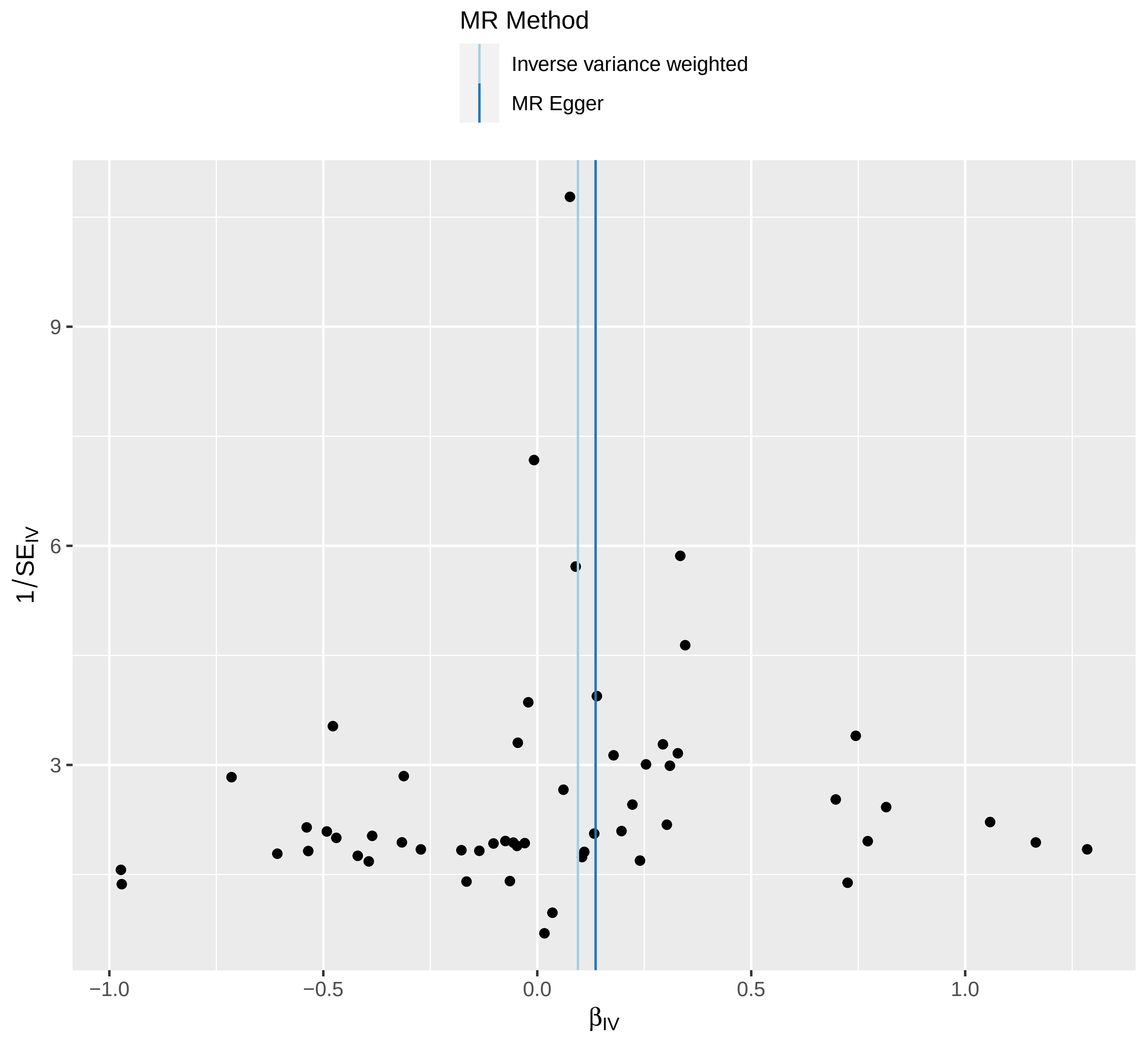
MR Method
Inverse variance weighted
MR Egger
9
6
3
$1/SE_{IV}$
−1.0
−0.5
0.0
0.5
1.0
β_{IV}

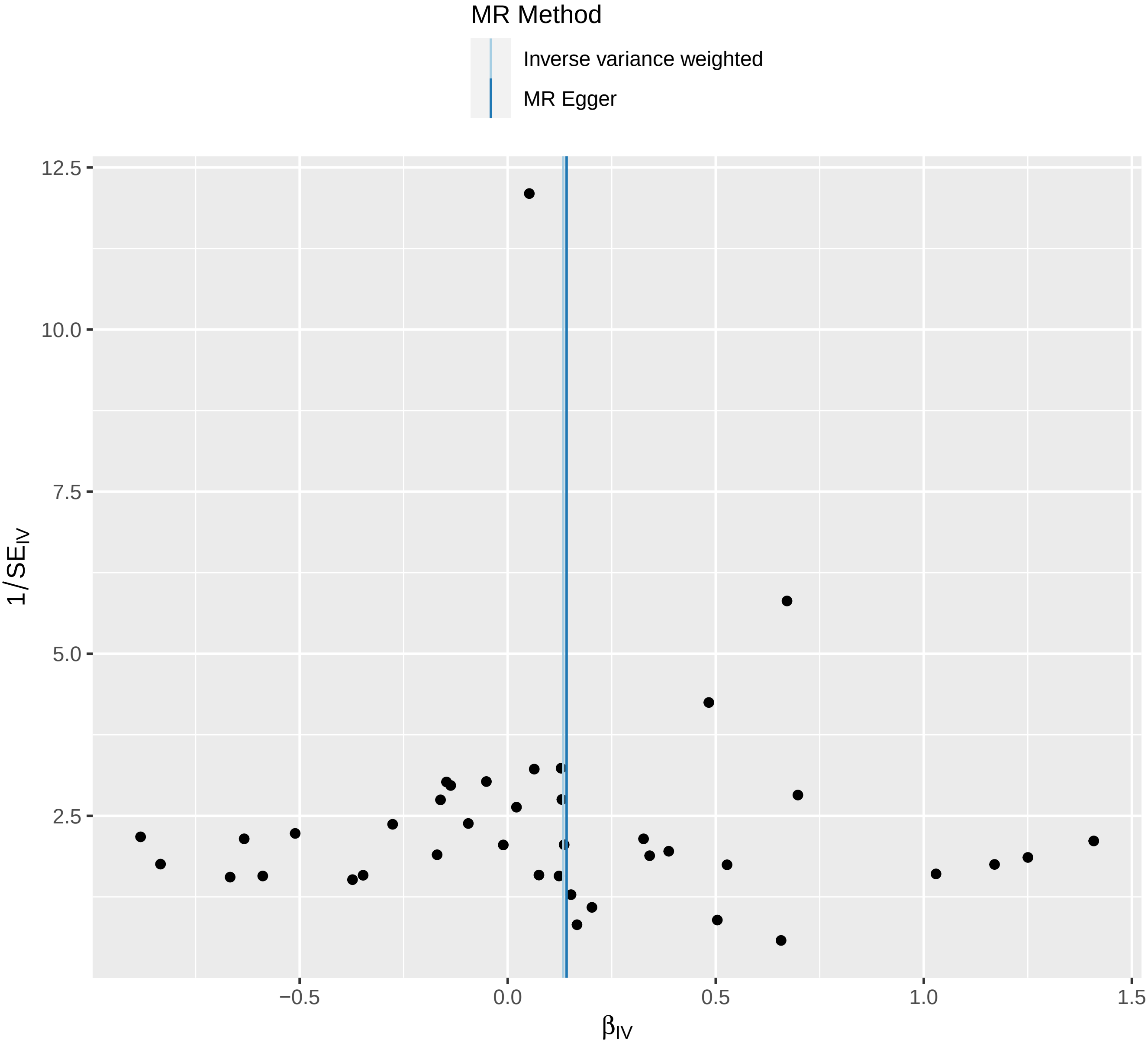
MR Method
Inverse variance weighted
MR Egger
12.5
10.0
7.5
5.0
2.5
1/SE_IV
−0.5
0.0
0.5
1.0
1.5
β_IV

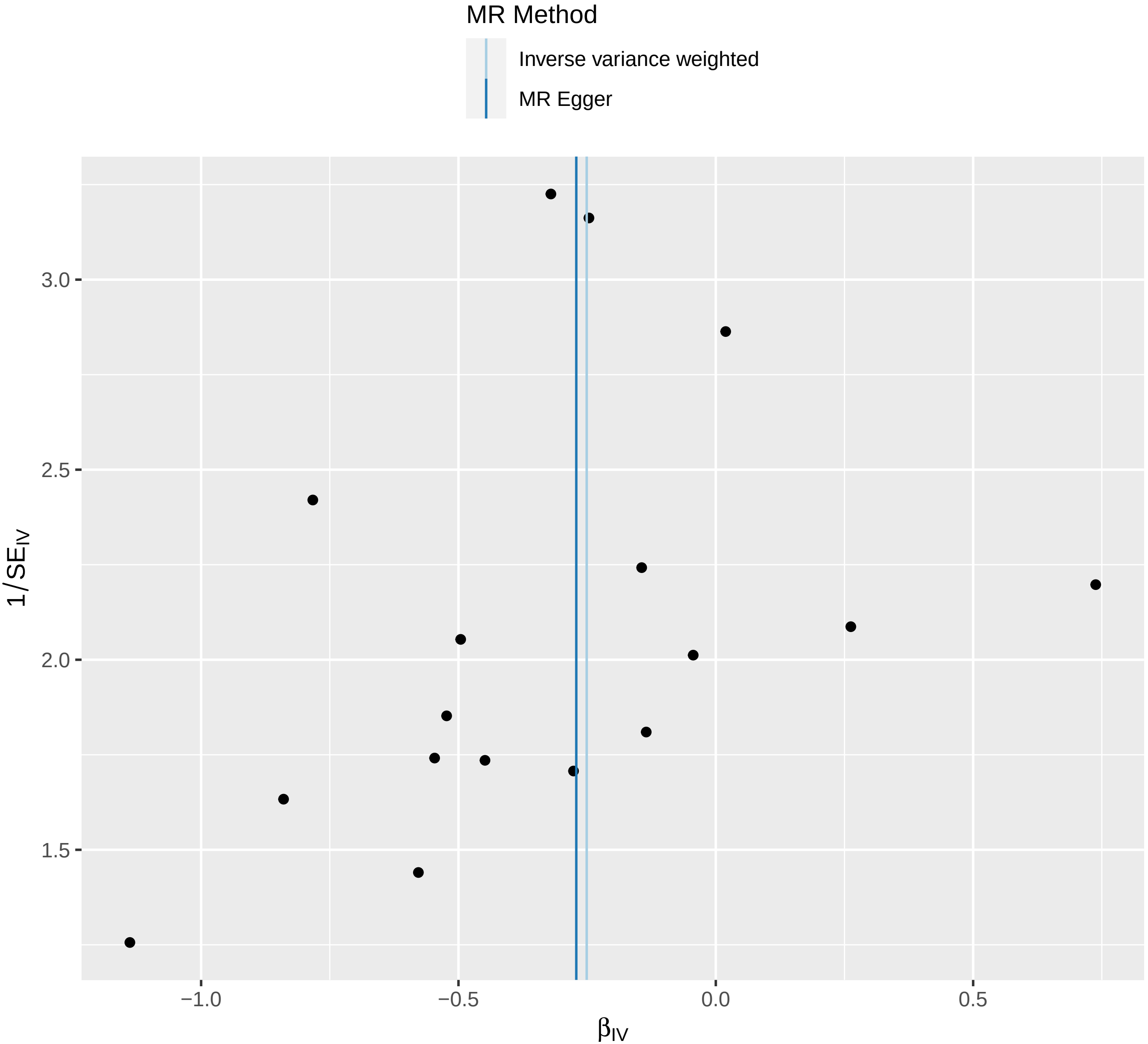
MR Method
Inverse variance weighted
MR Egger
3.0
2.5
2.0
1.5
$1/SE_{IV}$
−1.0
−0.5
0.0
0.5
β_{IV}

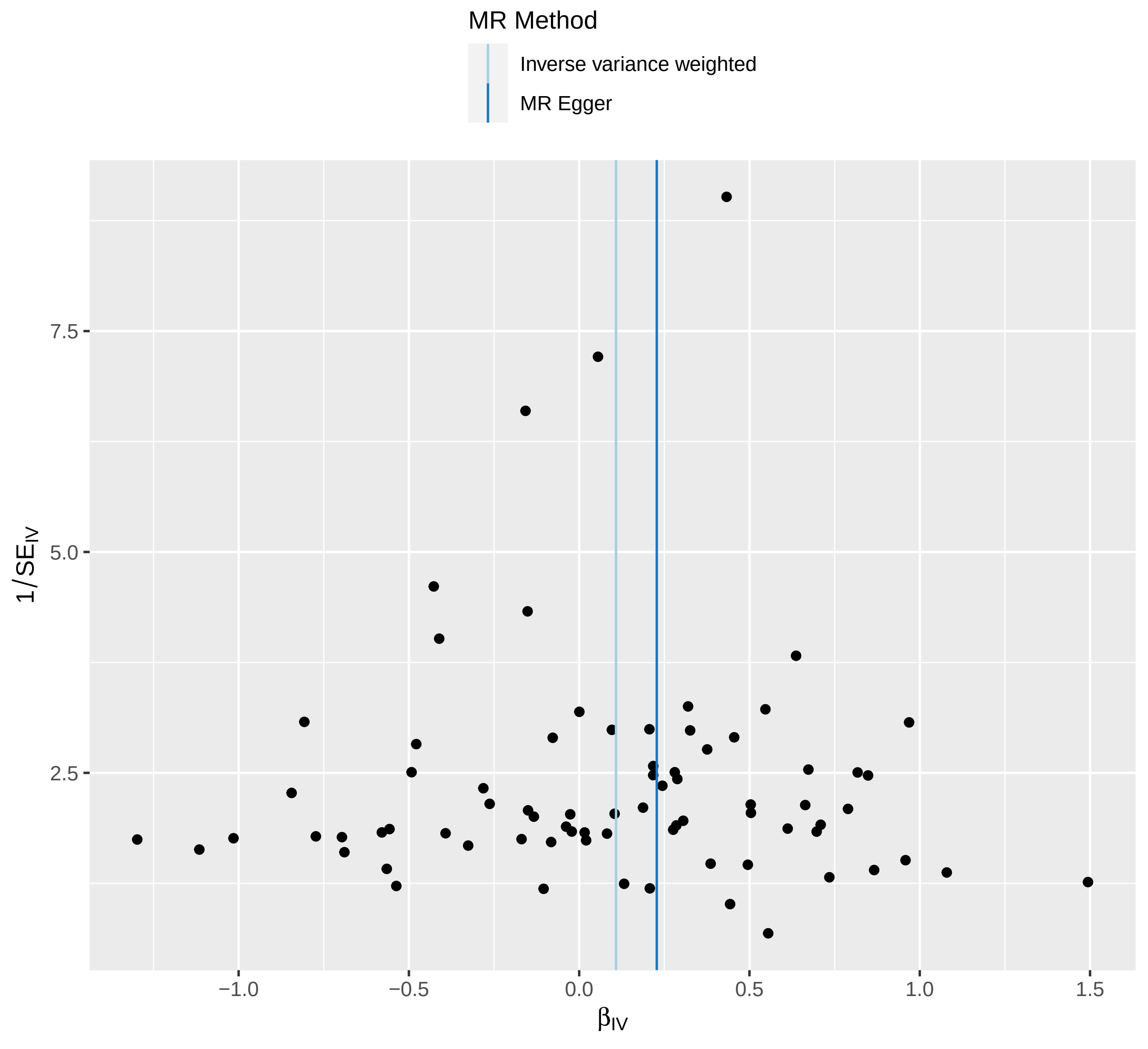
MR Method
Inverse variance weighted
MR Egger
7.5
5.0
2.5
1/SE_IV
−1.0
−0.5
0.0
0.5
1.0
1.5
β_IV

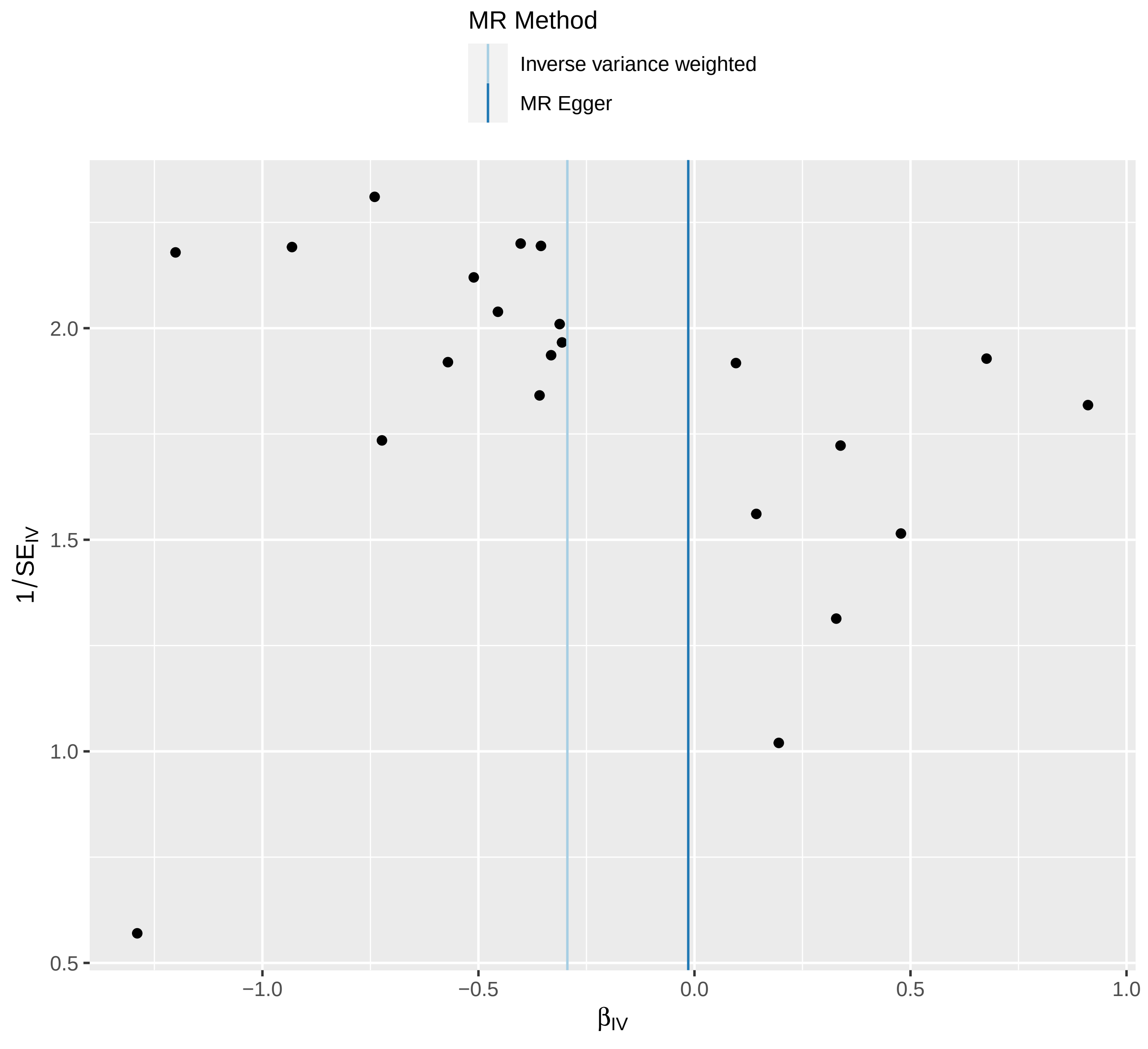
MR Method
Inverse variance weighted
MR Egger
2.0
1.5
1.0
0.5
−1.0
−0.5
0.0
0.5
1.0
1/SE$_{IV}$
β$_{IV}$

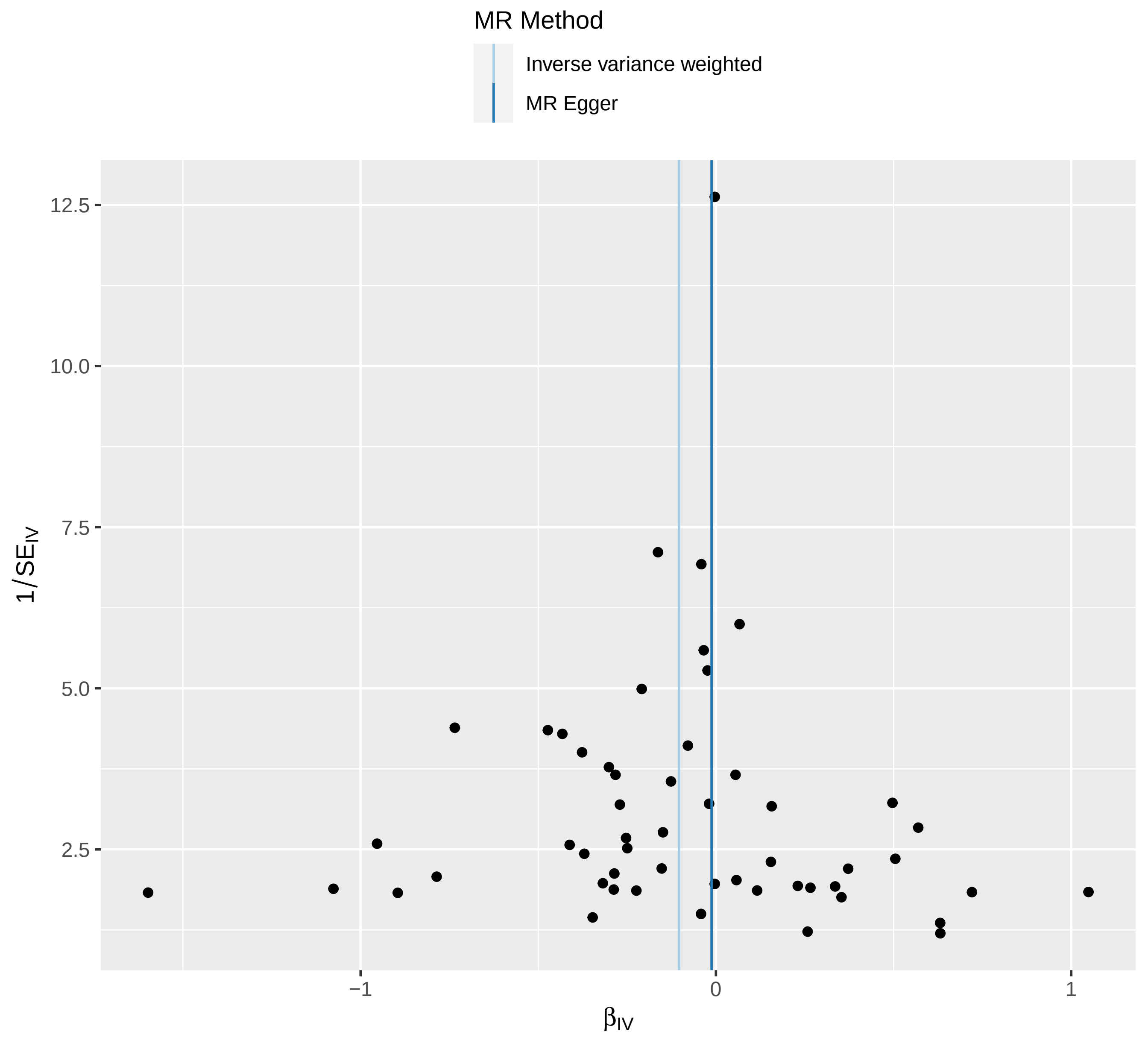
MR Method
Inverse variance weighted
MR Egger
12.5
10.0
7.5
5.0
2.5
$1/SE_{IV}$
−1
0
1
β_{IV}

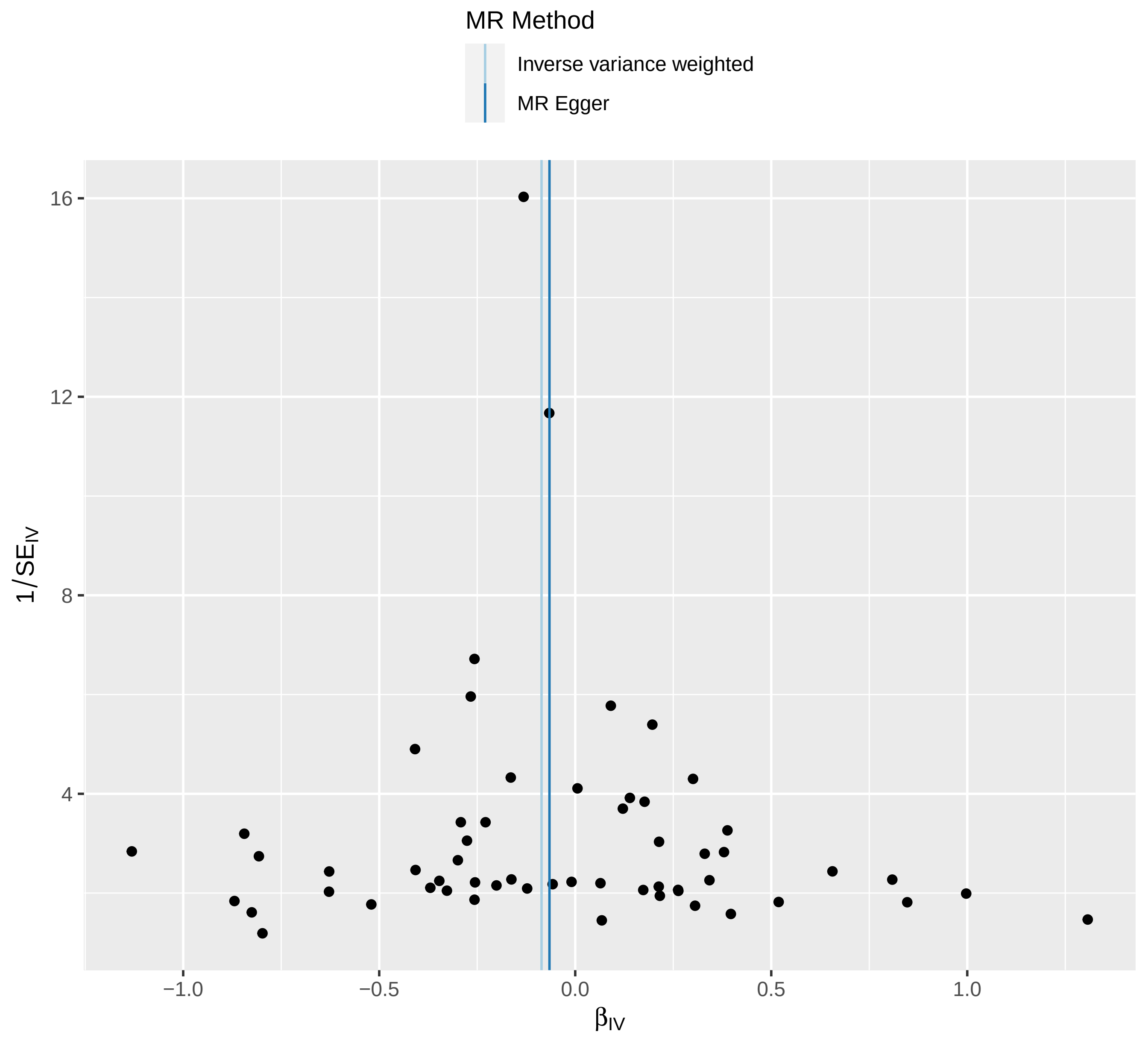
MR Method
Inverse variance weighted
MR Egger
16
12
8
4
1/SE_IV
−1.0
−0.5
0.0
0.5
1.0
β_IV

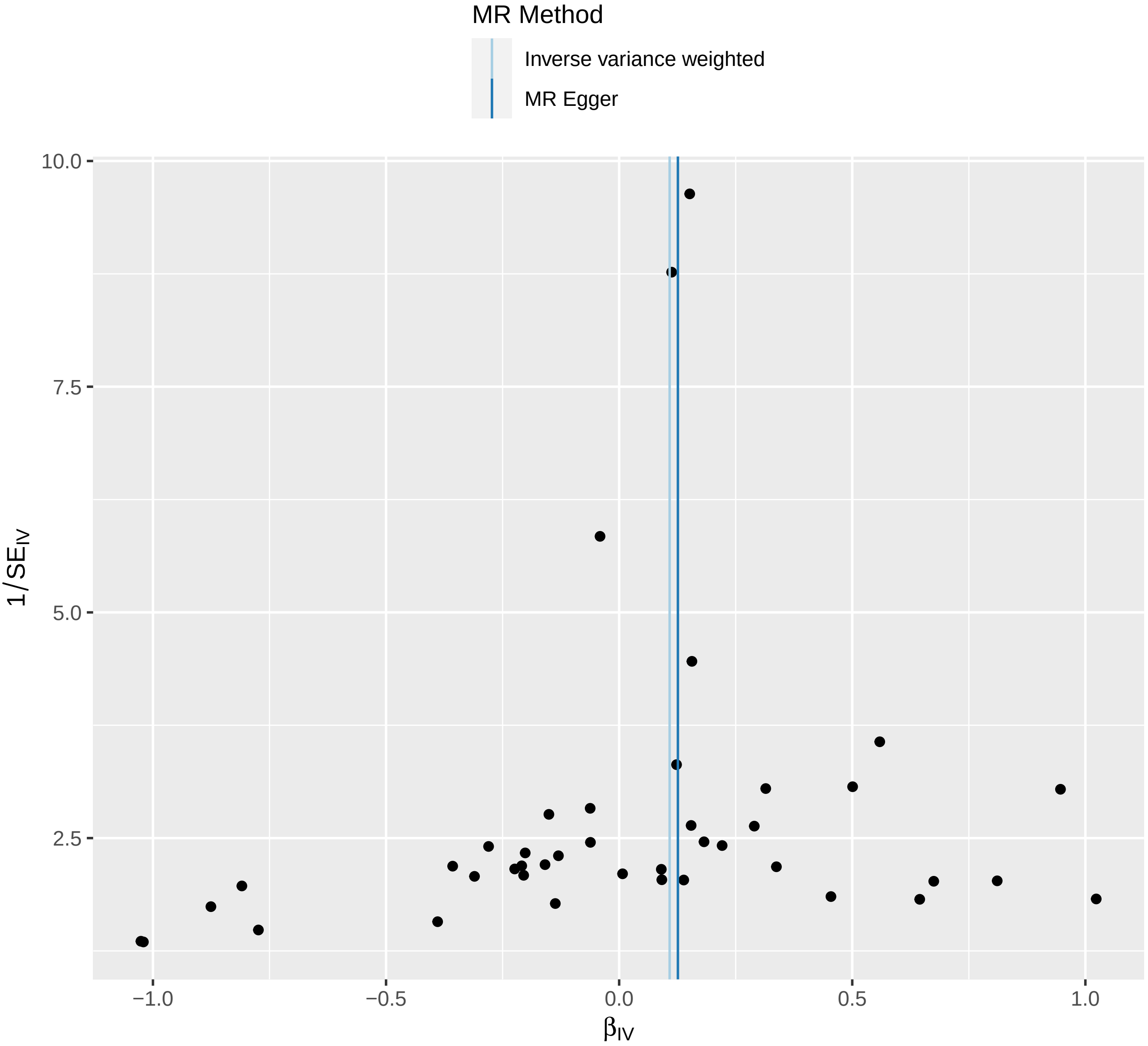
MR Method
Inverse variance weighted
MR Egger
10.0
7.5
5.0
2.5
1/SE_IV
−1.0
−0.5
0.0
0.5
1.0
β_IV

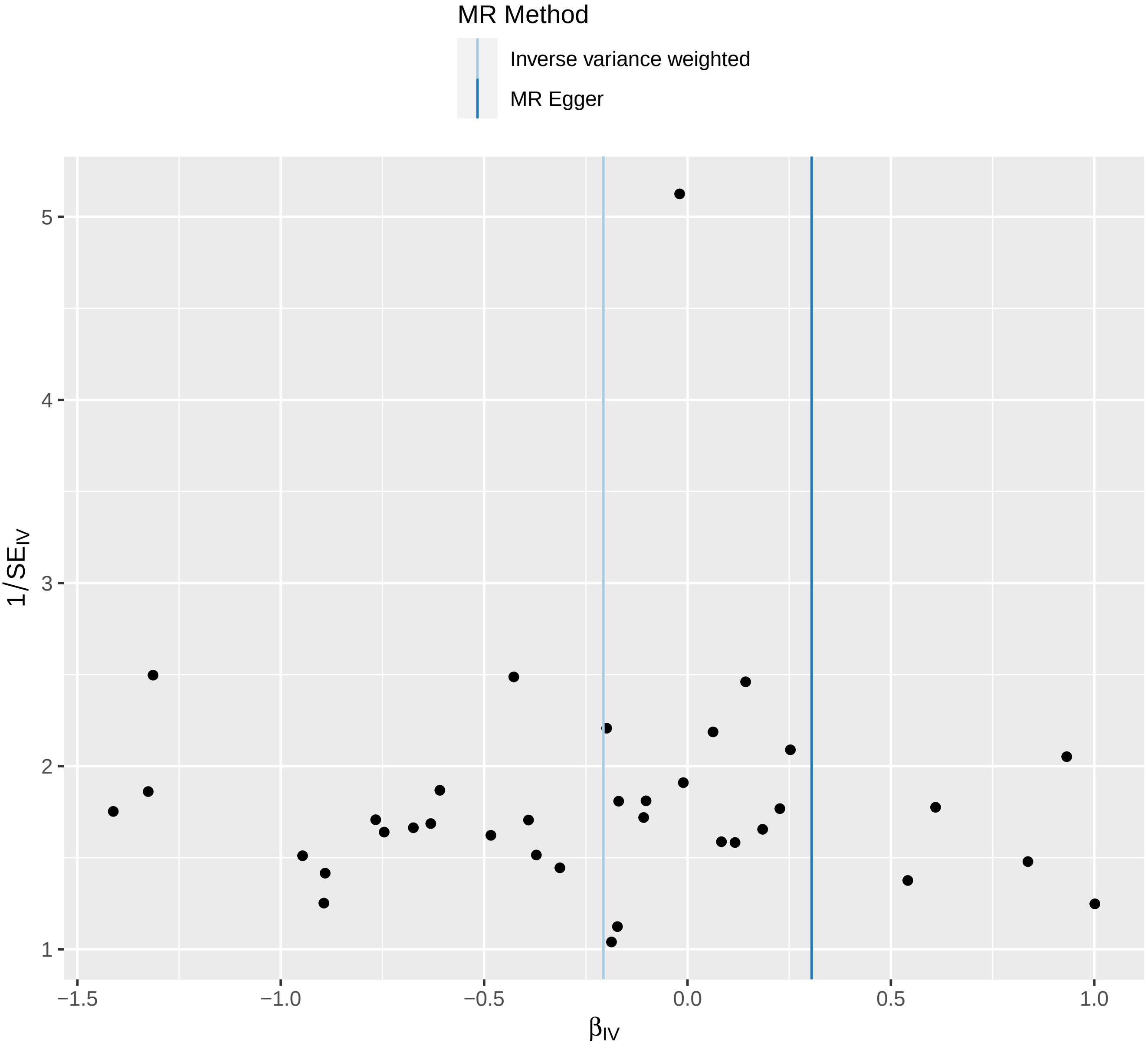

MR Method
Inverse variance weighted
MR Egger
1/SE_IV
β_IV
5
4
3
2
1
−1.5
−1.0
−0.5
0.0
0.5
1.0

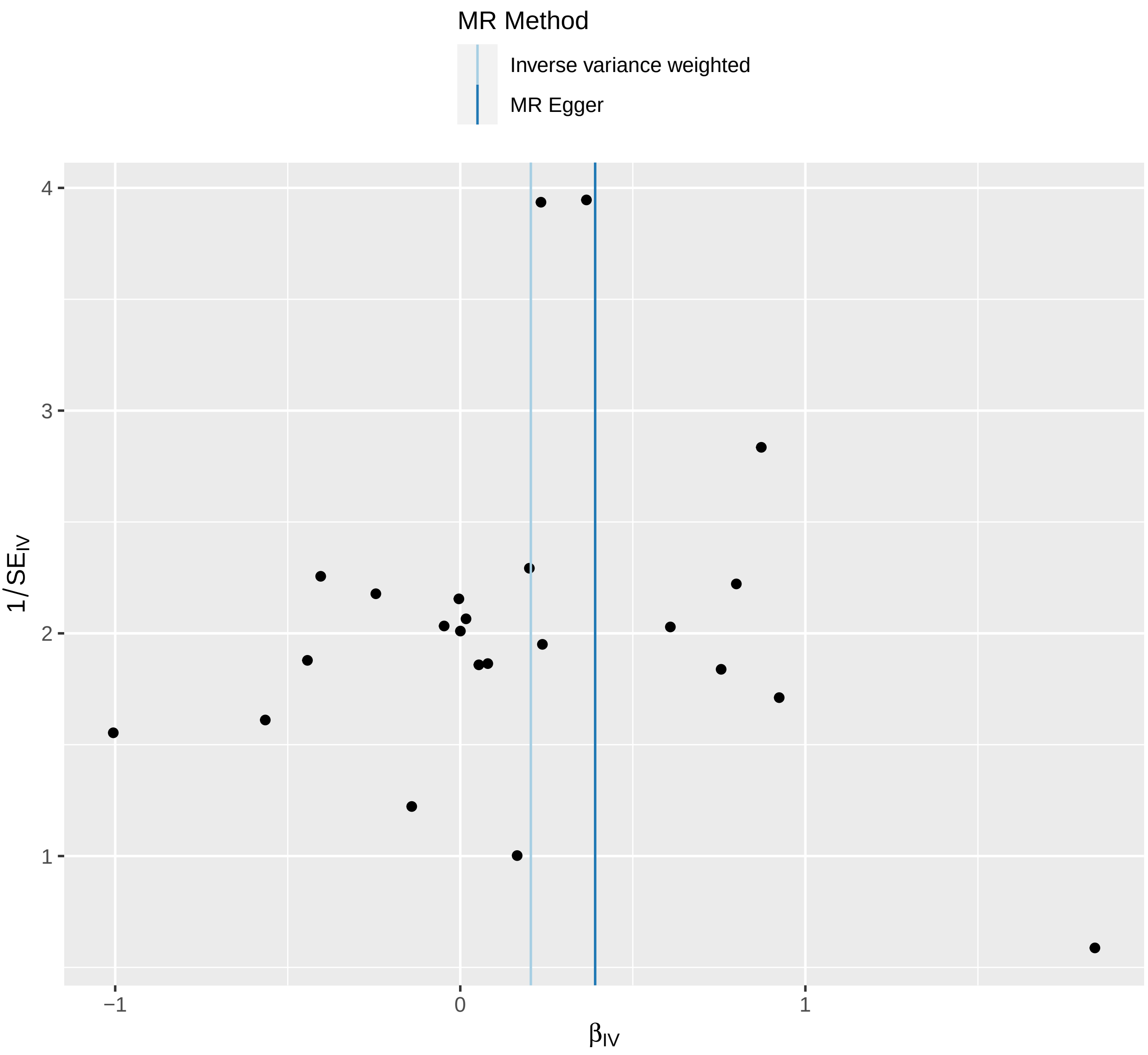
MR Method
Inverse variance weighted
MR Egger
4
3
2
1
−1
0
1
$1/SE_{IV}$
β_{IV}

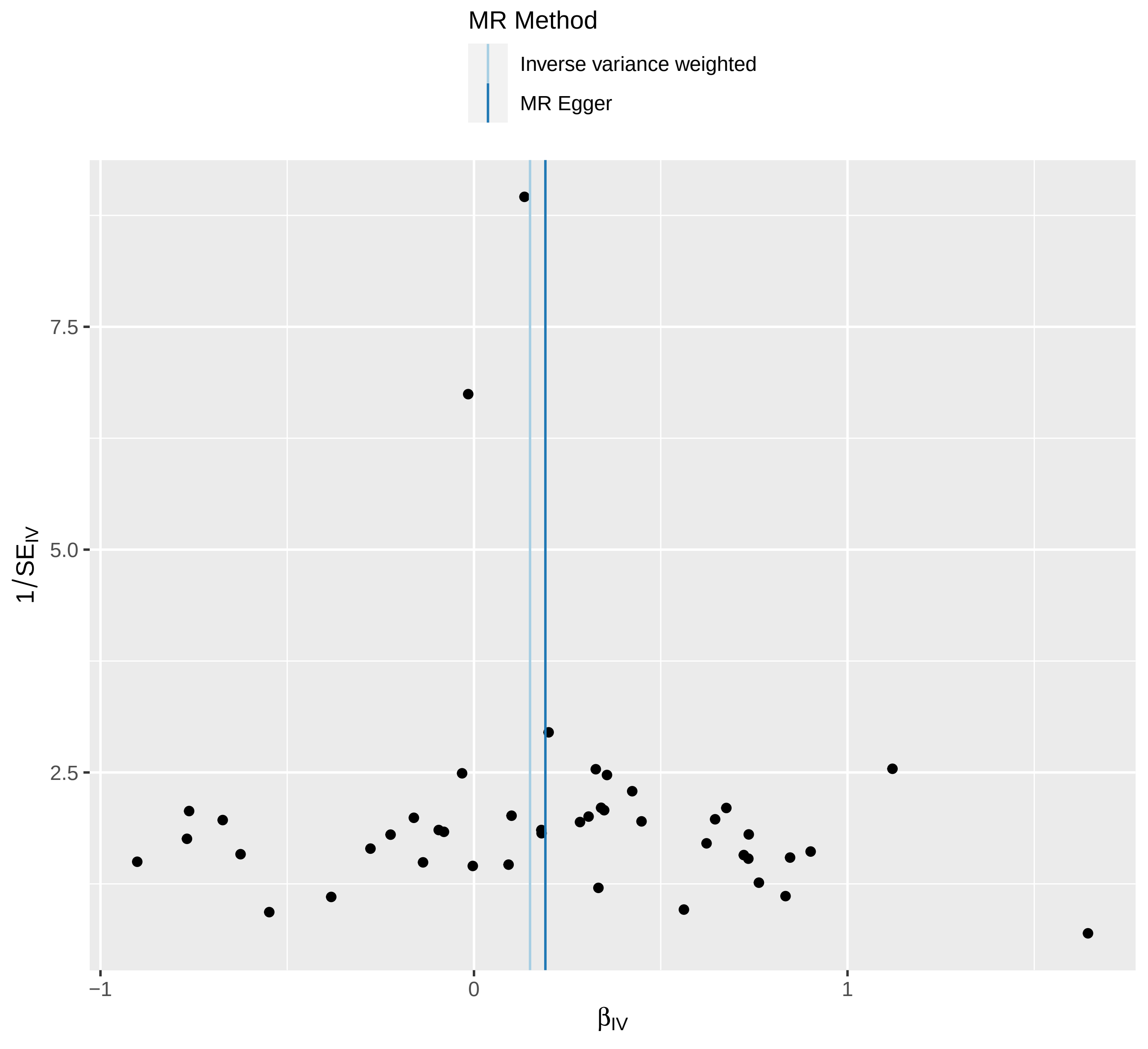
MR Method
Inverse variance weighted
MR Egger
7.5
5.0
2.5
1/SE$_{IV}$
−1
0
1
β$_{IV}$

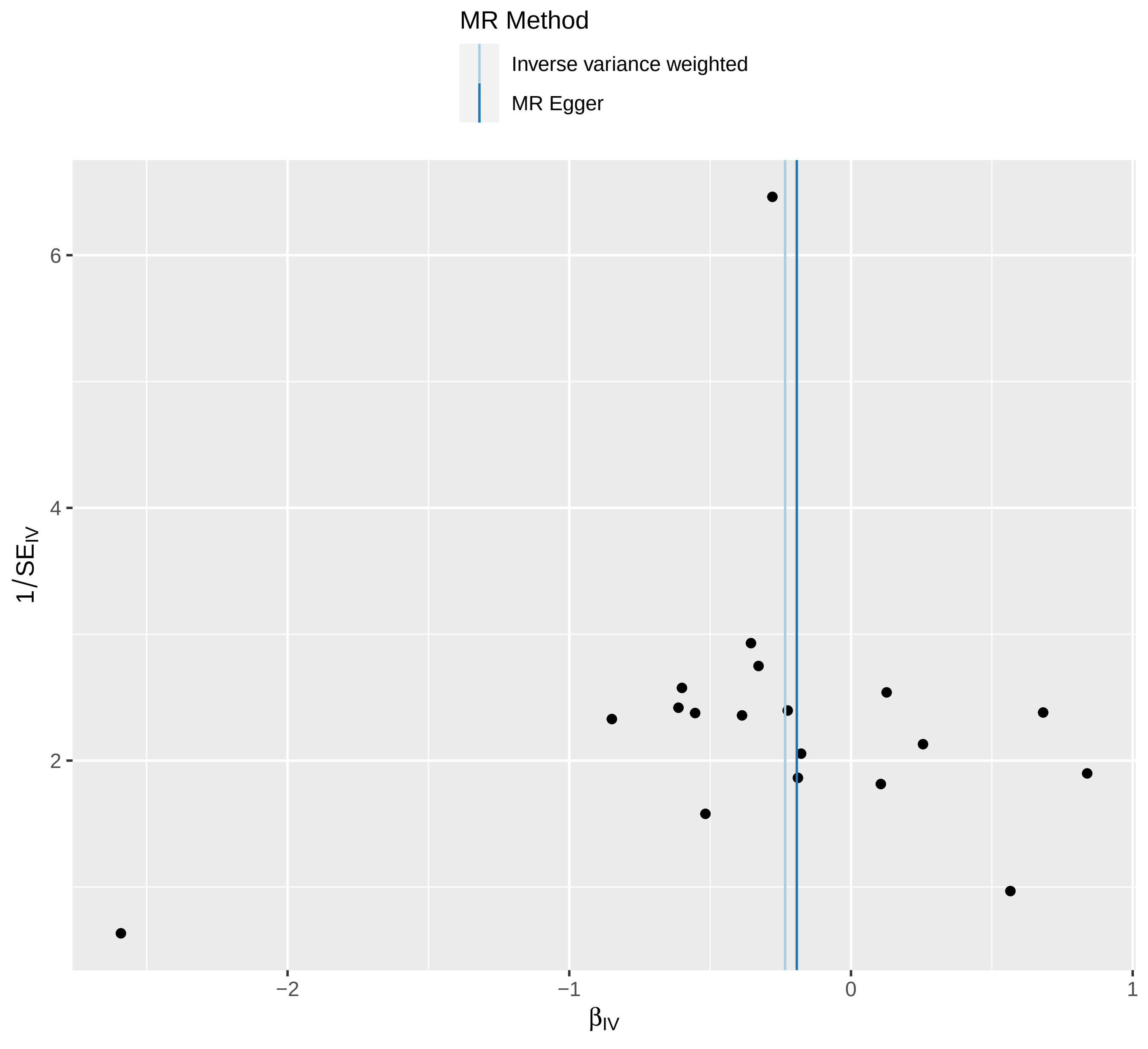

MR Method
Inverse variance weighted
MR Egger
6
4
2
1/SE_IV
−2
−1
0
1
β_IV

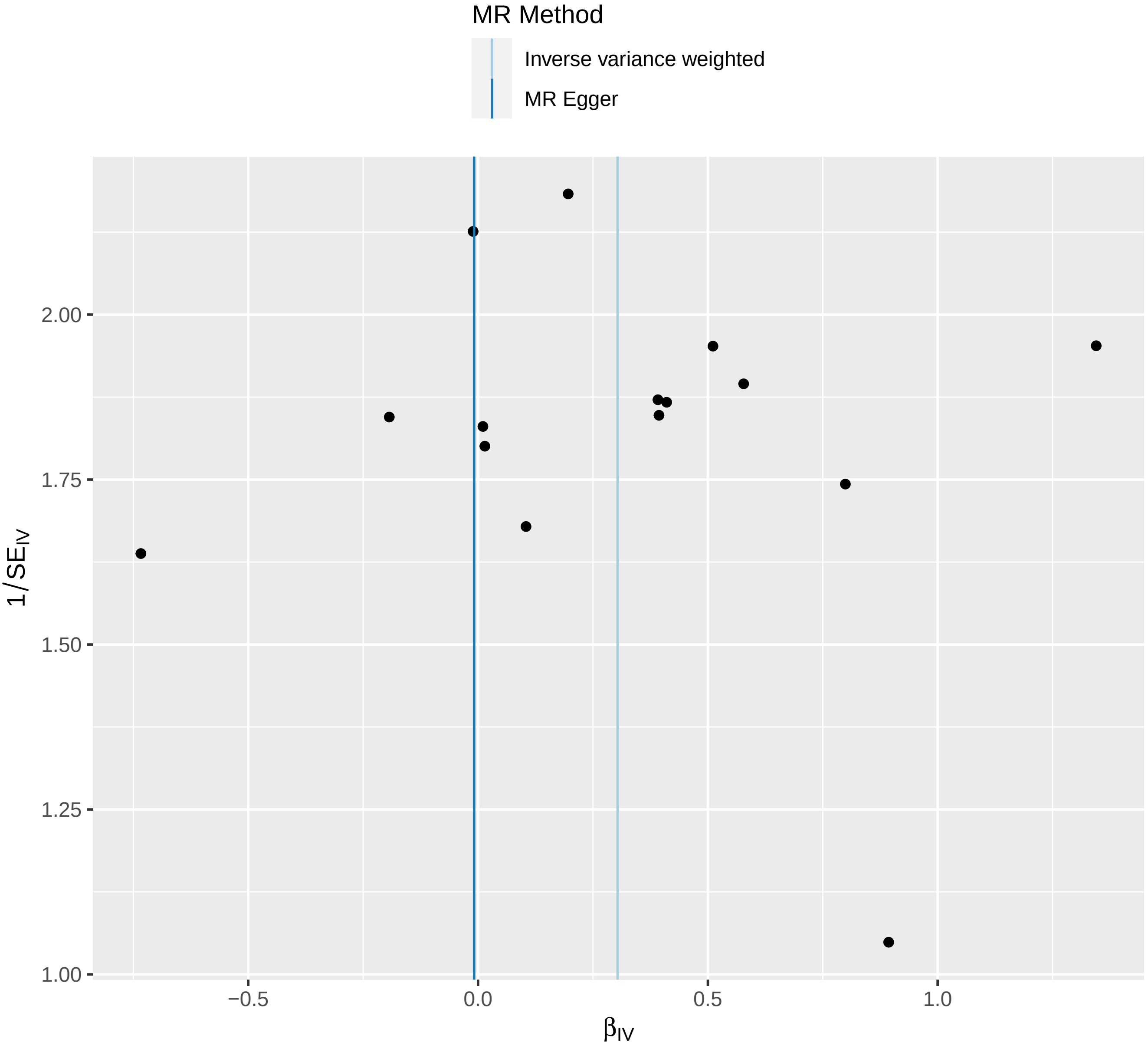

MR Method
Inverse variance weighted
MR Egger
2.00
1.75
1.50
1.25
1.00
1/SE_IV
−0.5
0.0
0.5
1.0
β_IV

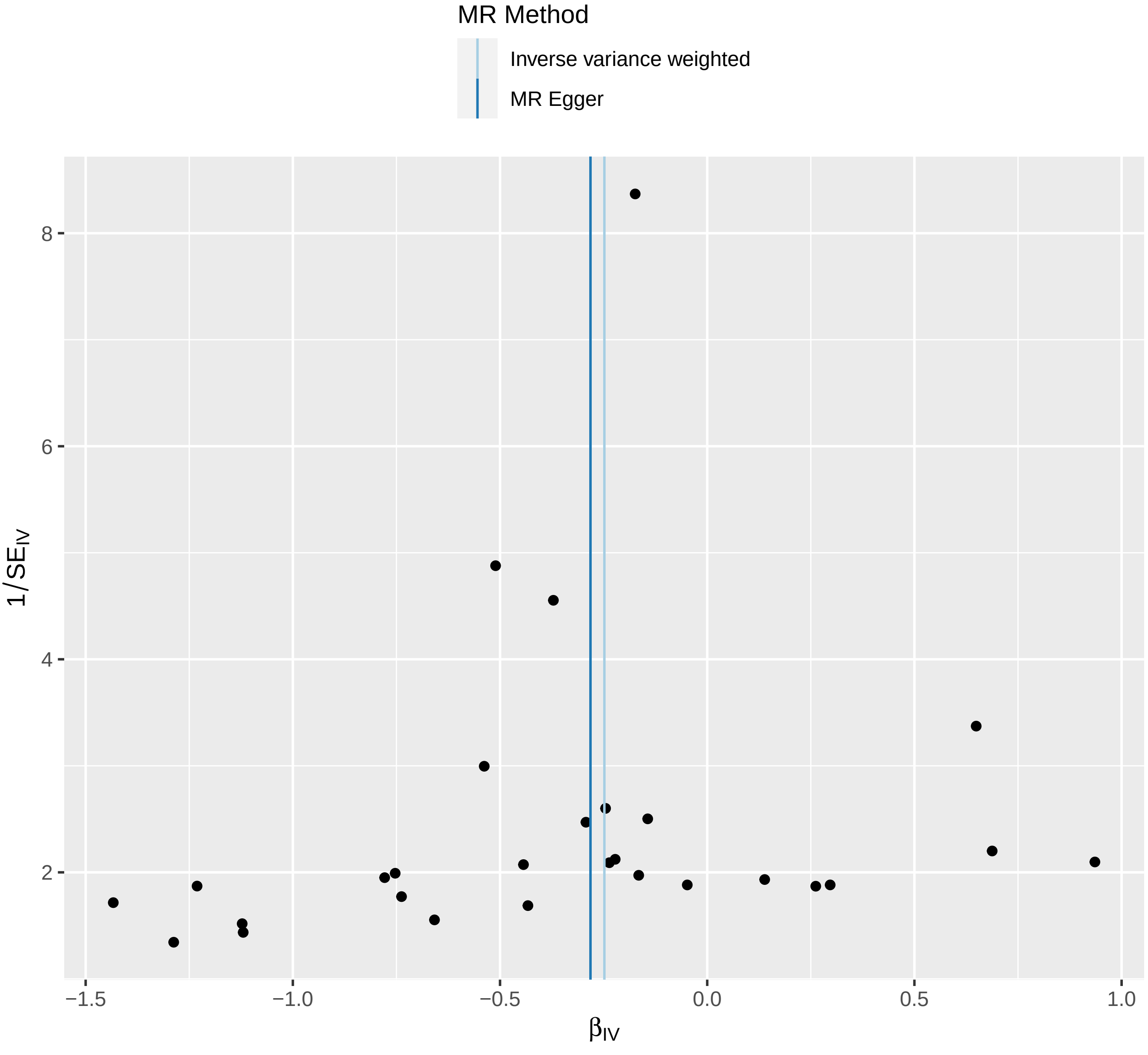

MR Method
Inverse variance weighted
MR Egger
8
6
4
2
$1/SE_{IV}$
−1.5
−1.0
−0.5
0.0
0.5
1.0
β_{IV}

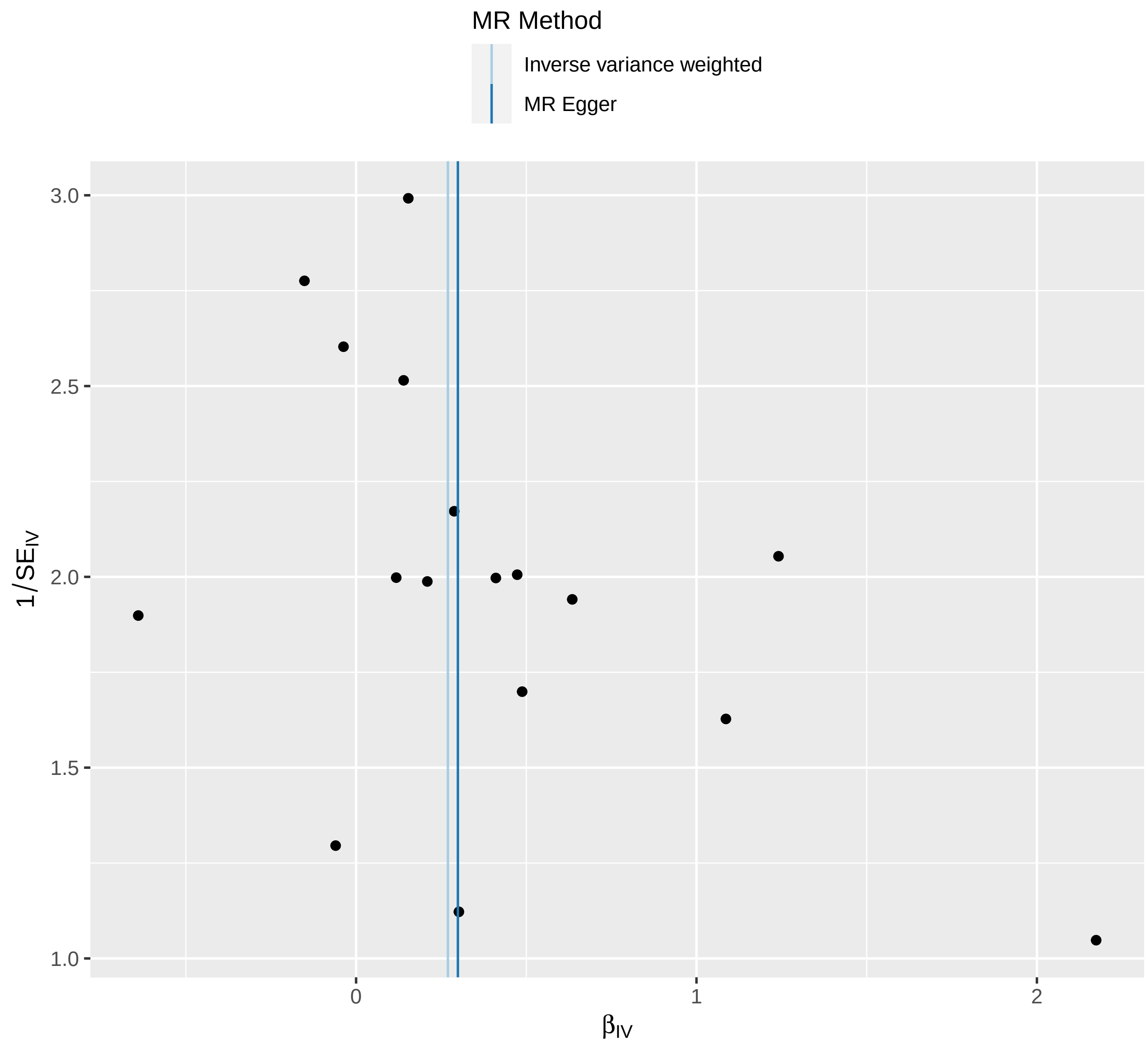

MR Method
Inverse variance weighted
MR Egger
3.0
2.5
2.0
1.5
1.0
1/SE_IV
0
1
2
β_IV

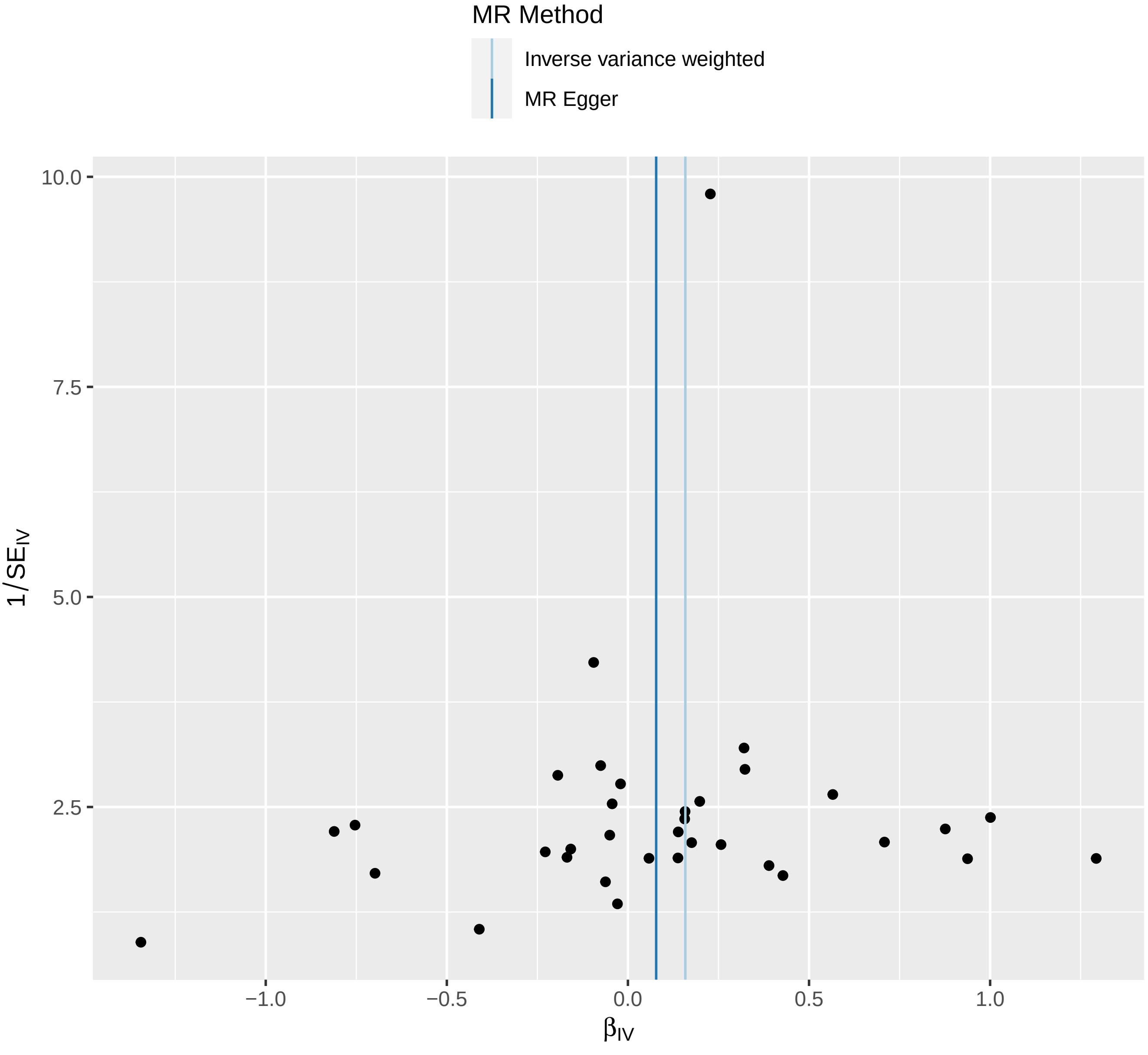

MR Method
Inverse variance weighted
MR Egger
10.0
7.5
5.0
2.5
$1/SE_{IV}$
−1.0
−0.5
0.0
0.5
1.0
β_{IV}

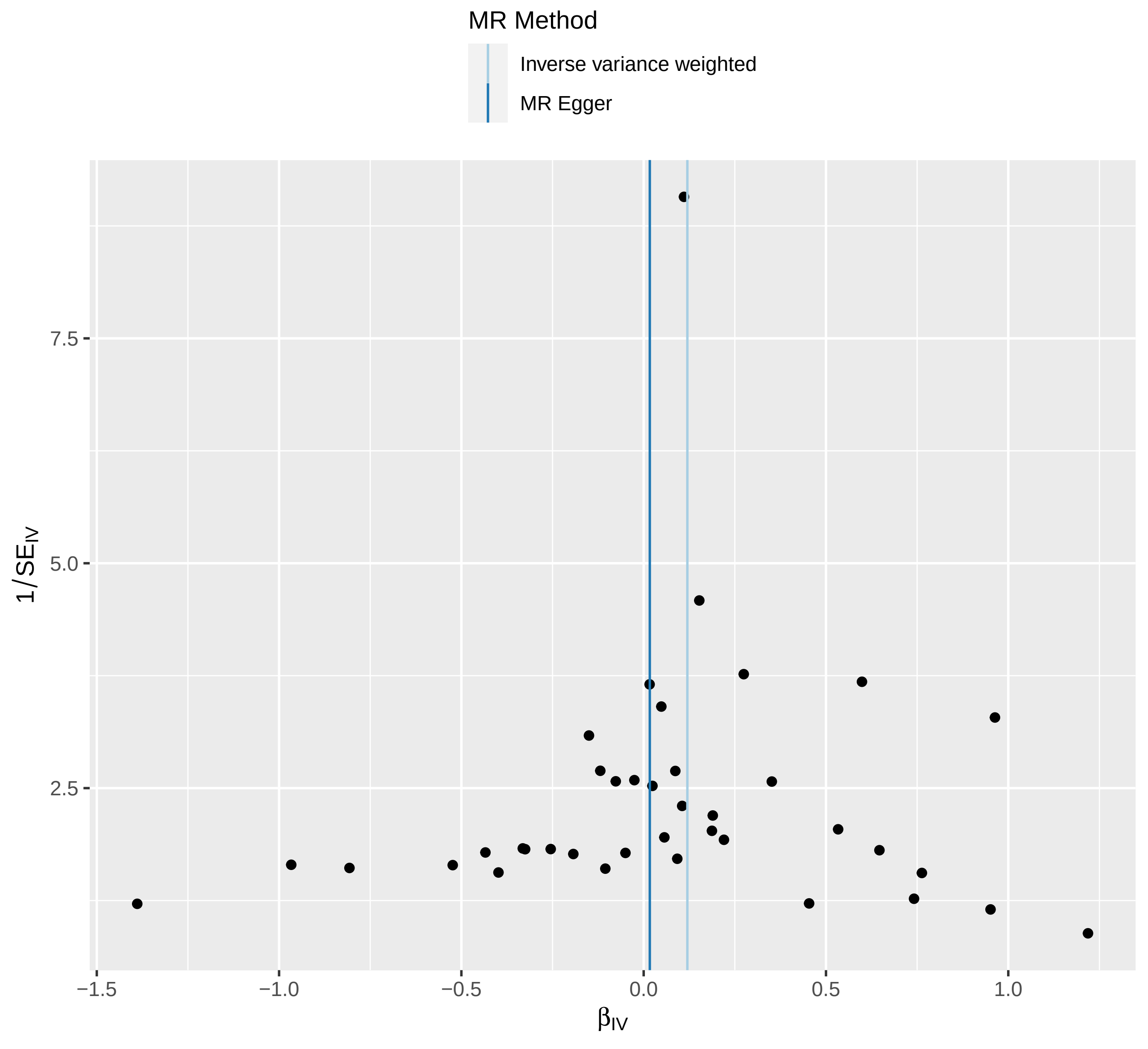
MR Method
Inverse variance weighted
MR Egger
1/SE$_{IV}$
7.5
5.0
2.5
−1.5
−1.0
−0.5
0.0
0.5
1.0
β$_{IV}$

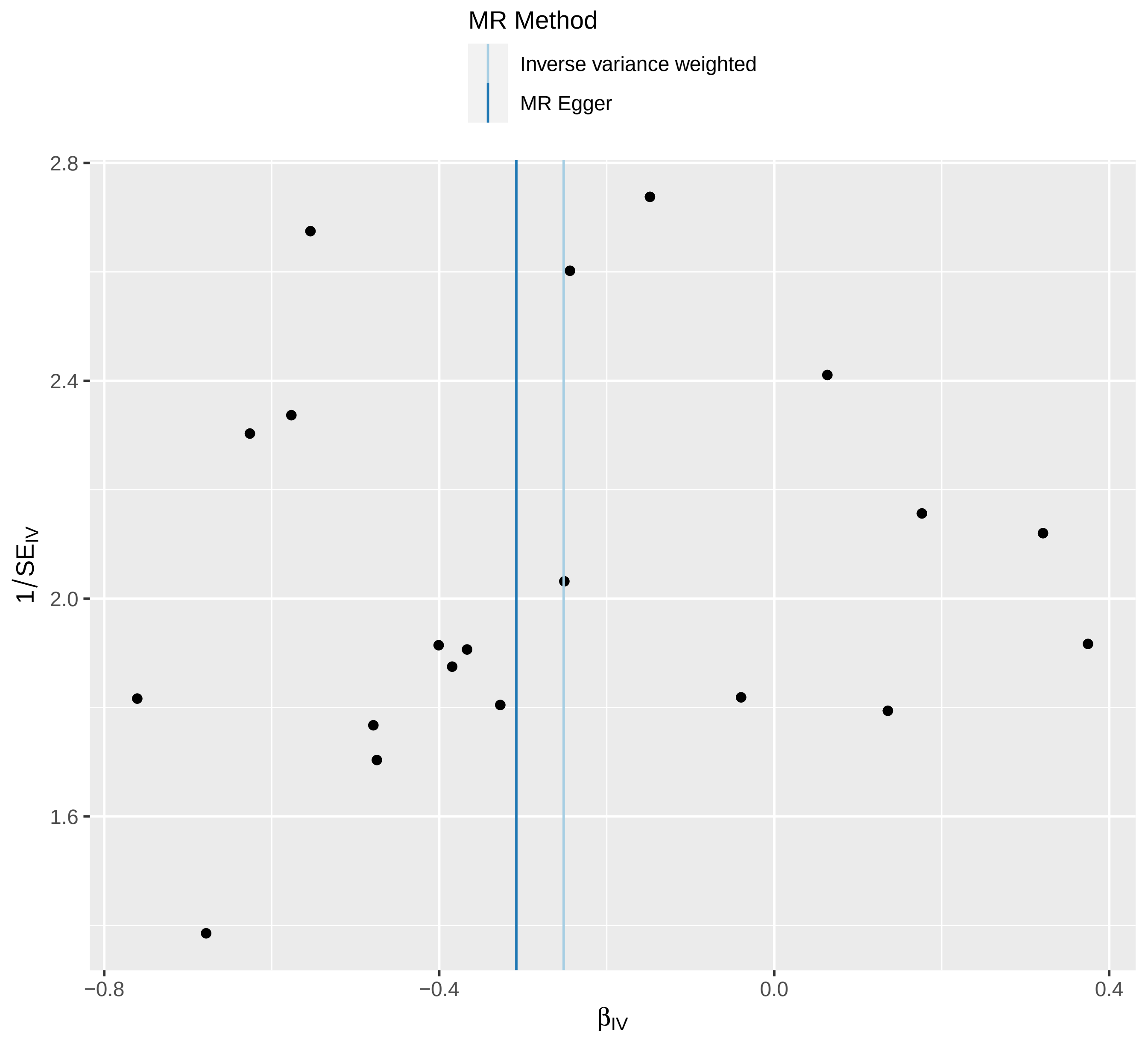

MR Method
Inverse variance weighted
MR Egger
2.8
2.4
2.0
1.6
1/SE_IV
−0.8
−0.4
0.0
0.4
β_IV

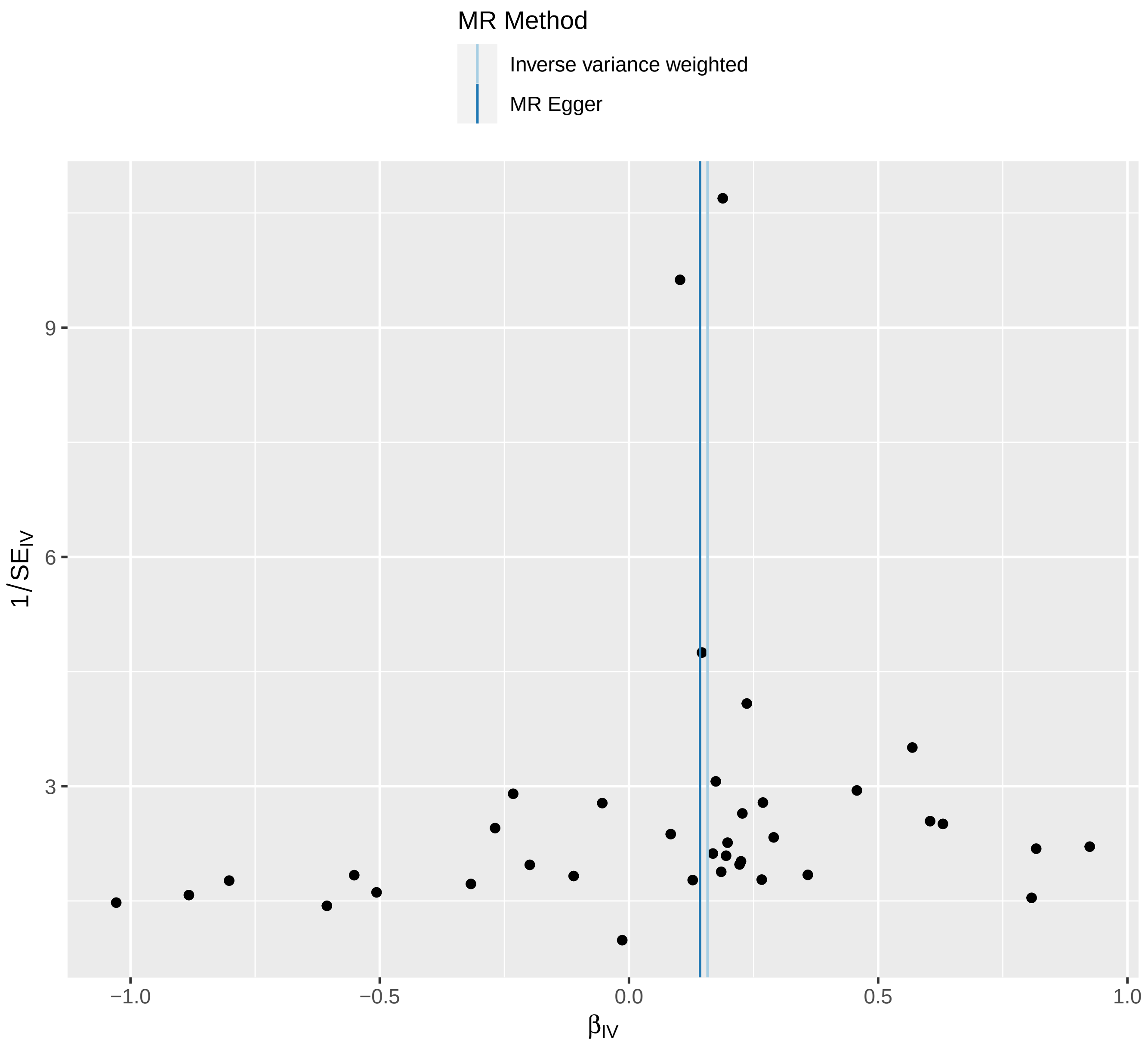

MR Method
Inverse variance weighted
MR Egger
1/SE$_{IV}$
9
6
3
−1.0
−0.5
0.0
0.5
1.0
β$_{IV}$

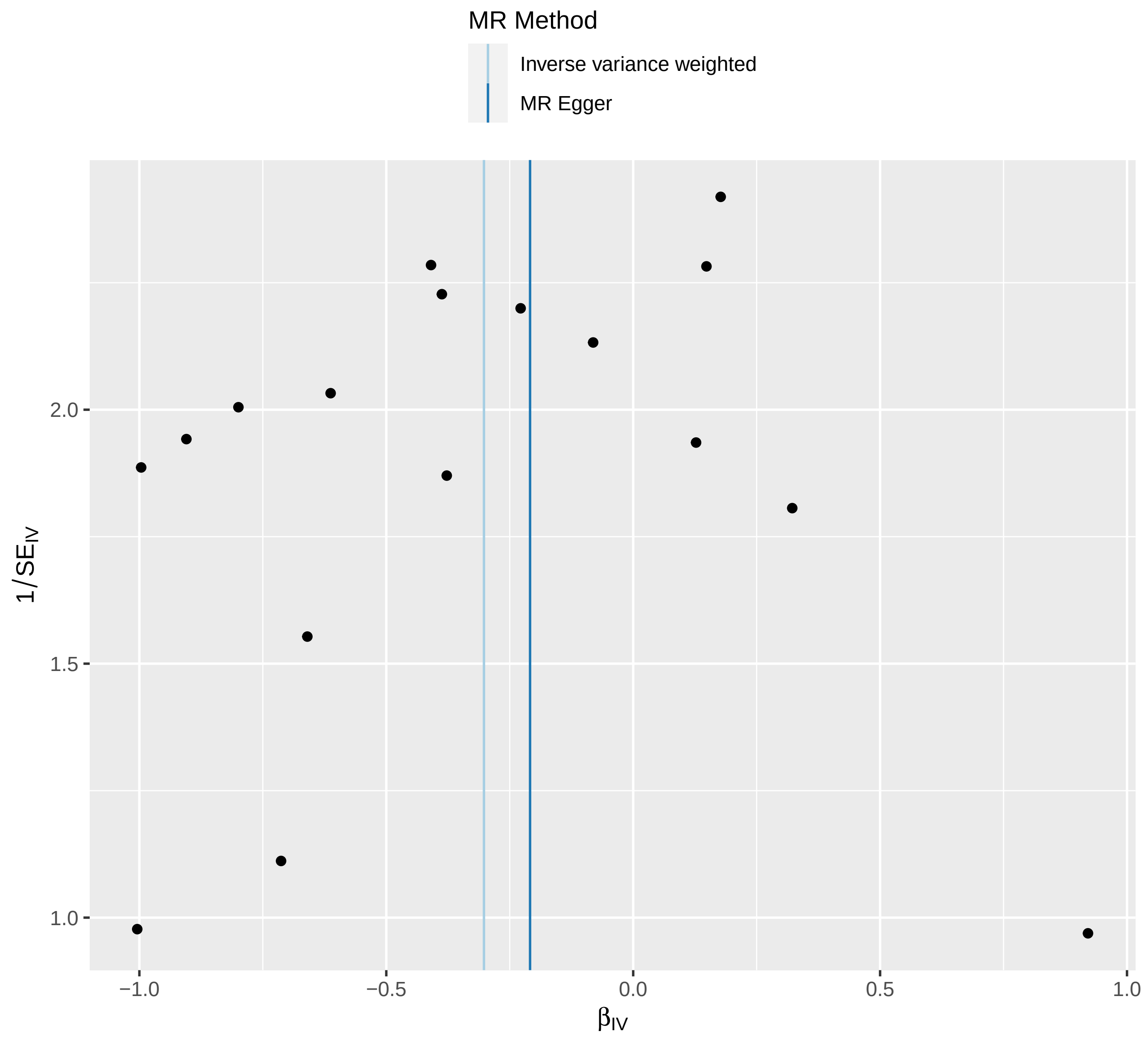
MR Method
Inverse variance weighted
MR Egger
1/SE_IV
2.0
1.5
1.0
−1.0
−0.5
0.0
0.5
1.0
β_IV

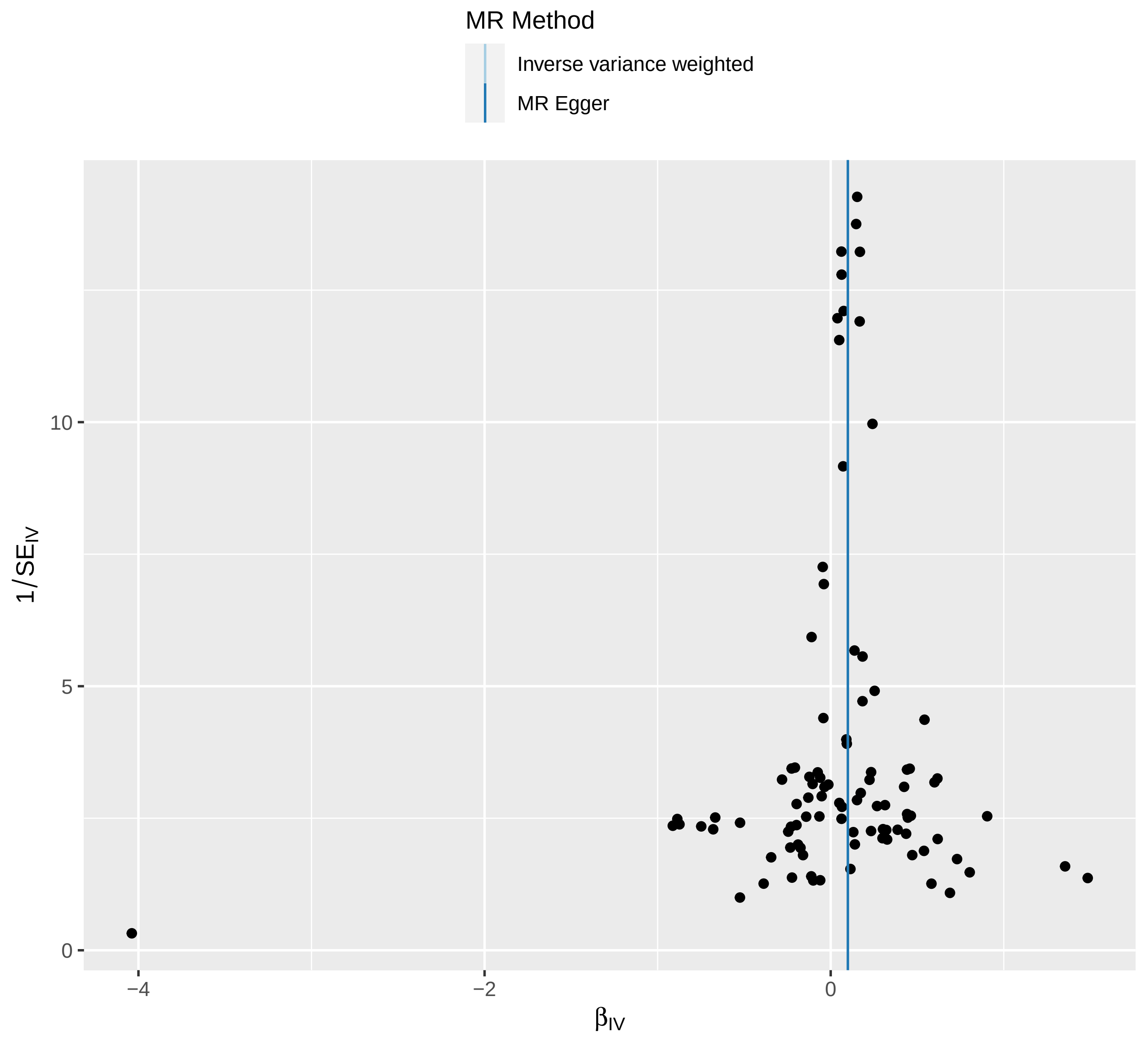
MR Method
Inverse variance weighted
MR Egger
$1/SE_{IV}$
10
5
0
−4
−2
0
β_{IV}

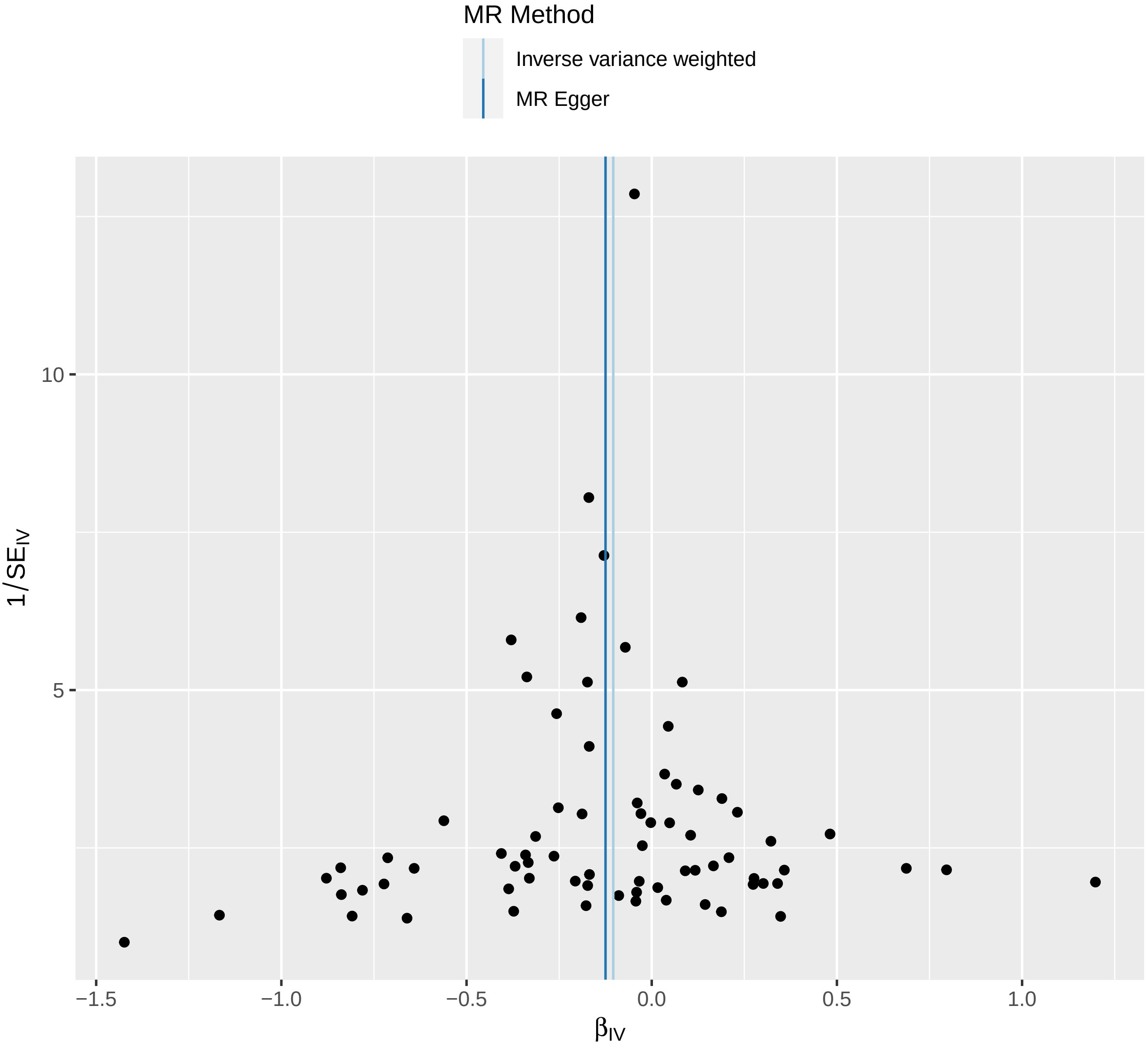
MR Method
Inverse variance weighted
MR Egger
10
5
−1.5
−1.0
−0.5
0.0
0.5
1.0
$1/SE_{IV}$
β_{IV}

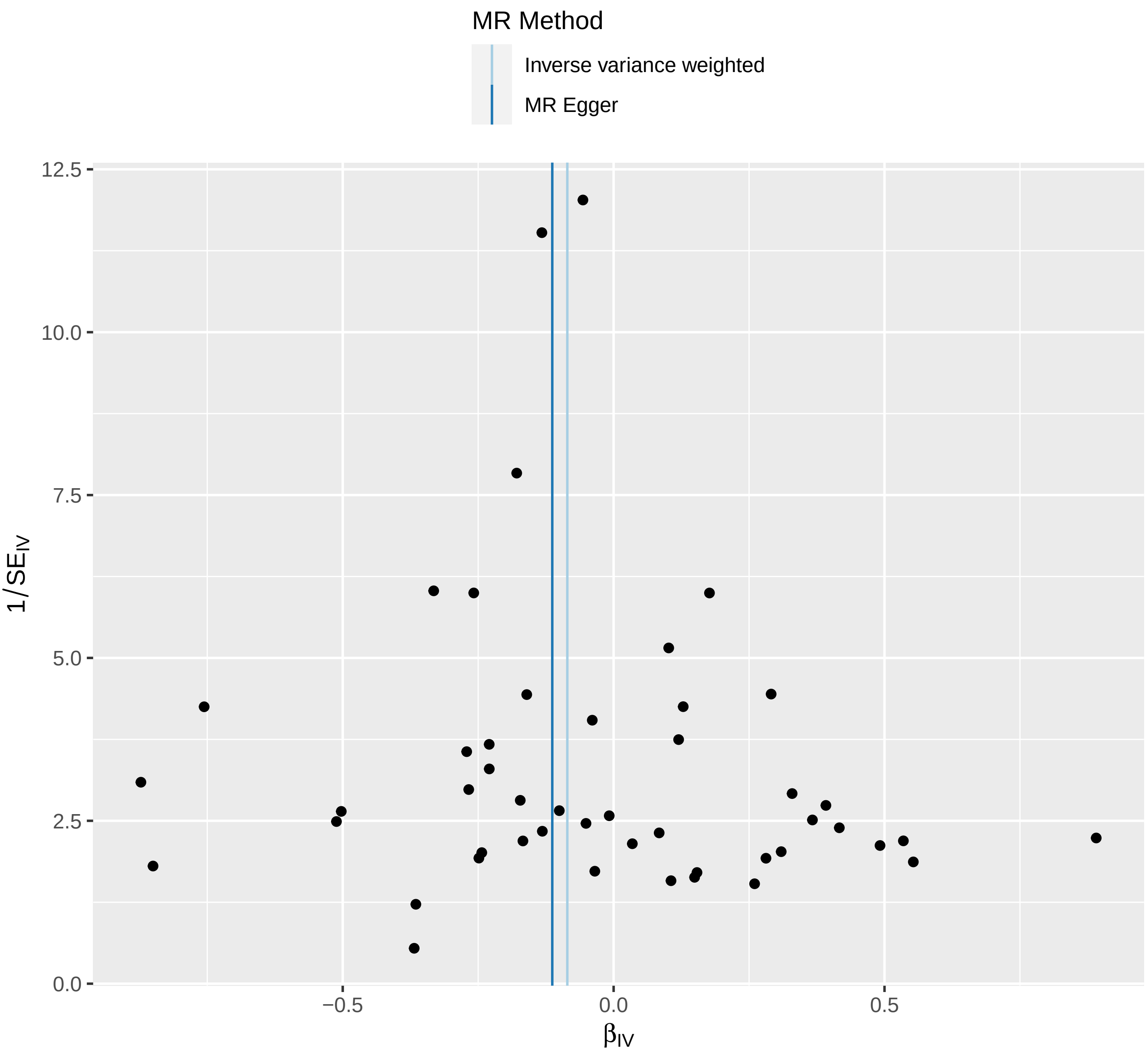

MR Method
Inverse variance weighted
MR Egger
12.5
10.0
7.5
5.0
2.5
0.0
1/SE_IV
−0.5
0.0
0.5
β_IV

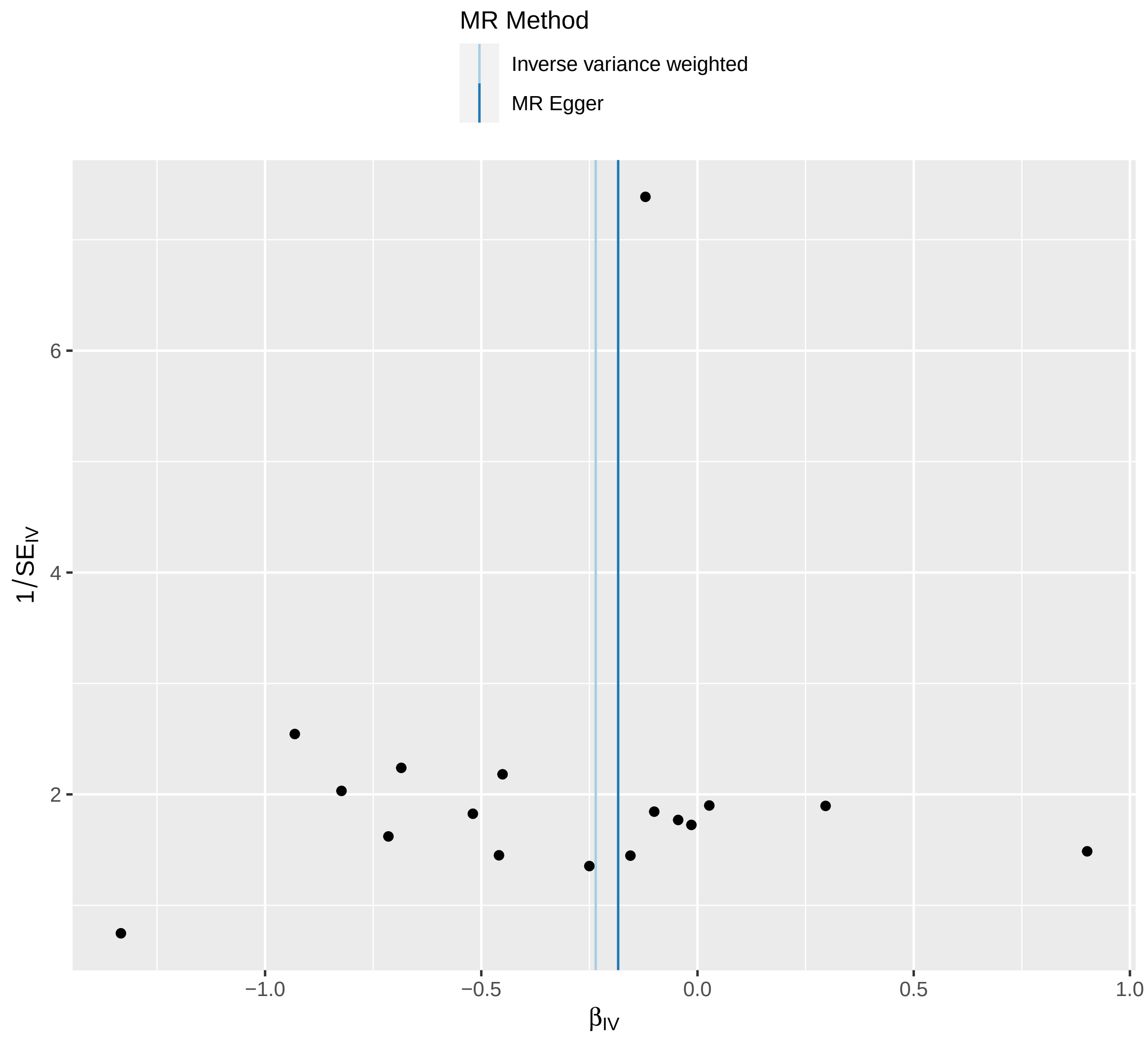
MR Method
Inverse variance weighted
MR Egger
6
4
2
$1/SE_{IV}$
−1.0
−0.5
0.0
0.5
1.0
β_{IV}

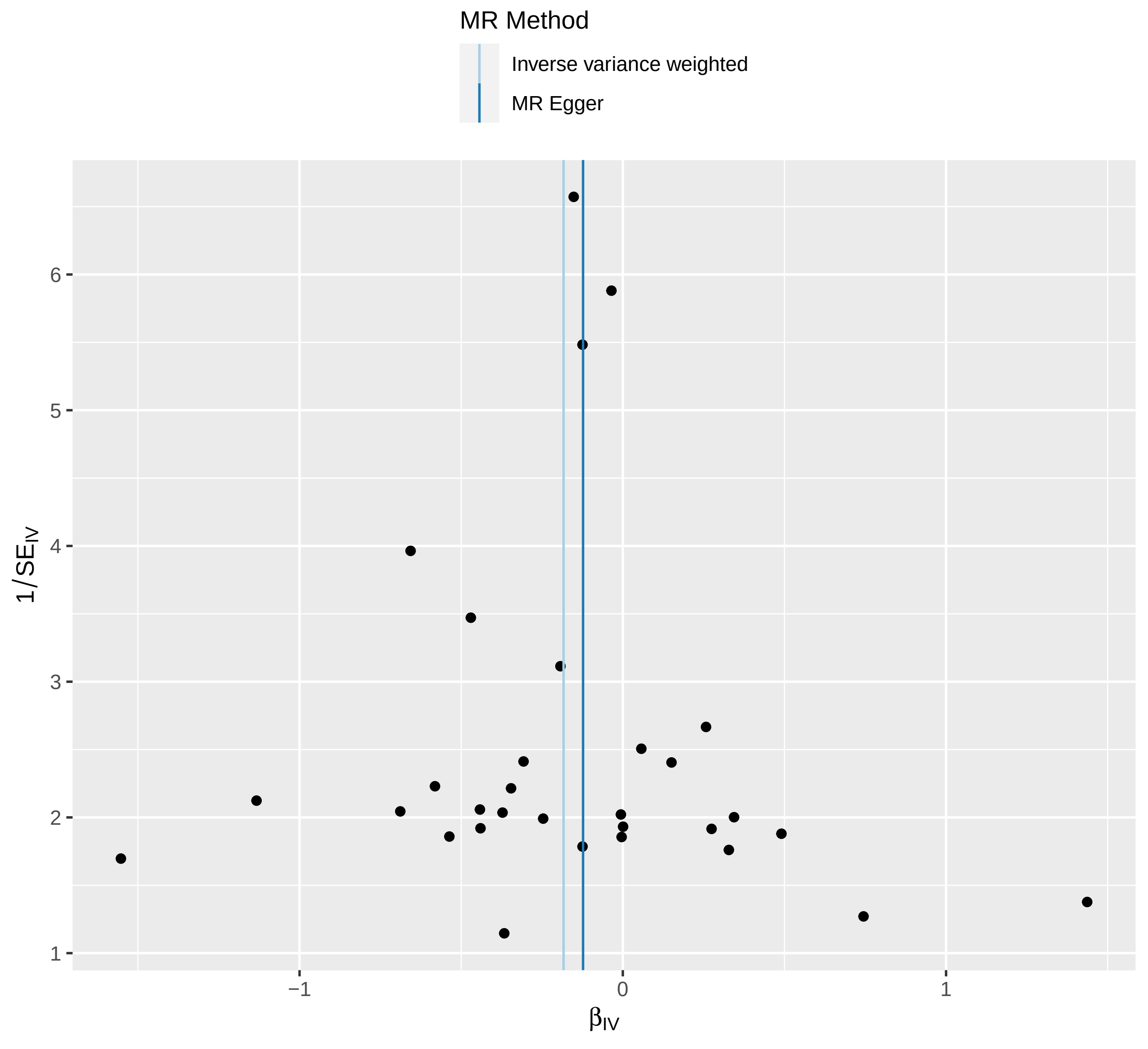

MR Method
Inverse variance weighted
MR Egger
6
5
4
3
2
1
$1/SE_{IV}$
−1
0
1
β_{IV}

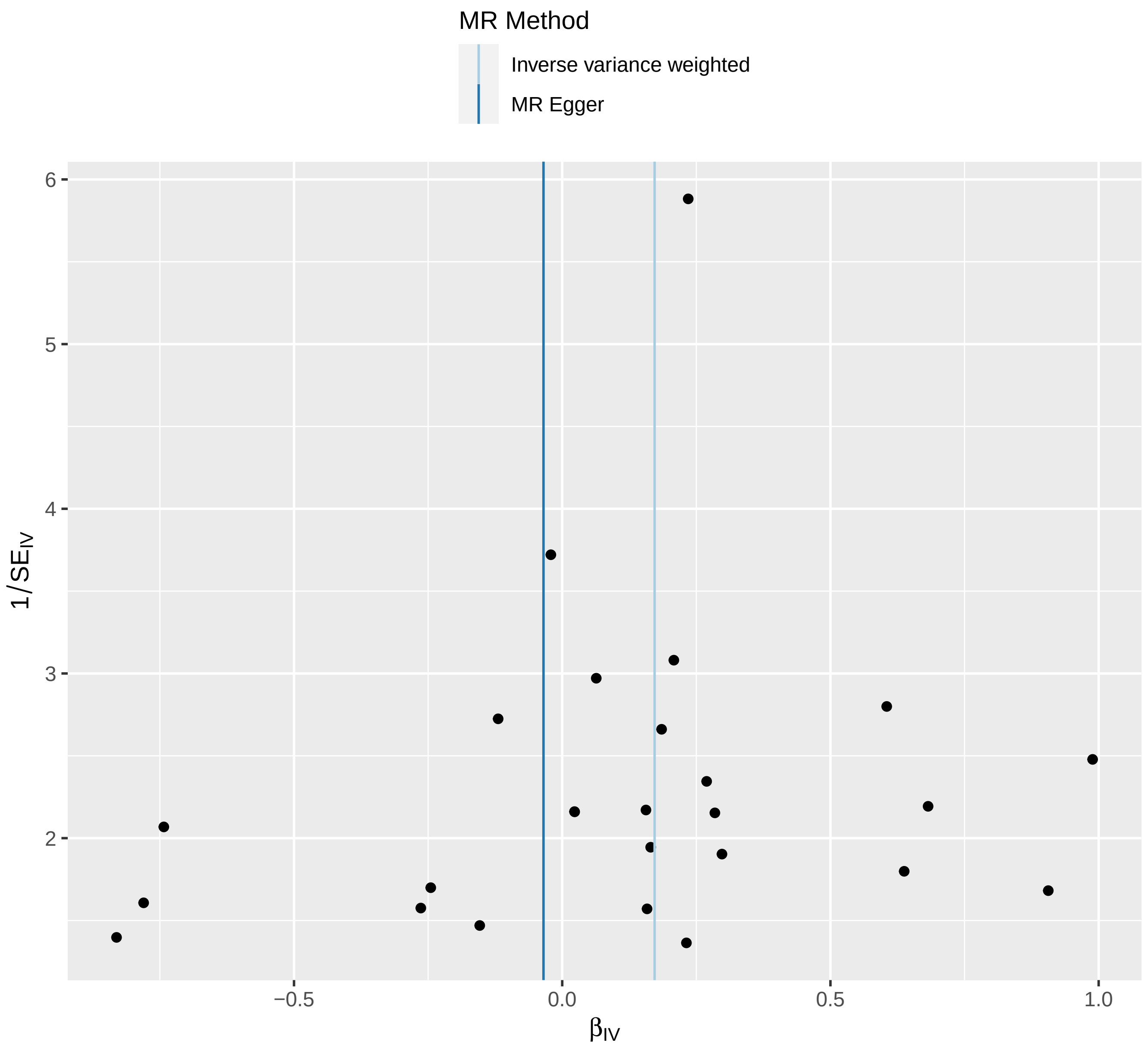
MR Method
Inverse variance weighted
MR Egger
6
5
4
3
2
1/SE_IV
−0.5
0.0
0.5
1.0
β_IV

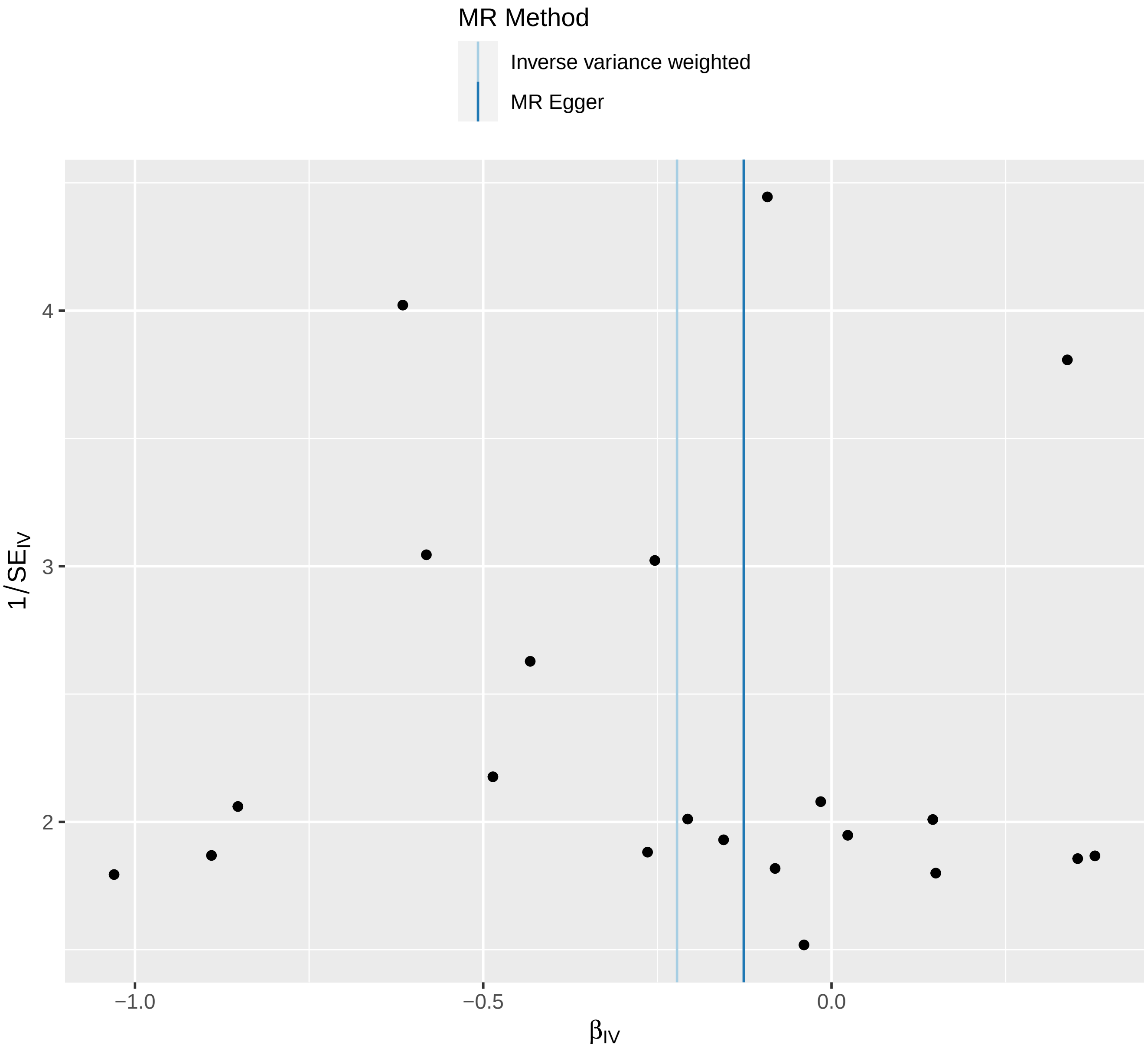
MR Method
Inverse variance weighted
MR Egger
4
3
2
1/SE_IV
−1.0
−0.5
0.0
β_IV

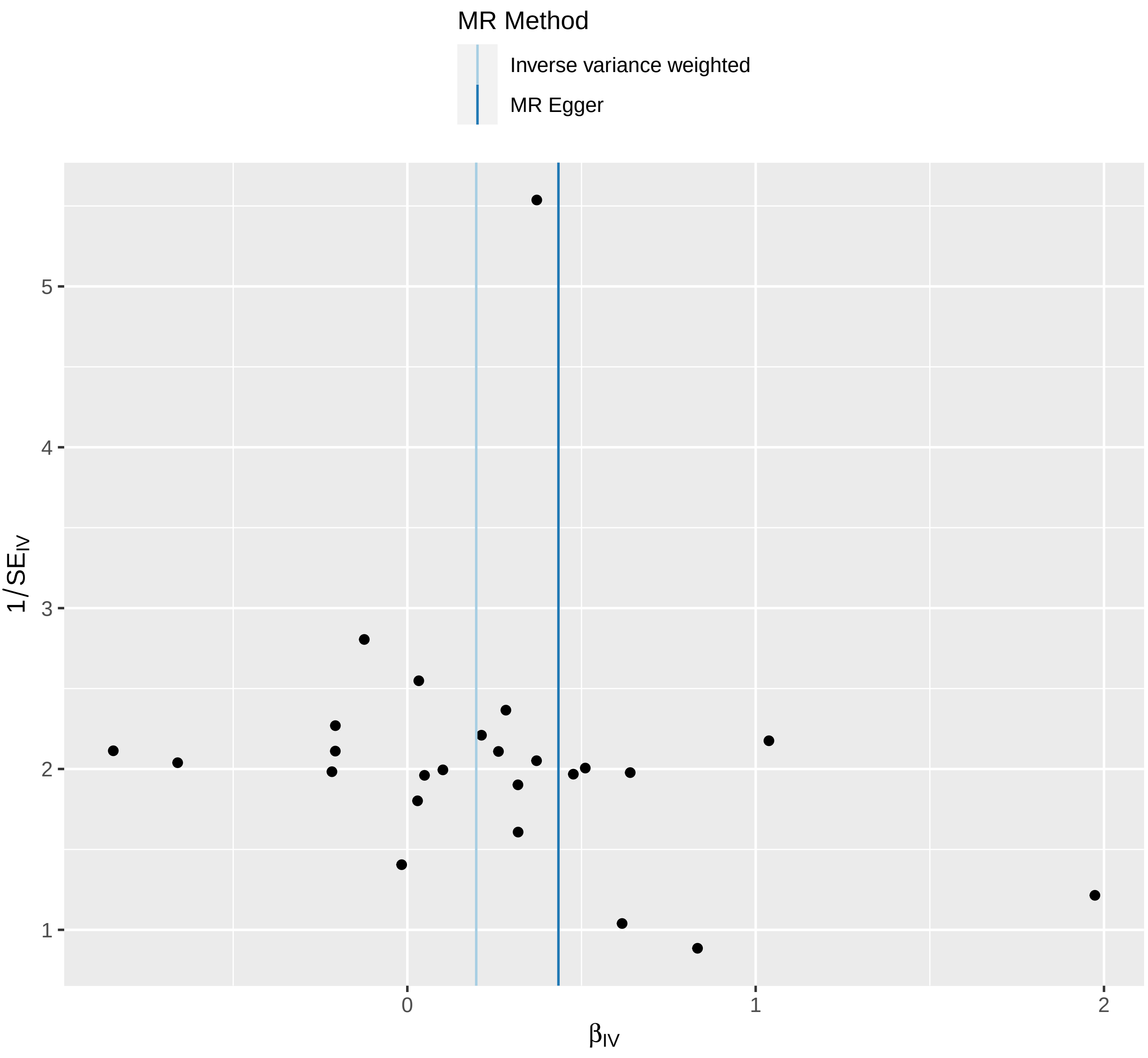
MR Method
Inverse variance weighted
MR Egger
1/SE_IV
5
4
3
2
1
0
1
2
β_IV

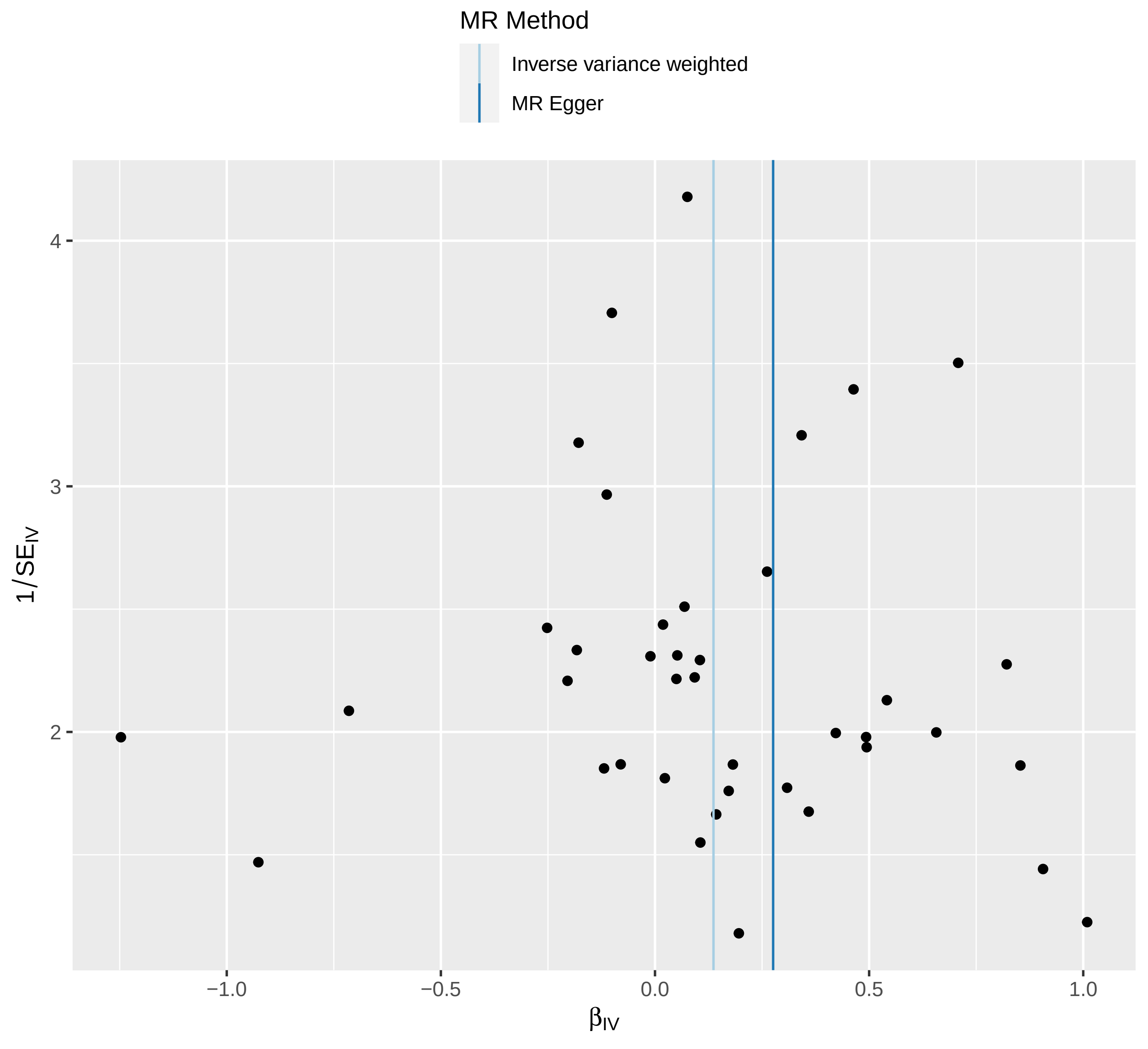

MR Method
Inverse variance weighted
MR Egger
4
3
2
1/SE_IV
−1.0
−0.5
0.0
0.5
1.0
β_IV

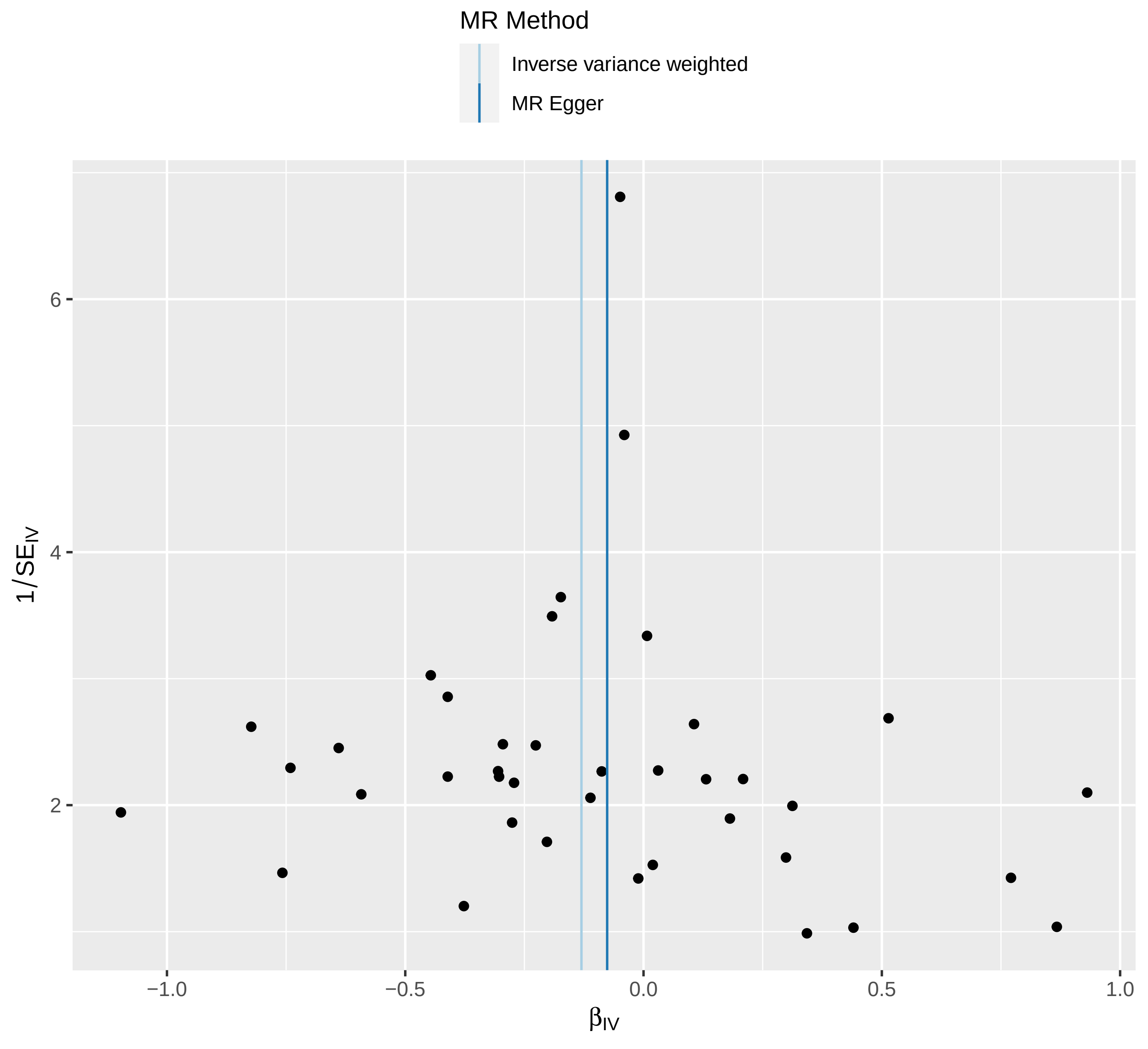
MR Method
Inverse variance weighted
MR Egger
6
4
2
1/SE$_{IV}$
−1.0
−0.5
0.0
0.5
1.0
β$_{IV}$

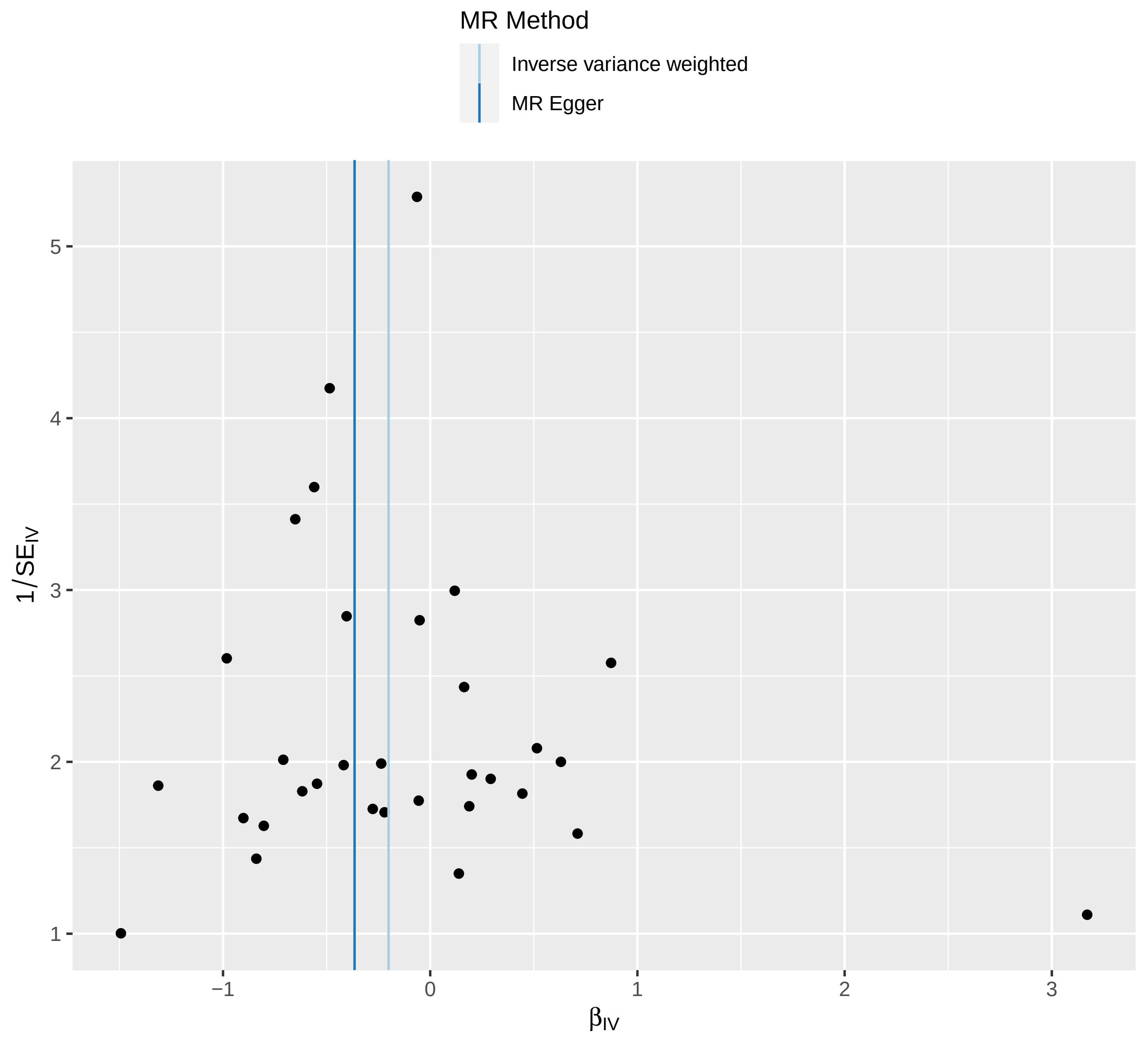
MR Method
Inverse variance weighted
MR Egger
1/SE_IV
5
4
3
2
1
−1
0
1
2
3
β_IV

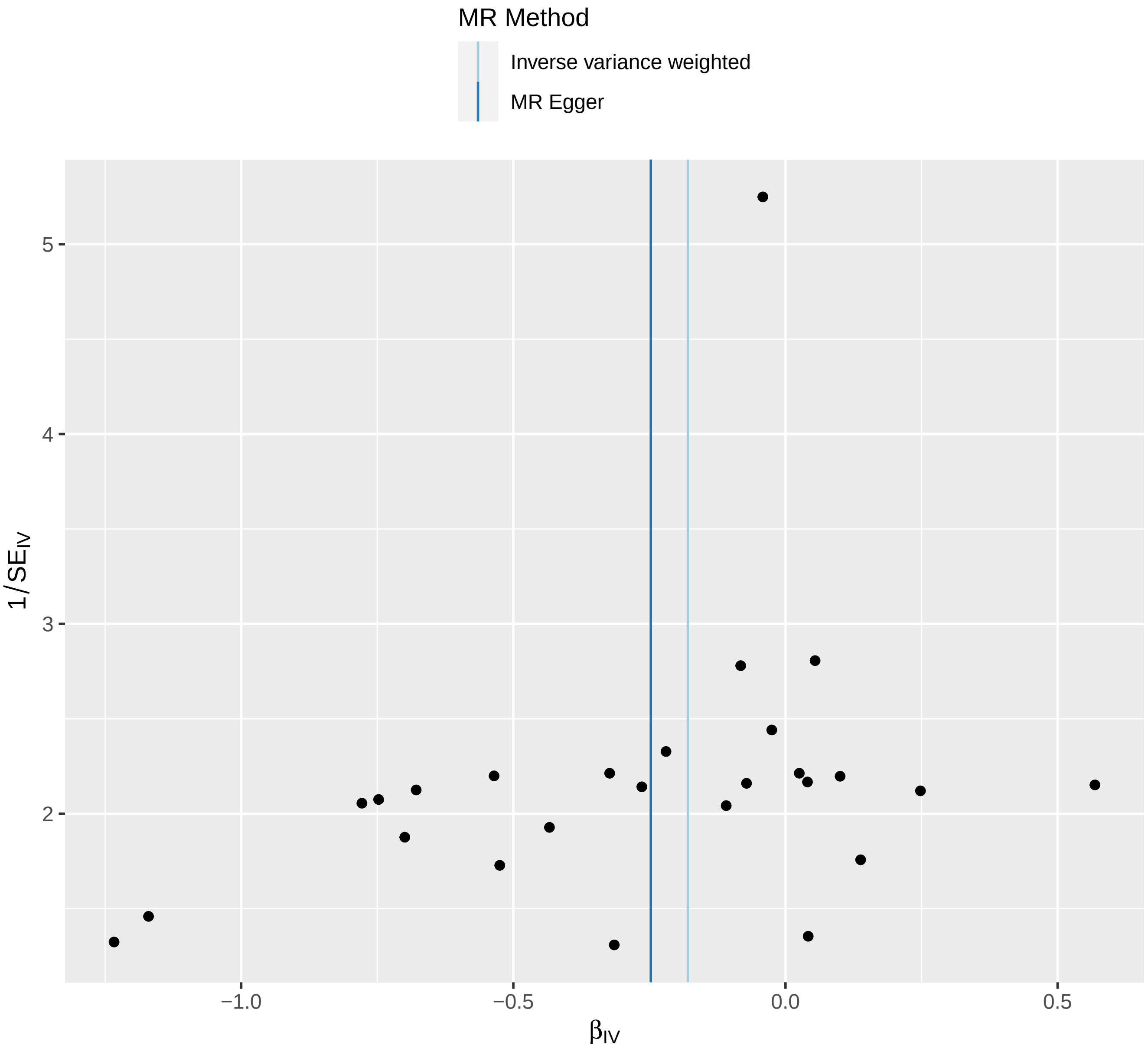
MR Method
Inverse variance weighted
MR Egger
1/SE_IV
β_IV
5
4
3
2
−1.0
−0.5
0.0
0.5

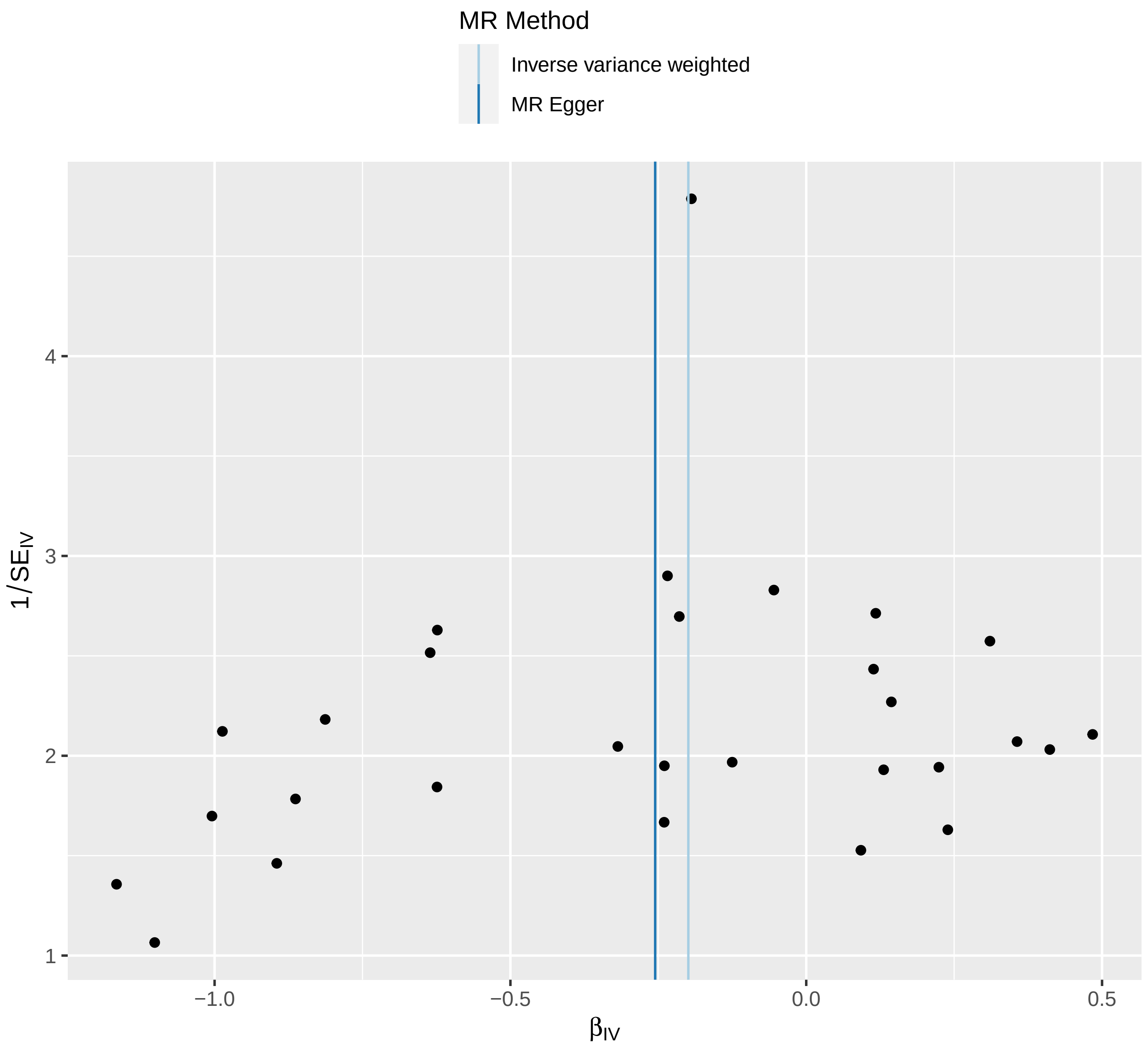

MR Method
Inverse variance weighted
MR Egger
1/SE_IV
β_IV
−1.0
−0.5
0.0
0.5
1
2
3
4

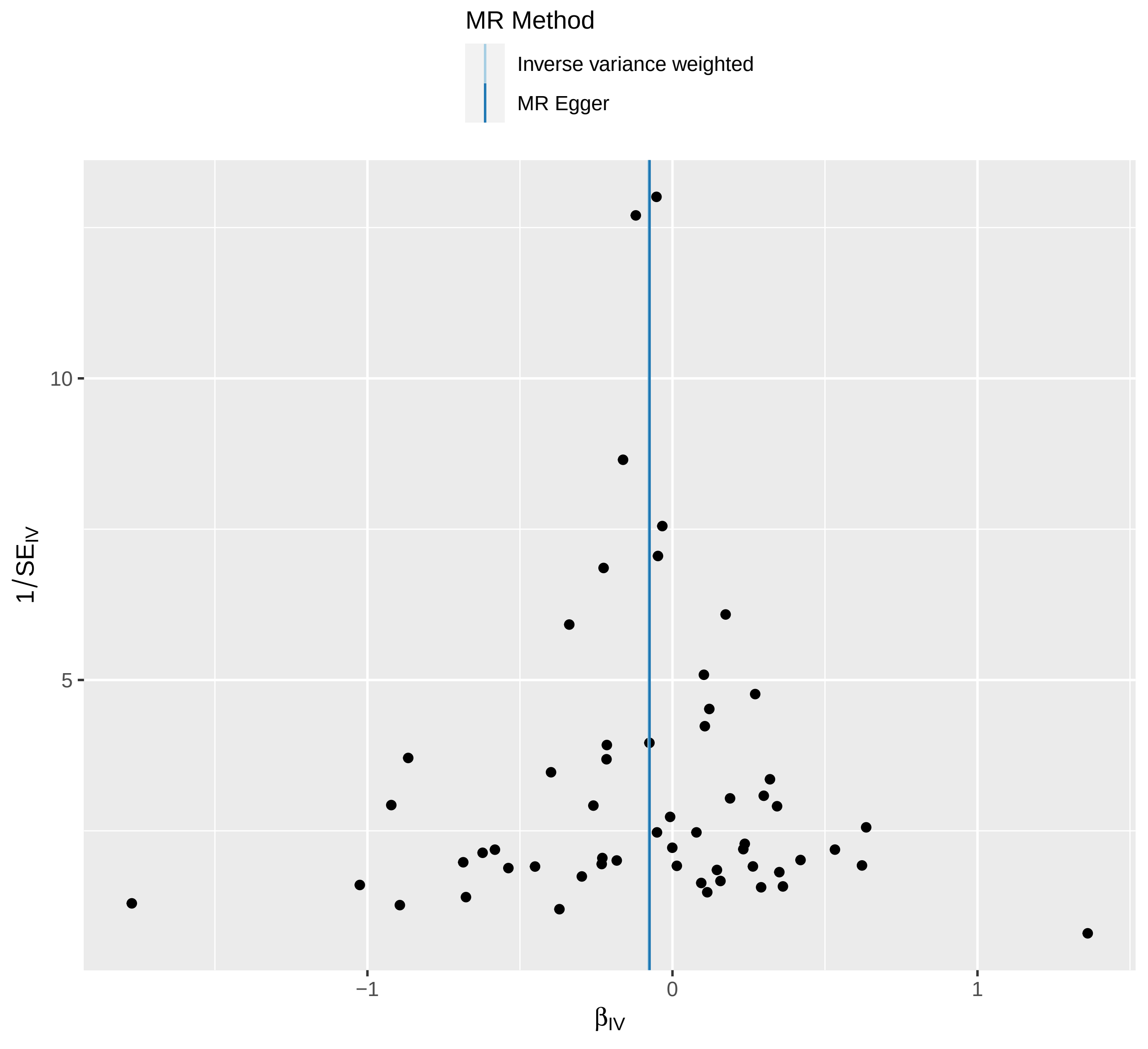
MR Method
Inverse variance weighted
MR Egger
10
5
1/SE$_{IV}$
−1
0
1
β$_{IV}$

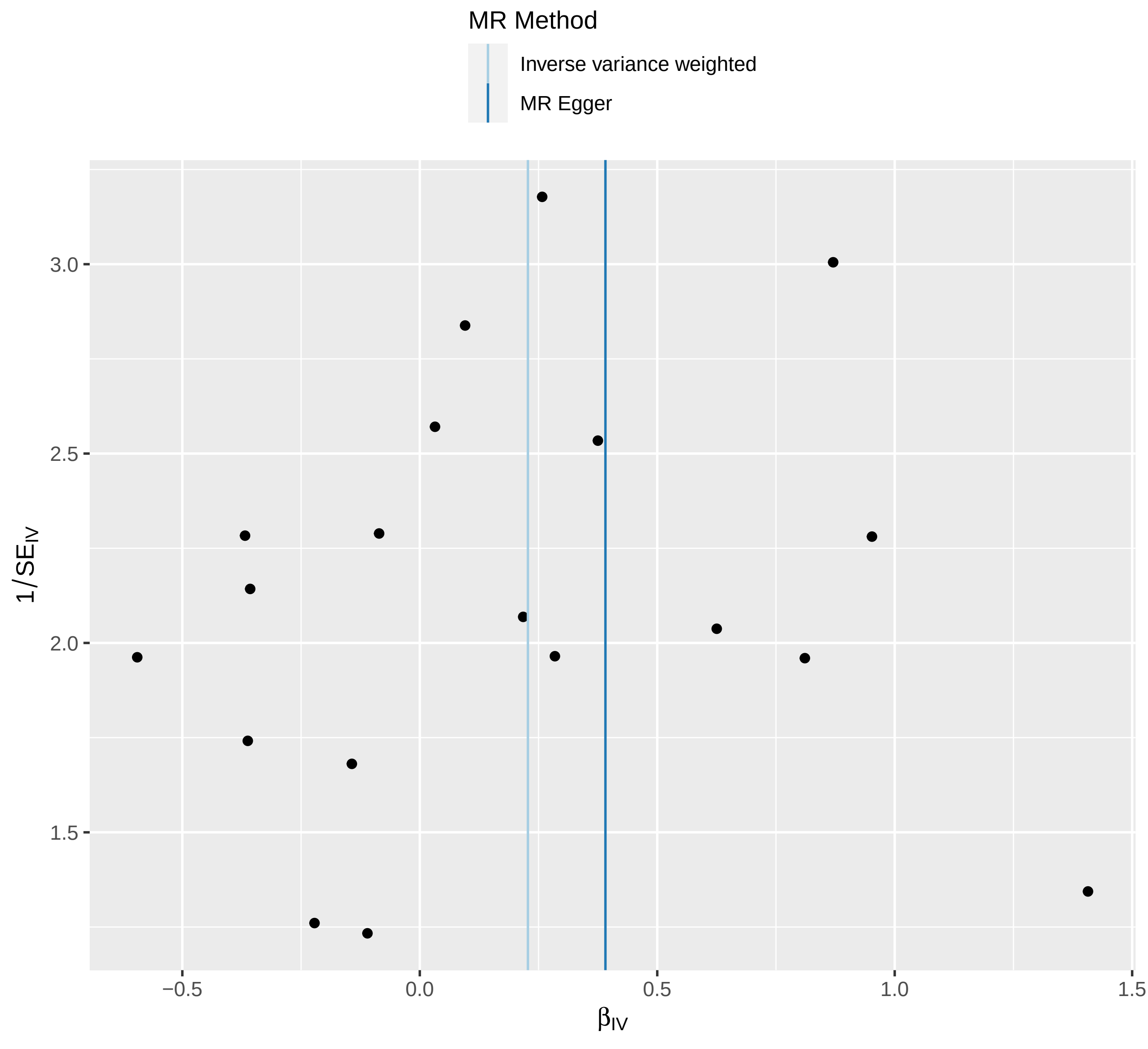

MR Method
Inverse variance weighted
MR Egger
3.0
2.5
2.0
1.5
1/SE$_{IV}$
−0.5
0.0
0.5
1.0
1.5
β$_{IV}$

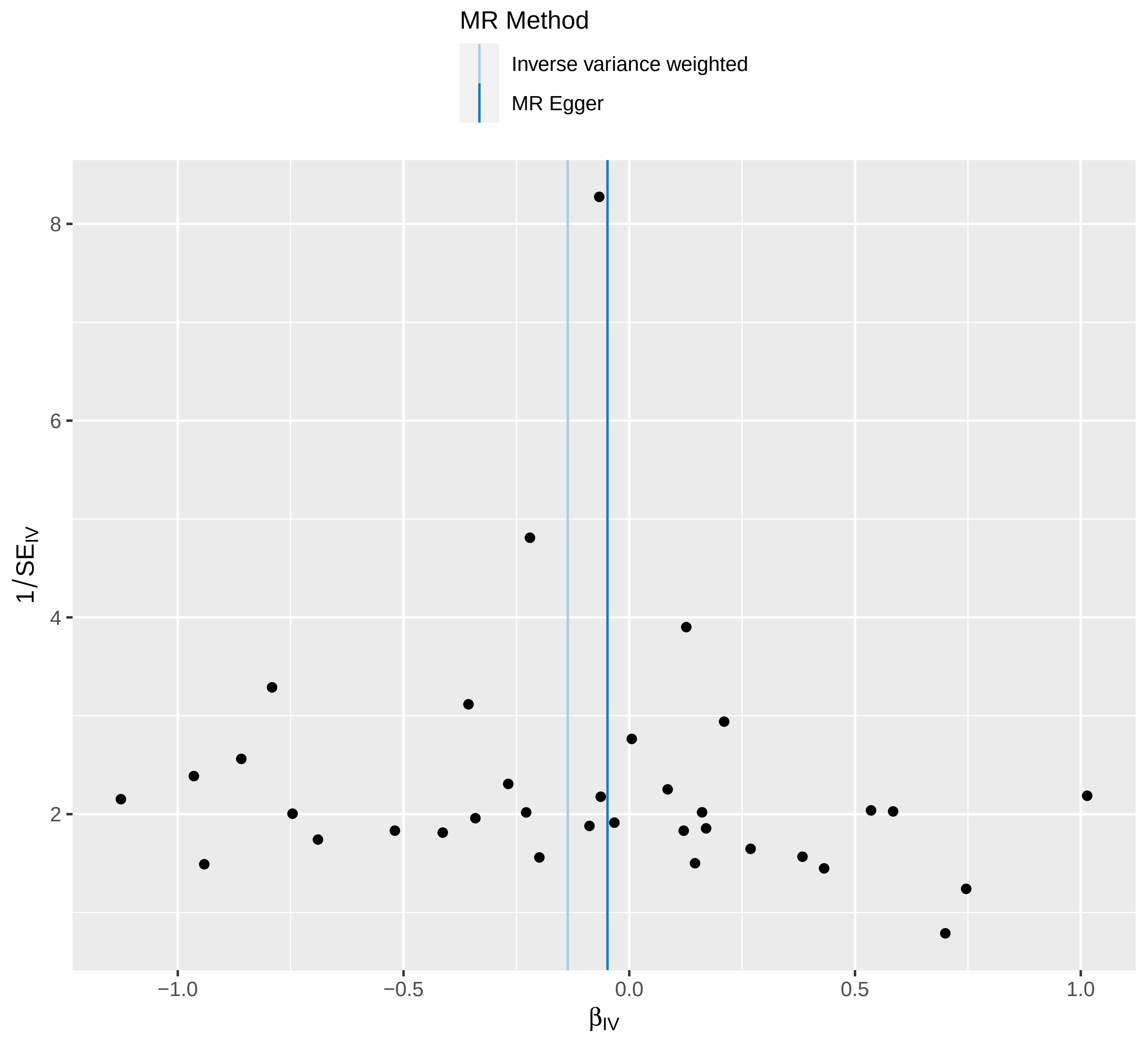

MR Method
Inverse variance weighted
MR Egger
8
6
4
2
1/SE$_{IV}$
−1.0
−0.5
0.0
0.5
1.0
β$_{IV}$

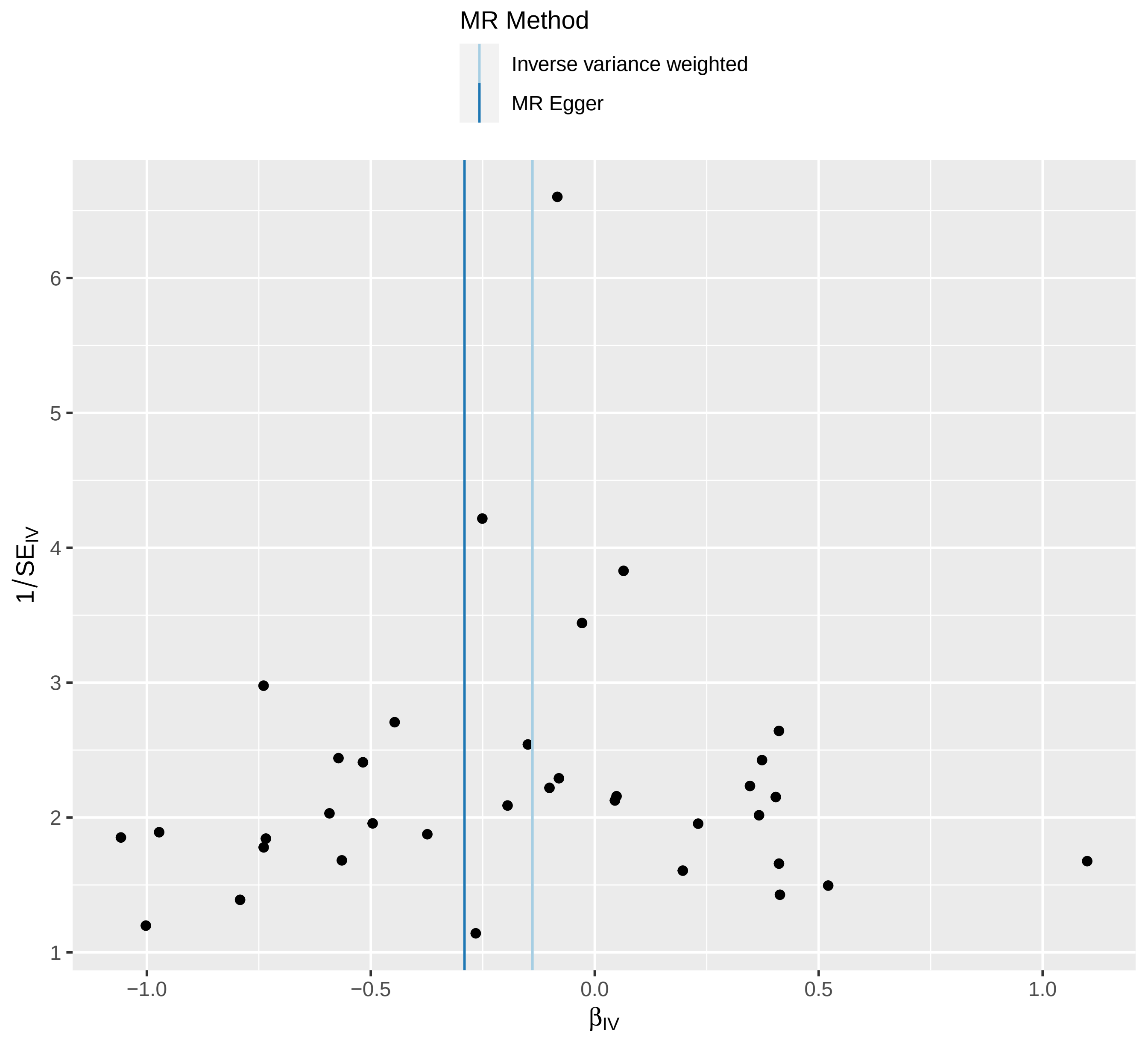
MR Method
Inverse variance weighted
MR Egger
1/SE_IV
6
5
4
3
2
1
−1.0
−0.5
0.0
0.5
1.0
β_IV

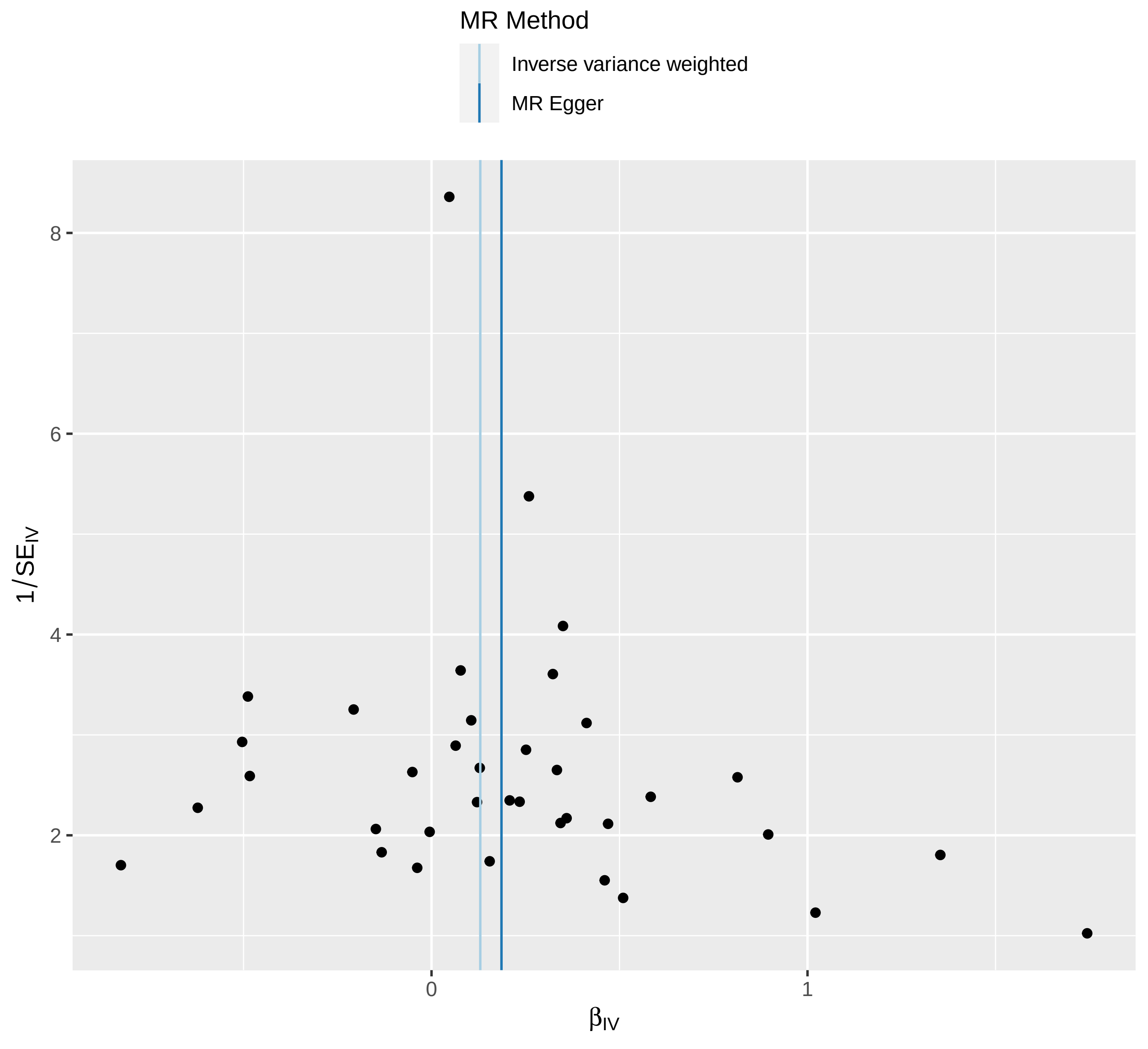

MR Method
Inverse variance weighted
MR Egger
8
6
4
2
1/SE_IV
0
1
β_IV

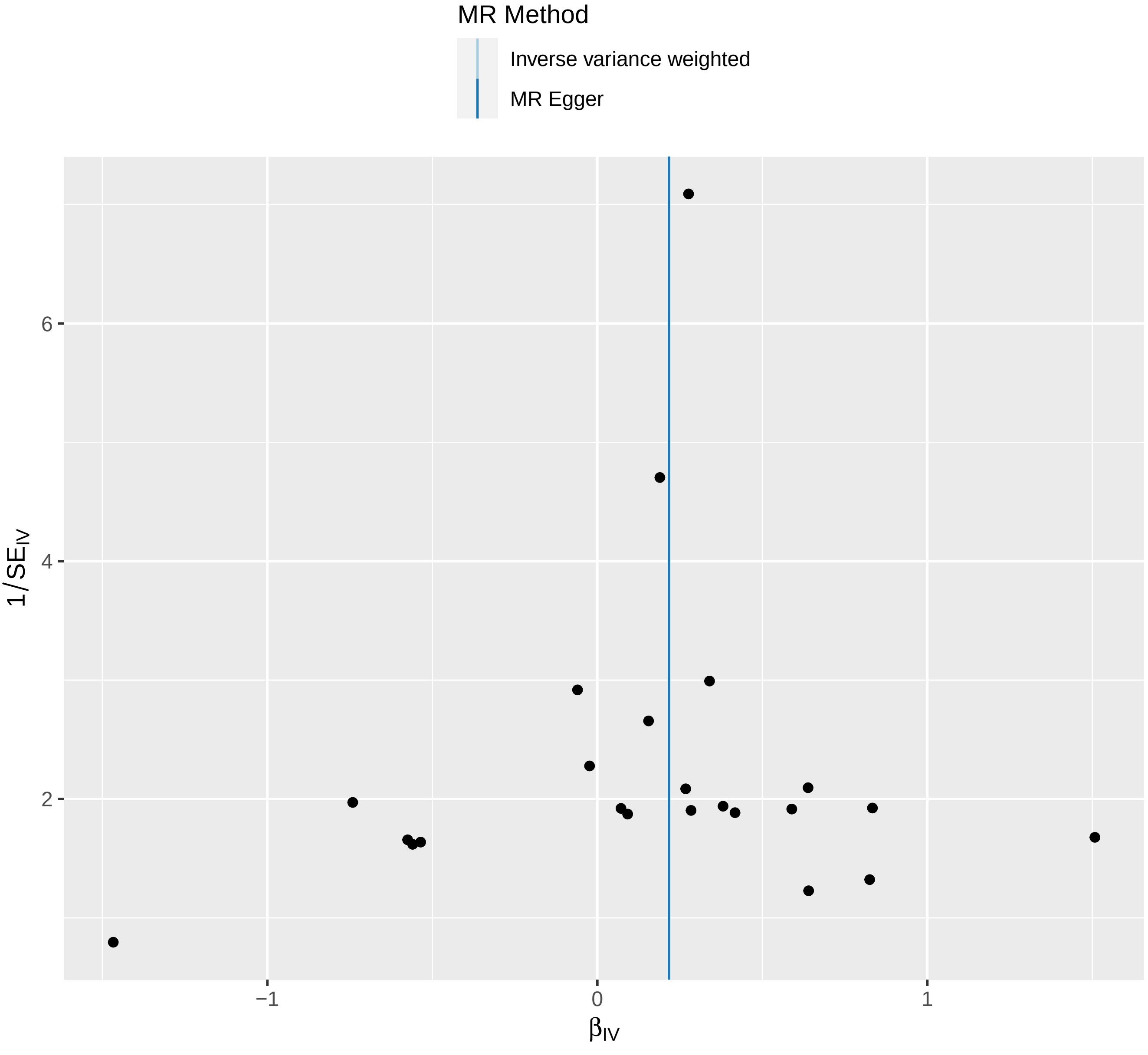

MR Method
Inverse variance weighted
MR Egger
6
4
2
1/SE_IV
−1
0
1
β_IV

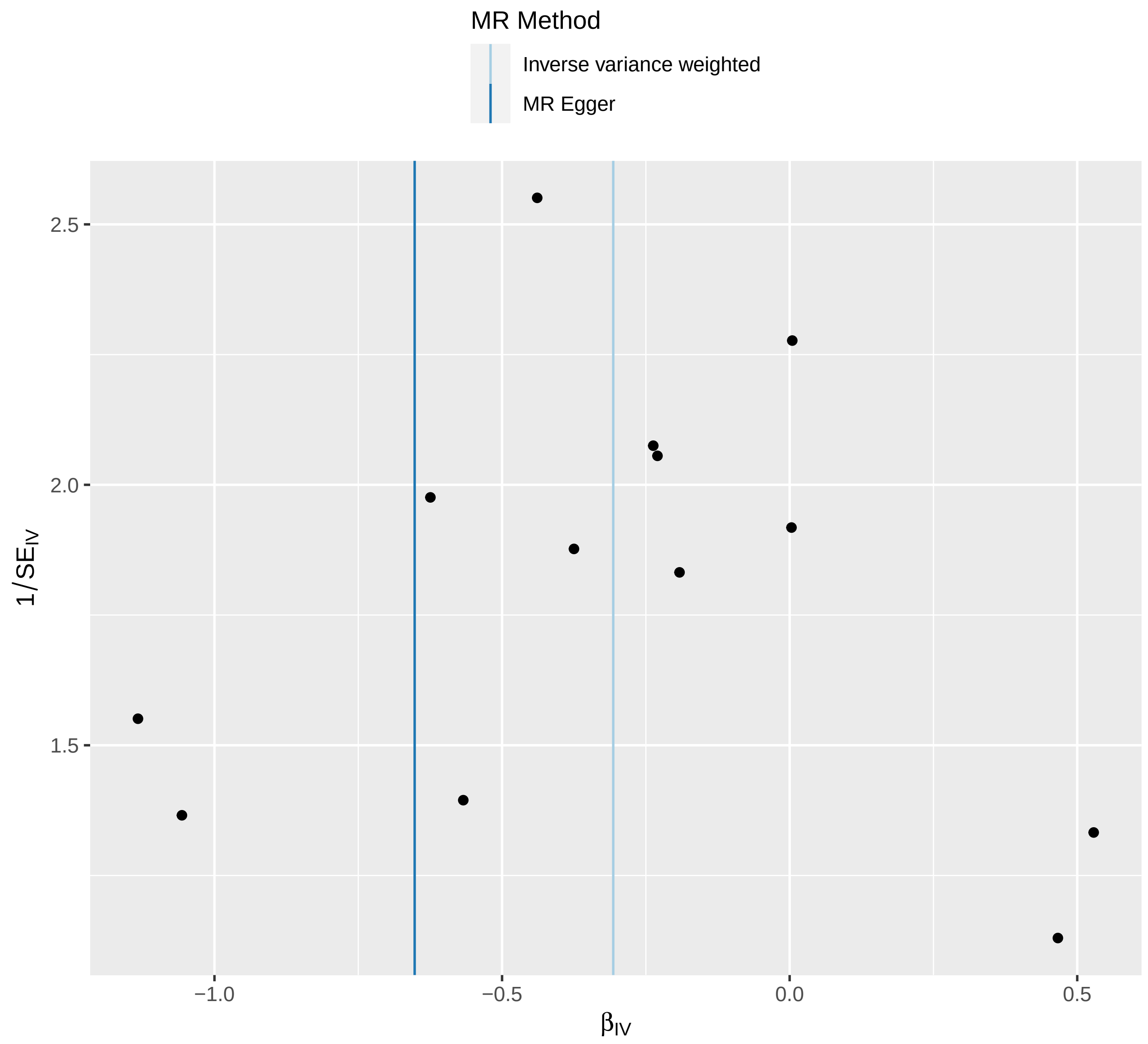
MR Method
Inverse variance weighted
MR Egger
2.5
2.0
1.5
$1/SE_{IV}$
−1.0
−0.5
0.0
0.5
β_{IV}

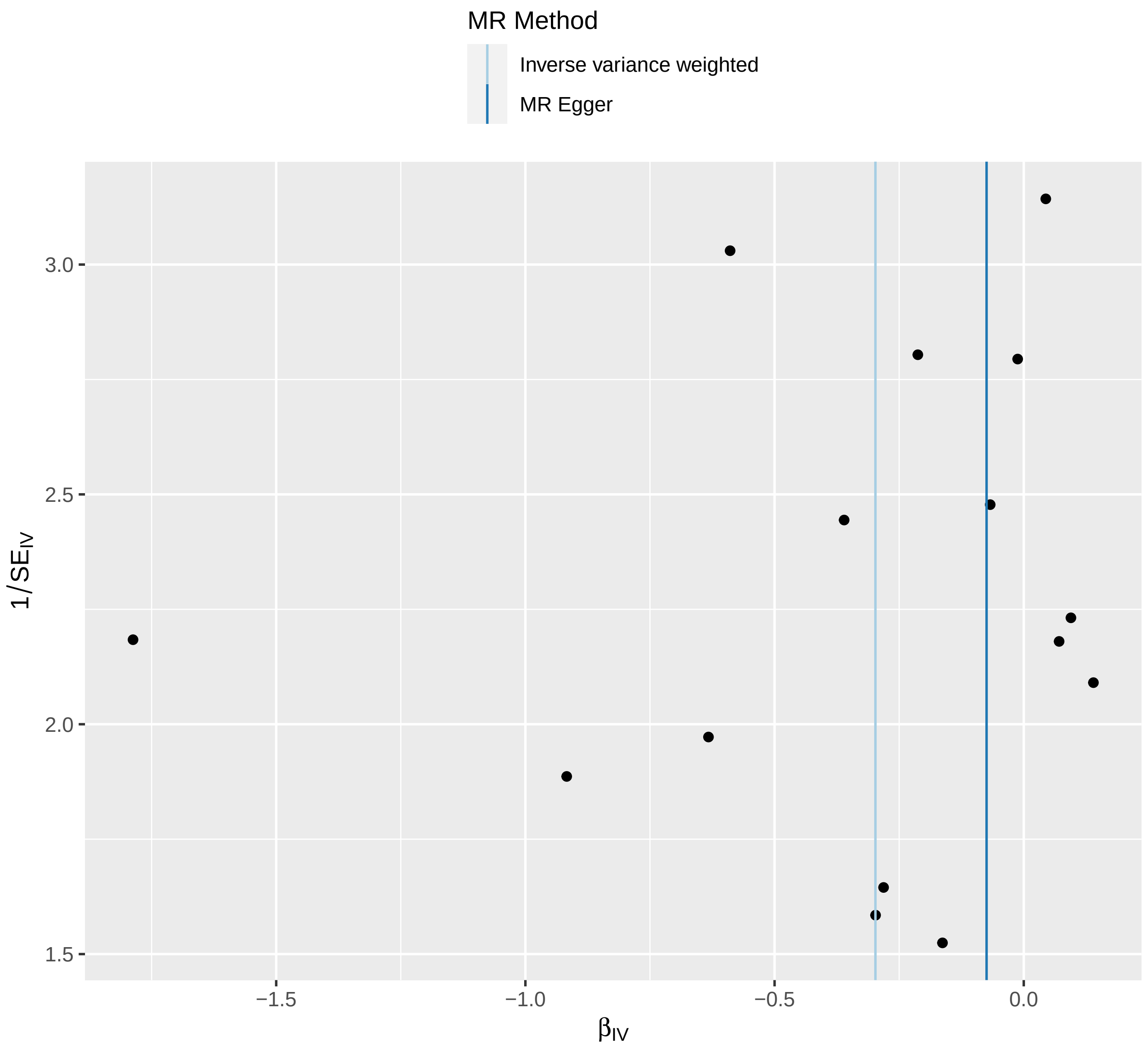
MR Method
Inverse variance weighted
MR Egger
3.0
2.5
2.0
1.5
$1/SE_{IV}$
−1.5
−1.0
−0.5
0.0
β_{IV}

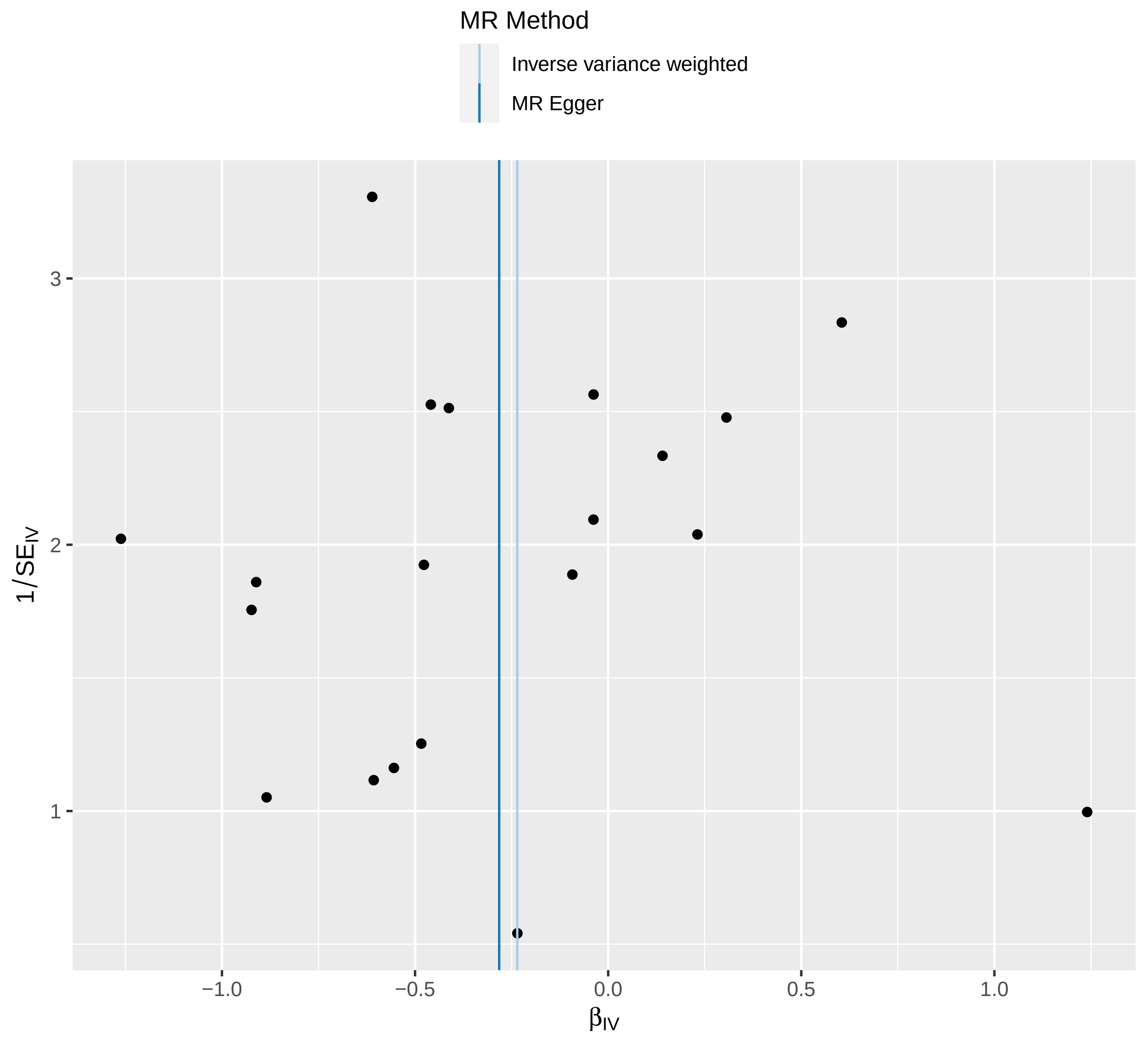
MR Method
Inverse variance weighted
MR Egger
3
2
1
1/SE_IV
−1.0
−0.5
0.0
0.5
1.0
β_IV

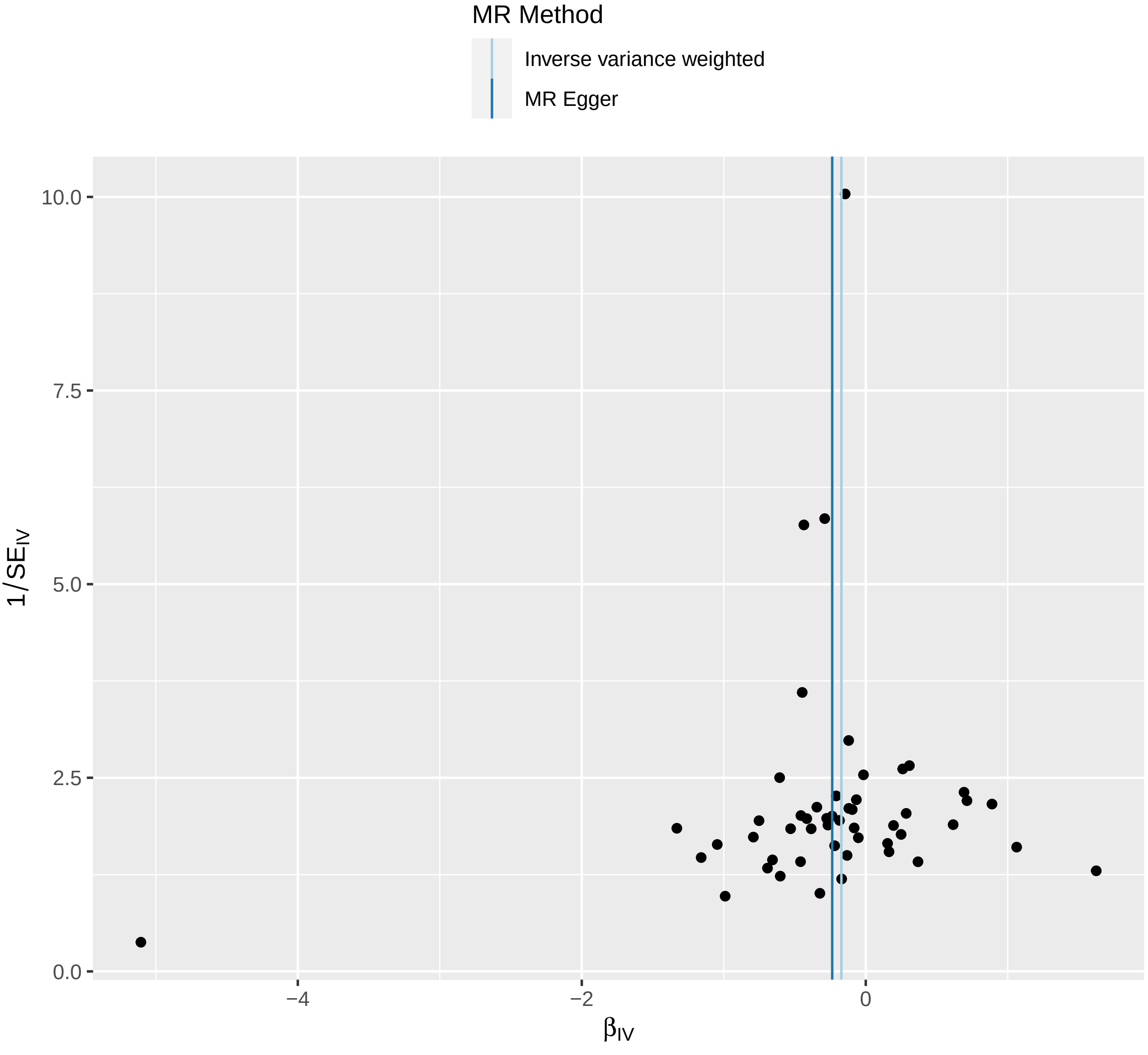

MR Method
Inverse variance weighted
MR Egger
10.0
7.5
5.0
2.5
0.0
1/SE_IV
−4
−2
0
β_IV

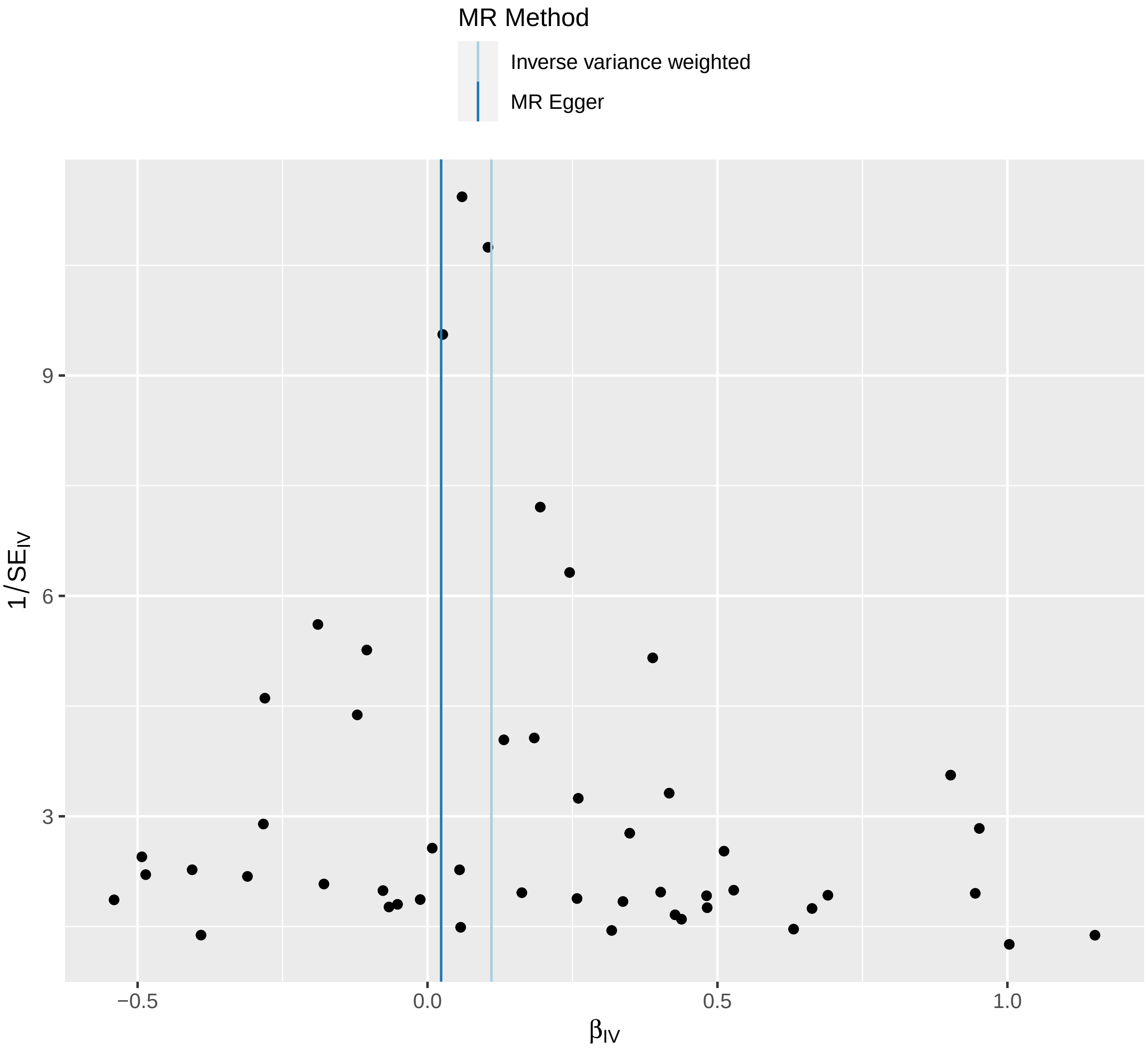

MR Method
Inverse variance weighted
MR Egger
9
6
3
1/SE$_{IV}$
−0.5
0.0
0.5
1.0
β$_{IV}$

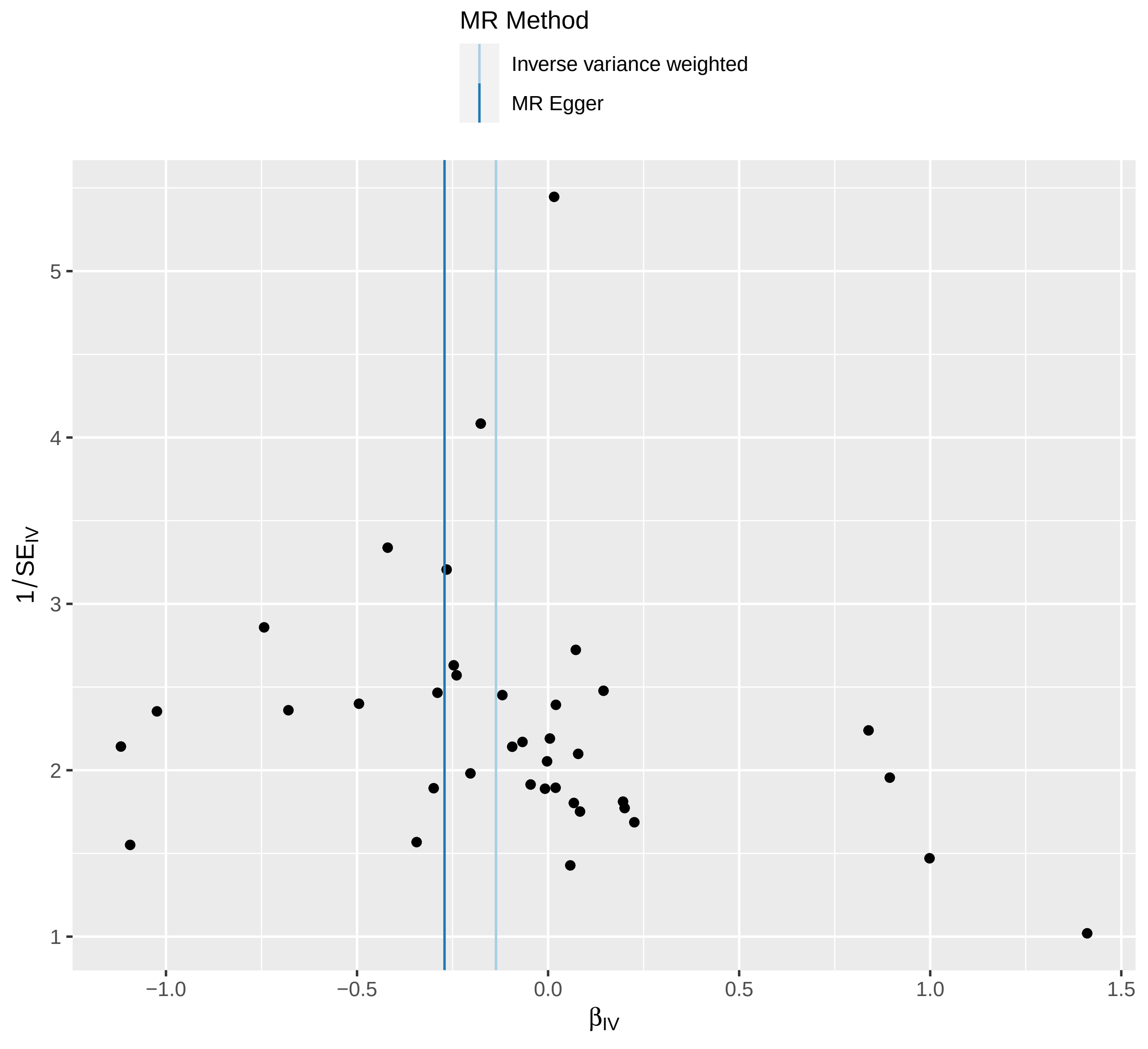

MR Method
Inverse variance weighted
MR Egger
1/SE$_{IV}$
5
4
3
2
1
−1.0
−0.5
0.0
0.5
1.0
1.5
β_{IV}

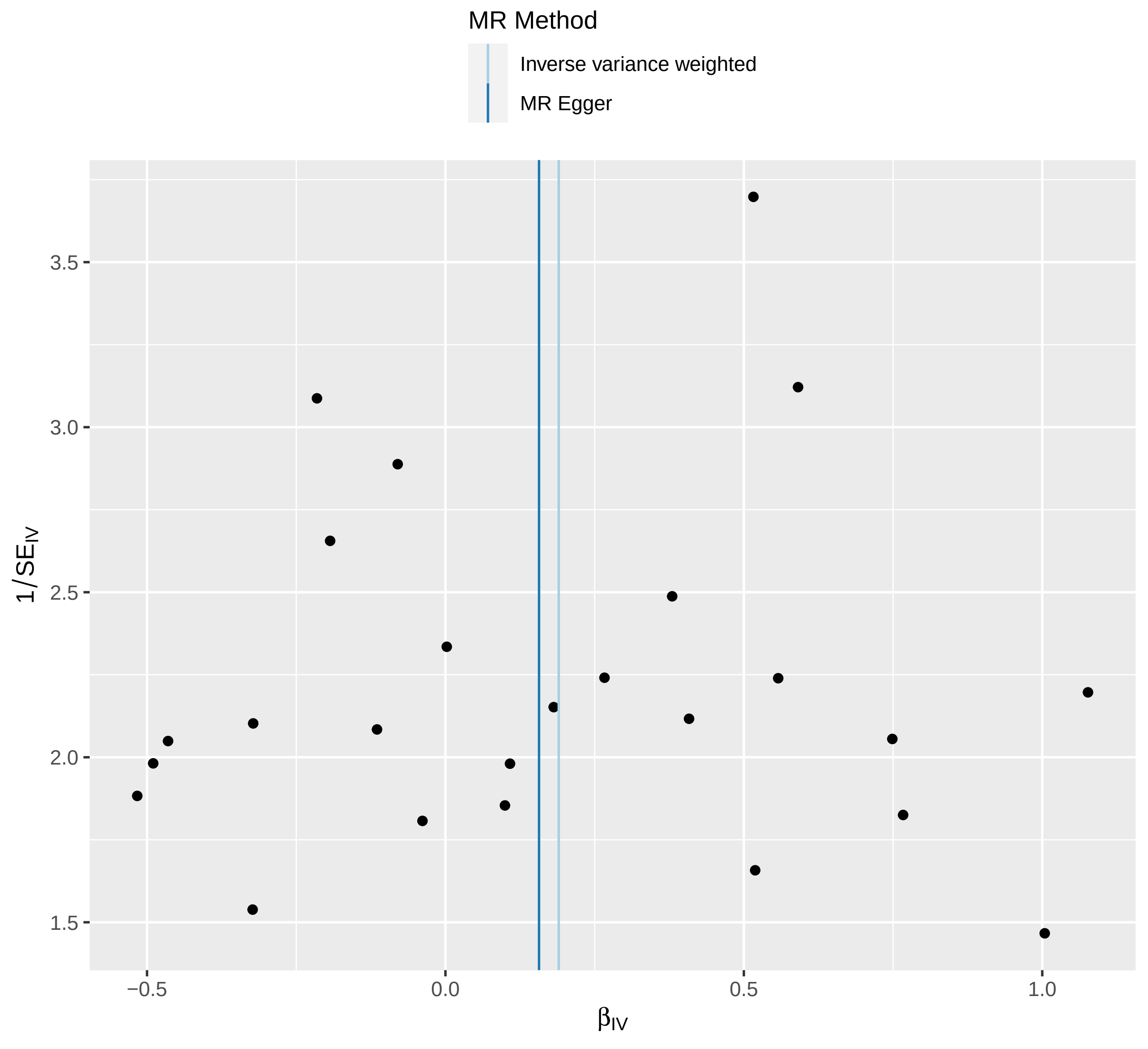

MR Method
Inverse variance weighted
MR Egger
1/SE_IV
β_IV
3.5
3.0
2.5
2.0
1.5
−0.5
0.0
0.5
1.0

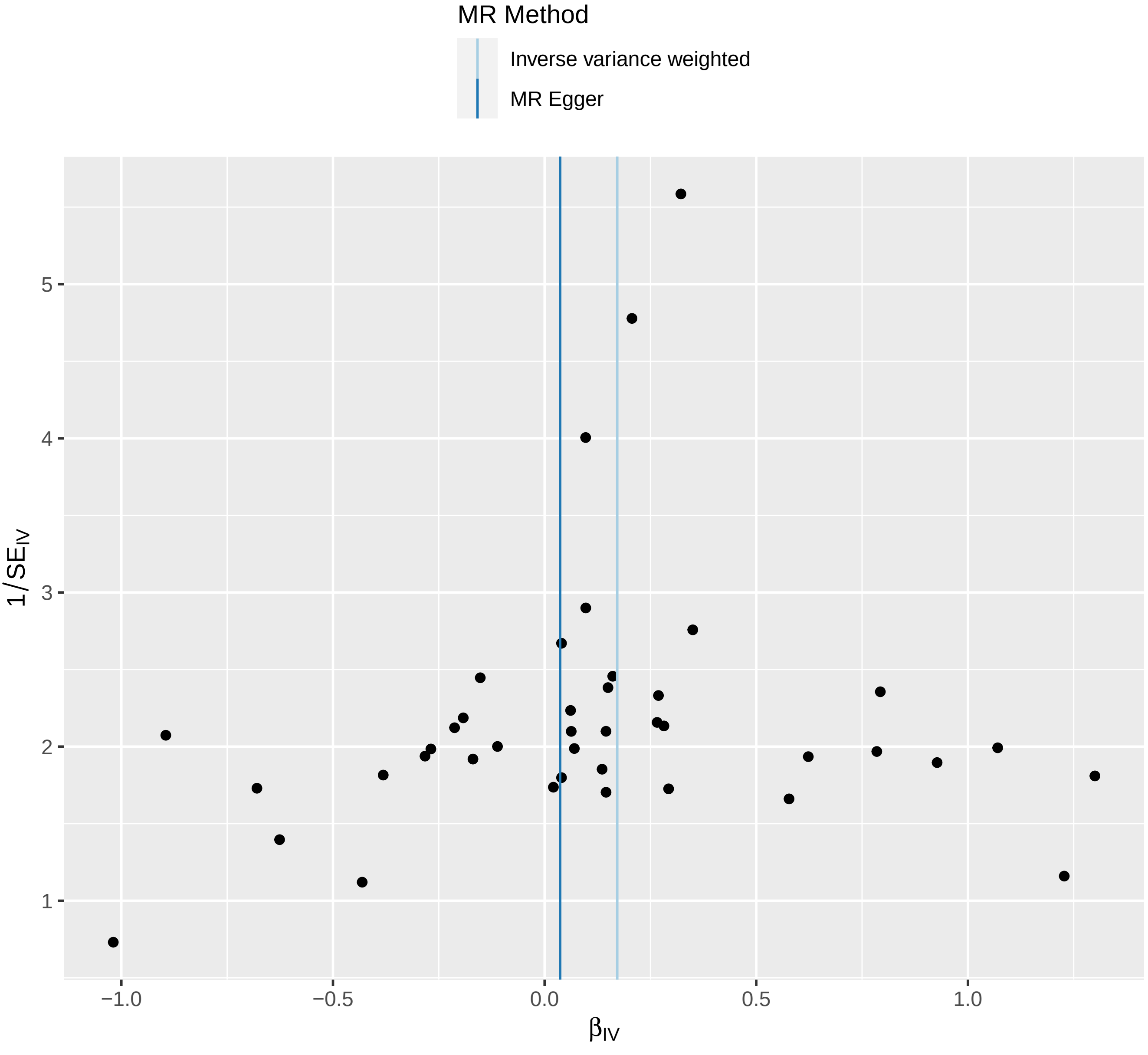
MR Method
Inverse variance weighted
MR Egger
1/SE$_{IV}$
5
4
3
2
1
−1.0
−0.5
0.0
0.5
1.0
β_{IV}

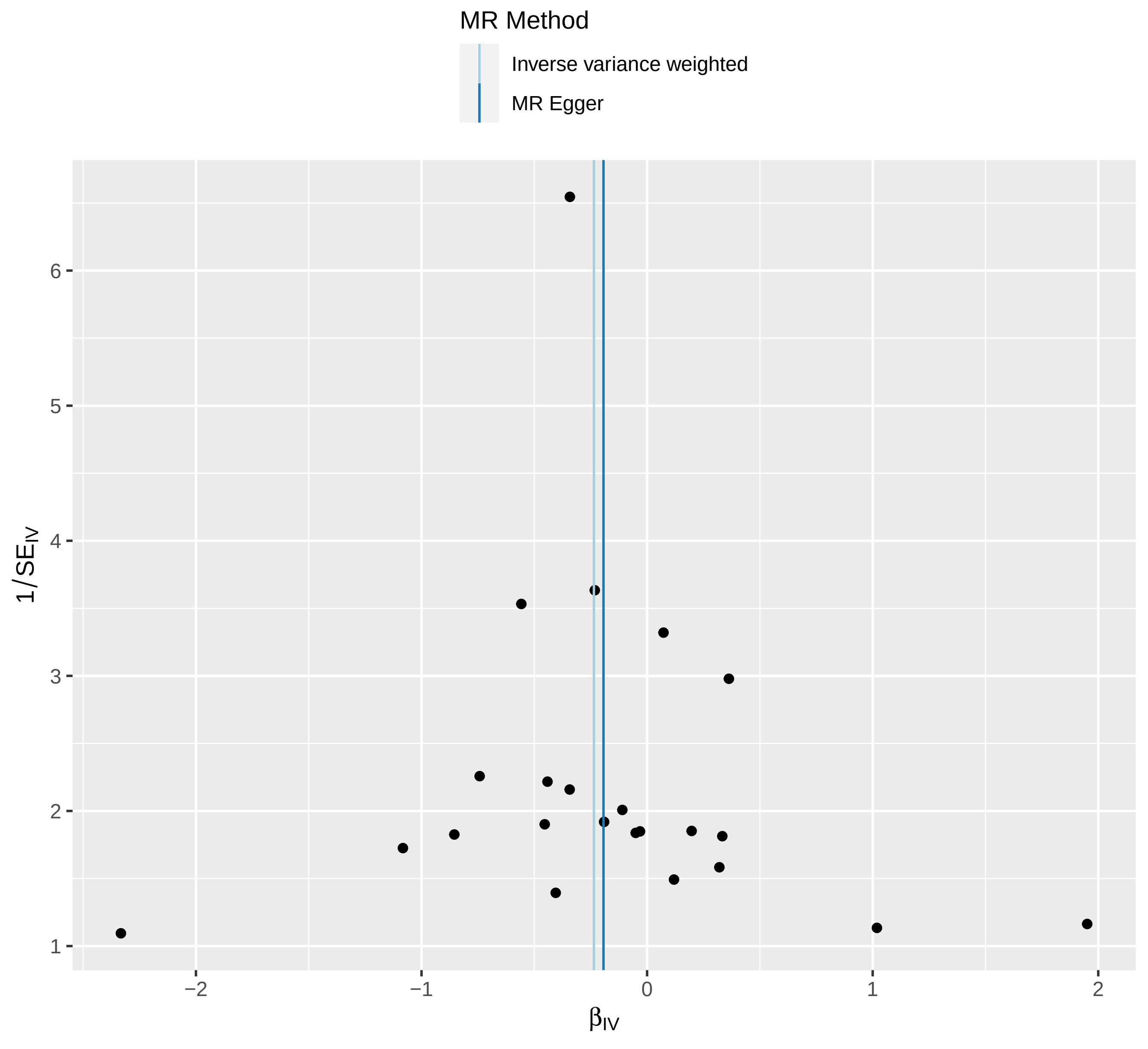
MR Method
Inverse variance weighted
MR Egger
1/SE_IV
β_IV
6
5
4
3
2
1
−2
−1
0
1
2

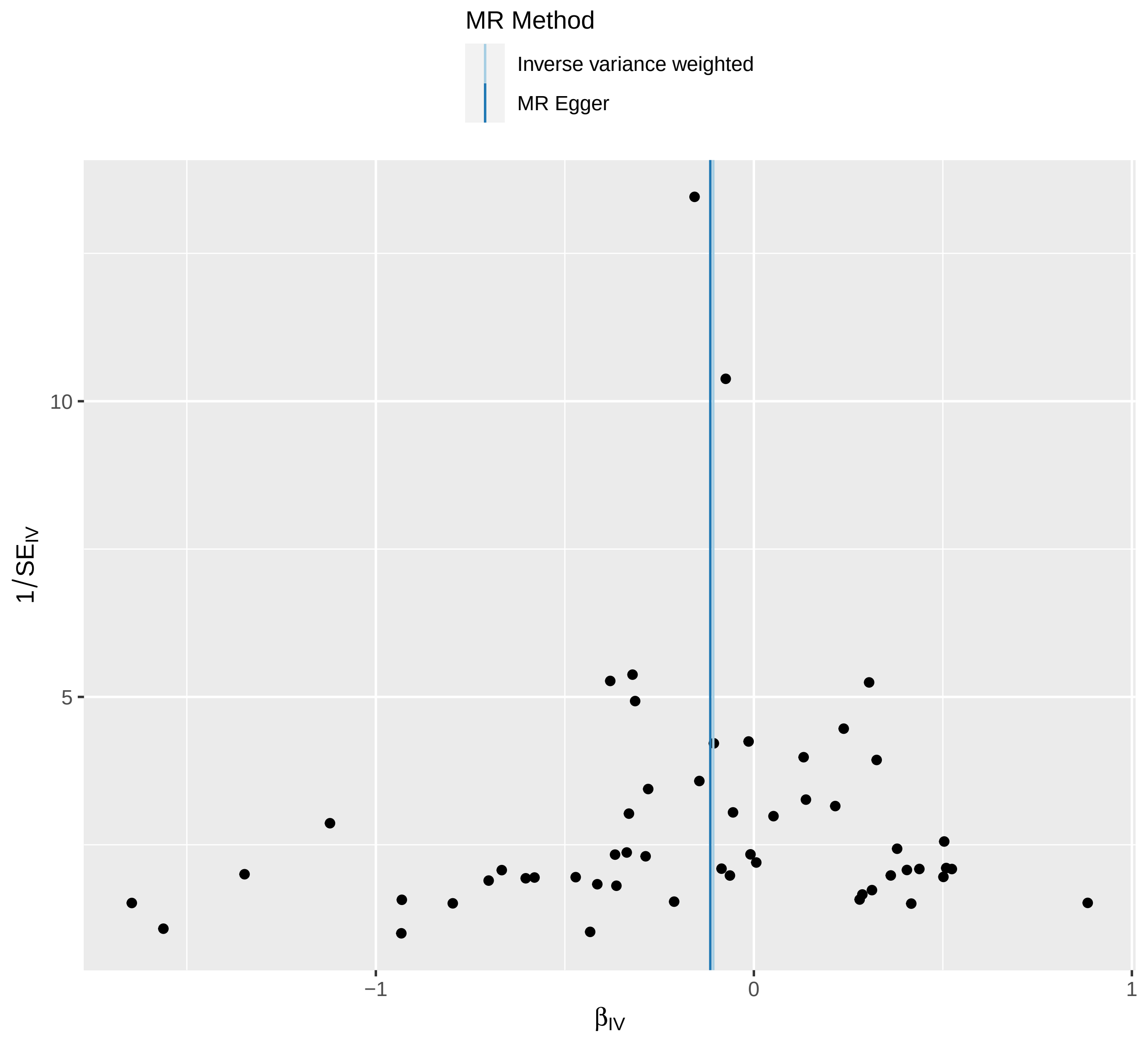

MR Method
Inverse variance weighted
MR Egger
10
5
1/SE$_{IV}$
−1
0
1
β$_{IV}$

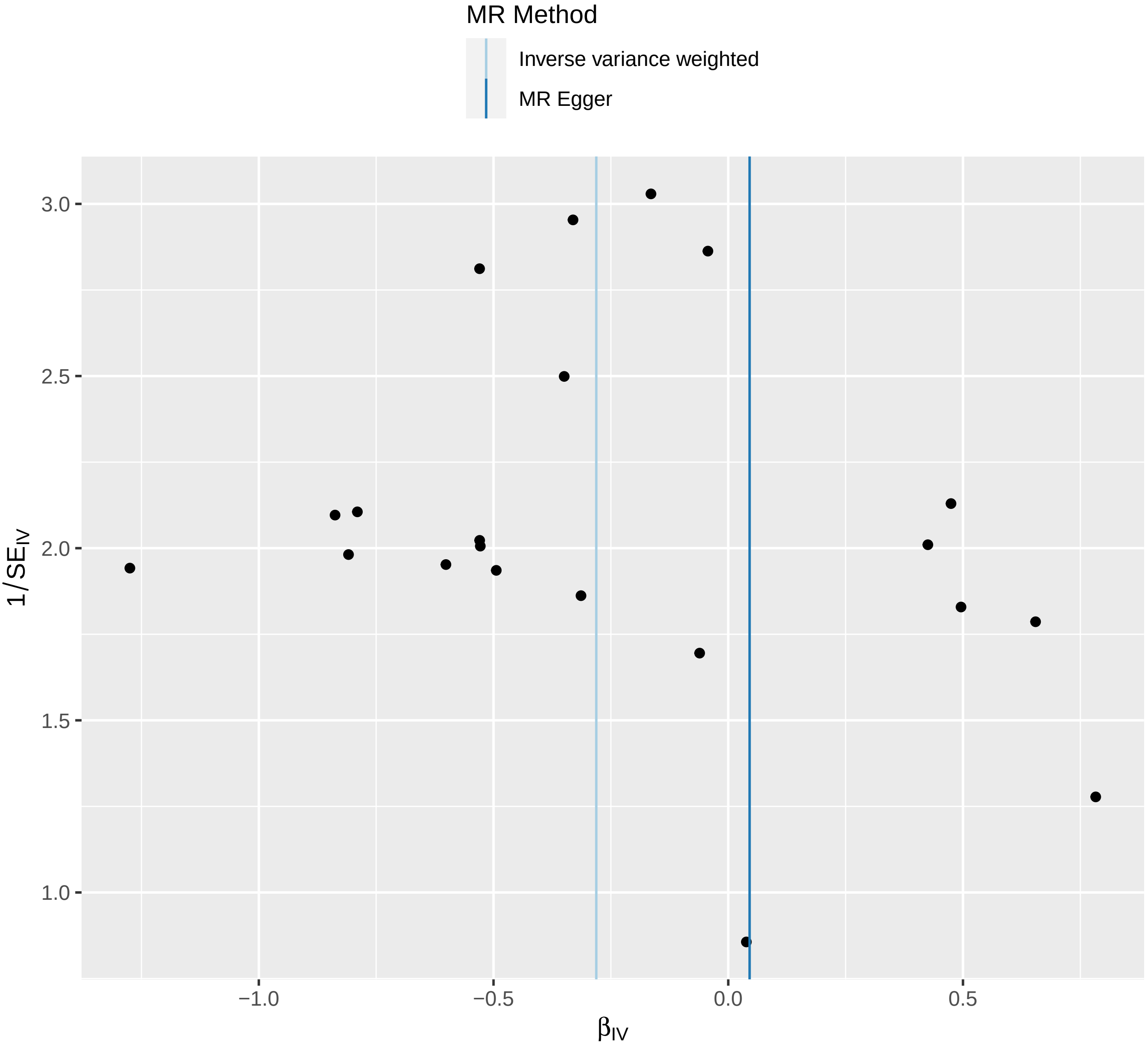
MR Method
Inverse variance weighted
MR Egger
3.0
2.5
2.0
1.5
1.0
1/SE_{IV}
−1.0
−0.5
0.0
0.5
β_{IV}

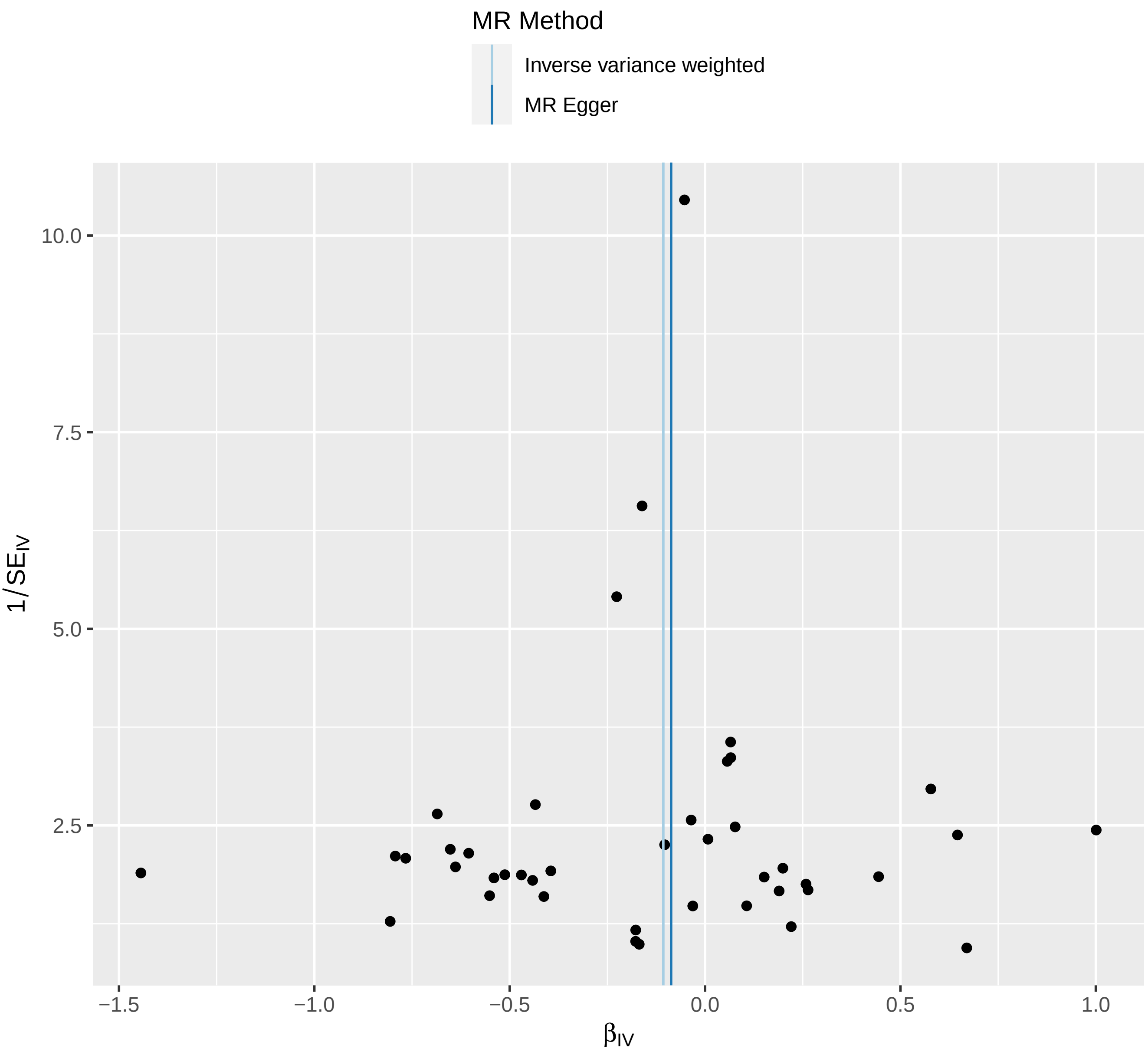
MR Method
Inverse variance weighted
MR Egger
10.0
7.5
5.0
2.5
1/SE_IV
−1.5
−1.0
−0.5
0.0
0.5
1.0
β_IV

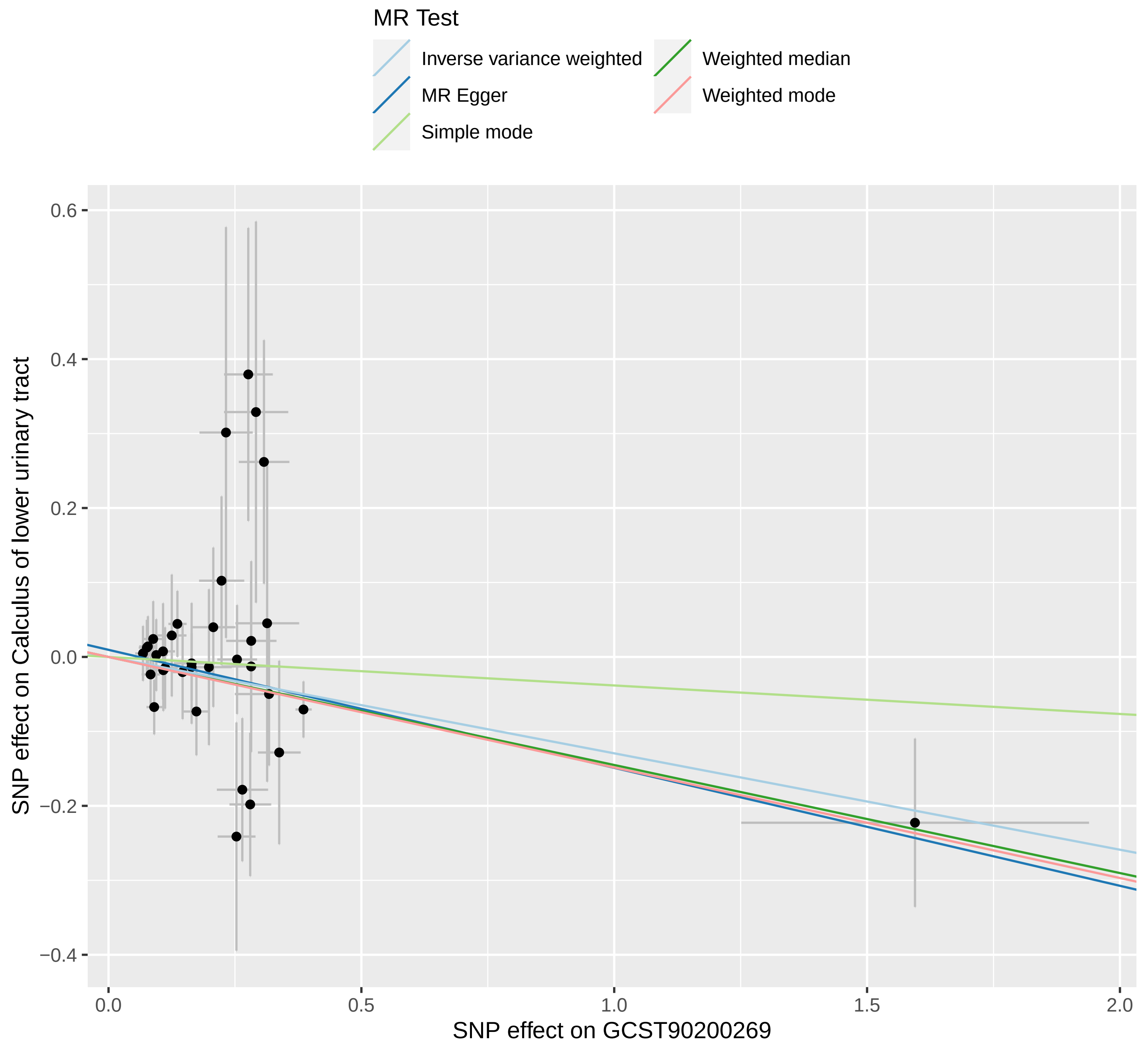

MR Test
Inverse variance weighted
MR Egger
Simple mode
Weighted median
Weighted mode
0.6
0.4
0.2
0.0
−0.2
−0.4
SNP effect on Calculus of lower urinary tract
0.0
0.5
1.0
1.5
2.0
SNP effect on GCST90200269

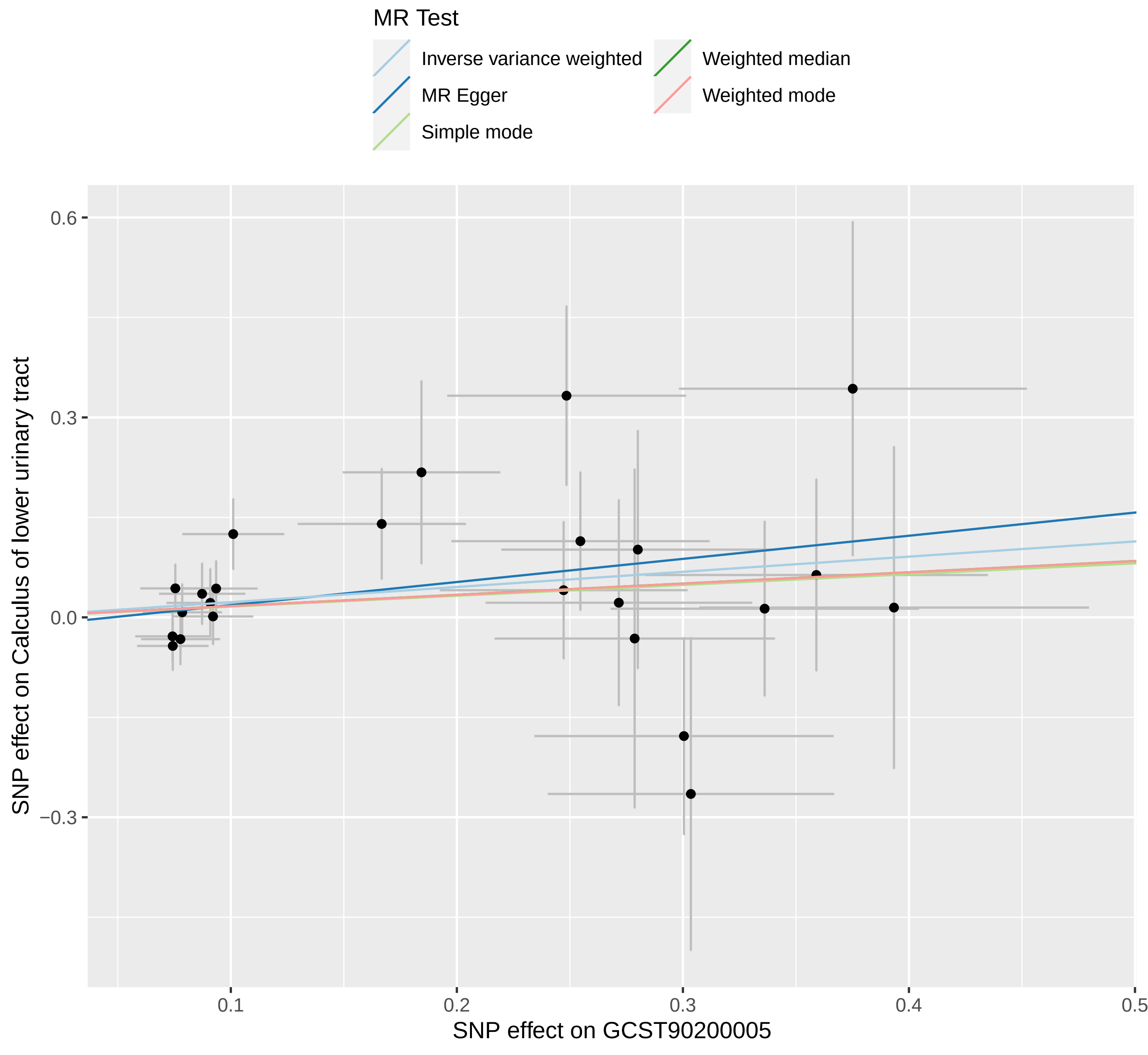

MR Test
Inverse variance weighted
MR Egger
Simple mode
Weighted median
Weighted mode
SNP effect on Calculus of lower urinary tract
0.6
0.3
0.0
−0.3
0.1
0.2
0.3
0.4
0.5
SNP effect on GCST90200005

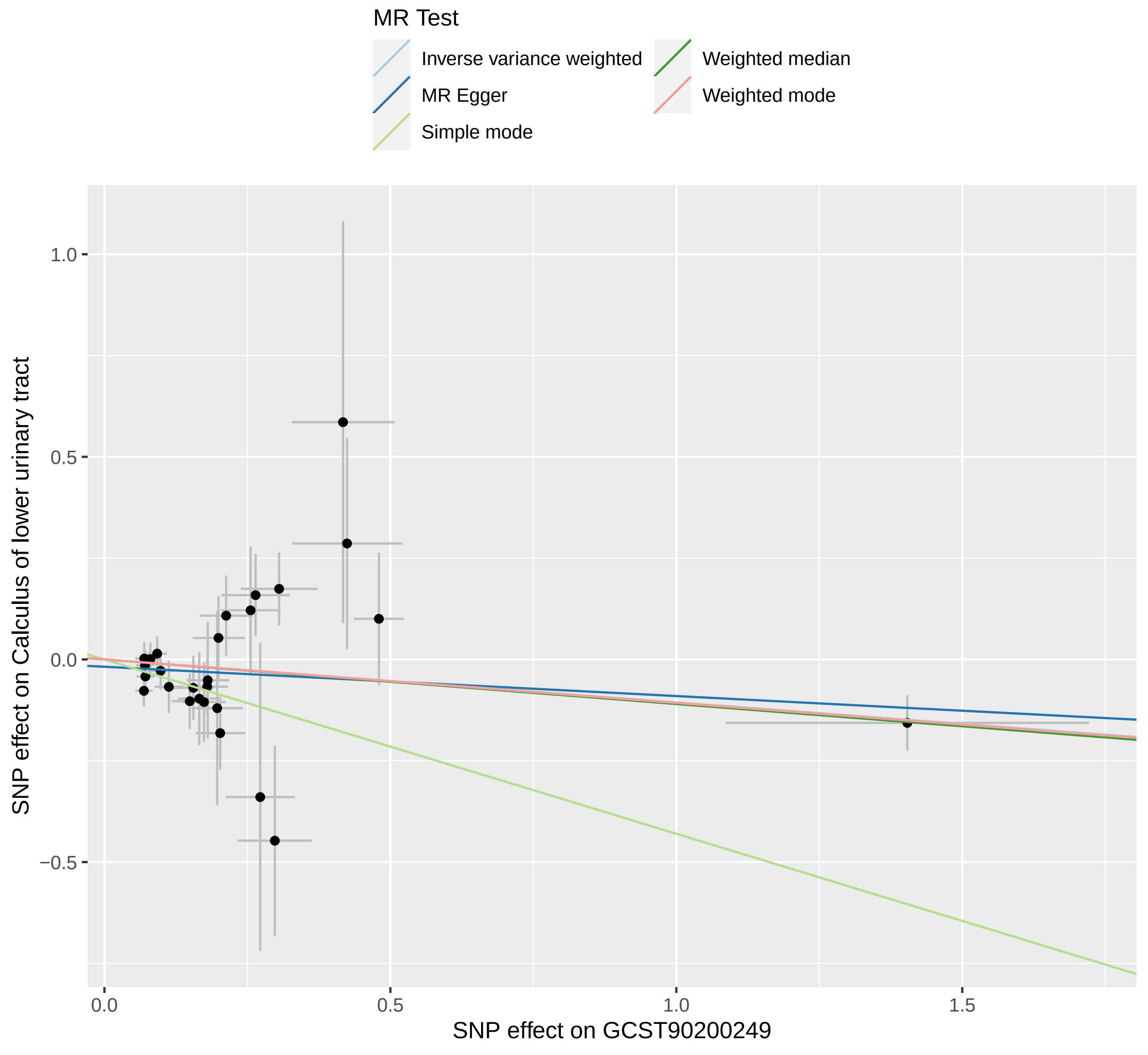
MR Test
Inverse variance weighted
MR Egger
Simple mode
Weighted median
Weighted mode
SNP effect on Calculus of lower urinary tract
SNP effect on GCST90200249
1.0
0.5
0.0
−0.5
0.0
0.5
1.0
1.5

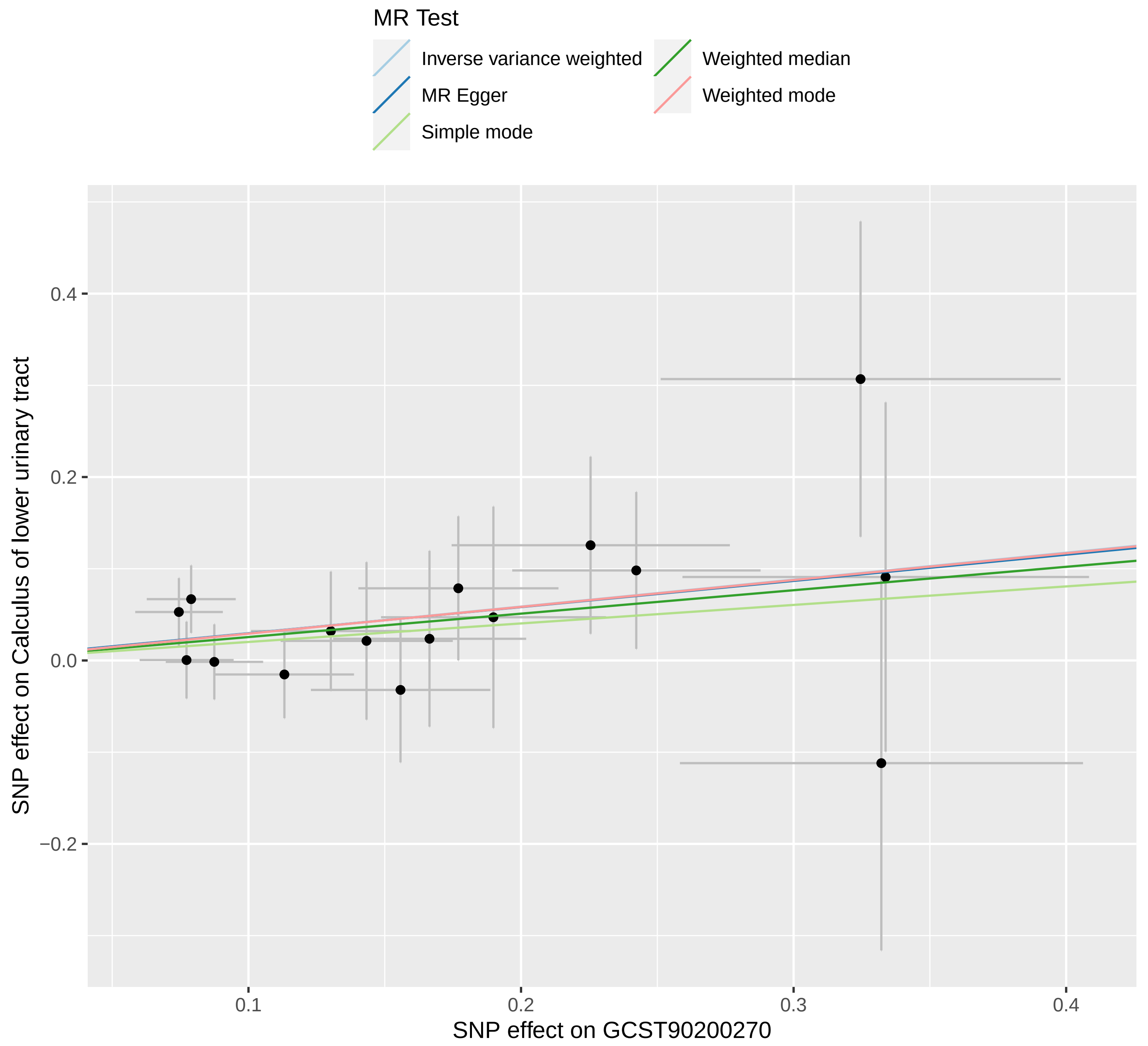

MR Test
Inverse variance weighted
MR Egger
Simple mode
Weighted median
Weighted mode
0.4
0.2
0.0
−0.2
0.1
0.2
0.3
0.4
SNP effect on Calculus of lower urinary tract
SNP effect on GCST90200270

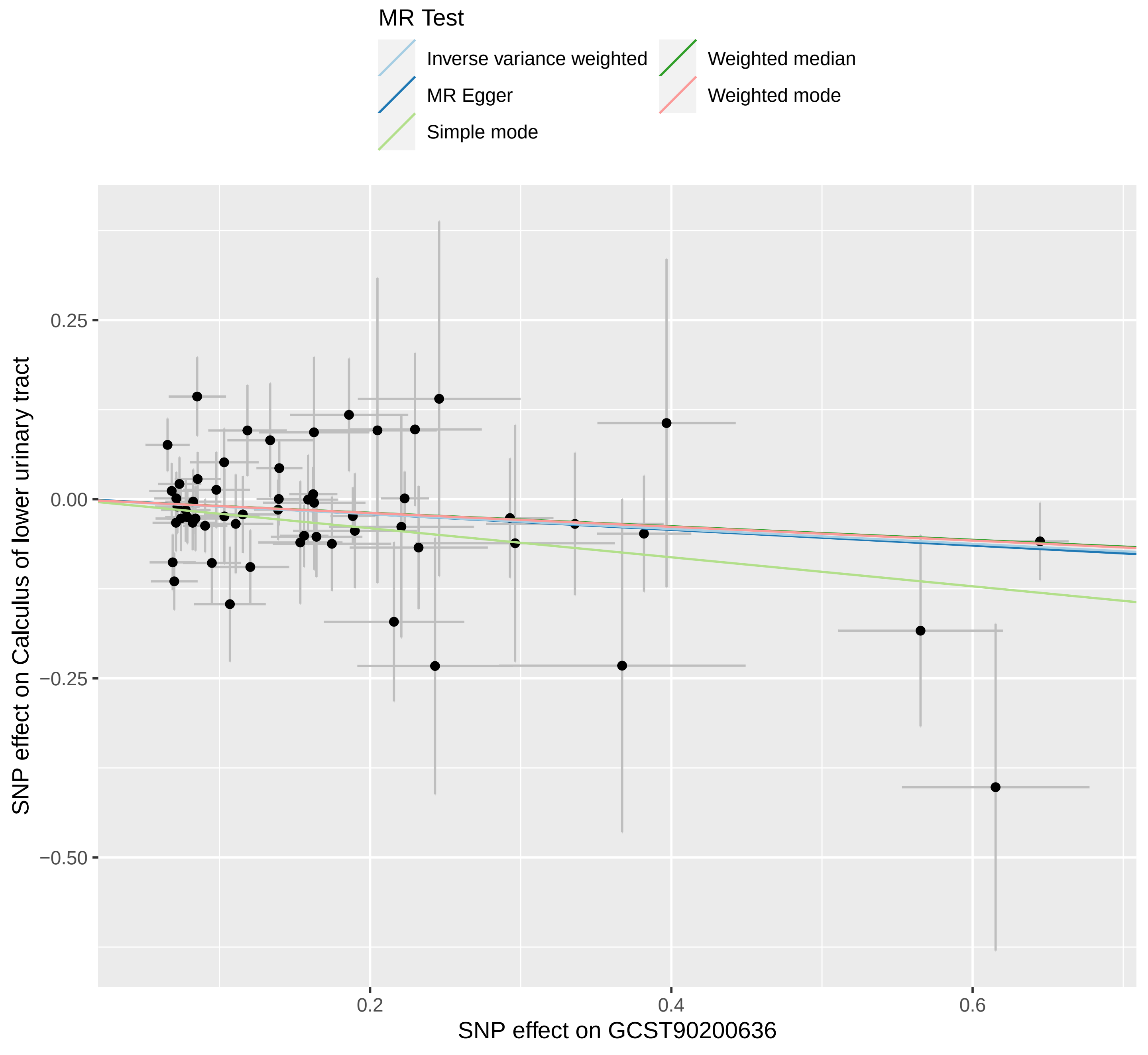
MR Test
Inverse variance weighted
MR Egger
Simple mode
Weighted median
Weighted mode
0.25
0.00
−0.25
−0.50
0.2
0.4
0.6
SNP effect on Calculus of lower urinary tract
SNP effect on GCST90200636

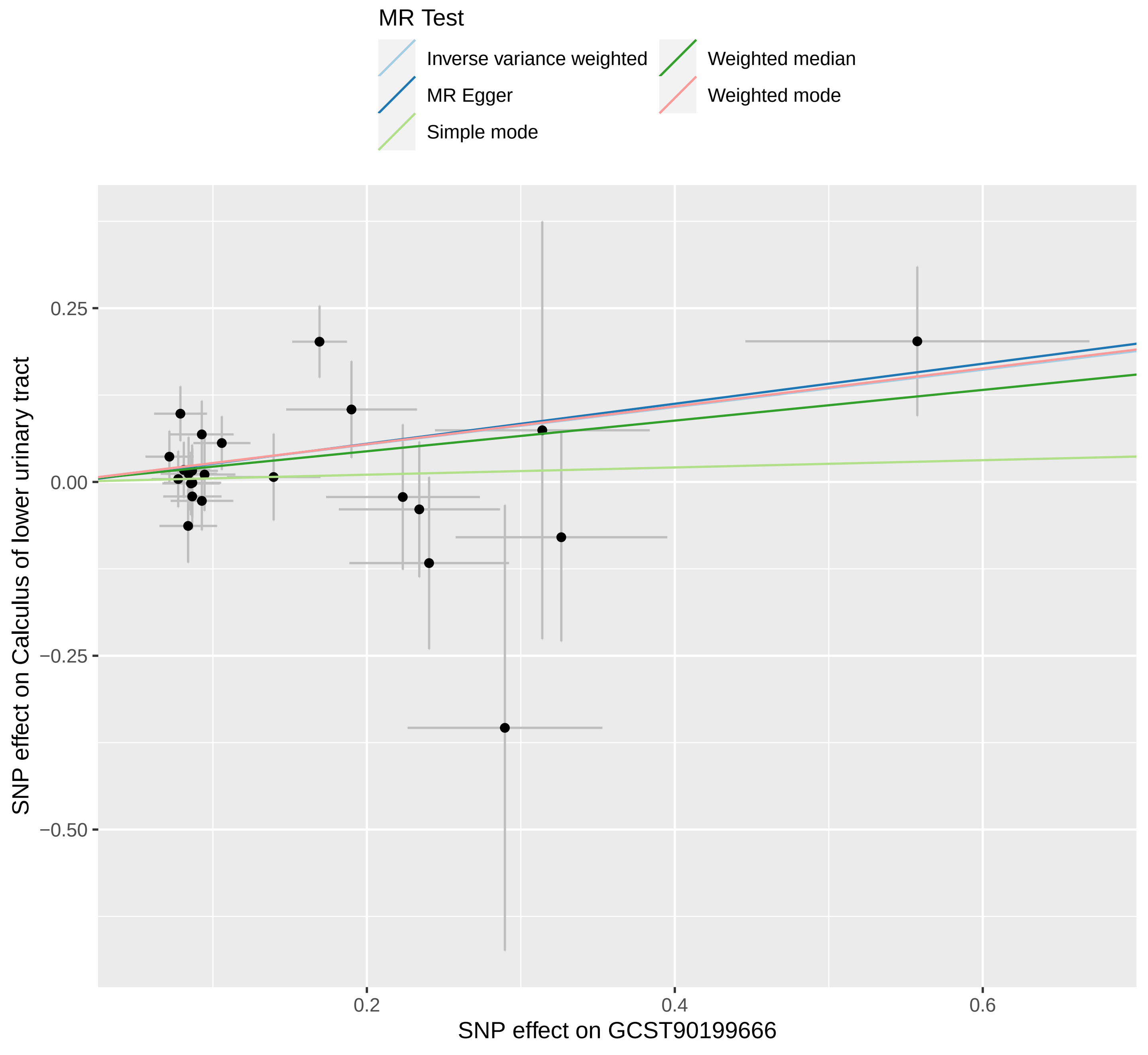
MR Test
Inverse variance weighted
MR Egger
Simple mode
Weighted median
Weighted mode
0.25
0.00
−0.25
−0.50
0.2
0.4
0.6
SNP effect on Calculus of lower urinary tract
SNP effect on GCST90199666

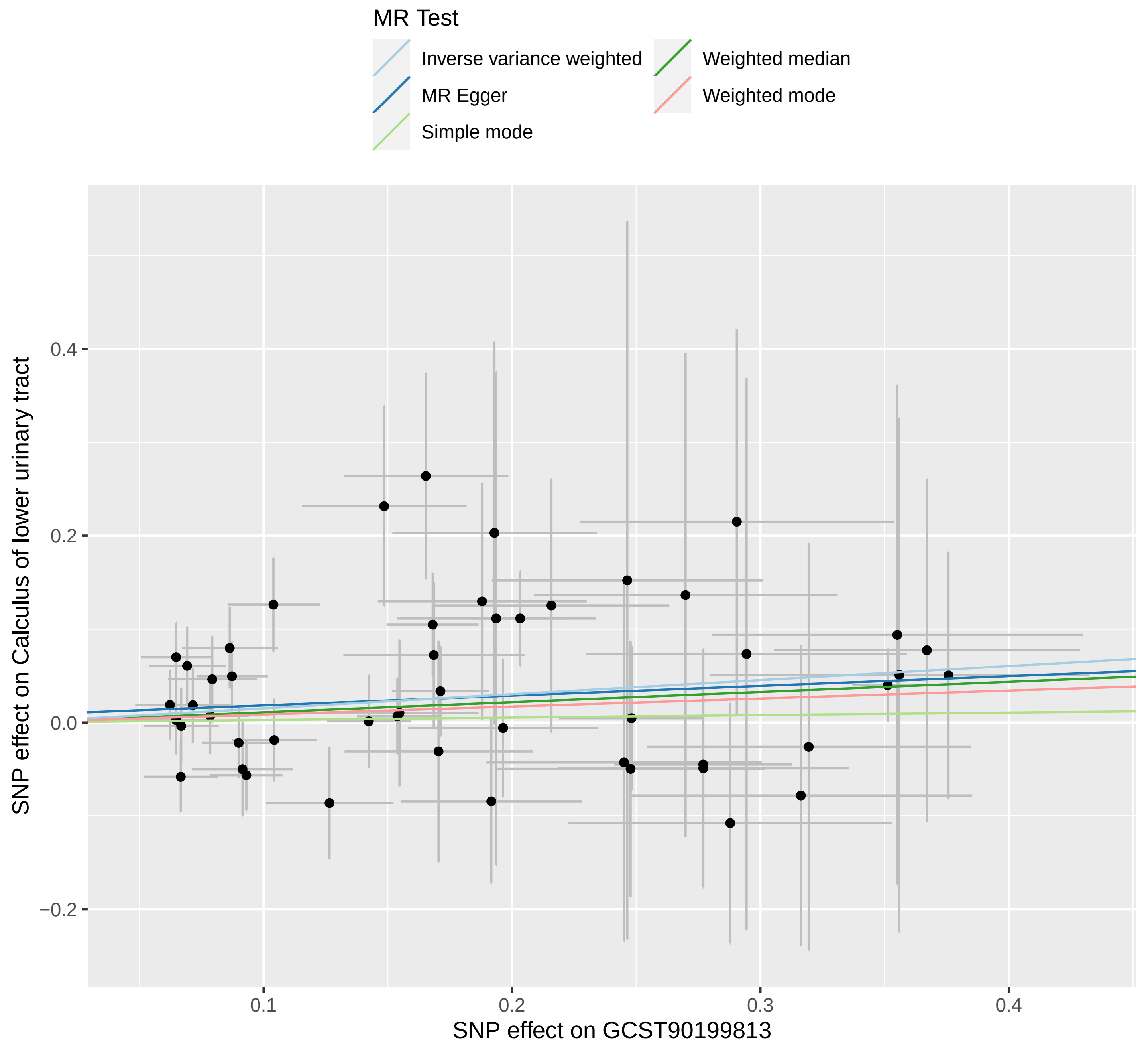

MR Test
Inverse variance weighted
MR Egger
Simple mode
Weighted median
Weighted mode
SNP effect on Calculus of lower urinary tract
0.4
0.2
0.0
−0.2
0.1
0.2
0.3
0.4
SNP effect on GCST90199813

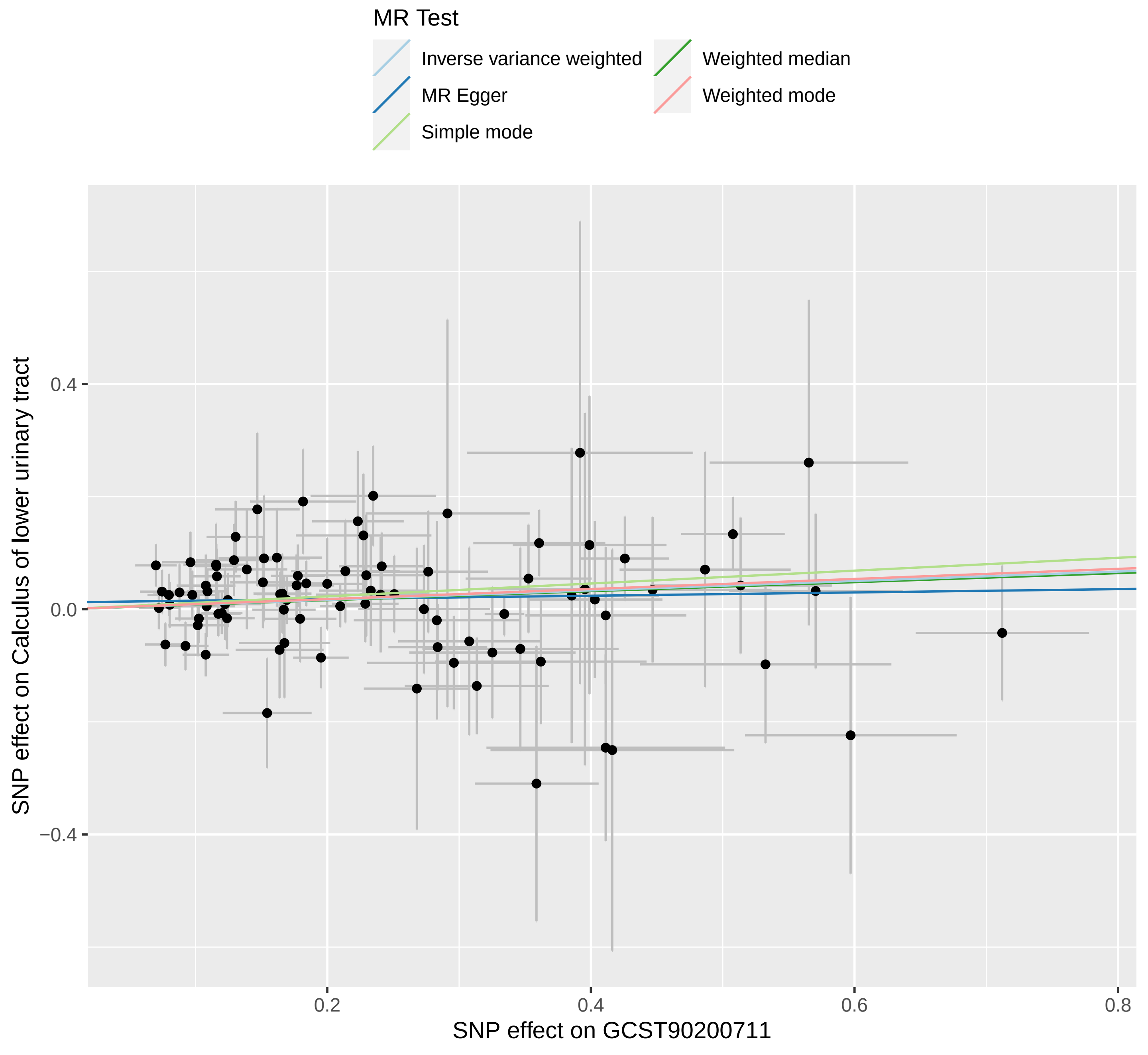
MR Test
Inverse variance weighted
MR Egger
Simple mode
Weighted median
Weighted mode
0.4
0.0
−0.4
0.2
0.4
0.6
0.8
SNP effect on Calculus of lower urinary tract
SNP effect on GCST90200711

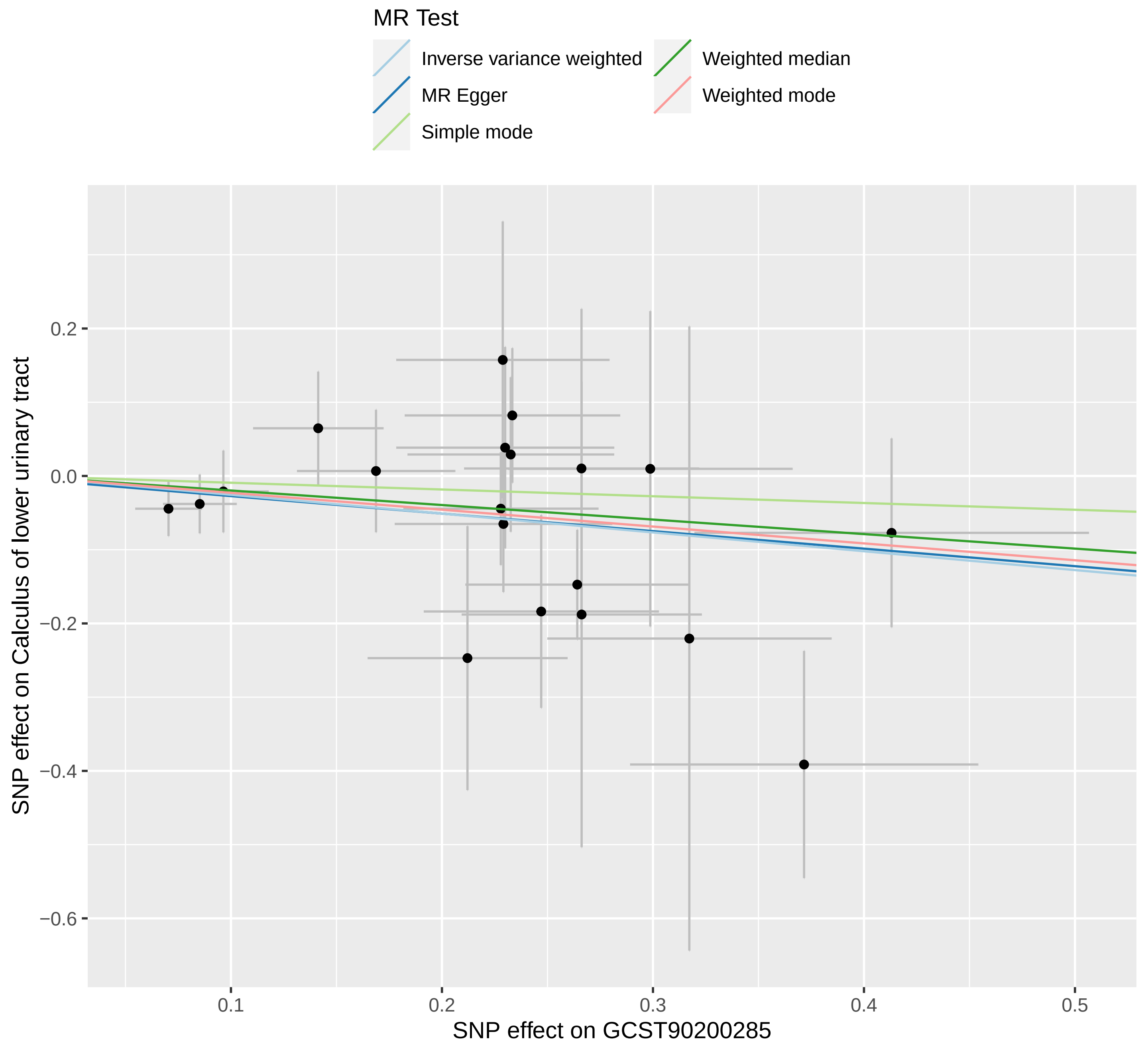
MR Test
Inverse variance weighted
MR Egger
Simple mode
Weighted median
Weighted mode
SNP effect on Calculus of lower urinary tract
0.2
0.0
−0.2
−0.4
−0.6
0.1
0.2
0.3
0.4
0.5
SNP effect on GCST90200285

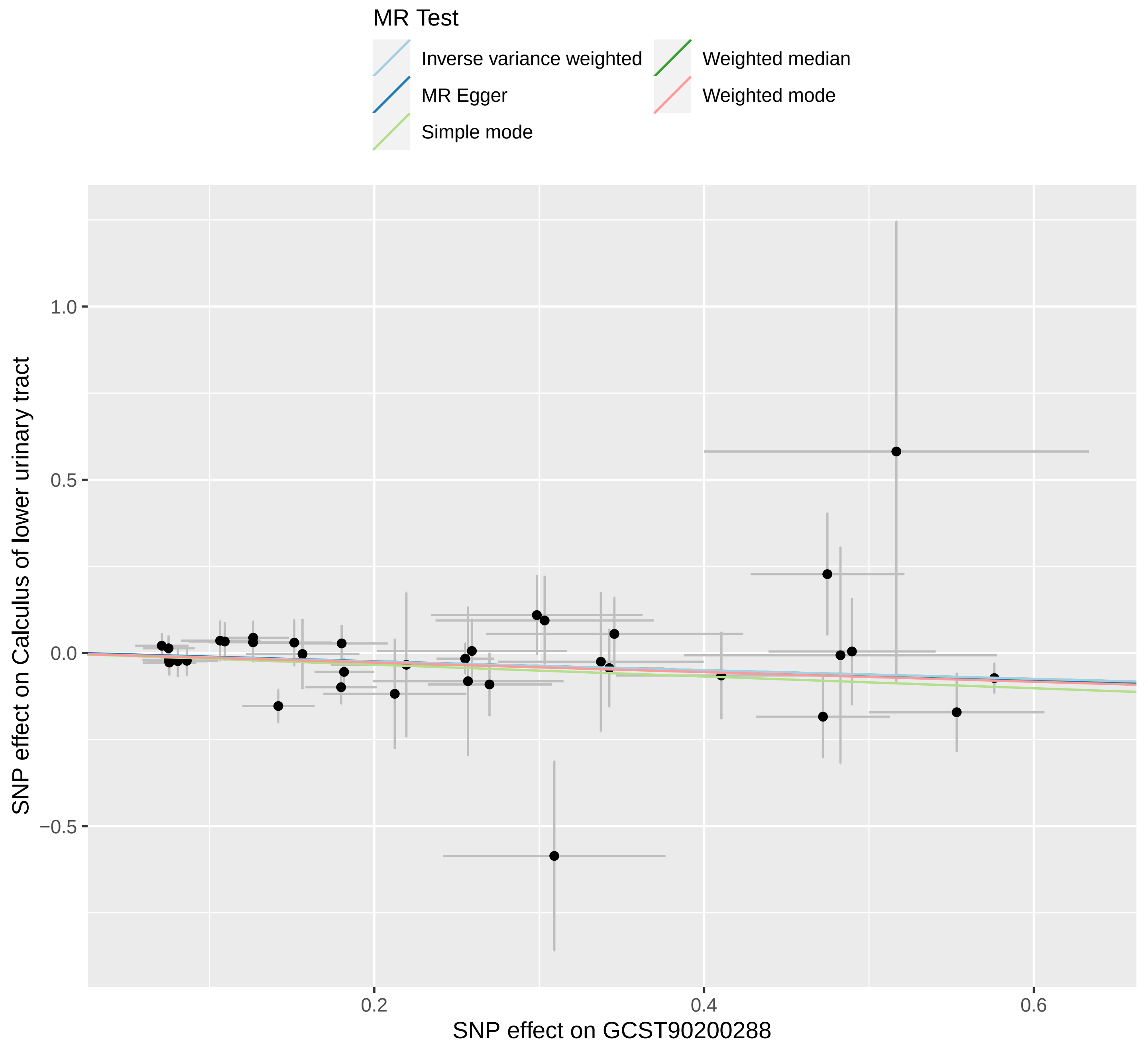

MR Test
Inverse variance weighted
MR Egger
Simple mode
Weighted median
Weighted mode
1.0
0.5
0.0
−0.5
0.2
0.4
0.6
SNP effect on Calculus of lower urinary tract
SNP effect on GCST90200288

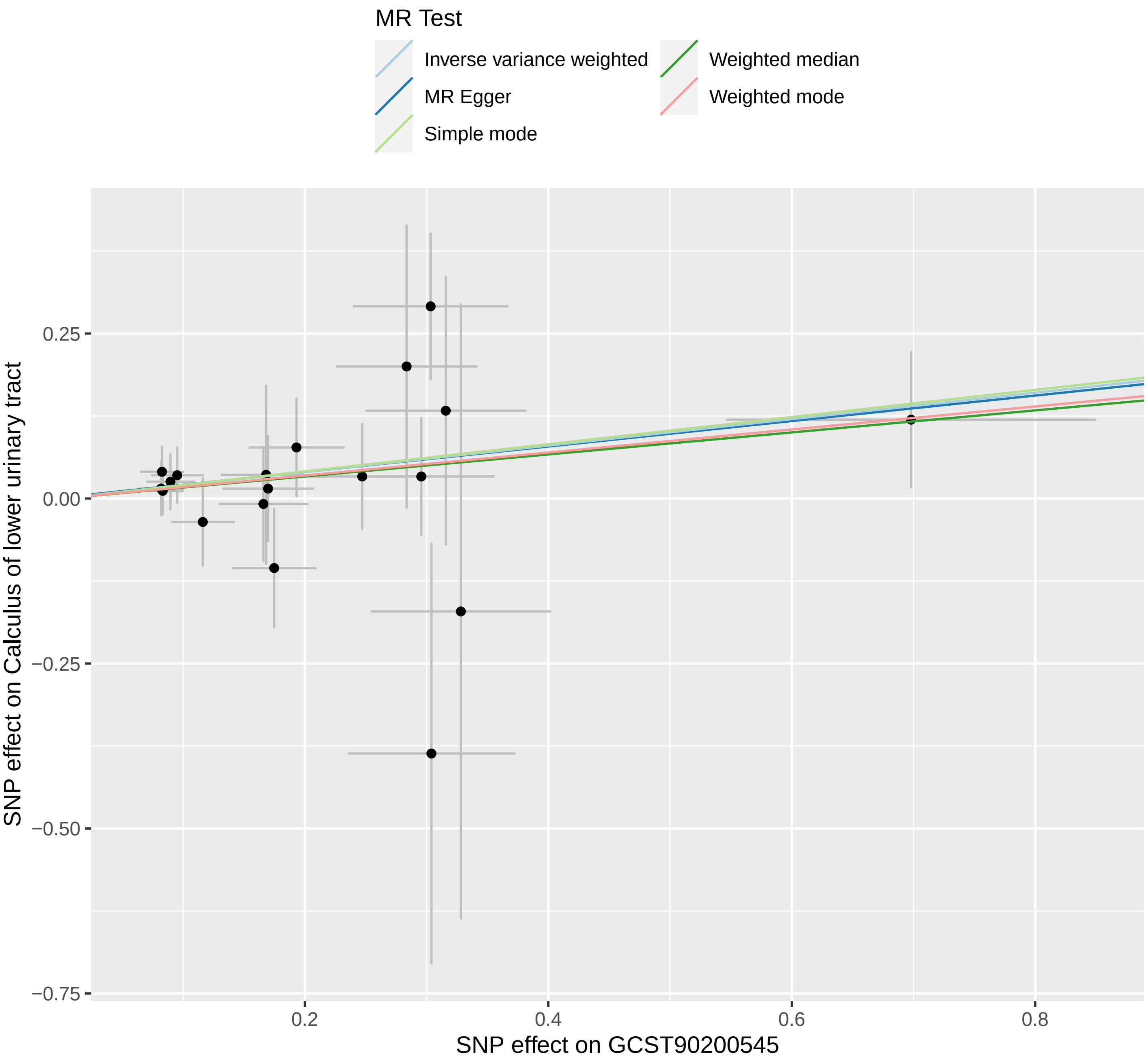

MR Test
Inverse variance weighted
MR Egger
Simple mode
Weighted median
Weighted mode
SNP effect on Calculus of lower urinary tract
0.25
0.00
−0.25
−0.50
−0.75
0.2
0.4
0.6
0.8
SNP effect on GCST90200545

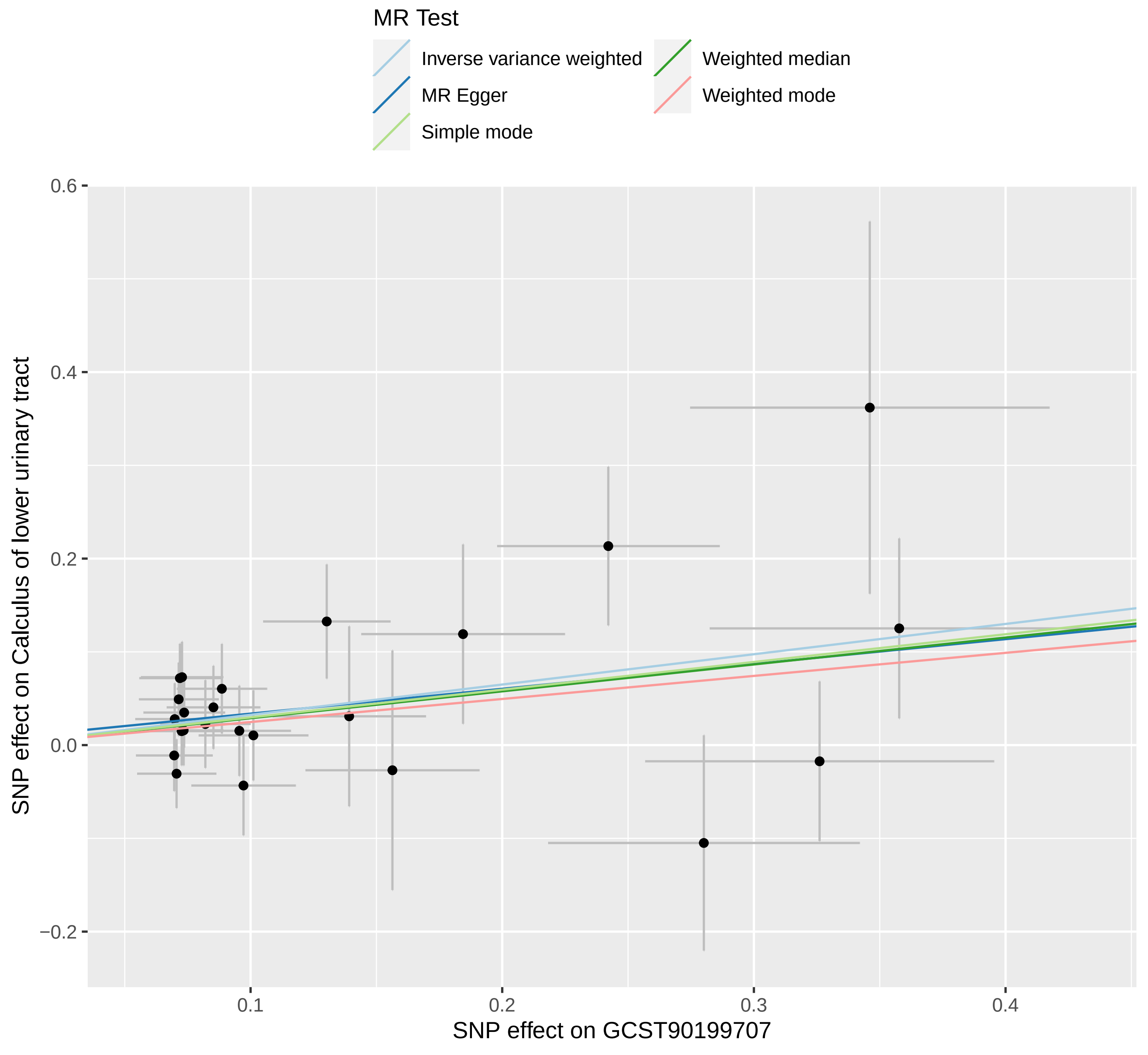
MR Test
Inverse variance weighted
MR Egger
Simple mode
Weighted median
Weighted mode
0.6
0.4
0.2
0.0
−0.2
0.1
0.2
0.3
0.4
SNP effect on Calculus of lower urinary tract
SNP effect on GCST90199707

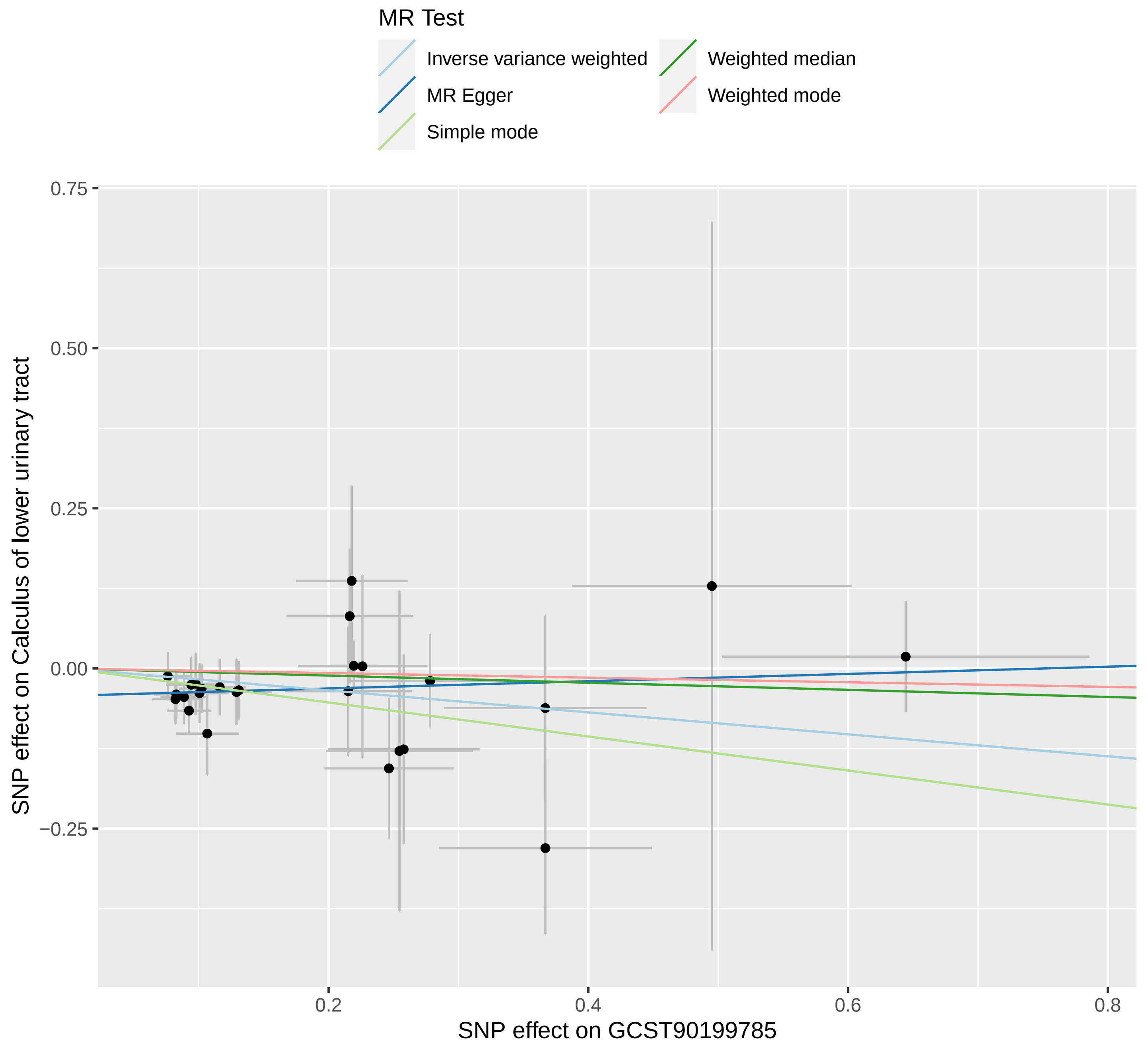

MR Test
Inverse variance weighted
MR Egger
Simple mode
Weighted median
Weighted mode
0.75
0.50
0.25
0.00
−0.25
0.2
0.4
0.6
0.8
SNP effect on Calculus of lower urinary tract
SNP effect on GCST90199785

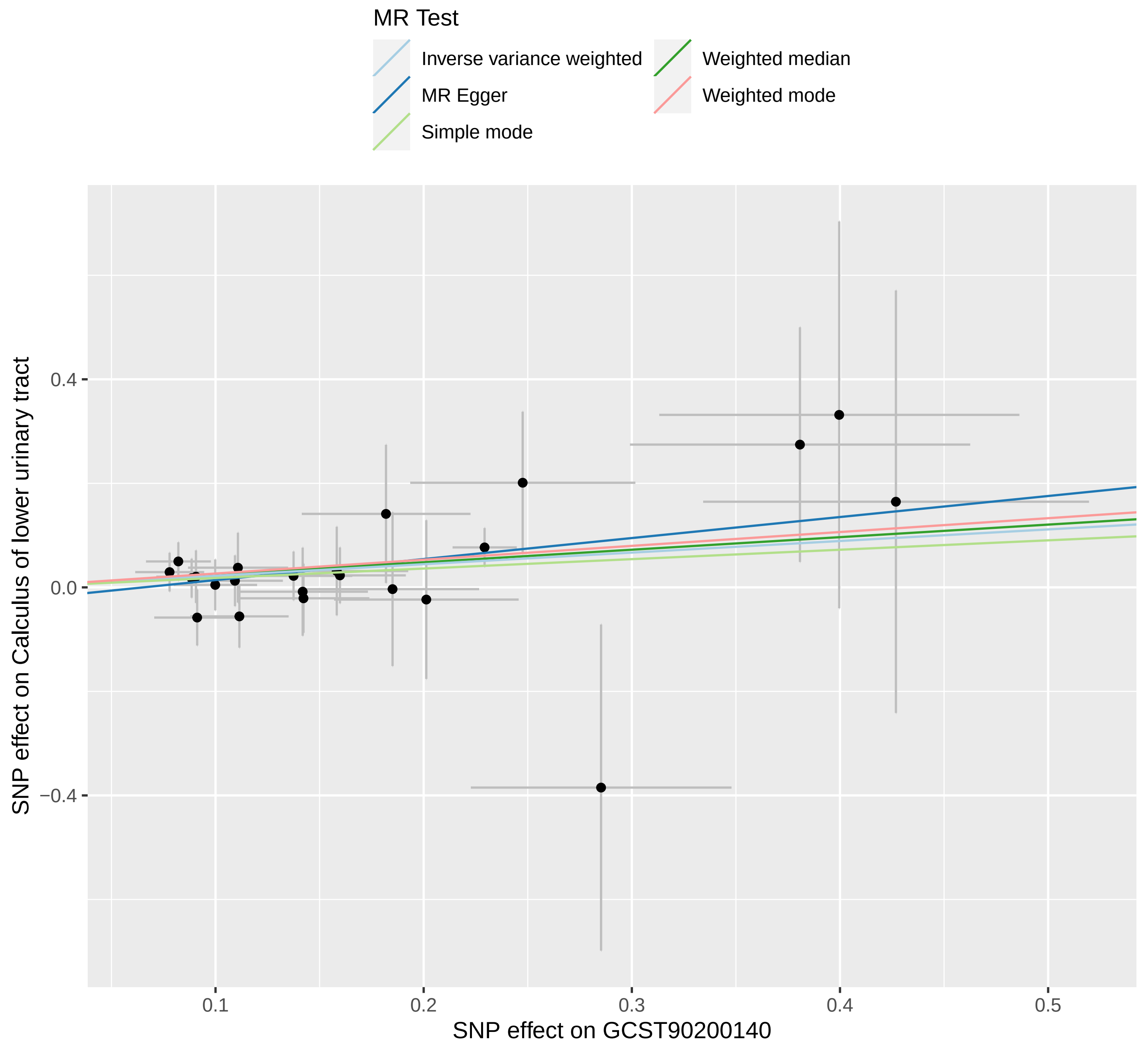

MR Test
Inverse variance weighted
MR Egger
Simple mode
Weighted median
Weighted mode
0.4
0.0
−0.4
0.1
0.2
0.3
0.4
0.5
SNP effect on GCST90200140
SNP effect on Calculus of lower urinary tract

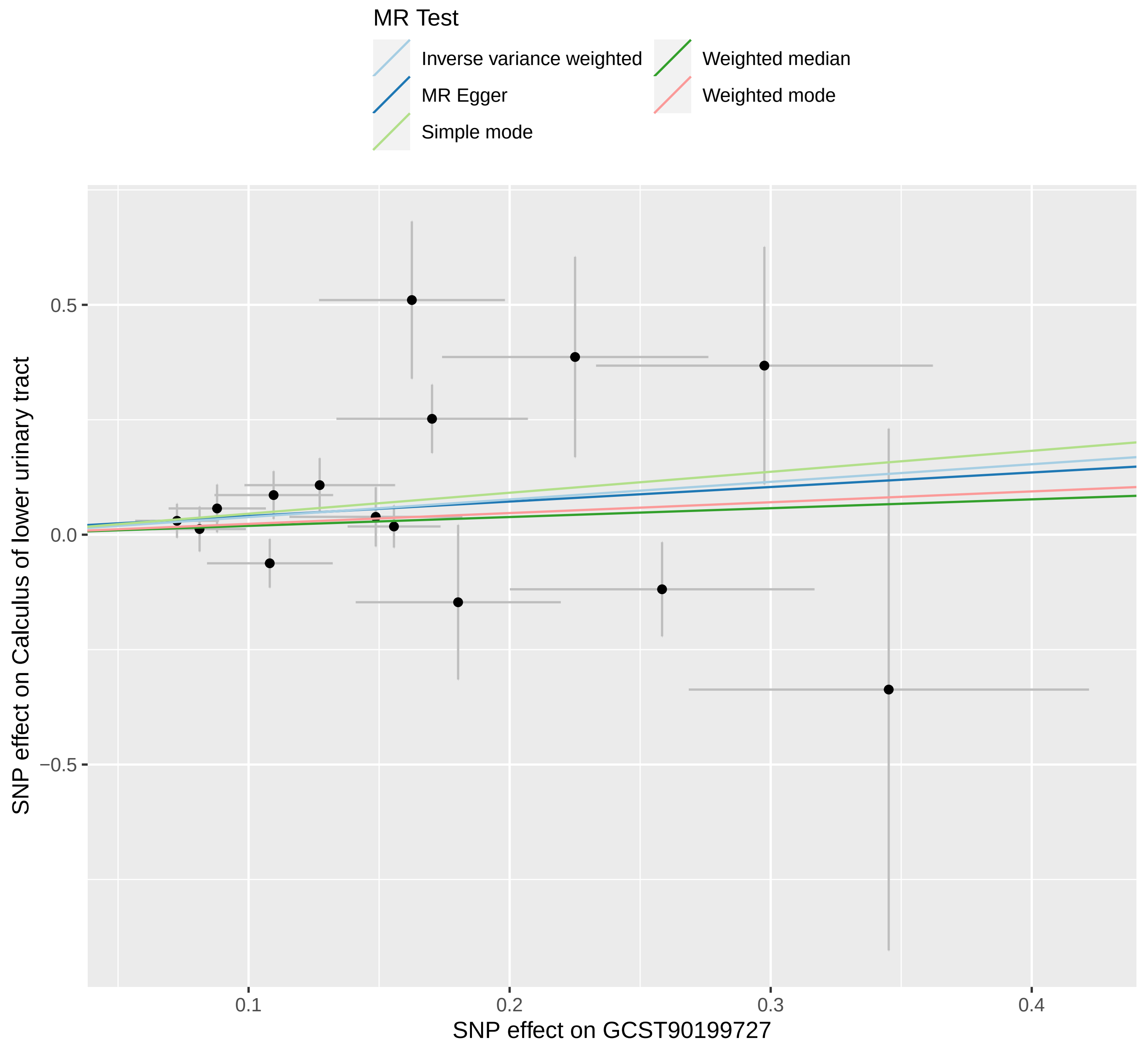
MR Test
Inverse variance weighted
MR Egger
Simple mode
Weighted median
Weighted mode
0.5
0.0
−0.5
0.1
0.2
0.3
0.4
SNP effect on Calculus of lower urinary tract
SNP effect on GCST90199727

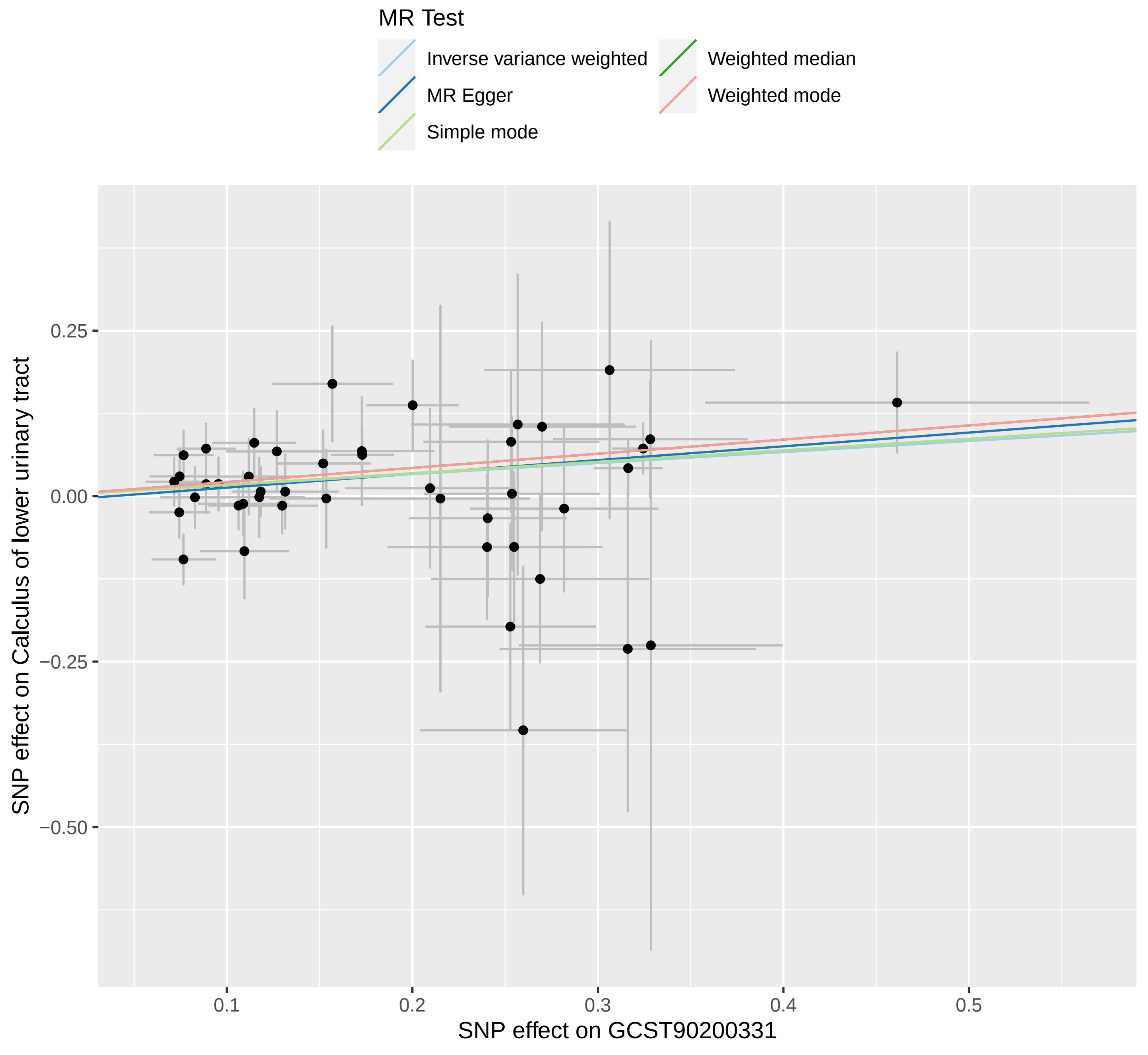

MR Test
Inverse variance weighted
MR Egger
Simple mode
Weighted median
Weighted mode
0.25
0.00
−0.25
−0.50
0.1
0.2
0.3
0.4
0.5
SNP effect on Calculus of lower urinary tract
SNP effect on GCST90200331

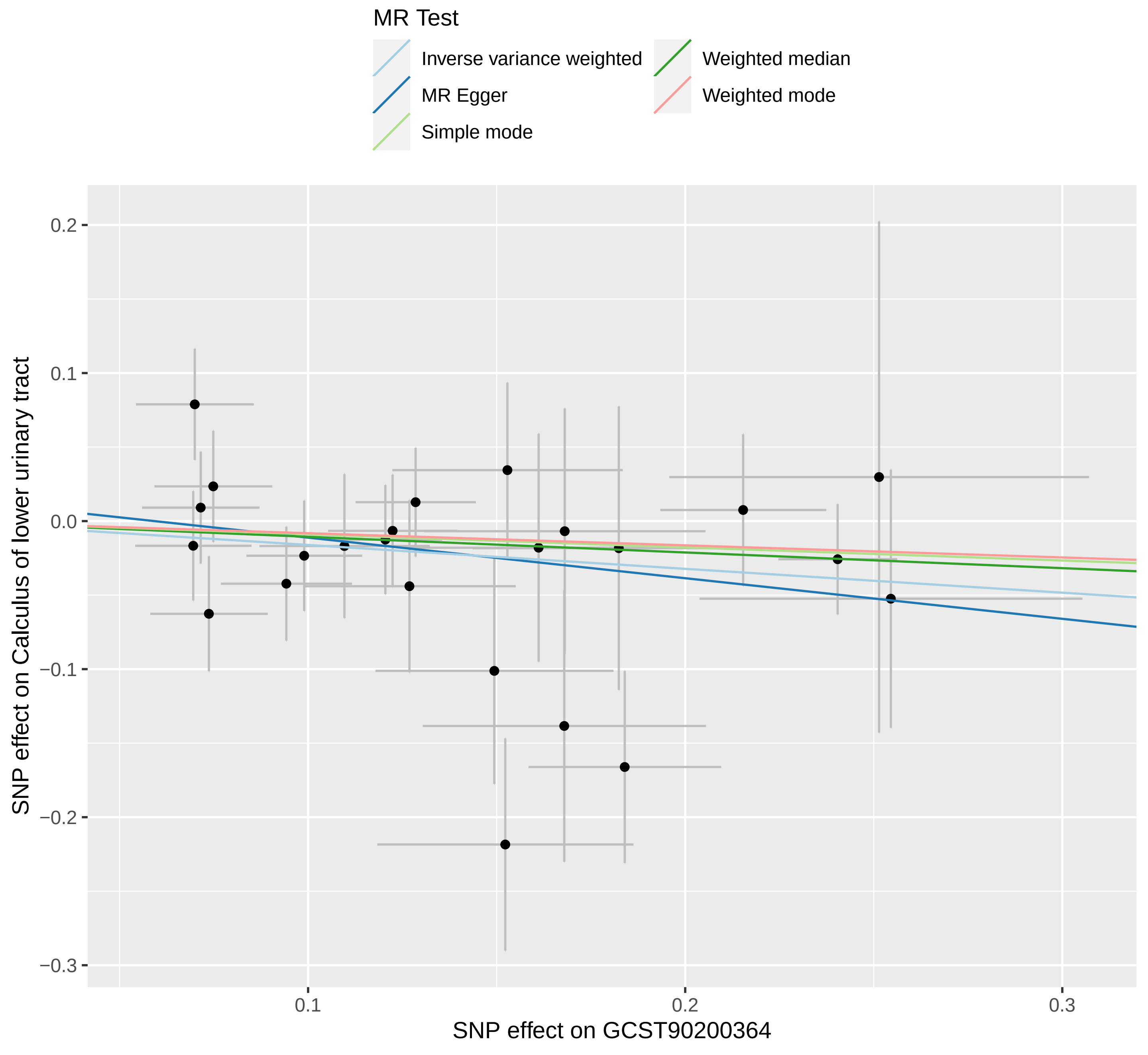

MR Test
Inverse variance weighted
MR Egger
Simple mode
Weighted median
Weighted mode
SNP effect on Calculus of lower urinary tract
0.2
0.1
0.0
−0.1
−0.2
−0.3
0.1
0.2
0.3
SNP effect on GCST90200364

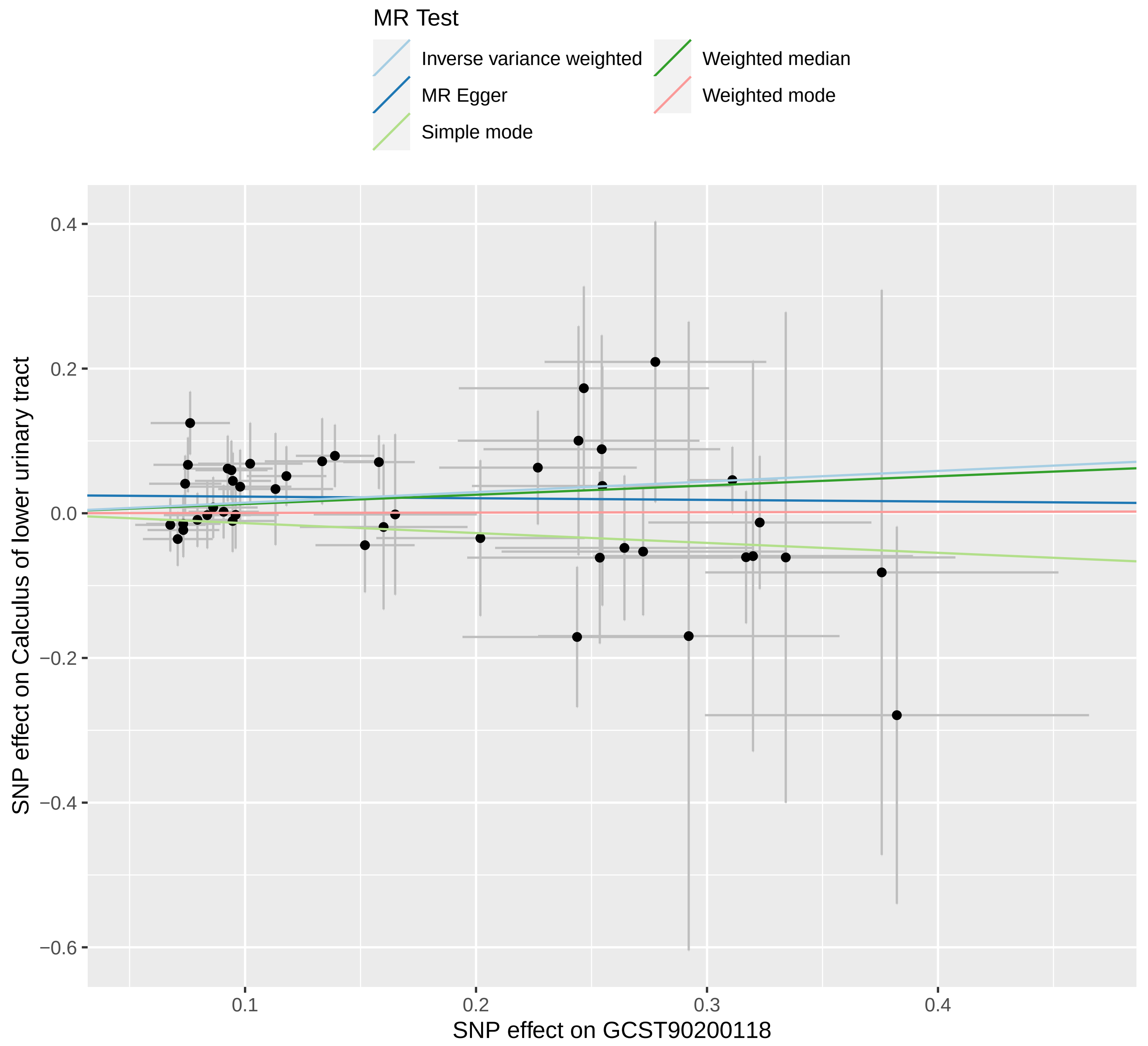
MR Test
Inverse variance weighted
MR Egger
Simple mode
Weighted median
Weighted mode
0.4
0.2
0.0
−0.2
−0.4
−0.6
0.1
0.2
0.3
0.4
SNP effect on Calculus of lower urinary tract
SNP effect on GCST90200118

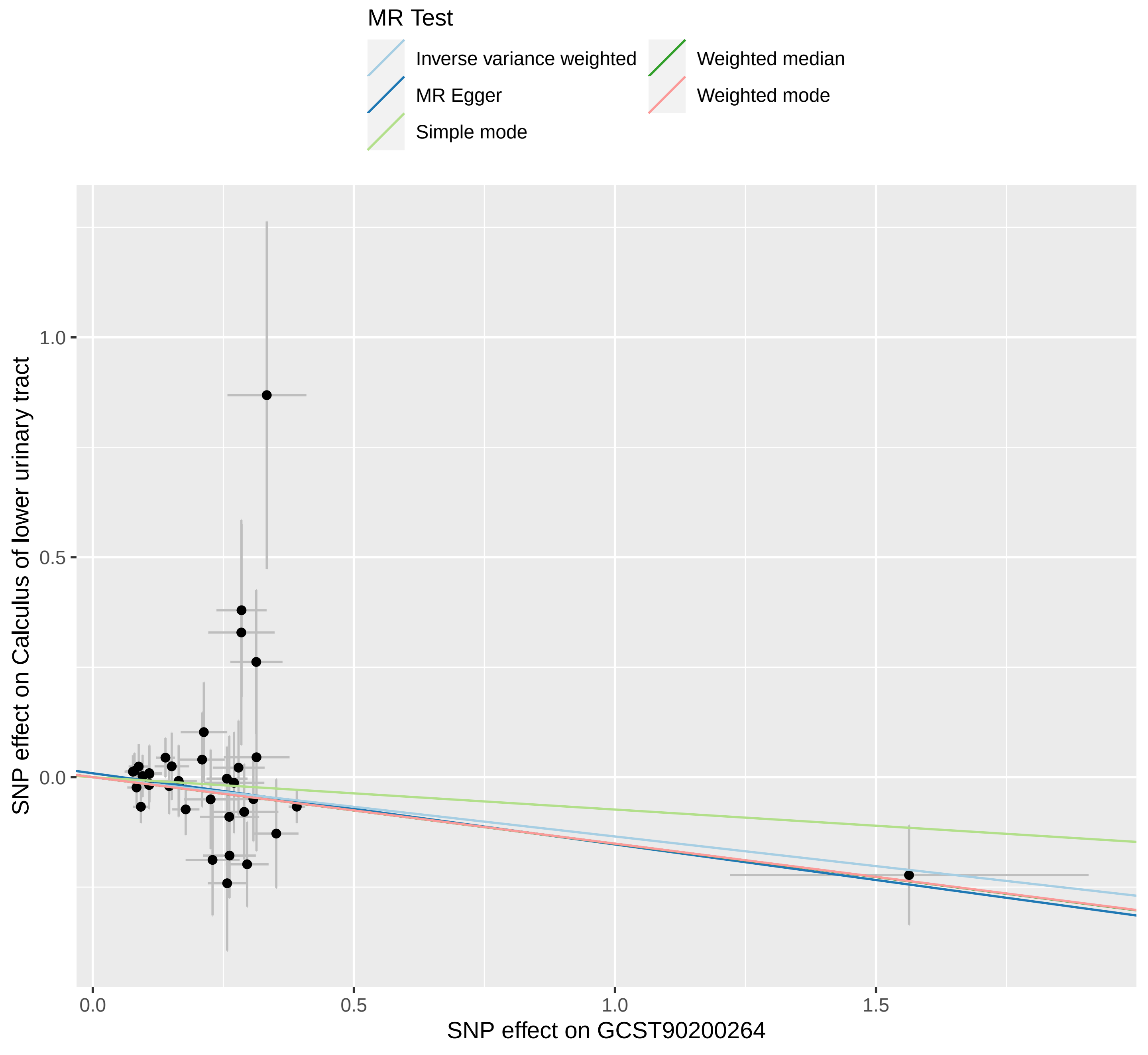
MR Test
Inverse variance weighted
MR Egger
Simple mode
Weighted median
Weighted mode
SNP effect on Calculus of lower urinary tract
1.0
0.5
0.0
0.0
0.5
1.0
1.5
SNP effect on GCST90200264

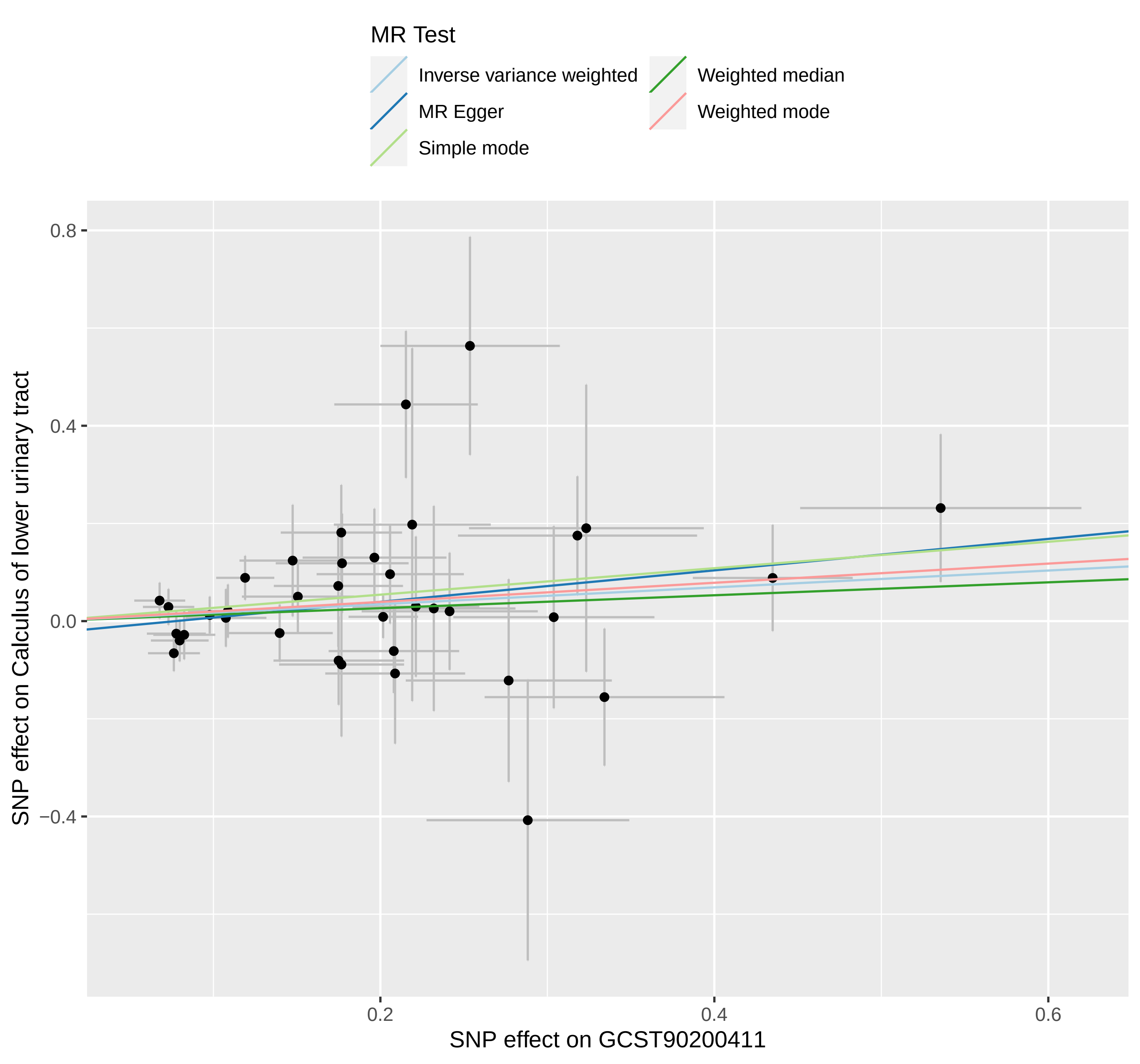

MR Test
Inverse variance weighted
MR Egger
Simple mode
Weighted median
Weighted mode
0.8
0.4
0.0
−0.4
SNP effect on Calculus of lower urinary tract
0.2
0.4
0.6
SNP effect on GCST90200411

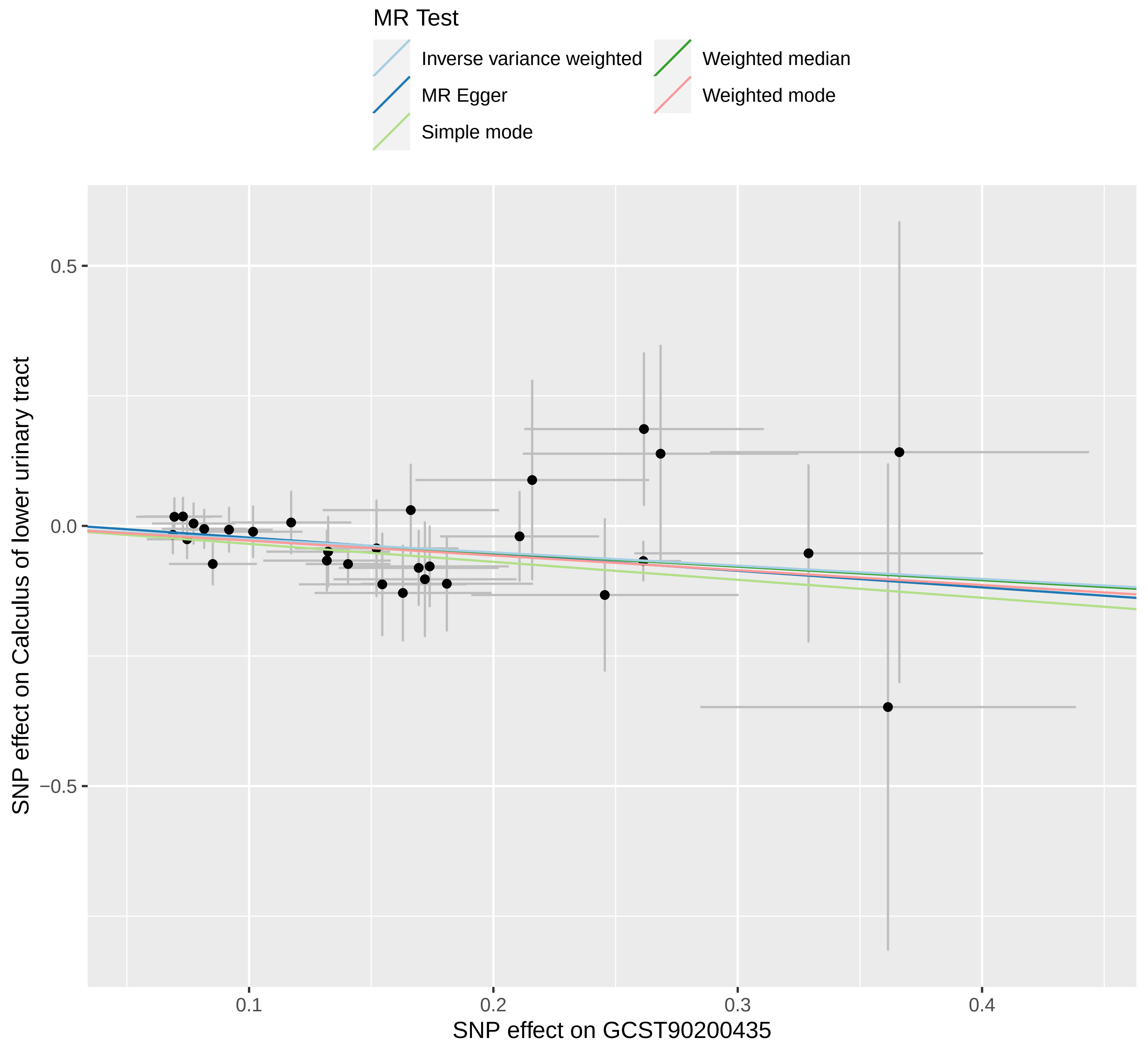

MR Test
Inverse variance weighted
MR Egger
Simple mode
Weighted median
Weighted mode
0.5
0.0
−0.5
0.1
0.2
0.3
0.4
SNP effect on GCST90200435
SNP effect on Calculus of lower urinary tract

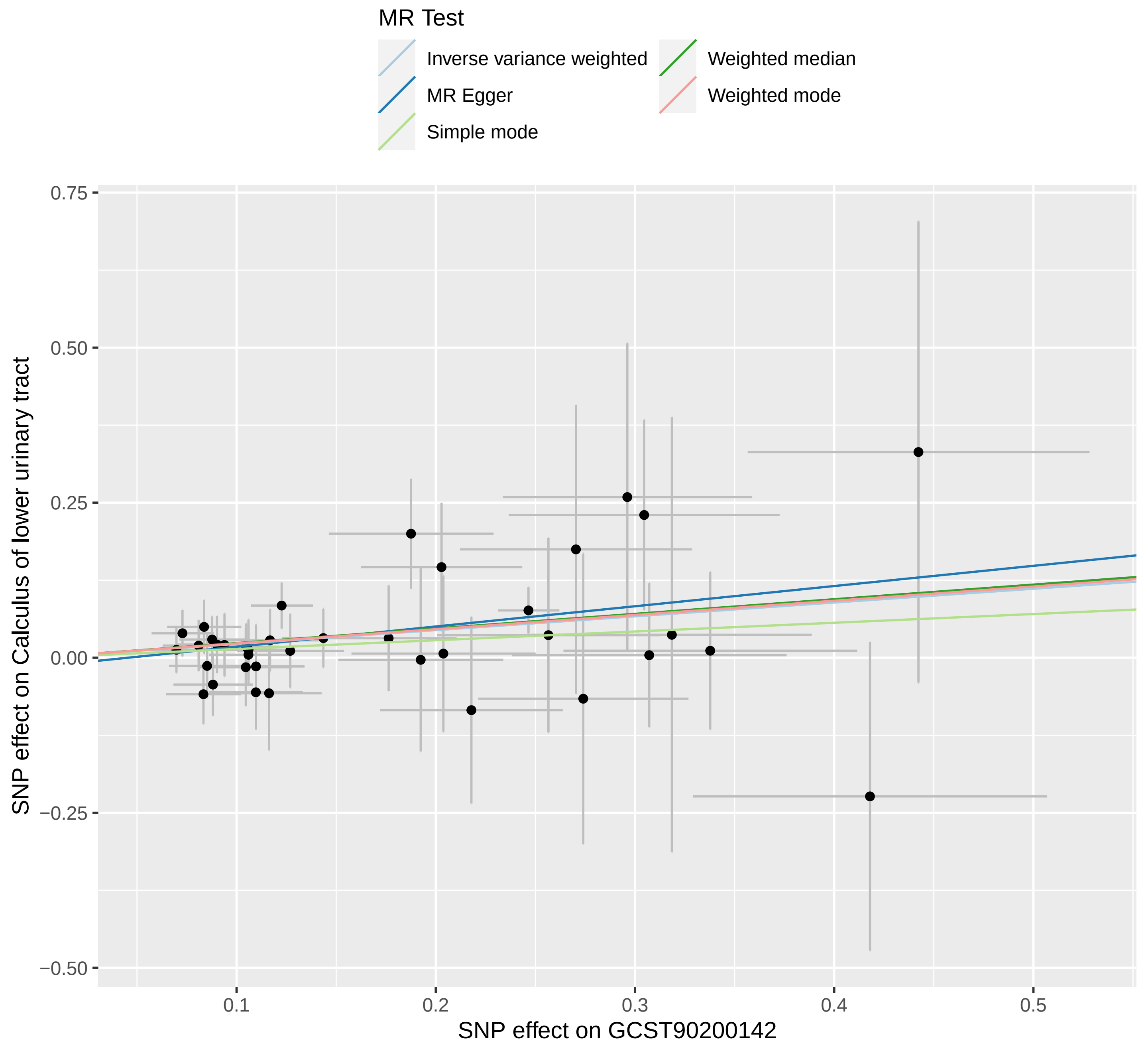

MR Test
Inverse variance weighted
MR Egger
Simple mode
Weighted median
Weighted mode
0.75
0.50
0.25
0.00
−0.25
−0.50
0.1
0.2
0.3
0.4
0.5
SNP effect on Calculus of lower urinary tract
SNP effect on GCST90200142

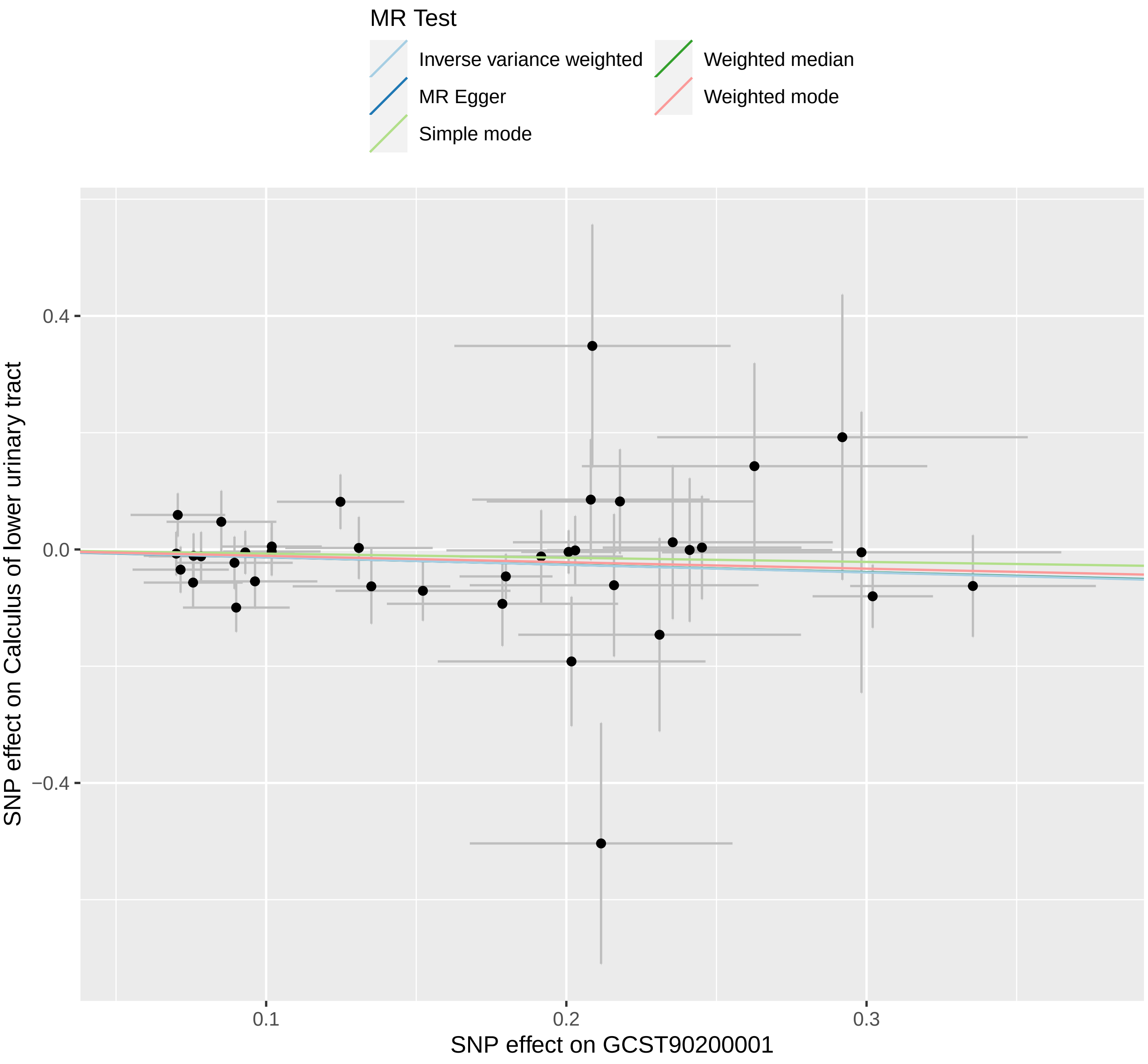
MR Test
Inverse variance weighted
MR Egger
Simple mode
Weighted median
Weighted mode
0.4
0.0
−0.4
0.1
0.2
0.3
SNP effect on Calculus of lower urinary tract
SNP effect on GCST90200001

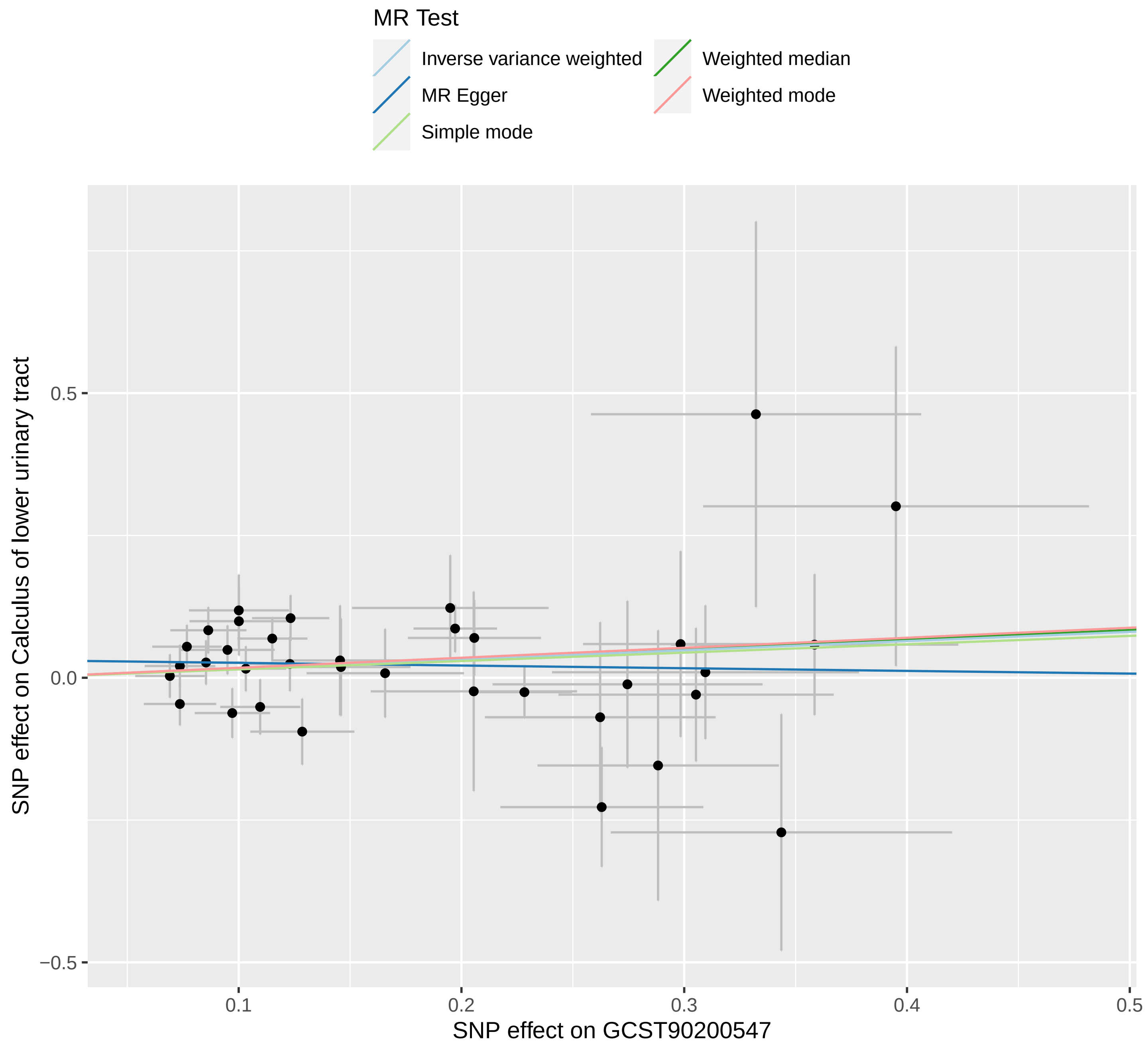
MR Test
Inverse variance weighted
MR Egger
Simple mode
Weighted median
Weighted mode
0.5
0.0
−0.5
0.1
0.2
0.3
0.4
0.5
SNP effect on GCST90200547
SNP effect on Calculus of lower urinary tract

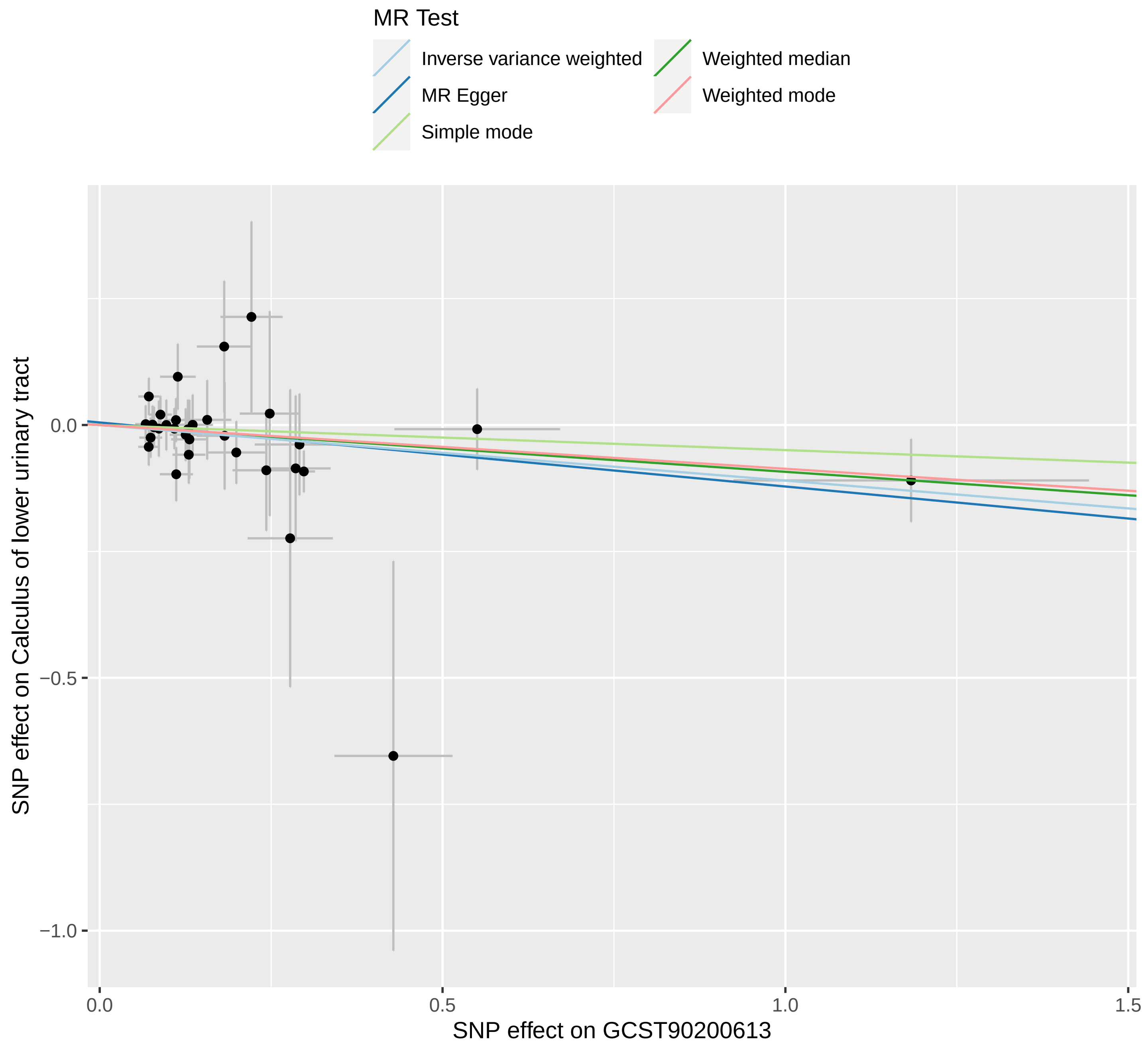

MR Test
Inverse variance weighted
MR Egger
Simple mode
Weighted median
Weighted mode
0.0
−0.5
−1.0
0.0
0.5
1.0
1.5
SNP effect on Calculus of lower urinary tract
SNP effect on GCST90200613

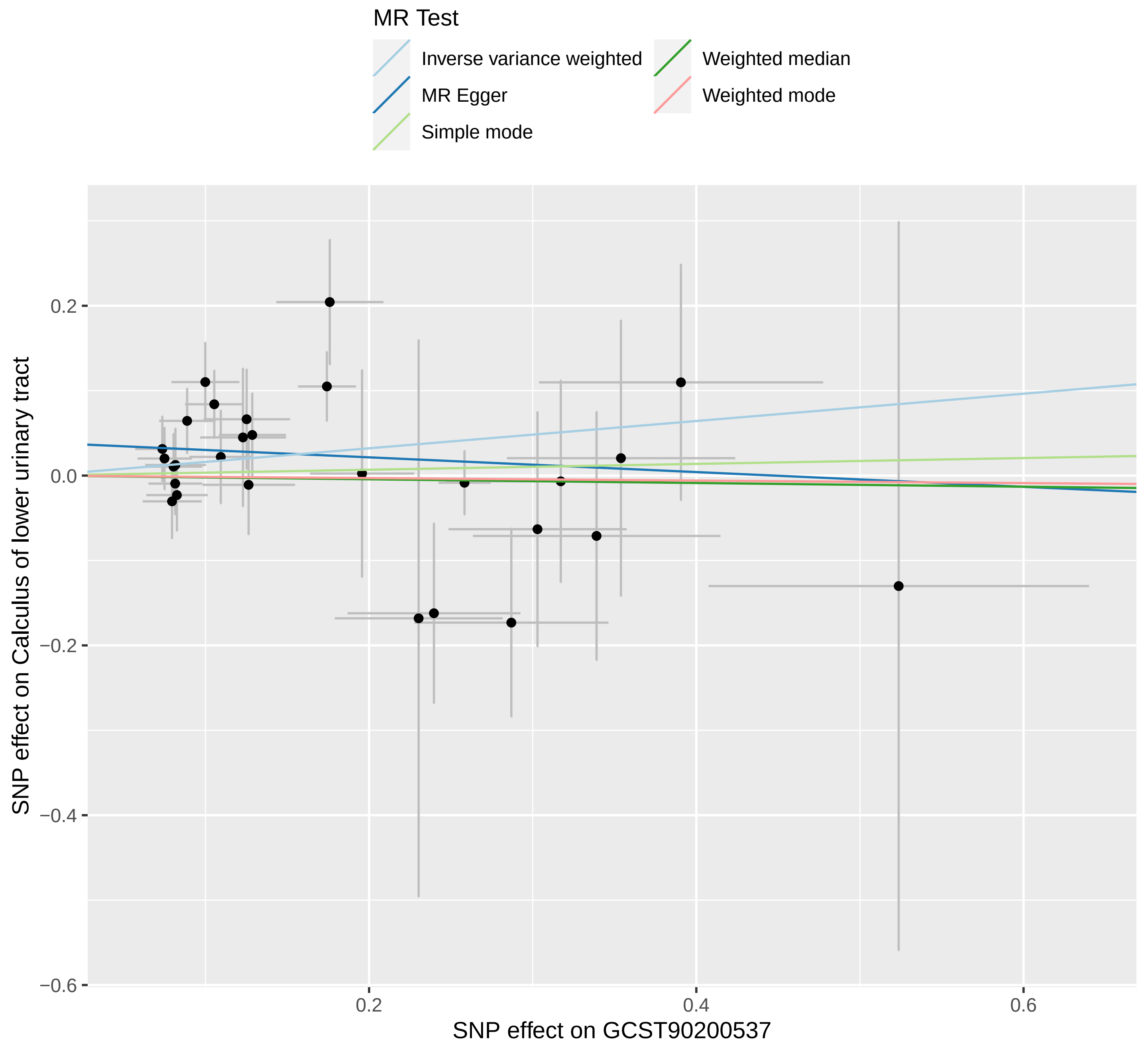

MR Test
Inverse variance weighted
MR Egger
Simple mode
Weighted median
Weighted mode
0.2
0.0
−0.2
−0.4
−0.6
SNP effect on Calculus of lower urinary tract
0.2
0.4
0.6
SNP effect on GCST90200537

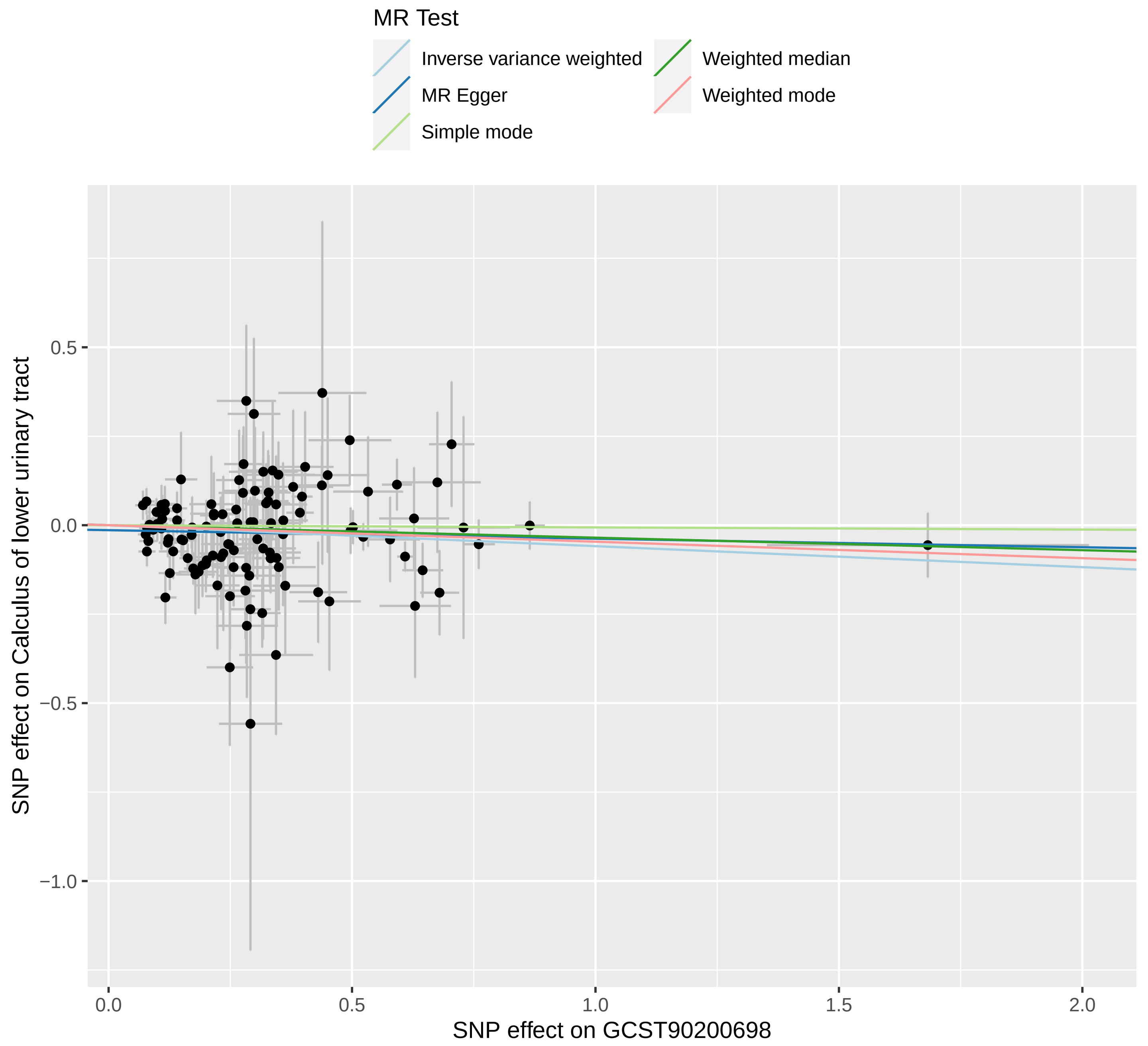
MR Test
Inverse variance weighted
MR Egger
Simple mode
Weighted median
Weighted mode
SNP effect on Calculus of lower urinary tract
0.5
0.0
−0.5
−1.0
0.0
0.5
1.0
1.5
2.0
SNP effect on GCST90200698

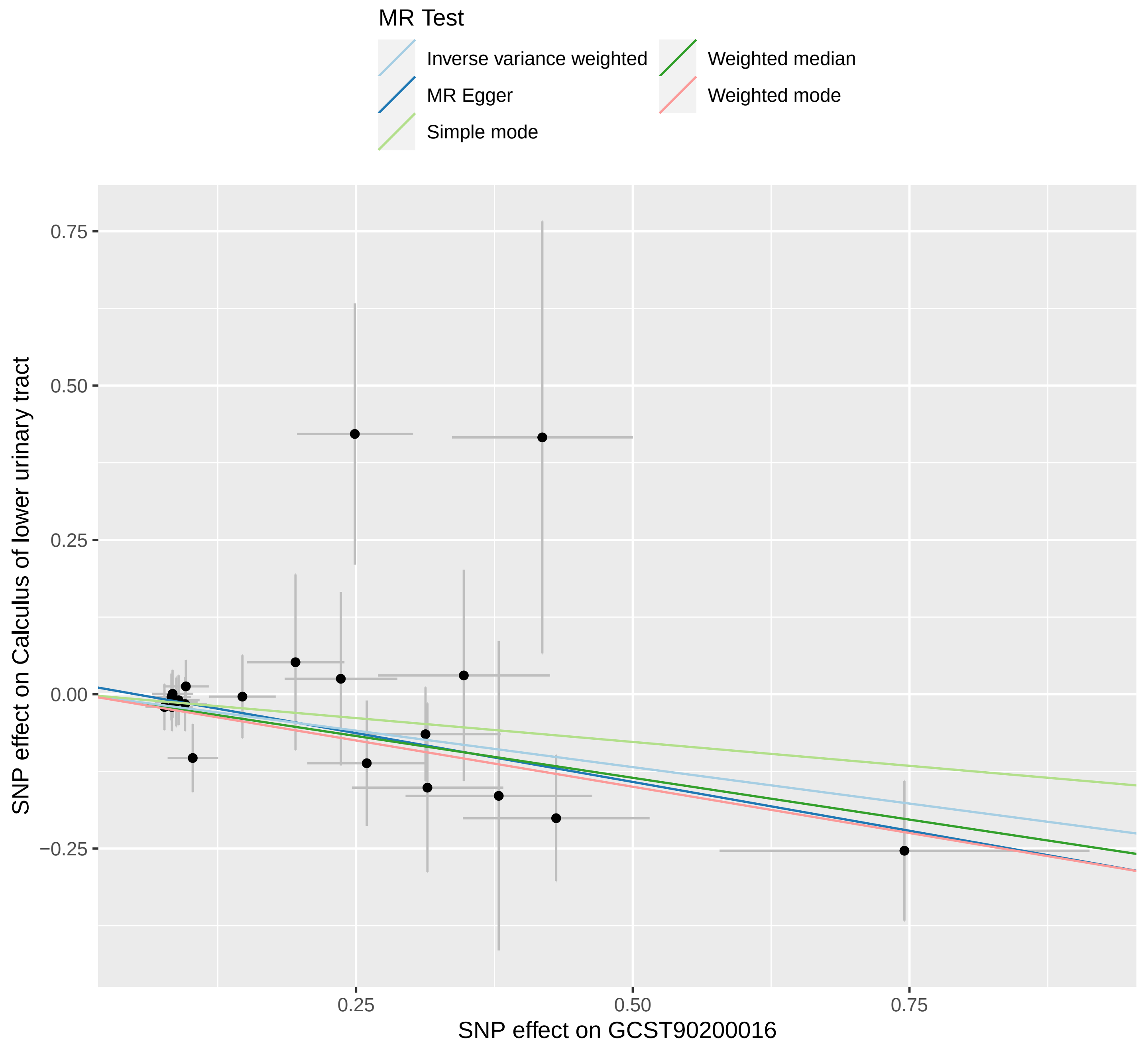

MR Test
Inverse variance weighted
MR Egger
Simple mode
Weighted median
Weighted mode
0.75
0.50
0.25
0.00
−0.25
SNP effect on Calculus of lower urinary tract
0.25
0.50
0.75
SNP effect on GCST90200016

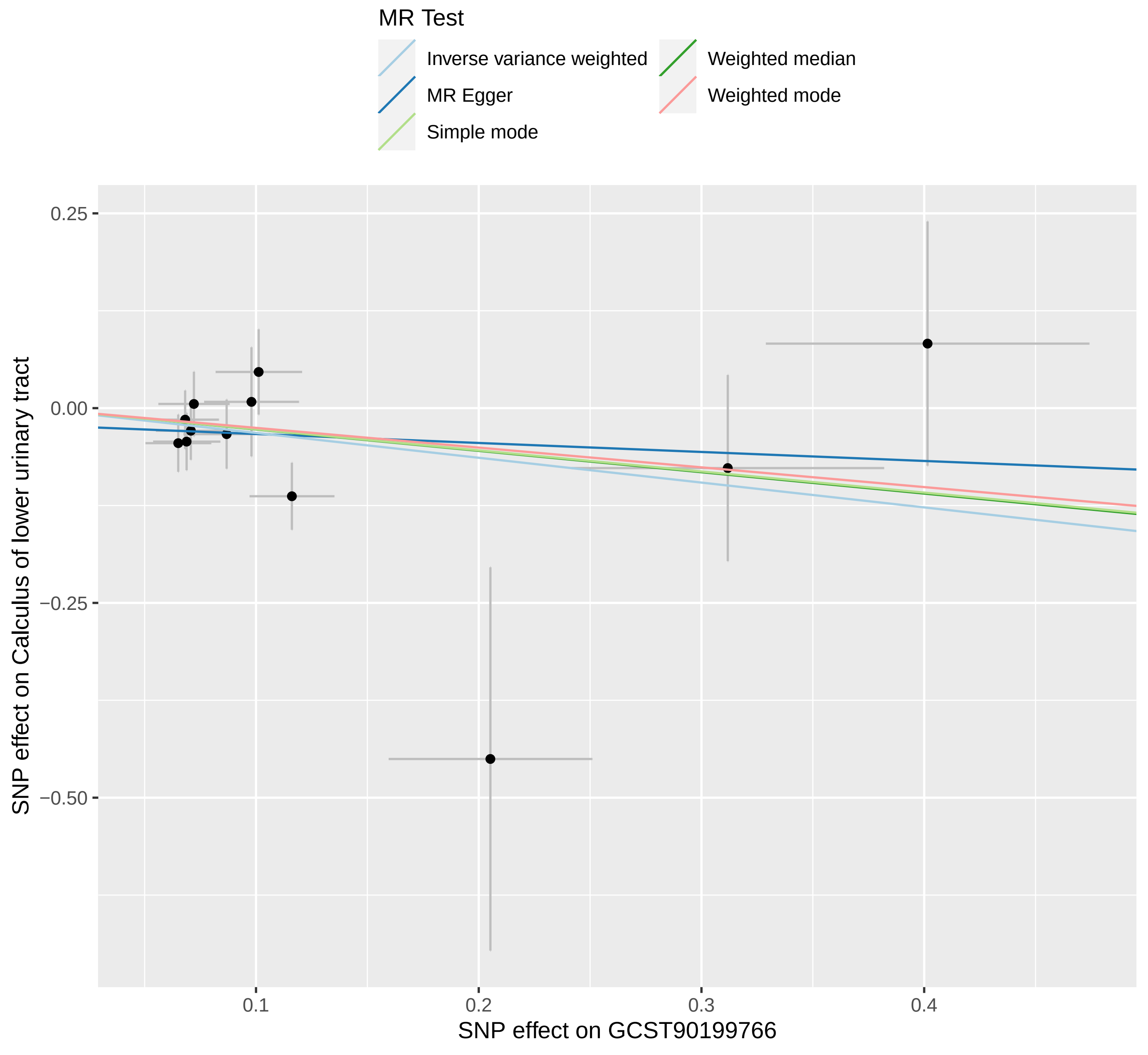
MR Test
Inverse variance weighted
MR Egger
Simple mode
Weighted median
Weighted mode
0.25
0.00
−0.25
−0.50
0.1
0.2
0.3
0.4
SNP effect on Calculus of lower urinary tract
SNP effect on GCST90199766

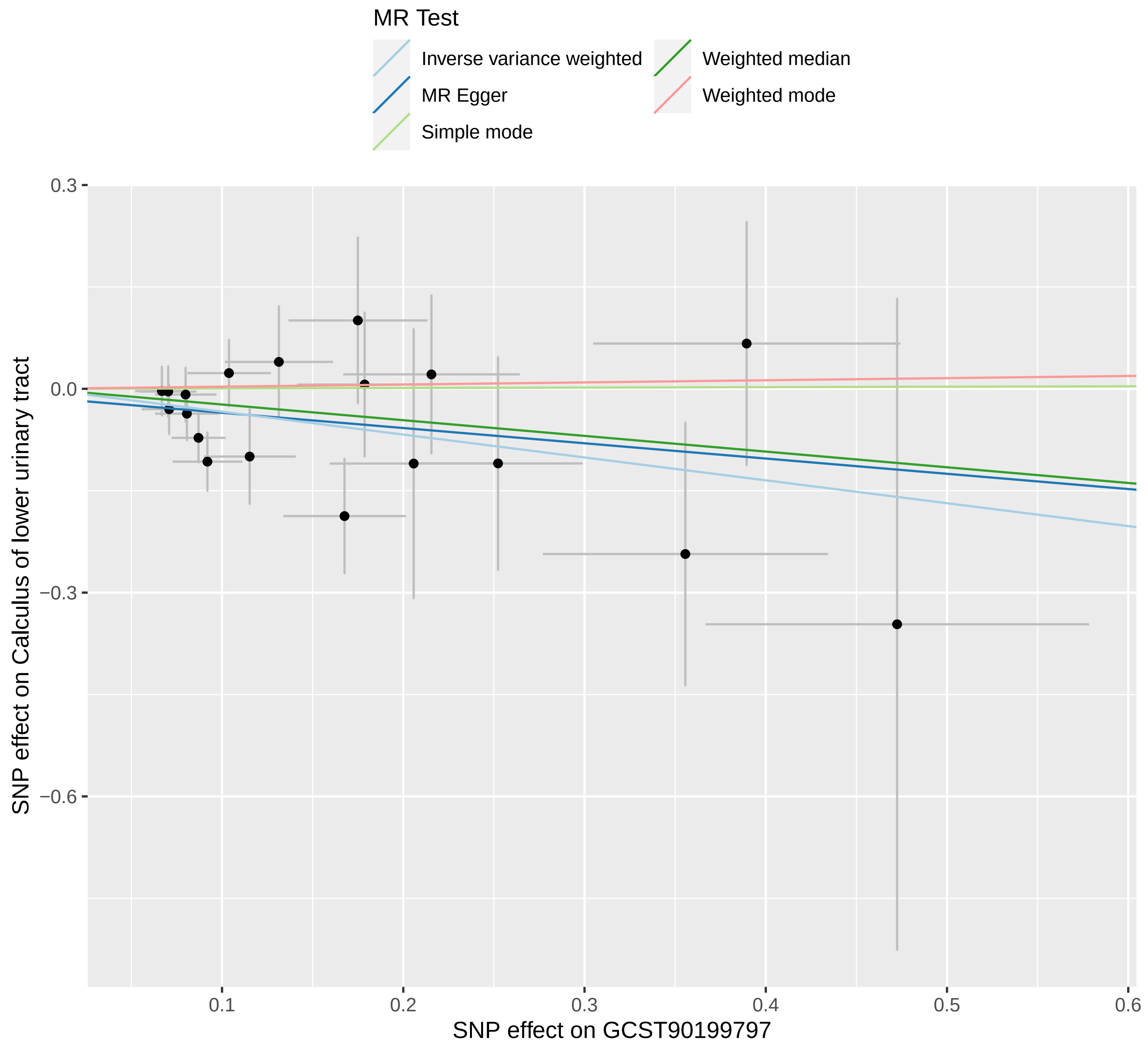

MR Test
Inverse variance weighted
MR Egger
Simple mode
Weighted median
Weighted mode
0.3
0.0
−0.3
−0.6
SNP effect on Calculus of lower urinary tract
0.1
0.2
0.3
0.4
0.5
0.6
SNP effect on GCST90199797

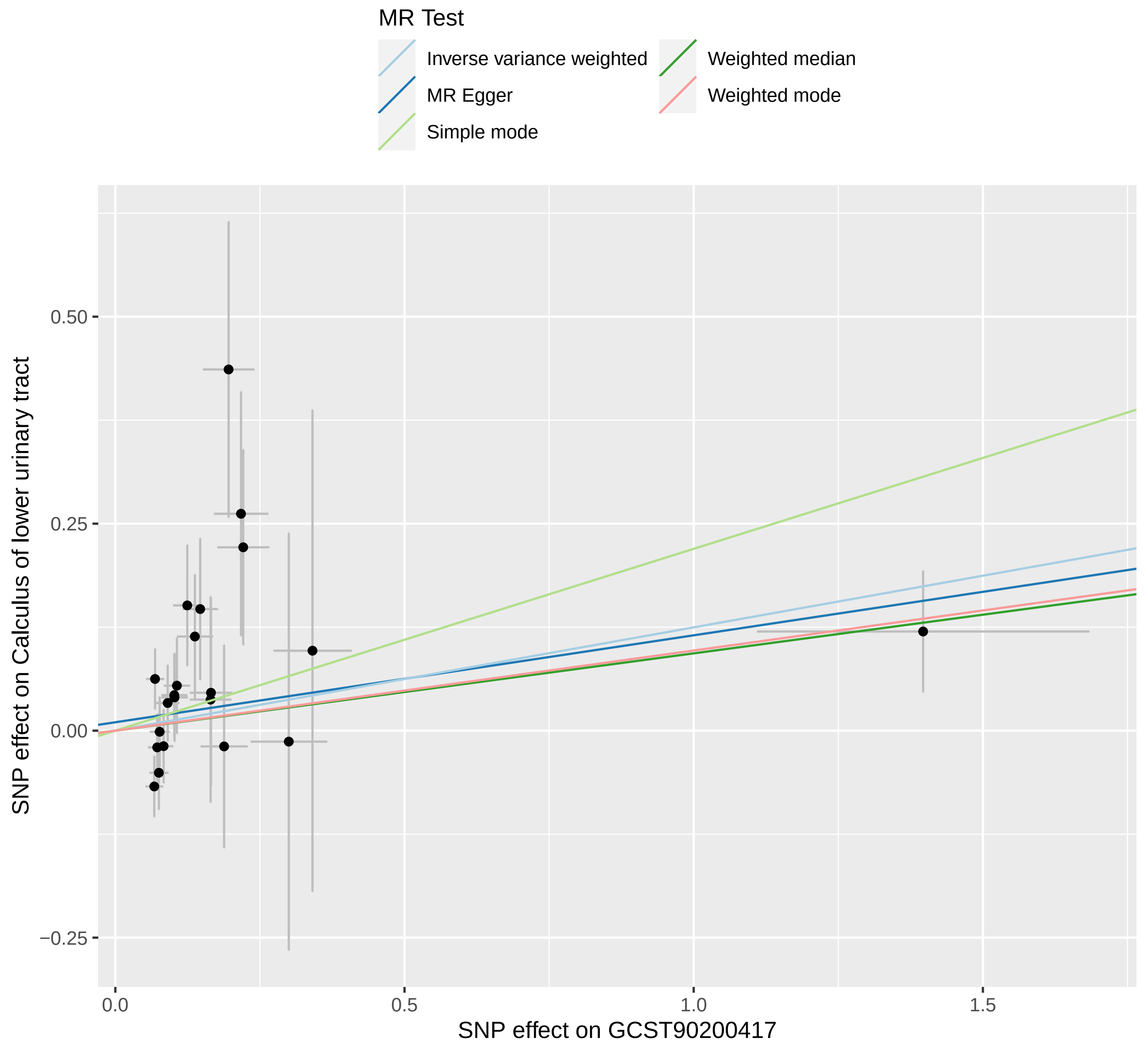

MR Test
Inverse variance weighted
MR Egger
Simple mode
Weighted median
Weighted mode
0.50
0.25
0.00
−0.25
0.0
0.5
1.0
1.5
SNP effect on Calculus of lower urinary tract
SNP effect on GCST90200417

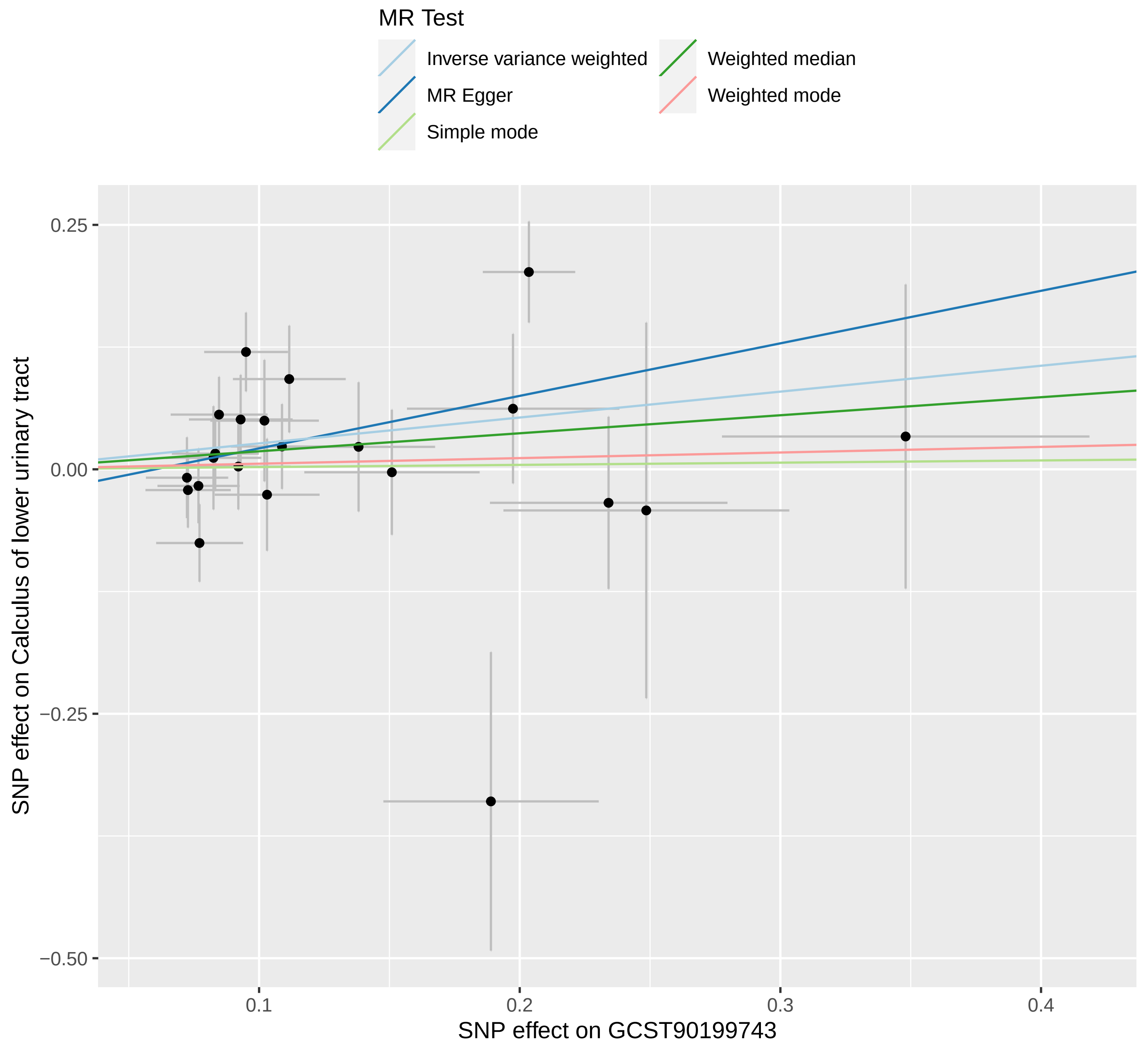

MR Test
Inverse variance weighted
MR Egger
Simple mode
Weighted median
Weighted mode
0.25
0.00
−0.25
−0.50
0.1
0.2
0.3
0.4
SNP effect on GCST90199743
SNP effect on Calculus of lower urinary tract

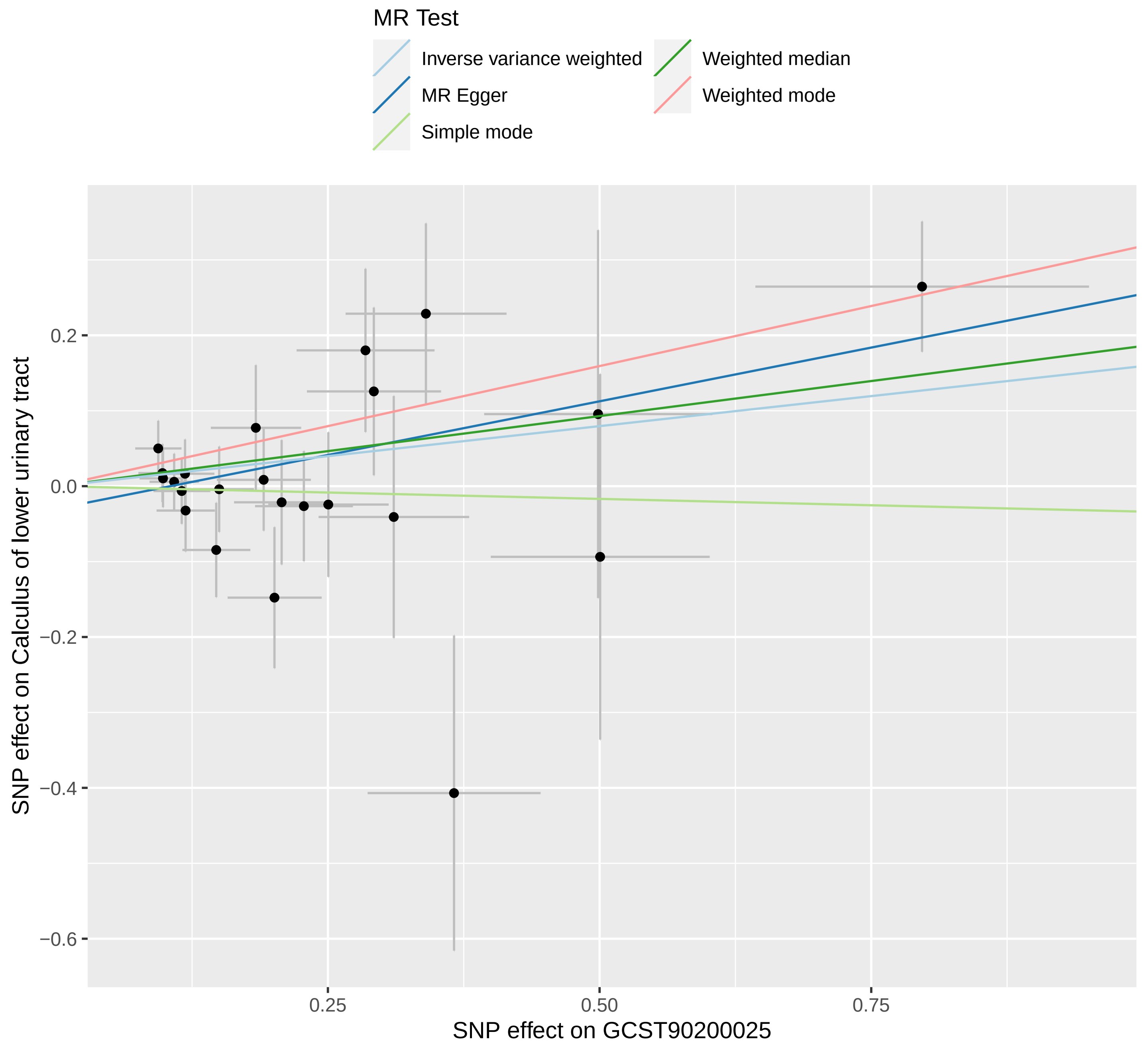
MR Test
Inverse variance weighted
MR Egger
Simple mode
Weighted median
Weighted mode
SNP effect on Calculus of lower urinary tract
0.2
0.0
−0.2
−0.4
−0.6
0.25
0.50
0.75
SNP effect on GCST90200025

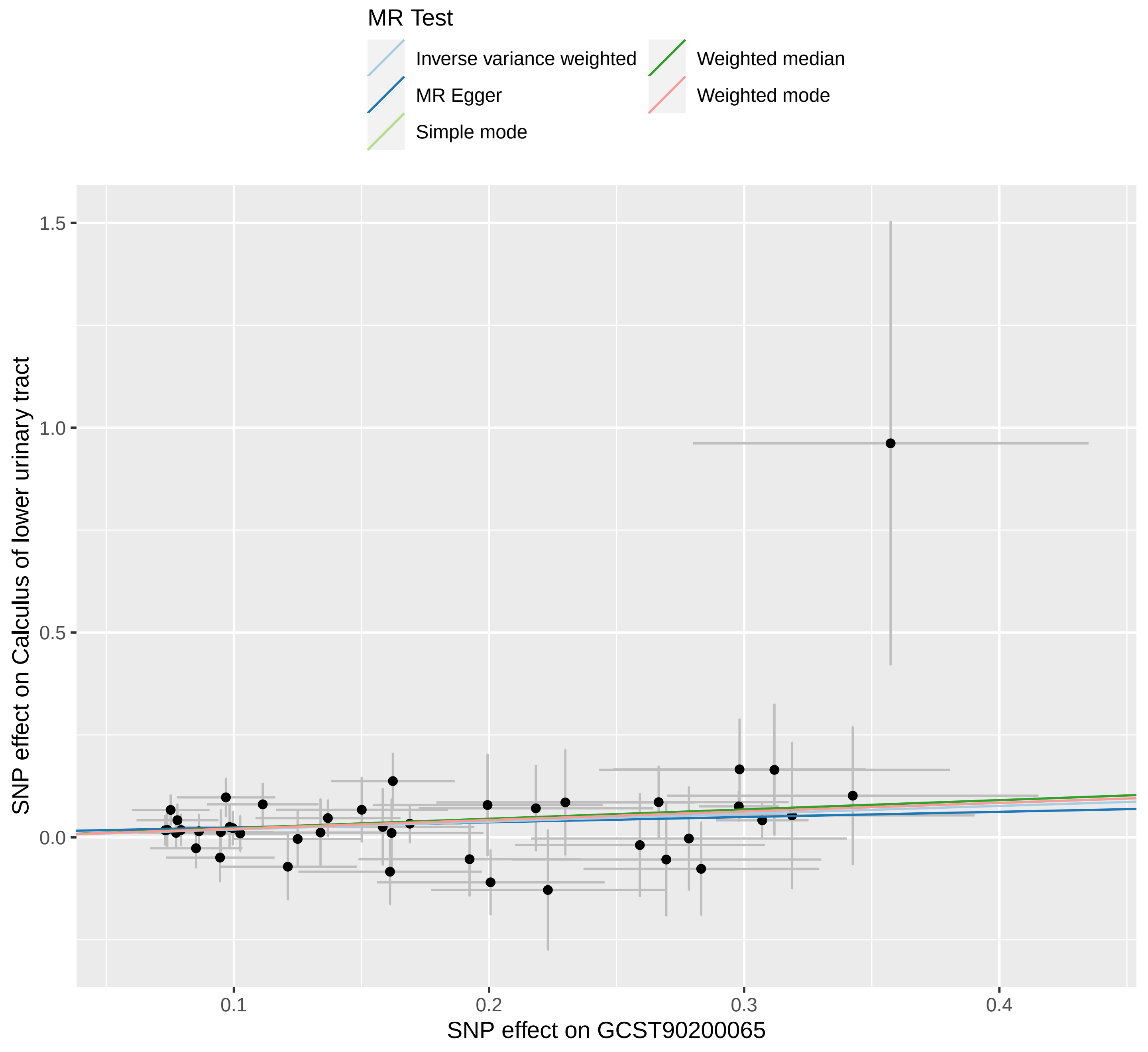

MR Test
Inverse variance weighted
MR Egger
Simple mode
Weighted median
Weighted mode
1.5
1.0
0.5
0.0
0.1
0.2
0.3
0.4
SNP effect on GCST90200065
SNP effect on Calculus of lower urinary tract

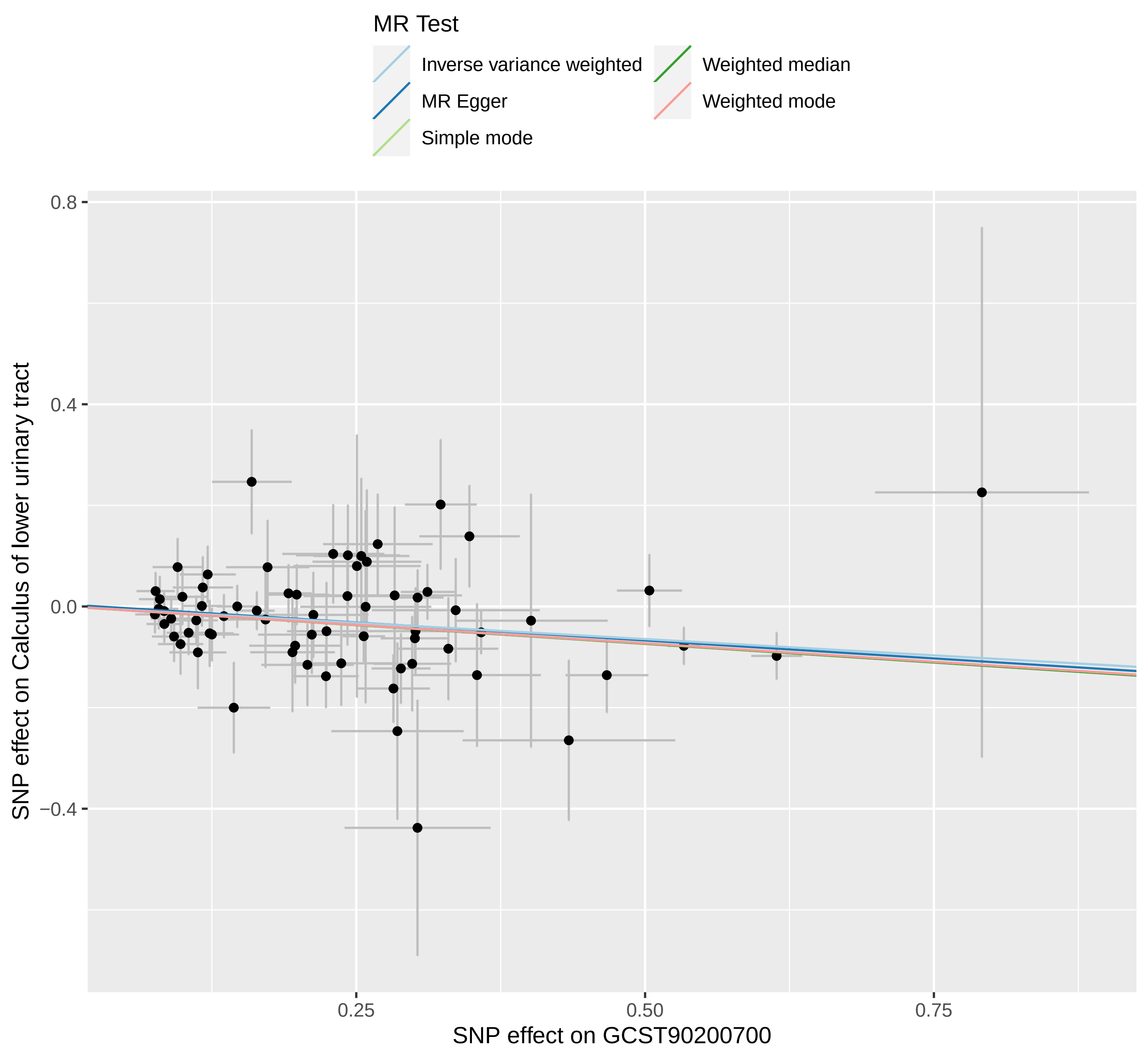
MR Test
Inverse variance weighted
MR Egger
Simple mode
Weighted median
Weighted mode
0.8
0.4
0.0
−0.4
0.25
0.50
0.75
SNP effect on Calculus of lower urinary tract
SNP effect on GCST90200700

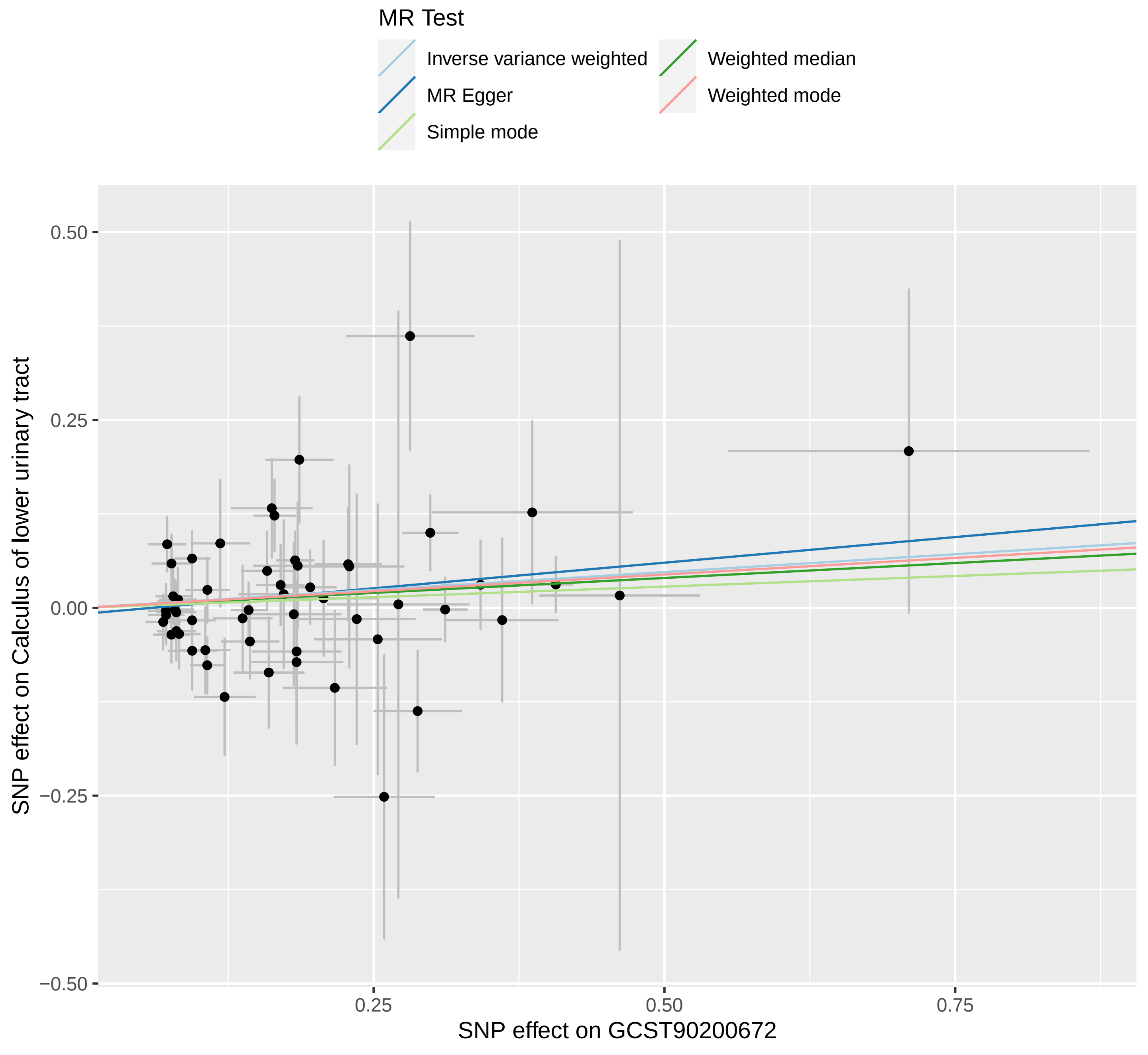

MR Test
Inverse variance weighted
MR Egger
Simple mode
Weighted median
Weighted mode
0.50
0.25
0.00
−0.25
−0.50
0.25
0.50
0.75
SNP effect on Calculus of lower urinary tract
SNP effect on GCST90200672

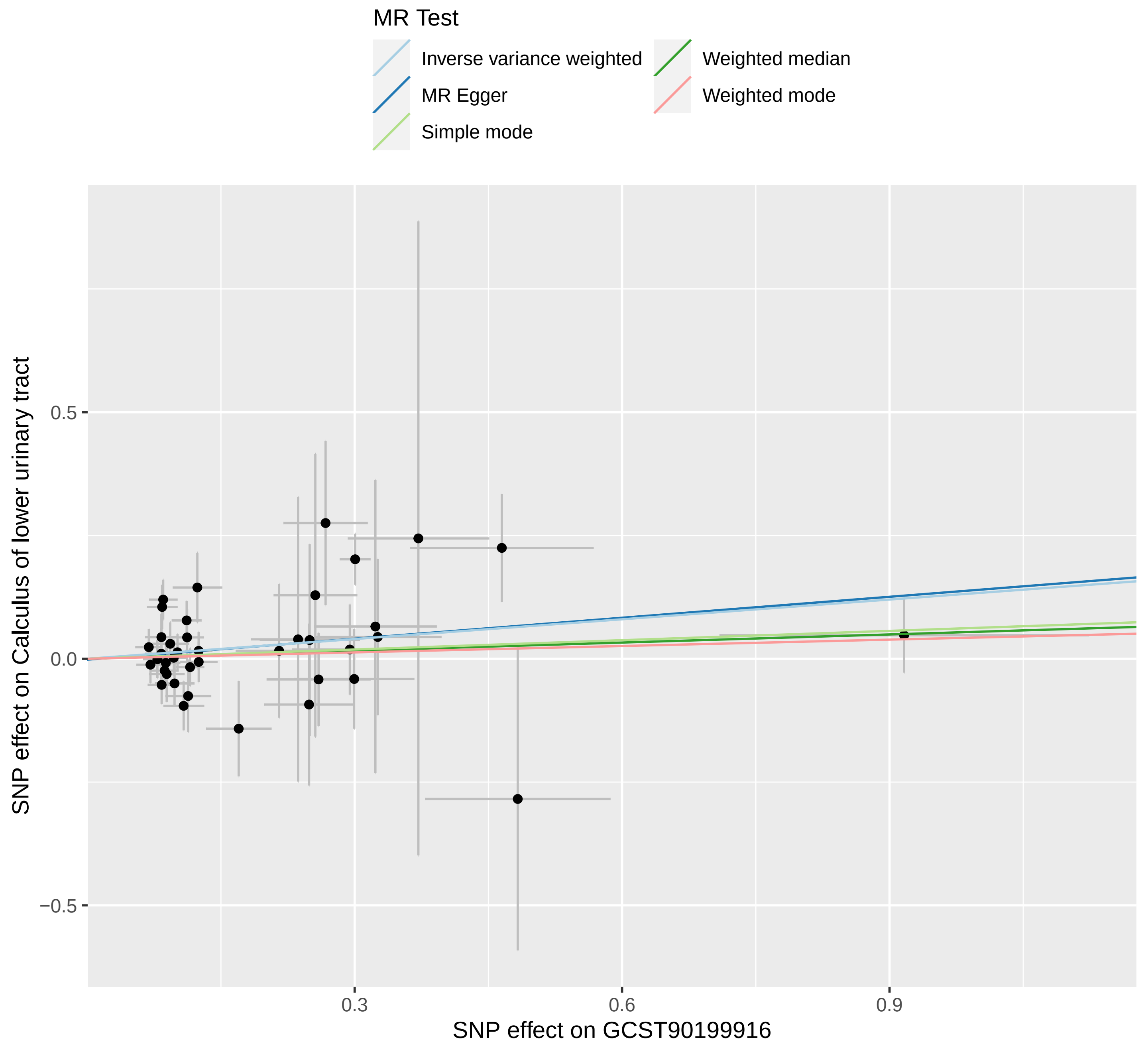
MR Test
Inverse variance weighted
MR Egger
Simple mode
Weighted median
Weighted mode
SNP effect on Calculus of lower urinary tract
0.5
0.0
−0.5
0.3
0.6
0.9
SNP effect on GCST90199916

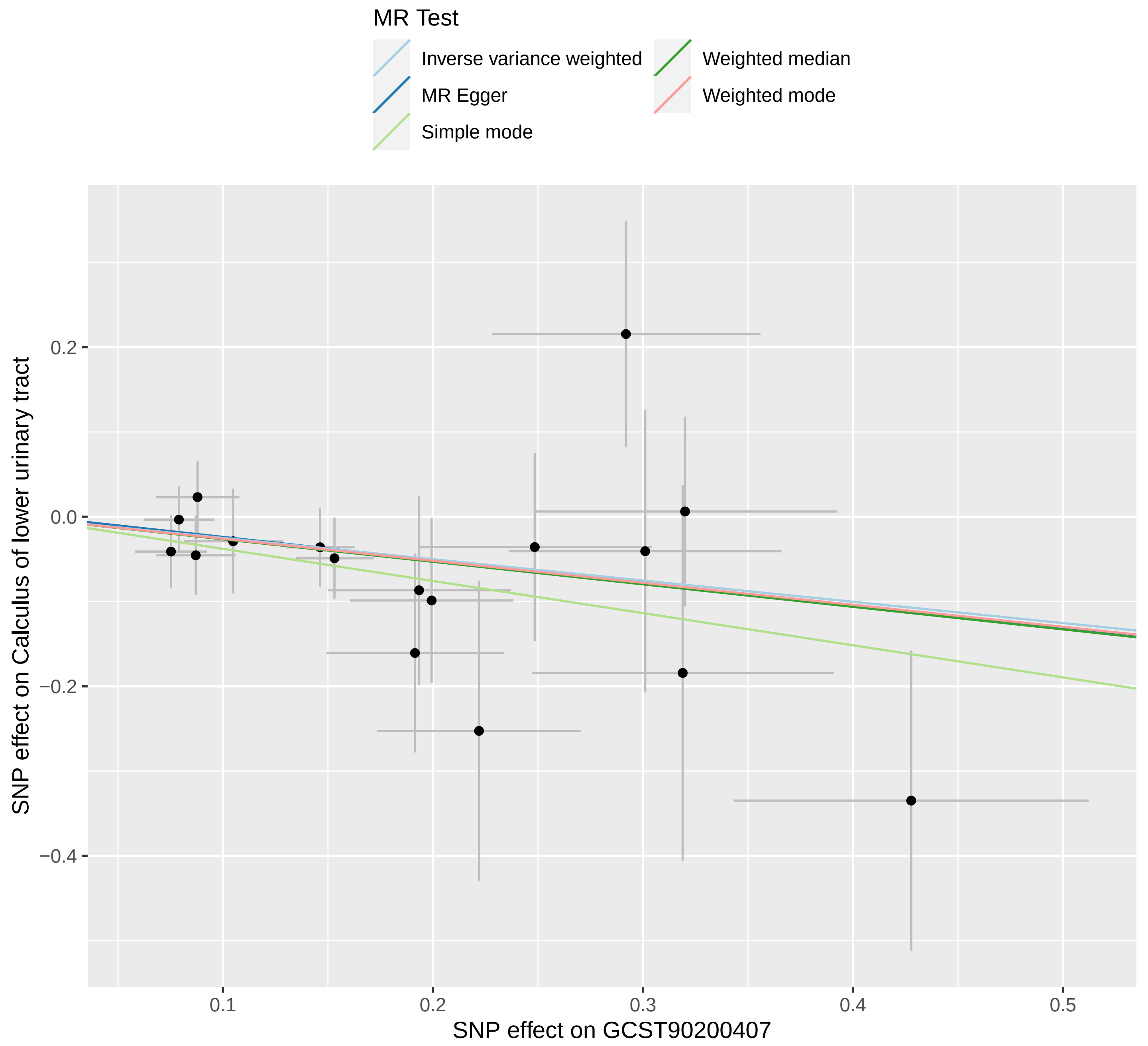

MR Test
Inverse variance weighted
MR Egger
Simple mode
Weighted median
Weighted mode
SNP effect on Calculus of lower urinary tract
0.2
0.0
−0.2
−0.4
0.1
0.2
0.3
0.4
0.5
SNP effect on GCST90200407

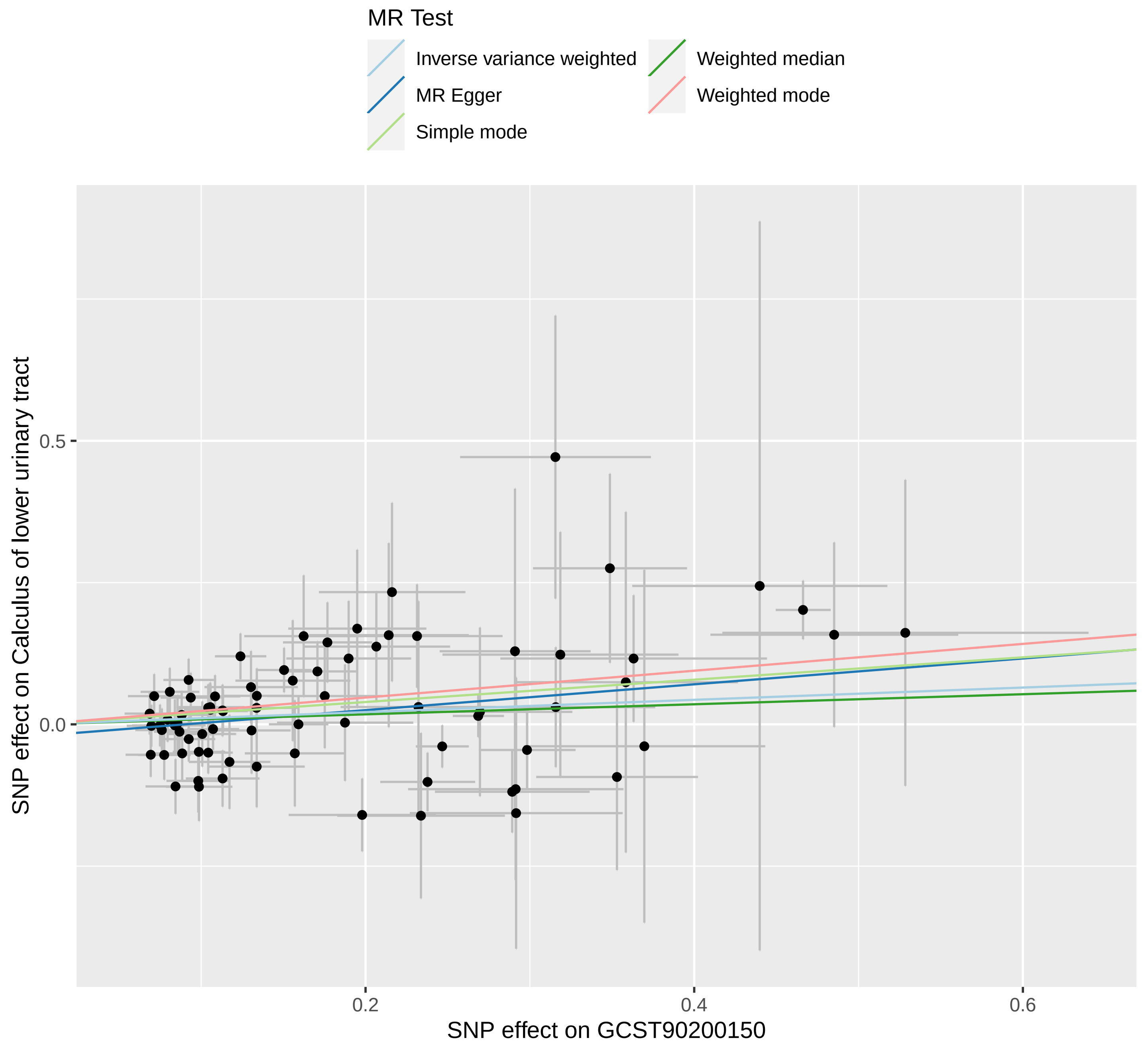

MR Test
Inverse variance weighted
MR Egger
Simple mode
Weighted median
Weighted mode
0.5
0.0
0.2
0.4
0.6
SNP effect on GCST90200150
SNP effect on Calculus of lower urinary tract

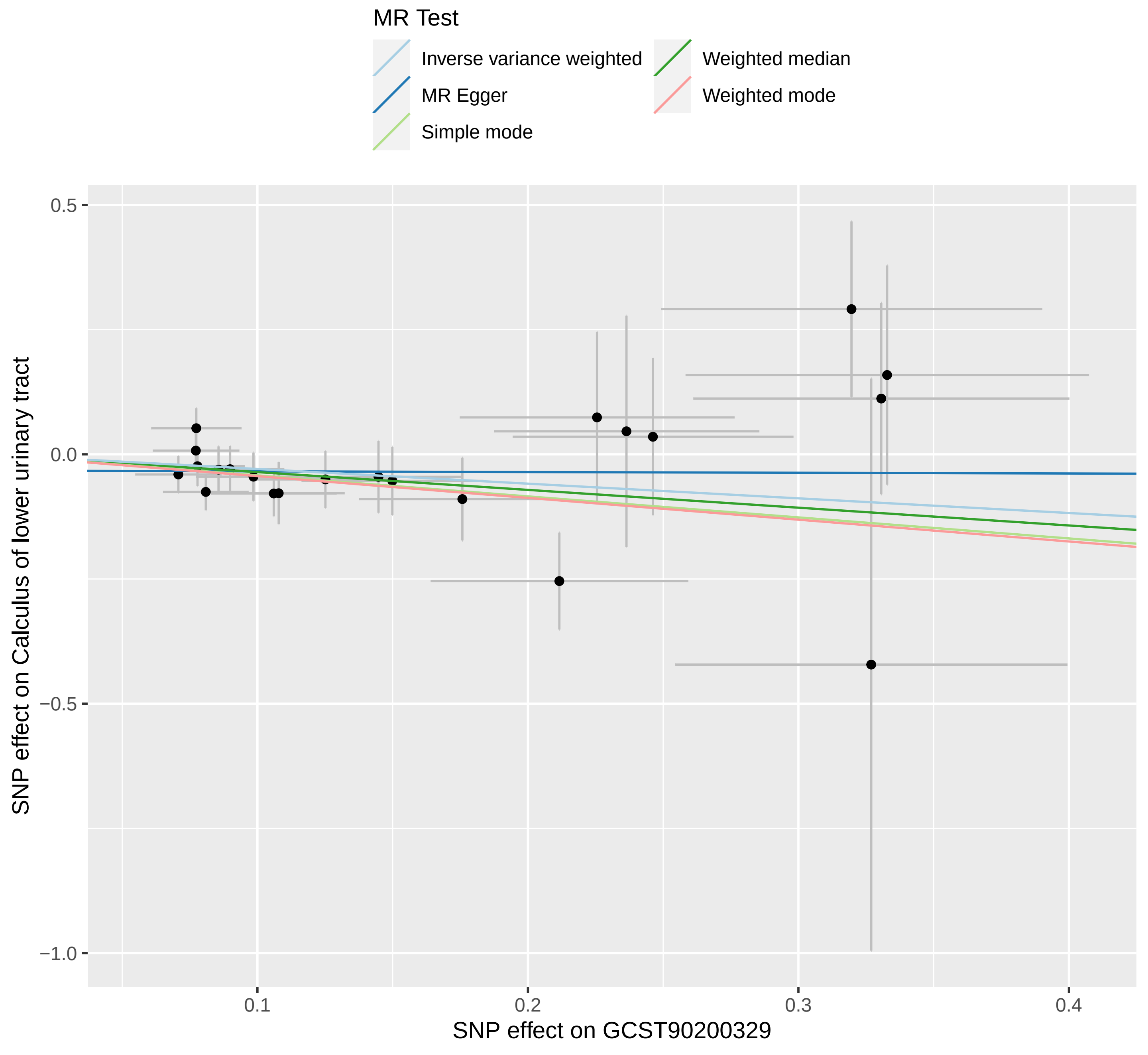
MR Test
Inverse variance weighted
MR Egger
Simple mode
Weighted median
Weighted mode
SNP effect on Calculus of lower urinary tract
0.5
0.0
−0.5
−1.0
0.1
0.2
0.3
0.4
SNP effect on GCST90200329

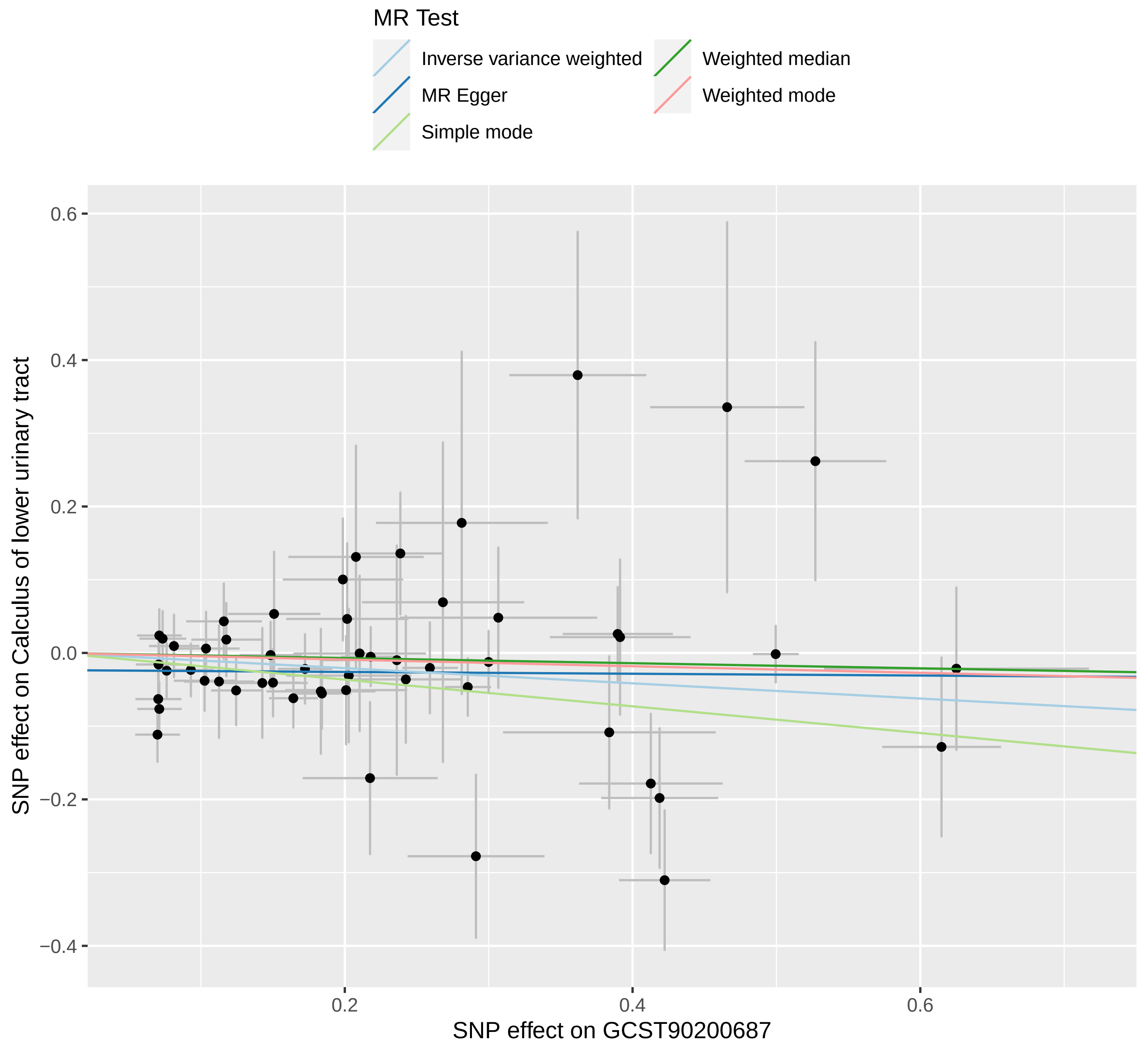

MR Test
Inverse variance weighted
MR Egger
Simple mode
Weighted median
Weighted mode
0.6
0.4
0.2
0.0
−0.2
−0.4
SNP effect on Calculus of lower urinary tract
0.2
0.4
0.6
SNP effect on GCST90200687

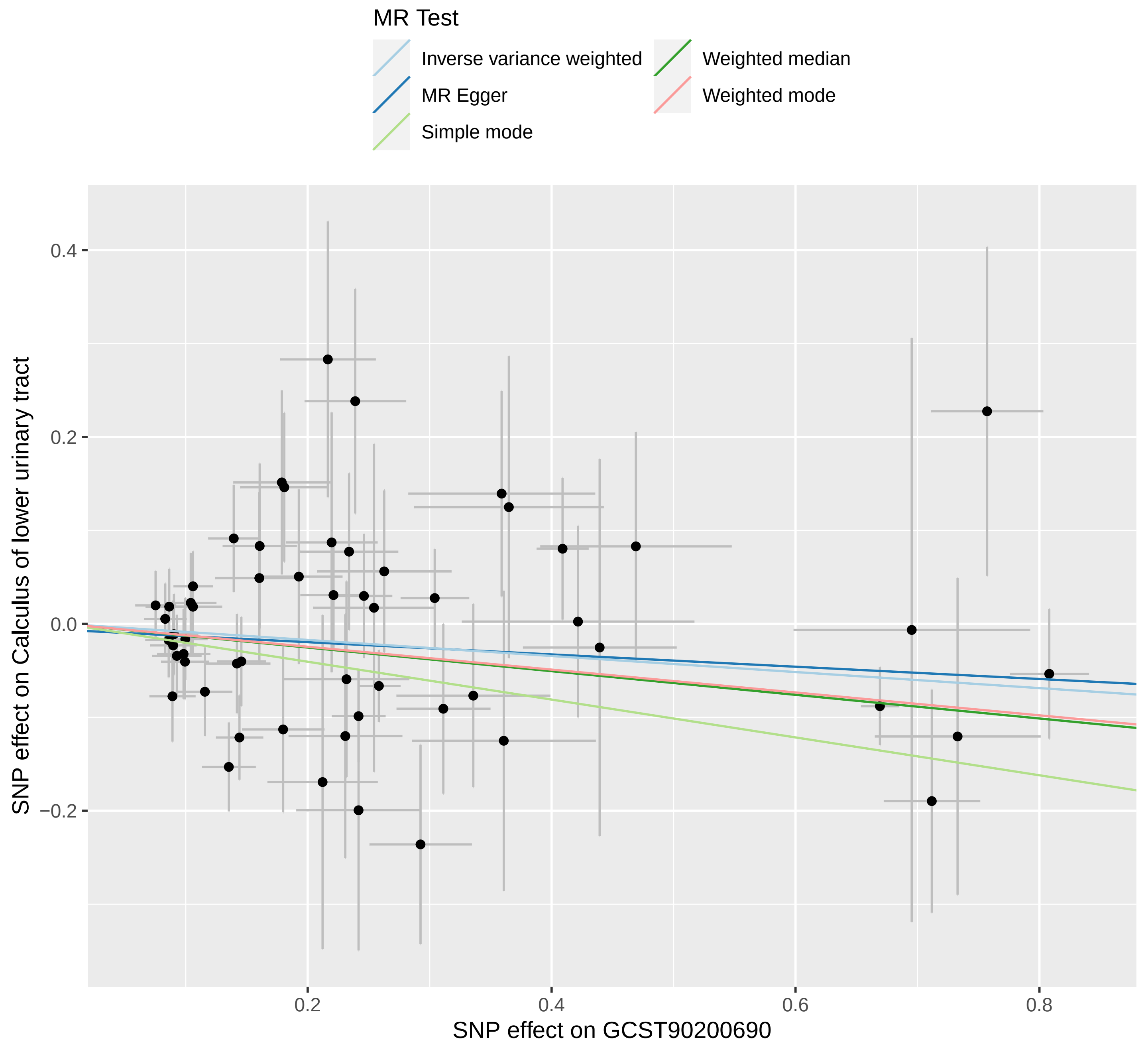

MR Test
Inverse variance weighted
MR Egger
Simple mode
Weighted median
Weighted mode
0.4
0.2
0.0
−0.2
0.2
0.4
0.6
0.8
SNP effect on Calculus of lower urinary tract
SNP effect on GCST90200690

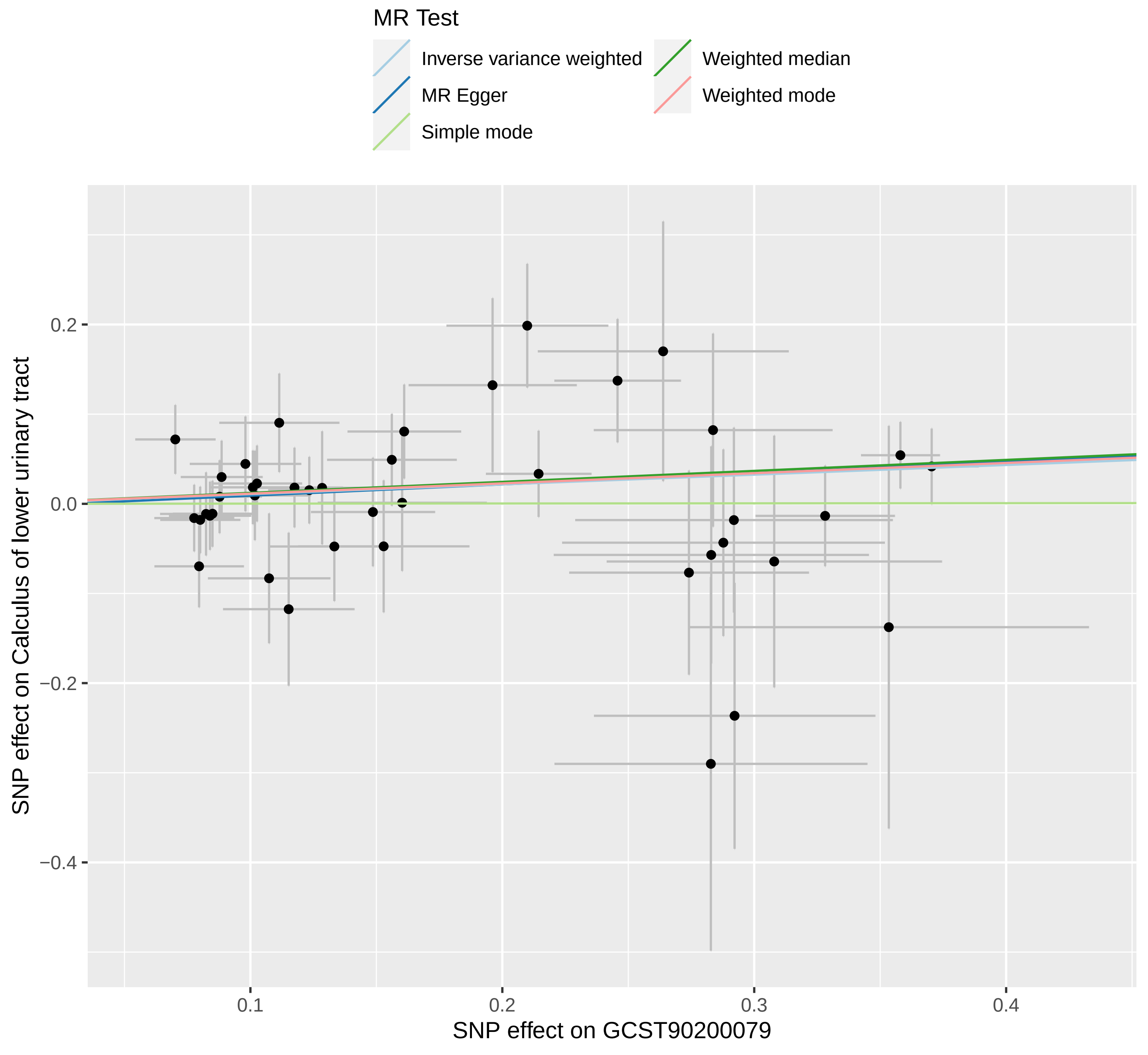

MR Test
Inverse variance weighted
MR Egger
Simple mode
Weighted median
Weighted mode
0.2
0.0
−0.2
−0.4
0.1
0.2
0.3
0.4
SNP effect on GCST90200079
SNP effect on Calculus of lower urinary tract

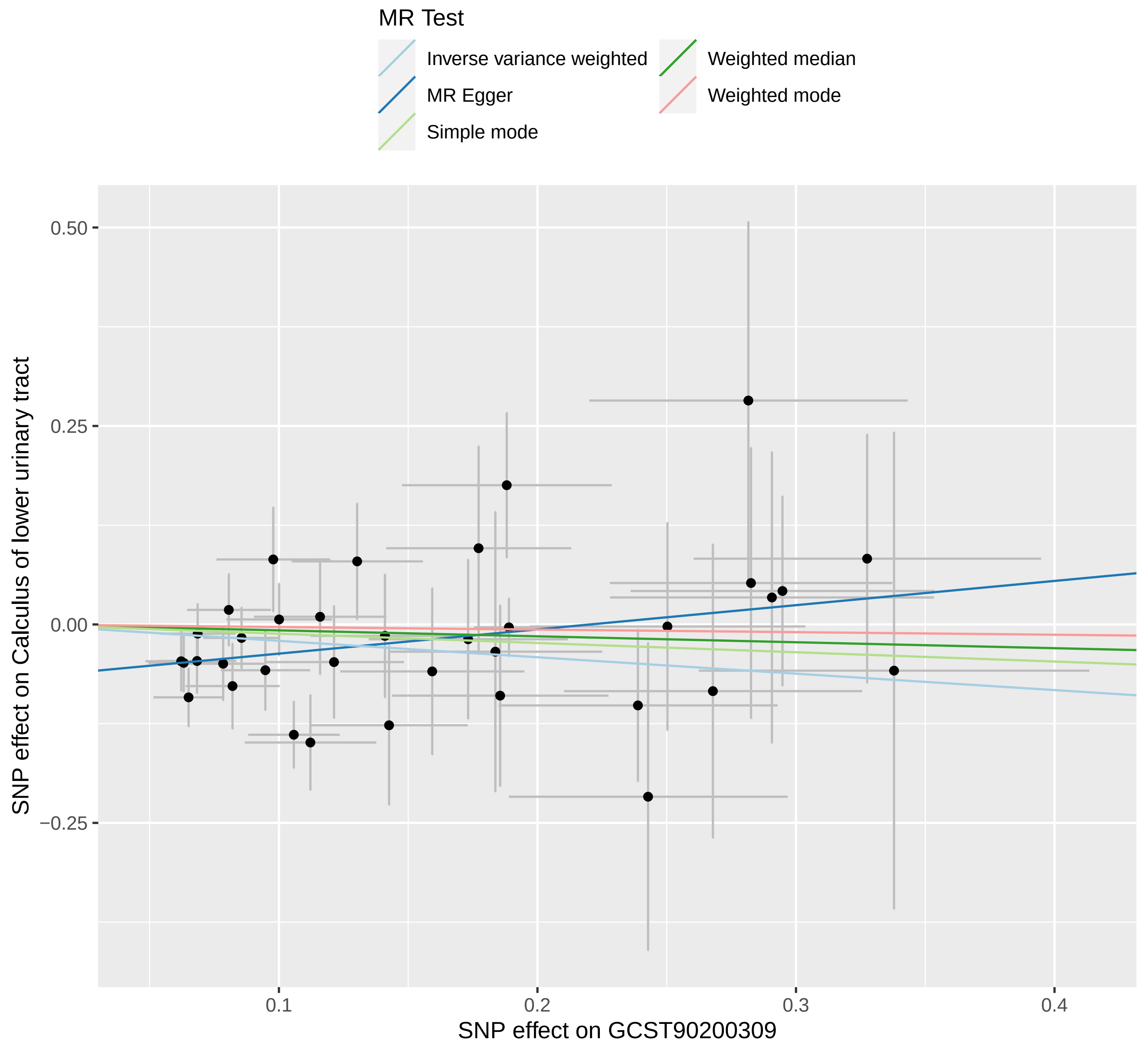

MR Test
Inverse variance weighted
MR Egger
Simple mode
Weighted median
Weighted mode
0.50
0.25
0.00
−0.25
0.1
0.2
0.3
0.4
SNP effect on GCST90200309
SNP effect on Calculus of lower urinary tract

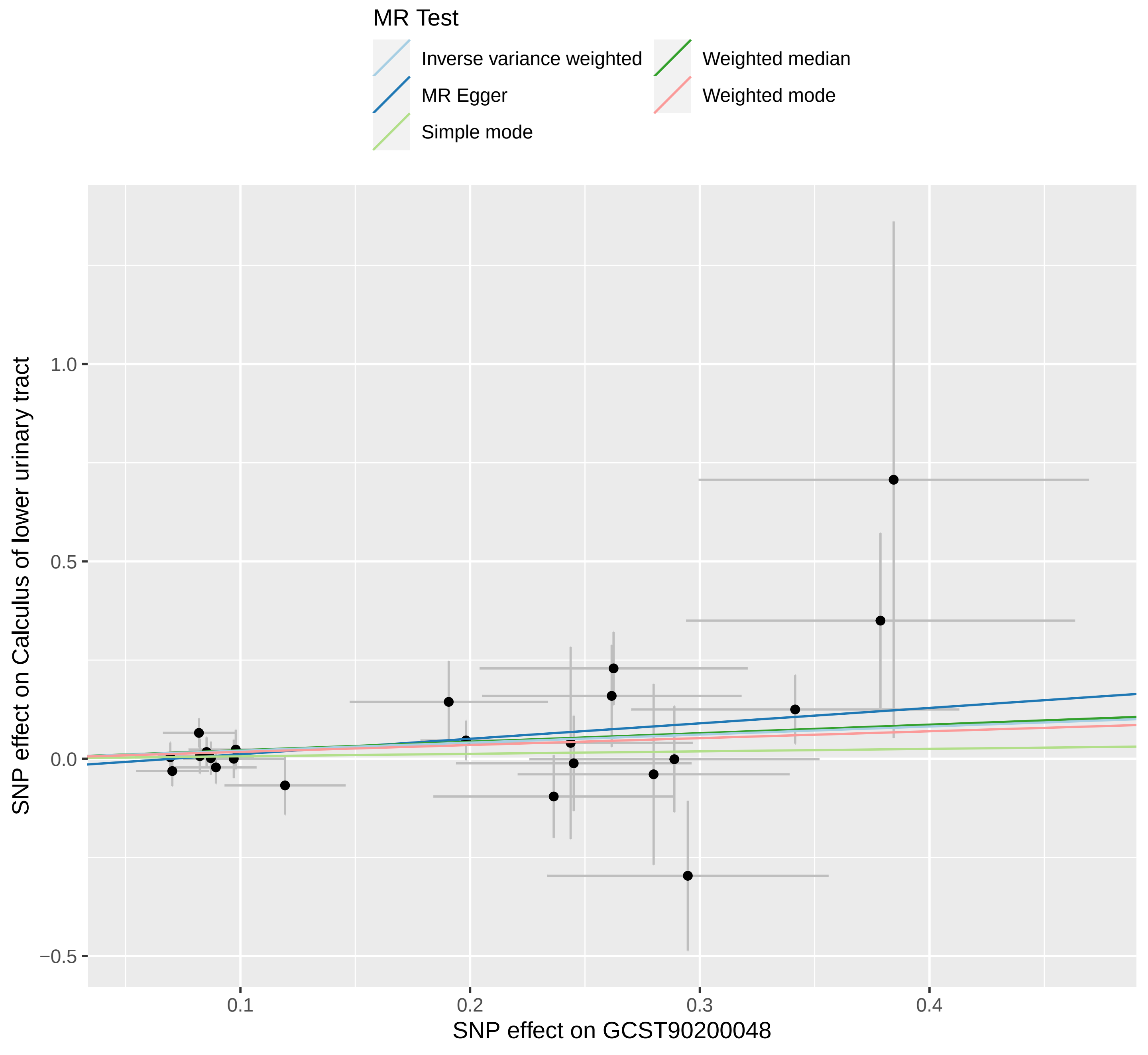
MR Test
Inverse variance weighted
MR Egger
Simple mode
Weighted median
Weighted mode
SNP effect on Calculus of lower urinary tract
1.0
0.5
0.0
−0.5
0.1
0.2
0.3
0.4
SNP effect on GCST90200048

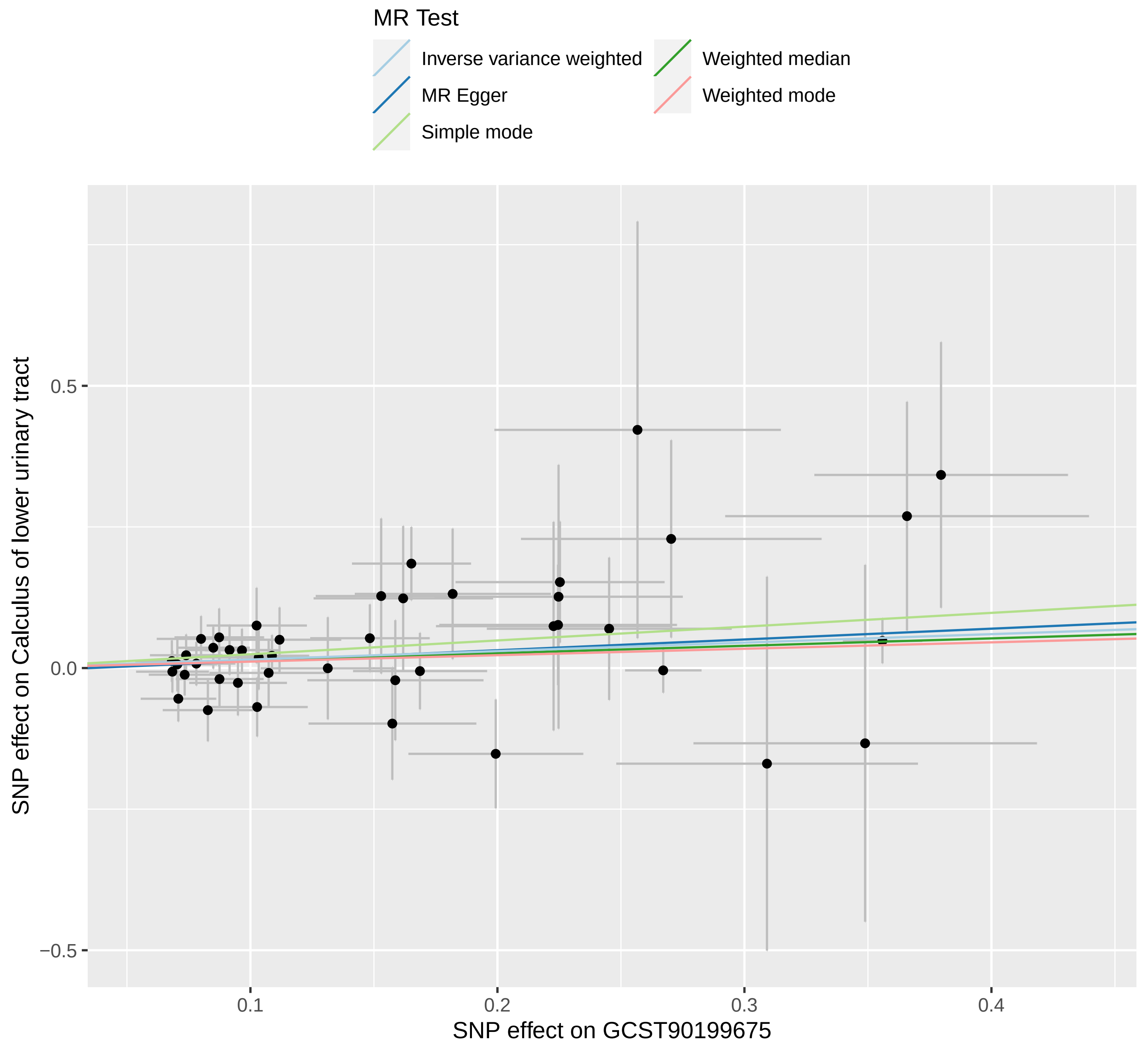

MR Test
Inverse variance weighted
MR Egger
Simple mode
Weighted median
Weighted mode
SNP effect on Calculus of lower urinary tract
0.5
0.0
−0.5
0.1
0.2
0.3
0.4
SNP effect on GCST90199675

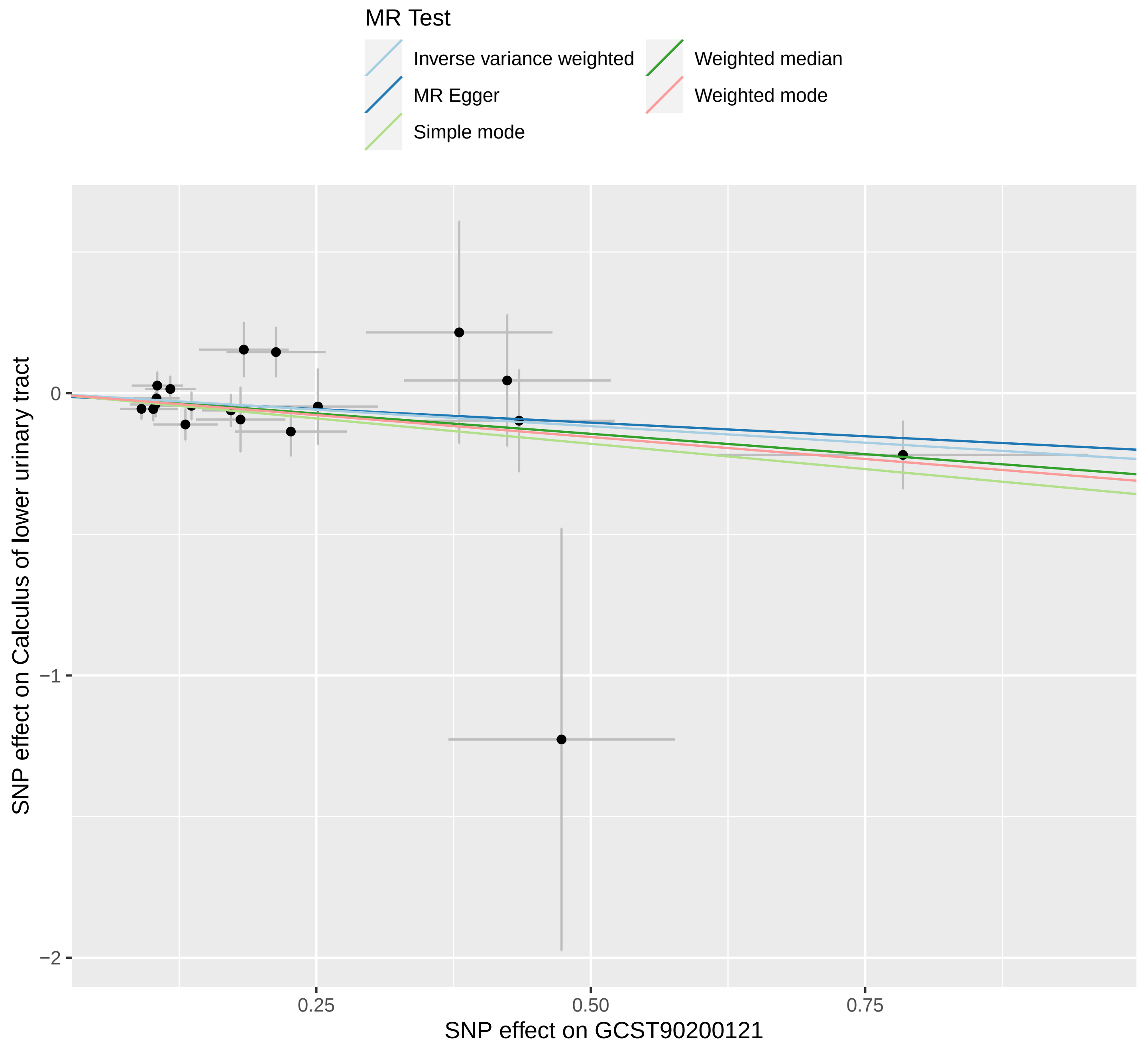

MR Test
Inverse variance weighted
MR Egger
Simple mode
Weighted median
Weighted mode
SNP effect on Calculus of lower urinary tract
0
−1
−2
0.25
0.50
0.75
SNP effect on GCST90200121

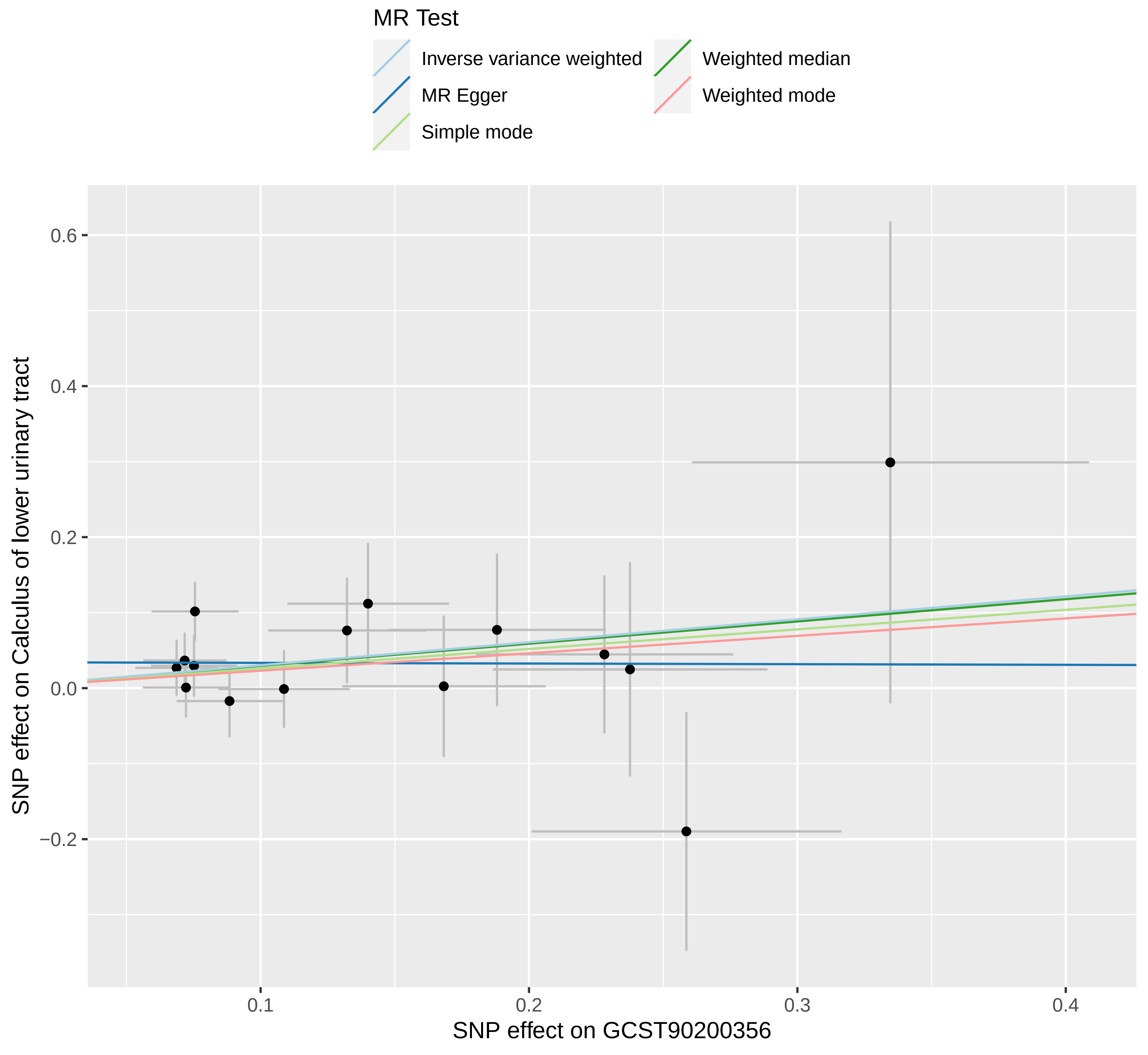

MR Test
Inverse variance weighted
MR Egger
Simple mode
Weighted median
Weighted mode
0.6
0.4
0.2
0.0
−0.2
SNP effect on Calculus of lower urinary tract
0.1
0.2
0.3
0.4
SNP effect on GCST90200356

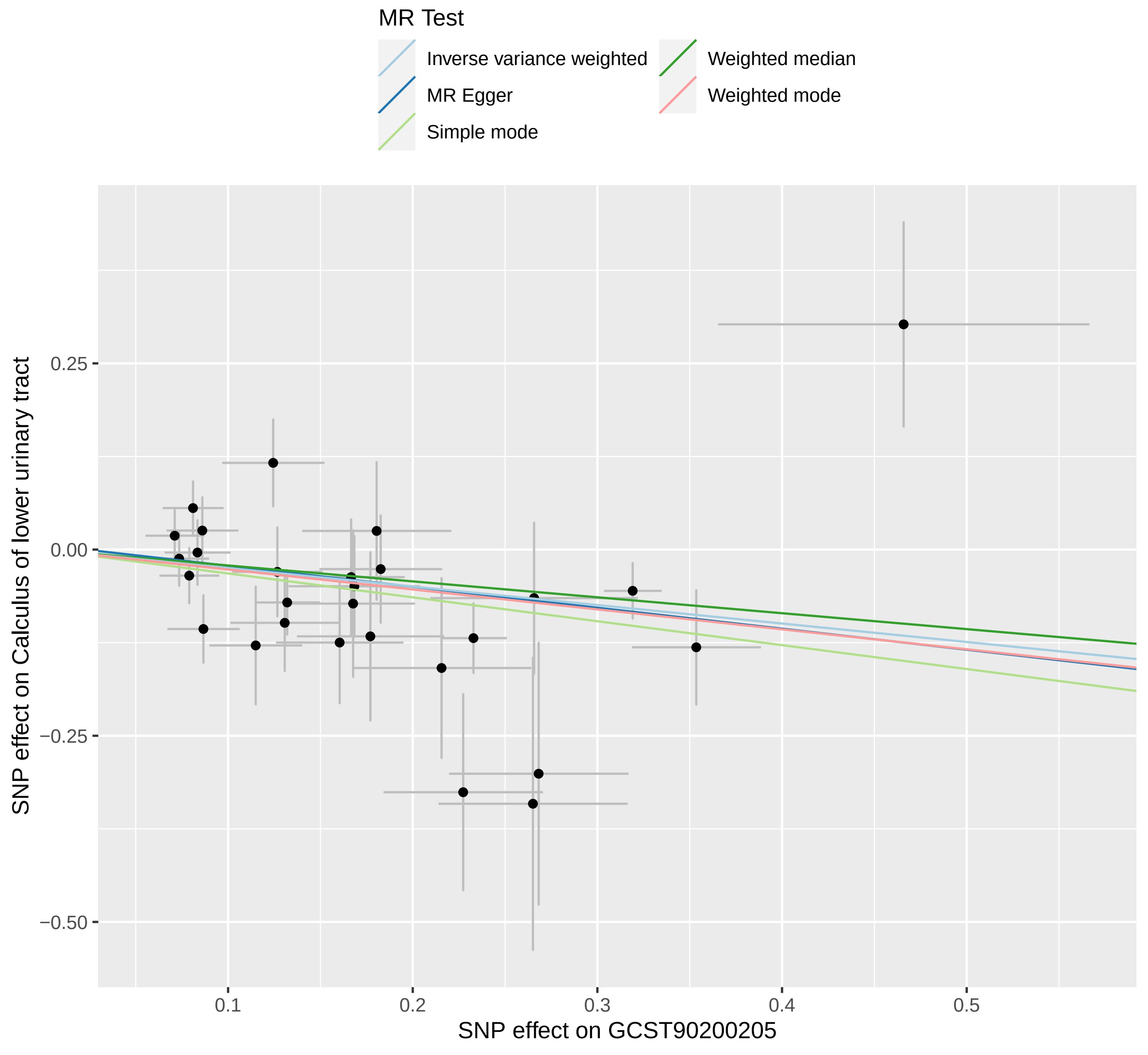
MR Test
Inverse variance weighted
MR Egger
Simple mode
Weighted median
Weighted mode
0.25
0.00
−0.25
−0.50
SNP effect on Calculus of lower urinary tract
0.1
0.2
0.3
0.4
0.5
SNP effect on GCST90200205

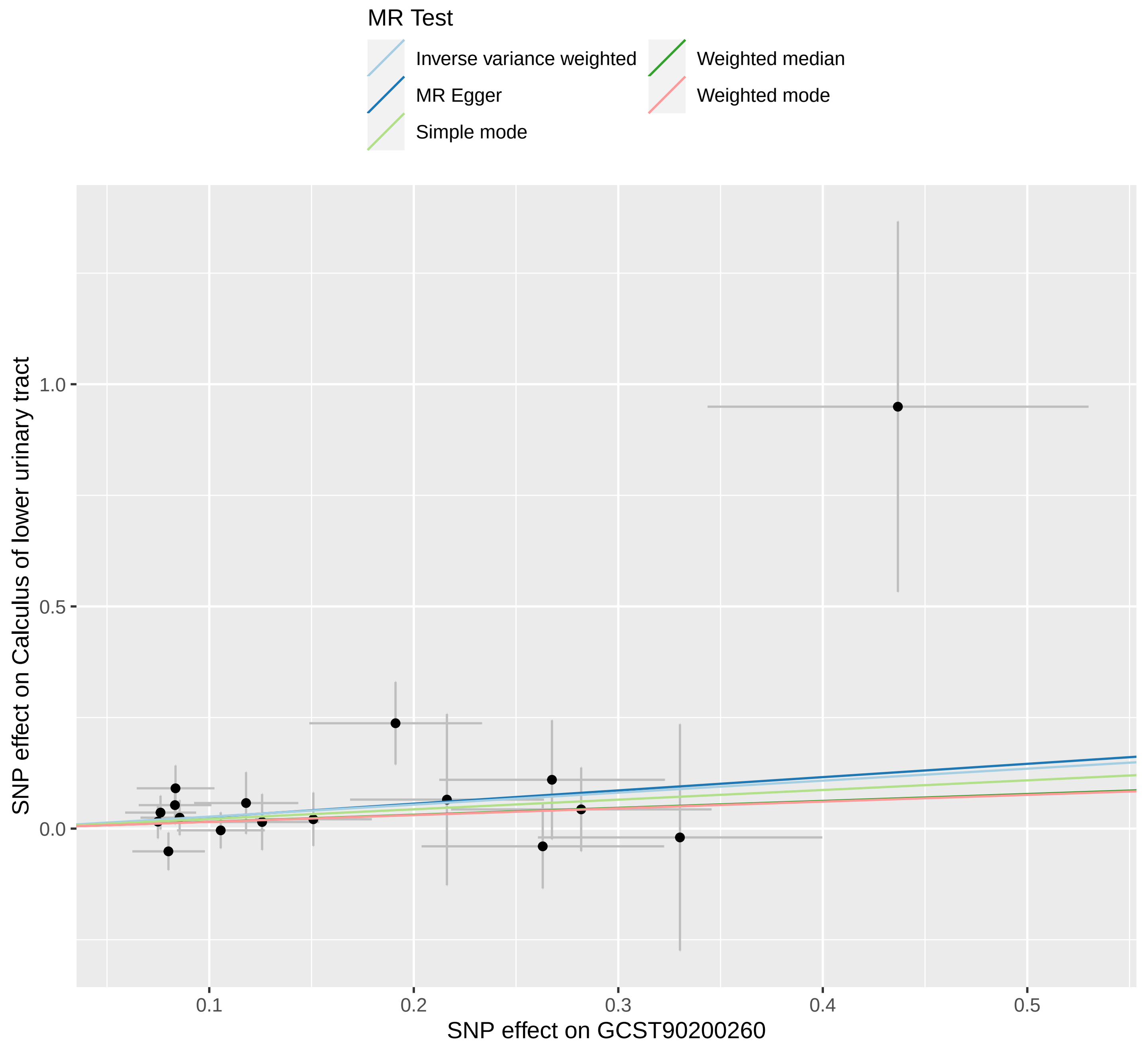
MR Test
Inverse variance weighted
MR Egger
Simple mode
Weighted median
Weighted mode
SNP effect on Calculus of lower urinary tract
1.0
0.5
0.0
0.1
0.2
0.3
0.4
0.5
SNP effect on GCST90200260

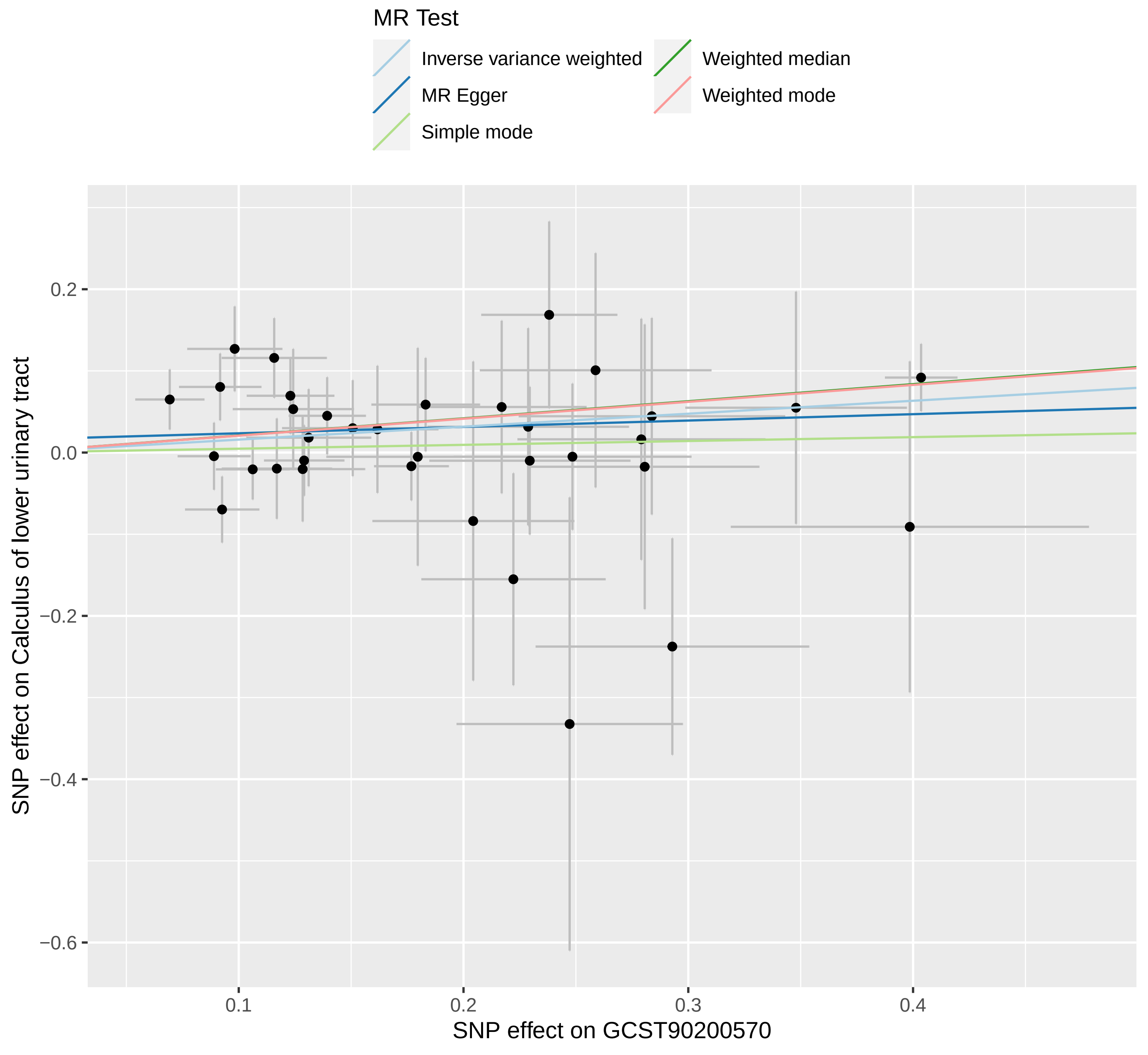
MR Test
Inverse variance weighted
MR Egger
Simple mode
Weighted median
Weighted mode
SNP effect on Calculus of lower urinary tract
0.2
0.0
−0.2
−0.4
−0.6
0.1
0.2
0.3
0.4
SNP effect on GCST90200570

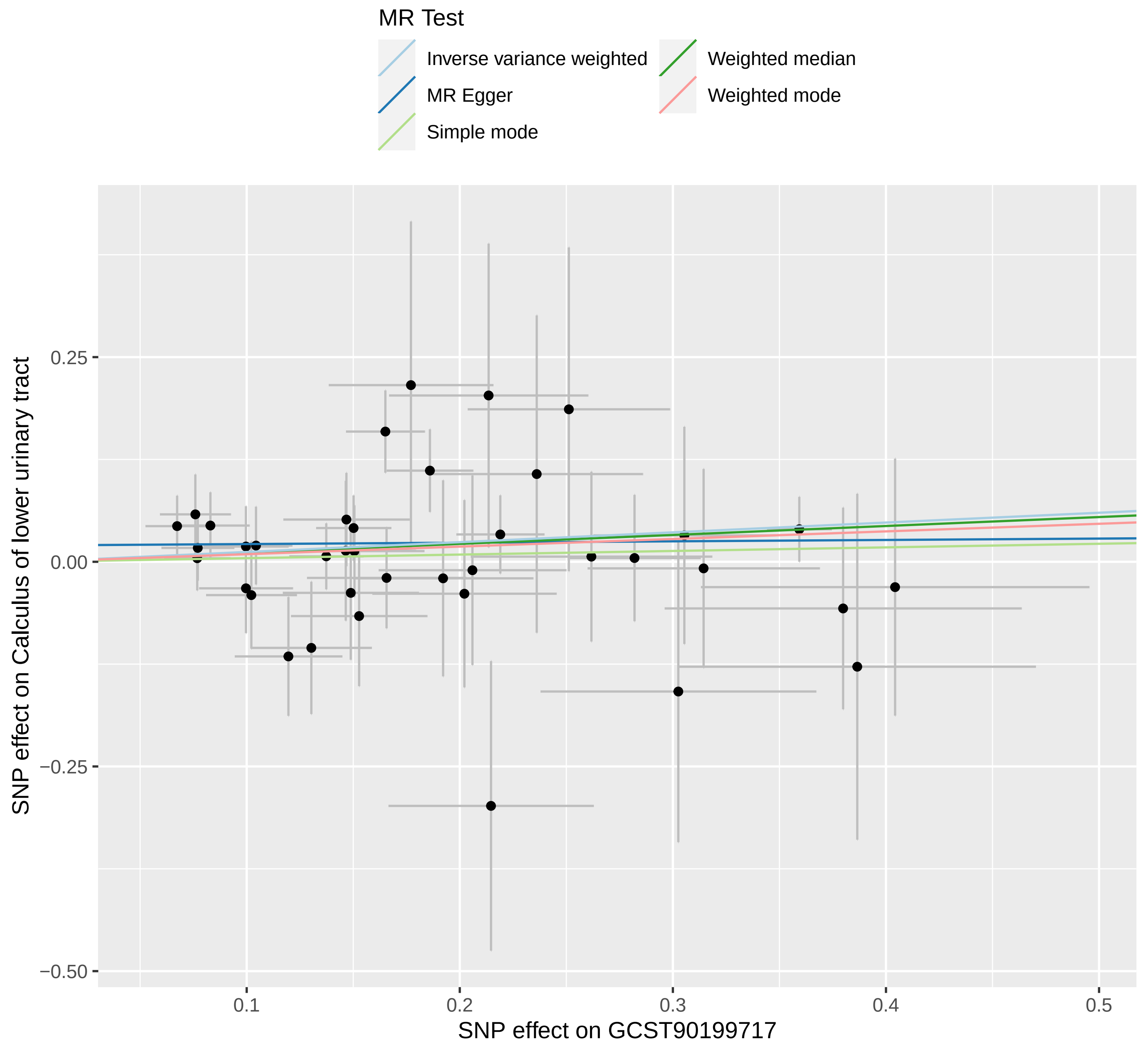

MR Test
Inverse variance weighted
MR Egger
Simple mode
Weighted median
Weighted mode
0.25
0.00
−0.25
−0.50
0.1
0.2
0.3
0.4
0.5
SNP effect on Calculus of lower urinary tract
SNP effect on GCST90199717

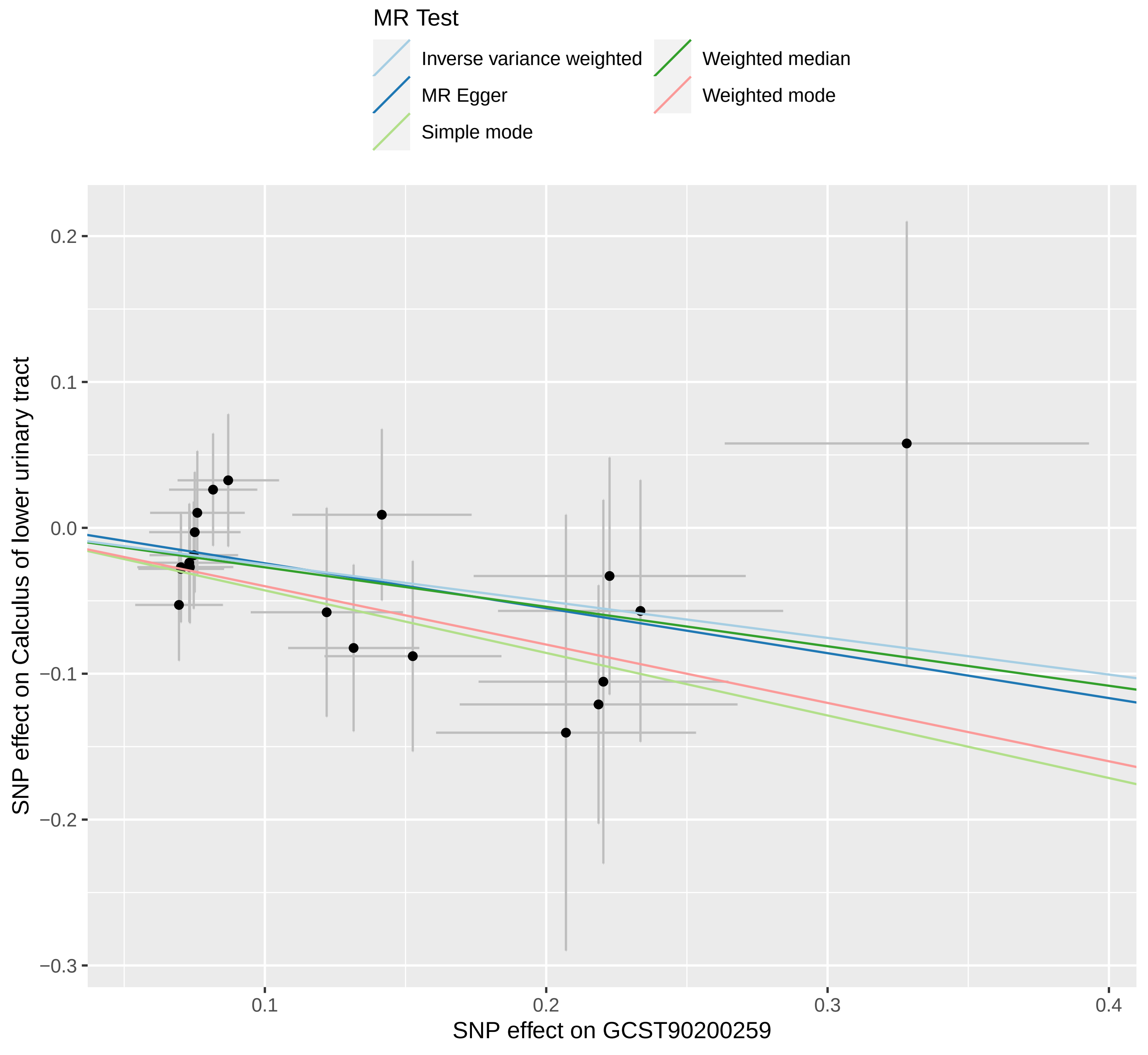
MR Test
Inverse variance weighted
MR Egger
Simple mode
Weighted median
Weighted mode
SNP effect on Calculus of lower urinary tract
0.2
0.1
0.0
−0.1
−0.2
−0.3
0.1
0.2
0.3
0.4
SNP effect on GCST90200259

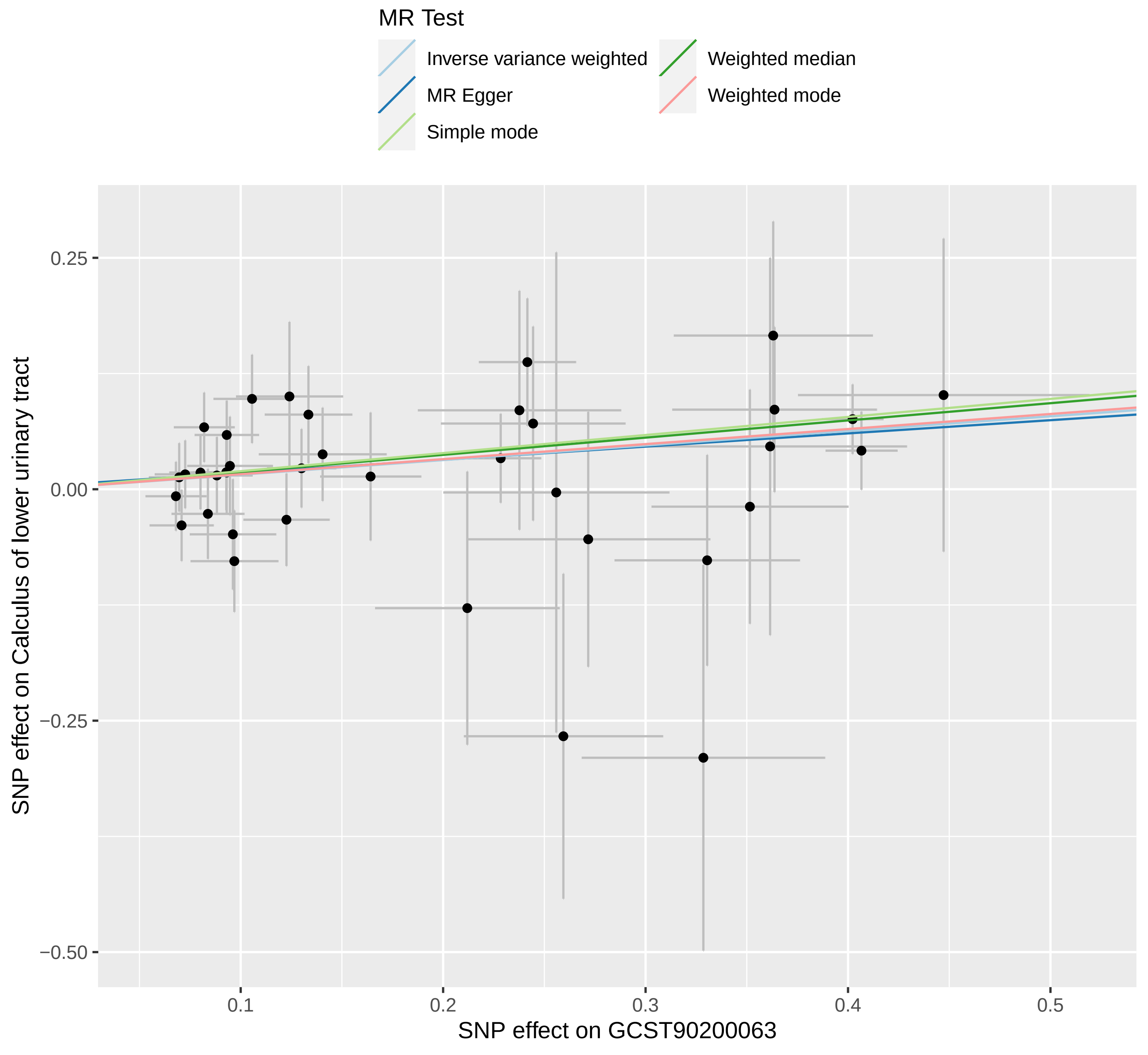
MR Test
Inverse variance weighted
MR Egger
Simple mode
Weighted median
Weighted mode
0.25
0.00
−0.25
−0.50
0.1
0.2
0.3
0.4
0.5
SNP effect on GCST90200063
SNP effect on Calculus of lower urinary tract

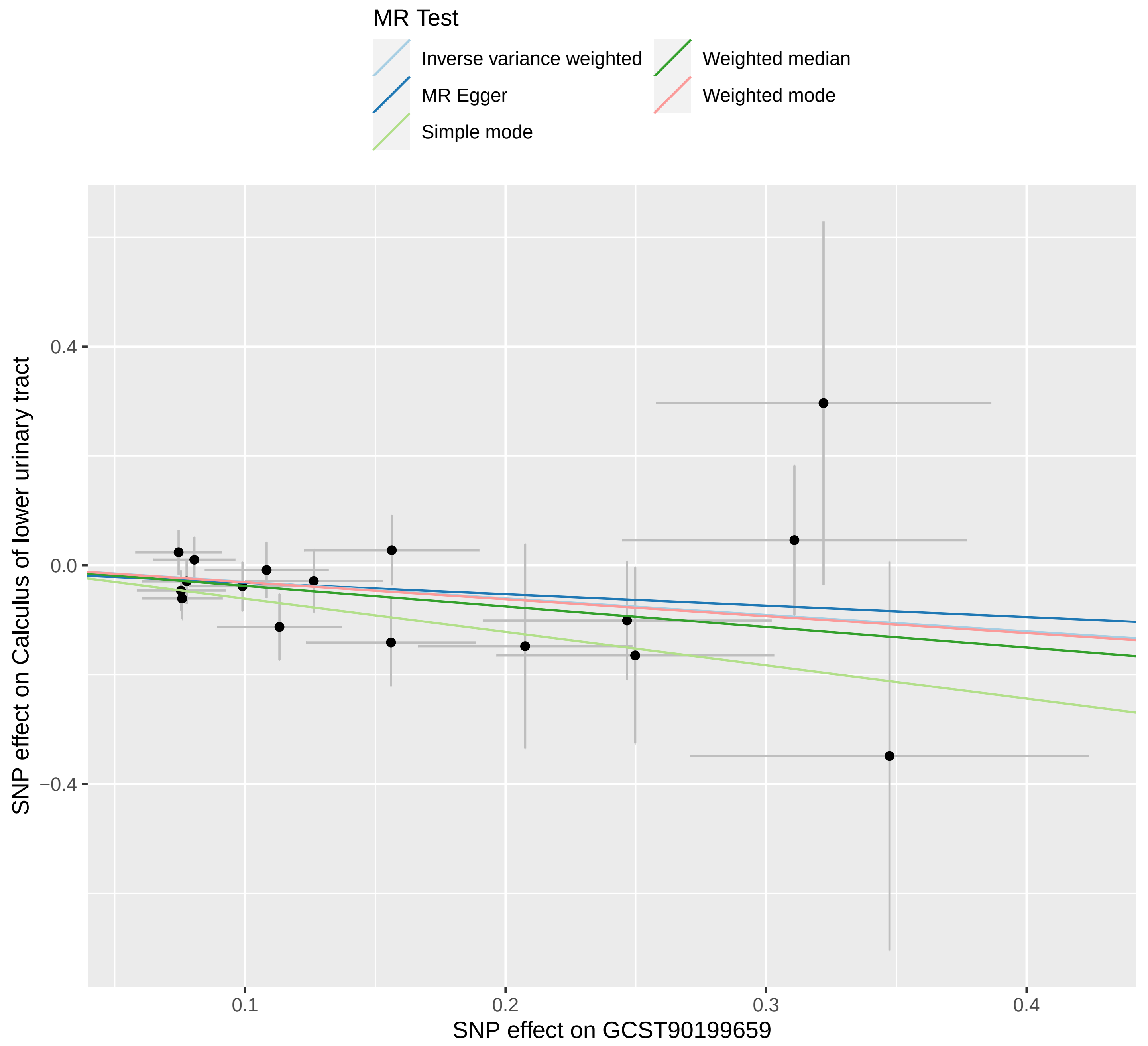
MR Test
Inverse variance weighted
MR Egger
Simple mode
Weighted median
Weighted mode
0.4
0.0
−0.4
0.1
0.2
0.3
0.4
SNP effect on Calculus of lower urinary tract
SNP effect on GCST90199659

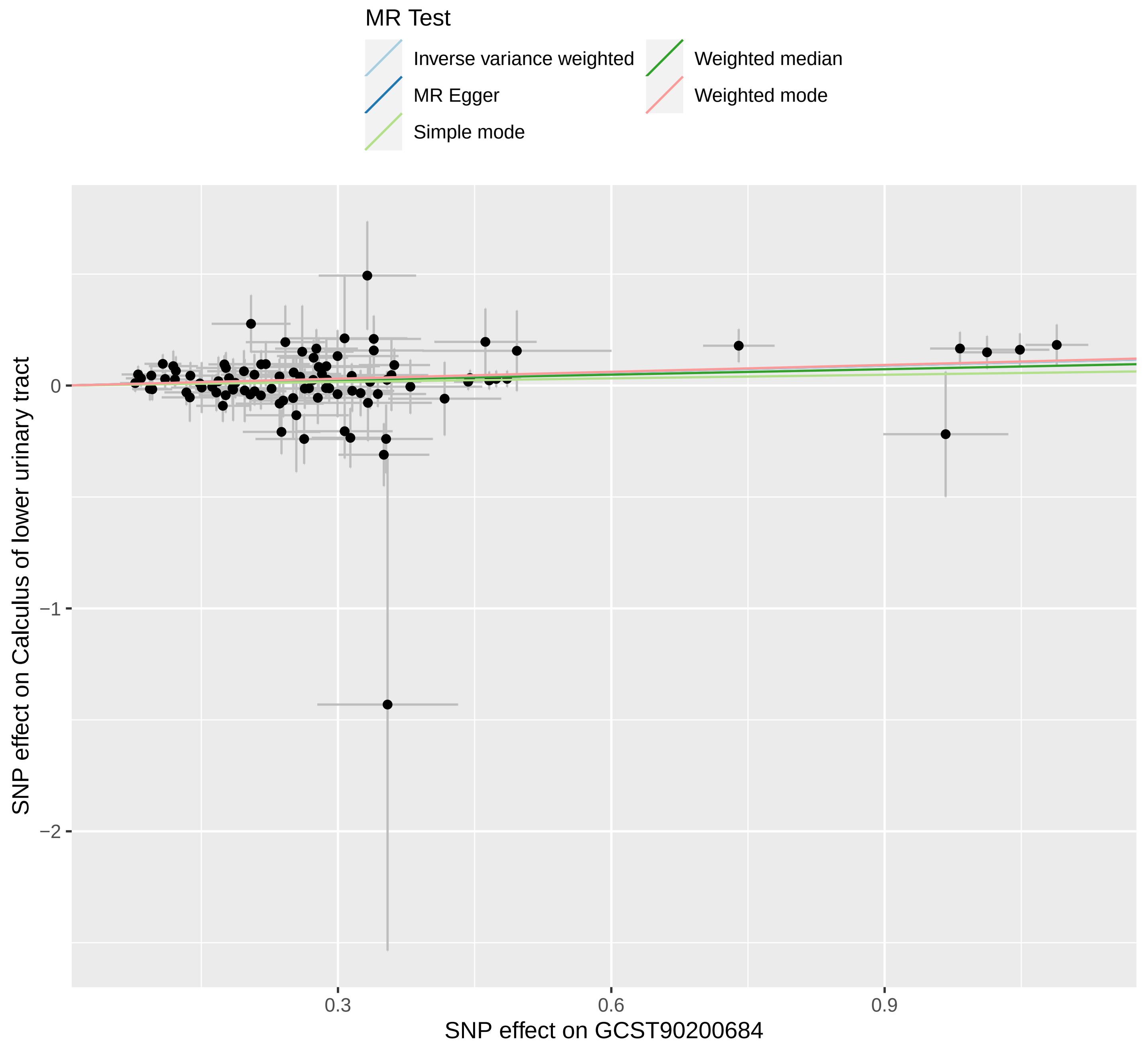
MR Test
Inverse variance weighted
MR Egger
Simple mode
Weighted median
Weighted mode
SNP effect on Calculus of lower urinary tract
0
−1
−2
0.3
0.6
0.9
SNP effect on GCST90200684

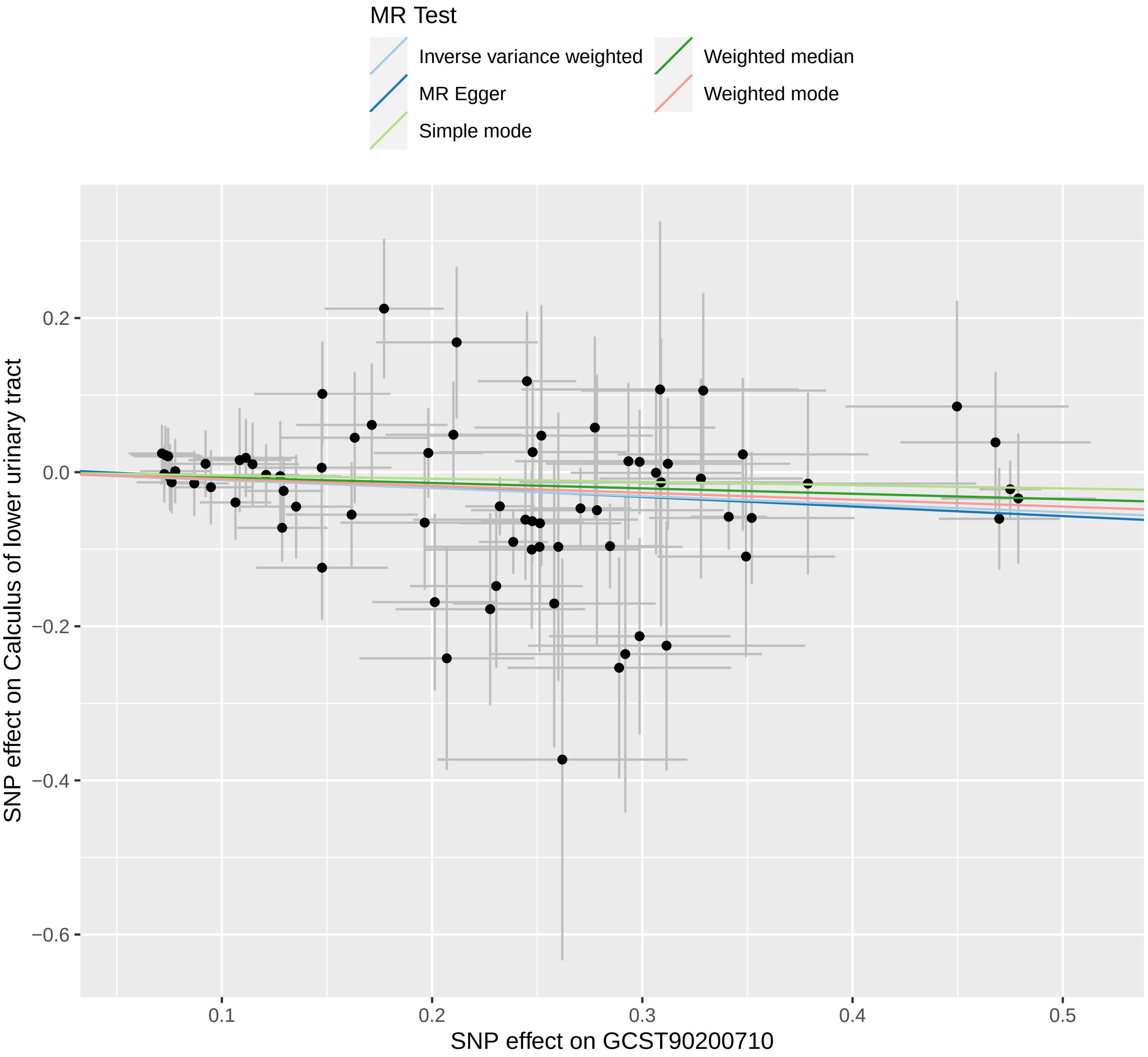
MR Test
Inverse variance weighted
MR Egger
Simple mode
Weighted median
Weighted mode
0.2
0.0
−0.2
−0.4
−0.6
0.1
0.2
0.3
0.4
0.5
SNP effect on Calculus of lower urinary tract
SNP effect on GCST90200710

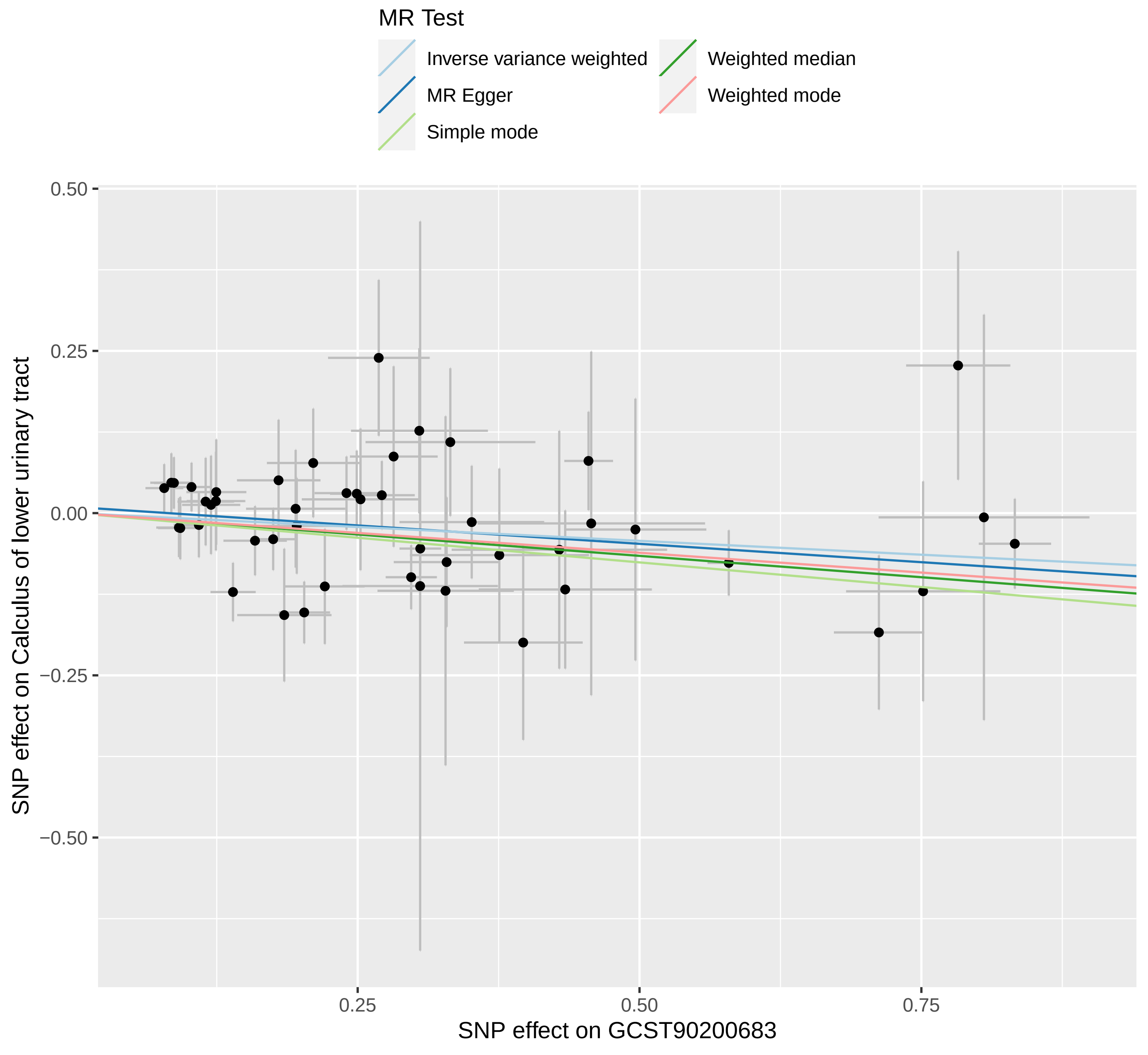
MR Test
Inverse variance weighted
MR Egger
Simple mode
Weighted median
Weighted mode
0.50
0.25
0.00
−0.25
−0.50
0.25
0.50
0.75
SNP effect on GCST90200683
SNP effect on Calculus of lower urinary tract

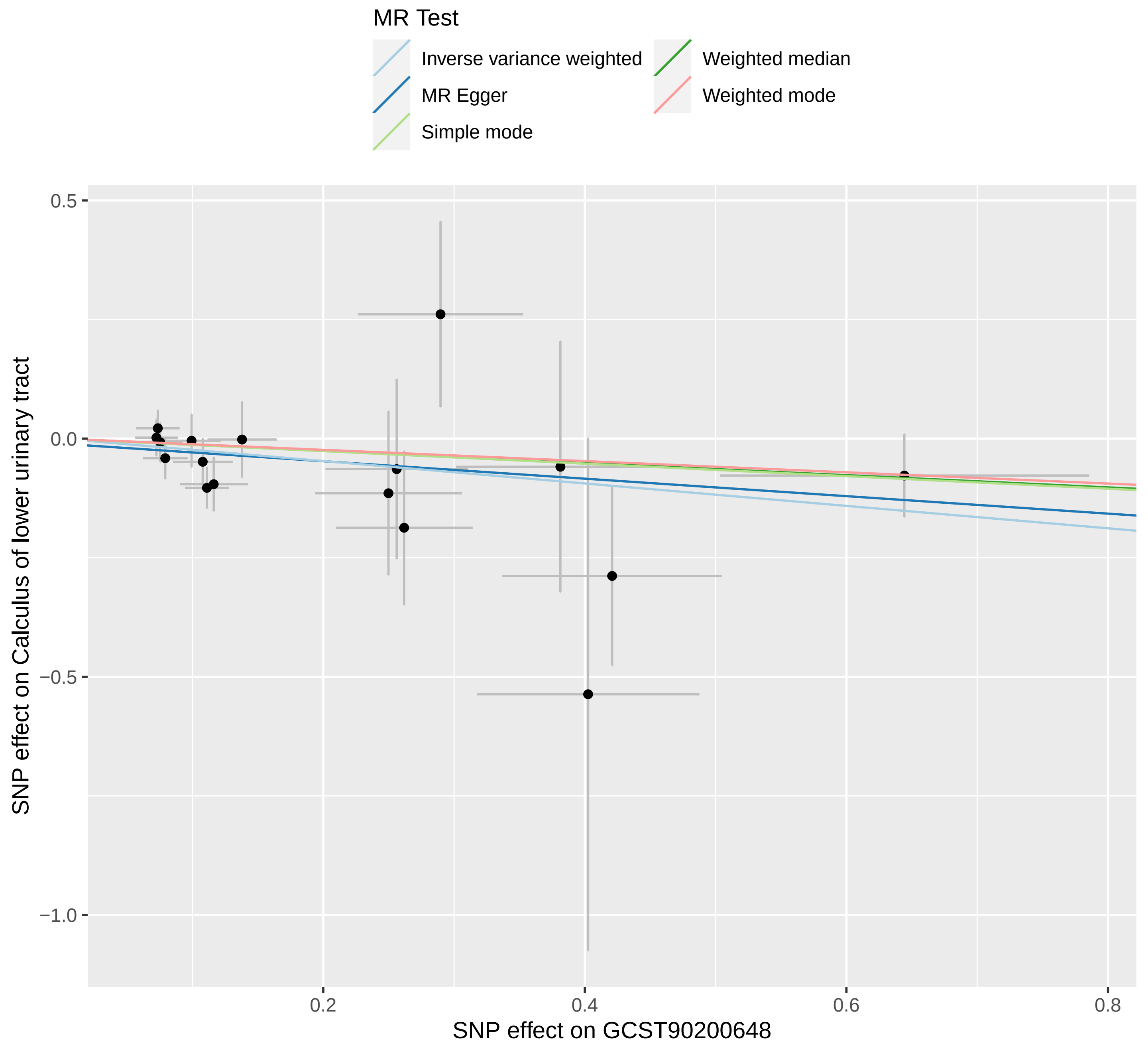

MR Test
Inverse variance weighted
MR Egger
Simple mode
Weighted median
Weighted mode
0.5
0.0
−0.5
−1.0
0.2
0.4
0.6
0.8
SNP effect on Calculus of lower urinary tract
SNP effect on GCST90200648

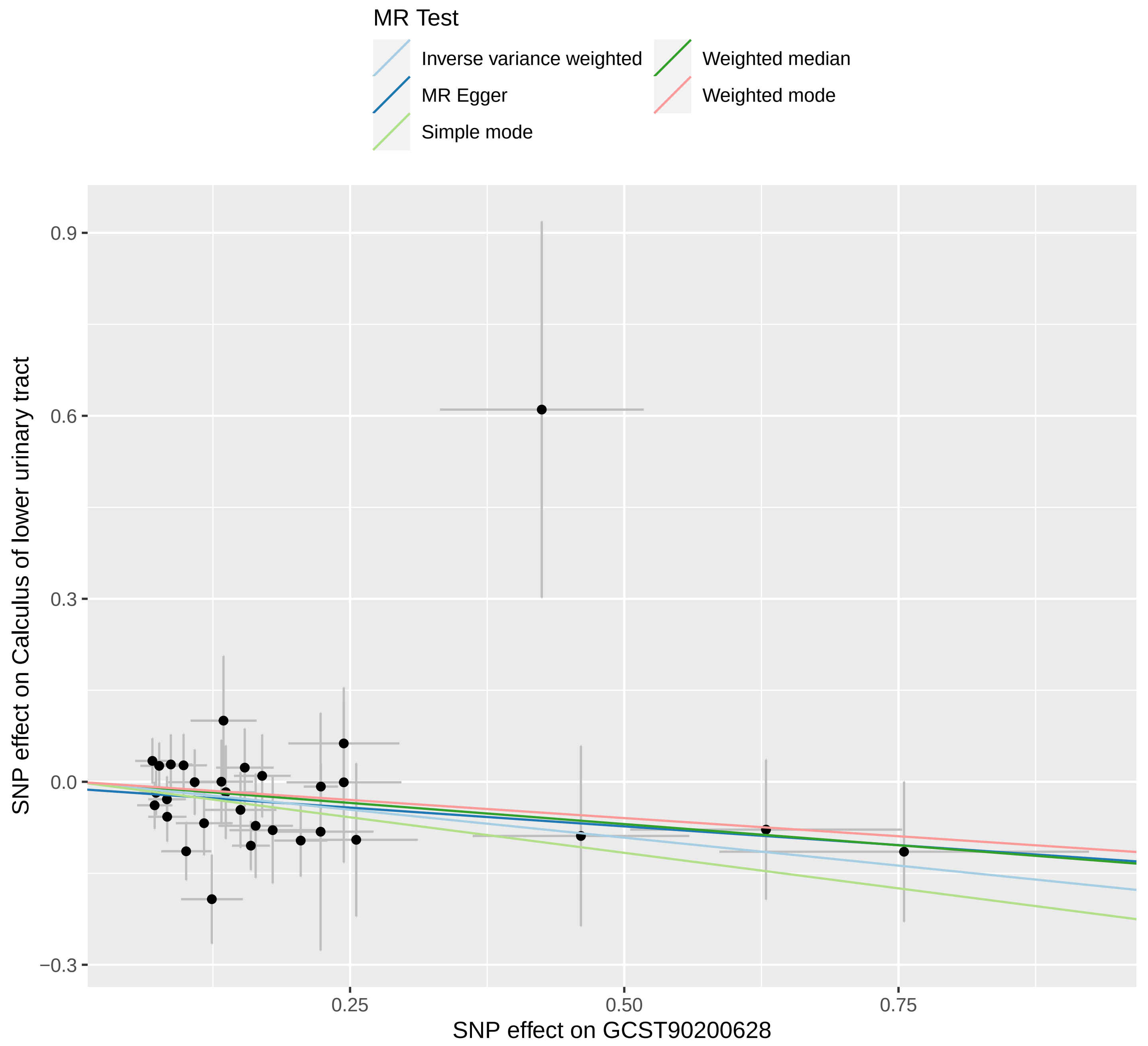
MR Test
Inverse variance weighted
MR Egger
Simple mode
Weighted median
Weighted mode
SNP effect on Calculus of lower urinary tract
0.9
0.6
0.3
0.0
−0.3
0.25
0.50
0.75
SNP effect on GCST90200628

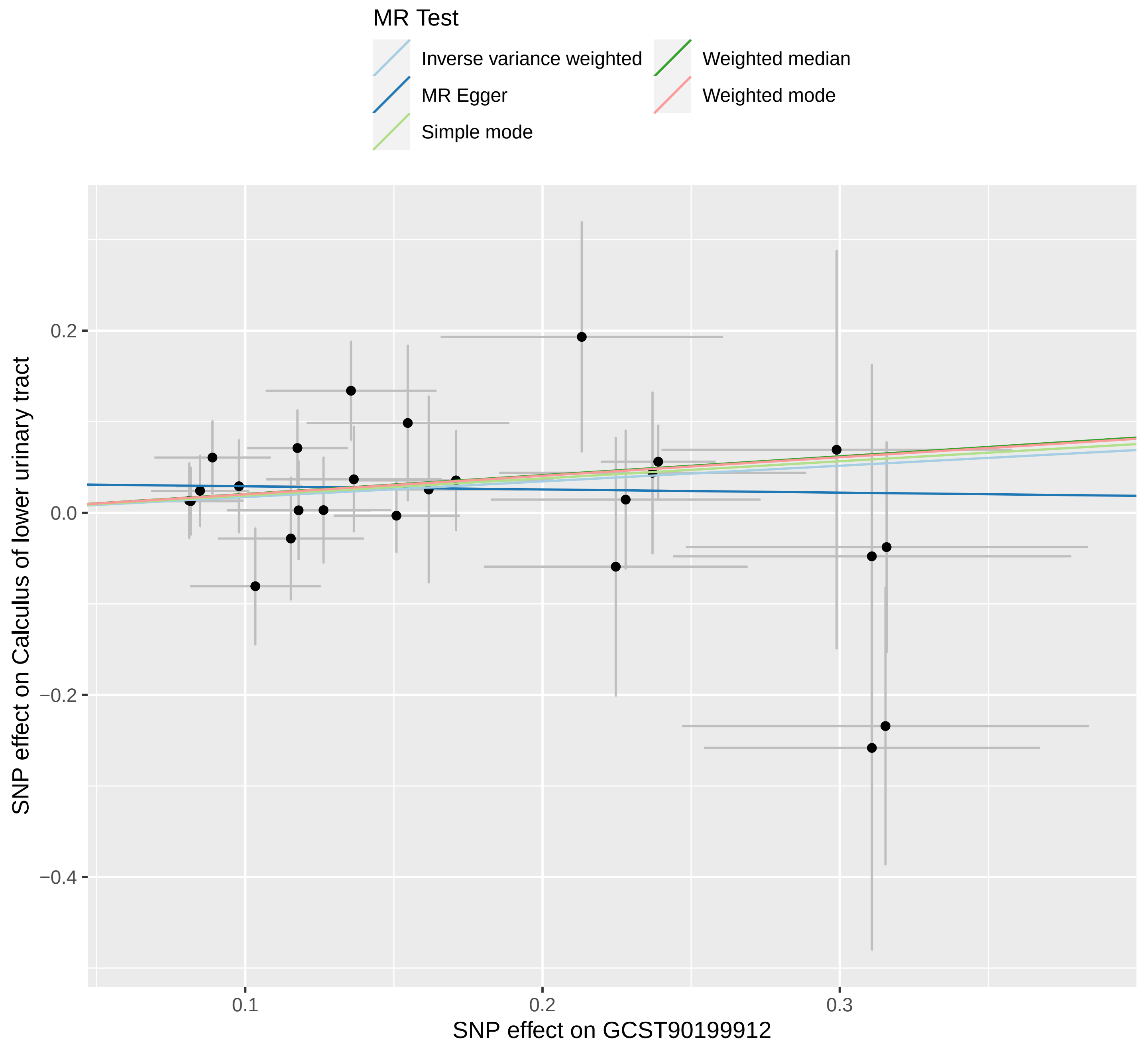

MR Test
Inverse variance weighted
MR Egger
Simple mode
Weighted median
Weighted mode
0.2
0.0
−0.2
−0.4
0.1
0.2
0.3
SNP effect on Calculus of lower urinary tract
SNP effect on GCST90199912

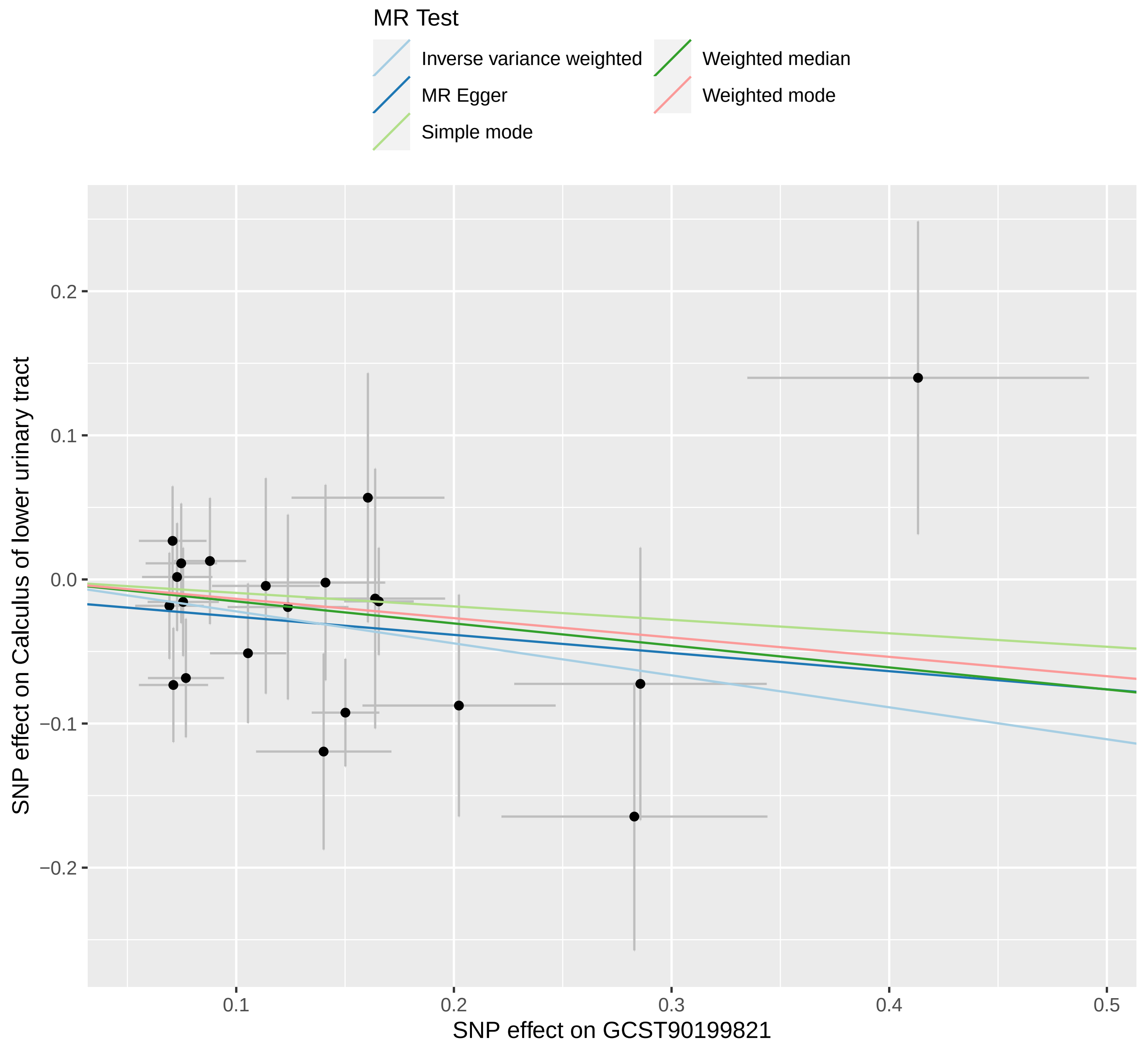
MR Test
Inverse variance weighted
MR Egger
Simple mode
Weighted median
Weighted mode
SNP effect on Calculus of lower urinary tract
0.2
0.1
0.0
−0.1
−0.2
0.1
0.2
0.3
0.4
0.5
SNP effect on GCST90199821

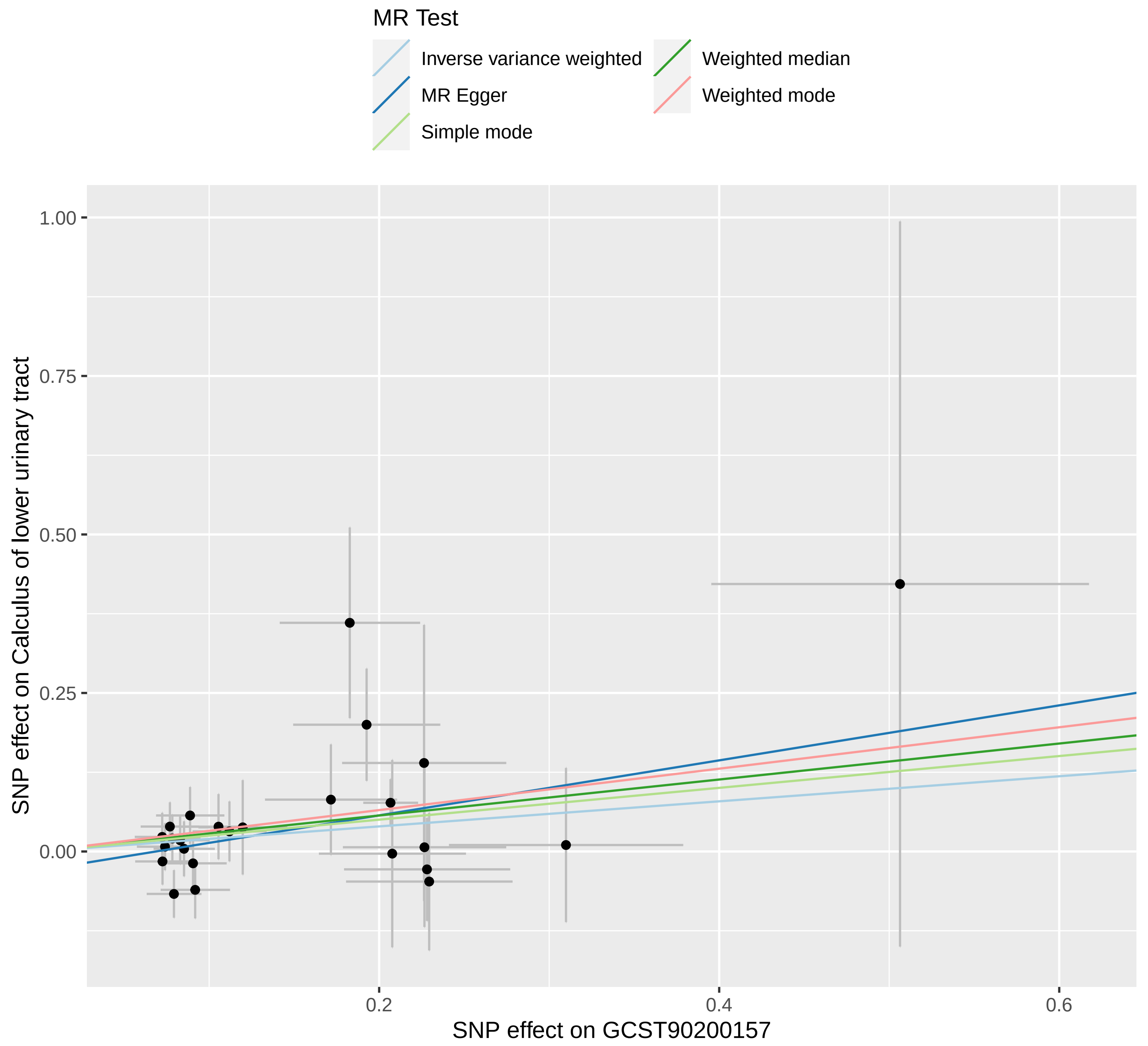
MR Test
Inverse variance weighted
MR Egger
Simple mode
Weighted median
Weighted mode
SNP effect on Calculus of lower urinary tract
1.00
0.75
0.50
0.25
0.00
0.2
0.4
0.6
SNP effect on GCST90200157

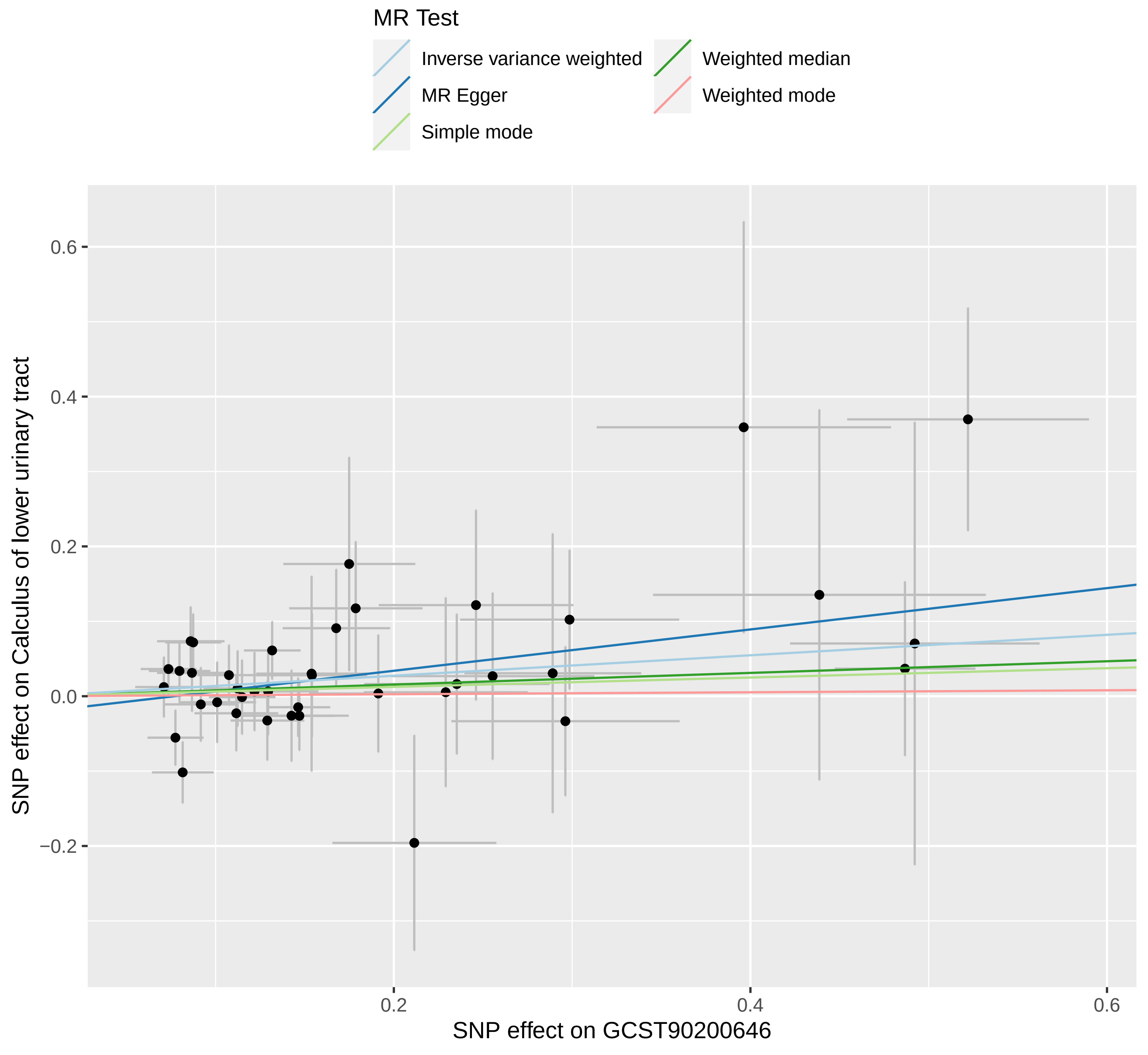

MR Test
Inverse variance weighted
MR Egger
Simple mode
Weighted median
Weighted mode
0.6
0.4
0.2
0.0
−0.2
0.2
0.4
0.6
SNP effect on Calculus of lower urinary tract
SNP effect on GCST90200646

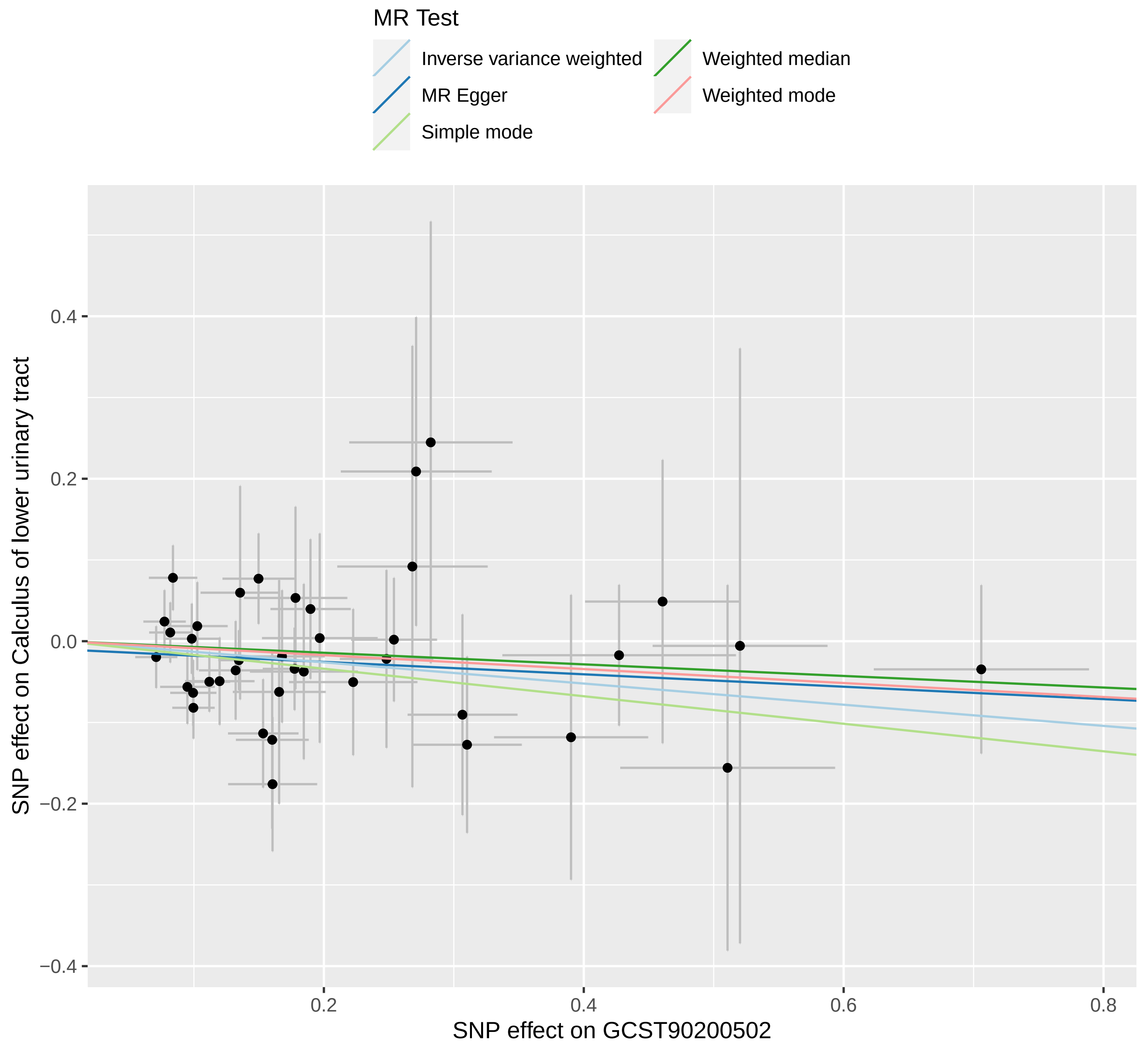

MR Test
Inverse variance weighted
MR Egger
Simple mode
Weighted median
Weighted mode
SNP effect on Calculus of lower urinary tract
0.4
0.2
0.0
−0.2
−0.4
0.2
0.4
0.6
0.8
SNP effect on GCST90200502

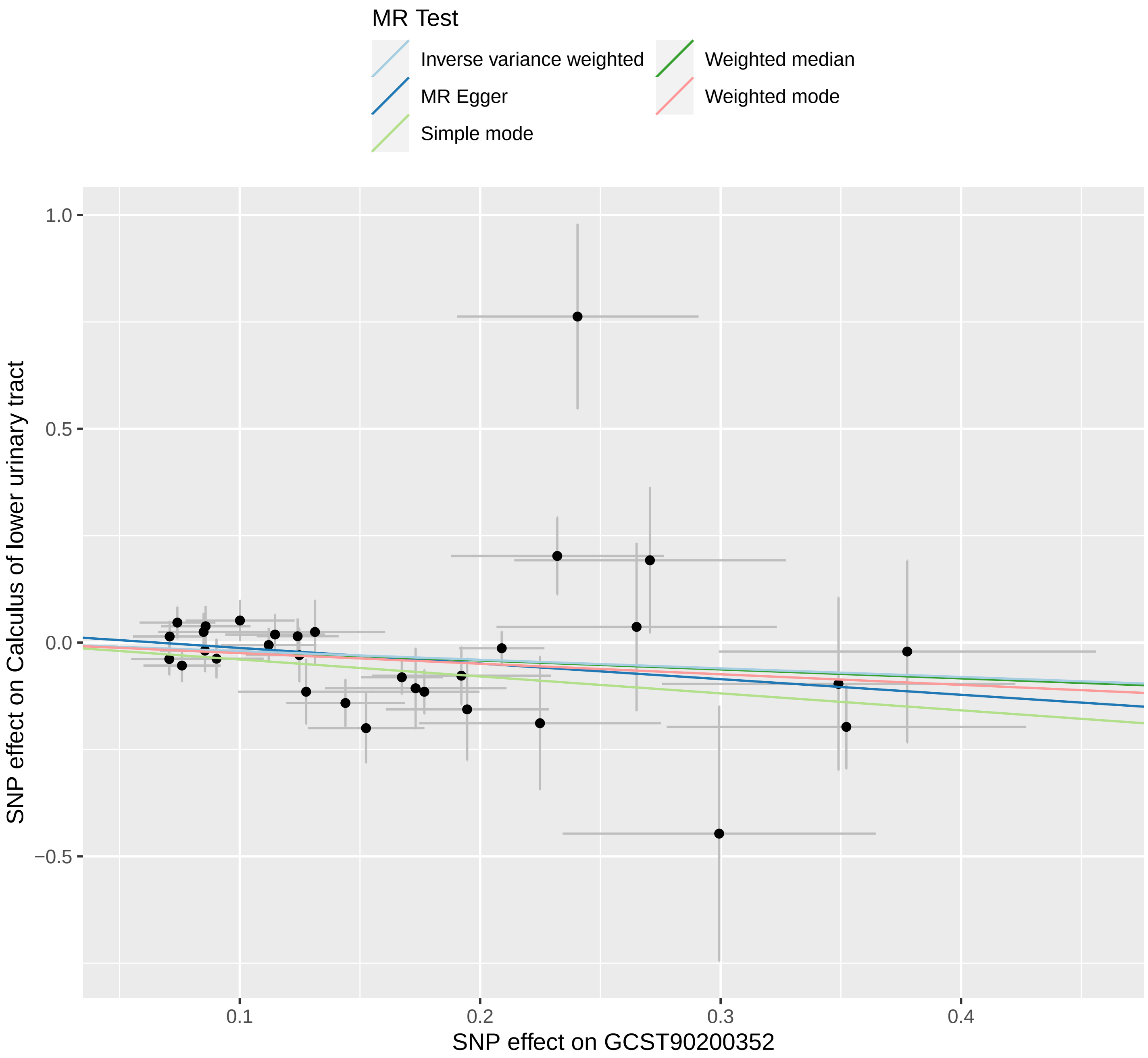
MR Test
Inverse variance weighted
MR Egger
Simple mode
Weighted median
Weighted mode
SNP effect on Calculus of lower urinary tract
1.0
0.5
0.0
−0.5
0.1
0.2
0.3
0.4
SNP effect on GCST90200352

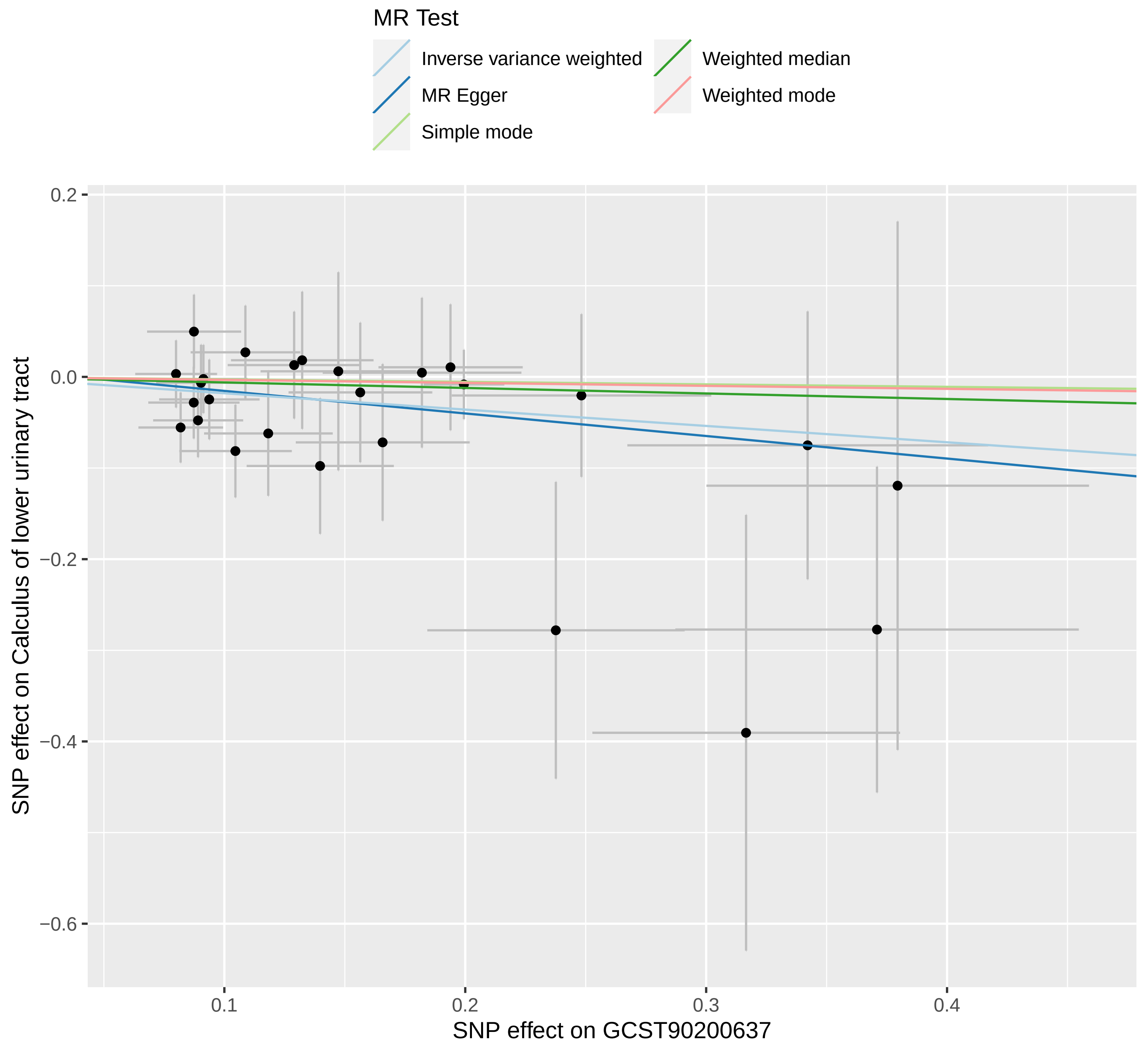
MR Test
Inverse variance weighted
MR Egger
Simple mode
Weighted median
Weighted mode
0.2
0.0
−0.2
−0.4
−0.6
0.1
0.2
0.3
0.4
SNP effect on Calculus of lower urinary tract
SNP effect on GCST90200637

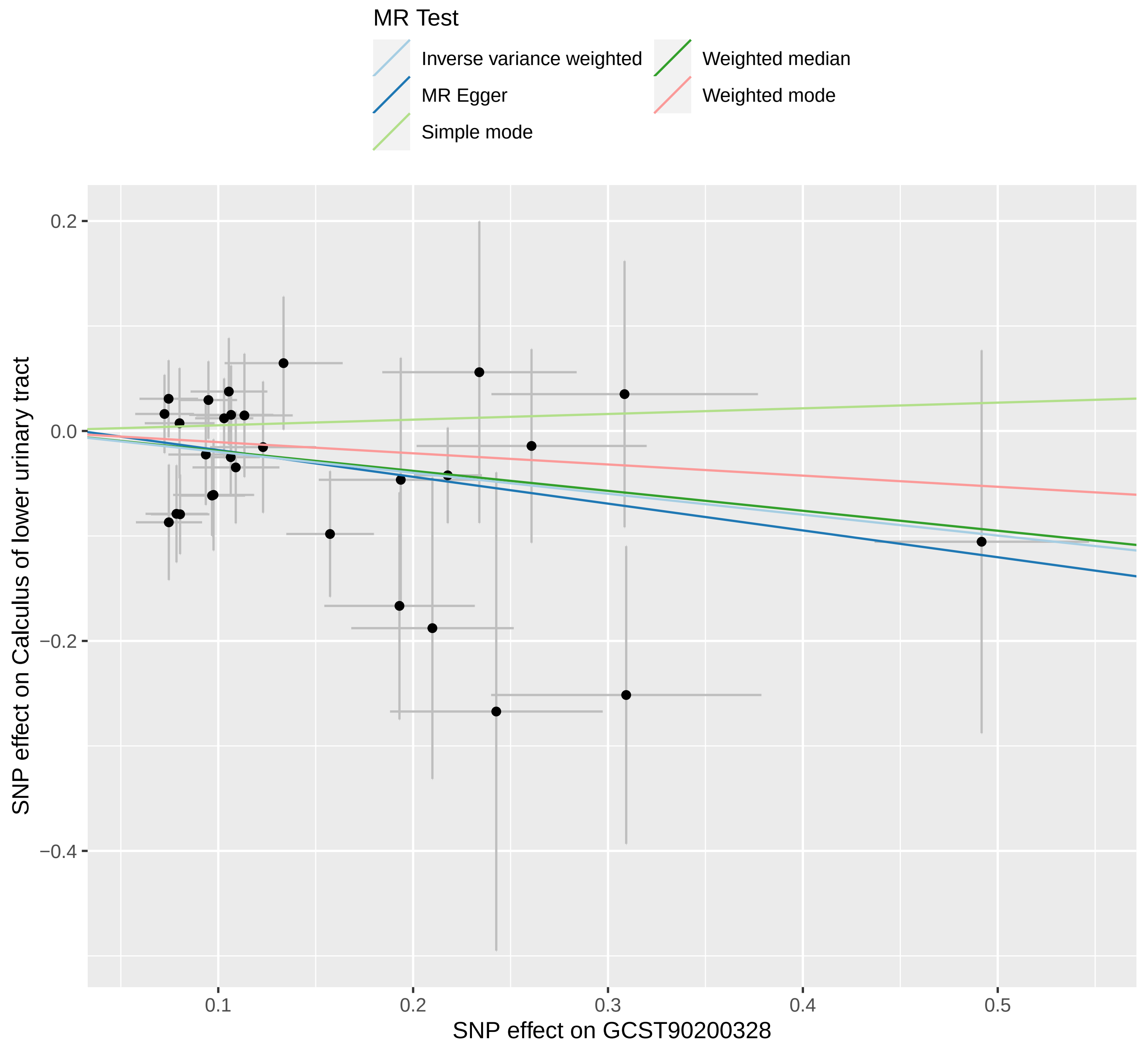

MR Test
Inverse variance weighted
MR Egger
Simple mode
Weighted median
Weighted mode
0.2
0.0
−0.2
−0.4
0.1
0.2
0.3
0.4
0.5
SNP effect on Calculus of lower urinary tract
SNP effect on GCST90200328

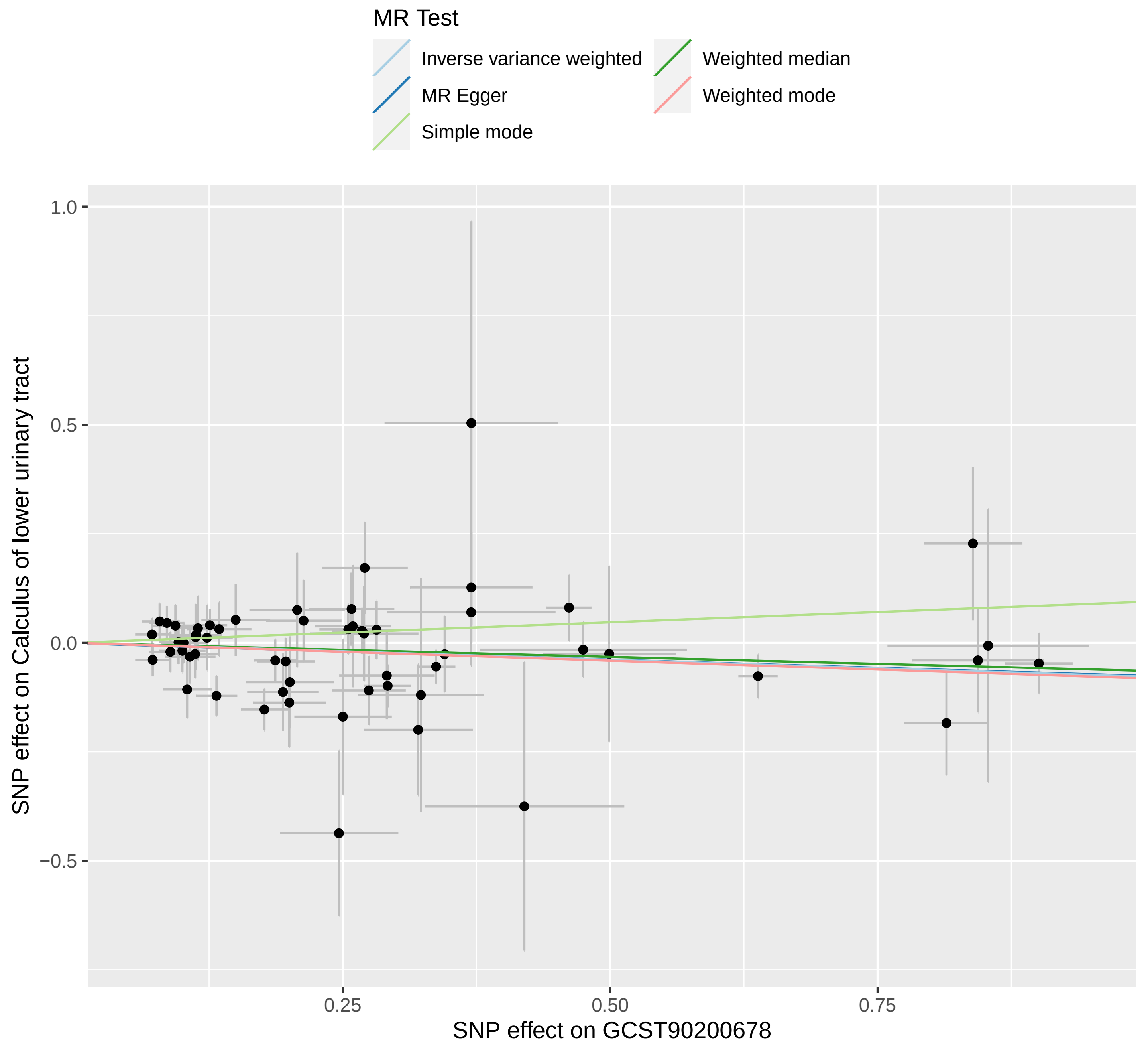
MR Test
Inverse variance weighted
MR Egger
Simple mode
Weighted median
Weighted mode
1.0
0.5
0.0
−0.5
0.25
0.50
0.75
SNP effect on Calculus of lower urinary tract
SNP effect on GCST90200678

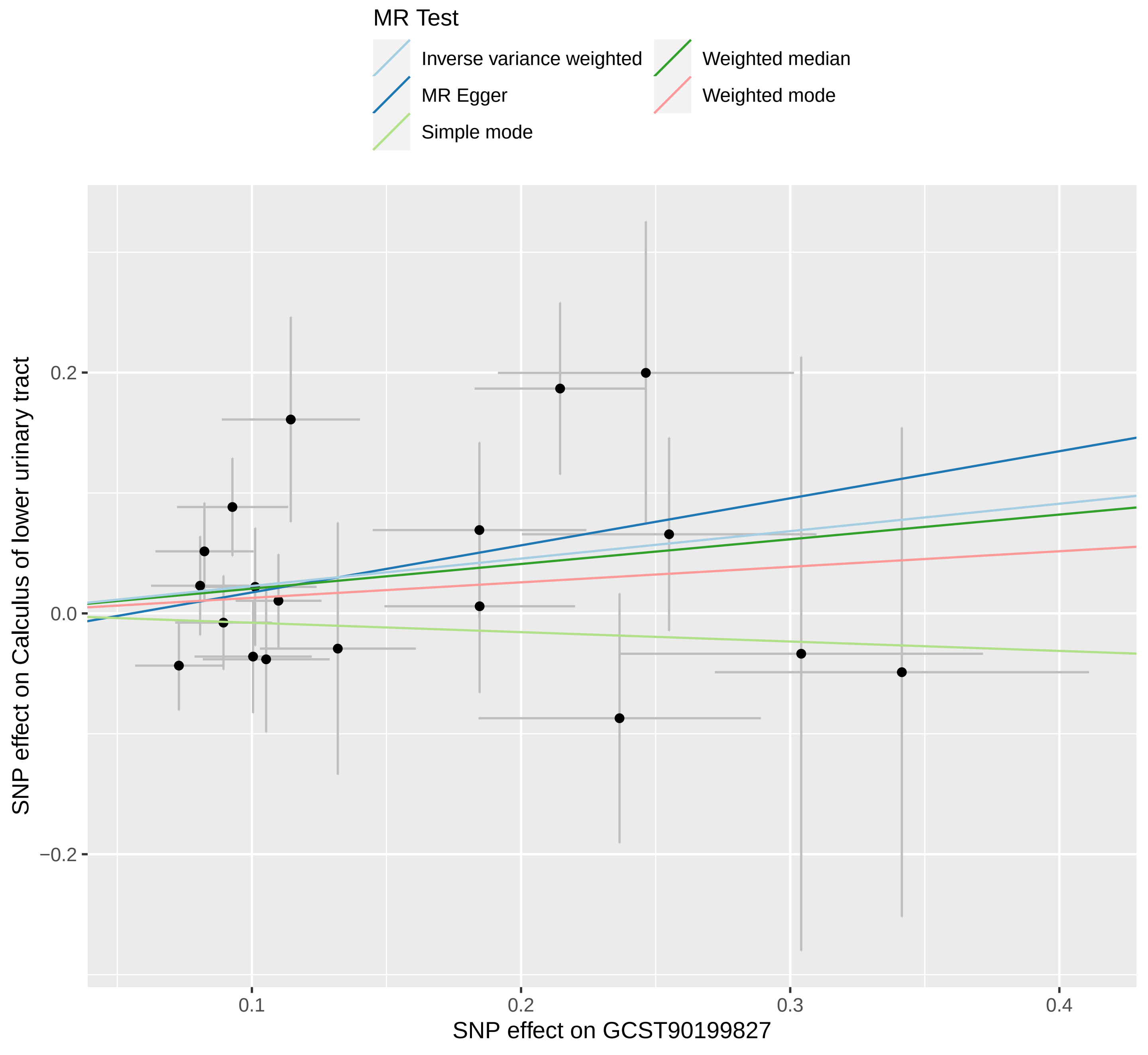

MR Test
Inverse variance weighted
MR Egger
Simple mode
Weighted median
Weighted mode
SNP effect on Calculus of lower urinary tract
0.2
0.0
−0.2
0.1
0.2
0.3
0.4
SNP effect on GCST90199827

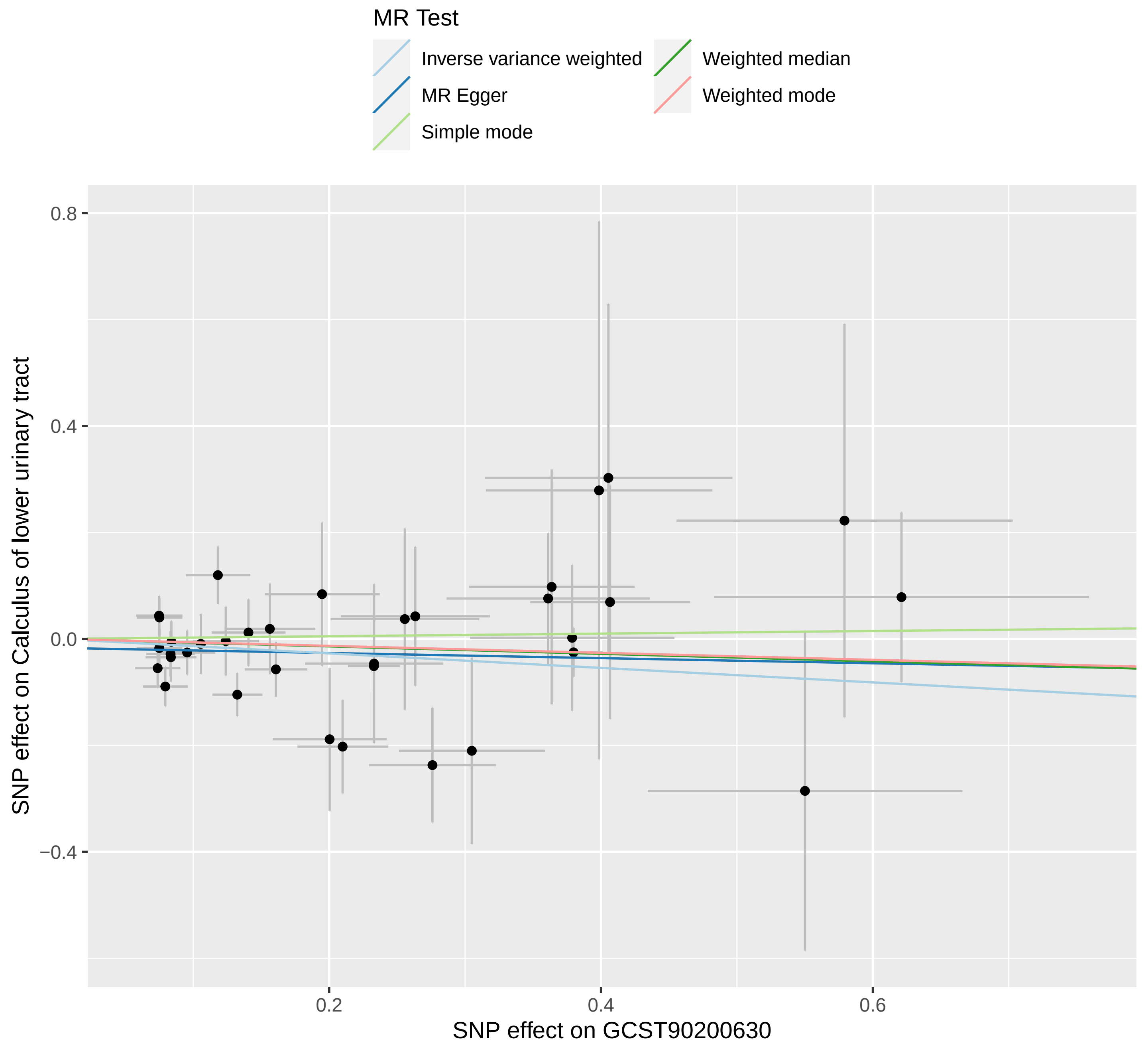

MR Test
Inverse variance weighted
MR Egger
Simple mode
Weighted median
Weighted mode
0.8
0.4
0.0
−0.4
0.2
0.4
0.6
SNP effect on Calculus of lower urinary tract
SNP effect on GCST90200630

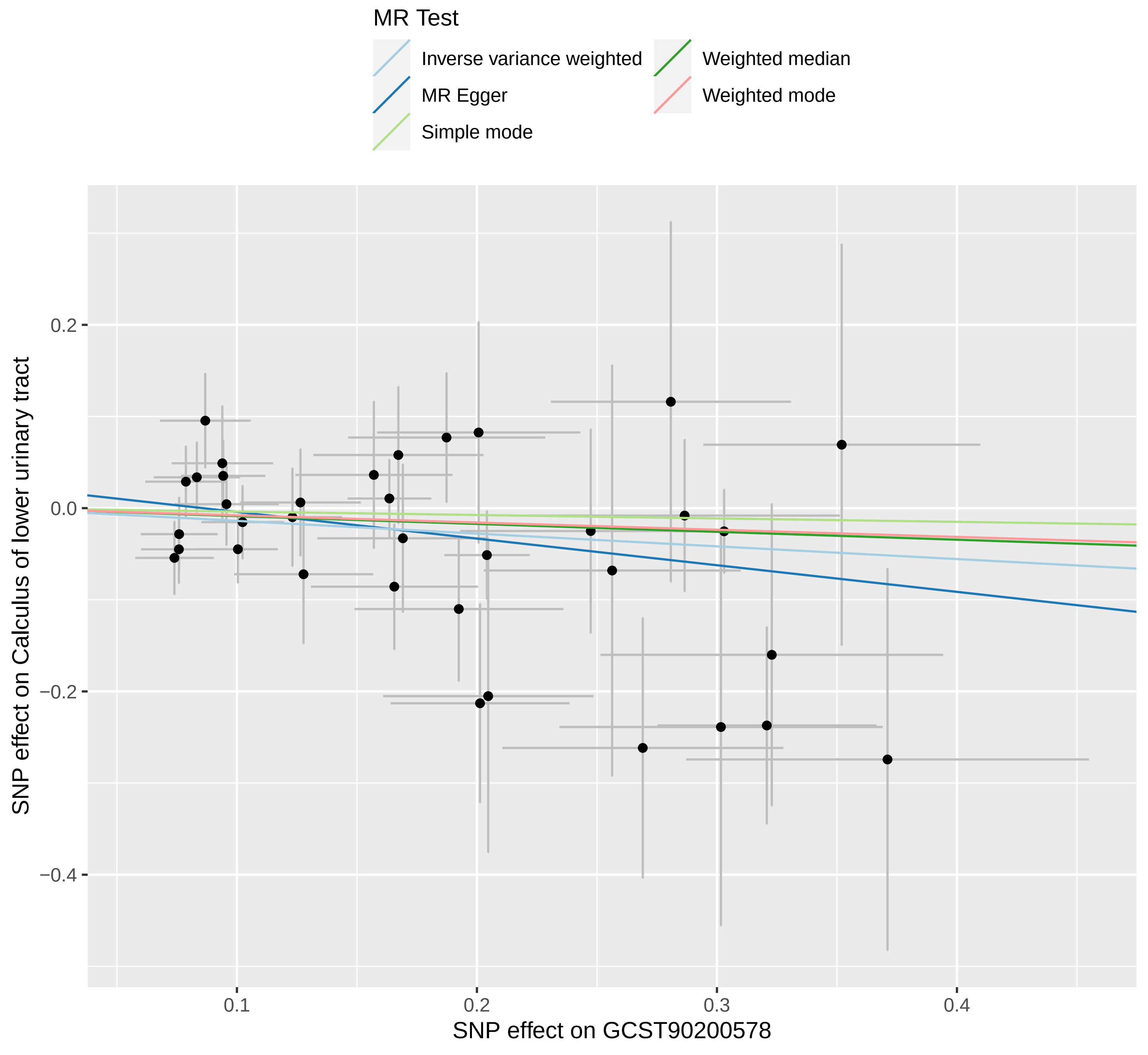

MR Test
Inverse variance weighted
MR Egger
Simple mode
Weighted median
Weighted mode
0.2
0.0
−0.2
−0.4
SNP effect on Calculus of lower urinary tract
0.1
0.2
0.3
0.4
SNP effect on GCST90200578

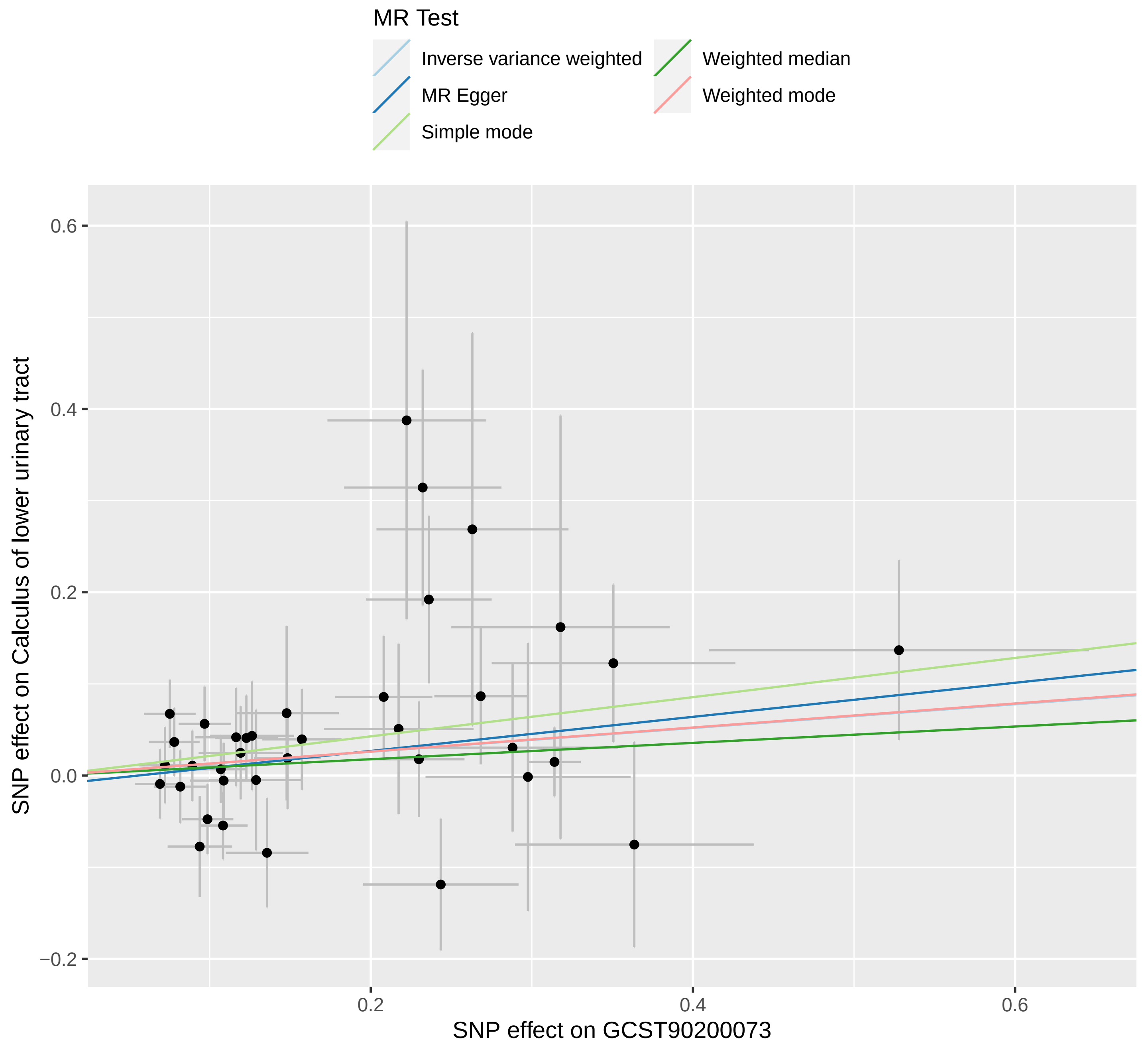
MR Test
Inverse variance weighted
MR Egger
Simple mode
Weighted median
Weighted mode
SNP effect on Calculus of lower urinary tract
0.6
0.4
0.2
0.0
−0.2
0.2
0.4
0.6
SNP effect on GCST90200073

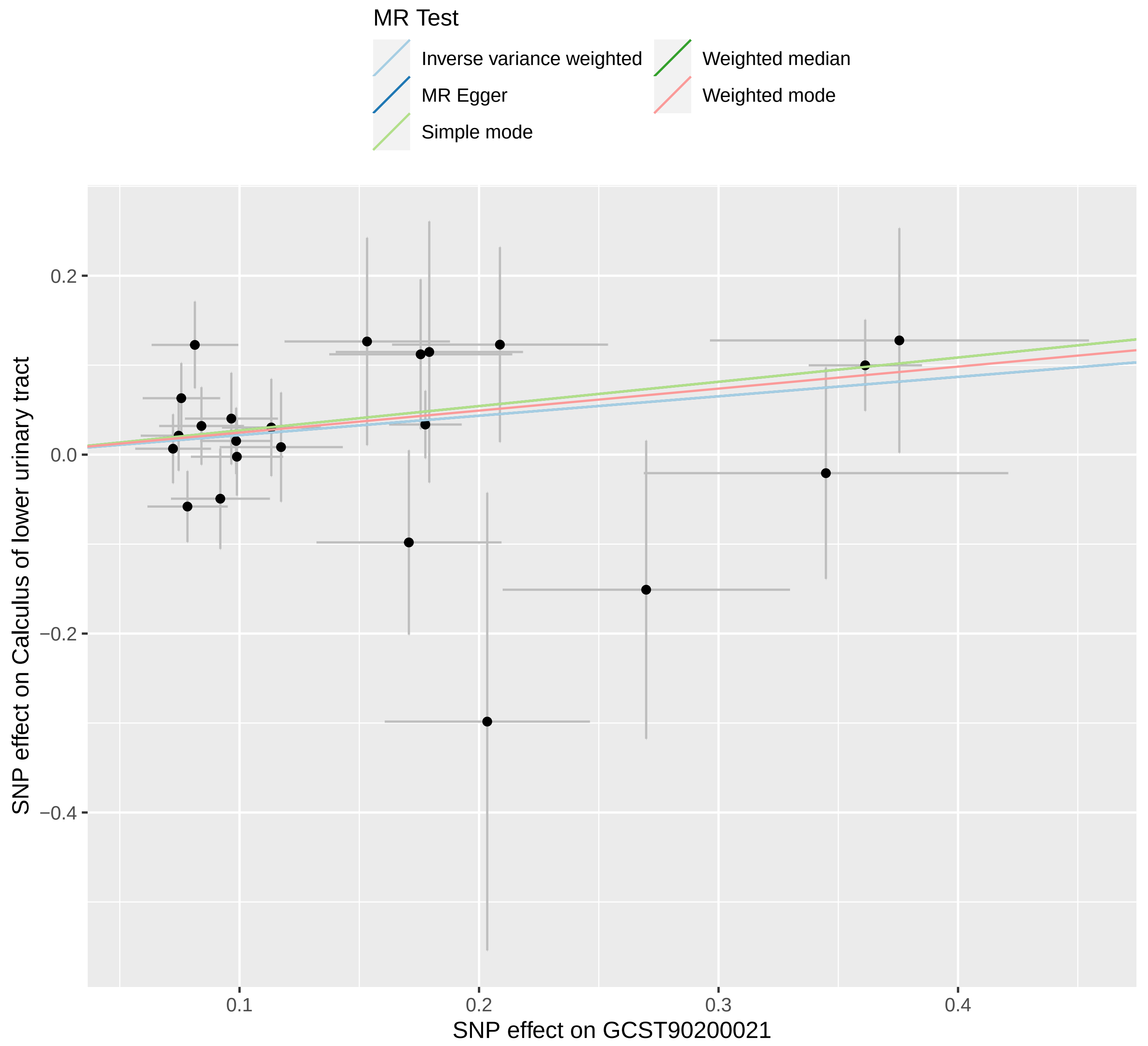
MR Test
Inverse variance weighted
MR Egger
Simple mode
Weighted median
Weighted mode
SNP effect on Calculus of lower urinary tract
SNP effect on GCST90200021
0.2
0.0
−0.2
−0.4
0.1
0.2
0.3
0.4

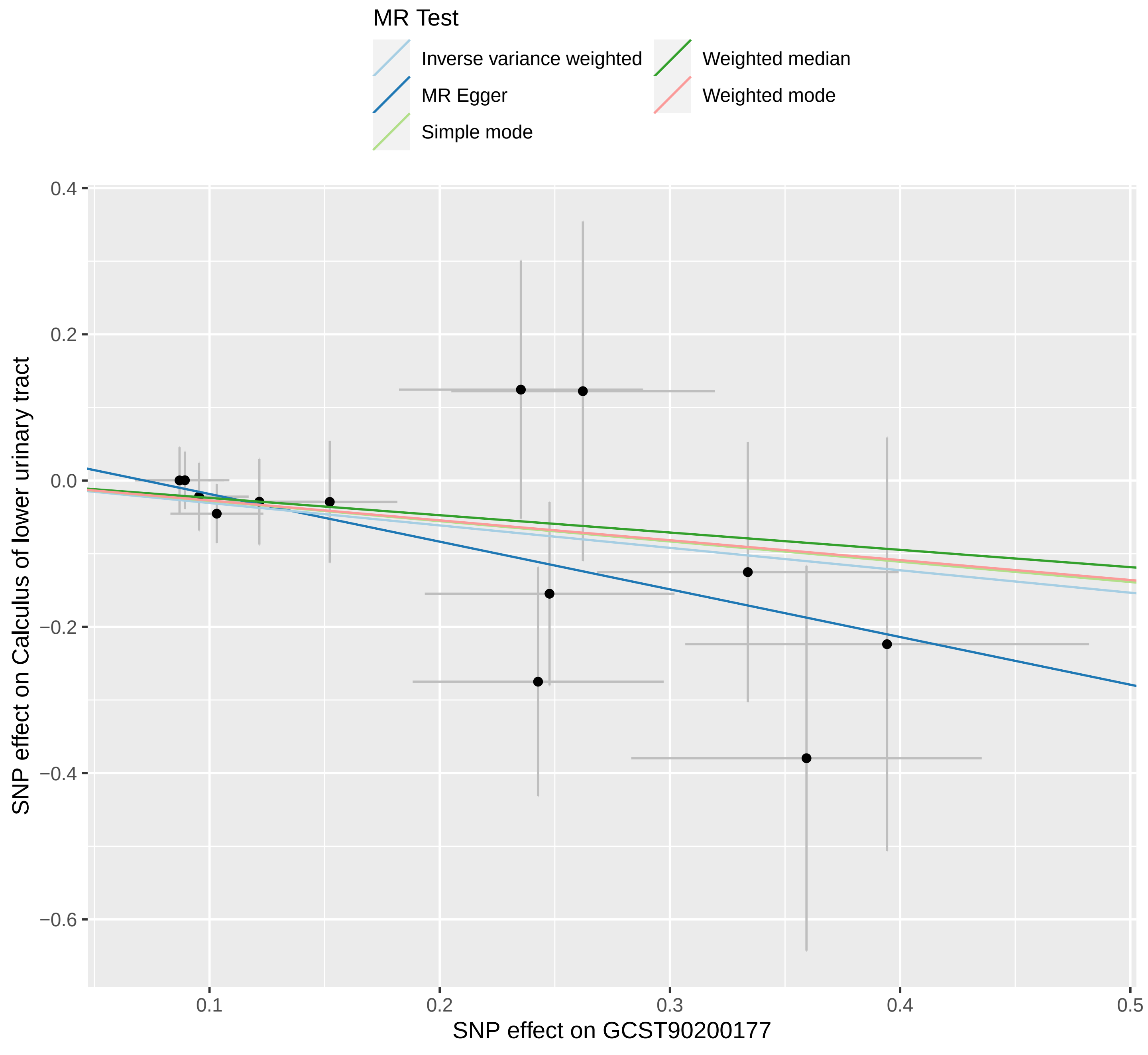

MR Test
Inverse variance weighted
MR Egger
Simple mode
Weighted median
Weighted mode
0.4
0.2
0.0
−0.2
−0.4
−0.6
SNP effect on Calculus of lower urinary tract
0.1
0.2
0.3
0.4
0.5
SNP effect on GCST90200177

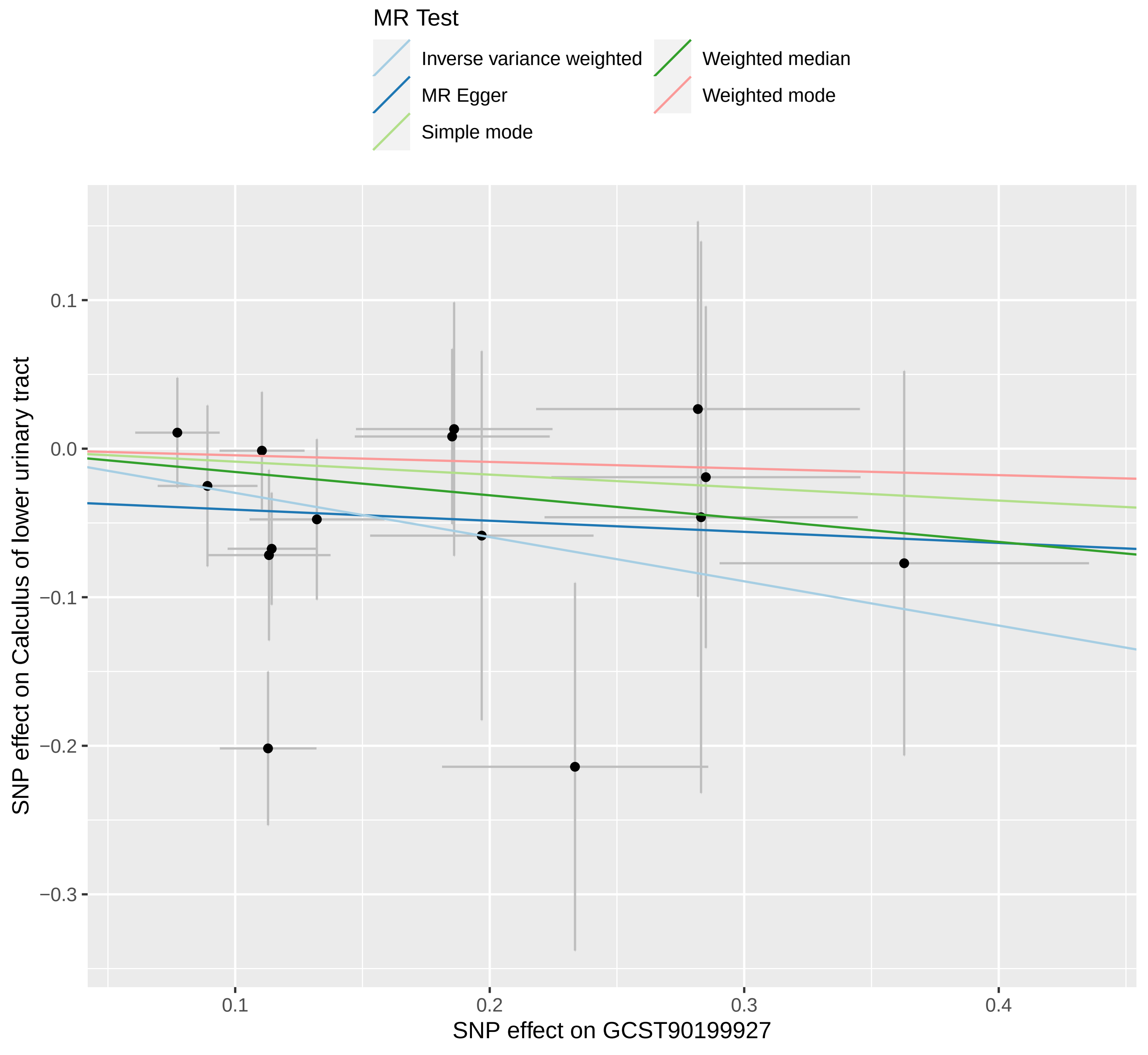

MR Test
Inverse variance weighted
MR Egger
Simple mode
Weighted median
Weighted mode
SNP effect on Calculus of lower urinary tract
0.1
0.0
−0.1
−0.2
−0.3
0.1
0.2
0.3
0.4
SNP effect on GCST90199927

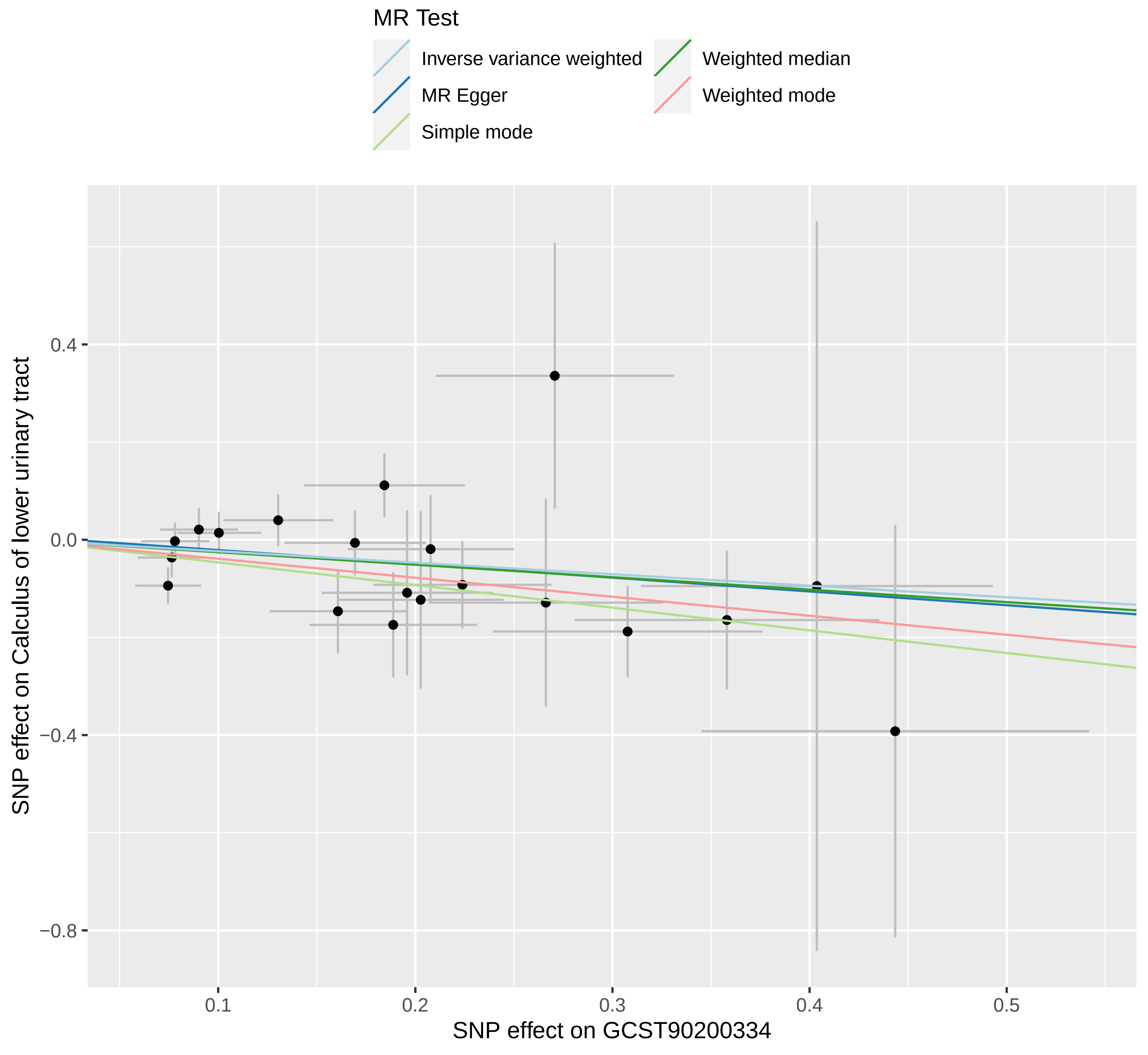
MR Test
Inverse variance weighted
MR Egger
Simple mode
Weighted median
Weighted mode
0.4
0.0
−0.4
−0.8
0.1
0.2
0.3
0.4
0.5
SNP effect on Calculus of lower urinary tract
SNP effect on GCST90200334

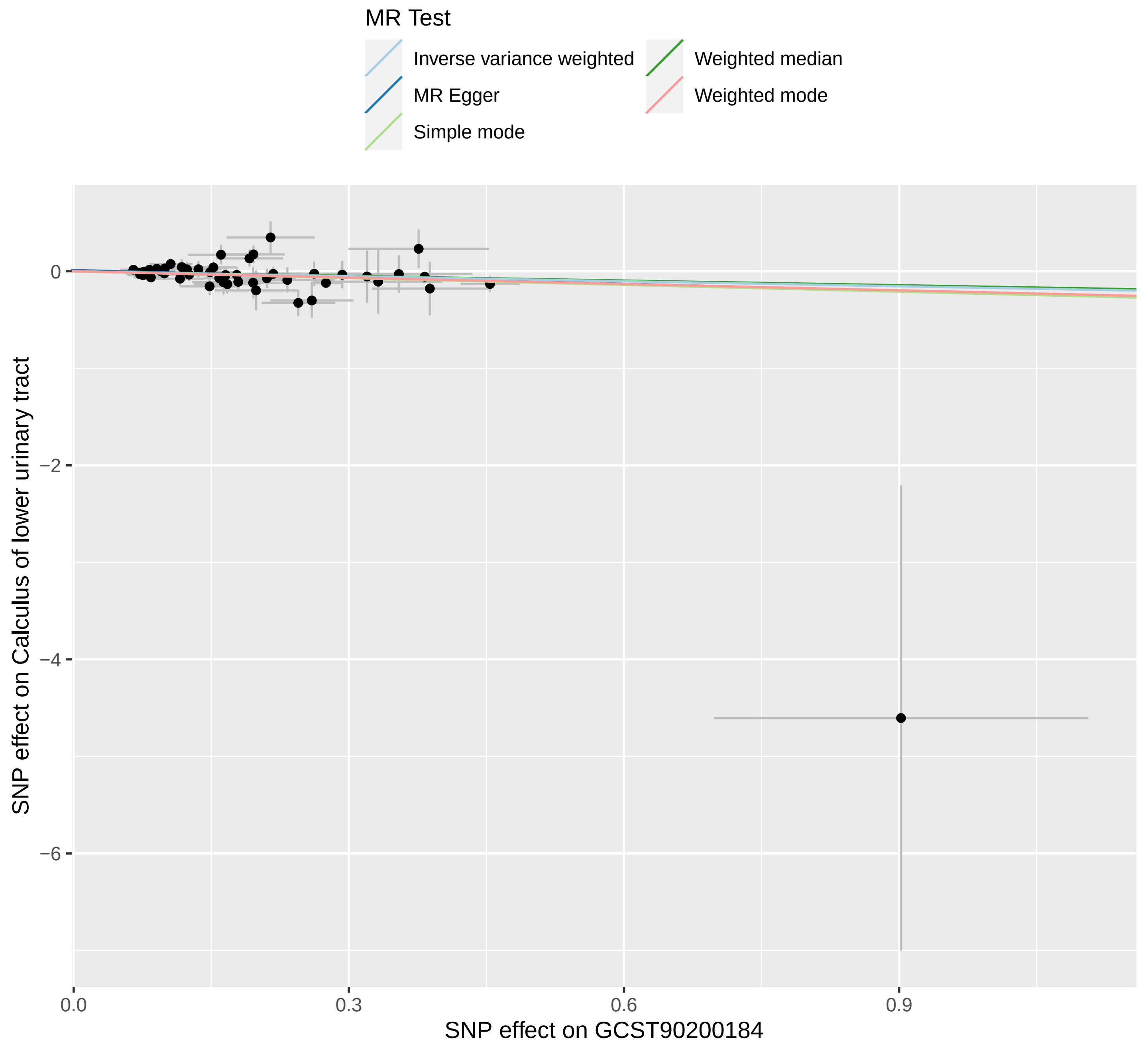

MR Test
Inverse variance weighted
MR Egger
Simple mode
Weighted median
Weighted mode
0
−2
−4
−6
0.0
0.3
0.6
0.9
SNP effect on Calculus of lower urinary tract
SNP effect on GCST90200184

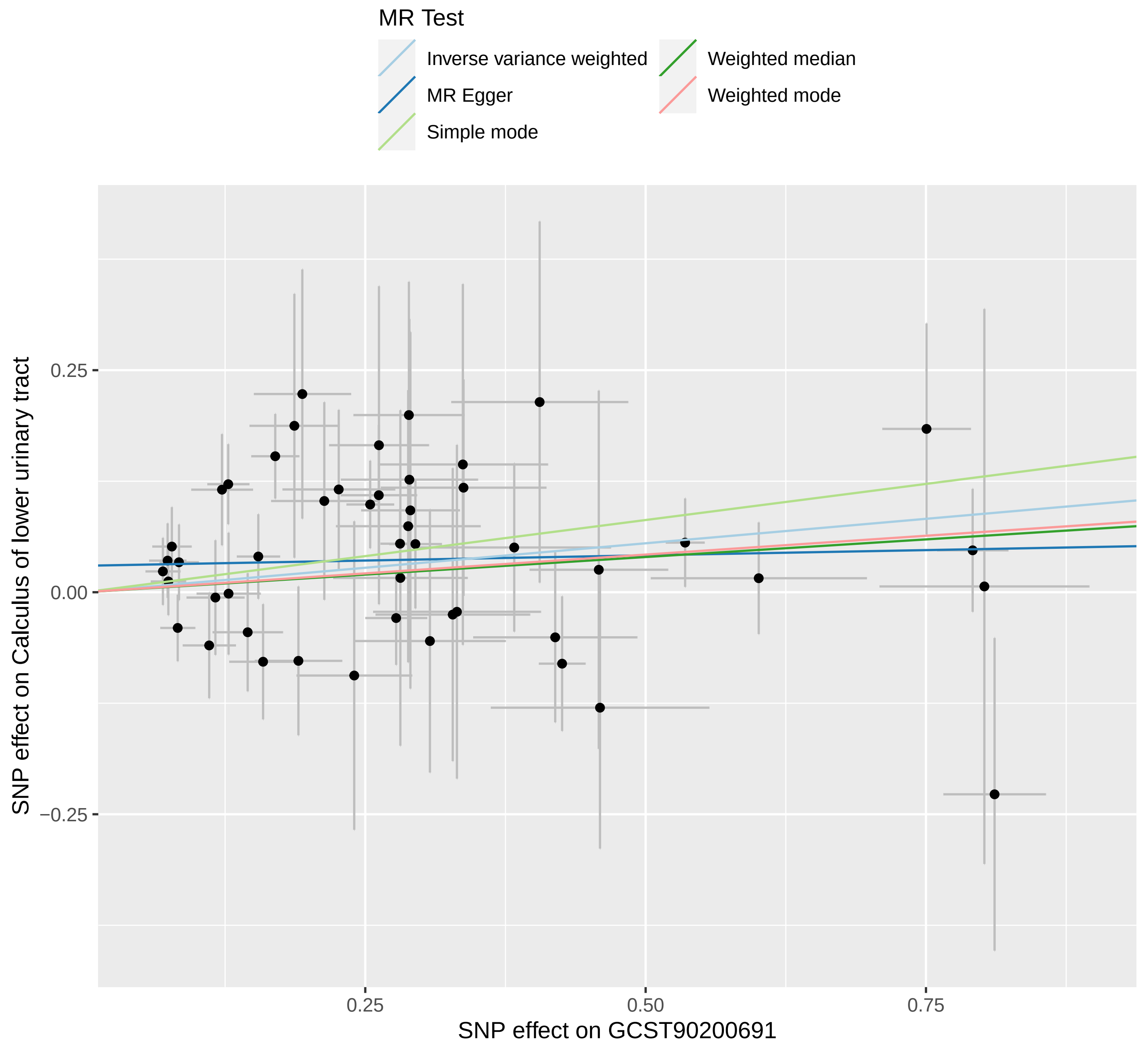
MR Test
Inverse variance weighted
MR Egger
Simple mode
Weighted median
Weighted mode
0.25
0.00
−0.25
SNP effect on Calculus of lower urinary tract
0.25
0.50
0.75
SNP effect on GCST90200691

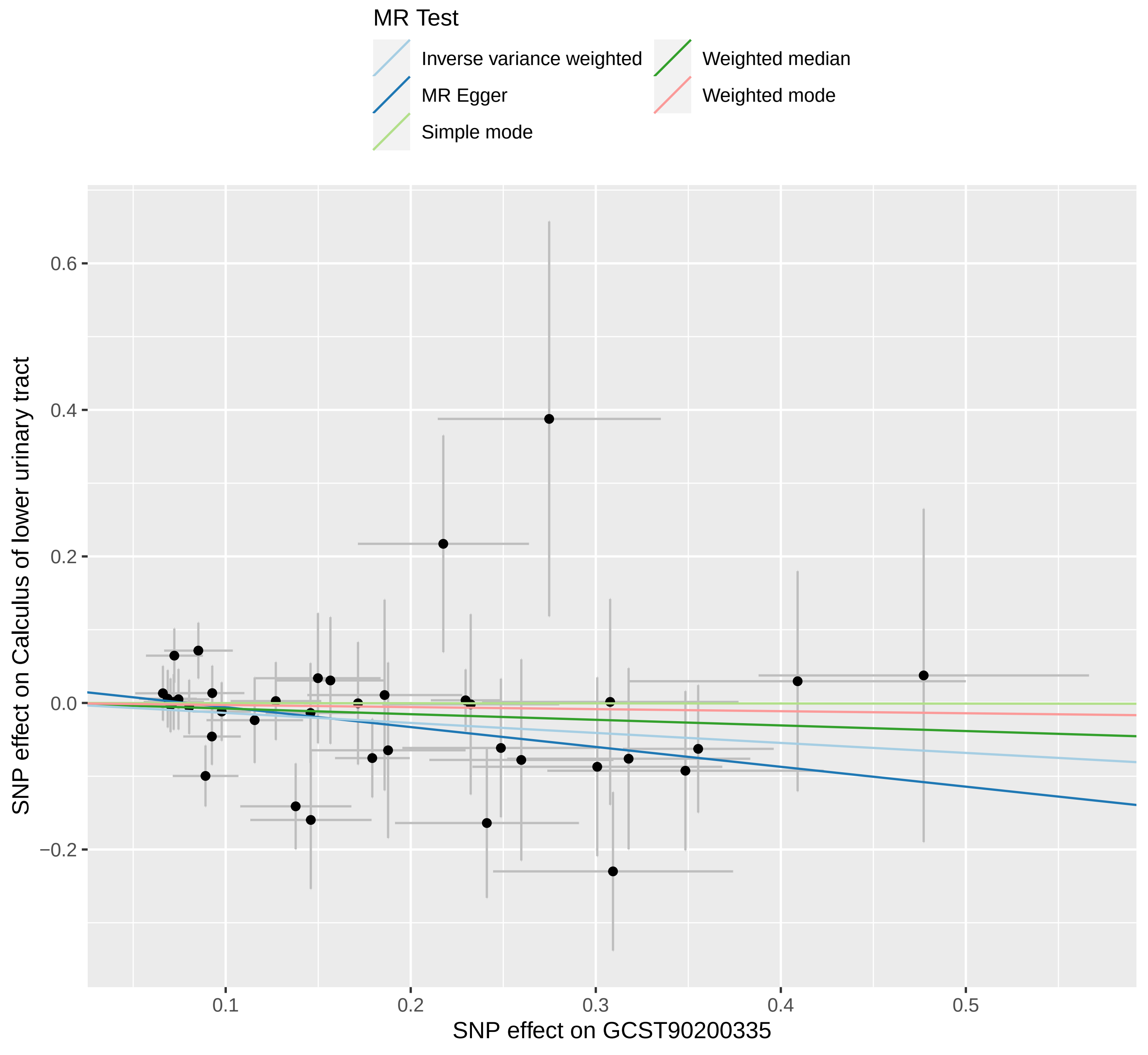
MR Test
Inverse variance weighted
MR Egger
Simple mode
Weighted median
Weighted mode
SNP effect on Calculus of lower urinary tract
SNP effect on GCST90200335
0.6
0.4
0.2
0.0
−0.2
0.1
0.2
0.3
0.4
0.5

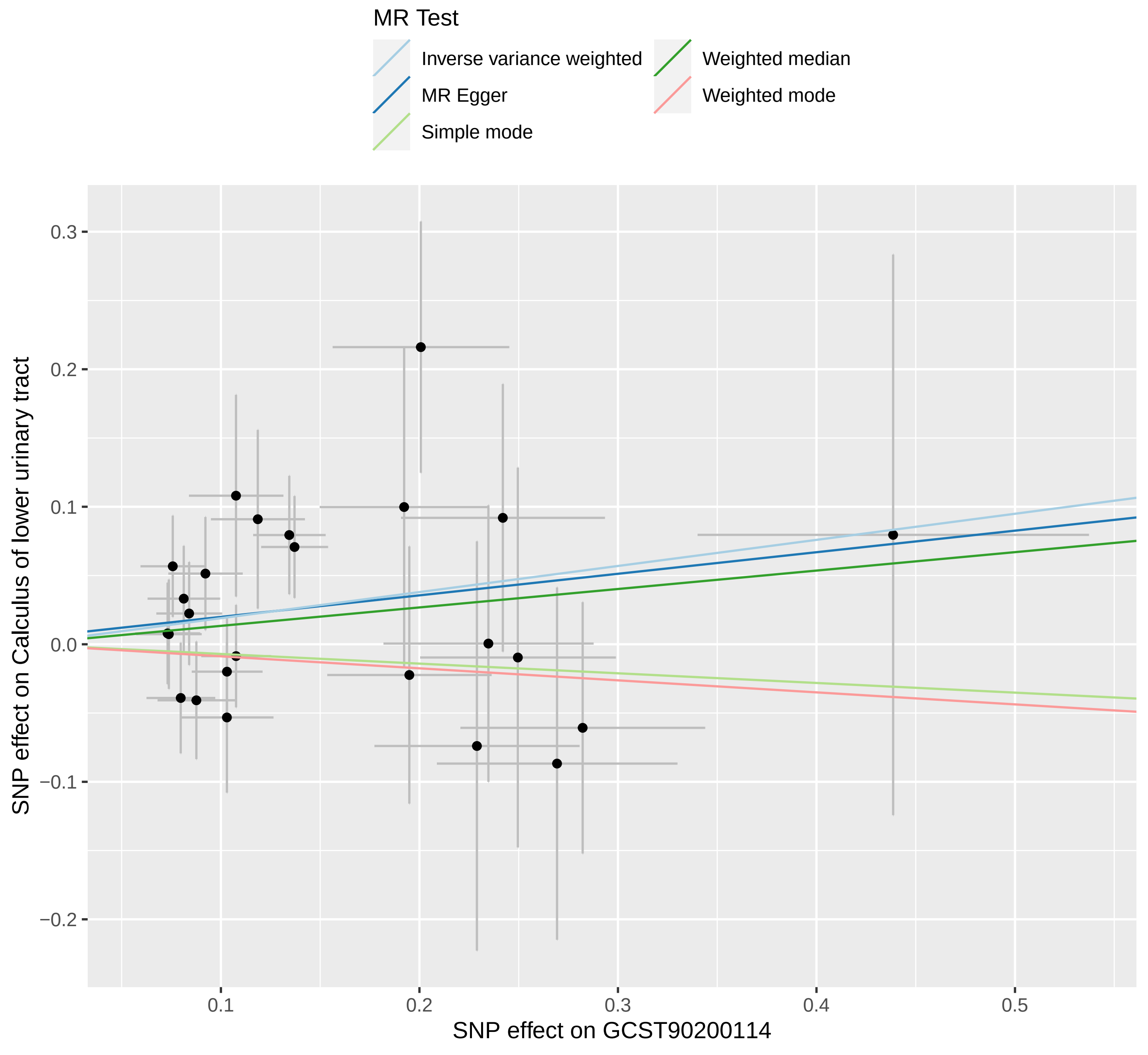
MR Test
Inverse variance weighted
MR Egger
Simple mode
Weighted median
Weighted mode
0.3
0.2
0.1
0.0
−0.1
−0.2
0.1
0.2
0.3
0.4
0.5
SNP effect on GCST90200114
SNP effect on Calculus of lower urinary tract

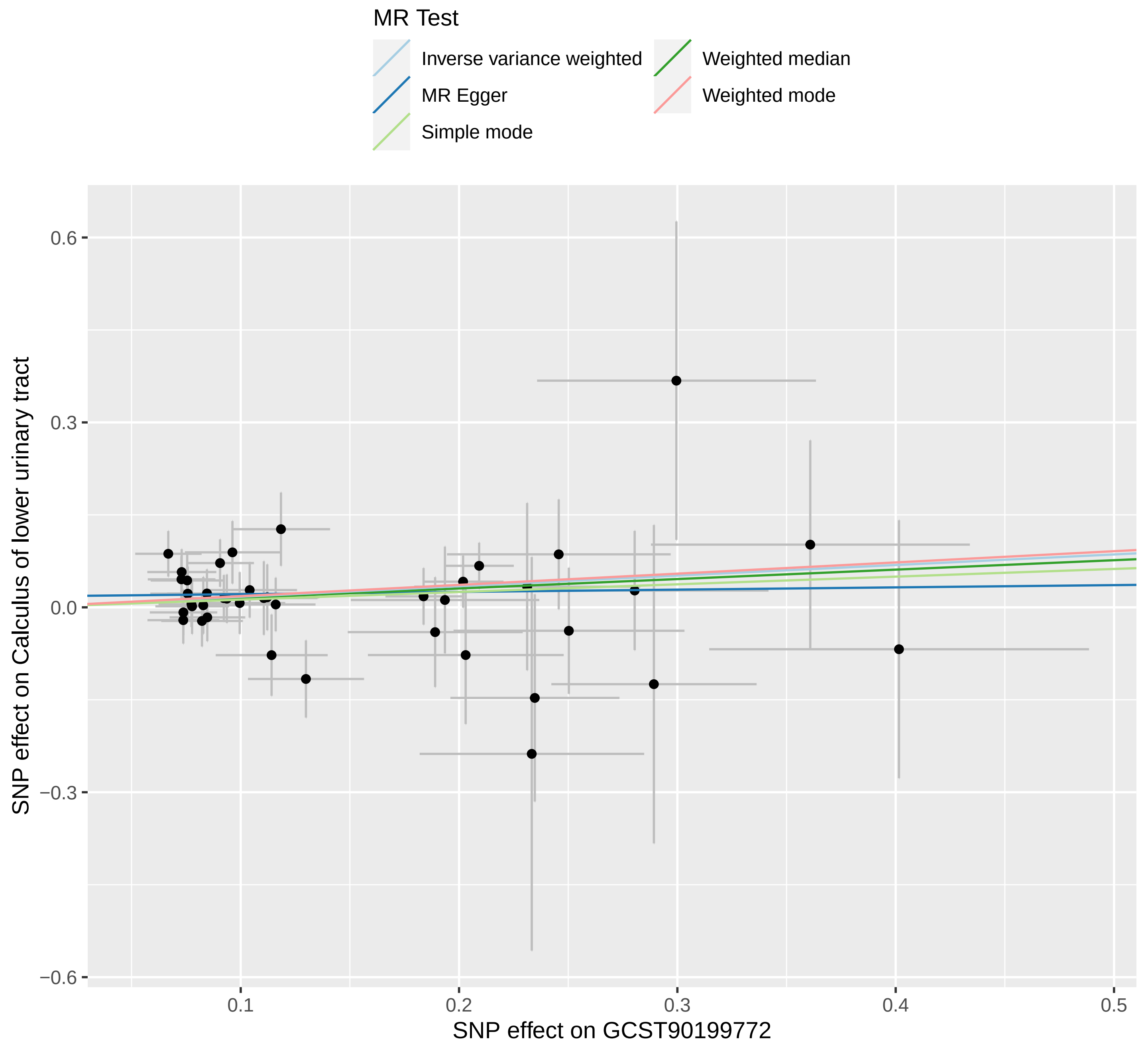

MR Test
Inverse variance weighted
MR Egger
Simple mode
Weighted median
Weighted mode
0.6
0.3
0.0
−0.3
−0.6
SNP effect on Calculus of lower urinary tract
0.1
0.2
0.3
0.4
0.5
SNP effect on GCST90199772

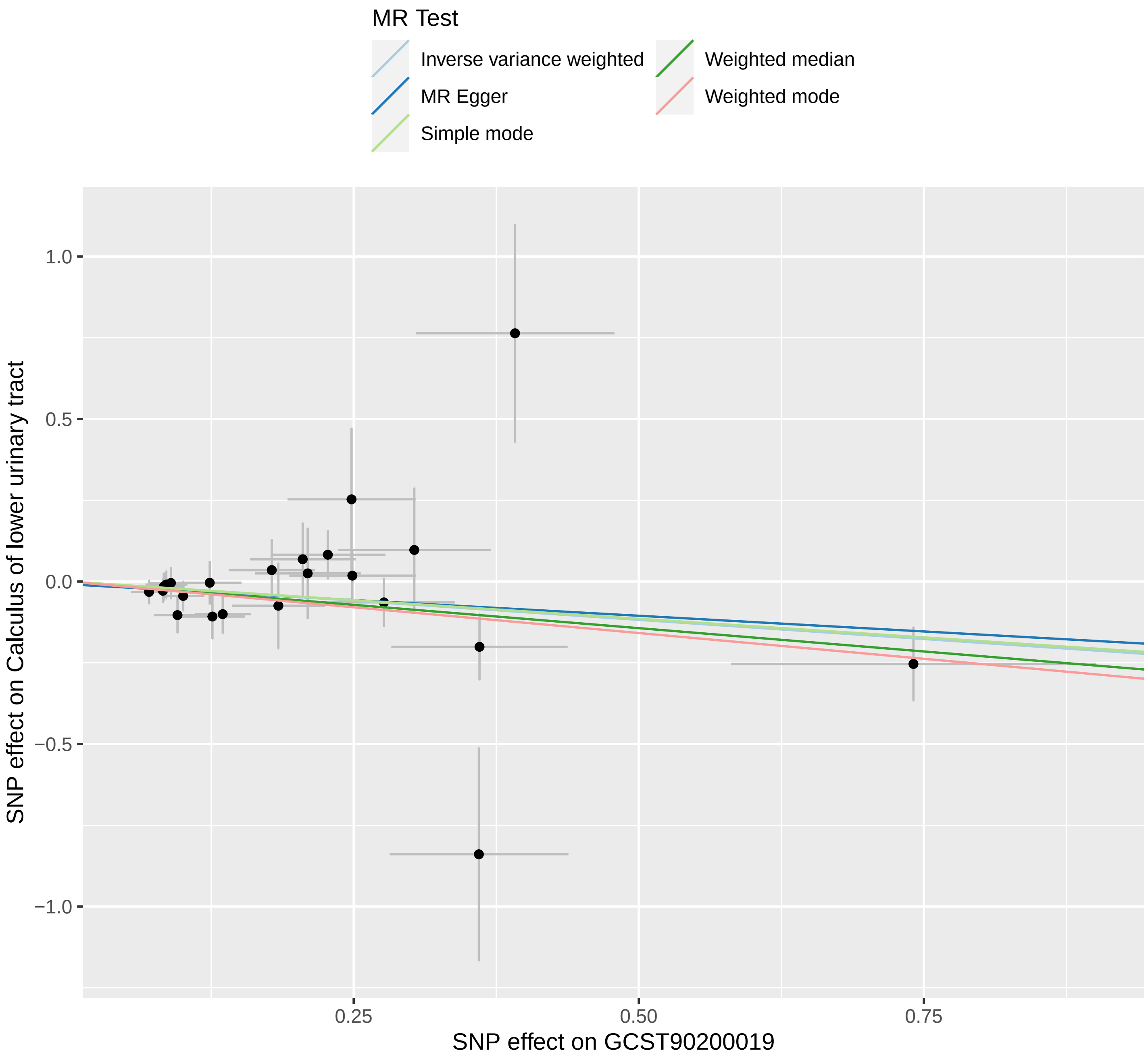
MR Test
Inverse variance weighted
MR Egger
Simple mode
Weighted median
Weighted mode
1.0
0.5
0.0
−0.5
−1.0
SNP effect on Calculus of lower urinary tract
0.25
0.50
0.75
SNP effect on GCST90200019

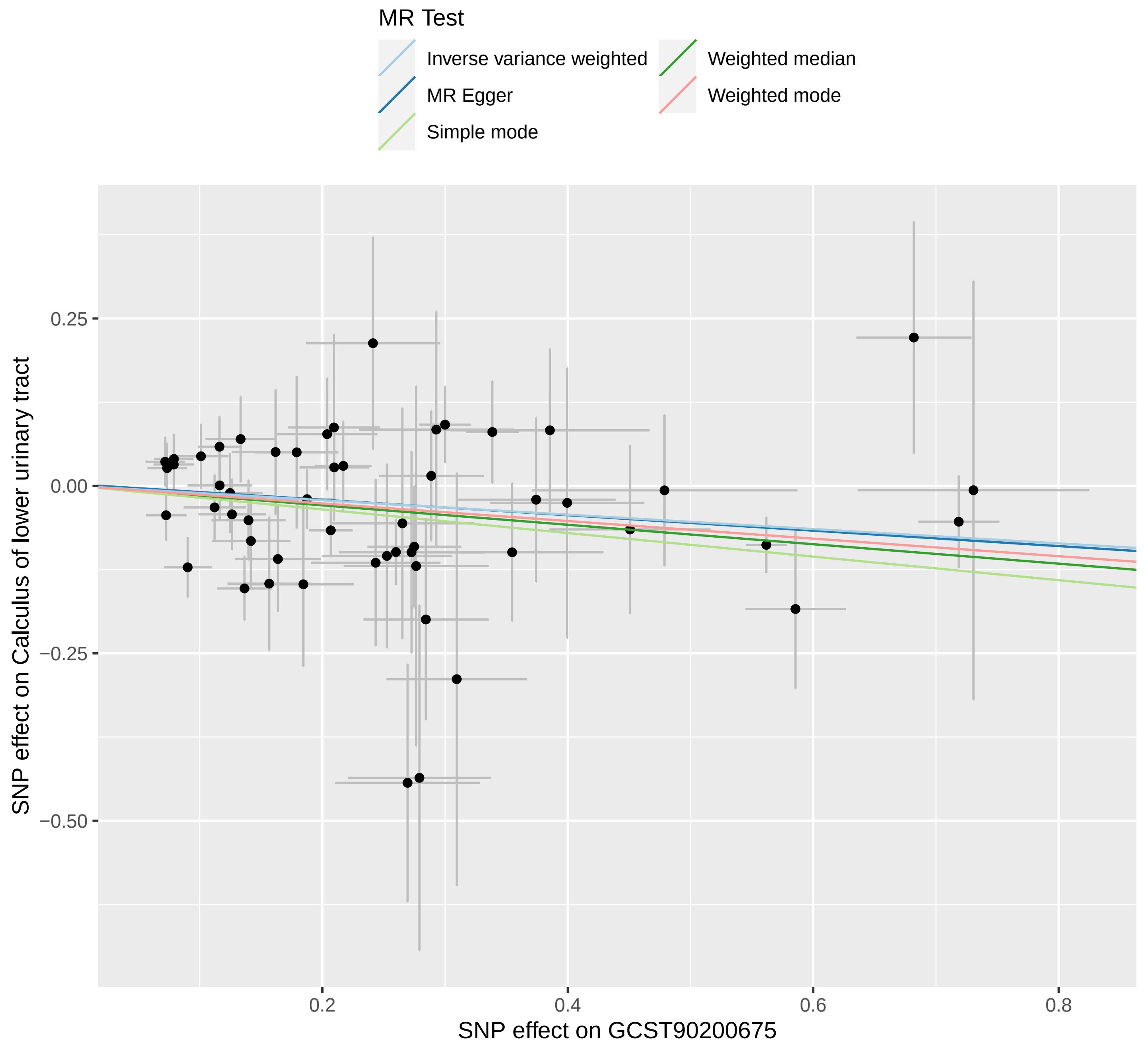

MR Test
Inverse variance weighted
MR Egger
Simple mode
Weighted median
Weighted mode
0.25
0.00
−0.25
−0.50
0.2
0.4
0.6
0.8
SNP effect on Calculus of lower urinary tract
SNP effect on GCST90200675

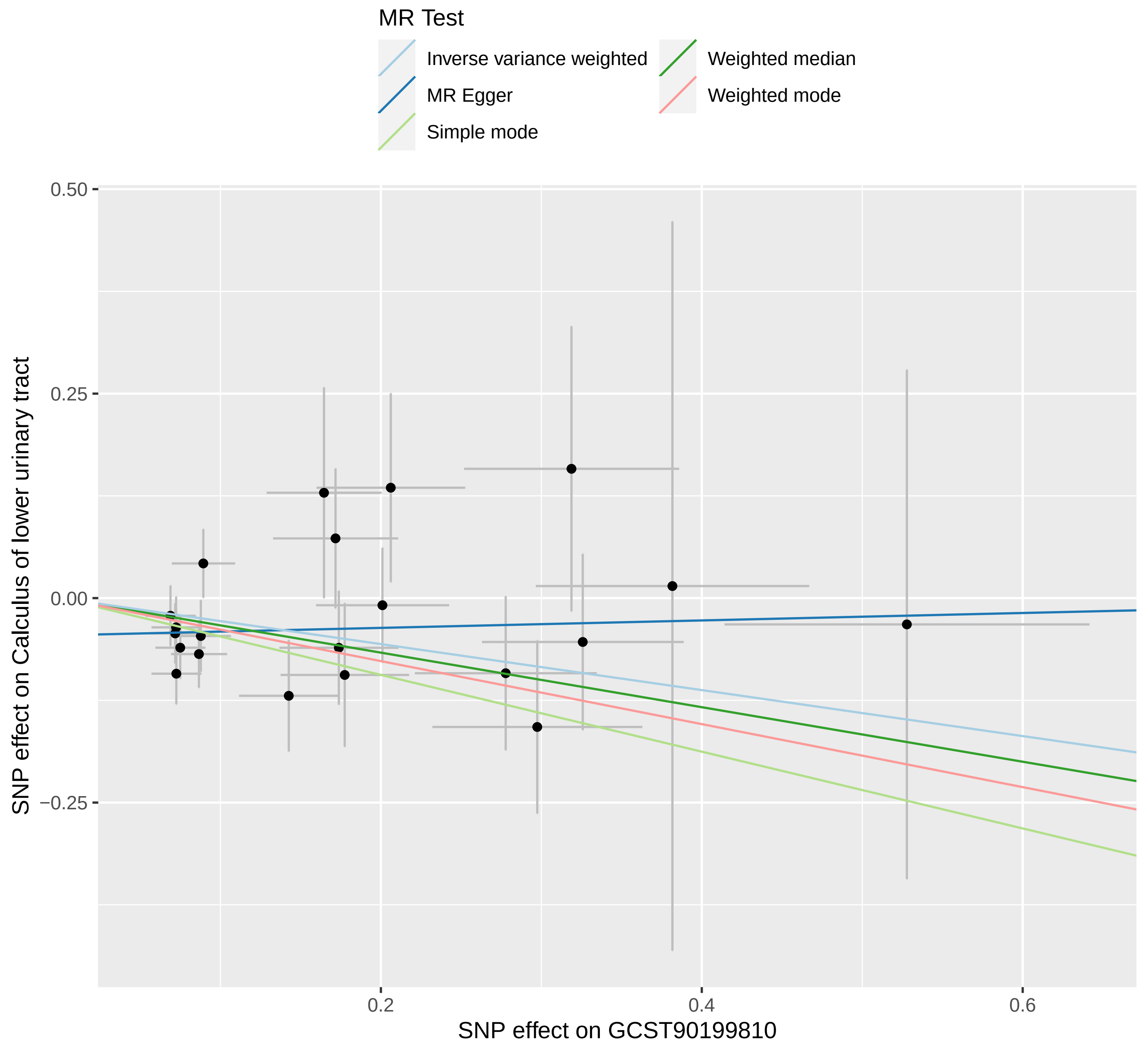
MR Test
Inverse variance weighted
MR Egger
Simple mode
Weighted median
Weighted mode
0.50
0.25
0.00
−0.25
0.2
0.4
0.6
SNP effect on Calculus of lower urinary tract
SNP effect on GCST90199810

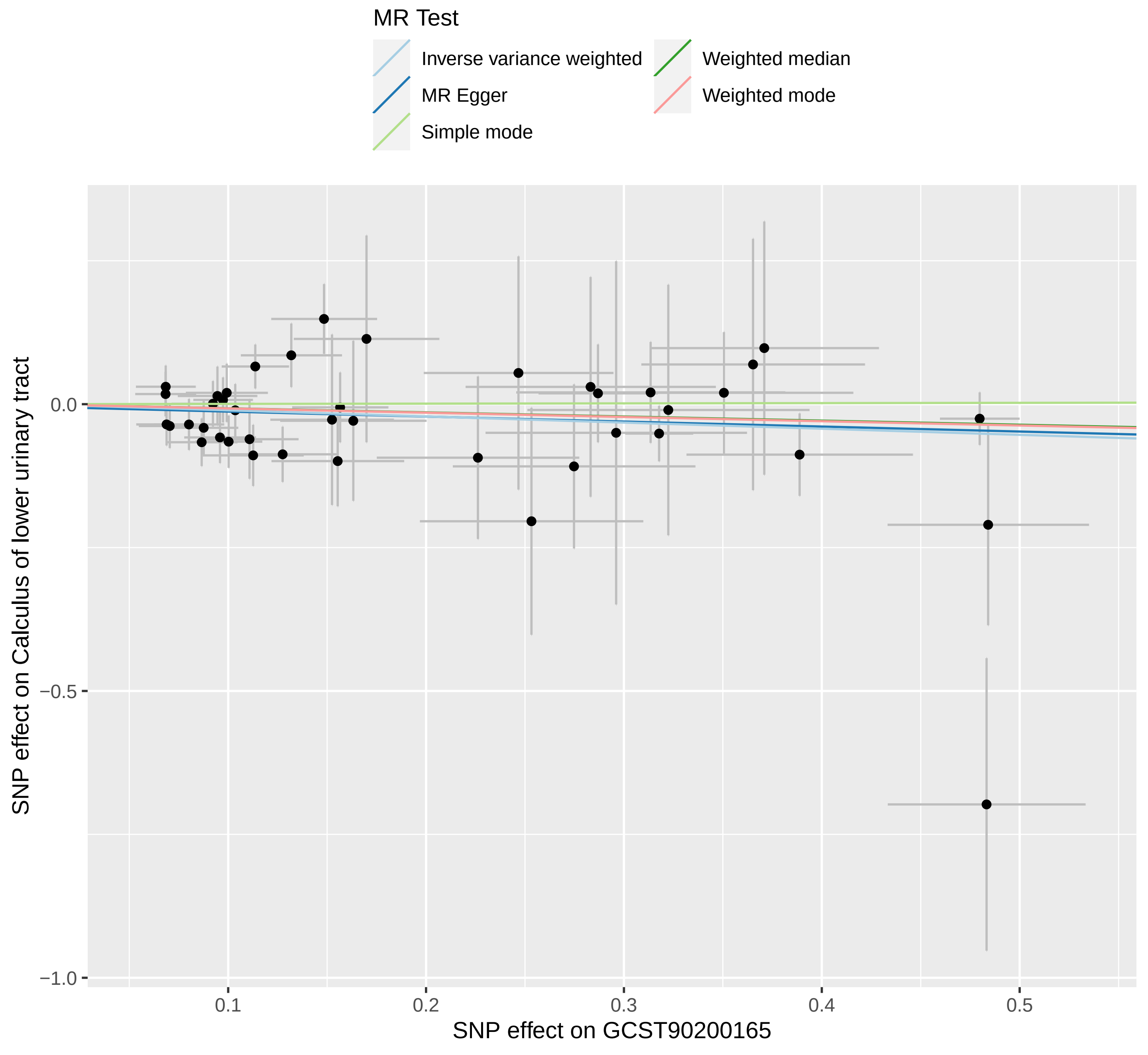

MR Test
Inverse variance weighted
MR Egger
Simple mode
Weighted median
Weighted mode
SNP effect on Calculus of lower urinary tract
0.0
−0.5
−1.0
0.1
0.2
0.3
0.4
0.5
SNP effect on GCST90200165

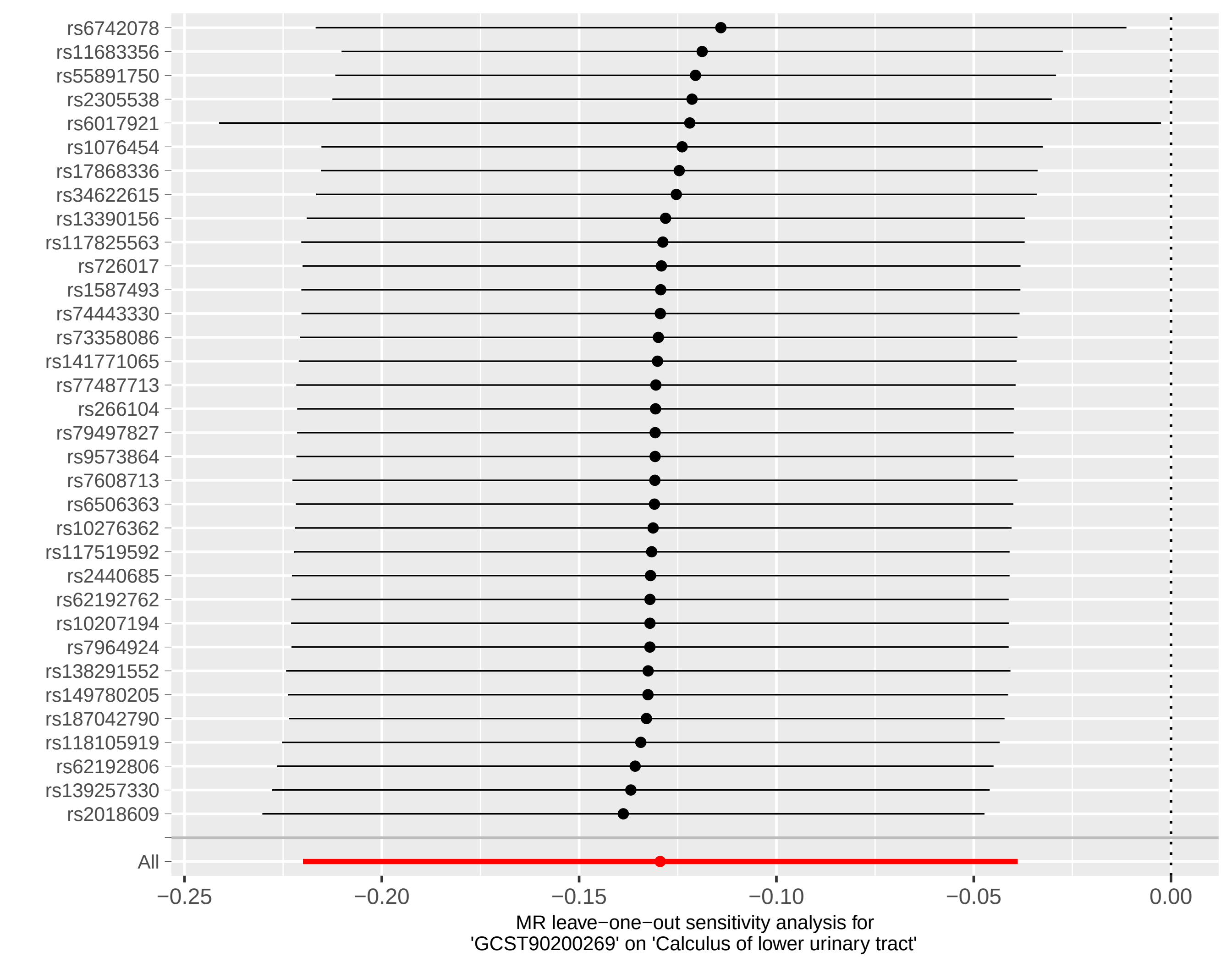
rs6742078
rs11683356
rs55891750
rs2305538
rs6017921
rs1076454
rs17868336
rs34622615
rs13390156
rs117825563
rs726017
rs1587493
rs74443330
rs73358086
rs141771065
rs77487713
rs266104
rs79497827
rs9573864
rs7608713
rs6506363
rs10276362
rs117519592
rs2440685
rs62192762
rs10207194
rs7964924
rs138291552
rs149780205
rs187042790
rs118105919
rs62192806
rs139257330
rs2018609
All
−0.25
−0.20
−0.15
−0.10
−0.05
0.00
MR leave−one−out sensitivity analysis for
'GCST90200269' on 'Calculus of lower urinary tract'

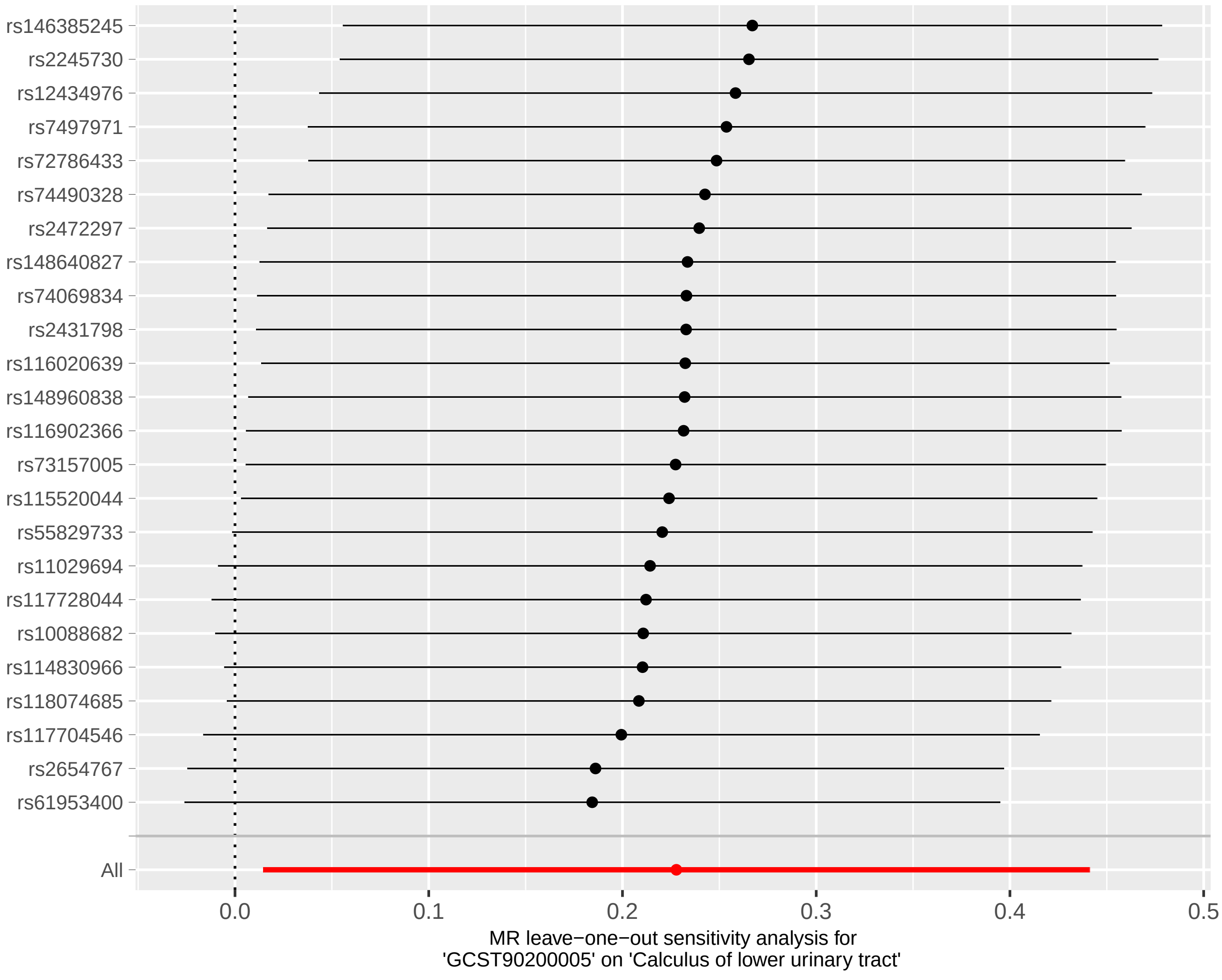
rs146385245
rs2245730
rs12434976
rs7497971
rs72786433
rs74490328
rs2472297
rs148640827
rs74069834
rs2431798
rs116020639
rs148960838
rs116902366
rs73157005
rs115520044
rs55829733
rs11029694
rs117728044
rs10088682
rs114830966
rs118074685
rs117704546
rs2654767
rs61953400
All
0.0
0.1
0.2
0.3
0.4
0.5
MR leave−one−out sensitivity analysis for
'GCST90200005' on 'Calculus of lower urinary tract'

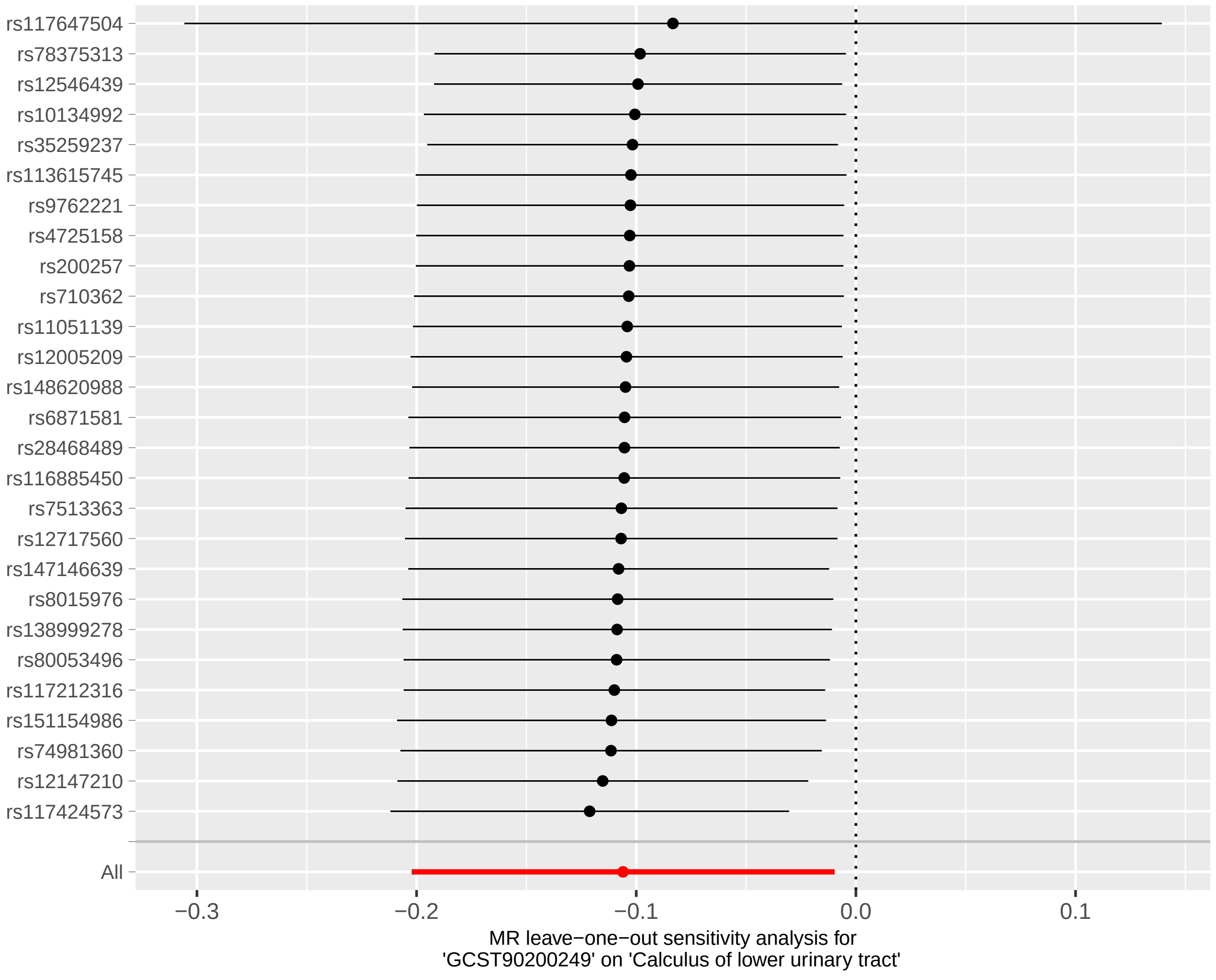
rs117647504
rs78375313
rs12546439
rs10134992
rs35259237
rs113615745
rs9762221
rs4725158
rs200257
rs710362
rs11051139
rs12005209
rs148620988
rs6871581
rs28468489
rs116885450
rs7513363
rs12717560
rs147146639
rs8015976
rs138999278
rs80053496
rs117212316
rs151154986
rs74981360
rs12147210
rs117424573
All
−0.3
−0.2
−0.1
0.0
0.1
MR leave−one−out sensitivity analysis for
'GCST90200249' on 'Calculus of lower urinary tract'

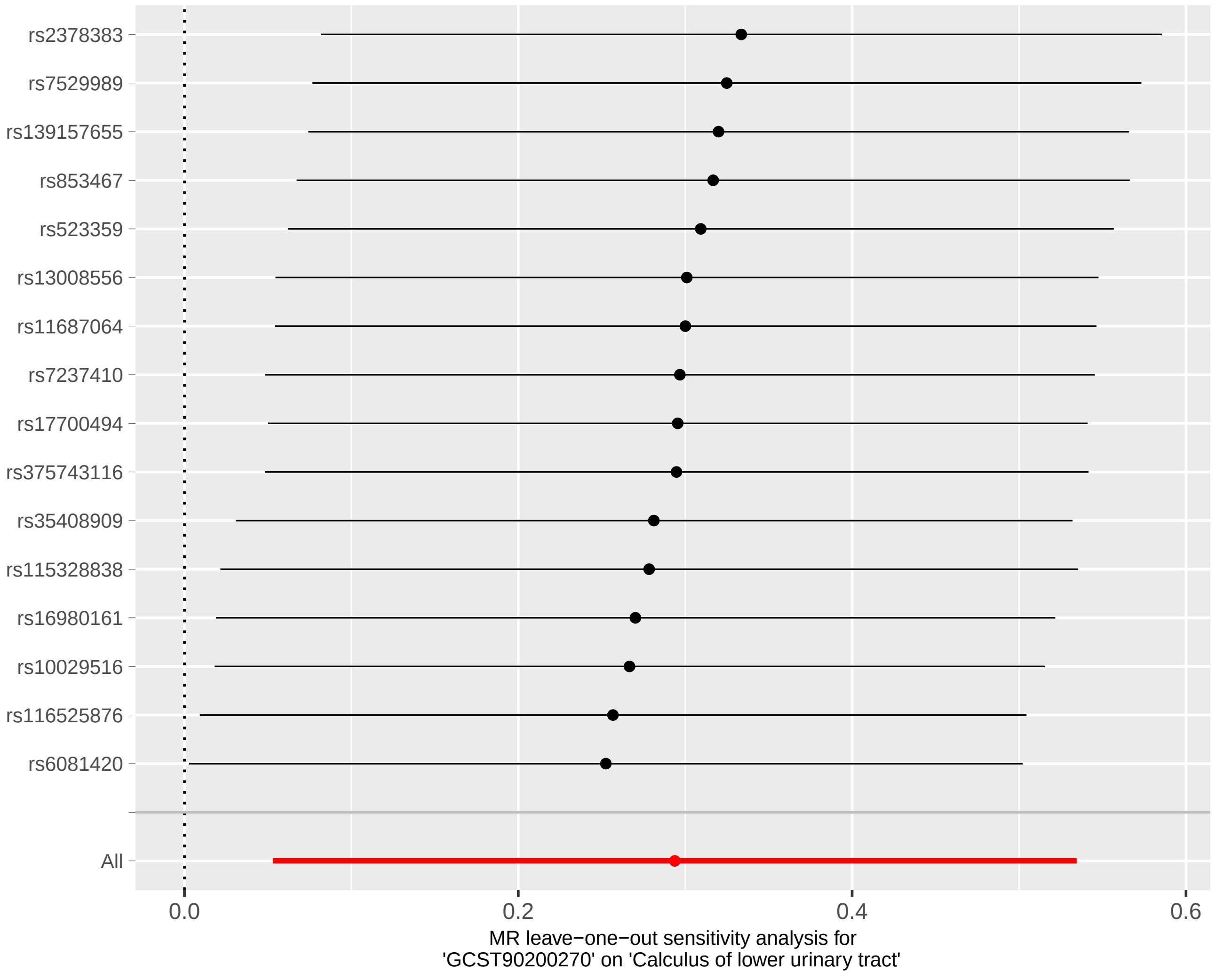
rs2378383
rs7529989
rs139157655
rs853467
rs523359
rs13008556
rs11687064
rs7237410
rs17700494
rs375743116
rs35408909
rs115328838
rs16980161
rs10029516
rs116525876
rs6081420
All
0.0
0.2
0.4
0.6
MR leave−one−out sensitivity analysis for
'GCST90200270' on 'Calculus of lower urinary tract'

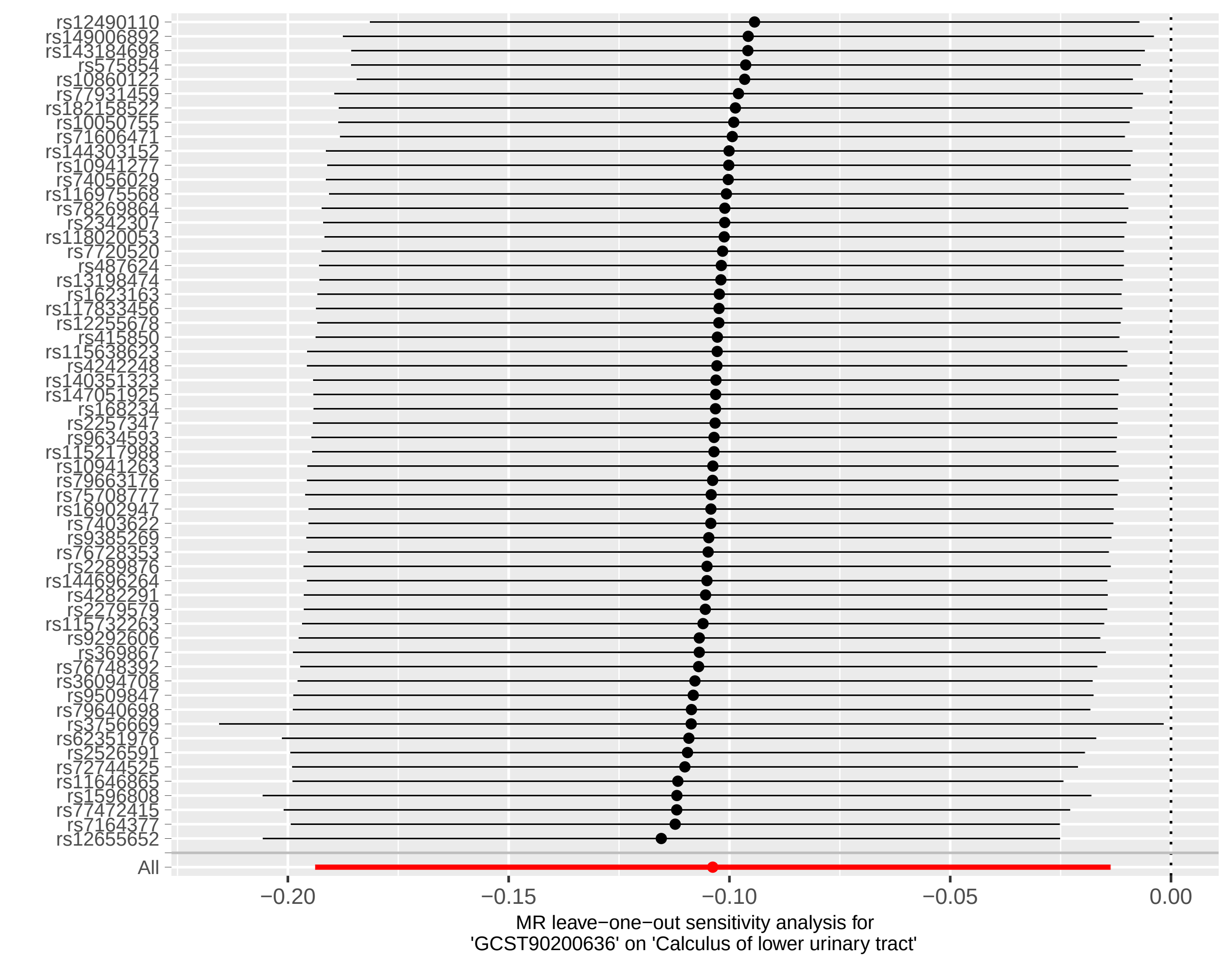

rs12490110
rs149006892
rs143184698
rs575854
rs10860122
rs77931459
rs182158522
rs10050755
rs71606471
rs144303152
rs10941277
rs74056029
rs116975568
rs78269864
rs2342307
rs118020053
rs7720520
rs487624
rs13198474
rs1623163
rs117833456
rs12255678
rs415850
rs115638623
rs4242248
rs140351323
rs147051925
rs168234
rs2257347
rs9634593
rs115217988
rs10941263
rs79663176
rs75708777
rs16902947
rs7403622
rs9385269
rs76728353
rs2289876
rs144696264
rs4282291
rs2279579
rs115732263
rs9292606
rs369867
rs76748392
rs36094708
rs9509847
rs79640698
rs3756669
rs62351976
rs2526591
rs72744525
rs11646865
rs1596808
rs77472415
rs7164377
rs12655652
All
−0.20
−0.15
−0.10
−0.05
0.00
MR leave−one−out sensitivity analysis for
'GCST90200636' on 'Calculus of lower urinary tract'

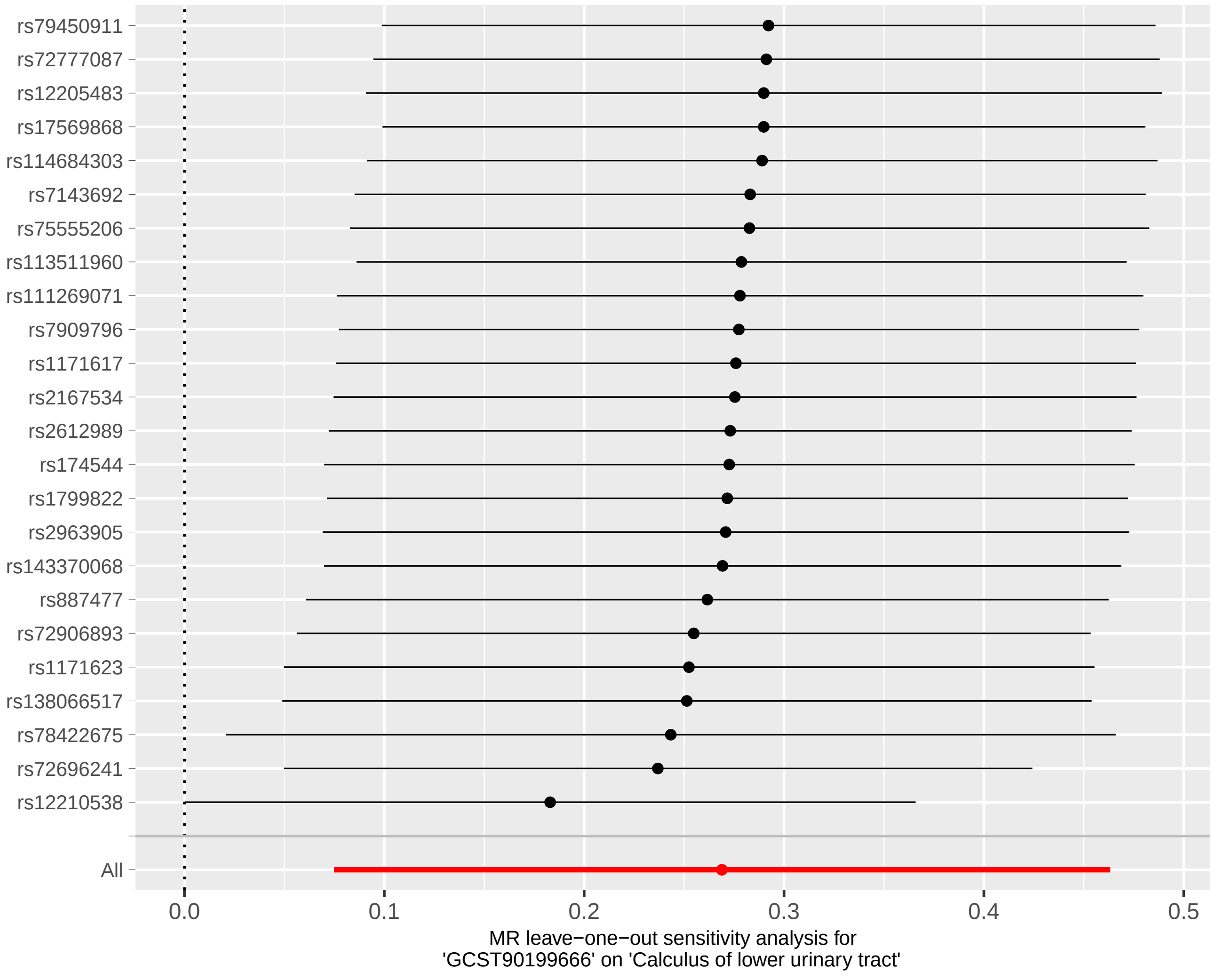
rs79450911
rs72777087
rs12205483
rs17569868
rs114684303
rs7143692
rs75555206
rs113511960
rs111269071
rs7909796
rs1171617
rs2167534
rs2612989
rs174544
rs1799822
rs2963905
rs143370068
rs887477
rs72906893
rs1171623
rs138066517
rs78422675
rs72696241
rs12210538
All
0.0
0.1
0.2
0.3
0.4
0.5
MR leave−one−out sensitivity analysis for
'GCST90199666' on 'Calculus of lower urinary tract'

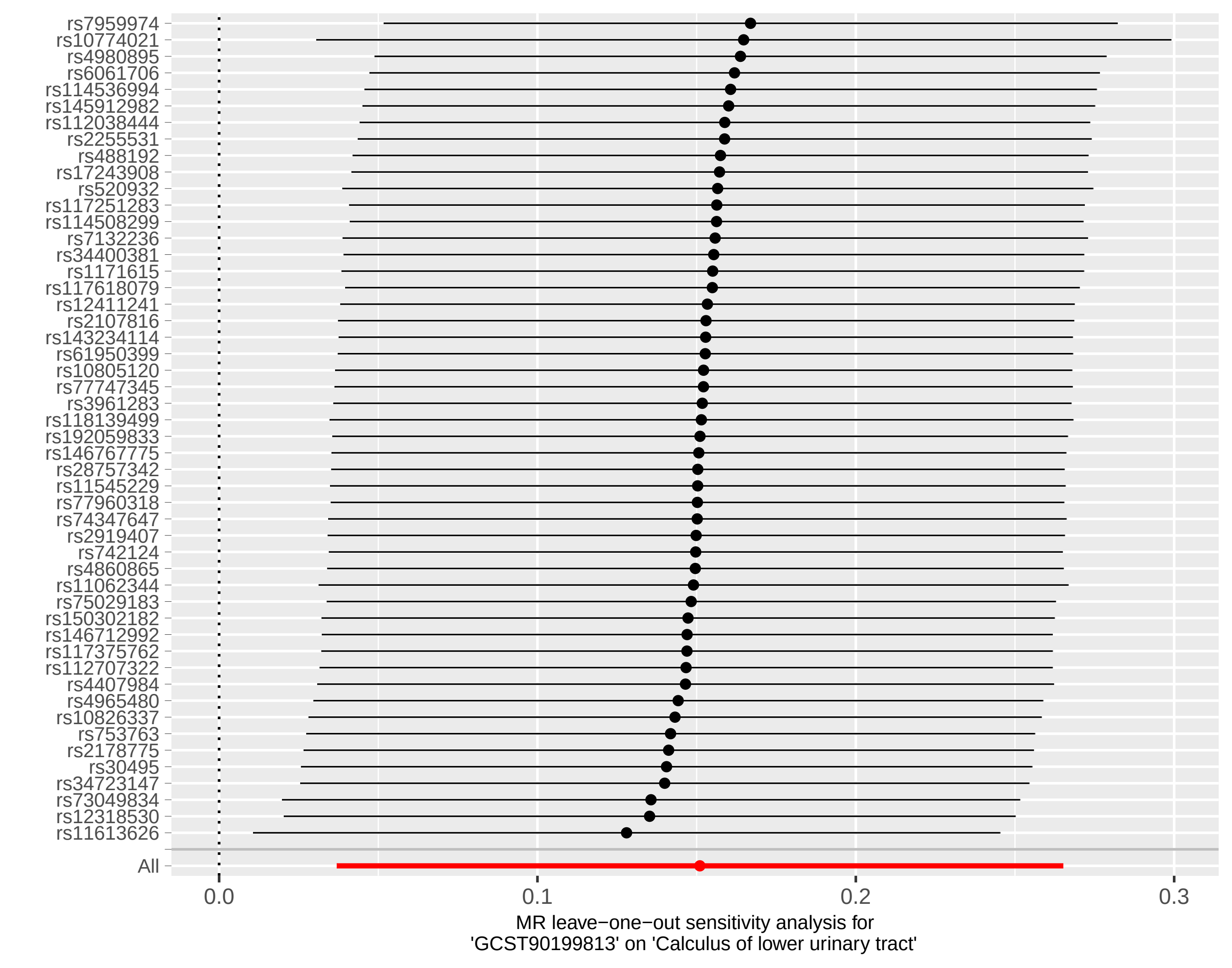
rs7959974
rs10774021
rs4980895
rs6061706
rs114536994
rs145912982
rs112038444
rs2255531
rs488192
rs17243908
rs520932
rs117251283
rs114508299
rs7132236
rs34400381
rs1171615
rs117618079
rs12411241
rs2107816
rs143234114
rs61950399
rs10805120
rs77747345
rs3961283
rs118139499
rs192059833
rs146767775
rs28757342
rs11545229
rs77960318
rs74347647
rs2919407
rs742124
rs4860865
rs11062344
rs75029183
rs150302182
rs146712992
rs117375762
rs112707322
rs4407984
rs4965480
rs10826337
rs753763
rs2178775
rs30495
rs34723147
rs73049834
rs12318530
rs11613626
All
0.0
0.1
0.2
0.3
MR leave−one−out sensitivity analysis for
'GCST90199813' on 'Calculus of lower urinary tract'

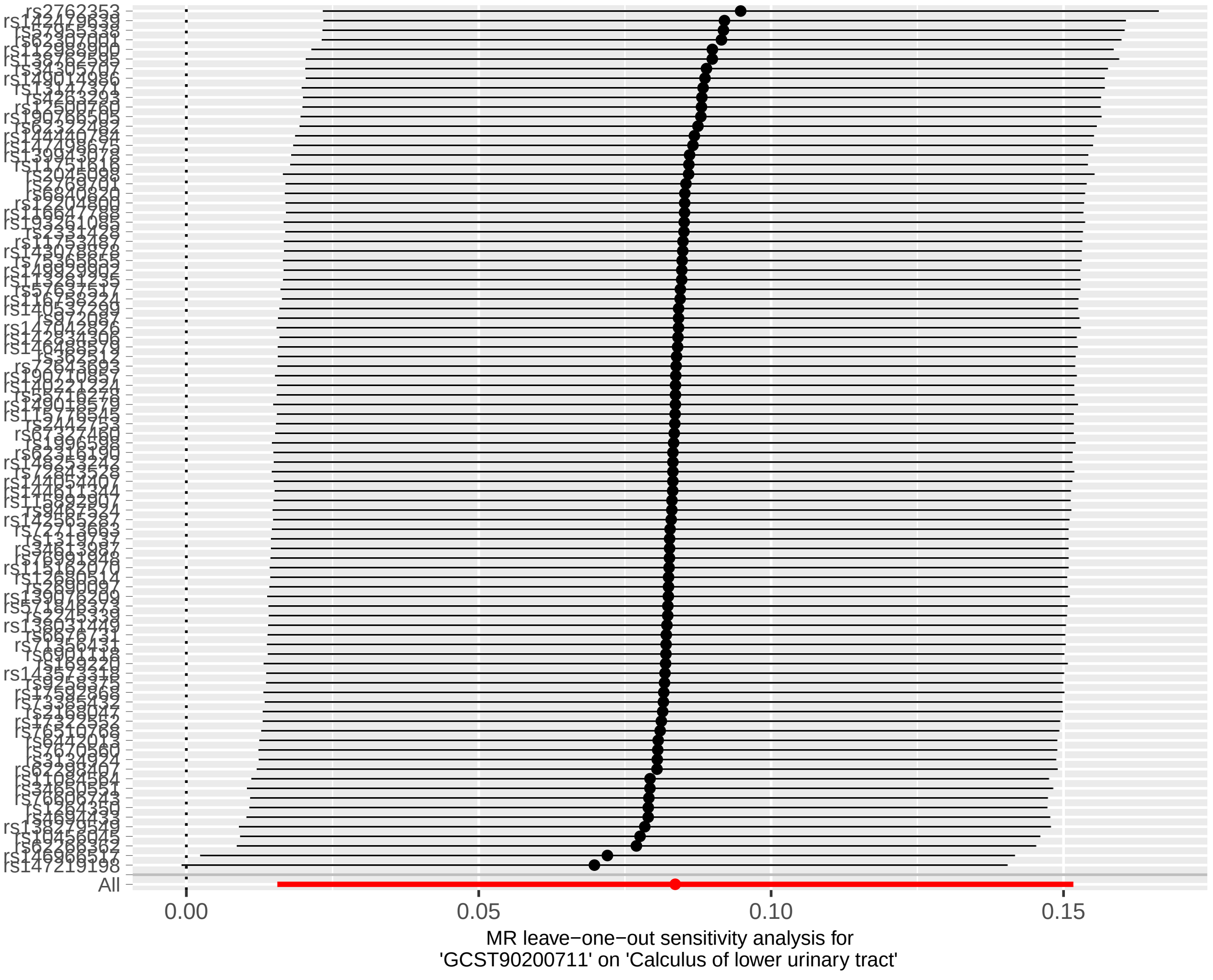
rs2762353
rs147219198
All
0.00
0.05
0.10
0.15
MR leave−one−out sensitivity analysis for
'GCST90200711' on 'Calculus of lower urinary tract'

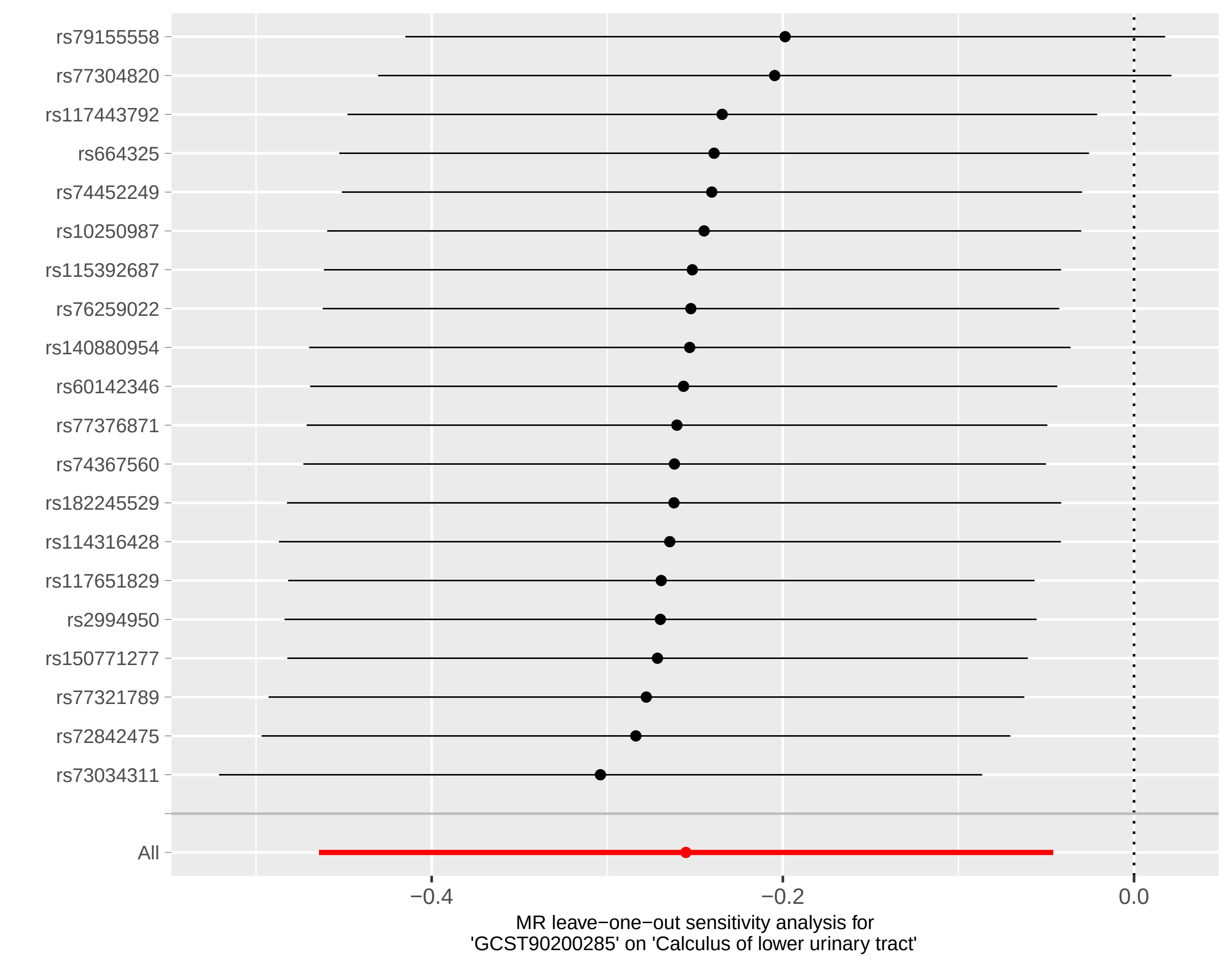
rs79155558
rs77304820
rs117443792
rs664325
rs74452249
rs10250987
rs115392687
rs76259022
rs140880954
rs60142346
rs77376871
rs74367560
rs182245529
rs114316428
rs117651829
rs2994950
rs150771277
rs77321789
rs72842475
rs73034311
All
−0.4
−0.2
0.0
MR leave−one−out sensitivity analysis for
'GCST90200285' on 'Calculus of lower urinary tract'

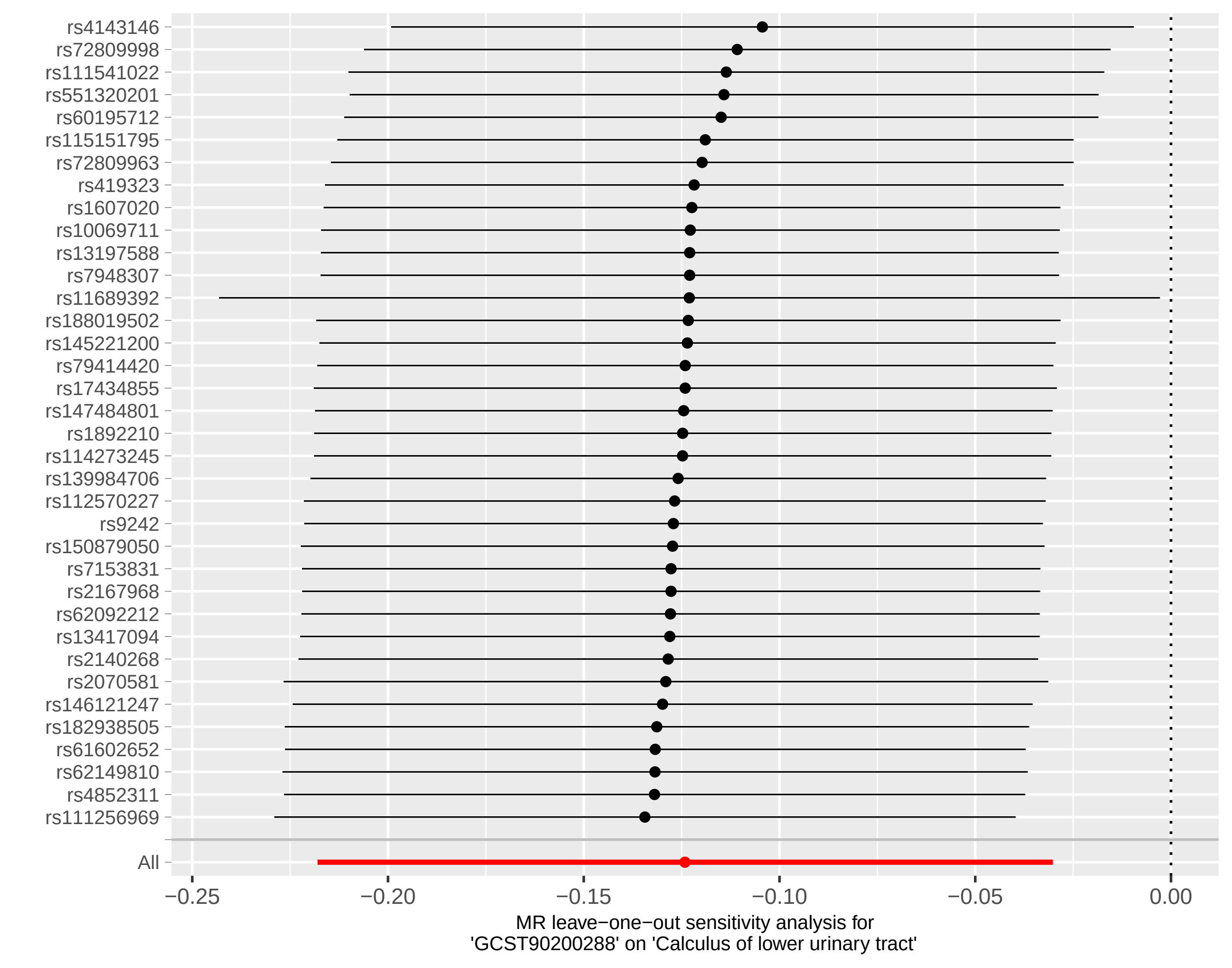

rs4143146
rs72809998
rs111541022
rs551320201
rs60195712
rs115151795
rs72809963
rs419323
rs1607020
rs10069711
rs13197588
rs7948307
rs11689392
rs188019502
rs145221200
rs79414420
rs17434855
rs147484801
rs1892210
rs114273245
rs139984706
rs112570227
rs9242
rs150879050
rs7153831
rs2167968
rs62092212
rs13417094
rs2140268
rs2070581
rs146121247
rs182938505
rs61602652
rs62149810
rs4852311
rs111256969
All
−0.25
−0.20
−0.15
−0.10
−0.05
0.00
MR leave−one−out sensitivity analysis for
'GCST90200288' on 'Calculus of lower urinary tract'

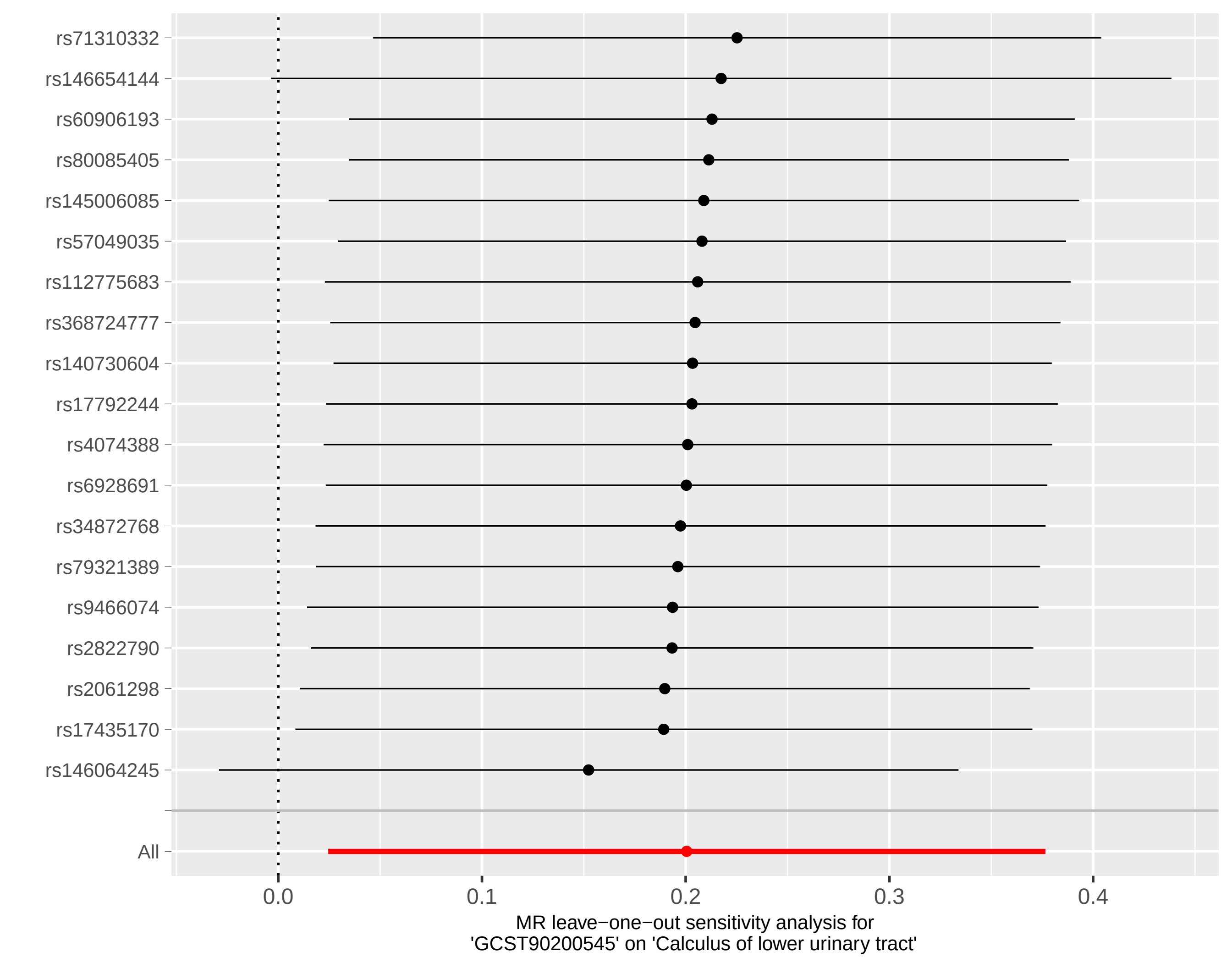

rs71310332
rs146654144
rs60906193
rs80085405
rs145006085
rs57049035
rs112775683
rs368724777
rs140730604
rs17792244
rs4074388
rs6928691
rs34872768
rs79321389
rs9466074
rs2822790
rs2061298
rs17435170
rs146064245
All
0.0
0.1
0.2
0.3
0.4
MR leave−one−out sensitivity analysis for
'GCST90200545' on 'Calculus of lower urinary tract'

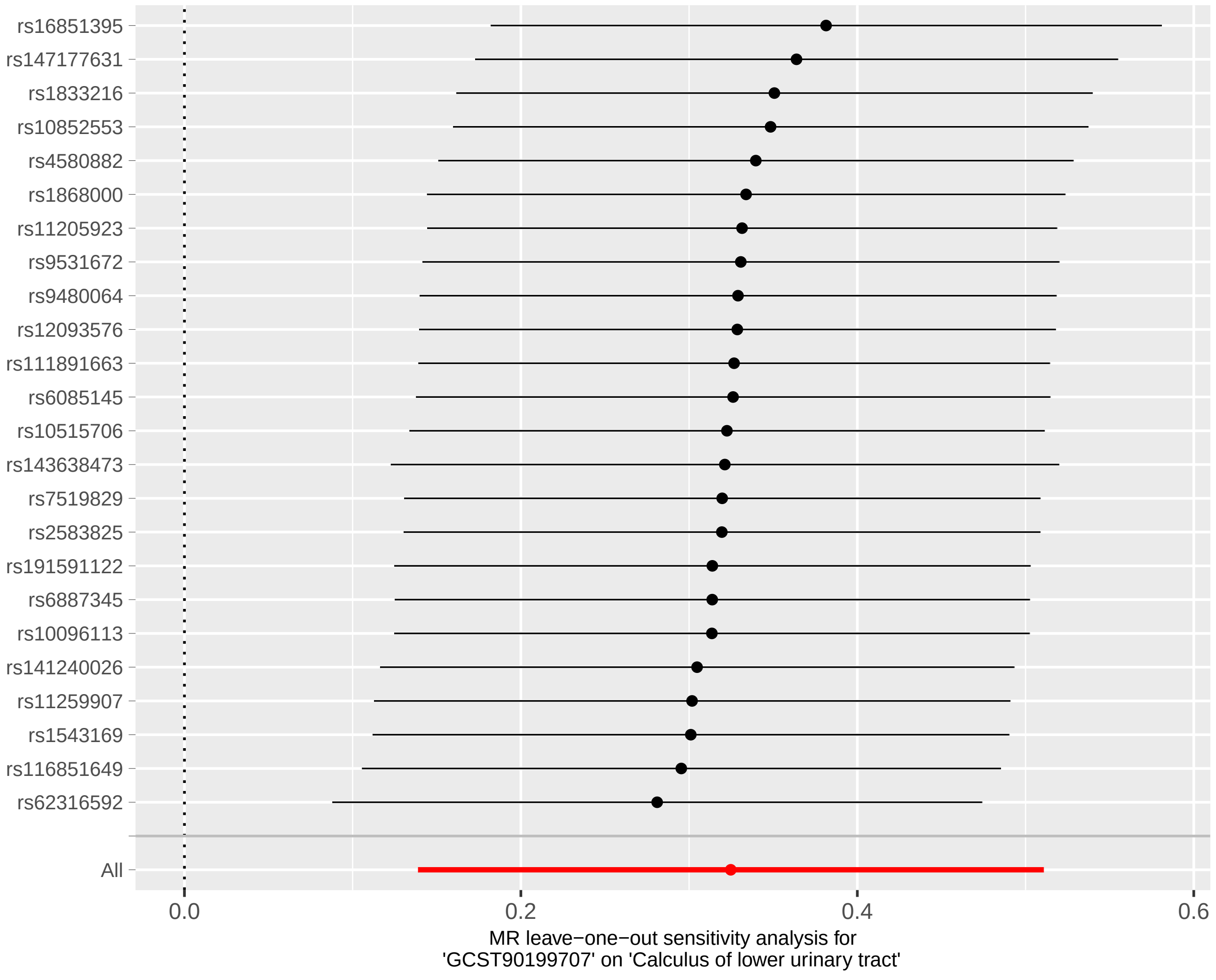
rs16851395
rs147177631
rs1833216
rs10852553
rs4580882
rs1868000
rs11205923
rs9531672
rs9480064
rs12093576
rs111891663
rs6085145
rs10515706
rs143638473
rs7519829
rs2583825
rs191591122
rs6887345
rs10096113
rs141240026
rs11259907
rs1543169
rs116851649
rs62316592
All
0.0
0.2
0.4
0.6
MR leave−one−out sensitivity analysis for
'GCST90199707' on 'Calculus of lower urinary tract'

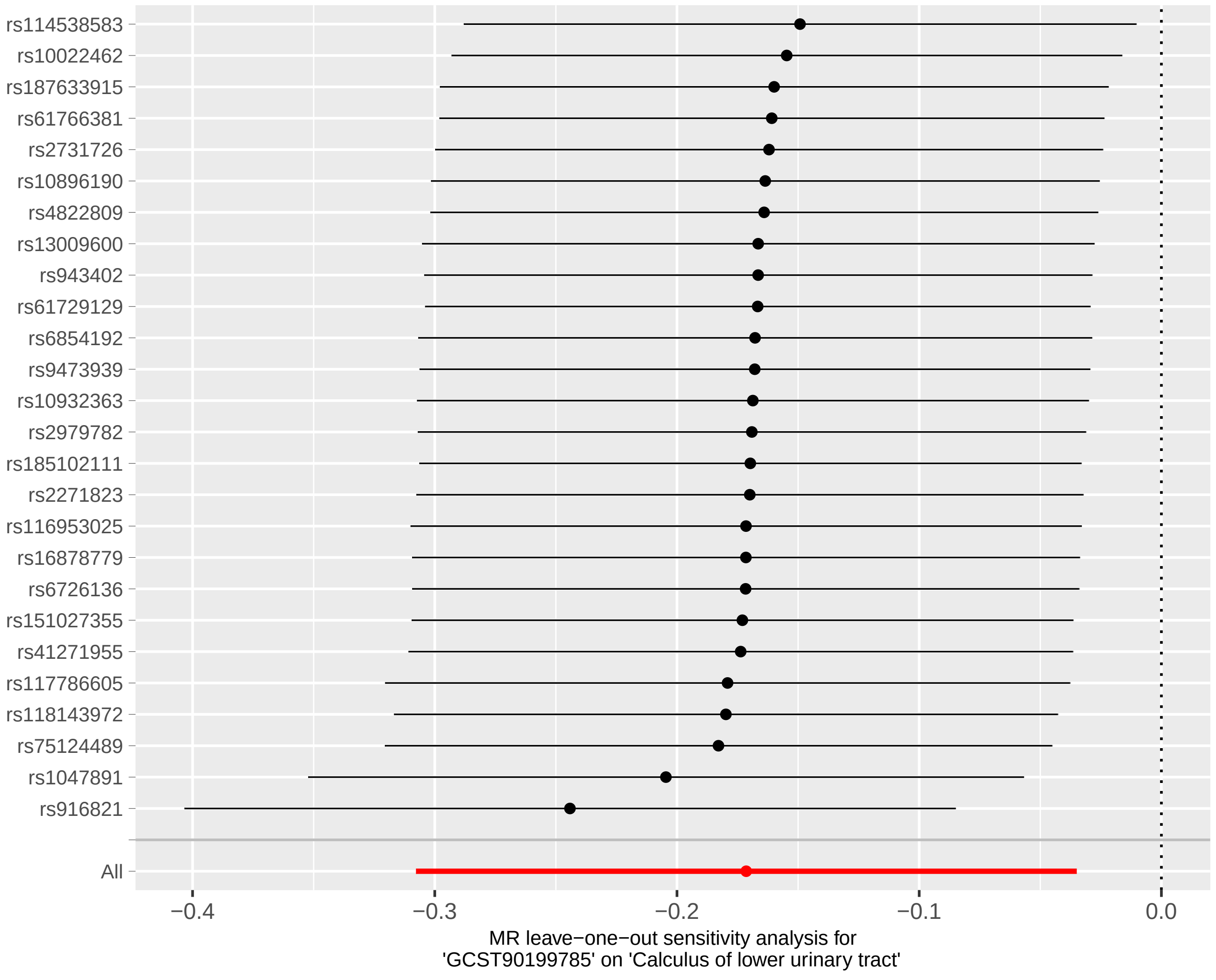
rs114538583
rs10022462
rs187633915
rs61766381
rs2731726
rs10896190
rs4822809
rs13009600
rs943402
rs61729129
rs6854192
rs9473939
rs10932363
rs2979782
rs185102111
rs2271823
rs116953025
rs16878779
rs6726136
rs151027355
rs41271955
rs117786605
rs118143972
rs75124489
rs1047891
rs916821
All
−0.4
−0.3
−0.2
−0.1
0.0
MR leave−one−out sensitivity analysis for
'GCST90199785' on 'Calculus of lower urinary tract'

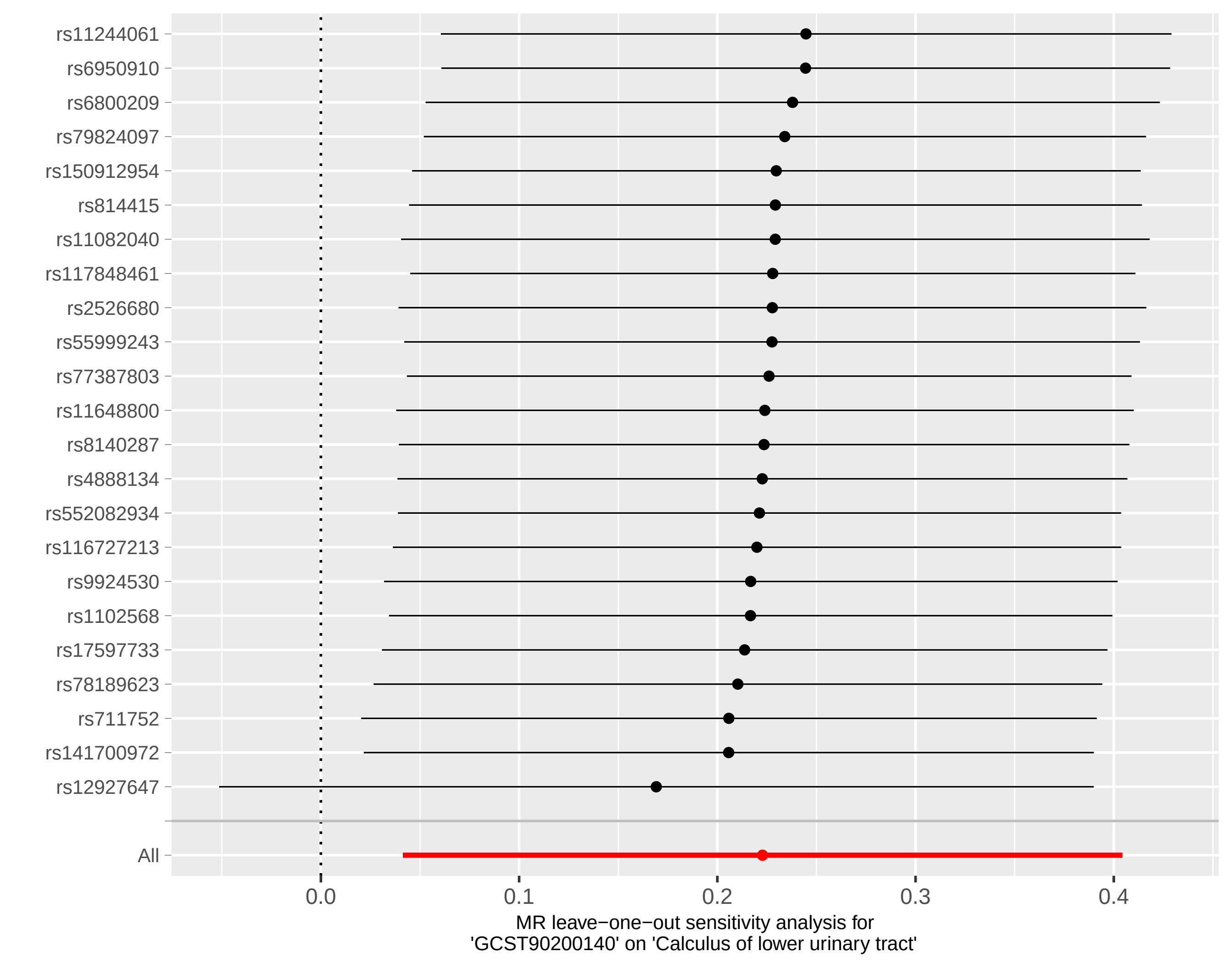
rs11244061
rs6950910
rs6800209
rs79824097
rs150912954
rs814415
rs11082040
rs117848461
rs2526680
rs55999243
rs77387803
rs11648800
rs8140287
rs4888134
rs552082934
rs116727213
rs9924530
rs1102568
rs17597733
rs78189623
rs711752
rs141700972
rs12927647
All
0.0
0.1
0.2
0.3
0.4
MR leave−one−out sensitivity analysis for
'GCST90200140' on 'Calculus of lower urinary tract'

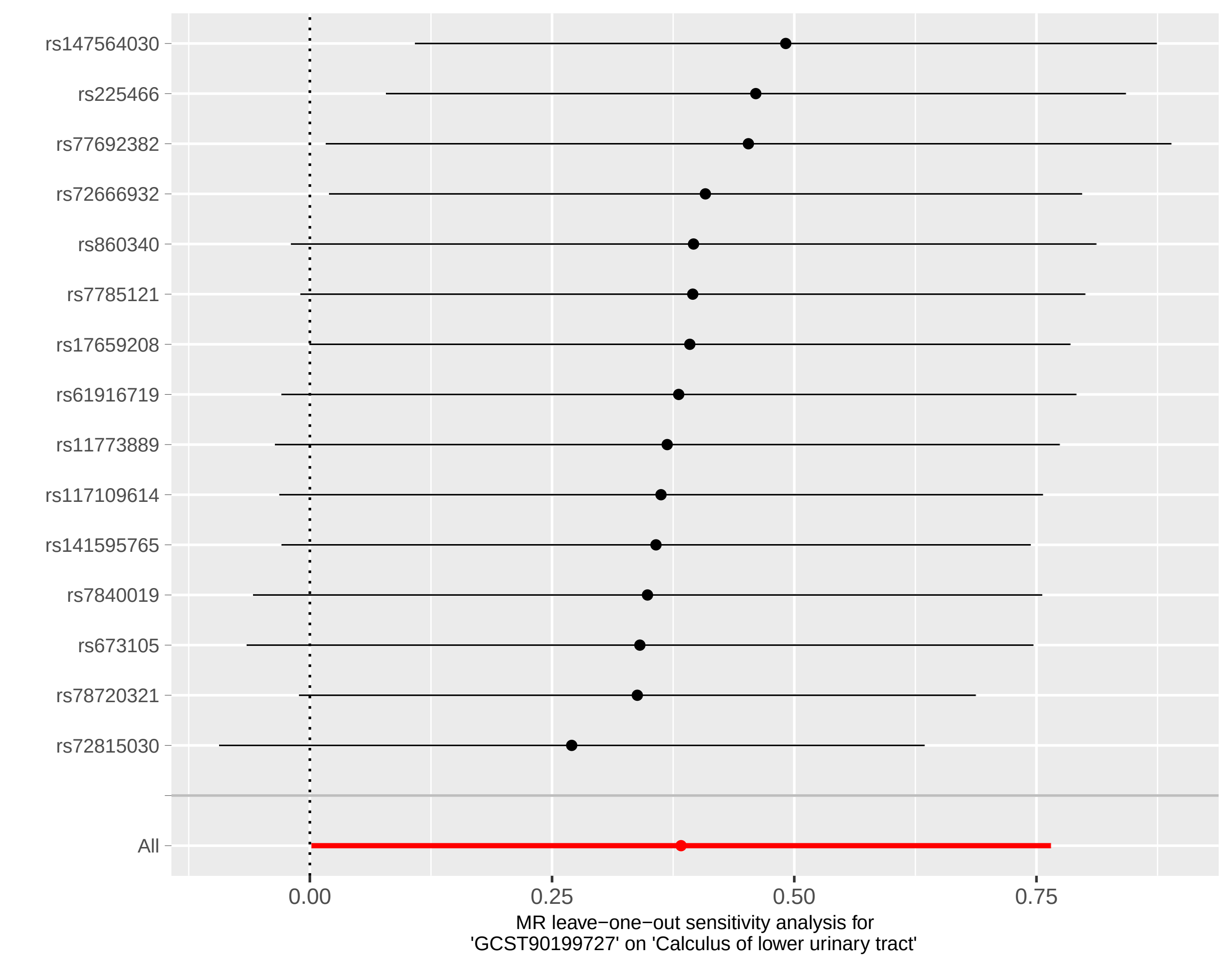
rs147564030
rs225466
rs77692382
rs72666932
rs860340
rs7785121
rs17659208
rs61916719
rs11773889
rs117109614
rs141595765
rs7840019
rs673105
rs78720321
rs72815030
All
0.00
0.25
0.50
0.75
MR leave−one−out sensitivity analysis for
'GCST90199727' on 'Calculus of lower urinary tract'

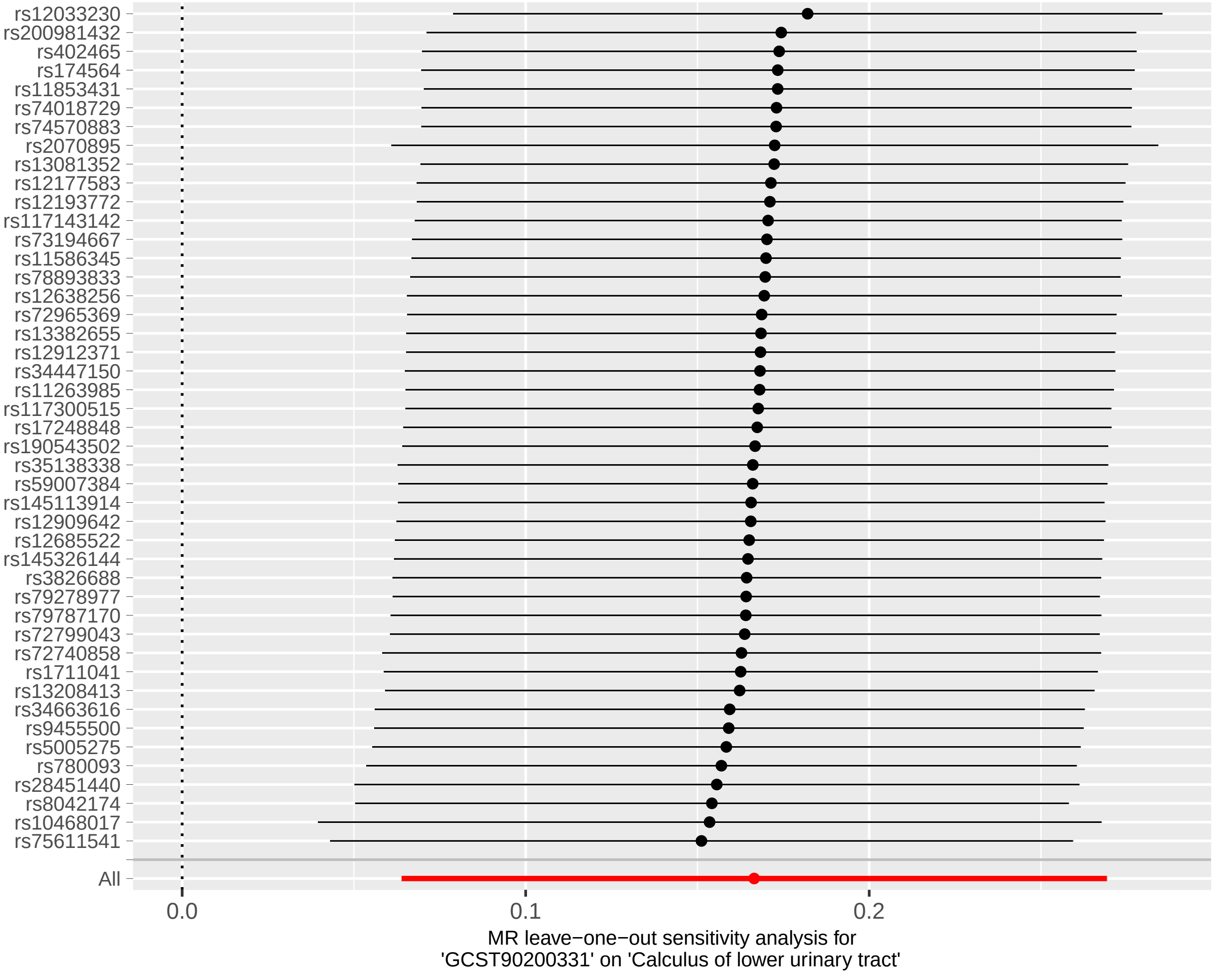
rs12033230
rs200981432
rs402465
rs174564
rs11853431
rs74018729
rs74570883
rs2070895
rs13081352
rs12177583
rs12193772
rs117143142
rs73194667
rs11586345
rs78893833
rs12638256
rs72965369
rs13382655
rs12912371
rs34447150
rs11263985
rs117300515
rs17248848
rs190543502
rs35138338
rs59007384
rs145113914
rs12909642
rs12685522
rs145326144
rs3826688
rs79278977
rs79787170
rs72799043
rs72740858
rs1711041
rs13208413
rs34663616
rs9455500
rs5005275
rs780093
rs28451440
rs8042174
rs10468017
rs75611541
All
0.0
0.1
0.2
MR leave−one−out sensitivity analysis for
'GCST90200331' on 'Calculus of lower urinary tract'

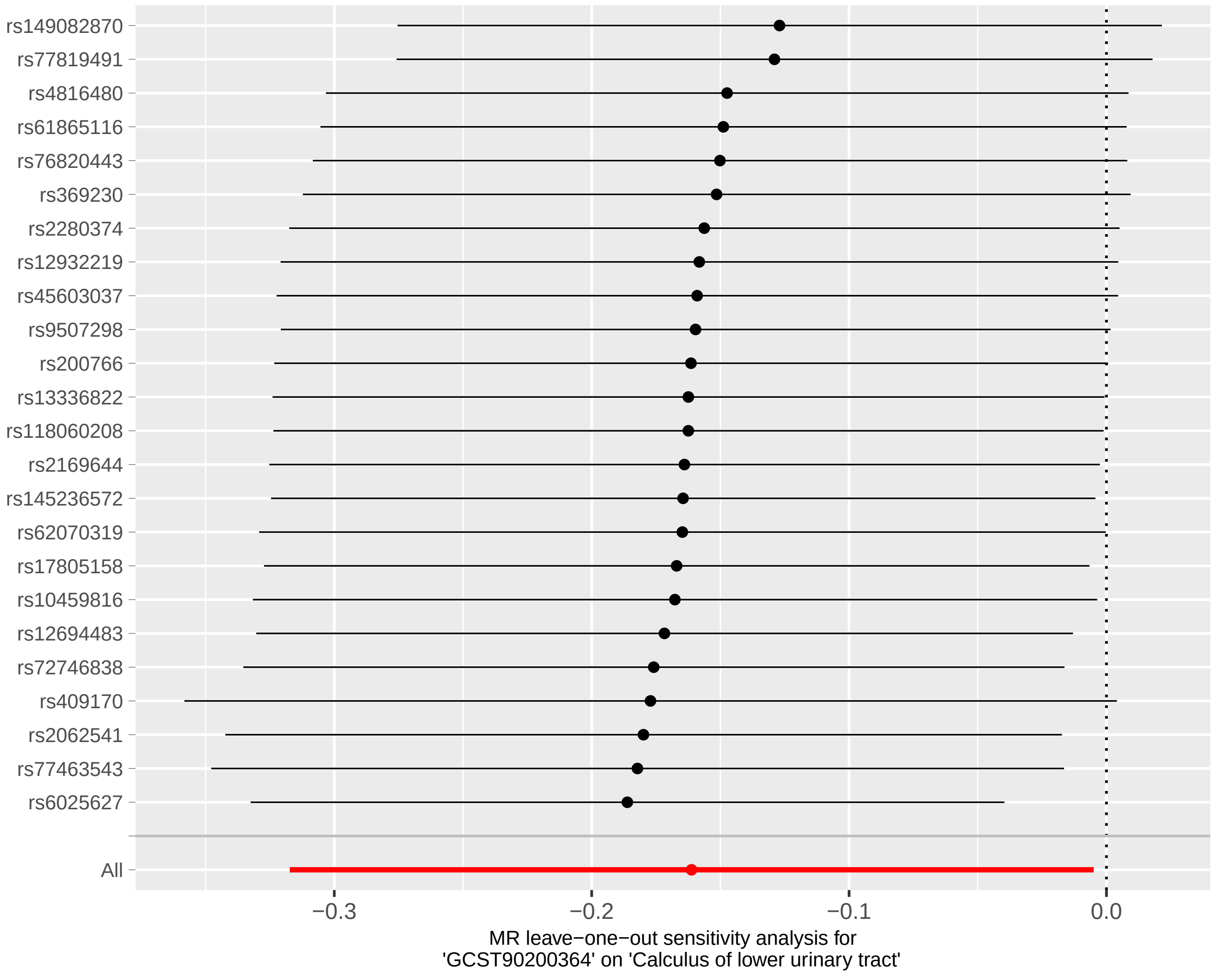
rs149082870
rs77819491
rs4816480
rs61865116
rs76820443
rs369230
rs2280374
rs12932219
rs45603037
rs9507298
rs200766
rs13336822
rs118060208
rs2169644
rs145236572
rs62070319
rs17805158
rs10459816
rs12694483
rs72746838
rs409170
rs2062541
rs77463543
rs6025627
All
−0.3
−0.2
−0.1
0.0
MR leave−one−out sensitivity analysis for
'GCST90200364' on 'Calculus of lower urinary tract'

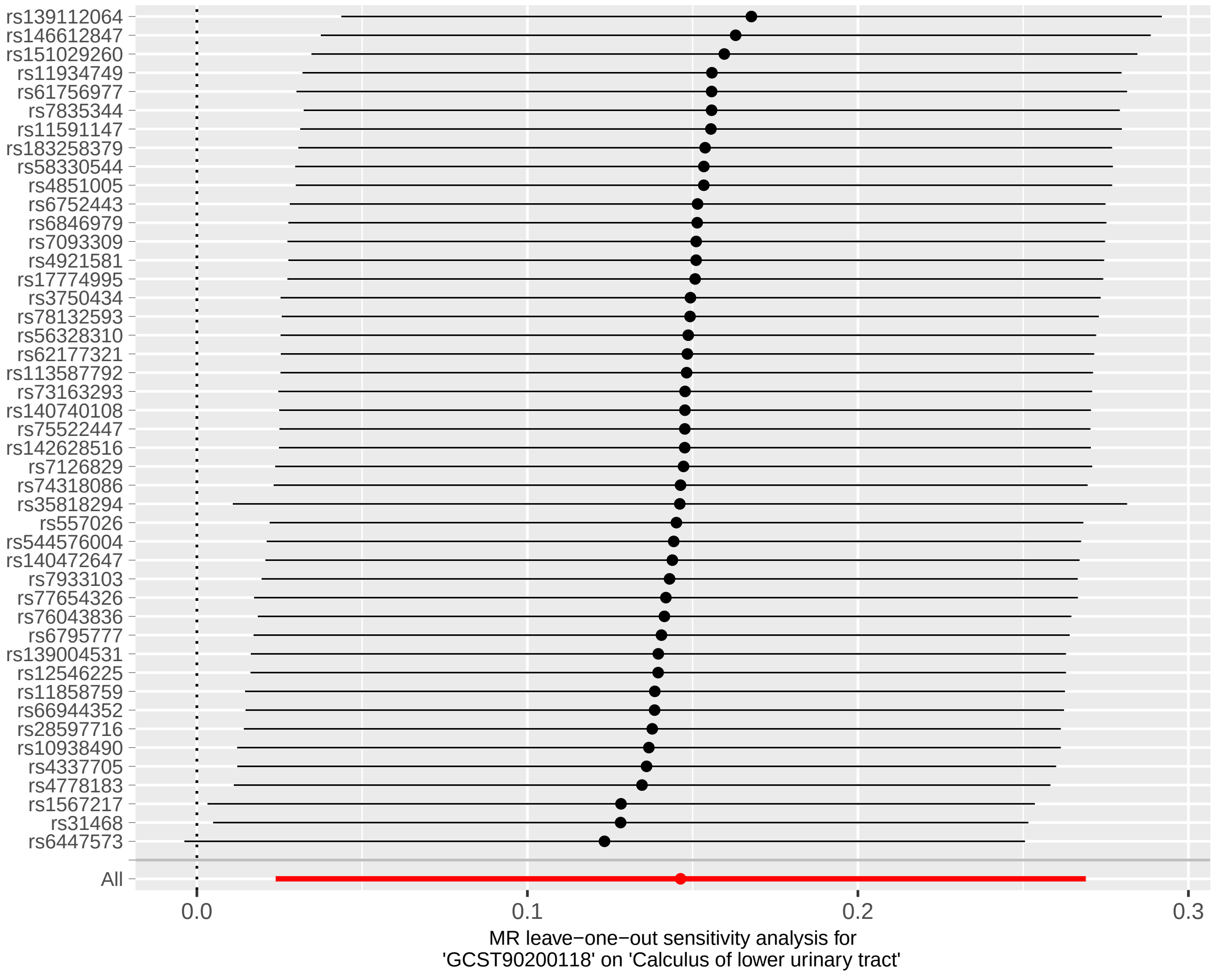
rs139112064
rs146612847
rs151029260
rs11934749
rs61756977
rs7835344
rs11591147
rs183258379
rs58330544
rs4851005
rs6752443
rs6846979
rs7093309
rs4921581
rs17774995
rs3750434
rs78132593
rs56328310
rs62177321
rs113587792
rs73163293
rs140740108
rs75522447
rs142628516
rs7126829
rs74318086
rs35818294
rs557026
rs544576004
rs140472647
rs7933103
rs77654326
rs76043836
rs6795777
rs139004531
rs12546225
rs11858759
rs66944352
rs28597716
rs10938490
rs4337705
rs4778183
rs1567217
rs31468
rs6447573
All
0.0
0.1
0.2
0.3
MR leave−one−out sensitivity analysis for
'GCST90200118' on 'Calculus of lower urinary tract'

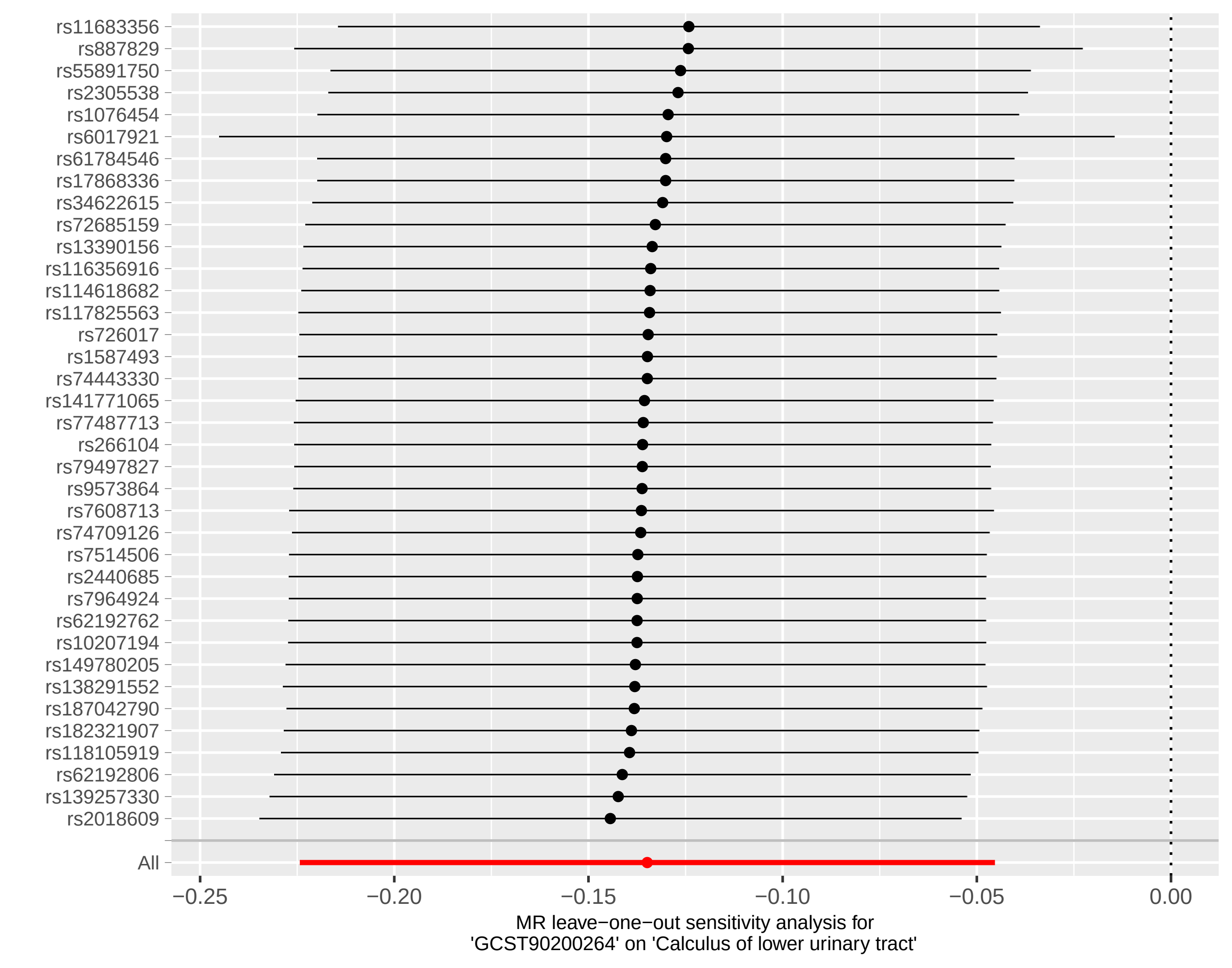
rs11683356
rs887829
rs55891750
rs2305538
rs1076454
rs6017921
rs61784546
rs17868336
rs34622615
rs72685159
rs13390156
rs116356916
rs114618682
rs117825563
rs726017
rs1587493
rs74443330
rs141771065
rs77487713
rs266104
rs79497827
rs9573864
rs7608713
rs74709126
rs7514506
rs2440685
rs7964924
rs62192762
rs10207194
rs149780205
rs138291552
rs187042790
rs182321907
rs118105919
rs62192806
rs139257330
rs2018609
All
−0.25
−0.20
−0.15
−0.10
−0.05
0.00
MR leave−one−out sensitivity analysis for
'GCST90200264' on 'Calculus of lower urinary tract'

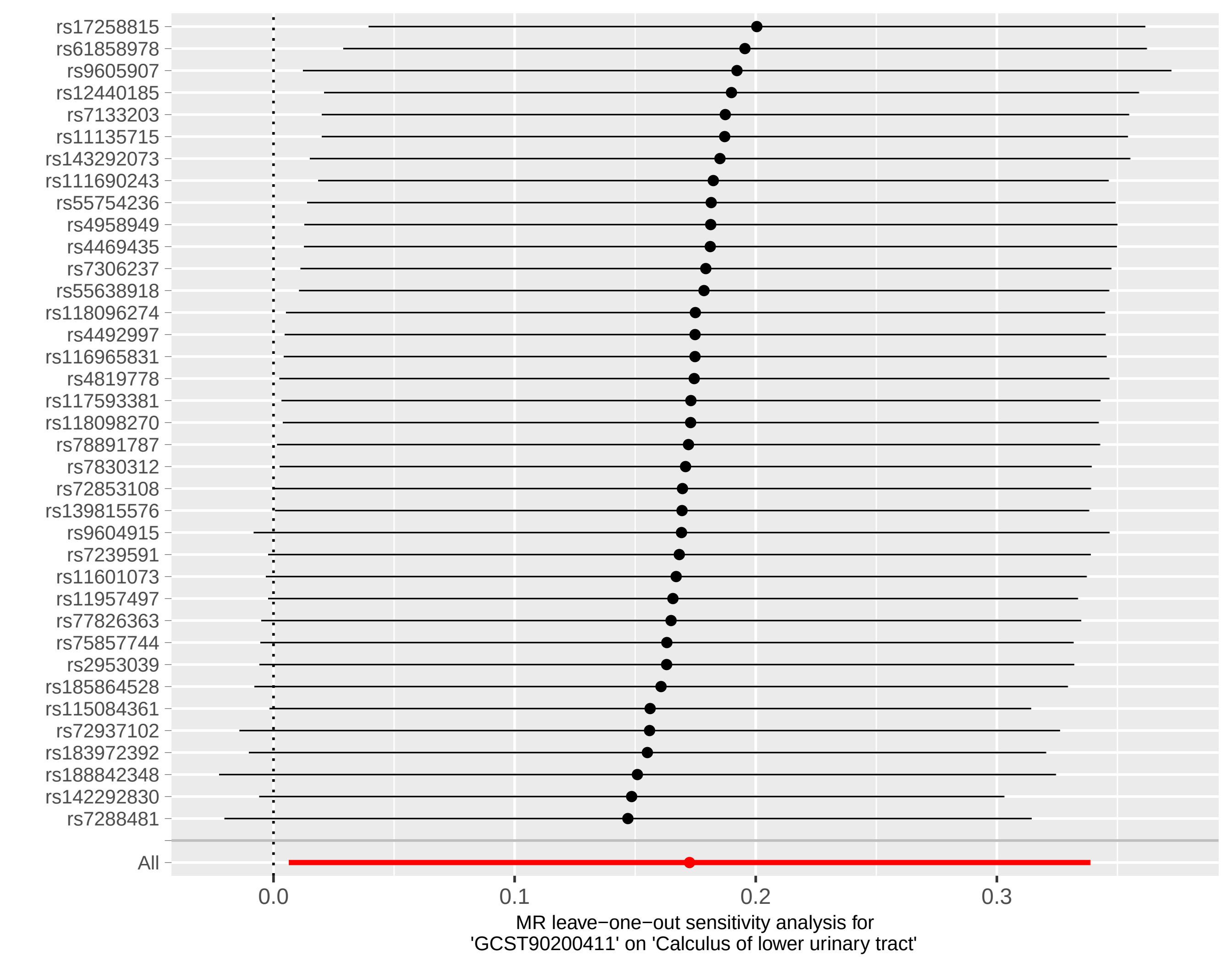
rs17258815
rs61858978
rs9605907
rs12440185
rs7133203
rs11135715
rs143292073
rs111690243
rs55754236
rs4958949
rs4469435
rs7306237
rs55638918
rs118096274
rs4492997
rs116965831
rs4819778
rs117593381
rs118098270
rs78891787
rs7830312
rs72853108
rs139815576
rs9604915
rs7239591
rs11601073
rs11957497
rs77826363
rs75857744
rs2953039
rs185864528
rs115084361
rs72937102
rs183972392
rs188842348
rs142292830
rs7288481
All
0.0
0.1
0.2
0.3
MR leave−one−out sensitivity analysis for
'GCST90200411' on 'Calculus of lower urinary tract'

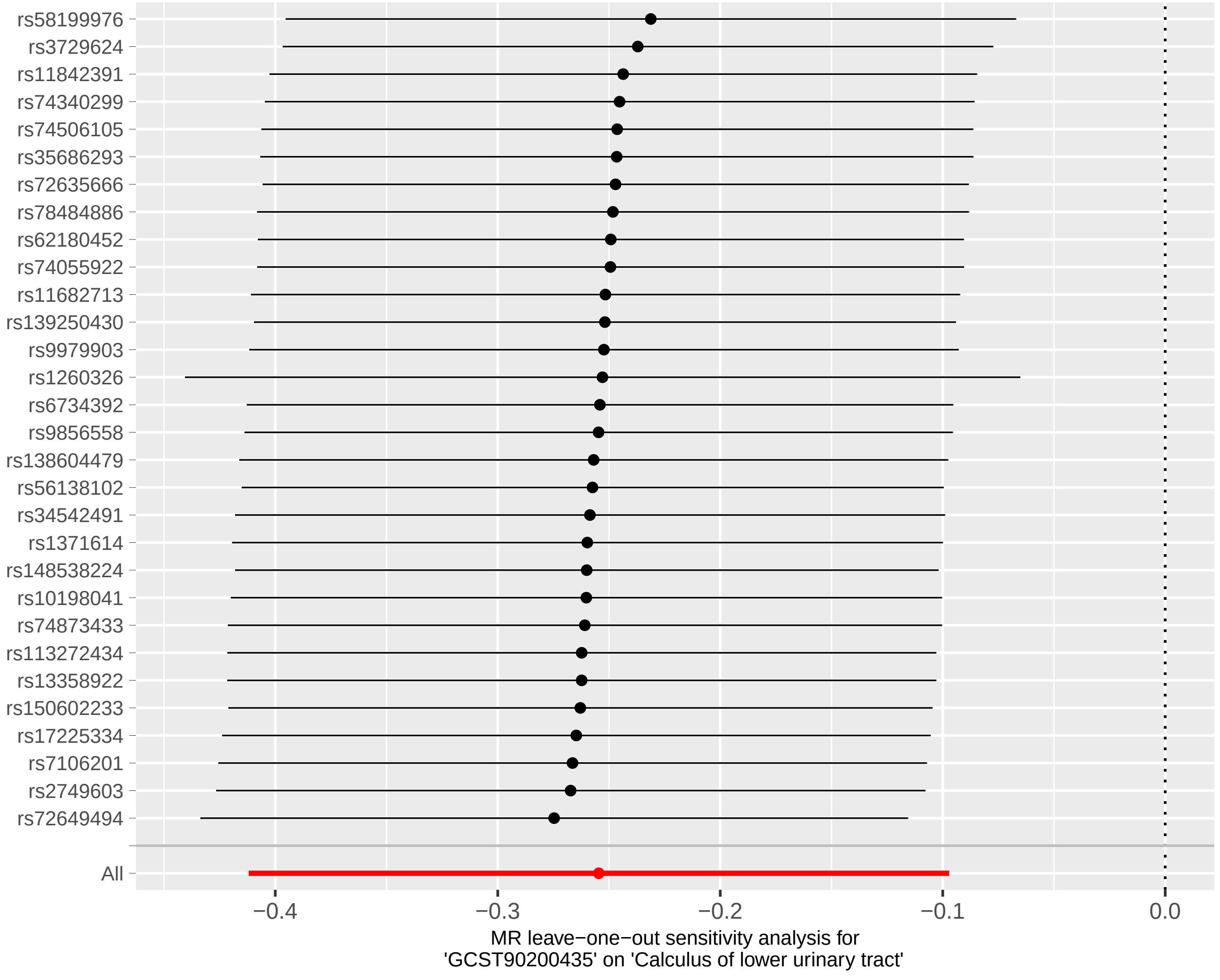

rs58199976
rs3729624
rs11842391
rs74340299
rs74506105
rs35686293
rs72635666
rs78484886
rs62180452
rs74055922
rs11682713
rs139250430
rs9979903
rs1260326
rs6734392
rs9856558
rs138604479
rs56138102
rs34542491
rs1371614
rs148538224
rs10198041
rs74873433
rs113272434
rs13358922
rs150602233
rs17225334
rs7106201
rs2749603
rs72649494
All
−0.4
−0.3
−0.2
−0.1
0.0
MR leave−one−out sensitivity analysis for
'GCST90200435' on 'Calculus of lower urinary tract'

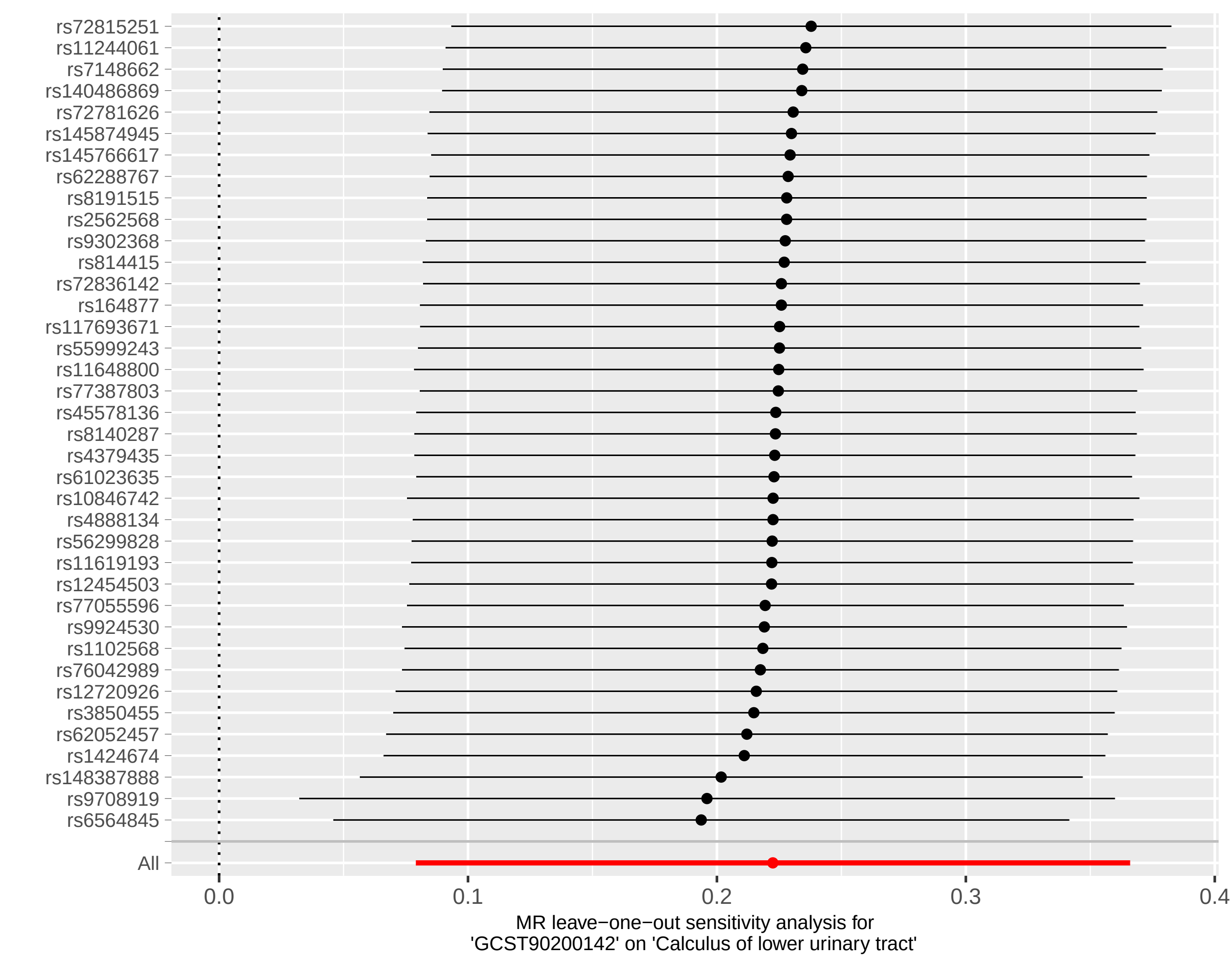
rs72815251
rs11244061
rs7148662
rs140486869
rs72781626
rs145874945
rs145766617
rs62288767
rs8191515
rs2562568
rs9302368
rs814415
rs72836142
rs164877
rs117693671
rs55999243
rs11648800
rs77387803
rs45578136
rs8140287
rs4379435
rs61023635
rs10846742
rs4888134
rs56299828
rs11619193
rs12454503
rs77055596
rs9924530
rs1102568
rs76042989
rs12720926
rs3850455
rs62052457
rs1424674
rs148387888
rs9708919
rs6564845
All
0.0
0.1
0.2
0.3
0.4
MR leave−one−out sensitivity analysis for
'GCST90200142' on 'Calculus of lower urinary tract'

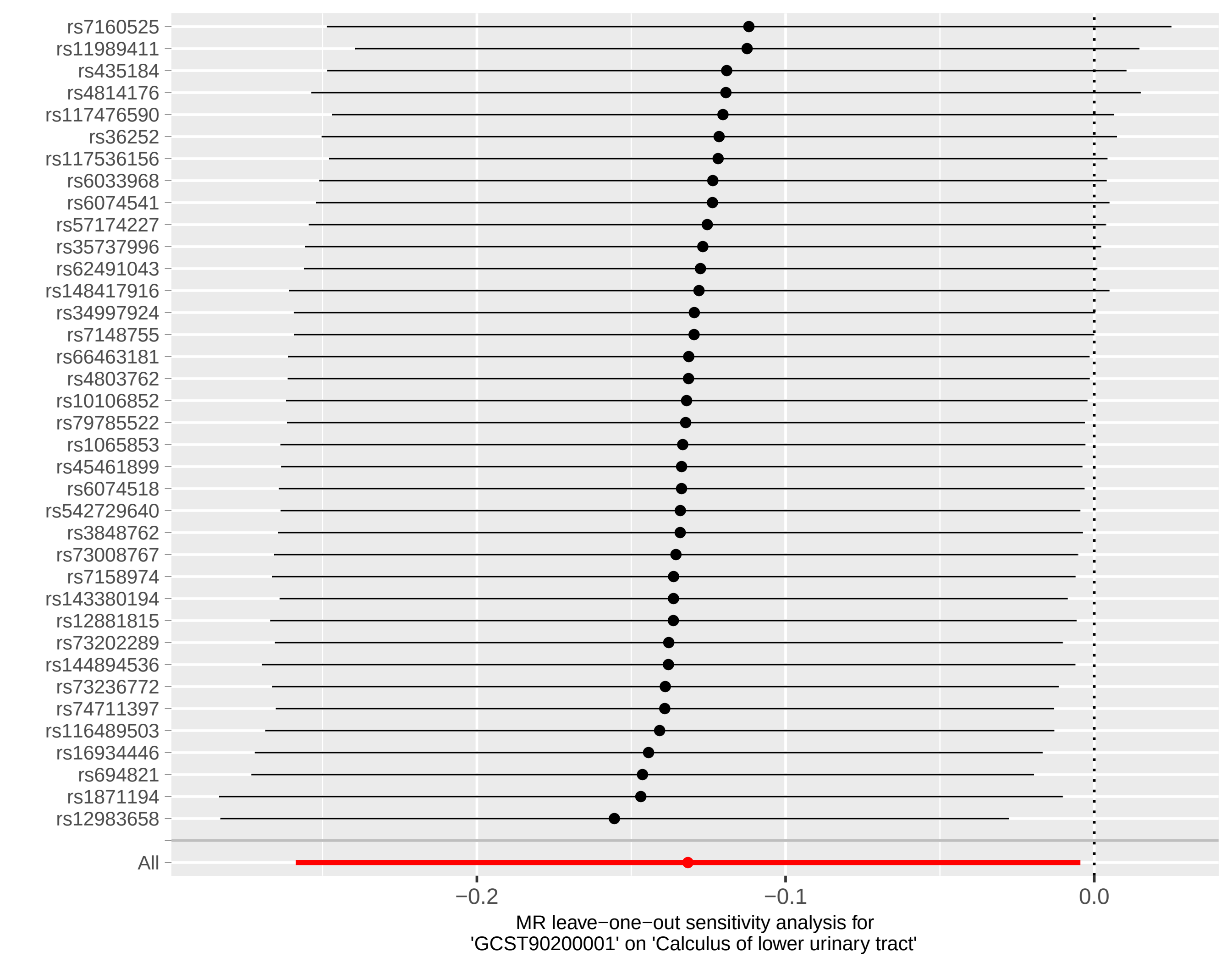
rs7160525
rs11989411
rs435184
rs4814176
rs117476590
rs36252
rs117536156
rs6033968
rs6074541
rs57174227
rs35737996
rs62491043
rs148417916
rs34997924
rs7148755
rs66463181
rs4803762
rs10106852
rs79785522
rs1065853
rs45461899
rs6074518
rs542729640
rs3848762
rs73008767
rs7158974
rs143380194
rs12881815
rs73202289
rs144894536
rs73236772
rs74711397
rs116489503
rs16934446
rs694821
rs1871194
rs12983658
All
−0.2
−0.1
0.0
MR leave−one−out sensitivity analysis for
'GCST90200001' on 'Calculus of lower urinary tract'

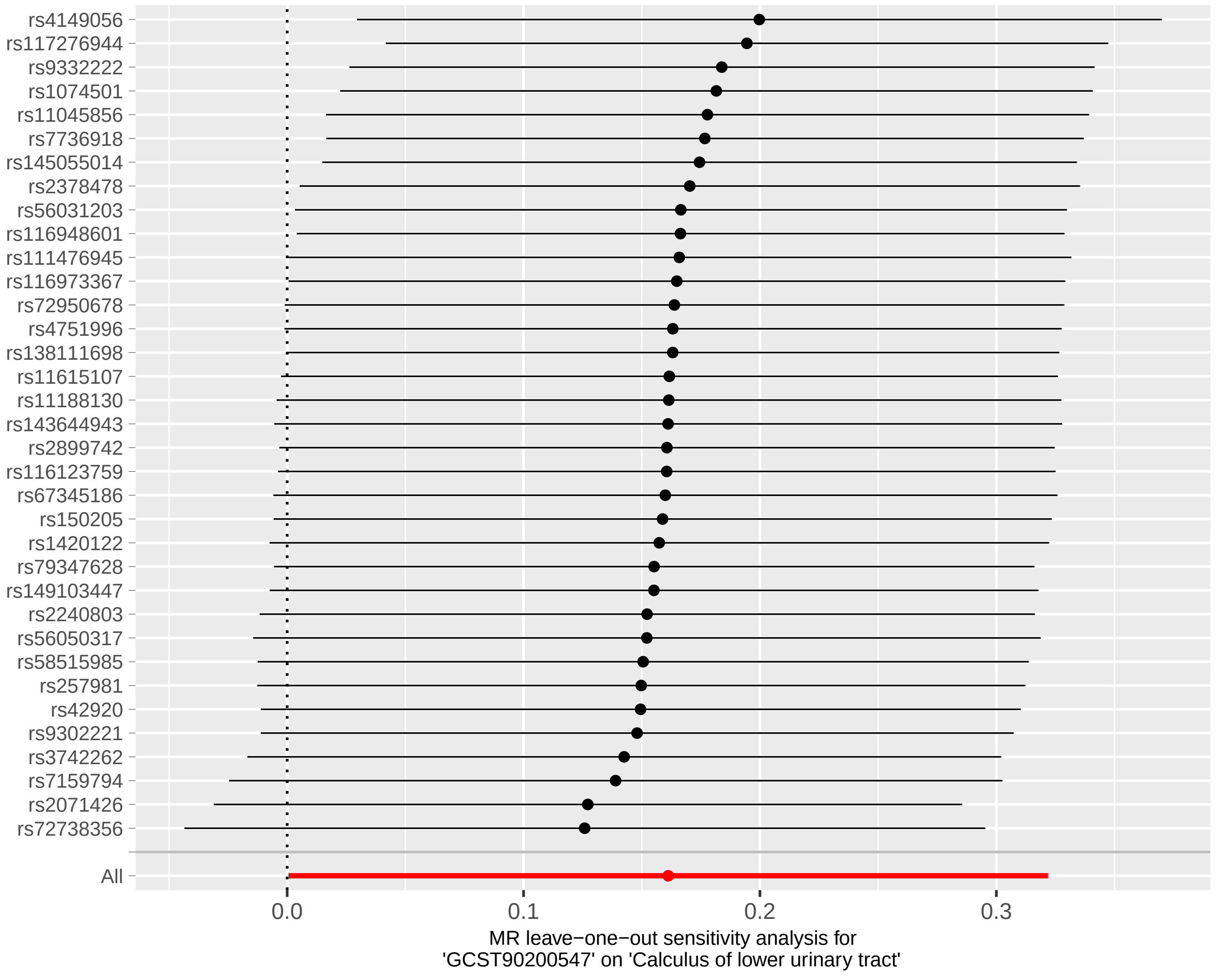
rs4149056
rs117276944
rs9332222
rs1074501
rs11045856
rs7736918
rs145055014
rs2378478
rs56031203
rs116948601
rs111476945
rs116973367
rs72950678
rs4751996
rs138111698
rs11615107
rs11188130
rs143644943
rs2899742
rs116123759
rs67345186
rs150205
rs1420122
rs79347628
rs149103447
rs2240803
rs56050317
rs58515985
rs257981
rs42920
rs9302221
rs3742262
rs7159794
rs2071426
rs72738356
All
0.0
0.1
0.2
0.3
MR leave−one−out sensitivity analysis for
'GCST90200547' on 'Calculus of lower urinary tract'

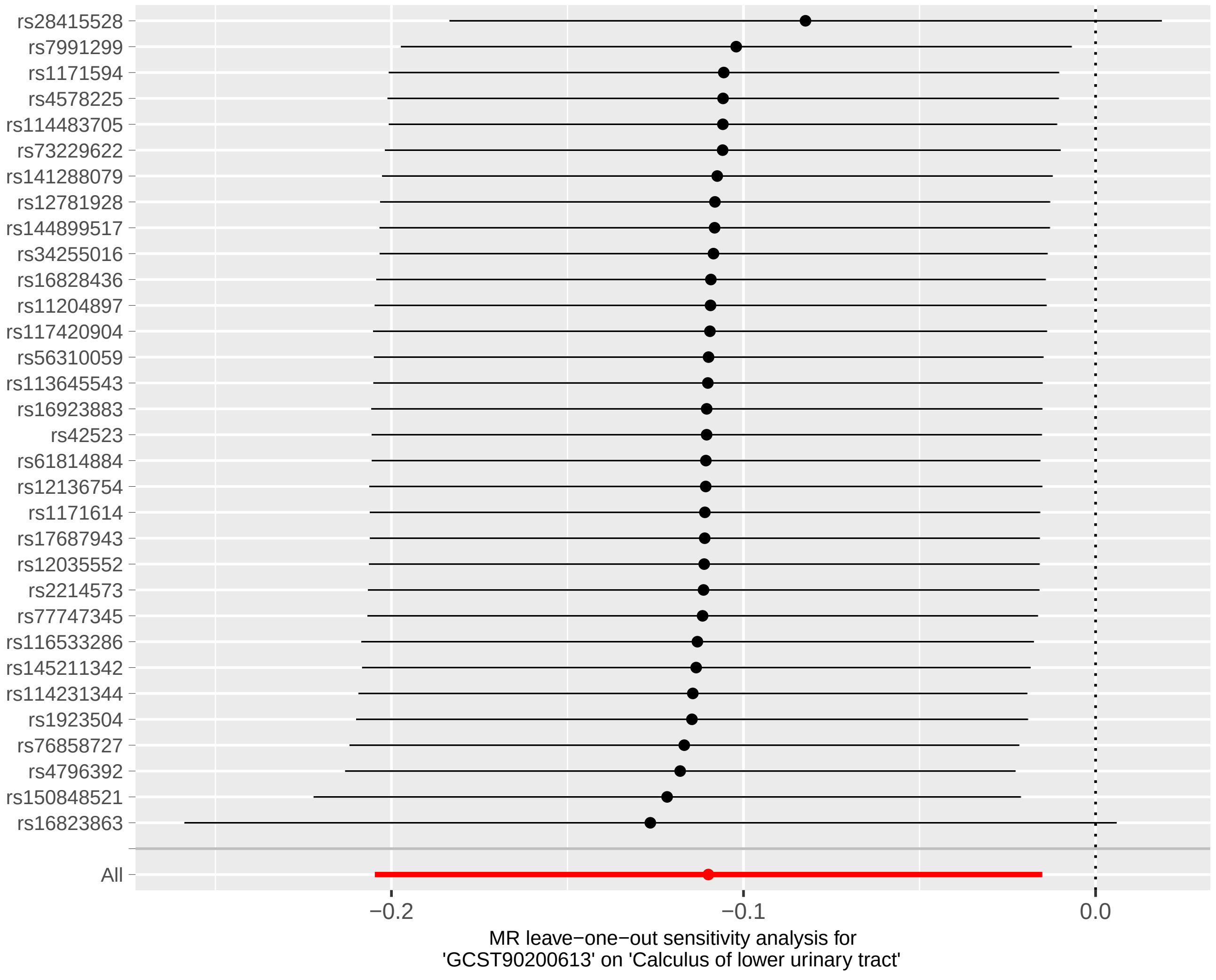
rs28415528
rs7991299
rs1171594
rs4578225
rs114483705
rs73229622
rs141288079
rs12781928
rs144899517
rs34255016
rs16828436
rs11204897
rs117420904
rs56310059
rs113645543
rs16923883
rs42523
rs61814884
rs12136754
rs1171614
rs17687943
rs12035552
rs2214573
rs77747345
rs116533286
rs145211342
rs114231344
rs1923504
rs76858727
rs4796392
rs150848521
rs16823863
All
−0.2
−0.1
0.0
MR leave−one−out sensitivity analysis for
'GCST90200613' on 'Calculus of lower urinary tract'

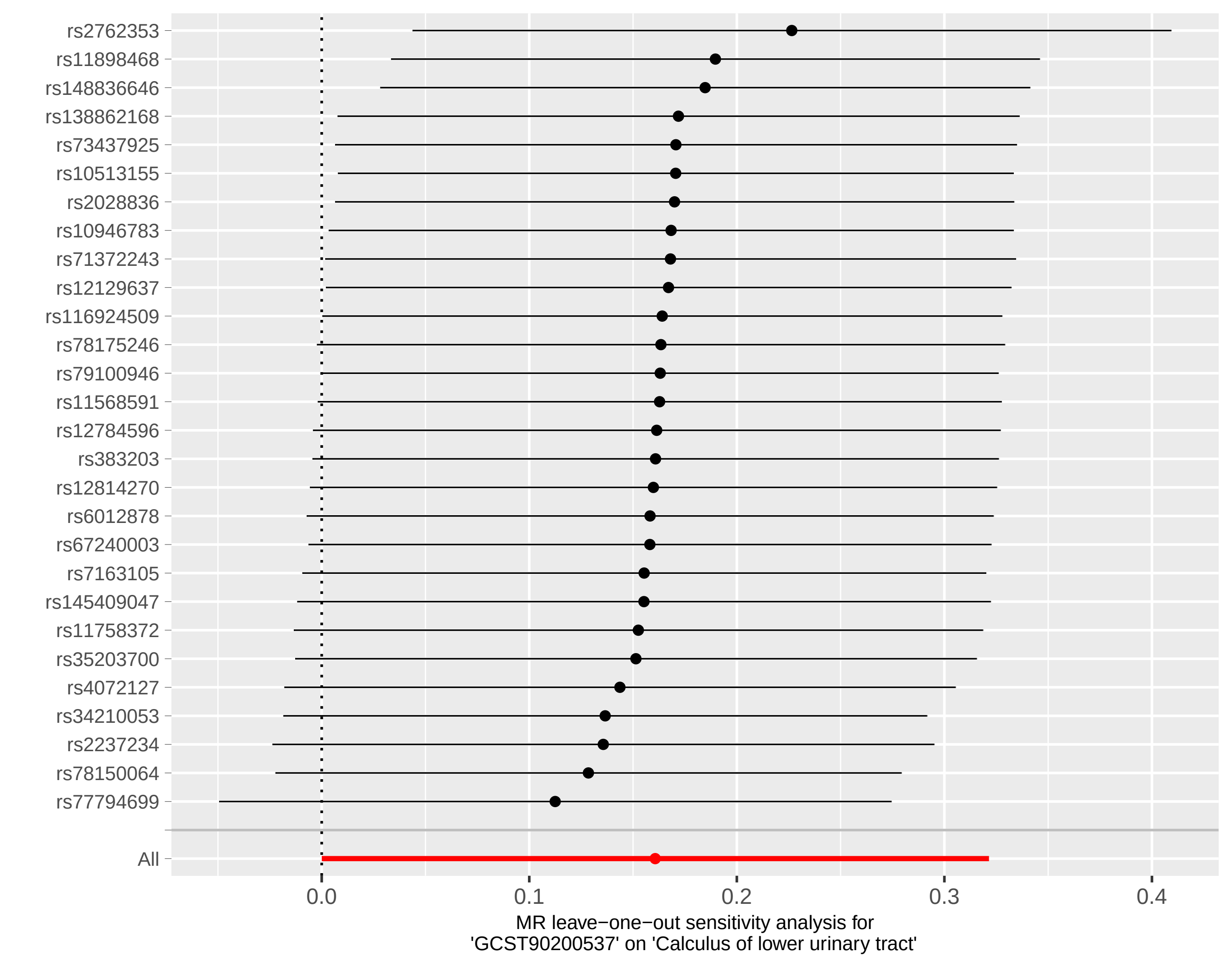
rs2762353
rs11898468
rs148836646
rs138862168
rs73437925
rs10513155
rs2028836
rs10946783
rs71372243
rs12129637
rs116924509
rs78175246
rs79100946
rs11568591
rs12784596
rs383203
rs12814270
rs6012878
rs67240003
rs7163105
rs145409047
rs11758372
rs35203700
rs4072127
rs34210053
rs2237234
rs78150064
rs77794699
All
0.0
0.1
0.2
0.3
0.4
MR leave−one−out sensitivity analysis for
'GCST90200537' on 'Calculus of lower urinary tract'

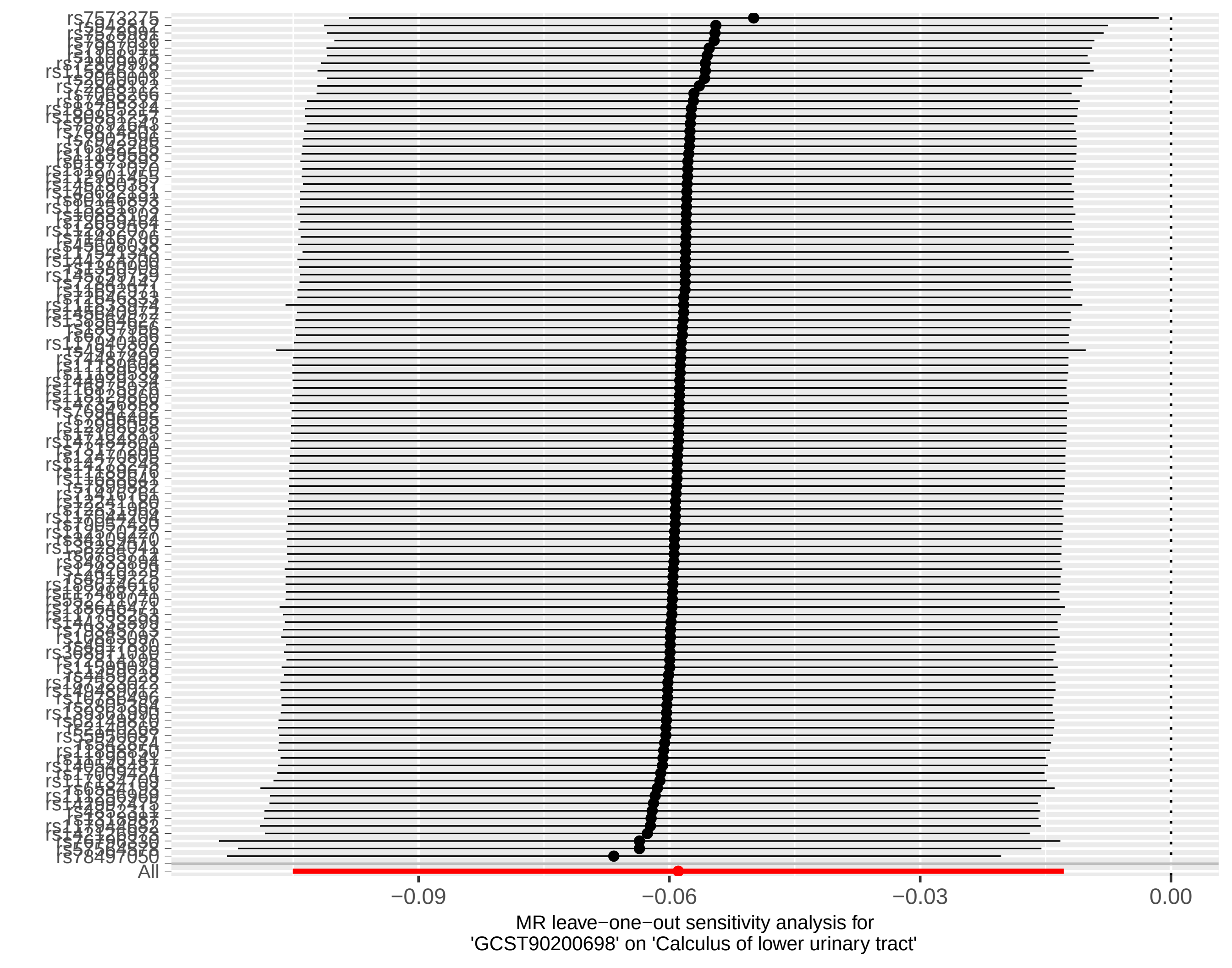
rs7573275
rs78497050
All
−0.09
−0.06
−0.03
0.00
MR leave−one−out sensitivity analysis for
'GCST90200698' on 'Calculus of lower urinary tract'

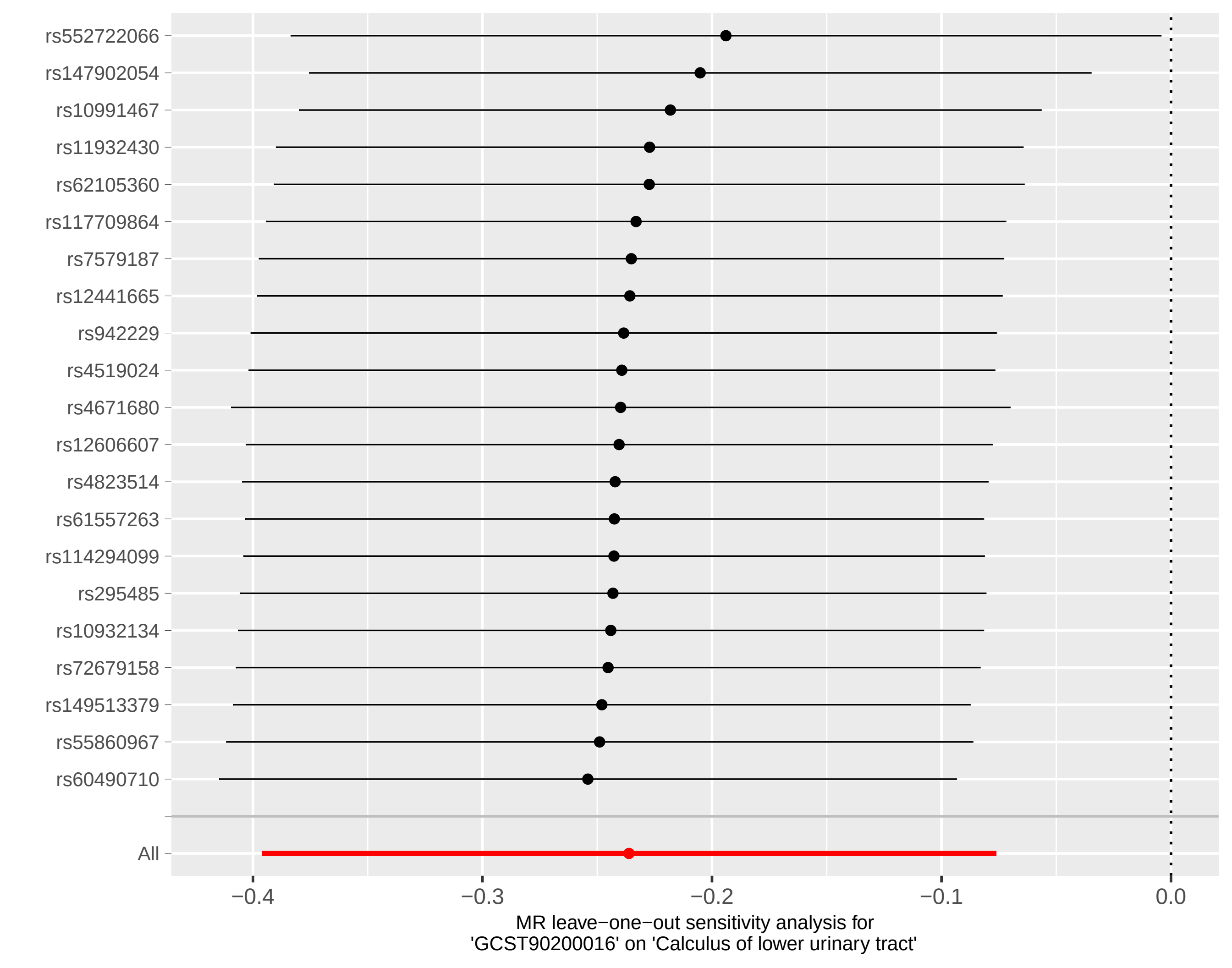
rs552722066
rs147902054
rs10991467
rs11932430
rs62105360
rs117709864
rs7579187
rs12441665
rs942229
rs4519024
rs4671680
rs12606607
rs4823514
rs61557263
rs114294099
rs295485
rs10932134
rs72679158
rs149513379
rs55860967
rs60490710
All
−0.4
−0.3
−0.2
−0.1
0.0
MR leave−one−out sensitivity analysis for
'GCST90200016' on 'Calculus of lower urinary tract'

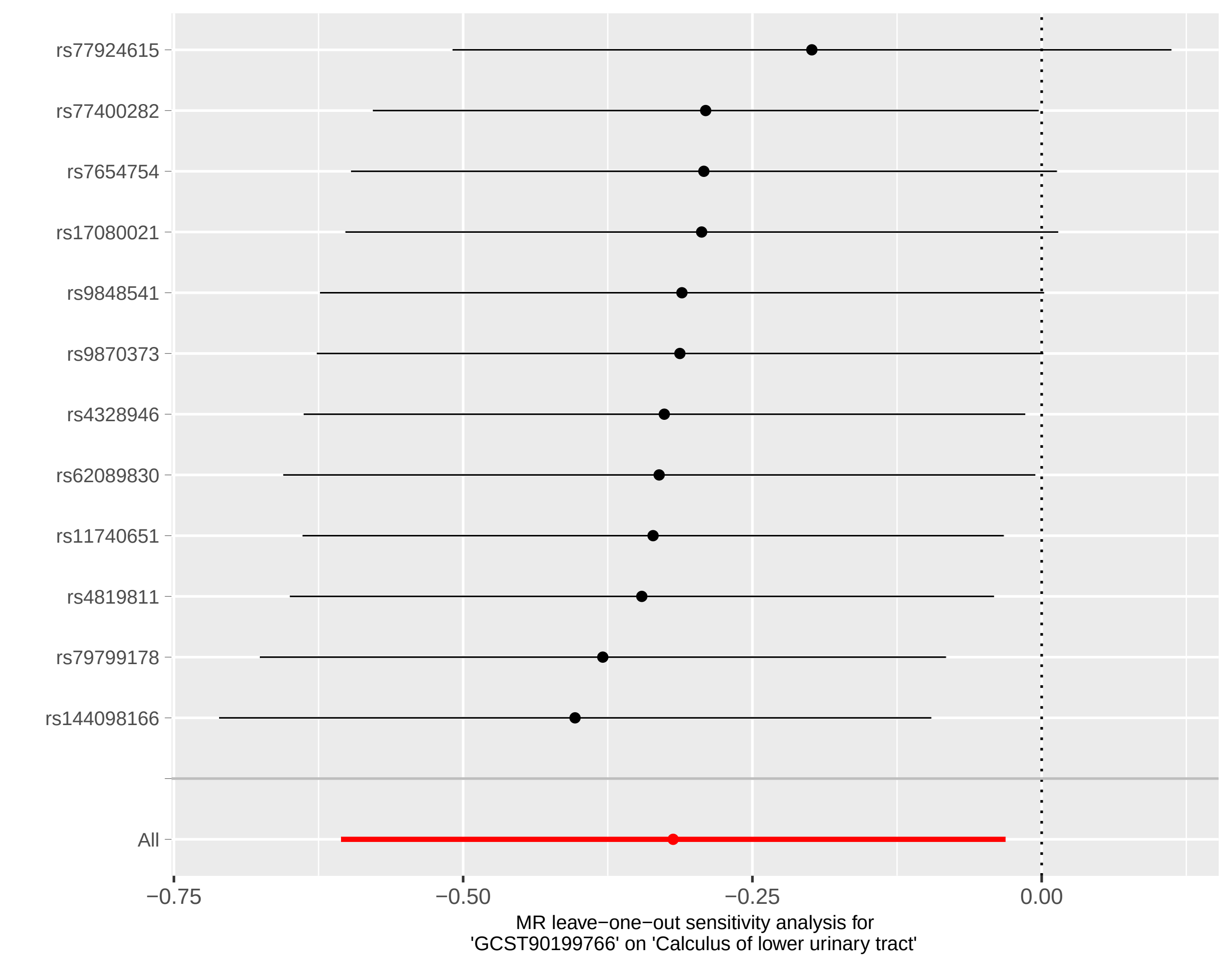
rs77924615
rs77400282
rs7654754
rs17080021
rs9848541
rs9870373
rs4328946
rs62089830
rs11740651
rs4819811
rs79799178
rs144098166
All
−0.75
−0.50
−0.25
0.00
MR leave−one−out sensitivity analysis for
'GCST90199766' on 'Calculus of lower urinary tract'

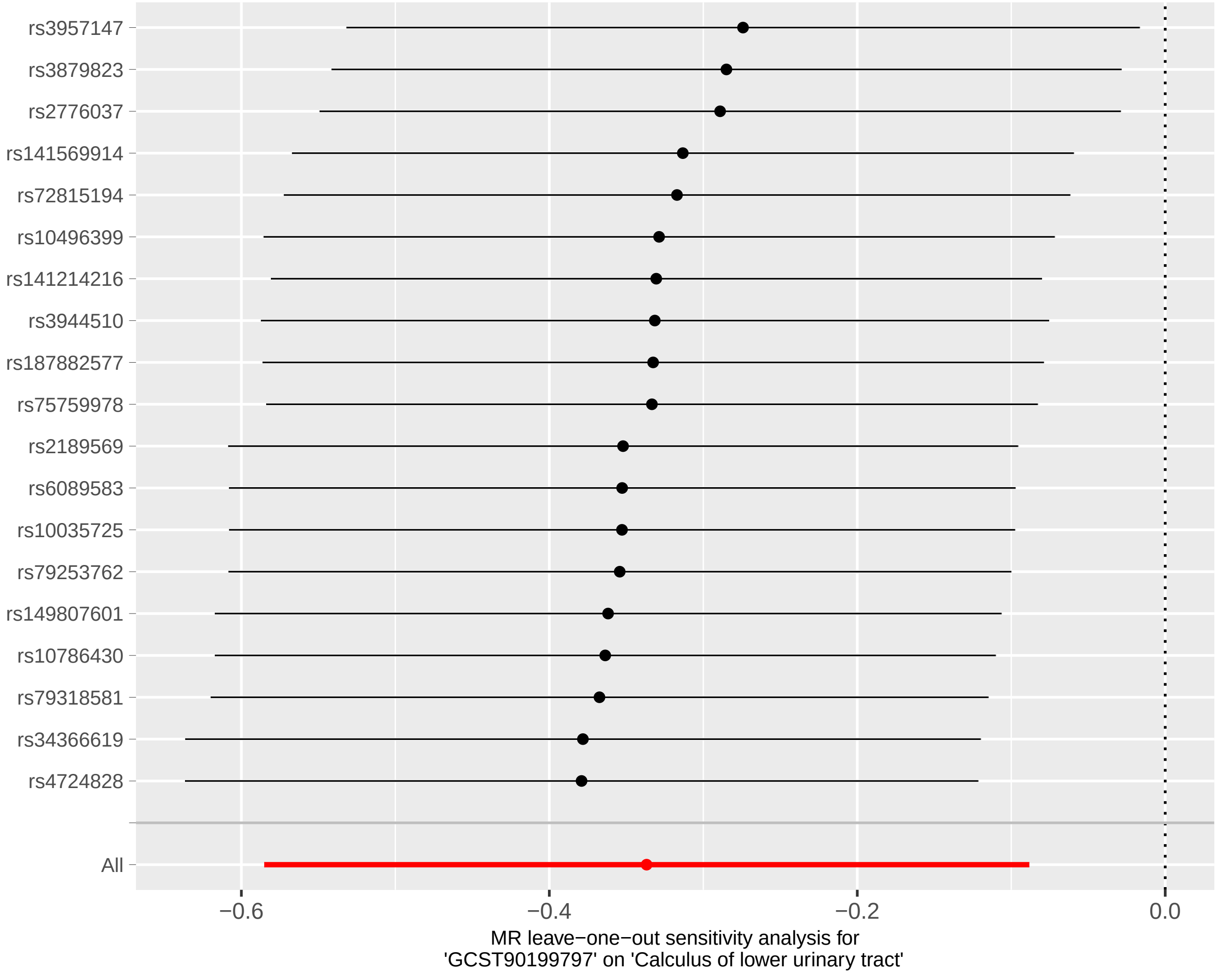
rs3957147
rs3879823
rs2776037
rs141569914
rs72815194
rs10496399
rs141214216
rs3944510
rs187882577
rs75759978
rs2189569
rs6089583
rs10035725
rs79253762
rs149807601
rs10786430
rs79318581
rs34366619
rs4724828
All
−0.6
−0.4
−0.2
0.0
MR leave−one−out sensitivity analysis for
'GCST90199797' on 'Calculus of lower urinary tract'

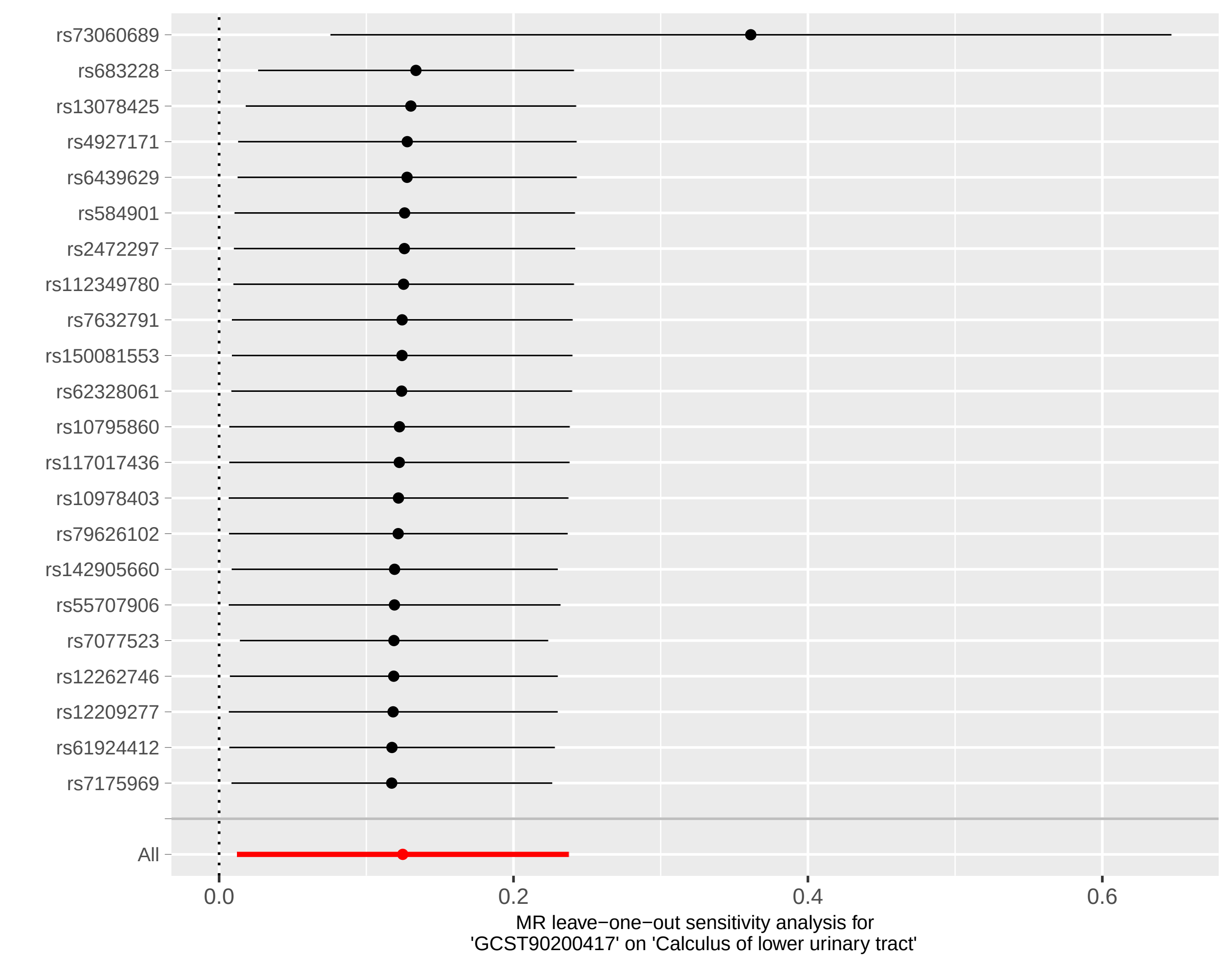
rs73060689
rs683228
rs13078425
rs4927171
rs6439629
rs584901
rs2472297
rs112349780
rs7632791
rs150081553
rs62328061
rs10795860
rs117017436
rs10978403
rs79626102
rs142905660
rs55707906
rs7077523
rs12262746
rs12209277
rs61924412
rs7175969
All
0.0
0.2
0.4
0.6
MR leave−one−out sensitivity analysis for
'GCST90200417' on 'Calculus of lower urinary tract'

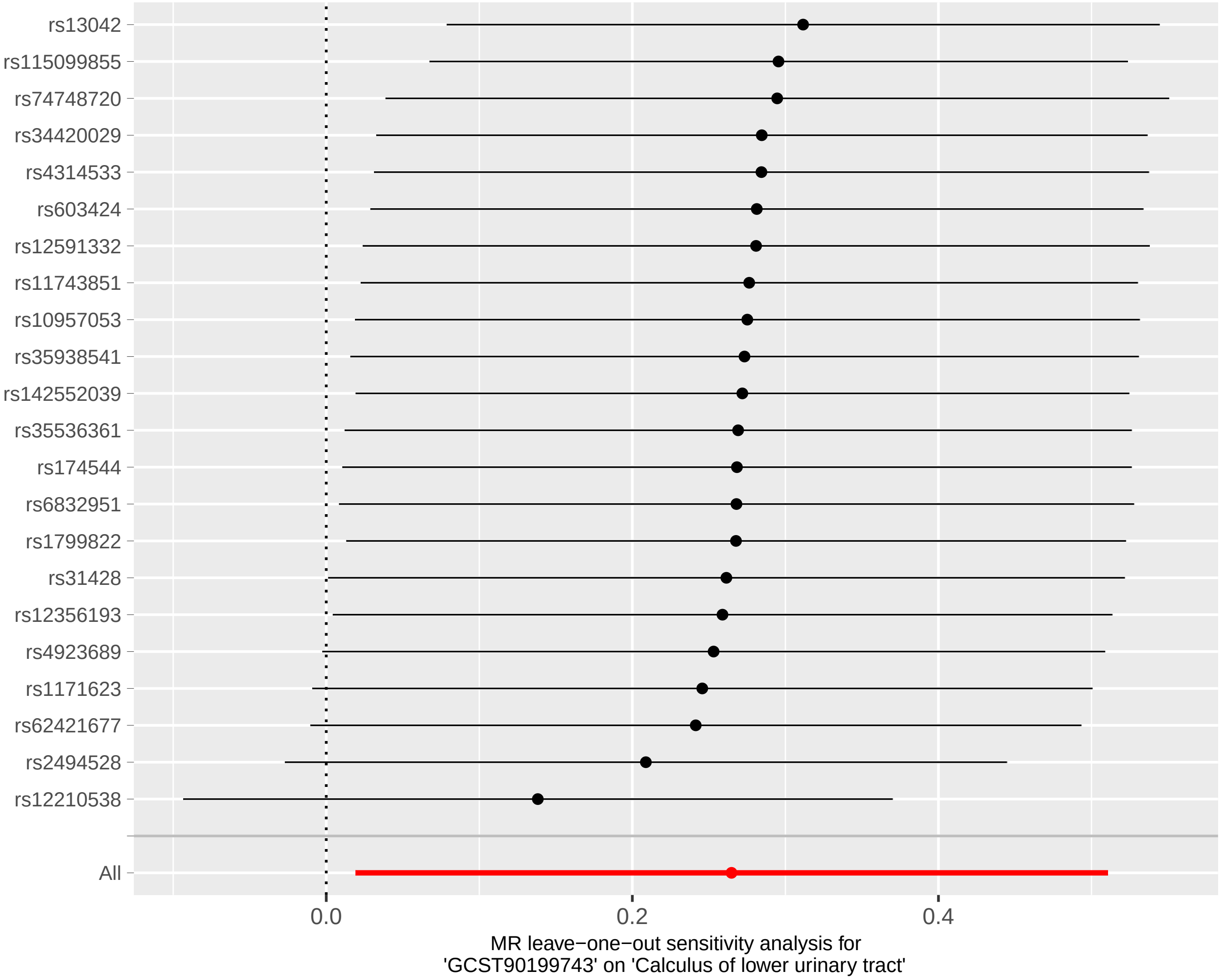
rs13042
rs115099855
rs74748720
rs34420029
rs4314533
rs603424
rs12591332
rs11743851
rs10957053
rs35938541
rs142552039
rs35536361
rs174544
rs6832951
rs1799822
rs31428
rs12356193
rs4923689
rs1171623
rs62421677
rs2494528
rs12210538
All
0.0
0.2
0.4
MR leave−one−out sensitivity analysis for
'GCST90199743' on 'Calculus of lower urinary tract'

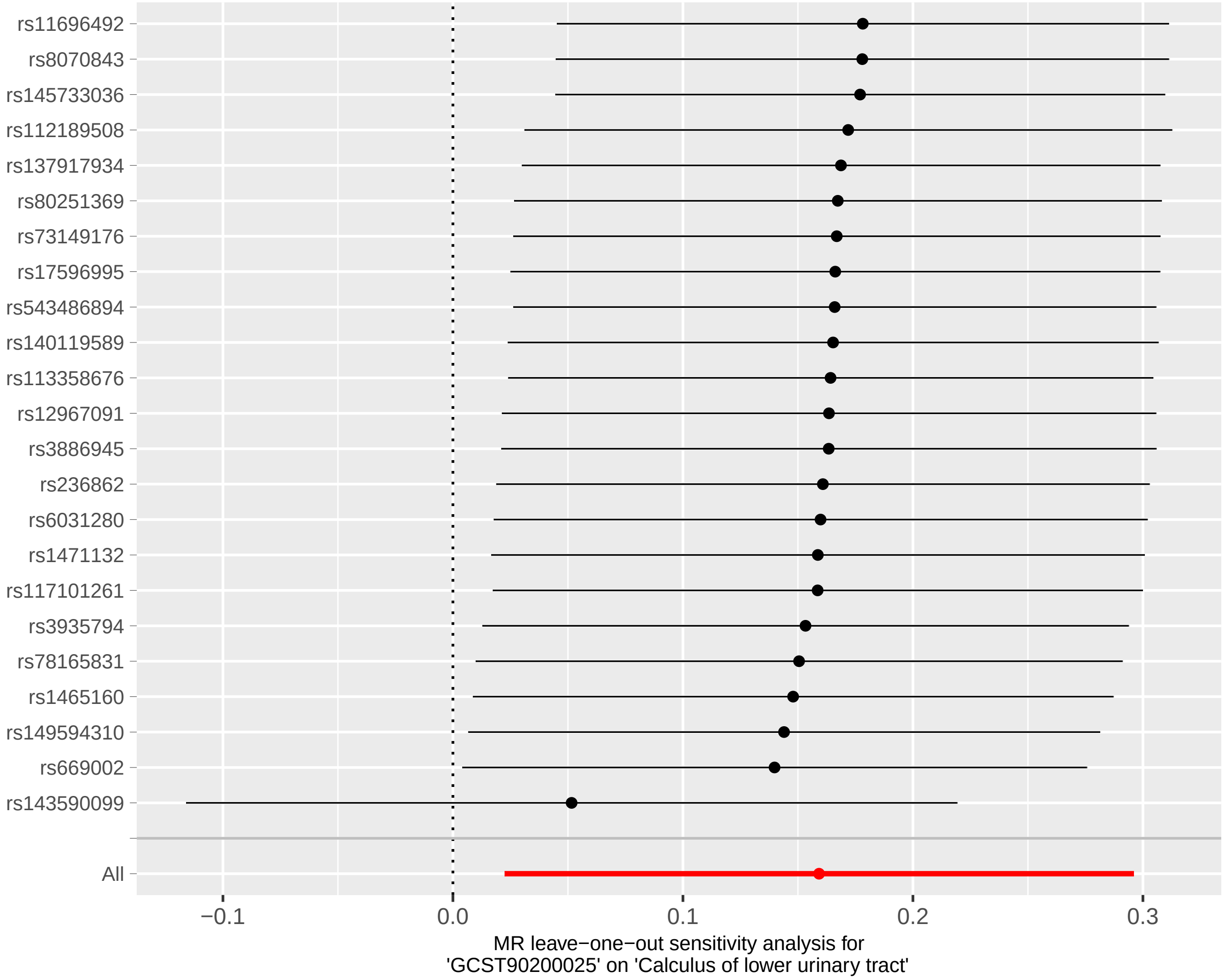

rs11696492
rs8070843
rs145733036
rs112189508
rs137917934
rs80251369
rs73149176
rs17596995
rs543486894
rs140119589
rs113358676
rs12967091
rs3886945
rs236862
rs6031280
rs1471132
rs117101261
rs3935794
rs78165831
rs1465160
rs149594310
rs669002
rs143590099
All
−0.1
0.0
0.1
0.2
0.3
MR leave−one−out sensitivity analysis for
'GCST90200025' on 'Calculus of lower urinary tract'

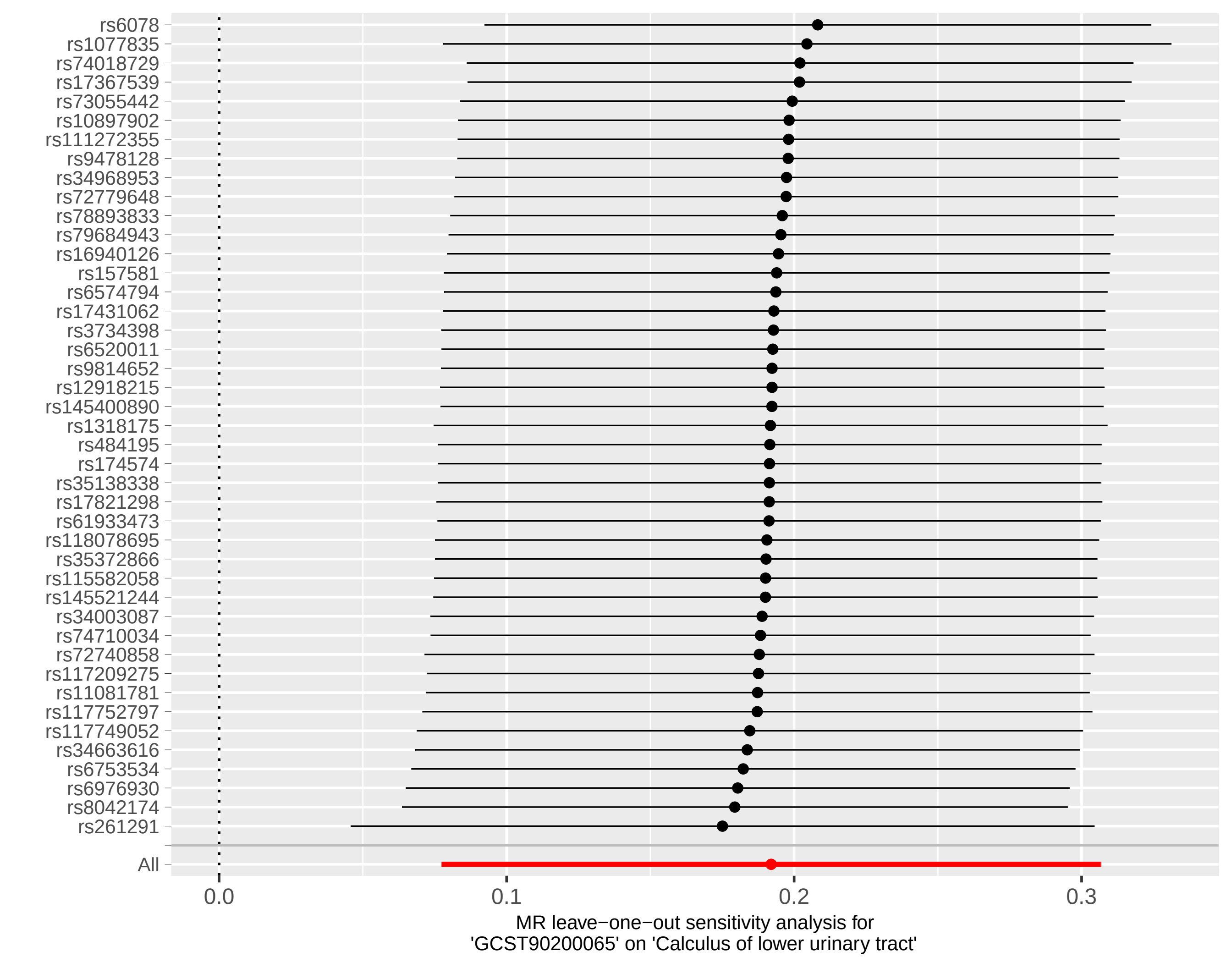
rs6078
rs1077835
rs74018729
rs17367539
rs73055442
rs10897902
rs111272355
rs9478128
rs34968953
rs72779648
rs78893833
rs79684943
rs16940126
rs157581
rs6574794
rs17431062
rs3734398
rs6520011
rs9814652
rs12918215
rs145400890
rs1318175
rs484195
rs174574
rs35138338
rs17821298
rs61933473
rs118078695
rs35372866
rs115582058
rs145521244
rs34003087
rs74710034
rs72740858
rs117209275
rs11081781
rs117752797
rs117749052
rs34663616
rs6753534
rs6976930
rs8042174
rs261291
All
0.0
0.1
0.2
0.3
MR leave−one−out sensitivity analysis for
'GCST90200065' on 'Calculus of lower urinary tract'

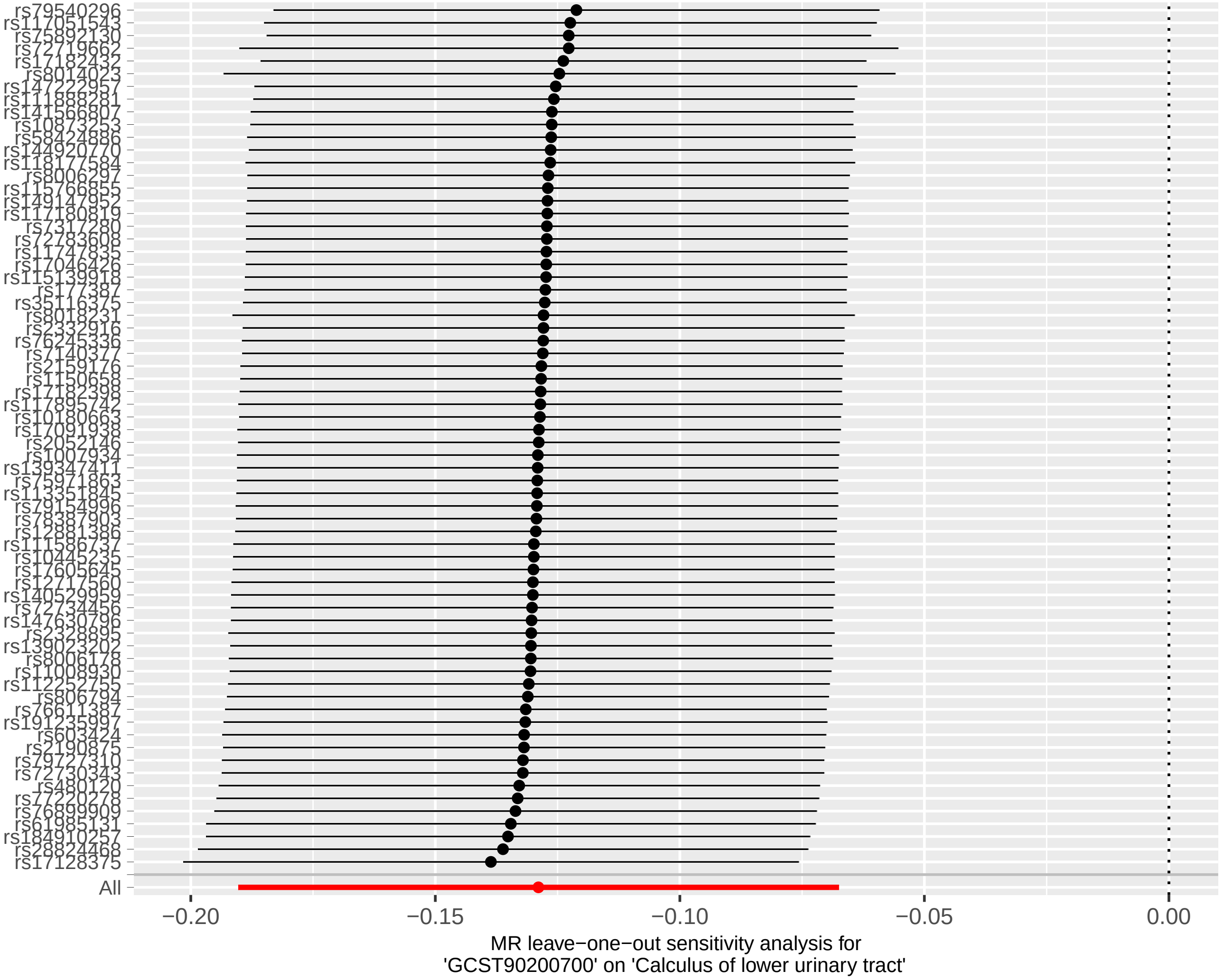

rs79540296
rs117051543
rs75892130
rs72719662
rs17182432
rs8014023
rs147222957
rs111888281
rs141566807
rs10873253
rs58424886
rs144920770
rs118177584
rs8006297
rs115766855
rs149147952
rs117180819
rs7317280
rs72783608
rs11747835
rs17046426
rs115139918
rs177387
rs35116375
rs8018231
rs2332916
rs76245336
rs7140377
rs2159176
rs1150658
rs17182398
rs117895742
rs10180663
rs17091938
rs2052146
rs1007934
rs139347411
rs75971863
rs113351845
rs79154996
rs78387903
rs12881386
rs111586737
rs10445235
rs17605645
rs12717560
rs140529959
rs72734456
rs147630796
rs2328895
rs139023202
rs8006178
rs11008930
rs112252755
rs806794
rs76611387
rs191235997
rs603424
rs2190875
rs79727310
rs72730343
rs480120
rs77220278
rs76899909
rs61985131
rs184910257
rs28824468
rs17128375
All
−0.20
−0.15
−0.10
−0.05
0.00
MR leave−one−out sensitivity analysis for
'GCST90200700' on 'Calculus of lower urinary tract'

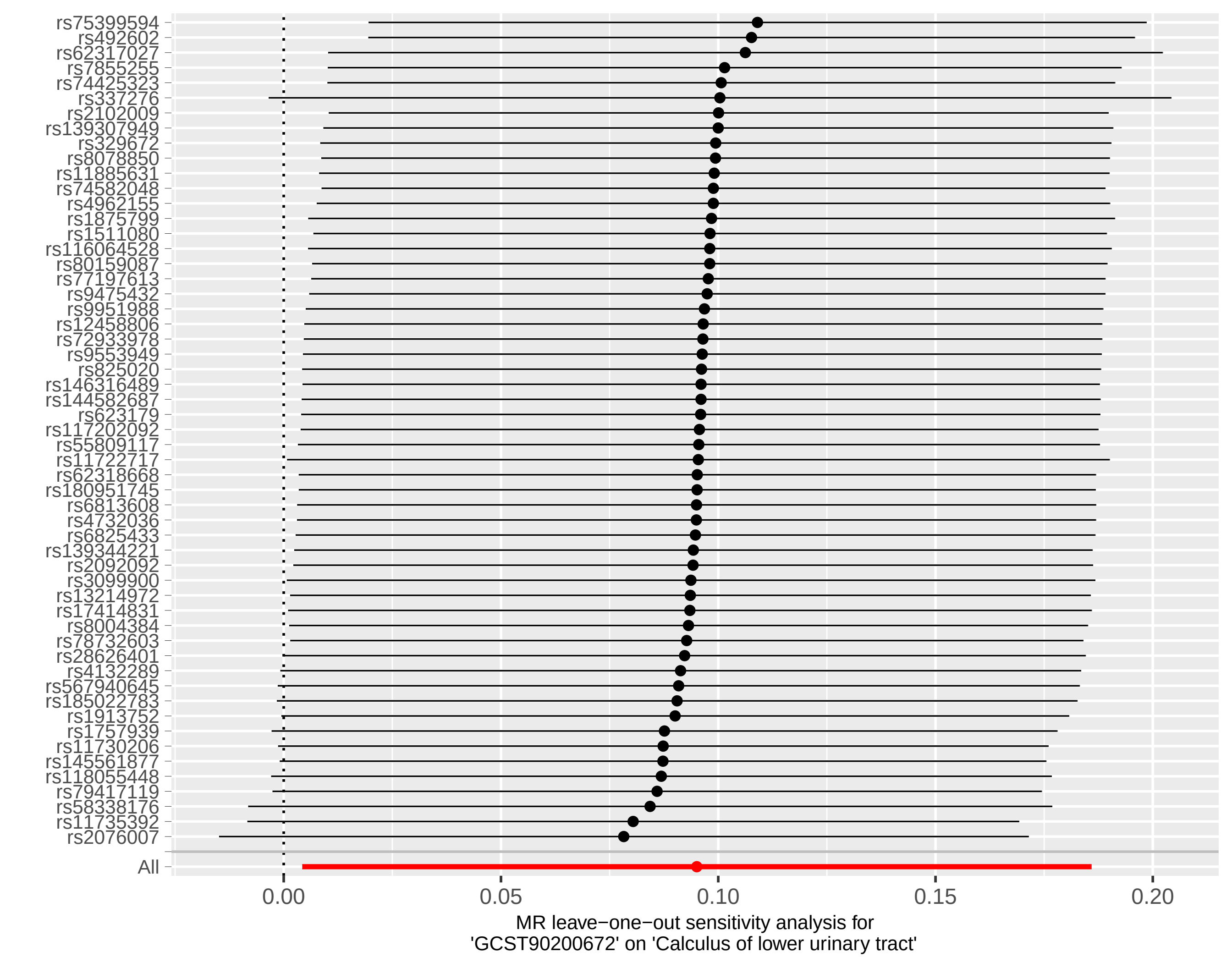

rs75399594
rs492602
rs62317027
rs7855255
rs74425323
rs337276
rs2102009
rs139307949
rs329672
rs8078850
rs11885631
rs74582048
rs4962155
rs1875799
rs1511080
rs116064528
rs80159087
rs77197613
rs9475432
rs9951988
rs12458806
rs72933978
rs9553949
rs825020
rs146316489
rs144582687
rs623179
rs117202092
rs55809117
rs11722717
rs62318668
rs180951745
rs6813608
rs4732036
rs6825433
rs139344221
rs2092092
rs3099900
rs13214972
rs17414831
rs8004384
rs78732603
rs28626401
rs4132289
rs567940645
rs185022783
rs1913752
rs1757939
rs11730206
rs145561877
rs118055448
rs79417119
rs58338176
rs11735392
rs2076007
All
0.00
0.05
0.10
0.15
0.20
MR leave−one−out sensitivity analysis for
'GCST90200672' on 'Calculus of lower urinary tract'

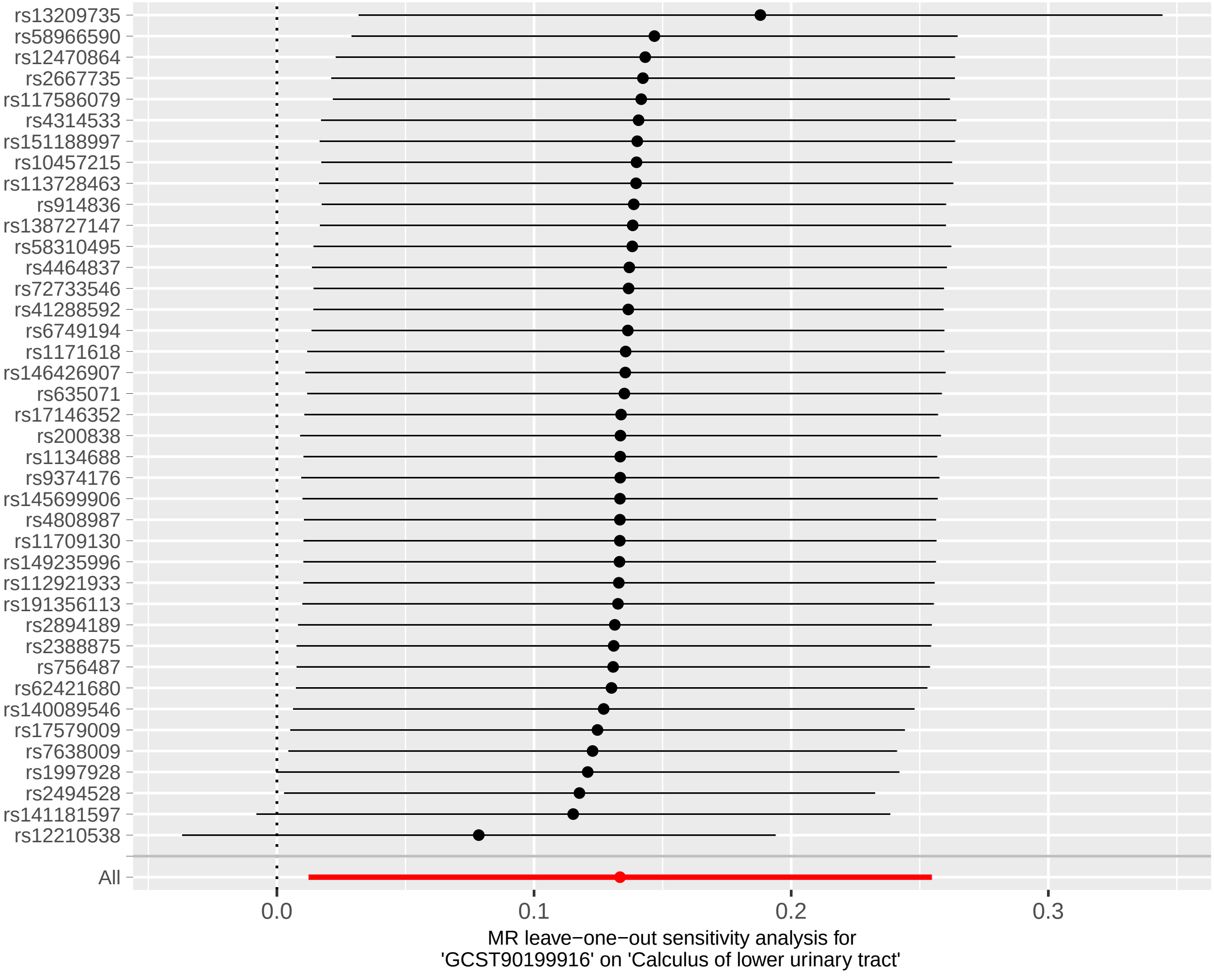

rs13209735
rs58966590
rs12470864
rs2667735
rs117586079
rs4314533
rs151188997
rs10457215
rs113728463
rs914836
rs138727147
rs58310495
rs4464837
rs72733546
rs41288592
rs6749194
rs1171618
rs146426907
rs635071
rs17146352
rs200838
rs1134688
rs9374176
rs145699906
rs4808987
rs11709130
rs149235996
rs112921933
rs191356113
rs2894189
rs2388875
rs756487
rs62421680
rs140089546
rs17579009
rs7638009
rs1997928
rs2494528
rs141181597
rs12210538
All
0.0
0.1
0.2
0.3
MR leave−one−out sensitivity analysis for
'GCST90199916' on 'Calculus of lower urinary tract'

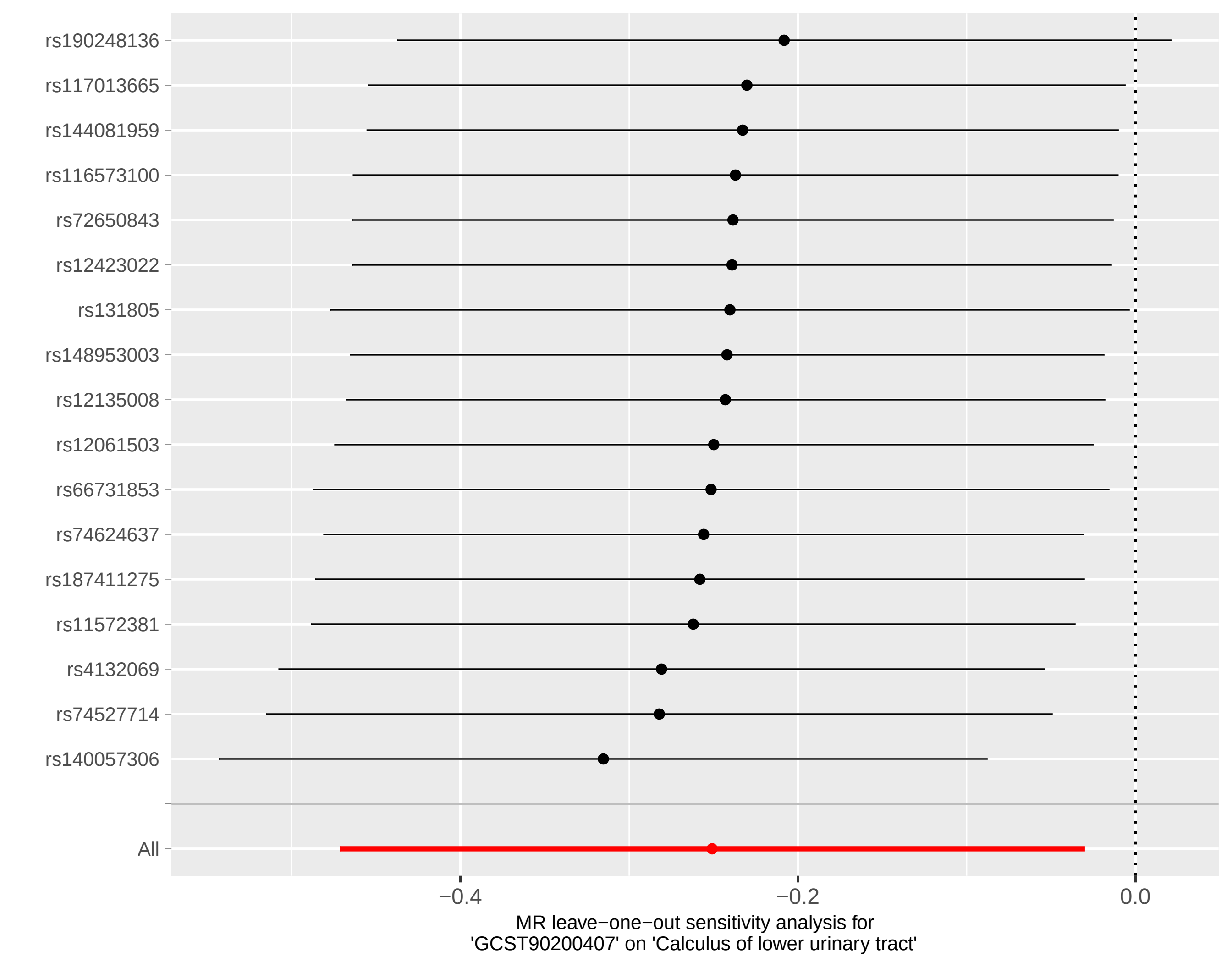
rs190248136
rs117013665
rs144081959
rs116573100
rs72650843
rs12423022
rs131805
rs148953003
rs12135008
rs12061503
rs66731853
rs74624637
rs187411275
rs11572381
rs4132069
rs74527714
rs140057306
All
−0.4
−0.2
0.0
MR leave−one−out sensitivity analysis for
'GCST90200407' on 'Calculus of lower urinary tract'

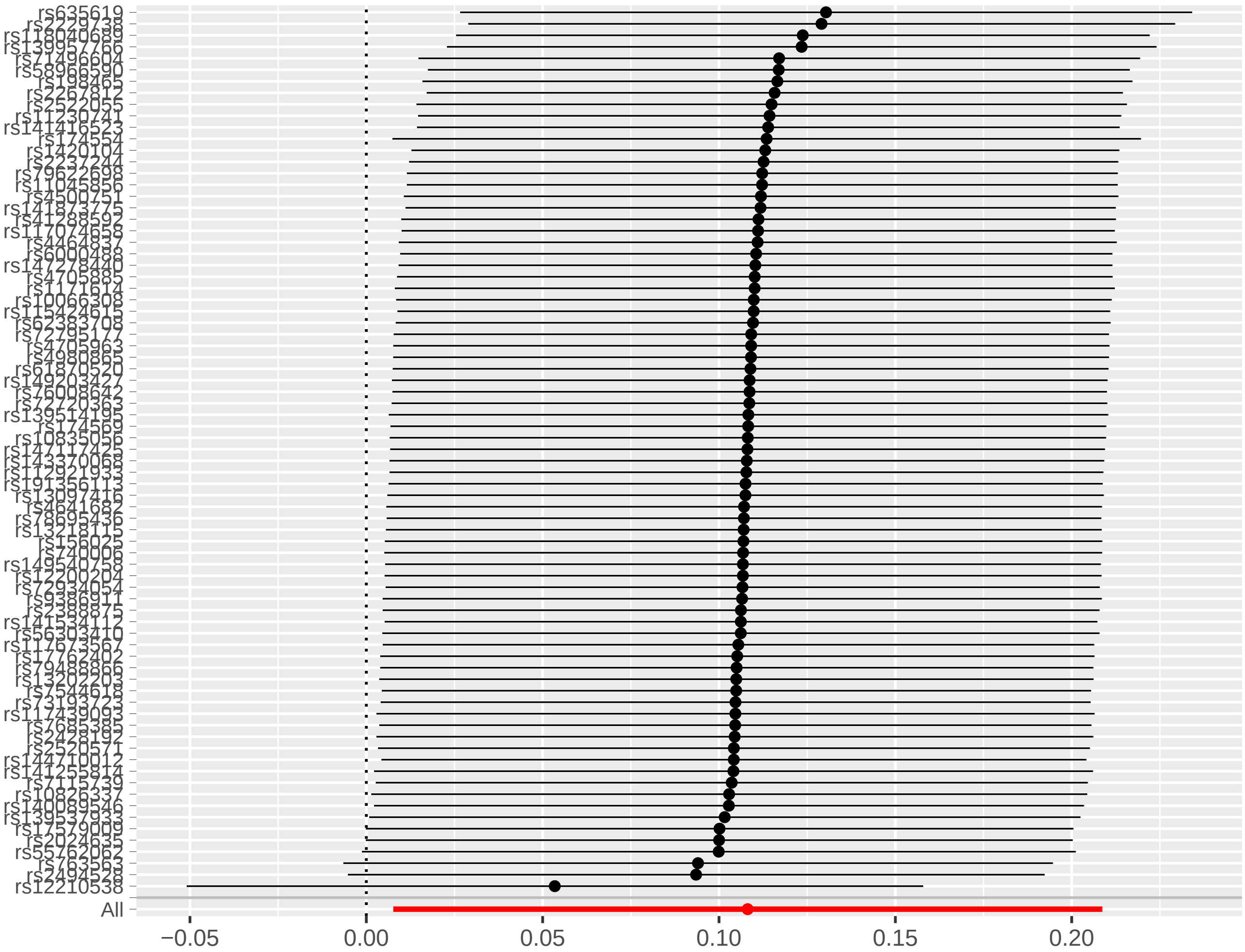
rs635619
rs12210538
All
−0.05
0.00
0.05
0.10
0.15
0.20
MR leave−one−out sensitivity analysis for
'GCST90200150' on 'Calculus of lower urinary tract'

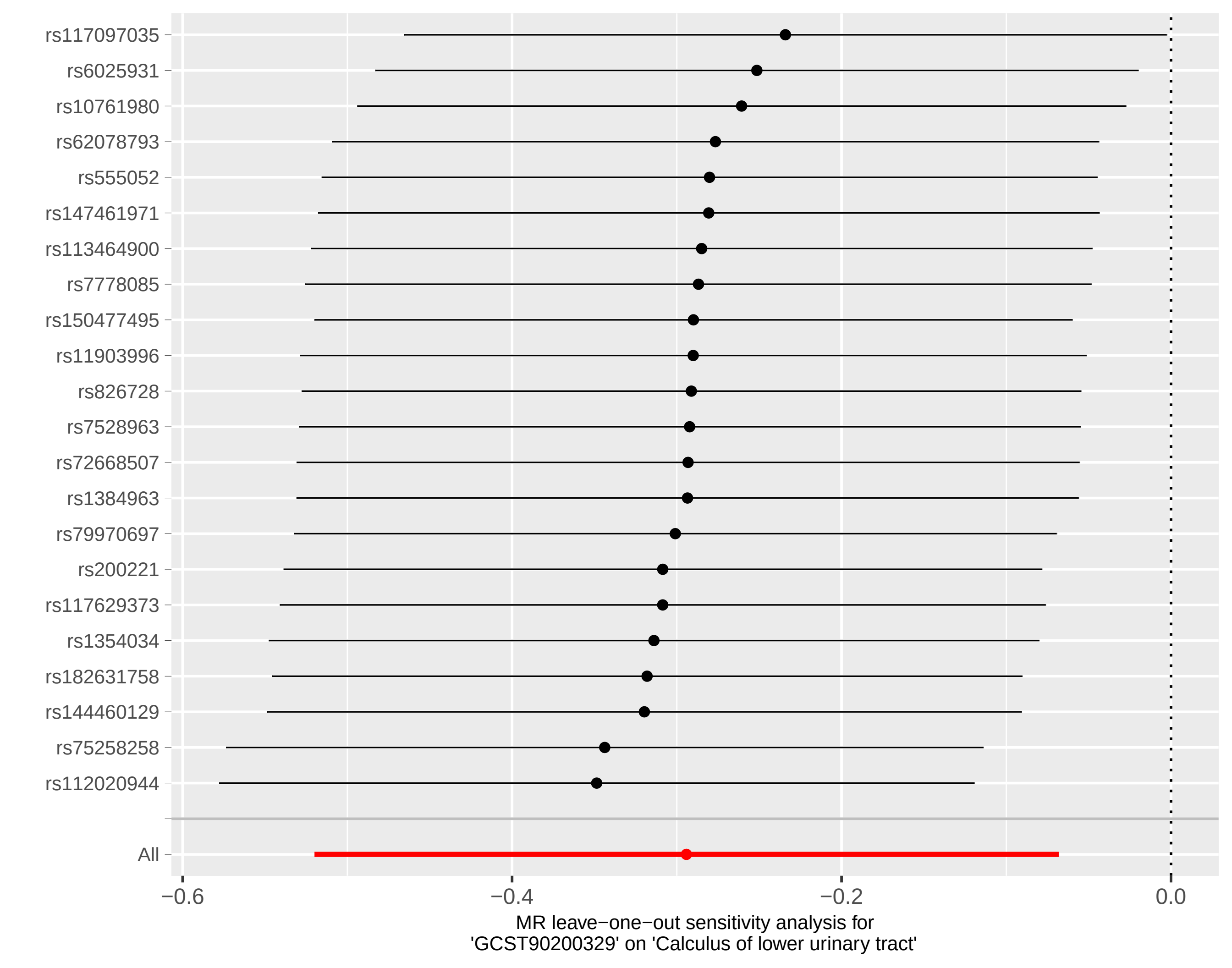

rs117097035
rs6025931
rs10761980
rs62078793
rs555052
rs147461971
rs113464900
rs7778085
rs150477495
rs11903996
rs826728
rs7528963
rs72668507
rs1384963
rs79970697
rs200221
rs117629373
rs1354034
rs182631758
rs144460129
rs75258258
rs112020944
All
−0.6
−0.4
−0.2
0.0
MR leave−one−out sensitivity analysis for
'GCST90200329' on 'Calculus of lower urinary tract'

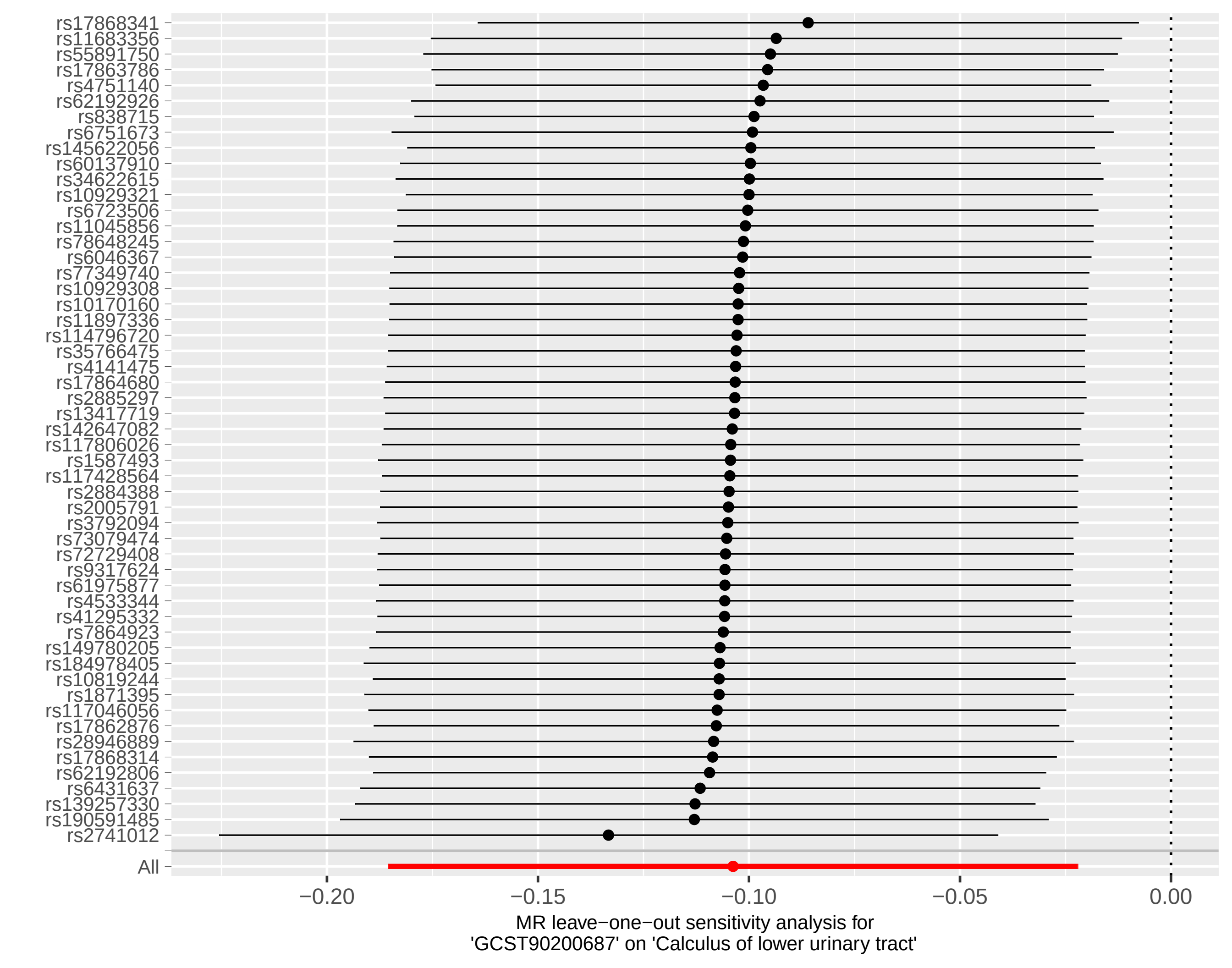
rs17868341
rs11683356
rs55891750
rs17863786
rs4751140
rs62192926
rs838715
rs6751673
rs145622056
rs60137910
rs34622615
rs10929321
rs6723506
rs11045856
rs78648245
rs6046367
rs77349740
rs10929308
rs10170160
rs11897336
rs114796720
rs35766475
rs4141475
rs17864680
rs2885297
rs13417719
rs142647082
rs117806026
rs1587493
rs117428564
rs2884388
rs2005791
rs3792094
rs73079474
rs72729408
rs9317624
rs61975877
rs4533344
rs41295332
rs7864923
rs149780205
rs184978405
rs10819244
rs1871395
rs117046056
rs17862876
rs28946889
rs17868314
rs62192806
rs6431637
rs139257330
rs190591485
rs2741012
All
−0.20
−0.15
−0.10
−0.05
0.00
MR leave−one−out sensitivity analysis for
'GCST90200687' on 'Calculus of lower urinary tract'

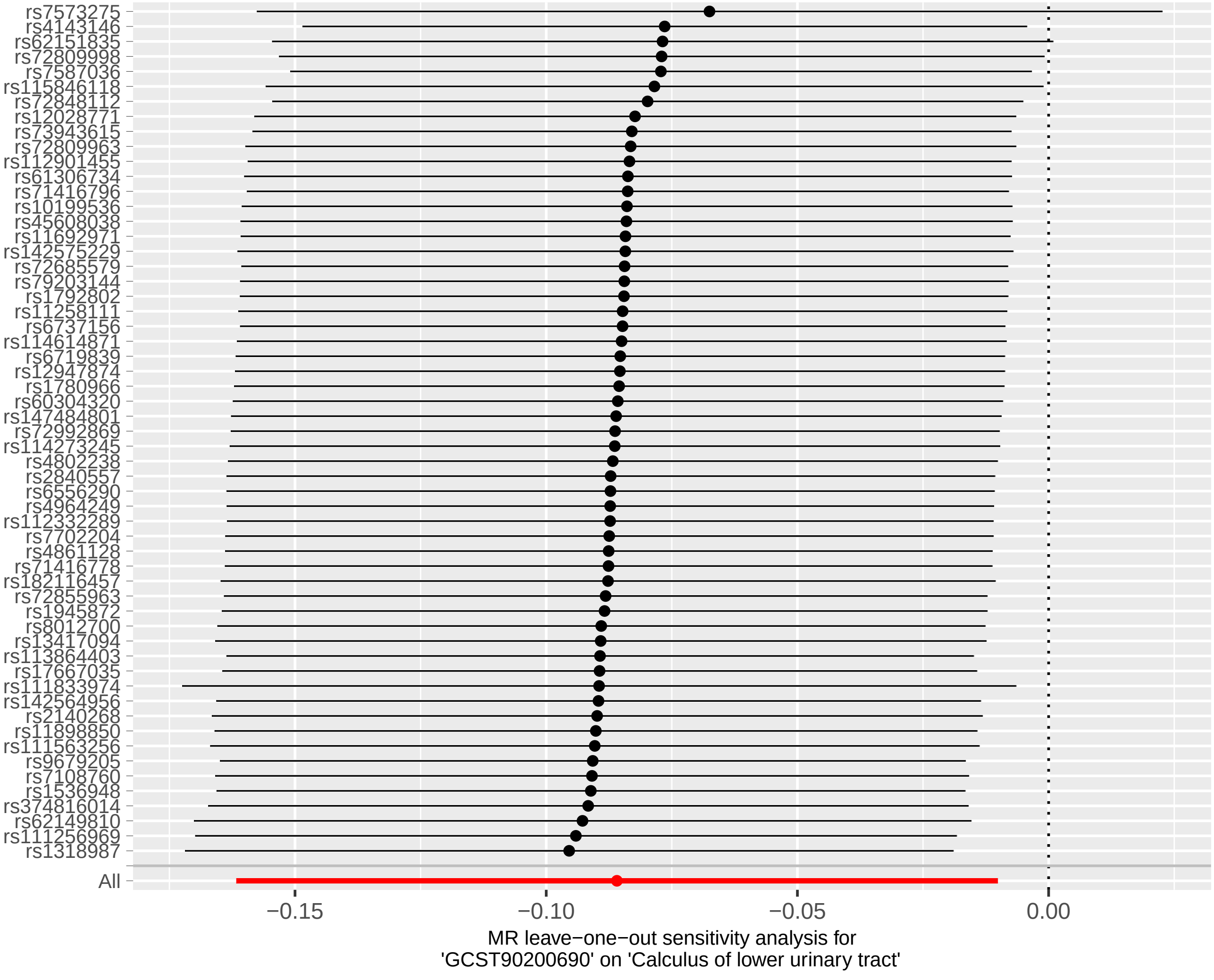
rs7573275
rs4143146
rs62151835
rs72809998
rs7587036
rs115846118
rs72848112
rs12028771
rs73943615
rs72809963
rs112901455
rs61306734
rs71416796
rs10199536
rs45608038
rs11692971
rs142575229
rs72685579
rs79203144
rs1792802
rs11258111
rs6737156
rs114614871
rs6719839
rs12947874
rs1780966
rs60304320
rs147484801
rs72992869
rs114273245
rs4802238
rs2840557
rs6556290
rs4964249
rs112332289
rs7702204
rs4861128
rs71416778
rs182116457
rs72855963
rs1945872
rs8012700
rs13417094
rs113864403
rs17667035
rs111833974
rs142564956
rs2140268
rs11898850
rs111563256
rs9679205
rs7108760
rs1536948
rs374816014
rs62149810
rs111256969
rs1318987
All
−0.15
−0.10
−0.05
0.00
MR leave−one−out sensitivity analysis for
'GCST90200690' on 'Calculus of lower urinary tract'

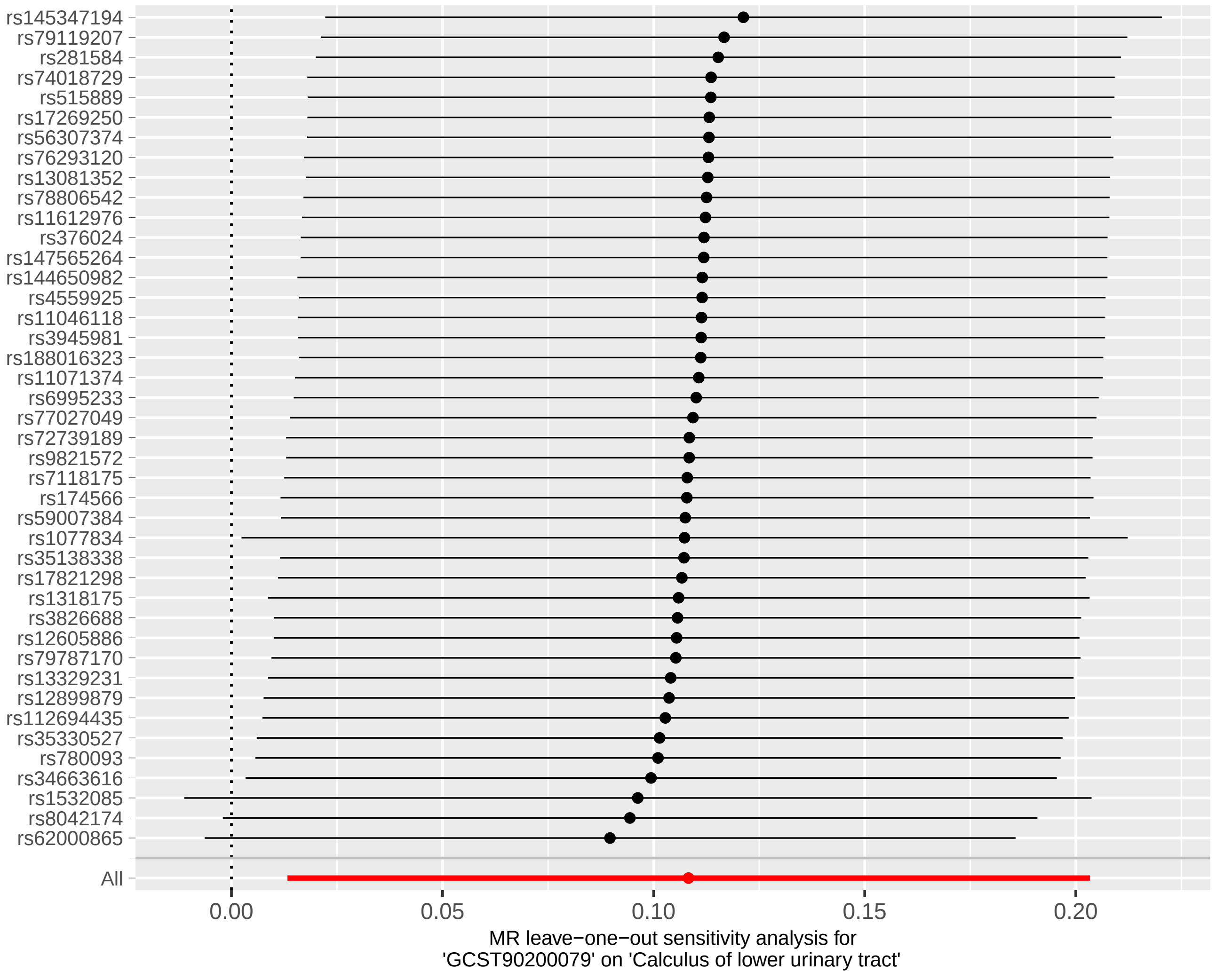
rs145347194
rs79119207
rs281584
rs74018729
rs515889
rs17269250
rs56307374
rs76293120
rs13081352
rs78806542
rs11612976
rs376024
rs147565264
rs144650982
rs4559925
rs11046118
rs3945981
rs188016323
rs11071374
rs6995233
rs77027049
rs72739189
rs9821572
rs7118175
rs174566
rs59007384
rs1077834
rs35138338
rs17821298
rs1318175
rs3826688
rs12605886
rs79787170
rs13329231
rs12899879
rs112694435
rs35330527
rs780093
rs34663616
rs1532085
rs8042174
rs62000865
All
0.00
0.05
0.10
0.15
0.20
MR leave−one−out sensitivity analysis for
'GCST90200079' on 'Calculus of lower urinary tract'

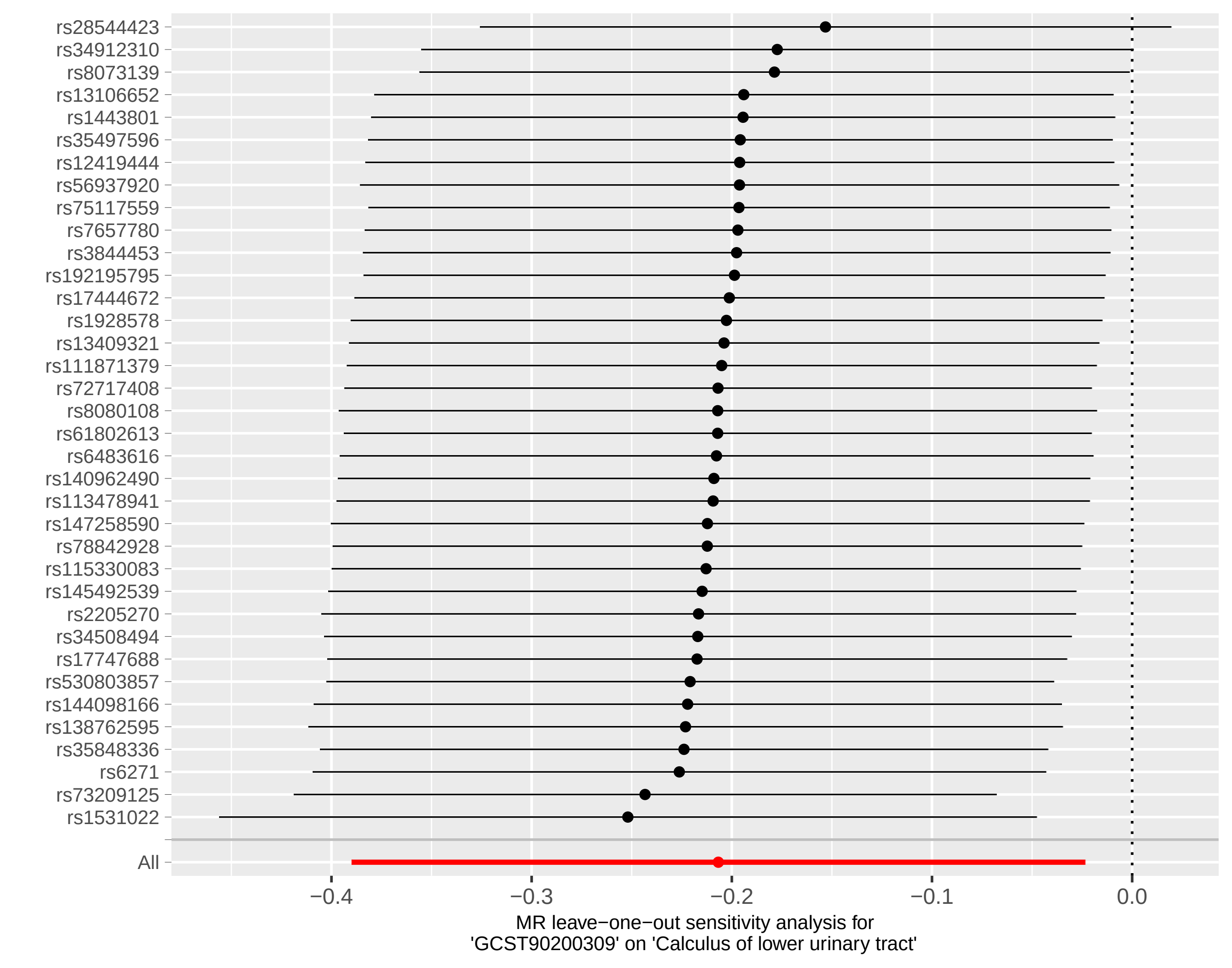
rs28544423
rs34912310
rs8073139
rs13106652
rs1443801
rs35497596
rs12419444
rs56937920
rs75117559
rs7657780
rs3844453
rs192195795
rs17444672
rs1928578
rs13409321
rs111871379
rs72717408
rs8080108
rs61802613
rs6483616
rs140962490
rs113478941
rs147258590
rs78842928
rs115330083
rs145492539
rs2205270
rs34508494
rs17747688
rs530803857
rs144098166
rs138762595
rs35848336
rs6271
rs73209125
rs1531022
All
−0.4
−0.3
−0.2
−0.1
0.0
MR leave−one−out sensitivity analysis for
'GCST90200309' on 'Calculus of lower urinary tract'

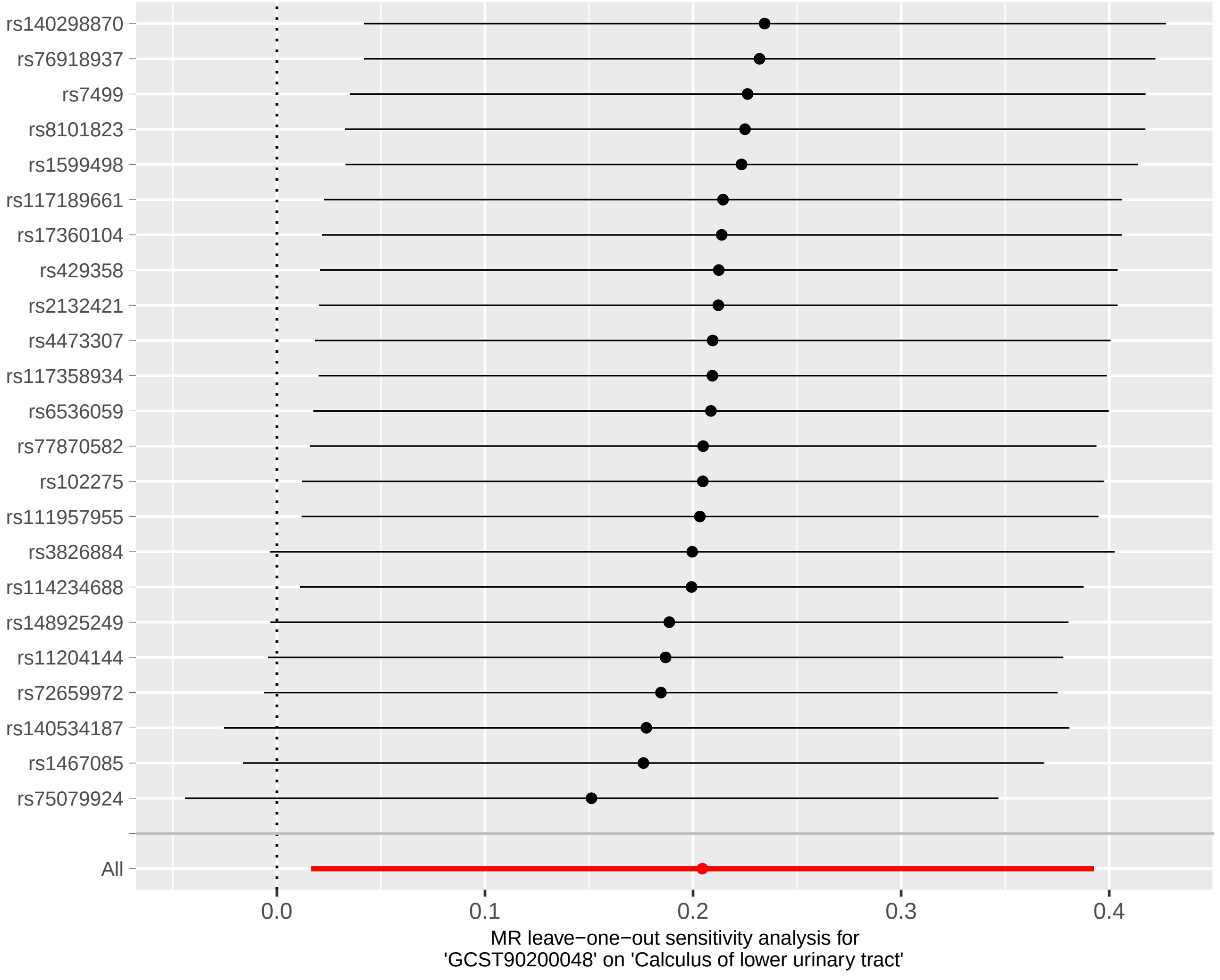
rs140298870
rs76918937
rs7499
rs8101823
rs1599498
rs117189661
rs17360104
rs429358
rs2132421
rs4473307
rs117358934
rs6536059
rs77870582
rs102275
rs111957955
rs3826884
rs114234688
rs148925249
rs11204144
rs72659972
rs140534187
rs1467085
rs75079924
All
0.0
0.1
0.2
0.3
0.4
MR leave−one−out sensitivity analysis for
'GCST90200048' on 'Calculus of lower urinary tract'

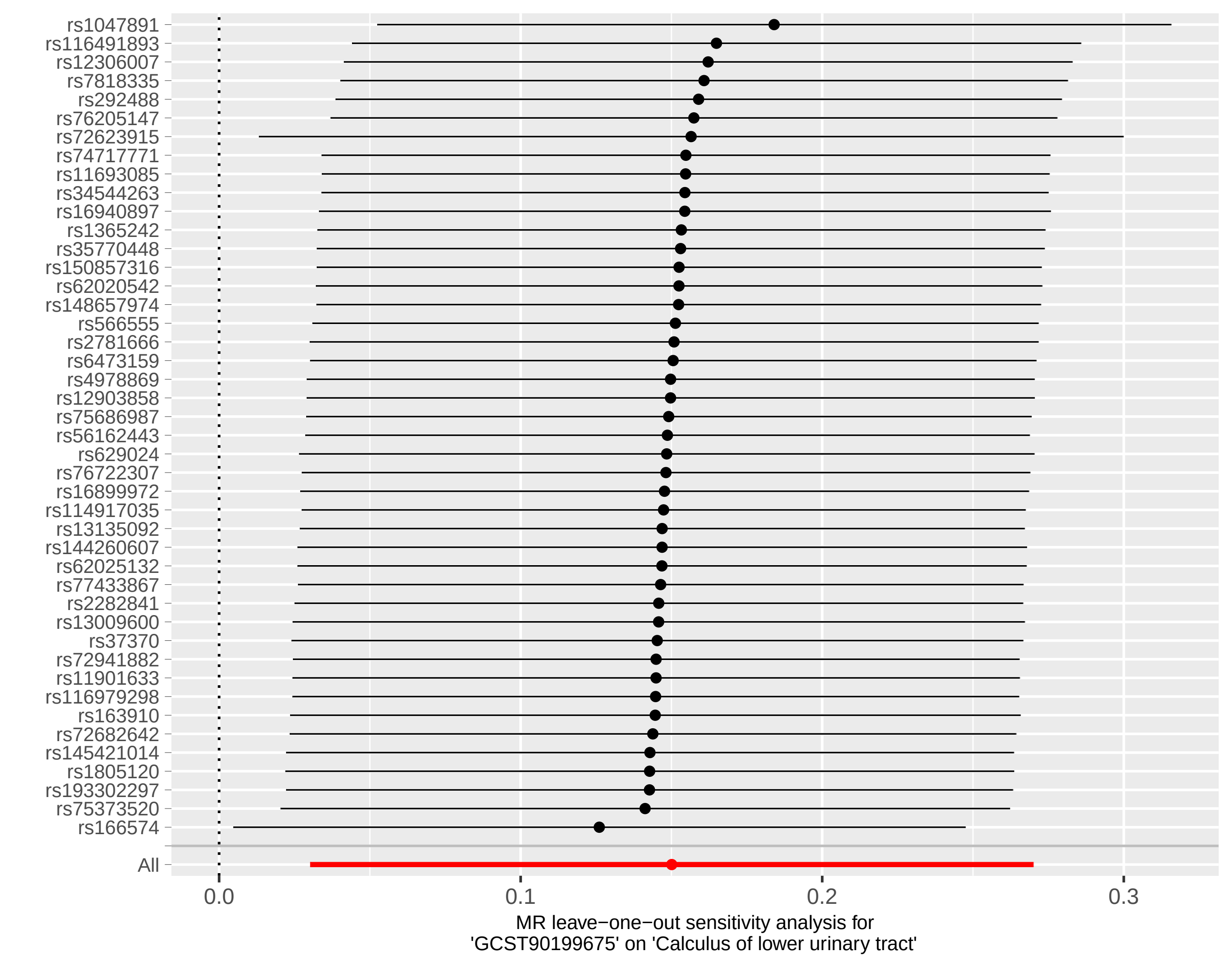

rs1047891
rs116491893
rs12306007
rs7818335
rs292488
rs76205147
rs72623915
rs74717771
rs11693085
rs34544263
rs16940897
rs1365242
rs35770448
rs150857316
rs62020542
rs148657974
rs566555
rs2781666
rs6473159
rs4978869
rs12903858
rs75686987
rs56162443
rs629024
rs76722307
rs16899972
rs114917035
rs13135092
rs144260607
rs62025132
rs77433867
rs2282841
rs13009600
rs37370
rs72941882
rs11901633
rs116979298
rs163910
rs72682642
rs145421014
rs1805120
rs193302297
rs75373520
rs166574
All
0.0
0.1
0.2
0.3
MR leave−one−out sensitivity analysis for
'GCST90199675' on 'Calculus of lower urinary tract'

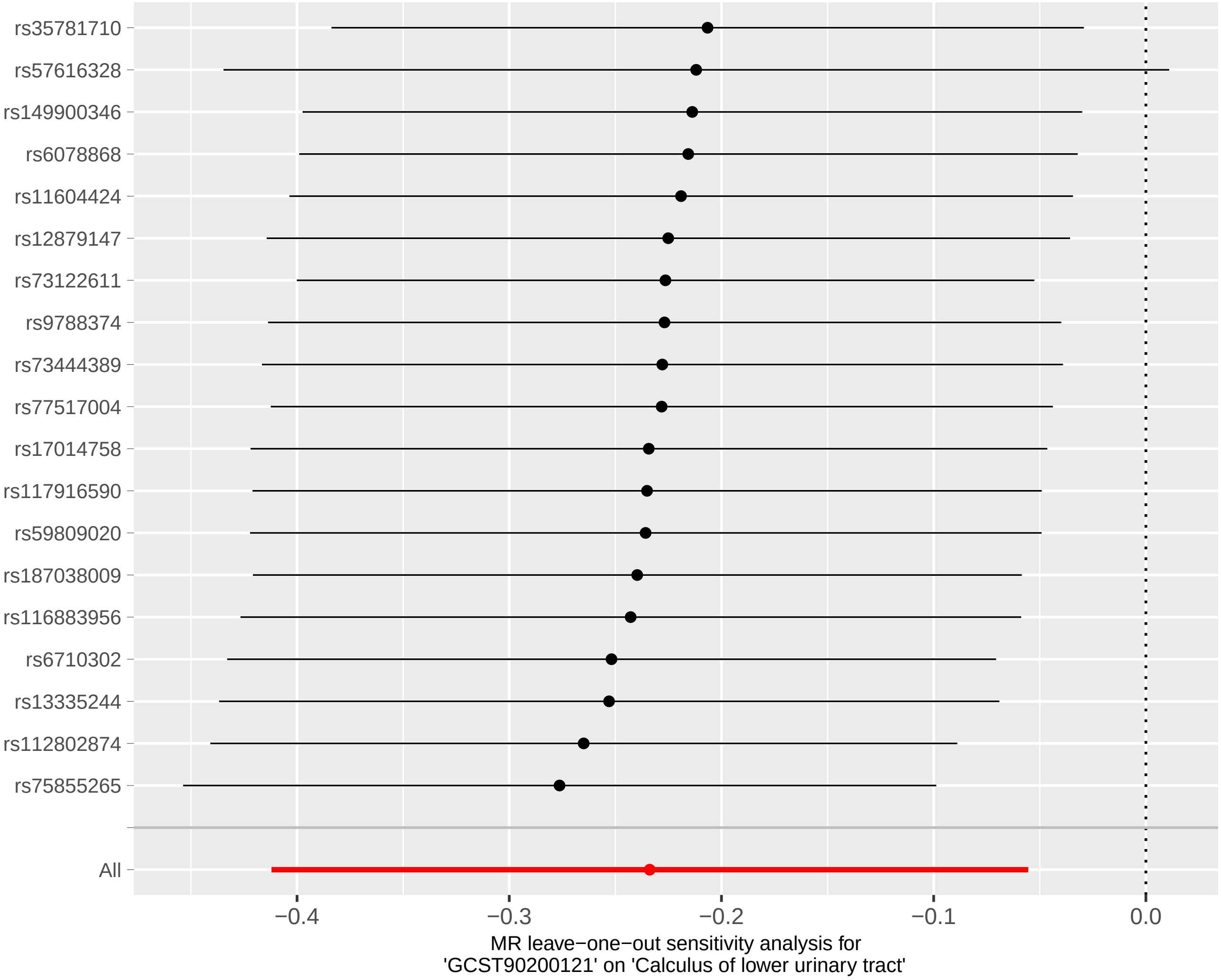
rs35781710
rs57616328
rs149900346
rs6078868
rs11604424
rs12879147
rs73122611
rs9788374
rs73444389
rs77517004
rs17014758
rs117916590
rs59809020
rs187038009
rs116883956
rs6710302
rs13335244
rs112802874
rs75855265
All
−0.4
−0.3
−0.2
−0.1
0.0
MR leave−one−out sensitivity analysis for
'GCST90200121' on 'Calculus of lower urinary tract'

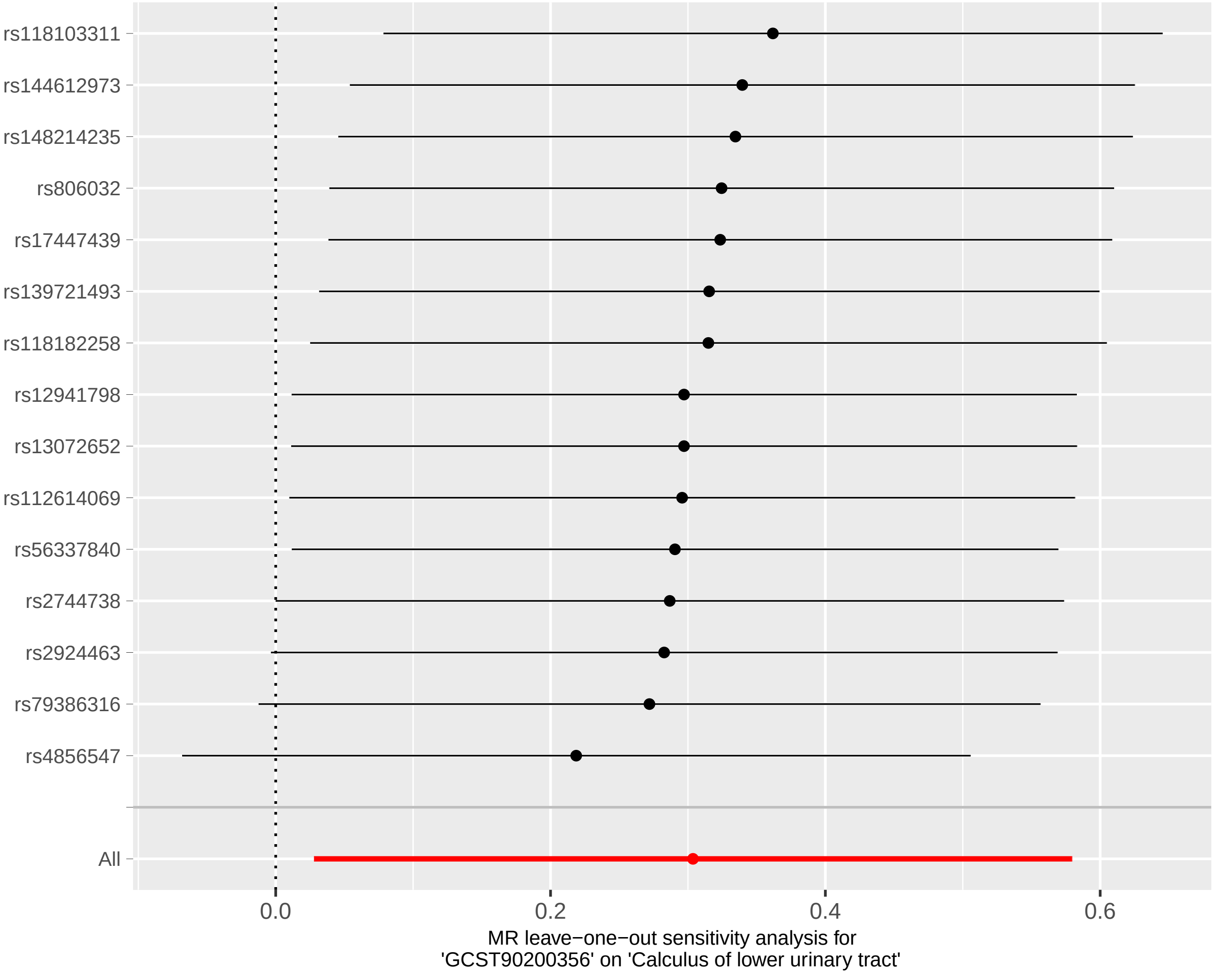
rs118103311
rs144612973
rs148214235
rs806032
rs17447439
rs139721493
rs118182258
rs12941798
rs13072652
rs112614069
rs56337840
rs2744738
rs2924463
rs79386316
rs4856547
All
0.0
0.2
0.4
0.6
MR leave−one−out sensitivity analysis for
'GCST90200356' on 'Calculus of lower urinary tract'

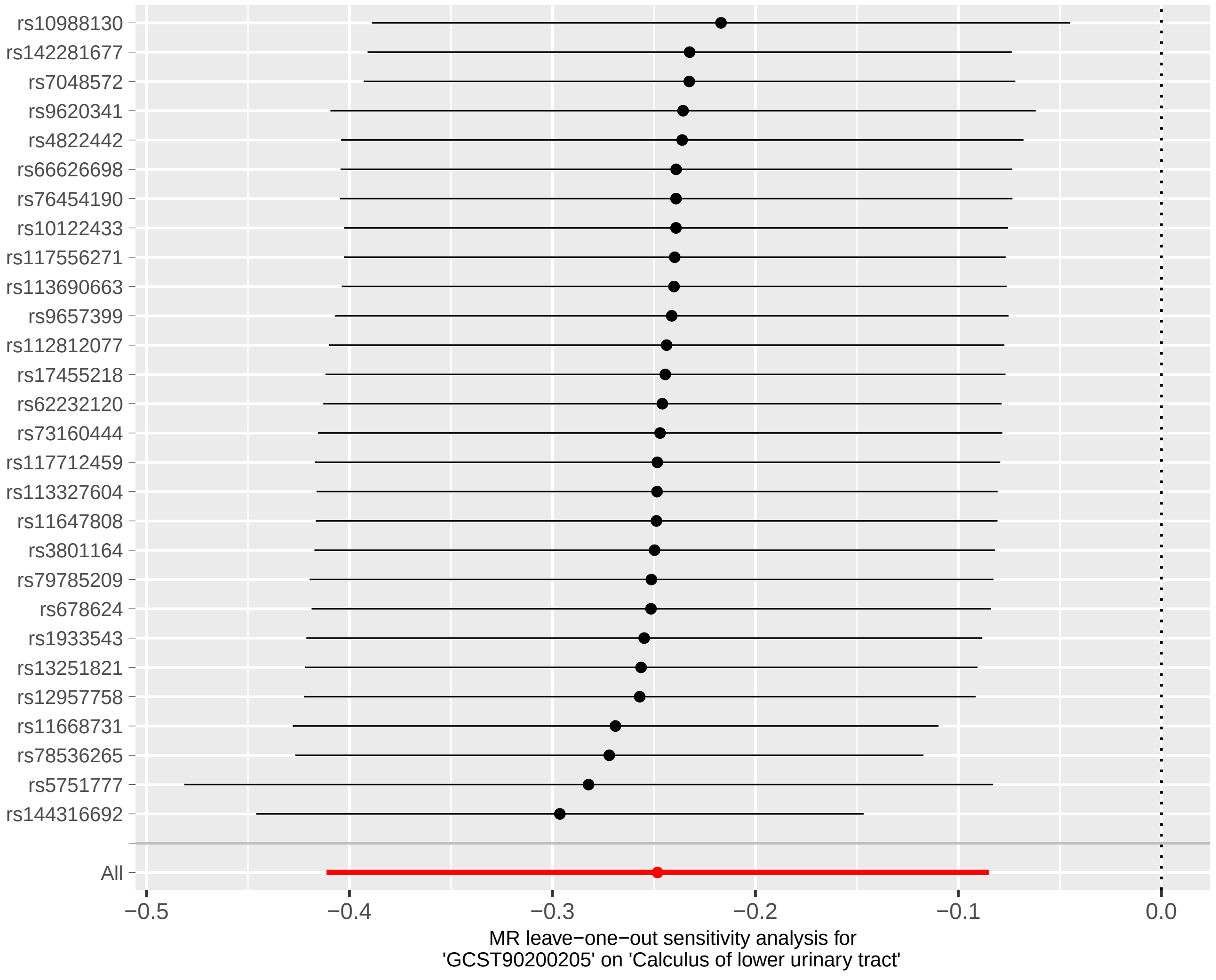
rs10988130
rs142281677
rs7048572
rs9620341
rs4822442
rs66626698
rs76454190
rs10122433
rs117556271
rs113690663
rs9657399
rs112812077
rs17455218
rs62232120
rs73160444
rs117712459
rs113327604
rs11647808
rs3801164
rs79785209
rs678624
rs1933543
rs13251821
rs12957758
rs11668731
rs78536265
rs5751777
rs144316692
All
−0.5
−0.4
−0.3
−0.2
−0.1
0.0
MR leave−one−out sensitivity analysis for
'GCST90200205' on 'Calculus of lower urinary tract'

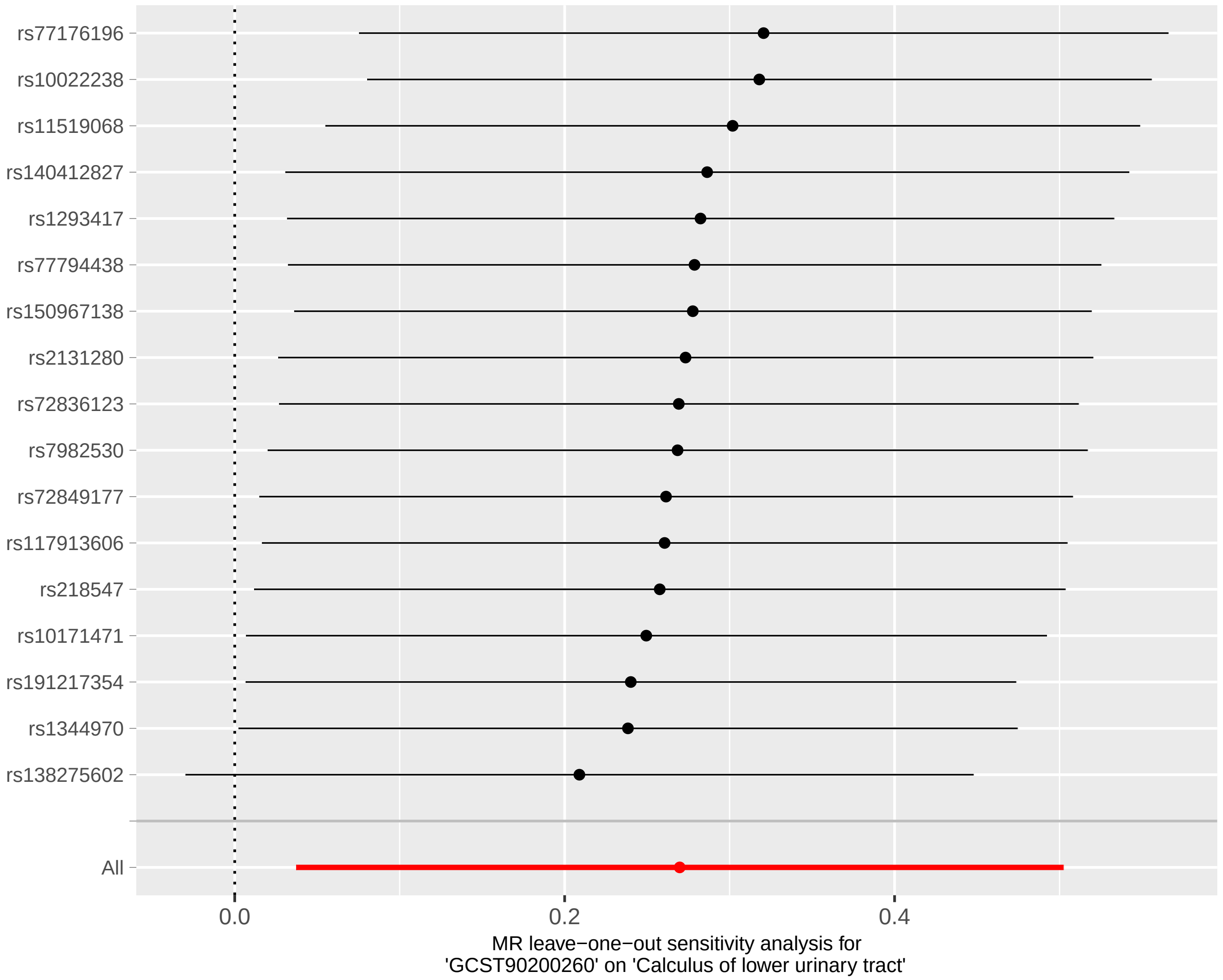

rs77176196
rs10022238
rs11519068
rs140412827
rs1293417
rs77794438
rs150967138
rs2131280
rs72836123
rs7982530
rs72849177
rs117913606
rs218547
rs10171471
rs191217354
rs1344970
rs138275602
All
0.0
0.2
0.4
MR leave−one−out sensitivity analysis for
'GCST90200260' on 'Calculus of lower urinary tract'

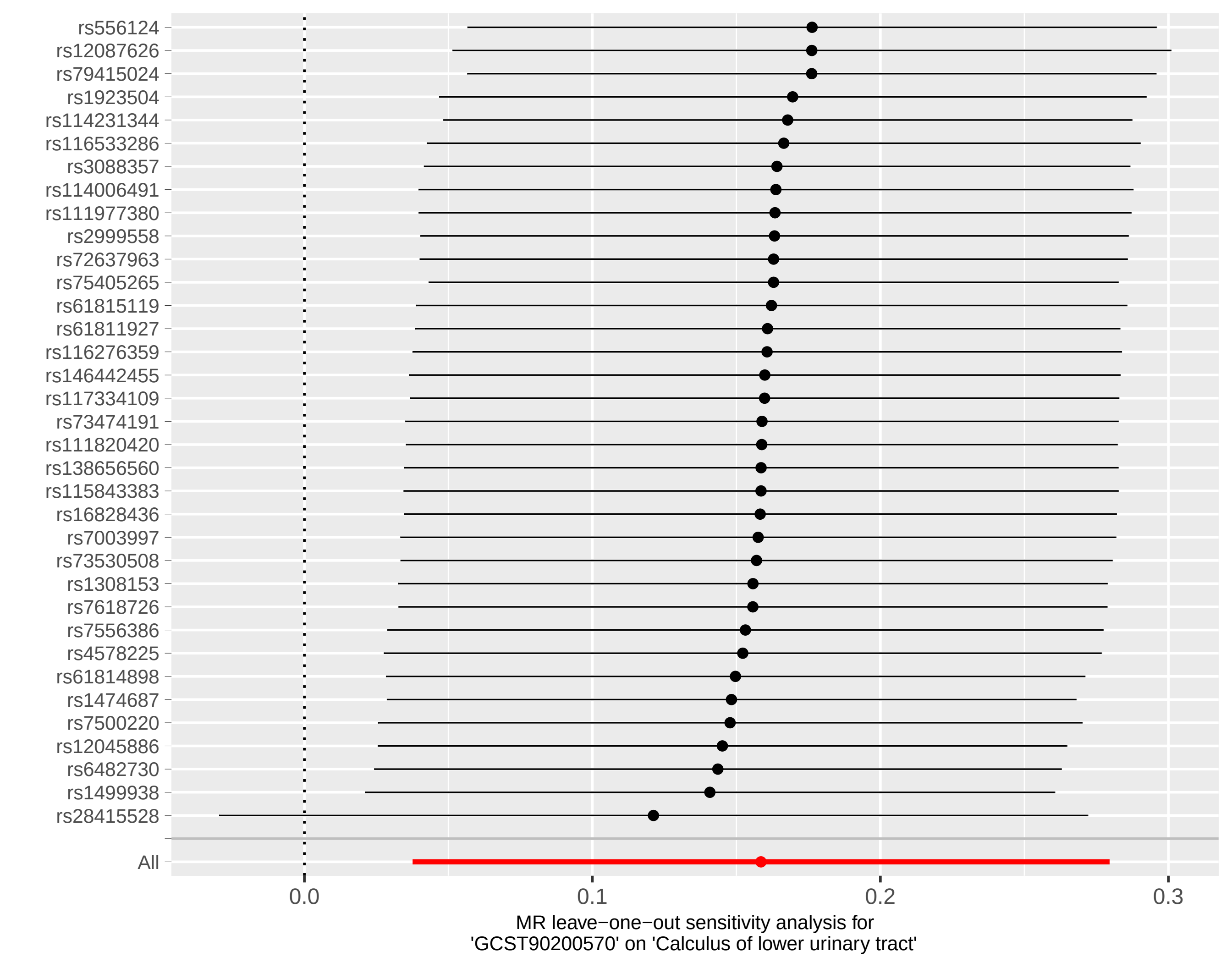
rs556124
rs12087626
rs79415024
rs1923504
rs114231344
rs116533286
rs3088357
rs114006491
rs111977380
rs2999558
rs72637963
rs75405265
rs61815119
rs61811927
rs116276359
rs146442455
rs117334109
rs73474191
rs111820420
rs138656560
rs115843383
rs16828436
rs7003997
rs73530508
rs1308153
rs7618726
rs7556386
rs4578225
rs61814898
rs1474687
rs7500220
rs12045886
rs6482730
rs1499938
rs28415528
All
0.0
0.1
0.2
0.3
MR leave−one−out sensitivity analysis for
'GCST90200570' on 'Calculus of lower urinary tract'

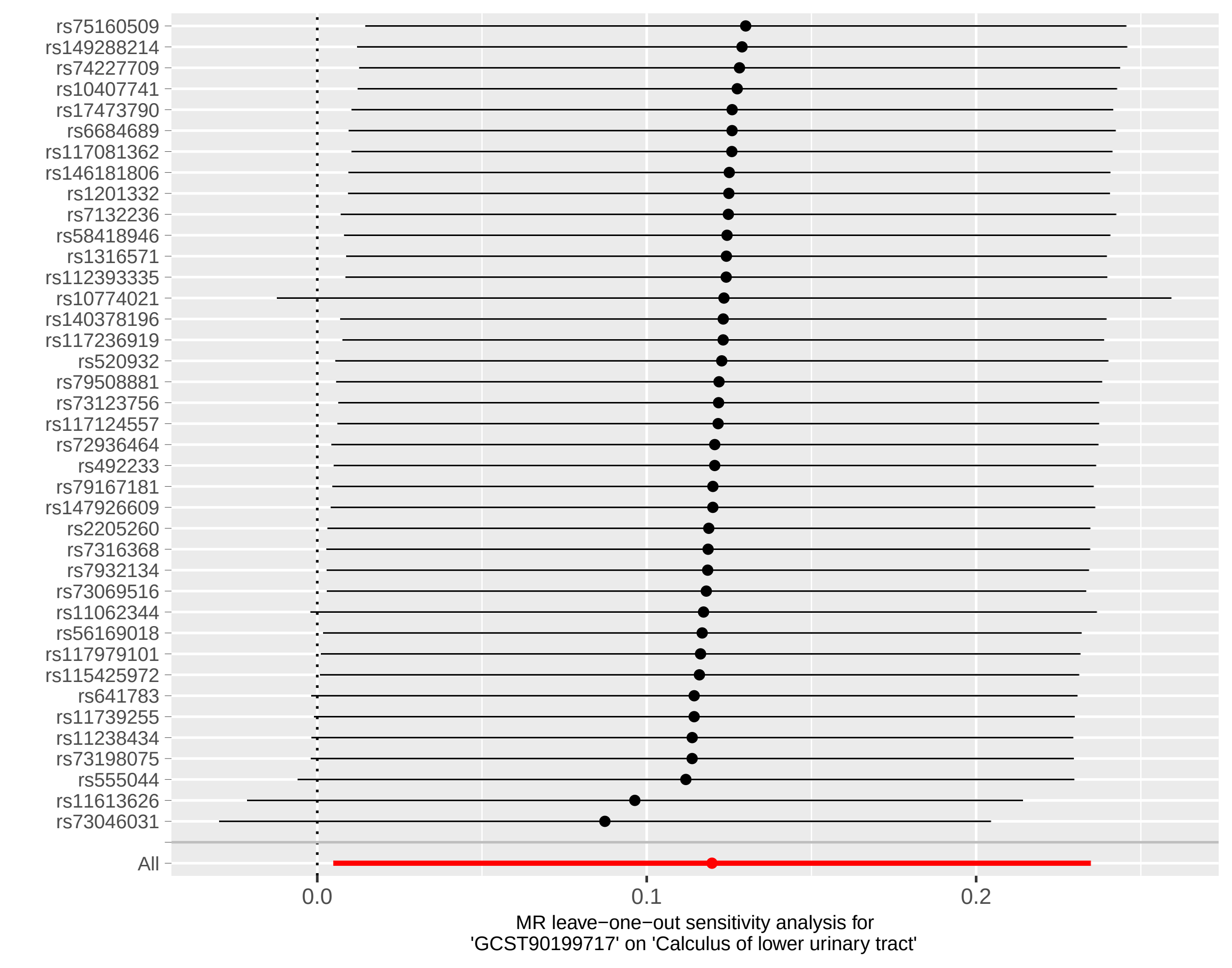
rs75160509
rs149288214
rs74227709
rs10407741
rs17473790
rs6684689
rs117081362
rs146181806
rs1201332
rs7132236
rs58418946
rs1316571
rs112393335
rs10774021
rs140378196
rs117236919
rs520932
rs79508881
rs73123756
rs117124557
rs72936464
rs492233
rs79167181
rs147926609
rs2205260
rs7316368
rs7932134
rs73069516
rs11062344
rs56169018
rs117979101
rs115425972
rs641783
rs11739255
rs11238434
rs73198075
rs555044
rs11613626
rs73046031
All
0.0
0.1
0.2
MR leave−one−out sensitivity analysis for
'GCST90199717' on 'Calculus of lower urinary tract'

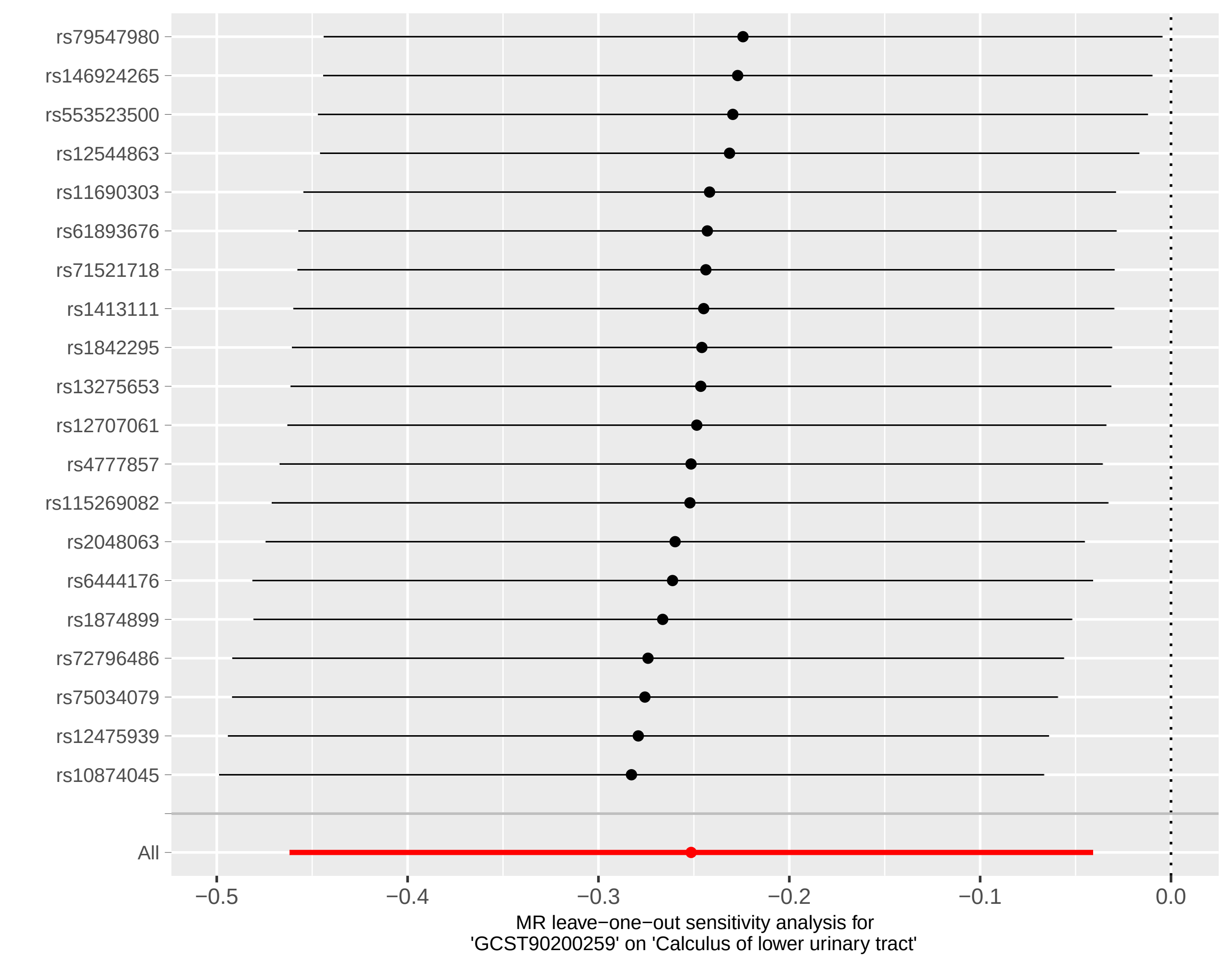
rs79547980
rs146924265
rs553523500
rs12544863
rs11690303
rs61893676
rs71521718
rs1413111
rs1842295
rs13275653
rs12707061
rs4777857
rs115269082
rs2048063
rs6444176
rs1874899
rs72796486
rs75034079
rs12475939
rs10874045
All
−0.5
−0.4
−0.3
−0.2
−0.1
0.0
MR leave−one−out sensitivity analysis for
'GCST90200259' on 'Calculus of lower urinary tract'

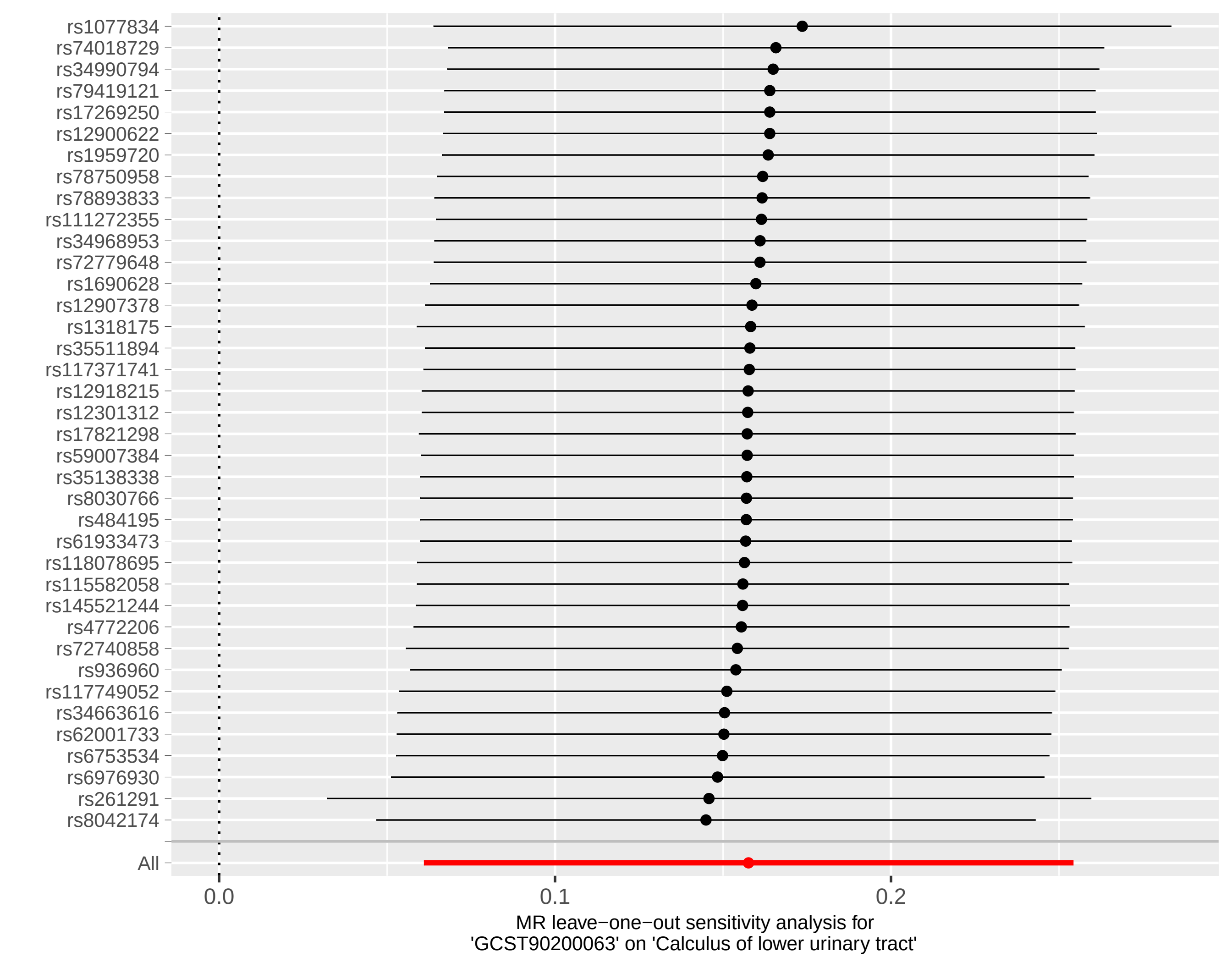
rs1077834
rs74018729
rs34990794
rs79419121
rs17269250
rs12900622
rs1959720
rs78750958
rs78893833
rs111272355
rs34968953
rs72779648
rs1690628
rs12907378
rs1318175
rs35511894
rs117371741
rs12918215
rs12301312
rs17821298
rs59007384
rs35138338
rs8030766
rs484195
rs61933473
rs118078695
rs115582058
rs145521244
rs4772206
rs72740858
rs936960
rs117749052
rs34663616
rs62001733
rs6753534
rs6976930
rs261291
rs8042174
All
0.0
0.1
0.2
MR leave−one−out sensitivity analysis for
'GCST90200063' on 'Calculus of lower urinary tract'

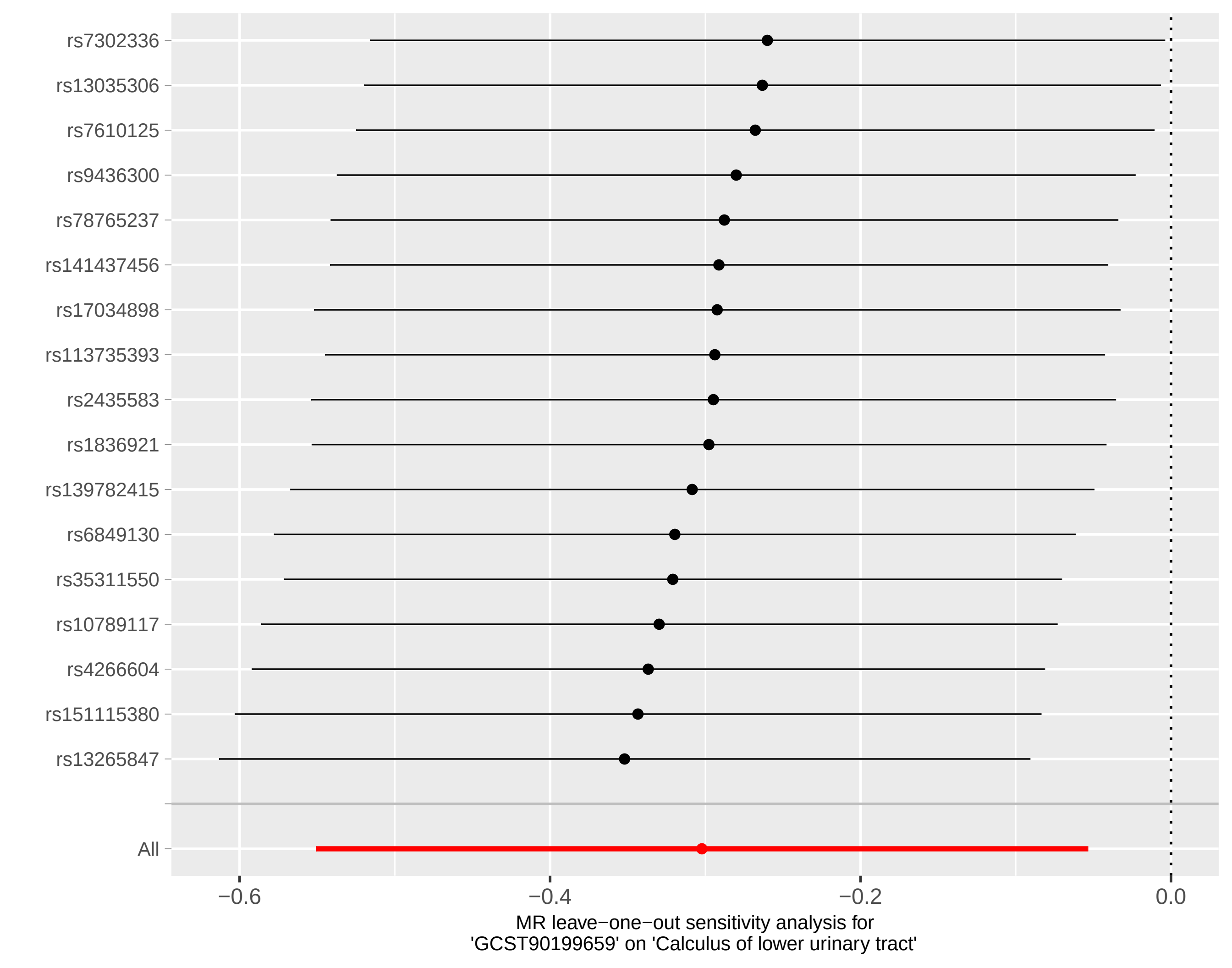
rs7302336
rs13035306
rs7610125
rs9436300
rs78765237
rs141437456
rs17034898
rs113735393
rs2435583
rs1836921
rs139782415
rs6849130
rs35311550
rs10789117
rs4266604
rs151115380
rs13265847
All
−0.6
−0.4
−0.2
0.0
MR leave−one−out sensitivity analysis for
'GCST90199659' on 'Calculus of lower urinary tract'

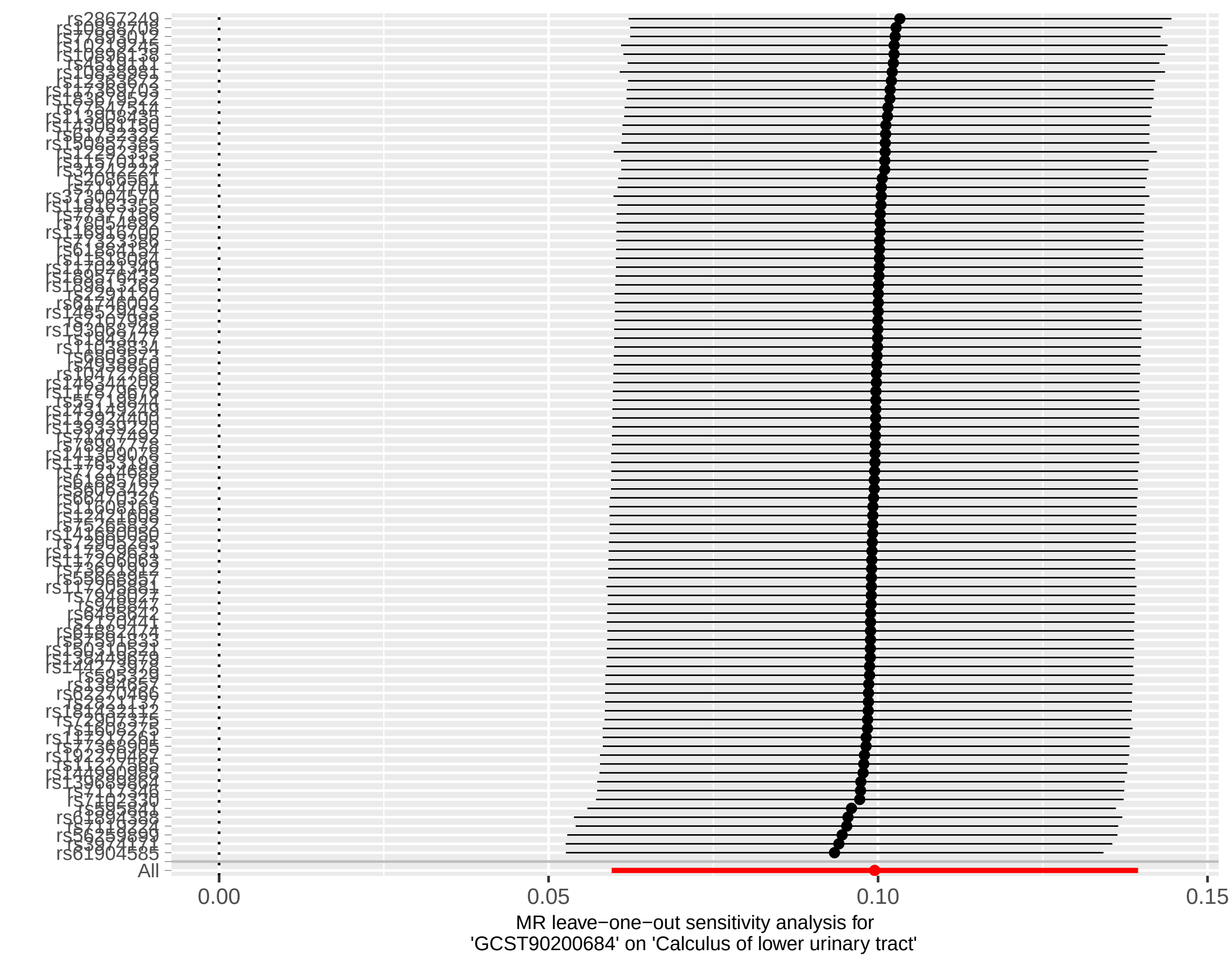
rs2867249
rs61904585
All
0.00
0.05
0.10
0.15
MR leave−one−out sensitivity analysis for
'GCST90200684' on 'Calculus of lower urinary tract'

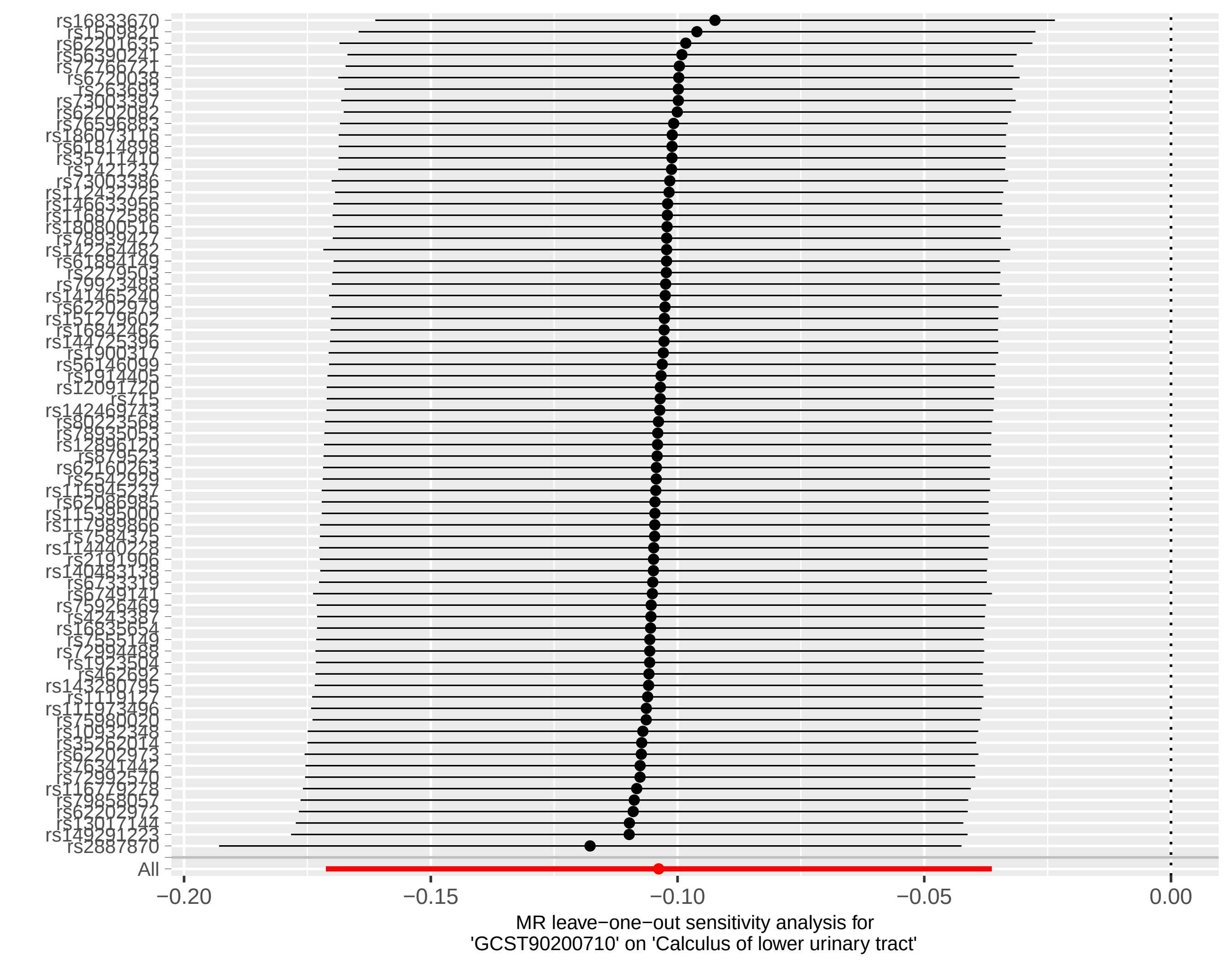
rs16833670
rs1509821
rs62201635
rs56390241
rs72766721
rs6720038
rs263693
rs73003397
rs62202082
rs76596883
rs186073116
rs61814898
rs35711410
rs1421237
rs73003386
rs112432725
rs146633956
rs116872586
rs180800516
rs78939427
rs142264482
rs61884149
rs2279503
rs79923488
rs141465240
rs62202979
rs151279602
rs16842462
rs144725396
rs1900317
rs56146099
rs1914405
rs12091720
rs715
rs142469743
rs80223568
rs78935053
rs12896120
rs879523
rs62160263
rs2542929
rs115945237
rs62086985
rs115395000
rs117989866
rs7584375
rs114440228
rs2191906
rs140483138
rs6733319
rs6749141
rs75926469
rs4243387
rs16835654
rs7555149
rs72994488
rs1923504
rs462692
rs143280795
rs1119127
rs11973496
rs75980020
rs10932348
rs35262014
rs62202973
rs76341442
rs72992570
rs116779278
rs79858057
rs62202972
rs13017144
rs149291223
rs2887870
All
−0.20
−0.15
−0.10
−0.05
0.00
MR leave−one−out sensitivity analysis for
'GCST90200710' on 'Calculus of lower urinary tract'

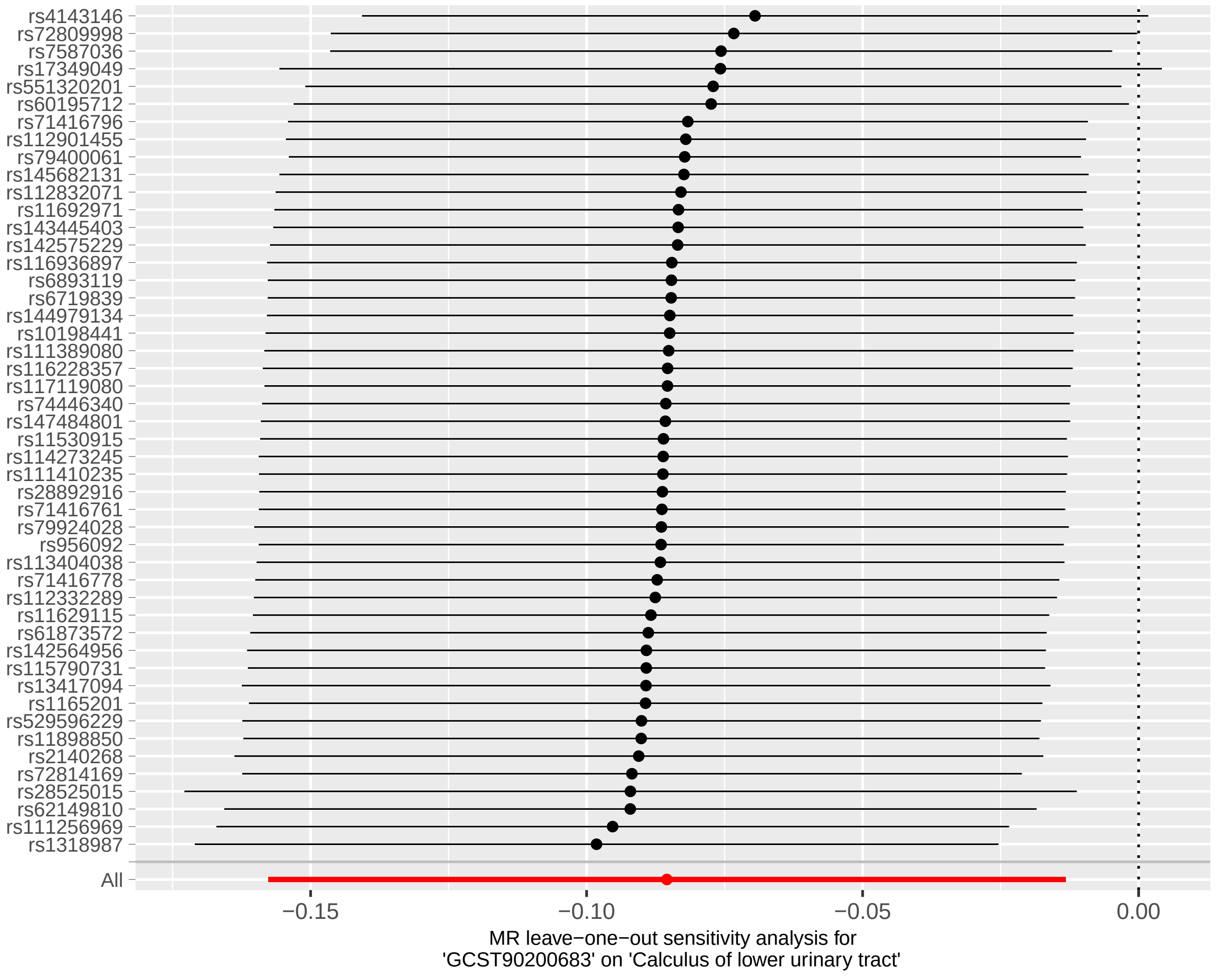

rs4143146
rs72809998
rs7587036
rs17349049
rs551320201
rs60195712
rs71416796
rs112901455
rs79400061
rs145682131
rs112832071
rs11692971
rs143445403
rs142575229
rs116936897
rs6893119
rs6719839
rs144979134
rs10198441
rs111389080
rs116228357
rs117119080
rs74446340
rs147484801
rs11530915
rs114273245
rs111410235
rs28892916
rs71416761
rs79924028
rs956092
rs113404038
rs71416778
rs112332289
rs11629115
rs61873572
rs142564956
rs115790731
rs13417094
rs1165201
rs529596229
rs11898850
rs2140268
rs72814169
rs28525015
rs62149810
rs111256969
rs1318987
All
−0.15
−0.10
−0.05
0.00
MR leave−one−out sensitivity analysis for
'GCST90200683' on 'Calculus of lower urinary tract'

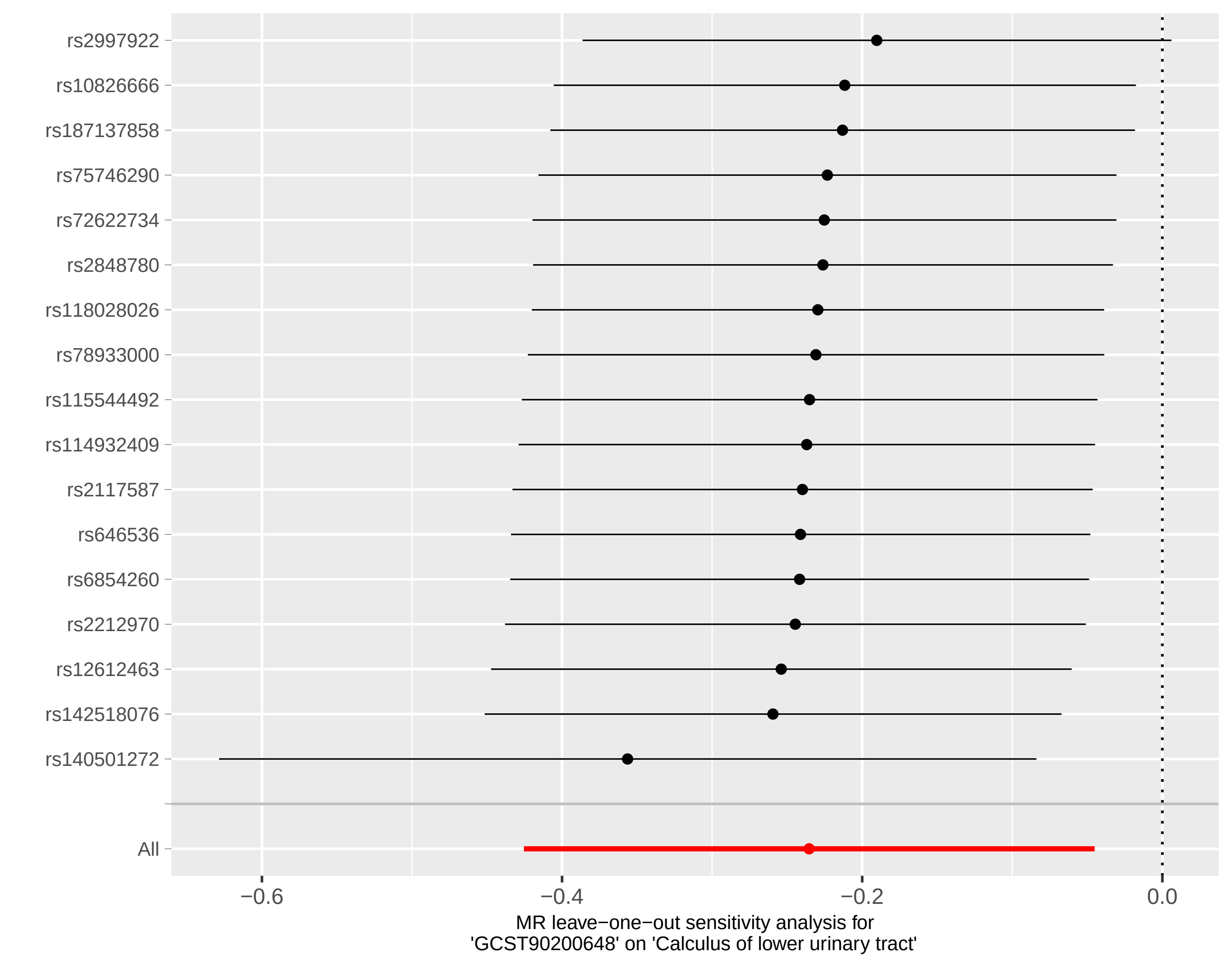
rs2997922
rs10826666
rs187137858
rs75746290
rs72622734
rs2848780
rs118028026
rs78933000
rs115544492
rs114932409
rs2117587
rs646536
rs6854260
rs2212970
rs12612463
rs142518076
rs140501272
All
−0.6
−0.4
−0.2
0.0
MR leave−one−out sensitivity analysis for
'GCST90200648' on 'Calculus of lower urinary tract'

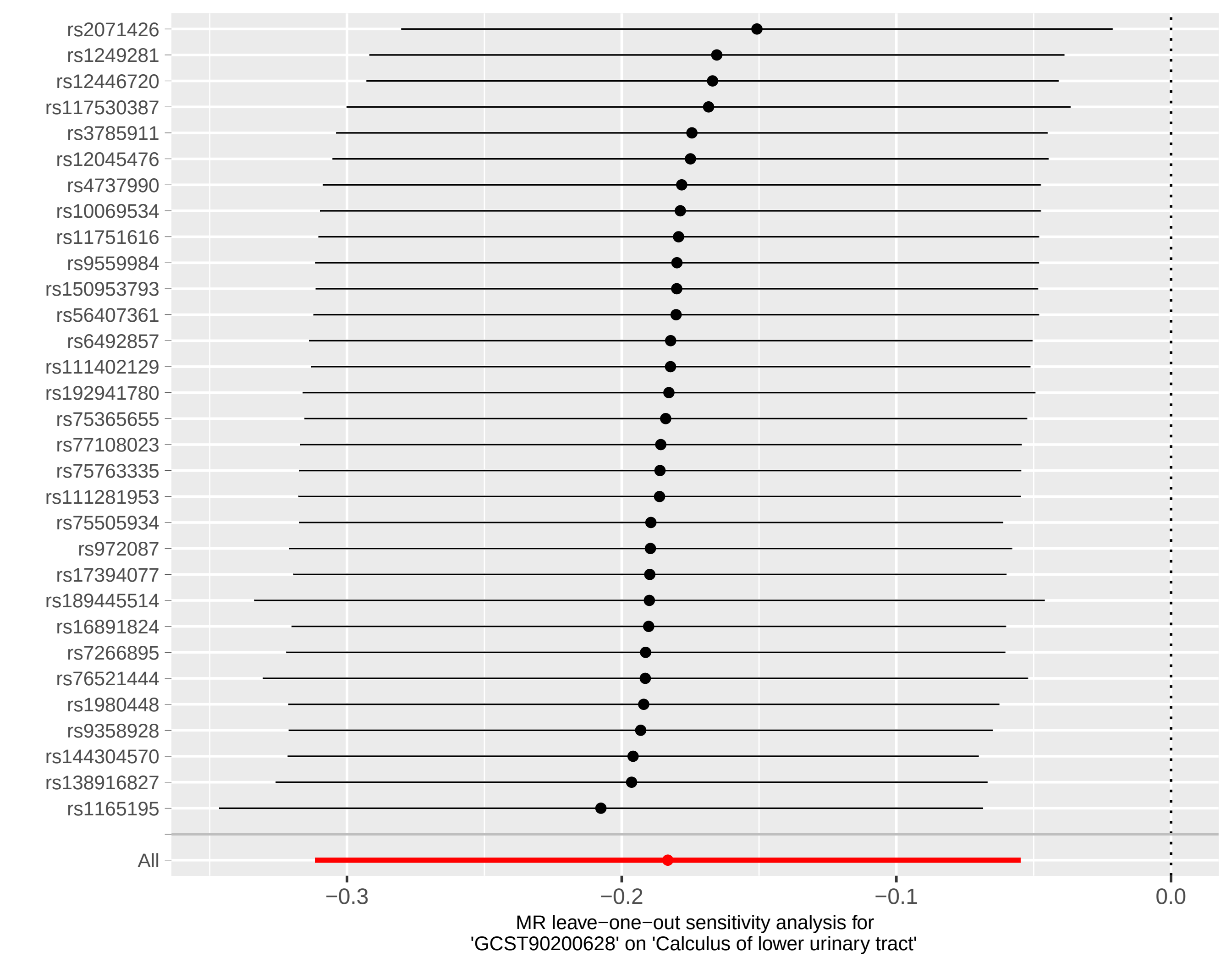

rs2071426
rs1249281
rs12446720
rs117530387
rs3785911
rs12045476
rs4737990
rs10069534
rs11751616
rs9559984
rs150953793
rs56407361
rs6492857
rs111402129
rs192941780
rs75365655
rs77108023
rs75763335
rs111281953
rs75505934
rs972087
rs17394077
rs189445514
rs16891824
rs7266895
rs76521444
rs1980448
rs9358928
rs144304570
rs138916827
rs1165195
All
−0.3
−0.2
−0.1
0.0
MR leave−one−out sensitivity analysis for
'GCST90200628' on 'Calculus of lower urinary tract'

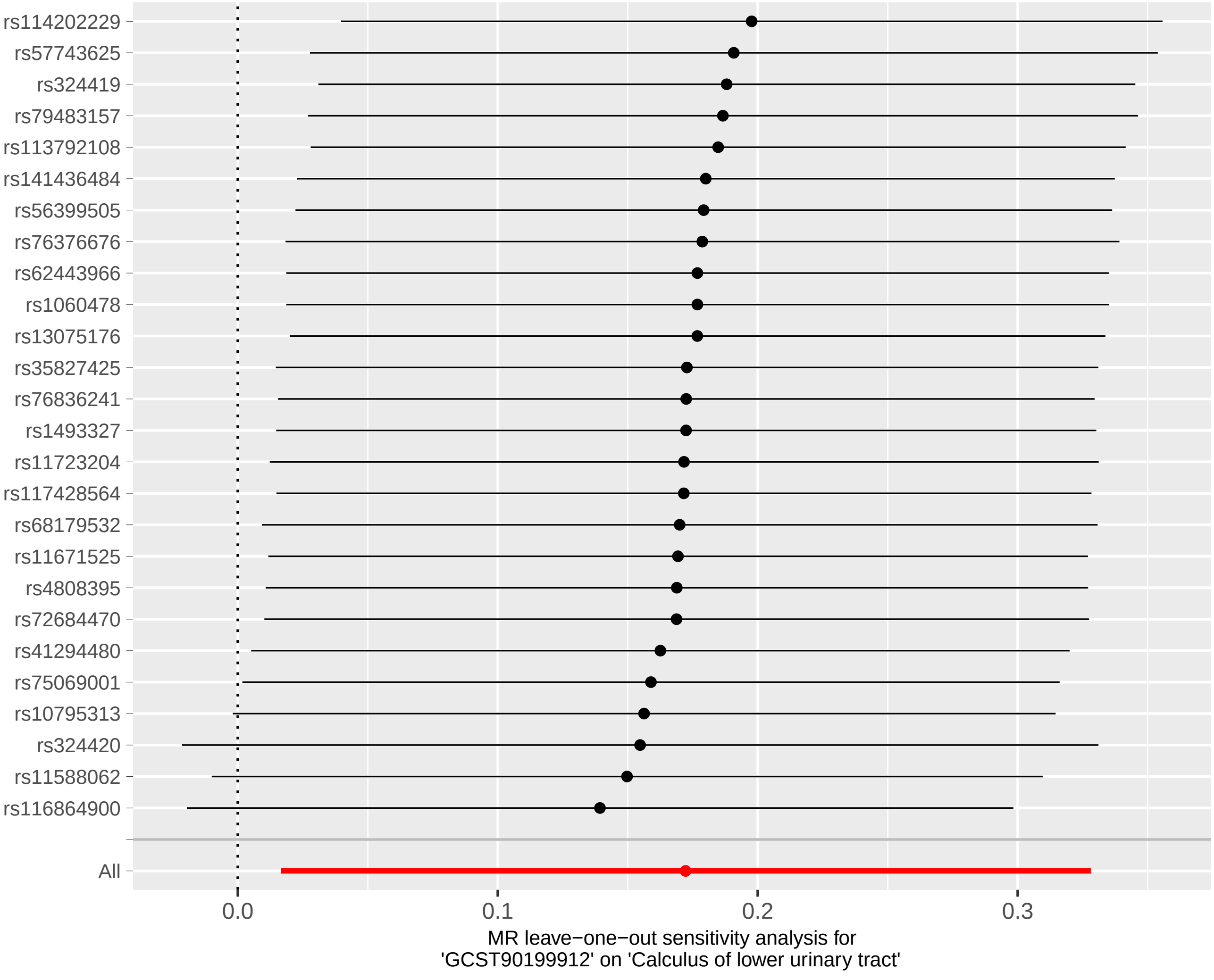
rs114202229
rs57743625
rs324419
rs79483157
rs113792108
rs141436484
rs56399505
rs76376676
rs62443966
rs1060478
rs13075176
rs35827425
rs76836241
rs1493327
rs11723204
rs117428564
rs68179532
rs11671525
rs4808395
rs72684470
rs41294480
rs75069001
rs10795313
rs324420
rs11588062
rs116864900
All
0.0
0.1
0.2
0.3
MR leave−one−out sensitivity analysis for
'GCST90199912' on 'Calculus of lower urinary tract'

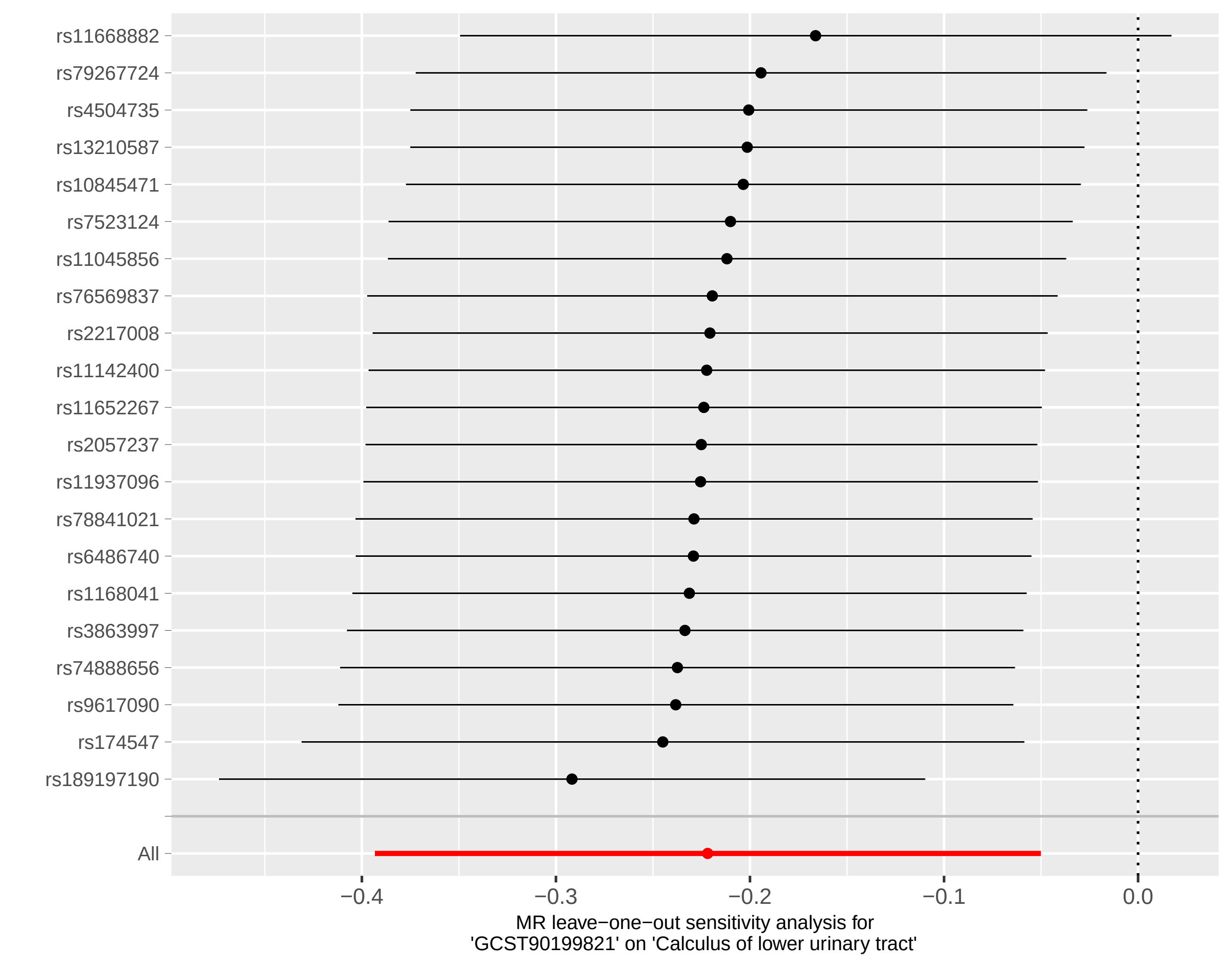
rs11668882
rs79267724
rs4504735
rs13210587
rs10845471
rs7523124
rs11045856
rs76569837
rs2217008
rs11142400
rs11652267
rs2057237
rs11937096
rs78841021
rs6486740
rs1168041
rs3863997
rs74888656
rs9617090
rs174547
rs189197190
All
−0.4
−0.3
−0.2
−0.1
0.0
MR leave−one−out sensitivity analysis for
'GCST90199821' on 'Calculus of lower urinary tract'

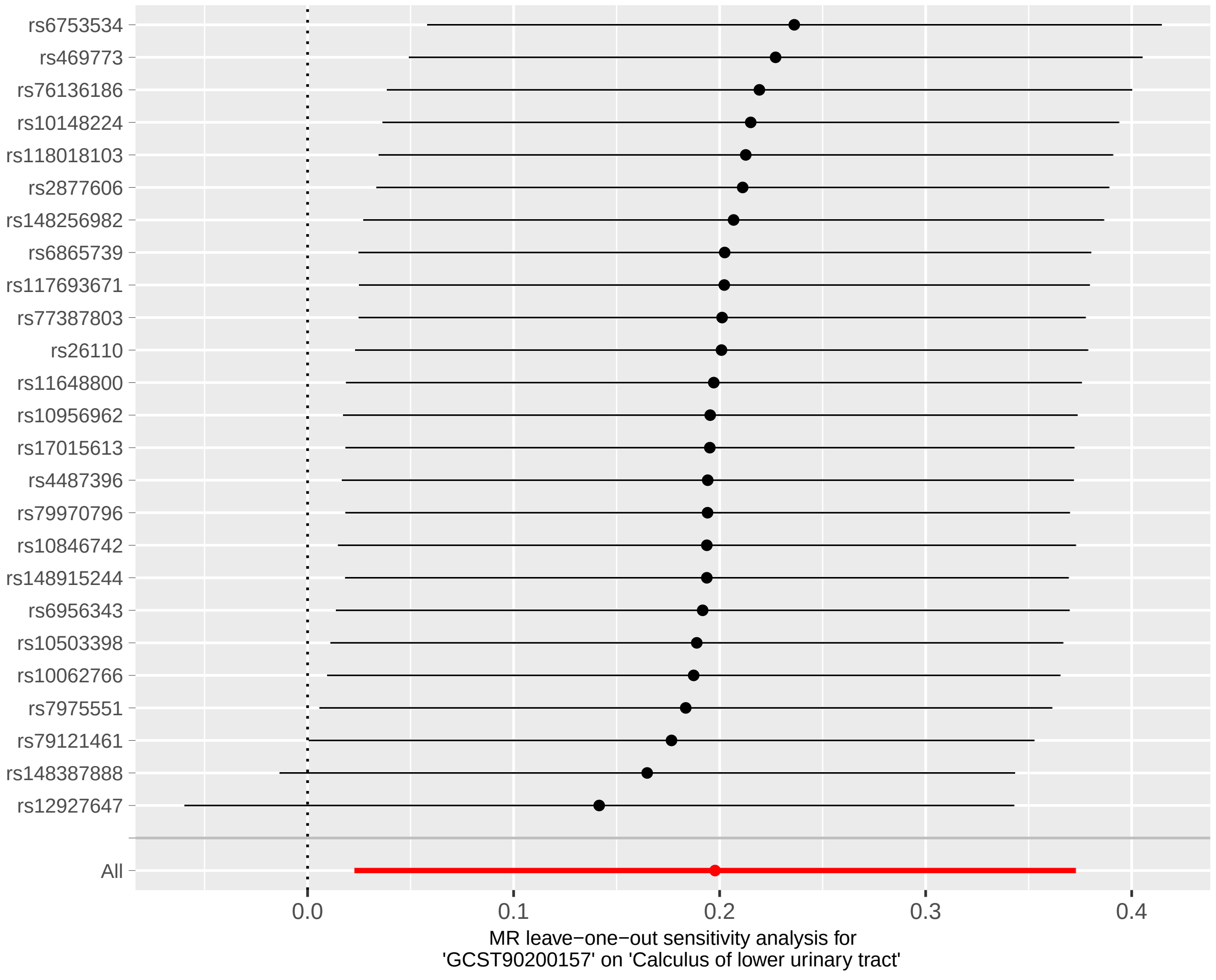
rs6753534
rs469773
rs76136186
rs10148224
rs118018103
rs2877606
rs148256982
rs6865739
rs117693671
rs77387803
rs26110
rs11648800
rs10956962
rs17015613
rs4487396
rs79970796
rs10846742
rs148915244
rs6956343
rs10503398
rs10062766
rs7975551
rs79121461
rs148387888
rs12927647
All
0.0
0.1
0.2
0.3
0.4
MR leave−one−out sensitivity analysis for
'GCST90200157' on 'Calculus of lower urinary tract'

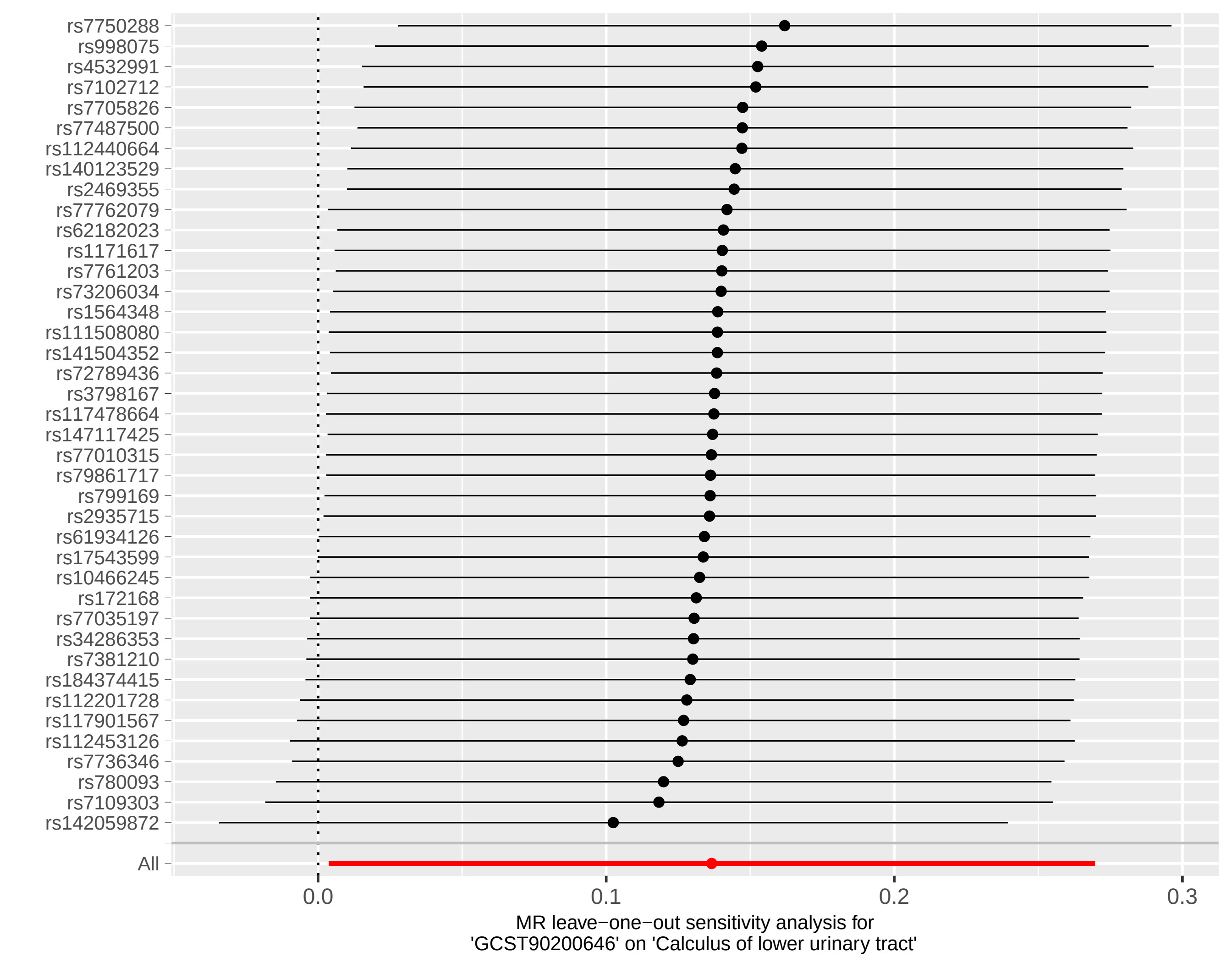
rs7750288
rs998075
rs4532991
rs7102712
rs7705826
rs77487500
rs112440664
rs140123529
rs2469355
rs77762079
rs62182023
rs1171617
rs7761203
rs73206034
rs1564348
rs111508080
rs141504352
rs72789436
rs3798167
rs117478664
rs147117425
rs77010315
rs79861717
rs799169
rs2935715
rs61934126
rs17543599
rs10466245
rs172168
rs77035197
rs34286353
rs7381210
rs184374415
rs112201728
rs117901567
rs112453126
rs7736346
rs780093
rs7109303
rs142059872
All
0.0
0.1
0.2
0.3
MR leave−one−out sensitivity analysis for
'GCST90200646' on 'Calculus of lower urinary tract'

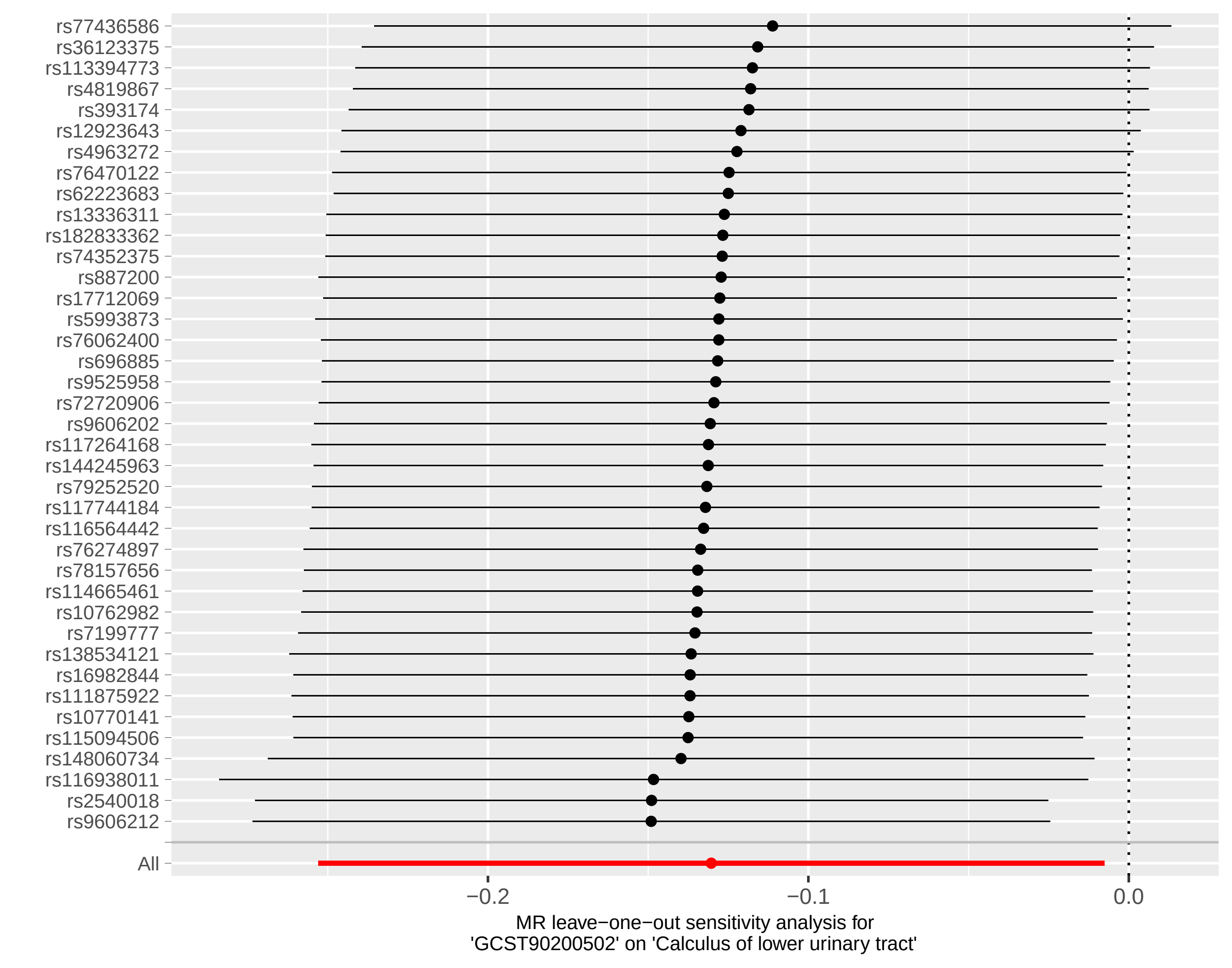

rs77436586
rs36123375
rs113394773
rs4819867
rs393174
rs12923643
rs4963272
rs76470122
rs62223683
rs13336311
rs182833362
rs74352375
rs887200
rs17712069
rs5993873
rs76062400
rs696885
rs9525958
rs72720906
rs9606202
rs117264168
rs144245963
rs79252520
rs117744184
rs116564442
rs76274897
rs78157656
rs114665461
rs10762982
rs7199777
rs138534121
rs16982844
rs111875922
rs10770141
rs115094506
rs148060734
rs116938011
rs2540018
rs9606212
All
−0.2
−0.1
0.0
MR leave−one−out sensitivity analysis for
'GCST90200502' on 'Calculus of lower urinary tract'

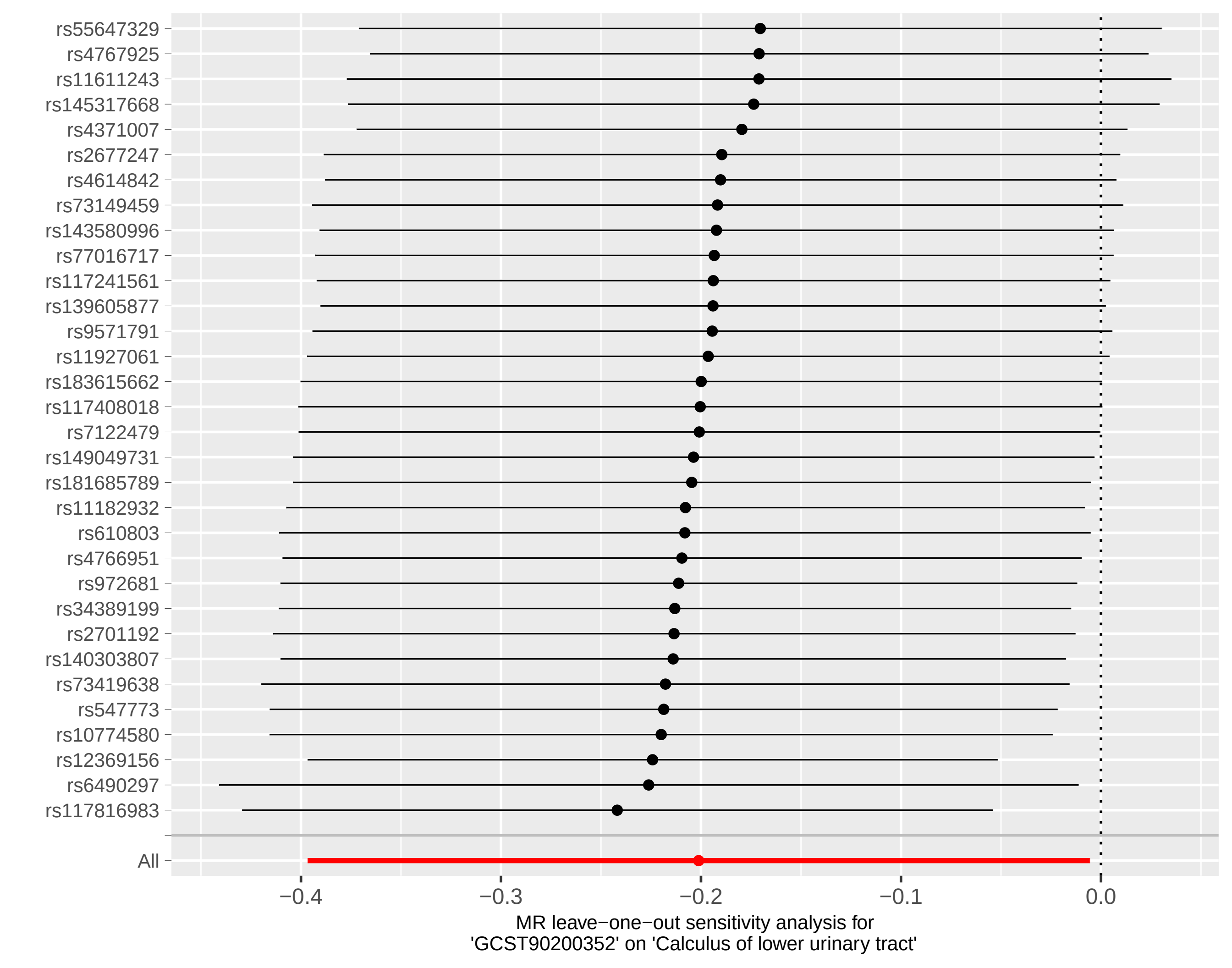
rs55647329
rs4767925
rs11611243
rs145317668
rs4371007
rs2677247
rs4614842
rs73149459
rs143580996
rs77016717
rs117241561
rs139605877
rs9571791
rs11927061
rs183615662
rs117408018
rs7122479
rs149049731
rs181685789
rs11182932
rs610803
rs4766951
rs972681
rs34389199
rs2701192
rs140303807
rs73419638
rs547773
rs10774580
rs12369156
rs6490297
rs117816983
All
−0.4
−0.3
−0.2
−0.1
0.0
MR leave−one−out sensitivity analysis for
'GCST90200352' on 'Calculus of lower urinary tract'

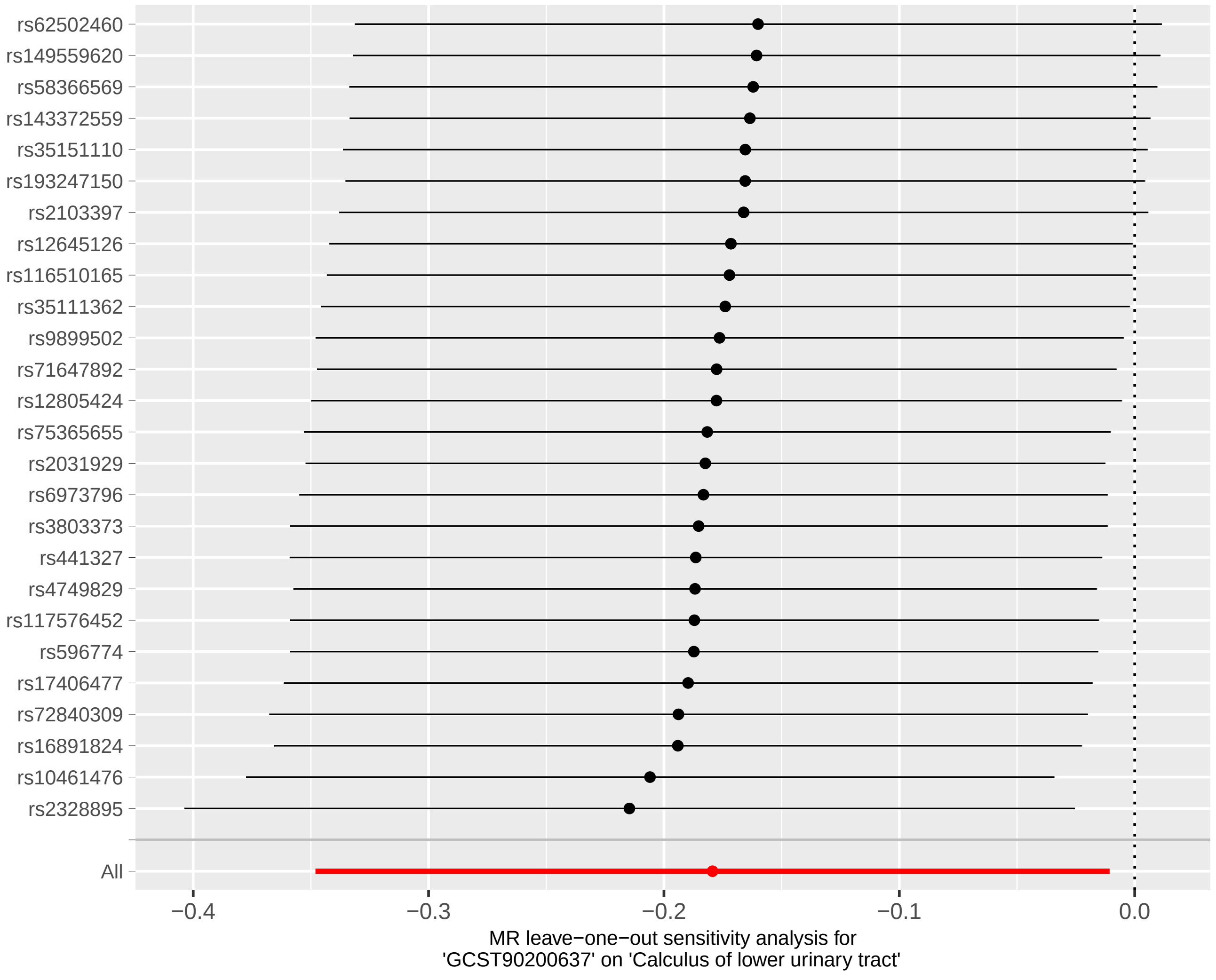
rs62502460
rs149559620
rs58366569
rs143372559
rs35151110
rs193247150
rs2103397
rs12645126
rs116510165
rs35111362
rs9899502
rs71647892
rs12805424
rs75365655
rs2031929
rs6973796
rs3803373
rs441327
rs4749829
rs117576452
rs596774
rs17406477
rs72840309
rs16891824
rs10461476
rs2328895
All
−0.4
−0.3
−0.2
−0.1
0.0
MR leave−one−out sensitivity analysis for
'GCST90200637' on 'Calculus of lower urinary tract'

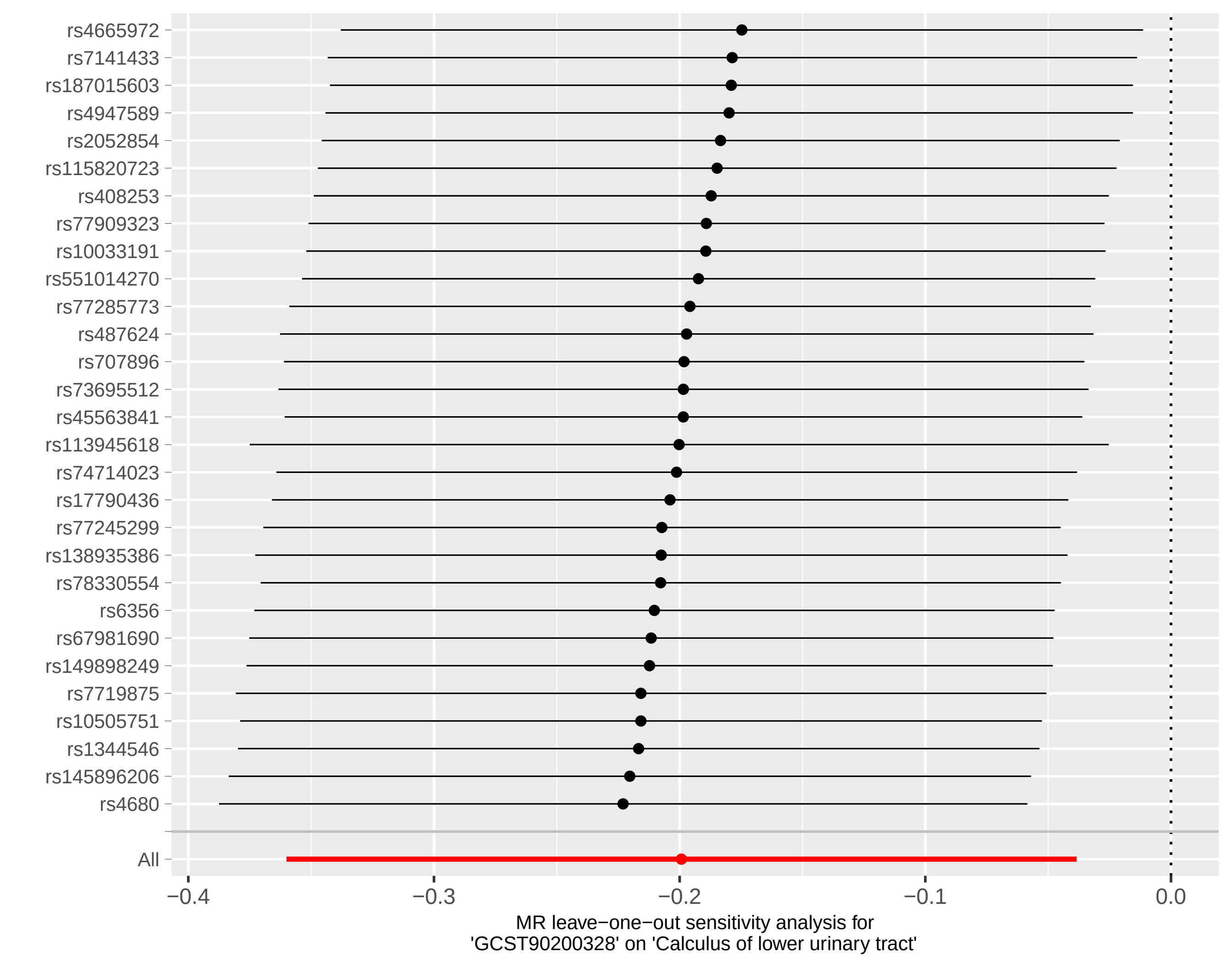
rs4665972
rs7141433
rs187015603
rs4947589
rs2052854
rs115820723
rs408253
rs77909323
rs10033191
rs551014270
rs77285773
rs487624
rs707896
rs73695512
rs45563841
rs113945618
rs74714023
rs17790436
rs77245299
rs138935386
rs78330554
rs6356
rs67981690
rs149898249
rs7719875
rs10505751
rs1344546
rs145896206
rs4680
All
−0.4
−0.3
−0.2
−0.1
0.0
MR leave−one−out sensitivity analysis for
'GCST90200328' on 'Calculus of lower urinary tract'

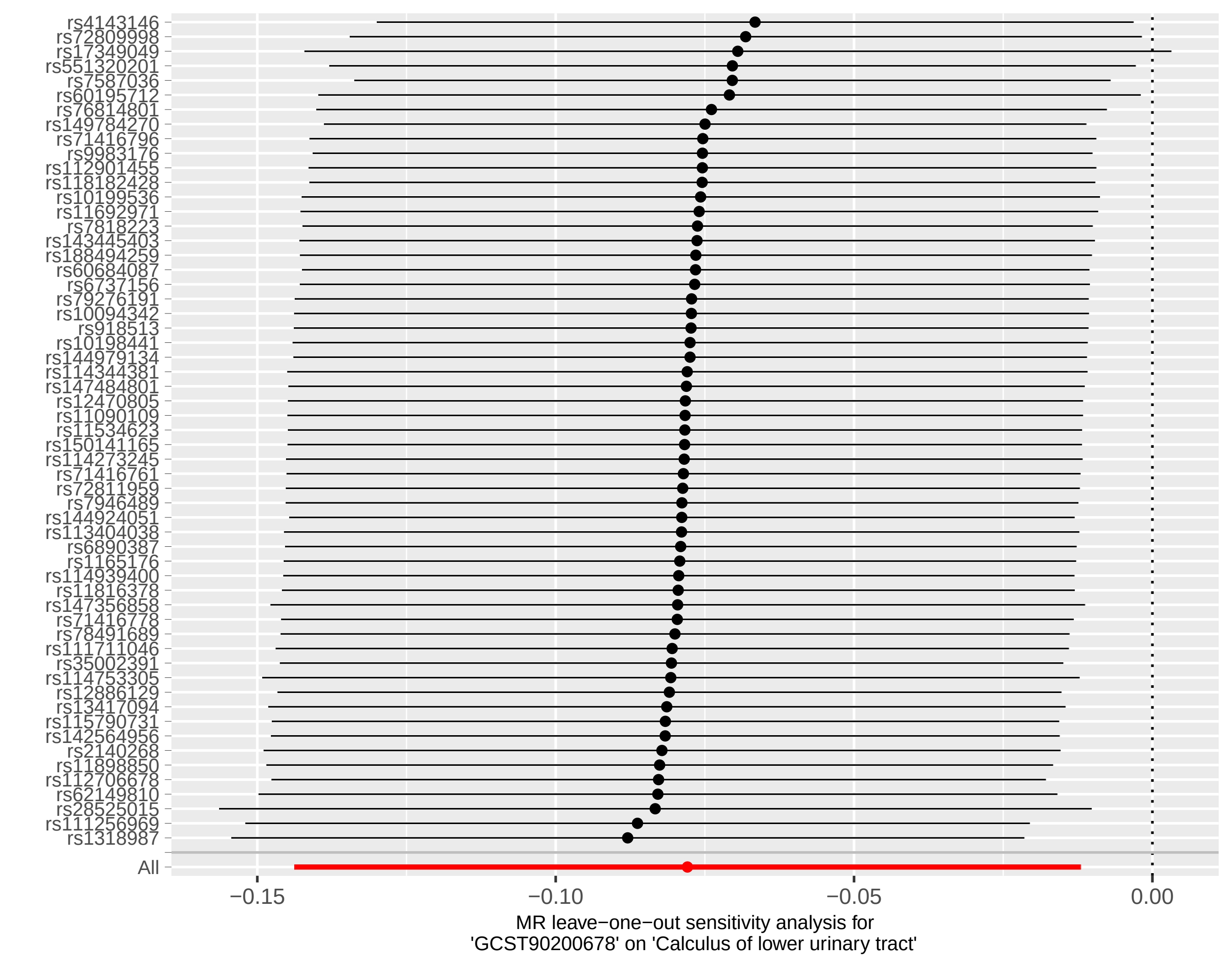
rs4143146
rs72809998
rs17349049
rs551320201
rs7587036
rs60195712
rs76814801
rs149784270
rs71416796
rs9983176
rs112901455
rs118182428
rs10199536
rs11692971
rs7818223
rs143445403
rs188494259
rs60684087
rs6737156
rs79276191
rs10094342
rs918513
rs10198441
rs144979134
rs114344381
rs147484801
rs12470805
rs11090109
rs11534623
rs150141165
rs114273245
rs71416761
rs72811959
rs7946489
rs144924051
rs113404038
rs6890387
rs1165176
rs114939400
rs11816378
rs147356858
rs71416778
rs78491689
rs111711046
rs35002391
rs114753305
rs12886129
rs13417094
rs115790731
rs142564956
rs2140268
rs11898850
rs112706678
rs62149810
rs28525015
rs111256969
rs1318987
All
−0.15
−0.10
−0.05
0.00
MR leave−one−out sensitivity analysis for
'GCST90200678' on 'Calculus of lower urinary tract'

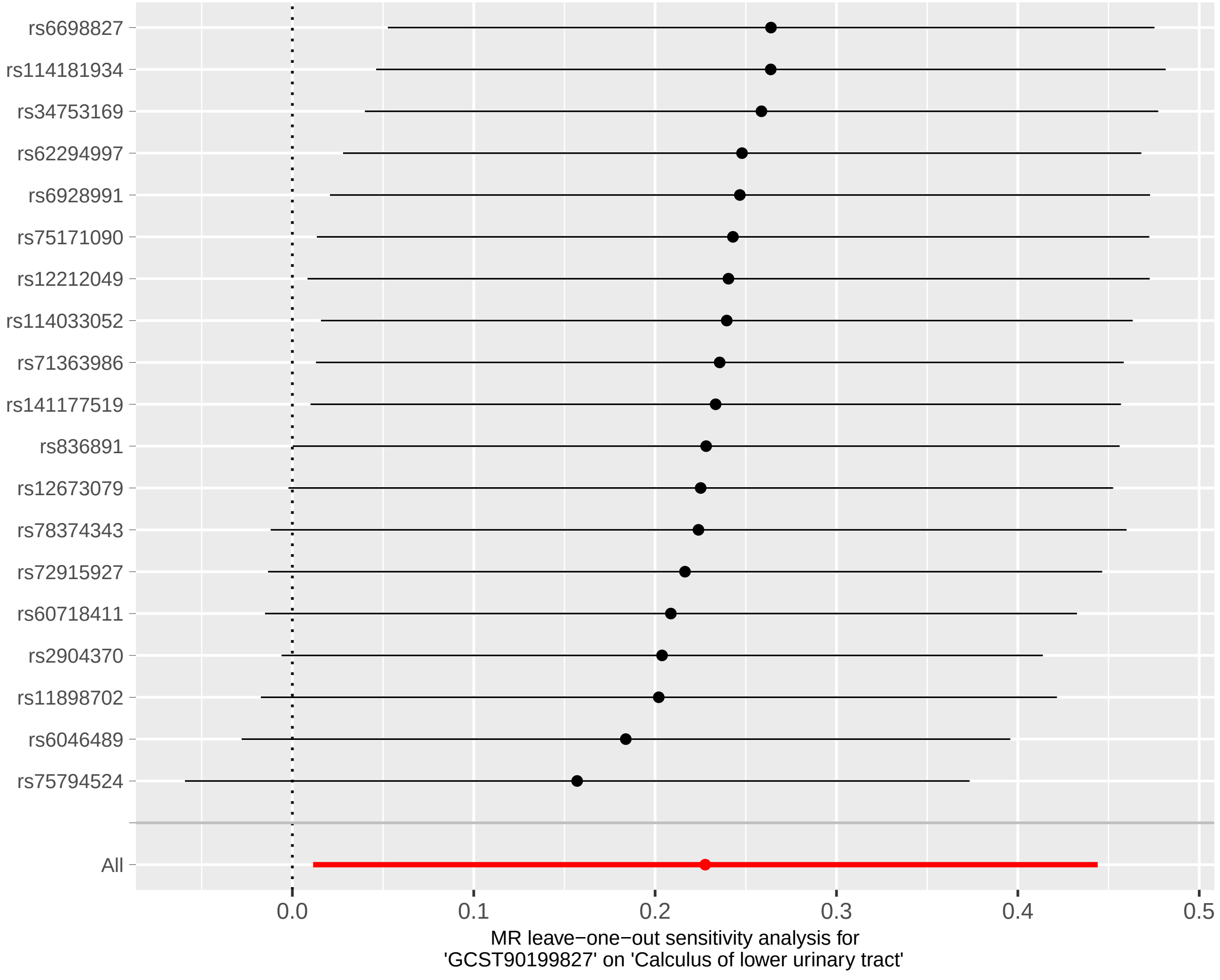
rs6698827
rs114181934
rs34753169
rs62294997
rs6928991
rs75171090
rs12212049
rs114033052
rs71363986
rs141177519
rs836891
rs12673079
rs78374343
rs72915927
rs60718411
rs2904370
rs11898702
rs6046489
rs75794524
All
0.0
0.1
0.2
0.3
0.4
0.5
MR leave−one−out sensitivity analysis for
'GCST90199827' on 'Calculus of lower urinary tract'

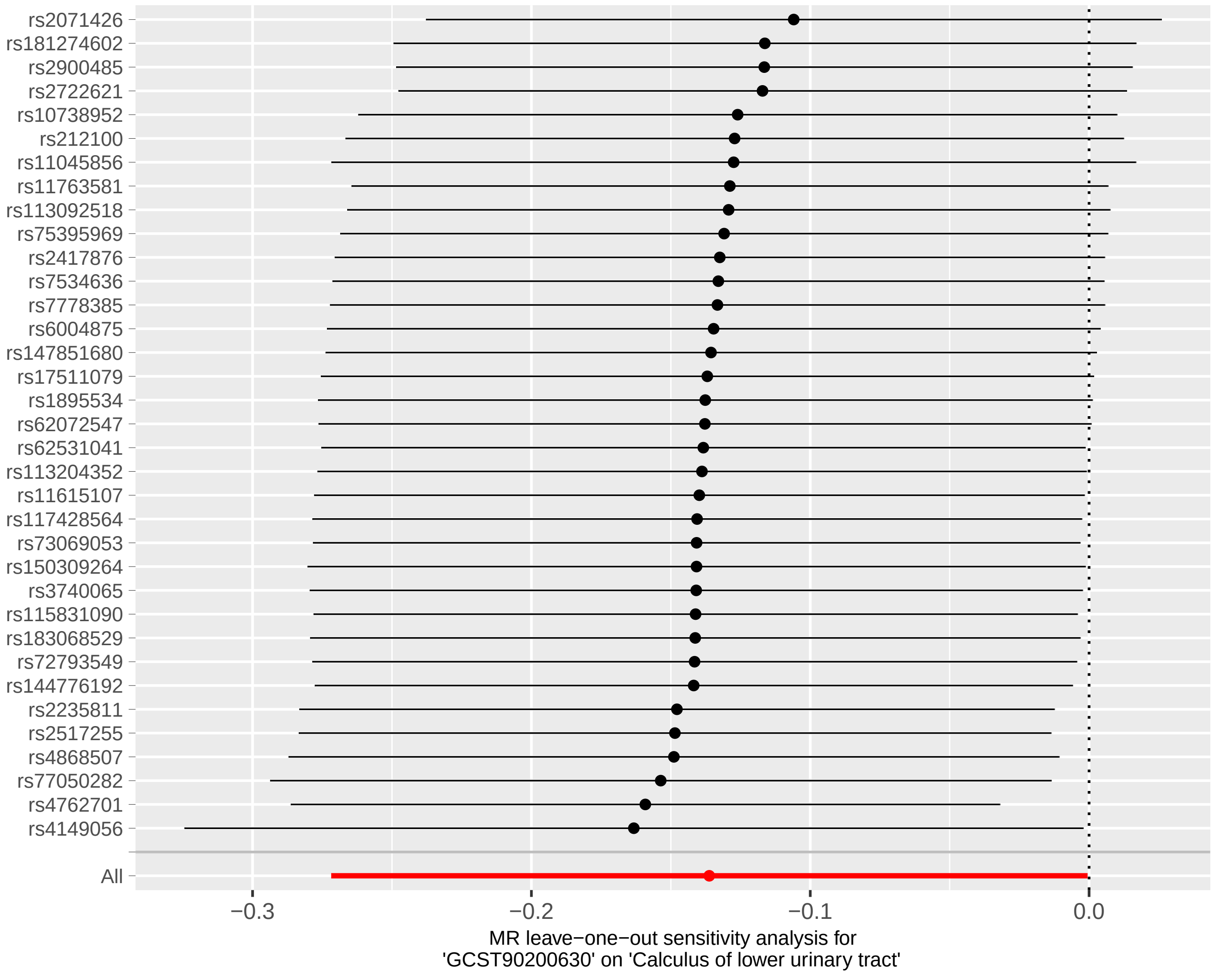
rs2071426
rs181274602
rs2900485
rs2722621
rs10738952
rs212100
rs11045856
rs11763581
rs113092518
rs75395969
rs2417876
rs7534636
rs7778385
rs6004875
rs147851680
rs17511079
rs1895534
rs62072547
rs62531041
rs113204352
rs11615107
rs117428564
rs73069053
rs150309264
rs3740065
rs115831090
rs183068529
rs72793549
rs144776192
rs2235811
rs2517255
rs4868507
rs77050282
rs4762701
rs4149056
All
−0.3
−0.2
−0.1
0.0
MR leave−one−out sensitivity analysis for
'GCST90200630' on 'Calculus of lower urinary tract'

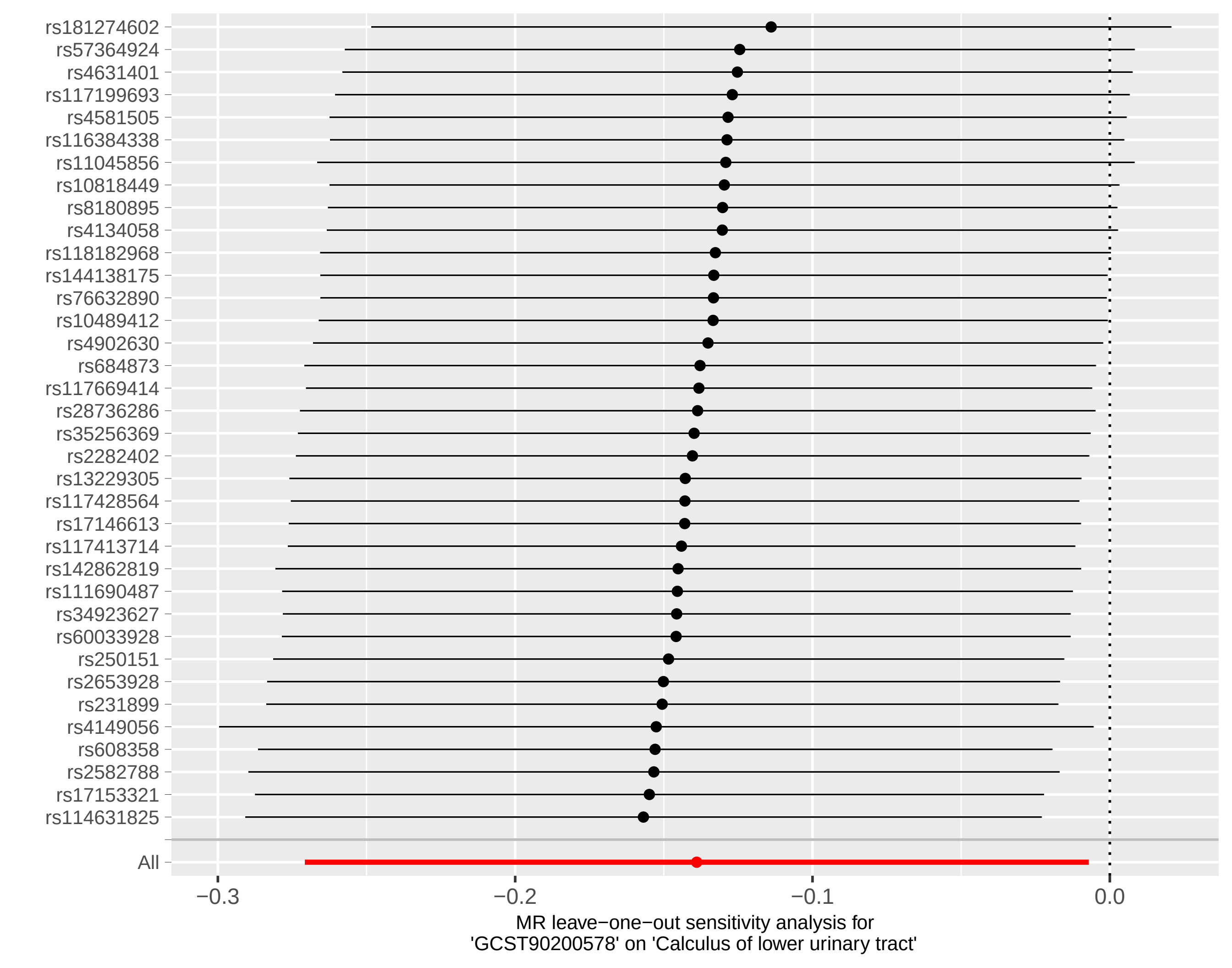
rs181274602
rs57364924
rs4631401
rs117199693
rs4581505
rs116384338
rs11045856
rs10818449
rs8180895
rs4134058
rs118182968
rs144138175
rs76632890
rs10489412
rs4902630
rs684873
rs117669414
rs28736286
rs35256369
rs2282402
rs13229305
rs117428564
rs17146613
rs117413714
rs142862819
rs111690487
rs34923627
rs60033928
rs250151
rs2653928
rs231899
rs4149056
rs608358
rs2582788
rs17153321
rs114631825
All
−0.3
−0.2
−0.1
0.0
MR leave−one−out sensitivity analysis for
'GCST90200578' on 'Calculus of lower urinary tract'

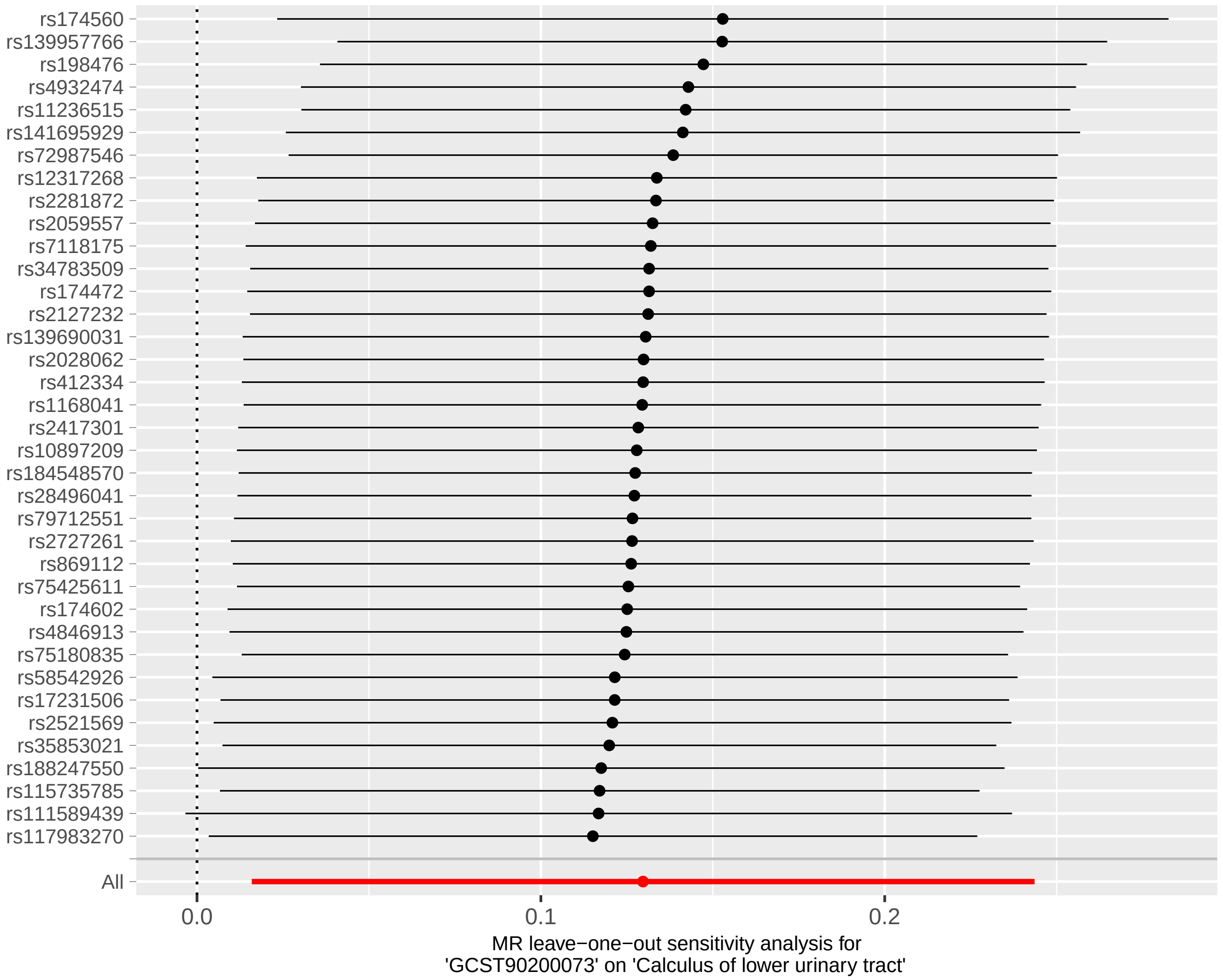

rs174560
rs139957766
rs198476
rs4932474
rs11236515
rs141695929
rs72987546
rs12317268
rs2281872
rs2059557
rs7118175
rs34783509
rs174472
rs2127232
rs139690031
rs2028062
rs412334
rs1168041
rs2417301
rs10897209
rs184548570
rs28496041
rs79712551
rs2727261
rs869112
rs75425611
rs174602
rs4846913
rs75180835
rs58542926
rs17231506
rs2521569
rs35853021
rs188247550
rs115735785
rs111589439
rs117983270
All
0.0
0.1
0.2
MR leave−one−out sensitivity analysis for
'GCST90200073' on 'Calculus of lower urinary tract'

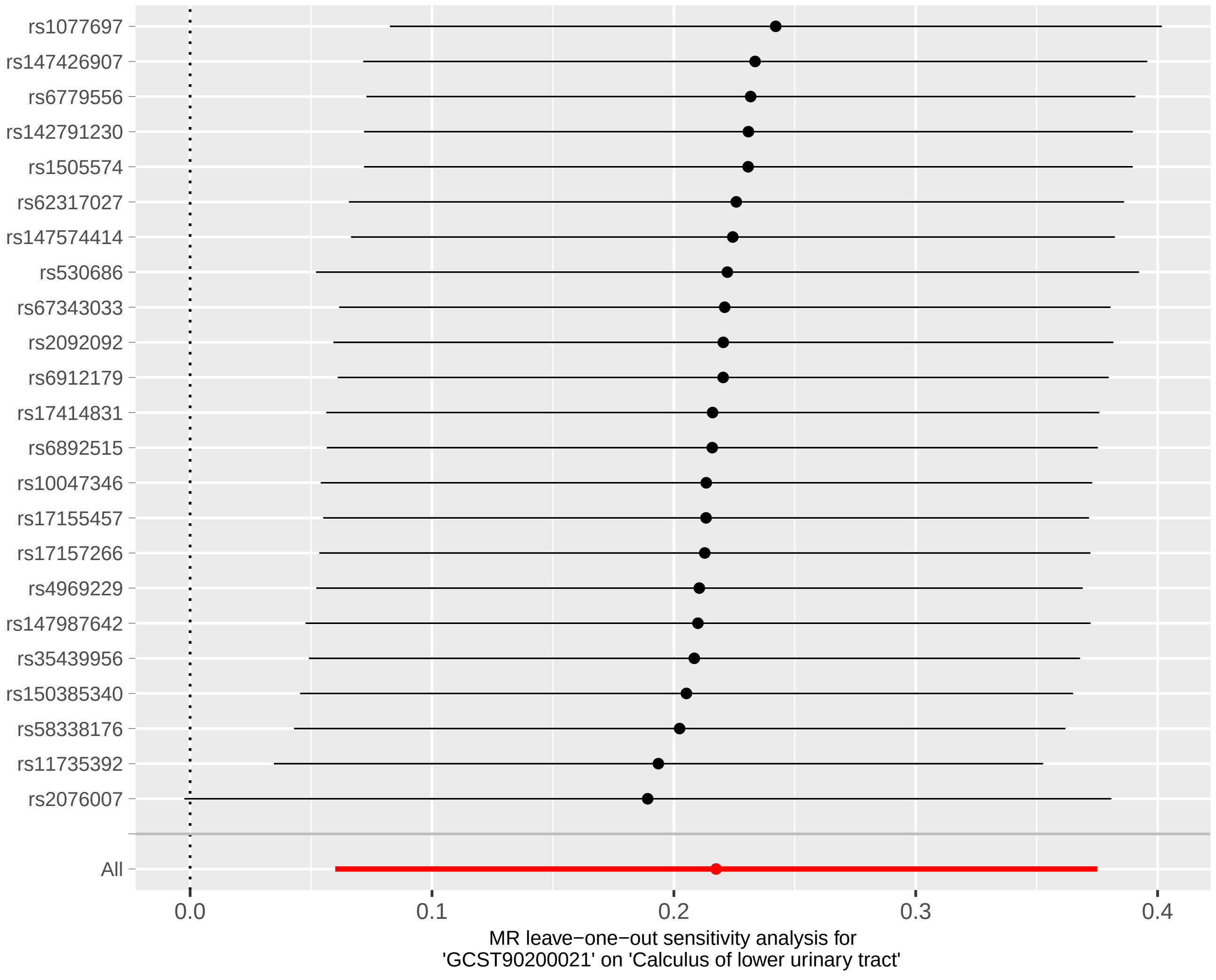
rs1077697
rs147426907
rs6779556
rs142791230
rs1505574
rs62317027
rs147574414
rs530686
rs67343033
rs2092092
rs6912179
rs17414831
rs6892515
rs10047346
rs17155457
rs17157266
rs4969229
rs147987642
rs35439956
rs150385340
rs58338176
rs11735392
rs2076007
All
0.0
0.1
0.2
0.3
0.4
MR leave−one−out sensitivity analysis for
'GCST90200021' on 'Calculus of lower urinary tract'

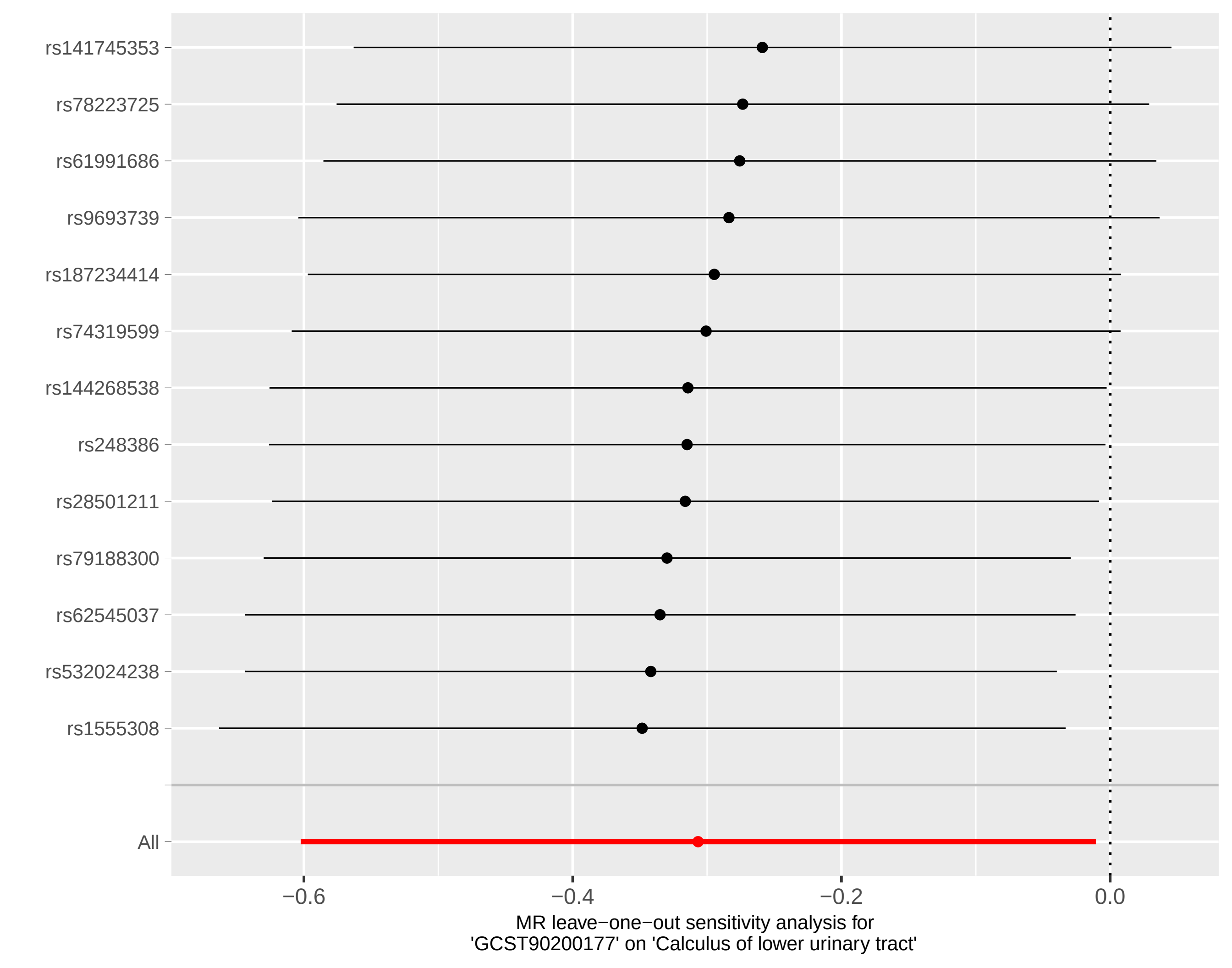

rs141745353
rs78223725
rs61991686
rs9693739
rs187234414
rs74319599
rs144268538
rs248386
rs28501211
rs79188300
rs62545037
rs532024238
rs1555308
All
−0.6
−0.4
−0.2
0.0
MR leave−one−out sensitivity analysis for
'GCST90200177' on 'Calculus of lower urinary tract'

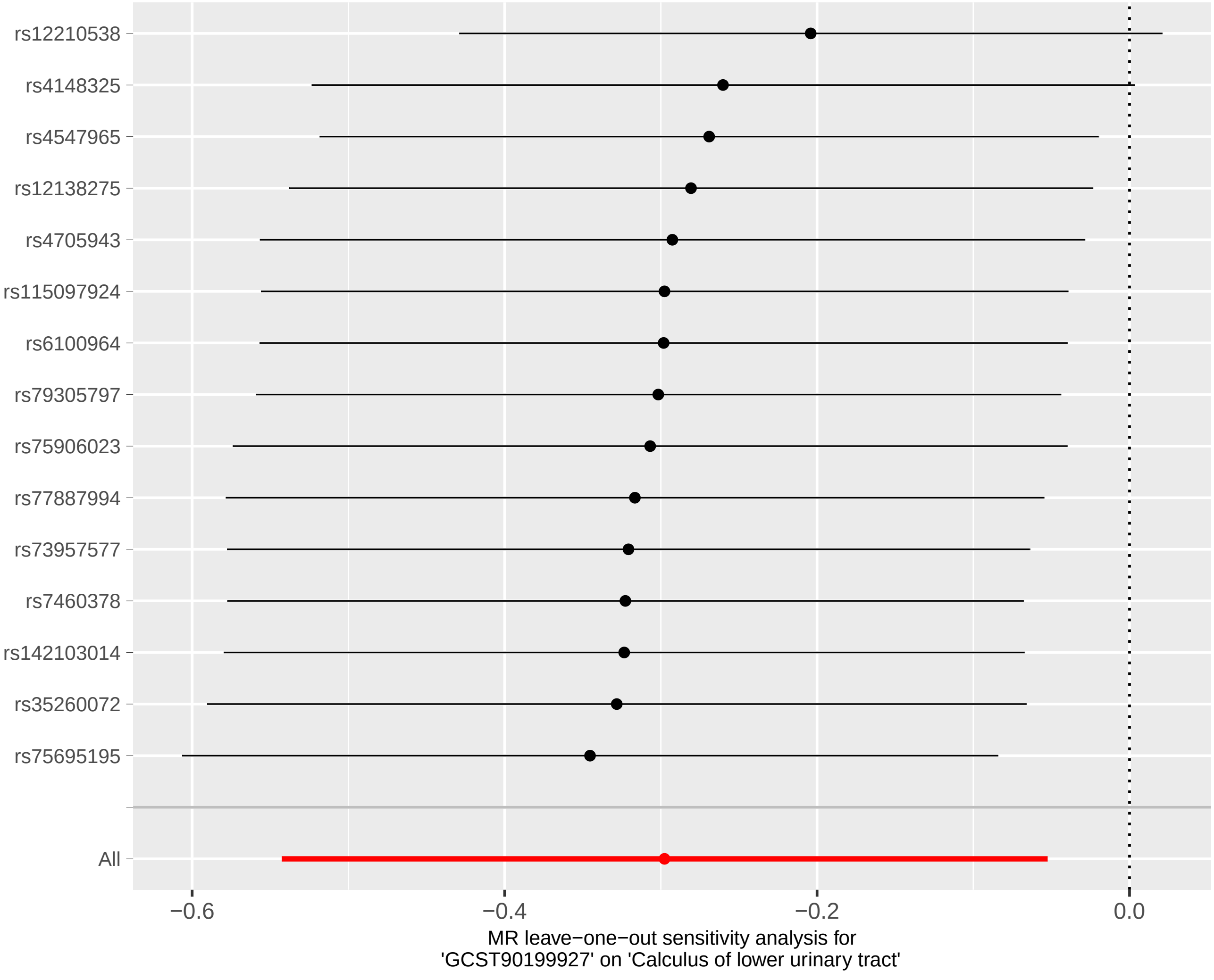
rs12210538
rs4148325
rs4547965
rs12138275
rs4705943
rs115097924
rs6100964
rs79305797
rs75906023
rs77887994
rs73957577
rs7460378
rs142103014
rs35260072
rs75695195
All
−0.6
−0.4
−0.2
0.0
MR leave−one−out sensitivity analysis for
'GCST90199927' on 'Calculus of lower urinary tract'

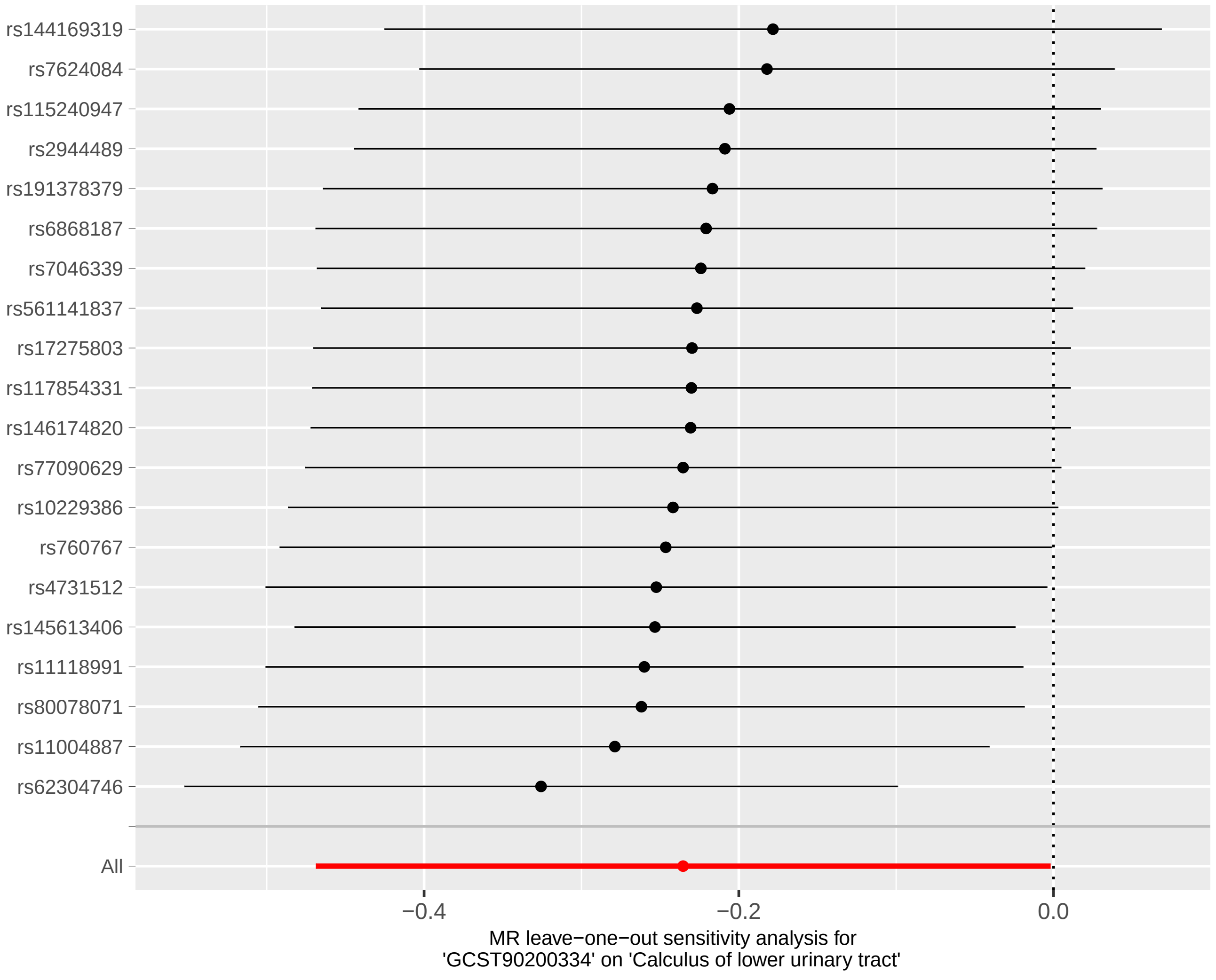
rs144169319
rs7624084
rs115240947
rs2944489
rs191378379
rs6868187
rs7046339
rs561141837
rs17275803
rs117854331
rs146174820
rs77090629
rs10229386
rs760767
rs4731512
rs145613406
rs11118991
rs80078071
rs11004887
rs62304746
All
−0.4
−0.2
0.0
MR leave−one−out sensitivity analysis for
'GCST90200334' on 'Calculus of lower urinary tract'

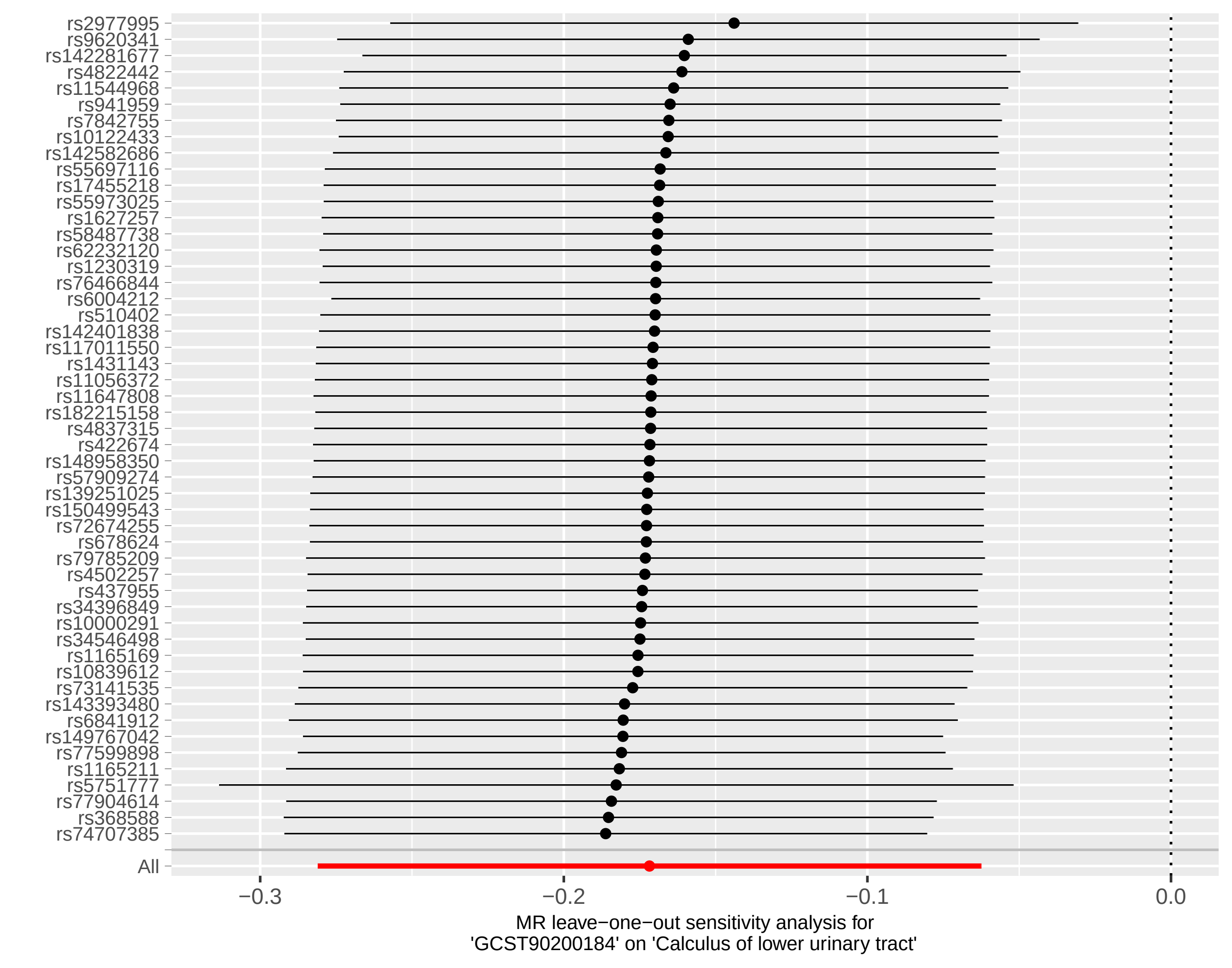
rs2977995
rs9620341
rs142281677
rs4822442
rs11544968
rs941959
rs7842755
rs10122433
rs142582686
rs55697116
rs17455218
rs55973025
rs1627257
rs58487738
rs62232120
rs1230319
rs76466844
rs6004212
rs510402
rs142401838
rs117011550
rs1431143
rs11056372
rs11647808
rs182215158
rs4837315
rs422674
rs148958350
rs57909274
rs139251025
rs150499543
rs72674255
rs678624
rs79785209
rs4502257
rs437955
rs34396849
rs10000291
rs34546498
rs1165169
rs10839612
rs73141535
rs143393480
rs6841912
rs149767042
rs77599898
rs1165211
rs5751777
rs77904614
rs368588
rs74707385
All
−0.3
−0.2
−0.1
0.0
MR leave−one−out sensitivity analysis for
'GCST90200184' on 'Calculus of lower urinary tract'

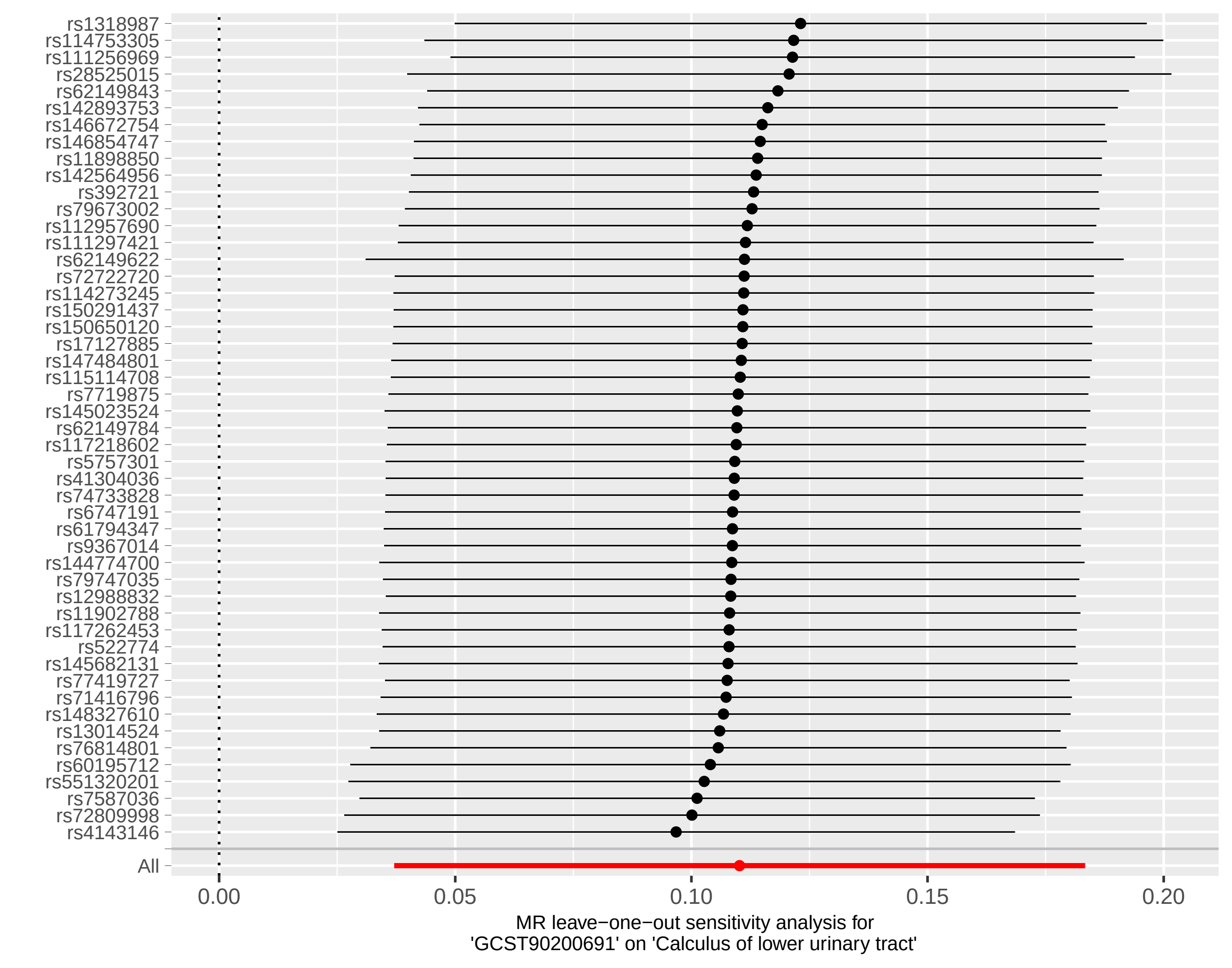

rs1318987
rs114753305
rs111256969
rs28525015
rs62149843
rs142893753
rs146672754
rs146854747
rs11898850
rs142564956
rs392721
rs79673002
rs112957690
rs111297421
rs62149622
rs72722720
rs114273245
rs150291437
rs150650120
rs17127885
rs147484801
rs115114708
rs7719875
rs145023524
rs62149784
rs117218602
rs5757301
rs41304036
rs74733828
rs6747191
rs61794347
rs9367014
rs144774700
rs79747035
rs12988832
rs11902788
rs117262453
rs522774
rs145682131
rs77419727
rs71416796
rs148327610
rs13014524
rs76814801
rs60195712
rs551320201
rs7587036
rs72809998
rs4143146
All
0.00
0.05
0.10
0.15
0.20
MR leave−one−out sensitivity analysis for
'GCST90200691' on 'Calculus of lower urinary tract'

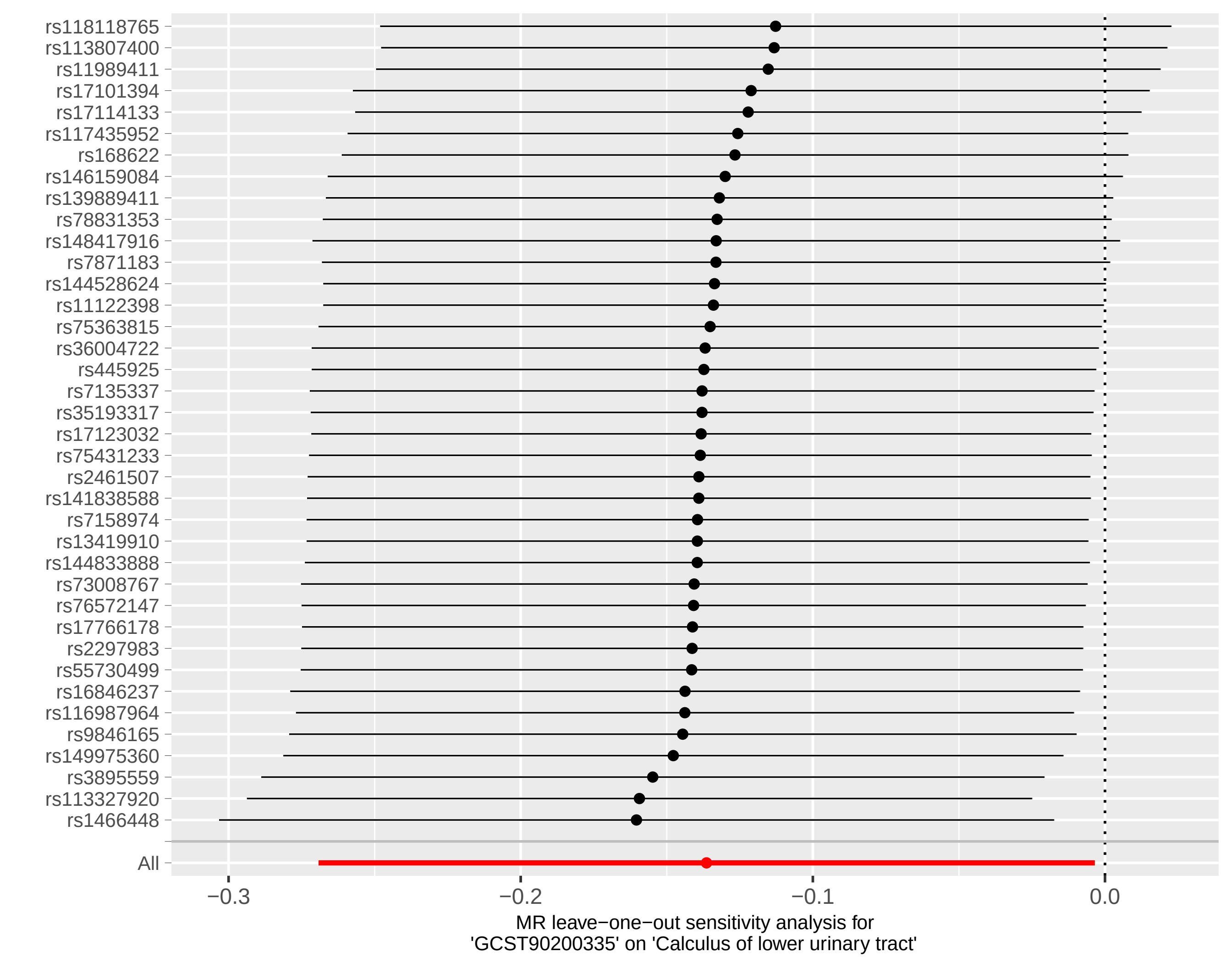

rs118118765
rs113807400
rs11989411
rs17101394
rs17114133
rs117435952
rs168622
rs146159084
rs139889411
rs78831353
rs148417916
rs7871183
rs144528624
rs11122398
rs75363815
rs36004722
rs445925
rs7135337
rs35193317
rs17123032
rs75431233
rs2461507
rs141838588
rs7158974
rs13419910
rs144833888
rs73008767
rs76572147
rs17766178
rs2297983
rs55730499
rs16846237
rs116987964
rs9846165
rs149975360
rs3895559
rs113327920
rs1466448
All
−0.3
−0.2
−0.1
0.0
MR leave−one−out sensitivity analysis for
'GCST90200335' on 'Calculus of lower urinary tract'

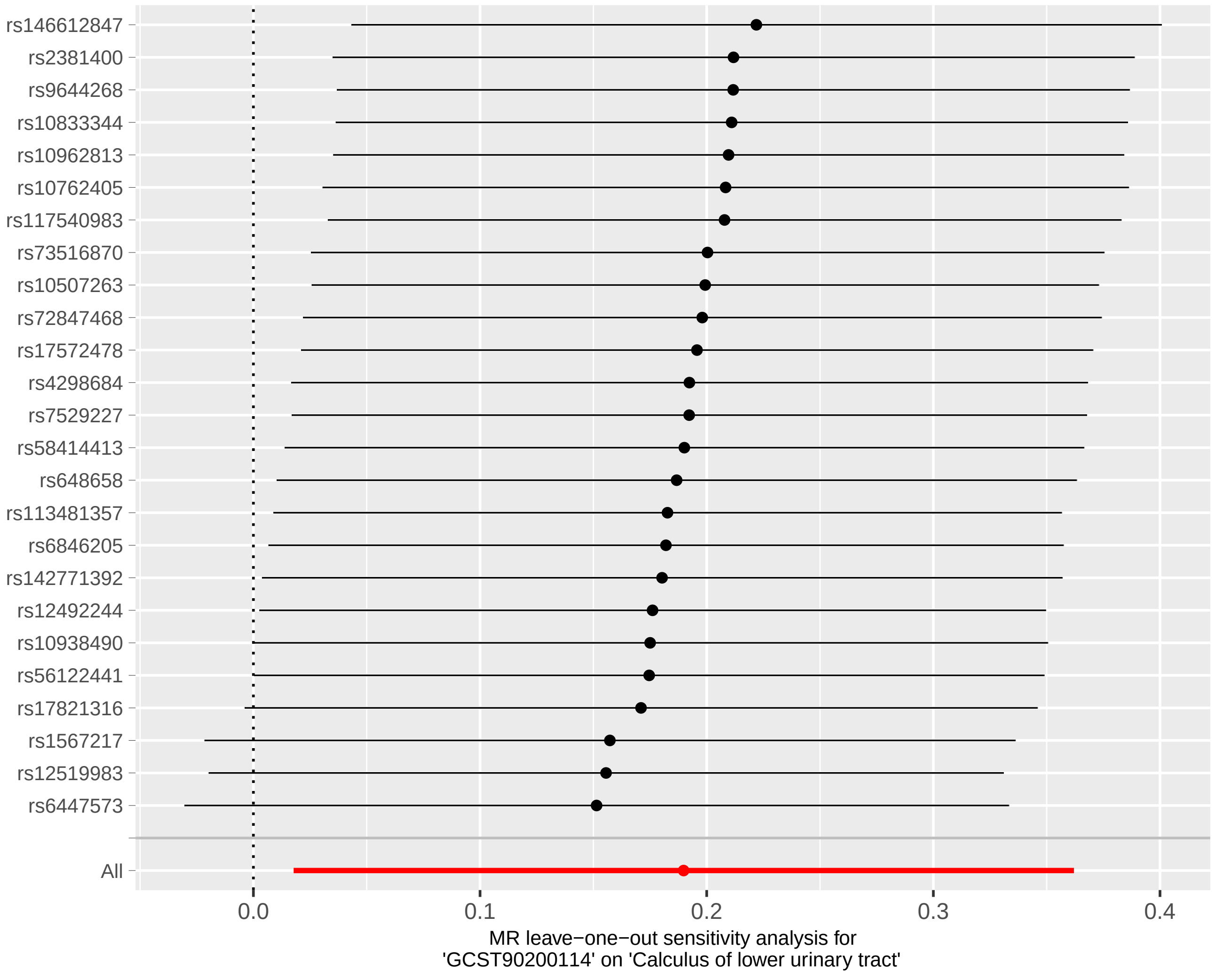
rs146612847
rs2381400
rs9644268
rs10833344
rs10962813
rs10762405
rs117540983
rs73516870
rs10507263
rs72847468
rs17572478
rs4298684
rs7529227
rs58414413
rs648658
rs113481357
rs6846205
rs142771392
rs12492244
rs10938490
rs56122441
rs17821316
rs1567217
rs12519983
rs6447573
All
0.0
0.1
0.2
0.3
0.4
MR leave−one−out sensitivity analysis for
'GCST90200114' on 'Calculus of lower urinary tract'

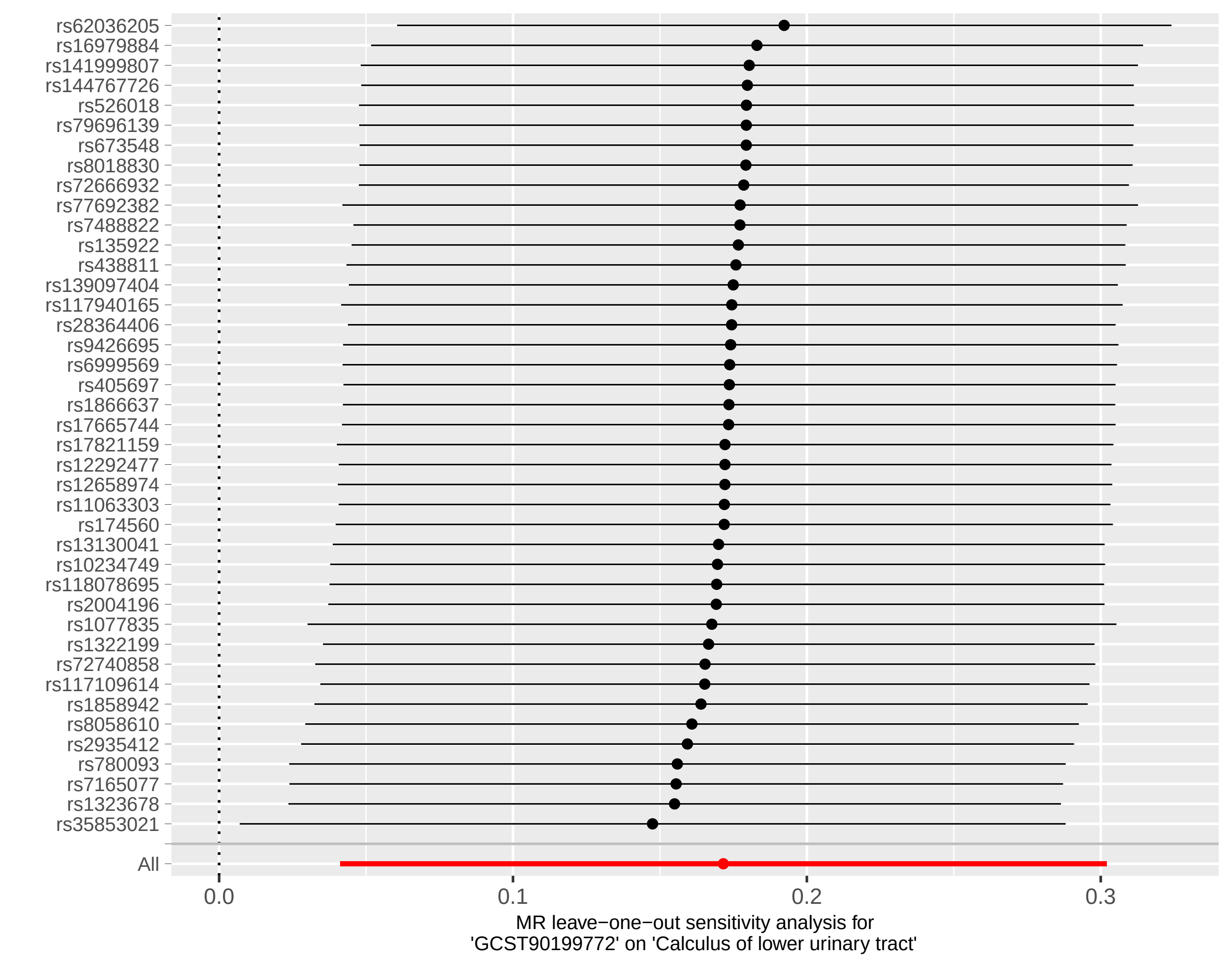

rs62036205
rs16979884
rs141999807
rs144767726
rs526018
rs79696139
rs673548
rs8018830
rs72666932
rs77692382
rs7488822
rs135922
rs438811
rs139097404
rs117940165
rs28364406
rs9426695
rs6999569
rs405697
rs1866637
rs17665744
rs17821159
rs12292477
rs12658974
rs11063303
rs174560
rs13130041
rs10234749
rs118078695
rs2004196
rs1077835
rs1322199
rs72740858
rs117109614
rs1858942
rs8058610
rs2935412
rs780093
rs7165077
rs1323678
rs35853021
All
0.0
0.1
0.2
0.3
MR leave−one−out sensitivity analysis for
'GCST90199772' on 'Calculus of lower urinary tract'

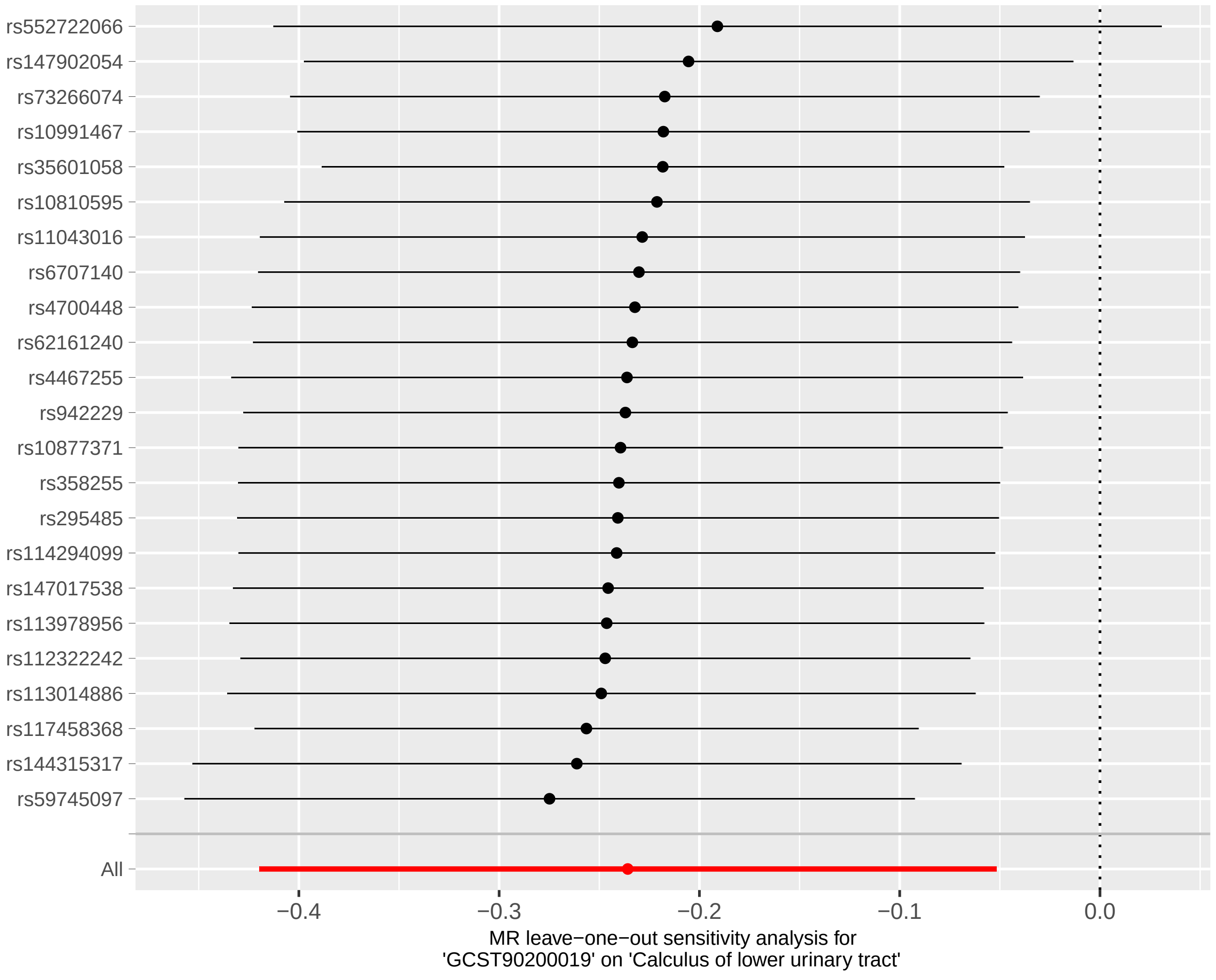
rs552722066
rs147902054
rs73266074
rs10991467
rs35601058
rs10810595
rs11043016
rs6707140
rs4700448
rs62161240
rs4467255
rs942229
rs10877371
rs358255
rs295485
rs114294099
rs147017538
rs113978956
rs112322242
rs113014886
rs117458368
rs144315317
rs59745097
All
−0.4
−0.3
−0.2
−0.1
0.0
MR leave−one−out sensitivity analysis for
'GCST90200019' on 'Calculus of lower urinary tract'

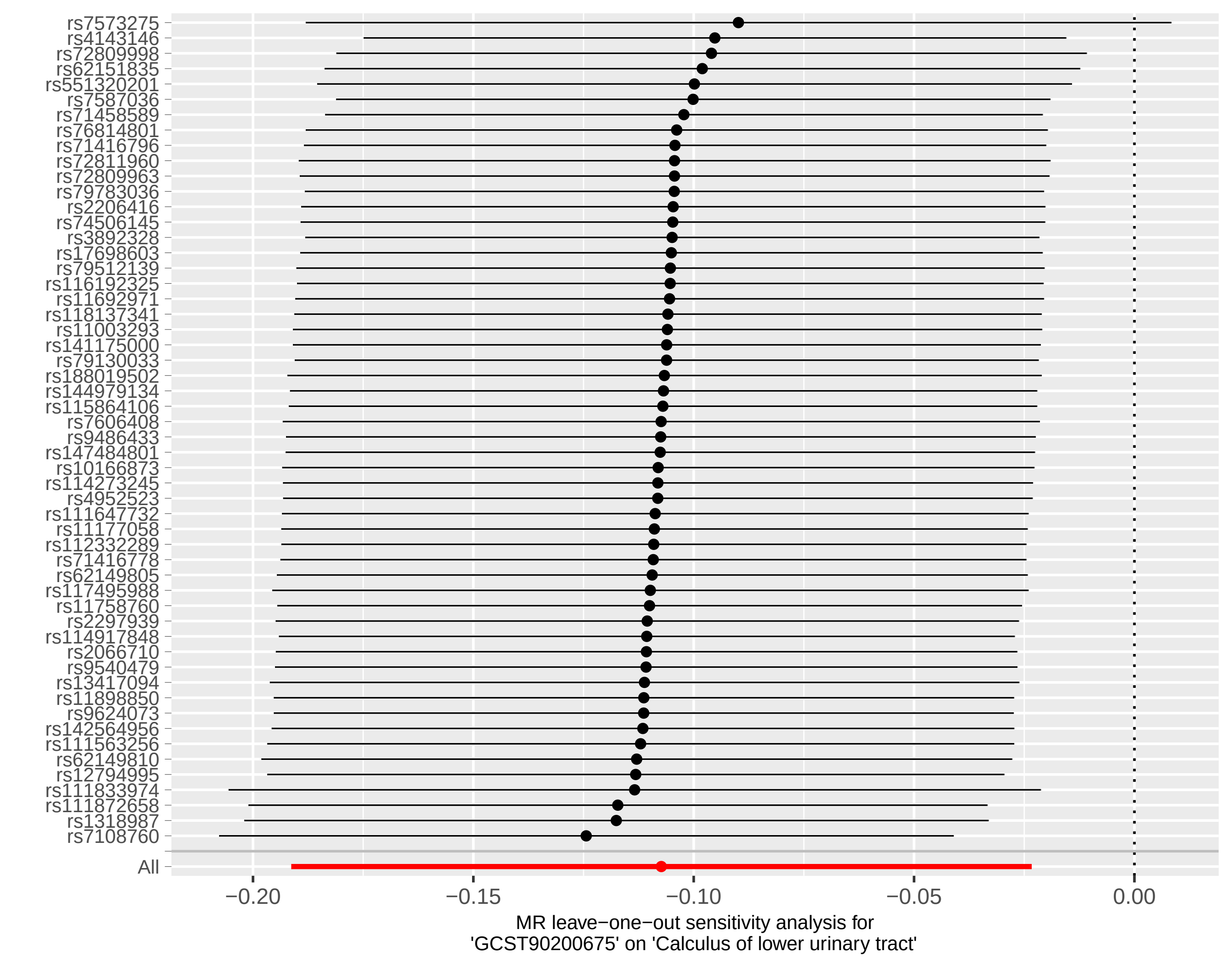
rs7573275
rs4143146
rs72809998
rs62151835
rs551320201
rs7587036
rs71458589
rs76814801
rs71416796
rs72811960
rs72809963
rs79783036
rs2206416
rs74506145
rs3892328
rs17698603
rs79512139
rs116192325
rs11692971
rs118137341
rs11003293
rs141175000
rs79130033
rs188019502
rs144979134
rs115864106
rs7606408
rs9486433
rs147484801
rs10166873
rs114273245
rs4952523
rs111647732
rs11177058
rs112332289
rs71416778
rs62149805
rs117495988
rs11758760
rs2297939
rs114917848
rs2066710
rs9540479
rs13417094
rs11898850
rs9624073
rs142564956
rs111563256
rs62149810
rs12794995
rs111833974
rs111872658
rs1318987
rs7108760
All
−0.20
−0.15
−0.10
−0.05
0.00
MR leave−one−out sensitivity analysis for
'GCST90200675' on 'Calculus of lower urinary tract'

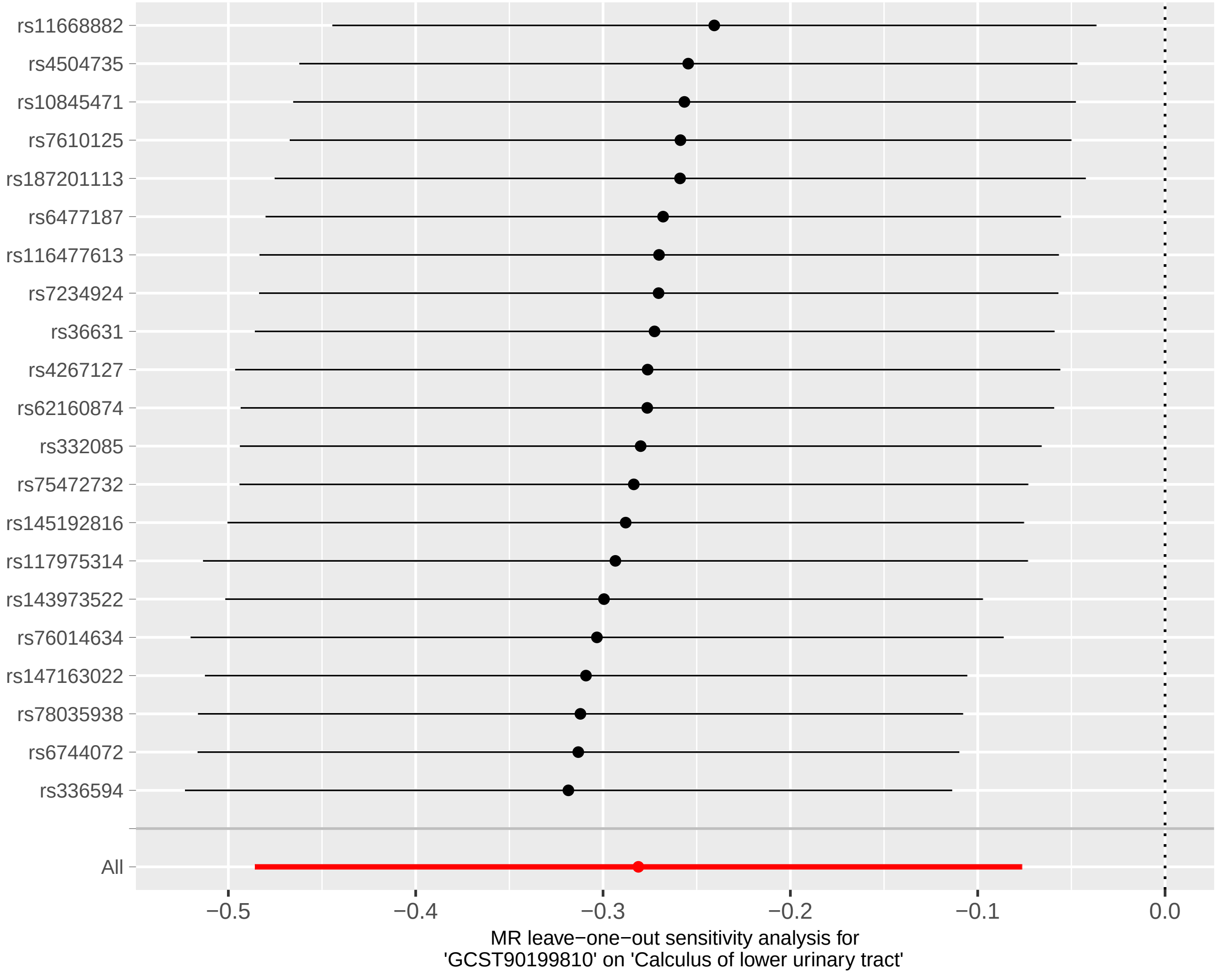
rs11668882
rs4504735
rs10845471
rs7610125
rs187201113
rs6477187
rs116477613
rs7234924
rs36631
rs4267127
rs62160874
rs332085
rs75472732
rs145192816
rs117975314
rs143973522
rs76014634
rs147163022
rs78035938
rs6744072
rs336594
All
−0.5
−0.4
−0.3
−0.2
−0.1
0.0
MR leave−one−out sensitivity analysis for
'GCST90199810' on 'Calculus of lower urinary tract'

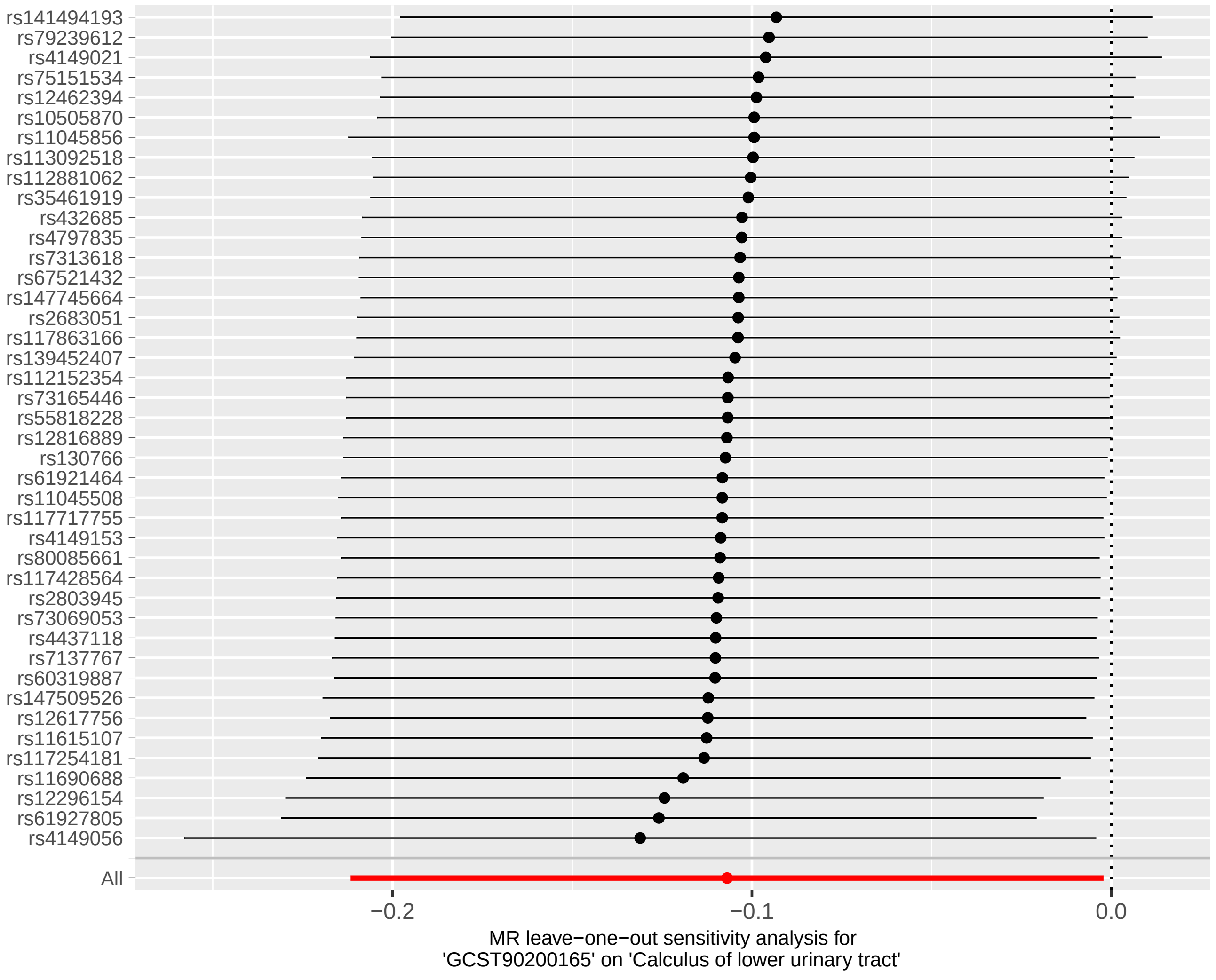
rs141494193
rs79239612
rs4149021
rs75151534
rs12462394
rs10505870
rs11045856
rs113092518
rs112881062
rs35461919
rs432685
rs4797835
rs7313618
rs67521432
rs147745664
rs2683051
rs117863166
rs139452407
rs112152354
rs73165446
rs55818228
rs12816889
rs130766
rs61921464
rs11045508
rs117717755
rs4149153
rs80085661
rs117428564
rs2803945
rs73069053
rs4437118
rs7137767
rs60319887
rs147509526
rs12617756
rs11615107
rs117254181
rs11690688
rs12296154
rs61927805
rs4149056
All
−0.2
−0.1
0.0
MR leave−one−out sensitivity analysis for
'GCST90200165' on 'Calculus of lower urinary tract'
